# Glial enriched gene expression profiling identifies novel factors regulating the proliferation of specific glial subtypes in the *Drosophila* brain

**DOI:** 10.1016/j.gep.2014.09.001

**Published:** 2014-09

**Authors:** Amélie Avet-Rochex, Katja T. Maierbrugger, Joseph M. Bateman

**Affiliations:** Wolfson Centre for Age-Related Diseases, King's College London, Guy's Campus, London SE1 1UL, UK

**Keywords:** Glia, *Drosophila*, Cortex, Perineurial, *foxO*, *Tramtrack*

## Abstract

Glial cells constitute a large proportion of the central nervous system (CNS) and are critical for the correct development and function of the adult CNS. Recent studies have shown that specific subtypes of glia are generated through the proliferation of differentiated glial cells in both the developing invertebrate and vertebrate nervous systems. However, the factors that regulate glial proliferation in specific glial subtypes are poorly understood. To address this we have performed global gene expression analysis of *Drosophila* post-embryonic CNS tissue enriched in glial cells, through glial specific overexpression of either the FGF or insulin receptor. Analysis of the differentially regulated genes in these tissues shows that the expression of known glial genes is significantly increased in both cases. Conversely, the expression of neuronal genes is significantly decreased. FGF and insulin signalling drive the expression of overlapping sets of genes in glial cells that then activate proliferation. We then used these data to identify novel transcription factors that are expressed in glia in the brain. We show that two of the transcription factors identified in the glial enriched gene expression profiles, *foxO* and *tramtrack69*, have novel roles in regulating the proliferation of cortex and perineurial glia. These studies provide new insight into the genes and molecular pathways that regulate the proliferation of specific glial subtypes in the *Drosophila* post-embryonic brain.

## Highlights

• Global gene expression analysis identifies glial specific transcriptomes.
• Different glial subtypes have distinct but overlapping transcriptomes.
• *foxO* and *tramtrack69* are novel regulators of glial subtype specific proliferation.

Glia play many critical roles in the development and maintenance of the nervous system. During development glia provide targets to ensure correct axonal pathfinding. In the mature nervous system glia provide trophic support by ensheathing neuronal cell bodies, processes and synapses. In the mammalian central nervous system (CNS) glia have also been shown to regulate synaptic transmission through modulation of neurotransmitter levels at ‘tripartite synapses’ (Perea et al., 2009). These functions are performed by different classes of glia, such as astrocytes that associate with neuronal cell bodies and synapses, and oligodendrocytes that form myelin sheaths around axons (Freeman and Doherty, 2006). The *Drosophila* CNS also contains several different essential glial classes, such as cortex glia that ensheath neuronal cell bodies and sub-perineurial/perineurial glia that form the blood brain barrier (Hartenstein, 2011).

Up to 50% of the cells in the human brain are glia (Azevedo et al., 2009). To provide sufficient glia for the mature CNS to function correctly, glial cells must be generated either from stem cell populations or through the proliferation of differentiated glia. In both the developing and adult mammalian CNS radial glia act as neural stem cells, which generate a variety of neuronal and glial subtypes (Rowitch and Kriegstein, 2010). Transcription factors (TFs) such as OLIG2, PAX6 and NKX6.1 control glial subtype differentiation from radial glial neural stem cells (Rowitch and Kriegstein, 2010).

In the *Drosophila* embryonic ventral nerve cord (VNC) glia are generated by asymmetric division of neuroglioblast stem cells (Ito et al., 1995). Glial cell fate in the embryonic VNC is regulated by the TF *glial cells missing* (*gcm*), which is necessary for and sufficient to induce gliogenesis (Hosoya et al, 1995, Jones et al, 1995). By contrast, in the *Drosophila* post-embryonic brain two major glial populations, cortex and perineurial glia, are generated by symmetric division of differentiated glial cells (Avet-Rochex et al, 2012, Awasaki et al, 2008, Colonques et al, 2007, Pereanu et al, 2005). Importantly, large scale genesis of glia through symmetric division of differentiated glial cells has also recently been observed in mammals, where differentiated astrocytes proliferate to generate large glial populations in the postnatal mouse brain (Ge et al., 2012). Therefore, gliogenesis through the proliferation of differentiated glia in the post-embryonic brain is conserved in flies and mammals. However, the genes that regulate the cell division of astrocytes are not known and the genetic regulation of proliferation of specific glial subtypes in *Drosophila* has only begun to be explored.

Two major questions arise from these studies of glial proliferation: (1) What are the factors that define glial subtype identity? (2) What are the factors and pathways that regulate the proliferation of specific glial subtypes? We have recently shown that proliferation of cortex and perineurial glia in the post-embryonic brain is driven by the fibroblast growth factor (FGF) and insulin receptor (InR)/mechanistic target of rapamycin (mTOR) pathways, which differentially regulate cortex and perineurial glial proliferation (Avet-Rochex et al., 2012). However, the molecular mechanism by which these pathways regulate the proliferation of these specific glial subtypes is not known. To address these questions we have characterised global gene expression profiles from *Drosophila* postembryonic CNS tissue that is enriched for proliferating glial cells driven by either FGF or InR signalling. These two pathways have differential effects on specific glial subtypes, which are reflected in the respective gene expression profiles. To test the efficacy of these expression datasets we focused on TFs. We show that two of the TFs identified, *kayak* and *hairy*, are indeed expressed specifically in glia. Finally we show that another two of the TFs identified, *foxO* and *tramtrack69*, regulate the proliferation of specific glial subtypes.

## 1 Results and discussion

### 1.1 Global gene expression profiling of glia in the post-embryonic CNS

**Fig. 1:**
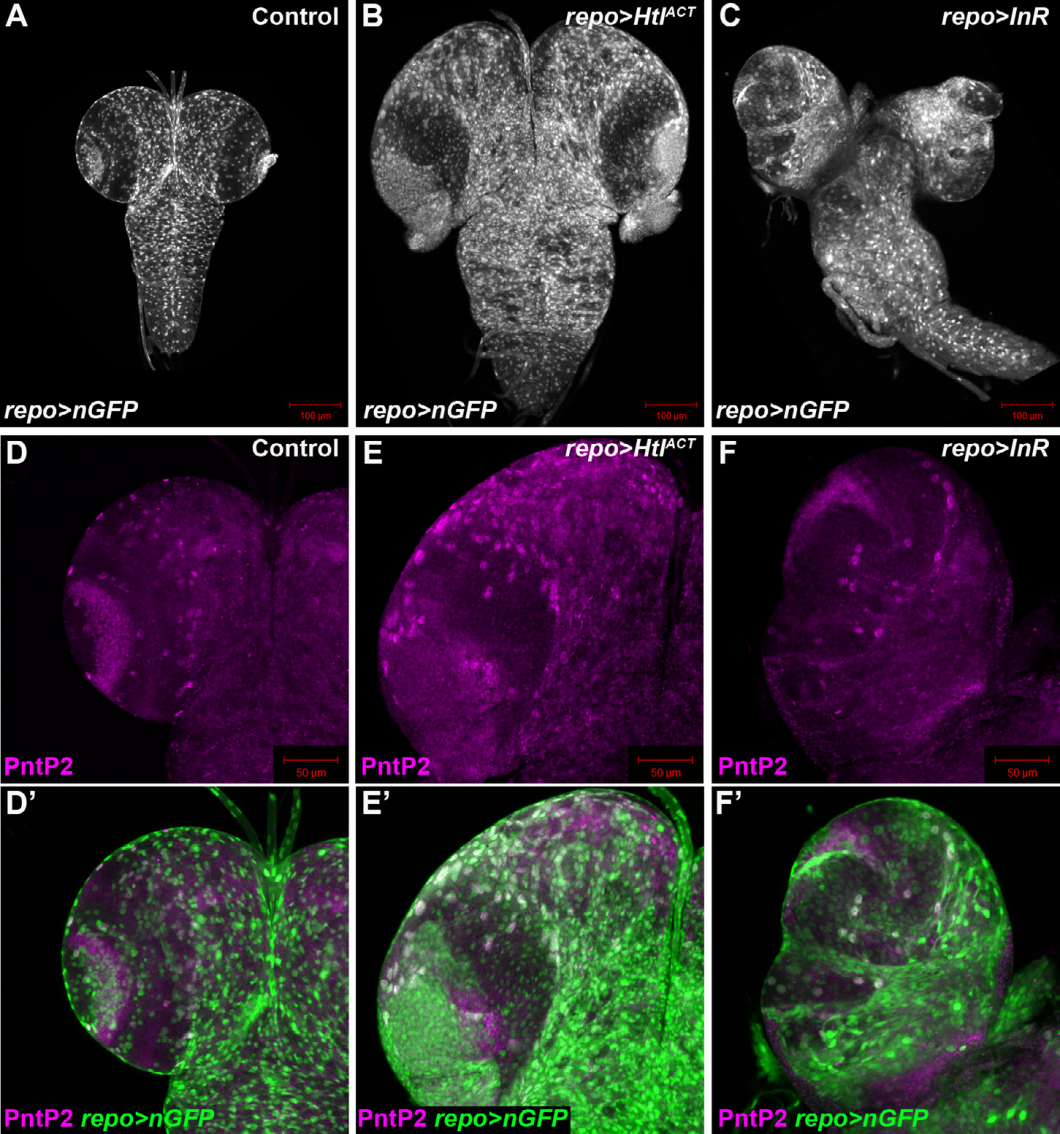
* *Generation of larval CNS tissue enriched in glia. (A) Late third instar larval CNS expressing nuclear GFP in glia using *repo-Gal4* (*repo>nGFP*). (B,C) Overexpression of *Htl^ACT^* (B), or the *InR* (C) in glia using *repo-Gal4* causes glial overproliferation. Glia are marked by the expression of nuclear GFP as in A. (D–F') Overexpression of *Htl^ACT^* (E), but not the *InR* (F), in glia using *repo-Gal4* causes overproliferation of PntP2 expressing cortex glia. PntP2 expression shown in magenta (D–F') and glia (green in D–E') are marked by the expression of nuclear GFP as in (A–C).

We have recently shown that the proliferation of two glial subtypes in the *Drosophila* post-embryonic brain is regulated through the concerted action of the FGF and InR/mTOR pathways. Cortex glia require FGF signalling and the InR, but not downstream components of the InR/mTOR pathway, whereas perineurial glia require both FGF and InR signalling pathways for proliferation. Pan-glial activation of either pathway causes glial overproliferation (Fig. 1B,C). However, specific glial sub-types respond differently to the expression of each receptor. The majority of superficial glia in larval brains from animals overexpressing an activated form of the FGF receptor (Htl^ACT^) in glia expressed both the pan-glial protein Repo and pointedP2 (PntP2), a marker of cortex glia (Fig. 1E,E'). By contrast, glial-specific overexpression of the *InR* resulted in the proliferation of Repo expressing, but not PntP2 expressing glia (Fig. 1F,F'). These data suggest that these two receptors promote glial proliferation, but that the glial subtypes that proliferate are partially distinct.

The glial overproliferation phenotype caused by overexpression of *Htl^ACT^* and the *InR* (Fig. 1B,C) provided the opportunity to determine the global gene expression profile of glia in these tissues by comparing transcript levels from CNS tissue overexpressing either *Htl^ACT^*, or the *InR* in glia, to that of control CNS tissue. We postulated that CNS tissue from larvae with increased glial numbers would be significantly enriched for the expression of glial genes, compared to CNS tissue from control larvae. Therefore, we dissected the CNS from third instar larvae overexpressing either *Htl^ACT^*, or the *InR* in glia (using *repo-Gal4*), or from control larvae. RNA isolated from CNS tissue was then used for microarray gene expression analysis (see Experimental procedures).

### 1.2 Glial specific FGF and InR pathway activation results in different but overlapping glial enriched gene expression profiles

**Fig. 2:**
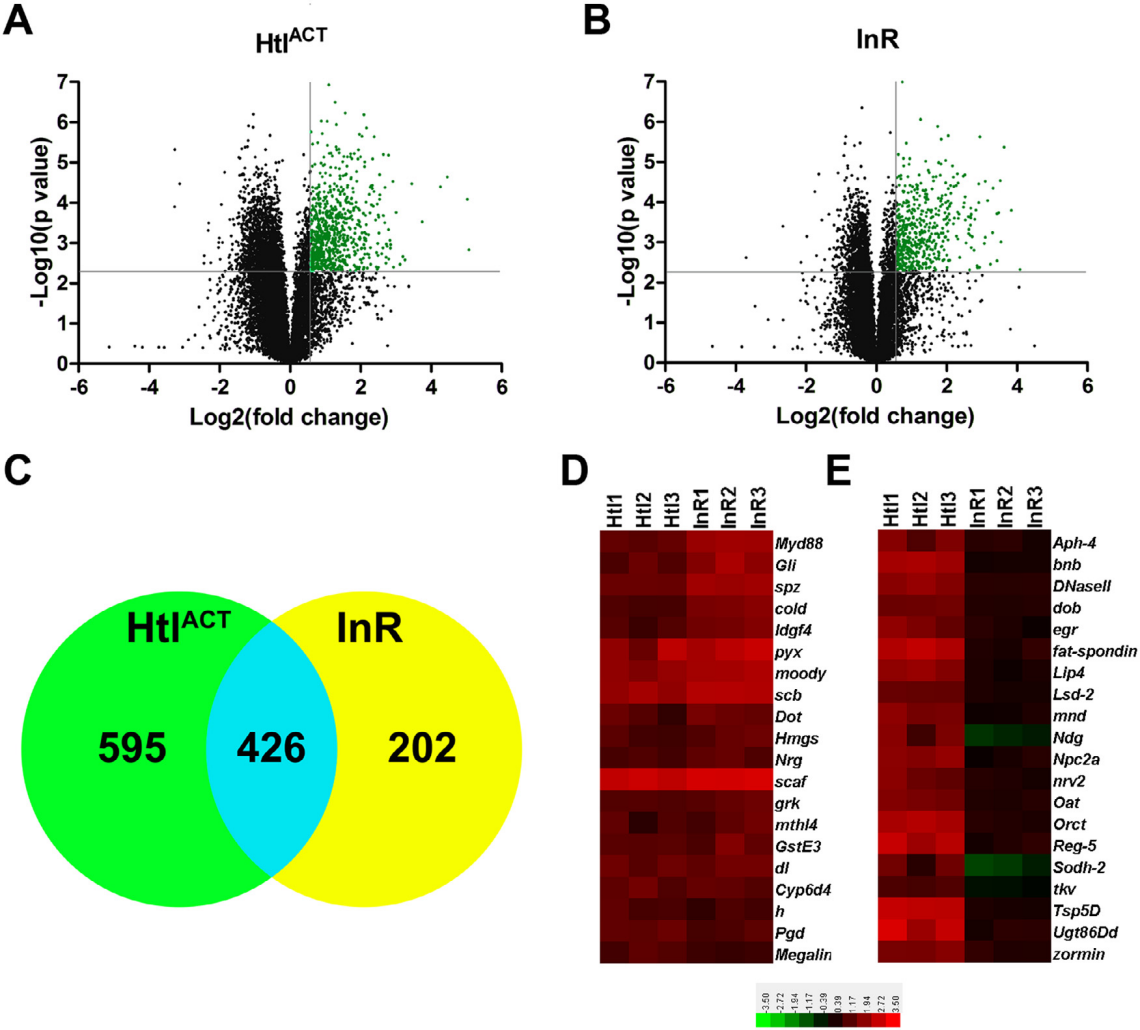
* *Glial enriched larval CNS gene expression profiles. (A,B) Volcano plots of transcript expression levels from larval CNS tissue overexpressing *Htl^ACT^* (A), or the *InR* (B) in glia using *repo-Gal4*. Transcripts whose expression increased ≥1.5 fold with a p value ≤0.05 are shown in green. (C) Venn diagram showing the numbers of genes whose expression was significantly increased ≥1.5 fold in either *Htl^ACT^* overexpressing CNS tissue (green circle), *InR* overexpressing CNS tissue (yellow circle), or in both conditions (blue overlap). (D,E) Heat maps representing expression levels (log_2_) of 20 genes whose expression was similar (D), or significantly different (E) in *Htl^ACT^* (Htl1-3) and *InR* (InR1-3) overexpressing CNS tissue.

Analysis of transcript expression levels showed that the expression of 1021 genes was increased ≥1.5 fold and 583 genes increased ≥2 fold in *Htl^ACT^* overexpressing CNS tissue (Fig. 2A, Supplementary Table S1). Expression of the glial-specific gene *repo* was increased 2.5 fold, while expression of *pnt* (the probe sequence was common to both *pntP1* and *pntP2* isoforms) was increased 4.96 fold (Supplementary Table S1). We previously showed that the number of Repo expressing superficial glia in *Htl^ACT^* overexpressing brains was increased 2.27 fold, while the number of PntP2 expressing cortex glia was increased 3.65 fold (Avet-Rochex et al., 2012). Therefore, the changes in expression of *repo* and *pnt* correlate with the increase in glial numbers in *Htl^ACT^* overexpressing tissue. Moreover, expression of other genes previously established to have roles in glial biology including *bangles* (*bnb*) (Ng et al., 1989), *wrapper* (Noordermeer et al., 1998), *gliotactin* (*Gli*) (Auld et al., 1995), *kruppel* (*Kr*) (Romani et al., 1996), *sinuous* (*sinu*), *pickel* (*pck*) (Stork et al., 2008), *myoglianin* (*myo*) (Lo and Frasch, 1999), *held out wings* (*how*) (Edenfeld et al., 2006), *glial lazarillo* (*Glaz*) (Sanchez et al., 2000), *inebriated* (*ine*) (Yager et al., 2001), *neuroglian* (*Nrg*) (Banerjee et al., 2006), *Contactin* (*Cont*) (Banerjee et al., 2006), *moody, G protein α i subunit* (*G-ialpha65A, Gαi*) and *locomotion defects* (*loco*) (Schwabe et al., 2005), were all significantly increased in *Htl^ACT^* overexpressing tissue (Supplementary Table S1). GO analysis of cellular processes of genes with significantly increased expression in *Htl^ACT^* tissue showed that the classes ‘establishment of the glial blood–brain barrier’ and ‘septate junction assembly’ were significantly over-represented (Supplementary Table S8). Taken together these data strongly suggest that this dataset is significantly enriched for glial-expressed genes. The GO analysis also showed that genes involved in small molecule, lipid and carbohydrate metabolism were significantly over-represented (Supplementary Table S8), suggesting that these proliferating glial cells are highly metabolically active.

In tissue overexpressing the *InR* in glia the expression of 628 genes were significantly increased ≥ 1.5 fold and 383 genes ≥ 2 fold (Fig. 2, Table S2). *repo* expression was significantly increased (1.68 fold), which correlates well with the 1.64-fold increase in Repo-expressing superficial glia in *InR* overexpressing brains (Avet-Rochex et al., 2012). The fact that there were fewer differentially upregulated genes in *InR* overexpressing tissue than in *Htl^ACT^* overexpressing tissue may reflect the smaller increase in glial numbers in *InR* overexpressing tissue, compared to *Htl^ACT^* overexpressing tissue (Fig. 1B,C) (Avet-Rochex et al., 2012). Of the 628 genes whose expression was increased in *InR* overexpressing tissue, 426 were also increased in *Htl^ACT^* overexpressing tissue (Fig. 2, Table S3). However, 32% (202) of genes with increased expression in *InR* overexpressing tissue were not significantly increased in *Htl^ACT^* overexpressing tissue (Fig. 2, Table S4), suggesting differences in the gene expression landscape, or glial subtypes, in these two tissues. As with *Htl^ACT^* expressing tissue, expression of a number of genes with characterised functions in glial biology were significantly increased in *InR* overexpressing tissue including *Gli, pck, sinu, moody, Cont* and *Nrg* (Supplementary TableS2), all of which were also increased in *Htl^ACT^* overexpressing tissue (Supplementary Table S1). As expected from the lack of increase in cortex glia in *InR* overexpressing tissue (Fig. 1F), expression of *pnt* was not significantly increased in *InR* overexpressing tissue. Similar to *Htl^ACT^* overexpressing tissue, GO analysis showed that genes involved in the establishment of the blood brain barrier and septate junction assembly were over-represented in tissue overexpressing the *InR* (Supplementary Table S9). However, unlike *Htl^ACT^* overexpressing tissue (Supplementary Table S8), metabolic genes were not over-represented in *InR* overexpressing tissue. Furthermore, genes involved in the innate immune response were enriched in this tissue, but not in *Htl^ACT^* overexpressing tissue (Supplementary Table S9). Thus, overexpression of the *InR* in glia results in a gene expression profile that overlaps with, but has significant differences to that of glia overexpressing *Htl^ACT^*.

We hypothesised that neuronal specific genes would be over-represented in the group of genes whose expression was significantly decreased in tissue overexpressing *Htl^ACT^* or the *InR* in glia. The expression of 1654 genes was significantly decreased ≥1.5 fold in CNS tissue overexpressing *Htl^ACT^* in glia (Supplementary Table S5), while the expression of 240 genes were significantly decreased ≥1.5 fold in *InR* overexpressing tissue (Supplementary Table S6). Of the 240 genes whose expression was significantly decreased in *InR* overexpressing tissue 89% (213) were also decreased in *Htl^ACT^* overexpressing tissue (Supplementary Table S7). GO analysis of genes with significantly decreased expression in tissue overexpressing *Htl^ACT^* in glia showed that cellular processes including ‘generation of neurons’, ‘neuron differentiation’, ‘neuron development’, ‘axonogenesis’, ‘axon guidance’, ‘neuroblast differentiation’ and ‘synaptic transmission’ were all over-represented (Supplementary Table S9). Very few GO classes were over-represented in the group of genes with significantly decreased expression from tissue overexpressing the *InR* in glia, but one of these was ‘neuropeptide signalling pathway’ (Supplementary Table  S11). These bioinformatic analyses suggest that the group of genes with differentially decreased expression is strongly enriched for genes expressed in neurons in the larval CNS. However, this group may also include genes whose expression in glia is suppressed by overexpression of *Htl^ACT^* or the *InR*.

### 1.3 Expression analysis of TFs expressed in superficial glia in the post-embryonic brain

**Fig. 3:**
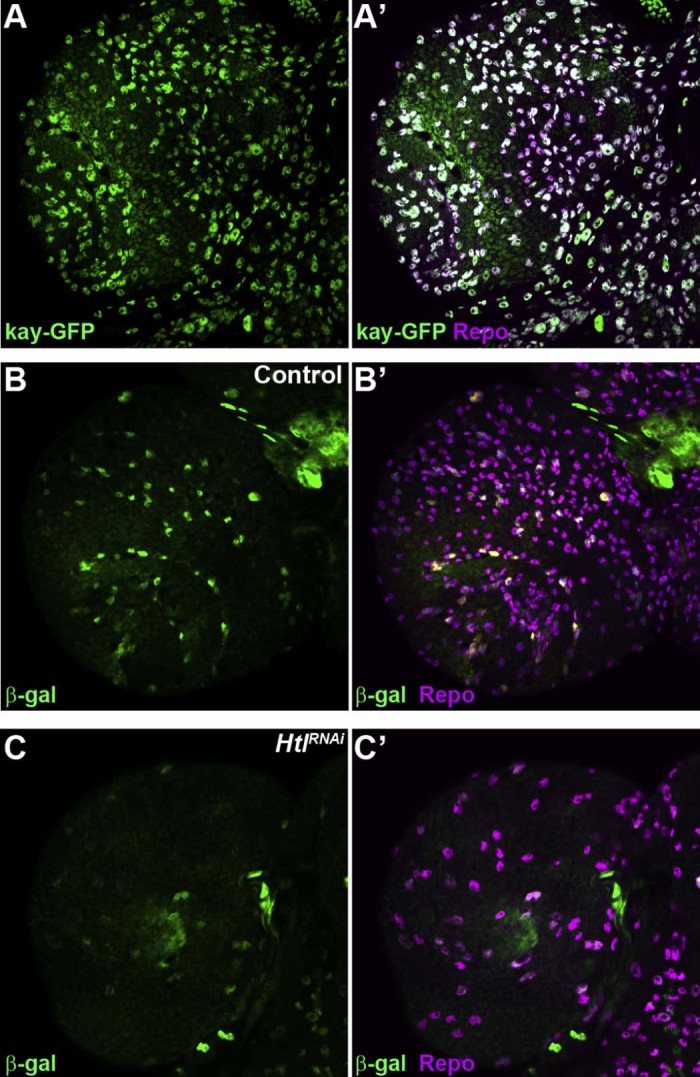
* kayak* and *hairy* are expressed in glia in the brain. (A,A') Superficial layer of a late third instar larval brain expressing kayak-GFP (kay-GFP) stained for GFP (green) and Repo (magenta) expression. (B,B') β-Galactosidase expression (green) in the superficial layer of a control brain from a *h^E11^* enhancer trap larva, co-stained for Repo expression (magenta). (C,C') β-Galactosidase expression (green) in the superficial layer of a *repo-Gal4>htl^RNAi^* third instar larval brain carrying the *h^E11^* enhancer trap, co-stained for Repo expression (magenta).

Although several of the genes whose expression was significantly increased in both *Htl^ACT^* and *InR* overexpressing CNS tissue had been previously shown to function in glia, we sought to experimentally test the efficacy of the microarray datasets as a source of genes that are expressed in cortex glia and/or surface glia (perineurial and sub-perineurial glia) in the brain. We focused on TFs, as these frequently play important roles in gliogenesis. The expression of 21 TFs was significantly increased in *Htl^ACT^* overexpressing tissue (Table 1), while the expression of 10 TFs was significantly increased in *InR* overexpressing tissue (Table 2). Fifteen of the TFs whose expression was increased in *Htl^ACT^* overexpressing tissue were not increased in *InR* tissue (Table 1), while four (*kni, kay, Usf* and *ci*) were unique to *InR* overexpressing tissue (Table 2). We tested antibodies against several of the TFs identified (Dorsal, Krüppel, Knirps, cubitus interruptus, FoxO and Mef2), but these gave either weak staining or high background staining in the larval brain (data not shown). However, a GFP fusion of *kayak* showed expression in both cortex and surface glia in the larval brain (Fig. 3A). Also, a *lacZ* enhancer trap in *hairy* (*h^E11^*) showed clear β-galactosidase expression specifically in cortex glia (Fig. 3B). Moreover, inhibition of glial proliferation by knock-down of *htl* using *repo-Gal4* caused a dramatic reduction in the number of *hairy* expressing glia (Fig. 3C). These results further validate the glial enriched gene expression datasets as a source of glial-expressed genes and also as a means of identifying genes whose expression is specific at least to cortex glia.

**Table 1:** TFs with significantly increased expression ≥ 1.5 fold in *repo-Gal4, UAS-Htl^Act^* CNS tissue.

Gene	Fold expression change	Characterised role in glia
*pointed* (*pnt*)*	4.96	Yes (Klambt, 1993)
*Kruppel* (*Kr*)*	3.9	Yes (Romani et al., 1996)
CG3328*	3.65	No
*Pif1A*	2.9	No
Hnf4*	2.17	No
*dorsal* (*dl*)	2.72	Yes (Kato et al., 2009)
CrebA*	2.62	No
*Repo*	2.5	Yes (Xiong et al., 1994)
*Hairy* (*h*)*	2.23	Yes (Giangrande, 1995)
*tramtrack* (*ttk*)	2.1	Yes, this study and (Badenhorst, 2001)
Xbp1*	1.88	Yes (Sone et al., 2013)
foxO*	1.87	Yes, this study and (Lavery et al., 2007)
*Gemini* (*gem*)*	1.83	No
Edl*	1.83	Yes (Yamada et al., 2003)
NFAT*	1.83	No
*CG2678*	1.71	No
Mef2*	1.66	No
*CG13188*	1.63	No
cup*	1.55	No
luna*	1.54	No
Eaf*	1.52	No

* Expression not significantly increased in *repo-Gal4, UAS-InR* CNS tissue.

**Table 2:** TFs with significantly increased expression ≥ 1.5 fold in *repo-Gal4, UAS-InR* CNS tissue.

Gene	Fold expression change	Characterised role in glia
*dorsal* (*dl*)	2.88	Yes (Kato et al., 2009)
*CG2678*	2.48	No
*knirps* (*kni*)*	2.31	No
*kayak* (*kay*)*	2.01	Yes (Macdonald et al., 2013)
*Pif1A*	2.0	No
Usf*	1.9	No
*tramtrack* (*ttk*)	1.82	Yes, this study and (Badenhorst, 2001)
*CG13188*	1.75	No
*repo*	1.68	Yes (Xiong et al., 1994)
*cubitus interruptus* (*ci*)*	1.54	Yes (Rangarajan et al., 2001)

* Expression not significantly increased in *repo-Gal4, UAS-Htl^ACT^* CNS tissue.

### 1.4 *foxO* and *tramtrack* regulate glial proliferation in the *Drosophila* post-embryonic brain

To test whether the glial enriched gene expression datasets could be used to identify genes involved in the regulation of glial proliferation in the post-embryonic brain we focused on two of the TFs identified in these datasets, *foxO* and *tramtrack* (*ttk*) (Table 1, Table 2). *foxO* and *ttk* have both been found previously to have roles in glial development in *Drosophila*, but their potential roles in glial proliferation in the post-embryonic brain are not known. FoxO is a negative regulator of growth, acting downstream of the InR and PI3K (Eijkelenboom and Burgering, 2013). FoxO has been shown to act in the InR pathway to regulate perineurial glial size in the peripheral nervous system (Lavery et al., 2007). *ttk* is a transcriptional repressor that acts to inhibit the expression of neuronal genes in embryonic glial development and to negatively regulate the proliferation of embryonic longitudinal glia (Badenhorst, 2001).

**Fig. 4:**
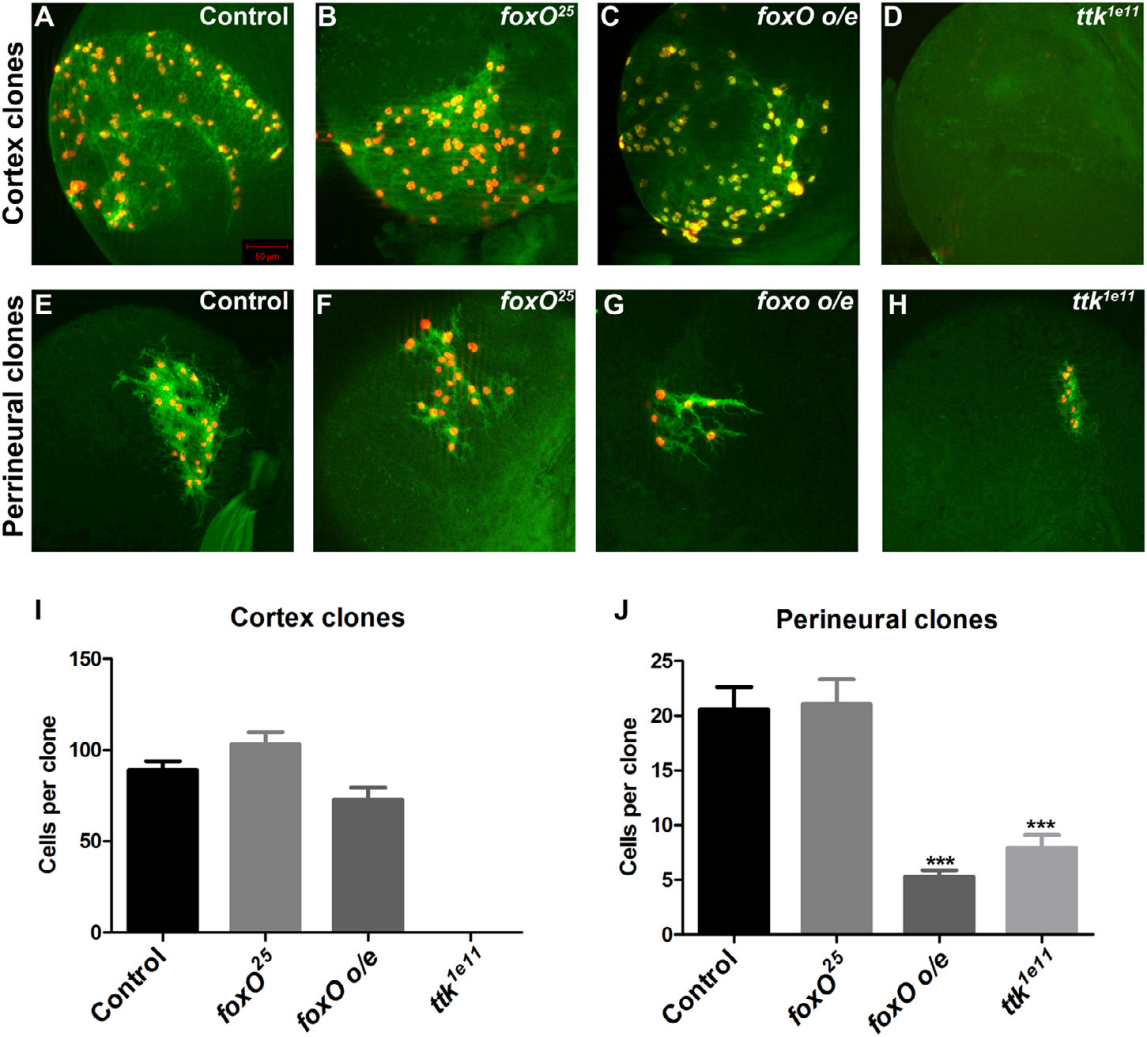
* foxO* and *ttk69* regulate glial proliferation in the postembryonic brain. (A–D) Representative *repo*-MARCM cortex clones marked with GFP (green) and nuclear-RFP (red) expression. (E–H) Representative *repo*-MARCM perineurial clones marked with GFP (green) and nuclear-RFP (red) expression. (I) Quantification of cortex *repo-*MARCM clone sizes. Average clone size of *FRT82B* control clones (n = 10), *foxO^25^* (n = 9), *foxO* overexpression (o/e) (n = 8) and *ttk^1e11^* clones (no cortex clones were observed in >50 brains). (J) Quantification of perineurial *repo-*MARCM clone sizes. Average clone size of *FRT82B* control clones (n = 34), *foxO^25^* (n = 49), *foxO* overexpression (o/e) (n = 24) and *ttk^1e11^* clones (n = 24). Data are represented as mean +/− SEM. ***p < 0.001.

To test the requirement for *foxO* in cortex and perineurial glia we generated *repo*-MARCM clones homozygous for a loss-of-function (LOF) mutation in *foxO* (*foxO^25^*). Loss of *foxO* did not affect the size of either cortex or perineurial glial clones (Fig. 4B,F,I,J). FoxO regulates growth control downstream of the InR, but *foxO* mutants do not have a growth phenotype, whereas overexpression of *foxO* inhibits growth (Junger et al., 2003). We therefore overexpressed *foxO* using *repo*-MARCM and found that this did not affect cortex clones but caused a significant reduction in perineurial glial clone size (Fig. 4C,G,I,J). Therefore, *foxO* is sufficient to inhibit glial proliferation specifically in perineurial glia.

Ttk is a transcriptional repressor and its first characterised functional role was in cell fate determination in the *Drosophila* eye (Xiong and Montell, 1993). *Drosophila* has two Ttk isoforms, Ttk88 and Ttk69, which differ in their carboxyl-terminal DNA binding zinc finger domains (Harrison, Travers, 1990, Read, Manley, 1992). *ttk88* is not required for glial development in the *Drosophila* embryo, whereas loss of *ttk69* causes increased proliferation of longitudinal glia (Badenhorst, 2001). Surprisingly, LOF *repo*-MARCM analysis of *ttk* using *ttk^1e11^*, an allele specific to the Ttk69 isoform (Lai and Li, 1999), demonstrated that *ttk69* is positively required in both cortex and perineurial glia. We did not observe a single cortex clone that was mutant for *ttk69* and perineurial *ttk69* clones were significantly smaller than control clones (Fig. 4D,H–J). Therefore, *ttk69* is a key regulator of both cortex and perineurial glial proliferation in the *Drosophila* post-embryonic brain.

The proliferative potential of differentiated glia has recently been demonstrated in both the *Drosophila* and vertebrate CNS, but the genetic regulation of this process is poorly understood. We profiled the global gene expression pattern of CNS tissue enriched for different subsets of glial cells through activation of either FGF or InR signalling. Our data and analyses strongly suggest that these glial transcriptomes are highly enriched for overlapping but distinct sets of glial genes and can be used as a resource for identification of novel glial genes expressed in specific glial subtypes. Conversely, the set of genes whose expression is decreased provides a resource of neuronally expressed genes. As a proof-of-principle we then used these data to identify two genes that specifically regulate cortex and perineurial glial proliferation in the post-embryonic brain.

Three studies have previously attempted to identify glial genes by gene expression profiling, all in the *Drosophila* embryo (Altenhein et al, 2006, Egger et al, 2002, Freeman, Doherty, 2006). The first two studies induced gliogenesis by ectopic expression of *gcm* in the embryonic nervous system (Egger et al, 2002, Freeman et al, 2003). Freeman et al. (2003) found a high rate of false positives (88%) when the differentially regulated genes were analysed by in situ hybridisation and suggested a similar rate of false positives in the genes identified by Egger et al. (2002). In addition to microarray analysis Freeman et al. combined expression databases and computational analysis of *gcm* target genes to identify 45 new *Drosophila* glial genes (Freeman et al., 2003). With the goal of improving on these earlier studies Altenhein et al., in addition to ectopic *gcm* expression, used *gcm* mutant embryos to identify glial genes (Altenhein et al., 2006). Surprisingly, there was not a great deal of overlap between the differentially regulated genes identified in these three studies (Altenhein et al., 2006). Similarly, we found a relatively low degree of overlap between the genes identified in these previous studies and the genes with significantly increased expression from larval CNS tissue overexpressing *Htl^ACT^* in glia. Twenty-one per cent (68 of 328) of the glial genes identified by Altenhein et al. (2006), 31% (14 of 45) of the glial genes identified by Freeman et al. (2003), and 9% (23 of 257) of the glial genes from the Egger et al. (2002), study were present in our *Htl^ACT^* significantly increased gene set (Supplementary Table S1). To some extent this is not surprising as our study used the late third instar larval CNS and induced gliogenesis through overexpression of *Htl^ACT^*, rather than overexpression or loss of *gcm*. The differences may also reflect the different gene expression patterns of glia generated through glial cell division and glia generated through ectopic differentiation from neuroglioblast precursors.

The first question we aimed to address using gene expression profiling was the identity of factors that define specific glial subtypes. Overexpression of *Htl^ACT^* and the *InR* drives the proliferation of different but overlapping glial subtypes and this is reflected in the sets of genes whose expression was significantly increased in either tissue. Focusing on TFs we found that *kayak* is expressed in cortex and surface glia, while *hairy* expression is specific to cortex glia. Taken together our data extend our previous work demonstrating that cortex and surface glial have distinct gene expression signatures that define each glial subtype.

The second question we aimed to address was the identity of novel genes and pathways that regulate the proliferation of specific glial subtypes. TFs such as *dorsal, foxO* and *ci*, whose expression was significantly increased in *Htl^ACT^* and *InR* overexpressing tissue (Table 1, Table 2), are known to regulate cell proliferation in other contexts and so are good candidates as regulators of glial proliferation. *Mef2* had not been previously shown to have a role in glia, but was differentially upregulated in *Htl^ACT^* (but not *InR*) overexpressing CNS tissue (Table 1). *Mef2* has recently been shown to act synergistically with Notch to activate cell proliferation by inducing the expression of the matrix metalloproteinase *Mmp1* and the TNF ligand *eiger* (*egr*) in *Drosophila* (Pallavi et al., 2012). Interestingly, the expression of both *Mmp1* and *egr* are also increased in *Htl^ACT^* overexpressing tissue (Supplementary Table S1). *Mef2* has also been identified as a transcriptional target of *dorsal* in the embryonic mesoderm (Stathopoulos et al., 2002), suggesting a potential hierarchical relationship between *dorsal* and *Mef2* in regulating glial proliferation in the larval CNS.

A second TF that had not been previously recognised to have a role in glia, but whose expression was significantly increased in *InR* overexpressing tissue (Table 2), is the gap gene *knirps. knirps* is required for embryonic segmentation and has also been shown to act downstream of Decapentaplegic (Dpp) signalling in the *Drosophila* tracheal system (Chen et al., 1998). Dpp signalling regulates glial proliferation in the *Drosophila* eye (Rangarajan et al., 2001) and Dpp expression is significantly increased in both *Htl^ACT^* and *InR* overexpressing tissue (Supplementary Tables S1 and S2). Expression of the Dpp receptors *thickvein* (*tkv*) and *glass bottom boat* (*gbb*) are also significantly increased in *Htl^ACT^* overexpressing tissue (Supplementary Table S1). Thus, *knirps* may act downstream of Dpp signalling to regulate the proliferation of cortex glia.

To test whether two of the TFs we identified were required for proliferation of either cortex or perineurial glia we used *repo*-MARCM LOF analysis. We found that *foxO* is not necessary for glial proliferation, but is sufficient to specifically inhibit the proliferation of perineurial glia. FoxO is a negative regulator of growth and upon activation of the InR pathway FoxO is phosphorylated by AKT, which causes FoxO to be sequestered in the cytoplasm (Junger et al., 2003). We previously proposed a model in which PI3K signalling acts together with the FGF pathway to regulate perineurial glial proliferation, whereas PI3K signalling is not required for cortex glia proliferation (Avet-Rochex et al., 2012). The inhibition of perineurial but not cortex glial proliferation by *foxO* overexpression fits well with this model and extends our previous findings, suggesting that FoxO acts as a negative regulator of perineurial glial proliferation downstream of InR/PI3K signalling specifically in perineurial glia.

We also found that *ttk69* is positively required for the proliferation of both cortex and perineurial glia. Although *ttk69* is a negative regulator of longitudinal glial proliferation in the *Drosophila* embryo (Badenhorst, 2001), *ttk69* is positively required to promote photoreceptor development in the late pupal stage during *Drosophila* eye development (Lai and Li, 1999), thus a positive role for *ttk69* is not unprecedented. *ttk69* is absolutely required for cortex glial proliferation but only partially required in perineurial glia. This phenotype is very similar to the requirement for components of the FGF pathway in glial proliferation (Avet-Rochex et al., 2012). We therefore suggest that Ttk69 acts downstream of FGF signalling to regulate cortex and perineurial glial proliferation in the larval brain.

### 1.5 Conclusions

Future studies will fully dissect the roles of *foxO* and *ttk* in glial proliferation, but our data demonstrate that the glial transcriptomes we have characterised can be used to identify genes that have key roles in regulating subtype specific glial proliferation in the larval brain.

## 2 Experimental procedures

### 2.1 *Drosophila* stocks

Flies were maintained on standard yeast, glucose, agar food at 25 °C unless otherwise stated. *h^E11^* was from David Ish-Horowicz and *FRT82B,foxO^25^* from Helen McNeill. *FRT82B, Kay-GFP, UAS-Htl^ACT^, UAS-InR, UAS-foxO, FRT82B, ttk^1e11^, UAS-RedStinger* and *repo-Gal4* were from the Bloomington Stock Center. The *repo-*MARCM stock genotype was as described previously (Avet-Rochex et al., 2012), but using UAS-RedStinger instead of UAS-nLacZ to visualise nuclei: UAS-*RedStinger*; *repo-flp, repo-Gal4, UAS-actinGFP*; *FRT82B, tub-Gal80*. Knock-down of *htl* was performed as described previously (Avet-Rochex et al., 2012).

### 2.2 Immunofluorescence and imaging

Antibody staining was performed as previously described (Avet-Rochex et al., 2012). Antibodies were mouse anti-Repo (DSHB, 1/100), rat anti-PntP2 (Avet-Rochex et al., 2012; 1/500), chicken anti-β-galactosidase (Abcam, 1/1000), rabbit anti-GFP (Molecular Probes, 1/1000). Secondary antibodies were from Invitrogen. Imaging was performed on a Zeiss LSM 710 and images were processed in Adobe Photoshop.

*repo-*MARCM clone sizes were quantified manually in ImageJ by quantifying numbers of RFP positive nuclei per clone. Statistical analysis was performed in GraphPad Prism using one way ANOVA with Dunnett's post hoc test.

### 2.3 Microarray experiments and data analysis

For microarray analysis, the complete CNS from 10–15 wandering third instar larvae were dissected in PBS on ice and then transferred into 100 µl of cold lysis buffer from the Absolutely RNA Microprep kit (Stratagene) and vortexed for 5 s. Total RNA was then prepared using this kit according to the manufacturer's instructions. For each genotype RNA samples were prepared in triplicate and stored at −80 °C. cRNA was prepared from 500 ng of total RNA using the Ambion Premier kit (Ambion) and hybridisations were performed using the Genechip 3'IVT kit (Affymetrix) on Genechip *Drosophila* Genome 2.0 Arrays (Affymetrix). Imaging of the arrays was performed using the Affymetrix GCS3000 microarray system.

Data normalisation was performed using the Microarray Suite version 5 (MAS 5.0) statistical algorithm using the Affymetrix Expression Console software. Probes where the detection p-value (calculated using the intensity value of a perfect match to a mismatch sequence) was >0.06 in any of the samples were classed as ‘absent’ (A) and excluded from further analysis. Using this criterion, 8638 and 8779 unique probes were included for control versus *Htl^ACT^* tissue and control versus *InR* tissue respectively. Relative differences in gene expression were calculated using the array statistical programme Significance Analysis of Microarrays (SAM) (Tusher et al., 2001). SAM uses gene expression measurements and a response variable to determine if the expression of any genes is significantly related to the response. We used a two class unpaired response type, using log_2_ of the raw expression values, selecting genes whose expression had increased either ≥1.5 or decreased ≤1.5 fold with a false discovery rate of 0.58% (*repo-Gal4>Htl^ACT^*) and 0.56% (*repo-Gal4>InR*).

Volcano plots were generated using GraphPad Prism 5. Heat maps were generated from log_2_ values of the expression change values using Cluster 3.0 (Eisen et al., 1998) and Java Treeview (Saldanha, 2004). The data discussed in this publication have been deposited in NCBI's Gene Expression Omnibus (Edgar et al., 2002) and are accessible through GEO Series accession number GSE46317 (http://www.ncbi.nlm.nih.gov/geo/query/acc.cgi?acc=GSE46317).

### 2.4 Gene ontology (GO) analysis

GO enriched cellular processes in the differentially regulated gene sets were determined using the Generic GO Term Finder (Boyle et al., 2004). The complete gene list (excluding absent probes) from which the differentially regulated genes were identified was used as the background population.

## Appendix Supplementary material

Table S1 Genes with significantly increased expression ≥ 1.5 fold in repo-Gal4, UAS-HtlACT CNS tissue.

Table S2 Genes with significantly increased expression ≥ 1.5 fold in repo-Gal4, UAS-InR CNS tissue.

Table S3 Genes with significantly increased expression ≥ 1.5 fold in both repo-Gal4, UAS-HtlACT CNS tissue and repo-Gal4, UAS-InR CNS tissue.

Table S4 Genes with significantly increased expression ≥ 1.5 fold in repo-Gal4, UAS-InR CNS tissue, but not repo-Gal4, UAS-HtlACT CNS tissue.

Table S5 Genes with significantly decreased expression ≤ 1.5 fold in repo-Gal4, UAS-HtlACT CNS tissue.

Table S6 Genes with significantly decreased expression ≤ 1.5 fold in repo-Gal4, UAS-InR CNS tissue. 22

Table S7 Genes with significantly decreased expression ≤ 1.5 fold in both repo-Gal4, UAS-HtlACT and repo-Gal4, UAS-InR CNS tissue.

Table S8 GO analysis (cellular processes) of genes with significantly increased expression ≥1.5 fold in repo-Gal4, UAS-HtlACT CNS tissue. p-value ≤0.01.

Table S9 GO analysis (cellular processes) of genes with significantly increased expression ≥1.5 fold in repo-Gal4, UAS-InR CNS tissue. p-value ≤0.01.

Table S10 GO analysis (cellular processes) of genes with significantly decreased expression ≤1.5 fold in repo-Gal4, UAS-HtlACT CNS tissue. p-value ≤0.01.

Table S11 GO analysis (cellular processes) of genes with significantly decreased expression ≤1.5 fold in repo-Gal4, UAS-InR CNS tissue. p-value ≤0.01.
