# Supplementary material for: Glial enriched gene expression profiling identifies novel factors regulating the proliferation of specific glial subtypes in the Drosophila brain
Source: Gene Expr Patterns. 2014 Sep;16(1):61–8. doi: 10.1016/j.gep.2014.09.001 (PMC4222725; doi:10.1016/j.gep.2014.09.001)
Supplement: Table S8 — GO analysis (cellular processes) of genes with significantly increased expression ≥1.5 fold in repo-Gal4, UAS-HtlACT CNS tissue. p-value ≤0.01. [file mmc8.docx]

*Supplementary table S8. GO analysis (cellular processes) of genes with significantly increased expression ≥1.5 fold in repo-Gal4, UAS-Htl^ACT^ CNS tissue. p-value ≤0.01.*

| **Gene Ontology term** | **Cluster frequency** | **Genome frequency** | **Corrected P-value** | **FDR** | **False Positives** | **Genes annotated to the term** |
| --- | --- | --- | --- | --- | --- | --- |
| [single-organism metabolic process](http://amigo.geneontology.org/cgi-bin/amigo/go.cgi?view=details&query=GO:0044710) | 204 of 1000 genes, 20.4% | 862 of 7634 genes, 11.3% | 7.25e-18 | 0.00% | 0.00 | [CG13667](http://flybase.bio.indiana.edu/.bin/fbidq.html?CG13667), [CG9339](http://flybase.bio.indiana.edu/.bin/fbidq.html?CG9339), [Arc42](http://flybase.bio.indiana.edu/.bin/fbidq.html?Arc42), [CG9362](http://flybase.bio.indiana.edu/.bin/fbidq.html?CG9362), [Eip55E](http://flybase.bio.indiana.edu/.bin/fbidq.html?Eip55E), [Got1](http://flybase.bio.indiana.edu/.bin/fbidq.html?Got1), [GXIVsPLA2](http://flybase.bio.indiana.edu/.bin/fbidq.html?GXIVsPLA2), [Nmdmc](http://flybase.bio.indiana.edu/.bin/fbidq.html?Nmdmc), [CG7461](http://flybase.bio.indiana.edu/.bin/fbidq.html?CG7461), [Pect](http://flybase.bio.indiana.edu/.bin/fbidq.html?Pect), [Inos](http://flybase.bio.indiana.edu/.bin/fbidq.html?Inos), [CG12140](http://flybase.bio.indiana.edu/.bin/fbidq.html?CG12140), [CG9547](http://flybase.bio.indiana.edu/.bin/fbidq.html?CG9547), [ry](http://flybase.bio.indiana.edu/.bin/fbidq.html?ry), [CG9904](http://flybase.bio.indiana.edu/.bin/fbidq.html?CG9904), [Git](http://flybase.bio.indiana.edu/.bin/fbidq.html?Git), [ifc](http://flybase.bio.indiana.edu/.bin/fbidq.html?ifc), [Phm](http://flybase.bio.indiana.edu/.bin/fbidq.html?Phm), [CG31683](http://flybase.bio.indiana.edu/.bin/fbidq.html?CG31683), [CG30000](http://flybase.bio.indiana.edu/.bin/fbidq.html?CG30000), [Glycogenin](http://flybase.bio.indiana.edu/.bin/fbidq.html?Glycogenin), [CG31674](http://flybase.bio.indiana.edu/.bin/fbidq.html?CG31674), [CG17544](http://flybase.bio.indiana.edu/.bin/fbidq.html?CG17544), [dysb](http://flybase.bio.indiana.edu/.bin/fbidq.html?dysb), [Cyp9h1](http://flybase.bio.indiana.edu/.bin/fbidq.html?Cyp9h1), [Spn27A](http://flybase.bio.indiana.edu/.bin/fbidq.html?Spn27A), [Thiolase](http://flybase.bio.indiana.edu/.bin/fbidq.html?Thiolase),[CG9629](http://flybase.bio.indiana.edu/.bin/fbidq.html?CG9629), [CG17896](http://flybase.bio.indiana.edu/.bin/fbidq.html?CG17896), [CG4829](http://flybase.bio.indiana.edu/.bin/fbidq.html?CG4829), [CG1998](http://flybase.bio.indiana.edu/.bin/fbidq.html?CG1998), [Ace](http://flybase.bio.indiana.edu/.bin/fbidq.html?Ace), [l(1)G0334](http://flybase.bio.indiana.edu/.bin/fbidq.html?l(1)G0334), [CG31414](http://flybase.bio.indiana.edu/.bin/fbidq.html?CG31414), [CG12262](http://flybase.bio.indiana.edu/.bin/fbidq.html?CG12262), [Lsd-2](http://flybase.bio.indiana.edu/.bin/fbidq.html?Lsd-2), [GstD2](http://flybase.bio.indiana.edu/.bin/fbidq.html?GstD2), [Efr](http://flybase.bio.indiana.edu/.bin/fbidq.html?Efr), [CG9331](http://flybase.bio.indiana.edu/.bin/fbidq.html?CG9331), [CG14945](http://flybase.bio.indiana.edu/.bin/fbidq.html?CG14945), [scu](http://flybase.bio.indiana.edu/.bin/fbidq.html?scu), [CG9503](http://flybase.bio.indiana.edu/.bin/fbidq.html?CG9503), [GstE1](http://flybase.bio.indiana.edu/.bin/fbidq.html?GstE1), [yip2](http://flybase.bio.indiana.edu/.bin/fbidq.html?yip2), [CG10184](http://flybase.bio.indiana.edu/.bin/fbidq.html?CG10184), [CG6543](http://flybase.bio.indiana.edu/.bin/fbidq.html?CG6543), [CG2118](http://flybase.bio.indiana.edu/.bin/fbidq.html?CG2118), [CG1140](http://flybase.bio.indiana.edu/.bin/fbidq.html?CG1140), [CaBP1](http://flybase.bio.indiana.edu/.bin/fbidq.html?CaBP1), [CG5991](http://flybase.bio.indiana.edu/.bin/fbidq.html?CG5991), [GstD1](http://flybase.bio.indiana.edu/.bin/fbidq.html?GstD1), [Nrg](http://flybase.bio.indiana.edu/.bin/fbidq.html?Nrg), [Rac2](http://flybase.bio.indiana.edu/.bin/fbidq.html?Rac2), [Ced-12](http://flybase.bio.indiana.edu/.bin/fbidq.html?Ced-12), [CG3376](http://flybase.bio.indiana.edu/.bin/fbidq.html?CG3376),[CG5599](http://flybase.bio.indiana.edu/.bin/fbidq.html?CG5599), [Cpr](http://flybase.bio.indiana.edu/.bin/fbidq.html?Cpr), [CG14997](http://flybase.bio.indiana.edu/.bin/fbidq.html?CG14997), [CG10863](http://flybase.bio.indiana.edu/.bin/fbidq.html?CG10863), [CG6142](http://flybase.bio.indiana.edu/.bin/fbidq.html?CG6142), [Aprt](http://flybase.bio.indiana.edu/.bin/fbidq.html?Aprt), [Ppat-Dpck](http://flybase.bio.indiana.edu/.bin/fbidq.html?Ppat-Dpck), [kar](http://flybase.bio.indiana.edu/.bin/fbidq.html?kar), [ttv](http://flybase.bio.indiana.edu/.bin/fbidq.html?ttv), [CG30410](http://flybase.bio.indiana.edu/.bin/fbidq.html?CG30410), [Mtp](http://flybase.bio.indiana.edu/.bin/fbidq.html?Mtp), [CG6726](http://flybase.bio.indiana.edu/.bin/fbidq.html?CG6726), [CG10361](http://flybase.bio.indiana.edu/.bin/fbidq.html?CG10361), [CG30499](http://flybase.bio.indiana.edu/.bin/fbidq.html?CG30499), [Gclc](http://flybase.bio.indiana.edu/.bin/fbidq.html?Gclc), [Sp7](http://flybase.bio.indiana.edu/.bin/fbidq.html?Sp7), [Fdh](http://flybase.bio.indiana.edu/.bin/fbidq.html?Fdh), [dob](http://flybase.bio.indiana.edu/.bin/fbidq.html?dob), [CG5167](http://flybase.bio.indiana.edu/.bin/fbidq.html?CG5167), [CG31673](http://flybase.bio.indiana.edu/.bin/fbidq.html?CG31673), [foxo](http://flybase.bio.indiana.edu/.bin/fbidq.html?foxo), [Cyp12b2](http://flybase.bio.indiana.edu/.bin/fbidq.html?Cyp12b2), [GstD9](http://flybase.bio.indiana.edu/.bin/fbidq.html?GstD9), [Hmgs](http://flybase.bio.indiana.edu/.bin/fbidq.html?Hmgs), [CG3940](http://flybase.bio.indiana.edu/.bin/fbidq.html?CG3940), [Irc](http://flybase.bio.indiana.edu/.bin/fbidq.html?Irc), [G-ialpha65A](http://flybase.bio.indiana.edu/.bin/fbidq.html?G-ialpha65A), [CG31915](http://flybase.bio.indiana.edu/.bin/fbidq.html?CG31915), [CG10383](http://flybase.bio.indiana.edu/.bin/fbidq.html?CG10383),[CG10932](http://flybase.bio.indiana.edu/.bin/fbidq.html?CG10932), [ox](http://flybase.bio.indiana.edu/.bin/fbidq.html?ox), [Npc2b](http://flybase.bio.indiana.edu/.bin/fbidq.html?Npc2b), [CG16936](http://flybase.bio.indiana.edu/.bin/fbidq.html?CG16936), [frj](http://flybase.bio.indiana.edu/.bin/fbidq.html?frj), [CG10627](http://flybase.bio.indiana.edu/.bin/fbidq.html?CG10627), [PhKgamma](http://flybase.bio.indiana.edu/.bin/fbidq.html?PhKgamma), [CG3621](http://flybase.bio.indiana.edu/.bin/fbidq.html?CG3621), [Npc1a](http://flybase.bio.indiana.edu/.bin/fbidq.html?Npc1a), [Cyp4g15](http://flybase.bio.indiana.edu/.bin/fbidq.html?Cyp4g15), [GstE3](http://flybase.bio.indiana.edu/.bin/fbidq.html?GstE3), [Cyp12e1](http://flybase.bio.indiana.edu/.bin/fbidq.html?Cyp12e1), [RhoL](http://flybase.bio.indiana.edu/.bin/fbidq.html?RhoL), [CG17333](http://flybase.bio.indiana.edu/.bin/fbidq.html?CG17333), [CG7737](http://flybase.bio.indiana.edu/.bin/fbidq.html?CG7737), [CG7149](http://flybase.bio.indiana.edu/.bin/fbidq.html?CG7149), [CG10512](http://flybase.bio.indiana.edu/.bin/fbidq.html?CG10512), [CG3523](http://flybase.bio.indiana.edu/.bin/fbidq.html?CG3523), [CG42271](http://flybase.bio.indiana.edu/.bin/fbidq.html?CG42271), [CG3609](http://flybase.bio.indiana.edu/.bin/fbidq.html?CG3609), [Spn28D](http://flybase.bio.indiana.edu/.bin/fbidq.html?Spn28D), [Atpalpha](http://flybase.bio.indiana.edu/.bin/fbidq.html?Atpalpha), [CG17026](http://flybase.bio.indiana.edu/.bin/fbidq.html?CG17026), [Oat](http://flybase.bio.indiana.edu/.bin/fbidq.html?Oat), [CG30005](http://flybase.bio.indiana.edu/.bin/fbidq.html?CG30005), [CG1544](http://flybase.bio.indiana.edu/.bin/fbidq.html?CG1544),[CG13200](http://flybase.bio.indiana.edu/.bin/fbidq.html?CG13200), [CG5362](http://flybase.bio.indiana.edu/.bin/fbidq.html?CG5362), [cert](http://flybase.bio.indiana.edu/.bin/fbidq.html?cert), [dare](http://flybase.bio.indiana.edu/.bin/fbidq.html?dare), [Npc2a](http://flybase.bio.indiana.edu/.bin/fbidq.html?Npc2a), [sktl](http://flybase.bio.indiana.edu/.bin/fbidq.html?sktl), [CG31523](http://flybase.bio.indiana.edu/.bin/fbidq.html?CG31523), [CG3590](http://flybase.bio.indiana.edu/.bin/fbidq.html?CG3590), [Cyp6v1](http://flybase.bio.indiana.edu/.bin/fbidq.html?Cyp6v1), [CG11255](http://flybase.bio.indiana.edu/.bin/fbidq.html?CG11255), [pyd3](http://flybase.bio.indiana.edu/.bin/fbidq.html?pyd3), [HLH106](http://flybase.bio.indiana.edu/.bin/fbidq.html?HLH106), [CG17029](http://flybase.bio.indiana.edu/.bin/fbidq.html?CG17029), [asparagine-synthetase](http://flybase.bio.indiana.edu/.bin/fbidq.html?asparagine-synthetase), [CG11784](http://flybase.bio.indiana.edu/.bin/fbidq.html?CG11784), [lace](http://flybase.bio.indiana.edu/.bin/fbidq.html?lace), [Adk3](http://flybase.bio.indiana.edu/.bin/fbidq.html?Adk3), [w](http://flybase.bio.indiana.edu/.bin/fbidq.html?w), [GstE6](http://flybase.bio.indiana.edu/.bin/fbidq.html?GstE6), [IP3K2](http://flybase.bio.indiana.edu/.bin/fbidq.html?IP3K2), [Ssadh](http://flybase.bio.indiana.edu/.bin/fbidq.html?Ssadh), [ade2](http://flybase.bio.indiana.edu/.bin/fbidq.html?ade2), [CG6638](http://flybase.bio.indiana.edu/.bin/fbidq.html?CG6638), [egr](http://flybase.bio.indiana.edu/.bin/fbidq.html?egr), [CG8360](http://flybase.bio.indiana.edu/.bin/fbidq.html?CG8360), [Pde9](http://flybase.bio.indiana.edu/.bin/fbidq.html?Pde9), [santa-maria](http://flybase.bio.indiana.edu/.bin/fbidq.html?santa-maria),[CG8417](http://flybase.bio.indiana.edu/.bin/fbidq.html?CG8417), [CG1702](http://flybase.bio.indiana.edu/.bin/fbidq.html?CG1702), [CG3267](http://flybase.bio.indiana.edu/.bin/fbidq.html?CG3267), [fu12](http://flybase.bio.indiana.edu/.bin/fbidq.html?fu12), [CG6045](http://flybase.bio.indiana.edu/.bin/fbidq.html?CG6045), [CG10688](http://flybase.bio.indiana.edu/.bin/fbidq.html?CG10688), [PH4alphaEFB](http://flybase.bio.indiana.edu/.bin/fbidq.html?PH4alphaEFB), [Hnf4](http://flybase.bio.indiana.edu/.bin/fbidq.html?Hnf4), [CG18135](http://flybase.bio.indiana.edu/.bin/fbidq.html?CG18135), [Coprox](http://flybase.bio.indiana.edu/.bin/fbidq.html?Coprox), [CG11015](http://flybase.bio.indiana.edu/.bin/fbidq.html?CG11015), [CG17292](http://flybase.bio.indiana.edu/.bin/fbidq.html?CG17292), [Gtp-bp](http://flybase.bio.indiana.edu/.bin/fbidq.html?Gtp-bp), [Pgd](http://flybase.bio.indiana.edu/.bin/fbidq.html?Pgd), [CG5895](http://flybase.bio.indiana.edu/.bin/fbidq.html?CG5895), [CG4389](http://flybase.bio.indiana.edu/.bin/fbidq.html?CG4389), [CG4860](http://flybase.bio.indiana.edu/.bin/fbidq.html?CG4860), [CG32068](http://flybase.bio.indiana.edu/.bin/fbidq.html?CG32068), [zetaCOP](http://flybase.bio.indiana.edu/.bin/fbidq.html?zetaCOP), [Sucb](http://flybase.bio.indiana.edu/.bin/fbidq.html?Sucb), [CG10399](http://flybase.bio.indiana.edu/.bin/fbidq.html?CG10399), [CG10157](http://flybase.bio.indiana.edu/.bin/fbidq.html?CG10157), [Aats-cys](http://flybase.bio.indiana.edu/.bin/fbidq.html?Aats-cys), [CG5508](http://flybase.bio.indiana.edu/.bin/fbidq.html?CG5508), [CG4306](http://flybase.bio.indiana.edu/.bin/fbidq.html?CG4306),[Cyp6d4](http://flybase.bio.indiana.edu/.bin/fbidq.html?Cyp6d4), [Vha68-2](http://flybase.bio.indiana.edu/.bin/fbidq.html?Vha68-2), [GstE2](http://flybase.bio.indiana.edu/.bin/fbidq.html?GstE2), [CG9302](http://flybase.bio.indiana.edu/.bin/fbidq.html?CG9302), [Cyp6t1](http://flybase.bio.indiana.edu/.bin/fbidq.html?Cyp6t1), [CG1673](http://flybase.bio.indiana.edu/.bin/fbidq.html?CG1673), [CG17224](http://flybase.bio.indiana.edu/.bin/fbidq.html?CG17224), [CG15093](http://flybase.bio.indiana.edu/.bin/fbidq.html?CG15093), [betaTub60D](http://flybase.bio.indiana.edu/.bin/fbidq.html?betaTub60D), [Mical](http://flybase.bio.indiana.edu/.bin/fbidq.html?Mical), [CG8525](http://flybase.bio.indiana.edu/.bin/fbidq.html?CG8525), [CG4585](http://flybase.bio.indiana.edu/.bin/fbidq.html?CG4585), [CG4670](http://flybase.bio.indiana.edu/.bin/fbidq.html?CG4670), [Acox57D-p](http://flybase.bio.indiana.edu/.bin/fbidq.html?Acox57D-p), [ACC](http://flybase.bio.indiana.edu/.bin/fbidq.html?ACC), [Lip4](http://flybase.bio.indiana.edu/.bin/fbidq.html?Lip4), [CG7997](http://flybase.bio.indiana.edu/.bin/fbidq.html?CG7997), [Sodh-2](http://flybase.bio.indiana.edu/.bin/fbidq.html?Sodh-2), [CG11883](http://flybase.bio.indiana.edu/.bin/fbidq.html?CG11883), [CDase](http://flybase.bio.indiana.edu/.bin/fbidq.html?CDase), [Aldh-III](http://flybase.bio.indiana.edu/.bin/fbidq.html?Aldh-III), [CG7433](http://flybase.bio.indiana.edu/.bin/fbidq.html?CG7433), [CG6084](http://flybase.bio.indiana.edu/.bin/fbidq.html?CG6084), [Gpdh](http://flybase.bio.indiana.edu/.bin/fbidq.html?Gpdh), [betaTub97EF](http://flybase.bio.indiana.edu/.bin/fbidq.html?betaTub97EF), [mdy](http://flybase.bio.indiana.edu/.bin/fbidq.html?mdy),[desat1](http://flybase.bio.indiana.edu/.bin/fbidq.html?desat1), [CG6074](http://flybase.bio.indiana.edu/.bin/fbidq.html?CG6074), [ade5](http://flybase.bio.indiana.edu/.bin/fbidq.html?ade5), [CG30104](http://flybase.bio.indiana.edu/.bin/fbidq.html?CG30104), [Prat](http://flybase.bio.indiana.edu/.bin/fbidq.html?Prat), [Tal](http://flybase.bio.indiana.edu/.bin/fbidq.html?Tal), [mbc](http://flybase.bio.indiana.edu/.bin/fbidq.html?mbc), [CG9436](http://flybase.bio.indiana.edu/.bin/fbidq.html?CG9436), [CG8709](http://flybase.bio.indiana.edu/.bin/fbidq.html?CG8709), [CG3835](http://flybase.bio.indiana.edu/.bin/fbidq.html?CG3835), [CG18547](http://flybase.bio.indiana.edu/.bin/fbidq.html?CG18547), [CG3902](http://flybase.bio.indiana.edu/.bin/fbidq.html?CG3902), [GstS1](http://flybase.bio.indiana.edu/.bin/fbidq.html?GstS1), [CG10639](http://flybase.bio.indiana.edu/.bin/fbidq.html?CG10639), [Pax](http://flybase.bio.indiana.edu/.bin/fbidq.html?Pax), [GstE7](http://flybase.bio.indiana.edu/.bin/fbidq.html?GstE7) |
| [oxidation-reduction process](http://amigo.geneontology.org/cgi-bin/amigo/go.cgi?view=details&query=GO:0055114) | 82 of 1000 genes, 8.2% | 273 of 7634 genes, 3.6% | 2.34e-11 | 0.00% | 0.00 | [CG13667](http://flybase.bio.indiana.edu/.bin/fbidq.html?CG13667), [CG1544](http://flybase.bio.indiana.edu/.bin/fbidq.html?CG1544), [Arc42](http://flybase.bio.indiana.edu/.bin/fbidq.html?Arc42), [CG13200](http://flybase.bio.indiana.edu/.bin/fbidq.html?CG13200), [CG5362](http://flybase.bio.indiana.edu/.bin/fbidq.html?CG5362), [Nmdmc](http://flybase.bio.indiana.edu/.bin/fbidq.html?Nmdmc), [dare](http://flybase.bio.indiana.edu/.bin/fbidq.html?dare), [CG7461](http://flybase.bio.indiana.edu/.bin/fbidq.html?CG7461), [CG12140](http://flybase.bio.indiana.edu/.bin/fbidq.html?CG12140), [CG9547](http://flybase.bio.indiana.edu/.bin/fbidq.html?CG9547), [ry](http://flybase.bio.indiana.edu/.bin/fbidq.html?ry), [ifc](http://flybase.bio.indiana.edu/.bin/fbidq.html?ifc), [Cyp6v1](http://flybase.bio.indiana.edu/.bin/fbidq.html?Cyp6v1), [Phm](http://flybase.bio.indiana.edu/.bin/fbidq.html?Phm), [Glycogenin](http://flybase.bio.indiana.edu/.bin/fbidq.html?Glycogenin), [CG31674](http://flybase.bio.indiana.edu/.bin/fbidq.html?CG31674), [CG17544](http://flybase.bio.indiana.edu/.bin/fbidq.html?CG17544), [Cyp9h1](http://flybase.bio.indiana.edu/.bin/fbidq.html?Cyp9h1), [Thiolase](http://flybase.bio.indiana.edu/.bin/fbidq.html?Thiolase), [CG9629](http://flybase.bio.indiana.edu/.bin/fbidq.html?CG9629), [CG17896](http://flybase.bio.indiana.edu/.bin/fbidq.html?CG17896), [Ssadh](http://flybase.bio.indiana.edu/.bin/fbidq.html?Ssadh), [CG1998](http://flybase.bio.indiana.edu/.bin/fbidq.html?CG1998), [l(1)G0334](http://flybase.bio.indiana.edu/.bin/fbidq.html?l(1)G0334), [CG12262](http://flybase.bio.indiana.edu/.bin/fbidq.html?CG12262), [CG9331](http://flybase.bio.indiana.edu/.bin/fbidq.html?CG9331),[CG6638](http://flybase.bio.indiana.edu/.bin/fbidq.html?CG6638), [CG9503](http://flybase.bio.indiana.edu/.bin/fbidq.html?CG9503), [CG6045](http://flybase.bio.indiana.edu/.bin/fbidq.html?CG6045), [yip2](http://flybase.bio.indiana.edu/.bin/fbidq.html?yip2), [PH4alphaEFB](http://flybase.bio.indiana.edu/.bin/fbidq.html?PH4alphaEFB), [CG6543](http://flybase.bio.indiana.edu/.bin/fbidq.html?CG6543), [Hnf4](http://flybase.bio.indiana.edu/.bin/fbidq.html?Hnf4), [Coprox](http://flybase.bio.indiana.edu/.bin/fbidq.html?Coprox), [CG11015](http://flybase.bio.indiana.edu/.bin/fbidq.html?CG11015), [CG5599](http://flybase.bio.indiana.edu/.bin/fbidq.html?CG5599), [Cpr](http://flybase.bio.indiana.edu/.bin/fbidq.html?Cpr), [CG14997](http://flybase.bio.indiana.edu/.bin/fbidq.html?CG14997), [Pgd](http://flybase.bio.indiana.edu/.bin/fbidq.html?Pgd), [CG10863](http://flybase.bio.indiana.edu/.bin/fbidq.html?CG10863), [CG6142](http://flybase.bio.indiana.edu/.bin/fbidq.html?CG6142), [CG30410](http://flybase.bio.indiana.edu/.bin/fbidq.html?CG30410), [CG4389](http://flybase.bio.indiana.edu/.bin/fbidq.html?CG4389), [CG30499](http://flybase.bio.indiana.edu/.bin/fbidq.html?CG30499), [CG4860](http://flybase.bio.indiana.edu/.bin/fbidq.html?CG4860), [CG32068](http://flybase.bio.indiana.edu/.bin/fbidq.html?CG32068), [zetaCOP](http://flybase.bio.indiana.edu/.bin/fbidq.html?zetaCOP), [Sucb](http://flybase.bio.indiana.edu/.bin/fbidq.html?Sucb), [CG10157](http://flybase.bio.indiana.edu/.bin/fbidq.html?CG10157), [Cyp6d4](http://flybase.bio.indiana.edu/.bin/fbidq.html?Cyp6d4), [Cyp6t1](http://flybase.bio.indiana.edu/.bin/fbidq.html?Cyp6t1), [Fdh](http://flybase.bio.indiana.edu/.bin/fbidq.html?Fdh),[CG31673](http://flybase.bio.indiana.edu/.bin/fbidq.html?CG31673), [CG5167](http://flybase.bio.indiana.edu/.bin/fbidq.html?CG5167), [CG15093](http://flybase.bio.indiana.edu/.bin/fbidq.html?CG15093), [foxo](http://flybase.bio.indiana.edu/.bin/fbidq.html?foxo), [Cyp12b2](http://flybase.bio.indiana.edu/.bin/fbidq.html?Cyp12b2), [Irc](http://flybase.bio.indiana.edu/.bin/fbidq.html?Irc), [Mical](http://flybase.bio.indiana.edu/.bin/fbidq.html?Mical), [CG4670](http://flybase.bio.indiana.edu/.bin/fbidq.html?CG4670), [Acox57D-p](http://flybase.bio.indiana.edu/.bin/fbidq.html?Acox57D-p), [ox](http://flybase.bio.indiana.edu/.bin/fbidq.html?ox), [PhKgamma](http://flybase.bio.indiana.edu/.bin/fbidq.html?PhKgamma), [Sodh-2](http://flybase.bio.indiana.edu/.bin/fbidq.html?Sodh-2), [CG3621](http://flybase.bio.indiana.edu/.bin/fbidq.html?CG3621), [Cyp4g15](http://flybase.bio.indiana.edu/.bin/fbidq.html?Cyp4g15), [Aldh-III](http://flybase.bio.indiana.edu/.bin/fbidq.html?Aldh-III), [Cyp12e1](http://flybase.bio.indiana.edu/.bin/fbidq.html?Cyp12e1), [CG6084](http://flybase.bio.indiana.edu/.bin/fbidq.html?CG6084), [CG17333](http://flybase.bio.indiana.edu/.bin/fbidq.html?CG17333), [Gpdh](http://flybase.bio.indiana.edu/.bin/fbidq.html?Gpdh), [CG7737](http://flybase.bio.indiana.edu/.bin/fbidq.html?CG7737), [desat1](http://flybase.bio.indiana.edu/.bin/fbidq.html?desat1), [Tal](http://flybase.bio.indiana.edu/.bin/fbidq.html?Tal), [CG10512](http://flybase.bio.indiana.edu/.bin/fbidq.html?CG10512), [CG9436](http://flybase.bio.indiana.edu/.bin/fbidq.html?CG9436), [CG3523](http://flybase.bio.indiana.edu/.bin/fbidq.html?CG3523), [CG3609](http://flybase.bio.indiana.edu/.bin/fbidq.html?CG3609),[CG3835](http://flybase.bio.indiana.edu/.bin/fbidq.html?CG3835), [CG3902](http://flybase.bio.indiana.edu/.bin/fbidq.html?CG3902), [CG18547](http://flybase.bio.indiana.edu/.bin/fbidq.html?CG18547), [CG10639](http://flybase.bio.indiana.edu/.bin/fbidq.html?CG10639) |
| [small molecule metabolic process](http://amigo.geneontology.org/cgi-bin/amigo/go.cgi?view=details&query=GO:0044281) | 114 of 1000 genes, 11.4% | 458 of 7634 genes, 6.0% | 3.69e-10 | 0.00% | 0.00 | [CG9339](http://flybase.bio.indiana.edu/.bin/fbidq.html?CG9339), [CG9362](http://flybase.bio.indiana.edu/.bin/fbidq.html?CG9362), [Eip55E](http://flybase.bio.indiana.edu/.bin/fbidq.html?Eip55E), [Got1](http://flybase.bio.indiana.edu/.bin/fbidq.html?Got1), [CG5362](http://flybase.bio.indiana.edu/.bin/fbidq.html?CG5362), [Nmdmc](http://flybase.bio.indiana.edu/.bin/fbidq.html?Nmdmc), [dare](http://flybase.bio.indiana.edu/.bin/fbidq.html?dare), [Npc2a](http://flybase.bio.indiana.edu/.bin/fbidq.html?Npc2a), [Pect](http://flybase.bio.indiana.edu/.bin/fbidq.html?Pect), [Inos](http://flybase.bio.indiana.edu/.bin/fbidq.html?Inos), [CG12140](http://flybase.bio.indiana.edu/.bin/fbidq.html?CG12140), [CG9547](http://flybase.bio.indiana.edu/.bin/fbidq.html?CG9547), [ry](http://flybase.bio.indiana.edu/.bin/fbidq.html?ry), [CG31523](http://flybase.bio.indiana.edu/.bin/fbidq.html?CG31523), [CG3590](http://flybase.bio.indiana.edu/.bin/fbidq.html?CG3590), [Git](http://flybase.bio.indiana.edu/.bin/fbidq.html?Git), [ifc](http://flybase.bio.indiana.edu/.bin/fbidq.html?ifc), [CG11255](http://flybase.bio.indiana.edu/.bin/fbidq.html?CG11255), [pyd3](http://flybase.bio.indiana.edu/.bin/fbidq.html?pyd3), [CG30000](http://flybase.bio.indiana.edu/.bin/fbidq.html?CG30000), [CG17544](http://flybase.bio.indiana.edu/.bin/fbidq.html?CG17544), [dysb](http://flybase.bio.indiana.edu/.bin/fbidq.html?dysb), [HLH106](http://flybase.bio.indiana.edu/.bin/fbidq.html?HLH106), [CG11784](http://flybase.bio.indiana.edu/.bin/fbidq.html?CG11784), [asparagine-synthetase](http://flybase.bio.indiana.edu/.bin/fbidq.html?asparagine-synthetase), [Adk3](http://flybase.bio.indiana.edu/.bin/fbidq.html?Adk3), [Thiolase](http://flybase.bio.indiana.edu/.bin/fbidq.html?Thiolase),[CG17896](http://flybase.bio.indiana.edu/.bin/fbidq.html?CG17896), [CG4829](http://flybase.bio.indiana.edu/.bin/fbidq.html?CG4829), [GstE6](http://flybase.bio.indiana.edu/.bin/fbidq.html?GstE6), [IP3K2](http://flybase.bio.indiana.edu/.bin/fbidq.html?IP3K2), [Ssadh](http://flybase.bio.indiana.edu/.bin/fbidq.html?Ssadh), [CG1998](http://flybase.bio.indiana.edu/.bin/fbidq.html?CG1998), [Ace](http://flybase.bio.indiana.edu/.bin/fbidq.html?Ace), [l(1)G0334](http://flybase.bio.indiana.edu/.bin/fbidq.html?l(1)G0334), [CG12262](http://flybase.bio.indiana.edu/.bin/fbidq.html?CG12262), [ade2](http://flybase.bio.indiana.edu/.bin/fbidq.html?ade2), [GstD2](http://flybase.bio.indiana.edu/.bin/fbidq.html?GstD2), [Efr](http://flybase.bio.indiana.edu/.bin/fbidq.html?Efr), [CG6638](http://flybase.bio.indiana.edu/.bin/fbidq.html?CG6638), [CG8360](http://flybase.bio.indiana.edu/.bin/fbidq.html?CG8360), [scu](http://flybase.bio.indiana.edu/.bin/fbidq.html?scu), [Pde9](http://flybase.bio.indiana.edu/.bin/fbidq.html?Pde9), [CG8417](http://flybase.bio.indiana.edu/.bin/fbidq.html?CG8417), [CG1702](http://flybase.bio.indiana.edu/.bin/fbidq.html?CG1702), [CG9503](http://flybase.bio.indiana.edu/.bin/fbidq.html?CG9503), [CG3267](http://flybase.bio.indiana.edu/.bin/fbidq.html?CG3267), [GstE1](http://flybase.bio.indiana.edu/.bin/fbidq.html?GstE1), [CG10688](http://flybase.bio.indiana.edu/.bin/fbidq.html?CG10688), [yip2](http://flybase.bio.indiana.edu/.bin/fbidq.html?yip2), [PH4alphaEFB](http://flybase.bio.indiana.edu/.bin/fbidq.html?PH4alphaEFB), [CG10184](http://flybase.bio.indiana.edu/.bin/fbidq.html?CG10184), [CG6543](http://flybase.bio.indiana.edu/.bin/fbidq.html?CG6543), [CG18135](http://flybase.bio.indiana.edu/.bin/fbidq.html?CG18135),[CG2118](http://flybase.bio.indiana.edu/.bin/fbidq.html?CG2118), [CG1140](http://flybase.bio.indiana.edu/.bin/fbidq.html?CG1140), [CaBP1](http://flybase.bio.indiana.edu/.bin/fbidq.html?CaBP1), [GstD1](http://flybase.bio.indiana.edu/.bin/fbidq.html?GstD1), [Ced-12](http://flybase.bio.indiana.edu/.bin/fbidq.html?Ced-12), [CG3376](http://flybase.bio.indiana.edu/.bin/fbidq.html?CG3376), [Gtp-bp](http://flybase.bio.indiana.edu/.bin/fbidq.html?Gtp-bp), [Pgd](http://flybase.bio.indiana.edu/.bin/fbidq.html?Pgd), [CG6142](http://flybase.bio.indiana.edu/.bin/fbidq.html?CG6142), [Aprt](http://flybase.bio.indiana.edu/.bin/fbidq.html?Aprt), [Ppat-Dpck](http://flybase.bio.indiana.edu/.bin/fbidq.html?Ppat-Dpck), [ttv](http://flybase.bio.indiana.edu/.bin/fbidq.html?ttv), [CG30410](http://flybase.bio.indiana.edu/.bin/fbidq.html?CG30410), [CG4389](http://flybase.bio.indiana.edu/.bin/fbidq.html?CG4389), [CG6726](http://flybase.bio.indiana.edu/.bin/fbidq.html?CG6726), [CG10361](http://flybase.bio.indiana.edu/.bin/fbidq.html?CG10361), [CG30499](http://flybase.bio.indiana.edu/.bin/fbidq.html?CG30499), [Gclc](http://flybase.bio.indiana.edu/.bin/fbidq.html?Gclc), [CG10399](http://flybase.bio.indiana.edu/.bin/fbidq.html?CG10399), [Aats-cys](http://flybase.bio.indiana.edu/.bin/fbidq.html?Aats-cys), [CG4306](http://flybase.bio.indiana.edu/.bin/fbidq.html?CG4306), [Vha68-2](http://flybase.bio.indiana.edu/.bin/fbidq.html?Vha68-2), [GstE2](http://flybase.bio.indiana.edu/.bin/fbidq.html?GstE2), [CG9302](http://flybase.bio.indiana.edu/.bin/fbidq.html?CG9302), [Fdh](http://flybase.bio.indiana.edu/.bin/fbidq.html?Fdh), [CG17224](http://flybase.bio.indiana.edu/.bin/fbidq.html?CG17224), [CG1673](http://flybase.bio.indiana.edu/.bin/fbidq.html?CG1673),[CG15093](http://flybase.bio.indiana.edu/.bin/fbidq.html?CG15093), [betaTub60D](http://flybase.bio.indiana.edu/.bin/fbidq.html?betaTub60D), [GstD9](http://flybase.bio.indiana.edu/.bin/fbidq.html?GstD9), [CG3940](http://flybase.bio.indiana.edu/.bin/fbidq.html?CG3940), [G-ialpha65A](http://flybase.bio.indiana.edu/.bin/fbidq.html?G-ialpha65A), [Mical](http://flybase.bio.indiana.edu/.bin/fbidq.html?Mical), [CG8525](http://flybase.bio.indiana.edu/.bin/fbidq.html?CG8525), [CG10932](http://flybase.bio.indiana.edu/.bin/fbidq.html?CG10932), [Acox57D-p](http://flybase.bio.indiana.edu/.bin/fbidq.html?Acox57D-p), [ACC](http://flybase.bio.indiana.edu/.bin/fbidq.html?ACC), [Npc2b](http://flybase.bio.indiana.edu/.bin/fbidq.html?Npc2b), [CG16936](http://flybase.bio.indiana.edu/.bin/fbidq.html?CG16936), [CG10627](http://flybase.bio.indiana.edu/.bin/fbidq.html?CG10627), [CG11883](http://flybase.bio.indiana.edu/.bin/fbidq.html?CG11883), [Npc1a](http://flybase.bio.indiana.edu/.bin/fbidq.html?Npc1a), [GstE3](http://flybase.bio.indiana.edu/.bin/fbidq.html?GstE3), [CG7433](http://flybase.bio.indiana.edu/.bin/fbidq.html?CG7433), [CG17333](http://flybase.bio.indiana.edu/.bin/fbidq.html?CG17333), [betaTub97EF](http://flybase.bio.indiana.edu/.bin/fbidq.html?betaTub97EF), [desat1](http://flybase.bio.indiana.edu/.bin/fbidq.html?desat1), [CG6074](http://flybase.bio.indiana.edu/.bin/fbidq.html?CG6074), [ade5](http://flybase.bio.indiana.edu/.bin/fbidq.html?ade5), [CG30104](http://flybase.bio.indiana.edu/.bin/fbidq.html?CG30104), [Prat](http://flybase.bio.indiana.edu/.bin/fbidq.html?Prat), [Tal](http://flybase.bio.indiana.edu/.bin/fbidq.html?Tal),[mbc](http://flybase.bio.indiana.edu/.bin/fbidq.html?mbc), [CG8709](http://flybase.bio.indiana.edu/.bin/fbidq.html?CG8709), [Atpalpha](http://flybase.bio.indiana.edu/.bin/fbidq.html?Atpalpha), [GstS1](http://flybase.bio.indiana.edu/.bin/fbidq.html?GstS1), [Pax](http://flybase.bio.indiana.edu/.bin/fbidq.html?Pax), [GstE7](http://flybase.bio.indiana.edu/.bin/fbidq.html?GstE7), [CG30005](http://flybase.bio.indiana.edu/.bin/fbidq.html?CG30005), [Oat](http://flybase.bio.indiana.edu/.bin/fbidq.html?Oat) |
| [carboxylic acid metabolic process](http://amigo.geneontology.org/cgi-bin/amigo/go.cgi?view=details&query=GO:0019752) | 58 of 1000 genes, 5.8% | 182 of 7634 genes, 2.4% | 1.38e-08 | 0.00% | 0.00 | [Gclc](http://flybase.bio.indiana.edu/.bin/fbidq.html?Gclc), [CG9362](http://flybase.bio.indiana.edu/.bin/fbidq.html?CG9362), [Eip55E](http://flybase.bio.indiana.edu/.bin/fbidq.html?Eip55E), [Got1](http://flybase.bio.indiana.edu/.bin/fbidq.html?Got1), [CG10399](http://flybase.bio.indiana.edu/.bin/fbidq.html?CG10399), [CG4306](http://flybase.bio.indiana.edu/.bin/fbidq.html?CG4306), [CG5362](http://flybase.bio.indiana.edu/.bin/fbidq.html?CG5362), [Aats-cys](http://flybase.bio.indiana.edu/.bin/fbidq.html?Aats-cys), [Nmdmc](http://flybase.bio.indiana.edu/.bin/fbidq.html?Nmdmc), [GstE2](http://flybase.bio.indiana.edu/.bin/fbidq.html?GstE2), [CG12140](http://flybase.bio.indiana.edu/.bin/fbidq.html?CG12140), [CG9547](http://flybase.bio.indiana.edu/.bin/fbidq.html?CG9547), [ry](http://flybase.bio.indiana.edu/.bin/fbidq.html?ry), [CG1673](http://flybase.bio.indiana.edu/.bin/fbidq.html?CG1673), [CG31523](http://flybase.bio.indiana.edu/.bin/fbidq.html?CG31523), [ifc](http://flybase.bio.indiana.edu/.bin/fbidq.html?ifc), [CG15093](http://flybase.bio.indiana.edu/.bin/fbidq.html?CG15093), [CG30000](http://flybase.bio.indiana.edu/.bin/fbidq.html?CG30000), [CG17544](http://flybase.bio.indiana.edu/.bin/fbidq.html?CG17544), [GstD9](http://flybase.bio.indiana.edu/.bin/fbidq.html?GstD9), [HLH106](http://flybase.bio.indiana.edu/.bin/fbidq.html?HLH106), [CG11784](http://flybase.bio.indiana.edu/.bin/fbidq.html?CG11784), [asparagine-synthetase](http://flybase.bio.indiana.edu/.bin/fbidq.html?asparagine-synthetase), [Thiolase](http://flybase.bio.indiana.edu/.bin/fbidq.html?Thiolase),[CG17896](http://flybase.bio.indiana.edu/.bin/fbidq.html?CG17896), [CG4829](http://flybase.bio.indiana.edu/.bin/fbidq.html?CG4829), [GstE6](http://flybase.bio.indiana.edu/.bin/fbidq.html?GstE6), [CG10932](http://flybase.bio.indiana.edu/.bin/fbidq.html?CG10932), [Acox57D-p](http://flybase.bio.indiana.edu/.bin/fbidq.html?Acox57D-p), [ACC](http://flybase.bio.indiana.edu/.bin/fbidq.html?ACC), [Ssadh](http://flybase.bio.indiana.edu/.bin/fbidq.html?Ssadh), [CG1998](http://flybase.bio.indiana.edu/.bin/fbidq.html?CG1998), [l(1)G0334](http://flybase.bio.indiana.edu/.bin/fbidq.html?l(1)G0334), [CG16936](http://flybase.bio.indiana.edu/.bin/fbidq.html?CG16936), [CG12262](http://flybase.bio.indiana.edu/.bin/fbidq.html?CG12262), [GstD2](http://flybase.bio.indiana.edu/.bin/fbidq.html?GstD2), [CG6638](http://flybase.bio.indiana.edu/.bin/fbidq.html?CG6638), [scu](http://flybase.bio.indiana.edu/.bin/fbidq.html?scu), [GstE3](http://flybase.bio.indiana.edu/.bin/fbidq.html?GstE3), [CG7433](http://flybase.bio.indiana.edu/.bin/fbidq.html?CG7433), [CG1702](http://flybase.bio.indiana.edu/.bin/fbidq.html?CG1702), [CG3267](http://flybase.bio.indiana.edu/.bin/fbidq.html?CG3267), [GstE1](http://flybase.bio.indiana.edu/.bin/fbidq.html?GstE1), [yip2](http://flybase.bio.indiana.edu/.bin/fbidq.html?yip2), [PH4alphaEFB](http://flybase.bio.indiana.edu/.bin/fbidq.html?PH4alphaEFB), [CG10184](http://flybase.bio.indiana.edu/.bin/fbidq.html?CG10184), [CG6543](http://flybase.bio.indiana.edu/.bin/fbidq.html?CG6543), [desat1](http://flybase.bio.indiana.edu/.bin/fbidq.html?desat1), [CG2118](http://flybase.bio.indiana.edu/.bin/fbidq.html?CG2118),[GstD1](http://flybase.bio.indiana.edu/.bin/fbidq.html?GstD1), [CG8709](http://flybase.bio.indiana.edu/.bin/fbidq.html?CG8709), [CG4389](http://flybase.bio.indiana.edu/.bin/fbidq.html?CG4389), [GstS1](http://flybase.bio.indiana.edu/.bin/fbidq.html?GstS1), [CG6726](http://flybase.bio.indiana.edu/.bin/fbidq.html?CG6726), [CG10361](http://flybase.bio.indiana.edu/.bin/fbidq.html?CG10361), [GstE7](http://flybase.bio.indiana.edu/.bin/fbidq.html?GstE7), [CG30005](http://flybase.bio.indiana.edu/.bin/fbidq.html?CG30005), [Oat](http://flybase.bio.indiana.edu/.bin/fbidq.html?Oat) |
| [organic acid metabolic process](http://amigo.geneontology.org/cgi-bin/amigo/go.cgi?view=details&query=GO:0006082) | 60 of 1000 genes, 6.0% | 194 of 7634 genes, 2.5% | 2.46e-08 | 0.00% | 0.00 | [Gclc](http://flybase.bio.indiana.edu/.bin/fbidq.html?Gclc), [CG9362](http://flybase.bio.indiana.edu/.bin/fbidq.html?CG9362), [Eip55E](http://flybase.bio.indiana.edu/.bin/fbidq.html?Eip55E), [Got1](http://flybase.bio.indiana.edu/.bin/fbidq.html?Got1), [CG10399](http://flybase.bio.indiana.edu/.bin/fbidq.html?CG10399), [CG4306](http://flybase.bio.indiana.edu/.bin/fbidq.html?CG4306), [CG5362](http://flybase.bio.indiana.edu/.bin/fbidq.html?CG5362), [Aats-cys](http://flybase.bio.indiana.edu/.bin/fbidq.html?Aats-cys), [Nmdmc](http://flybase.bio.indiana.edu/.bin/fbidq.html?Nmdmc), [GstE2](http://flybase.bio.indiana.edu/.bin/fbidq.html?GstE2), [CG12140](http://flybase.bio.indiana.edu/.bin/fbidq.html?CG12140), [CG9547](http://flybase.bio.indiana.edu/.bin/fbidq.html?CG9547), [ry](http://flybase.bio.indiana.edu/.bin/fbidq.html?ry), [CG1673](http://flybase.bio.indiana.edu/.bin/fbidq.html?CG1673), [CG31523](http://flybase.bio.indiana.edu/.bin/fbidq.html?CG31523), [ifc](http://flybase.bio.indiana.edu/.bin/fbidq.html?ifc), [CG15093](http://flybase.bio.indiana.edu/.bin/fbidq.html?CG15093), [CG30000](http://flybase.bio.indiana.edu/.bin/fbidq.html?CG30000), [CG17544](http://flybase.bio.indiana.edu/.bin/fbidq.html?CG17544), [GstD9](http://flybase.bio.indiana.edu/.bin/fbidq.html?GstD9), [HLH106](http://flybase.bio.indiana.edu/.bin/fbidq.html?HLH106), [CG11784](http://flybase.bio.indiana.edu/.bin/fbidq.html?CG11784), [asparagine-synthetase](http://flybase.bio.indiana.edu/.bin/fbidq.html?asparagine-synthetase), [Thiolase](http://flybase.bio.indiana.edu/.bin/fbidq.html?Thiolase),[CG17896](http://flybase.bio.indiana.edu/.bin/fbidq.html?CG17896), [CG4829](http://flybase.bio.indiana.edu/.bin/fbidq.html?CG4829), [GstE6](http://flybase.bio.indiana.edu/.bin/fbidq.html?GstE6), [CG10932](http://flybase.bio.indiana.edu/.bin/fbidq.html?CG10932), [Acox57D-p](http://flybase.bio.indiana.edu/.bin/fbidq.html?Acox57D-p), [ACC](http://flybase.bio.indiana.edu/.bin/fbidq.html?ACC), [Ssadh](http://flybase.bio.indiana.edu/.bin/fbidq.html?Ssadh), [CG1998](http://flybase.bio.indiana.edu/.bin/fbidq.html?CG1998), [l(1)G0334](http://flybase.bio.indiana.edu/.bin/fbidq.html?l(1)G0334), [CG16936](http://flybase.bio.indiana.edu/.bin/fbidq.html?CG16936), [CG12262](http://flybase.bio.indiana.edu/.bin/fbidq.html?CG12262), [GstD2](http://flybase.bio.indiana.edu/.bin/fbidq.html?GstD2), [Efr](http://flybase.bio.indiana.edu/.bin/fbidq.html?Efr), [CG6638](http://flybase.bio.indiana.edu/.bin/fbidq.html?CG6638), [scu](http://flybase.bio.indiana.edu/.bin/fbidq.html?scu), [GstE3](http://flybase.bio.indiana.edu/.bin/fbidq.html?GstE3), [CG7433](http://flybase.bio.indiana.edu/.bin/fbidq.html?CG7433), [CG1702](http://flybase.bio.indiana.edu/.bin/fbidq.html?CG1702), [CG3267](http://flybase.bio.indiana.edu/.bin/fbidq.html?CG3267), [GstE1](http://flybase.bio.indiana.edu/.bin/fbidq.html?GstE1), [yip2](http://flybase.bio.indiana.edu/.bin/fbidq.html?yip2), [PH4alphaEFB](http://flybase.bio.indiana.edu/.bin/fbidq.html?PH4alphaEFB), [CG10184](http://flybase.bio.indiana.edu/.bin/fbidq.html?CG10184), [CG6543](http://flybase.bio.indiana.edu/.bin/fbidq.html?CG6543), [desat1](http://flybase.bio.indiana.edu/.bin/fbidq.html?desat1), [CG2118](http://flybase.bio.indiana.edu/.bin/fbidq.html?CG2118),[GstD1](http://flybase.bio.indiana.edu/.bin/fbidq.html?GstD1), [CG8709](http://flybase.bio.indiana.edu/.bin/fbidq.html?CG8709), [ttv](http://flybase.bio.indiana.edu/.bin/fbidq.html?ttv), [CG4389](http://flybase.bio.indiana.edu/.bin/fbidq.html?CG4389), [GstS1](http://flybase.bio.indiana.edu/.bin/fbidq.html?GstS1), [CG6726](http://flybase.bio.indiana.edu/.bin/fbidq.html?CG6726), [CG10361](http://flybase.bio.indiana.edu/.bin/fbidq.html?CG10361), [GstE7](http://flybase.bio.indiana.edu/.bin/fbidq.html?GstE7), [CG30005](http://flybase.bio.indiana.edu/.bin/fbidq.html?CG30005), [Oat](http://flybase.bio.indiana.edu/.bin/fbidq.html?Oat) |
| [oxoacid metabolic process](http://amigo.geneontology.org/cgi-bin/amigo/go.cgi?view=details&query=GO:0043436) | 60 of 1000 genes, 6.0% | 194 of 7634 genes, 2.5% | 2.46e-08 | 0.00% | 0.00 | [Gclc](http://flybase.bio.indiana.edu/.bin/fbidq.html?Gclc), [CG9362](http://flybase.bio.indiana.edu/.bin/fbidq.html?CG9362), [Eip55E](http://flybase.bio.indiana.edu/.bin/fbidq.html?Eip55E), [Got1](http://flybase.bio.indiana.edu/.bin/fbidq.html?Got1), [CG10399](http://flybase.bio.indiana.edu/.bin/fbidq.html?CG10399), [CG4306](http://flybase.bio.indiana.edu/.bin/fbidq.html?CG4306), [CG5362](http://flybase.bio.indiana.edu/.bin/fbidq.html?CG5362), [Aats-cys](http://flybase.bio.indiana.edu/.bin/fbidq.html?Aats-cys), [Nmdmc](http://flybase.bio.indiana.edu/.bin/fbidq.html?Nmdmc), [GstE2](http://flybase.bio.indiana.edu/.bin/fbidq.html?GstE2), [CG12140](http://flybase.bio.indiana.edu/.bin/fbidq.html?CG12140), [CG9547](http://flybase.bio.indiana.edu/.bin/fbidq.html?CG9547), [ry](http://flybase.bio.indiana.edu/.bin/fbidq.html?ry), [CG1673](http://flybase.bio.indiana.edu/.bin/fbidq.html?CG1673), [CG31523](http://flybase.bio.indiana.edu/.bin/fbidq.html?CG31523), [ifc](http://flybase.bio.indiana.edu/.bin/fbidq.html?ifc), [CG15093](http://flybase.bio.indiana.edu/.bin/fbidq.html?CG15093), [CG30000](http://flybase.bio.indiana.edu/.bin/fbidq.html?CG30000), [CG17544](http://flybase.bio.indiana.edu/.bin/fbidq.html?CG17544), [GstD9](http://flybase.bio.indiana.edu/.bin/fbidq.html?GstD9), [HLH106](http://flybase.bio.indiana.edu/.bin/fbidq.html?HLH106), [CG11784](http://flybase.bio.indiana.edu/.bin/fbidq.html?CG11784), [asparagine-synthetase](http://flybase.bio.indiana.edu/.bin/fbidq.html?asparagine-synthetase), [Thiolase](http://flybase.bio.indiana.edu/.bin/fbidq.html?Thiolase),[CG17896](http://flybase.bio.indiana.edu/.bin/fbidq.html?CG17896), [CG4829](http://flybase.bio.indiana.edu/.bin/fbidq.html?CG4829), [GstE6](http://flybase.bio.indiana.edu/.bin/fbidq.html?GstE6), [CG10932](http://flybase.bio.indiana.edu/.bin/fbidq.html?CG10932), [Acox57D-p](http://flybase.bio.indiana.edu/.bin/fbidq.html?Acox57D-p), [ACC](http://flybase.bio.indiana.edu/.bin/fbidq.html?ACC), [Ssadh](http://flybase.bio.indiana.edu/.bin/fbidq.html?Ssadh), [CG1998](http://flybase.bio.indiana.edu/.bin/fbidq.html?CG1998), [l(1)G0334](http://flybase.bio.indiana.edu/.bin/fbidq.html?l(1)G0334), [CG16936](http://flybase.bio.indiana.edu/.bin/fbidq.html?CG16936), [CG12262](http://flybase.bio.indiana.edu/.bin/fbidq.html?CG12262), [GstD2](http://flybase.bio.indiana.edu/.bin/fbidq.html?GstD2), [Efr](http://flybase.bio.indiana.edu/.bin/fbidq.html?Efr), [CG6638](http://flybase.bio.indiana.edu/.bin/fbidq.html?CG6638), [scu](http://flybase.bio.indiana.edu/.bin/fbidq.html?scu), [GstE3](http://flybase.bio.indiana.edu/.bin/fbidq.html?GstE3), [CG7433](http://flybase.bio.indiana.edu/.bin/fbidq.html?CG7433), [CG1702](http://flybase.bio.indiana.edu/.bin/fbidq.html?CG1702), [CG3267](http://flybase.bio.indiana.edu/.bin/fbidq.html?CG3267), [GstE1](http://flybase.bio.indiana.edu/.bin/fbidq.html?GstE1), [yip2](http://flybase.bio.indiana.edu/.bin/fbidq.html?yip2), [PH4alphaEFB](http://flybase.bio.indiana.edu/.bin/fbidq.html?PH4alphaEFB), [CG10184](http://flybase.bio.indiana.edu/.bin/fbidq.html?CG10184), [CG6543](http://flybase.bio.indiana.edu/.bin/fbidq.html?CG6543), [desat1](http://flybase.bio.indiana.edu/.bin/fbidq.html?desat1), [CG2118](http://flybase.bio.indiana.edu/.bin/fbidq.html?CG2118),[GstD1](http://flybase.bio.indiana.edu/.bin/fbidq.html?GstD1), [CG8709](http://flybase.bio.indiana.edu/.bin/fbidq.html?CG8709), [ttv](http://flybase.bio.indiana.edu/.bin/fbidq.html?ttv), [CG4389](http://flybase.bio.indiana.edu/.bin/fbidq.html?CG4389), [GstS1](http://flybase.bio.indiana.edu/.bin/fbidq.html?GstS1), [CG6726](http://flybase.bio.indiana.edu/.bin/fbidq.html?CG6726), [CG10361](http://flybase.bio.indiana.edu/.bin/fbidq.html?CG10361), [GstE7](http://flybase.bio.indiana.edu/.bin/fbidq.html?GstE7), [CG30005](http://flybase.bio.indiana.edu/.bin/fbidq.html?CG30005), [Oat](http://flybase.bio.indiana.edu/.bin/fbidq.html?Oat) |
| [transmembrane transport](http://amigo.geneontology.org/cgi-bin/amigo/go.cgi?view=details&query=GO:0055085) | 66 of 1000 genes, 6.6% | 230 of 7634 genes, 3.0% | 9.48e-08 | 0.00% | 0.00 | [Sur](http://flybase.bio.indiana.edu/.bin/fbidq.html?Sur), [CG5130](http://flybase.bio.indiana.edu/.bin/fbidq.html?CG5130), [CG10960](http://flybase.bio.indiana.edu/.bin/fbidq.html?CG10960), [ClC-a](http://flybase.bio.indiana.edu/.bin/fbidq.html?ClC-a), [Ent2](http://flybase.bio.indiana.edu/.bin/fbidq.html?Ent2), [Ctr1A](http://flybase.bio.indiana.edu/.bin/fbidq.html?Ctr1A), [CG3036](http://flybase.bio.indiana.edu/.bin/fbidq.html?CG3036), [CG17119](http://flybase.bio.indiana.edu/.bin/fbidq.html?CG17119), [Orct2](http://flybase.bio.indiana.edu/.bin/fbidq.html?Orct2), [VhaPPA1-1](http://flybase.bio.indiana.edu/.bin/fbidq.html?VhaPPA1-1), [JhI-21](http://flybase.bio.indiana.edu/.bin/fbidq.html?JhI-21), [hoe1](http://flybase.bio.indiana.edu/.bin/fbidq.html?hoe1), [CG6672](http://flybase.bio.indiana.edu/.bin/fbidq.html?CG6672), [CG3168](http://flybase.bio.indiana.edu/.bin/fbidq.html?CG3168), [CG7342](http://flybase.bio.indiana.edu/.bin/fbidq.html?CG7342), [CG10444](http://flybase.bio.indiana.edu/.bin/fbidq.html?CG10444), [Indy](http://flybase.bio.indiana.edu/.bin/fbidq.html?Indy), [Efr](http://flybase.bio.indiana.edu/.bin/fbidq.html?Efr), [Mdr65](http://flybase.bio.indiana.edu/.bin/fbidq.html?Mdr65), [Itp-r83A](http://flybase.bio.indiana.edu/.bin/fbidq.html?Itp-r83A), [mnd](http://flybase.bio.indiana.edu/.bin/fbidq.html?mnd), [CG10226](http://flybase.bio.indiana.edu/.bin/fbidq.html?CG10226), [CG6812](http://flybase.bio.indiana.edu/.bin/fbidq.html?CG6812), [CG10006](http://flybase.bio.indiana.edu/.bin/fbidq.html?CG10006), [aralar1](http://flybase.bio.indiana.edu/.bin/fbidq.html?aralar1), [CG15438](http://flybase.bio.indiana.edu/.bin/fbidq.html?CG15438), [VhaAC39-1](http://flybase.bio.indiana.edu/.bin/fbidq.html?VhaAC39-1),[CG1607](http://flybase.bio.indiana.edu/.bin/fbidq.html?CG1607), [CG1213](http://flybase.bio.indiana.edu/.bin/fbidq.html?CG1213), [CG10069](http://flybase.bio.indiana.edu/.bin/fbidq.html?CG10069), [kar](http://flybase.bio.indiana.edu/.bin/fbidq.html?kar), [CG42235](http://flybase.bio.indiana.edu/.bin/fbidq.html?CG42235), [CG2893](http://flybase.bio.indiana.edu/.bin/fbidq.html?CG2893), [CG15094](http://flybase.bio.indiana.edu/.bin/fbidq.html?CG15094), [pyx](http://flybase.bio.indiana.edu/.bin/fbidq.html?pyx), [CG8596](http://flybase.bio.indiana.edu/.bin/fbidq.html?CG8596), [VhaM9.7-c](http://flybase.bio.indiana.edu/.bin/fbidq.html?VhaM9.7-c), [Sec61alpha](http://flybase.bio.indiana.edu/.bin/fbidq.html?Sec61alpha), [CG6356](http://flybase.bio.indiana.edu/.bin/fbidq.html?CG6356), [Vha68-2](http://flybase.bio.indiana.edu/.bin/fbidq.html?Vha68-2), [CG11163](http://flybase.bio.indiana.edu/.bin/fbidq.html?CG11163), [VhaM9.7-b](http://flybase.bio.indiana.edu/.bin/fbidq.html?VhaM9.7-b), [Orct](http://flybase.bio.indiana.edu/.bin/fbidq.html?Orct), [CG8602](http://flybase.bio.indiana.edu/.bin/fbidq.html?CG8602), [CG14511](http://flybase.bio.indiana.edu/.bin/fbidq.html?CG14511), [CG4288](http://flybase.bio.indiana.edu/.bin/fbidq.html?CG4288), [VhaM8.9](http://flybase.bio.indiana.edu/.bin/fbidq.html?VhaM8.9), [CG8790](http://flybase.bio.indiana.edu/.bin/fbidq.html?CG8790), [CG7816](http://flybase.bio.indiana.edu/.bin/fbidq.html?CG7816), [Picot](http://flybase.bio.indiana.edu/.bin/fbidq.html?Picot), [CG16700](http://flybase.bio.indiana.edu/.bin/fbidq.html?CG16700), [ClC-c](http://flybase.bio.indiana.edu/.bin/fbidq.html?ClC-c),[CG15890](http://flybase.bio.indiana.edu/.bin/fbidq.html?CG15890), [CG5802](http://flybase.bio.indiana.edu/.bin/fbidq.html?CG5802), [wtrw](http://flybase.bio.indiana.edu/.bin/fbidq.html?wtrw), [trpm](http://flybase.bio.indiana.edu/.bin/fbidq.html?trpm), [CG6723](http://flybase.bio.indiana.edu/.bin/fbidq.html?CG6723), [CG6293](http://flybase.bio.indiana.edu/.bin/fbidq.html?CG6293), [Vha100-2](http://flybase.bio.indiana.edu/.bin/fbidq.html?Vha100-2), [gb](http://flybase.bio.indiana.edu/.bin/fbidq.html?gb), [CG7777](http://flybase.bio.indiana.edu/.bin/fbidq.html?CG7777), [spin](http://flybase.bio.indiana.edu/.bin/fbidq.html?spin), [CG1628](http://flybase.bio.indiana.edu/.bin/fbidq.html?CG1628), [Ork1](http://flybase.bio.indiana.edu/.bin/fbidq.html?Ork1), [CG10420](http://flybase.bio.indiana.edu/.bin/fbidq.html?CG10420), [bib](http://flybase.bio.indiana.edu/.bin/fbidq.html?bib) |
| [lipid metabolic process](http://amigo.geneontology.org/cgi-bin/amigo/go.cgi?view=details&query=GO:0006629) | 59 of 1000 genes, 5.9% | 196 of 7634 genes, 2.6% | 1.25e-07 | 0.00% | 0.00 | [GXIVsPLA2](http://flybase.bio.indiana.edu/.bin/fbidq.html?GXIVsPLA2), [cert](http://flybase.bio.indiana.edu/.bin/fbidq.html?cert), [CG5508](http://flybase.bio.indiana.edu/.bin/fbidq.html?CG5508), [dare](http://flybase.bio.indiana.edu/.bin/fbidq.html?dare), [Npc2a](http://flybase.bio.indiana.edu/.bin/fbidq.html?Npc2a), [Inos](http://flybase.bio.indiana.edu/.bin/fbidq.html?Inos), [CG12140](http://flybase.bio.indiana.edu/.bin/fbidq.html?CG12140), [sktl](http://flybase.bio.indiana.edu/.bin/fbidq.html?sktl), [ry](http://flybase.bio.indiana.edu/.bin/fbidq.html?ry), [CG9904](http://flybase.bio.indiana.edu/.bin/fbidq.html?CG9904), [CG31523](http://flybase.bio.indiana.edu/.bin/fbidq.html?CG31523), [dob](http://flybase.bio.indiana.edu/.bin/fbidq.html?dob), [ifc](http://flybase.bio.indiana.edu/.bin/fbidq.html?ifc), [CG31683](http://flybase.bio.indiana.edu/.bin/fbidq.html?CG31683), [CG17544](http://flybase.bio.indiana.edu/.bin/fbidq.html?CG17544), [HLH106](http://flybase.bio.indiana.edu/.bin/fbidq.html?HLH106), [CG17029](http://flybase.bio.indiana.edu/.bin/fbidq.html?CG17029), [Hmgs](http://flybase.bio.indiana.edu/.bin/fbidq.html?Hmgs), [lace](http://flybase.bio.indiana.edu/.bin/fbidq.html?lace), [Thiolase](http://flybase.bio.indiana.edu/.bin/fbidq.html?Thiolase), [CG31915](http://flybase.bio.indiana.edu/.bin/fbidq.html?CG31915), [CG4585](http://flybase.bio.indiana.edu/.bin/fbidq.html?CG4585), [CG10383](http://flybase.bio.indiana.edu/.bin/fbidq.html?CG10383), [CG10932](http://flybase.bio.indiana.edu/.bin/fbidq.html?CG10932), [Acox57D-p](http://flybase.bio.indiana.edu/.bin/fbidq.html?Acox57D-p), [ACC](http://flybase.bio.indiana.edu/.bin/fbidq.html?ACC), [CG1998](http://flybase.bio.indiana.edu/.bin/fbidq.html?CG1998), [Lip4](http://flybase.bio.indiana.edu/.bin/fbidq.html?Lip4),[CG31414](http://flybase.bio.indiana.edu/.bin/fbidq.html?CG31414), [Npc2b](http://flybase.bio.indiana.edu/.bin/fbidq.html?Npc2b), [frj](http://flybase.bio.indiana.edu/.bin/fbidq.html?frj), [CG12262](http://flybase.bio.indiana.edu/.bin/fbidq.html?CG12262), [Lsd-2](http://flybase.bio.indiana.edu/.bin/fbidq.html?Lsd-2), [CG7997](http://flybase.bio.indiana.edu/.bin/fbidq.html?CG7997), [CG14945](http://flybase.bio.indiana.edu/.bin/fbidq.html?CG14945), [CDase](http://flybase.bio.indiana.edu/.bin/fbidq.html?CDase), [Npc1a](http://flybase.bio.indiana.edu/.bin/fbidq.html?Npc1a), [Cyp4g15](http://flybase.bio.indiana.edu/.bin/fbidq.html?Cyp4g15), [scu](http://flybase.bio.indiana.edu/.bin/fbidq.html?scu), [santa-maria](http://flybase.bio.indiana.edu/.bin/fbidq.html?santa-maria), [CG3267](http://flybase.bio.indiana.edu/.bin/fbidq.html?CG3267), [Gpdh](http://flybase.bio.indiana.edu/.bin/fbidq.html?Gpdh), [fu12](http://flybase.bio.indiana.edu/.bin/fbidq.html?fu12), [yip2](http://flybase.bio.indiana.edu/.bin/fbidq.html?yip2), [mdy](http://flybase.bio.indiana.edu/.bin/fbidq.html?mdy), [CG6543](http://flybase.bio.indiana.edu/.bin/fbidq.html?CG6543), [desat1](http://flybase.bio.indiana.edu/.bin/fbidq.html?desat1), [Hnf4](http://flybase.bio.indiana.edu/.bin/fbidq.html?Hnf4), [CG18135](http://flybase.bio.indiana.edu/.bin/fbidq.html?CG18135), [CG7149](http://flybase.bio.indiana.edu/.bin/fbidq.html?CG7149), [CG5991](http://flybase.bio.indiana.edu/.bin/fbidq.html?CG5991), [CG42271](http://flybase.bio.indiana.edu/.bin/fbidq.html?CG42271), [CG3376](http://flybase.bio.indiana.edu/.bin/fbidq.html?CG3376), [CG17292](http://flybase.bio.indiana.edu/.bin/fbidq.html?CG17292), [CG8709](http://flybase.bio.indiana.edu/.bin/fbidq.html?CG8709),[CG5895](http://flybase.bio.indiana.edu/.bin/fbidq.html?CG5895), [CG4389](http://flybase.bio.indiana.edu/.bin/fbidq.html?CG4389), [Mtp](http://flybase.bio.indiana.edu/.bin/fbidq.html?Mtp), [CG17026](http://flybase.bio.indiana.edu/.bin/fbidq.html?CG17026) |
| [glutathione metabolic process](http://amigo.geneontology.org/cgi-bin/amigo/go.cgi?view=details&query=GO:0006749) | 18 of 1000 genes, 1.8% | 27 of 7634 genes, 0.4% | 1.78e-07 | 0.00% | 0.00 | [GstD2](http://flybase.bio.indiana.edu/.bin/fbidq.html?GstD2), [Gclc](http://flybase.bio.indiana.edu/.bin/fbidq.html?Gclc), [GstE3](http://flybase.bio.indiana.edu/.bin/fbidq.html?GstE3), [CG9362](http://flybase.bio.indiana.edu/.bin/fbidq.html?CG9362), [CG1702](http://flybase.bio.indiana.edu/.bin/fbidq.html?CG1702), [CG4306](http://flybase.bio.indiana.edu/.bin/fbidq.html?CG4306), [GstE1](http://flybase.bio.indiana.edu/.bin/fbidq.html?GstE1), [GstE2](http://flybase.bio.indiana.edu/.bin/fbidq.html?GstE2), [CG30000](http://flybase.bio.indiana.edu/.bin/fbidq.html?CG30000), [GstD1](http://flybase.bio.indiana.edu/.bin/fbidq.html?GstD1), [GstD9](http://flybase.bio.indiana.edu/.bin/fbidq.html?GstD9), [CG11784](http://flybase.bio.indiana.edu/.bin/fbidq.html?CG11784), [CG4829](http://flybase.bio.indiana.edu/.bin/fbidq.html?CG4829), [GstE6](http://flybase.bio.indiana.edu/.bin/fbidq.html?GstE6), [CG16936](http://flybase.bio.indiana.edu/.bin/fbidq.html?CG16936), [GstS1](http://flybase.bio.indiana.edu/.bin/fbidq.html?GstS1), [GstE7](http://flybase.bio.indiana.edu/.bin/fbidq.html?GstE7), [CG30005](http://flybase.bio.indiana.edu/.bin/fbidq.html?CG30005) |
| [ion transport](http://amigo.geneontology.org/cgi-bin/amigo/go.cgi?view=details&query=GO:0006811) | 63 of 1000 genes, 6.3% | 220 of 7634 genes, 2.9% | 2.89e-07 | 0.00% | 0.00 | [pain](http://flybase.bio.indiana.edu/.bin/fbidq.html?pain), [CG15094](http://flybase.bio.indiana.edu/.bin/fbidq.html?CG15094), [pyx](http://flybase.bio.indiana.edu/.bin/fbidq.html?pyx), [Oatp58Dc](http://flybase.bio.indiana.edu/.bin/fbidq.html?Oatp58Dc), [VhaM9.7-c](http://flybase.bio.indiana.edu/.bin/fbidq.html?VhaM9.7-c), [CG5130](http://flybase.bio.indiana.edu/.bin/fbidq.html?CG5130), [nrv1](http://flybase.bio.indiana.edu/.bin/fbidq.html?nrv1), [ClC-a](http://flybase.bio.indiana.edu/.bin/fbidq.html?ClC-a), [colt](http://flybase.bio.indiana.edu/.bin/fbidq.html?colt), [Vha68-2](http://flybase.bio.indiana.edu/.bin/fbidq.html?Vha68-2), [Ent2](http://flybase.bio.indiana.edu/.bin/fbidq.html?Ent2), [Ctr1A](http://flybase.bio.indiana.edu/.bin/fbidq.html?Ctr1A), [CG11163](http://flybase.bio.indiana.edu/.bin/fbidq.html?CG11163), [CG3036](http://flybase.bio.indiana.edu/.bin/fbidq.html?CG3036), [VhaM9.7-b](http://flybase.bio.indiana.edu/.bin/fbidq.html?VhaM9.7-b), [Ir](http://flybase.bio.indiana.edu/.bin/fbidq.html?Ir), [Atox1](http://flybase.bio.indiana.edu/.bin/fbidq.html?Atox1), [CG17119](http://flybase.bio.indiana.edu/.bin/fbidq.html?CG17119), [CG31729](http://flybase.bio.indiana.edu/.bin/fbidq.html?CG31729), [Orct](http://flybase.bio.indiana.edu/.bin/fbidq.html?Orct), [Orct2](http://flybase.bio.indiana.edu/.bin/fbidq.html?Orct2), [VhaPPA1-1](http://flybase.bio.indiana.edu/.bin/fbidq.html?VhaPPA1-1), [JhI-21](http://flybase.bio.indiana.edu/.bin/fbidq.html?JhI-21), [hoe1](http://flybase.bio.indiana.edu/.bin/fbidq.html?hoe1), [CG9523](http://flybase.bio.indiana.edu/.bin/fbidq.html?CG9523), [nrv2](http://flybase.bio.indiana.edu/.bin/fbidq.html?nrv2), [CG6672](http://flybase.bio.indiana.edu/.bin/fbidq.html?CG6672), [VhaM8.9](http://flybase.bio.indiana.edu/.bin/fbidq.html?VhaM8.9), [CD98hc](http://flybase.bio.indiana.edu/.bin/fbidq.html?CD98hc),[w](http://flybase.bio.indiana.edu/.bin/fbidq.html?w), [CG8790](http://flybase.bio.indiana.edu/.bin/fbidq.html?CG8790), [Picot](http://flybase.bio.indiana.edu/.bin/fbidq.html?Picot), [CG7816](http://flybase.bio.indiana.edu/.bin/fbidq.html?CG7816), [Indy](http://flybase.bio.indiana.edu/.bin/fbidq.html?Indy), [CG16700](http://flybase.bio.indiana.edu/.bin/fbidq.html?CG16700), [Irk2](http://flybase.bio.indiana.edu/.bin/fbidq.html?Irk2), [Oatp33Ea](http://flybase.bio.indiana.edu/.bin/fbidq.html?Oatp33Ea), [ClC-c](http://flybase.bio.indiana.edu/.bin/fbidq.html?ClC-c), [CG4301](http://flybase.bio.indiana.edu/.bin/fbidq.html?CG4301), [Efr](http://flybase.bio.indiana.edu/.bin/fbidq.html?Efr), [CG5002](http://flybase.bio.indiana.edu/.bin/fbidq.html?CG5002), [Itp-r83A](http://flybase.bio.indiana.edu/.bin/fbidq.html?Itp-r83A), [NKAIN](http://flybase.bio.indiana.edu/.bin/fbidq.html?NKAIN), [mnd](http://flybase.bio.indiana.edu/.bin/fbidq.html?mnd), [CG6125](http://flybase.bio.indiana.edu/.bin/fbidq.html?CG6125), [Irk3](http://flybase.bio.indiana.edu/.bin/fbidq.html?Irk3), [wtrw](http://flybase.bio.indiana.edu/.bin/fbidq.html?wtrw), [trpm](http://flybase.bio.indiana.edu/.bin/fbidq.html?trpm), [Tsf2](http://flybase.bio.indiana.edu/.bin/fbidq.html?Tsf2), [CG12344](http://flybase.bio.indiana.edu/.bin/fbidq.html?CG12344), [CG6812](http://flybase.bio.indiana.edu/.bin/fbidq.html?CG6812), [CG10006](http://flybase.bio.indiana.edu/.bin/fbidq.html?CG10006), [Vha100-2](http://flybase.bio.indiana.edu/.bin/fbidq.html?Vha100-2), [CG11655](http://flybase.bio.indiana.edu/.bin/fbidq.html?CG11655), [gb](http://flybase.bio.indiana.edu/.bin/fbidq.html?gb), [VhaAC39-1](http://flybase.bio.indiana.edu/.bin/fbidq.html?VhaAC39-1), [CG1607](http://flybase.bio.indiana.edu/.bin/fbidq.html?CG1607), [Eaat1](http://flybase.bio.indiana.edu/.bin/fbidq.html?Eaat1),[CG1628](http://flybase.bio.indiana.edu/.bin/fbidq.html?CG1628), [Atpalpha](http://flybase.bio.indiana.edu/.bin/fbidq.html?Atpalpha), [Ork1](http://flybase.bio.indiana.edu/.bin/fbidq.html?Ork1), [CG1907](http://flybase.bio.indiana.edu/.bin/fbidq.html?CG1907), [Fatp](http://flybase.bio.indiana.edu/.bin/fbidq.html?Fatp) |
| [cellular amide metabolic process](http://amigo.geneontology.org/cgi-bin/amigo/go.cgi?view=details&query=GO:0043603) | 28 of 1000 genes, 2.8% | 67 of 7634 genes, 0.9% | 4.47e-06 | 0.00% | 0.00 | [GstD2](http://flybase.bio.indiana.edu/.bin/fbidq.html?GstD2), [Gclc](http://flybase.bio.indiana.edu/.bin/fbidq.html?Gclc), [GstE3](http://flybase.bio.indiana.edu/.bin/fbidq.html?GstE3), [spz](http://flybase.bio.indiana.edu/.bin/fbidq.html?spz), [imd](http://flybase.bio.indiana.edu/.bin/fbidq.html?imd), [CG9362](http://flybase.bio.indiana.edu/.bin/fbidq.html?CG9362), [CG1702](http://flybase.bio.indiana.edu/.bin/fbidq.html?CG1702), [CG4306](http://flybase.bio.indiana.edu/.bin/fbidq.html?CG4306), [GstE1](http://flybase.bio.indiana.edu/.bin/fbidq.html?GstE1), [GstE2](http://flybase.bio.indiana.edu/.bin/fbidq.html?GstE2), [Phm](http://flybase.bio.indiana.edu/.bin/fbidq.html?Phm), [CG30000](http://flybase.bio.indiana.edu/.bin/fbidq.html?CG30000), [GstD9](http://flybase.bio.indiana.edu/.bin/fbidq.html?GstD9), [GstD1](http://flybase.bio.indiana.edu/.bin/fbidq.html?GstD1), [asparagine-synthetase](http://flybase.bio.indiana.edu/.bin/fbidq.html?asparagine-synthetase), [CG11784](http://flybase.bio.indiana.edu/.bin/fbidq.html?CG11784), [CG3376](http://flybase.bio.indiana.edu/.bin/fbidq.html?CG3376), [Myd88](http://flybase.bio.indiana.edu/.bin/fbidq.html?Myd88), [BG4](http://flybase.bio.indiana.edu/.bin/fbidq.html?BG4), [PGRP-SA](http://flybase.bio.indiana.edu/.bin/fbidq.html?PGRP-SA), [CG4829](http://flybase.bio.indiana.edu/.bin/fbidq.html?CG4829), [GstE6](http://flybase.bio.indiana.edu/.bin/fbidq.html?GstE6), [ird5](http://flybase.bio.indiana.edu/.bin/fbidq.html?ird5), [GstS1](http://flybase.bio.indiana.edu/.bin/fbidq.html?GstS1), [Tollo](http://flybase.bio.indiana.edu/.bin/fbidq.html?Tollo), [CG16936](http://flybase.bio.indiana.edu/.bin/fbidq.html?CG16936), [GstE7](http://flybase.bio.indiana.edu/.bin/fbidq.html?GstE7),[CG30005](http://flybase.bio.indiana.edu/.bin/fbidq.html?CG30005) |
| [cellular modified amino acid metabolic process](http://amigo.geneontology.org/cgi-bin/amigo/go.cgi?view=details&query=GO:0006575) | 21 of 1000 genes, 2.1% | 42 of 7634 genes, 0.6% | 8.77e-06 | 0.00% | 0.00 | [GstD2](http://flybase.bio.indiana.edu/.bin/fbidq.html?GstD2), [Gclc](http://flybase.bio.indiana.edu/.bin/fbidq.html?Gclc), [GstE3](http://flybase.bio.indiana.edu/.bin/fbidq.html?GstE3), [CG9362](http://flybase.bio.indiana.edu/.bin/fbidq.html?CG9362), [CG1702](http://flybase.bio.indiana.edu/.bin/fbidq.html?CG1702), [CG4306](http://flybase.bio.indiana.edu/.bin/fbidq.html?CG4306), [GstE1](http://flybase.bio.indiana.edu/.bin/fbidq.html?GstE1), [Nmdmc](http://flybase.bio.indiana.edu/.bin/fbidq.html?Nmdmc), [GstE2](http://flybase.bio.indiana.edu/.bin/fbidq.html?GstE2), [CG9547](http://flybase.bio.indiana.edu/.bin/fbidq.html?CG9547), [PH4alphaEFB](http://flybase.bio.indiana.edu/.bin/fbidq.html?PH4alphaEFB), [CG30000](http://flybase.bio.indiana.edu/.bin/fbidq.html?CG30000), [GstD1](http://flybase.bio.indiana.edu/.bin/fbidq.html?GstD1), [GstD9](http://flybase.bio.indiana.edu/.bin/fbidq.html?GstD9), [CG11784](http://flybase.bio.indiana.edu/.bin/fbidq.html?CG11784), [CG4829](http://flybase.bio.indiana.edu/.bin/fbidq.html?CG4829), [GstE6](http://flybase.bio.indiana.edu/.bin/fbidq.html?GstE6), [CG16936](http://flybase.bio.indiana.edu/.bin/fbidq.html?CG16936), [GstS1](http://flybase.bio.indiana.edu/.bin/fbidq.html?GstS1), [GstE7](http://flybase.bio.indiana.edu/.bin/fbidq.html?GstE7), [CG30005](http://flybase.bio.indiana.edu/.bin/fbidq.html?CG30005) |
| [carbohydrate metabolic process](http://amigo.geneontology.org/cgi-bin/amigo/go.cgi?view=details&query=GO:0005975) | 51 of 1000 genes, 5.1% | 176 of 7634 genes, 2.3% | 1.06e-05 | 0.00% | 0.00 | [Pfk](http://flybase.bio.indiana.edu/.bin/fbidq.html?Pfk), [Treh](http://flybase.bio.indiana.edu/.bin/fbidq.html?Treh), [fbp](http://flybase.bio.indiana.edu/.bin/fbidq.html?fbp), [GalNAc-T1](http://flybase.bio.indiana.edu/.bin/fbidq.html?GalNAc-T1), [CG5362](http://flybase.bio.indiana.edu/.bin/fbidq.html?CG5362), [Nmdmc](http://flybase.bio.indiana.edu/.bin/fbidq.html?Nmdmc), [Inos](http://flybase.bio.indiana.edu/.bin/fbidq.html?Inos), [CG10467](http://flybase.bio.indiana.edu/.bin/fbidq.html?CG10467), [CG12582](http://flybase.bio.indiana.edu/.bin/fbidq.html?CG12582), [GNBP1](http://flybase.bio.indiana.edu/.bin/fbidq.html?GNBP1), [CG15093](http://flybase.bio.indiana.edu/.bin/fbidq.html?CG15093), [foxo](http://flybase.bio.indiana.edu/.bin/fbidq.html?foxo), [Glycogenin](http://flybase.bio.indiana.edu/.bin/fbidq.html?Glycogenin), [Oscillin](http://flybase.bio.indiana.edu/.bin/fbidq.html?Oscillin), [CG9232](http://flybase.bio.indiana.edu/.bin/fbidq.html?CG9232), [CG31849](http://flybase.bio.indiana.edu/.bin/fbidq.html?CG31849), [fng](http://flybase.bio.indiana.edu/.bin/fbidq.html?fng), [CG31915](http://flybase.bio.indiana.edu/.bin/fbidq.html?CG31915), [CD98hc](http://flybase.bio.indiana.edu/.bin/fbidq.html?CD98hc), [CG6453](http://flybase.bio.indiana.edu/.bin/fbidq.html?CG6453), [CG31414](http://flybase.bio.indiana.edu/.bin/fbidq.html?CG31414), [l(1)G0334](http://flybase.bio.indiana.edu/.bin/fbidq.html?l(1)G0334), [OstStt3](http://flybase.bio.indiana.edu/.bin/fbidq.html?OstStt3), [Tollo](http://flybase.bio.indiana.edu/.bin/fbidq.html?Tollo), [Arf51F](http://flybase.bio.indiana.edu/.bin/fbidq.html?Arf51F), [CG10627](http://flybase.bio.indiana.edu/.bin/fbidq.html?CG10627), [Idgf4](http://flybase.bio.indiana.edu/.bin/fbidq.html?Idgf4),[CG7997](http://flybase.bio.indiana.edu/.bin/fbidq.html?CG7997), [PhKgamma](http://flybase.bio.indiana.edu/.bin/fbidq.html?PhKgamma), [Efr](http://flybase.bio.indiana.edu/.bin/fbidq.html?Efr), [CG33080](http://flybase.bio.indiana.edu/.bin/fbidq.html?CG33080), [GalNAc-T2](http://flybase.bio.indiana.edu/.bin/fbidq.html?GalNAc-T2), [CG8417](http://flybase.bio.indiana.edu/.bin/fbidq.html?CG8417), [CG17333](http://flybase.bio.indiana.edu/.bin/fbidq.html?CG17333), [Gpdh](http://flybase.bio.indiana.edu/.bin/fbidq.html?Gpdh), [Hexo2](http://flybase.bio.indiana.edu/.bin/fbidq.html?Hexo2), [CG10688](http://flybase.bio.indiana.edu/.bin/fbidq.html?CG10688), [CG15117](http://flybase.bio.indiana.edu/.bin/fbidq.html?CG15117), [Idgf2](http://flybase.bio.indiana.edu/.bin/fbidq.html?Idgf2), [CG18135](http://flybase.bio.indiana.edu/.bin/fbidq.html?CG18135), [Pepck](http://flybase.bio.indiana.edu/.bin/fbidq.html?Pepck), [Tal](http://flybase.bio.indiana.edu/.bin/fbidq.html?Tal), [Cht3](http://flybase.bio.indiana.edu/.bin/fbidq.html?Cht3), [CG5210](http://flybase.bio.indiana.edu/.bin/fbidq.html?CG5210), [Pgd](http://flybase.bio.indiana.edu/.bin/fbidq.html?Pgd), [Idgf3](http://flybase.bio.indiana.edu/.bin/fbidq.html?Idgf3), [CG3792](http://flybase.bio.indiana.edu/.bin/fbidq.html?CG3792), [CG13937](http://flybase.bio.indiana.edu/.bin/fbidq.html?CG13937), [ttv](http://flybase.bio.indiana.edu/.bin/fbidq.html?ttv), [CG30410](http://flybase.bio.indiana.edu/.bin/fbidq.html?CG30410), [CG30499](http://flybase.bio.indiana.edu/.bin/fbidq.html?CG30499) |
| [cellular lipid metabolic process](http://amigo.geneontology.org/cgi-bin/amigo/go.cgi?view=details&query=GO:0044255) | 47 of 1000 genes, 4.7% | 156 of 7634 genes, 2.0% | 1.07e-05 | 0.00% | 0.00 | [GXIVsPLA2](http://flybase.bio.indiana.edu/.bin/fbidq.html?GXIVsPLA2), [cert](http://flybase.bio.indiana.edu/.bin/fbidq.html?cert), [CG5508](http://flybase.bio.indiana.edu/.bin/fbidq.html?CG5508), [Inos](http://flybase.bio.indiana.edu/.bin/fbidq.html?Inos), [CG12140](http://flybase.bio.indiana.edu/.bin/fbidq.html?CG12140), [sktl](http://flybase.bio.indiana.edu/.bin/fbidq.html?sktl), [ry](http://flybase.bio.indiana.edu/.bin/fbidq.html?ry), [CG9904](http://flybase.bio.indiana.edu/.bin/fbidq.html?CG9904), [CG31523](http://flybase.bio.indiana.edu/.bin/fbidq.html?CG31523), [ifc](http://flybase.bio.indiana.edu/.bin/fbidq.html?ifc), [CG17544](http://flybase.bio.indiana.edu/.bin/fbidq.html?CG17544), [HLH106](http://flybase.bio.indiana.edu/.bin/fbidq.html?HLH106), [CG17029](http://flybase.bio.indiana.edu/.bin/fbidq.html?CG17029), [Hmgs](http://flybase.bio.indiana.edu/.bin/fbidq.html?Hmgs), [lace](http://flybase.bio.indiana.edu/.bin/fbidq.html?lace), [Thiolase](http://flybase.bio.indiana.edu/.bin/fbidq.html?Thiolase), [CG31915](http://flybase.bio.indiana.edu/.bin/fbidq.html?CG31915), [CG4585](http://flybase.bio.indiana.edu/.bin/fbidq.html?CG4585), [CG10383](http://flybase.bio.indiana.edu/.bin/fbidq.html?CG10383), [CG10932](http://flybase.bio.indiana.edu/.bin/fbidq.html?CG10932), [Acox57D-p](http://flybase.bio.indiana.edu/.bin/fbidq.html?Acox57D-p), [ACC](http://flybase.bio.indiana.edu/.bin/fbidq.html?ACC), [CG1998](http://flybase.bio.indiana.edu/.bin/fbidq.html?CG1998), [CG31414](http://flybase.bio.indiana.edu/.bin/fbidq.html?CG31414), [frj](http://flybase.bio.indiana.edu/.bin/fbidq.html?frj), [CG12262](http://flybase.bio.indiana.edu/.bin/fbidq.html?CG12262), [CG7997](http://flybase.bio.indiana.edu/.bin/fbidq.html?CG7997),[CG14945](http://flybase.bio.indiana.edu/.bin/fbidq.html?CG14945), [CDase](http://flybase.bio.indiana.edu/.bin/fbidq.html?CDase), [scu](http://flybase.bio.indiana.edu/.bin/fbidq.html?scu), [santa-maria](http://flybase.bio.indiana.edu/.bin/fbidq.html?santa-maria), [CG3267](http://flybase.bio.indiana.edu/.bin/fbidq.html?CG3267), [Gpdh](http://flybase.bio.indiana.edu/.bin/fbidq.html?Gpdh), [fu12](http://flybase.bio.indiana.edu/.bin/fbidq.html?fu12), [yip2](http://flybase.bio.indiana.edu/.bin/fbidq.html?yip2), [mdy](http://flybase.bio.indiana.edu/.bin/fbidq.html?mdy), [CG6543](http://flybase.bio.indiana.edu/.bin/fbidq.html?CG6543), [desat1](http://flybase.bio.indiana.edu/.bin/fbidq.html?desat1), [Hnf4](http://flybase.bio.indiana.edu/.bin/fbidq.html?Hnf4), [CG7149](http://flybase.bio.indiana.edu/.bin/fbidq.html?CG7149), [CG5991](http://flybase.bio.indiana.edu/.bin/fbidq.html?CG5991), [CG3376](http://flybase.bio.indiana.edu/.bin/fbidq.html?CG3376), [CG42271](http://flybase.bio.indiana.edu/.bin/fbidq.html?CG42271), [CG8709](http://flybase.bio.indiana.edu/.bin/fbidq.html?CG8709), [CG4389](http://flybase.bio.indiana.edu/.bin/fbidq.html?CG4389), [Mtp](http://flybase.bio.indiana.edu/.bin/fbidq.html?Mtp), [CG17026](http://flybase.bio.indiana.edu/.bin/fbidq.html?CG17026) |
| [cation transport](http://amigo.geneontology.org/cgi-bin/amigo/go.cgi?view=details&query=GO:0006812) | 44 of 1000 genes, 4.4% | 142 of 7634 genes, 1.9% | 1.27e-05 | 0.00% | 0.00 | [pain](http://flybase.bio.indiana.edu/.bin/fbidq.html?pain), [CG15094](http://flybase.bio.indiana.edu/.bin/fbidq.html?CG15094), [pyx](http://flybase.bio.indiana.edu/.bin/fbidq.html?pyx), [VhaM9.7-c](http://flybase.bio.indiana.edu/.bin/fbidq.html?VhaM9.7-c), [CG5130](http://flybase.bio.indiana.edu/.bin/fbidq.html?CG5130), [nrv1](http://flybase.bio.indiana.edu/.bin/fbidq.html?nrv1), [colt](http://flybase.bio.indiana.edu/.bin/fbidq.html?colt), [Vha68-2](http://flybase.bio.indiana.edu/.bin/fbidq.html?Vha68-2), [Ent2](http://flybase.bio.indiana.edu/.bin/fbidq.html?Ent2), [Ctr1A](http://flybase.bio.indiana.edu/.bin/fbidq.html?Ctr1A), [CG11163](http://flybase.bio.indiana.edu/.bin/fbidq.html?CG11163), [VhaM9.7-b](http://flybase.bio.indiana.edu/.bin/fbidq.html?VhaM9.7-b), [Ir](http://flybase.bio.indiana.edu/.bin/fbidq.html?Ir), [Atox1](http://flybase.bio.indiana.edu/.bin/fbidq.html?Atox1), [CG31729](http://flybase.bio.indiana.edu/.bin/fbidq.html?CG31729), [Orct](http://flybase.bio.indiana.edu/.bin/fbidq.html?Orct), [Orct2](http://flybase.bio.indiana.edu/.bin/fbidq.html?Orct2), [VhaPPA1-1](http://flybase.bio.indiana.edu/.bin/fbidq.html?VhaPPA1-1), [JhI-21](http://flybase.bio.indiana.edu/.bin/fbidq.html?JhI-21), [CG9523](http://flybase.bio.indiana.edu/.bin/fbidq.html?CG9523), [nrv2](http://flybase.bio.indiana.edu/.bin/fbidq.html?nrv2), [CG6672](http://flybase.bio.indiana.edu/.bin/fbidq.html?CG6672), [VhaM8.9](http://flybase.bio.indiana.edu/.bin/fbidq.html?VhaM8.9), [CD98hc](http://flybase.bio.indiana.edu/.bin/fbidq.html?CD98hc), [CG7816](http://flybase.bio.indiana.edu/.bin/fbidq.html?CG7816), [Indy](http://flybase.bio.indiana.edu/.bin/fbidq.html?Indy), [Irk2](http://flybase.bio.indiana.edu/.bin/fbidq.html?Irk2), [CG4301](http://flybase.bio.indiana.edu/.bin/fbidq.html?CG4301), [Itp-r83A](http://flybase.bio.indiana.edu/.bin/fbidq.html?Itp-r83A), [NKAIN](http://flybase.bio.indiana.edu/.bin/fbidq.html?NKAIN),[mnd](http://flybase.bio.indiana.edu/.bin/fbidq.html?mnd), [Irk3](http://flybase.bio.indiana.edu/.bin/fbidq.html?Irk3), [trpm](http://flybase.bio.indiana.edu/.bin/fbidq.html?trpm), [wtrw](http://flybase.bio.indiana.edu/.bin/fbidq.html?wtrw), [Tsf2](http://flybase.bio.indiana.edu/.bin/fbidq.html?Tsf2), [CG6812](http://flybase.bio.indiana.edu/.bin/fbidq.html?CG6812), [CG10006](http://flybase.bio.indiana.edu/.bin/fbidq.html?CG10006), [Vha100-2](http://flybase.bio.indiana.edu/.bin/fbidq.html?Vha100-2), [gb](http://flybase.bio.indiana.edu/.bin/fbidq.html?gb), [CG11655](http://flybase.bio.indiana.edu/.bin/fbidq.html?CG11655), [VhaAC39-1](http://flybase.bio.indiana.edu/.bin/fbidq.html?VhaAC39-1), [Atpalpha](http://flybase.bio.indiana.edu/.bin/fbidq.html?Atpalpha), [CG1628](http://flybase.bio.indiana.edu/.bin/fbidq.html?CG1628), [Ork1](http://flybase.bio.indiana.edu/.bin/fbidq.html?Ork1) |
| [peptide metabolic process](http://amigo.geneontology.org/cgi-bin/amigo/go.cgi?view=details&query=GO:0006518) | 26 of 1000 genes, 2.6% | 62 of 7634 genes, 0.8% | 1.52e-05 | 0.00% | 0.00 | [GstD2](http://flybase.bio.indiana.edu/.bin/fbidq.html?GstD2), [Gclc](http://flybase.bio.indiana.edu/.bin/fbidq.html?Gclc), [GstE3](http://flybase.bio.indiana.edu/.bin/fbidq.html?GstE3), [spz](http://flybase.bio.indiana.edu/.bin/fbidq.html?spz), [imd](http://flybase.bio.indiana.edu/.bin/fbidq.html?imd), [CG9362](http://flybase.bio.indiana.edu/.bin/fbidq.html?CG9362), [CG1702](http://flybase.bio.indiana.edu/.bin/fbidq.html?CG1702), [CG4306](http://flybase.bio.indiana.edu/.bin/fbidq.html?CG4306), [GstE1](http://flybase.bio.indiana.edu/.bin/fbidq.html?GstE1), [GstE2](http://flybase.bio.indiana.edu/.bin/fbidq.html?GstE2), [Phm](http://flybase.bio.indiana.edu/.bin/fbidq.html?Phm), [CG30000](http://flybase.bio.indiana.edu/.bin/fbidq.html?CG30000), [GstD1](http://flybase.bio.indiana.edu/.bin/fbidq.html?GstD1), [GstD9](http://flybase.bio.indiana.edu/.bin/fbidq.html?GstD9), [CG11784](http://flybase.bio.indiana.edu/.bin/fbidq.html?CG11784), [Myd88](http://flybase.bio.indiana.edu/.bin/fbidq.html?Myd88), [BG4](http://flybase.bio.indiana.edu/.bin/fbidq.html?BG4), [PGRP-SA](http://flybase.bio.indiana.edu/.bin/fbidq.html?PGRP-SA), [CG4829](http://flybase.bio.indiana.edu/.bin/fbidq.html?CG4829), [GstE6](http://flybase.bio.indiana.edu/.bin/fbidq.html?GstE6), [ird5](http://flybase.bio.indiana.edu/.bin/fbidq.html?ird5), [GstS1](http://flybase.bio.indiana.edu/.bin/fbidq.html?GstS1), [Tollo](http://flybase.bio.indiana.edu/.bin/fbidq.html?Tollo), [CG16936](http://flybase.bio.indiana.edu/.bin/fbidq.html?CG16936), [GstE7](http://flybase.bio.indiana.edu/.bin/fbidq.html?GstE7), [CG30005](http://flybase.bio.indiana.edu/.bin/fbidq.html?CG30005) |
| [fatty acid metabolic process](http://amigo.geneontology.org/cgi-bin/amigo/go.cgi?view=details&query=GO:0006631) | 18 of 1000 genes, 1.8% | 33 of 7634 genes, 0.4% | 1.85e-05 | 0.00% | 0.00 | [scu](http://flybase.bio.indiana.edu/.bin/fbidq.html?scu), [CG3267](http://flybase.bio.indiana.edu/.bin/fbidq.html?CG3267), [CG12140](http://flybase.bio.indiana.edu/.bin/fbidq.html?CG12140), [yip2](http://flybase.bio.indiana.edu/.bin/fbidq.html?yip2), [CG31523](http://flybase.bio.indiana.edu/.bin/fbidq.html?CG31523), [CG6543](http://flybase.bio.indiana.edu/.bin/fbidq.html?CG6543), [desat1](http://flybase.bio.indiana.edu/.bin/fbidq.html?desat1), [ifc](http://flybase.bio.indiana.edu/.bin/fbidq.html?ifc), [CG17544](http://flybase.bio.indiana.edu/.bin/fbidq.html?CG17544), [HLH106](http://flybase.bio.indiana.edu/.bin/fbidq.html?HLH106), [Thiolase](http://flybase.bio.indiana.edu/.bin/fbidq.html?Thiolase), [CG8709](http://flybase.bio.indiana.edu/.bin/fbidq.html?CG8709), [Acox57D-p](http://flybase.bio.indiana.edu/.bin/fbidq.html?Acox57D-p), [CG10932](http://flybase.bio.indiana.edu/.bin/fbidq.html?CG10932), [ACC](http://flybase.bio.indiana.edu/.bin/fbidq.html?ACC), [CG1998](http://flybase.bio.indiana.edu/.bin/fbidq.html?CG1998), [CG4389](http://flybase.bio.indiana.edu/.bin/fbidq.html?CG4389), [CG12262](http://flybase.bio.indiana.edu/.bin/fbidq.html?CG12262) |
| [monocarboxylic acid metabolic process](http://amigo.geneontology.org/cgi-bin/amigo/go.cgi?view=details&query=GO:0032787) | 22 of 1000 genes, 2.2% | 49 of 7634 genes, 0.6% | 4.72e-05 | 0.00% | 0.00 | [scu](http://flybase.bio.indiana.edu/.bin/fbidq.html?scu), [CG7433](http://flybase.bio.indiana.edu/.bin/fbidq.html?CG7433), [CG3267](http://flybase.bio.indiana.edu/.bin/fbidq.html?CG3267), [CG12140](http://flybase.bio.indiana.edu/.bin/fbidq.html?CG12140), [yip2](http://flybase.bio.indiana.edu/.bin/fbidq.html?yip2), [PH4alphaEFB](http://flybase.bio.indiana.edu/.bin/fbidq.html?PH4alphaEFB), [CG31523](http://flybase.bio.indiana.edu/.bin/fbidq.html?CG31523), [CG6543](http://flybase.bio.indiana.edu/.bin/fbidq.html?CG6543), [desat1](http://flybase.bio.indiana.edu/.bin/fbidq.html?desat1), [ifc](http://flybase.bio.indiana.edu/.bin/fbidq.html?ifc), [CG17544](http://flybase.bio.indiana.edu/.bin/fbidq.html?CG17544), [HLH106](http://flybase.bio.indiana.edu/.bin/fbidq.html?HLH106), [Thiolase](http://flybase.bio.indiana.edu/.bin/fbidq.html?Thiolase), [CG8709](http://flybase.bio.indiana.edu/.bin/fbidq.html?CG8709), [Acox57D-p](http://flybase.bio.indiana.edu/.bin/fbidq.html?Acox57D-p), [CG10932](http://flybase.bio.indiana.edu/.bin/fbidq.html?CG10932), [ACC](http://flybase.bio.indiana.edu/.bin/fbidq.html?ACC), [Ssadh](http://flybase.bio.indiana.edu/.bin/fbidq.html?Ssadh), [l(1)G0334](http://flybase.bio.indiana.edu/.bin/fbidq.html?l(1)G0334), [CG1998](http://flybase.bio.indiana.edu/.bin/fbidq.html?CG1998), [CG4389](http://flybase.bio.indiana.edu/.bin/fbidq.html?CG4389), [CG12262](http://flybase.bio.indiana.edu/.bin/fbidq.html?CG12262) |
| [septate junction assembly](http://amigo.geneontology.org/cgi-bin/amigo/go.cgi?view=details&query=GO:0019991) | 16 of 1000 genes, 1.6% | 28 of 7634 genes, 0.4% | 5.05e-05 | 0.00% | 0.00 | [crok](http://flybase.bio.indiana.edu/.bin/fbidq.html?crok), [Cont](http://flybase.bio.indiana.edu/.bin/fbidq.html?Cont), [sinu](http://flybase.bio.indiana.edu/.bin/fbidq.html?sinu), [Nrg](http://flybase.bio.indiana.edu/.bin/fbidq.html?Nrg), [kune](http://flybase.bio.indiana.edu/.bin/fbidq.html?kune), [moody](http://flybase.bio.indiana.edu/.bin/fbidq.html?moody), [vari](http://flybase.bio.indiana.edu/.bin/fbidq.html?vari), [G-ialpha65A](http://flybase.bio.indiana.edu/.bin/fbidq.html?G-ialpha65A), [crim](http://flybase.bio.indiana.edu/.bin/fbidq.html?crim), [cold](http://flybase.bio.indiana.edu/.bin/fbidq.html?cold), [wun](http://flybase.bio.indiana.edu/.bin/fbidq.html?wun), [nrv2](http://flybase.bio.indiana.edu/.bin/fbidq.html?nrv2), [loco](http://flybase.bio.indiana.edu/.bin/fbidq.html?loco), [Atpalpha](http://flybase.bio.indiana.edu/.bin/fbidq.html?Atpalpha), [Tsf2](http://flybase.bio.indiana.edu/.bin/fbidq.html?Tsf2), [pck](http://flybase.bio.indiana.edu/.bin/fbidq.html?pck) |
| [sulfur compound metabolic process](http://amigo.geneontology.org/cgi-bin/amigo/go.cgi?view=details&query=GO:0006790) | 24 of 1000 genes, 2.4% | 57 of 7634 genes, 0.7% | 5.16e-05 | 0.00% | 0.00 | [GstD2](http://flybase.bio.indiana.edu/.bin/fbidq.html?GstD2), [Efr](http://flybase.bio.indiana.edu/.bin/fbidq.html?Efr), [Gclc](http://flybase.bio.indiana.edu/.bin/fbidq.html?Gclc), [GstE3](http://flybase.bio.indiana.edu/.bin/fbidq.html?GstE3), [CG9362](http://flybase.bio.indiana.edu/.bin/fbidq.html?CG9362), [Eip55E](http://flybase.bio.indiana.edu/.bin/fbidq.html?Eip55E), [CG1702](http://flybase.bio.indiana.edu/.bin/fbidq.html?CG1702), [CG4306](http://flybase.bio.indiana.edu/.bin/fbidq.html?CG4306), [CG3267](http://flybase.bio.indiana.edu/.bin/fbidq.html?CG3267), [GstE1](http://flybase.bio.indiana.edu/.bin/fbidq.html?GstE1), [GstE2](http://flybase.bio.indiana.edu/.bin/fbidq.html?GstE2), [CG30000](http://flybase.bio.indiana.edu/.bin/fbidq.html?CG30000), [GstD1](http://flybase.bio.indiana.edu/.bin/fbidq.html?GstD1), [GstD9](http://flybase.bio.indiana.edu/.bin/fbidq.html?GstD9), [CG11784](http://flybase.bio.indiana.edu/.bin/fbidq.html?CG11784), [Mical](http://flybase.bio.indiana.edu/.bin/fbidq.html?Mical), [Papss](http://flybase.bio.indiana.edu/.bin/fbidq.html?Papss), [CG4829](http://flybase.bio.indiana.edu/.bin/fbidq.html?CG4829), [GstE6](http://flybase.bio.indiana.edu/.bin/fbidq.html?GstE6), [ttv](http://flybase.bio.indiana.edu/.bin/fbidq.html?ttv), [GstS1](http://flybase.bio.indiana.edu/.bin/fbidq.html?GstS1), [CG16936](http://flybase.bio.indiana.edu/.bin/fbidq.html?CG16936), [GstE7](http://flybase.bio.indiana.edu/.bin/fbidq.html?GstE7), [CG30005](http://flybase.bio.indiana.edu/.bin/fbidq.html?CG30005) |
| [organonitrogen compound metabolic process](http://amigo.geneontology.org/cgi-bin/amigo/go.cgi?view=details&query=GO:1901564) | 96 of 1000 genes, 9.6% | 443 of 7634 genes, 5.8% | 8.83e-05 | 0.00% | 0.00 | [CG9339](http://flybase.bio.indiana.edu/.bin/fbidq.html?CG9339), [CG9362](http://flybase.bio.indiana.edu/.bin/fbidq.html?CG9362), [Eip55E](http://flybase.bio.indiana.edu/.bin/fbidq.html?Eip55E), [Got1](http://flybase.bio.indiana.edu/.bin/fbidq.html?Got1), [cert](http://flybase.bio.indiana.edu/.bin/fbidq.html?cert), [Nmdmc](http://flybase.bio.indiana.edu/.bin/fbidq.html?Nmdmc), [Pect](http://flybase.bio.indiana.edu/.bin/fbidq.html?Pect), [CG9547](http://flybase.bio.indiana.edu/.bin/fbidq.html?CG9547), [ry](http://flybase.bio.indiana.edu/.bin/fbidq.html?ry), [CG3590](http://flybase.bio.indiana.edu/.bin/fbidq.html?CG3590), [Git](http://flybase.bio.indiana.edu/.bin/fbidq.html?Git), [CG11255](http://flybase.bio.indiana.edu/.bin/fbidq.html?CG11255), [Phm](http://flybase.bio.indiana.edu/.bin/fbidq.html?Phm), [pyd3](http://flybase.bio.indiana.edu/.bin/fbidq.html?pyd3), [CG30000](http://flybase.bio.indiana.edu/.bin/fbidq.html?CG30000), [dysb](http://flybase.bio.indiana.edu/.bin/fbidq.html?dysb), [CG11784](http://flybase.bio.indiana.edu/.bin/fbidq.html?CG11784), [asparagine-synthetase](http://flybase.bio.indiana.edu/.bin/fbidq.html?asparagine-synthetase), [lace](http://flybase.bio.indiana.edu/.bin/fbidq.html?lace), [Adk3](http://flybase.bio.indiana.edu/.bin/fbidq.html?Adk3), [Myd88](http://flybase.bio.indiana.edu/.bin/fbidq.html?Myd88), [CG17896](http://flybase.bio.indiana.edu/.bin/fbidq.html?CG17896), [CG4829](http://flybase.bio.indiana.edu/.bin/fbidq.html?CG4829), [w](http://flybase.bio.indiana.edu/.bin/fbidq.html?w), [GstE6](http://flybase.bio.indiana.edu/.bin/fbidq.html?GstE6), [ird5](http://flybase.bio.indiana.edu/.bin/fbidq.html?ird5), [Ssadh](http://flybase.bio.indiana.edu/.bin/fbidq.html?Ssadh), [Ace](http://flybase.bio.indiana.edu/.bin/fbidq.html?Ace), [CG31414](http://flybase.bio.indiana.edu/.bin/fbidq.html?CG31414), [Tollo](http://flybase.bio.indiana.edu/.bin/fbidq.html?Tollo),[ade2](http://flybase.bio.indiana.edu/.bin/fbidq.html?ade2), [GstD2](http://flybase.bio.indiana.edu/.bin/fbidq.html?GstD2), [CG6638](http://flybase.bio.indiana.edu/.bin/fbidq.html?CG6638), [CG8360](http://flybase.bio.indiana.edu/.bin/fbidq.html?CG8360), [imd](http://flybase.bio.indiana.edu/.bin/fbidq.html?imd), [CG1702](http://flybase.bio.indiana.edu/.bin/fbidq.html?CG1702), [CG3267](http://flybase.bio.indiana.edu/.bin/fbidq.html?CG3267), [GstE1](http://flybase.bio.indiana.edu/.bin/fbidq.html?GstE1), [l(3)02640](http://flybase.bio.indiana.edu/.bin/fbidq.html?l(3)02640), [PH4alphaEFB](http://flybase.bio.indiana.edu/.bin/fbidq.html?PH4alphaEFB), [CG10184](http://flybase.bio.indiana.edu/.bin/fbidq.html?CG10184), [Idgf2](http://flybase.bio.indiana.edu/.bin/fbidq.html?Idgf2), [Coprox](http://flybase.bio.indiana.edu/.bin/fbidq.html?Coprox), [CG2118](http://flybase.bio.indiana.edu/.bin/fbidq.html?CG2118), [CG5210](http://flybase.bio.indiana.edu/.bin/fbidq.html?CG5210), [GstD1](http://flybase.bio.indiana.edu/.bin/fbidq.html?GstD1), [Ced-12](http://flybase.bio.indiana.edu/.bin/fbidq.html?Ced-12), [Pbgs](http://flybase.bio.indiana.edu/.bin/fbidq.html?Pbgs), [CG3376](http://flybase.bio.indiana.edu/.bin/fbidq.html?CG3376), [BG4](http://flybase.bio.indiana.edu/.bin/fbidq.html?BG4), [Gtp-bp](http://flybase.bio.indiana.edu/.bin/fbidq.html?Gtp-bp), [Pgd](http://flybase.bio.indiana.edu/.bin/fbidq.html?Pgd), [Idgf3](http://flybase.bio.indiana.edu/.bin/fbidq.html?Idgf3), [Aprt](http://flybase.bio.indiana.edu/.bin/fbidq.html?Aprt), [Ppat-Dpck](http://flybase.bio.indiana.edu/.bin/fbidq.html?Ppat-Dpck), [ttv](http://flybase.bio.indiana.edu/.bin/fbidq.html?ttv), [CG30410](http://flybase.bio.indiana.edu/.bin/fbidq.html?CG30410), [CG6726](http://flybase.bio.indiana.edu/.bin/fbidq.html?CG6726),[CG10361](http://flybase.bio.indiana.edu/.bin/fbidq.html?CG10361), [CG30499](http://flybase.bio.indiana.edu/.bin/fbidq.html?CG30499), [Gclc](http://flybase.bio.indiana.edu/.bin/fbidq.html?Gclc), [CG10399](http://flybase.bio.indiana.edu/.bin/fbidq.html?CG10399), [Aats-cys](http://flybase.bio.indiana.edu/.bin/fbidq.html?Aats-cys), [CG4306](http://flybase.bio.indiana.edu/.bin/fbidq.html?CG4306), [Vha68-2](http://flybase.bio.indiana.edu/.bin/fbidq.html?Vha68-2), [GstE2](http://flybase.bio.indiana.edu/.bin/fbidq.html?GstE2), [CG17224](http://flybase.bio.indiana.edu/.bin/fbidq.html?CG17224), [CG1673](http://flybase.bio.indiana.edu/.bin/fbidq.html?CG1673), [GNBP1](http://flybase.bio.indiana.edu/.bin/fbidq.html?GNBP1), [CG15093](http://flybase.bio.indiana.edu/.bin/fbidq.html?CG15093), [betaTub60D](http://flybase.bio.indiana.edu/.bin/fbidq.html?betaTub60D), [GstD9](http://flybase.bio.indiana.edu/.bin/fbidq.html?GstD9), [G-ialpha65A](http://flybase.bio.indiana.edu/.bin/fbidq.html?G-ialpha65A), [Mical](http://flybase.bio.indiana.edu/.bin/fbidq.html?Mical), [PGRP-SA](http://flybase.bio.indiana.edu/.bin/fbidq.html?PGRP-SA), [CG16936](http://flybase.bio.indiana.edu/.bin/fbidq.html?CG16936), [Idgf4](http://flybase.bio.indiana.edu/.bin/fbidq.html?Idgf4), [CG7997](http://flybase.bio.indiana.edu/.bin/fbidq.html?CG7997), [CDase](http://flybase.bio.indiana.edu/.bin/fbidq.html?CDase), [GstE3](http://flybase.bio.indiana.edu/.bin/fbidq.html?GstE3), [spz](http://flybase.bio.indiana.edu/.bin/fbidq.html?spz), [CG7433](http://flybase.bio.indiana.edu/.bin/fbidq.html?CG7433), [CG17333](http://flybase.bio.indiana.edu/.bin/fbidq.html?CG17333),[betaTub97EF](http://flybase.bio.indiana.edu/.bin/fbidq.html?betaTub97EF), [ade5](http://flybase.bio.indiana.edu/.bin/fbidq.html?ade5), [Prat](http://flybase.bio.indiana.edu/.bin/fbidq.html?Prat), [Tal](http://flybase.bio.indiana.edu/.bin/fbidq.html?Tal), [mbc](http://flybase.bio.indiana.edu/.bin/fbidq.html?mbc), [Cht3](http://flybase.bio.indiana.edu/.bin/fbidq.html?Cht3), [Atpalpha](http://flybase.bio.indiana.edu/.bin/fbidq.html?Atpalpha), [PGRP-LB](http://flybase.bio.indiana.edu/.bin/fbidq.html?PGRP-LB), [GstS1](http://flybase.bio.indiana.edu/.bin/fbidq.html?GstS1), [Pax](http://flybase.bio.indiana.edu/.bin/fbidq.html?Pax), [GstE7](http://flybase.bio.indiana.edu/.bin/fbidq.html?GstE7), [CG30005](http://flybase.bio.indiana.edu/.bin/fbidq.html?CG30005), [Oat](http://flybase.bio.indiana.edu/.bin/fbidq.html?Oat) |
| [single-organism transport](http://amigo.geneontology.org/cgi-bin/amigo/go.cgi?view=details&query=GO:0044765) | 166 of 1000 genes, 16.6% | 898 of 7634 genes, 11.8% | 0.00019 | 0.00% | 0.00 | [CG16791](http://flybase.bio.indiana.edu/.bin/fbidq.html?CG16791), [CG10960](http://flybase.bio.indiana.edu/.bin/fbidq.html?CG10960), [CG14040](http://flybase.bio.indiana.edu/.bin/fbidq.html?CG14040), [Ir](http://flybase.bio.indiana.edu/.bin/fbidq.html?Ir), [AnnIX](http://flybase.bio.indiana.edu/.bin/fbidq.html?AnnIX), [JhI-21](http://flybase.bio.indiana.edu/.bin/fbidq.html?JhI-21), [hoe1](http://flybase.bio.indiana.edu/.bin/fbidq.html?hoe1), [scb](http://flybase.bio.indiana.edu/.bin/fbidq.html?scb), [pll](http://flybase.bio.indiana.edu/.bin/fbidq.html?pll), [CG7342](http://flybase.bio.indiana.edu/.bin/fbidq.html?CG7342), [Arf51F](http://flybase.bio.indiana.edu/.bin/fbidq.html?Arf51F), [Lsd-2](http://flybase.bio.indiana.edu/.bin/fbidq.html?Lsd-2), [membrin](http://flybase.bio.indiana.edu/.bin/fbidq.html?membrin), [Efr](http://flybase.bio.indiana.edu/.bin/fbidq.html?Efr), [CG31150](http://flybase.bio.indiana.edu/.bin/fbidq.html?CG31150), [Su(fu)](http://flybase.bio.indiana.edu/.bin/fbidq.html?Su(fu)), [CG5002](http://flybase.bio.indiana.edu/.bin/fbidq.html?CG5002), [Mcr](http://flybase.bio.indiana.edu/.bin/fbidq.html?Mcr), [mnd](http://flybase.bio.indiana.edu/.bin/fbidq.html?mnd), [CG10226](http://flybase.bio.indiana.edu/.bin/fbidq.html?CG10226), [CG6812](http://flybase.bio.indiana.edu/.bin/fbidq.html?CG6812), [CG1732](http://flybase.bio.indiana.edu/.bin/fbidq.html?CG1732), [svr](http://flybase.bio.indiana.edu/.bin/fbidq.html?svr), [CG10006](http://flybase.bio.indiana.edu/.bin/fbidq.html?CG10006), [CG11655](http://flybase.bio.indiana.edu/.bin/fbidq.html?CG11655), [CaBP1](http://flybase.bio.indiana.edu/.bin/fbidq.html?CaBP1), [Rac2](http://flybase.bio.indiana.edu/.bin/fbidq.html?Rac2), [Ced-12](http://flybase.bio.indiana.edu/.bin/fbidq.html?Ced-12), [CG1607](http://flybase.bio.indiana.edu/.bin/fbidq.html?CG1607),[CG5599](http://flybase.bio.indiana.edu/.bin/fbidq.html?CG5599), [Dscam](http://flybase.bio.indiana.edu/.bin/fbidq.html?Dscam), [mth](http://flybase.bio.indiana.edu/.bin/fbidq.html?mth), [gbb](http://flybase.bio.indiana.edu/.bin/fbidq.html?gbb), [Srp54k](http://flybase.bio.indiana.edu/.bin/fbidq.html?Srp54k), [Ppat-Dpck](http://flybase.bio.indiana.edu/.bin/fbidq.html?Ppat-Dpck), [kar](http://flybase.bio.indiana.edu/.bin/fbidq.html?kar), [ttv](http://flybase.bio.indiana.edu/.bin/fbidq.html?ttv), [CG42235](http://flybase.bio.indiana.edu/.bin/fbidq.html?CG42235), [Amph](http://flybase.bio.indiana.edu/.bin/fbidq.html?Amph), [Mtp](http://flybase.bio.indiana.edu/.bin/fbidq.html?Mtp), [Scamp](http://flybase.bio.indiana.edu/.bin/fbidq.html?Scamp), [Oatp58Dc](http://flybase.bio.indiana.edu/.bin/fbidq.html?Oatp58Dc), [VhaM9.7-c](http://flybase.bio.indiana.edu/.bin/fbidq.html?VhaM9.7-c), [Sec61alpha](http://flybase.bio.indiana.edu/.bin/fbidq.html?Sec61alpha), [nrv1](http://flybase.bio.indiana.edu/.bin/fbidq.html?nrv1), [CG12918](http://flybase.bio.indiana.edu/.bin/fbidq.html?CG12918), [colt](http://flybase.bio.indiana.edu/.bin/fbidq.html?colt), [Rfabg](http://flybase.bio.indiana.edu/.bin/fbidq.html?Rfabg), [NFAT](http://flybase.bio.indiana.edu/.bin/fbidq.html?NFAT), [Atox1](http://flybase.bio.indiana.edu/.bin/fbidq.html?Atox1), [Orct](http://flybase.bio.indiana.edu/.bin/fbidq.html?Orct), [CG8602](http://flybase.bio.indiana.edu/.bin/fbidq.html?CG8602), [CG14511](http://flybase.bio.indiana.edu/.bin/fbidq.html?CG14511), [Syb](http://flybase.bio.indiana.edu/.bin/fbidq.html?Syb), [CG9523](http://flybase.bio.indiana.edu/.bin/fbidq.html?CG9523), [CD98hc](http://flybase.bio.indiana.edu/.bin/fbidq.html?CD98hc), [CG8790](http://flybase.bio.indiana.edu/.bin/fbidq.html?CG8790), [Picot](http://flybase.bio.indiana.edu/.bin/fbidq.html?Picot), [Npc2b](http://flybase.bio.indiana.edu/.bin/fbidq.html?Npc2b),[CG16700](http://flybase.bio.indiana.edu/.bin/fbidq.html?CG16700), [Irk2](http://flybase.bio.indiana.edu/.bin/fbidq.html?Irk2), [ClC-c](http://flybase.bio.indiana.edu/.bin/fbidq.html?ClC-c), [Npc1a](http://flybase.bio.indiana.edu/.bin/fbidq.html?Npc1a), [CG15890](http://flybase.bio.indiana.edu/.bin/fbidq.html?CG15890), [NKAIN](http://flybase.bio.indiana.edu/.bin/fbidq.html?NKAIN), [Aph-4](http://flybase.bio.indiana.edu/.bin/fbidq.html?Aph-4), [trpm](http://flybase.bio.indiana.edu/.bin/fbidq.html?trpm), [CG12344](http://flybase.bio.indiana.edu/.bin/fbidq.html?CG12344), [CG6723](http://flybase.bio.indiana.edu/.bin/fbidq.html?CG6723), [Vha100-2](http://flybase.bio.indiana.edu/.bin/fbidq.html?Vha100-2), [CG9449](http://flybase.bio.indiana.edu/.bin/fbidq.html?CG9449), [CG7149](http://flybase.bio.indiana.edu/.bin/fbidq.html?CG7149), [gb](http://flybase.bio.indiana.edu/.bin/fbidq.html?gb), [CG7777](http://flybase.bio.indiana.edu/.bin/fbidq.html?CG7777), [spin](http://flybase.bio.indiana.edu/.bin/fbidq.html?spin), [Eaat1](http://flybase.bio.indiana.edu/.bin/fbidq.html?Eaat1), [SrpRbeta](http://flybase.bio.indiana.edu/.bin/fbidq.html?SrpRbeta), [CanB2](http://flybase.bio.indiana.edu/.bin/fbidq.html?CanB2), [loco](http://flybase.bio.indiana.edu/.bin/fbidq.html?loco), [Atpalpha](http://flybase.bio.indiana.edu/.bin/fbidq.html?Atpalpha), [Tig](http://flybase.bio.indiana.edu/.bin/fbidq.html?Tig), [CG5885](http://flybase.bio.indiana.edu/.bin/fbidq.html?CG5885), [CG31229](http://flybase.bio.indiana.edu/.bin/fbidq.html?CG31229), [bib](http://flybase.bio.indiana.edu/.bin/fbidq.html?bib), [Sur](http://flybase.bio.indiana.edu/.bin/fbidq.html?Sur), [CG5130](http://flybase.bio.indiana.edu/.bin/fbidq.html?CG5130), [CG9053](http://flybase.bio.indiana.edu/.bin/fbidq.html?CG9053), [ClC-a](http://flybase.bio.indiana.edu/.bin/fbidq.html?ClC-a), [cert](http://flybase.bio.indiana.edu/.bin/fbidq.html?cert),[Npc2a](http://flybase.bio.indiana.edu/.bin/fbidq.html?Npc2a), [Vinc](http://flybase.bio.indiana.edu/.bin/fbidq.html?Vinc), [Ent2](http://flybase.bio.indiana.edu/.bin/fbidq.html?Ent2), [kel](http://flybase.bio.indiana.edu/.bin/fbidq.html?kel), [Ctr1A](http://flybase.bio.indiana.edu/.bin/fbidq.html?Ctr1A), [CG3036](http://flybase.bio.indiana.edu/.bin/fbidq.html?CG3036), [CG17119](http://flybase.bio.indiana.edu/.bin/fbidq.html?CG17119), [HLH106](http://flybase.bio.indiana.edu/.bin/fbidq.html?HLH106), [drpr](http://flybase.bio.indiana.edu/.bin/fbidq.html?drpr), [CG31729](http://flybase.bio.indiana.edu/.bin/fbidq.html?CG31729), [pnt](http://flybase.bio.indiana.edu/.bin/fbidq.html?pnt), [VhaPPA1-1](http://flybase.bio.indiana.edu/.bin/fbidq.html?VhaPPA1-1), [Orct2](http://flybase.bio.indiana.edu/.bin/fbidq.html?Orct2), [ine](http://flybase.bio.indiana.edu/.bin/fbidq.html?ine), [CG6672](http://flybase.bio.indiana.edu/.bin/fbidq.html?CG6672), [w](http://flybase.bio.indiana.edu/.bin/fbidq.html?w), [CG3168](http://flybase.bio.indiana.edu/.bin/fbidq.html?CG3168), [CG10444](http://flybase.bio.indiana.edu/.bin/fbidq.html?CG10444), [Indy](http://flybase.bio.indiana.edu/.bin/fbidq.html?Indy), [btsz](http://flybase.bio.indiana.edu/.bin/fbidq.html?btsz), [Mdr65](http://flybase.bio.indiana.edu/.bin/fbidq.html?Mdr65), [Itp-r83A](http://flybase.bio.indiana.edu/.bin/fbidq.html?Itp-r83A), [debcl](http://flybase.bio.indiana.edu/.bin/fbidq.html?debcl), [Mer](http://flybase.bio.indiana.edu/.bin/fbidq.html?Mer), [Syx4](http://flybase.bio.indiana.edu/.bin/fbidq.html?Syx4), [Irk3](http://flybase.bio.indiana.edu/.bin/fbidq.html?Irk3), [Tsf2](http://flybase.bio.indiana.edu/.bin/fbidq.html?Tsf2), [aralar1](http://flybase.bio.indiana.edu/.bin/fbidq.html?aralar1), [CG15438](http://flybase.bio.indiana.edu/.bin/fbidq.html?CG15438), [VhaAC39-1](http://flybase.bio.indiana.edu/.bin/fbidq.html?VhaAC39-1), [CG1213](http://flybase.bio.indiana.edu/.bin/fbidq.html?CG1213), [Gtp-bp](http://flybase.bio.indiana.edu/.bin/fbidq.html?Gtp-bp), [CG10069](http://flybase.bio.indiana.edu/.bin/fbidq.html?CG10069), [CG1907](http://flybase.bio.indiana.edu/.bin/fbidq.html?CG1907), [Fatp](http://flybase.bio.indiana.edu/.bin/fbidq.html?Fatp), [CG2893](http://flybase.bio.indiana.edu/.bin/fbidq.html?CG2893), [glob1](http://flybase.bio.indiana.edu/.bin/fbidq.html?glob1), [pain](http://flybase.bio.indiana.edu/.bin/fbidq.html?pain), [zetaCOP](http://flybase.bio.indiana.edu/.bin/fbidq.html?zetaCOP), [CG15094](http://flybase.bio.indiana.edu/.bin/fbidq.html?CG15094), [pyx](http://flybase.bio.indiana.edu/.bin/fbidq.html?pyx), [CG8596](http://flybase.bio.indiana.edu/.bin/fbidq.html?CG8596), [ced-6](http://flybase.bio.indiana.edu/.bin/fbidq.html?ced-6), [CHOp24](http://flybase.bio.indiana.edu/.bin/fbidq.html?CHOp24), [CG33635](http://flybase.bio.indiana.edu/.bin/fbidq.html?CG33635), [CG6356](http://flybase.bio.indiana.edu/.bin/fbidq.html?CG6356), [CG11110](http://flybase.bio.indiana.edu/.bin/fbidq.html?CG11110), [Vha68-2](http://flybase.bio.indiana.edu/.bin/fbidq.html?Vha68-2), [CG11163](http://flybase.bio.indiana.edu/.bin/fbidq.html?CG11163), [VhaM9.7-b](http://flybase.bio.indiana.edu/.bin/fbidq.html?VhaM9.7-b), [jagn](http://flybase.bio.indiana.edu/.bin/fbidq.html?jagn), [eca](http://flybase.bio.indiana.edu/.bin/fbidq.html?eca), [CG4288](http://flybase.bio.indiana.edu/.bin/fbidq.html?CG4288), [nrv2](http://flybase.bio.indiana.edu/.bin/fbidq.html?nrv2), [TepII](http://flybase.bio.indiana.edu/.bin/fbidq.html?TepII), [VhaM8.9](http://flybase.bio.indiana.edu/.bin/fbidq.html?VhaM8.9), [CG4484](http://flybase.bio.indiana.edu/.bin/fbidq.html?CG4484), [CG7816](http://flybase.bio.indiana.edu/.bin/fbidq.html?CG7816), [CG4301](http://flybase.bio.indiana.edu/.bin/fbidq.html?CG4301),[Oatp33Ea](http://flybase.bio.indiana.edu/.bin/fbidq.html?Oatp33Ea), [CDase](http://flybase.bio.indiana.edu/.bin/fbidq.html?CDase), [ScpX](http://flybase.bio.indiana.edu/.bin/fbidq.html?ScpX), [Gpdh](http://flybase.bio.indiana.edu/.bin/fbidq.html?Gpdh), [CG6125](http://flybase.bio.indiana.edu/.bin/fbidq.html?CG6125), [Tango5](http://flybase.bio.indiana.edu/.bin/fbidq.html?Tango5), [CG5802](http://flybase.bio.indiana.edu/.bin/fbidq.html?CG5802), [wtrw](http://flybase.bio.indiana.edu/.bin/fbidq.html?wtrw), [TRAM](http://flybase.bio.indiana.edu/.bin/fbidq.html?TRAM), [CG6293](http://flybase.bio.indiana.edu/.bin/fbidq.html?CG6293), [p24-1](http://flybase.bio.indiana.edu/.bin/fbidq.html?p24-1), [BM-40-SPARC](http://flybase.bio.indiana.edu/.bin/fbidq.html?BM-40-SPARC), [CG1628](http://flybase.bio.indiana.edu/.bin/fbidq.html?CG1628), [Ork1](http://flybase.bio.indiana.edu/.bin/fbidq.html?Ork1), [CG10420](http://flybase.bio.indiana.edu/.bin/fbidq.html?CG10420), [yin](http://flybase.bio.indiana.edu/.bin/fbidq.html?yin), [KdelR](http://flybase.bio.indiana.edu/.bin/fbidq.html?KdelR), [shark](http://flybase.bio.indiana.edu/.bin/fbidq.html?shark) |
| [anion transport](http://amigo.geneontology.org/cgi-bin/amigo/go.cgi?view=details&query=GO:0006820) | 25 of 1000 genes, 2.5% | 66 of 7634 genes, 0.9% | 0.00032 | 0.00% | 0.00 | [CG15094](http://flybase.bio.indiana.edu/.bin/fbidq.html?CG15094), [Efr](http://flybase.bio.indiana.edu/.bin/fbidq.html?Efr), [Oatp58Dc](http://flybase.bio.indiana.edu/.bin/fbidq.html?Oatp58Dc), [ClC-a](http://flybase.bio.indiana.edu/.bin/fbidq.html?ClC-a), [CG5002](http://flybase.bio.indiana.edu/.bin/fbidq.html?CG5002), [colt](http://flybase.bio.indiana.edu/.bin/fbidq.html?colt), [mnd](http://flybase.bio.indiana.edu/.bin/fbidq.html?mnd), [CG6125](http://flybase.bio.indiana.edu/.bin/fbidq.html?CG6125), [CG3036](http://flybase.bio.indiana.edu/.bin/fbidq.html?CG3036), [gb](http://flybase.bio.indiana.edu/.bin/fbidq.html?gb), [CG17119](http://flybase.bio.indiana.edu/.bin/fbidq.html?CG17119), [CG1607](http://flybase.bio.indiana.edu/.bin/fbidq.html?CG1607), [Eaat1](http://flybase.bio.indiana.edu/.bin/fbidq.html?Eaat1), [JhI-21](http://flybase.bio.indiana.edu/.bin/fbidq.html?JhI-21), [hoe1](http://flybase.bio.indiana.edu/.bin/fbidq.html?hoe1), [CG1628](http://flybase.bio.indiana.edu/.bin/fbidq.html?CG1628), [w](http://flybase.bio.indiana.edu/.bin/fbidq.html?w), [CD98hc](http://flybase.bio.indiana.edu/.bin/fbidq.html?CD98hc), [CG8790](http://flybase.bio.indiana.edu/.bin/fbidq.html?CG8790), [CG1907](http://flybase.bio.indiana.edu/.bin/fbidq.html?CG1907), [Picot](http://flybase.bio.indiana.edu/.bin/fbidq.html?Picot), [Fatp](http://flybase.bio.indiana.edu/.bin/fbidq.html?Fatp), [CG16700](http://flybase.bio.indiana.edu/.bin/fbidq.html?CG16700), [Oatp33Ea](http://flybase.bio.indiana.edu/.bin/fbidq.html?Oatp33Ea), [ClC-c](http://flybase.bio.indiana.edu/.bin/fbidq.html?ClC-c) |
| [cellular amino acid metabolic process](http://amigo.geneontology.org/cgi-bin/amigo/go.cgi?view=details&query=GO:0006520) | 39 of 1000 genes, 3.9% | 132 of 7634 genes, 1.7% | 0.00037 | 0.00% | 0.00 | [Gclc](http://flybase.bio.indiana.edu/.bin/fbidq.html?Gclc), [CG9362](http://flybase.bio.indiana.edu/.bin/fbidq.html?CG9362), [Eip55E](http://flybase.bio.indiana.edu/.bin/fbidq.html?Eip55E), [Got1](http://flybase.bio.indiana.edu/.bin/fbidq.html?Got1), [CG10399](http://flybase.bio.indiana.edu/.bin/fbidq.html?CG10399), [CG4306](http://flybase.bio.indiana.edu/.bin/fbidq.html?CG4306), [Aats-cys](http://flybase.bio.indiana.edu/.bin/fbidq.html?Aats-cys), [Nmdmc](http://flybase.bio.indiana.edu/.bin/fbidq.html?Nmdmc), [GstE2](http://flybase.bio.indiana.edu/.bin/fbidq.html?GstE2), [CG9547](http://flybase.bio.indiana.edu/.bin/fbidq.html?CG9547), [ry](http://flybase.bio.indiana.edu/.bin/fbidq.html?ry), [CG1673](http://flybase.bio.indiana.edu/.bin/fbidq.html?CG1673), [CG15093](http://flybase.bio.indiana.edu/.bin/fbidq.html?CG15093), [CG30000](http://flybase.bio.indiana.edu/.bin/fbidq.html?CG30000), [GstD9](http://flybase.bio.indiana.edu/.bin/fbidq.html?GstD9), [CG11784](http://flybase.bio.indiana.edu/.bin/fbidq.html?CG11784), [asparagine-synthetase](http://flybase.bio.indiana.edu/.bin/fbidq.html?asparagine-synthetase), [CG17896](http://flybase.bio.indiana.edu/.bin/fbidq.html?CG17896), [CG4829](http://flybase.bio.indiana.edu/.bin/fbidq.html?CG4829), [GstE6](http://flybase.bio.indiana.edu/.bin/fbidq.html?GstE6), [Ssadh](http://flybase.bio.indiana.edu/.bin/fbidq.html?Ssadh), [CG16936](http://flybase.bio.indiana.edu/.bin/fbidq.html?CG16936), [GstD2](http://flybase.bio.indiana.edu/.bin/fbidq.html?GstD2), [CG6638](http://flybase.bio.indiana.edu/.bin/fbidq.html?CG6638), [GstE3](http://flybase.bio.indiana.edu/.bin/fbidq.html?GstE3),[CG7433](http://flybase.bio.indiana.edu/.bin/fbidq.html?CG7433), [CG1702](http://flybase.bio.indiana.edu/.bin/fbidq.html?CG1702), [CG3267](http://flybase.bio.indiana.edu/.bin/fbidq.html?CG3267), [GstE1](http://flybase.bio.indiana.edu/.bin/fbidq.html?GstE1), [PH4alphaEFB](http://flybase.bio.indiana.edu/.bin/fbidq.html?PH4alphaEFB), [CG10184](http://flybase.bio.indiana.edu/.bin/fbidq.html?CG10184), [CG2118](http://flybase.bio.indiana.edu/.bin/fbidq.html?CG2118), [GstD1](http://flybase.bio.indiana.edu/.bin/fbidq.html?GstD1), [GstS1](http://flybase.bio.indiana.edu/.bin/fbidq.html?GstS1), [GstE7](http://flybase.bio.indiana.edu/.bin/fbidq.html?GstE7), [CG10361](http://flybase.bio.indiana.edu/.bin/fbidq.html?CG10361), [CG6726](http://flybase.bio.indiana.edu/.bin/fbidq.html?CG6726), [CG30005](http://flybase.bio.indiana.edu/.bin/fbidq.html?CG30005), [Oat](http://flybase.bio.indiana.edu/.bin/fbidq.html?Oat) |
| [establishment of blood-brain barrier](http://amigo.geneontology.org/cgi-bin/amigo/go.cgi?view=details&query=GO:0060856) | 11 of 1000 genes, 1.1% | 16 of 7634 genes, 0.2% | 0.00055 | 0.00% | 0.00 | [Cont](http://flybase.bio.indiana.edu/.bin/fbidq.html?Cont), [sinu](http://flybase.bio.indiana.edu/.bin/fbidq.html?sinu), [Nrg](http://flybase.bio.indiana.edu/.bin/fbidq.html?Nrg), [kune](http://flybase.bio.indiana.edu/.bin/fbidq.html?kune), [moody](http://flybase.bio.indiana.edu/.bin/fbidq.html?moody), [G-ialpha65A](http://flybase.bio.indiana.edu/.bin/fbidq.html?G-ialpha65A), [cold](http://flybase.bio.indiana.edu/.bin/fbidq.html?cold), [Mdr65](http://flybase.bio.indiana.edu/.bin/fbidq.html?Mdr65), [nrv2](http://flybase.bio.indiana.edu/.bin/fbidq.html?nrv2), [loco](http://flybase.bio.indiana.edu/.bin/fbidq.html?loco), [pck](http://flybase.bio.indiana.edu/.bin/fbidq.html?pck) |
| [apical junction assembly](http://amigo.geneontology.org/cgi-bin/amigo/go.cgi?view=details&query=GO:0043297) | 17 of 1000 genes, 1.7% | 36 of 7634 genes, 0.5% | 0.00074 | 0.00% | 0.00 | [crok](http://flybase.bio.indiana.edu/.bin/fbidq.html?crok), [Cont](http://flybase.bio.indiana.edu/.bin/fbidq.html?Cont), [sinu](http://flybase.bio.indiana.edu/.bin/fbidq.html?sinu), [Nrg](http://flybase.bio.indiana.edu/.bin/fbidq.html?Nrg), [btsz](http://flybase.bio.indiana.edu/.bin/fbidq.html?btsz), [kune](http://flybase.bio.indiana.edu/.bin/fbidq.html?kune), [moody](http://flybase.bio.indiana.edu/.bin/fbidq.html?moody), [vari](http://flybase.bio.indiana.edu/.bin/fbidq.html?vari), [G-ialpha65A](http://flybase.bio.indiana.edu/.bin/fbidq.html?G-ialpha65A), [crim](http://flybase.bio.indiana.edu/.bin/fbidq.html?crim), [cold](http://flybase.bio.indiana.edu/.bin/fbidq.html?cold), [wun](http://flybase.bio.indiana.edu/.bin/fbidq.html?wun), [nrv2](http://flybase.bio.indiana.edu/.bin/fbidq.html?nrv2), [loco](http://flybase.bio.indiana.edu/.bin/fbidq.html?loco), [Atpalpha](http://flybase.bio.indiana.edu/.bin/fbidq.html?Atpalpha), [Tsf2](http://flybase.bio.indiana.edu/.bin/fbidq.html?Tsf2), [pck](http://flybase.bio.indiana.edu/.bin/fbidq.html?pck) |
| [cell-cell junction assembly](http://amigo.geneontology.org/cgi-bin/amigo/go.cgi?view=details&query=GO:0007043) | 18 of 1000 genes, 1.8% | 40 of 7634 genes, 0.5% | 0.00083 | 0.00% | 0.00 | [crok](http://flybase.bio.indiana.edu/.bin/fbidq.html?crok), [Cont](http://flybase.bio.indiana.edu/.bin/fbidq.html?Cont), [sinu](http://flybase.bio.indiana.edu/.bin/fbidq.html?sinu), [btsz](http://flybase.bio.indiana.edu/.bin/fbidq.html?btsz), [cold](http://flybase.bio.indiana.edu/.bin/fbidq.html?cold), [Tsf2](http://flybase.bio.indiana.edu/.bin/fbidq.html?Tsf2), [pck](http://flybase.bio.indiana.edu/.bin/fbidq.html?pck), [Nrg](http://flybase.bio.indiana.edu/.bin/fbidq.html?Nrg), [moody](http://flybase.bio.indiana.edu/.bin/fbidq.html?moody), [kune](http://flybase.bio.indiana.edu/.bin/fbidq.html?kune), [G-ialpha65A](http://flybase.bio.indiana.edu/.bin/fbidq.html?G-ialpha65A), [vari](http://flybase.bio.indiana.edu/.bin/fbidq.html?vari), [crim](http://flybase.bio.indiana.edu/.bin/fbidq.html?crim), [nrv2](http://flybase.bio.indiana.edu/.bin/fbidq.html?nrv2), [wun](http://flybase.bio.indiana.edu/.bin/fbidq.html?wun), [Atpalpha](http://flybase.bio.indiana.edu/.bin/fbidq.html?Atpalpha), [loco](http://flybase.bio.indiana.edu/.bin/fbidq.html?loco), [pyd](http://flybase.bio.indiana.edu/.bin/fbidq.html?pyd) |
| [branched-chain amino acid metabolic process](http://amigo.geneontology.org/cgi-bin/amigo/go.cgi?view=details&query=GO:0009081) | 7 of 1000 genes, 0.7% | 7 of 7634 genes, 0.1% | 0.00090 | 0.00% | 0.00 | [CG17896](http://flybase.bio.indiana.edu/.bin/fbidq.html?CG17896), [CG6638](http://flybase.bio.indiana.edu/.bin/fbidq.html?CG6638), [CG1673](http://flybase.bio.indiana.edu/.bin/fbidq.html?CG1673), [CG15093](http://flybase.bio.indiana.edu/.bin/fbidq.html?CG15093), [CG2118](http://flybase.bio.indiana.edu/.bin/fbidq.html?CG2118), [CG10399](http://flybase.bio.indiana.edu/.bin/fbidq.html?CG10399), [CG3267](http://flybase.bio.indiana.edu/.bin/fbidq.html?CG3267) |
| [transport](http://amigo.geneontology.org/cgi-bin/amigo/go.cgi?view=details&query=GO:0006810) | 183 of 1000 genes, 18.3% | 1036 of 7634 genes, 13.6% | 0.00109 | 0.00% | 0.00 | [CG16791](http://flybase.bio.indiana.edu/.bin/fbidq.html?CG16791), [CG10960](http://flybase.bio.indiana.edu/.bin/fbidq.html?CG10960), [CG14040](http://flybase.bio.indiana.edu/.bin/fbidq.html?CG14040), [Ppt1](http://flybase.bio.indiana.edu/.bin/fbidq.html?Ppt1), [Ir](http://flybase.bio.indiana.edu/.bin/fbidq.html?Ir), [AnnIX](http://flybase.bio.indiana.edu/.bin/fbidq.html?AnnIX), [JhI-21](http://flybase.bio.indiana.edu/.bin/fbidq.html?JhI-21), [hoe1](http://flybase.bio.indiana.edu/.bin/fbidq.html?hoe1), [scb](http://flybase.bio.indiana.edu/.bin/fbidq.html?scb), [pll](http://flybase.bio.indiana.edu/.bin/fbidq.html?pll), [CG7342](http://flybase.bio.indiana.edu/.bin/fbidq.html?CG7342), [Arf51F](http://flybase.bio.indiana.edu/.bin/fbidq.html?Arf51F), [Lsd-2](http://flybase.bio.indiana.edu/.bin/fbidq.html?Lsd-2), [membrin](http://flybase.bio.indiana.edu/.bin/fbidq.html?membrin), [Efr](http://flybase.bio.indiana.edu/.bin/fbidq.html?Efr), [CG31150](http://flybase.bio.indiana.edu/.bin/fbidq.html?CG31150), [Su(fu)](http://flybase.bio.indiana.edu/.bin/fbidq.html?Su(fu)), [CG5002](http://flybase.bio.indiana.edu/.bin/fbidq.html?CG5002), [Sop2](http://flybase.bio.indiana.edu/.bin/fbidq.html?Sop2), [Mcr](http://flybase.bio.indiana.edu/.bin/fbidq.html?Mcr), [mnd](http://flybase.bio.indiana.edu/.bin/fbidq.html?mnd), [CG10226](http://flybase.bio.indiana.edu/.bin/fbidq.html?CG10226), [CG6812](http://flybase.bio.indiana.edu/.bin/fbidq.html?CG6812), [CG1732](http://flybase.bio.indiana.edu/.bin/fbidq.html?CG1732), [svr](http://flybase.bio.indiana.edu/.bin/fbidq.html?svr), [CG10006](http://flybase.bio.indiana.edu/.bin/fbidq.html?CG10006), [CG11655](http://flybase.bio.indiana.edu/.bin/fbidq.html?CG11655), [CaBP1](http://flybase.bio.indiana.edu/.bin/fbidq.html?CaBP1), [loj](http://flybase.bio.indiana.edu/.bin/fbidq.html?loj), [Rac2](http://flybase.bio.indiana.edu/.bin/fbidq.html?Rac2), [Ced-12](http://flybase.bio.indiana.edu/.bin/fbidq.html?Ced-12),[CG1607](http://flybase.bio.indiana.edu/.bin/fbidq.html?CG1607), [CG5599](http://flybase.bio.indiana.edu/.bin/fbidq.html?CG5599), [Dscam](http://flybase.bio.indiana.edu/.bin/fbidq.html?Dscam), [bai](http://flybase.bio.indiana.edu/.bin/fbidq.html?bai), [mth](http://flybase.bio.indiana.edu/.bin/fbidq.html?mth), [gbb](http://flybase.bio.indiana.edu/.bin/fbidq.html?gbb), [Srp54k](http://flybase.bio.indiana.edu/.bin/fbidq.html?Srp54k), [Ppat-Dpck](http://flybase.bio.indiana.edu/.bin/fbidq.html?Ppat-Dpck), [kar](http://flybase.bio.indiana.edu/.bin/fbidq.html?kar), [ttv](http://flybase.bio.indiana.edu/.bin/fbidq.html?ttv), [Amph](http://flybase.bio.indiana.edu/.bin/fbidq.html?Amph), [CG42235](http://flybase.bio.indiana.edu/.bin/fbidq.html?CG42235), [Mtp](http://flybase.bio.indiana.edu/.bin/fbidq.html?Mtp), [Scamp](http://flybase.bio.indiana.edu/.bin/fbidq.html?Scamp), [Oatp58Dc](http://flybase.bio.indiana.edu/.bin/fbidq.html?Oatp58Dc), [VhaM9.7-c](http://flybase.bio.indiana.edu/.bin/fbidq.html?VhaM9.7-c), [Sec61alpha](http://flybase.bio.indiana.edu/.bin/fbidq.html?Sec61alpha), [nrv1](http://flybase.bio.indiana.edu/.bin/fbidq.html?nrv1), [CG12918](http://flybase.bio.indiana.edu/.bin/fbidq.html?CG12918), [colt](http://flybase.bio.indiana.edu/.bin/fbidq.html?colt), [Rfabg](http://flybase.bio.indiana.edu/.bin/fbidq.html?Rfabg), [CG3091](http://flybase.bio.indiana.edu/.bin/fbidq.html?CG3091), [NFAT](http://flybase.bio.indiana.edu/.bin/fbidq.html?NFAT), [Atox1](http://flybase.bio.indiana.edu/.bin/fbidq.html?Atox1), [Orct](http://flybase.bio.indiana.edu/.bin/fbidq.html?Orct), [CG8602](http://flybase.bio.indiana.edu/.bin/fbidq.html?CG8602), [CG14511](http://flybase.bio.indiana.edu/.bin/fbidq.html?CG14511), [Syb](http://flybase.bio.indiana.edu/.bin/fbidq.html?Syb), [CG9523](http://flybase.bio.indiana.edu/.bin/fbidq.html?CG9523), [CG10383](http://flybase.bio.indiana.edu/.bin/fbidq.html?CG10383),[CD98hc](http://flybase.bio.indiana.edu/.bin/fbidq.html?CD98hc), [CG8790](http://flybase.bio.indiana.edu/.bin/fbidq.html?CG8790), [Picot](http://flybase.bio.indiana.edu/.bin/fbidq.html?Picot), [Npc2b](http://flybase.bio.indiana.edu/.bin/fbidq.html?Npc2b), [CG16700](http://flybase.bio.indiana.edu/.bin/fbidq.html?CG16700), [Irk2](http://flybase.bio.indiana.edu/.bin/fbidq.html?Irk2), [ClC-c](http://flybase.bio.indiana.edu/.bin/fbidq.html?ClC-c), [Npc1a](http://flybase.bio.indiana.edu/.bin/fbidq.html?Npc1a), [CG15890](http://flybase.bio.indiana.edu/.bin/fbidq.html?CG15890), [NKAIN](http://flybase.bio.indiana.edu/.bin/fbidq.html?NKAIN), [Aph-4](http://flybase.bio.indiana.edu/.bin/fbidq.html?Aph-4), [trpm](http://flybase.bio.indiana.edu/.bin/fbidq.html?trpm), [CG12344](http://flybase.bio.indiana.edu/.bin/fbidq.html?CG12344), [CG6723](http://flybase.bio.indiana.edu/.bin/fbidq.html?CG6723), [Vha100-2](http://flybase.bio.indiana.edu/.bin/fbidq.html?Vha100-2), [CG9449](http://flybase.bio.indiana.edu/.bin/fbidq.html?CG9449), [CG7149](http://flybase.bio.indiana.edu/.bin/fbidq.html?CG7149), [gb](http://flybase.bio.indiana.edu/.bin/fbidq.html?gb), [CG7777](http://flybase.bio.indiana.edu/.bin/fbidq.html?CG7777), [mol](http://flybase.bio.indiana.edu/.bin/fbidq.html?mol), [spin](http://flybase.bio.indiana.edu/.bin/fbidq.html?spin), [Past1](http://flybase.bio.indiana.edu/.bin/fbidq.html?Past1), [Eaat1](http://flybase.bio.indiana.edu/.bin/fbidq.html?Eaat1), [SrpRbeta](http://flybase.bio.indiana.edu/.bin/fbidq.html?SrpRbeta), [CanB2](http://flybase.bio.indiana.edu/.bin/fbidq.html?CanB2), [loco](http://flybase.bio.indiana.edu/.bin/fbidq.html?loco), [Atpalpha](http://flybase.bio.indiana.edu/.bin/fbidq.html?Atpalpha), [Tig](http://flybase.bio.indiana.edu/.bin/fbidq.html?Tig), [CG5104](http://flybase.bio.indiana.edu/.bin/fbidq.html?CG5104),[CG5885](http://flybase.bio.indiana.edu/.bin/fbidq.html?CG5885), [CG31229](http://flybase.bio.indiana.edu/.bin/fbidq.html?CG31229), [bib](http://flybase.bio.indiana.edu/.bin/fbidq.html?bib), [Sur](http://flybase.bio.indiana.edu/.bin/fbidq.html?Sur), [CG5130](http://flybase.bio.indiana.edu/.bin/fbidq.html?CG5130), [CG9053](http://flybase.bio.indiana.edu/.bin/fbidq.html?CG9053), [ClC-a](http://flybase.bio.indiana.edu/.bin/fbidq.html?ClC-a), [cert](http://flybase.bio.indiana.edu/.bin/fbidq.html?cert), [Npc2a](http://flybase.bio.indiana.edu/.bin/fbidq.html?Npc2a), [Vinc](http://flybase.bio.indiana.edu/.bin/fbidq.html?Vinc), [Ent2](http://flybase.bio.indiana.edu/.bin/fbidq.html?Ent2), [kel](http://flybase.bio.indiana.edu/.bin/fbidq.html?kel), [Ctr1A](http://flybase.bio.indiana.edu/.bin/fbidq.html?Ctr1A), [CG3036](http://flybase.bio.indiana.edu/.bin/fbidq.html?CG3036), [CG17119](http://flybase.bio.indiana.edu/.bin/fbidq.html?CG17119), [HLH106](http://flybase.bio.indiana.edu/.bin/fbidq.html?HLH106), [drpr](http://flybase.bio.indiana.edu/.bin/fbidq.html?drpr), [CG31729](http://flybase.bio.indiana.edu/.bin/fbidq.html?CG31729), [pnt](http://flybase.bio.indiana.edu/.bin/fbidq.html?pnt), [VhaPPA1-1](http://flybase.bio.indiana.edu/.bin/fbidq.html?VhaPPA1-1), [Orct2](http://flybase.bio.indiana.edu/.bin/fbidq.html?Orct2), [ine](http://flybase.bio.indiana.edu/.bin/fbidq.html?ine), [CG6672](http://flybase.bio.indiana.edu/.bin/fbidq.html?CG6672), [w](http://flybase.bio.indiana.edu/.bin/fbidq.html?w), [CG3168](http://flybase.bio.indiana.edu/.bin/fbidq.html?CG3168), [CG10444](http://flybase.bio.indiana.edu/.bin/fbidq.html?CG10444), [Indy](http://flybase.bio.indiana.edu/.bin/fbidq.html?Indy), [btsz](http://flybase.bio.indiana.edu/.bin/fbidq.html?btsz), [Mdr65](http://flybase.bio.indiana.edu/.bin/fbidq.html?Mdr65), [CG13887](http://flybase.bio.indiana.edu/.bin/fbidq.html?CG13887), [Itp-r83A](http://flybase.bio.indiana.edu/.bin/fbidq.html?Itp-r83A),[debcl](http://flybase.bio.indiana.edu/.bin/fbidq.html?debcl), [Mer](http://flybase.bio.indiana.edu/.bin/fbidq.html?Mer), [Syx4](http://flybase.bio.indiana.edu/.bin/fbidq.html?Syx4), [Irk3](http://flybase.bio.indiana.edu/.bin/fbidq.html?Irk3), [CG17036](http://flybase.bio.indiana.edu/.bin/fbidq.html?CG17036), [Tsf2](http://flybase.bio.indiana.edu/.bin/fbidq.html?Tsf2), [aralar1](http://flybase.bio.indiana.edu/.bin/fbidq.html?aralar1), [CG15438](http://flybase.bio.indiana.edu/.bin/fbidq.html?CG15438), [VhaAC39-1](http://flybase.bio.indiana.edu/.bin/fbidq.html?VhaAC39-1), [CG1213](http://flybase.bio.indiana.edu/.bin/fbidq.html?CG1213), [Bet1](http://flybase.bio.indiana.edu/.bin/fbidq.html?Bet1), [Gtp-bp](http://flybase.bio.indiana.edu/.bin/fbidq.html?Gtp-bp), [CG10069](http://flybase.bio.indiana.edu/.bin/fbidq.html?CG10069), [CG1907](http://flybase.bio.indiana.edu/.bin/fbidq.html?CG1907), [Fatp](http://flybase.bio.indiana.edu/.bin/fbidq.html?Fatp), [CG33970](http://flybase.bio.indiana.edu/.bin/fbidq.html?CG33970), [CG2893](http://flybase.bio.indiana.edu/.bin/fbidq.html?CG2893), [glob1](http://flybase.bio.indiana.edu/.bin/fbidq.html?glob1), [pain](http://flybase.bio.indiana.edu/.bin/fbidq.html?pain), [zetaCOP](http://flybase.bio.indiana.edu/.bin/fbidq.html?zetaCOP), [CG15094](http://flybase.bio.indiana.edu/.bin/fbidq.html?CG15094), [pyx](http://flybase.bio.indiana.edu/.bin/fbidq.html?pyx), [edl](http://flybase.bio.indiana.edu/.bin/fbidq.html?edl), [CG8596](http://flybase.bio.indiana.edu/.bin/fbidq.html?CG8596), [ced-6](http://flybase.bio.indiana.edu/.bin/fbidq.html?ced-6), [CHOp24](http://flybase.bio.indiana.edu/.bin/fbidq.html?CHOp24), [CG33635](http://flybase.bio.indiana.edu/.bin/fbidq.html?CG33635), [CG6356](http://flybase.bio.indiana.edu/.bin/fbidq.html?CG6356),[CG11110](http://flybase.bio.indiana.edu/.bin/fbidq.html?CG11110), [Vha68-2](http://flybase.bio.indiana.edu/.bin/fbidq.html?Vha68-2), [CG11163](http://flybase.bio.indiana.edu/.bin/fbidq.html?CG11163), [VhaM9.7-b](http://flybase.bio.indiana.edu/.bin/fbidq.html?VhaM9.7-b), [CG9664](http://flybase.bio.indiana.edu/.bin/fbidq.html?CG9664), [jagn](http://flybase.bio.indiana.edu/.bin/fbidq.html?jagn), [eca](http://flybase.bio.indiana.edu/.bin/fbidq.html?eca), [CG4288](http://flybase.bio.indiana.edu/.bin/fbidq.html?CG4288), [nrv2](http://flybase.bio.indiana.edu/.bin/fbidq.html?nrv2), [TepII](http://flybase.bio.indiana.edu/.bin/fbidq.html?TepII), [VhaM8.9](http://flybase.bio.indiana.edu/.bin/fbidq.html?VhaM8.9), [CG4484](http://flybase.bio.indiana.edu/.bin/fbidq.html?CG4484), [CG7816](http://flybase.bio.indiana.edu/.bin/fbidq.html?CG7816), [CG4301](http://flybase.bio.indiana.edu/.bin/fbidq.html?CG4301), [Oatp33Ea](http://flybase.bio.indiana.edu/.bin/fbidq.html?Oatp33Ea), [CDase](http://flybase.bio.indiana.edu/.bin/fbidq.html?CDase), [ScpX](http://flybase.bio.indiana.edu/.bin/fbidq.html?ScpX), [Gpdh](http://flybase.bio.indiana.edu/.bin/fbidq.html?Gpdh), [CG6125](http://flybase.bio.indiana.edu/.bin/fbidq.html?CG6125), [Tango5](http://flybase.bio.indiana.edu/.bin/fbidq.html?Tango5), [CG5802](http://flybase.bio.indiana.edu/.bin/fbidq.html?CG5802), [wtrw](http://flybase.bio.indiana.edu/.bin/fbidq.html?wtrw), [CG2774](http://flybase.bio.indiana.edu/.bin/fbidq.html?CG2774), [TRAM](http://flybase.bio.indiana.edu/.bin/fbidq.html?TRAM), [CG6293](http://flybase.bio.indiana.edu/.bin/fbidq.html?CG6293), [p24-1](http://flybase.bio.indiana.edu/.bin/fbidq.html?p24-1), [BM-40-SPARC](http://flybase.bio.indiana.edu/.bin/fbidq.html?BM-40-SPARC),[CG1628](http://flybase.bio.indiana.edu/.bin/fbidq.html?CG1628), [Ork1](http://flybase.bio.indiana.edu/.bin/fbidq.html?Ork1), [CG10420](http://flybase.bio.indiana.edu/.bin/fbidq.html?CG10420), [SH3PX1](http://flybase.bio.indiana.edu/.bin/fbidq.html?SH3PX1), [yin](http://flybase.bio.indiana.edu/.bin/fbidq.html?yin), [KdelR](http://flybase.bio.indiana.edu/.bin/fbidq.html?KdelR), [shark](http://flybase.bio.indiana.edu/.bin/fbidq.html?shark) |
| [monocarboxylic acid catabolic process](http://amigo.geneontology.org/cgi-bin/amigo/go.cgi?view=details&query=GO:0072329) | 10 of 1000 genes, 1.0% | 14 of 7634 genes, 0.2% | 0.00113 | 0.00% | 0.00 | [CG17544](http://flybase.bio.indiana.edu/.bin/fbidq.html?CG17544), [Thiolase](http://flybase.bio.indiana.edu/.bin/fbidq.html?Thiolase), [CG3267](http://flybase.bio.indiana.edu/.bin/fbidq.html?CG3267), [yip2](http://flybase.bio.indiana.edu/.bin/fbidq.html?yip2), [Acox57D-p](http://flybase.bio.indiana.edu/.bin/fbidq.html?Acox57D-p), [CG12140](http://flybase.bio.indiana.edu/.bin/fbidq.html?CG12140), [CG6543](http://flybase.bio.indiana.edu/.bin/fbidq.html?CG6543), [Ssadh](http://flybase.bio.indiana.edu/.bin/fbidq.html?Ssadh), [CG4389](http://flybase.bio.indiana.edu/.bin/fbidq.html?CG4389), [CG12262](http://flybase.bio.indiana.edu/.bin/fbidq.html?CG12262) |
| [small molecule catabolic process](http://amigo.geneontology.org/cgi-bin/amigo/go.cgi?view=details&query=GO:0044282) | 17 of 1000 genes, 1.7% | 38 of 7634 genes, 0.5% | 0.00193 | 0.00% | 0.00 | [CG6638](http://flybase.bio.indiana.edu/.bin/fbidq.html?CG6638), [CG9362](http://flybase.bio.indiana.edu/.bin/fbidq.html?CG9362), [CG3267](http://flybase.bio.indiana.edu/.bin/fbidq.html?CG3267), [dare](http://flybase.bio.indiana.edu/.bin/fbidq.html?dare), [CG12140](http://flybase.bio.indiana.edu/.bin/fbidq.html?CG12140), [CG9547](http://flybase.bio.indiana.edu/.bin/fbidq.html?CG9547), [yip2](http://flybase.bio.indiana.edu/.bin/fbidq.html?yip2), [CG6543](http://flybase.bio.indiana.edu/.bin/fbidq.html?CG6543), [CG1140](http://flybase.bio.indiana.edu/.bin/fbidq.html?CG1140), [CG17544](http://flybase.bio.indiana.edu/.bin/fbidq.html?CG17544), [CG3376](http://flybase.bio.indiana.edu/.bin/fbidq.html?CG3376), [Thiolase](http://flybase.bio.indiana.edu/.bin/fbidq.html?Thiolase), [Acox57D-p](http://flybase.bio.indiana.edu/.bin/fbidq.html?Acox57D-p), [Ssadh](http://flybase.bio.indiana.edu/.bin/fbidq.html?Ssadh), [Ace](http://flybase.bio.indiana.edu/.bin/fbidq.html?Ace), [CG4389](http://flybase.bio.indiana.edu/.bin/fbidq.html?CG4389), [CG12262](http://flybase.bio.indiana.edu/.bin/fbidq.html?CG12262) |
| [single-organism catabolic process](http://amigo.geneontology.org/cgi-bin/amigo/go.cgi?view=details&query=GO:0044712) | 17 of 1000 genes, 1.7% | 38 of 7634 genes, 0.5% | 0.00193 | 0.00% | 0.00 | [CG6638](http://flybase.bio.indiana.edu/.bin/fbidq.html?CG6638), [CG9362](http://flybase.bio.indiana.edu/.bin/fbidq.html?CG9362), [CG3267](http://flybase.bio.indiana.edu/.bin/fbidq.html?CG3267), [dare](http://flybase.bio.indiana.edu/.bin/fbidq.html?dare), [CG12140](http://flybase.bio.indiana.edu/.bin/fbidq.html?CG12140), [CG9547](http://flybase.bio.indiana.edu/.bin/fbidq.html?CG9547), [yip2](http://flybase.bio.indiana.edu/.bin/fbidq.html?yip2), [CG6543](http://flybase.bio.indiana.edu/.bin/fbidq.html?CG6543), [CG1140](http://flybase.bio.indiana.edu/.bin/fbidq.html?CG1140), [CG17544](http://flybase.bio.indiana.edu/.bin/fbidq.html?CG17544), [CG3376](http://flybase.bio.indiana.edu/.bin/fbidq.html?CG3376), [Thiolase](http://flybase.bio.indiana.edu/.bin/fbidq.html?Thiolase), [Acox57D-p](http://flybase.bio.indiana.edu/.bin/fbidq.html?Acox57D-p), [Ssadh](http://flybase.bio.indiana.edu/.bin/fbidq.html?Ssadh), [Ace](http://flybase.bio.indiana.edu/.bin/fbidq.html?Ace), [CG4389](http://flybase.bio.indiana.edu/.bin/fbidq.html?CG4389), [CG12262](http://flybase.bio.indiana.edu/.bin/fbidq.html?CG12262) |
| [cell junction assembly](http://amigo.geneontology.org/cgi-bin/amigo/go.cgi?view=details&query=GO:0034329) | 18 of 1000 genes, 1.8% | 42 of 7634 genes, 0.6% | 0.00203 | 0.00% | 0.00 | [crok](http://flybase.bio.indiana.edu/.bin/fbidq.html?crok), [Cont](http://flybase.bio.indiana.edu/.bin/fbidq.html?Cont), [sinu](http://flybase.bio.indiana.edu/.bin/fbidq.html?sinu), [btsz](http://flybase.bio.indiana.edu/.bin/fbidq.html?btsz), [cold](http://flybase.bio.indiana.edu/.bin/fbidq.html?cold), [Tsf2](http://flybase.bio.indiana.edu/.bin/fbidq.html?Tsf2), [pck](http://flybase.bio.indiana.edu/.bin/fbidq.html?pck), [Nrg](http://flybase.bio.indiana.edu/.bin/fbidq.html?Nrg), [moody](http://flybase.bio.indiana.edu/.bin/fbidq.html?moody), [kune](http://flybase.bio.indiana.edu/.bin/fbidq.html?kune), [G-ialpha65A](http://flybase.bio.indiana.edu/.bin/fbidq.html?G-ialpha65A), [vari](http://flybase.bio.indiana.edu/.bin/fbidq.html?vari), [crim](http://flybase.bio.indiana.edu/.bin/fbidq.html?crim), [nrv2](http://flybase.bio.indiana.edu/.bin/fbidq.html?nrv2), [wun](http://flybase.bio.indiana.edu/.bin/fbidq.html?wun), [Atpalpha](http://flybase.bio.indiana.edu/.bin/fbidq.html?Atpalpha), [loco](http://flybase.bio.indiana.edu/.bin/fbidq.html?loco), [pyd](http://flybase.bio.indiana.edu/.bin/fbidq.html?pyd) |
| [fatty acid catabolic process](http://amigo.geneontology.org/cgi-bin/amigo/go.cgi?view=details&query=GO:0009062) | 9 of 1000 genes, 0.9% | 13 of 7634 genes, 0.2% | 0.00645 | 0.06% | 0.02 | [CG17544](http://flybase.bio.indiana.edu/.bin/fbidq.html?CG17544), [CG12140](http://flybase.bio.indiana.edu/.bin/fbidq.html?CG12140), [Acox57D-p](http://flybase.bio.indiana.edu/.bin/fbidq.html?Acox57D-p), [yip2](http://flybase.bio.indiana.edu/.bin/fbidq.html?yip2), [CG6543](http://flybase.bio.indiana.edu/.bin/fbidq.html?CG6543), [CG4389](http://flybase.bio.indiana.edu/.bin/fbidq.html?CG4389), [Thiolase](http://flybase.bio.indiana.edu/.bin/fbidq.html?Thiolase), [CG12262](http://flybase.bio.indiana.edu/.bin/fbidq.html?CG12262), [CG3267](http://flybase.bio.indiana.edu/.bin/fbidq.html?CG3267) |
| [establishment of glial blood-brain barrier](http://amigo.geneontology.org/cgi-bin/amigo/go.cgi?view=details&query=GO:0060857) | 9 of 1000 genes, 0.9% | 13 of 7634 genes, 0.2% | 0.00645 | 0.06% | 0.02 | [Cont](http://flybase.bio.indiana.edu/.bin/fbidq.html?Cont), [sinu](http://flybase.bio.indiana.edu/.bin/fbidq.html?sinu), [Nrg](http://flybase.bio.indiana.edu/.bin/fbidq.html?Nrg), [kune](http://flybase.bio.indiana.edu/.bin/fbidq.html?kune), [moody](http://flybase.bio.indiana.edu/.bin/fbidq.html?moody), [G-ialpha65A](http://flybase.bio.indiana.edu/.bin/fbidq.html?G-ialpha65A), [nrv2](http://flybase.bio.indiana.edu/.bin/fbidq.html?nrv2), [loco](http://flybase.bio.indiana.edu/.bin/fbidq.html?loco), [pck](http://flybase.bio.indiana.edu/.bin/fbidq.html?pck) |
| [establishment of localization](http://amigo.geneontology.org/cgi-bin/amigo/go.cgi?view=details&query=GO:0051234) | 186 of 1000 genes, 18.6% | 1088 of 7634 genes, 14.3% | 0.00839 | 0.06% | 0.02 | [CG16791](http://flybase.bio.indiana.edu/.bin/fbidq.html?CG16791), [CG10960](http://flybase.bio.indiana.edu/.bin/fbidq.html?CG10960), [CG14040](http://flybase.bio.indiana.edu/.bin/fbidq.html?CG14040), [Ppt1](http://flybase.bio.indiana.edu/.bin/fbidq.html?Ppt1), [Ir](http://flybase.bio.indiana.edu/.bin/fbidq.html?Ir), [AnnIX](http://flybase.bio.indiana.edu/.bin/fbidq.html?AnnIX), [JhI-21](http://flybase.bio.indiana.edu/.bin/fbidq.html?JhI-21), [hoe1](http://flybase.bio.indiana.edu/.bin/fbidq.html?hoe1), [scb](http://flybase.bio.indiana.edu/.bin/fbidq.html?scb), [pll](http://flybase.bio.indiana.edu/.bin/fbidq.html?pll), [CG7342](http://flybase.bio.indiana.edu/.bin/fbidq.html?CG7342), [Arf51F](http://flybase.bio.indiana.edu/.bin/fbidq.html?Arf51F), [Lsd-2](http://flybase.bio.indiana.edu/.bin/fbidq.html?Lsd-2), [membrin](http://flybase.bio.indiana.edu/.bin/fbidq.html?membrin), [Efr](http://flybase.bio.indiana.edu/.bin/fbidq.html?Efr), [CG31150](http://flybase.bio.indiana.edu/.bin/fbidq.html?CG31150), [Su(fu)](http://flybase.bio.indiana.edu/.bin/fbidq.html?Su(fu)), [CG5002](http://flybase.bio.indiana.edu/.bin/fbidq.html?CG5002), [Sop2](http://flybase.bio.indiana.edu/.bin/fbidq.html?Sop2), [Mcr](http://flybase.bio.indiana.edu/.bin/fbidq.html?Mcr), [mnd](http://flybase.bio.indiana.edu/.bin/fbidq.html?mnd), [CG10226](http://flybase.bio.indiana.edu/.bin/fbidq.html?CG10226), [CG6812](http://flybase.bio.indiana.edu/.bin/fbidq.html?CG6812), [CG1732](http://flybase.bio.indiana.edu/.bin/fbidq.html?CG1732), [svr](http://flybase.bio.indiana.edu/.bin/fbidq.html?svr), [CG10006](http://flybase.bio.indiana.edu/.bin/fbidq.html?CG10006), [CG11655](http://flybase.bio.indiana.edu/.bin/fbidq.html?CG11655), [CaBP1](http://flybase.bio.indiana.edu/.bin/fbidq.html?CaBP1), [loj](http://flybase.bio.indiana.edu/.bin/fbidq.html?loj), [Rac2](http://flybase.bio.indiana.edu/.bin/fbidq.html?Rac2), [Ced-12](http://flybase.bio.indiana.edu/.bin/fbidq.html?Ced-12),[CG1607](http://flybase.bio.indiana.edu/.bin/fbidq.html?CG1607), [CG5599](http://flybase.bio.indiana.edu/.bin/fbidq.html?CG5599), [Dscam](http://flybase.bio.indiana.edu/.bin/fbidq.html?Dscam), [bai](http://flybase.bio.indiana.edu/.bin/fbidq.html?bai), [mth](http://flybase.bio.indiana.edu/.bin/fbidq.html?mth), [gbb](http://flybase.bio.indiana.edu/.bin/fbidq.html?gbb), [Srp54k](http://flybase.bio.indiana.edu/.bin/fbidq.html?Srp54k), [Ppat-Dpck](http://flybase.bio.indiana.edu/.bin/fbidq.html?Ppat-Dpck), [kar](http://flybase.bio.indiana.edu/.bin/fbidq.html?kar), [ttv](http://flybase.bio.indiana.edu/.bin/fbidq.html?ttv), [Amph](http://flybase.bio.indiana.edu/.bin/fbidq.html?Amph), [CG42235](http://flybase.bio.indiana.edu/.bin/fbidq.html?CG42235), [Mtp](http://flybase.bio.indiana.edu/.bin/fbidq.html?Mtp), [Scamp](http://flybase.bio.indiana.edu/.bin/fbidq.html?Scamp), [Oatp58Dc](http://flybase.bio.indiana.edu/.bin/fbidq.html?Oatp58Dc), [VhaM9.7-c](http://flybase.bio.indiana.edu/.bin/fbidq.html?VhaM9.7-c), [Sec61alpha](http://flybase.bio.indiana.edu/.bin/fbidq.html?Sec61alpha), [nrv1](http://flybase.bio.indiana.edu/.bin/fbidq.html?nrv1), [CG12918](http://flybase.bio.indiana.edu/.bin/fbidq.html?CG12918), [colt](http://flybase.bio.indiana.edu/.bin/fbidq.html?colt), [Rfabg](http://flybase.bio.indiana.edu/.bin/fbidq.html?Rfabg), [CG3091](http://flybase.bio.indiana.edu/.bin/fbidq.html?CG3091), [NFAT](http://flybase.bio.indiana.edu/.bin/fbidq.html?NFAT), [Atox1](http://flybase.bio.indiana.edu/.bin/fbidq.html?Atox1), [Orct](http://flybase.bio.indiana.edu/.bin/fbidq.html?Orct), [CG8602](http://flybase.bio.indiana.edu/.bin/fbidq.html?CG8602), [G-ialpha65A](http://flybase.bio.indiana.edu/.bin/fbidq.html?G-ialpha65A), [CG14511](http://flybase.bio.indiana.edu/.bin/fbidq.html?CG14511), [Syb](http://flybase.bio.indiana.edu/.bin/fbidq.html?Syb),[CG9523](http://flybase.bio.indiana.edu/.bin/fbidq.html?CG9523), [CG10383](http://flybase.bio.indiana.edu/.bin/fbidq.html?CG10383), [CD98hc](http://flybase.bio.indiana.edu/.bin/fbidq.html?CD98hc), [CG8790](http://flybase.bio.indiana.edu/.bin/fbidq.html?CG8790), [Picot](http://flybase.bio.indiana.edu/.bin/fbidq.html?Picot), [Npc2b](http://flybase.bio.indiana.edu/.bin/fbidq.html?Npc2b), [CG16700](http://flybase.bio.indiana.edu/.bin/fbidq.html?CG16700), [Irk2](http://flybase.bio.indiana.edu/.bin/fbidq.html?Irk2), [ClC-c](http://flybase.bio.indiana.edu/.bin/fbidq.html?ClC-c), [Npc1a](http://flybase.bio.indiana.edu/.bin/fbidq.html?Npc1a), [CG15890](http://flybase.bio.indiana.edu/.bin/fbidq.html?CG15890), [NKAIN](http://flybase.bio.indiana.edu/.bin/fbidq.html?NKAIN), [Aph-4](http://flybase.bio.indiana.edu/.bin/fbidq.html?Aph-4), [trpm](http://flybase.bio.indiana.edu/.bin/fbidq.html?trpm), [CG12344](http://flybase.bio.indiana.edu/.bin/fbidq.html?CG12344), [CG6723](http://flybase.bio.indiana.edu/.bin/fbidq.html?CG6723), [Vha100-2](http://flybase.bio.indiana.edu/.bin/fbidq.html?Vha100-2), [CG9449](http://flybase.bio.indiana.edu/.bin/fbidq.html?CG9449), [CG7149](http://flybase.bio.indiana.edu/.bin/fbidq.html?CG7149), [gb](http://flybase.bio.indiana.edu/.bin/fbidq.html?gb), [CG7777](http://flybase.bio.indiana.edu/.bin/fbidq.html?CG7777), [mol](http://flybase.bio.indiana.edu/.bin/fbidq.html?mol), [spin](http://flybase.bio.indiana.edu/.bin/fbidq.html?spin), [Past1](http://flybase.bio.indiana.edu/.bin/fbidq.html?Past1), [Eaat1](http://flybase.bio.indiana.edu/.bin/fbidq.html?Eaat1), [CG3074](http://flybase.bio.indiana.edu/.bin/fbidq.html?CG3074), [SrpRbeta](http://flybase.bio.indiana.edu/.bin/fbidq.html?SrpRbeta), [CanB2](http://flybase.bio.indiana.edu/.bin/fbidq.html?CanB2), [loco](http://flybase.bio.indiana.edu/.bin/fbidq.html?loco),[Atpalpha](http://flybase.bio.indiana.edu/.bin/fbidq.html?Atpalpha), [Tig](http://flybase.bio.indiana.edu/.bin/fbidq.html?Tig), [CG5104](http://flybase.bio.indiana.edu/.bin/fbidq.html?CG5104), [CG5885](http://flybase.bio.indiana.edu/.bin/fbidq.html?CG5885), [CG31229](http://flybase.bio.indiana.edu/.bin/fbidq.html?CG31229), [bib](http://flybase.bio.indiana.edu/.bin/fbidq.html?bib), [Sur](http://flybase.bio.indiana.edu/.bin/fbidq.html?Sur), [CG5130](http://flybase.bio.indiana.edu/.bin/fbidq.html?CG5130), [CG9053](http://flybase.bio.indiana.edu/.bin/fbidq.html?CG9053), [ClC-a](http://flybase.bio.indiana.edu/.bin/fbidq.html?ClC-a), [cert](http://flybase.bio.indiana.edu/.bin/fbidq.html?cert), [Npc2a](http://flybase.bio.indiana.edu/.bin/fbidq.html?Npc2a), [Vinc](http://flybase.bio.indiana.edu/.bin/fbidq.html?Vinc), [Ent2](http://flybase.bio.indiana.edu/.bin/fbidq.html?Ent2), [kel](http://flybase.bio.indiana.edu/.bin/fbidq.html?kel), [Ctr1A](http://flybase.bio.indiana.edu/.bin/fbidq.html?Ctr1A), [CG3036](http://flybase.bio.indiana.edu/.bin/fbidq.html?CG3036), [CG17119](http://flybase.bio.indiana.edu/.bin/fbidq.html?CG17119), [HLH106](http://flybase.bio.indiana.edu/.bin/fbidq.html?HLH106), [drpr](http://flybase.bio.indiana.edu/.bin/fbidq.html?drpr), [CG31729](http://flybase.bio.indiana.edu/.bin/fbidq.html?CG31729), [pnt](http://flybase.bio.indiana.edu/.bin/fbidq.html?pnt), [VhaPPA1-1](http://flybase.bio.indiana.edu/.bin/fbidq.html?VhaPPA1-1), [Orct2](http://flybase.bio.indiana.edu/.bin/fbidq.html?Orct2), [ine](http://flybase.bio.indiana.edu/.bin/fbidq.html?ine), [CG6672](http://flybase.bio.indiana.edu/.bin/fbidq.html?CG6672), [w](http://flybase.bio.indiana.edu/.bin/fbidq.html?w), [CG3168](http://flybase.bio.indiana.edu/.bin/fbidq.html?CG3168), [CG10444](http://flybase.bio.indiana.edu/.bin/fbidq.html?CG10444), [Indy](http://flybase.bio.indiana.edu/.bin/fbidq.html?Indy), [btsz](http://flybase.bio.indiana.edu/.bin/fbidq.html?btsz), [Mdr65](http://flybase.bio.indiana.edu/.bin/fbidq.html?Mdr65),[CG13887](http://flybase.bio.indiana.edu/.bin/fbidq.html?CG13887), [Itp-r83A](http://flybase.bio.indiana.edu/.bin/fbidq.html?Itp-r83A), [debcl](http://flybase.bio.indiana.edu/.bin/fbidq.html?debcl), [Mer](http://flybase.bio.indiana.edu/.bin/fbidq.html?Mer), [Syx4](http://flybase.bio.indiana.edu/.bin/fbidq.html?Syx4), [Irk3](http://flybase.bio.indiana.edu/.bin/fbidq.html?Irk3), [CG17036](http://flybase.bio.indiana.edu/.bin/fbidq.html?CG17036), [Tsf2](http://flybase.bio.indiana.edu/.bin/fbidq.html?Tsf2), [aralar1](http://flybase.bio.indiana.edu/.bin/fbidq.html?aralar1), [CG15438](http://flybase.bio.indiana.edu/.bin/fbidq.html?CG15438), [VhaAC39-1](http://flybase.bio.indiana.edu/.bin/fbidq.html?VhaAC39-1), [CG1213](http://flybase.bio.indiana.edu/.bin/fbidq.html?CG1213), [Bet1](http://flybase.bio.indiana.edu/.bin/fbidq.html?Bet1), [Gtp-bp](http://flybase.bio.indiana.edu/.bin/fbidq.html?Gtp-bp), [CG10069](http://flybase.bio.indiana.edu/.bin/fbidq.html?CG10069), [CG1907](http://flybase.bio.indiana.edu/.bin/fbidq.html?CG1907), [Fatp](http://flybase.bio.indiana.edu/.bin/fbidq.html?Fatp), [CG33970](http://flybase.bio.indiana.edu/.bin/fbidq.html?CG33970), [CG2893](http://flybase.bio.indiana.edu/.bin/fbidq.html?CG2893), [glob1](http://flybase.bio.indiana.edu/.bin/fbidq.html?glob1), [pain](http://flybase.bio.indiana.edu/.bin/fbidq.html?pain), [zetaCOP](http://flybase.bio.indiana.edu/.bin/fbidq.html?zetaCOP), [CG15094](http://flybase.bio.indiana.edu/.bin/fbidq.html?CG15094), [pyx](http://flybase.bio.indiana.edu/.bin/fbidq.html?pyx), [edl](http://flybase.bio.indiana.edu/.bin/fbidq.html?edl), [CG8596](http://flybase.bio.indiana.edu/.bin/fbidq.html?CG8596), [ced-6](http://flybase.bio.indiana.edu/.bin/fbidq.html?ced-6), [CHOp24](http://flybase.bio.indiana.edu/.bin/fbidq.html?CHOp24),[CG33635](http://flybase.bio.indiana.edu/.bin/fbidq.html?CG33635), [CG6356](http://flybase.bio.indiana.edu/.bin/fbidq.html?CG6356), [CG11110](http://flybase.bio.indiana.edu/.bin/fbidq.html?CG11110), [Vha68-2](http://flybase.bio.indiana.edu/.bin/fbidq.html?Vha68-2), [CG11163](http://flybase.bio.indiana.edu/.bin/fbidq.html?CG11163), [VhaM9.7-b](http://flybase.bio.indiana.edu/.bin/fbidq.html?VhaM9.7-b), [CG9664](http://flybase.bio.indiana.edu/.bin/fbidq.html?CG9664), [jagn](http://flybase.bio.indiana.edu/.bin/fbidq.html?jagn), [eca](http://flybase.bio.indiana.edu/.bin/fbidq.html?eca), [CG4288](http://flybase.bio.indiana.edu/.bin/fbidq.html?CG4288), [nrv2](http://flybase.bio.indiana.edu/.bin/fbidq.html?nrv2), [TepII](http://flybase.bio.indiana.edu/.bin/fbidq.html?TepII), [VhaM8.9](http://flybase.bio.indiana.edu/.bin/fbidq.html?VhaM8.9), [CG4484](http://flybase.bio.indiana.edu/.bin/fbidq.html?CG4484), [CG7816](http://flybase.bio.indiana.edu/.bin/fbidq.html?CG7816), [CG4301](http://flybase.bio.indiana.edu/.bin/fbidq.html?CG4301), [Oatp33Ea](http://flybase.bio.indiana.edu/.bin/fbidq.html?Oatp33Ea), [CDase](http://flybase.bio.indiana.edu/.bin/fbidq.html?CDase), [grk](http://flybase.bio.indiana.edu/.bin/fbidq.html?grk), [ScpX](http://flybase.bio.indiana.edu/.bin/fbidq.html?ScpX), [Gpdh](http://flybase.bio.indiana.edu/.bin/fbidq.html?Gpdh), [CG6125](http://flybase.bio.indiana.edu/.bin/fbidq.html?CG6125), [Tango5](http://flybase.bio.indiana.edu/.bin/fbidq.html?Tango5), [CG5802](http://flybase.bio.indiana.edu/.bin/fbidq.html?CG5802), [wtrw](http://flybase.bio.indiana.edu/.bin/fbidq.html?wtrw), [CG2774](http://flybase.bio.indiana.edu/.bin/fbidq.html?CG2774), [TRAM](http://flybase.bio.indiana.edu/.bin/fbidq.html?TRAM),[CG6293](http://flybase.bio.indiana.edu/.bin/fbidq.html?CG6293), [p24-1](http://flybase.bio.indiana.edu/.bin/fbidq.html?p24-1), [BM-40-SPARC](http://flybase.bio.indiana.edu/.bin/fbidq.html?BM-40-SPARC), [CG1628](http://flybase.bio.indiana.edu/.bin/fbidq.html?CG1628), [Ork1](http://flybase.bio.indiana.edu/.bin/fbidq.html?Ork1), [CG10420](http://flybase.bio.indiana.edu/.bin/fbidq.html?CG10420), [SH3PX1](http://flybase.bio.indiana.edu/.bin/fbidq.html?SH3PX1), [yin](http://flybase.bio.indiana.edu/.bin/fbidq.html?yin), [shark](http://flybase.bio.indiana.edu/.bin/fbidq.html?shark), [KdelR](http://flybase.bio.indiana.edu/.bin/fbidq.html?KdelR) |
