# Supplementary material for: Glial enriched gene expression profiling identifies novel factors regulating the proliferation of specific glial subtypes in the Drosophila brain
Source: Gene Expr Patterns. 2014 Sep;16(1):61–8. doi: 10.1016/j.gep.2014.09.001 (PMC4222725; doi:10.1016/j.gep.2014.09.001)
Supplement: Table S9 — GO analysis (cellular processes) of genes with significantly increased expression ≥1.5 fold in repo-Gal4, UAS-InR CNS tissue. p-value ≤0.01. [file mmc9.docx]

*Supplementary table S9. GO analysis (cellular processes) of genes with significantly increased expression ≥1.5 fold in repo-Gal4, UAS-InR CNS tissue. p-value ≤0.01.*

| **Gene Ontology term** | **Cluster frequency** | **Genome frequency** | **Corrected P-value** | **FDR** | **False Positives** | **Genes annotated to the term** |
| --- | --- | --- | --- | --- | --- | --- |
| [apical junction assembly](http://amigo.geneontology.org/cgi-bin/amigo/go.cgi?view=details&query=GO:0043297) | 17 of 616 genes, 2.8% | 36 of 7732 genes, 0.5% | 2.82e-07 | 0.00% | 0.00 | [crok](http://flybase.bio.indiana.edu/.bin/fbidq.html?crok), [Cont](http://flybase.bio.indiana.edu/.bin/fbidq.html?Cont), [sinu](http://flybase.bio.indiana.edu/.bin/fbidq.html?sinu), [kst](http://flybase.bio.indiana.edu/.bin/fbidq.html?kst), [Nrg](http://flybase.bio.indiana.edu/.bin/fbidq.html?Nrg), [btsz](http://flybase.bio.indiana.edu/.bin/fbidq.html?btsz), [kune](http://flybase.bio.indiana.edu/.bin/fbidq.html?kune), [moody](http://flybase.bio.indiana.edu/.bin/fbidq.html?moody), [vari](http://flybase.bio.indiana.edu/.bin/fbidq.html?vari), [crim](http://flybase.bio.indiana.edu/.bin/fbidq.html?crim), [cold](http://flybase.bio.indiana.edu/.bin/fbidq.html?cold), [wun](http://flybase.bio.indiana.edu/.bin/fbidq.html?wun), [wol](http://flybase.bio.indiana.edu/.bin/fbidq.html?wol), [cora](http://flybase.bio.indiana.edu/.bin/fbidq.html?cora), [Tsf2](http://flybase.bio.indiana.edu/.bin/fbidq.html?Tsf2), [pck](http://flybase.bio.indiana.edu/.bin/fbidq.html?pck), [scrib](http://flybase.bio.indiana.edu/.bin/fbidq.html?scrib) |
| [septate junction assembly](http://amigo.geneontology.org/cgi-bin/amigo/go.cgi?view=details&query=GO:0019991) | 15 of 616 genes, 2.4% | 28 of 7732 genes, 0.4% | 3.38e-07 | 0.00% | 0.00 | [crok](http://flybase.bio.indiana.edu/.bin/fbidq.html?crok), [Cont](http://flybase.bio.indiana.edu/.bin/fbidq.html?Cont), [sinu](http://flybase.bio.indiana.edu/.bin/fbidq.html?sinu), [Nrg](http://flybase.bio.indiana.edu/.bin/fbidq.html?Nrg), [kune](http://flybase.bio.indiana.edu/.bin/fbidq.html?kune), [moody](http://flybase.bio.indiana.edu/.bin/fbidq.html?moody), [vari](http://flybase.bio.indiana.edu/.bin/fbidq.html?vari), [crim](http://flybase.bio.indiana.edu/.bin/fbidq.html?crim), [cold](http://flybase.bio.indiana.edu/.bin/fbidq.html?cold), [wun](http://flybase.bio.indiana.edu/.bin/fbidq.html?wun), [wol](http://flybase.bio.indiana.edu/.bin/fbidq.html?wol), [cora](http://flybase.bio.indiana.edu/.bin/fbidq.html?cora), [Tsf2](http://flybase.bio.indiana.edu/.bin/fbidq.html?Tsf2), [pck](http://flybase.bio.indiana.edu/.bin/fbidq.html?pck), [scrib](http://flybase.bio.indiana.edu/.bin/fbidq.html?scrib) |
| [cell-cell junction assembly](http://amigo.geneontology.org/cgi-bin/amigo/go.cgi?view=details&query=GO:0007043) | 17 of 616 genes, 2.8% | 40 of 7732 genes, 0.5% | 2.17e-06 | 0.00% | 0.00 | [crok](http://flybase.bio.indiana.edu/.bin/fbidq.html?crok), [Cont](http://flybase.bio.indiana.edu/.bin/fbidq.html?Cont), [sinu](http://flybase.bio.indiana.edu/.bin/fbidq.html?sinu), [kst](http://flybase.bio.indiana.edu/.bin/fbidq.html?kst), [Nrg](http://flybase.bio.indiana.edu/.bin/fbidq.html?Nrg), [btsz](http://flybase.bio.indiana.edu/.bin/fbidq.html?btsz), [kune](http://flybase.bio.indiana.edu/.bin/fbidq.html?kune), [moody](http://flybase.bio.indiana.edu/.bin/fbidq.html?moody), [vari](http://flybase.bio.indiana.edu/.bin/fbidq.html?vari), [crim](http://flybase.bio.indiana.edu/.bin/fbidq.html?crim), [cold](http://flybase.bio.indiana.edu/.bin/fbidq.html?cold), [wun](http://flybase.bio.indiana.edu/.bin/fbidq.html?wun), [wol](http://flybase.bio.indiana.edu/.bin/fbidq.html?wol), [cora](http://flybase.bio.indiana.edu/.bin/fbidq.html?cora), [Tsf2](http://flybase.bio.indiana.edu/.bin/fbidq.html?Tsf2), [pck](http://flybase.bio.indiana.edu/.bin/fbidq.html?pck), [scrib](http://flybase.bio.indiana.edu/.bin/fbidq.html?scrib) |
| [cell junction assembly](http://amigo.geneontology.org/cgi-bin/amigo/go.cgi?view=details&query=GO:0034329) | 17 of 616 genes, 2.8% | 42 of 7732 genes, 0.5% | 5.38e-06 | 0.00% | 0.00 | [crok](http://flybase.bio.indiana.edu/.bin/fbidq.html?crok), [Cont](http://flybase.bio.indiana.edu/.bin/fbidq.html?Cont), [sinu](http://flybase.bio.indiana.edu/.bin/fbidq.html?sinu), [kst](http://flybase.bio.indiana.edu/.bin/fbidq.html?kst), [Nrg](http://flybase.bio.indiana.edu/.bin/fbidq.html?Nrg), [btsz](http://flybase.bio.indiana.edu/.bin/fbidq.html?btsz), [kune](http://flybase.bio.indiana.edu/.bin/fbidq.html?kune), [moody](http://flybase.bio.indiana.edu/.bin/fbidq.html?moody), [vari](http://flybase.bio.indiana.edu/.bin/fbidq.html?vari), [crim](http://flybase.bio.indiana.edu/.bin/fbidq.html?crim), [cold](http://flybase.bio.indiana.edu/.bin/fbidq.html?cold), [wun](http://flybase.bio.indiana.edu/.bin/fbidq.html?wun), [wol](http://flybase.bio.indiana.edu/.bin/fbidq.html?wol), [cora](http://flybase.bio.indiana.edu/.bin/fbidq.html?cora), [Tsf2](http://flybase.bio.indiana.edu/.bin/fbidq.html?Tsf2), [pck](http://flybase.bio.indiana.edu/.bin/fbidq.html?pck), [scrib](http://flybase.bio.indiana.edu/.bin/fbidq.html?scrib) |
| [defense response](http://amigo.geneontology.org/cgi-bin/amigo/go.cgi?view=details&query=GO:0006952) | 31 of 616 genes, 5.0% | 139 of 7732 genes, 1.8% | 6.30e-05 | 0.00% | 0.00 | [LanA](http://flybase.bio.indiana.edu/.bin/fbidq.html?LanA), [egr](http://flybase.bio.indiana.edu/.bin/fbidq.html?egr), [spz](http://flybase.bio.indiana.edu/.bin/fbidq.html?spz), [santa-maria](http://flybase.bio.indiana.edu/.bin/fbidq.html?santa-maria), [Sp7](http://flybase.bio.indiana.edu/.bin/fbidq.html?Sp7), [Mcr](http://flybase.bio.indiana.edu/.bin/fbidq.html?Mcr), [Mpk2](http://flybase.bio.indiana.edu/.bin/fbidq.html?Mpk2), [CG10433](http://flybase.bio.indiana.edu/.bin/fbidq.html?CG10433), [Tsf1](http://flybase.bio.indiana.edu/.bin/fbidq.html?Tsf1), [He](http://flybase.bio.indiana.edu/.bin/fbidq.html?He), [scrib](http://flybase.bio.indiana.edu/.bin/fbidq.html?scrib), [PGRP-LA](http://flybase.bio.indiana.edu/.bin/fbidq.html?PGRP-LA), [Duox](http://flybase.bio.indiana.edu/.bin/fbidq.html?Duox), [Cpr49Ac](http://flybase.bio.indiana.edu/.bin/fbidq.html?Cpr49Ac), [Nrg](http://flybase.bio.indiana.edu/.bin/fbidq.html?Nrg), [Rac2](http://flybase.bio.indiana.edu/.bin/fbidq.html?Rac2), [Spn27A](http://flybase.bio.indiana.edu/.bin/fbidq.html?Spn27A), [Myo61F](http://flybase.bio.indiana.edu/.bin/fbidq.html?Myo61F), [TepIV](http://flybase.bio.indiana.edu/.bin/fbidq.html?TepIV), [PGRP-LE](http://flybase.bio.indiana.edu/.bin/fbidq.html?PGRP-LE), [pes](http://flybase.bio.indiana.edu/.bin/fbidq.html?pes), [Myd88](http://flybase.bio.indiana.edu/.bin/fbidq.html?Myd88), [TepII](http://flybase.bio.indiana.edu/.bin/fbidq.html?TepII), [PGRP-SA](http://flybase.bio.indiana.edu/.bin/fbidq.html?PGRP-SA), [Spn28D](http://flybase.bio.indiana.edu/.bin/fbidq.html?Spn28D), [nimB4](http://flybase.bio.indiana.edu/.bin/fbidq.html?nimB4), [PGRP-LB](http://flybase.bio.indiana.edu/.bin/fbidq.html?PGRP-LB),[ird5](http://flybase.bio.indiana.edu/.bin/fbidq.html?ird5), [Hml](http://flybase.bio.indiana.edu/.bin/fbidq.html?Hml), [vir-1](http://flybase.bio.indiana.edu/.bin/fbidq.html?vir-1), [modSP](http://flybase.bio.indiana.edu/.bin/fbidq.html?modSP) |
| [cell junction organization](http://amigo.geneontology.org/cgi-bin/amigo/go.cgi?view=details&query=GO:0034330) | 18 of 616 genes, 2.9% | 55 of 7732 genes, 0.7% | 9.44e-05 | 0.00% | 0.00 | [crok](http://flybase.bio.indiana.edu/.bin/fbidq.html?crok), [Cont](http://flybase.bio.indiana.edu/.bin/fbidq.html?Cont), [sinu](http://flybase.bio.indiana.edu/.bin/fbidq.html?sinu), [kst](http://flybase.bio.indiana.edu/.bin/fbidq.html?kst), [Nrg](http://flybase.bio.indiana.edu/.bin/fbidq.html?Nrg), [Rac2](http://flybase.bio.indiana.edu/.bin/fbidq.html?Rac2), [btsz](http://flybase.bio.indiana.edu/.bin/fbidq.html?btsz), [kune](http://flybase.bio.indiana.edu/.bin/fbidq.html?kune), [moody](http://flybase.bio.indiana.edu/.bin/fbidq.html?moody), [vari](http://flybase.bio.indiana.edu/.bin/fbidq.html?vari), [crim](http://flybase.bio.indiana.edu/.bin/fbidq.html?crim), [cold](http://flybase.bio.indiana.edu/.bin/fbidq.html?cold), [wun](http://flybase.bio.indiana.edu/.bin/fbidq.html?wun), [wol](http://flybase.bio.indiana.edu/.bin/fbidq.html?wol), [cora](http://flybase.bio.indiana.edu/.bin/fbidq.html?cora), [Tsf2](http://flybase.bio.indiana.edu/.bin/fbidq.html?Tsf2), [pck](http://flybase.bio.indiana.edu/.bin/fbidq.html?pck), [scrib](http://flybase.bio.indiana.edu/.bin/fbidq.html?scrib) |
| [cell-cell junction organization](http://amigo.geneontology.org/cgi-bin/amigo/go.cgi?view=details&query=GO:0045216) | 18 of 616 genes, 2.9% | 55 of 7732 genes, 0.7% | 9.44e-05 | 0.00% | 0.00 | [crok](http://flybase.bio.indiana.edu/.bin/fbidq.html?crok), [Cont](http://flybase.bio.indiana.edu/.bin/fbidq.html?Cont), [sinu](http://flybase.bio.indiana.edu/.bin/fbidq.html?sinu), [kst](http://flybase.bio.indiana.edu/.bin/fbidq.html?kst), [Nrg](http://flybase.bio.indiana.edu/.bin/fbidq.html?Nrg), [Rac2](http://flybase.bio.indiana.edu/.bin/fbidq.html?Rac2), [btsz](http://flybase.bio.indiana.edu/.bin/fbidq.html?btsz), [kune](http://flybase.bio.indiana.edu/.bin/fbidq.html?kune), [moody](http://flybase.bio.indiana.edu/.bin/fbidq.html?moody), [vari](http://flybase.bio.indiana.edu/.bin/fbidq.html?vari), [crim](http://flybase.bio.indiana.edu/.bin/fbidq.html?crim), [cold](http://flybase.bio.indiana.edu/.bin/fbidq.html?cold), [wun](http://flybase.bio.indiana.edu/.bin/fbidq.html?wun), [wol](http://flybase.bio.indiana.edu/.bin/fbidq.html?wol), [cora](http://flybase.bio.indiana.edu/.bin/fbidq.html?cora), [Tsf2](http://flybase.bio.indiana.edu/.bin/fbidq.html?Tsf2), [pck](http://flybase.bio.indiana.edu/.bin/fbidq.html?pck), [scrib](http://flybase.bio.indiana.edu/.bin/fbidq.html?scrib) |
| [regulation of tube size, open tracheal system](http://amigo.geneontology.org/cgi-bin/amigo/go.cgi?view=details&query=GO:0035151) | 15 of 616 genes, 2.4% | 43 of 7732 genes, 0.6% | 0.00045 | 0.00% | 0.00 | [dpp](http://flybase.bio.indiana.edu/.bin/fbidq.html?dpp), [crok](http://flybase.bio.indiana.edu/.bin/fbidq.html?crok), [sinu](http://flybase.bio.indiana.edu/.bin/fbidq.html?sinu), [Fas2](http://flybase.bio.indiana.edu/.bin/fbidq.html?Fas2), [kni](http://flybase.bio.indiana.edu/.bin/fbidq.html?kni), [Nrg](http://flybase.bio.indiana.edu/.bin/fbidq.html?Nrg), [kune](http://flybase.bio.indiana.edu/.bin/fbidq.html?kune), [vari](http://flybase.bio.indiana.edu/.bin/fbidq.html?vari), [knk](http://flybase.bio.indiana.edu/.bin/fbidq.html?knk), [crim](http://flybase.bio.indiana.edu/.bin/fbidq.html?crim), [cold](http://flybase.bio.indiana.edu/.bin/fbidq.html?cold), [cora](http://flybase.bio.indiana.edu/.bin/fbidq.html?cora), [ttk](http://flybase.bio.indiana.edu/.bin/fbidq.html?ttk), [Mmp1](http://flybase.bio.indiana.edu/.bin/fbidq.html?Mmp1), [pck](http://flybase.bio.indiana.edu/.bin/fbidq.html?pck) |
| [establishment of blood-brain barrier](http://amigo.geneontology.org/cgi-bin/amigo/go.cgi?view=details&query=GO:0060856) | 9 of 616 genes, 1.5% | 16 of 7732 genes, 0.2% | 0.00085 | 0.00% | 0.00 | [Cont](http://flybase.bio.indiana.edu/.bin/fbidq.html?Cont), [sinu](http://flybase.bio.indiana.edu/.bin/fbidq.html?sinu), [Nrg](http://flybase.bio.indiana.edu/.bin/fbidq.html?Nrg), [kune](http://flybase.bio.indiana.edu/.bin/fbidq.html?kune), [moody](http://flybase.bio.indiana.edu/.bin/fbidq.html?moody), [cold](http://flybase.bio.indiana.edu/.bin/fbidq.html?cold), [Mdr65](http://flybase.bio.indiana.edu/.bin/fbidq.html?Mdr65), [cora](http://flybase.bio.indiana.edu/.bin/fbidq.html?cora), [pck](http://flybase.bio.indiana.edu/.bin/fbidq.html?pck) |
| [regulation of tube size](http://amigo.geneontology.org/cgi-bin/amigo/go.cgi?view=details&query=GO:0035150) | 15 of 616 genes, 2.4% | 45 of 7732 genes, 0.6% | 0.00090 | 0.00% | 0.00 | [dpp](http://flybase.bio.indiana.edu/.bin/fbidq.html?dpp), [crok](http://flybase.bio.indiana.edu/.bin/fbidq.html?crok), [sinu](http://flybase.bio.indiana.edu/.bin/fbidq.html?sinu), [Fas2](http://flybase.bio.indiana.edu/.bin/fbidq.html?Fas2), [kni](http://flybase.bio.indiana.edu/.bin/fbidq.html?kni), [Nrg](http://flybase.bio.indiana.edu/.bin/fbidq.html?Nrg), [kune](http://flybase.bio.indiana.edu/.bin/fbidq.html?kune), [vari](http://flybase.bio.indiana.edu/.bin/fbidq.html?vari), [knk](http://flybase.bio.indiana.edu/.bin/fbidq.html?knk), [crim](http://flybase.bio.indiana.edu/.bin/fbidq.html?crim), [cold](http://flybase.bio.indiana.edu/.bin/fbidq.html?cold), [cora](http://flybase.bio.indiana.edu/.bin/fbidq.html?cora), [ttk](http://flybase.bio.indiana.edu/.bin/fbidq.html?ttk), [Mmp1](http://flybase.bio.indiana.edu/.bin/fbidq.html?Mmp1), [pck](http://flybase.bio.indiana.edu/.bin/fbidq.html?pck) |
| [immune response](http://amigo.geneontology.org/cgi-bin/amigo/go.cgi?view=details&query=GO:0006955) | 28 of 616 genes, 4.5% | 134 of 7732 genes, 1.7% | 0.00117 | 0.00% | 0.00 | [Mkp3](http://flybase.bio.indiana.edu/.bin/fbidq.html?Mkp3), [LanA](http://flybase.bio.indiana.edu/.bin/fbidq.html?LanA), [egr](http://flybase.bio.indiana.edu/.bin/fbidq.html?egr), [spz](http://flybase.bio.indiana.edu/.bin/fbidq.html?spz), [Sp7](http://flybase.bio.indiana.edu/.bin/fbidq.html?Sp7), [Mcr](http://flybase.bio.indiana.edu/.bin/fbidq.html?Mcr), [Mpk2](http://flybase.bio.indiana.edu/.bin/fbidq.html?Mpk2), [Pvr](http://flybase.bio.indiana.edu/.bin/fbidq.html?Pvr), [He](http://flybase.bio.indiana.edu/.bin/fbidq.html?He), [scrib](http://flybase.bio.indiana.edu/.bin/fbidq.html?scrib), [PGRP-LA](http://flybase.bio.indiana.edu/.bin/fbidq.html?PGRP-LA), [Duox](http://flybase.bio.indiana.edu/.bin/fbidq.html?Duox), [Cpr49Ac](http://flybase.bio.indiana.edu/.bin/fbidq.html?Cpr49Ac), [Nrg](http://flybase.bio.indiana.edu/.bin/fbidq.html?Nrg), [Rac2](http://flybase.bio.indiana.edu/.bin/fbidq.html?Rac2), [Spn27A](http://flybase.bio.indiana.edu/.bin/fbidq.html?Spn27A), [TepIV](http://flybase.bio.indiana.edu/.bin/fbidq.html?TepIV), [PGRP-LE](http://flybase.bio.indiana.edu/.bin/fbidq.html?PGRP-LE), [CG3074](http://flybase.bio.indiana.edu/.bin/fbidq.html?CG3074), [Myd88](http://flybase.bio.indiana.edu/.bin/fbidq.html?Myd88), [TepII](http://flybase.bio.indiana.edu/.bin/fbidq.html?TepII), [PGRP-SA](http://flybase.bio.indiana.edu/.bin/fbidq.html?PGRP-SA), [Spn28D](http://flybase.bio.indiana.edu/.bin/fbidq.html?Spn28D), [PGRP-LB](http://flybase.bio.indiana.edu/.bin/fbidq.html?PGRP-LB), [Hml](http://flybase.bio.indiana.edu/.bin/fbidq.html?Hml), [ird5](http://flybase.bio.indiana.edu/.bin/fbidq.html?ird5), [modSP](http://flybase.bio.indiana.edu/.bin/fbidq.html?modSP), [kay](http://flybase.bio.indiana.edu/.bin/fbidq.html?kay) |
| [response to bacterium](http://amigo.geneontology.org/cgi-bin/amigo/go.cgi?view=details&query=GO:0009617) | 18 of 616 genes, 2.9% | 66 of 7732 genes, 0.9% | 0.00200 | 0.33% | 0.04 | [egr](http://flybase.bio.indiana.edu/.bin/fbidq.html?egr), [spz](http://flybase.bio.indiana.edu/.bin/fbidq.html?spz), [Mcr](http://flybase.bio.indiana.edu/.bin/fbidq.html?Mcr), [Mpk2](http://flybase.bio.indiana.edu/.bin/fbidq.html?Mpk2), [Ance](http://flybase.bio.indiana.edu/.bin/fbidq.html?Ance), [scrib](http://flybase.bio.indiana.edu/.bin/fbidq.html?scrib), [PGRP-LA](http://flybase.bio.indiana.edu/.bin/fbidq.html?PGRP-LA), [Duox](http://flybase.bio.indiana.edu/.bin/fbidq.html?Duox), [Rac2](http://flybase.bio.indiana.edu/.bin/fbidq.html?Rac2), [TepIV](http://flybase.bio.indiana.edu/.bin/fbidq.html?TepIV), [Myo61F](http://flybase.bio.indiana.edu/.bin/fbidq.html?Myo61F), [PGRP-LE](http://flybase.bio.indiana.edu/.bin/fbidq.html?PGRP-LE), [Myd88](http://flybase.bio.indiana.edu/.bin/fbidq.html?Myd88), [TepII](http://flybase.bio.indiana.edu/.bin/fbidq.html?TepII), [PGRP-SA](http://flybase.bio.indiana.edu/.bin/fbidq.html?PGRP-SA), [nimB4](http://flybase.bio.indiana.edu/.bin/fbidq.html?nimB4), [PGRP-LB](http://flybase.bio.indiana.edu/.bin/fbidq.html?PGRP-LB), [ird5](http://flybase.bio.indiana.edu/.bin/fbidq.html?ird5) |
| [regulation of tube architecture, open tracheal system](http://amigo.geneontology.org/cgi-bin/amigo/go.cgi?view=details&query=GO:0035152) | 17 of 616 genes, 2.8% | 64 of 7732 genes, 0.8% | 0.00582 | 0.31% | 0.04 | [Mkp3](http://flybase.bio.indiana.edu/.bin/fbidq.html?Mkp3), [crok](http://flybase.bio.indiana.edu/.bin/fbidq.html?crok), [sinu](http://flybase.bio.indiana.edu/.bin/fbidq.html?sinu), [Fas2](http://flybase.bio.indiana.edu/.bin/fbidq.html?Fas2), [kni](http://flybase.bio.indiana.edu/.bin/fbidq.html?kni), [cold](http://flybase.bio.indiana.edu/.bin/fbidq.html?cold), [cora](http://flybase.bio.indiana.edu/.bin/fbidq.html?cora), [pck](http://flybase.bio.indiana.edu/.bin/fbidq.html?pck), [dpp](http://flybase.bio.indiana.edu/.bin/fbidq.html?dpp), [Nrg](http://flybase.bio.indiana.edu/.bin/fbidq.html?Nrg), [kune](http://flybase.bio.indiana.edu/.bin/fbidq.html?kune), [knk](http://flybase.bio.indiana.edu/.bin/fbidq.html?knk), [vari](http://flybase.bio.indiana.edu/.bin/fbidq.html?vari), [Syb](http://flybase.bio.indiana.edu/.bin/fbidq.html?Syb), [crim](http://flybase.bio.indiana.edu/.bin/fbidq.html?crim), [ttk](http://flybase.bio.indiana.edu/.bin/fbidq.html?ttk), [Mmp1](http://flybase.bio.indiana.edu/.bin/fbidq.html?Mmp1) |
| [defense response to bacterium](http://amigo.geneontology.org/cgi-bin/amigo/go.cgi?view=details&query=GO:0042742) | 16 of 616 genes, 2.6% | 59 of 7732 genes, 0.8% | 0.00830 | 0.29% | 0.04 | [egr](http://flybase.bio.indiana.edu/.bin/fbidq.html?egr), [spz](http://flybase.bio.indiana.edu/.bin/fbidq.html?spz), [Mcr](http://flybase.bio.indiana.edu/.bin/fbidq.html?Mcr), [Mpk2](http://flybase.bio.indiana.edu/.bin/fbidq.html?Mpk2), [scrib](http://flybase.bio.indiana.edu/.bin/fbidq.html?scrib), [Duox](http://flybase.bio.indiana.edu/.bin/fbidq.html?Duox), [Rac2](http://flybase.bio.indiana.edu/.bin/fbidq.html?Rac2), [TepIV](http://flybase.bio.indiana.edu/.bin/fbidq.html?TepIV), [Myo61F](http://flybase.bio.indiana.edu/.bin/fbidq.html?Myo61F), [PGRP-LE](http://flybase.bio.indiana.edu/.bin/fbidq.html?PGRP-LE), [Myd88](http://flybase.bio.indiana.edu/.bin/fbidq.html?Myd88), [TepII](http://flybase.bio.indiana.edu/.bin/fbidq.html?TepII), [PGRP-SA](http://flybase.bio.indiana.edu/.bin/fbidq.html?PGRP-SA), [nimB4](http://flybase.bio.indiana.edu/.bin/fbidq.html?nimB4), [ird5](http://flybase.bio.indiana.edu/.bin/fbidq.html?ird5), [PGRP-LB](http://flybase.bio.indiana.edu/.bin/fbidq.html?PGRP-LB) |
