# Supplementary material for: Glial enriched gene expression profiling identifies novel factors regulating the proliferation of specific glial subtypes in the Drosophila brain
Source: Gene Expr Patterns. 2014 Sep;16(1):61–8. doi: 10.1016/j.gep.2014.09.001 (PMC4222725; doi:10.1016/j.gep.2014.09.001)
Supplement: Table S10 — GO analysis (cellular processes) of genes with significantly decreased expression ≤1.5 fold in repo-Gal4, UAS-HtlACT CNS tissue. p-value ≤0.01. [file mmc10.docx]

*Supplementary table S10. GO analysis (cellular processes) of genes with significantly decreased expression ≤1.5 fold in repo-Gal4, UAS-Htl^ACT^ CNS tissue. p-value ≤0.01.*

| **Gene Ontology term** | **Cluster frequency** | **Genome frequency** | **Corrected P-value** | **FDR** | **False Positives** | **Genes annotated to the term** |
| --- | --- | --- | --- | --- | --- | --- |
| [regulation of cellular process](http://amigo.geneontology.org/cgi-bin/amigo/go.cgi?view=details&query=GO:0050794) | 545 of 1594 genes, 34.2% | 1961 of 7634 genes, 25.7% | 3.23e-20 | 0.00% | 0.00 | [Sh](http://flybase.bio.indiana.edu/.bin/fbidq.html?Sh), [dock](http://flybase.bio.indiana.edu/.bin/fbidq.html?dock), [tna](http://flybase.bio.indiana.edu/.bin/fbidq.html?tna), [CG8500](http://flybase.bio.indiana.edu/.bin/fbidq.html?CG8500), [mub](http://flybase.bio.indiana.edu/.bin/fbidq.html?mub), [rn](http://flybase.bio.indiana.edu/.bin/fbidq.html?rn), [ap](http://flybase.bio.indiana.edu/.bin/fbidq.html?ap), [cenG1A](http://flybase.bio.indiana.edu/.bin/fbidq.html?cenG1A), [tlk](http://flybase.bio.indiana.edu/.bin/fbidq.html?tlk), [malpha](http://flybase.bio.indiana.edu/.bin/fbidq.html?malpha), [os](http://flybase.bio.indiana.edu/.bin/fbidq.html?os), [Axn](http://flybase.bio.indiana.edu/.bin/fbidq.html?Axn), [mtrm](http://flybase.bio.indiana.edu/.bin/fbidq.html?mtrm), [Hcf](http://flybase.bio.indiana.edu/.bin/fbidq.html?Hcf), [Traf6](http://flybase.bio.indiana.edu/.bin/fbidq.html?Traf6), [Leucokinin](http://flybase.bio.indiana.edu/.bin/fbidq.html?Leucokinin), [NetB](http://flybase.bio.indiana.edu/.bin/fbidq.html?NetB), [rdgC](http://flybase.bio.indiana.edu/.bin/fbidq.html?rdgC), [Ssrp](http://flybase.bio.indiana.edu/.bin/fbidq.html?Ssrp), [Lar](http://flybase.bio.indiana.edu/.bin/fbidq.html?Lar), [scrib](http://flybase.bio.indiana.edu/.bin/fbidq.html?scrib), [HGTX](http://flybase.bio.indiana.edu/.bin/fbidq.html?HGTX), [not](http://flybase.bio.indiana.edu/.bin/fbidq.html?not), [nvy](http://flybase.bio.indiana.edu/.bin/fbidq.html?nvy), [CG7650](http://flybase.bio.indiana.edu/.bin/fbidq.html?CG7650), [nab](http://flybase.bio.indiana.edu/.bin/fbidq.html?nab), [U2af38](http://flybase.bio.indiana.edu/.bin/fbidq.html?U2af38), [HLHmdelta](http://flybase.bio.indiana.edu/.bin/fbidq.html?HLHmdelta), [ATbp](http://flybase.bio.indiana.edu/.bin/fbidq.html?ATbp), [7B2](http://flybase.bio.indiana.edu/.bin/fbidq.html?7B2), [alph](http://flybase.bio.indiana.edu/.bin/fbidq.html?alph), [Traf4](http://flybase.bio.indiana.edu/.bin/fbidq.html?Traf4), [TBPH](http://flybase.bio.indiana.edu/.bin/fbidq.html?TBPH), [Snap25](http://flybase.bio.indiana.edu/.bin/fbidq.html?Snap25), [Fer2](http://flybase.bio.indiana.edu/.bin/fbidq.html?Fer2), [CG34400](http://flybase.bio.indiana.edu/.bin/fbidq.html?CG34400),[mGluRA](http://flybase.bio.indiana.edu/.bin/fbidq.html?mGluRA), [ena](http://flybase.bio.indiana.edu/.bin/fbidq.html?ena), [dsh](http://flybase.bio.indiana.edu/.bin/fbidq.html?dsh), [Nf-YC](http://flybase.bio.indiana.edu/.bin/fbidq.html?Nf-YC), [Pli](http://flybase.bio.indiana.edu/.bin/fbidq.html?Pli), [Hel25E](http://flybase.bio.indiana.edu/.bin/fbidq.html?Hel25E), [comt](http://flybase.bio.indiana.edu/.bin/fbidq.html?comt), [CG31140](http://flybase.bio.indiana.edu/.bin/fbidq.html?CG31140), [PP2A-B](http://flybase.bio.indiana.edu/.bin/fbidq.html?PP2A-B), [qvr](http://flybase.bio.indiana.edu/.bin/fbidq.html?qvr), [DAAM](http://flybase.bio.indiana.edu/.bin/fbidq.html?DAAM), [rho-5](http://flybase.bio.indiana.edu/.bin/fbidq.html?rho-5), [nmo](http://flybase.bio.indiana.edu/.bin/fbidq.html?nmo), [Tusp](http://flybase.bio.indiana.edu/.bin/fbidq.html?Tusp), [5-HT1A](http://flybase.bio.indiana.edu/.bin/fbidq.html?5-HT1A), [Psi](http://flybase.bio.indiana.edu/.bin/fbidq.html?Psi), [Gug](http://flybase.bio.indiana.edu/.bin/fbidq.html?Gug), [ed](http://flybase.bio.indiana.edu/.bin/fbidq.html?ed), [pad](http://flybase.bio.indiana.edu/.bin/fbidq.html?pad), [daw](http://flybase.bio.indiana.edu/.bin/fbidq.html?daw), [CG5036](http://flybase.bio.indiana.edu/.bin/fbidq.html?CG5036), [5-HT1B](http://flybase.bio.indiana.edu/.bin/fbidq.html?5-HT1B), [Rx](http://flybase.bio.indiana.edu/.bin/fbidq.html?Rx), [MED1](http://flybase.bio.indiana.edu/.bin/fbidq.html?MED1), [salr](http://flybase.bio.indiana.edu/.bin/fbidq.html?salr), [Apc](http://flybase.bio.indiana.edu/.bin/fbidq.html?Apc), [CG8795](http://flybase.bio.indiana.edu/.bin/fbidq.html?CG8795), [d4](http://flybase.bio.indiana.edu/.bin/fbidq.html?d4), [dan](http://flybase.bio.indiana.edu/.bin/fbidq.html?dan), [cort](http://flybase.bio.indiana.edu/.bin/fbidq.html?cort), [Jarid2](http://flybase.bio.indiana.edu/.bin/fbidq.html?Jarid2), [RN-tre](http://flybase.bio.indiana.edu/.bin/fbidq.html?RN-tre), [trx](http://flybase.bio.indiana.edu/.bin/fbidq.html?trx), [Btk29A](http://flybase.bio.indiana.edu/.bin/fbidq.html?Btk29A), [MED25](http://flybase.bio.indiana.edu/.bin/fbidq.html?MED25), [ETH](http://flybase.bio.indiana.edu/.bin/fbidq.html?ETH), [unc-5](http://flybase.bio.indiana.edu/.bin/fbidq.html?unc-5),[cdi](http://flybase.bio.indiana.edu/.bin/fbidq.html?cdi), [vfl](http://flybase.bio.indiana.edu/.bin/fbidq.html?vfl), [NPFR1](http://flybase.bio.indiana.edu/.bin/fbidq.html?NPFR1), [pygo](http://flybase.bio.indiana.edu/.bin/fbidq.html?pygo), [z](http://flybase.bio.indiana.edu/.bin/fbidq.html?z), [HLHmgamma](http://flybase.bio.indiana.edu/.bin/fbidq.html?HLHmgamma), [CG15556](http://flybase.bio.indiana.edu/.bin/fbidq.html?CG15556), [jar](http://flybase.bio.indiana.edu/.bin/fbidq.html?jar), [Ror](http://flybase.bio.indiana.edu/.bin/fbidq.html?Ror), [wor](http://flybase.bio.indiana.edu/.bin/fbidq.html?wor), [Atg1](http://flybase.bio.indiana.edu/.bin/fbidq.html?Atg1), [pUf68](http://flybase.bio.indiana.edu/.bin/fbidq.html?pUf68), [seq](http://flybase.bio.indiana.edu/.bin/fbidq.html?seq), [HDAC4](http://flybase.bio.indiana.edu/.bin/fbidq.html?HDAC4), [Cbl](http://flybase.bio.indiana.edu/.bin/fbidq.html?Cbl), [thr](http://flybase.bio.indiana.edu/.bin/fbidq.html?thr), [pog](http://flybase.bio.indiana.edu/.bin/fbidq.html?pog), [Pdp1](http://flybase.bio.indiana.edu/.bin/fbidq.html?Pdp1), [CASK](http://flybase.bio.indiana.edu/.bin/fbidq.html?CASK), [CG32758](http://flybase.bio.indiana.edu/.bin/fbidq.html?CG32758), [mtt](http://flybase.bio.indiana.edu/.bin/fbidq.html?mtt), [disp](http://flybase.bio.indiana.edu/.bin/fbidq.html?disp), [mip120](http://flybase.bio.indiana.edu/.bin/fbidq.html?mip120), [cpx](http://flybase.bio.indiana.edu/.bin/fbidq.html?cpx), [CG4022](http://flybase.bio.indiana.edu/.bin/fbidq.html?CG4022), [jumu](http://flybase.bio.indiana.edu/.bin/fbidq.html?jumu), [Usp7](http://flybase.bio.indiana.edu/.bin/fbidq.html?Usp7), [fd59A](http://flybase.bio.indiana.edu/.bin/fbidq.html?fd59A), [brat](http://flybase.bio.indiana.edu/.bin/fbidq.html?brat), [FBX011](http://flybase.bio.indiana.edu/.bin/fbidq.html?FBX011), [CG17343](http://flybase.bio.indiana.edu/.bin/fbidq.html?CG17343), [GABA-B-R2](http://flybase.bio.indiana.edu/.bin/fbidq.html?GABA-B-R2), [futsch](http://flybase.bio.indiana.edu/.bin/fbidq.html?futsch), [Ubx](http://flybase.bio.indiana.edu/.bin/fbidq.html?Ubx), [Jupiter](http://flybase.bio.indiana.edu/.bin/fbidq.html?Jupiter),[chm](http://flybase.bio.indiana.edu/.bin/fbidq.html?chm), [Rim](http://flybase.bio.indiana.edu/.bin/fbidq.html?Rim), [corn](http://flybase.bio.indiana.edu/.bin/fbidq.html?corn), [Sur-8](http://flybase.bio.indiana.edu/.bin/fbidq.html?Sur-8), [Rgl](http://flybase.bio.indiana.edu/.bin/fbidq.html?Rgl), [CG9007](http://flybase.bio.indiana.edu/.bin/fbidq.html?CG9007), [mav](http://flybase.bio.indiana.edu/.bin/fbidq.html?mav), [gol](http://flybase.bio.indiana.edu/.bin/fbidq.html?gol), [Ilp7](http://flybase.bio.indiana.edu/.bin/fbidq.html?Ilp7), [CG33275](http://flybase.bio.indiana.edu/.bin/fbidq.html?CG33275), [toy](http://flybase.bio.indiana.edu/.bin/fbidq.html?toy), [CG40351](http://flybase.bio.indiana.edu/.bin/fbidq.html?CG40351), [Lim3](http://flybase.bio.indiana.edu/.bin/fbidq.html?Lim3), [sd](http://flybase.bio.indiana.edu/.bin/fbidq.html?sd), [Aplip1](http://flybase.bio.indiana.edu/.bin/fbidq.html?Aplip1), [fs(1)h](http://flybase.bio.indiana.edu/.bin/fbidq.html?fs(1)h), [CG11347](http://flybase.bio.indiana.edu/.bin/fbidq.html?CG11347), [Ggamma30A](http://flybase.bio.indiana.edu/.bin/fbidq.html?Ggamma30A), [CG11294](http://flybase.bio.indiana.edu/.bin/fbidq.html?CG11294), [wls](http://flybase.bio.indiana.edu/.bin/fbidq.html?wls), [skl](http://flybase.bio.indiana.edu/.bin/fbidq.html?skl), [hiw](http://flybase.bio.indiana.edu/.bin/fbidq.html?hiw), [Atf-2](http://flybase.bio.indiana.edu/.bin/fbidq.html?Atf-2), [skd](http://flybase.bio.indiana.edu/.bin/fbidq.html?skd), [jing](http://flybase.bio.indiana.edu/.bin/fbidq.html?jing), [katanin-60](http://flybase.bio.indiana.edu/.bin/fbidq.html?katanin-60), [Camta](http://flybase.bio.indiana.edu/.bin/fbidq.html?Camta), [tll](http://flybase.bio.indiana.edu/.bin/fbidq.html?tll), [ato](http://flybase.bio.indiana.edu/.bin/fbidq.html?ato), [Fmrf](http://flybase.bio.indiana.edu/.bin/fbidq.html?Fmrf), [Iswi](http://flybase.bio.indiana.edu/.bin/fbidq.html?Iswi), [Spt6](http://flybase.bio.indiana.edu/.bin/fbidq.html?Spt6), [obst-A](http://flybase.bio.indiana.edu/.bin/fbidq.html?obst-A), [Pka-R1](http://flybase.bio.indiana.edu/.bin/fbidq.html?Pka-R1), [mago](http://flybase.bio.indiana.edu/.bin/fbidq.html?mago), [enc](http://flybase.bio.indiana.edu/.bin/fbidq.html?enc),[CG42533](http://flybase.bio.indiana.edu/.bin/fbidq.html?CG42533), [ksr](http://flybase.bio.indiana.edu/.bin/fbidq.html?ksr), [CG3618](http://flybase.bio.indiana.edu/.bin/fbidq.html?CG3618), [Gyc-89Da](http://flybase.bio.indiana.edu/.bin/fbidq.html?Gyc-89Da), [brp](http://flybase.bio.indiana.edu/.bin/fbidq.html?brp), [chn](http://flybase.bio.indiana.edu/.bin/fbidq.html?chn), [CG17124](http://flybase.bio.indiana.edu/.bin/fbidq.html?CG17124), [chif](http://flybase.bio.indiana.edu/.bin/fbidq.html?chif), [D2R](http://flybase.bio.indiana.edu/.bin/fbidq.html?D2R), [ewg](http://flybase.bio.indiana.edu/.bin/fbidq.html?ewg), [PHDP](http://flybase.bio.indiana.edu/.bin/fbidq.html?PHDP), [Cirl](http://flybase.bio.indiana.edu/.bin/fbidq.html?Cirl), [APC4](http://flybase.bio.indiana.edu/.bin/fbidq.html?APC4), [l(2)NC136](http://flybase.bio.indiana.edu/.bin/fbidq.html?l(2)NC136), [Su(var)3-3](http://flybase.bio.indiana.edu/.bin/fbidq.html?Su(var)3-3), [Bili](http://flybase.bio.indiana.edu/.bin/fbidq.html?Bili), [Dh44-R1](http://flybase.bio.indiana.edu/.bin/fbidq.html?Dh44-R1), [stet](http://flybase.bio.indiana.edu/.bin/fbidq.html?stet), [Sema-1a](http://flybase.bio.indiana.edu/.bin/fbidq.html?Sema-1a), [Eh](http://flybase.bio.indiana.edu/.bin/fbidq.html?Eh), [Hs3st-B](http://flybase.bio.indiana.edu/.bin/fbidq.html?Hs3st-B), [Sos](http://flybase.bio.indiana.edu/.bin/fbidq.html?Sos), [Fas2](http://flybase.bio.indiana.edu/.bin/fbidq.html?Fas2), [Klp61F](http://flybase.bio.indiana.edu/.bin/fbidq.html?Klp61F), [eya](http://flybase.bio.indiana.edu/.bin/fbidq.html?eya), [mAcR-60C](http://flybase.bio.indiana.edu/.bin/fbidq.html?mAcR-60C), [CG8557](http://flybase.bio.indiana.edu/.bin/fbidq.html?CG8557), [Vsx2](http://flybase.bio.indiana.edu/.bin/fbidq.html?Vsx2), [kat-60L1](http://flybase.bio.indiana.edu/.bin/fbidq.html?kat-60L1), [mtg](http://flybase.bio.indiana.edu/.bin/fbidq.html?mtg), [ft](http://flybase.bio.indiana.edu/.bin/fbidq.html?ft), [Crz](http://flybase.bio.indiana.edu/.bin/fbidq.html?Crz),[l(2)k16918](http://flybase.bio.indiana.edu/.bin/fbidq.html?l(2)k16918), [Takr86C](http://flybase.bio.indiana.edu/.bin/fbidq.html?Takr86C), [Dll](http://flybase.bio.indiana.edu/.bin/fbidq.html?Dll), [disco](http://flybase.bio.indiana.edu/.bin/fbidq.html?disco), [DopR](http://flybase.bio.indiana.edu/.bin/fbidq.html?DopR), [D12](http://flybase.bio.indiana.edu/.bin/fbidq.html?D12), [didum](http://flybase.bio.indiana.edu/.bin/fbidq.html?didum), [Lis-1](http://flybase.bio.indiana.edu/.bin/fbidq.html?Lis-1), [ems](http://flybase.bio.indiana.edu/.bin/fbidq.html?ems), [CG16896](http://flybase.bio.indiana.edu/.bin/fbidq.html?CG16896), [tup](http://flybase.bio.indiana.edu/.bin/fbidq.html?tup), [Rab3](http://flybase.bio.indiana.edu/.bin/fbidq.html?Rab3), [CG6191](http://flybase.bio.indiana.edu/.bin/fbidq.html?CG6191), [trio](http://flybase.bio.indiana.edu/.bin/fbidq.html?trio), [pdm2](http://flybase.bio.indiana.edu/.bin/fbidq.html?pdm2), [RabX4](http://flybase.bio.indiana.edu/.bin/fbidq.html?RabX4), [rgr](http://flybase.bio.indiana.edu/.bin/fbidq.html?rgr), [Lkr](http://flybase.bio.indiana.edu/.bin/fbidq.html?Lkr), [spel1](http://flybase.bio.indiana.edu/.bin/fbidq.html?spel1), [CG31665](http://flybase.bio.indiana.edu/.bin/fbidq.html?CG31665), [CG7918](http://flybase.bio.indiana.edu/.bin/fbidq.html?CG7918), [CaMKII](http://flybase.bio.indiana.edu/.bin/fbidq.html?CaMKII), [DopR2](http://flybase.bio.indiana.edu/.bin/fbidq.html?DopR2), [l(3)psg2](http://flybase.bio.indiana.edu/.bin/fbidq.html?l(3)psg2), [nerfin-1](http://flybase.bio.indiana.edu/.bin/fbidq.html?nerfin-1), [pigs](http://flybase.bio.indiana.edu/.bin/fbidq.html?pigs), [5-HT7](http://flybase.bio.indiana.edu/.bin/fbidq.html?5-HT7), [Lgr3](http://flybase.bio.indiana.edu/.bin/fbidq.html?Lgr3), [polo](http://flybase.bio.indiana.edu/.bin/fbidq.html?polo), [Syn1](http://flybase.bio.indiana.edu/.bin/fbidq.html?Syn1), [Ac13E](http://flybase.bio.indiana.edu/.bin/fbidq.html?Ac13E), [Ssdp](http://flybase.bio.indiana.edu/.bin/fbidq.html?Ssdp),[CG8155](http://flybase.bio.indiana.edu/.bin/fbidq.html?CG8155), [CG32105](http://flybase.bio.indiana.edu/.bin/fbidq.html?CG32105), [Fer3](http://flybase.bio.indiana.edu/.bin/fbidq.html?Fer3), [dalao](http://flybase.bio.indiana.edu/.bin/fbidq.html?dalao), [sr](http://flybase.bio.indiana.edu/.bin/fbidq.html?sr), [Lim1](http://flybase.bio.indiana.edu/.bin/fbidq.html?Lim1), [nkd](http://flybase.bio.indiana.edu/.bin/fbidq.html?nkd), [RhoGEF3](http://flybase.bio.indiana.edu/.bin/fbidq.html?RhoGEF3), [danr](http://flybase.bio.indiana.edu/.bin/fbidq.html?danr), [Dsk](http://flybase.bio.indiana.edu/.bin/fbidq.html?Dsk), [RhoGEF4](http://flybase.bio.indiana.edu/.bin/fbidq.html?RhoGEF4), [vvl](http://flybase.bio.indiana.edu/.bin/fbidq.html?vvl), [betaInt-nu](http://flybase.bio.indiana.edu/.bin/fbidq.html?betaInt-nu), [sgg](http://flybase.bio.indiana.edu/.bin/fbidq.html?sgg), [Brd](http://flybase.bio.indiana.edu/.bin/fbidq.html?Brd), [Nmdar1](http://flybase.bio.indiana.edu/.bin/fbidq.html?Nmdar1), [Eip93F](http://flybase.bio.indiana.edu/.bin/fbidq.html?Eip93F), [gcm](http://flybase.bio.indiana.edu/.bin/fbidq.html?gcm), [Hsp67Bc](http://flybase.bio.indiana.edu/.bin/fbidq.html?Hsp67Bc), [ase](http://flybase.bio.indiana.edu/.bin/fbidq.html?ase), [scro](http://flybase.bio.indiana.edu/.bin/fbidq.html?scro), [elav](http://flybase.bio.indiana.edu/.bin/fbidq.html?elav), [MESK2](http://flybase.bio.indiana.edu/.bin/fbidq.html?MESK2), [CG6227](http://flybase.bio.indiana.edu/.bin/fbidq.html?CG6227), [Poxn](http://flybase.bio.indiana.edu/.bin/fbidq.html?Poxn), [sals](http://flybase.bio.indiana.edu/.bin/fbidq.html?sals), [CG15376](http://flybase.bio.indiana.edu/.bin/fbidq.html?CG15376), [fred](http://flybase.bio.indiana.edu/.bin/fbidq.html?fred), [ush](http://flybase.bio.indiana.edu/.bin/fbidq.html?ush), [Fak56D](http://flybase.bio.indiana.edu/.bin/fbidq.html?Fak56D), [Takr99D](http://flybase.bio.indiana.edu/.bin/fbidq.html?Takr99D), [Alh](http://flybase.bio.indiana.edu/.bin/fbidq.html?Alh), [Sox102F](http://flybase.bio.indiana.edu/.bin/fbidq.html?Sox102F),[egg](http://flybase.bio.indiana.edu/.bin/fbidq.html?egg), [CG13692](http://flybase.bio.indiana.edu/.bin/fbidq.html?CG13692), [CG5337](http://flybase.bio.indiana.edu/.bin/fbidq.html?CG5337), [CG12424](http://flybase.bio.indiana.edu/.bin/fbidq.html?CG12424), [Sp1](http://flybase.bio.indiana.edu/.bin/fbidq.html?Sp1), [Wnt5](http://flybase.bio.indiana.edu/.bin/fbidq.html?Wnt5), [fus](http://flybase.bio.indiana.edu/.bin/fbidq.html?fus), [sif](http://flybase.bio.indiana.edu/.bin/fbidq.html?sif), [Tbh](http://flybase.bio.indiana.edu/.bin/fbidq.html?Tbh), [Aac11](http://flybase.bio.indiana.edu/.bin/fbidq.html?Aac11), [MTA1-like](http://flybase.bio.indiana.edu/.bin/fbidq.html?MTA1-like), [ssh](http://flybase.bio.indiana.edu/.bin/fbidq.html?ssh), [CG42541](http://flybase.bio.indiana.edu/.bin/fbidq.html?CG42541), [PIP5K59B](http://flybase.bio.indiana.edu/.bin/fbidq.html?PIP5K59B), [mle](http://flybase.bio.indiana.edu/.bin/fbidq.html?mle), [Hr51](http://flybase.bio.indiana.edu/.bin/fbidq.html?Hr51), [CG11155](http://flybase.bio.indiana.edu/.bin/fbidq.html?CG11155), [wg](http://flybase.bio.indiana.edu/.bin/fbidq.html?wg), [fkh](http://flybase.bio.indiana.edu/.bin/fbidq.html?fkh), [Spps](http://flybase.bio.indiana.edu/.bin/fbidq.html?Spps), [Rhp](http://flybase.bio.indiana.edu/.bin/fbidq.html?Rhp), [fne](http://flybase.bio.indiana.edu/.bin/fbidq.html?fne), [en](http://flybase.bio.indiana.edu/.bin/fbidq.html?en), [sfl](http://flybase.bio.indiana.edu/.bin/fbidq.html?sfl), [CG13229](http://flybase.bio.indiana.edu/.bin/fbidq.html?CG13229), [Pde11](http://flybase.bio.indiana.edu/.bin/fbidq.html?Pde11), [Utx](http://flybase.bio.indiana.edu/.bin/fbidq.html?Utx), [Ct](http://flybase.bio.indiana.edu/.bin/fbidq.html?Ct), [fz2](http://flybase.bio.indiana.edu/.bin/fbidq.html?fz2), [numb](http://flybase.bio.indiana.edu/.bin/fbidq.html?numb), [Ocho](http://flybase.bio.indiana.edu/.bin/fbidq.html?Ocho), [CG14375](http://flybase.bio.indiana.edu/.bin/fbidq.html?CG14375), [Ets65A](http://flybase.bio.indiana.edu/.bin/fbidq.html?Ets65A), [Sxl](http://flybase.bio.indiana.edu/.bin/fbidq.html?Sxl), [stj](http://flybase.bio.indiana.edu/.bin/fbidq.html?stj), [JIL-1](http://flybase.bio.indiana.edu/.bin/fbidq.html?JIL-1),[Nf1](http://flybase.bio.indiana.edu/.bin/fbidq.html?Nf1), [AR-2](http://flybase.bio.indiana.edu/.bin/fbidq.html?AR-2), [bi](http://flybase.bio.indiana.edu/.bin/fbidq.html?bi), [NC2alpha](http://flybase.bio.indiana.edu/.bin/fbidq.html?NC2alpha), [blue](http://flybase.bio.indiana.edu/.bin/fbidq.html?blue), [mod(mdg4)](http://flybase.bio.indiana.edu/.bin/fbidq.html?mod(mdg4)), [bab1](http://flybase.bio.indiana.edu/.bin/fbidq.html?bab1), [Galpha49B](http://flybase.bio.indiana.edu/.bin/fbidq.html?Galpha49B), [Snoo](http://flybase.bio.indiana.edu/.bin/fbidq.html?Snoo), [rut](http://flybase.bio.indiana.edu/.bin/fbidq.html?rut), [RhoGAP100F](http://flybase.bio.indiana.edu/.bin/fbidq.html?RhoGAP100F), [uif](http://flybase.bio.indiana.edu/.bin/fbidq.html?uif), [nub](http://flybase.bio.indiana.edu/.bin/fbidq.html?nub), [btd](http://flybase.bio.indiana.edu/.bin/fbidq.html?btd), [Mmp2](http://flybase.bio.indiana.edu/.bin/fbidq.html?Mmp2), [fd68A](http://flybase.bio.indiana.edu/.bin/fbidq.html?fd68A), [Nplp1](http://flybase.bio.indiana.edu/.bin/fbidq.html?Nplp1), [B-H1](http://flybase.bio.indiana.edu/.bin/fbidq.html?B-H1), [CG32206](http://flybase.bio.indiana.edu/.bin/fbidq.html?CG32206), [Nipped-A](http://flybase.bio.indiana.edu/.bin/fbidq.html?Nipped-A), [fd102C](http://flybase.bio.indiana.edu/.bin/fbidq.html?fd102C), [Spf45](http://flybase.bio.indiana.edu/.bin/fbidq.html?Spf45), [hth](http://flybase.bio.indiana.edu/.bin/fbidq.html?hth), [14-3-3zeta](http://flybase.bio.indiana.edu/.bin/fbidq.html?14-3-3zeta), [pzg](http://flybase.bio.indiana.edu/.bin/fbidq.html?pzg), [lea](http://flybase.bio.indiana.edu/.bin/fbidq.html?lea), [shakB](http://flybase.bio.indiana.edu/.bin/fbidq.html?shakB), [Hey](http://flybase.bio.indiana.edu/.bin/fbidq.html?Hey), [plexB](http://flybase.bio.indiana.edu/.bin/fbidq.html?plexB), [AlstR](http://flybase.bio.indiana.edu/.bin/fbidq.html?AlstR), [loqs](http://flybase.bio.indiana.edu/.bin/fbidq.html?loqs), [mars](http://flybase.bio.indiana.edu/.bin/fbidq.html?mars), [IM10](http://flybase.bio.indiana.edu/.bin/fbidq.html?IM10),[neur](http://flybase.bio.indiana.edu/.bin/fbidq.html?neur), [Brf](http://flybase.bio.indiana.edu/.bin/fbidq.html?Brf), [CycE](http://flybase.bio.indiana.edu/.bin/fbidq.html?CycE), [CG32149](http://flybase.bio.indiana.edu/.bin/fbidq.html?CG32149), [Vdup1](http://flybase.bio.indiana.edu/.bin/fbidq.html?Vdup1), [mr](http://flybase.bio.indiana.edu/.bin/fbidq.html?mr), [orb2](http://flybase.bio.indiana.edu/.bin/fbidq.html?orb2), [wdn](http://flybase.bio.indiana.edu/.bin/fbidq.html?wdn), [synaptogyrin](http://flybase.bio.indiana.edu/.bin/fbidq.html?synaptogyrin), [sim](http://flybase.bio.indiana.edu/.bin/fbidq.html?sim), [gro](http://flybase.bio.indiana.edu/.bin/fbidq.html?gro), [Rya-r44F](http://flybase.bio.indiana.edu/.bin/fbidq.html?Rya-r44F), [scyl](http://flybase.bio.indiana.edu/.bin/fbidq.html?scyl), [hkl](http://flybase.bio.indiana.edu/.bin/fbidq.html?hkl), [bin3](http://flybase.bio.indiana.edu/.bin/fbidq.html?bin3), [Rpd3](http://flybase.bio.indiana.edu/.bin/fbidq.html?Rpd3), [tyf](http://flybase.bio.indiana.edu/.bin/fbidq.html?tyf), [CG10107](http://flybase.bio.indiana.edu/.bin/fbidq.html?CG10107), [aret](http://flybase.bio.indiana.edu/.bin/fbidq.html?aret), [HLHm7](http://flybase.bio.indiana.edu/.bin/fbidq.html?HLHm7), [Sox21b](http://flybase.bio.indiana.edu/.bin/fbidq.html?Sox21b), [tow](http://flybase.bio.indiana.edu/.bin/fbidq.html?tow), [CG15609](http://flybase.bio.indiana.edu/.bin/fbidq.html?CG15609), [CG12187](http://flybase.bio.indiana.edu/.bin/fbidq.html?CG12187), [Pten](http://flybase.bio.indiana.edu/.bin/fbidq.html?Pten), [unc-13](http://flybase.bio.indiana.edu/.bin/fbidq.html?unc-13), [wda](http://flybase.bio.indiana.edu/.bin/fbidq.html?wda), [npf](http://flybase.bio.indiana.edu/.bin/fbidq.html?npf), [mira](http://flybase.bio.indiana.edu/.bin/fbidq.html?mira), [MED14](http://flybase.bio.indiana.edu/.bin/fbidq.html?MED14), [Spindly](http://flybase.bio.indiana.edu/.bin/fbidq.html?Spindly), [Drep-2](http://flybase.bio.indiana.edu/.bin/fbidq.html?Drep-2), [Dip3](http://flybase.bio.indiana.edu/.bin/fbidq.html?Dip3), [oa2](http://flybase.bio.indiana.edu/.bin/fbidq.html?oa2), [elk](http://flybase.bio.indiana.edu/.bin/fbidq.html?elk),[Rab26](http://flybase.bio.indiana.edu/.bin/fbidq.html?Rab26), [phl](http://flybase.bio.indiana.edu/.bin/fbidq.html?phl), [sNPF](http://flybase.bio.indiana.edu/.bin/fbidq.html?sNPF), [Pde6](http://flybase.bio.indiana.edu/.bin/fbidq.html?Pde6), [plx](http://flybase.bio.indiana.edu/.bin/fbidq.html?plx), [CG9098](http://flybase.bio.indiana.edu/.bin/fbidq.html?CG9098), [CG8108](http://flybase.bio.indiana.edu/.bin/fbidq.html?CG8108), [IFa](http://flybase.bio.indiana.edu/.bin/fbidq.html?IFa), [Alk](http://flybase.bio.indiana.edu/.bin/fbidq.html?Alk), [Pde1c](http://flybase.bio.indiana.edu/.bin/fbidq.html?Pde1c), [Sin3A](http://flybase.bio.indiana.edu/.bin/fbidq.html?Sin3A), [casp](http://flybase.bio.indiana.edu/.bin/fbidq.html?casp), [Smox](http://flybase.bio.indiana.edu/.bin/fbidq.html?Smox), [Plc21C](http://flybase.bio.indiana.edu/.bin/fbidq.html?Plc21C), [Antp](http://flybase.bio.indiana.edu/.bin/fbidq.html?Antp), [Rdl](http://flybase.bio.indiana.edu/.bin/fbidq.html?Rdl), [GRHRII](http://flybase.bio.indiana.edu/.bin/fbidq.html?GRHRII), [l(3)neo38](http://flybase.bio.indiana.edu/.bin/fbidq.html?l(3)neo38), [CG10362](http://flybase.bio.indiana.edu/.bin/fbidq.html?CG10362), [Ast-C](http://flybase.bio.indiana.edu/.bin/fbidq.html?Ast-C), [cindr](http://flybase.bio.indiana.edu/.bin/fbidq.html?cindr), [Gbeta5](http://flybase.bio.indiana.edu/.bin/fbidq.html?Gbeta5), [Ptx1](http://flybase.bio.indiana.edu/.bin/fbidq.html?Ptx1), [hug](http://flybase.bio.indiana.edu/.bin/fbidq.html?hug), [hbn](http://flybase.bio.indiana.edu/.bin/fbidq.html?hbn), [Syn2](http://flybase.bio.indiana.edu/.bin/fbidq.html?Syn2), [tsh](http://flybase.bio.indiana.edu/.bin/fbidq.html?tsh), [e(y)3](http://flybase.bio.indiana.edu/.bin/fbidq.html?e(y)3), [CRMP](http://flybase.bio.indiana.edu/.bin/fbidq.html?CRMP), [Cdk5](http://flybase.bio.indiana.edu/.bin/fbidq.html?Cdk5), [oc](http://flybase.bio.indiana.edu/.bin/fbidq.html?oc), [Cep135](http://flybase.bio.indiana.edu/.bin/fbidq.html?Cep135), [spt4](http://flybase.bio.indiana.edu/.bin/fbidq.html?spt4), [CG4641](http://flybase.bio.indiana.edu/.bin/fbidq.html?CG4641), [ey](http://flybase.bio.indiana.edu/.bin/fbidq.html?ey), [baz](http://flybase.bio.indiana.edu/.bin/fbidq.html?baz),[Mip](http://flybase.bio.indiana.edu/.bin/fbidq.html?Mip), [Oamb](http://flybase.bio.indiana.edu/.bin/fbidq.html?Oamb), [CG34381](http://flybase.bio.indiana.edu/.bin/fbidq.html?CG34381), [l(1)sc](http://flybase.bio.indiana.edu/.bin/fbidq.html?l(1)sc), [Bgb](http://flybase.bio.indiana.edu/.bin/fbidq.html?Bgb), [comm](http://flybase.bio.indiana.edu/.bin/fbidq.html?comm), [grh](http://flybase.bio.indiana.edu/.bin/fbidq.html?grh), [Cdk5alpha](http://flybase.bio.indiana.edu/.bin/fbidq.html?Cdk5alpha), [Vsx1](http://flybase.bio.indiana.edu/.bin/fbidq.html?Vsx1), [CG34362](http://flybase.bio.indiana.edu/.bin/fbidq.html?CG34362), [crol](http://flybase.bio.indiana.edu/.bin/fbidq.html?crol), [retn](http://flybase.bio.indiana.edu/.bin/fbidq.html?retn), [Ac3](http://flybase.bio.indiana.edu/.bin/fbidq.html?Ac3), [Syt1](http://flybase.bio.indiana.edu/.bin/fbidq.html?Syt1), [rl](http://flybase.bio.indiana.edu/.bin/fbidq.html?rl), [CG17760](http://flybase.bio.indiana.edu/.bin/fbidq.html?CG17760), [CG32683](http://flybase.bio.indiana.edu/.bin/fbidq.html?CG32683), [Nup153](http://flybase.bio.indiana.edu/.bin/fbidq.html?Nup153), [Saf-B](http://flybase.bio.indiana.edu/.bin/fbidq.html?Saf-B), [tey](http://flybase.bio.indiana.edu/.bin/fbidq.html?tey), [CG33960](http://flybase.bio.indiana.edu/.bin/fbidq.html?CG33960), [dnr1](http://flybase.bio.indiana.edu/.bin/fbidq.html?dnr1), [acj6](http://flybase.bio.indiana.edu/.bin/fbidq.html?acj6), [caup](http://flybase.bio.indiana.edu/.bin/fbidq.html?caup), [Cbp80](http://flybase.bio.indiana.edu/.bin/fbidq.html?Cbp80), [vimar](http://flybase.bio.indiana.edu/.bin/fbidq.html?vimar), [Rox8](http://flybase.bio.indiana.edu/.bin/fbidq.html?Rox8), [Dg](http://flybase.bio.indiana.edu/.bin/fbidq.html?Dg), [Trim9](http://flybase.bio.indiana.edu/.bin/fbidq.html?Trim9), [GABA-B-R3](http://flybase.bio.indiana.edu/.bin/fbidq.html?GABA-B-R3), [Evi5](http://flybase.bio.indiana.edu/.bin/fbidq.html?Evi5), [Gycalpha99B](http://flybase.bio.indiana.edu/.bin/fbidq.html?Gycalpha99B), [br](http://flybase.bio.indiana.edu/.bin/fbidq.html?br),[neo](http://flybase.bio.indiana.edu/.bin/fbidq.html?neo), [CG11376](http://flybase.bio.indiana.edu/.bin/fbidq.html?CG11376), [PNUTS](http://flybase.bio.indiana.edu/.bin/fbidq.html?PNUTS), [Gsc](http://flybase.bio.indiana.edu/.bin/fbidq.html?Gsc), [lola](http://flybase.bio.indiana.edu/.bin/fbidq.html?lola), [HLH4C](http://flybase.bio.indiana.edu/.bin/fbidq.html?HLH4C), [Pcl](http://flybase.bio.indiana.edu/.bin/fbidq.html?Pcl), [simj](http://flybase.bio.indiana.edu/.bin/fbidq.html?simj), [CG31158](http://flybase.bio.indiana.edu/.bin/fbidq.html?CG31158), [tap](http://flybase.bio.indiana.edu/.bin/fbidq.html?tap), [Appl](http://flybase.bio.indiana.edu/.bin/fbidq.html?Appl), [Awh](http://flybase.bio.indiana.edu/.bin/fbidq.html?Awh), [metro](http://flybase.bio.indiana.edu/.bin/fbidq.html?metro), [Nrk](http://flybase.bio.indiana.edu/.bin/fbidq.html?Nrk), [erm](http://flybase.bio.indiana.edu/.bin/fbidq.html?erm), [CG4328](http://flybase.bio.indiana.edu/.bin/fbidq.html?CG4328), [Taf4](http://flybase.bio.indiana.edu/.bin/fbidq.html?Taf4), [Ilp3](http://flybase.bio.indiana.edu/.bin/fbidq.html?Ilp3), [Nrg](http://flybase.bio.indiana.edu/.bin/fbidq.html?Nrg), [CG34384](http://flybase.bio.indiana.edu/.bin/fbidq.html?CG34384), [insc](http://flybase.bio.indiana.edu/.bin/fbidq.html?insc), [CG32447](http://flybase.bio.indiana.edu/.bin/fbidq.html?CG32447), [stau](http://flybase.bio.indiana.edu/.bin/fbidq.html?stau), [otk](http://flybase.bio.indiana.edu/.bin/fbidq.html?otk), [phyl](http://flybase.bio.indiana.edu/.bin/fbidq.html?phyl), [vg](http://flybase.bio.indiana.edu/.bin/fbidq.html?vg), [brm](http://flybase.bio.indiana.edu/.bin/fbidq.html?brm), [bsk](http://flybase.bio.indiana.edu/.bin/fbidq.html?bsk), [robo](http://flybase.bio.indiana.edu/.bin/fbidq.html?robo), [fz](http://flybase.bio.indiana.edu/.bin/fbidq.html?fz), [sno](http://flybase.bio.indiana.edu/.bin/fbidq.html?sno), [CG13830](http://flybase.bio.indiana.edu/.bin/fbidq.html?CG13830), [RhoGAPp190](http://flybase.bio.indiana.edu/.bin/fbidq.html?RhoGAPp190), [ara](http://flybase.bio.indiana.edu/.bin/fbidq.html?ara), [Sfmbt](http://flybase.bio.indiana.edu/.bin/fbidq.html?Sfmbt), [mam](http://flybase.bio.indiana.edu/.bin/fbidq.html?mam),[CG30158](http://flybase.bio.indiana.edu/.bin/fbidq.html?CG30158), [pan](http://flybase.bio.indiana.edu/.bin/fbidq.html?pan), [PKD](http://flybase.bio.indiana.edu/.bin/fbidq.html?PKD), [grn](http://flybase.bio.indiana.edu/.bin/fbidq.html?grn), [HLHm5](http://flybase.bio.indiana.edu/.bin/fbidq.html?HLHm5), [dap](http://flybase.bio.indiana.edu/.bin/fbidq.html?dap), [pros](http://flybase.bio.indiana.edu/.bin/fbidq.html?pros), [mthl8](http://flybase.bio.indiana.edu/.bin/fbidq.html?mthl8), [Ccap](http://flybase.bio.indiana.edu/.bin/fbidq.html?Ccap), [Wnt4](http://flybase.bio.indiana.edu/.bin/fbidq.html?Wnt4), [Mad1](http://flybase.bio.indiana.edu/.bin/fbidq.html?Mad1), [CG10188](http://flybase.bio.indiana.edu/.bin/fbidq.html?CG10188), [CG30372](http://flybase.bio.indiana.edu/.bin/fbidq.html?CG30372), [CG42629](http://flybase.bio.indiana.edu/.bin/fbidq.html?CG42629), [CG31760](http://flybase.bio.indiana.edu/.bin/fbidq.html?CG31760), [bchs](http://flybase.bio.indiana.edu/.bin/fbidq.html?bchs), [pr-set7](http://flybase.bio.indiana.edu/.bin/fbidq.html?pr-set7), [Klp54D](http://flybase.bio.indiana.edu/.bin/fbidq.html?Klp54D), [dsx](http://flybase.bio.indiana.edu/.bin/fbidq.html?dsx), [Dh](http://flybase.bio.indiana.edu/.bin/fbidq.html?Dh), [sqz](http://flybase.bio.indiana.edu/.bin/fbidq.html?sqz), [SPR](http://flybase.bio.indiana.edu/.bin/fbidq.html?SPR), [klu](http://flybase.bio.indiana.edu/.bin/fbidq.html?klu), [tld](http://flybase.bio.indiana.edu/.bin/fbidq.html?tld), [CG7879](http://flybase.bio.indiana.edu/.bin/fbidq.html?CG7879), [CG9121](http://flybase.bio.indiana.edu/.bin/fbidq.html?CG9121), [gcm2](http://flybase.bio.indiana.edu/.bin/fbidq.html?gcm2), [inaE](http://flybase.bio.indiana.edu/.bin/fbidq.html?inaE), [CanB](http://flybase.bio.indiana.edu/.bin/fbidq.html?CanB), [can](http://flybase.bio.indiana.edu/.bin/fbidq.html?can), [Aef1](http://flybase.bio.indiana.edu/.bin/fbidq.html?Aef1), [Pask](http://flybase.bio.indiana.edu/.bin/fbidq.html?Pask), [rab3-GEF](http://flybase.bio.indiana.edu/.bin/fbidq.html?rab3-GEF),[CG13253](http://flybase.bio.indiana.edu/.bin/fbidq.html?CG13253), [ovo](http://flybase.bio.indiana.edu/.bin/fbidq.html?ovo), [CG6197](http://flybase.bio.indiana.edu/.bin/fbidq.html?CG6197), [Dsp1](http://flybase.bio.indiana.edu/.bin/fbidq.html?Dsp1), [CG14216](http://flybase.bio.indiana.edu/.bin/fbidq.html?CG14216), [Oli](http://flybase.bio.indiana.edu/.bin/fbidq.html?Oli), [Nelf-A](http://flybase.bio.indiana.edu/.bin/fbidq.html?Nelf-A), [GATAd](http://flybase.bio.indiana.edu/.bin/fbidq.html?GATAd), [Syt4](http://flybase.bio.indiana.edu/.bin/fbidq.html?Syt4), [fbl6](http://flybase.bio.indiana.edu/.bin/fbidq.html?fbl6), [SIFR](http://flybase.bio.indiana.edu/.bin/fbidq.html?SIFR), [CG2061](http://flybase.bio.indiana.edu/.bin/fbidq.html?CG2061), [Ast](http://flybase.bio.indiana.edu/.bin/fbidq.html?Ast), [Drl-2](http://flybase.bio.indiana.edu/.bin/fbidq.html?Drl-2), [CG32944](http://flybase.bio.indiana.edu/.bin/fbidq.html?CG32944), [Tk](http://flybase.bio.indiana.edu/.bin/fbidq.html?Tk), [Ilp5](http://flybase.bio.indiana.edu/.bin/fbidq.html?Ilp5), [CG10336](http://flybase.bio.indiana.edu/.bin/fbidq.html?CG10336), [Gap1](http://flybase.bio.indiana.edu/.bin/fbidq.html?Gap1), [Toll-6](http://flybase.bio.indiana.edu/.bin/fbidq.html?Toll-6), [CG3227](http://flybase.bio.indiana.edu/.bin/fbidq.html?CG3227), [run](http://flybase.bio.indiana.edu/.bin/fbidq.html?run), [MESR4](http://flybase.bio.indiana.edu/.bin/fbidq.html?MESR4), [sNPF-R](http://flybase.bio.indiana.edu/.bin/fbidq.html?sNPF-R), [tara](http://flybase.bio.indiana.edu/.bin/fbidq.html?tara), [Hel89B](http://flybase.bio.indiana.edu/.bin/fbidq.html?Hel89B), [koko](http://flybase.bio.indiana.edu/.bin/fbidq.html?koko), [stan](http://flybase.bio.indiana.edu/.bin/fbidq.html?stan), [CanA-14F](http://flybase.bio.indiana.edu/.bin/fbidq.html?CanA-14F), [tutl](http://flybase.bio.indiana.edu/.bin/fbidq.html?tutl), [Ptth](http://flybase.bio.indiana.edu/.bin/fbidq.html?Ptth), [Rab2](http://flybase.bio.indiana.edu/.bin/fbidq.html?Rab2), [Ilp2](http://flybase.bio.indiana.edu/.bin/fbidq.html?Ilp2), [ph-p](http://flybase.bio.indiana.edu/.bin/fbidq.html?ph-p),[onecut](http://flybase.bio.indiana.edu/.bin/fbidq.html?onecut), [Frq1](http://flybase.bio.indiana.edu/.bin/fbidq.html?Frq1), [CG7757](http://flybase.bio.indiana.edu/.bin/fbidq.html?CG7757), [SPoCk](http://flybase.bio.indiana.edu/.bin/fbidq.html?SPoCk), [sna](http://flybase.bio.indiana.edu/.bin/fbidq.html?sna), [Taf1](http://flybase.bio.indiana.edu/.bin/fbidq.html?Taf1), [Ggamma1](http://flybase.bio.indiana.edu/.bin/fbidq.html?Ggamma1), [MBD-like](http://flybase.bio.indiana.edu/.bin/fbidq.html?MBD-like), [esg](http://flybase.bio.indiana.edu/.bin/fbidq.html?esg), [RSG7](http://flybase.bio.indiana.edu/.bin/fbidq.html?RSG7), [m4](http://flybase.bio.indiana.edu/.bin/fbidq.html?m4), [dom](http://flybase.bio.indiana.edu/.bin/fbidq.html?dom), [CG32532](http://flybase.bio.indiana.edu/.bin/fbidq.html?CG32532), [Dh31](http://flybase.bio.indiana.edu/.bin/fbidq.html?Dh31), [shi](http://flybase.bio.indiana.edu/.bin/fbidq.html?shi), [Spred](http://flybase.bio.indiana.edu/.bin/fbidq.html?Spred), [siz](http://flybase.bio.indiana.edu/.bin/fbidq.html?siz), [B-H2](http://flybase.bio.indiana.edu/.bin/fbidq.html?B-H2), [tyn](http://flybase.bio.indiana.edu/.bin/fbidq.html?tyn), [CG3822](http://flybase.bio.indiana.edu/.bin/fbidq.html?CG3822), [CadN](http://flybase.bio.indiana.edu/.bin/fbidq.html?CadN), [Mfap1](http://flybase.bio.indiana.edu/.bin/fbidq.html?Mfap1), [CG13995](http://flybase.bio.indiana.edu/.bin/fbidq.html?CG13995), [E(bx)](http://flybase.bio.indiana.edu/.bin/fbidq.html?E(bx)), [Dms](http://flybase.bio.indiana.edu/.bin/fbidq.html?Dms), [Pitslre](http://flybase.bio.indiana.edu/.bin/fbidq.html?Pitslre), [pburs](http://flybase.bio.indiana.edu/.bin/fbidq.html?pburs), [cos](http://flybase.bio.indiana.edu/.bin/fbidq.html?cos) |
| [G-protein coupled receptor signaling pathway](http://amigo.geneontology.org/cgi-bin/amigo/go.cgi?view=details&query=GO:0007186) | 76 of 1594 genes, 4.8% | 128 of 7634 genes, 1.7% | 1.00e-19 | 0.00% | 0.00 | [CG15556](http://flybase.bio.indiana.edu/.bin/fbidq.html?CG15556), [CG14375](http://flybase.bio.indiana.edu/.bin/fbidq.html?CG14375), [Dsk](http://flybase.bio.indiana.edu/.bin/fbidq.html?Dsk), [npf](http://flybase.bio.indiana.edu/.bin/fbidq.html?npf), [Camta](http://flybase.bio.indiana.edu/.bin/fbidq.html?Camta), [Fmrf](http://flybase.bio.indiana.edu/.bin/fbidq.html?Fmrf), [GABA-B-R3](http://flybase.bio.indiana.edu/.bin/fbidq.html?GABA-B-R3), [oa2](http://flybase.bio.indiana.edu/.bin/fbidq.html?oa2), [Gycalpha99B](http://flybase.bio.indiana.edu/.bin/fbidq.html?Gycalpha99B), [AR-2](http://flybase.bio.indiana.edu/.bin/fbidq.html?AR-2), [sNPF](http://flybase.bio.indiana.edu/.bin/fbidq.html?sNPF), [pog](http://flybase.bio.indiana.edu/.bin/fbidq.html?pog), [Axn](http://flybase.bio.indiana.edu/.bin/fbidq.html?Axn), [Leucokinin](http://flybase.bio.indiana.edu/.bin/fbidq.html?Leucokinin), [IFa](http://flybase.bio.indiana.edu/.bin/fbidq.html?IFa), [D2R](http://flybase.bio.indiana.edu/.bin/fbidq.html?D2R), [mtt](http://flybase.bio.indiana.edu/.bin/fbidq.html?mtt), [rdgC](http://flybase.bio.indiana.edu/.bin/fbidq.html?rdgC), [SIFR](http://flybase.bio.indiana.edu/.bin/fbidq.html?SIFR), [CG2061](http://flybase.bio.indiana.edu/.bin/fbidq.html?CG2061), [Cirl](http://flybase.bio.indiana.edu/.bin/fbidq.html?Cirl), [Galpha49B](http://flybase.bio.indiana.edu/.bin/fbidq.html?Galpha49B), [Ast](http://flybase.bio.indiana.edu/.bin/fbidq.html?Ast), [rut](http://flybase.bio.indiana.edu/.bin/fbidq.html?rut), [Tk](http://flybase.bio.indiana.edu/.bin/fbidq.html?Tk), [CG34384](http://flybase.bio.indiana.edu/.bin/fbidq.html?CG34384), [Dh44-R1](http://flybase.bio.indiana.edu/.bin/fbidq.html?Dh44-R1), [Nplp1](http://flybase.bio.indiana.edu/.bin/fbidq.html?Nplp1), [CG32447](http://flybase.bio.indiana.edu/.bin/fbidq.html?CG32447), [7B2](http://flybase.bio.indiana.edu/.bin/fbidq.html?7B2), [Takr99D](http://flybase.bio.indiana.edu/.bin/fbidq.html?Takr99D), [sNPF-R](http://flybase.bio.indiana.edu/.bin/fbidq.html?sNPF-R), [GRHRII](http://flybase.bio.indiana.edu/.bin/fbidq.html?GRHRII), [Eh](http://flybase.bio.indiana.edu/.bin/fbidq.html?Eh), [stan](http://flybase.bio.indiana.edu/.bin/fbidq.html?stan), [GABA-B-R2](http://flybase.bio.indiana.edu/.bin/fbidq.html?GABA-B-R2), [mGluRA](http://flybase.bio.indiana.edu/.bin/fbidq.html?mGluRA), [Ast-C](http://flybase.bio.indiana.edu/.bin/fbidq.html?Ast-C), [Gbeta5](http://flybase.bio.indiana.edu/.bin/fbidq.html?Gbeta5), [mAcR-60C](http://flybase.bio.indiana.edu/.bin/fbidq.html?mAcR-60C), [CG31140](http://flybase.bio.indiana.edu/.bin/fbidq.html?CG31140), [Crz](http://flybase.bio.indiana.edu/.bin/fbidq.html?Crz), [AlstR](http://flybase.bio.indiana.edu/.bin/fbidq.html?AlstR), [Takr86C](http://flybase.bio.indiana.edu/.bin/fbidq.html?Takr86C), [hug](http://flybase.bio.indiana.edu/.bin/fbidq.html?hug), [Ggamma1](http://flybase.bio.indiana.edu/.bin/fbidq.html?Ggamma1), [DopR](http://flybase.bio.indiana.edu/.bin/fbidq.html?DopR), [5-HT1A](http://flybase.bio.indiana.edu/.bin/fbidq.html?5-HT1A), [RSG7](http://flybase.bio.indiana.edu/.bin/fbidq.html?RSG7), [Tbh](http://flybase.bio.indiana.edu/.bin/fbidq.html?Tbh), [mthl8](http://flybase.bio.indiana.edu/.bin/fbidq.html?mthl8), [Dh31](http://flybase.bio.indiana.edu/.bin/fbidq.html?Dh31), [Mip](http://flybase.bio.indiana.edu/.bin/fbidq.html?Mip), [Oamb](http://flybase.bio.indiana.edu/.bin/fbidq.html?Oamb), [Lkr](http://flybase.bio.indiana.edu/.bin/fbidq.html?Lkr), [Ccap](http://flybase.bio.indiana.edu/.bin/fbidq.html?Ccap), [5-HT1B](http://flybase.bio.indiana.edu/.bin/fbidq.html?5-HT1B), [CG5036](http://flybase.bio.indiana.edu/.bin/fbidq.html?CG5036), [CG34381](http://flybase.bio.indiana.edu/.bin/fbidq.html?CG34381), [CG7918](http://flybase.bio.indiana.edu/.bin/fbidq.html?CG7918), [CG8795](http://flybase.bio.indiana.edu/.bin/fbidq.html?CG8795),[DopR2](http://flybase.bio.indiana.edu/.bin/fbidq.html?DopR2), [CG31760](http://flybase.bio.indiana.edu/.bin/fbidq.html?CG31760), [Klp54D](http://flybase.bio.indiana.edu/.bin/fbidq.html?Klp54D), [CG13995](http://flybase.bio.indiana.edu/.bin/fbidq.html?CG13995), [5-HT7](http://flybase.bio.indiana.edu/.bin/fbidq.html?5-HT7), [Lgr3](http://flybase.bio.indiana.edu/.bin/fbidq.html?Lgr3), [CG13229](http://flybase.bio.indiana.edu/.bin/fbidq.html?CG13229), [Dh](http://flybase.bio.indiana.edu/.bin/fbidq.html?Dh), [CG17760](http://flybase.bio.indiana.edu/.bin/fbidq.html?CG17760), [Dms](http://flybase.bio.indiana.edu/.bin/fbidq.html?Dms), [ETH](http://flybase.bio.indiana.edu/.bin/fbidq.html?ETH), [SPR](http://flybase.bio.indiana.edu/.bin/fbidq.html?SPR), [NPFR1](http://flybase.bio.indiana.edu/.bin/fbidq.html?NPFR1), [Ggamma30A](http://flybase.bio.indiana.edu/.bin/fbidq.html?Ggamma30A), [pburs](http://flybase.bio.indiana.edu/.bin/fbidq.html?pburs) |
| [regulation of biological process](http://amigo.geneontology.org/cgi-bin/amigo/go.cgi?view=details&query=GO:0050789) | 569 of 1594 genes, 35.7% | 2101 of 7634 genes, 27.5% | 1.99e-18 | 0.00% | 0.00 | [Sh](http://flybase.bio.indiana.edu/.bin/fbidq.html?Sh), [dock](http://flybase.bio.indiana.edu/.bin/fbidq.html?dock), [tna](http://flybase.bio.indiana.edu/.bin/fbidq.html?tna), [CG8500](http://flybase.bio.indiana.edu/.bin/fbidq.html?CG8500), [mub](http://flybase.bio.indiana.edu/.bin/fbidq.html?mub), [rn](http://flybase.bio.indiana.edu/.bin/fbidq.html?rn), [ap](http://flybase.bio.indiana.edu/.bin/fbidq.html?ap), [cenG1A](http://flybase.bio.indiana.edu/.bin/fbidq.html?cenG1A), [tlk](http://flybase.bio.indiana.edu/.bin/fbidq.html?tlk), [malpha](http://flybase.bio.indiana.edu/.bin/fbidq.html?malpha), [os](http://flybase.bio.indiana.edu/.bin/fbidq.html?os), [Axn](http://flybase.bio.indiana.edu/.bin/fbidq.html?Axn), [mtrm](http://flybase.bio.indiana.edu/.bin/fbidq.html?mtrm), [Hcf](http://flybase.bio.indiana.edu/.bin/fbidq.html?Hcf), [Traf6](http://flybase.bio.indiana.edu/.bin/fbidq.html?Traf6), [Leucokinin](http://flybase.bio.indiana.edu/.bin/fbidq.html?Leucokinin), [NetB](http://flybase.bio.indiana.edu/.bin/fbidq.html?NetB), [rdgC](http://flybase.bio.indiana.edu/.bin/fbidq.html?rdgC), [Ssrp](http://flybase.bio.indiana.edu/.bin/fbidq.html?Ssrp), [Lar](http://flybase.bio.indiana.edu/.bin/fbidq.html?Lar), [scrib](http://flybase.bio.indiana.edu/.bin/fbidq.html?scrib), [HGTX](http://flybase.bio.indiana.edu/.bin/fbidq.html?HGTX), [not](http://flybase.bio.indiana.edu/.bin/fbidq.html?not), [nvy](http://flybase.bio.indiana.edu/.bin/fbidq.html?nvy), [CG7650](http://flybase.bio.indiana.edu/.bin/fbidq.html?CG7650), [nab](http://flybase.bio.indiana.edu/.bin/fbidq.html?nab), [Ddc](http://flybase.bio.indiana.edu/.bin/fbidq.html?Ddc), [U2af38](http://flybase.bio.indiana.edu/.bin/fbidq.html?U2af38), [HLHmdelta](http://flybase.bio.indiana.edu/.bin/fbidq.html?HLHmdelta), [ATbp](http://flybase.bio.indiana.edu/.bin/fbidq.html?ATbp), [7B2](http://flybase.bio.indiana.edu/.bin/fbidq.html?7B2), [alph](http://flybase.bio.indiana.edu/.bin/fbidq.html?alph), [Traf4](http://flybase.bio.indiana.edu/.bin/fbidq.html?Traf4), [TBPH](http://flybase.bio.indiana.edu/.bin/fbidq.html?TBPH), [Snap25](http://flybase.bio.indiana.edu/.bin/fbidq.html?Snap25), [Teh2](http://flybase.bio.indiana.edu/.bin/fbidq.html?Teh2), [Fer2](http://flybase.bio.indiana.edu/.bin/fbidq.html?Fer2),[CG34400](http://flybase.bio.indiana.edu/.bin/fbidq.html?CG34400), [mGluRA](http://flybase.bio.indiana.edu/.bin/fbidq.html?mGluRA), [ena](http://flybase.bio.indiana.edu/.bin/fbidq.html?ena), [dsh](http://flybase.bio.indiana.edu/.bin/fbidq.html?dsh), [Nf-YC](http://flybase.bio.indiana.edu/.bin/fbidq.html?Nf-YC), [Pli](http://flybase.bio.indiana.edu/.bin/fbidq.html?Pli), [Hel25E](http://flybase.bio.indiana.edu/.bin/fbidq.html?Hel25E), [comt](http://flybase.bio.indiana.edu/.bin/fbidq.html?comt), [CG31140](http://flybase.bio.indiana.edu/.bin/fbidq.html?CG31140), [PP2A-B](http://flybase.bio.indiana.edu/.bin/fbidq.html?PP2A-B), [qvr](http://flybase.bio.indiana.edu/.bin/fbidq.html?qvr), [jet](http://flybase.bio.indiana.edu/.bin/fbidq.html?jet), [DAAM](http://flybase.bio.indiana.edu/.bin/fbidq.html?DAAM), [rho-5](http://flybase.bio.indiana.edu/.bin/fbidq.html?rho-5), [nmo](http://flybase.bio.indiana.edu/.bin/fbidq.html?nmo), [Tusp](http://flybase.bio.indiana.edu/.bin/fbidq.html?Tusp), [5-HT1A](http://flybase.bio.indiana.edu/.bin/fbidq.html?5-HT1A), [Psi](http://flybase.bio.indiana.edu/.bin/fbidq.html?Psi), [Gug](http://flybase.bio.indiana.edu/.bin/fbidq.html?Gug), [ed](http://flybase.bio.indiana.edu/.bin/fbidq.html?ed), [pad](http://flybase.bio.indiana.edu/.bin/fbidq.html?pad), [daw](http://flybase.bio.indiana.edu/.bin/fbidq.html?daw), [CG5036](http://flybase.bio.indiana.edu/.bin/fbidq.html?CG5036), [5-HT1B](http://flybase.bio.indiana.edu/.bin/fbidq.html?5-HT1B), [Rx](http://flybase.bio.indiana.edu/.bin/fbidq.html?Rx), [MED1](http://flybase.bio.indiana.edu/.bin/fbidq.html?MED1), [salr](http://flybase.bio.indiana.edu/.bin/fbidq.html?salr), [Apc](http://flybase.bio.indiana.edu/.bin/fbidq.html?Apc), [CG8795](http://flybase.bio.indiana.edu/.bin/fbidq.html?CG8795), [d4](http://flybase.bio.indiana.edu/.bin/fbidq.html?d4), [dan](http://flybase.bio.indiana.edu/.bin/fbidq.html?dan), [cort](http://flybase.bio.indiana.edu/.bin/fbidq.html?cort), [Jarid2](http://flybase.bio.indiana.edu/.bin/fbidq.html?Jarid2), [RN-tre](http://flybase.bio.indiana.edu/.bin/fbidq.html?RN-tre), [trx](http://flybase.bio.indiana.edu/.bin/fbidq.html?trx), [Btk29A](http://flybase.bio.indiana.edu/.bin/fbidq.html?Btk29A), [MED25](http://flybase.bio.indiana.edu/.bin/fbidq.html?MED25),[ETH](http://flybase.bio.indiana.edu/.bin/fbidq.html?ETH), [unc-5](http://flybase.bio.indiana.edu/.bin/fbidq.html?unc-5), [cdi](http://flybase.bio.indiana.edu/.bin/fbidq.html?cdi), [vfl](http://flybase.bio.indiana.edu/.bin/fbidq.html?vfl), [NPFR1](http://flybase.bio.indiana.edu/.bin/fbidq.html?NPFR1), [pygo](http://flybase.bio.indiana.edu/.bin/fbidq.html?pygo), [z](http://flybase.bio.indiana.edu/.bin/fbidq.html?z), [HLHmgamma](http://flybase.bio.indiana.edu/.bin/fbidq.html?HLHmgamma), [CG15556](http://flybase.bio.indiana.edu/.bin/fbidq.html?CG15556), [jar](http://flybase.bio.indiana.edu/.bin/fbidq.html?jar), [Ror](http://flybase.bio.indiana.edu/.bin/fbidq.html?Ror), [wor](http://flybase.bio.indiana.edu/.bin/fbidq.html?wor), [Atg1](http://flybase.bio.indiana.edu/.bin/fbidq.html?Atg1), [pUf68](http://flybase.bio.indiana.edu/.bin/fbidq.html?pUf68), [seq](http://flybase.bio.indiana.edu/.bin/fbidq.html?seq), [HDAC4](http://flybase.bio.indiana.edu/.bin/fbidq.html?HDAC4), [Cbl](http://flybase.bio.indiana.edu/.bin/fbidq.html?Cbl), [thr](http://flybase.bio.indiana.edu/.bin/fbidq.html?thr), [pog](http://flybase.bio.indiana.edu/.bin/fbidq.html?pog), [Pdp1](http://flybase.bio.indiana.edu/.bin/fbidq.html?Pdp1), [CASK](http://flybase.bio.indiana.edu/.bin/fbidq.html?CASK), [CG32758](http://flybase.bio.indiana.edu/.bin/fbidq.html?CG32758), [mtt](http://flybase.bio.indiana.edu/.bin/fbidq.html?mtt), [disp](http://flybase.bio.indiana.edu/.bin/fbidq.html?disp), [mip120](http://flybase.bio.indiana.edu/.bin/fbidq.html?mip120), [cpx](http://flybase.bio.indiana.edu/.bin/fbidq.html?cpx), [CG4022](http://flybase.bio.indiana.edu/.bin/fbidq.html?CG4022), [jumu](http://flybase.bio.indiana.edu/.bin/fbidq.html?jumu), [Su(var)2-HP2](http://flybase.bio.indiana.edu/.bin/fbidq.html?Su(var)2-HP2), [Usp7](http://flybase.bio.indiana.edu/.bin/fbidq.html?Usp7), [fd59A](http://flybase.bio.indiana.edu/.bin/fbidq.html?fd59A), [brat](http://flybase.bio.indiana.edu/.bin/fbidq.html?brat), [FBX011](http://flybase.bio.indiana.edu/.bin/fbidq.html?FBX011), [CG17343](http://flybase.bio.indiana.edu/.bin/fbidq.html?CG17343),[GABA-B-R2](http://flybase.bio.indiana.edu/.bin/fbidq.html?GABA-B-R2), [futsch](http://flybase.bio.indiana.edu/.bin/fbidq.html?futsch), [Ubx](http://flybase.bio.indiana.edu/.bin/fbidq.html?Ubx), [Jupiter](http://flybase.bio.indiana.edu/.bin/fbidq.html?Jupiter), [chm](http://flybase.bio.indiana.edu/.bin/fbidq.html?chm), [Rim](http://flybase.bio.indiana.edu/.bin/fbidq.html?Rim), [msl-1](http://flybase.bio.indiana.edu/.bin/fbidq.html?msl-1), [corn](http://flybase.bio.indiana.edu/.bin/fbidq.html?corn), [Sur-8](http://flybase.bio.indiana.edu/.bin/fbidq.html?Sur-8), [CG42574](http://flybase.bio.indiana.edu/.bin/fbidq.html?CG42574), [Rgl](http://flybase.bio.indiana.edu/.bin/fbidq.html?Rgl), [dac](http://flybase.bio.indiana.edu/.bin/fbidq.html?dac), [CG9007](http://flybase.bio.indiana.edu/.bin/fbidq.html?CG9007), [mav](http://flybase.bio.indiana.edu/.bin/fbidq.html?mav), [gol](http://flybase.bio.indiana.edu/.bin/fbidq.html?gol), [Ilp7](http://flybase.bio.indiana.edu/.bin/fbidq.html?Ilp7), [CG33275](http://flybase.bio.indiana.edu/.bin/fbidq.html?CG33275), [toy](http://flybase.bio.indiana.edu/.bin/fbidq.html?toy), [CG40351](http://flybase.bio.indiana.edu/.bin/fbidq.html?CG40351), [Lim3](http://flybase.bio.indiana.edu/.bin/fbidq.html?Lim3), [sd](http://flybase.bio.indiana.edu/.bin/fbidq.html?sd), [Aplip1](http://flybase.bio.indiana.edu/.bin/fbidq.html?Aplip1), [fs(1)h](http://flybase.bio.indiana.edu/.bin/fbidq.html?fs(1)h), [CG11347](http://flybase.bio.indiana.edu/.bin/fbidq.html?CG11347), [Ggamma30A](http://flybase.bio.indiana.edu/.bin/fbidq.html?Ggamma30A), [CG11294](http://flybase.bio.indiana.edu/.bin/fbidq.html?CG11294), [wls](http://flybase.bio.indiana.edu/.bin/fbidq.html?wls), [skl](http://flybase.bio.indiana.edu/.bin/fbidq.html?skl), [hiw](http://flybase.bio.indiana.edu/.bin/fbidq.html?hiw), [CG32653](http://flybase.bio.indiana.edu/.bin/fbidq.html?CG32653), [Atf-2](http://flybase.bio.indiana.edu/.bin/fbidq.html?Atf-2), [skd](http://flybase.bio.indiana.edu/.bin/fbidq.html?skd), [jing](http://flybase.bio.indiana.edu/.bin/fbidq.html?jing),[katanin-60](http://flybase.bio.indiana.edu/.bin/fbidq.html?katanin-60), [Camta](http://flybase.bio.indiana.edu/.bin/fbidq.html?Camta), [tll](http://flybase.bio.indiana.edu/.bin/fbidq.html?tll), [ato](http://flybase.bio.indiana.edu/.bin/fbidq.html?ato), [Fmrf](http://flybase.bio.indiana.edu/.bin/fbidq.html?Fmrf), [Iswi](http://flybase.bio.indiana.edu/.bin/fbidq.html?Iswi), [Spt6](http://flybase.bio.indiana.edu/.bin/fbidq.html?Spt6), [obst-A](http://flybase.bio.indiana.edu/.bin/fbidq.html?obst-A), [Pka-R1](http://flybase.bio.indiana.edu/.bin/fbidq.html?Pka-R1), [mago](http://flybase.bio.indiana.edu/.bin/fbidq.html?mago), [enc](http://flybase.bio.indiana.edu/.bin/fbidq.html?enc), [CG42533](http://flybase.bio.indiana.edu/.bin/fbidq.html?CG42533), [ksr](http://flybase.bio.indiana.edu/.bin/fbidq.html?ksr), [CG3618](http://flybase.bio.indiana.edu/.bin/fbidq.html?CG3618), [Gyc-89Da](http://flybase.bio.indiana.edu/.bin/fbidq.html?Gyc-89Da), [brp](http://flybase.bio.indiana.edu/.bin/fbidq.html?brp), [chn](http://flybase.bio.indiana.edu/.bin/fbidq.html?chn), [CG17124](http://flybase.bio.indiana.edu/.bin/fbidq.html?CG17124), [chif](http://flybase.bio.indiana.edu/.bin/fbidq.html?chif), [D2R](http://flybase.bio.indiana.edu/.bin/fbidq.html?D2R), [ewg](http://flybase.bio.indiana.edu/.bin/fbidq.html?ewg), [PHDP](http://flybase.bio.indiana.edu/.bin/fbidq.html?PHDP), [Cirl](http://flybase.bio.indiana.edu/.bin/fbidq.html?Cirl), [APC4](http://flybase.bio.indiana.edu/.bin/fbidq.html?APC4), [l(2)NC136](http://flybase.bio.indiana.edu/.bin/fbidq.html?l(2)NC136), [Su(var)3-3](http://flybase.bio.indiana.edu/.bin/fbidq.html?Su(var)3-3), [Bili](http://flybase.bio.indiana.edu/.bin/fbidq.html?Bili), [Dh44-R1](http://flybase.bio.indiana.edu/.bin/fbidq.html?Dh44-R1), [stet](http://flybase.bio.indiana.edu/.bin/fbidq.html?stet), [Sema-1a](http://flybase.bio.indiana.edu/.bin/fbidq.html?Sema-1a), [Eh](http://flybase.bio.indiana.edu/.bin/fbidq.html?Eh), [Hs3st-B](http://flybase.bio.indiana.edu/.bin/fbidq.html?Hs3st-B), [Sos](http://flybase.bio.indiana.edu/.bin/fbidq.html?Sos),[Fas2](http://flybase.bio.indiana.edu/.bin/fbidq.html?Fas2), [Klp61F](http://flybase.bio.indiana.edu/.bin/fbidq.html?Klp61F), [eya](http://flybase.bio.indiana.edu/.bin/fbidq.html?eya), [mAcR-60C](http://flybase.bio.indiana.edu/.bin/fbidq.html?mAcR-60C), [CG8557](http://flybase.bio.indiana.edu/.bin/fbidq.html?CG8557), [Vsx2](http://flybase.bio.indiana.edu/.bin/fbidq.html?Vsx2), [kat-60L1](http://flybase.bio.indiana.edu/.bin/fbidq.html?kat-60L1), [mtg](http://flybase.bio.indiana.edu/.bin/fbidq.html?mtg), [ft](http://flybase.bio.indiana.edu/.bin/fbidq.html?ft), [Crz](http://flybase.bio.indiana.edu/.bin/fbidq.html?Crz), [l(2)k16918](http://flybase.bio.indiana.edu/.bin/fbidq.html?l(2)k16918), [Takr86C](http://flybase.bio.indiana.edu/.bin/fbidq.html?Takr86C), [Dll](http://flybase.bio.indiana.edu/.bin/fbidq.html?Dll), [disco](http://flybase.bio.indiana.edu/.bin/fbidq.html?disco), [DopR](http://flybase.bio.indiana.edu/.bin/fbidq.html?DopR), [D12](http://flybase.bio.indiana.edu/.bin/fbidq.html?D12), [didum](http://flybase.bio.indiana.edu/.bin/fbidq.html?didum), [Lis-1](http://flybase.bio.indiana.edu/.bin/fbidq.html?Lis-1), [bmm](http://flybase.bio.indiana.edu/.bin/fbidq.html?bmm), [ems](http://flybase.bio.indiana.edu/.bin/fbidq.html?ems), [CG16896](http://flybase.bio.indiana.edu/.bin/fbidq.html?CG16896), [eys](http://flybase.bio.indiana.edu/.bin/fbidq.html?eys), [tup](http://flybase.bio.indiana.edu/.bin/fbidq.html?tup), [Rab3](http://flybase.bio.indiana.edu/.bin/fbidq.html?Rab3), [CG6191](http://flybase.bio.indiana.edu/.bin/fbidq.html?CG6191), [trio](http://flybase.bio.indiana.edu/.bin/fbidq.html?trio), [pdm2](http://flybase.bio.indiana.edu/.bin/fbidq.html?pdm2), [RabX4](http://flybase.bio.indiana.edu/.bin/fbidq.html?RabX4), [rgr](http://flybase.bio.indiana.edu/.bin/fbidq.html?rgr), [Lkr](http://flybase.bio.indiana.edu/.bin/fbidq.html?Lkr), [spel1](http://flybase.bio.indiana.edu/.bin/fbidq.html?spel1), [CG31665](http://flybase.bio.indiana.edu/.bin/fbidq.html?CG31665), [CG7918](http://flybase.bio.indiana.edu/.bin/fbidq.html?CG7918), [CaMKII](http://flybase.bio.indiana.edu/.bin/fbidq.html?CaMKII),[DopR2](http://flybase.bio.indiana.edu/.bin/fbidq.html?DopR2), [l(3)psg2](http://flybase.bio.indiana.edu/.bin/fbidq.html?l(3)psg2), [nerfin-1](http://flybase.bio.indiana.edu/.bin/fbidq.html?nerfin-1), [pigs](http://flybase.bio.indiana.edu/.bin/fbidq.html?pigs), [5-HT7](http://flybase.bio.indiana.edu/.bin/fbidq.html?5-HT7), [Lgr3](http://flybase.bio.indiana.edu/.bin/fbidq.html?Lgr3), [polo](http://flybase.bio.indiana.edu/.bin/fbidq.html?polo), [Syn1](http://flybase.bio.indiana.edu/.bin/fbidq.html?Syn1), [Ac13E](http://flybase.bio.indiana.edu/.bin/fbidq.html?Ac13E), [Ssdp](http://flybase.bio.indiana.edu/.bin/fbidq.html?Ssdp), [CG8155](http://flybase.bio.indiana.edu/.bin/fbidq.html?CG8155), [CG32105](http://flybase.bio.indiana.edu/.bin/fbidq.html?CG32105), [Fer3](http://flybase.bio.indiana.edu/.bin/fbidq.html?Fer3), [dalao](http://flybase.bio.indiana.edu/.bin/fbidq.html?dalao), [CG1868](http://flybase.bio.indiana.edu/.bin/fbidq.html?CG1868), [sr](http://flybase.bio.indiana.edu/.bin/fbidq.html?sr), [Lim1](http://flybase.bio.indiana.edu/.bin/fbidq.html?Lim1), [nkd](http://flybase.bio.indiana.edu/.bin/fbidq.html?nkd), [RhoGEF3](http://flybase.bio.indiana.edu/.bin/fbidq.html?RhoGEF3), [danr](http://flybase.bio.indiana.edu/.bin/fbidq.html?danr), [Dsk](http://flybase.bio.indiana.edu/.bin/fbidq.html?Dsk), [RhoGEF4](http://flybase.bio.indiana.edu/.bin/fbidq.html?RhoGEF4), [vvl](http://flybase.bio.indiana.edu/.bin/fbidq.html?vvl), [Brd8](http://flybase.bio.indiana.edu/.bin/fbidq.html?Brd8), [betaInt-nu](http://flybase.bio.indiana.edu/.bin/fbidq.html?betaInt-nu), [sgg](http://flybase.bio.indiana.edu/.bin/fbidq.html?sgg), [Brd](http://flybase.bio.indiana.edu/.bin/fbidq.html?Brd), [Eip93F](http://flybase.bio.indiana.edu/.bin/fbidq.html?Eip93F), [Nmdar1](http://flybase.bio.indiana.edu/.bin/fbidq.html?Nmdar1), [gcm](http://flybase.bio.indiana.edu/.bin/fbidq.html?gcm), [Suv4-20](http://flybase.bio.indiana.edu/.bin/fbidq.html?Suv4-20), [Hsp67Bc](http://flybase.bio.indiana.edu/.bin/fbidq.html?Hsp67Bc),[ase](http://flybase.bio.indiana.edu/.bin/fbidq.html?ase), [scro](http://flybase.bio.indiana.edu/.bin/fbidq.html?scro), [elav](http://flybase.bio.indiana.edu/.bin/fbidq.html?elav), [MESK2](http://flybase.bio.indiana.edu/.bin/fbidq.html?MESK2), [CG6227](http://flybase.bio.indiana.edu/.bin/fbidq.html?CG6227), [Poxn](http://flybase.bio.indiana.edu/.bin/fbidq.html?Poxn), [sals](http://flybase.bio.indiana.edu/.bin/fbidq.html?sals), [Dat](http://flybase.bio.indiana.edu/.bin/fbidq.html?Dat), [CG15376](http://flybase.bio.indiana.edu/.bin/fbidq.html?CG15376), [fred](http://flybase.bio.indiana.edu/.bin/fbidq.html?fred), [ush](http://flybase.bio.indiana.edu/.bin/fbidq.html?ush), [Fak56D](http://flybase.bio.indiana.edu/.bin/fbidq.html?Fak56D), [Takr99D](http://flybase.bio.indiana.edu/.bin/fbidq.html?Takr99D), [Teh4](http://flybase.bio.indiana.edu/.bin/fbidq.html?Teh4), [Alh](http://flybase.bio.indiana.edu/.bin/fbidq.html?Alh), [Sox102F](http://flybase.bio.indiana.edu/.bin/fbidq.html?Sox102F), [egg](http://flybase.bio.indiana.edu/.bin/fbidq.html?egg), [CG13692](http://flybase.bio.indiana.edu/.bin/fbidq.html?CG13692), [CG5337](http://flybase.bio.indiana.edu/.bin/fbidq.html?CG5337), [CG12424](http://flybase.bio.indiana.edu/.bin/fbidq.html?CG12424), [Sp1](http://flybase.bio.indiana.edu/.bin/fbidq.html?Sp1), [Wnt5](http://flybase.bio.indiana.edu/.bin/fbidq.html?Wnt5), [fus](http://flybase.bio.indiana.edu/.bin/fbidq.html?fus), [sif](http://flybase.bio.indiana.edu/.bin/fbidq.html?sif), [Tbh](http://flybase.bio.indiana.edu/.bin/fbidq.html?Tbh), [Aac11](http://flybase.bio.indiana.edu/.bin/fbidq.html?Aac11), [MTA1-like](http://flybase.bio.indiana.edu/.bin/fbidq.html?MTA1-like), [ssh](http://flybase.bio.indiana.edu/.bin/fbidq.html?ssh), [CG42541](http://flybase.bio.indiana.edu/.bin/fbidq.html?CG42541), [PIP5K59B](http://flybase.bio.indiana.edu/.bin/fbidq.html?PIP5K59B), [mle](http://flybase.bio.indiana.edu/.bin/fbidq.html?mle), [Hr51](http://flybase.bio.indiana.edu/.bin/fbidq.html?Hr51), [CG11155](http://flybase.bio.indiana.edu/.bin/fbidq.html?CG11155),[CG8786](http://flybase.bio.indiana.edu/.bin/fbidq.html?CG8786), [wg](http://flybase.bio.indiana.edu/.bin/fbidq.html?wg), [fkh](http://flybase.bio.indiana.edu/.bin/fbidq.html?fkh), [Spps](http://flybase.bio.indiana.edu/.bin/fbidq.html?Spps), [Rhp](http://flybase.bio.indiana.edu/.bin/fbidq.html?Rhp), [fne](http://flybase.bio.indiana.edu/.bin/fbidq.html?fne), [en](http://flybase.bio.indiana.edu/.bin/fbidq.html?en), [sfl](http://flybase.bio.indiana.edu/.bin/fbidq.html?sfl), [CG13229](http://flybase.bio.indiana.edu/.bin/fbidq.html?CG13229), [Pde11](http://flybase.bio.indiana.edu/.bin/fbidq.html?Pde11), [Utx](http://flybase.bio.indiana.edu/.bin/fbidq.html?Utx), [Ct](http://flybase.bio.indiana.edu/.bin/fbidq.html?Ct), [fz2](http://flybase.bio.indiana.edu/.bin/fbidq.html?fz2), [numb](http://flybase.bio.indiana.edu/.bin/fbidq.html?numb), [Ocho](http://flybase.bio.indiana.edu/.bin/fbidq.html?Ocho), [CG14375](http://flybase.bio.indiana.edu/.bin/fbidq.html?CG14375), [Ets65A](http://flybase.bio.indiana.edu/.bin/fbidq.html?Ets65A), [Sxl](http://flybase.bio.indiana.edu/.bin/fbidq.html?Sxl), [stj](http://flybase.bio.indiana.edu/.bin/fbidq.html?stj), [JIL-1](http://flybase.bio.indiana.edu/.bin/fbidq.html?JIL-1), [Nf1](http://flybase.bio.indiana.edu/.bin/fbidq.html?Nf1), [AR-2](http://flybase.bio.indiana.edu/.bin/fbidq.html?AR-2), [jigr1](http://flybase.bio.indiana.edu/.bin/fbidq.html?jigr1), [bi](http://flybase.bio.indiana.edu/.bin/fbidq.html?bi), [tok](http://flybase.bio.indiana.edu/.bin/fbidq.html?tok), [NC2alpha](http://flybase.bio.indiana.edu/.bin/fbidq.html?NC2alpha), [blue](http://flybase.bio.indiana.edu/.bin/fbidq.html?blue), [mod(mdg4)](http://flybase.bio.indiana.edu/.bin/fbidq.html?mod(mdg4)), [bab1](http://flybase.bio.indiana.edu/.bin/fbidq.html?bab1), [Galpha49B](http://flybase.bio.indiana.edu/.bin/fbidq.html?Galpha49B), [Snoo](http://flybase.bio.indiana.edu/.bin/fbidq.html?Snoo), [rut](http://flybase.bio.indiana.edu/.bin/fbidq.html?rut), [RhoGAP100F](http://flybase.bio.indiana.edu/.bin/fbidq.html?RhoGAP100F), [uif](http://flybase.bio.indiana.edu/.bin/fbidq.html?uif), [nub](http://flybase.bio.indiana.edu/.bin/fbidq.html?nub), [btd](http://flybase.bio.indiana.edu/.bin/fbidq.html?btd), [Mmp2](http://flybase.bio.indiana.edu/.bin/fbidq.html?Mmp2),[fd68A](http://flybase.bio.indiana.edu/.bin/fbidq.html?fd68A), [Nplp1](http://flybase.bio.indiana.edu/.bin/fbidq.html?Nplp1), [B-H1](http://flybase.bio.indiana.edu/.bin/fbidq.html?B-H1), [CG32206](http://flybase.bio.indiana.edu/.bin/fbidq.html?CG32206), [Nipped-A](http://flybase.bio.indiana.edu/.bin/fbidq.html?Nipped-A), [fd102C](http://flybase.bio.indiana.edu/.bin/fbidq.html?fd102C), [Spf45](http://flybase.bio.indiana.edu/.bin/fbidq.html?Spf45), [hth](http://flybase.bio.indiana.edu/.bin/fbidq.html?hth), [14-3-3zeta](http://flybase.bio.indiana.edu/.bin/fbidq.html?14-3-3zeta), [pzg](http://flybase.bio.indiana.edu/.bin/fbidq.html?pzg), [lea](http://flybase.bio.indiana.edu/.bin/fbidq.html?lea), [shakB](http://flybase.bio.indiana.edu/.bin/fbidq.html?shakB), [Hey](http://flybase.bio.indiana.edu/.bin/fbidq.html?Hey), [plexB](http://flybase.bio.indiana.edu/.bin/fbidq.html?plexB), [AlstR](http://flybase.bio.indiana.edu/.bin/fbidq.html?AlstR), [loqs](http://flybase.bio.indiana.edu/.bin/fbidq.html?loqs), [mars](http://flybase.bio.indiana.edu/.bin/fbidq.html?mars), [IM10](http://flybase.bio.indiana.edu/.bin/fbidq.html?IM10), [neur](http://flybase.bio.indiana.edu/.bin/fbidq.html?neur), [Brf](http://flybase.bio.indiana.edu/.bin/fbidq.html?Brf), [CycE](http://flybase.bio.indiana.edu/.bin/fbidq.html?CycE), [CG32149](http://flybase.bio.indiana.edu/.bin/fbidq.html?CG32149), [Vdup1](http://flybase.bio.indiana.edu/.bin/fbidq.html?Vdup1), [mr](http://flybase.bio.indiana.edu/.bin/fbidq.html?mr), [orb2](http://flybase.bio.indiana.edu/.bin/fbidq.html?orb2), [wdn](http://flybase.bio.indiana.edu/.bin/fbidq.html?wdn), [synaptogyrin](http://flybase.bio.indiana.edu/.bin/fbidq.html?synaptogyrin), [sim](http://flybase.bio.indiana.edu/.bin/fbidq.html?sim), [gro](http://flybase.bio.indiana.edu/.bin/fbidq.html?gro), [Rya-r44F](http://flybase.bio.indiana.edu/.bin/fbidq.html?Rya-r44F), [scyl](http://flybase.bio.indiana.edu/.bin/fbidq.html?scyl), [hkl](http://flybase.bio.indiana.edu/.bin/fbidq.html?hkl), [bin3](http://flybase.bio.indiana.edu/.bin/fbidq.html?bin3), [Rpd3](http://flybase.bio.indiana.edu/.bin/fbidq.html?Rpd3), [tyf](http://flybase.bio.indiana.edu/.bin/fbidq.html?tyf),[CG10107](http://flybase.bio.indiana.edu/.bin/fbidq.html?CG10107), [aret](http://flybase.bio.indiana.edu/.bin/fbidq.html?aret), [HLHm7](http://flybase.bio.indiana.edu/.bin/fbidq.html?HLHm7), [Sox21b](http://flybase.bio.indiana.edu/.bin/fbidq.html?Sox21b), [tow](http://flybase.bio.indiana.edu/.bin/fbidq.html?tow), [CG15609](http://flybase.bio.indiana.edu/.bin/fbidq.html?CG15609), [CG12187](http://flybase.bio.indiana.edu/.bin/fbidq.html?CG12187), [Pten](http://flybase.bio.indiana.edu/.bin/fbidq.html?Pten), [unc-13](http://flybase.bio.indiana.edu/.bin/fbidq.html?unc-13), [wda](http://flybase.bio.indiana.edu/.bin/fbidq.html?wda), [npf](http://flybase.bio.indiana.edu/.bin/fbidq.html?npf), [mira](http://flybase.bio.indiana.edu/.bin/fbidq.html?mira), [MED14](http://flybase.bio.indiana.edu/.bin/fbidq.html?MED14), [Spindly](http://flybase.bio.indiana.edu/.bin/fbidq.html?Spindly), [Drep-2](http://flybase.bio.indiana.edu/.bin/fbidq.html?Drep-2), [Dip3](http://flybase.bio.indiana.edu/.bin/fbidq.html?Dip3), [oa2](http://flybase.bio.indiana.edu/.bin/fbidq.html?oa2), [elk](http://flybase.bio.indiana.edu/.bin/fbidq.html?elk), [Rab26](http://flybase.bio.indiana.edu/.bin/fbidq.html?Rab26), [phl](http://flybase.bio.indiana.edu/.bin/fbidq.html?phl), [sNPF](http://flybase.bio.indiana.edu/.bin/fbidq.html?sNPF), [Pde6](http://flybase.bio.indiana.edu/.bin/fbidq.html?Pde6), [plx](http://flybase.bio.indiana.edu/.bin/fbidq.html?plx), [CG9098](http://flybase.bio.indiana.edu/.bin/fbidq.html?CG9098), [CG8108](http://flybase.bio.indiana.edu/.bin/fbidq.html?CG8108), [IFa](http://flybase.bio.indiana.edu/.bin/fbidq.html?IFa), [Alk](http://flybase.bio.indiana.edu/.bin/fbidq.html?Alk), [Vang](http://flybase.bio.indiana.edu/.bin/fbidq.html?Vang), [Pde1c](http://flybase.bio.indiana.edu/.bin/fbidq.html?Pde1c), [Sin3A](http://flybase.bio.indiana.edu/.bin/fbidq.html?Sin3A), [casp](http://flybase.bio.indiana.edu/.bin/fbidq.html?casp), [Smox](http://flybase.bio.indiana.edu/.bin/fbidq.html?Smox), [Plc21C](http://flybase.bio.indiana.edu/.bin/fbidq.html?Plc21C), [Antp](http://flybase.bio.indiana.edu/.bin/fbidq.html?Antp), [Rdl](http://flybase.bio.indiana.edu/.bin/fbidq.html?Rdl),[GRHRII](http://flybase.bio.indiana.edu/.bin/fbidq.html?GRHRII), [l(3)neo38](http://flybase.bio.indiana.edu/.bin/fbidq.html?l(3)neo38), [CG10362](http://flybase.bio.indiana.edu/.bin/fbidq.html?CG10362), [Ast-C](http://flybase.bio.indiana.edu/.bin/fbidq.html?Ast-C), [cindr](http://flybase.bio.indiana.edu/.bin/fbidq.html?cindr), [Gbeta5](http://flybase.bio.indiana.edu/.bin/fbidq.html?Gbeta5), [Ptx1](http://flybase.bio.indiana.edu/.bin/fbidq.html?Ptx1), [hug](http://flybase.bio.indiana.edu/.bin/fbidq.html?hug), [hbn](http://flybase.bio.indiana.edu/.bin/fbidq.html?hbn), [Syn2](http://flybase.bio.indiana.edu/.bin/fbidq.html?Syn2), [tsh](http://flybase.bio.indiana.edu/.bin/fbidq.html?tsh), [e(y)3](http://flybase.bio.indiana.edu/.bin/fbidq.html?e(y)3), [CRMP](http://flybase.bio.indiana.edu/.bin/fbidq.html?CRMP), [Cdk5](http://flybase.bio.indiana.edu/.bin/fbidq.html?Cdk5), [oc](http://flybase.bio.indiana.edu/.bin/fbidq.html?oc), [Cep135](http://flybase.bio.indiana.edu/.bin/fbidq.html?Cep135), [spt4](http://flybase.bio.indiana.edu/.bin/fbidq.html?spt4), [CG4641](http://flybase.bio.indiana.edu/.bin/fbidq.html?CG4641), [ey](http://flybase.bio.indiana.edu/.bin/fbidq.html?ey), [baz](http://flybase.bio.indiana.edu/.bin/fbidq.html?baz), [Mip](http://flybase.bio.indiana.edu/.bin/fbidq.html?Mip), [Oamb](http://flybase.bio.indiana.edu/.bin/fbidq.html?Oamb), [rst](http://flybase.bio.indiana.edu/.bin/fbidq.html?rst), [CG34381](http://flybase.bio.indiana.edu/.bin/fbidq.html?CG34381), [l(1)sc](http://flybase.bio.indiana.edu/.bin/fbidq.html?l(1)sc), [Bgb](http://flybase.bio.indiana.edu/.bin/fbidq.html?Bgb), [comm](http://flybase.bio.indiana.edu/.bin/fbidq.html?comm), [grh](http://flybase.bio.indiana.edu/.bin/fbidq.html?grh), [Cdk5alpha](http://flybase.bio.indiana.edu/.bin/fbidq.html?Cdk5alpha), [Vsx1](http://flybase.bio.indiana.edu/.bin/fbidq.html?Vsx1), [CG34362](http://flybase.bio.indiana.edu/.bin/fbidq.html?CG34362), [crol](http://flybase.bio.indiana.edu/.bin/fbidq.html?crol), [retn](http://flybase.bio.indiana.edu/.bin/fbidq.html?retn), [Ac3](http://flybase.bio.indiana.edu/.bin/fbidq.html?Ac3), [Syt1](http://flybase.bio.indiana.edu/.bin/fbidq.html?Syt1), [rl](http://flybase.bio.indiana.edu/.bin/fbidq.html?rl),[CG17760](http://flybase.bio.indiana.edu/.bin/fbidq.html?CG17760), [CG32683](http://flybase.bio.indiana.edu/.bin/fbidq.html?CG32683), [Nup153](http://flybase.bio.indiana.edu/.bin/fbidq.html?Nup153), [Saf-B](http://flybase.bio.indiana.edu/.bin/fbidq.html?Saf-B), [tey](http://flybase.bio.indiana.edu/.bin/fbidq.html?tey), [CG33960](http://flybase.bio.indiana.edu/.bin/fbidq.html?CG33960), [dnr1](http://flybase.bio.indiana.edu/.bin/fbidq.html?dnr1), [acj6](http://flybase.bio.indiana.edu/.bin/fbidq.html?acj6), [caup](http://flybase.bio.indiana.edu/.bin/fbidq.html?caup), [Cbp80](http://flybase.bio.indiana.edu/.bin/fbidq.html?Cbp80), [vimar](http://flybase.bio.indiana.edu/.bin/fbidq.html?vimar), [Rox8](http://flybase.bio.indiana.edu/.bin/fbidq.html?Rox8), [Dg](http://flybase.bio.indiana.edu/.bin/fbidq.html?Dg), [Trim9](http://flybase.bio.indiana.edu/.bin/fbidq.html?Trim9), [GABA-B-R3](http://flybase.bio.indiana.edu/.bin/fbidq.html?GABA-B-R3), [Evi5](http://flybase.bio.indiana.edu/.bin/fbidq.html?Evi5), [Gycalpha99B](http://flybase.bio.indiana.edu/.bin/fbidq.html?Gycalpha99B), [br](http://flybase.bio.indiana.edu/.bin/fbidq.html?br), [neo](http://flybase.bio.indiana.edu/.bin/fbidq.html?neo), [CG11376](http://flybase.bio.indiana.edu/.bin/fbidq.html?CG11376), [PNUTS](http://flybase.bio.indiana.edu/.bin/fbidq.html?PNUTS), [Gsc](http://flybase.bio.indiana.edu/.bin/fbidq.html?Gsc), [lola](http://flybase.bio.indiana.edu/.bin/fbidq.html?lola), [HLH4C](http://flybase.bio.indiana.edu/.bin/fbidq.html?HLH4C), [Pcl](http://flybase.bio.indiana.edu/.bin/fbidq.html?Pcl), [simj](http://flybase.bio.indiana.edu/.bin/fbidq.html?simj), [CG31158](http://flybase.bio.indiana.edu/.bin/fbidq.html?CG31158), [tap](http://flybase.bio.indiana.edu/.bin/fbidq.html?tap), [Appl](http://flybase.bio.indiana.edu/.bin/fbidq.html?Appl), [Awh](http://flybase.bio.indiana.edu/.bin/fbidq.html?Awh), [metro](http://flybase.bio.indiana.edu/.bin/fbidq.html?metro), [Nrk](http://flybase.bio.indiana.edu/.bin/fbidq.html?Nrk), [erm](http://flybase.bio.indiana.edu/.bin/fbidq.html?erm),[CG4328](http://flybase.bio.indiana.edu/.bin/fbidq.html?CG4328), [Taf4](http://flybase.bio.indiana.edu/.bin/fbidq.html?Taf4), [Ilp3](http://flybase.bio.indiana.edu/.bin/fbidq.html?Ilp3), [Nrg](http://flybase.bio.indiana.edu/.bin/fbidq.html?Nrg), [CG34384](http://flybase.bio.indiana.edu/.bin/fbidq.html?CG34384), [insc](http://flybase.bio.indiana.edu/.bin/fbidq.html?insc), [CG32447](http://flybase.bio.indiana.edu/.bin/fbidq.html?CG32447), [stau](http://flybase.bio.indiana.edu/.bin/fbidq.html?stau), [otk](http://flybase.bio.indiana.edu/.bin/fbidq.html?otk), [phyl](http://flybase.bio.indiana.edu/.bin/fbidq.html?phyl), [vg](http://flybase.bio.indiana.edu/.bin/fbidq.html?vg), [msl-2](http://flybase.bio.indiana.edu/.bin/fbidq.html?msl-2), [brm](http://flybase.bio.indiana.edu/.bin/fbidq.html?brm), [bsk](http://flybase.bio.indiana.edu/.bin/fbidq.html?bsk), [robo](http://flybase.bio.indiana.edu/.bin/fbidq.html?robo), [fz](http://flybase.bio.indiana.edu/.bin/fbidq.html?fz), [sno](http://flybase.bio.indiana.edu/.bin/fbidq.html?sno), [CG13830](http://flybase.bio.indiana.edu/.bin/fbidq.html?CG13830), [RhoGAPp190](http://flybase.bio.indiana.edu/.bin/fbidq.html?RhoGAPp190), [ara](http://flybase.bio.indiana.edu/.bin/fbidq.html?ara), [Sfmbt](http://flybase.bio.indiana.edu/.bin/fbidq.html?Sfmbt), [mam](http://flybase.bio.indiana.edu/.bin/fbidq.html?mam), [CG30158](http://flybase.bio.indiana.edu/.bin/fbidq.html?CG30158), [pan](http://flybase.bio.indiana.edu/.bin/fbidq.html?pan), [PKD](http://flybase.bio.indiana.edu/.bin/fbidq.html?PKD), [grn](http://flybase.bio.indiana.edu/.bin/fbidq.html?grn), [HLHm5](http://flybase.bio.indiana.edu/.bin/fbidq.html?HLHm5), [dap](http://flybase.bio.indiana.edu/.bin/fbidq.html?dap), [pros](http://flybase.bio.indiana.edu/.bin/fbidq.html?pros), [mthl8](http://flybase.bio.indiana.edu/.bin/fbidq.html?mthl8), [Ccap](http://flybase.bio.indiana.edu/.bin/fbidq.html?Ccap), [tipE](http://flybase.bio.indiana.edu/.bin/fbidq.html?tipE), [Wnt4](http://flybase.bio.indiana.edu/.bin/fbidq.html?Wnt4), [Mad1](http://flybase.bio.indiana.edu/.bin/fbidq.html?Mad1), [CG30372](http://flybase.bio.indiana.edu/.bin/fbidq.html?CG30372),[CG10188](http://flybase.bio.indiana.edu/.bin/fbidq.html?CG10188), [CG42629](http://flybase.bio.indiana.edu/.bin/fbidq.html?CG42629), [CG31760](http://flybase.bio.indiana.edu/.bin/fbidq.html?CG31760), [Klp54D](http://flybase.bio.indiana.edu/.bin/fbidq.html?Klp54D), [pr-set7](http://flybase.bio.indiana.edu/.bin/fbidq.html?pr-set7), [bchs](http://flybase.bio.indiana.edu/.bin/fbidq.html?bchs), [dsx](http://flybase.bio.indiana.edu/.bin/fbidq.html?dsx), [Dh](http://flybase.bio.indiana.edu/.bin/fbidq.html?Dh), [klu](http://flybase.bio.indiana.edu/.bin/fbidq.html?klu), [SPR](http://flybase.bio.indiana.edu/.bin/fbidq.html?SPR), [sqz](http://flybase.bio.indiana.edu/.bin/fbidq.html?sqz), [tld](http://flybase.bio.indiana.edu/.bin/fbidq.html?tld), [CG7879](http://flybase.bio.indiana.edu/.bin/fbidq.html?CG7879), [CG9121](http://flybase.bio.indiana.edu/.bin/fbidq.html?CG9121), [gcm2](http://flybase.bio.indiana.edu/.bin/fbidq.html?gcm2), [inaE](http://flybase.bio.indiana.edu/.bin/fbidq.html?inaE), [CanB](http://flybase.bio.indiana.edu/.bin/fbidq.html?CanB), [Aef1](http://flybase.bio.indiana.edu/.bin/fbidq.html?Aef1), [can](http://flybase.bio.indiana.edu/.bin/fbidq.html?can), [Pask](http://flybase.bio.indiana.edu/.bin/fbidq.html?Pask), [rab3-GEF](http://flybase.bio.indiana.edu/.bin/fbidq.html?rab3-GEF), [CG13253](http://flybase.bio.indiana.edu/.bin/fbidq.html?CG13253), [ovo](http://flybase.bio.indiana.edu/.bin/fbidq.html?ovo), [CG6197](http://flybase.bio.indiana.edu/.bin/fbidq.html?CG6197), [phol](http://flybase.bio.indiana.edu/.bin/fbidq.html?phol), [Dsp1](http://flybase.bio.indiana.edu/.bin/fbidq.html?Dsp1), [CG14216](http://flybase.bio.indiana.edu/.bin/fbidq.html?CG14216), [ds](http://flybase.bio.indiana.edu/.bin/fbidq.html?ds), [Oli](http://flybase.bio.indiana.edu/.bin/fbidq.html?Oli), [Nelf-A](http://flybase.bio.indiana.edu/.bin/fbidq.html?Nelf-A), [Syt4](http://flybase.bio.indiana.edu/.bin/fbidq.html?Syt4), [GATAd](http://flybase.bio.indiana.edu/.bin/fbidq.html?GATAd), [fbl6](http://flybase.bio.indiana.edu/.bin/fbidq.html?fbl6), [SIFR](http://flybase.bio.indiana.edu/.bin/fbidq.html?SIFR),[CG2061](http://flybase.bio.indiana.edu/.bin/fbidq.html?CG2061), [Ast](http://flybase.bio.indiana.edu/.bin/fbidq.html?Ast), [Drl-2](http://flybase.bio.indiana.edu/.bin/fbidq.html?Drl-2), [CG32944](http://flybase.bio.indiana.edu/.bin/fbidq.html?CG32944), [Tk](http://flybase.bio.indiana.edu/.bin/fbidq.html?Tk), [Ilp5](http://flybase.bio.indiana.edu/.bin/fbidq.html?Ilp5), [CG10336](http://flybase.bio.indiana.edu/.bin/fbidq.html?CG10336), [Gap1](http://flybase.bio.indiana.edu/.bin/fbidq.html?Gap1), [Toll-6](http://flybase.bio.indiana.edu/.bin/fbidq.html?Toll-6), [CG3227](http://flybase.bio.indiana.edu/.bin/fbidq.html?CG3227), [run](http://flybase.bio.indiana.edu/.bin/fbidq.html?run), [MESR4](http://flybase.bio.indiana.edu/.bin/fbidq.html?MESR4), [sNPF-R](http://flybase.bio.indiana.edu/.bin/fbidq.html?sNPF-R), [tara](http://flybase.bio.indiana.edu/.bin/fbidq.html?tara), [Hel89B](http://flybase.bio.indiana.edu/.bin/fbidq.html?Hel89B), [koko](http://flybase.bio.indiana.edu/.bin/fbidq.html?koko), [stan](http://flybase.bio.indiana.edu/.bin/fbidq.html?stan), [CanA-14F](http://flybase.bio.indiana.edu/.bin/fbidq.html?CanA-14F), [tutl](http://flybase.bio.indiana.edu/.bin/fbidq.html?tutl), [Ptth](http://flybase.bio.indiana.edu/.bin/fbidq.html?Ptth), [Rab2](http://flybase.bio.indiana.edu/.bin/fbidq.html?Rab2), [Ilp2](http://flybase.bio.indiana.edu/.bin/fbidq.html?Ilp2), [ph-p](http://flybase.bio.indiana.edu/.bin/fbidq.html?ph-p), [onecut](http://flybase.bio.indiana.edu/.bin/fbidq.html?onecut), [Frq1](http://flybase.bio.indiana.edu/.bin/fbidq.html?Frq1), [CG7757](http://flybase.bio.indiana.edu/.bin/fbidq.html?CG7757), [SPoCk](http://flybase.bio.indiana.edu/.bin/fbidq.html?SPoCk), [sna](http://flybase.bio.indiana.edu/.bin/fbidq.html?sna), [Taf1](http://flybase.bio.indiana.edu/.bin/fbidq.html?Taf1), [Ggamma1](http://flybase.bio.indiana.edu/.bin/fbidq.html?Ggamma1), [MBD-like](http://flybase.bio.indiana.edu/.bin/fbidq.html?MBD-like), [esg](http://flybase.bio.indiana.edu/.bin/fbidq.html?esg), [RSG7](http://flybase.bio.indiana.edu/.bin/fbidq.html?RSG7),[m4](http://flybase.bio.indiana.edu/.bin/fbidq.html?m4), [dom](http://flybase.bio.indiana.edu/.bin/fbidq.html?dom), [CG32532](http://flybase.bio.indiana.edu/.bin/fbidq.html?CG32532), [Dh31](http://flybase.bio.indiana.edu/.bin/fbidq.html?Dh31), [shi](http://flybase.bio.indiana.edu/.bin/fbidq.html?shi), [Spred](http://flybase.bio.indiana.edu/.bin/fbidq.html?Spred), [siz](http://flybase.bio.indiana.edu/.bin/fbidq.html?siz), [B-H2](http://flybase.bio.indiana.edu/.bin/fbidq.html?B-H2), [tyn](http://flybase.bio.indiana.edu/.bin/fbidq.html?tyn), [CG3822](http://flybase.bio.indiana.edu/.bin/fbidq.html?CG3822), [CadN](http://flybase.bio.indiana.edu/.bin/fbidq.html?CadN), [Mfap1](http://flybase.bio.indiana.edu/.bin/fbidq.html?Mfap1), [CG13995](http://flybase.bio.indiana.edu/.bin/fbidq.html?CG13995), [E(bx)](http://flybase.bio.indiana.edu/.bin/fbidq.html?E(bx)), [Dms](http://flybase.bio.indiana.edu/.bin/fbidq.html?Dms), [Pitslre](http://flybase.bio.indiana.edu/.bin/fbidq.html?Pitslre), [pburs](http://flybase.bio.indiana.edu/.bin/fbidq.html?pburs), [cos](http://flybase.bio.indiana.edu/.bin/fbidq.html?cos) |
| [biological regulation](http://amigo.geneontology.org/cgi-bin/amigo/go.cgi?view=details&query=GO:0065007) | 605 of 1594 genes, 38.0% | 2277 of 7634 genes, 29.8% | 6.91e-18 | 0.00% | 0.00 | [endoA](http://flybase.bio.indiana.edu/.bin/fbidq.html?endoA), [Sh](http://flybase.bio.indiana.edu/.bin/fbidq.html?Sh), [dock](http://flybase.bio.indiana.edu/.bin/fbidq.html?dock), [tna](http://flybase.bio.indiana.edu/.bin/fbidq.html?tna), [CG8500](http://flybase.bio.indiana.edu/.bin/fbidq.html?CG8500), [mub](http://flybase.bio.indiana.edu/.bin/fbidq.html?mub), [rn](http://flybase.bio.indiana.edu/.bin/fbidq.html?rn), [ap](http://flybase.bio.indiana.edu/.bin/fbidq.html?ap), [cenG1A](http://flybase.bio.indiana.edu/.bin/fbidq.html?cenG1A), [kkv](http://flybase.bio.indiana.edu/.bin/fbidq.html?kkv), [tlk](http://flybase.bio.indiana.edu/.bin/fbidq.html?tlk), [malpha](http://flybase.bio.indiana.edu/.bin/fbidq.html?malpha), [os](http://flybase.bio.indiana.edu/.bin/fbidq.html?os), [Axn](http://flybase.bio.indiana.edu/.bin/fbidq.html?Axn), [mtrm](http://flybase.bio.indiana.edu/.bin/fbidq.html?mtrm), [Hcf](http://flybase.bio.indiana.edu/.bin/fbidq.html?Hcf), [Traf6](http://flybase.bio.indiana.edu/.bin/fbidq.html?Traf6), [Leucokinin](http://flybase.bio.indiana.edu/.bin/fbidq.html?Leucokinin), [NetB](http://flybase.bio.indiana.edu/.bin/fbidq.html?NetB), [rdgC](http://flybase.bio.indiana.edu/.bin/fbidq.html?rdgC), [Ssrp](http://flybase.bio.indiana.edu/.bin/fbidq.html?Ssrp), [Lar](http://flybase.bio.indiana.edu/.bin/fbidq.html?Lar), [scrib](http://flybase.bio.indiana.edu/.bin/fbidq.html?scrib), [HGTX](http://flybase.bio.indiana.edu/.bin/fbidq.html?HGTX), [not](http://flybase.bio.indiana.edu/.bin/fbidq.html?not), [nvy](http://flybase.bio.indiana.edu/.bin/fbidq.html?nvy), [CG7650](http://flybase.bio.indiana.edu/.bin/fbidq.html?CG7650), [nab](http://flybase.bio.indiana.edu/.bin/fbidq.html?nab), [Ddc](http://flybase.bio.indiana.edu/.bin/fbidq.html?Ddc), [U2af38](http://flybase.bio.indiana.edu/.bin/fbidq.html?U2af38), [HLHmdelta](http://flybase.bio.indiana.edu/.bin/fbidq.html?HLHmdelta), [ATbp](http://flybase.bio.indiana.edu/.bin/fbidq.html?ATbp), [7B2](http://flybase.bio.indiana.edu/.bin/fbidq.html?7B2), [alph](http://flybase.bio.indiana.edu/.bin/fbidq.html?alph), [Traf4](http://flybase.bio.indiana.edu/.bin/fbidq.html?Traf4), [TBPH](http://flybase.bio.indiana.edu/.bin/fbidq.html?TBPH), [Snap25](http://flybase.bio.indiana.edu/.bin/fbidq.html?Snap25), [Teh2](http://flybase.bio.indiana.edu/.bin/fbidq.html?Teh2),[Fer2](http://flybase.bio.indiana.edu/.bin/fbidq.html?Fer2), [CG34400](http://flybase.bio.indiana.edu/.bin/fbidq.html?CG34400), [mGluRA](http://flybase.bio.indiana.edu/.bin/fbidq.html?mGluRA), [ena](http://flybase.bio.indiana.edu/.bin/fbidq.html?ena), [dsh](http://flybase.bio.indiana.edu/.bin/fbidq.html?dsh), [Nf-YC](http://flybase.bio.indiana.edu/.bin/fbidq.html?Nf-YC), [Pli](http://flybase.bio.indiana.edu/.bin/fbidq.html?Pli), [Hel25E](http://flybase.bio.indiana.edu/.bin/fbidq.html?Hel25E), [comt](http://flybase.bio.indiana.edu/.bin/fbidq.html?comt), [CG31140](http://flybase.bio.indiana.edu/.bin/fbidq.html?CG31140), [PP2A-B](http://flybase.bio.indiana.edu/.bin/fbidq.html?PP2A-B), [qvr](http://flybase.bio.indiana.edu/.bin/fbidq.html?qvr), [jet](http://flybase.bio.indiana.edu/.bin/fbidq.html?jet), [DAAM](http://flybase.bio.indiana.edu/.bin/fbidq.html?DAAM), [rho-5](http://flybase.bio.indiana.edu/.bin/fbidq.html?rho-5), [nmo](http://flybase.bio.indiana.edu/.bin/fbidq.html?nmo), [Tusp](http://flybase.bio.indiana.edu/.bin/fbidq.html?Tusp), [5-HT1A](http://flybase.bio.indiana.edu/.bin/fbidq.html?5-HT1A), [Psi](http://flybase.bio.indiana.edu/.bin/fbidq.html?Psi), [Gug](http://flybase.bio.indiana.edu/.bin/fbidq.html?Gug), [Actn](http://flybase.bio.indiana.edu/.bin/fbidq.html?Actn), [ed](http://flybase.bio.indiana.edu/.bin/fbidq.html?ed), [pad](http://flybase.bio.indiana.edu/.bin/fbidq.html?pad), [daw](http://flybase.bio.indiana.edu/.bin/fbidq.html?daw), [CG5036](http://flybase.bio.indiana.edu/.bin/fbidq.html?CG5036), [5-HT1B](http://flybase.bio.indiana.edu/.bin/fbidq.html?5-HT1B), [Fhos](http://flybase.bio.indiana.edu/.bin/fbidq.html?Fhos), [Rx](http://flybase.bio.indiana.edu/.bin/fbidq.html?Rx), [MED1](http://flybase.bio.indiana.edu/.bin/fbidq.html?MED1), [salr](http://flybase.bio.indiana.edu/.bin/fbidq.html?salr), [Apc](http://flybase.bio.indiana.edu/.bin/fbidq.html?Apc), [CG8795](http://flybase.bio.indiana.edu/.bin/fbidq.html?CG8795), [d4](http://flybase.bio.indiana.edu/.bin/fbidq.html?d4), [dan](http://flybase.bio.indiana.edu/.bin/fbidq.html?dan), [cort](http://flybase.bio.indiana.edu/.bin/fbidq.html?cort), [Jarid2](http://flybase.bio.indiana.edu/.bin/fbidq.html?Jarid2), [RN-tre](http://flybase.bio.indiana.edu/.bin/fbidq.html?RN-tre),[trx](http://flybase.bio.indiana.edu/.bin/fbidq.html?trx), [Btk29A](http://flybase.bio.indiana.edu/.bin/fbidq.html?Btk29A), [MED25](http://flybase.bio.indiana.edu/.bin/fbidq.html?MED25), [ETH](http://flybase.bio.indiana.edu/.bin/fbidq.html?ETH), [unc-5](http://flybase.bio.indiana.edu/.bin/fbidq.html?unc-5), [cdi](http://flybase.bio.indiana.edu/.bin/fbidq.html?cdi), [vfl](http://flybase.bio.indiana.edu/.bin/fbidq.html?vfl), [NPFR1](http://flybase.bio.indiana.edu/.bin/fbidq.html?NPFR1), [pygo](http://flybase.bio.indiana.edu/.bin/fbidq.html?pygo), [z](http://flybase.bio.indiana.edu/.bin/fbidq.html?z), [HLHmgamma](http://flybase.bio.indiana.edu/.bin/fbidq.html?HLHmgamma), [CG15556](http://flybase.bio.indiana.edu/.bin/fbidq.html?CG15556), [jar](http://flybase.bio.indiana.edu/.bin/fbidq.html?jar), [Ror](http://flybase.bio.indiana.edu/.bin/fbidq.html?Ror), [wor](http://flybase.bio.indiana.edu/.bin/fbidq.html?wor), [Atg1](http://flybase.bio.indiana.edu/.bin/fbidq.html?Atg1), [pUf68](http://flybase.bio.indiana.edu/.bin/fbidq.html?pUf68), [seq](http://flybase.bio.indiana.edu/.bin/fbidq.html?seq), [HDAC4](http://flybase.bio.indiana.edu/.bin/fbidq.html?HDAC4), [Cbl](http://flybase.bio.indiana.edu/.bin/fbidq.html?Cbl), [thr](http://flybase.bio.indiana.edu/.bin/fbidq.html?thr), [pog](http://flybase.bio.indiana.edu/.bin/fbidq.html?pog), [Pdp1](http://flybase.bio.indiana.edu/.bin/fbidq.html?Pdp1), [CASK](http://flybase.bio.indiana.edu/.bin/fbidq.html?CASK), [CG32758](http://flybase.bio.indiana.edu/.bin/fbidq.html?CG32758), [mtt](http://flybase.bio.indiana.edu/.bin/fbidq.html?mtt), [disp](http://flybase.bio.indiana.edu/.bin/fbidq.html?disp), [mip120](http://flybase.bio.indiana.edu/.bin/fbidq.html?mip120), [cpx](http://flybase.bio.indiana.edu/.bin/fbidq.html?cpx), [CG4022](http://flybase.bio.indiana.edu/.bin/fbidq.html?CG4022), [jumu](http://flybase.bio.indiana.edu/.bin/fbidq.html?jumu), [Su(var)2-HP2](http://flybase.bio.indiana.edu/.bin/fbidq.html?Su(var)2-HP2), [gfA](http://flybase.bio.indiana.edu/.bin/fbidq.html?gfA), [Usp7](http://flybase.bio.indiana.edu/.bin/fbidq.html?Usp7), [fd59A](http://flybase.bio.indiana.edu/.bin/fbidq.html?fd59A), [Rph](http://flybase.bio.indiana.edu/.bin/fbidq.html?Rph),[brat](http://flybase.bio.indiana.edu/.bin/fbidq.html?brat), [FBX011](http://flybase.bio.indiana.edu/.bin/fbidq.html?FBX011), [CG17343](http://flybase.bio.indiana.edu/.bin/fbidq.html?CG17343), [GABA-B-R2](http://flybase.bio.indiana.edu/.bin/fbidq.html?GABA-B-R2), [futsch](http://flybase.bio.indiana.edu/.bin/fbidq.html?futsch), [Ubx](http://flybase.bio.indiana.edu/.bin/fbidq.html?Ubx), [Cbp53E](http://flybase.bio.indiana.edu/.bin/fbidq.html?Cbp53E), [Jupiter](http://flybase.bio.indiana.edu/.bin/fbidq.html?Jupiter), [chm](http://flybase.bio.indiana.edu/.bin/fbidq.html?chm), [Rim](http://flybase.bio.indiana.edu/.bin/fbidq.html?Rim), [msl-1](http://flybase.bio.indiana.edu/.bin/fbidq.html?msl-1), [corn](http://flybase.bio.indiana.edu/.bin/fbidq.html?corn), [Sur-8](http://flybase.bio.indiana.edu/.bin/fbidq.html?Sur-8), [CG42574](http://flybase.bio.indiana.edu/.bin/fbidq.html?CG42574), [Rgl](http://flybase.bio.indiana.edu/.bin/fbidq.html?Rgl), [dac](http://flybase.bio.indiana.edu/.bin/fbidq.html?dac), [CG9007](http://flybase.bio.indiana.edu/.bin/fbidq.html?CG9007), [mav](http://flybase.bio.indiana.edu/.bin/fbidq.html?mav), [gol](http://flybase.bio.indiana.edu/.bin/fbidq.html?gol), [Ilp7](http://flybase.bio.indiana.edu/.bin/fbidq.html?Ilp7), [CG33275](http://flybase.bio.indiana.edu/.bin/fbidq.html?CG33275), [toy](http://flybase.bio.indiana.edu/.bin/fbidq.html?toy), [CG40351](http://flybase.bio.indiana.edu/.bin/fbidq.html?CG40351), [Lim3](http://flybase.bio.indiana.edu/.bin/fbidq.html?Lim3), [CG6136](http://flybase.bio.indiana.edu/.bin/fbidq.html?CG6136), [sd](http://flybase.bio.indiana.edu/.bin/fbidq.html?sd), [Aplip1](http://flybase.bio.indiana.edu/.bin/fbidq.html?Aplip1), [fs(1)h](http://flybase.bio.indiana.edu/.bin/fbidq.html?fs(1)h), [CG11347](http://flybase.bio.indiana.edu/.bin/fbidq.html?CG11347), [Ggamma30A](http://flybase.bio.indiana.edu/.bin/fbidq.html?Ggamma30A), [yrt](http://flybase.bio.indiana.edu/.bin/fbidq.html?yrt),[CG11294](http://flybase.bio.indiana.edu/.bin/fbidq.html?CG11294), [wls](http://flybase.bio.indiana.edu/.bin/fbidq.html?wls), [skl](http://flybase.bio.indiana.edu/.bin/fbidq.html?skl), [hiw](http://flybase.bio.indiana.edu/.bin/fbidq.html?hiw), [CG32653](http://flybase.bio.indiana.edu/.bin/fbidq.html?CG32653), [Atf-2](http://flybase.bio.indiana.edu/.bin/fbidq.html?Atf-2), [n-syb](http://flybase.bio.indiana.edu/.bin/fbidq.html?n-syb), [skd](http://flybase.bio.indiana.edu/.bin/fbidq.html?skd), [jing](http://flybase.bio.indiana.edu/.bin/fbidq.html?jing), [katanin-60](http://flybase.bio.indiana.edu/.bin/fbidq.html?katanin-60), [Camta](http://flybase.bio.indiana.edu/.bin/fbidq.html?Camta), [tll](http://flybase.bio.indiana.edu/.bin/fbidq.html?tll), [ato](http://flybase.bio.indiana.edu/.bin/fbidq.html?ato), [Fmrf](http://flybase.bio.indiana.edu/.bin/fbidq.html?Fmrf), [Iswi](http://flybase.bio.indiana.edu/.bin/fbidq.html?Iswi), [Spt6](http://flybase.bio.indiana.edu/.bin/fbidq.html?Spt6), [obst-A](http://flybase.bio.indiana.edu/.bin/fbidq.html?obst-A), [Pka-R1](http://flybase.bio.indiana.edu/.bin/fbidq.html?Pka-R1), [mago](http://flybase.bio.indiana.edu/.bin/fbidq.html?mago), [enc](http://flybase.bio.indiana.edu/.bin/fbidq.html?enc), [CG42533](http://flybase.bio.indiana.edu/.bin/fbidq.html?CG42533), [ksr](http://flybase.bio.indiana.edu/.bin/fbidq.html?ksr), [PMCA](http://flybase.bio.indiana.edu/.bin/fbidq.html?PMCA), [CG3618](http://flybase.bio.indiana.edu/.bin/fbidq.html?CG3618), [Gyc-89Da](http://flybase.bio.indiana.edu/.bin/fbidq.html?Gyc-89Da), [brp](http://flybase.bio.indiana.edu/.bin/fbidq.html?brp), [chn](http://flybase.bio.indiana.edu/.bin/fbidq.html?chn), [CG17124](http://flybase.bio.indiana.edu/.bin/fbidq.html?CG17124), [Khc-73](http://flybase.bio.indiana.edu/.bin/fbidq.html?Khc-73), [chif](http://flybase.bio.indiana.edu/.bin/fbidq.html?chif), [D2R](http://flybase.bio.indiana.edu/.bin/fbidq.html?D2R), [p130CAS](http://flybase.bio.indiana.edu/.bin/fbidq.html?p130CAS), [ewg](http://flybase.bio.indiana.edu/.bin/fbidq.html?ewg), [PHDP](http://flybase.bio.indiana.edu/.bin/fbidq.html?PHDP),[sano](http://flybase.bio.indiana.edu/.bin/fbidq.html?sano), [Cirl](http://flybase.bio.indiana.edu/.bin/fbidq.html?Cirl), [APC4](http://flybase.bio.indiana.edu/.bin/fbidq.html?APC4), [l(2)NC136](http://flybase.bio.indiana.edu/.bin/fbidq.html?l(2)NC136), [Su(var)3-3](http://flybase.bio.indiana.edu/.bin/fbidq.html?Su(var)3-3), [Bili](http://flybase.bio.indiana.edu/.bin/fbidq.html?Bili), [unc-13-4A](http://flybase.bio.indiana.edu/.bin/fbidq.html?unc-13-4A), [Dh44-R1](http://flybase.bio.indiana.edu/.bin/fbidq.html?Dh44-R1), [stet](http://flybase.bio.indiana.edu/.bin/fbidq.html?stet), [Sema-1a](http://flybase.bio.indiana.edu/.bin/fbidq.html?Sema-1a), [Eh](http://flybase.bio.indiana.edu/.bin/fbidq.html?Eh), [Hs3st-B](http://flybase.bio.indiana.edu/.bin/fbidq.html?Hs3st-B), [Sos](http://flybase.bio.indiana.edu/.bin/fbidq.html?Sos), [Fas2](http://flybase.bio.indiana.edu/.bin/fbidq.html?Fas2), [Klp61F](http://flybase.bio.indiana.edu/.bin/fbidq.html?Klp61F), [eya](http://flybase.bio.indiana.edu/.bin/fbidq.html?eya), [mAcR-60C](http://flybase.bio.indiana.edu/.bin/fbidq.html?mAcR-60C), [CG8557](http://flybase.bio.indiana.edu/.bin/fbidq.html?CG8557), [Vsx2](http://flybase.bio.indiana.edu/.bin/fbidq.html?Vsx2), [kat-60L1](http://flybase.bio.indiana.edu/.bin/fbidq.html?kat-60L1), [mtg](http://flybase.bio.indiana.edu/.bin/fbidq.html?mtg), [ft](http://flybase.bio.indiana.edu/.bin/fbidq.html?ft), [Crz](http://flybase.bio.indiana.edu/.bin/fbidq.html?Crz), [l(2)k16918](http://flybase.bio.indiana.edu/.bin/fbidq.html?l(2)k16918), [Takr86C](http://flybase.bio.indiana.edu/.bin/fbidq.html?Takr86C), [Dll](http://flybase.bio.indiana.edu/.bin/fbidq.html?Dll), [disco](http://flybase.bio.indiana.edu/.bin/fbidq.html?disco), [DopR](http://flybase.bio.indiana.edu/.bin/fbidq.html?DopR), [D12](http://flybase.bio.indiana.edu/.bin/fbidq.html?D12), [mew](http://flybase.bio.indiana.edu/.bin/fbidq.html?mew), [didum](http://flybase.bio.indiana.edu/.bin/fbidq.html?didum), [Lis-1](http://flybase.bio.indiana.edu/.bin/fbidq.html?Lis-1),[bmm](http://flybase.bio.indiana.edu/.bin/fbidq.html?bmm), [ems](http://flybase.bio.indiana.edu/.bin/fbidq.html?ems), [CG16896](http://flybase.bio.indiana.edu/.bin/fbidq.html?CG16896), [eys](http://flybase.bio.indiana.edu/.bin/fbidq.html?eys), [tup](http://flybase.bio.indiana.edu/.bin/fbidq.html?tup), [Rab3](http://flybase.bio.indiana.edu/.bin/fbidq.html?Rab3), [CG6191](http://flybase.bio.indiana.edu/.bin/fbidq.html?CG6191), [trio](http://flybase.bio.indiana.edu/.bin/fbidq.html?trio), [pdm2](http://flybase.bio.indiana.edu/.bin/fbidq.html?pdm2), [RabX4](http://flybase.bio.indiana.edu/.bin/fbidq.html?RabX4), [rgr](http://flybase.bio.indiana.edu/.bin/fbidq.html?rgr), [Lkr](http://flybase.bio.indiana.edu/.bin/fbidq.html?Lkr), [spel1](http://flybase.bio.indiana.edu/.bin/fbidq.html?spel1), [CG31665](http://flybase.bio.indiana.edu/.bin/fbidq.html?CG31665), [Gad1](http://flybase.bio.indiana.edu/.bin/fbidq.html?Gad1), [CG7918](http://flybase.bio.indiana.edu/.bin/fbidq.html?CG7918), [CaMKII](http://flybase.bio.indiana.edu/.bin/fbidq.html?CaMKII), [DopR2](http://flybase.bio.indiana.edu/.bin/fbidq.html?DopR2), [l(3)psg2](http://flybase.bio.indiana.edu/.bin/fbidq.html?l(3)psg2), [nerfin-1](http://flybase.bio.indiana.edu/.bin/fbidq.html?nerfin-1), [pigs](http://flybase.bio.indiana.edu/.bin/fbidq.html?pigs), [5-HT7](http://flybase.bio.indiana.edu/.bin/fbidq.html?5-HT7), [Lgr3](http://flybase.bio.indiana.edu/.bin/fbidq.html?Lgr3), [polo](http://flybase.bio.indiana.edu/.bin/fbidq.html?polo), [Syn1](http://flybase.bio.indiana.edu/.bin/fbidq.html?Syn1), [Ac13E](http://flybase.bio.indiana.edu/.bin/fbidq.html?Ac13E), [Ssdp](http://flybase.bio.indiana.edu/.bin/fbidq.html?Ssdp), [CG8155](http://flybase.bio.indiana.edu/.bin/fbidq.html?CG8155), [CG32105](http://flybase.bio.indiana.edu/.bin/fbidq.html?CG32105), [Fer3](http://flybase.bio.indiana.edu/.bin/fbidq.html?Fer3), [dalao](http://flybase.bio.indiana.edu/.bin/fbidq.html?dalao), [CG1868](http://flybase.bio.indiana.edu/.bin/fbidq.html?CG1868), [sr](http://flybase.bio.indiana.edu/.bin/fbidq.html?sr),[Lim1](http://flybase.bio.indiana.edu/.bin/fbidq.html?Lim1), [nkd](http://flybase.bio.indiana.edu/.bin/fbidq.html?nkd), [RhoGEF3](http://flybase.bio.indiana.edu/.bin/fbidq.html?RhoGEF3), [danr](http://flybase.bio.indiana.edu/.bin/fbidq.html?danr), [Dsk](http://flybase.bio.indiana.edu/.bin/fbidq.html?Dsk), [RhoGEF4](http://flybase.bio.indiana.edu/.bin/fbidq.html?RhoGEF4), [vvl](http://flybase.bio.indiana.edu/.bin/fbidq.html?vvl), [Brd8](http://flybase.bio.indiana.edu/.bin/fbidq.html?Brd8), [betaInt-nu](http://flybase.bio.indiana.edu/.bin/fbidq.html?betaInt-nu), [sgg](http://flybase.bio.indiana.edu/.bin/fbidq.html?sgg), [Brd](http://flybase.bio.indiana.edu/.bin/fbidq.html?Brd), [Eip93F](http://flybase.bio.indiana.edu/.bin/fbidq.html?Eip93F), [Nmdar1](http://flybase.bio.indiana.edu/.bin/fbidq.html?Nmdar1), [gcm](http://flybase.bio.indiana.edu/.bin/fbidq.html?gcm), [Suv4-20](http://flybase.bio.indiana.edu/.bin/fbidq.html?Suv4-20), [Hsp67Bc](http://flybase.bio.indiana.edu/.bin/fbidq.html?Hsp67Bc), [ase](http://flybase.bio.indiana.edu/.bin/fbidq.html?ase), [scro](http://flybase.bio.indiana.edu/.bin/fbidq.html?scro), [elav](http://flybase.bio.indiana.edu/.bin/fbidq.html?elav), [MESK2](http://flybase.bio.indiana.edu/.bin/fbidq.html?MESK2), [CG6227](http://flybase.bio.indiana.edu/.bin/fbidq.html?CG6227), [Poxn](http://flybase.bio.indiana.edu/.bin/fbidq.html?Poxn), [sals](http://flybase.bio.indiana.edu/.bin/fbidq.html?sals), [Dat](http://flybase.bio.indiana.edu/.bin/fbidq.html?Dat), [CG15376](http://flybase.bio.indiana.edu/.bin/fbidq.html?CG15376), [fred](http://flybase.bio.indiana.edu/.bin/fbidq.html?fred), [ush](http://flybase.bio.indiana.edu/.bin/fbidq.html?ush), [Fak56D](http://flybase.bio.indiana.edu/.bin/fbidq.html?Fak56D), [Takr99D](http://flybase.bio.indiana.edu/.bin/fbidq.html?Takr99D), [Teh4](http://flybase.bio.indiana.edu/.bin/fbidq.html?Teh4), [Alh](http://flybase.bio.indiana.edu/.bin/fbidq.html?Alh), [Sox102F](http://flybase.bio.indiana.edu/.bin/fbidq.html?Sox102F), [Hex-A](http://flybase.bio.indiana.edu/.bin/fbidq.html?Hex-A),[egg](http://flybase.bio.indiana.edu/.bin/fbidq.html?egg), [CG13692](http://flybase.bio.indiana.edu/.bin/fbidq.html?CG13692), [CG5337](http://flybase.bio.indiana.edu/.bin/fbidq.html?CG5337), [CG12424](http://flybase.bio.indiana.edu/.bin/fbidq.html?CG12424), [Sp1](http://flybase.bio.indiana.edu/.bin/fbidq.html?Sp1), [Wnt5](http://flybase.bio.indiana.edu/.bin/fbidq.html?Wnt5), [fus](http://flybase.bio.indiana.edu/.bin/fbidq.html?fus), [sif](http://flybase.bio.indiana.edu/.bin/fbidq.html?sif), [Tbh](http://flybase.bio.indiana.edu/.bin/fbidq.html?Tbh), [Aac11](http://flybase.bio.indiana.edu/.bin/fbidq.html?Aac11), [MTA1-like](http://flybase.bio.indiana.edu/.bin/fbidq.html?MTA1-like), [ssh](http://flybase.bio.indiana.edu/.bin/fbidq.html?ssh), [CG42541](http://flybase.bio.indiana.edu/.bin/fbidq.html?CG42541), [PIP5K59B](http://flybase.bio.indiana.edu/.bin/fbidq.html?PIP5K59B), [mle](http://flybase.bio.indiana.edu/.bin/fbidq.html?mle), [Hr51](http://flybase.bio.indiana.edu/.bin/fbidq.html?Hr51), [lap](http://flybase.bio.indiana.edu/.bin/fbidq.html?lap), [CG11155](http://flybase.bio.indiana.edu/.bin/fbidq.html?CG11155), [CG8786](http://flybase.bio.indiana.edu/.bin/fbidq.html?CG8786), [wg](http://flybase.bio.indiana.edu/.bin/fbidq.html?wg), [CG11077](http://flybase.bio.indiana.edu/.bin/fbidq.html?CG11077), [fkh](http://flybase.bio.indiana.edu/.bin/fbidq.html?fkh), [Spps](http://flybase.bio.indiana.edu/.bin/fbidq.html?Spps), [Rhp](http://flybase.bio.indiana.edu/.bin/fbidq.html?Rhp), [fne](http://flybase.bio.indiana.edu/.bin/fbidq.html?fne), [en](http://flybase.bio.indiana.edu/.bin/fbidq.html?en), [sfl](http://flybase.bio.indiana.edu/.bin/fbidq.html?sfl), [CG13229](http://flybase.bio.indiana.edu/.bin/fbidq.html?CG13229), [Pde11](http://flybase.bio.indiana.edu/.bin/fbidq.html?Pde11), [SerT](http://flybase.bio.indiana.edu/.bin/fbidq.html?SerT), [Utx](http://flybase.bio.indiana.edu/.bin/fbidq.html?Utx), [Ct](http://flybase.bio.indiana.edu/.bin/fbidq.html?Ct), [fz2](http://flybase.bio.indiana.edu/.bin/fbidq.html?fz2), [numb](http://flybase.bio.indiana.edu/.bin/fbidq.html?numb), [Ocho](http://flybase.bio.indiana.edu/.bin/fbidq.html?Ocho),[CG14375](http://flybase.bio.indiana.edu/.bin/fbidq.html?CG14375), [Ets65A](http://flybase.bio.indiana.edu/.bin/fbidq.html?Ets65A), [Sxl](http://flybase.bio.indiana.edu/.bin/fbidq.html?Sxl), [stj](http://flybase.bio.indiana.edu/.bin/fbidq.html?stj), [JIL-1](http://flybase.bio.indiana.edu/.bin/fbidq.html?JIL-1), [Nf1](http://flybase.bio.indiana.edu/.bin/fbidq.html?Nf1), [AR-2](http://flybase.bio.indiana.edu/.bin/fbidq.html?AR-2), [jigr1](http://flybase.bio.indiana.edu/.bin/fbidq.html?jigr1), [bi](http://flybase.bio.indiana.edu/.bin/fbidq.html?bi), [tok](http://flybase.bio.indiana.edu/.bin/fbidq.html?tok), [Sytalpha](http://flybase.bio.indiana.edu/.bin/fbidq.html?Sytalpha), [NC2alpha](http://flybase.bio.indiana.edu/.bin/fbidq.html?NC2alpha), [blue](http://flybase.bio.indiana.edu/.bin/fbidq.html?blue), [mod(mdg4)](http://flybase.bio.indiana.edu/.bin/fbidq.html?mod(mdg4)), [bab1](http://flybase.bio.indiana.edu/.bin/fbidq.html?bab1), [Galpha49B](http://flybase.bio.indiana.edu/.bin/fbidq.html?Galpha49B), [Snoo](http://flybase.bio.indiana.edu/.bin/fbidq.html?Snoo), [rut](http://flybase.bio.indiana.edu/.bin/fbidq.html?rut), [RhoGAP100F](http://flybase.bio.indiana.edu/.bin/fbidq.html?RhoGAP100F), [uif](http://flybase.bio.indiana.edu/.bin/fbidq.html?uif), [nub](http://flybase.bio.indiana.edu/.bin/fbidq.html?nub), [btd](http://flybase.bio.indiana.edu/.bin/fbidq.html?btd), [Mmp2](http://flybase.bio.indiana.edu/.bin/fbidq.html?Mmp2), [amon](http://flybase.bio.indiana.edu/.bin/fbidq.html?amon), [fd68A](http://flybase.bio.indiana.edu/.bin/fbidq.html?fd68A), [Nplp1](http://flybase.bio.indiana.edu/.bin/fbidq.html?Nplp1), [B-H1](http://flybase.bio.indiana.edu/.bin/fbidq.html?B-H1), [CG32206](http://flybase.bio.indiana.edu/.bin/fbidq.html?CG32206), [Nipped-A](http://flybase.bio.indiana.edu/.bin/fbidq.html?Nipped-A), [fd102C](http://flybase.bio.indiana.edu/.bin/fbidq.html?fd102C), [Spf45](http://flybase.bio.indiana.edu/.bin/fbidq.html?Spf45), [hth](http://flybase.bio.indiana.edu/.bin/fbidq.html?hth), [14-3-3zeta](http://flybase.bio.indiana.edu/.bin/fbidq.html?14-3-3zeta), [pzg](http://flybase.bio.indiana.edu/.bin/fbidq.html?pzg), [lea](http://flybase.bio.indiana.edu/.bin/fbidq.html?lea), [shakB](http://flybase.bio.indiana.edu/.bin/fbidq.html?shakB), [Hey](http://flybase.bio.indiana.edu/.bin/fbidq.html?Hey), [plexB](http://flybase.bio.indiana.edu/.bin/fbidq.html?plexB), [AlstR](http://flybase.bio.indiana.edu/.bin/fbidq.html?AlstR), [loqs](http://flybase.bio.indiana.edu/.bin/fbidq.html?loqs), [icln](http://flybase.bio.indiana.edu/.bin/fbidq.html?icln), [mars](http://flybase.bio.indiana.edu/.bin/fbidq.html?mars), [IM10](http://flybase.bio.indiana.edu/.bin/fbidq.html?IM10), [neur](http://flybase.bio.indiana.edu/.bin/fbidq.html?neur), [Brf](http://flybase.bio.indiana.edu/.bin/fbidq.html?Brf), [CycE](http://flybase.bio.indiana.edu/.bin/fbidq.html?CycE), [CG32149](http://flybase.bio.indiana.edu/.bin/fbidq.html?CG32149), [Vdup1](http://flybase.bio.indiana.edu/.bin/fbidq.html?Vdup1), [mr](http://flybase.bio.indiana.edu/.bin/fbidq.html?mr), [orb2](http://flybase.bio.indiana.edu/.bin/fbidq.html?orb2), [wdn](http://flybase.bio.indiana.edu/.bin/fbidq.html?wdn), [synaptogyrin](http://flybase.bio.indiana.edu/.bin/fbidq.html?synaptogyrin), [sim](http://flybase.bio.indiana.edu/.bin/fbidq.html?sim), [gro](http://flybase.bio.indiana.edu/.bin/fbidq.html?gro), [Rya-r44F](http://flybase.bio.indiana.edu/.bin/fbidq.html?Rya-r44F), [scyl](http://flybase.bio.indiana.edu/.bin/fbidq.html?scyl), [hkl](http://flybase.bio.indiana.edu/.bin/fbidq.html?hkl), [bin3](http://flybase.bio.indiana.edu/.bin/fbidq.html?bin3), [Rpd3](http://flybase.bio.indiana.edu/.bin/fbidq.html?Rpd3), [tyf](http://flybase.bio.indiana.edu/.bin/fbidq.html?tyf), [CG10107](http://flybase.bio.indiana.edu/.bin/fbidq.html?CG10107), [aret](http://flybase.bio.indiana.edu/.bin/fbidq.html?aret), [HLHm7](http://flybase.bio.indiana.edu/.bin/fbidq.html?HLHm7), [Sox21b](http://flybase.bio.indiana.edu/.bin/fbidq.html?Sox21b), [tow](http://flybase.bio.indiana.edu/.bin/fbidq.html?tow), [CG15609](http://flybase.bio.indiana.edu/.bin/fbidq.html?CG15609), [CG12187](http://flybase.bio.indiana.edu/.bin/fbidq.html?CG12187), [Pten](http://flybase.bio.indiana.edu/.bin/fbidq.html?Pten),[unc-13](http://flybase.bio.indiana.edu/.bin/fbidq.html?unc-13), [wda](http://flybase.bio.indiana.edu/.bin/fbidq.html?wda), [npf](http://flybase.bio.indiana.edu/.bin/fbidq.html?npf), [mira](http://flybase.bio.indiana.edu/.bin/fbidq.html?mira), [MED14](http://flybase.bio.indiana.edu/.bin/fbidq.html?MED14), [Spindly](http://flybase.bio.indiana.edu/.bin/fbidq.html?Spindly), [Drep-2](http://flybase.bio.indiana.edu/.bin/fbidq.html?Drep-2), [Dip3](http://flybase.bio.indiana.edu/.bin/fbidq.html?Dip3), [oa2](http://flybase.bio.indiana.edu/.bin/fbidq.html?oa2), [elk](http://flybase.bio.indiana.edu/.bin/fbidq.html?elk), [Rab26](http://flybase.bio.indiana.edu/.bin/fbidq.html?Rab26), [phl](http://flybase.bio.indiana.edu/.bin/fbidq.html?phl), [sNPF](http://flybase.bio.indiana.edu/.bin/fbidq.html?sNPF), [Pde6](http://flybase.bio.indiana.edu/.bin/fbidq.html?Pde6), [plx](http://flybase.bio.indiana.edu/.bin/fbidq.html?plx), [CG9098](http://flybase.bio.indiana.edu/.bin/fbidq.html?CG9098), [CG8108](http://flybase.bio.indiana.edu/.bin/fbidq.html?CG8108), [IFa](http://flybase.bio.indiana.edu/.bin/fbidq.html?IFa), [CG7708](http://flybase.bio.indiana.edu/.bin/fbidq.html?CG7708), [Alk](http://flybase.bio.indiana.edu/.bin/fbidq.html?Alk), [Vang](http://flybase.bio.indiana.edu/.bin/fbidq.html?Vang), [Pde1c](http://flybase.bio.indiana.edu/.bin/fbidq.html?Pde1c), [Sin3A](http://flybase.bio.indiana.edu/.bin/fbidq.html?Sin3A), [casp](http://flybase.bio.indiana.edu/.bin/fbidq.html?casp), [Smox](http://flybase.bio.indiana.edu/.bin/fbidq.html?Smox), [Plc21C](http://flybase.bio.indiana.edu/.bin/fbidq.html?Plc21C), [Antp](http://flybase.bio.indiana.edu/.bin/fbidq.html?Antp), [Rdl](http://flybase.bio.indiana.edu/.bin/fbidq.html?Rdl), [GRHRII](http://flybase.bio.indiana.edu/.bin/fbidq.html?GRHRII), [l(3)neo38](http://flybase.bio.indiana.edu/.bin/fbidq.html?l(3)neo38), [CG10362](http://flybase.bio.indiana.edu/.bin/fbidq.html?CG10362), [Ast-C](http://flybase.bio.indiana.edu/.bin/fbidq.html?Ast-C), [cindr](http://flybase.bio.indiana.edu/.bin/fbidq.html?cindr), [Gbeta5](http://flybase.bio.indiana.edu/.bin/fbidq.html?Gbeta5),[Ptx1](http://flybase.bio.indiana.edu/.bin/fbidq.html?Ptx1), [Nhe3](http://flybase.bio.indiana.edu/.bin/fbidq.html?Nhe3), [hug](http://flybase.bio.indiana.edu/.bin/fbidq.html?hug), [hbn](http://flybase.bio.indiana.edu/.bin/fbidq.html?hbn), [Syn2](http://flybase.bio.indiana.edu/.bin/fbidq.html?Syn2), [tsh](http://flybase.bio.indiana.edu/.bin/fbidq.html?tsh), [e(y)3](http://flybase.bio.indiana.edu/.bin/fbidq.html?e(y)3), [CRMP](http://flybase.bio.indiana.edu/.bin/fbidq.html?CRMP), [Cdk5](http://flybase.bio.indiana.edu/.bin/fbidq.html?Cdk5), [oc](http://flybase.bio.indiana.edu/.bin/fbidq.html?oc), [Cep135](http://flybase.bio.indiana.edu/.bin/fbidq.html?Cep135), [spt4](http://flybase.bio.indiana.edu/.bin/fbidq.html?spt4), [CG4641](http://flybase.bio.indiana.edu/.bin/fbidq.html?CG4641), [ey](http://flybase.bio.indiana.edu/.bin/fbidq.html?ey), [Mip](http://flybase.bio.indiana.edu/.bin/fbidq.html?Mip), [baz](http://flybase.bio.indiana.edu/.bin/fbidq.html?baz), [Oamb](http://flybase.bio.indiana.edu/.bin/fbidq.html?Oamb), [SK](http://flybase.bio.indiana.edu/.bin/fbidq.html?SK), [CG34381](http://flybase.bio.indiana.edu/.bin/fbidq.html?CG34381), [rst](http://flybase.bio.indiana.edu/.bin/fbidq.html?rst), [Bgb](http://flybase.bio.indiana.edu/.bin/fbidq.html?Bgb), [l(1)sc](http://flybase.bio.indiana.edu/.bin/fbidq.html?l(1)sc), [comm](http://flybase.bio.indiana.edu/.bin/fbidq.html?comm), [grh](http://flybase.bio.indiana.edu/.bin/fbidq.html?grh), [Vsx1](http://flybase.bio.indiana.edu/.bin/fbidq.html?Vsx1), [Cdk5alpha](http://flybase.bio.indiana.edu/.bin/fbidq.html?Cdk5alpha), [CG34362](http://flybase.bio.indiana.edu/.bin/fbidq.html?CG34362), [Cha](http://flybase.bio.indiana.edu/.bin/fbidq.html?Cha), [crol](http://flybase.bio.indiana.edu/.bin/fbidq.html?crol), [retn](http://flybase.bio.indiana.edu/.bin/fbidq.html?retn), [Ac3](http://flybase.bio.indiana.edu/.bin/fbidq.html?Ac3), [rl](http://flybase.bio.indiana.edu/.bin/fbidq.html?rl), [Syt1](http://flybase.bio.indiana.edu/.bin/fbidq.html?Syt1), [CG17760](http://flybase.bio.indiana.edu/.bin/fbidq.html?CG17760), [Nup153](http://flybase.bio.indiana.edu/.bin/fbidq.html?Nup153), [CG32683](http://flybase.bio.indiana.edu/.bin/fbidq.html?CG32683), [tey](http://flybase.bio.indiana.edu/.bin/fbidq.html?tey),[Saf-B](http://flybase.bio.indiana.edu/.bin/fbidq.html?Saf-B), [CG33960](http://flybase.bio.indiana.edu/.bin/fbidq.html?CG33960), [dnr1](http://flybase.bio.indiana.edu/.bin/fbidq.html?dnr1), [acj6](http://flybase.bio.indiana.edu/.bin/fbidq.html?acj6), [caup](http://flybase.bio.indiana.edu/.bin/fbidq.html?caup), [Cbp80](http://flybase.bio.indiana.edu/.bin/fbidq.html?Cbp80), [vimar](http://flybase.bio.indiana.edu/.bin/fbidq.html?vimar), [Rox8](http://flybase.bio.indiana.edu/.bin/fbidq.html?Rox8), [Dg](http://flybase.bio.indiana.edu/.bin/fbidq.html?Dg), [Ast-CC](http://flybase.bio.indiana.edu/.bin/fbidq.html?Ast-CC), [Trim9](http://flybase.bio.indiana.edu/.bin/fbidq.html?Trim9), [GABA-B-R3](http://flybase.bio.indiana.edu/.bin/fbidq.html?GABA-B-R3), [Evi5](http://flybase.bio.indiana.edu/.bin/fbidq.html?Evi5), [br](http://flybase.bio.indiana.edu/.bin/fbidq.html?br), [Gycalpha99B](http://flybase.bio.indiana.edu/.bin/fbidq.html?Gycalpha99B), [CG15537](http://flybase.bio.indiana.edu/.bin/fbidq.html?CG15537), [neo](http://flybase.bio.indiana.edu/.bin/fbidq.html?neo), [CG11376](http://flybase.bio.indiana.edu/.bin/fbidq.html?CG11376), [PNUTS](http://flybase.bio.indiana.edu/.bin/fbidq.html?PNUTS), [Gsc](http://flybase.bio.indiana.edu/.bin/fbidq.html?Gsc), [lola](http://flybase.bio.indiana.edu/.bin/fbidq.html?lola), [HLH4C](http://flybase.bio.indiana.edu/.bin/fbidq.html?HLH4C), [Pcl](http://flybase.bio.indiana.edu/.bin/fbidq.html?Pcl), [simj](http://flybase.bio.indiana.edu/.bin/fbidq.html?simj), [CG31158](http://flybase.bio.indiana.edu/.bin/fbidq.html?CG31158), [Awh](http://flybase.bio.indiana.edu/.bin/fbidq.html?Awh), [tap](http://flybase.bio.indiana.edu/.bin/fbidq.html?tap), [Appl](http://flybase.bio.indiana.edu/.bin/fbidq.html?Appl), [metro](http://flybase.bio.indiana.edu/.bin/fbidq.html?metro), [erm](http://flybase.bio.indiana.edu/.bin/fbidq.html?erm), [Nrk](http://flybase.bio.indiana.edu/.bin/fbidq.html?Nrk), [CG4328](http://flybase.bio.indiana.edu/.bin/fbidq.html?CG4328), [Ilp3](http://flybase.bio.indiana.edu/.bin/fbidq.html?Ilp3), [Taf4](http://flybase.bio.indiana.edu/.bin/fbidq.html?Taf4),[Nrg](http://flybase.bio.indiana.edu/.bin/fbidq.html?Nrg), [CG34384](http://flybase.bio.indiana.edu/.bin/fbidq.html?CG34384), [insc](http://flybase.bio.indiana.edu/.bin/fbidq.html?insc), [CG32447](http://flybase.bio.indiana.edu/.bin/fbidq.html?CG32447), [stau](http://flybase.bio.indiana.edu/.bin/fbidq.html?stau), [otk](http://flybase.bio.indiana.edu/.bin/fbidq.html?otk), [phyl](http://flybase.bio.indiana.edu/.bin/fbidq.html?phyl), [msl-2](http://flybase.bio.indiana.edu/.bin/fbidq.html?msl-2), [vg](http://flybase.bio.indiana.edu/.bin/fbidq.html?vg), [fz](http://flybase.bio.indiana.edu/.bin/fbidq.html?fz), [robo](http://flybase.bio.indiana.edu/.bin/fbidq.html?robo), [bsk](http://flybase.bio.indiana.edu/.bin/fbidq.html?bsk), [brm](http://flybase.bio.indiana.edu/.bin/fbidq.html?brm), [nAcRalpha-30D](http://flybase.bio.indiana.edu/.bin/fbidq.html?nAcRalpha-30D), [CG13830](http://flybase.bio.indiana.edu/.bin/fbidq.html?CG13830), [sno](http://flybase.bio.indiana.edu/.bin/fbidq.html?sno), [RhoGAPp190](http://flybase.bio.indiana.edu/.bin/fbidq.html?RhoGAPp190), [Cad96Ca](http://flybase.bio.indiana.edu/.bin/fbidq.html?Cad96Ca), [Sfmbt](http://flybase.bio.indiana.edu/.bin/fbidq.html?Sfmbt), [ara](http://flybase.bio.indiana.edu/.bin/fbidq.html?ara), [mam](http://flybase.bio.indiana.edu/.bin/fbidq.html?mam), [CG30158](http://flybase.bio.indiana.edu/.bin/fbidq.html?CG30158), [pan](http://flybase.bio.indiana.edu/.bin/fbidq.html?pan), [PKD](http://flybase.bio.indiana.edu/.bin/fbidq.html?PKD), [grn](http://flybase.bio.indiana.edu/.bin/fbidq.html?grn), [HLHm5](http://flybase.bio.indiana.edu/.bin/fbidq.html?HLHm5), [dap](http://flybase.bio.indiana.edu/.bin/fbidq.html?dap), [pros](http://flybase.bio.indiana.edu/.bin/fbidq.html?pros), [mthl8](http://flybase.bio.indiana.edu/.bin/fbidq.html?mthl8), [Ccap](http://flybase.bio.indiana.edu/.bin/fbidq.html?Ccap), [tipE](http://flybase.bio.indiana.edu/.bin/fbidq.html?tipE), [Wnt4](http://flybase.bio.indiana.edu/.bin/fbidq.html?Wnt4), [Mad1](http://flybase.bio.indiana.edu/.bin/fbidq.html?Mad1),[CG30372](http://flybase.bio.indiana.edu/.bin/fbidq.html?CG30372), [CG10188](http://flybase.bio.indiana.edu/.bin/fbidq.html?CG10188), [CG42629](http://flybase.bio.indiana.edu/.bin/fbidq.html?CG42629), [CG31760](http://flybase.bio.indiana.edu/.bin/fbidq.html?CG31760), [Klp54D](http://flybase.bio.indiana.edu/.bin/fbidq.html?Klp54D), [pr-set7](http://flybase.bio.indiana.edu/.bin/fbidq.html?pr-set7), [bchs](http://flybase.bio.indiana.edu/.bin/fbidq.html?bchs), [pk](http://flybase.bio.indiana.edu/.bin/fbidq.html?pk), [dsx](http://flybase.bio.indiana.edu/.bin/fbidq.html?dsx), [Dh](http://flybase.bio.indiana.edu/.bin/fbidq.html?Dh), [klu](http://flybase.bio.indiana.edu/.bin/fbidq.html?klu), [SPR](http://flybase.bio.indiana.edu/.bin/fbidq.html?SPR), [sqz](http://flybase.bio.indiana.edu/.bin/fbidq.html?sqz), [tld](http://flybase.bio.indiana.edu/.bin/fbidq.html?tld), [CG7879](http://flybase.bio.indiana.edu/.bin/fbidq.html?CG7879), [CG9121](http://flybase.bio.indiana.edu/.bin/fbidq.html?CG9121), [gcm2](http://flybase.bio.indiana.edu/.bin/fbidq.html?gcm2), [inaE](http://flybase.bio.indiana.edu/.bin/fbidq.html?inaE), [CanB](http://flybase.bio.indiana.edu/.bin/fbidq.html?CanB), [Aef1](http://flybase.bio.indiana.edu/.bin/fbidq.html?Aef1), [SP2353](http://flybase.bio.indiana.edu/.bin/fbidq.html?SP2353), [can](http://flybase.bio.indiana.edu/.bin/fbidq.html?can), [Pask](http://flybase.bio.indiana.edu/.bin/fbidq.html?Pask), [rab3-GEF](http://flybase.bio.indiana.edu/.bin/fbidq.html?rab3-GEF), [CG13253](http://flybase.bio.indiana.edu/.bin/fbidq.html?CG13253), [ovo](http://flybase.bio.indiana.edu/.bin/fbidq.html?ovo), [CG6197](http://flybase.bio.indiana.edu/.bin/fbidq.html?CG6197), [phol](http://flybase.bio.indiana.edu/.bin/fbidq.html?phol), [Dsp1](http://flybase.bio.indiana.edu/.bin/fbidq.html?Dsp1), [CG14216](http://flybase.bio.indiana.edu/.bin/fbidq.html?CG14216), [ds](http://flybase.bio.indiana.edu/.bin/fbidq.html?ds), [Oli](http://flybase.bio.indiana.edu/.bin/fbidq.html?Oli), [Nelf-A](http://flybase.bio.indiana.edu/.bin/fbidq.html?Nelf-A),[Syt4](http://flybase.bio.indiana.edu/.bin/fbidq.html?Syt4), [GATAd](http://flybase.bio.indiana.edu/.bin/fbidq.html?GATAd), [fbl6](http://flybase.bio.indiana.edu/.bin/fbidq.html?fbl6), [SIFR](http://flybase.bio.indiana.edu/.bin/fbidq.html?SIFR), [CG2061](http://flybase.bio.indiana.edu/.bin/fbidq.html?CG2061), [Ast](http://flybase.bio.indiana.edu/.bin/fbidq.html?Ast), [Drl-2](http://flybase.bio.indiana.edu/.bin/fbidq.html?Drl-2), [CG32944](http://flybase.bio.indiana.edu/.bin/fbidq.html?CG32944), [Tk](http://flybase.bio.indiana.edu/.bin/fbidq.html?Tk), [Ilp5](http://flybase.bio.indiana.edu/.bin/fbidq.html?Ilp5), [CG10336](http://flybase.bio.indiana.edu/.bin/fbidq.html?CG10336), [Gap1](http://flybase.bio.indiana.edu/.bin/fbidq.html?Gap1), [Toll-6](http://flybase.bio.indiana.edu/.bin/fbidq.html?Toll-6), [CG3227](http://flybase.bio.indiana.edu/.bin/fbidq.html?CG3227), [run](http://flybase.bio.indiana.edu/.bin/fbidq.html?run), [MESR4](http://flybase.bio.indiana.edu/.bin/fbidq.html?MESR4), [sNPF-R](http://flybase.bio.indiana.edu/.bin/fbidq.html?sNPF-R), [tara](http://flybase.bio.indiana.edu/.bin/fbidq.html?tara), [Hel89B](http://flybase.bio.indiana.edu/.bin/fbidq.html?Hel89B), [koko](http://flybase.bio.indiana.edu/.bin/fbidq.html?koko), [stan](http://flybase.bio.indiana.edu/.bin/fbidq.html?stan), [CanA-14F](http://flybase.bio.indiana.edu/.bin/fbidq.html?CanA-14F), [tutl](http://flybase.bio.indiana.edu/.bin/fbidq.html?tutl), [Ptth](http://flybase.bio.indiana.edu/.bin/fbidq.html?Ptth), [Rab2](http://flybase.bio.indiana.edu/.bin/fbidq.html?Rab2), [Ilp2](http://flybase.bio.indiana.edu/.bin/fbidq.html?Ilp2), [ph-p](http://flybase.bio.indiana.edu/.bin/fbidq.html?ph-p), [onecut](http://flybase.bio.indiana.edu/.bin/fbidq.html?onecut), [Frq1](http://flybase.bio.indiana.edu/.bin/fbidq.html?Frq1), [CG7757](http://flybase.bio.indiana.edu/.bin/fbidq.html?CG7757), [SPoCk](http://flybase.bio.indiana.edu/.bin/fbidq.html?SPoCk), [sna](http://flybase.bio.indiana.edu/.bin/fbidq.html?sna), [Taf1](http://flybase.bio.indiana.edu/.bin/fbidq.html?Taf1), [Ggamma1](http://flybase.bio.indiana.edu/.bin/fbidq.html?Ggamma1),[MBD-like](http://flybase.bio.indiana.edu/.bin/fbidq.html?MBD-like), [esg](http://flybase.bio.indiana.edu/.bin/fbidq.html?esg), [RSG7](http://flybase.bio.indiana.edu/.bin/fbidq.html?RSG7), [m4](http://flybase.bio.indiana.edu/.bin/fbidq.html?m4), [dom](http://flybase.bio.indiana.edu/.bin/fbidq.html?dom), [CG32532](http://flybase.bio.indiana.edu/.bin/fbidq.html?CG32532), [Dh31](http://flybase.bio.indiana.edu/.bin/fbidq.html?Dh31), [klar](http://flybase.bio.indiana.edu/.bin/fbidq.html?klar), [shi](http://flybase.bio.indiana.edu/.bin/fbidq.html?shi), [Spred](http://flybase.bio.indiana.edu/.bin/fbidq.html?Spred), [siz](http://flybase.bio.indiana.edu/.bin/fbidq.html?siz), [B-H2](http://flybase.bio.indiana.edu/.bin/fbidq.html?B-H2), [tyn](http://flybase.bio.indiana.edu/.bin/fbidq.html?tyn), [CG3822](http://flybase.bio.indiana.edu/.bin/fbidq.html?CG3822), [CadN](http://flybase.bio.indiana.edu/.bin/fbidq.html?CadN), [Mfap1](http://flybase.bio.indiana.edu/.bin/fbidq.html?Mfap1), [CG13995](http://flybase.bio.indiana.edu/.bin/fbidq.html?CG13995), [E(bx)](http://flybase.bio.indiana.edu/.bin/fbidq.html?E(bx)), [Dms](http://flybase.bio.indiana.edu/.bin/fbidq.html?Dms), [Snap](http://flybase.bio.indiana.edu/.bin/fbidq.html?Snap), [Pitslre](http://flybase.bio.indiana.edu/.bin/fbidq.html?Pitslre), [pburs](http://flybase.bio.indiana.edu/.bin/fbidq.html?pburs), [cos](http://flybase.bio.indiana.edu/.bin/fbidq.html?cos) |
| [cell surface receptor signaling pathway](http://amigo.geneontology.org/cgi-bin/amigo/go.cgi?view=details&query=GO:0007166) | 188 of 1594 genes, 11.8% | 529 of 7634 genes, 6.9% | 3.32e-15 | 0.00% | 0.00 | [fz2](http://flybase.bio.indiana.edu/.bin/fbidq.html?fz2), [CG14375](http://flybase.bio.indiana.edu/.bin/fbidq.html?CG14375), [Ocho](http://flybase.bio.indiana.edu/.bin/fbidq.html?Ocho), [numb](http://flybase.bio.indiana.edu/.bin/fbidq.html?numb), [skd](http://flybase.bio.indiana.edu/.bin/fbidq.html?skd), [dock](http://flybase.bio.indiana.edu/.bin/fbidq.html?dock), [tll](http://flybase.bio.indiana.edu/.bin/fbidq.html?tll), [Camta](http://flybase.bio.indiana.edu/.bin/fbidq.html?Camta), [ato](http://flybase.bio.indiana.edu/.bin/fbidq.html?ato), [Fmrf](http://flybase.bio.indiana.edu/.bin/fbidq.html?Fmrf), [Sxl](http://flybase.bio.indiana.edu/.bin/fbidq.html?Sxl), [Trim9](http://flybase.bio.indiana.edu/.bin/fbidq.html?Trim9), [GABA-B-R3](http://flybase.bio.indiana.edu/.bin/fbidq.html?GABA-B-R3), [mago](http://flybase.bio.indiana.edu/.bin/fbidq.html?mago), [Gycalpha99B](http://flybase.bio.indiana.edu/.bin/fbidq.html?Gycalpha99B), [malpha](http://flybase.bio.indiana.edu/.bin/fbidq.html?malpha), [AR-2](http://flybase.bio.indiana.edu/.bin/fbidq.html?AR-2), [ksr](http://flybase.bio.indiana.edu/.bin/fbidq.html?ksr), [Axn](http://flybase.bio.indiana.edu/.bin/fbidq.html?Axn), [Traf6](http://flybase.bio.indiana.edu/.bin/fbidq.html?Traf6), [Leucokinin](http://flybase.bio.indiana.edu/.bin/fbidq.html?Leucokinin), [D2R](http://flybase.bio.indiana.edu/.bin/fbidq.html?D2R), [rdgC](http://flybase.bio.indiana.edu/.bin/fbidq.html?rdgC), [ewg](http://flybase.bio.indiana.edu/.bin/fbidq.html?ewg), [Nrk](http://flybase.bio.indiana.edu/.bin/fbidq.html?Nrk), [Cirl](http://flybase.bio.indiana.edu/.bin/fbidq.html?Cirl), [Galpha49B](http://flybase.bio.indiana.edu/.bin/fbidq.html?Galpha49B), [Snoo](http://flybase.bio.indiana.edu/.bin/fbidq.html?Snoo), [Ilp3](http://flybase.bio.indiana.edu/.bin/fbidq.html?Ilp3), [Su(var)3-3](http://flybase.bio.indiana.edu/.bin/fbidq.html?Su(var)3-3), [Bili](http://flybase.bio.indiana.edu/.bin/fbidq.html?Bili), [rut](http://flybase.bio.indiana.edu/.bin/fbidq.html?rut), [Nrg](http://flybase.bio.indiana.edu/.bin/fbidq.html?Nrg), [uif](http://flybase.bio.indiana.edu/.bin/fbidq.html?uif), [CG34384](http://flybase.bio.indiana.edu/.bin/fbidq.html?CG34384),[Mmp2](http://flybase.bio.indiana.edu/.bin/fbidq.html?Mmp2), [HLHmdelta](http://flybase.bio.indiana.edu/.bin/fbidq.html?HLHmdelta), [Dh44-R1](http://flybase.bio.indiana.edu/.bin/fbidq.html?Dh44-R1), [stet](http://flybase.bio.indiana.edu/.bin/fbidq.html?stet), [Nplp1](http://flybase.bio.indiana.edu/.bin/fbidq.html?Nplp1), [CG32447](http://flybase.bio.indiana.edu/.bin/fbidq.html?CG32447), [Nipped-A](http://flybase.bio.indiana.edu/.bin/fbidq.html?Nipped-A), [CG32206](http://flybase.bio.indiana.edu/.bin/fbidq.html?CG32206), [7B2](http://flybase.bio.indiana.edu/.bin/fbidq.html?7B2), [Sema-1a](http://flybase.bio.indiana.edu/.bin/fbidq.html?Sema-1a), [Eh](http://flybase.bio.indiana.edu/.bin/fbidq.html?Eh), [Hs3st-B](http://flybase.bio.indiana.edu/.bin/fbidq.html?Hs3st-B), [Traf4](http://flybase.bio.indiana.edu/.bin/fbidq.html?Traf4), [otk](http://flybase.bio.indiana.edu/.bin/fbidq.html?otk), [Sos](http://flybase.bio.indiana.edu/.bin/fbidq.html?Sos), [phyl](http://flybase.bio.indiana.edu/.bin/fbidq.html?phyl), [mGluRA](http://flybase.bio.indiana.edu/.bin/fbidq.html?mGluRA), [dsh](http://flybase.bio.indiana.edu/.bin/fbidq.html?dsh), [pzg](http://flybase.bio.indiana.edu/.bin/fbidq.html?pzg), [Fas2](http://flybase.bio.indiana.edu/.bin/fbidq.html?Fas2), [Nf-YC](http://flybase.bio.indiana.edu/.bin/fbidq.html?Nf-YC), [fz](http://flybase.bio.indiana.edu/.bin/fbidq.html?fz), [robo](http://flybase.bio.indiana.edu/.bin/fbidq.html?robo), [mAcR-60C](http://flybase.bio.indiana.edu/.bin/fbidq.html?mAcR-60C), [brm](http://flybase.bio.indiana.edu/.bin/fbidq.html?brm), [bsk](http://flybase.bio.indiana.edu/.bin/fbidq.html?bsk), [Pli](http://flybase.bio.indiana.edu/.bin/fbidq.html?Pli), [CG31140](http://flybase.bio.indiana.edu/.bin/fbidq.html?CG31140), [ft](http://flybase.bio.indiana.edu/.bin/fbidq.html?ft), [Crz](http://flybase.bio.indiana.edu/.bin/fbidq.html?Crz), [Hey](http://flybase.bio.indiana.edu/.bin/fbidq.html?Hey), [sno](http://flybase.bio.indiana.edu/.bin/fbidq.html?sno), [AlstR](http://flybase.bio.indiana.edu/.bin/fbidq.html?AlstR), [mam](http://flybase.bio.indiana.edu/.bin/fbidq.html?mam),[l(2)k16918](http://flybase.bio.indiana.edu/.bin/fbidq.html?l(2)k16918), [Takr86C](http://flybase.bio.indiana.edu/.bin/fbidq.html?Takr86C), [pan](http://flybase.bio.indiana.edu/.bin/fbidq.html?pan), [IM10](http://flybase.bio.indiana.edu/.bin/fbidq.html?IM10), [DopR](http://flybase.bio.indiana.edu/.bin/fbidq.html?DopR), [neur](http://flybase.bio.indiana.edu/.bin/fbidq.html?neur), [Lis-1](http://flybase.bio.indiana.edu/.bin/fbidq.html?Lis-1), [rho-5](http://flybase.bio.indiana.edu/.bin/fbidq.html?rho-5), [nmo](http://flybase.bio.indiana.edu/.bin/fbidq.html?nmo), [5-HT1A](http://flybase.bio.indiana.edu/.bin/fbidq.html?5-HT1A), [tup](http://flybase.bio.indiana.edu/.bin/fbidq.html?tup), [mthl8](http://flybase.bio.indiana.edu/.bin/fbidq.html?mthl8), [Gug](http://flybase.bio.indiana.edu/.bin/fbidq.html?Gug), [ed](http://flybase.bio.indiana.edu/.bin/fbidq.html?ed), [Lkr](http://flybase.bio.indiana.edu/.bin/fbidq.html?Lkr), [Ccap](http://flybase.bio.indiana.edu/.bin/fbidq.html?Ccap), [daw](http://flybase.bio.indiana.edu/.bin/fbidq.html?daw), [CG31665](http://flybase.bio.indiana.edu/.bin/fbidq.html?CG31665), [CG5036](http://flybase.bio.indiana.edu/.bin/fbidq.html?CG5036), [5-HT1B](http://flybase.bio.indiana.edu/.bin/fbidq.html?5-HT1B), [Wnt4](http://flybase.bio.indiana.edu/.bin/fbidq.html?Wnt4), [CG7918](http://flybase.bio.indiana.edu/.bin/fbidq.html?CG7918), [Apc](http://flybase.bio.indiana.edu/.bin/fbidq.html?Apc), [CG8795](http://flybase.bio.indiana.edu/.bin/fbidq.html?CG8795), [DopR2](http://flybase.bio.indiana.edu/.bin/fbidq.html?DopR2), [gro](http://flybase.bio.indiana.edu/.bin/fbidq.html?gro), [pigs](http://flybase.bio.indiana.edu/.bin/fbidq.html?pigs), [CG31760](http://flybase.bio.indiana.edu/.bin/fbidq.html?CG31760), [Klp54D](http://flybase.bio.indiana.edu/.bin/fbidq.html?Klp54D), [5-HT7](http://flybase.bio.indiana.edu/.bin/fbidq.html?5-HT7), [Lgr3](http://flybase.bio.indiana.edu/.bin/fbidq.html?Lgr3), [Dh](http://flybase.bio.indiana.edu/.bin/fbidq.html?Dh), [ETH](http://flybase.bio.indiana.edu/.bin/fbidq.html?ETH), [cdi](http://flybase.bio.indiana.edu/.bin/fbidq.html?cdi),[SPR](http://flybase.bio.indiana.edu/.bin/fbidq.html?SPR), [NPFR1](http://flybase.bio.indiana.edu/.bin/fbidq.html?NPFR1), [pygo](http://flybase.bio.indiana.edu/.bin/fbidq.html?pygo), [HLHmgamma](http://flybase.bio.indiana.edu/.bin/fbidq.html?HLHmgamma), [nkd](http://flybase.bio.indiana.edu/.bin/fbidq.html?nkd), [CG15556](http://flybase.bio.indiana.edu/.bin/fbidq.html?CG15556), [tld](http://flybase.bio.indiana.edu/.bin/fbidq.html?tld), [Ror](http://flybase.bio.indiana.edu/.bin/fbidq.html?Ror), [tow](http://flybase.bio.indiana.edu/.bin/fbidq.html?tow), [CG15609](http://flybase.bio.indiana.edu/.bin/fbidq.html?CG15609), [Dsk](http://flybase.bio.indiana.edu/.bin/fbidq.html?Dsk), [Pten](http://flybase.bio.indiana.edu/.bin/fbidq.html?Pten), [npf](http://flybase.bio.indiana.edu/.bin/fbidq.html?npf), [sgg](http://flybase.bio.indiana.edu/.bin/fbidq.html?sgg), [Brd](http://flybase.bio.indiana.edu/.bin/fbidq.html?Brd), [Nmdar1](http://flybase.bio.indiana.edu/.bin/fbidq.html?Nmdar1), [oa2](http://flybase.bio.indiana.edu/.bin/fbidq.html?oa2), [phl](http://flybase.bio.indiana.edu/.bin/fbidq.html?phl), [Cbl](http://flybase.bio.indiana.edu/.bin/fbidq.html?Cbl), [CG13253](http://flybase.bio.indiana.edu/.bin/fbidq.html?CG13253), [sNPF](http://flybase.bio.indiana.edu/.bin/fbidq.html?sNPF), [pog](http://flybase.bio.indiana.edu/.bin/fbidq.html?pog), [IFa](http://flybase.bio.indiana.edu/.bin/fbidq.html?IFa), [mtt](http://flybase.bio.indiana.edu/.bin/fbidq.html?mtt), [Alk](http://flybase.bio.indiana.edu/.bin/fbidq.html?Alk), [SIFR](http://flybase.bio.indiana.edu/.bin/fbidq.html?SIFR), [CG2061](http://flybase.bio.indiana.edu/.bin/fbidq.html?CG2061), [disp](http://flybase.bio.indiana.edu/.bin/fbidq.html?disp), [casp](http://flybase.bio.indiana.edu/.bin/fbidq.html?casp), [Ast](http://flybase.bio.indiana.edu/.bin/fbidq.html?Ast), [Smox](http://flybase.bio.indiana.edu/.bin/fbidq.html?Smox), [fred](http://flybase.bio.indiana.edu/.bin/fbidq.html?fred), [Drl-2](http://flybase.bio.indiana.edu/.bin/fbidq.html?Drl-2), [ush](http://flybase.bio.indiana.edu/.bin/fbidq.html?ush), [Fak56D](http://flybase.bio.indiana.edu/.bin/fbidq.html?Fak56D), [Tk](http://flybase.bio.indiana.edu/.bin/fbidq.html?Tk), [Ilp5](http://flybase.bio.indiana.edu/.bin/fbidq.html?Ilp5), [Gap1](http://flybase.bio.indiana.edu/.bin/fbidq.html?Gap1),[CG3227](http://flybase.bio.indiana.edu/.bin/fbidq.html?CG3227), [Takr99D](http://flybase.bio.indiana.edu/.bin/fbidq.html?Takr99D), [sNPF-R](http://flybase.bio.indiana.edu/.bin/fbidq.html?sNPF-R), [Hel89B](http://flybase.bio.indiana.edu/.bin/fbidq.html?Hel89B), [GRHRII](http://flybase.bio.indiana.edu/.bin/fbidq.html?GRHRII), [koko](http://flybase.bio.indiana.edu/.bin/fbidq.html?koko), [stan](http://flybase.bio.indiana.edu/.bin/fbidq.html?stan), [CG12424](http://flybase.bio.indiana.edu/.bin/fbidq.html?CG12424), [GABA-B-R2](http://flybase.bio.indiana.edu/.bin/fbidq.html?GABA-B-R2), [Ptth](http://flybase.bio.indiana.edu/.bin/fbidq.html?Ptth), [Ast-C](http://flybase.bio.indiana.edu/.bin/fbidq.html?Ast-C), [Gbeta5](http://flybase.bio.indiana.edu/.bin/fbidq.html?Gbeta5), [Ilp2](http://flybase.bio.indiana.edu/.bin/fbidq.html?Ilp2), [Wnt5](http://flybase.bio.indiana.edu/.bin/fbidq.html?Wnt5), [hug](http://flybase.bio.indiana.edu/.bin/fbidq.html?hug), [Ggamma1](http://flybase.bio.indiana.edu/.bin/fbidq.html?Ggamma1), [fus](http://flybase.bio.indiana.edu/.bin/fbidq.html?fus), [mav](http://flybase.bio.indiana.edu/.bin/fbidq.html?mav), [RSG7](http://flybase.bio.indiana.edu/.bin/fbidq.html?RSG7), [m4](http://flybase.bio.indiana.edu/.bin/fbidq.html?m4), [dom](http://flybase.bio.indiana.edu/.bin/fbidq.html?dom), [Tbh](http://flybase.bio.indiana.edu/.bin/fbidq.html?Tbh), [Ilp7](http://flybase.bio.indiana.edu/.bin/fbidq.html?Ilp7), [Dh31](http://flybase.bio.indiana.edu/.bin/fbidq.html?Dh31), [ey](http://flybase.bio.indiana.edu/.bin/fbidq.html?ey), [Mip](http://flybase.bio.indiana.edu/.bin/fbidq.html?Mip), [baz](http://flybase.bio.indiana.edu/.bin/fbidq.html?baz), [Oamb](http://flybase.bio.indiana.edu/.bin/fbidq.html?Oamb), [shi](http://flybase.bio.indiana.edu/.bin/fbidq.html?shi), [CG34381](http://flybase.bio.indiana.edu/.bin/fbidq.html?CG34381), [wg](http://flybase.bio.indiana.edu/.bin/fbidq.html?wg), [crol](http://flybase.bio.indiana.edu/.bin/fbidq.html?crol), [CG13995](http://flybase.bio.indiana.edu/.bin/fbidq.html?CG13995), [E(bx)](http://flybase.bio.indiana.edu/.bin/fbidq.html?E(bx)), [sfl](http://flybase.bio.indiana.edu/.bin/fbidq.html?sfl),[rl](http://flybase.bio.indiana.edu/.bin/fbidq.html?rl), [CG13229](http://flybase.bio.indiana.edu/.bin/fbidq.html?CG13229), [CG17760](http://flybase.bio.indiana.edu/.bin/fbidq.html?CG17760), [Dms](http://flybase.bio.indiana.edu/.bin/fbidq.html?Dms), [Nup153](http://flybase.bio.indiana.edu/.bin/fbidq.html?Nup153), [Ggamma30A](http://flybase.bio.indiana.edu/.bin/fbidq.html?Ggamma30A), [Utx](http://flybase.bio.indiana.edu/.bin/fbidq.html?Utx), [wls](http://flybase.bio.indiana.edu/.bin/fbidq.html?wls), [pburs](http://flybase.bio.indiana.edu/.bin/fbidq.html?pburs), [hiw](http://flybase.bio.indiana.edu/.bin/fbidq.html?hiw), [cos](http://flybase.bio.indiana.edu/.bin/fbidq.html?cos), [Ct](http://flybase.bio.indiana.edu/.bin/fbidq.html?Ct) |
| [generation of neurons](http://amigo.geneontology.org/cgi-bin/amigo/go.cgi?view=details&query=GO:0048699) | 215 of 1594 genes, 13.5% | 637 of 7634 genes, 8.3% | 8.74e-15 | 0.00% | 0.00 | [fz2](http://flybase.bio.indiana.edu/.bin/fbidq.html?fz2), [Sh](http://flybase.bio.indiana.edu/.bin/fbidq.html?Sh), [numb](http://flybase.bio.indiana.edu/.bin/fbidq.html?numb), [acj6](http://flybase.bio.indiana.edu/.bin/fbidq.html?acj6), [jing](http://flybase.bio.indiana.edu/.bin/fbidq.html?jing), [dock](http://flybase.bio.indiana.edu/.bin/fbidq.html?dock), [CG42256](http://flybase.bio.indiana.edu/.bin/fbidq.html?CG42256), [CG3703](http://flybase.bio.indiana.edu/.bin/fbidq.html?CG3703), [tll](http://flybase.bio.indiana.edu/.bin/fbidq.html?tll), [vimar](http://flybase.bio.indiana.edu/.bin/fbidq.html?vimar), [ato](http://flybase.bio.indiana.edu/.bin/fbidq.html?ato), [Iswi](http://flybase.bio.indiana.edu/.bin/fbidq.html?Iswi), [ap](http://flybase.bio.indiana.edu/.bin/fbidq.html?ap), [Dg](http://flybase.bio.indiana.edu/.bin/fbidq.html?Dg), [Trim9](http://flybase.bio.indiana.edu/.bin/fbidq.html?Trim9), [br](http://flybase.bio.indiana.edu/.bin/fbidq.html?br), [CG7154](http://flybase.bio.indiana.edu/.bin/fbidq.html?CG7154), [robo3](http://flybase.bio.indiana.edu/.bin/fbidq.html?robo3), [os](http://flybase.bio.indiana.edu/.bin/fbidq.html?os), [tok](http://flybase.bio.indiana.edu/.bin/fbidq.html?tok), [Ptp99A](http://flybase.bio.indiana.edu/.bin/fbidq.html?Ptp99A), [lola](http://flybase.bio.indiana.edu/.bin/fbidq.html?lola), [unc-104](http://flybase.bio.indiana.edu/.bin/fbidq.html?unc-104), [chif](http://flybase.bio.indiana.edu/.bin/fbidq.html?chif), [NetB](http://flybase.bio.indiana.edu/.bin/fbidq.html?NetB), [p130CAS](http://flybase.bio.indiana.edu/.bin/fbidq.html?p130CAS), [Tango10](http://flybase.bio.indiana.edu/.bin/fbidq.html?Tango10), [CG31158](http://flybase.bio.indiana.edu/.bin/fbidq.html?CG31158), [Appl](http://flybase.bio.indiana.edu/.bin/fbidq.html?Appl), [metro](http://flybase.bio.indiana.edu/.bin/fbidq.html?metro), [CG32137](http://flybase.bio.indiana.edu/.bin/fbidq.html?CG32137), [erm](http://flybase.bio.indiana.edu/.bin/fbidq.html?erm), [Nrk](http://flybase.bio.indiana.edu/.bin/fbidq.html?Nrk), [CG4328](http://flybase.bio.indiana.edu/.bin/fbidq.html?CG4328), [Galpha49B](http://flybase.bio.indiana.edu/.bin/fbidq.html?Galpha49B),[Snoo](http://flybase.bio.indiana.edu/.bin/fbidq.html?Snoo), [Lar](http://flybase.bio.indiana.edu/.bin/fbidq.html?Lar), [Taf4](http://flybase.bio.indiana.edu/.bin/fbidq.html?Taf4), [CG10249](http://flybase.bio.indiana.edu/.bin/fbidq.html?CG10249), [scrib](http://flybase.bio.indiana.edu/.bin/fbidq.html?scrib), [HGTX](http://flybase.bio.indiana.edu/.bin/fbidq.html?HGTX), [not](http://flybase.bio.indiana.edu/.bin/fbidq.html?not), [rut](http://flybase.bio.indiana.edu/.bin/fbidq.html?rut), [nvy](http://flybase.bio.indiana.edu/.bin/fbidq.html?nvy), [Nrg](http://flybase.bio.indiana.edu/.bin/fbidq.html?Nrg), [gogo](http://flybase.bio.indiana.edu/.bin/fbidq.html?gogo), [nub](http://flybase.bio.indiana.edu/.bin/fbidq.html?nub), [nab](http://flybase.bio.indiana.edu/.bin/fbidq.html?nab), [insc](http://flybase.bio.indiana.edu/.bin/fbidq.html?insc), [HLHmdelta](http://flybase.bio.indiana.edu/.bin/fbidq.html?HLHmdelta), [jeb](http://flybase.bio.indiana.edu/.bin/fbidq.html?jeb), [B-H1](http://flybase.bio.indiana.edu/.bin/fbidq.html?B-H1), [GluClalpha](http://flybase.bio.indiana.edu/.bin/fbidq.html?GluClalpha), [alph](http://flybase.bio.indiana.edu/.bin/fbidq.html?alph), [Actbeta](http://flybase.bio.indiana.edu/.bin/fbidq.html?Actbeta), [stau](http://flybase.bio.indiana.edu/.bin/fbidq.html?stau), [Sema-1a](http://flybase.bio.indiana.edu/.bin/fbidq.html?Sema-1a), [beat-Ib](http://flybase.bio.indiana.edu/.bin/fbidq.html?beat-Ib), [Hs3st-B](http://flybase.bio.indiana.edu/.bin/fbidq.html?Hs3st-B), [TBPH](http://flybase.bio.indiana.edu/.bin/fbidq.html?TBPH), [otk](http://flybase.bio.indiana.edu/.bin/fbidq.html?otk), [Sos](http://flybase.bio.indiana.edu/.bin/fbidq.html?Sos), [Fer2](http://flybase.bio.indiana.edu/.bin/fbidq.html?Fer2), [CG34400](http://flybase.bio.indiana.edu/.bin/fbidq.html?CG34400), [phyl](http://flybase.bio.indiana.edu/.bin/fbidq.html?phyl), [Ulp1](http://flybase.bio.indiana.edu/.bin/fbidq.html?Ulp1), [ena](http://flybase.bio.indiana.edu/.bin/fbidq.html?ena), [dsh](http://flybase.bio.indiana.edu/.bin/fbidq.html?dsh), [hth](http://flybase.bio.indiana.edu/.bin/fbidq.html?hth), [Fas2](http://flybase.bio.indiana.edu/.bin/fbidq.html?Fas2), [Nf-YC](http://flybase.bio.indiana.edu/.bin/fbidq.html?Nf-YC), [eya](http://flybase.bio.indiana.edu/.bin/fbidq.html?eya),[14-3-3zeta](http://flybase.bio.indiana.edu/.bin/fbidq.html?14-3-3zeta), [fz](http://flybase.bio.indiana.edu/.bin/fbidq.html?fz), [robo](http://flybase.bio.indiana.edu/.bin/fbidq.html?robo), [lea](http://flybase.bio.indiana.edu/.bin/fbidq.html?lea), [brm](http://flybase.bio.indiana.edu/.bin/fbidq.html?brm), [bsk](http://flybase.bio.indiana.edu/.bin/fbidq.html?bsk), [beat-Ia](http://flybase.bio.indiana.edu/.bin/fbidq.html?beat-Ia), [kat-60L1](http://flybase.bio.indiana.edu/.bin/fbidq.html?kat-60L1), [hig](http://flybase.bio.indiana.edu/.bin/fbidq.html?hig), [RhoGAPp190](http://flybase.bio.indiana.edu/.bin/fbidq.html?RhoGAPp190), [CG12004](http://flybase.bio.indiana.edu/.bin/fbidq.html?CG12004), [plexB](http://flybase.bio.indiana.edu/.bin/fbidq.html?plexB), [Klp64D](http://flybase.bio.indiana.edu/.bin/fbidq.html?Klp64D), [grn](http://flybase.bio.indiana.edu/.bin/fbidq.html?grn), [DAAM](http://flybase.bio.indiana.edu/.bin/fbidq.html?DAAM), [mew](http://flybase.bio.indiana.edu/.bin/fbidq.html?mew), [neur](http://flybase.bio.indiana.edu/.bin/fbidq.html?neur), [didum](http://flybase.bio.indiana.edu/.bin/fbidq.html?didum), [Lis-1](http://flybase.bio.indiana.edu/.bin/fbidq.html?Lis-1), [CycE](http://flybase.bio.indiana.edu/.bin/fbidq.html?CycE), [Brf](http://flybase.bio.indiana.edu/.bin/fbidq.html?Brf), [ems](http://flybase.bio.indiana.edu/.bin/fbidq.html?ems), [eys](http://flybase.bio.indiana.edu/.bin/fbidq.html?eys), [Mob2](http://flybase.bio.indiana.edu/.bin/fbidq.html?Mob2), [pros](http://flybase.bio.indiana.edu/.bin/fbidq.html?pros), [tup](http://flybase.bio.indiana.edu/.bin/fbidq.html?tup), [trio](http://flybase.bio.indiana.edu/.bin/fbidq.html?trio), [pdm2](http://flybase.bio.indiana.edu/.bin/fbidq.html?pdm2), [ed](http://flybase.bio.indiana.edu/.bin/fbidq.html?ed), [CG1463](http://flybase.bio.indiana.edu/.bin/fbidq.html?CG1463), [daw](http://flybase.bio.indiana.edu/.bin/fbidq.html?daw), [Wnt4](http://flybase.bio.indiana.edu/.bin/fbidq.html?Wnt4), [rictor](http://flybase.bio.indiana.edu/.bin/fbidq.html?rictor), [Apc](http://flybase.bio.indiana.edu/.bin/fbidq.html?Apc), [sim](http://flybase.bio.indiana.edu/.bin/fbidq.html?sim), [d4](http://flybase.bio.indiana.edu/.bin/fbidq.html?d4), [gro](http://flybase.bio.indiana.edu/.bin/fbidq.html?gro),[nerfin-1](http://flybase.bio.indiana.edu/.bin/fbidq.html?nerfin-1), [bchs](http://flybase.bio.indiana.edu/.bin/fbidq.html?bchs), [wnd](http://flybase.bio.indiana.edu/.bin/fbidq.html?wnd), [trx](http://flybase.bio.indiana.edu/.bin/fbidq.html?trx), [Rpd3](http://flybase.bio.indiana.edu/.bin/fbidq.html?Rpd3), [polo](http://flybase.bio.indiana.edu/.bin/fbidq.html?polo), [dsx](http://flybase.bio.indiana.edu/.bin/fbidq.html?dsx), [chinmo](http://flybase.bio.indiana.edu/.bin/fbidq.html?chinmo), [unc-5](http://flybase.bio.indiana.edu/.bin/fbidq.html?unc-5), [Ssdp](http://flybase.bio.indiana.edu/.bin/fbidq.html?Ssdp), [cdi](http://flybase.bio.indiana.edu/.bin/fbidq.html?cdi), [klu](http://flybase.bio.indiana.edu/.bin/fbidq.html?klu), [CG10107](http://flybase.bio.indiana.edu/.bin/fbidq.html?CG10107), [sqz](http://flybase.bio.indiana.edu/.bin/fbidq.html?sqz), [fend](http://flybase.bio.indiana.edu/.bin/fbidq.html?fend), [pygo](http://flybase.bio.indiana.edu/.bin/fbidq.html?pygo), [HLHm7](http://flybase.bio.indiana.edu/.bin/fbidq.html?HLHm7), [HLHmgamma](http://flybase.bio.indiana.edu/.bin/fbidq.html?HLHmgamma), [nkd](http://flybase.bio.indiana.edu/.bin/fbidq.html?nkd), [jar](http://flybase.bio.indiana.edu/.bin/fbidq.html?jar), [wor](http://flybase.bio.indiana.edu/.bin/fbidq.html?wor), [vvl](http://flybase.bio.indiana.edu/.bin/fbidq.html?vvl), [Pten](http://flybase.bio.indiana.edu/.bin/fbidq.html?Pten), [mira](http://flybase.bio.indiana.edu/.bin/fbidq.html?mira), [Ptp69D](http://flybase.bio.indiana.edu/.bin/fbidq.html?Ptp69D), [gcm2](http://flybase.bio.indiana.edu/.bin/fbidq.html?gcm2), [hts](http://flybase.bio.indiana.edu/.bin/fbidq.html?hts), [mp](http://flybase.bio.indiana.edu/.bin/fbidq.html?mp), [gcm](http://flybase.bio.indiana.edu/.bin/fbidq.html?gcm), [seq](http://flybase.bio.indiana.edu/.bin/fbidq.html?seq), [CG5890](http://flybase.bio.indiana.edu/.bin/fbidq.html?CG5890), [Dip3](http://flybase.bio.indiana.edu/.bin/fbidq.html?Dip3), [SP2353](http://flybase.bio.indiana.edu/.bin/fbidq.html?SP2353), [phl](http://flybase.bio.indiana.edu/.bin/fbidq.html?phl), [ase](http://flybase.bio.indiana.edu/.bin/fbidq.html?ase), [Cbl](http://flybase.bio.indiana.edu/.bin/fbidq.html?Cbl), [CG32685](http://flybase.bio.indiana.edu/.bin/fbidq.html?CG32685),[CG4893](http://flybase.bio.indiana.edu/.bin/fbidq.html?CG4893), [CG9098](http://flybase.bio.indiana.edu/.bin/fbidq.html?CG9098), [Alk](http://flybase.bio.indiana.edu/.bin/fbidq.html?Alk), [esn](http://flybase.bio.indiana.edu/.bin/fbidq.html?esn), [Vang](http://flybase.bio.indiana.edu/.bin/fbidq.html?Vang), [Sin3A](http://flybase.bio.indiana.edu/.bin/fbidq.html?Sin3A), [pot](http://flybase.bio.indiana.edu/.bin/fbidq.html?pot), [jumu](http://flybase.bio.indiana.edu/.bin/fbidq.html?jumu), [Smox](http://flybase.bio.indiana.edu/.bin/fbidq.html?Smox), [CG31475](http://flybase.bio.indiana.edu/.bin/fbidq.html?CG31475), [beat-Ic](http://flybase.bio.indiana.edu/.bin/fbidq.html?beat-Ic), [fd59A](http://flybase.bio.indiana.edu/.bin/fbidq.html?fd59A), [Gap1](http://flybase.bio.indiana.edu/.bin/fbidq.html?Gap1), [run](http://flybase.bio.indiana.edu/.bin/fbidq.html?run), [brat](http://flybase.bio.indiana.edu/.bin/fbidq.html?brat), [Antp](http://flybase.bio.indiana.edu/.bin/fbidq.html?Antp), [ko](http://flybase.bio.indiana.edu/.bin/fbidq.html?ko), [Eip71CD](http://flybase.bio.indiana.edu/.bin/fbidq.html?Eip71CD), [stan](http://flybase.bio.indiana.edu/.bin/fbidq.html?stan), [CG12424](http://flybase.bio.indiana.edu/.bin/fbidq.html?CG12424), [tutl](http://flybase.bio.indiana.edu/.bin/fbidq.html?tutl), [futsch](http://flybase.bio.indiana.edu/.bin/fbidq.html?futsch), [Ptx1](http://flybase.bio.indiana.edu/.bin/fbidq.html?Ptx1), [ph-p](http://flybase.bio.indiana.edu/.bin/fbidq.html?ph-p), [gukh](http://flybase.bio.indiana.edu/.bin/fbidq.html?gukh), [chm](http://flybase.bio.indiana.edu/.bin/fbidq.html?chm), [Rim](http://flybase.bio.indiana.edu/.bin/fbidq.html?Rim), [sna](http://flybase.bio.indiana.edu/.bin/fbidq.html?sna), [Wnt5](http://flybase.bio.indiana.edu/.bin/fbidq.html?Wnt5), [beat-IIa](http://flybase.bio.indiana.edu/.bin/fbidq.html?beat-IIa), [Ggamma1](http://flybase.bio.indiana.edu/.bin/fbidq.html?Ggamma1), [sm](http://flybase.bio.indiana.edu/.bin/fbidq.html?sm), [Syn2](http://flybase.bio.indiana.edu/.bin/fbidq.html?Syn2), [esg](http://flybase.bio.indiana.edu/.bin/fbidq.html?esg), [eas](http://flybase.bio.indiana.edu/.bin/fbidq.html?eas), [dac](http://flybase.bio.indiana.edu/.bin/fbidq.html?dac), [sif](http://flybase.bio.indiana.edu/.bin/fbidq.html?sif),[Cdk5](http://flybase.bio.indiana.edu/.bin/fbidq.html?Cdk5), [oc](http://flybase.bio.indiana.edu/.bin/fbidq.html?oc), [dom](http://flybase.bio.indiana.edu/.bin/fbidq.html?dom), [ey](http://flybase.bio.indiana.edu/.bin/fbidq.html?ey), [ssh](http://flybase.bio.indiana.edu/.bin/fbidq.html?ssh), [baz](http://flybase.bio.indiana.edu/.bin/fbidq.html?baz), [klar](http://flybase.bio.indiana.edu/.bin/fbidq.html?klar), [mle](http://flybase.bio.indiana.edu/.bin/fbidq.html?mle), [Hr51](http://flybase.bio.indiana.edu/.bin/fbidq.html?Hr51), [CG11155](http://flybase.bio.indiana.edu/.bin/fbidq.html?CG11155), [rst](http://flybase.bio.indiana.edu/.bin/fbidq.html?rst), [l(1)sc](http://flybase.bio.indiana.edu/.bin/fbidq.html?l(1)sc), [B-H2](http://flybase.bio.indiana.edu/.bin/fbidq.html?B-H2), [grh](http://flybase.bio.indiana.edu/.bin/fbidq.html?grh), [comm](http://flybase.bio.indiana.edu/.bin/fbidq.html?comm), [Cdk5alpha](http://flybase.bio.indiana.edu/.bin/fbidq.html?Cdk5alpha), [wg](http://flybase.bio.indiana.edu/.bin/fbidq.html?wg), [Lim3](http://flybase.bio.indiana.edu/.bin/fbidq.html?Lim3), [CadN](http://flybase.bio.indiana.edu/.bin/fbidq.html?CadN), [Aplip1](http://flybase.bio.indiana.edu/.bin/fbidq.html?Aplip1), [E(Pc)](http://flybase.bio.indiana.edu/.bin/fbidq.html?E(Pc)), [retn](http://flybase.bio.indiana.edu/.bin/fbidq.html?retn), [klg](http://flybase.bio.indiana.edu/.bin/fbidq.html?klg), [en](http://flybase.bio.indiana.edu/.bin/fbidq.html?en), [E(bx)](http://flybase.bio.indiana.edu/.bin/fbidq.html?E(bx)), [rl](http://flybase.bio.indiana.edu/.bin/fbidq.html?rl), [Snap](http://flybase.bio.indiana.edu/.bin/fbidq.html?Snap), [trn](http://flybase.bio.indiana.edu/.bin/fbidq.html?trn), [tey](http://flybase.bio.indiana.edu/.bin/fbidq.html?tey), [yrt](http://flybase.bio.indiana.edu/.bin/fbidq.html?yrt), [CG33960](http://flybase.bio.indiana.edu/.bin/fbidq.html?CG33960), [Ct](http://flybase.bio.indiana.edu/.bin/fbidq.html?Ct) |
| [signaling](http://amigo.geneontology.org/cgi-bin/amigo/go.cgi?view=details&query=GO:0023052) | 352 of 1594 genes, 22.1% | 1226 of 7634 genes, 16.1% | 4.10e-13 | 0.00% | 0.00 | [fz2](http://flybase.bio.indiana.edu/.bin/fbidq.html?fz2), [endoA](http://flybase.bio.indiana.edu/.bin/fbidq.html?endoA), [CG14375](http://flybase.bio.indiana.edu/.bin/fbidq.html?CG14375), [Ocho](http://flybase.bio.indiana.edu/.bin/fbidq.html?Ocho), [Sh](http://flybase.bio.indiana.edu/.bin/fbidq.html?Sh), [numb](http://flybase.bio.indiana.edu/.bin/fbidq.html?numb), [dock](http://flybase.bio.indiana.edu/.bin/fbidq.html?dock), [stj](http://flybase.bio.indiana.edu/.bin/fbidq.html?stj), [Sxl](http://flybase.bio.indiana.edu/.bin/fbidq.html?Sxl), [CG8500](http://flybase.bio.indiana.edu/.bin/fbidq.html?CG8500), [cenG1A](http://flybase.bio.indiana.edu/.bin/fbidq.html?cenG1A), [Nf1](http://flybase.bio.indiana.edu/.bin/fbidq.html?Nf1), [malpha](http://flybase.bio.indiana.edu/.bin/fbidq.html?malpha), [AR-2](http://flybase.bio.indiana.edu/.bin/fbidq.html?AR-2), [os](http://flybase.bio.indiana.edu/.bin/fbidq.html?os), [Axn](http://flybase.bio.indiana.edu/.bin/fbidq.html?Axn), [Sytalpha](http://flybase.bio.indiana.edu/.bin/fbidq.html?Sytalpha), [Traf6](http://flybase.bio.indiana.edu/.bin/fbidq.html?Traf6), [Leucokinin](http://flybase.bio.indiana.edu/.bin/fbidq.html?Leucokinin), [rdgC](http://flybase.bio.indiana.edu/.bin/fbidq.html?rdgC), [Galpha49B](http://flybase.bio.indiana.edu/.bin/fbidq.html?Galpha49B), [Snoo](http://flybase.bio.indiana.edu/.bin/fbidq.html?Snoo), [scrib](http://flybase.bio.indiana.edu/.bin/fbidq.html?scrib), [rut](http://flybase.bio.indiana.edu/.bin/fbidq.html?rut), [CG7650](http://flybase.bio.indiana.edu/.bin/fbidq.html?CG7650), [RhoGAP100F](http://flybase.bio.indiana.edu/.bin/fbidq.html?RhoGAP100F), [uif](http://flybase.bio.indiana.edu/.bin/fbidq.html?uif), [Mmp2](http://flybase.bio.indiana.edu/.bin/fbidq.html?Mmp2), [HLHmdelta](http://flybase.bio.indiana.edu/.bin/fbidq.html?HLHmdelta), [Nplp1](http://flybase.bio.indiana.edu/.bin/fbidq.html?Nplp1), [Nipped-A](http://flybase.bio.indiana.edu/.bin/fbidq.html?Nipped-A),[CG32206](http://flybase.bio.indiana.edu/.bin/fbidq.html?CG32206), [7B2](http://flybase.bio.indiana.edu/.bin/fbidq.html?7B2), [alph](http://flybase.bio.indiana.edu/.bin/fbidq.html?alph), [Traf4](http://flybase.bio.indiana.edu/.bin/fbidq.html?Traf4), [TBPH](http://flybase.bio.indiana.edu/.bin/fbidq.html?TBPH), [Snap25](http://flybase.bio.indiana.edu/.bin/fbidq.html?Snap25), [mGluRA](http://flybase.bio.indiana.edu/.bin/fbidq.html?mGluRA), [dsh](http://flybase.bio.indiana.edu/.bin/fbidq.html?dsh), [pzg](http://flybase.bio.indiana.edu/.bin/fbidq.html?pzg), [Nf-YC](http://flybase.bio.indiana.edu/.bin/fbidq.html?Nf-YC), [14-3-3zeta](http://flybase.bio.indiana.edu/.bin/fbidq.html?14-3-3zeta), [Pli](http://flybase.bio.indiana.edu/.bin/fbidq.html?Pli), [shakB](http://flybase.bio.indiana.edu/.bin/fbidq.html?shakB), [comt](http://flybase.bio.indiana.edu/.bin/fbidq.html?comt), [CG31140](http://flybase.bio.indiana.edu/.bin/fbidq.html?CG31140), [Hey](http://flybase.bio.indiana.edu/.bin/fbidq.html?Hey), [plexB](http://flybase.bio.indiana.edu/.bin/fbidq.html?plexB), [AlstR](http://flybase.bio.indiana.edu/.bin/fbidq.html?AlstR), [PP2A-B](http://flybase.bio.indiana.edu/.bin/fbidq.html?PP2A-B), [qvr](http://flybase.bio.indiana.edu/.bin/fbidq.html?qvr), [mars](http://flybase.bio.indiana.edu/.bin/fbidq.html?mars), [IM10](http://flybase.bio.indiana.edu/.bin/fbidq.html?IM10), [neur](http://flybase.bio.indiana.edu/.bin/fbidq.html?neur), [CycE](http://flybase.bio.indiana.edu/.bin/fbidq.html?CycE), [rho-5](http://flybase.bio.indiana.edu/.bin/fbidq.html?rho-5), [nmo](http://flybase.bio.indiana.edu/.bin/fbidq.html?nmo), [Tusp](http://flybase.bio.indiana.edu/.bin/fbidq.html?Tusp), [5-HT1A](http://flybase.bio.indiana.edu/.bin/fbidq.html?5-HT1A), [CG32149](http://flybase.bio.indiana.edu/.bin/fbidq.html?CG32149), [Vdup1](http://flybase.bio.indiana.edu/.bin/fbidq.html?Vdup1), [Gug](http://flybase.bio.indiana.edu/.bin/fbidq.html?Gug), [ed](http://flybase.bio.indiana.edu/.bin/fbidq.html?ed), [mr](http://flybase.bio.indiana.edu/.bin/fbidq.html?mr), [daw](http://flybase.bio.indiana.edu/.bin/fbidq.html?daw),[CG5036](http://flybase.bio.indiana.edu/.bin/fbidq.html?CG5036), [5-HT1B](http://flybase.bio.indiana.edu/.bin/fbidq.html?5-HT1B), [synaptogyrin](http://flybase.bio.indiana.edu/.bin/fbidq.html?synaptogyrin), [Apc](http://flybase.bio.indiana.edu/.bin/fbidq.html?Apc), [sim](http://flybase.bio.indiana.edu/.bin/fbidq.html?sim), [CG8795](http://flybase.bio.indiana.edu/.bin/fbidq.html?CG8795), [gro](http://flybase.bio.indiana.edu/.bin/fbidq.html?gro), [RN-tre](http://flybase.bio.indiana.edu/.bin/fbidq.html?RN-tre), [scyl](http://flybase.bio.indiana.edu/.bin/fbidq.html?scyl), [Btk29A](http://flybase.bio.indiana.edu/.bin/fbidq.html?Btk29A), [ETH](http://flybase.bio.indiana.edu/.bin/fbidq.html?ETH), [unc-5](http://flybase.bio.indiana.edu/.bin/fbidq.html?unc-5), [cdi](http://flybase.bio.indiana.edu/.bin/fbidq.html?cdi), [NPFR1](http://flybase.bio.indiana.edu/.bin/fbidq.html?NPFR1), [pygo](http://flybase.bio.indiana.edu/.bin/fbidq.html?pygo), [HLHmgamma](http://flybase.bio.indiana.edu/.bin/fbidq.html?HLHmgamma), [CG15556](http://flybase.bio.indiana.edu/.bin/fbidq.html?CG15556), [Ror](http://flybase.bio.indiana.edu/.bin/fbidq.html?Ror), [tow](http://flybase.bio.indiana.edu/.bin/fbidq.html?tow), [CG15609](http://flybase.bio.indiana.edu/.bin/fbidq.html?CG15609), [wor](http://flybase.bio.indiana.edu/.bin/fbidq.html?wor), [Atg1](http://flybase.bio.indiana.edu/.bin/fbidq.html?Atg1), [CG12187](http://flybase.bio.indiana.edu/.bin/fbidq.html?CG12187), [Pten](http://flybase.bio.indiana.edu/.bin/fbidq.html?Pten), [unc-13](http://flybase.bio.indiana.edu/.bin/fbidq.html?unc-13), [npf](http://flybase.bio.indiana.edu/.bin/fbidq.html?npf), [Ptp69D](http://flybase.bio.indiana.edu/.bin/fbidq.html?Ptp69D), [bowl](http://flybase.bio.indiana.edu/.bin/fbidq.html?bowl), [oa2](http://flybase.bio.indiana.edu/.bin/fbidq.html?oa2), [elk](http://flybase.bio.indiana.edu/.bin/fbidq.html?elk), [phl](http://flybase.bio.indiana.edu/.bin/fbidq.html?phl), [Cbl](http://flybase.bio.indiana.edu/.bin/fbidq.html?Cbl), [Rab26](http://flybase.bio.indiana.edu/.bin/fbidq.html?Rab26),[Pde6](http://flybase.bio.indiana.edu/.bin/fbidq.html?Pde6), [sNPF](http://flybase.bio.indiana.edu/.bin/fbidq.html?sNPF), [pog](http://flybase.bio.indiana.edu/.bin/fbidq.html?pog), [plx](http://flybase.bio.indiana.edu/.bin/fbidq.html?plx), [CASK](http://flybase.bio.indiana.edu/.bin/fbidq.html?CASK), [CG9098](http://flybase.bio.indiana.edu/.bin/fbidq.html?CG9098), [CG8108](http://flybase.bio.indiana.edu/.bin/fbidq.html?CG8108), [IFa](http://flybase.bio.indiana.edu/.bin/fbidq.html?IFa), [mtt](http://flybase.bio.indiana.edu/.bin/fbidq.html?mtt), [CG32758](http://flybase.bio.indiana.edu/.bin/fbidq.html?CG32758), [Alk](http://flybase.bio.indiana.edu/.bin/fbidq.html?Alk), [CG7708](http://flybase.bio.indiana.edu/.bin/fbidq.html?CG7708), [Pde1c](http://flybase.bio.indiana.edu/.bin/fbidq.html?Pde1c), [disp](http://flybase.bio.indiana.edu/.bin/fbidq.html?disp), [cpx](http://flybase.bio.indiana.edu/.bin/fbidq.html?cpx), [casp](http://flybase.bio.indiana.edu/.bin/fbidq.html?casp), [CG4022](http://flybase.bio.indiana.edu/.bin/fbidq.html?CG4022), [Smox](http://flybase.bio.indiana.edu/.bin/fbidq.html?Smox), [gfA](http://flybase.bio.indiana.edu/.bin/fbidq.html?gfA), [Plc21C](http://flybase.bio.indiana.edu/.bin/fbidq.html?Plc21C), [CG31781](http://flybase.bio.indiana.edu/.bin/fbidq.html?CG31781), [Rph](http://flybase.bio.indiana.edu/.bin/fbidq.html?Rph), [srpk79D](http://flybase.bio.indiana.edu/.bin/fbidq.html?srpk79D), [GRHRII](http://flybase.bio.indiana.edu/.bin/fbidq.html?GRHRII), [Rdl](http://flybase.bio.indiana.edu/.bin/fbidq.html?Rdl), [CG10362](http://flybase.bio.indiana.edu/.bin/fbidq.html?CG10362), [GABA-B-R2](http://flybase.bio.indiana.edu/.bin/fbidq.html?GABA-B-R2), [Ast-C](http://flybase.bio.indiana.edu/.bin/fbidq.html?Ast-C), [Gbeta5](http://flybase.bio.indiana.edu/.bin/fbidq.html?Gbeta5), [cindr](http://flybase.bio.indiana.edu/.bin/fbidq.html?cindr), [futsch](http://flybase.bio.indiana.edu/.bin/fbidq.html?futsch), [Rim](http://flybase.bio.indiana.edu/.bin/fbidq.html?Rim), [Shal](http://flybase.bio.indiana.edu/.bin/fbidq.html?Shal),[hug](http://flybase.bio.indiana.edu/.bin/fbidq.html?hug), [para](http://flybase.bio.indiana.edu/.bin/fbidq.html?para), [Sur-8](http://flybase.bio.indiana.edu/.bin/fbidq.html?Sur-8), [CG11597](http://flybase.bio.indiana.edu/.bin/fbidq.html?CG11597), [Syn2](http://flybase.bio.indiana.edu/.bin/fbidq.html?Syn2), [Rgl](http://flybase.bio.indiana.edu/.bin/fbidq.html?Rgl), [e(y)3](http://flybase.bio.indiana.edu/.bin/fbidq.html?e(y)3), [Nrx-1](http://flybase.bio.indiana.edu/.bin/fbidq.html?Nrx-1), [CRMP](http://flybase.bio.indiana.edu/.bin/fbidq.html?CRMP), [Cdk5](http://flybase.bio.indiana.edu/.bin/fbidq.html?Cdk5), [Cep135](http://flybase.bio.indiana.edu/.bin/fbidq.html?Cep135), [mav](http://flybase.bio.indiana.edu/.bin/fbidq.html?mav), [CG33203](http://flybase.bio.indiana.edu/.bin/fbidq.html?CG33203), [CG4641](http://flybase.bio.indiana.edu/.bin/fbidq.html?CG4641), [Ilp7](http://flybase.bio.indiana.edu/.bin/fbidq.html?Ilp7), [ey](http://flybase.bio.indiana.edu/.bin/fbidq.html?ey), [CG31122](http://flybase.bio.indiana.edu/.bin/fbidq.html?CG31122), [CG15439](http://flybase.bio.indiana.edu/.bin/fbidq.html?CG15439), [Mip](http://flybase.bio.indiana.edu/.bin/fbidq.html?Mip), [baz](http://flybase.bio.indiana.edu/.bin/fbidq.html?baz), [CG33275](http://flybase.bio.indiana.edu/.bin/fbidq.html?CG33275), [Oamb](http://flybase.bio.indiana.edu/.bin/fbidq.html?Oamb), [CG34381](http://flybase.bio.indiana.edu/.bin/fbidq.html?CG34381), [rst](http://flybase.bio.indiana.edu/.bin/fbidq.html?rst), [Cdk5alpha](http://flybase.bio.indiana.edu/.bin/fbidq.html?Cdk5alpha), [CG40351](http://flybase.bio.indiana.edu/.bin/fbidq.html?CG40351), [Vmat](http://flybase.bio.indiana.edu/.bin/fbidq.html?Vmat), [qtc](http://flybase.bio.indiana.edu/.bin/fbidq.html?qtc), [Cha](http://flybase.bio.indiana.edu/.bin/fbidq.html?Cha), [crol](http://flybase.bio.indiana.edu/.bin/fbidq.html?crol), [sd](http://flybase.bio.indiana.edu/.bin/fbidq.html?sd), [Aplip1](http://flybase.bio.indiana.edu/.bin/fbidq.html?Aplip1), [Ac3](http://flybase.bio.indiana.edu/.bin/fbidq.html?Ac3), [rl](http://flybase.bio.indiana.edu/.bin/fbidq.html?rl),[Syt1](http://flybase.bio.indiana.edu/.bin/fbidq.html?Syt1), [CG17760](http://flybase.bio.indiana.edu/.bin/fbidq.html?CG17760), [Nup153](http://flybase.bio.indiana.edu/.bin/fbidq.html?Nup153), [CG32683](http://flybase.bio.indiana.edu/.bin/fbidq.html?CG32683), [CG11347](http://flybase.bio.indiana.edu/.bin/fbidq.html?CG11347), [Ggamma30A](http://flybase.bio.indiana.edu/.bin/fbidq.html?Ggamma30A), [wls](http://flybase.bio.indiana.edu/.bin/fbidq.html?wls), [hiw](http://flybase.bio.indiana.edu/.bin/fbidq.html?hiw), [Atf-2](http://flybase.bio.indiana.edu/.bin/fbidq.html?Atf-2), [n-syb](http://flybase.bio.indiana.edu/.bin/fbidq.html?n-syb), [skd](http://flybase.bio.indiana.edu/.bin/fbidq.html?skd), [Camta](http://flybase.bio.indiana.edu/.bin/fbidq.html?Camta), [tll](http://flybase.bio.indiana.edu/.bin/fbidq.html?tll), [ato](http://flybase.bio.indiana.edu/.bin/fbidq.html?ato), [Fmrf](http://flybase.bio.indiana.edu/.bin/fbidq.html?Fmrf), [Iswi](http://flybase.bio.indiana.edu/.bin/fbidq.html?Iswi), [Dg](http://flybase.bio.indiana.edu/.bin/fbidq.html?Dg), [Trim9](http://flybase.bio.indiana.edu/.bin/fbidq.html?Trim9), [GABA-B-R3](http://flybase.bio.indiana.edu/.bin/fbidq.html?GABA-B-R3), [Pka-R1](http://flybase.bio.indiana.edu/.bin/fbidq.html?Pka-R1), [Evi5](http://flybase.bio.indiana.edu/.bin/fbidq.html?Evi5), [mago](http://flybase.bio.indiana.edu/.bin/fbidq.html?mago), [Gycalpha99B](http://flybase.bio.indiana.edu/.bin/fbidq.html?Gycalpha99B), [ksr](http://flybase.bio.indiana.edu/.bin/fbidq.html?ksr), [CG42533](http://flybase.bio.indiana.edu/.bin/fbidq.html?CG42533), [CG11376](http://flybase.bio.indiana.edu/.bin/fbidq.html?CG11376), [Gyc-89Da](http://flybase.bio.indiana.edu/.bin/fbidq.html?Gyc-89Da), [brp](http://flybase.bio.indiana.edu/.bin/fbidq.html?brp), [unc-104](http://flybase.bio.indiana.edu/.bin/fbidq.html?unc-104), [Khc-73](http://flybase.bio.indiana.edu/.bin/fbidq.html?Khc-73), [D2R](http://flybase.bio.indiana.edu/.bin/fbidq.html?D2R),[CG31158](http://flybase.bio.indiana.edu/.bin/fbidq.html?CG31158), [metro](http://flybase.bio.indiana.edu/.bin/fbidq.html?metro), [ewg](http://flybase.bio.indiana.edu/.bin/fbidq.html?ewg), [Nrk](http://flybase.bio.indiana.edu/.bin/fbidq.html?Nrk), [Cirl](http://flybase.bio.indiana.edu/.bin/fbidq.html?Cirl), [Ilp3](http://flybase.bio.indiana.edu/.bin/fbidq.html?Ilp3), [Su(var)3-3](http://flybase.bio.indiana.edu/.bin/fbidq.html?Su(var)3-3), [Bili](http://flybase.bio.indiana.edu/.bin/fbidq.html?Bili), [Nrg](http://flybase.bio.indiana.edu/.bin/fbidq.html?Nrg), [unc-13-4A](http://flybase.bio.indiana.edu/.bin/fbidq.html?unc-13-4A), [CG34384](http://flybase.bio.indiana.edu/.bin/fbidq.html?CG34384), [stet](http://flybase.bio.indiana.edu/.bin/fbidq.html?stet), [Dh44-R1](http://flybase.bio.indiana.edu/.bin/fbidq.html?Dh44-R1), [CG32447](http://flybase.bio.indiana.edu/.bin/fbidq.html?CG32447), [Eh](http://flybase.bio.indiana.edu/.bin/fbidq.html?Eh), [Sema-1a](http://flybase.bio.indiana.edu/.bin/fbidq.html?Sema-1a), [Hs3st-B](http://flybase.bio.indiana.edu/.bin/fbidq.html?Hs3st-B), [otk](http://flybase.bio.indiana.edu/.bin/fbidq.html?otk), [Sos](http://flybase.bio.indiana.edu/.bin/fbidq.html?Sos), [phyl](http://flybase.bio.indiana.edu/.bin/fbidq.html?phyl), [vg](http://flybase.bio.indiana.edu/.bin/fbidq.html?vg), [Fas2](http://flybase.bio.indiana.edu/.bin/fbidq.html?Fas2), [fz](http://flybase.bio.indiana.edu/.bin/fbidq.html?fz), [robo](http://flybase.bio.indiana.edu/.bin/fbidq.html?robo), [CG8557](http://flybase.bio.indiana.edu/.bin/fbidq.html?CG8557), [bsk](http://flybase.bio.indiana.edu/.bin/fbidq.html?bsk), [brm](http://flybase.bio.indiana.edu/.bin/fbidq.html?brm), [mAcR-60C](http://flybase.bio.indiana.edu/.bin/fbidq.html?mAcR-60C), [Crz](http://flybase.bio.indiana.edu/.bin/fbidq.html?Crz), [ft](http://flybase.bio.indiana.edu/.bin/fbidq.html?ft), [mtg](http://flybase.bio.indiana.edu/.bin/fbidq.html?mtg), [CG13830](http://flybase.bio.indiana.edu/.bin/fbidq.html?CG13830), [sno](http://flybase.bio.indiana.edu/.bin/fbidq.html?sno), [RhoGAPp190](http://flybase.bio.indiana.edu/.bin/fbidq.html?RhoGAPp190),[l(2)k16918](http://flybase.bio.indiana.edu/.bin/fbidq.html?l(2)k16918), [mam](http://flybase.bio.indiana.edu/.bin/fbidq.html?mam), [CG30158](http://flybase.bio.indiana.edu/.bin/fbidq.html?CG30158), [Takr86C](http://flybase.bio.indiana.edu/.bin/fbidq.html?Takr86C), [pan](http://flybase.bio.indiana.edu/.bin/fbidq.html?pan), [PKD](http://flybase.bio.indiana.edu/.bin/fbidq.html?PKD), [DopR](http://flybase.bio.indiana.edu/.bin/fbidq.html?DopR), [Lis-1](http://flybase.bio.indiana.edu/.bin/fbidq.html?Lis-1), [CG16896](http://flybase.bio.indiana.edu/.bin/fbidq.html?CG16896), [Rab3](http://flybase.bio.indiana.edu/.bin/fbidq.html?Rab3), [tup](http://flybase.bio.indiana.edu/.bin/fbidq.html?tup), [mthl8](http://flybase.bio.indiana.edu/.bin/fbidq.html?mthl8), [trio](http://flybase.bio.indiana.edu/.bin/fbidq.html?trio), [RabX4](http://flybase.bio.indiana.edu/.bin/fbidq.html?RabX4), [spel1](http://flybase.bio.indiana.edu/.bin/fbidq.html?spel1), [Lkr](http://flybase.bio.indiana.edu/.bin/fbidq.html?Lkr), [CG31665](http://flybase.bio.indiana.edu/.bin/fbidq.html?CG31665), [Ccap](http://flybase.bio.indiana.edu/.bin/fbidq.html?Ccap), [Gad1](http://flybase.bio.indiana.edu/.bin/fbidq.html?Gad1), [Wnt4](http://flybase.bio.indiana.edu/.bin/fbidq.html?Wnt4), [CG7918](http://flybase.bio.indiana.edu/.bin/fbidq.html?CG7918), [CaMKII](http://flybase.bio.indiana.edu/.bin/fbidq.html?CaMKII), [DopR2](http://flybase.bio.indiana.edu/.bin/fbidq.html?DopR2), [CG30372](http://flybase.bio.indiana.edu/.bin/fbidq.html?CG30372), [CG10188](http://flybase.bio.indiana.edu/.bin/fbidq.html?CG10188), [CG42629](http://flybase.bio.indiana.edu/.bin/fbidq.html?CG42629), [pigs](http://flybase.bio.indiana.edu/.bin/fbidq.html?pigs), [CG31760](http://flybase.bio.indiana.edu/.bin/fbidq.html?CG31760), [Klp54D](http://flybase.bio.indiana.edu/.bin/fbidq.html?Klp54D), [bchs](http://flybase.bio.indiana.edu/.bin/fbidq.html?bchs), [5-HT7](http://flybase.bio.indiana.edu/.bin/fbidq.html?5-HT7), [Lgr3](http://flybase.bio.indiana.edu/.bin/fbidq.html?Lgr3), [Dh](http://flybase.bio.indiana.edu/.bin/fbidq.html?Dh), [Syn1](http://flybase.bio.indiana.edu/.bin/fbidq.html?Syn1), [Ac13E](http://flybase.bio.indiana.edu/.bin/fbidq.html?Ac13E), [klu](http://flybase.bio.indiana.edu/.bin/fbidq.html?klu), [SPR](http://flybase.bio.indiana.edu/.bin/fbidq.html?SPR), [CG8155](http://flybase.bio.indiana.edu/.bin/fbidq.html?CG8155), [sr](http://flybase.bio.indiana.edu/.bin/fbidq.html?sr), [nkd](http://flybase.bio.indiana.edu/.bin/fbidq.html?nkd), [tld](http://flybase.bio.indiana.edu/.bin/fbidq.html?tld), [RhoGEF3](http://flybase.bio.indiana.edu/.bin/fbidq.html?RhoGEF3), [Dsk](http://flybase.bio.indiana.edu/.bin/fbidq.html?Dsk), [RhoGEF4](http://flybase.bio.indiana.edu/.bin/fbidq.html?RhoGEF4), [CG9121](http://flybase.bio.indiana.edu/.bin/fbidq.html?CG9121), [betaInt-nu](http://flybase.bio.indiana.edu/.bin/fbidq.html?betaInt-nu), [CG1909](http://flybase.bio.indiana.edu/.bin/fbidq.html?CG1909), [Brd](http://flybase.bio.indiana.edu/.bin/fbidq.html?Brd), [sgg](http://flybase.bio.indiana.edu/.bin/fbidq.html?sgg), [Nmdar1](http://flybase.bio.indiana.edu/.bin/fbidq.html?Nmdar1), [Eip93F](http://flybase.bio.indiana.edu/.bin/fbidq.html?Eip93F), [inaE](http://flybase.bio.indiana.edu/.bin/fbidq.html?inaE), [CanB](http://flybase.bio.indiana.edu/.bin/fbidq.html?CanB), [Pask](http://flybase.bio.indiana.edu/.bin/fbidq.html?Pask), [rab3-GEF](http://flybase.bio.indiana.edu/.bin/fbidq.html?rab3-GEF), [CG13253](http://flybase.bio.indiana.edu/.bin/fbidq.html?CG13253), [MESK2](http://flybase.bio.indiana.edu/.bin/fbidq.html?MESK2), [Syt4](http://flybase.bio.indiana.edu/.bin/fbidq.html?Syt4), [Dat](http://flybase.bio.indiana.edu/.bin/fbidq.html?Dat), [SIFR](http://flybase.bio.indiana.edu/.bin/fbidq.html?SIFR), [CG2061](http://flybase.bio.indiana.edu/.bin/fbidq.html?CG2061), [Ast](http://flybase.bio.indiana.edu/.bin/fbidq.html?Ast), [fred](http://flybase.bio.indiana.edu/.bin/fbidq.html?fred), [Drl-2](http://flybase.bio.indiana.edu/.bin/fbidq.html?Drl-2),[ush](http://flybase.bio.indiana.edu/.bin/fbidq.html?ush), [Tk](http://flybase.bio.indiana.edu/.bin/fbidq.html?Tk), [Fak56D](http://flybase.bio.indiana.edu/.bin/fbidq.html?Fak56D), [Ilp5](http://flybase.bio.indiana.edu/.bin/fbidq.html?Ilp5), [Gap1](http://flybase.bio.indiana.edu/.bin/fbidq.html?Gap1), [Toll-6](http://flybase.bio.indiana.edu/.bin/fbidq.html?Toll-6), [CG3227](http://flybase.bio.indiana.edu/.bin/fbidq.html?CG3227), [MESR4](http://flybase.bio.indiana.edu/.bin/fbidq.html?MESR4), [Takr99D](http://flybase.bio.indiana.edu/.bin/fbidq.html?Takr99D), [sNPF-R](http://flybase.bio.indiana.edu/.bin/fbidq.html?sNPF-R), [tara](http://flybase.bio.indiana.edu/.bin/fbidq.html?tara), [Hel89B](http://flybase.bio.indiana.edu/.bin/fbidq.html?Hel89B), [CG5337](http://flybase.bio.indiana.edu/.bin/fbidq.html?CG5337), [CG13692](http://flybase.bio.indiana.edu/.bin/fbidq.html?CG13692), [koko](http://flybase.bio.indiana.edu/.bin/fbidq.html?koko), [stan](http://flybase.bio.indiana.edu/.bin/fbidq.html?stan), [CG12424](http://flybase.bio.indiana.edu/.bin/fbidq.html?CG12424), [tutl](http://flybase.bio.indiana.edu/.bin/fbidq.html?tutl), [Ptth](http://flybase.bio.indiana.edu/.bin/fbidq.html?Ptth), [Rab2](http://flybase.bio.indiana.edu/.bin/fbidq.html?Rab2), [Ilp2](http://flybase.bio.indiana.edu/.bin/fbidq.html?Ilp2), [Frq1](http://flybase.bio.indiana.edu/.bin/fbidq.html?Frq1), [SPoCk](http://flybase.bio.indiana.edu/.bin/fbidq.html?SPoCk), [sna](http://flybase.bio.indiana.edu/.bin/fbidq.html?sna), [Wnt5](http://flybase.bio.indiana.edu/.bin/fbidq.html?Wnt5), [Taf1](http://flybase.bio.indiana.edu/.bin/fbidq.html?Taf1), [Ggamma1](http://flybase.bio.indiana.edu/.bin/fbidq.html?Ggamma1), [fus](http://flybase.bio.indiana.edu/.bin/fbidq.html?fus), [sif](http://flybase.bio.indiana.edu/.bin/fbidq.html?sif), [RSG7](http://flybase.bio.indiana.edu/.bin/fbidq.html?RSG7), [m4](http://flybase.bio.indiana.edu/.bin/fbidq.html?m4), [dom](http://flybase.bio.indiana.edu/.bin/fbidq.html?dom), [Tbh](http://flybase.bio.indiana.edu/.bin/fbidq.html?Tbh), [Dh31](http://flybase.bio.indiana.edu/.bin/fbidq.html?Dh31),[CG42541](http://flybase.bio.indiana.edu/.bin/fbidq.html?CG42541), [PIP5K59B](http://flybase.bio.indiana.edu/.bin/fbidq.html?PIP5K59B), [Hr51](http://flybase.bio.indiana.edu/.bin/fbidq.html?Hr51), [lap](http://flybase.bio.indiana.edu/.bin/fbidq.html?lap), [shi](http://flybase.bio.indiana.edu/.bin/fbidq.html?shi), [CG11155](http://flybase.bio.indiana.edu/.bin/fbidq.html?CG11155), [Spred](http://flybase.bio.indiana.edu/.bin/fbidq.html?Spred), [siz](http://flybase.bio.indiana.edu/.bin/fbidq.html?siz), [B-H2](http://flybase.bio.indiana.edu/.bin/fbidq.html?B-H2), [CG3822](http://flybase.bio.indiana.edu/.bin/fbidq.html?CG3822), [wg](http://flybase.bio.indiana.edu/.bin/fbidq.html?wg), [Rhp](http://flybase.bio.indiana.edu/.bin/fbidq.html?Rhp), [CG13995](http://flybase.bio.indiana.edu/.bin/fbidq.html?CG13995), [E(bx)](http://flybase.bio.indiana.edu/.bin/fbidq.html?E(bx)), [sfl](http://flybase.bio.indiana.edu/.bin/fbidq.html?sfl), [CG13229](http://flybase.bio.indiana.edu/.bin/fbidq.html?CG13229), [Pde11](http://flybase.bio.indiana.edu/.bin/fbidq.html?Pde11), [Dms](http://flybase.bio.indiana.edu/.bin/fbidq.html?Dms), [Snap](http://flybase.bio.indiana.edu/.bin/fbidq.html?Snap), [SerT](http://flybase.bio.indiana.edu/.bin/fbidq.html?SerT), [Utx](http://flybase.bio.indiana.edu/.bin/fbidq.html?Utx), [pburs](http://flybase.bio.indiana.edu/.bin/fbidq.html?pburs), [cos](http://flybase.bio.indiana.edu/.bin/fbidq.html?cos), [Ct](http://flybase.bio.indiana.edu/.bin/fbidq.html?Ct) |
| [single organism signaling](http://amigo.geneontology.org/cgi-bin/amigo/go.cgi?view=details&query=GO:0044700) | 352 of 1594 genes, 22.1% | 1226 of 7634 genes, 16.1% | 4.10e-13 | 0.00% | 0.00 | [fz2](http://flybase.bio.indiana.edu/.bin/fbidq.html?fz2), [endoA](http://flybase.bio.indiana.edu/.bin/fbidq.html?endoA), [CG14375](http://flybase.bio.indiana.edu/.bin/fbidq.html?CG14375), [Ocho](http://flybase.bio.indiana.edu/.bin/fbidq.html?Ocho), [Sh](http://flybase.bio.indiana.edu/.bin/fbidq.html?Sh), [numb](http://flybase.bio.indiana.edu/.bin/fbidq.html?numb), [dock](http://flybase.bio.indiana.edu/.bin/fbidq.html?dock), [stj](http://flybase.bio.indiana.edu/.bin/fbidq.html?stj), [Sxl](http://flybase.bio.indiana.edu/.bin/fbidq.html?Sxl), [CG8500](http://flybase.bio.indiana.edu/.bin/fbidq.html?CG8500), [cenG1A](http://flybase.bio.indiana.edu/.bin/fbidq.html?cenG1A), [Nf1](http://flybase.bio.indiana.edu/.bin/fbidq.html?Nf1), [malpha](http://flybase.bio.indiana.edu/.bin/fbidq.html?malpha), [AR-2](http://flybase.bio.indiana.edu/.bin/fbidq.html?AR-2), [os](http://flybase.bio.indiana.edu/.bin/fbidq.html?os), [Axn](http://flybase.bio.indiana.edu/.bin/fbidq.html?Axn), [Sytalpha](http://flybase.bio.indiana.edu/.bin/fbidq.html?Sytalpha), [Traf6](http://flybase.bio.indiana.edu/.bin/fbidq.html?Traf6), [Leucokinin](http://flybase.bio.indiana.edu/.bin/fbidq.html?Leucokinin), [rdgC](http://flybase.bio.indiana.edu/.bin/fbidq.html?rdgC), [Galpha49B](http://flybase.bio.indiana.edu/.bin/fbidq.html?Galpha49B), [Snoo](http://flybase.bio.indiana.edu/.bin/fbidq.html?Snoo), [scrib](http://flybase.bio.indiana.edu/.bin/fbidq.html?scrib), [rut](http://flybase.bio.indiana.edu/.bin/fbidq.html?rut), [CG7650](http://flybase.bio.indiana.edu/.bin/fbidq.html?CG7650), [RhoGAP100F](http://flybase.bio.indiana.edu/.bin/fbidq.html?RhoGAP100F), [uif](http://flybase.bio.indiana.edu/.bin/fbidq.html?uif), [Mmp2](http://flybase.bio.indiana.edu/.bin/fbidq.html?Mmp2), [HLHmdelta](http://flybase.bio.indiana.edu/.bin/fbidq.html?HLHmdelta), [Nplp1](http://flybase.bio.indiana.edu/.bin/fbidq.html?Nplp1), [Nipped-A](http://flybase.bio.indiana.edu/.bin/fbidq.html?Nipped-A),[CG32206](http://flybase.bio.indiana.edu/.bin/fbidq.html?CG32206), [7B2](http://flybase.bio.indiana.edu/.bin/fbidq.html?7B2), [alph](http://flybase.bio.indiana.edu/.bin/fbidq.html?alph), [Traf4](http://flybase.bio.indiana.edu/.bin/fbidq.html?Traf4), [TBPH](http://flybase.bio.indiana.edu/.bin/fbidq.html?TBPH), [Snap25](http://flybase.bio.indiana.edu/.bin/fbidq.html?Snap25), [mGluRA](http://flybase.bio.indiana.edu/.bin/fbidq.html?mGluRA), [dsh](http://flybase.bio.indiana.edu/.bin/fbidq.html?dsh), [pzg](http://flybase.bio.indiana.edu/.bin/fbidq.html?pzg), [Nf-YC](http://flybase.bio.indiana.edu/.bin/fbidq.html?Nf-YC), [14-3-3zeta](http://flybase.bio.indiana.edu/.bin/fbidq.html?14-3-3zeta), [Pli](http://flybase.bio.indiana.edu/.bin/fbidq.html?Pli), [shakB](http://flybase.bio.indiana.edu/.bin/fbidq.html?shakB), [comt](http://flybase.bio.indiana.edu/.bin/fbidq.html?comt), [CG31140](http://flybase.bio.indiana.edu/.bin/fbidq.html?CG31140), [Hey](http://flybase.bio.indiana.edu/.bin/fbidq.html?Hey), [plexB](http://flybase.bio.indiana.edu/.bin/fbidq.html?plexB), [AlstR](http://flybase.bio.indiana.edu/.bin/fbidq.html?AlstR), [PP2A-B](http://flybase.bio.indiana.edu/.bin/fbidq.html?PP2A-B), [qvr](http://flybase.bio.indiana.edu/.bin/fbidq.html?qvr), [mars](http://flybase.bio.indiana.edu/.bin/fbidq.html?mars), [IM10](http://flybase.bio.indiana.edu/.bin/fbidq.html?IM10), [neur](http://flybase.bio.indiana.edu/.bin/fbidq.html?neur), [CycE](http://flybase.bio.indiana.edu/.bin/fbidq.html?CycE), [rho-5](http://flybase.bio.indiana.edu/.bin/fbidq.html?rho-5), [nmo](http://flybase.bio.indiana.edu/.bin/fbidq.html?nmo), [Tusp](http://flybase.bio.indiana.edu/.bin/fbidq.html?Tusp), [5-HT1A](http://flybase.bio.indiana.edu/.bin/fbidq.html?5-HT1A), [CG32149](http://flybase.bio.indiana.edu/.bin/fbidq.html?CG32149), [Vdup1](http://flybase.bio.indiana.edu/.bin/fbidq.html?Vdup1), [Gug](http://flybase.bio.indiana.edu/.bin/fbidq.html?Gug), [ed](http://flybase.bio.indiana.edu/.bin/fbidq.html?ed), [mr](http://flybase.bio.indiana.edu/.bin/fbidq.html?mr), [daw](http://flybase.bio.indiana.edu/.bin/fbidq.html?daw),[CG5036](http://flybase.bio.indiana.edu/.bin/fbidq.html?CG5036), [5-HT1B](http://flybase.bio.indiana.edu/.bin/fbidq.html?5-HT1B), [synaptogyrin](http://flybase.bio.indiana.edu/.bin/fbidq.html?synaptogyrin), [Apc](http://flybase.bio.indiana.edu/.bin/fbidq.html?Apc), [sim](http://flybase.bio.indiana.edu/.bin/fbidq.html?sim), [CG8795](http://flybase.bio.indiana.edu/.bin/fbidq.html?CG8795), [gro](http://flybase.bio.indiana.edu/.bin/fbidq.html?gro), [RN-tre](http://flybase.bio.indiana.edu/.bin/fbidq.html?RN-tre), [scyl](http://flybase.bio.indiana.edu/.bin/fbidq.html?scyl), [Btk29A](http://flybase.bio.indiana.edu/.bin/fbidq.html?Btk29A), [ETH](http://flybase.bio.indiana.edu/.bin/fbidq.html?ETH), [unc-5](http://flybase.bio.indiana.edu/.bin/fbidq.html?unc-5), [cdi](http://flybase.bio.indiana.edu/.bin/fbidq.html?cdi), [NPFR1](http://flybase.bio.indiana.edu/.bin/fbidq.html?NPFR1), [pygo](http://flybase.bio.indiana.edu/.bin/fbidq.html?pygo), [HLHmgamma](http://flybase.bio.indiana.edu/.bin/fbidq.html?HLHmgamma), [CG15556](http://flybase.bio.indiana.edu/.bin/fbidq.html?CG15556), [Ror](http://flybase.bio.indiana.edu/.bin/fbidq.html?Ror), [tow](http://flybase.bio.indiana.edu/.bin/fbidq.html?tow), [CG15609](http://flybase.bio.indiana.edu/.bin/fbidq.html?CG15609), [wor](http://flybase.bio.indiana.edu/.bin/fbidq.html?wor), [Atg1](http://flybase.bio.indiana.edu/.bin/fbidq.html?Atg1), [CG12187](http://flybase.bio.indiana.edu/.bin/fbidq.html?CG12187), [Pten](http://flybase.bio.indiana.edu/.bin/fbidq.html?Pten), [unc-13](http://flybase.bio.indiana.edu/.bin/fbidq.html?unc-13), [npf](http://flybase.bio.indiana.edu/.bin/fbidq.html?npf), [Ptp69D](http://flybase.bio.indiana.edu/.bin/fbidq.html?Ptp69D), [bowl](http://flybase.bio.indiana.edu/.bin/fbidq.html?bowl), [oa2](http://flybase.bio.indiana.edu/.bin/fbidq.html?oa2), [elk](http://flybase.bio.indiana.edu/.bin/fbidq.html?elk), [phl](http://flybase.bio.indiana.edu/.bin/fbidq.html?phl), [Cbl](http://flybase.bio.indiana.edu/.bin/fbidq.html?Cbl), [Rab26](http://flybase.bio.indiana.edu/.bin/fbidq.html?Rab26),[Pde6](http://flybase.bio.indiana.edu/.bin/fbidq.html?Pde6), [sNPF](http://flybase.bio.indiana.edu/.bin/fbidq.html?sNPF), [pog](http://flybase.bio.indiana.edu/.bin/fbidq.html?pog), [plx](http://flybase.bio.indiana.edu/.bin/fbidq.html?plx), [CASK](http://flybase.bio.indiana.edu/.bin/fbidq.html?CASK), [CG9098](http://flybase.bio.indiana.edu/.bin/fbidq.html?CG9098), [CG8108](http://flybase.bio.indiana.edu/.bin/fbidq.html?CG8108), [IFa](http://flybase.bio.indiana.edu/.bin/fbidq.html?IFa), [mtt](http://flybase.bio.indiana.edu/.bin/fbidq.html?mtt), [CG32758](http://flybase.bio.indiana.edu/.bin/fbidq.html?CG32758), [Alk](http://flybase.bio.indiana.edu/.bin/fbidq.html?Alk), [CG7708](http://flybase.bio.indiana.edu/.bin/fbidq.html?CG7708), [Pde1c](http://flybase.bio.indiana.edu/.bin/fbidq.html?Pde1c), [disp](http://flybase.bio.indiana.edu/.bin/fbidq.html?disp), [cpx](http://flybase.bio.indiana.edu/.bin/fbidq.html?cpx), [casp](http://flybase.bio.indiana.edu/.bin/fbidq.html?casp), [CG4022](http://flybase.bio.indiana.edu/.bin/fbidq.html?CG4022), [Smox](http://flybase.bio.indiana.edu/.bin/fbidq.html?Smox), [gfA](http://flybase.bio.indiana.edu/.bin/fbidq.html?gfA), [Plc21C](http://flybase.bio.indiana.edu/.bin/fbidq.html?Plc21C), [CG31781](http://flybase.bio.indiana.edu/.bin/fbidq.html?CG31781), [Rph](http://flybase.bio.indiana.edu/.bin/fbidq.html?Rph), [srpk79D](http://flybase.bio.indiana.edu/.bin/fbidq.html?srpk79D), [GRHRII](http://flybase.bio.indiana.edu/.bin/fbidq.html?GRHRII), [Rdl](http://flybase.bio.indiana.edu/.bin/fbidq.html?Rdl), [CG10362](http://flybase.bio.indiana.edu/.bin/fbidq.html?CG10362), [GABA-B-R2](http://flybase.bio.indiana.edu/.bin/fbidq.html?GABA-B-R2), [Ast-C](http://flybase.bio.indiana.edu/.bin/fbidq.html?Ast-C), [Gbeta5](http://flybase.bio.indiana.edu/.bin/fbidq.html?Gbeta5), [cindr](http://flybase.bio.indiana.edu/.bin/fbidq.html?cindr), [futsch](http://flybase.bio.indiana.edu/.bin/fbidq.html?futsch), [Rim](http://flybase.bio.indiana.edu/.bin/fbidq.html?Rim), [Shal](http://flybase.bio.indiana.edu/.bin/fbidq.html?Shal),[hug](http://flybase.bio.indiana.edu/.bin/fbidq.html?hug), [para](http://flybase.bio.indiana.edu/.bin/fbidq.html?para), [Sur-8](http://flybase.bio.indiana.edu/.bin/fbidq.html?Sur-8), [CG11597](http://flybase.bio.indiana.edu/.bin/fbidq.html?CG11597), [Syn2](http://flybase.bio.indiana.edu/.bin/fbidq.html?Syn2), [Rgl](http://flybase.bio.indiana.edu/.bin/fbidq.html?Rgl), [e(y)3](http://flybase.bio.indiana.edu/.bin/fbidq.html?e(y)3), [Nrx-1](http://flybase.bio.indiana.edu/.bin/fbidq.html?Nrx-1), [CRMP](http://flybase.bio.indiana.edu/.bin/fbidq.html?CRMP), [Cdk5](http://flybase.bio.indiana.edu/.bin/fbidq.html?Cdk5), [Cep135](http://flybase.bio.indiana.edu/.bin/fbidq.html?Cep135), [mav](http://flybase.bio.indiana.edu/.bin/fbidq.html?mav), [CG33203](http://flybase.bio.indiana.edu/.bin/fbidq.html?CG33203), [CG4641](http://flybase.bio.indiana.edu/.bin/fbidq.html?CG4641), [Ilp7](http://flybase.bio.indiana.edu/.bin/fbidq.html?Ilp7), [ey](http://flybase.bio.indiana.edu/.bin/fbidq.html?ey), [CG31122](http://flybase.bio.indiana.edu/.bin/fbidq.html?CG31122), [CG15439](http://flybase.bio.indiana.edu/.bin/fbidq.html?CG15439), [Mip](http://flybase.bio.indiana.edu/.bin/fbidq.html?Mip), [baz](http://flybase.bio.indiana.edu/.bin/fbidq.html?baz), [CG33275](http://flybase.bio.indiana.edu/.bin/fbidq.html?CG33275), [Oamb](http://flybase.bio.indiana.edu/.bin/fbidq.html?Oamb), [CG34381](http://flybase.bio.indiana.edu/.bin/fbidq.html?CG34381), [rst](http://flybase.bio.indiana.edu/.bin/fbidq.html?rst), [Cdk5alpha](http://flybase.bio.indiana.edu/.bin/fbidq.html?Cdk5alpha), [CG40351](http://flybase.bio.indiana.edu/.bin/fbidq.html?CG40351), [Vmat](http://flybase.bio.indiana.edu/.bin/fbidq.html?Vmat), [qtc](http://flybase.bio.indiana.edu/.bin/fbidq.html?qtc), [Cha](http://flybase.bio.indiana.edu/.bin/fbidq.html?Cha), [crol](http://flybase.bio.indiana.edu/.bin/fbidq.html?crol), [sd](http://flybase.bio.indiana.edu/.bin/fbidq.html?sd), [Aplip1](http://flybase.bio.indiana.edu/.bin/fbidq.html?Aplip1), [Ac3](http://flybase.bio.indiana.edu/.bin/fbidq.html?Ac3), [rl](http://flybase.bio.indiana.edu/.bin/fbidq.html?rl),[Syt1](http://flybase.bio.indiana.edu/.bin/fbidq.html?Syt1), [CG17760](http://flybase.bio.indiana.edu/.bin/fbidq.html?CG17760), [Nup153](http://flybase.bio.indiana.edu/.bin/fbidq.html?Nup153), [CG32683](http://flybase.bio.indiana.edu/.bin/fbidq.html?CG32683), [CG11347](http://flybase.bio.indiana.edu/.bin/fbidq.html?CG11347), [Ggamma30A](http://flybase.bio.indiana.edu/.bin/fbidq.html?Ggamma30A), [wls](http://flybase.bio.indiana.edu/.bin/fbidq.html?wls), [hiw](http://flybase.bio.indiana.edu/.bin/fbidq.html?hiw), [Atf-2](http://flybase.bio.indiana.edu/.bin/fbidq.html?Atf-2), [n-syb](http://flybase.bio.indiana.edu/.bin/fbidq.html?n-syb), [skd](http://flybase.bio.indiana.edu/.bin/fbidq.html?skd), [Camta](http://flybase.bio.indiana.edu/.bin/fbidq.html?Camta), [tll](http://flybase.bio.indiana.edu/.bin/fbidq.html?tll), [ato](http://flybase.bio.indiana.edu/.bin/fbidq.html?ato), [Fmrf](http://flybase.bio.indiana.edu/.bin/fbidq.html?Fmrf), [Iswi](http://flybase.bio.indiana.edu/.bin/fbidq.html?Iswi), [Dg](http://flybase.bio.indiana.edu/.bin/fbidq.html?Dg), [Trim9](http://flybase.bio.indiana.edu/.bin/fbidq.html?Trim9), [GABA-B-R3](http://flybase.bio.indiana.edu/.bin/fbidq.html?GABA-B-R3), [Pka-R1](http://flybase.bio.indiana.edu/.bin/fbidq.html?Pka-R1), [Evi5](http://flybase.bio.indiana.edu/.bin/fbidq.html?Evi5), [mago](http://flybase.bio.indiana.edu/.bin/fbidq.html?mago), [Gycalpha99B](http://flybase.bio.indiana.edu/.bin/fbidq.html?Gycalpha99B), [ksr](http://flybase.bio.indiana.edu/.bin/fbidq.html?ksr), [CG42533](http://flybase.bio.indiana.edu/.bin/fbidq.html?CG42533), [CG11376](http://flybase.bio.indiana.edu/.bin/fbidq.html?CG11376), [Gyc-89Da](http://flybase.bio.indiana.edu/.bin/fbidq.html?Gyc-89Da), [brp](http://flybase.bio.indiana.edu/.bin/fbidq.html?brp), [unc-104](http://flybase.bio.indiana.edu/.bin/fbidq.html?unc-104), [Khc-73](http://flybase.bio.indiana.edu/.bin/fbidq.html?Khc-73), [D2R](http://flybase.bio.indiana.edu/.bin/fbidq.html?D2R),[CG31158](http://flybase.bio.indiana.edu/.bin/fbidq.html?CG31158), [metro](http://flybase.bio.indiana.edu/.bin/fbidq.html?metro), [ewg](http://flybase.bio.indiana.edu/.bin/fbidq.html?ewg), [Nrk](http://flybase.bio.indiana.edu/.bin/fbidq.html?Nrk), [Cirl](http://flybase.bio.indiana.edu/.bin/fbidq.html?Cirl), [Ilp3](http://flybase.bio.indiana.edu/.bin/fbidq.html?Ilp3), [Su(var)3-3](http://flybase.bio.indiana.edu/.bin/fbidq.html?Su(var)3-3), [Bili](http://flybase.bio.indiana.edu/.bin/fbidq.html?Bili), [Nrg](http://flybase.bio.indiana.edu/.bin/fbidq.html?Nrg), [unc-13-4A](http://flybase.bio.indiana.edu/.bin/fbidq.html?unc-13-4A), [CG34384](http://flybase.bio.indiana.edu/.bin/fbidq.html?CG34384), [stet](http://flybase.bio.indiana.edu/.bin/fbidq.html?stet), [Dh44-R1](http://flybase.bio.indiana.edu/.bin/fbidq.html?Dh44-R1), [CG32447](http://flybase.bio.indiana.edu/.bin/fbidq.html?CG32447), [Eh](http://flybase.bio.indiana.edu/.bin/fbidq.html?Eh), [Sema-1a](http://flybase.bio.indiana.edu/.bin/fbidq.html?Sema-1a), [Hs3st-B](http://flybase.bio.indiana.edu/.bin/fbidq.html?Hs3st-B), [otk](http://flybase.bio.indiana.edu/.bin/fbidq.html?otk), [Sos](http://flybase.bio.indiana.edu/.bin/fbidq.html?Sos), [phyl](http://flybase.bio.indiana.edu/.bin/fbidq.html?phyl), [vg](http://flybase.bio.indiana.edu/.bin/fbidq.html?vg), [Fas2](http://flybase.bio.indiana.edu/.bin/fbidq.html?Fas2), [fz](http://flybase.bio.indiana.edu/.bin/fbidq.html?fz), [robo](http://flybase.bio.indiana.edu/.bin/fbidq.html?robo), [CG8557](http://flybase.bio.indiana.edu/.bin/fbidq.html?CG8557), [bsk](http://flybase.bio.indiana.edu/.bin/fbidq.html?bsk), [brm](http://flybase.bio.indiana.edu/.bin/fbidq.html?brm), [mAcR-60C](http://flybase.bio.indiana.edu/.bin/fbidq.html?mAcR-60C), [Crz](http://flybase.bio.indiana.edu/.bin/fbidq.html?Crz), [ft](http://flybase.bio.indiana.edu/.bin/fbidq.html?ft), [mtg](http://flybase.bio.indiana.edu/.bin/fbidq.html?mtg), [CG13830](http://flybase.bio.indiana.edu/.bin/fbidq.html?CG13830), [sno](http://flybase.bio.indiana.edu/.bin/fbidq.html?sno), [RhoGAPp190](http://flybase.bio.indiana.edu/.bin/fbidq.html?RhoGAPp190),[l(2)k16918](http://flybase.bio.indiana.edu/.bin/fbidq.html?l(2)k16918), [mam](http://flybase.bio.indiana.edu/.bin/fbidq.html?mam), [CG30158](http://flybase.bio.indiana.edu/.bin/fbidq.html?CG30158), [Takr86C](http://flybase.bio.indiana.edu/.bin/fbidq.html?Takr86C), [pan](http://flybase.bio.indiana.edu/.bin/fbidq.html?pan), [PKD](http://flybase.bio.indiana.edu/.bin/fbidq.html?PKD), [DopR](http://flybase.bio.indiana.edu/.bin/fbidq.html?DopR), [Lis-1](http://flybase.bio.indiana.edu/.bin/fbidq.html?Lis-1), [CG16896](http://flybase.bio.indiana.edu/.bin/fbidq.html?CG16896), [Rab3](http://flybase.bio.indiana.edu/.bin/fbidq.html?Rab3), [tup](http://flybase.bio.indiana.edu/.bin/fbidq.html?tup), [mthl8](http://flybase.bio.indiana.edu/.bin/fbidq.html?mthl8), [trio](http://flybase.bio.indiana.edu/.bin/fbidq.html?trio), [RabX4](http://flybase.bio.indiana.edu/.bin/fbidq.html?RabX4), [spel1](http://flybase.bio.indiana.edu/.bin/fbidq.html?spel1), [Lkr](http://flybase.bio.indiana.edu/.bin/fbidq.html?Lkr), [CG31665](http://flybase.bio.indiana.edu/.bin/fbidq.html?CG31665), [Ccap](http://flybase.bio.indiana.edu/.bin/fbidq.html?Ccap), [Gad1](http://flybase.bio.indiana.edu/.bin/fbidq.html?Gad1), [Wnt4](http://flybase.bio.indiana.edu/.bin/fbidq.html?Wnt4), [CG7918](http://flybase.bio.indiana.edu/.bin/fbidq.html?CG7918), [CaMKII](http://flybase.bio.indiana.edu/.bin/fbidq.html?CaMKII), [DopR2](http://flybase.bio.indiana.edu/.bin/fbidq.html?DopR2), [CG30372](http://flybase.bio.indiana.edu/.bin/fbidq.html?CG30372), [CG10188](http://flybase.bio.indiana.edu/.bin/fbidq.html?CG10188), [CG42629](http://flybase.bio.indiana.edu/.bin/fbidq.html?CG42629), [pigs](http://flybase.bio.indiana.edu/.bin/fbidq.html?pigs), [CG31760](http://flybase.bio.indiana.edu/.bin/fbidq.html?CG31760), [Klp54D](http://flybase.bio.indiana.edu/.bin/fbidq.html?Klp54D), [bchs](http://flybase.bio.indiana.edu/.bin/fbidq.html?bchs), [5-HT7](http://flybase.bio.indiana.edu/.bin/fbidq.html?5-HT7), [Lgr3](http://flybase.bio.indiana.edu/.bin/fbidq.html?Lgr3), [Dh](http://flybase.bio.indiana.edu/.bin/fbidq.html?Dh), [Syn1](http://flybase.bio.indiana.edu/.bin/fbidq.html?Syn1), [Ac13E](http://flybase.bio.indiana.edu/.bin/fbidq.html?Ac13E), [klu](http://flybase.bio.indiana.edu/.bin/fbidq.html?klu), [SPR](http://flybase.bio.indiana.edu/.bin/fbidq.html?SPR), [CG8155](http://flybase.bio.indiana.edu/.bin/fbidq.html?CG8155), [sr](http://flybase.bio.indiana.edu/.bin/fbidq.html?sr), [nkd](http://flybase.bio.indiana.edu/.bin/fbidq.html?nkd), [tld](http://flybase.bio.indiana.edu/.bin/fbidq.html?tld), [RhoGEF3](http://flybase.bio.indiana.edu/.bin/fbidq.html?RhoGEF3), [Dsk](http://flybase.bio.indiana.edu/.bin/fbidq.html?Dsk), [RhoGEF4](http://flybase.bio.indiana.edu/.bin/fbidq.html?RhoGEF4), [CG9121](http://flybase.bio.indiana.edu/.bin/fbidq.html?CG9121), [betaInt-nu](http://flybase.bio.indiana.edu/.bin/fbidq.html?betaInt-nu), [CG1909](http://flybase.bio.indiana.edu/.bin/fbidq.html?CG1909), [Brd](http://flybase.bio.indiana.edu/.bin/fbidq.html?Brd), [sgg](http://flybase.bio.indiana.edu/.bin/fbidq.html?sgg), [Nmdar1](http://flybase.bio.indiana.edu/.bin/fbidq.html?Nmdar1), [Eip93F](http://flybase.bio.indiana.edu/.bin/fbidq.html?Eip93F), [inaE](http://flybase.bio.indiana.edu/.bin/fbidq.html?inaE), [CanB](http://flybase.bio.indiana.edu/.bin/fbidq.html?CanB), [Pask](http://flybase.bio.indiana.edu/.bin/fbidq.html?Pask), [rab3-GEF](http://flybase.bio.indiana.edu/.bin/fbidq.html?rab3-GEF), [CG13253](http://flybase.bio.indiana.edu/.bin/fbidq.html?CG13253), [MESK2](http://flybase.bio.indiana.edu/.bin/fbidq.html?MESK2), [Syt4](http://flybase.bio.indiana.edu/.bin/fbidq.html?Syt4), [Dat](http://flybase.bio.indiana.edu/.bin/fbidq.html?Dat), [SIFR](http://flybase.bio.indiana.edu/.bin/fbidq.html?SIFR), [CG2061](http://flybase.bio.indiana.edu/.bin/fbidq.html?CG2061), [Ast](http://flybase.bio.indiana.edu/.bin/fbidq.html?Ast), [fred](http://flybase.bio.indiana.edu/.bin/fbidq.html?fred), [Drl-2](http://flybase.bio.indiana.edu/.bin/fbidq.html?Drl-2),[ush](http://flybase.bio.indiana.edu/.bin/fbidq.html?ush), [Tk](http://flybase.bio.indiana.edu/.bin/fbidq.html?Tk), [Fak56D](http://flybase.bio.indiana.edu/.bin/fbidq.html?Fak56D), [Ilp5](http://flybase.bio.indiana.edu/.bin/fbidq.html?Ilp5), [Gap1](http://flybase.bio.indiana.edu/.bin/fbidq.html?Gap1), [Toll-6](http://flybase.bio.indiana.edu/.bin/fbidq.html?Toll-6), [CG3227](http://flybase.bio.indiana.edu/.bin/fbidq.html?CG3227), [MESR4](http://flybase.bio.indiana.edu/.bin/fbidq.html?MESR4), [Takr99D](http://flybase.bio.indiana.edu/.bin/fbidq.html?Takr99D), [sNPF-R](http://flybase.bio.indiana.edu/.bin/fbidq.html?sNPF-R), [tara](http://flybase.bio.indiana.edu/.bin/fbidq.html?tara), [Hel89B](http://flybase.bio.indiana.edu/.bin/fbidq.html?Hel89B), [CG5337](http://flybase.bio.indiana.edu/.bin/fbidq.html?CG5337), [CG13692](http://flybase.bio.indiana.edu/.bin/fbidq.html?CG13692), [koko](http://flybase.bio.indiana.edu/.bin/fbidq.html?koko), [stan](http://flybase.bio.indiana.edu/.bin/fbidq.html?stan), [CG12424](http://flybase.bio.indiana.edu/.bin/fbidq.html?CG12424), [tutl](http://flybase.bio.indiana.edu/.bin/fbidq.html?tutl), [Ptth](http://flybase.bio.indiana.edu/.bin/fbidq.html?Ptth), [Rab2](http://flybase.bio.indiana.edu/.bin/fbidq.html?Rab2), [Ilp2](http://flybase.bio.indiana.edu/.bin/fbidq.html?Ilp2), [Frq1](http://flybase.bio.indiana.edu/.bin/fbidq.html?Frq1), [SPoCk](http://flybase.bio.indiana.edu/.bin/fbidq.html?SPoCk), [sna](http://flybase.bio.indiana.edu/.bin/fbidq.html?sna), [Wnt5](http://flybase.bio.indiana.edu/.bin/fbidq.html?Wnt5), [Taf1](http://flybase.bio.indiana.edu/.bin/fbidq.html?Taf1), [Ggamma1](http://flybase.bio.indiana.edu/.bin/fbidq.html?Ggamma1), [fus](http://flybase.bio.indiana.edu/.bin/fbidq.html?fus), [sif](http://flybase.bio.indiana.edu/.bin/fbidq.html?sif), [RSG7](http://flybase.bio.indiana.edu/.bin/fbidq.html?RSG7), [m4](http://flybase.bio.indiana.edu/.bin/fbidq.html?m4), [dom](http://flybase.bio.indiana.edu/.bin/fbidq.html?dom), [Tbh](http://flybase.bio.indiana.edu/.bin/fbidq.html?Tbh), [Dh31](http://flybase.bio.indiana.edu/.bin/fbidq.html?Dh31),[CG42541](http://flybase.bio.indiana.edu/.bin/fbidq.html?CG42541), [PIP5K59B](http://flybase.bio.indiana.edu/.bin/fbidq.html?PIP5K59B), [Hr51](http://flybase.bio.indiana.edu/.bin/fbidq.html?Hr51), [lap](http://flybase.bio.indiana.edu/.bin/fbidq.html?lap), [shi](http://flybase.bio.indiana.edu/.bin/fbidq.html?shi), [CG11155](http://flybase.bio.indiana.edu/.bin/fbidq.html?CG11155), [Spred](http://flybase.bio.indiana.edu/.bin/fbidq.html?Spred), [siz](http://flybase.bio.indiana.edu/.bin/fbidq.html?siz), [B-H2](http://flybase.bio.indiana.edu/.bin/fbidq.html?B-H2), [CG3822](http://flybase.bio.indiana.edu/.bin/fbidq.html?CG3822), [wg](http://flybase.bio.indiana.edu/.bin/fbidq.html?wg), [Rhp](http://flybase.bio.indiana.edu/.bin/fbidq.html?Rhp), [CG13995](http://flybase.bio.indiana.edu/.bin/fbidq.html?CG13995), [E(bx)](http://flybase.bio.indiana.edu/.bin/fbidq.html?E(bx)), [sfl](http://flybase.bio.indiana.edu/.bin/fbidq.html?sfl), [CG13229](http://flybase.bio.indiana.edu/.bin/fbidq.html?CG13229), [Pde11](http://flybase.bio.indiana.edu/.bin/fbidq.html?Pde11), [Dms](http://flybase.bio.indiana.edu/.bin/fbidq.html?Dms), [Snap](http://flybase.bio.indiana.edu/.bin/fbidq.html?Snap), [SerT](http://flybase.bio.indiana.edu/.bin/fbidq.html?SerT), [Utx](http://flybase.bio.indiana.edu/.bin/fbidq.html?Utx), [pburs](http://flybase.bio.indiana.edu/.bin/fbidq.html?pburs), [cos](http://flybase.bio.indiana.edu/.bin/fbidq.html?cos), [Ct](http://flybase.bio.indiana.edu/.bin/fbidq.html?Ct) |
| [regulation of RNA metabolic process](http://amigo.geneontology.org/cgi-bin/amigo/go.cgi?view=details&query=GO:0051252) | 213 of 1594 genes, 13.4% | 661 of 7634 genes, 8.7% | 4.50e-12 | 0.00% | 0.00 | [fz2](http://flybase.bio.indiana.edu/.bin/fbidq.html?fz2), [acj6](http://flybase.bio.indiana.edu/.bin/fbidq.html?acj6), [skd](http://flybase.bio.indiana.edu/.bin/fbidq.html?skd), [jing](http://flybase.bio.indiana.edu/.bin/fbidq.html?jing), [caup](http://flybase.bio.indiana.edu/.bin/fbidq.html?caup), [tll](http://flybase.bio.indiana.edu/.bin/fbidq.html?tll), [Camta](http://flybase.bio.indiana.edu/.bin/fbidq.html?Camta), [ato](http://flybase.bio.indiana.edu/.bin/fbidq.html?ato), [Ets65A](http://flybase.bio.indiana.edu/.bin/fbidq.html?Ets65A), [Rox8](http://flybase.bio.indiana.edu/.bin/fbidq.html?Rox8), [tna](http://flybase.bio.indiana.edu/.bin/fbidq.html?tna), [Sxl](http://flybase.bio.indiana.edu/.bin/fbidq.html?Sxl), [mub](http://flybase.bio.indiana.edu/.bin/fbidq.html?mub), [JIL-1](http://flybase.bio.indiana.edu/.bin/fbidq.html?JIL-1), [Spt6](http://flybase.bio.indiana.edu/.bin/fbidq.html?Spt6), [Iswi](http://flybase.bio.indiana.edu/.bin/fbidq.html?Iswi), [rn](http://flybase.bio.indiana.edu/.bin/fbidq.html?rn), [ap](http://flybase.bio.indiana.edu/.bin/fbidq.html?ap), [br](http://flybase.bio.indiana.edu/.bin/fbidq.html?br), [ksr](http://flybase.bio.indiana.edu/.bin/fbidq.html?ksr), [bi](http://flybase.bio.indiana.edu/.bin/fbidq.html?bi), [chn](http://flybase.bio.indiana.edu/.bin/fbidq.html?chn), [Gsc](http://flybase.bio.indiana.edu/.bin/fbidq.html?Gsc), [Hcf](http://flybase.bio.indiana.edu/.bin/fbidq.html?Hcf), [lola](http://flybase.bio.indiana.edu/.bin/fbidq.html?lola), [HLH4C](http://flybase.bio.indiana.edu/.bin/fbidq.html?HLH4C), [Pcl](http://flybase.bio.indiana.edu/.bin/fbidq.html?Pcl), [simj](http://flybase.bio.indiana.edu/.bin/fbidq.html?simj), [Awh](http://flybase.bio.indiana.edu/.bin/fbidq.html?Awh), [tap](http://flybase.bio.indiana.edu/.bin/fbidq.html?tap), [NC2alpha](http://flybase.bio.indiana.edu/.bin/fbidq.html?NC2alpha), [PHDP](http://flybase.bio.indiana.edu/.bin/fbidq.html?PHDP), [bab1](http://flybase.bio.indiana.edu/.bin/fbidq.html?bab1), [CG4328](http://flybase.bio.indiana.edu/.bin/fbidq.html?CG4328), [Snoo](http://flybase.bio.indiana.edu/.bin/fbidq.html?Snoo), [l(2)NC136](http://flybase.bio.indiana.edu/.bin/fbidq.html?l(2)NC136), [Taf4](http://flybase.bio.indiana.edu/.bin/fbidq.html?Taf4), [Su(var)3-3](http://flybase.bio.indiana.edu/.bin/fbidq.html?Su(var)3-3), [HGTX](http://flybase.bio.indiana.edu/.bin/fbidq.html?HGTX), [not](http://flybase.bio.indiana.edu/.bin/fbidq.html?not), [nvy](http://flybase.bio.indiana.edu/.bin/fbidq.html?nvy),[nub](http://flybase.bio.indiana.edu/.bin/fbidq.html?nub), [nab](http://flybase.bio.indiana.edu/.bin/fbidq.html?nab), [btd](http://flybase.bio.indiana.edu/.bin/fbidq.html?btd), [fd68A](http://flybase.bio.indiana.edu/.bin/fbidq.html?fd68A), [U2af38](http://flybase.bio.indiana.edu/.bin/fbidq.html?U2af38), [HLHmdelta](http://flybase.bio.indiana.edu/.bin/fbidq.html?HLHmdelta), [B-H1](http://flybase.bio.indiana.edu/.bin/fbidq.html?B-H1), [Nipped-A](http://flybase.bio.indiana.edu/.bin/fbidq.html?Nipped-A), [ATbp](http://flybase.bio.indiana.edu/.bin/fbidq.html?ATbp), [Sos](http://flybase.bio.indiana.edu/.bin/fbidq.html?Sos), [Spf45](http://flybase.bio.indiana.edu/.bin/fbidq.html?Spf45), [Fer2](http://flybase.bio.indiana.edu/.bin/fbidq.html?Fer2), [fd102C](http://flybase.bio.indiana.edu/.bin/fbidq.html?fd102C), [vg](http://flybase.bio.indiana.edu/.bin/fbidq.html?vg), [hth](http://flybase.bio.indiana.edu/.bin/fbidq.html?hth), [Nf-YC](http://flybase.bio.indiana.edu/.bin/fbidq.html?Nf-YC), [eya](http://flybase.bio.indiana.edu/.bin/fbidq.html?eya), [fz](http://flybase.bio.indiana.edu/.bin/fbidq.html?fz), [brm](http://flybase.bio.indiana.edu/.bin/fbidq.html?brm), [Vsx2](http://flybase.bio.indiana.edu/.bin/fbidq.html?Vsx2), [Hel25E](http://flybase.bio.indiana.edu/.bin/fbidq.html?Hel25E), [Hey](http://flybase.bio.indiana.edu/.bin/fbidq.html?Hey), [sno](http://flybase.bio.indiana.edu/.bin/fbidq.html?sno), [Sfmbt](http://flybase.bio.indiana.edu/.bin/fbidq.html?Sfmbt), [ara](http://flybase.bio.indiana.edu/.bin/fbidq.html?ara), [mam](http://flybase.bio.indiana.edu/.bin/fbidq.html?mam), [loqs](http://flybase.bio.indiana.edu/.bin/fbidq.html?loqs), [pan](http://flybase.bio.indiana.edu/.bin/fbidq.html?pan), [Dll](http://flybase.bio.indiana.edu/.bin/fbidq.html?Dll), [disco](http://flybase.bio.indiana.edu/.bin/fbidq.html?disco), [grn](http://flybase.bio.indiana.edu/.bin/fbidq.html?grn), [HLHm5](http://flybase.bio.indiana.edu/.bin/fbidq.html?HLHm5), [D12](http://flybase.bio.indiana.edu/.bin/fbidq.html?D12), [Lis-1](http://flybase.bio.indiana.edu/.bin/fbidq.html?Lis-1), [Brf](http://flybase.bio.indiana.edu/.bin/fbidq.html?Brf), [ems](http://flybase.bio.indiana.edu/.bin/fbidq.html?ems), [pros](http://flybase.bio.indiana.edu/.bin/fbidq.html?pros), [tup](http://flybase.bio.indiana.edu/.bin/fbidq.html?tup), [Psi](http://flybase.bio.indiana.edu/.bin/fbidq.html?Psi), [pdm2](http://flybase.bio.indiana.edu/.bin/fbidq.html?pdm2),[Gug](http://flybase.bio.indiana.edu/.bin/fbidq.html?Gug), [pad](http://flybase.bio.indiana.edu/.bin/fbidq.html?pad), [rgr](http://flybase.bio.indiana.edu/.bin/fbidq.html?rgr), [Wnt4](http://flybase.bio.indiana.edu/.bin/fbidq.html?Wnt4), [Rx](http://flybase.bio.indiana.edu/.bin/fbidq.html?Rx), [MED1](http://flybase.bio.indiana.edu/.bin/fbidq.html?MED1), [salr](http://flybase.bio.indiana.edu/.bin/fbidq.html?salr), [wdn](http://flybase.bio.indiana.edu/.bin/fbidq.html?wdn), [sim](http://flybase.bio.indiana.edu/.bin/fbidq.html?sim), [d4](http://flybase.bio.indiana.edu/.bin/fbidq.html?d4), [dan](http://flybase.bio.indiana.edu/.bin/fbidq.html?dan), [gro](http://flybase.bio.indiana.edu/.bin/fbidq.html?gro), [nerfin-1](http://flybase.bio.indiana.edu/.bin/fbidq.html?nerfin-1), [trx](http://flybase.bio.indiana.edu/.bin/fbidq.html?trx), [Rpd3](http://flybase.bio.indiana.edu/.bin/fbidq.html?Rpd3), [dsx](http://flybase.bio.indiana.edu/.bin/fbidq.html?dsx), [Ssdp](http://flybase.bio.indiana.edu/.bin/fbidq.html?Ssdp), [vfl](http://flybase.bio.indiana.edu/.bin/fbidq.html?vfl), [sqz](http://flybase.bio.indiana.edu/.bin/fbidq.html?sqz), [CG32105](http://flybase.bio.indiana.edu/.bin/fbidq.html?CG32105), [Fer3](http://flybase.bio.indiana.edu/.bin/fbidq.html?Fer3), [aret](http://flybase.bio.indiana.edu/.bin/fbidq.html?aret), [pygo](http://flybase.bio.indiana.edu/.bin/fbidq.html?pygo), [dalao](http://flybase.bio.indiana.edu/.bin/fbidq.html?dalao), [HLHm7](http://flybase.bio.indiana.edu/.bin/fbidq.html?HLHm7), [z](http://flybase.bio.indiana.edu/.bin/fbidq.html?z), [HLHmgamma](http://flybase.bio.indiana.edu/.bin/fbidq.html?HLHmgamma), [Sox21b](http://flybase.bio.indiana.edu/.bin/fbidq.html?Sox21b), [sr](http://flybase.bio.indiana.edu/.bin/fbidq.html?sr), [Lim1](http://flybase.bio.indiana.edu/.bin/fbidq.html?Lim1), [tld](http://flybase.bio.indiana.edu/.bin/fbidq.html?tld), [danr](http://flybase.bio.indiana.edu/.bin/fbidq.html?danr), [wor](http://flybase.bio.indiana.edu/.bin/fbidq.html?wor), [vvl](http://flybase.bio.indiana.edu/.bin/fbidq.html?vvl), [CG7879](http://flybase.bio.indiana.edu/.bin/fbidq.html?CG7879), [wda](http://flybase.bio.indiana.edu/.bin/fbidq.html?wda), [sgg](http://flybase.bio.indiana.edu/.bin/fbidq.html?sgg), [MED14](http://flybase.bio.indiana.edu/.bin/fbidq.html?MED14), [gcm2](http://flybase.bio.indiana.edu/.bin/fbidq.html?gcm2), [pUf68](http://flybase.bio.indiana.edu/.bin/fbidq.html?pUf68),[Eip93F](http://flybase.bio.indiana.edu/.bin/fbidq.html?Eip93F), [gcm](http://flybase.bio.indiana.edu/.bin/fbidq.html?gcm), [seq](http://flybase.bio.indiana.edu/.bin/fbidq.html?seq), [Dip3](http://flybase.bio.indiana.edu/.bin/fbidq.html?Dip3), [Aef1](http://flybase.bio.indiana.edu/.bin/fbidq.html?Aef1), [HDAC4](http://flybase.bio.indiana.edu/.bin/fbidq.html?HDAC4), [can](http://flybase.bio.indiana.edu/.bin/fbidq.html?can), [elk](http://flybase.bio.indiana.edu/.bin/fbidq.html?elk), [phl](http://flybase.bio.indiana.edu/.bin/fbidq.html?phl), [ase](http://flybase.bio.indiana.edu/.bin/fbidq.html?ase), [CG13253](http://flybase.bio.indiana.edu/.bin/fbidq.html?CG13253), [ovo](http://flybase.bio.indiana.edu/.bin/fbidq.html?ovo), [scro](http://flybase.bio.indiana.edu/.bin/fbidq.html?scro), [CG6197](http://flybase.bio.indiana.edu/.bin/fbidq.html?CG6197), [elav](http://flybase.bio.indiana.edu/.bin/fbidq.html?elav), [Dsp1](http://flybase.bio.indiana.edu/.bin/fbidq.html?Dsp1), [CG14216](http://flybase.bio.indiana.edu/.bin/fbidq.html?CG14216), [Pdp1](http://flybase.bio.indiana.edu/.bin/fbidq.html?Pdp1), [Oli](http://flybase.bio.indiana.edu/.bin/fbidq.html?Oli), [Nelf-A](http://flybase.bio.indiana.edu/.bin/fbidq.html?Nelf-A), [GATAd](http://flybase.bio.indiana.edu/.bin/fbidq.html?GATAd), [CG6227](http://flybase.bio.indiana.edu/.bin/fbidq.html?CG6227), [Poxn](http://flybase.bio.indiana.edu/.bin/fbidq.html?Poxn), [CG15376](http://flybase.bio.indiana.edu/.bin/fbidq.html?CG15376), [Sin3A](http://flybase.bio.indiana.edu/.bin/fbidq.html?Sin3A), [mip120](http://flybase.bio.indiana.edu/.bin/fbidq.html?mip120), [jumu](http://flybase.bio.indiana.edu/.bin/fbidq.html?jumu), [Smox](http://flybase.bio.indiana.edu/.bin/fbidq.html?Smox), [Usp7](http://flybase.bio.indiana.edu/.bin/fbidq.html?Usp7), [ush](http://flybase.bio.indiana.edu/.bin/fbidq.html?ush), [fd59A](http://flybase.bio.indiana.edu/.bin/fbidq.html?fd59A), [Gap1](http://flybase.bio.indiana.edu/.bin/fbidq.html?Gap1), [run](http://flybase.bio.indiana.edu/.bin/fbidq.html?run), [FBX011](http://flybase.bio.indiana.edu/.bin/fbidq.html?FBX011), [Antp](http://flybase.bio.indiana.edu/.bin/fbidq.html?Antp),[tara](http://flybase.bio.indiana.edu/.bin/fbidq.html?tara), [Alh](http://flybase.bio.indiana.edu/.bin/fbidq.html?Alh), [Sox102F](http://flybase.bio.indiana.edu/.bin/fbidq.html?Sox102F), [egg](http://flybase.bio.indiana.edu/.bin/fbidq.html?egg), [l(3)neo38](http://flybase.bio.indiana.edu/.bin/fbidq.html?l(3)neo38), [koko](http://flybase.bio.indiana.edu/.bin/fbidq.html?koko), [Ptth](http://flybase.bio.indiana.edu/.bin/fbidq.html?Ptth), [Sp1](http://flybase.bio.indiana.edu/.bin/fbidq.html?Sp1), [Ptx1](http://flybase.bio.indiana.edu/.bin/fbidq.html?Ptx1), [ph-p](http://flybase.bio.indiana.edu/.bin/fbidq.html?ph-p), [Ubx](http://flybase.bio.indiana.edu/.bin/fbidq.html?Ubx), [onecut](http://flybase.bio.indiana.edu/.bin/fbidq.html?onecut), [CG7757](http://flybase.bio.indiana.edu/.bin/fbidq.html?CG7757), [chm](http://flybase.bio.indiana.edu/.bin/fbidq.html?chm), [sna](http://flybase.bio.indiana.edu/.bin/fbidq.html?sna), [Wnt5](http://flybase.bio.indiana.edu/.bin/fbidq.html?Wnt5), [Taf1](http://flybase.bio.indiana.edu/.bin/fbidq.html?Taf1), [hbn](http://flybase.bio.indiana.edu/.bin/fbidq.html?hbn), [MBD-like](http://flybase.bio.indiana.edu/.bin/fbidq.html?MBD-like), [tsh](http://flybase.bio.indiana.edu/.bin/fbidq.html?tsh), [esg](http://flybase.bio.indiana.edu/.bin/fbidq.html?esg), [e(y)3](http://flybase.bio.indiana.edu/.bin/fbidq.html?e(y)3), [CG9007](http://flybase.bio.indiana.edu/.bin/fbidq.html?CG9007), [oc](http://flybase.bio.indiana.edu/.bin/fbidq.html?oc), [dom](http://flybase.bio.indiana.edu/.bin/fbidq.html?dom), [spt4](http://flybase.bio.indiana.edu/.bin/fbidq.html?spt4), [CG32532](http://flybase.bio.indiana.edu/.bin/fbidq.html?CG32532), [MTA1-like](http://flybase.bio.indiana.edu/.bin/fbidq.html?MTA1-like), [gol](http://flybase.bio.indiana.edu/.bin/fbidq.html?gol), [ey](http://flybase.bio.indiana.edu/.bin/fbidq.html?ey), [mle](http://flybase.bio.indiana.edu/.bin/fbidq.html?mle), [Hr51](http://flybase.bio.indiana.edu/.bin/fbidq.html?Hr51), [Bgb](http://flybase.bio.indiana.edu/.bin/fbidq.html?Bgb), [l(1)sc](http://flybase.bio.indiana.edu/.bin/fbidq.html?l(1)sc), [B-H2](http://flybase.bio.indiana.edu/.bin/fbidq.html?B-H2), [grh](http://flybase.bio.indiana.edu/.bin/fbidq.html?grh), [Vsx1](http://flybase.bio.indiana.edu/.bin/fbidq.html?Vsx1), [wg](http://flybase.bio.indiana.edu/.bin/fbidq.html?wg),[toy](http://flybase.bio.indiana.edu/.bin/fbidq.html?toy), [fkh](http://flybase.bio.indiana.edu/.bin/fbidq.html?fkh), [Lim3](http://flybase.bio.indiana.edu/.bin/fbidq.html?Lim3), [CG34362](http://flybase.bio.indiana.edu/.bin/fbidq.html?CG34362), [crol](http://flybase.bio.indiana.edu/.bin/fbidq.html?crol), [sd](http://flybase.bio.indiana.edu/.bin/fbidq.html?sd), [fne](http://flybase.bio.indiana.edu/.bin/fbidq.html?fne), [retn](http://flybase.bio.indiana.edu/.bin/fbidq.html?retn), [en](http://flybase.bio.indiana.edu/.bin/fbidq.html?en), [fs(1)h](http://flybase.bio.indiana.edu/.bin/fbidq.html?fs(1)h), [rl](http://flybase.bio.indiana.edu/.bin/fbidq.html?rl), [CG11347](http://flybase.bio.indiana.edu/.bin/fbidq.html?CG11347), [tey](http://flybase.bio.indiana.edu/.bin/fbidq.html?tey), [Saf-B](http://flybase.bio.indiana.edu/.bin/fbidq.html?Saf-B), [CG11294](http://flybase.bio.indiana.edu/.bin/fbidq.html?CG11294), [hiw](http://flybase.bio.indiana.edu/.bin/fbidq.html?hiw), [cos](http://flybase.bio.indiana.edu/.bin/fbidq.html?cos), [Ct](http://flybase.bio.indiana.edu/.bin/fbidq.html?Ct), [Atf-2](http://flybase.bio.indiana.edu/.bin/fbidq.html?Atf-2) |
| [signal transduction](http://amigo.geneontology.org/cgi-bin/amigo/go.cgi?view=details&query=GO:0007165) | 280 of 1594 genes, 17.6% | 940 of 7634 genes, 12.3% | 8.63e-12 | 0.00% | 0.00 | [fz2](http://flybase.bio.indiana.edu/.bin/fbidq.html?fz2), [CG14375](http://flybase.bio.indiana.edu/.bin/fbidq.html?CG14375), [Ocho](http://flybase.bio.indiana.edu/.bin/fbidq.html?Ocho), [numb](http://flybase.bio.indiana.edu/.bin/fbidq.html?numb), [dock](http://flybase.bio.indiana.edu/.bin/fbidq.html?dock), [Sxl](http://flybase.bio.indiana.edu/.bin/fbidq.html?Sxl), [CG8500](http://flybase.bio.indiana.edu/.bin/fbidq.html?CG8500), [cenG1A](http://flybase.bio.indiana.edu/.bin/fbidq.html?cenG1A), [Nf1](http://flybase.bio.indiana.edu/.bin/fbidq.html?Nf1), [malpha](http://flybase.bio.indiana.edu/.bin/fbidq.html?malpha), [AR-2](http://flybase.bio.indiana.edu/.bin/fbidq.html?AR-2), [os](http://flybase.bio.indiana.edu/.bin/fbidq.html?os), [Axn](http://flybase.bio.indiana.edu/.bin/fbidq.html?Axn), [Traf6](http://flybase.bio.indiana.edu/.bin/fbidq.html?Traf6), [Leucokinin](http://flybase.bio.indiana.edu/.bin/fbidq.html?Leucokinin), [rdgC](http://flybase.bio.indiana.edu/.bin/fbidq.html?rdgC), [Galpha49B](http://flybase.bio.indiana.edu/.bin/fbidq.html?Galpha49B), [Snoo](http://flybase.bio.indiana.edu/.bin/fbidq.html?Snoo), [rut](http://flybase.bio.indiana.edu/.bin/fbidq.html?rut), [CG7650](http://flybase.bio.indiana.edu/.bin/fbidq.html?CG7650), [RhoGAP100F](http://flybase.bio.indiana.edu/.bin/fbidq.html?RhoGAP100F), [uif](http://flybase.bio.indiana.edu/.bin/fbidq.html?uif), [Mmp2](http://flybase.bio.indiana.edu/.bin/fbidq.html?Mmp2), [HLHmdelta](http://flybase.bio.indiana.edu/.bin/fbidq.html?HLHmdelta), [Nplp1](http://flybase.bio.indiana.edu/.bin/fbidq.html?Nplp1), [Nipped-A](http://flybase.bio.indiana.edu/.bin/fbidq.html?Nipped-A), [CG32206](http://flybase.bio.indiana.edu/.bin/fbidq.html?CG32206), [7B2](http://flybase.bio.indiana.edu/.bin/fbidq.html?7B2), [alph](http://flybase.bio.indiana.edu/.bin/fbidq.html?alph), [Traf4](http://flybase.bio.indiana.edu/.bin/fbidq.html?Traf4), [mGluRA](http://flybase.bio.indiana.edu/.bin/fbidq.html?mGluRA),[dsh](http://flybase.bio.indiana.edu/.bin/fbidq.html?dsh), [pzg](http://flybase.bio.indiana.edu/.bin/fbidq.html?pzg), [Nf-YC](http://flybase.bio.indiana.edu/.bin/fbidq.html?Nf-YC), [14-3-3zeta](http://flybase.bio.indiana.edu/.bin/fbidq.html?14-3-3zeta), [Pli](http://flybase.bio.indiana.edu/.bin/fbidq.html?Pli), [shakB](http://flybase.bio.indiana.edu/.bin/fbidq.html?shakB), [CG31140](http://flybase.bio.indiana.edu/.bin/fbidq.html?CG31140), [Hey](http://flybase.bio.indiana.edu/.bin/fbidq.html?Hey), [plexB](http://flybase.bio.indiana.edu/.bin/fbidq.html?plexB), [AlstR](http://flybase.bio.indiana.edu/.bin/fbidq.html?AlstR), [PP2A-B](http://flybase.bio.indiana.edu/.bin/fbidq.html?PP2A-B), [IM10](http://flybase.bio.indiana.edu/.bin/fbidq.html?IM10), [neur](http://flybase.bio.indiana.edu/.bin/fbidq.html?neur), [CycE](http://flybase.bio.indiana.edu/.bin/fbidq.html?CycE), [rho-5](http://flybase.bio.indiana.edu/.bin/fbidq.html?rho-5), [nmo](http://flybase.bio.indiana.edu/.bin/fbidq.html?nmo), [Tusp](http://flybase.bio.indiana.edu/.bin/fbidq.html?Tusp), [5-HT1A](http://flybase.bio.indiana.edu/.bin/fbidq.html?5-HT1A), [CG32149](http://flybase.bio.indiana.edu/.bin/fbidq.html?CG32149), [Vdup1](http://flybase.bio.indiana.edu/.bin/fbidq.html?Vdup1), [Gug](http://flybase.bio.indiana.edu/.bin/fbidq.html?Gug), [ed](http://flybase.bio.indiana.edu/.bin/fbidq.html?ed), [daw](http://flybase.bio.indiana.edu/.bin/fbidq.html?daw), [CG5036](http://flybase.bio.indiana.edu/.bin/fbidq.html?CG5036), [5-HT1B](http://flybase.bio.indiana.edu/.bin/fbidq.html?5-HT1B), [Apc](http://flybase.bio.indiana.edu/.bin/fbidq.html?Apc), [sim](http://flybase.bio.indiana.edu/.bin/fbidq.html?sim), [CG8795](http://flybase.bio.indiana.edu/.bin/fbidq.html?CG8795), [gro](http://flybase.bio.indiana.edu/.bin/fbidq.html?gro), [RN-tre](http://flybase.bio.indiana.edu/.bin/fbidq.html?RN-tre), [scyl](http://flybase.bio.indiana.edu/.bin/fbidq.html?scyl), [Btk29A](http://flybase.bio.indiana.edu/.bin/fbidq.html?Btk29A), [ETH](http://flybase.bio.indiana.edu/.bin/fbidq.html?ETH), [unc-5](http://flybase.bio.indiana.edu/.bin/fbidq.html?unc-5), [cdi](http://flybase.bio.indiana.edu/.bin/fbidq.html?cdi),[NPFR1](http://flybase.bio.indiana.edu/.bin/fbidq.html?NPFR1), [pygo](http://flybase.bio.indiana.edu/.bin/fbidq.html?pygo), [HLHmgamma](http://flybase.bio.indiana.edu/.bin/fbidq.html?HLHmgamma), [CG15556](http://flybase.bio.indiana.edu/.bin/fbidq.html?CG15556), [Ror](http://flybase.bio.indiana.edu/.bin/fbidq.html?Ror), [tow](http://flybase.bio.indiana.edu/.bin/fbidq.html?tow), [CG15609](http://flybase.bio.indiana.edu/.bin/fbidq.html?CG15609), [Atg1](http://flybase.bio.indiana.edu/.bin/fbidq.html?Atg1), [Pten](http://flybase.bio.indiana.edu/.bin/fbidq.html?Pten), [unc-13](http://flybase.bio.indiana.edu/.bin/fbidq.html?unc-13), [npf](http://flybase.bio.indiana.edu/.bin/fbidq.html?npf), [oa2](http://flybase.bio.indiana.edu/.bin/fbidq.html?oa2), [elk](http://flybase.bio.indiana.edu/.bin/fbidq.html?elk), [phl](http://flybase.bio.indiana.edu/.bin/fbidq.html?phl), [Cbl](http://flybase.bio.indiana.edu/.bin/fbidq.html?Cbl), [Rab26](http://flybase.bio.indiana.edu/.bin/fbidq.html?Rab26), [Pde6](http://flybase.bio.indiana.edu/.bin/fbidq.html?Pde6), [sNPF](http://flybase.bio.indiana.edu/.bin/fbidq.html?sNPF), [pog](http://flybase.bio.indiana.edu/.bin/fbidq.html?pog), [plx](http://flybase.bio.indiana.edu/.bin/fbidq.html?plx), [CG9098](http://flybase.bio.indiana.edu/.bin/fbidq.html?CG9098), [CG8108](http://flybase.bio.indiana.edu/.bin/fbidq.html?CG8108), [IFa](http://flybase.bio.indiana.edu/.bin/fbidq.html?IFa), [mtt](http://flybase.bio.indiana.edu/.bin/fbidq.html?mtt), [CG32758](http://flybase.bio.indiana.edu/.bin/fbidq.html?CG32758), [Alk](http://flybase.bio.indiana.edu/.bin/fbidq.html?Alk), [Pde1c](http://flybase.bio.indiana.edu/.bin/fbidq.html?Pde1c), [disp](http://flybase.bio.indiana.edu/.bin/fbidq.html?disp), [casp](http://flybase.bio.indiana.edu/.bin/fbidq.html?casp), [CG4022](http://flybase.bio.indiana.edu/.bin/fbidq.html?CG4022), [Smox](http://flybase.bio.indiana.edu/.bin/fbidq.html?Smox), [Plc21C](http://flybase.bio.indiana.edu/.bin/fbidq.html?Plc21C), [GRHRII](http://flybase.bio.indiana.edu/.bin/fbidq.html?GRHRII),[CG10362](http://flybase.bio.indiana.edu/.bin/fbidq.html?CG10362), [GABA-B-R2](http://flybase.bio.indiana.edu/.bin/fbidq.html?GABA-B-R2), [Ast-C](http://flybase.bio.indiana.edu/.bin/fbidq.html?Ast-C), [Gbeta5](http://flybase.bio.indiana.edu/.bin/fbidq.html?Gbeta5), [hug](http://flybase.bio.indiana.edu/.bin/fbidq.html?hug), [Sur-8](http://flybase.bio.indiana.edu/.bin/fbidq.html?Sur-8), [Rgl](http://flybase.bio.indiana.edu/.bin/fbidq.html?Rgl), [e(y)3](http://flybase.bio.indiana.edu/.bin/fbidq.html?e(y)3), [CRMP](http://flybase.bio.indiana.edu/.bin/fbidq.html?CRMP), [Cdk5](http://flybase.bio.indiana.edu/.bin/fbidq.html?Cdk5), [mav](http://flybase.bio.indiana.edu/.bin/fbidq.html?mav), [Ilp7](http://flybase.bio.indiana.edu/.bin/fbidq.html?Ilp7), [ey](http://flybase.bio.indiana.edu/.bin/fbidq.html?ey), [Mip](http://flybase.bio.indiana.edu/.bin/fbidq.html?Mip), [baz](http://flybase.bio.indiana.edu/.bin/fbidq.html?baz), [CG33275](http://flybase.bio.indiana.edu/.bin/fbidq.html?CG33275), [Oamb](http://flybase.bio.indiana.edu/.bin/fbidq.html?Oamb), [CG34381](http://flybase.bio.indiana.edu/.bin/fbidq.html?CG34381), [Cdk5alpha](http://flybase.bio.indiana.edu/.bin/fbidq.html?Cdk5alpha), [CG40351](http://flybase.bio.indiana.edu/.bin/fbidq.html?CG40351), [crol](http://flybase.bio.indiana.edu/.bin/fbidq.html?crol), [sd](http://flybase.bio.indiana.edu/.bin/fbidq.html?sd), [Aplip1](http://flybase.bio.indiana.edu/.bin/fbidq.html?Aplip1), [Ac3](http://flybase.bio.indiana.edu/.bin/fbidq.html?Ac3), [rl](http://flybase.bio.indiana.edu/.bin/fbidq.html?rl), [CG17760](http://flybase.bio.indiana.edu/.bin/fbidq.html?CG17760), [Nup153](http://flybase.bio.indiana.edu/.bin/fbidq.html?Nup153), [CG32683](http://flybase.bio.indiana.edu/.bin/fbidq.html?CG32683), [CG11347](http://flybase.bio.indiana.edu/.bin/fbidq.html?CG11347), [Ggamma30A](http://flybase.bio.indiana.edu/.bin/fbidq.html?Ggamma30A), [wls](http://flybase.bio.indiana.edu/.bin/fbidq.html?wls),[hiw](http://flybase.bio.indiana.edu/.bin/fbidq.html?hiw), [Atf-2](http://flybase.bio.indiana.edu/.bin/fbidq.html?Atf-2), [skd](http://flybase.bio.indiana.edu/.bin/fbidq.html?skd), [Camta](http://flybase.bio.indiana.edu/.bin/fbidq.html?Camta), [tll](http://flybase.bio.indiana.edu/.bin/fbidq.html?tll), [ato](http://flybase.bio.indiana.edu/.bin/fbidq.html?ato), [Fmrf](http://flybase.bio.indiana.edu/.bin/fbidq.html?Fmrf), [Iswi](http://flybase.bio.indiana.edu/.bin/fbidq.html?Iswi), [Trim9](http://flybase.bio.indiana.edu/.bin/fbidq.html?Trim9), [GABA-B-R3](http://flybase.bio.indiana.edu/.bin/fbidq.html?GABA-B-R3), [Pka-R1](http://flybase.bio.indiana.edu/.bin/fbidq.html?Pka-R1), [mago](http://flybase.bio.indiana.edu/.bin/fbidq.html?mago), [Evi5](http://flybase.bio.indiana.edu/.bin/fbidq.html?Evi5), [Gycalpha99B](http://flybase.bio.indiana.edu/.bin/fbidq.html?Gycalpha99B), [ksr](http://flybase.bio.indiana.edu/.bin/fbidq.html?ksr), [CG42533](http://flybase.bio.indiana.edu/.bin/fbidq.html?CG42533), [CG11376](http://flybase.bio.indiana.edu/.bin/fbidq.html?CG11376), [Gyc-89Da](http://flybase.bio.indiana.edu/.bin/fbidq.html?Gyc-89Da), [D2R](http://flybase.bio.indiana.edu/.bin/fbidq.html?D2R), [CG31158](http://flybase.bio.indiana.edu/.bin/fbidq.html?CG31158), [ewg](http://flybase.bio.indiana.edu/.bin/fbidq.html?ewg), [Nrk](http://flybase.bio.indiana.edu/.bin/fbidq.html?Nrk), [Cirl](http://flybase.bio.indiana.edu/.bin/fbidq.html?Cirl), [Su(var)3-3](http://flybase.bio.indiana.edu/.bin/fbidq.html?Su(var)3-3), [Ilp3](http://flybase.bio.indiana.edu/.bin/fbidq.html?Ilp3), [Bili](http://flybase.bio.indiana.edu/.bin/fbidq.html?Bili), [Nrg](http://flybase.bio.indiana.edu/.bin/fbidq.html?Nrg), [CG34384](http://flybase.bio.indiana.edu/.bin/fbidq.html?CG34384), [stet](http://flybase.bio.indiana.edu/.bin/fbidq.html?stet), [Dh44-R1](http://flybase.bio.indiana.edu/.bin/fbidq.html?Dh44-R1), [CG32447](http://flybase.bio.indiana.edu/.bin/fbidq.html?CG32447), [Eh](http://flybase.bio.indiana.edu/.bin/fbidq.html?Eh), [Sema-1a](http://flybase.bio.indiana.edu/.bin/fbidq.html?Sema-1a), [Hs3st-B](http://flybase.bio.indiana.edu/.bin/fbidq.html?Hs3st-B), [otk](http://flybase.bio.indiana.edu/.bin/fbidq.html?otk), [Sos](http://flybase.bio.indiana.edu/.bin/fbidq.html?Sos), [phyl](http://flybase.bio.indiana.edu/.bin/fbidq.html?phyl), [Fas2](http://flybase.bio.indiana.edu/.bin/fbidq.html?Fas2), [CG8557](http://flybase.bio.indiana.edu/.bin/fbidq.html?CG8557), [bsk](http://flybase.bio.indiana.edu/.bin/fbidq.html?bsk), [brm](http://flybase.bio.indiana.edu/.bin/fbidq.html?brm), [mAcR-60C](http://flybase.bio.indiana.edu/.bin/fbidq.html?mAcR-60C), [robo](http://flybase.bio.indiana.edu/.bin/fbidq.html?robo), [fz](http://flybase.bio.indiana.edu/.bin/fbidq.html?fz), [Crz](http://flybase.bio.indiana.edu/.bin/fbidq.html?Crz), [ft](http://flybase.bio.indiana.edu/.bin/fbidq.html?ft), [CG13830](http://flybase.bio.indiana.edu/.bin/fbidq.html?CG13830), [sno](http://flybase.bio.indiana.edu/.bin/fbidq.html?sno), [RhoGAPp190](http://flybase.bio.indiana.edu/.bin/fbidq.html?RhoGAPp190), [l(2)k16918](http://flybase.bio.indiana.edu/.bin/fbidq.html?l(2)k16918), [mam](http://flybase.bio.indiana.edu/.bin/fbidq.html?mam), [Takr86C](http://flybase.bio.indiana.edu/.bin/fbidq.html?Takr86C), [CG30158](http://flybase.bio.indiana.edu/.bin/fbidq.html?CG30158), [pan](http://flybase.bio.indiana.edu/.bin/fbidq.html?pan), [PKD](http://flybase.bio.indiana.edu/.bin/fbidq.html?PKD), [DopR](http://flybase.bio.indiana.edu/.bin/fbidq.html?DopR), [Lis-1](http://flybase.bio.indiana.edu/.bin/fbidq.html?Lis-1), [CG16896](http://flybase.bio.indiana.edu/.bin/fbidq.html?CG16896), [Rab3](http://flybase.bio.indiana.edu/.bin/fbidq.html?Rab3), [tup](http://flybase.bio.indiana.edu/.bin/fbidq.html?tup), [mthl8](http://flybase.bio.indiana.edu/.bin/fbidq.html?mthl8), [trio](http://flybase.bio.indiana.edu/.bin/fbidq.html?trio), [RabX4](http://flybase.bio.indiana.edu/.bin/fbidq.html?RabX4), [spel1](http://flybase.bio.indiana.edu/.bin/fbidq.html?spel1), [Lkr](http://flybase.bio.indiana.edu/.bin/fbidq.html?Lkr), [CG31665](http://flybase.bio.indiana.edu/.bin/fbidq.html?CG31665),[Ccap](http://flybase.bio.indiana.edu/.bin/fbidq.html?Ccap), [Wnt4](http://flybase.bio.indiana.edu/.bin/fbidq.html?Wnt4), [CG7918](http://flybase.bio.indiana.edu/.bin/fbidq.html?CG7918), [DopR2](http://flybase.bio.indiana.edu/.bin/fbidq.html?DopR2), [CG10188](http://flybase.bio.indiana.edu/.bin/fbidq.html?CG10188), [CG30372](http://flybase.bio.indiana.edu/.bin/fbidq.html?CG30372), [CG42629](http://flybase.bio.indiana.edu/.bin/fbidq.html?CG42629), [pigs](http://flybase.bio.indiana.edu/.bin/fbidq.html?pigs), [CG31760](http://flybase.bio.indiana.edu/.bin/fbidq.html?CG31760), [Klp54D](http://flybase.bio.indiana.edu/.bin/fbidq.html?Klp54D), [5-HT7](http://flybase.bio.indiana.edu/.bin/fbidq.html?5-HT7), [Lgr3](http://flybase.bio.indiana.edu/.bin/fbidq.html?Lgr3), [Dh](http://flybase.bio.indiana.edu/.bin/fbidq.html?Dh), [Ac13E](http://flybase.bio.indiana.edu/.bin/fbidq.html?Ac13E), [klu](http://flybase.bio.indiana.edu/.bin/fbidq.html?klu), [SPR](http://flybase.bio.indiana.edu/.bin/fbidq.html?SPR), [CG8155](http://flybase.bio.indiana.edu/.bin/fbidq.html?CG8155), [nkd](http://flybase.bio.indiana.edu/.bin/fbidq.html?nkd), [tld](http://flybase.bio.indiana.edu/.bin/fbidq.html?tld), [RhoGEF3](http://flybase.bio.indiana.edu/.bin/fbidq.html?RhoGEF3), [Dsk](http://flybase.bio.indiana.edu/.bin/fbidq.html?Dsk), [RhoGEF4](http://flybase.bio.indiana.edu/.bin/fbidq.html?RhoGEF4), [CG9121](http://flybase.bio.indiana.edu/.bin/fbidq.html?CG9121), [Brd](http://flybase.bio.indiana.edu/.bin/fbidq.html?Brd), [sgg](http://flybase.bio.indiana.edu/.bin/fbidq.html?sgg), [Nmdar1](http://flybase.bio.indiana.edu/.bin/fbidq.html?Nmdar1), [Eip93F](http://flybase.bio.indiana.edu/.bin/fbidq.html?Eip93F), [inaE](http://flybase.bio.indiana.edu/.bin/fbidq.html?inaE), [Pask](http://flybase.bio.indiana.edu/.bin/fbidq.html?Pask), [rab3-GEF](http://flybase.bio.indiana.edu/.bin/fbidq.html?rab3-GEF),[CG13253](http://flybase.bio.indiana.edu/.bin/fbidq.html?CG13253), [MESK2](http://flybase.bio.indiana.edu/.bin/fbidq.html?MESK2), [SIFR](http://flybase.bio.indiana.edu/.bin/fbidq.html?SIFR), [CG2061](http://flybase.bio.indiana.edu/.bin/fbidq.html?CG2061), [Ast](http://flybase.bio.indiana.edu/.bin/fbidq.html?Ast), [fred](http://flybase.bio.indiana.edu/.bin/fbidq.html?fred), [Drl-2](http://flybase.bio.indiana.edu/.bin/fbidq.html?Drl-2), [ush](http://flybase.bio.indiana.edu/.bin/fbidq.html?ush), [Tk](http://flybase.bio.indiana.edu/.bin/fbidq.html?Tk), [Fak56D](http://flybase.bio.indiana.edu/.bin/fbidq.html?Fak56D), [Ilp5](http://flybase.bio.indiana.edu/.bin/fbidq.html?Ilp5), [Gap1](http://flybase.bio.indiana.edu/.bin/fbidq.html?Gap1), [Toll-6](http://flybase.bio.indiana.edu/.bin/fbidq.html?Toll-6), [CG3227](http://flybase.bio.indiana.edu/.bin/fbidq.html?CG3227), [MESR4](http://flybase.bio.indiana.edu/.bin/fbidq.html?MESR4), [Takr99D](http://flybase.bio.indiana.edu/.bin/fbidq.html?Takr99D), [sNPF-R](http://flybase.bio.indiana.edu/.bin/fbidq.html?sNPF-R), [Hel89B](http://flybase.bio.indiana.edu/.bin/fbidq.html?Hel89B), [CG5337](http://flybase.bio.indiana.edu/.bin/fbidq.html?CG5337), [CG13692](http://flybase.bio.indiana.edu/.bin/fbidq.html?CG13692), [koko](http://flybase.bio.indiana.edu/.bin/fbidq.html?koko), [stan](http://flybase.bio.indiana.edu/.bin/fbidq.html?stan), [CG12424](http://flybase.bio.indiana.edu/.bin/fbidq.html?CG12424), [Ptth](http://flybase.bio.indiana.edu/.bin/fbidq.html?Ptth), [Rab2](http://flybase.bio.indiana.edu/.bin/fbidq.html?Rab2), [Ilp2](http://flybase.bio.indiana.edu/.bin/fbidq.html?Ilp2), [SPoCk](http://flybase.bio.indiana.edu/.bin/fbidq.html?SPoCk), [Wnt5](http://flybase.bio.indiana.edu/.bin/fbidq.html?Wnt5), [Taf1](http://flybase.bio.indiana.edu/.bin/fbidq.html?Taf1), [Ggamma1](http://flybase.bio.indiana.edu/.bin/fbidq.html?Ggamma1), [fus](http://flybase.bio.indiana.edu/.bin/fbidq.html?fus), [sif](http://flybase.bio.indiana.edu/.bin/fbidq.html?sif), [RSG7](http://flybase.bio.indiana.edu/.bin/fbidq.html?RSG7),[m4](http://flybase.bio.indiana.edu/.bin/fbidq.html?m4), [dom](http://flybase.bio.indiana.edu/.bin/fbidq.html?dom), [Tbh](http://flybase.bio.indiana.edu/.bin/fbidq.html?Tbh), [Dh31](http://flybase.bio.indiana.edu/.bin/fbidq.html?Dh31), [CG42541](http://flybase.bio.indiana.edu/.bin/fbidq.html?CG42541), [Hr51](http://flybase.bio.indiana.edu/.bin/fbidq.html?Hr51), [shi](http://flybase.bio.indiana.edu/.bin/fbidq.html?shi), [Spred](http://flybase.bio.indiana.edu/.bin/fbidq.html?Spred), [siz](http://flybase.bio.indiana.edu/.bin/fbidq.html?siz), [wg](http://flybase.bio.indiana.edu/.bin/fbidq.html?wg), [Rhp](http://flybase.bio.indiana.edu/.bin/fbidq.html?Rhp), [CG13995](http://flybase.bio.indiana.edu/.bin/fbidq.html?CG13995), [E(bx)](http://flybase.bio.indiana.edu/.bin/fbidq.html?E(bx)), [sfl](http://flybase.bio.indiana.edu/.bin/fbidq.html?sfl), [CG13229](http://flybase.bio.indiana.edu/.bin/fbidq.html?CG13229), [Dms](http://flybase.bio.indiana.edu/.bin/fbidq.html?Dms), [Pde11](http://flybase.bio.indiana.edu/.bin/fbidq.html?Pde11), [Utx](http://flybase.bio.indiana.edu/.bin/fbidq.html?Utx), [pburs](http://flybase.bio.indiana.edu/.bin/fbidq.html?pburs), [cos](http://flybase.bio.indiana.edu/.bin/fbidq.html?cos), [Ct](http://flybase.bio.indiana.edu/.bin/fbidq.html?Ct) |
| [cell communication](http://amigo.geneontology.org/cgi-bin/amigo/go.cgi?view=details&query=GO:0007154) | 355 of 1594 genes, 22.3% | 1262 of 7634 genes, 16.5% | 9.15e-12 | 0.00% | 0.00 | [fz2](http://flybase.bio.indiana.edu/.bin/fbidq.html?fz2), [endoA](http://flybase.bio.indiana.edu/.bin/fbidq.html?endoA), [CG14375](http://flybase.bio.indiana.edu/.bin/fbidq.html?CG14375), [Ocho](http://flybase.bio.indiana.edu/.bin/fbidq.html?Ocho), [Sh](http://flybase.bio.indiana.edu/.bin/fbidq.html?Sh), [numb](http://flybase.bio.indiana.edu/.bin/fbidq.html?numb), [dock](http://flybase.bio.indiana.edu/.bin/fbidq.html?dock), [stj](http://flybase.bio.indiana.edu/.bin/fbidq.html?stj), [Sxl](http://flybase.bio.indiana.edu/.bin/fbidq.html?Sxl), [CG8500](http://flybase.bio.indiana.edu/.bin/fbidq.html?CG8500), [cenG1A](http://flybase.bio.indiana.edu/.bin/fbidq.html?cenG1A), [Nf1](http://flybase.bio.indiana.edu/.bin/fbidq.html?Nf1), [malpha](http://flybase.bio.indiana.edu/.bin/fbidq.html?malpha), [AR-2](http://flybase.bio.indiana.edu/.bin/fbidq.html?AR-2), [os](http://flybase.bio.indiana.edu/.bin/fbidq.html?os), [Axn](http://flybase.bio.indiana.edu/.bin/fbidq.html?Axn), [Sytalpha](http://flybase.bio.indiana.edu/.bin/fbidq.html?Sytalpha), [Traf6](http://flybase.bio.indiana.edu/.bin/fbidq.html?Traf6), [Leucokinin](http://flybase.bio.indiana.edu/.bin/fbidq.html?Leucokinin), [rdgC](http://flybase.bio.indiana.edu/.bin/fbidq.html?rdgC), [Galpha49B](http://flybase.bio.indiana.edu/.bin/fbidq.html?Galpha49B), [Snoo](http://flybase.bio.indiana.edu/.bin/fbidq.html?Snoo), [Lar](http://flybase.bio.indiana.edu/.bin/fbidq.html?Lar), [scrib](http://flybase.bio.indiana.edu/.bin/fbidq.html?scrib), [not](http://flybase.bio.indiana.edu/.bin/fbidq.html?not), [rut](http://flybase.bio.indiana.edu/.bin/fbidq.html?rut), [CG7650](http://flybase.bio.indiana.edu/.bin/fbidq.html?CG7650), [RhoGAP100F](http://flybase.bio.indiana.edu/.bin/fbidq.html?RhoGAP100F), [uif](http://flybase.bio.indiana.edu/.bin/fbidq.html?uif), [Mmp2](http://flybase.bio.indiana.edu/.bin/fbidq.html?Mmp2), [HLHmdelta](http://flybase.bio.indiana.edu/.bin/fbidq.html?HLHmdelta), [Nplp1](http://flybase.bio.indiana.edu/.bin/fbidq.html?Nplp1), [Nipped-A](http://flybase.bio.indiana.edu/.bin/fbidq.html?Nipped-A),[CG32206](http://flybase.bio.indiana.edu/.bin/fbidq.html?CG32206), [7B2](http://flybase.bio.indiana.edu/.bin/fbidq.html?7B2), [alph](http://flybase.bio.indiana.edu/.bin/fbidq.html?alph), [Traf4](http://flybase.bio.indiana.edu/.bin/fbidq.html?Traf4), [TBPH](http://flybase.bio.indiana.edu/.bin/fbidq.html?TBPH), [Snap25](http://flybase.bio.indiana.edu/.bin/fbidq.html?Snap25), [mGluRA](http://flybase.bio.indiana.edu/.bin/fbidq.html?mGluRA), [dsh](http://flybase.bio.indiana.edu/.bin/fbidq.html?dsh), [pzg](http://flybase.bio.indiana.edu/.bin/fbidq.html?pzg), [Nf-YC](http://flybase.bio.indiana.edu/.bin/fbidq.html?Nf-YC), [14-3-3zeta](http://flybase.bio.indiana.edu/.bin/fbidq.html?14-3-3zeta), [Pli](http://flybase.bio.indiana.edu/.bin/fbidq.html?Pli), [shakB](http://flybase.bio.indiana.edu/.bin/fbidq.html?shakB), [comt](http://flybase.bio.indiana.edu/.bin/fbidq.html?comt), [CG31140](http://flybase.bio.indiana.edu/.bin/fbidq.html?CG31140), [Hey](http://flybase.bio.indiana.edu/.bin/fbidq.html?Hey), [plexB](http://flybase.bio.indiana.edu/.bin/fbidq.html?plexB), [AlstR](http://flybase.bio.indiana.edu/.bin/fbidq.html?AlstR), [PP2A-B](http://flybase.bio.indiana.edu/.bin/fbidq.html?PP2A-B), [qvr](http://flybase.bio.indiana.edu/.bin/fbidq.html?qvr), [mars](http://flybase.bio.indiana.edu/.bin/fbidq.html?mars), [IM10](http://flybase.bio.indiana.edu/.bin/fbidq.html?IM10), [neur](http://flybase.bio.indiana.edu/.bin/fbidq.html?neur), [CycE](http://flybase.bio.indiana.edu/.bin/fbidq.html?CycE), [rho-5](http://flybase.bio.indiana.edu/.bin/fbidq.html?rho-5), [nmo](http://flybase.bio.indiana.edu/.bin/fbidq.html?nmo), [Tusp](http://flybase.bio.indiana.edu/.bin/fbidq.html?Tusp), [5-HT1A](http://flybase.bio.indiana.edu/.bin/fbidq.html?5-HT1A), [CG32149](http://flybase.bio.indiana.edu/.bin/fbidq.html?CG32149), [Vdup1](http://flybase.bio.indiana.edu/.bin/fbidq.html?Vdup1), [Gug](http://flybase.bio.indiana.edu/.bin/fbidq.html?Gug), [ed](http://flybase.bio.indiana.edu/.bin/fbidq.html?ed), [mr](http://flybase.bio.indiana.edu/.bin/fbidq.html?mr), [daw](http://flybase.bio.indiana.edu/.bin/fbidq.html?daw),[CG5036](http://flybase.bio.indiana.edu/.bin/fbidq.html?CG5036), [5-HT1B](http://flybase.bio.indiana.edu/.bin/fbidq.html?5-HT1B), [synaptogyrin](http://flybase.bio.indiana.edu/.bin/fbidq.html?synaptogyrin), [Apc](http://flybase.bio.indiana.edu/.bin/fbidq.html?Apc), [sim](http://flybase.bio.indiana.edu/.bin/fbidq.html?sim), [CG8795](http://flybase.bio.indiana.edu/.bin/fbidq.html?CG8795), [gro](http://flybase.bio.indiana.edu/.bin/fbidq.html?gro), [RN-tre](http://flybase.bio.indiana.edu/.bin/fbidq.html?RN-tre), [scyl](http://flybase.bio.indiana.edu/.bin/fbidq.html?scyl), [Btk29A](http://flybase.bio.indiana.edu/.bin/fbidq.html?Btk29A), [ETH](http://flybase.bio.indiana.edu/.bin/fbidq.html?ETH), [unc-5](http://flybase.bio.indiana.edu/.bin/fbidq.html?unc-5), [cdi](http://flybase.bio.indiana.edu/.bin/fbidq.html?cdi), [NPFR1](http://flybase.bio.indiana.edu/.bin/fbidq.html?NPFR1), [pygo](http://flybase.bio.indiana.edu/.bin/fbidq.html?pygo), [HLHmgamma](http://flybase.bio.indiana.edu/.bin/fbidq.html?HLHmgamma), [CG15556](http://flybase.bio.indiana.edu/.bin/fbidq.html?CG15556), [Ror](http://flybase.bio.indiana.edu/.bin/fbidq.html?Ror), [tow](http://flybase.bio.indiana.edu/.bin/fbidq.html?tow), [CG15609](http://flybase.bio.indiana.edu/.bin/fbidq.html?CG15609), [wor](http://flybase.bio.indiana.edu/.bin/fbidq.html?wor), [Atg1](http://flybase.bio.indiana.edu/.bin/fbidq.html?Atg1), [CG12187](http://flybase.bio.indiana.edu/.bin/fbidq.html?CG12187), [Pten](http://flybase.bio.indiana.edu/.bin/fbidq.html?Pten), [unc-13](http://flybase.bio.indiana.edu/.bin/fbidq.html?unc-13), [npf](http://flybase.bio.indiana.edu/.bin/fbidq.html?npf), [Ptp69D](http://flybase.bio.indiana.edu/.bin/fbidq.html?Ptp69D), [bowl](http://flybase.bio.indiana.edu/.bin/fbidq.html?bowl), [oa2](http://flybase.bio.indiana.edu/.bin/fbidq.html?oa2), [elk](http://flybase.bio.indiana.edu/.bin/fbidq.html?elk), [phl](http://flybase.bio.indiana.edu/.bin/fbidq.html?phl), [Cbl](http://flybase.bio.indiana.edu/.bin/fbidq.html?Cbl), [Rab26](http://flybase.bio.indiana.edu/.bin/fbidq.html?Rab26),[Pde6](http://flybase.bio.indiana.edu/.bin/fbidq.html?Pde6), [sNPF](http://flybase.bio.indiana.edu/.bin/fbidq.html?sNPF), [pog](http://flybase.bio.indiana.edu/.bin/fbidq.html?pog), [plx](http://flybase.bio.indiana.edu/.bin/fbidq.html?plx), [CASK](http://flybase.bio.indiana.edu/.bin/fbidq.html?CASK), [CG9098](http://flybase.bio.indiana.edu/.bin/fbidq.html?CG9098), [CG8108](http://flybase.bio.indiana.edu/.bin/fbidq.html?CG8108), [IFa](http://flybase.bio.indiana.edu/.bin/fbidq.html?IFa), [mtt](http://flybase.bio.indiana.edu/.bin/fbidq.html?mtt), [CG32758](http://flybase.bio.indiana.edu/.bin/fbidq.html?CG32758), [Alk](http://flybase.bio.indiana.edu/.bin/fbidq.html?Alk), [CG7708](http://flybase.bio.indiana.edu/.bin/fbidq.html?CG7708), [Pde1c](http://flybase.bio.indiana.edu/.bin/fbidq.html?Pde1c), [disp](http://flybase.bio.indiana.edu/.bin/fbidq.html?disp), [cpx](http://flybase.bio.indiana.edu/.bin/fbidq.html?cpx), [casp](http://flybase.bio.indiana.edu/.bin/fbidq.html?casp), [CG4022](http://flybase.bio.indiana.edu/.bin/fbidq.html?CG4022), [Smox](http://flybase.bio.indiana.edu/.bin/fbidq.html?Smox), [gfA](http://flybase.bio.indiana.edu/.bin/fbidq.html?gfA), [Plc21C](http://flybase.bio.indiana.edu/.bin/fbidq.html?Plc21C), [CG31781](http://flybase.bio.indiana.edu/.bin/fbidq.html?CG31781), [Rph](http://flybase.bio.indiana.edu/.bin/fbidq.html?Rph), [srpk79D](http://flybase.bio.indiana.edu/.bin/fbidq.html?srpk79D), [GRHRII](http://flybase.bio.indiana.edu/.bin/fbidq.html?GRHRII), [Rdl](http://flybase.bio.indiana.edu/.bin/fbidq.html?Rdl), [CG10362](http://flybase.bio.indiana.edu/.bin/fbidq.html?CG10362), [GABA-B-R2](http://flybase.bio.indiana.edu/.bin/fbidq.html?GABA-B-R2), [Ast-C](http://flybase.bio.indiana.edu/.bin/fbidq.html?Ast-C), [Gbeta5](http://flybase.bio.indiana.edu/.bin/fbidq.html?Gbeta5), [cindr](http://flybase.bio.indiana.edu/.bin/fbidq.html?cindr), [futsch](http://flybase.bio.indiana.edu/.bin/fbidq.html?futsch), [Rim](http://flybase.bio.indiana.edu/.bin/fbidq.html?Rim), [Shal](http://flybase.bio.indiana.edu/.bin/fbidq.html?Shal),[hug](http://flybase.bio.indiana.edu/.bin/fbidq.html?hug), [para](http://flybase.bio.indiana.edu/.bin/fbidq.html?para), [Sur-8](http://flybase.bio.indiana.edu/.bin/fbidq.html?Sur-8), [CG11597](http://flybase.bio.indiana.edu/.bin/fbidq.html?CG11597), [Syn2](http://flybase.bio.indiana.edu/.bin/fbidq.html?Syn2), [Rgl](http://flybase.bio.indiana.edu/.bin/fbidq.html?Rgl), [e(y)3](http://flybase.bio.indiana.edu/.bin/fbidq.html?e(y)3), [Nrx-1](http://flybase.bio.indiana.edu/.bin/fbidq.html?Nrx-1), [CRMP](http://flybase.bio.indiana.edu/.bin/fbidq.html?CRMP), [Cdk5](http://flybase.bio.indiana.edu/.bin/fbidq.html?Cdk5), [Cep135](http://flybase.bio.indiana.edu/.bin/fbidq.html?Cep135), [mav](http://flybase.bio.indiana.edu/.bin/fbidq.html?mav), [CG33203](http://flybase.bio.indiana.edu/.bin/fbidq.html?CG33203), [CG4641](http://flybase.bio.indiana.edu/.bin/fbidq.html?CG4641), [Ilp7](http://flybase.bio.indiana.edu/.bin/fbidq.html?Ilp7), [ey](http://flybase.bio.indiana.edu/.bin/fbidq.html?ey), [CG31122](http://flybase.bio.indiana.edu/.bin/fbidq.html?CG31122), [CG15439](http://flybase.bio.indiana.edu/.bin/fbidq.html?CG15439), [Mip](http://flybase.bio.indiana.edu/.bin/fbidq.html?Mip), [baz](http://flybase.bio.indiana.edu/.bin/fbidq.html?baz), [CG33275](http://flybase.bio.indiana.edu/.bin/fbidq.html?CG33275), [Oamb](http://flybase.bio.indiana.edu/.bin/fbidq.html?Oamb), [CG34381](http://flybase.bio.indiana.edu/.bin/fbidq.html?CG34381), [rst](http://flybase.bio.indiana.edu/.bin/fbidq.html?rst), [Cdk5alpha](http://flybase.bio.indiana.edu/.bin/fbidq.html?Cdk5alpha), [CG40351](http://flybase.bio.indiana.edu/.bin/fbidq.html?CG40351), [Vmat](http://flybase.bio.indiana.edu/.bin/fbidq.html?Vmat), [qtc](http://flybase.bio.indiana.edu/.bin/fbidq.html?qtc), [Cha](http://flybase.bio.indiana.edu/.bin/fbidq.html?Cha), [crol](http://flybase.bio.indiana.edu/.bin/fbidq.html?crol), [sd](http://flybase.bio.indiana.edu/.bin/fbidq.html?sd), [Aplip1](http://flybase.bio.indiana.edu/.bin/fbidq.html?Aplip1), [Ac3](http://flybase.bio.indiana.edu/.bin/fbidq.html?Ac3), [rl](http://flybase.bio.indiana.edu/.bin/fbidq.html?rl),[Syt1](http://flybase.bio.indiana.edu/.bin/fbidq.html?Syt1), [CG17760](http://flybase.bio.indiana.edu/.bin/fbidq.html?CG17760), [Nup153](http://flybase.bio.indiana.edu/.bin/fbidq.html?Nup153), [CG32683](http://flybase.bio.indiana.edu/.bin/fbidq.html?CG32683), [CG11347](http://flybase.bio.indiana.edu/.bin/fbidq.html?CG11347), [Ggamma30A](http://flybase.bio.indiana.edu/.bin/fbidq.html?Ggamma30A), [wls](http://flybase.bio.indiana.edu/.bin/fbidq.html?wls), [hiw](http://flybase.bio.indiana.edu/.bin/fbidq.html?hiw), [Atf-2](http://flybase.bio.indiana.edu/.bin/fbidq.html?Atf-2), [n-syb](http://flybase.bio.indiana.edu/.bin/fbidq.html?n-syb), [skd](http://flybase.bio.indiana.edu/.bin/fbidq.html?skd), [Camta](http://flybase.bio.indiana.edu/.bin/fbidq.html?Camta), [tll](http://flybase.bio.indiana.edu/.bin/fbidq.html?tll), [ato](http://flybase.bio.indiana.edu/.bin/fbidq.html?ato), [Fmrf](http://flybase.bio.indiana.edu/.bin/fbidq.html?Fmrf), [Iswi](http://flybase.bio.indiana.edu/.bin/fbidq.html?Iswi), [Dg](http://flybase.bio.indiana.edu/.bin/fbidq.html?Dg), [Trim9](http://flybase.bio.indiana.edu/.bin/fbidq.html?Trim9), [GABA-B-R3](http://flybase.bio.indiana.edu/.bin/fbidq.html?GABA-B-R3), [Pka-R1](http://flybase.bio.indiana.edu/.bin/fbidq.html?Pka-R1), [Evi5](http://flybase.bio.indiana.edu/.bin/fbidq.html?Evi5), [mago](http://flybase.bio.indiana.edu/.bin/fbidq.html?mago), [Gycalpha99B](http://flybase.bio.indiana.edu/.bin/fbidq.html?Gycalpha99B), [ksr](http://flybase.bio.indiana.edu/.bin/fbidq.html?ksr), [CG42533](http://flybase.bio.indiana.edu/.bin/fbidq.html?CG42533), [CG11376](http://flybase.bio.indiana.edu/.bin/fbidq.html?CG11376), [Gyc-89Da](http://flybase.bio.indiana.edu/.bin/fbidq.html?Gyc-89Da), [brp](http://flybase.bio.indiana.edu/.bin/fbidq.html?brp), [unc-104](http://flybase.bio.indiana.edu/.bin/fbidq.html?unc-104), [Khc-73](http://flybase.bio.indiana.edu/.bin/fbidq.html?Khc-73), [D2R](http://flybase.bio.indiana.edu/.bin/fbidq.html?D2R),[CG31158](http://flybase.bio.indiana.edu/.bin/fbidq.html?CG31158), [metro](http://flybase.bio.indiana.edu/.bin/fbidq.html?metro), [ewg](http://flybase.bio.indiana.edu/.bin/fbidq.html?ewg), [Nrk](http://flybase.bio.indiana.edu/.bin/fbidq.html?Nrk), [Cirl](http://flybase.bio.indiana.edu/.bin/fbidq.html?Cirl), [Ilp3](http://flybase.bio.indiana.edu/.bin/fbidq.html?Ilp3), [Su(var)3-3](http://flybase.bio.indiana.edu/.bin/fbidq.html?Su(var)3-3), [Bili](http://flybase.bio.indiana.edu/.bin/fbidq.html?Bili), [Nrg](http://flybase.bio.indiana.edu/.bin/fbidq.html?Nrg), [unc-13-4A](http://flybase.bio.indiana.edu/.bin/fbidq.html?unc-13-4A), [CG34384](http://flybase.bio.indiana.edu/.bin/fbidq.html?CG34384), [stet](http://flybase.bio.indiana.edu/.bin/fbidq.html?stet), [Dh44-R1](http://flybase.bio.indiana.edu/.bin/fbidq.html?Dh44-R1), [CG32447](http://flybase.bio.indiana.edu/.bin/fbidq.html?CG32447), [Eh](http://flybase.bio.indiana.edu/.bin/fbidq.html?Eh), [Sema-1a](http://flybase.bio.indiana.edu/.bin/fbidq.html?Sema-1a), [Hs3st-B](http://flybase.bio.indiana.edu/.bin/fbidq.html?Hs3st-B), [otk](http://flybase.bio.indiana.edu/.bin/fbidq.html?otk), [Sos](http://flybase.bio.indiana.edu/.bin/fbidq.html?Sos), [phyl](http://flybase.bio.indiana.edu/.bin/fbidq.html?phyl), [vg](http://flybase.bio.indiana.edu/.bin/fbidq.html?vg), [Fas2](http://flybase.bio.indiana.edu/.bin/fbidq.html?Fas2), [fz](http://flybase.bio.indiana.edu/.bin/fbidq.html?fz), [robo](http://flybase.bio.indiana.edu/.bin/fbidq.html?robo), [CG8557](http://flybase.bio.indiana.edu/.bin/fbidq.html?CG8557), [bsk](http://flybase.bio.indiana.edu/.bin/fbidq.html?bsk), [brm](http://flybase.bio.indiana.edu/.bin/fbidq.html?brm), [mAcR-60C](http://flybase.bio.indiana.edu/.bin/fbidq.html?mAcR-60C), [Crz](http://flybase.bio.indiana.edu/.bin/fbidq.html?Crz), [ft](http://flybase.bio.indiana.edu/.bin/fbidq.html?ft), [mtg](http://flybase.bio.indiana.edu/.bin/fbidq.html?mtg), [CG13830](http://flybase.bio.indiana.edu/.bin/fbidq.html?CG13830), [sno](http://flybase.bio.indiana.edu/.bin/fbidq.html?sno), [RhoGAPp190](http://flybase.bio.indiana.edu/.bin/fbidq.html?RhoGAPp190),[l(2)k16918](http://flybase.bio.indiana.edu/.bin/fbidq.html?l(2)k16918), [mam](http://flybase.bio.indiana.edu/.bin/fbidq.html?mam), [CG30158](http://flybase.bio.indiana.edu/.bin/fbidq.html?CG30158), [Takr86C](http://flybase.bio.indiana.edu/.bin/fbidq.html?Takr86C), [pan](http://flybase.bio.indiana.edu/.bin/fbidq.html?pan), [PKD](http://flybase.bio.indiana.edu/.bin/fbidq.html?PKD), [DopR](http://flybase.bio.indiana.edu/.bin/fbidq.html?DopR), [Lis-1](http://flybase.bio.indiana.edu/.bin/fbidq.html?Lis-1), [CG16896](http://flybase.bio.indiana.edu/.bin/fbidq.html?CG16896), [Rab3](http://flybase.bio.indiana.edu/.bin/fbidq.html?Rab3), [tup](http://flybase.bio.indiana.edu/.bin/fbidq.html?tup), [mthl8](http://flybase.bio.indiana.edu/.bin/fbidq.html?mthl8), [trio](http://flybase.bio.indiana.edu/.bin/fbidq.html?trio), [RabX4](http://flybase.bio.indiana.edu/.bin/fbidq.html?RabX4), [spel1](http://flybase.bio.indiana.edu/.bin/fbidq.html?spel1), [Lkr](http://flybase.bio.indiana.edu/.bin/fbidq.html?Lkr), [CG31665](http://flybase.bio.indiana.edu/.bin/fbidq.html?CG31665), [Ccap](http://flybase.bio.indiana.edu/.bin/fbidq.html?Ccap), [Gad1](http://flybase.bio.indiana.edu/.bin/fbidq.html?Gad1), [Wnt4](http://flybase.bio.indiana.edu/.bin/fbidq.html?Wnt4), [CG7918](http://flybase.bio.indiana.edu/.bin/fbidq.html?CG7918), [CaMKII](http://flybase.bio.indiana.edu/.bin/fbidq.html?CaMKII), [DopR2](http://flybase.bio.indiana.edu/.bin/fbidq.html?DopR2), [CG30372](http://flybase.bio.indiana.edu/.bin/fbidq.html?CG30372), [CG10188](http://flybase.bio.indiana.edu/.bin/fbidq.html?CG10188), [CG42629](http://flybase.bio.indiana.edu/.bin/fbidq.html?CG42629), [pigs](http://flybase.bio.indiana.edu/.bin/fbidq.html?pigs), [CG31760](http://flybase.bio.indiana.edu/.bin/fbidq.html?CG31760), [Klp54D](http://flybase.bio.indiana.edu/.bin/fbidq.html?Klp54D), [bchs](http://flybase.bio.indiana.edu/.bin/fbidq.html?bchs), [5-HT7](http://flybase.bio.indiana.edu/.bin/fbidq.html?5-HT7), [Lgr3](http://flybase.bio.indiana.edu/.bin/fbidq.html?Lgr3), [Dh](http://flybase.bio.indiana.edu/.bin/fbidq.html?Dh), [Syn1](http://flybase.bio.indiana.edu/.bin/fbidq.html?Syn1), [Ac13E](http://flybase.bio.indiana.edu/.bin/fbidq.html?Ac13E), [klu](http://flybase.bio.indiana.edu/.bin/fbidq.html?klu), [SPR](http://flybase.bio.indiana.edu/.bin/fbidq.html?SPR), [CG8155](http://flybase.bio.indiana.edu/.bin/fbidq.html?CG8155), [sr](http://flybase.bio.indiana.edu/.bin/fbidq.html?sr), [nkd](http://flybase.bio.indiana.edu/.bin/fbidq.html?nkd), [tld](http://flybase.bio.indiana.edu/.bin/fbidq.html?tld), [RhoGEF3](http://flybase.bio.indiana.edu/.bin/fbidq.html?RhoGEF3), [Dsk](http://flybase.bio.indiana.edu/.bin/fbidq.html?Dsk), [RhoGEF4](http://flybase.bio.indiana.edu/.bin/fbidq.html?RhoGEF4), [CG9121](http://flybase.bio.indiana.edu/.bin/fbidq.html?CG9121), [betaInt-nu](http://flybase.bio.indiana.edu/.bin/fbidq.html?betaInt-nu), [CG1909](http://flybase.bio.indiana.edu/.bin/fbidq.html?CG1909), [Brd](http://flybase.bio.indiana.edu/.bin/fbidq.html?Brd), [sgg](http://flybase.bio.indiana.edu/.bin/fbidq.html?sgg), [Nmdar1](http://flybase.bio.indiana.edu/.bin/fbidq.html?Nmdar1), [Eip93F](http://flybase.bio.indiana.edu/.bin/fbidq.html?Eip93F), [inaE](http://flybase.bio.indiana.edu/.bin/fbidq.html?inaE), [CanB](http://flybase.bio.indiana.edu/.bin/fbidq.html?CanB), [Pask](http://flybase.bio.indiana.edu/.bin/fbidq.html?Pask), [rab3-GEF](http://flybase.bio.indiana.edu/.bin/fbidq.html?rab3-GEF), [CG13253](http://flybase.bio.indiana.edu/.bin/fbidq.html?CG13253), [MESK2](http://flybase.bio.indiana.edu/.bin/fbidq.html?MESK2), [Syt4](http://flybase.bio.indiana.edu/.bin/fbidq.html?Syt4), [Dat](http://flybase.bio.indiana.edu/.bin/fbidq.html?Dat), [SIFR](http://flybase.bio.indiana.edu/.bin/fbidq.html?SIFR), [CG2061](http://flybase.bio.indiana.edu/.bin/fbidq.html?CG2061), [Ast](http://flybase.bio.indiana.edu/.bin/fbidq.html?Ast), [fred](http://flybase.bio.indiana.edu/.bin/fbidq.html?fred), [Drl-2](http://flybase.bio.indiana.edu/.bin/fbidq.html?Drl-2),[ush](http://flybase.bio.indiana.edu/.bin/fbidq.html?ush), [Tk](http://flybase.bio.indiana.edu/.bin/fbidq.html?Tk), [Fak56D](http://flybase.bio.indiana.edu/.bin/fbidq.html?Fak56D), [Ilp5](http://flybase.bio.indiana.edu/.bin/fbidq.html?Ilp5), [Gap1](http://flybase.bio.indiana.edu/.bin/fbidq.html?Gap1), [Toll-6](http://flybase.bio.indiana.edu/.bin/fbidq.html?Toll-6), [CG3227](http://flybase.bio.indiana.edu/.bin/fbidq.html?CG3227), [MESR4](http://flybase.bio.indiana.edu/.bin/fbidq.html?MESR4), [Takr99D](http://flybase.bio.indiana.edu/.bin/fbidq.html?Takr99D), [sNPF-R](http://flybase.bio.indiana.edu/.bin/fbidq.html?sNPF-R), [tara](http://flybase.bio.indiana.edu/.bin/fbidq.html?tara), [Hel89B](http://flybase.bio.indiana.edu/.bin/fbidq.html?Hel89B), [CG5337](http://flybase.bio.indiana.edu/.bin/fbidq.html?CG5337), [CG13692](http://flybase.bio.indiana.edu/.bin/fbidq.html?CG13692), [koko](http://flybase.bio.indiana.edu/.bin/fbidq.html?koko), [stan](http://flybase.bio.indiana.edu/.bin/fbidq.html?stan), [CG12424](http://flybase.bio.indiana.edu/.bin/fbidq.html?CG12424), [tutl](http://flybase.bio.indiana.edu/.bin/fbidq.html?tutl), [Ptth](http://flybase.bio.indiana.edu/.bin/fbidq.html?Ptth), [Rab2](http://flybase.bio.indiana.edu/.bin/fbidq.html?Rab2), [Ilp2](http://flybase.bio.indiana.edu/.bin/fbidq.html?Ilp2), [Frq1](http://flybase.bio.indiana.edu/.bin/fbidq.html?Frq1), [SPoCk](http://flybase.bio.indiana.edu/.bin/fbidq.html?SPoCk), [sna](http://flybase.bio.indiana.edu/.bin/fbidq.html?sna), [Wnt5](http://flybase.bio.indiana.edu/.bin/fbidq.html?Wnt5), [Taf1](http://flybase.bio.indiana.edu/.bin/fbidq.html?Taf1), [Ggamma1](http://flybase.bio.indiana.edu/.bin/fbidq.html?Ggamma1), [fus](http://flybase.bio.indiana.edu/.bin/fbidq.html?fus), [sif](http://flybase.bio.indiana.edu/.bin/fbidq.html?sif), [RSG7](http://flybase.bio.indiana.edu/.bin/fbidq.html?RSG7), [m4](http://flybase.bio.indiana.edu/.bin/fbidq.html?m4), [dom](http://flybase.bio.indiana.edu/.bin/fbidq.html?dom), [Tbh](http://flybase.bio.indiana.edu/.bin/fbidq.html?Tbh), [Dh31](http://flybase.bio.indiana.edu/.bin/fbidq.html?Dh31),[CG42541](http://flybase.bio.indiana.edu/.bin/fbidq.html?CG42541), [PIP5K59B](http://flybase.bio.indiana.edu/.bin/fbidq.html?PIP5K59B), [Hr51](http://flybase.bio.indiana.edu/.bin/fbidq.html?Hr51), [lap](http://flybase.bio.indiana.edu/.bin/fbidq.html?lap), [shi](http://flybase.bio.indiana.edu/.bin/fbidq.html?shi), [CG11155](http://flybase.bio.indiana.edu/.bin/fbidq.html?CG11155), [Spred](http://flybase.bio.indiana.edu/.bin/fbidq.html?Spred), [siz](http://flybase.bio.indiana.edu/.bin/fbidq.html?siz), [B-H2](http://flybase.bio.indiana.edu/.bin/fbidq.html?B-H2), [CG3822](http://flybase.bio.indiana.edu/.bin/fbidq.html?CG3822), [wg](http://flybase.bio.indiana.edu/.bin/fbidq.html?wg), [CadN](http://flybase.bio.indiana.edu/.bin/fbidq.html?CadN), [Rhp](http://flybase.bio.indiana.edu/.bin/fbidq.html?Rhp), [CG13995](http://flybase.bio.indiana.edu/.bin/fbidq.html?CG13995), [E(bx)](http://flybase.bio.indiana.edu/.bin/fbidq.html?E(bx)), [sfl](http://flybase.bio.indiana.edu/.bin/fbidq.html?sfl), [CG13229](http://flybase.bio.indiana.edu/.bin/fbidq.html?CG13229), [Pde11](http://flybase.bio.indiana.edu/.bin/fbidq.html?Pde11), [Dms](http://flybase.bio.indiana.edu/.bin/fbidq.html?Dms), [Snap](http://flybase.bio.indiana.edu/.bin/fbidq.html?Snap), [SerT](http://flybase.bio.indiana.edu/.bin/fbidq.html?SerT), [Utx](http://flybase.bio.indiana.edu/.bin/fbidq.html?Utx), [pburs](http://flybase.bio.indiana.edu/.bin/fbidq.html?pburs), [cos](http://flybase.bio.indiana.edu/.bin/fbidq.html?cos), [Ct](http://flybase.bio.indiana.edu/.bin/fbidq.html?Ct) |
| [neuron differentiation](http://amigo.geneontology.org/cgi-bin/amigo/go.cgi?view=details&query=GO:0030182) | 192 of 1594 genes, 12.0% | 582 of 7634 genes, 7.6% | 1.01e-11 | 0.00% | 0.00 | [fz2](http://flybase.bio.indiana.edu/.bin/fbidq.html?fz2), [Sh](http://flybase.bio.indiana.edu/.bin/fbidq.html?Sh), [acj6](http://flybase.bio.indiana.edu/.bin/fbidq.html?acj6), [jing](http://flybase.bio.indiana.edu/.bin/fbidq.html?jing), [dock](http://flybase.bio.indiana.edu/.bin/fbidq.html?dock), [CG42256](http://flybase.bio.indiana.edu/.bin/fbidq.html?CG42256), [CG3703](http://flybase.bio.indiana.edu/.bin/fbidq.html?CG3703), [vimar](http://flybase.bio.indiana.edu/.bin/fbidq.html?vimar), [ato](http://flybase.bio.indiana.edu/.bin/fbidq.html?ato), [Iswi](http://flybase.bio.indiana.edu/.bin/fbidq.html?Iswi), [ap](http://flybase.bio.indiana.edu/.bin/fbidq.html?ap), [Dg](http://flybase.bio.indiana.edu/.bin/fbidq.html?Dg), [Trim9](http://flybase.bio.indiana.edu/.bin/fbidq.html?Trim9), [br](http://flybase.bio.indiana.edu/.bin/fbidq.html?br), [CG7154](http://flybase.bio.indiana.edu/.bin/fbidq.html?CG7154), [robo3](http://flybase.bio.indiana.edu/.bin/fbidq.html?robo3), [os](http://flybase.bio.indiana.edu/.bin/fbidq.html?os), [tok](http://flybase.bio.indiana.edu/.bin/fbidq.html?tok), [Ptp99A](http://flybase.bio.indiana.edu/.bin/fbidq.html?Ptp99A), [lola](http://flybase.bio.indiana.edu/.bin/fbidq.html?lola), [unc-104](http://flybase.bio.indiana.edu/.bin/fbidq.html?unc-104), [chif](http://flybase.bio.indiana.edu/.bin/fbidq.html?chif), [NetB](http://flybase.bio.indiana.edu/.bin/fbidq.html?NetB), [p130CAS](http://flybase.bio.indiana.edu/.bin/fbidq.html?p130CAS), [Tango10](http://flybase.bio.indiana.edu/.bin/fbidq.html?Tango10), [CG31158](http://flybase.bio.indiana.edu/.bin/fbidq.html?CG31158), [Appl](http://flybase.bio.indiana.edu/.bin/fbidq.html?Appl), [metro](http://flybase.bio.indiana.edu/.bin/fbidq.html?metro), [CG32137](http://flybase.bio.indiana.edu/.bin/fbidq.html?CG32137), [Nrk](http://flybase.bio.indiana.edu/.bin/fbidq.html?Nrk), [CG4328](http://flybase.bio.indiana.edu/.bin/fbidq.html?CG4328), [Galpha49B](http://flybase.bio.indiana.edu/.bin/fbidq.html?Galpha49B), [Snoo](http://flybase.bio.indiana.edu/.bin/fbidq.html?Snoo), [Lar](http://flybase.bio.indiana.edu/.bin/fbidq.html?Lar), [Taf4](http://flybase.bio.indiana.edu/.bin/fbidq.html?Taf4),[CG10249](http://flybase.bio.indiana.edu/.bin/fbidq.html?CG10249), [scrib](http://flybase.bio.indiana.edu/.bin/fbidq.html?scrib), [HGTX](http://flybase.bio.indiana.edu/.bin/fbidq.html?HGTX), [not](http://flybase.bio.indiana.edu/.bin/fbidq.html?not), [rut](http://flybase.bio.indiana.edu/.bin/fbidq.html?rut), [nvy](http://flybase.bio.indiana.edu/.bin/fbidq.html?nvy), [Nrg](http://flybase.bio.indiana.edu/.bin/fbidq.html?Nrg), [gogo](http://flybase.bio.indiana.edu/.bin/fbidq.html?gogo), [nab](http://flybase.bio.indiana.edu/.bin/fbidq.html?nab), [HLHmdelta](http://flybase.bio.indiana.edu/.bin/fbidq.html?HLHmdelta), [jeb](http://flybase.bio.indiana.edu/.bin/fbidq.html?jeb), [B-H1](http://flybase.bio.indiana.edu/.bin/fbidq.html?B-H1), [GluClalpha](http://flybase.bio.indiana.edu/.bin/fbidq.html?GluClalpha), [alph](http://flybase.bio.indiana.edu/.bin/fbidq.html?alph), [Actbeta](http://flybase.bio.indiana.edu/.bin/fbidq.html?Actbeta), [Sema-1a](http://flybase.bio.indiana.edu/.bin/fbidq.html?Sema-1a), [beat-Ib](http://flybase.bio.indiana.edu/.bin/fbidq.html?beat-Ib), [TBPH](http://flybase.bio.indiana.edu/.bin/fbidq.html?TBPH), [otk](http://flybase.bio.indiana.edu/.bin/fbidq.html?otk), [Sos](http://flybase.bio.indiana.edu/.bin/fbidq.html?Sos), [CG34400](http://flybase.bio.indiana.edu/.bin/fbidq.html?CG34400), [phyl](http://flybase.bio.indiana.edu/.bin/fbidq.html?phyl), [Ulp1](http://flybase.bio.indiana.edu/.bin/fbidq.html?Ulp1), [ena](http://flybase.bio.indiana.edu/.bin/fbidq.html?ena), [dsh](http://flybase.bio.indiana.edu/.bin/fbidq.html?dsh), [hth](http://flybase.bio.indiana.edu/.bin/fbidq.html?hth), [Fas2](http://flybase.bio.indiana.edu/.bin/fbidq.html?Fas2), [Nf-YC](http://flybase.bio.indiana.edu/.bin/fbidq.html?Nf-YC), [eya](http://flybase.bio.indiana.edu/.bin/fbidq.html?eya), [14-3-3zeta](http://flybase.bio.indiana.edu/.bin/fbidq.html?14-3-3zeta), [fz](http://flybase.bio.indiana.edu/.bin/fbidq.html?fz), [robo](http://flybase.bio.indiana.edu/.bin/fbidq.html?robo), [lea](http://flybase.bio.indiana.edu/.bin/fbidq.html?lea), [brm](http://flybase.bio.indiana.edu/.bin/fbidq.html?brm), [bsk](http://flybase.bio.indiana.edu/.bin/fbidq.html?bsk), [beat-Ia](http://flybase.bio.indiana.edu/.bin/fbidq.html?beat-Ia), [kat-60L1](http://flybase.bio.indiana.edu/.bin/fbidq.html?kat-60L1), [hig](http://flybase.bio.indiana.edu/.bin/fbidq.html?hig), [RhoGAPp190](http://flybase.bio.indiana.edu/.bin/fbidq.html?RhoGAPp190), [CG12004](http://flybase.bio.indiana.edu/.bin/fbidq.html?CG12004), [plexB](http://flybase.bio.indiana.edu/.bin/fbidq.html?plexB), [Klp64D](http://flybase.bio.indiana.edu/.bin/fbidq.html?Klp64D), [grn](http://flybase.bio.indiana.edu/.bin/fbidq.html?grn), [DAAM](http://flybase.bio.indiana.edu/.bin/fbidq.html?DAAM), [mew](http://flybase.bio.indiana.edu/.bin/fbidq.html?mew), [neur](http://flybase.bio.indiana.edu/.bin/fbidq.html?neur), [didum](http://flybase.bio.indiana.edu/.bin/fbidq.html?didum), [Lis-1](http://flybase.bio.indiana.edu/.bin/fbidq.html?Lis-1), [CycE](http://flybase.bio.indiana.edu/.bin/fbidq.html?CycE), [Brf](http://flybase.bio.indiana.edu/.bin/fbidq.html?Brf), [ems](http://flybase.bio.indiana.edu/.bin/fbidq.html?ems), [eys](http://flybase.bio.indiana.edu/.bin/fbidq.html?eys), [Mob2](http://flybase.bio.indiana.edu/.bin/fbidq.html?Mob2), [pros](http://flybase.bio.indiana.edu/.bin/fbidq.html?pros), [tup](http://flybase.bio.indiana.edu/.bin/fbidq.html?tup), [trio](http://flybase.bio.indiana.edu/.bin/fbidq.html?trio), [CG1463](http://flybase.bio.indiana.edu/.bin/fbidq.html?CG1463), [daw](http://flybase.bio.indiana.edu/.bin/fbidq.html?daw), [Wnt4](http://flybase.bio.indiana.edu/.bin/fbidq.html?Wnt4), [rictor](http://flybase.bio.indiana.edu/.bin/fbidq.html?rictor), [Apc](http://flybase.bio.indiana.edu/.bin/fbidq.html?Apc), [sim](http://flybase.bio.indiana.edu/.bin/fbidq.html?sim), [d4](http://flybase.bio.indiana.edu/.bin/fbidq.html?d4), [gro](http://flybase.bio.indiana.edu/.bin/fbidq.html?gro), [nerfin-1](http://flybase.bio.indiana.edu/.bin/fbidq.html?nerfin-1), [bchs](http://flybase.bio.indiana.edu/.bin/fbidq.html?bchs), [wnd](http://flybase.bio.indiana.edu/.bin/fbidq.html?wnd), [trx](http://flybase.bio.indiana.edu/.bin/fbidq.html?trx), [Rpd3](http://flybase.bio.indiana.edu/.bin/fbidq.html?Rpd3), [dsx](http://flybase.bio.indiana.edu/.bin/fbidq.html?dsx), [chinmo](http://flybase.bio.indiana.edu/.bin/fbidq.html?chinmo), [unc-5](http://flybase.bio.indiana.edu/.bin/fbidq.html?unc-5), [Ssdp](http://flybase.bio.indiana.edu/.bin/fbidq.html?Ssdp), [cdi](http://flybase.bio.indiana.edu/.bin/fbidq.html?cdi),[CG10107](http://flybase.bio.indiana.edu/.bin/fbidq.html?CG10107), [sqz](http://flybase.bio.indiana.edu/.bin/fbidq.html?sqz), [fend](http://flybase.bio.indiana.edu/.bin/fbidq.html?fend), [pygo](http://flybase.bio.indiana.edu/.bin/fbidq.html?pygo), [HLHm7](http://flybase.bio.indiana.edu/.bin/fbidq.html?HLHm7), [wor](http://flybase.bio.indiana.edu/.bin/fbidq.html?wor), [vvl](http://flybase.bio.indiana.edu/.bin/fbidq.html?vvl), [Pten](http://flybase.bio.indiana.edu/.bin/fbidq.html?Pten), [Ptp69D](http://flybase.bio.indiana.edu/.bin/fbidq.html?Ptp69D), [gcm2](http://flybase.bio.indiana.edu/.bin/fbidq.html?gcm2), [hts](http://flybase.bio.indiana.edu/.bin/fbidq.html?hts), [mp](http://flybase.bio.indiana.edu/.bin/fbidq.html?mp), [gcm](http://flybase.bio.indiana.edu/.bin/fbidq.html?gcm), [seq](http://flybase.bio.indiana.edu/.bin/fbidq.html?seq), [CG5890](http://flybase.bio.indiana.edu/.bin/fbidq.html?CG5890), [Dip3](http://flybase.bio.indiana.edu/.bin/fbidq.html?Dip3), [SP2353](http://flybase.bio.indiana.edu/.bin/fbidq.html?SP2353), [phl](http://flybase.bio.indiana.edu/.bin/fbidq.html?phl), [Cbl](http://flybase.bio.indiana.edu/.bin/fbidq.html?Cbl), [CG32685](http://flybase.bio.indiana.edu/.bin/fbidq.html?CG32685), [CG4893](http://flybase.bio.indiana.edu/.bin/fbidq.html?CG4893), [CG9098](http://flybase.bio.indiana.edu/.bin/fbidq.html?CG9098), [Alk](http://flybase.bio.indiana.edu/.bin/fbidq.html?Alk), [esn](http://flybase.bio.indiana.edu/.bin/fbidq.html?esn), [Vang](http://flybase.bio.indiana.edu/.bin/fbidq.html?Vang), [Sin3A](http://flybase.bio.indiana.edu/.bin/fbidq.html?Sin3A), [pot](http://flybase.bio.indiana.edu/.bin/fbidq.html?pot), [jumu](http://flybase.bio.indiana.edu/.bin/fbidq.html?jumu), [Smox](http://flybase.bio.indiana.edu/.bin/fbidq.html?Smox), [CG31475](http://flybase.bio.indiana.edu/.bin/fbidq.html?CG31475), [beat-Ic](http://flybase.bio.indiana.edu/.bin/fbidq.html?beat-Ic), [fd59A](http://flybase.bio.indiana.edu/.bin/fbidq.html?fd59A), [Gap1](http://flybase.bio.indiana.edu/.bin/fbidq.html?Gap1), [run](http://flybase.bio.indiana.edu/.bin/fbidq.html?run), [brat](http://flybase.bio.indiana.edu/.bin/fbidq.html?brat), [ko](http://flybase.bio.indiana.edu/.bin/fbidq.html?ko),[Eip71CD](http://flybase.bio.indiana.edu/.bin/fbidq.html?Eip71CD), [stan](http://flybase.bio.indiana.edu/.bin/fbidq.html?stan), [CG12424](http://flybase.bio.indiana.edu/.bin/fbidq.html?CG12424), [tutl](http://flybase.bio.indiana.edu/.bin/fbidq.html?tutl), [futsch](http://flybase.bio.indiana.edu/.bin/fbidq.html?futsch), [Ptx1](http://flybase.bio.indiana.edu/.bin/fbidq.html?Ptx1), [ph-p](http://flybase.bio.indiana.edu/.bin/fbidq.html?ph-p), [gukh](http://flybase.bio.indiana.edu/.bin/fbidq.html?gukh), [chm](http://flybase.bio.indiana.edu/.bin/fbidq.html?chm), [Rim](http://flybase.bio.indiana.edu/.bin/fbidq.html?Rim), [sna](http://flybase.bio.indiana.edu/.bin/fbidq.html?sna), [Wnt5](http://flybase.bio.indiana.edu/.bin/fbidq.html?Wnt5), [beat-IIa](http://flybase.bio.indiana.edu/.bin/fbidq.html?beat-IIa), [sm](http://flybase.bio.indiana.edu/.bin/fbidq.html?sm), [Syn2](http://flybase.bio.indiana.edu/.bin/fbidq.html?Syn2), [dac](http://flybase.bio.indiana.edu/.bin/fbidq.html?dac), [Cdk5](http://flybase.bio.indiana.edu/.bin/fbidq.html?Cdk5), [sif](http://flybase.bio.indiana.edu/.bin/fbidq.html?sif), [oc](http://flybase.bio.indiana.edu/.bin/fbidq.html?oc), [dom](http://flybase.bio.indiana.edu/.bin/fbidq.html?dom), [ey](http://flybase.bio.indiana.edu/.bin/fbidq.html?ey), [ssh](http://flybase.bio.indiana.edu/.bin/fbidq.html?ssh), [baz](http://flybase.bio.indiana.edu/.bin/fbidq.html?baz), [klar](http://flybase.bio.indiana.edu/.bin/fbidq.html?klar), [mle](http://flybase.bio.indiana.edu/.bin/fbidq.html?mle), [Hr51](http://flybase.bio.indiana.edu/.bin/fbidq.html?Hr51), [CG11155](http://flybase.bio.indiana.edu/.bin/fbidq.html?CG11155), [rst](http://flybase.bio.indiana.edu/.bin/fbidq.html?rst), [B-H2](http://flybase.bio.indiana.edu/.bin/fbidq.html?B-H2), [grh](http://flybase.bio.indiana.edu/.bin/fbidq.html?grh), [comm](http://flybase.bio.indiana.edu/.bin/fbidq.html?comm), [Cdk5alpha](http://flybase.bio.indiana.edu/.bin/fbidq.html?Cdk5alpha), [Lim3](http://flybase.bio.indiana.edu/.bin/fbidq.html?Lim3), [CadN](http://flybase.bio.indiana.edu/.bin/fbidq.html?CadN), [Aplip1](http://flybase.bio.indiana.edu/.bin/fbidq.html?Aplip1), [E(Pc)](http://flybase.bio.indiana.edu/.bin/fbidq.html?E(Pc)), [retn](http://flybase.bio.indiana.edu/.bin/fbidq.html?retn), [E(bx)](http://flybase.bio.indiana.edu/.bin/fbidq.html?E(bx)), [en](http://flybase.bio.indiana.edu/.bin/fbidq.html?en),[klg](http://flybase.bio.indiana.edu/.bin/fbidq.html?klg), [rl](http://flybase.bio.indiana.edu/.bin/fbidq.html?rl), [Snap](http://flybase.bio.indiana.edu/.bin/fbidq.html?Snap), [trn](http://flybase.bio.indiana.edu/.bin/fbidq.html?trn), [tey](http://flybase.bio.indiana.edu/.bin/fbidq.html?tey), [yrt](http://flybase.bio.indiana.edu/.bin/fbidq.html?yrt), [CG33960](http://flybase.bio.indiana.edu/.bin/fbidq.html?CG33960), [Ct](http://flybase.bio.indiana.edu/.bin/fbidq.html?Ct) |
| [regulation of nucleobase-containing compound metabolic process](http://amigo.geneontology.org/cgi-bin/amigo/go.cgi?view=details&query=GO:0019219) | 229 of 1594 genes, 14.4% | 731 of 7634 genes, 9.6% | 1.19e-11 | 0.00% | 0.00 | [fz2](http://flybase.bio.indiana.edu/.bin/fbidq.html?fz2), [acj6](http://flybase.bio.indiana.edu/.bin/fbidq.html?acj6), [skd](http://flybase.bio.indiana.edu/.bin/fbidq.html?skd), [jing](http://flybase.bio.indiana.edu/.bin/fbidq.html?jing), [caup](http://flybase.bio.indiana.edu/.bin/fbidq.html?caup), [tll](http://flybase.bio.indiana.edu/.bin/fbidq.html?tll), [Camta](http://flybase.bio.indiana.edu/.bin/fbidq.html?Camta), [ato](http://flybase.bio.indiana.edu/.bin/fbidq.html?ato), [Ets65A](http://flybase.bio.indiana.edu/.bin/fbidq.html?Ets65A), [Rox8](http://flybase.bio.indiana.edu/.bin/fbidq.html?Rox8), [tna](http://flybase.bio.indiana.edu/.bin/fbidq.html?tna), [Sxl](http://flybase.bio.indiana.edu/.bin/fbidq.html?Sxl), [mub](http://flybase.bio.indiana.edu/.bin/fbidq.html?mub), [JIL-1](http://flybase.bio.indiana.edu/.bin/fbidq.html?JIL-1), [Spt6](http://flybase.bio.indiana.edu/.bin/fbidq.html?Spt6), [Iswi](http://flybase.bio.indiana.edu/.bin/fbidq.html?Iswi), [rn](http://flybase.bio.indiana.edu/.bin/fbidq.html?rn), [ap](http://flybase.bio.indiana.edu/.bin/fbidq.html?ap), [cenG1A](http://flybase.bio.indiana.edu/.bin/fbidq.html?cenG1A), [Nf1](http://flybase.bio.indiana.edu/.bin/fbidq.html?Nf1), [Evi5](http://flybase.bio.indiana.edu/.bin/fbidq.html?Evi5), [br](http://flybase.bio.indiana.edu/.bin/fbidq.html?br), [ksr](http://flybase.bio.indiana.edu/.bin/fbidq.html?ksr), [bi](http://flybase.bio.indiana.edu/.bin/fbidq.html?bi), [chn](http://flybase.bio.indiana.edu/.bin/fbidq.html?chn), [Gsc](http://flybase.bio.indiana.edu/.bin/fbidq.html?Gsc), [Hcf](http://flybase.bio.indiana.edu/.bin/fbidq.html?Hcf), [lola](http://flybase.bio.indiana.edu/.bin/fbidq.html?lola), [HLH4C](http://flybase.bio.indiana.edu/.bin/fbidq.html?HLH4C), [Pcl](http://flybase.bio.indiana.edu/.bin/fbidq.html?Pcl), [chif](http://flybase.bio.indiana.edu/.bin/fbidq.html?chif), [simj](http://flybase.bio.indiana.edu/.bin/fbidq.html?simj), [Awh](http://flybase.bio.indiana.edu/.bin/fbidq.html?Awh), [tap](http://flybase.bio.indiana.edu/.bin/fbidq.html?tap), [NC2alpha](http://flybase.bio.indiana.edu/.bin/fbidq.html?NC2alpha), [PHDP](http://flybase.bio.indiana.edu/.bin/fbidq.html?PHDP), [bab1](http://flybase.bio.indiana.edu/.bin/fbidq.html?bab1), [CG4328](http://flybase.bio.indiana.edu/.bin/fbidq.html?CG4328), [Snoo](http://flybase.bio.indiana.edu/.bin/fbidq.html?Snoo), [l(2)NC136](http://flybase.bio.indiana.edu/.bin/fbidq.html?l(2)NC136), [Taf4](http://flybase.bio.indiana.edu/.bin/fbidq.html?Taf4),[Su(var)3-3](http://flybase.bio.indiana.edu/.bin/fbidq.html?Su(var)3-3), [HGTX](http://flybase.bio.indiana.edu/.bin/fbidq.html?HGTX), [not](http://flybase.bio.indiana.edu/.bin/fbidq.html?not), [nvy](http://flybase.bio.indiana.edu/.bin/fbidq.html?nvy), [nub](http://flybase.bio.indiana.edu/.bin/fbidq.html?nub), [nab](http://flybase.bio.indiana.edu/.bin/fbidq.html?nab), [btd](http://flybase.bio.indiana.edu/.bin/fbidq.html?btd), [fd68A](http://flybase.bio.indiana.edu/.bin/fbidq.html?fd68A), [U2af38](http://flybase.bio.indiana.edu/.bin/fbidq.html?U2af38), [HLHmdelta](http://flybase.bio.indiana.edu/.bin/fbidq.html?HLHmdelta), [B-H1](http://flybase.bio.indiana.edu/.bin/fbidq.html?B-H1), [Nipped-A](http://flybase.bio.indiana.edu/.bin/fbidq.html?Nipped-A), [ATbp](http://flybase.bio.indiana.edu/.bin/fbidq.html?ATbp), [Sos](http://flybase.bio.indiana.edu/.bin/fbidq.html?Sos), [Spf45](http://flybase.bio.indiana.edu/.bin/fbidq.html?Spf45), [Fer2](http://flybase.bio.indiana.edu/.bin/fbidq.html?Fer2), [fd102C](http://flybase.bio.indiana.edu/.bin/fbidq.html?fd102C), [vg](http://flybase.bio.indiana.edu/.bin/fbidq.html?vg), [hth](http://flybase.bio.indiana.edu/.bin/fbidq.html?hth), [Nf-YC](http://flybase.bio.indiana.edu/.bin/fbidq.html?Nf-YC), [eya](http://flybase.bio.indiana.edu/.bin/fbidq.html?eya), [fz](http://flybase.bio.indiana.edu/.bin/fbidq.html?fz), [brm](http://flybase.bio.indiana.edu/.bin/fbidq.html?brm), [Vsx2](http://flybase.bio.indiana.edu/.bin/fbidq.html?Vsx2), [Hel25E](http://flybase.bio.indiana.edu/.bin/fbidq.html?Hel25E), [Hey](http://flybase.bio.indiana.edu/.bin/fbidq.html?Hey), [sno](http://flybase.bio.indiana.edu/.bin/fbidq.html?sno), [Sfmbt](http://flybase.bio.indiana.edu/.bin/fbidq.html?Sfmbt), [ara](http://flybase.bio.indiana.edu/.bin/fbidq.html?ara), [mam](http://flybase.bio.indiana.edu/.bin/fbidq.html?mam), [loqs](http://flybase.bio.indiana.edu/.bin/fbidq.html?loqs), [pan](http://flybase.bio.indiana.edu/.bin/fbidq.html?pan), [Dll](http://flybase.bio.indiana.edu/.bin/fbidq.html?Dll), [disco](http://flybase.bio.indiana.edu/.bin/fbidq.html?disco), [grn](http://flybase.bio.indiana.edu/.bin/fbidq.html?grn), [HLHm5](http://flybase.bio.indiana.edu/.bin/fbidq.html?HLHm5), [D12](http://flybase.bio.indiana.edu/.bin/fbidq.html?D12), [Lis-1](http://flybase.bio.indiana.edu/.bin/fbidq.html?Lis-1),[CycE](http://flybase.bio.indiana.edu/.bin/fbidq.html?CycE), [Brf](http://flybase.bio.indiana.edu/.bin/fbidq.html?Brf), [ems](http://flybase.bio.indiana.edu/.bin/fbidq.html?ems), [CG16896](http://flybase.bio.indiana.edu/.bin/fbidq.html?CG16896), [pros](http://flybase.bio.indiana.edu/.bin/fbidq.html?pros), [tup](http://flybase.bio.indiana.edu/.bin/fbidq.html?tup), [Psi](http://flybase.bio.indiana.edu/.bin/fbidq.html?Psi), [pdm2](http://flybase.bio.indiana.edu/.bin/fbidq.html?pdm2), [Gug](http://flybase.bio.indiana.edu/.bin/fbidq.html?Gug), [pad](http://flybase.bio.indiana.edu/.bin/fbidq.html?pad), [rgr](http://flybase.bio.indiana.edu/.bin/fbidq.html?rgr), [spel1](http://flybase.bio.indiana.edu/.bin/fbidq.html?spel1), [Wnt4](http://flybase.bio.indiana.edu/.bin/fbidq.html?Wnt4), [Rx](http://flybase.bio.indiana.edu/.bin/fbidq.html?Rx), [MED1](http://flybase.bio.indiana.edu/.bin/fbidq.html?MED1), [salr](http://flybase.bio.indiana.edu/.bin/fbidq.html?salr), [wdn](http://flybase.bio.indiana.edu/.bin/fbidq.html?wdn), [sim](http://flybase.bio.indiana.edu/.bin/fbidq.html?sim), [d4](http://flybase.bio.indiana.edu/.bin/fbidq.html?d4), [dan](http://flybase.bio.indiana.edu/.bin/fbidq.html?dan), [CG30372](http://flybase.bio.indiana.edu/.bin/fbidq.html?CG30372), [gro](http://flybase.bio.indiana.edu/.bin/fbidq.html?gro), [CG42629](http://flybase.bio.indiana.edu/.bin/fbidq.html?CG42629), [RN-tre](http://flybase.bio.indiana.edu/.bin/fbidq.html?RN-tre), [nerfin-1](http://flybase.bio.indiana.edu/.bin/fbidq.html?nerfin-1), [trx](http://flybase.bio.indiana.edu/.bin/fbidq.html?trx), [Rpd3](http://flybase.bio.indiana.edu/.bin/fbidq.html?Rpd3), [dsx](http://flybase.bio.indiana.edu/.bin/fbidq.html?dsx), [Ssdp](http://flybase.bio.indiana.edu/.bin/fbidq.html?Ssdp), [vfl](http://flybase.bio.indiana.edu/.bin/fbidq.html?vfl), [sqz](http://flybase.bio.indiana.edu/.bin/fbidq.html?sqz), [CG8155](http://flybase.bio.indiana.edu/.bin/fbidq.html?CG8155), [CG32105](http://flybase.bio.indiana.edu/.bin/fbidq.html?CG32105), [Fer3](http://flybase.bio.indiana.edu/.bin/fbidq.html?Fer3), [aret](http://flybase.bio.indiana.edu/.bin/fbidq.html?aret), [pygo](http://flybase.bio.indiana.edu/.bin/fbidq.html?pygo), [dalao](http://flybase.bio.indiana.edu/.bin/fbidq.html?dalao), [HLHm7](http://flybase.bio.indiana.edu/.bin/fbidq.html?HLHm7), [z](http://flybase.bio.indiana.edu/.bin/fbidq.html?z),[HLHmgamma](http://flybase.bio.indiana.edu/.bin/fbidq.html?HLHmgamma), [Sox21b](http://flybase.bio.indiana.edu/.bin/fbidq.html?Sox21b), [sr](http://flybase.bio.indiana.edu/.bin/fbidq.html?sr), [Lim1](http://flybase.bio.indiana.edu/.bin/fbidq.html?Lim1), [tld](http://flybase.bio.indiana.edu/.bin/fbidq.html?tld), [danr](http://flybase.bio.indiana.edu/.bin/fbidq.html?danr), [wor](http://flybase.bio.indiana.edu/.bin/fbidq.html?wor), [vvl](http://flybase.bio.indiana.edu/.bin/fbidq.html?vvl), [CG7879](http://flybase.bio.indiana.edu/.bin/fbidq.html?CG7879), [wda](http://flybase.bio.indiana.edu/.bin/fbidq.html?wda), [sgg](http://flybase.bio.indiana.edu/.bin/fbidq.html?sgg), [MED14](http://flybase.bio.indiana.edu/.bin/fbidq.html?MED14), [gcm2](http://flybase.bio.indiana.edu/.bin/fbidq.html?gcm2), [pUf68](http://flybase.bio.indiana.edu/.bin/fbidq.html?pUf68), [Eip93F](http://flybase.bio.indiana.edu/.bin/fbidq.html?Eip93F), [seq](http://flybase.bio.indiana.edu/.bin/fbidq.html?seq), [gcm](http://flybase.bio.indiana.edu/.bin/fbidq.html?gcm), [Dip3](http://flybase.bio.indiana.edu/.bin/fbidq.html?Dip3), [Aef1](http://flybase.bio.indiana.edu/.bin/fbidq.html?Aef1), [HDAC4](http://flybase.bio.indiana.edu/.bin/fbidq.html?HDAC4), [can](http://flybase.bio.indiana.edu/.bin/fbidq.html?can), [elk](http://flybase.bio.indiana.edu/.bin/fbidq.html?elk), [phl](http://flybase.bio.indiana.edu/.bin/fbidq.html?phl), [ase](http://flybase.bio.indiana.edu/.bin/fbidq.html?ase), [CG13253](http://flybase.bio.indiana.edu/.bin/fbidq.html?CG13253), [ovo](http://flybase.bio.indiana.edu/.bin/fbidq.html?ovo), [scro](http://flybase.bio.indiana.edu/.bin/fbidq.html?scro), [CG6197](http://flybase.bio.indiana.edu/.bin/fbidq.html?CG6197), [elav](http://flybase.bio.indiana.edu/.bin/fbidq.html?elav), [Dsp1](http://flybase.bio.indiana.edu/.bin/fbidq.html?Dsp1), [CG14216](http://flybase.bio.indiana.edu/.bin/fbidq.html?CG14216), [Pdp1](http://flybase.bio.indiana.edu/.bin/fbidq.html?Pdp1), [plx](http://flybase.bio.indiana.edu/.bin/fbidq.html?plx), [Oli](http://flybase.bio.indiana.edu/.bin/fbidq.html?Oli), [Nelf-A](http://flybase.bio.indiana.edu/.bin/fbidq.html?Nelf-A), [GATAd](http://flybase.bio.indiana.edu/.bin/fbidq.html?GATAd),[CG6227](http://flybase.bio.indiana.edu/.bin/fbidq.html?CG6227), [Poxn](http://flybase.bio.indiana.edu/.bin/fbidq.html?Poxn), [CG15376](http://flybase.bio.indiana.edu/.bin/fbidq.html?CG15376), [Sin3A](http://flybase.bio.indiana.edu/.bin/fbidq.html?Sin3A), [mip120](http://flybase.bio.indiana.edu/.bin/fbidq.html?mip120), [jumu](http://flybase.bio.indiana.edu/.bin/fbidq.html?jumu), [Smox](http://flybase.bio.indiana.edu/.bin/fbidq.html?Smox), [Usp7](http://flybase.bio.indiana.edu/.bin/fbidq.html?Usp7), [ush](http://flybase.bio.indiana.edu/.bin/fbidq.html?ush), [fd59A](http://flybase.bio.indiana.edu/.bin/fbidq.html?fd59A), [CG10336](http://flybase.bio.indiana.edu/.bin/fbidq.html?CG10336), [Gap1](http://flybase.bio.indiana.edu/.bin/fbidq.html?Gap1), [run](http://flybase.bio.indiana.edu/.bin/fbidq.html?run), [FBX011](http://flybase.bio.indiana.edu/.bin/fbidq.html?FBX011), [Antp](http://flybase.bio.indiana.edu/.bin/fbidq.html?Antp), [tara](http://flybase.bio.indiana.edu/.bin/fbidq.html?tara), [Alh](http://flybase.bio.indiana.edu/.bin/fbidq.html?Alh), [Sox102F](http://flybase.bio.indiana.edu/.bin/fbidq.html?Sox102F), [egg](http://flybase.bio.indiana.edu/.bin/fbidq.html?egg), [CG5337](http://flybase.bio.indiana.edu/.bin/fbidq.html?CG5337), [l(3)neo38](http://flybase.bio.indiana.edu/.bin/fbidq.html?l(3)neo38), [koko](http://flybase.bio.indiana.edu/.bin/fbidq.html?koko), [Ptth](http://flybase.bio.indiana.edu/.bin/fbidq.html?Ptth), [Sp1](http://flybase.bio.indiana.edu/.bin/fbidq.html?Sp1), [Ubx](http://flybase.bio.indiana.edu/.bin/fbidq.html?Ubx), [Ptx1](http://flybase.bio.indiana.edu/.bin/fbidq.html?Ptx1), [ph-p](http://flybase.bio.indiana.edu/.bin/fbidq.html?ph-p), [onecut](http://flybase.bio.indiana.edu/.bin/fbidq.html?onecut), [CG7757](http://flybase.bio.indiana.edu/.bin/fbidq.html?CG7757), [chm](http://flybase.bio.indiana.edu/.bin/fbidq.html?chm), [sna](http://flybase.bio.indiana.edu/.bin/fbidq.html?sna), [Wnt5](http://flybase.bio.indiana.edu/.bin/fbidq.html?Wnt5), [Taf1](http://flybase.bio.indiana.edu/.bin/fbidq.html?Taf1), [hbn](http://flybase.bio.indiana.edu/.bin/fbidq.html?hbn), [MBD-like](http://flybase.bio.indiana.edu/.bin/fbidq.html?MBD-like), [tsh](http://flybase.bio.indiana.edu/.bin/fbidq.html?tsh), [esg](http://flybase.bio.indiana.edu/.bin/fbidq.html?esg), [Rgl](http://flybase.bio.indiana.edu/.bin/fbidq.html?Rgl), [e(y)3](http://flybase.bio.indiana.edu/.bin/fbidq.html?e(y)3), [sif](http://flybase.bio.indiana.edu/.bin/fbidq.html?sif), [CG9007](http://flybase.bio.indiana.edu/.bin/fbidq.html?CG9007), [oc](http://flybase.bio.indiana.edu/.bin/fbidq.html?oc), [dom](http://flybase.bio.indiana.edu/.bin/fbidq.html?dom), [spt4](http://flybase.bio.indiana.edu/.bin/fbidq.html?spt4), [CG32532](http://flybase.bio.indiana.edu/.bin/fbidq.html?CG32532), [MTA1-like](http://flybase.bio.indiana.edu/.bin/fbidq.html?MTA1-like), [gol](http://flybase.bio.indiana.edu/.bin/fbidq.html?gol), [ey](http://flybase.bio.indiana.edu/.bin/fbidq.html?ey), [mle](http://flybase.bio.indiana.edu/.bin/fbidq.html?mle), [Hr51](http://flybase.bio.indiana.edu/.bin/fbidq.html?Hr51), [Bgb](http://flybase.bio.indiana.edu/.bin/fbidq.html?Bgb), [l(1)sc](http://flybase.bio.indiana.edu/.bin/fbidq.html?l(1)sc), [B-H2](http://flybase.bio.indiana.edu/.bin/fbidq.html?B-H2), [grh](http://flybase.bio.indiana.edu/.bin/fbidq.html?grh), [Vsx1](http://flybase.bio.indiana.edu/.bin/fbidq.html?Vsx1), [wg](http://flybase.bio.indiana.edu/.bin/fbidq.html?wg), [toy](http://flybase.bio.indiana.edu/.bin/fbidq.html?toy), [fkh](http://flybase.bio.indiana.edu/.bin/fbidq.html?fkh), [Lim3](http://flybase.bio.indiana.edu/.bin/fbidq.html?Lim3), [CG34362](http://flybase.bio.indiana.edu/.bin/fbidq.html?CG34362), [crol](http://flybase.bio.indiana.edu/.bin/fbidq.html?crol), [sd](http://flybase.bio.indiana.edu/.bin/fbidq.html?sd), [fne](http://flybase.bio.indiana.edu/.bin/fbidq.html?fne), [retn](http://flybase.bio.indiana.edu/.bin/fbidq.html?retn), [en](http://flybase.bio.indiana.edu/.bin/fbidq.html?en), [fs(1)h](http://flybase.bio.indiana.edu/.bin/fbidq.html?fs(1)h), [rl](http://flybase.bio.indiana.edu/.bin/fbidq.html?rl), [CG11347](http://flybase.bio.indiana.edu/.bin/fbidq.html?CG11347), [tey](http://flybase.bio.indiana.edu/.bin/fbidq.html?tey), [Saf-B](http://flybase.bio.indiana.edu/.bin/fbidq.html?Saf-B), [CG11294](http://flybase.bio.indiana.edu/.bin/fbidq.html?CG11294), [hiw](http://flybase.bio.indiana.edu/.bin/fbidq.html?hiw), [cos](http://flybase.bio.indiana.edu/.bin/fbidq.html?cos), [Ct](http://flybase.bio.indiana.edu/.bin/fbidq.html?Ct), [Atf-2](http://flybase.bio.indiana.edu/.bin/fbidq.html?Atf-2) |
| [regulation of nitrogen compound metabolic process](http://amigo.geneontology.org/cgi-bin/amigo/go.cgi?view=details&query=GO:0051171) | 235 of 1594 genes, 14.7% | 756 of 7634 genes, 9.9% | 1.30e-11 | 0.00% | 0.00 | [fz2](http://flybase.bio.indiana.edu/.bin/fbidq.html?fz2), [acj6](http://flybase.bio.indiana.edu/.bin/fbidq.html?acj6), [skd](http://flybase.bio.indiana.edu/.bin/fbidq.html?skd), [jing](http://flybase.bio.indiana.edu/.bin/fbidq.html?jing), [caup](http://flybase.bio.indiana.edu/.bin/fbidq.html?caup), [tll](http://flybase.bio.indiana.edu/.bin/fbidq.html?tll), [Camta](http://flybase.bio.indiana.edu/.bin/fbidq.html?Camta), [ato](http://flybase.bio.indiana.edu/.bin/fbidq.html?ato), [Ets65A](http://flybase.bio.indiana.edu/.bin/fbidq.html?Ets65A), [Rox8](http://flybase.bio.indiana.edu/.bin/fbidq.html?Rox8), [tna](http://flybase.bio.indiana.edu/.bin/fbidq.html?tna), [Sxl](http://flybase.bio.indiana.edu/.bin/fbidq.html?Sxl), [mub](http://flybase.bio.indiana.edu/.bin/fbidq.html?mub), [JIL-1](http://flybase.bio.indiana.edu/.bin/fbidq.html?JIL-1), [Spt6](http://flybase.bio.indiana.edu/.bin/fbidq.html?Spt6), [Iswi](http://flybase.bio.indiana.edu/.bin/fbidq.html?Iswi), [rn](http://flybase.bio.indiana.edu/.bin/fbidq.html?rn), [ap](http://flybase.bio.indiana.edu/.bin/fbidq.html?ap), [cenG1A](http://flybase.bio.indiana.edu/.bin/fbidq.html?cenG1A), [Nf1](http://flybase.bio.indiana.edu/.bin/fbidq.html?Nf1), [Evi5](http://flybase.bio.indiana.edu/.bin/fbidq.html?Evi5), [br](http://flybase.bio.indiana.edu/.bin/fbidq.html?br), [ksr](http://flybase.bio.indiana.edu/.bin/fbidq.html?ksr), [bi](http://flybase.bio.indiana.edu/.bin/fbidq.html?bi), [chn](http://flybase.bio.indiana.edu/.bin/fbidq.html?chn), [Gsc](http://flybase.bio.indiana.edu/.bin/fbidq.html?Gsc), [Hcf](http://flybase.bio.indiana.edu/.bin/fbidq.html?Hcf), [lola](http://flybase.bio.indiana.edu/.bin/fbidq.html?lola), [HLH4C](http://flybase.bio.indiana.edu/.bin/fbidq.html?HLH4C), [Pcl](http://flybase.bio.indiana.edu/.bin/fbidq.html?Pcl), [chif](http://flybase.bio.indiana.edu/.bin/fbidq.html?chif), [simj](http://flybase.bio.indiana.edu/.bin/fbidq.html?simj), [Awh](http://flybase.bio.indiana.edu/.bin/fbidq.html?Awh), [tap](http://flybase.bio.indiana.edu/.bin/fbidq.html?tap), [NC2alpha](http://flybase.bio.indiana.edu/.bin/fbidq.html?NC2alpha), [PHDP](http://flybase.bio.indiana.edu/.bin/fbidq.html?PHDP), [bab1](http://flybase.bio.indiana.edu/.bin/fbidq.html?bab1), [CG4328](http://flybase.bio.indiana.edu/.bin/fbidq.html?CG4328), [Snoo](http://flybase.bio.indiana.edu/.bin/fbidq.html?Snoo), [l(2)NC136](http://flybase.bio.indiana.edu/.bin/fbidq.html?l(2)NC136), [Taf4](http://flybase.bio.indiana.edu/.bin/fbidq.html?Taf4),[Su(var)3-3](http://flybase.bio.indiana.edu/.bin/fbidq.html?Su(var)3-3), [scrib](http://flybase.bio.indiana.edu/.bin/fbidq.html?scrib), [HGTX](http://flybase.bio.indiana.edu/.bin/fbidq.html?HGTX), [not](http://flybase.bio.indiana.edu/.bin/fbidq.html?not), [nvy](http://flybase.bio.indiana.edu/.bin/fbidq.html?nvy), [nub](http://flybase.bio.indiana.edu/.bin/fbidq.html?nub), [nab](http://flybase.bio.indiana.edu/.bin/fbidq.html?nab), [btd](http://flybase.bio.indiana.edu/.bin/fbidq.html?btd), [fd68A](http://flybase.bio.indiana.edu/.bin/fbidq.html?fd68A), [U2af38](http://flybase.bio.indiana.edu/.bin/fbidq.html?U2af38), [HLHmdelta](http://flybase.bio.indiana.edu/.bin/fbidq.html?HLHmdelta), [B-H1](http://flybase.bio.indiana.edu/.bin/fbidq.html?B-H1), [Nipped-A](http://flybase.bio.indiana.edu/.bin/fbidq.html?Nipped-A), [ATbp](http://flybase.bio.indiana.edu/.bin/fbidq.html?ATbp), [Sos](http://flybase.bio.indiana.edu/.bin/fbidq.html?Sos), [Spf45](http://flybase.bio.indiana.edu/.bin/fbidq.html?Spf45), [Fer2](http://flybase.bio.indiana.edu/.bin/fbidq.html?Fer2), [fd102C](http://flybase.bio.indiana.edu/.bin/fbidq.html?fd102C), [vg](http://flybase.bio.indiana.edu/.bin/fbidq.html?vg), [hth](http://flybase.bio.indiana.edu/.bin/fbidq.html?hth), [Nf-YC](http://flybase.bio.indiana.edu/.bin/fbidq.html?Nf-YC), [eya](http://flybase.bio.indiana.edu/.bin/fbidq.html?eya), [fz](http://flybase.bio.indiana.edu/.bin/fbidq.html?fz), [brm](http://flybase.bio.indiana.edu/.bin/fbidq.html?brm), [Vsx2](http://flybase.bio.indiana.edu/.bin/fbidq.html?Vsx2), [Hel25E](http://flybase.bio.indiana.edu/.bin/fbidq.html?Hel25E), [Hey](http://flybase.bio.indiana.edu/.bin/fbidq.html?Hey), [sno](http://flybase.bio.indiana.edu/.bin/fbidq.html?sno), [Sfmbt](http://flybase.bio.indiana.edu/.bin/fbidq.html?Sfmbt), [ara](http://flybase.bio.indiana.edu/.bin/fbidq.html?ara), [mam](http://flybase.bio.indiana.edu/.bin/fbidq.html?mam), [loqs](http://flybase.bio.indiana.edu/.bin/fbidq.html?loqs), [pan](http://flybase.bio.indiana.edu/.bin/fbidq.html?pan), [Dll](http://flybase.bio.indiana.edu/.bin/fbidq.html?Dll), [disco](http://flybase.bio.indiana.edu/.bin/fbidq.html?disco), [grn](http://flybase.bio.indiana.edu/.bin/fbidq.html?grn), [HLHm5](http://flybase.bio.indiana.edu/.bin/fbidq.html?HLHm5), [D12](http://flybase.bio.indiana.edu/.bin/fbidq.html?D12),[Lis-1](http://flybase.bio.indiana.edu/.bin/fbidq.html?Lis-1), [CycE](http://flybase.bio.indiana.edu/.bin/fbidq.html?CycE), [Brf](http://flybase.bio.indiana.edu/.bin/fbidq.html?Brf), [ems](http://flybase.bio.indiana.edu/.bin/fbidq.html?ems), [CG16896](http://flybase.bio.indiana.edu/.bin/fbidq.html?CG16896), [pros](http://flybase.bio.indiana.edu/.bin/fbidq.html?pros), [tup](http://flybase.bio.indiana.edu/.bin/fbidq.html?tup), [Psi](http://flybase.bio.indiana.edu/.bin/fbidq.html?Psi), [pdm2](http://flybase.bio.indiana.edu/.bin/fbidq.html?pdm2), [Gug](http://flybase.bio.indiana.edu/.bin/fbidq.html?Gug), [pad](http://flybase.bio.indiana.edu/.bin/fbidq.html?pad), [rgr](http://flybase.bio.indiana.edu/.bin/fbidq.html?rgr), [spel1](http://flybase.bio.indiana.edu/.bin/fbidq.html?spel1), [Wnt4](http://flybase.bio.indiana.edu/.bin/fbidq.html?Wnt4), [Rx](http://flybase.bio.indiana.edu/.bin/fbidq.html?Rx), [MED1](http://flybase.bio.indiana.edu/.bin/fbidq.html?MED1), [salr](http://flybase.bio.indiana.edu/.bin/fbidq.html?salr), [wdn](http://flybase.bio.indiana.edu/.bin/fbidq.html?wdn), [sim](http://flybase.bio.indiana.edu/.bin/fbidq.html?sim), [d4](http://flybase.bio.indiana.edu/.bin/fbidq.html?d4), [dan](http://flybase.bio.indiana.edu/.bin/fbidq.html?dan), [CG30372](http://flybase.bio.indiana.edu/.bin/fbidq.html?CG30372), [gro](http://flybase.bio.indiana.edu/.bin/fbidq.html?gro), [CG42629](http://flybase.bio.indiana.edu/.bin/fbidq.html?CG42629), [RN-tre](http://flybase.bio.indiana.edu/.bin/fbidq.html?RN-tre), [nerfin-1](http://flybase.bio.indiana.edu/.bin/fbidq.html?nerfin-1), [trx](http://flybase.bio.indiana.edu/.bin/fbidq.html?trx), [Rpd3](http://flybase.bio.indiana.edu/.bin/fbidq.html?Rpd3), [dsx](http://flybase.bio.indiana.edu/.bin/fbidq.html?dsx), [MED25](http://flybase.bio.indiana.edu/.bin/fbidq.html?MED25), [Ssdp](http://flybase.bio.indiana.edu/.bin/fbidq.html?Ssdp), [vfl](http://flybase.bio.indiana.edu/.bin/fbidq.html?vfl), [sqz](http://flybase.bio.indiana.edu/.bin/fbidq.html?sqz), [CG8155](http://flybase.bio.indiana.edu/.bin/fbidq.html?CG8155), [CG32105](http://flybase.bio.indiana.edu/.bin/fbidq.html?CG32105), [Fer3](http://flybase.bio.indiana.edu/.bin/fbidq.html?Fer3), [aret](http://flybase.bio.indiana.edu/.bin/fbidq.html?aret), [pygo](http://flybase.bio.indiana.edu/.bin/fbidq.html?pygo),[dalao](http://flybase.bio.indiana.edu/.bin/fbidq.html?dalao), [HLHm7](http://flybase.bio.indiana.edu/.bin/fbidq.html?HLHm7), [z](http://flybase.bio.indiana.edu/.bin/fbidq.html?z), [HLHmgamma](http://flybase.bio.indiana.edu/.bin/fbidq.html?HLHmgamma), [Sox21b](http://flybase.bio.indiana.edu/.bin/fbidq.html?Sox21b), [sr](http://flybase.bio.indiana.edu/.bin/fbidq.html?sr), [Lim1](http://flybase.bio.indiana.edu/.bin/fbidq.html?Lim1), [tld](http://flybase.bio.indiana.edu/.bin/fbidq.html?tld), [danr](http://flybase.bio.indiana.edu/.bin/fbidq.html?danr), [wor](http://flybase.bio.indiana.edu/.bin/fbidq.html?wor), [vvl](http://flybase.bio.indiana.edu/.bin/fbidq.html?vvl), [CG7879](http://flybase.bio.indiana.edu/.bin/fbidq.html?CG7879), [wda](http://flybase.bio.indiana.edu/.bin/fbidq.html?wda), [sgg](http://flybase.bio.indiana.edu/.bin/fbidq.html?sgg), [MED14](http://flybase.bio.indiana.edu/.bin/fbidq.html?MED14), [gcm2](http://flybase.bio.indiana.edu/.bin/fbidq.html?gcm2), [pUf68](http://flybase.bio.indiana.edu/.bin/fbidq.html?pUf68), [Eip93F](http://flybase.bio.indiana.edu/.bin/fbidq.html?Eip93F), [seq](http://flybase.bio.indiana.edu/.bin/fbidq.html?seq), [gcm](http://flybase.bio.indiana.edu/.bin/fbidq.html?gcm), [Dip3](http://flybase.bio.indiana.edu/.bin/fbidq.html?Dip3), [Aef1](http://flybase.bio.indiana.edu/.bin/fbidq.html?Aef1), [HDAC4](http://flybase.bio.indiana.edu/.bin/fbidq.html?HDAC4), [can](http://flybase.bio.indiana.edu/.bin/fbidq.html?can), [elk](http://flybase.bio.indiana.edu/.bin/fbidq.html?elk), [phl](http://flybase.bio.indiana.edu/.bin/fbidq.html?phl), [ase](http://flybase.bio.indiana.edu/.bin/fbidq.html?ase), [CG13253](http://flybase.bio.indiana.edu/.bin/fbidq.html?CG13253), [ovo](http://flybase.bio.indiana.edu/.bin/fbidq.html?ovo), [scro](http://flybase.bio.indiana.edu/.bin/fbidq.html?scro), [CG6197](http://flybase.bio.indiana.edu/.bin/fbidq.html?CG6197), [elav](http://flybase.bio.indiana.edu/.bin/fbidq.html?elav), [pog](http://flybase.bio.indiana.edu/.bin/fbidq.html?pog), [Dsp1](http://flybase.bio.indiana.edu/.bin/fbidq.html?Dsp1), [CG14216](http://flybase.bio.indiana.edu/.bin/fbidq.html?CG14216), [Pdp1](http://flybase.bio.indiana.edu/.bin/fbidq.html?Pdp1), [plx](http://flybase.bio.indiana.edu/.bin/fbidq.html?plx),[Oli](http://flybase.bio.indiana.edu/.bin/fbidq.html?Oli), [Nelf-A](http://flybase.bio.indiana.edu/.bin/fbidq.html?Nelf-A), [GATAd](http://flybase.bio.indiana.edu/.bin/fbidq.html?GATAd), [CG6227](http://flybase.bio.indiana.edu/.bin/fbidq.html?CG6227), [Poxn](http://flybase.bio.indiana.edu/.bin/fbidq.html?Poxn), [CG15376](http://flybase.bio.indiana.edu/.bin/fbidq.html?CG15376), [Sin3A](http://flybase.bio.indiana.edu/.bin/fbidq.html?Sin3A), [mip120](http://flybase.bio.indiana.edu/.bin/fbidq.html?mip120), [casp](http://flybase.bio.indiana.edu/.bin/fbidq.html?casp), [jumu](http://flybase.bio.indiana.edu/.bin/fbidq.html?jumu), [Smox](http://flybase.bio.indiana.edu/.bin/fbidq.html?Smox), [Usp7](http://flybase.bio.indiana.edu/.bin/fbidq.html?Usp7), [ush](http://flybase.bio.indiana.edu/.bin/fbidq.html?ush), [fd59A](http://flybase.bio.indiana.edu/.bin/fbidq.html?fd59A), [CG10336](http://flybase.bio.indiana.edu/.bin/fbidq.html?CG10336), [Gap1](http://flybase.bio.indiana.edu/.bin/fbidq.html?Gap1), [run](http://flybase.bio.indiana.edu/.bin/fbidq.html?run), [FBX011](http://flybase.bio.indiana.edu/.bin/fbidq.html?FBX011), [Antp](http://flybase.bio.indiana.edu/.bin/fbidq.html?Antp), [tara](http://flybase.bio.indiana.edu/.bin/fbidq.html?tara), [Alh](http://flybase.bio.indiana.edu/.bin/fbidq.html?Alh), [Hel89B](http://flybase.bio.indiana.edu/.bin/fbidq.html?Hel89B), [Sox102F](http://flybase.bio.indiana.edu/.bin/fbidq.html?Sox102F), [egg](http://flybase.bio.indiana.edu/.bin/fbidq.html?egg), [CG5337](http://flybase.bio.indiana.edu/.bin/fbidq.html?CG5337), [l(3)neo38](http://flybase.bio.indiana.edu/.bin/fbidq.html?l(3)neo38), [koko](http://flybase.bio.indiana.edu/.bin/fbidq.html?koko), [Ptth](http://flybase.bio.indiana.edu/.bin/fbidq.html?Ptth), [Sp1](http://flybase.bio.indiana.edu/.bin/fbidq.html?Sp1), [Ubx](http://flybase.bio.indiana.edu/.bin/fbidq.html?Ubx), [Ptx1](http://flybase.bio.indiana.edu/.bin/fbidq.html?Ptx1), [ph-p](http://flybase.bio.indiana.edu/.bin/fbidq.html?ph-p), [onecut](http://flybase.bio.indiana.edu/.bin/fbidq.html?onecut),[CG7757](http://flybase.bio.indiana.edu/.bin/fbidq.html?CG7757), [chm](http://flybase.bio.indiana.edu/.bin/fbidq.html?chm), [sna](http://flybase.bio.indiana.edu/.bin/fbidq.html?sna), [Wnt5](http://flybase.bio.indiana.edu/.bin/fbidq.html?Wnt5), [Taf1](http://flybase.bio.indiana.edu/.bin/fbidq.html?Taf1), [hbn](http://flybase.bio.indiana.edu/.bin/fbidq.html?hbn), [MBD-like](http://flybase.bio.indiana.edu/.bin/fbidq.html?MBD-like), [tsh](http://flybase.bio.indiana.edu/.bin/fbidq.html?tsh), [esg](http://flybase.bio.indiana.edu/.bin/fbidq.html?esg), [Rgl](http://flybase.bio.indiana.edu/.bin/fbidq.html?Rgl), [e(y)3](http://flybase.bio.indiana.edu/.bin/fbidq.html?e(y)3), [sif](http://flybase.bio.indiana.edu/.bin/fbidq.html?sif), [CG9007](http://flybase.bio.indiana.edu/.bin/fbidq.html?CG9007), [oc](http://flybase.bio.indiana.edu/.bin/fbidq.html?oc), [dom](http://flybase.bio.indiana.edu/.bin/fbidq.html?dom), [spt4](http://flybase.bio.indiana.edu/.bin/fbidq.html?spt4), [CG32532](http://flybase.bio.indiana.edu/.bin/fbidq.html?CG32532), [MTA1-like](http://flybase.bio.indiana.edu/.bin/fbidq.html?MTA1-like), [gol](http://flybase.bio.indiana.edu/.bin/fbidq.html?gol), [ey](http://flybase.bio.indiana.edu/.bin/fbidq.html?ey), [mle](http://flybase.bio.indiana.edu/.bin/fbidq.html?mle), [Hr51](http://flybase.bio.indiana.edu/.bin/fbidq.html?Hr51), [Bgb](http://flybase.bio.indiana.edu/.bin/fbidq.html?Bgb), [l(1)sc](http://flybase.bio.indiana.edu/.bin/fbidq.html?l(1)sc), [B-H2](http://flybase.bio.indiana.edu/.bin/fbidq.html?B-H2), [grh](http://flybase.bio.indiana.edu/.bin/fbidq.html?grh), [Vsx1](http://flybase.bio.indiana.edu/.bin/fbidq.html?Vsx1), [wg](http://flybase.bio.indiana.edu/.bin/fbidq.html?wg), [toy](http://flybase.bio.indiana.edu/.bin/fbidq.html?toy), [fkh](http://flybase.bio.indiana.edu/.bin/fbidq.html?fkh), [Lim3](http://flybase.bio.indiana.edu/.bin/fbidq.html?Lim3), [CG34362](http://flybase.bio.indiana.edu/.bin/fbidq.html?CG34362), [crol](http://flybase.bio.indiana.edu/.bin/fbidq.html?crol), [sd](http://flybase.bio.indiana.edu/.bin/fbidq.html?sd), [fne](http://flybase.bio.indiana.edu/.bin/fbidq.html?fne), [retn](http://flybase.bio.indiana.edu/.bin/fbidq.html?retn), [en](http://flybase.bio.indiana.edu/.bin/fbidq.html?en), [fs(1)h](http://flybase.bio.indiana.edu/.bin/fbidq.html?fs(1)h), [rl](http://flybase.bio.indiana.edu/.bin/fbidq.html?rl), [CG11347](http://flybase.bio.indiana.edu/.bin/fbidq.html?CG11347),[tey](http://flybase.bio.indiana.edu/.bin/fbidq.html?tey), [Saf-B](http://flybase.bio.indiana.edu/.bin/fbidq.html?Saf-B), [CG11294](http://flybase.bio.indiana.edu/.bin/fbidq.html?CG11294), [hiw](http://flybase.bio.indiana.edu/.bin/fbidq.html?hiw), [cos](http://flybase.bio.indiana.edu/.bin/fbidq.html?cos), [Ct](http://flybase.bio.indiana.edu/.bin/fbidq.html?Ct), [Atf-2](http://flybase.bio.indiana.edu/.bin/fbidq.html?Atf-2), [dnr1](http://flybase.bio.indiana.edu/.bin/fbidq.html?dnr1) |
| [behavior](http://amigo.geneontology.org/cgi-bin/amigo/go.cgi?view=details&query=GO:0007610) | 133 of 1594 genes, 8.3% | 360 of 7634 genes, 4.7% | 1.85e-11 | 0.00% | 0.00 | [Sh](http://flybase.bio.indiana.edu/.bin/fbidq.html?Sh), [numb](http://flybase.bio.indiana.edu/.bin/fbidq.html?numb), [acj6](http://flybase.bio.indiana.edu/.bin/fbidq.html?acj6), [ato](http://flybase.bio.indiana.edu/.bin/fbidq.html?ato), [Spn](http://flybase.bio.indiana.edu/.bin/fbidq.html?Spn), [CG17734](http://flybase.bio.indiana.edu/.bin/fbidq.html?CG17734), [Nf1](http://flybase.bio.indiana.edu/.bin/fbidq.html?Nf1), [Pka-R1](http://flybase.bio.indiana.edu/.bin/fbidq.html?Pka-R1), [CG14478](http://flybase.bio.indiana.edu/.bin/fbidq.html?CG14478), [sol](http://flybase.bio.indiana.edu/.bin/fbidq.html?sol), [os](http://flybase.bio.indiana.edu/.bin/fbidq.html?os), [brp](http://flybase.bio.indiana.edu/.bin/fbidq.html?brp), [nonA](http://flybase.bio.indiana.edu/.bin/fbidq.html?nonA), [lola](http://flybase.bio.indiana.edu/.bin/fbidq.html?lola), [unc-104](http://flybase.bio.indiana.edu/.bin/fbidq.html?unc-104), [NetB](http://flybase.bio.indiana.edu/.bin/fbidq.html?NetB), [Appl](http://flybase.bio.indiana.edu/.bin/fbidq.html?Appl), [CG4476](http://flybase.bio.indiana.edu/.bin/fbidq.html?CG4476), [Galpha49B](http://flybase.bio.indiana.edu/.bin/fbidq.html?Galpha49B), [Ilp3](http://flybase.bio.indiana.edu/.bin/fbidq.html?Ilp3), [Lar](http://flybase.bio.indiana.edu/.bin/fbidq.html?Lar), [scrib](http://flybase.bio.indiana.edu/.bin/fbidq.html?scrib), [rut](http://flybase.bio.indiana.edu/.bin/fbidq.html?rut), [Nrg](http://flybase.bio.indiana.edu/.bin/fbidq.html?Nrg), [nab](http://flybase.bio.indiana.edu/.bin/fbidq.html?nab), [Ddc](http://flybase.bio.indiana.edu/.bin/fbidq.html?Ddc), [amon](http://flybase.bio.indiana.edu/.bin/fbidq.html?amon), [DAT](http://flybase.bio.indiana.edu/.bin/fbidq.html?DAT), [stau](http://flybase.bio.indiana.edu/.bin/fbidq.html?stau), [Sema-1a](http://flybase.bio.indiana.edu/.bin/fbidq.html?Sema-1a), [Eh](http://flybase.bio.indiana.edu/.bin/fbidq.html?Eh), [TBPH](http://flybase.bio.indiana.edu/.bin/fbidq.html?TBPH), [Fer2](http://flybase.bio.indiana.edu/.bin/fbidq.html?Fer2), [CG34400](http://flybase.bio.indiana.edu/.bin/fbidq.html?CG34400), [lid](http://flybase.bio.indiana.edu/.bin/fbidq.html?lid), [Fas2](http://flybase.bio.indiana.edu/.bin/fbidq.html?Fas2), [14-3-3zeta](http://flybase.bio.indiana.edu/.bin/fbidq.html?14-3-3zeta), [robo](http://flybase.bio.indiana.edu/.bin/fbidq.html?robo), [shakB](http://flybase.bio.indiana.edu/.bin/fbidq.html?shakB), [qvr](http://flybase.bio.indiana.edu/.bin/fbidq.html?qvr), [smi35A](http://flybase.bio.indiana.edu/.bin/fbidq.html?smi35A), [jet](http://flybase.bio.indiana.edu/.bin/fbidq.html?jet), [Dll](http://flybase.bio.indiana.edu/.bin/fbidq.html?Dll), [disco](http://flybase.bio.indiana.edu/.bin/fbidq.html?disco), [DopR](http://flybase.bio.indiana.edu/.bin/fbidq.html?DopR), [neur](http://flybase.bio.indiana.edu/.bin/fbidq.html?neur), [5-HT1A](http://flybase.bio.indiana.edu/.bin/fbidq.html?5-HT1A), [Bx](http://flybase.bio.indiana.edu/.bin/fbidq.html?Bx), [Mob2](http://flybase.bio.indiana.edu/.bin/fbidq.html?Mob2), [pros](http://flybase.bio.indiana.edu/.bin/fbidq.html?pros), [Actn](http://flybase.bio.indiana.edu/.bin/fbidq.html?Actn), [ed](http://flybase.bio.indiana.edu/.bin/fbidq.html?ed), [orb2](http://flybase.bio.indiana.edu/.bin/fbidq.html?orb2), [tipE](http://flybase.bio.indiana.edu/.bin/fbidq.html?tipE), [Gad1](http://flybase.bio.indiana.edu/.bin/fbidq.html?Gad1), [rogdi](http://flybase.bio.indiana.edu/.bin/fbidq.html?rogdi), [sim](http://flybase.bio.indiana.edu/.bin/fbidq.html?sim), [VGAT](http://flybase.bio.indiana.edu/.bin/fbidq.html?VGAT), [CaMKII](http://flybase.bio.indiana.edu/.bin/fbidq.html?CaMKII), [tau](http://flybase.bio.indiana.edu/.bin/fbidq.html?tau), [CG42629](http://flybase.bio.indiana.edu/.bin/fbidq.html?CG42629), [bin3](http://flybase.bio.indiana.edu/.bin/fbidq.html?bin3), [5-HT7](http://flybase.bio.indiana.edu/.bin/fbidq.html?5-HT7), [Btk29A](http://flybase.bio.indiana.edu/.bin/fbidq.html?Btk29A), [dsx](http://flybase.bio.indiana.edu/.bin/fbidq.html?dsx), [PQBP-1](http://flybase.bio.indiana.edu/.bin/fbidq.html?PQBP-1), [tyf](http://flybase.bio.indiana.edu/.bin/fbidq.html?tyf), [klu](http://flybase.bio.indiana.edu/.bin/fbidq.html?klu), [SPR](http://flybase.bio.indiana.edu/.bin/fbidq.html?SPR), [NPFR1](http://flybase.bio.indiana.edu/.bin/fbidq.html?NPFR1), [aret](http://flybase.bio.indiana.edu/.bin/fbidq.html?aret), [HLHm7](http://flybase.bio.indiana.edu/.bin/fbidq.html?HLHm7), [NaCP60E](http://flybase.bio.indiana.edu/.bin/fbidq.html?NaCP60E),[CG14509](http://flybase.bio.indiana.edu/.bin/fbidq.html?CG14509), [Dsk](http://flybase.bio.indiana.edu/.bin/fbidq.html?Dsk), [rg](http://flybase.bio.indiana.edu/.bin/fbidq.html?rg), [betaInt-nu](http://flybase.bio.indiana.edu/.bin/fbidq.html?betaInt-nu), [npf](http://flybase.bio.indiana.edu/.bin/fbidq.html?npf), [sgg](http://flybase.bio.indiana.edu/.bin/fbidq.html?sgg), [mnb](http://flybase.bio.indiana.edu/.bin/fbidq.html?mnb), [Nmdar1](http://flybase.bio.indiana.edu/.bin/fbidq.html?Nmdar1), [dpr9](http://flybase.bio.indiana.edu/.bin/fbidq.html?dpr9), [tay](http://flybase.bio.indiana.edu/.bin/fbidq.html?tay), [l(3)L1231](http://flybase.bio.indiana.edu/.bin/fbidq.html?l(3)L1231), [ovo](http://flybase.bio.indiana.edu/.bin/fbidq.html?ovo), [hang](http://flybase.bio.indiana.edu/.bin/fbidq.html?hang), [sNPF](http://flybase.bio.indiana.edu/.bin/fbidq.html?sNPF), [CG10251](http://flybase.bio.indiana.edu/.bin/fbidq.html?CG10251), [CASK](http://flybase.bio.indiana.edu/.bin/fbidq.html?CASK), [mtt](http://flybase.bio.indiana.edu/.bin/fbidq.html?mtt), [Poxn](http://flybase.bio.indiana.edu/.bin/fbidq.html?Poxn), [Alk](http://flybase.bio.indiana.edu/.bin/fbidq.html?Alk), [Dat](http://flybase.bio.indiana.edu/.bin/fbidq.html?Dat), [Pde1c](http://flybase.bio.indiana.edu/.bin/fbidq.html?Pde1c), [gfA](http://flybase.bio.indiana.edu/.bin/fbidq.html?gfA), [Tk](http://flybase.bio.indiana.edu/.bin/fbidq.html?Tk), [Plc21C](http://flybase.bio.indiana.edu/.bin/fbidq.html?Plc21C), [Ilp5](http://flybase.bio.indiana.edu/.bin/fbidq.html?Ilp5), [Rdl](http://flybase.bio.indiana.edu/.bin/fbidq.html?Rdl), [GABA-B-R2](http://flybase.bio.indiana.edu/.bin/fbidq.html?GABA-B-R2), [tutl](http://flybase.bio.indiana.edu/.bin/fbidq.html?tutl), [futsch](http://flybase.bio.indiana.edu/.bin/fbidq.html?futsch), [Ilp2](http://flybase.bio.indiana.edu/.bin/fbidq.html?Ilp2), [CG3967](http://flybase.bio.indiana.edu/.bin/fbidq.html?CG3967), [hug](http://flybase.bio.indiana.edu/.bin/fbidq.html?hug), [pigeon](http://flybase.bio.indiana.edu/.bin/fbidq.html?pigeon), [Ggamma1](http://flybase.bio.indiana.edu/.bin/fbidq.html?Ggamma1), [Gpo-1](http://flybase.bio.indiana.edu/.bin/fbidq.html?Gpo-1),[para](http://flybase.bio.indiana.edu/.bin/fbidq.html?para), [sm](http://flybase.bio.indiana.edu/.bin/fbidq.html?sm), [esg](http://flybase.bio.indiana.edu/.bin/fbidq.html?esg), [eas](http://flybase.bio.indiana.edu/.bin/fbidq.html?eas), [CRMP](http://flybase.bio.indiana.edu/.bin/fbidq.html?CRMP), [Nrx-1](http://flybase.bio.indiana.edu/.bin/fbidq.html?Nrx-1), [Cdk5](http://flybase.bio.indiana.edu/.bin/fbidq.html?Cdk5), [oc](http://flybase.bio.indiana.edu/.bin/fbidq.html?oc), [Tbh](http://flybase.bio.indiana.edu/.bin/fbidq.html?Tbh), [ey](http://flybase.bio.indiana.edu/.bin/fbidq.html?ey), [Oamb](http://flybase.bio.indiana.edu/.bin/fbidq.html?Oamb), [mle](http://flybase.bio.indiana.edu/.bin/fbidq.html?mle), [shi](http://flybase.bio.indiana.edu/.bin/fbidq.html?shi), [siz](http://flybase.bio.indiana.edu/.bin/fbidq.html?siz), [Cdk5alpha](http://flybase.bio.indiana.edu/.bin/fbidq.html?Cdk5alpha), [qtc](http://flybase.bio.indiana.edu/.bin/fbidq.html?qtc), [CadN](http://flybase.bio.indiana.edu/.bin/fbidq.html?CadN), [fne](http://flybase.bio.indiana.edu/.bin/fbidq.html?fne), [retn](http://flybase.bio.indiana.edu/.bin/fbidq.html?retn), [Ac3](http://flybase.bio.indiana.edu/.bin/fbidq.html?Ac3), [klg](http://flybase.bio.indiana.edu/.bin/fbidq.html?klg), [Syt1](http://flybase.bio.indiana.edu/.bin/fbidq.html?Syt1), [rl](http://flybase.bio.indiana.edu/.bin/fbidq.html?rl), [Sip1](http://flybase.bio.indiana.edu/.bin/fbidq.html?Sip1), [hiw](http://flybase.bio.indiana.edu/.bin/fbidq.html?hiw) |
| [regulation of transcription, DNA-dependent](http://amigo.geneontology.org/cgi-bin/amigo/go.cgi?view=details&query=GO:0006355) | 195 of 1594 genes, 12.2% | 600 of 7634 genes, 7.9% | 3.28e-11 | 0.00% | 0.00 | [fz2](http://flybase.bio.indiana.edu/.bin/fbidq.html?fz2), [acj6](http://flybase.bio.indiana.edu/.bin/fbidq.html?acj6), [skd](http://flybase.bio.indiana.edu/.bin/fbidq.html?skd), [jing](http://flybase.bio.indiana.edu/.bin/fbidq.html?jing), [caup](http://flybase.bio.indiana.edu/.bin/fbidq.html?caup), [tll](http://flybase.bio.indiana.edu/.bin/fbidq.html?tll), [Camta](http://flybase.bio.indiana.edu/.bin/fbidq.html?Camta), [ato](http://flybase.bio.indiana.edu/.bin/fbidq.html?ato), [Ets65A](http://flybase.bio.indiana.edu/.bin/fbidq.html?Ets65A), [tna](http://flybase.bio.indiana.edu/.bin/fbidq.html?tna), [JIL-1](http://flybase.bio.indiana.edu/.bin/fbidq.html?JIL-1), [Spt6](http://flybase.bio.indiana.edu/.bin/fbidq.html?Spt6), [Iswi](http://flybase.bio.indiana.edu/.bin/fbidq.html?Iswi), [rn](http://flybase.bio.indiana.edu/.bin/fbidq.html?rn), [ap](http://flybase.bio.indiana.edu/.bin/fbidq.html?ap), [br](http://flybase.bio.indiana.edu/.bin/fbidq.html?br), [ksr](http://flybase.bio.indiana.edu/.bin/fbidq.html?ksr), [bi](http://flybase.bio.indiana.edu/.bin/fbidq.html?bi), [chn](http://flybase.bio.indiana.edu/.bin/fbidq.html?chn), [Gsc](http://flybase.bio.indiana.edu/.bin/fbidq.html?Gsc), [Hcf](http://flybase.bio.indiana.edu/.bin/fbidq.html?Hcf), [lola](http://flybase.bio.indiana.edu/.bin/fbidq.html?lola), [HLH4C](http://flybase.bio.indiana.edu/.bin/fbidq.html?HLH4C), [Pcl](http://flybase.bio.indiana.edu/.bin/fbidq.html?Pcl), [simj](http://flybase.bio.indiana.edu/.bin/fbidq.html?simj), [Awh](http://flybase.bio.indiana.edu/.bin/fbidq.html?Awh), [tap](http://flybase.bio.indiana.edu/.bin/fbidq.html?tap), [NC2alpha](http://flybase.bio.indiana.edu/.bin/fbidq.html?NC2alpha), [PHDP](http://flybase.bio.indiana.edu/.bin/fbidq.html?PHDP), [bab1](http://flybase.bio.indiana.edu/.bin/fbidq.html?bab1), [CG4328](http://flybase.bio.indiana.edu/.bin/fbidq.html?CG4328), [Snoo](http://flybase.bio.indiana.edu/.bin/fbidq.html?Snoo), [l(2)NC136](http://flybase.bio.indiana.edu/.bin/fbidq.html?l(2)NC136), [Taf4](http://flybase.bio.indiana.edu/.bin/fbidq.html?Taf4), [Su(var)3-3](http://flybase.bio.indiana.edu/.bin/fbidq.html?Su(var)3-3), [HGTX](http://flybase.bio.indiana.edu/.bin/fbidq.html?HGTX), [not](http://flybase.bio.indiana.edu/.bin/fbidq.html?not), [nvy](http://flybase.bio.indiana.edu/.bin/fbidq.html?nvy), [nub](http://flybase.bio.indiana.edu/.bin/fbidq.html?nub), [nab](http://flybase.bio.indiana.edu/.bin/fbidq.html?nab), [btd](http://flybase.bio.indiana.edu/.bin/fbidq.html?btd),[fd68A](http://flybase.bio.indiana.edu/.bin/fbidq.html?fd68A), [HLHmdelta](http://flybase.bio.indiana.edu/.bin/fbidq.html?HLHmdelta), [B-H1](http://flybase.bio.indiana.edu/.bin/fbidq.html?B-H1), [Nipped-A](http://flybase.bio.indiana.edu/.bin/fbidq.html?Nipped-A), [ATbp](http://flybase.bio.indiana.edu/.bin/fbidq.html?ATbp), [Sos](http://flybase.bio.indiana.edu/.bin/fbidq.html?Sos), [Fer2](http://flybase.bio.indiana.edu/.bin/fbidq.html?Fer2), [fd102C](http://flybase.bio.indiana.edu/.bin/fbidq.html?fd102C), [vg](http://flybase.bio.indiana.edu/.bin/fbidq.html?vg), [hth](http://flybase.bio.indiana.edu/.bin/fbidq.html?hth), [Nf-YC](http://flybase.bio.indiana.edu/.bin/fbidq.html?Nf-YC), [eya](http://flybase.bio.indiana.edu/.bin/fbidq.html?eya), [fz](http://flybase.bio.indiana.edu/.bin/fbidq.html?fz), [brm](http://flybase.bio.indiana.edu/.bin/fbidq.html?brm), [Vsx2](http://flybase.bio.indiana.edu/.bin/fbidq.html?Vsx2), [Hey](http://flybase.bio.indiana.edu/.bin/fbidq.html?Hey), [sno](http://flybase.bio.indiana.edu/.bin/fbidq.html?sno), [Sfmbt](http://flybase.bio.indiana.edu/.bin/fbidq.html?Sfmbt), [ara](http://flybase.bio.indiana.edu/.bin/fbidq.html?ara), [mam](http://flybase.bio.indiana.edu/.bin/fbidq.html?mam), [pan](http://flybase.bio.indiana.edu/.bin/fbidq.html?pan), [Dll](http://flybase.bio.indiana.edu/.bin/fbidq.html?Dll), [disco](http://flybase.bio.indiana.edu/.bin/fbidq.html?disco), [grn](http://flybase.bio.indiana.edu/.bin/fbidq.html?grn), [HLHm5](http://flybase.bio.indiana.edu/.bin/fbidq.html?HLHm5), [D12](http://flybase.bio.indiana.edu/.bin/fbidq.html?D12), [Lis-1](http://flybase.bio.indiana.edu/.bin/fbidq.html?Lis-1), [Brf](http://flybase.bio.indiana.edu/.bin/fbidq.html?Brf), [ems](http://flybase.bio.indiana.edu/.bin/fbidq.html?ems), [pros](http://flybase.bio.indiana.edu/.bin/fbidq.html?pros), [tup](http://flybase.bio.indiana.edu/.bin/fbidq.html?tup), [pdm2](http://flybase.bio.indiana.edu/.bin/fbidq.html?pdm2), [Gug](http://flybase.bio.indiana.edu/.bin/fbidq.html?Gug), [pad](http://flybase.bio.indiana.edu/.bin/fbidq.html?pad), [rgr](http://flybase.bio.indiana.edu/.bin/fbidq.html?rgr), [Wnt4](http://flybase.bio.indiana.edu/.bin/fbidq.html?Wnt4), [Rx](http://flybase.bio.indiana.edu/.bin/fbidq.html?Rx), [MED1](http://flybase.bio.indiana.edu/.bin/fbidq.html?MED1), [salr](http://flybase.bio.indiana.edu/.bin/fbidq.html?salr), [wdn](http://flybase.bio.indiana.edu/.bin/fbidq.html?wdn), [sim](http://flybase.bio.indiana.edu/.bin/fbidq.html?sim),[d4](http://flybase.bio.indiana.edu/.bin/fbidq.html?d4), [dan](http://flybase.bio.indiana.edu/.bin/fbidq.html?dan), [gro](http://flybase.bio.indiana.edu/.bin/fbidq.html?gro), [nerfin-1](http://flybase.bio.indiana.edu/.bin/fbidq.html?nerfin-1), [trx](http://flybase.bio.indiana.edu/.bin/fbidq.html?trx), [Rpd3](http://flybase.bio.indiana.edu/.bin/fbidq.html?Rpd3), [dsx](http://flybase.bio.indiana.edu/.bin/fbidq.html?dsx), [Ssdp](http://flybase.bio.indiana.edu/.bin/fbidq.html?Ssdp), [vfl](http://flybase.bio.indiana.edu/.bin/fbidq.html?vfl), [sqz](http://flybase.bio.indiana.edu/.bin/fbidq.html?sqz), [CG32105](http://flybase.bio.indiana.edu/.bin/fbidq.html?CG32105), [Fer3](http://flybase.bio.indiana.edu/.bin/fbidq.html?Fer3), [pygo](http://flybase.bio.indiana.edu/.bin/fbidq.html?pygo), [dalao](http://flybase.bio.indiana.edu/.bin/fbidq.html?dalao), [HLHm7](http://flybase.bio.indiana.edu/.bin/fbidq.html?HLHm7), [z](http://flybase.bio.indiana.edu/.bin/fbidq.html?z), [HLHmgamma](http://flybase.bio.indiana.edu/.bin/fbidq.html?HLHmgamma), [Sox21b](http://flybase.bio.indiana.edu/.bin/fbidq.html?Sox21b), [sr](http://flybase.bio.indiana.edu/.bin/fbidq.html?sr), [Lim1](http://flybase.bio.indiana.edu/.bin/fbidq.html?Lim1), [tld](http://flybase.bio.indiana.edu/.bin/fbidq.html?tld), [danr](http://flybase.bio.indiana.edu/.bin/fbidq.html?danr), [wor](http://flybase.bio.indiana.edu/.bin/fbidq.html?wor), [vvl](http://flybase.bio.indiana.edu/.bin/fbidq.html?vvl), [wda](http://flybase.bio.indiana.edu/.bin/fbidq.html?wda), [sgg](http://flybase.bio.indiana.edu/.bin/fbidq.html?sgg), [MED14](http://flybase.bio.indiana.edu/.bin/fbidq.html?MED14), [gcm2](http://flybase.bio.indiana.edu/.bin/fbidq.html?gcm2), [Eip93F](http://flybase.bio.indiana.edu/.bin/fbidq.html?Eip93F), [gcm](http://flybase.bio.indiana.edu/.bin/fbidq.html?gcm), [seq](http://flybase.bio.indiana.edu/.bin/fbidq.html?seq), [Dip3](http://flybase.bio.indiana.edu/.bin/fbidq.html?Dip3), [Aef1](http://flybase.bio.indiana.edu/.bin/fbidq.html?Aef1), [HDAC4](http://flybase.bio.indiana.edu/.bin/fbidq.html?HDAC4), [can](http://flybase.bio.indiana.edu/.bin/fbidq.html?can), [elk](http://flybase.bio.indiana.edu/.bin/fbidq.html?elk), [phl](http://flybase.bio.indiana.edu/.bin/fbidq.html?phl), [ase](http://flybase.bio.indiana.edu/.bin/fbidq.html?ase), [CG13253](http://flybase.bio.indiana.edu/.bin/fbidq.html?CG13253),[ovo](http://flybase.bio.indiana.edu/.bin/fbidq.html?ovo), [scro](http://flybase.bio.indiana.edu/.bin/fbidq.html?scro), [Dsp1](http://flybase.bio.indiana.edu/.bin/fbidq.html?Dsp1), [CG14216](http://flybase.bio.indiana.edu/.bin/fbidq.html?CG14216), [Pdp1](http://flybase.bio.indiana.edu/.bin/fbidq.html?Pdp1), [Oli](http://flybase.bio.indiana.edu/.bin/fbidq.html?Oli), [Nelf-A](http://flybase.bio.indiana.edu/.bin/fbidq.html?Nelf-A), [GATAd](http://flybase.bio.indiana.edu/.bin/fbidq.html?GATAd), [Poxn](http://flybase.bio.indiana.edu/.bin/fbidq.html?Poxn), [CG15376](http://flybase.bio.indiana.edu/.bin/fbidq.html?CG15376), [Sin3A](http://flybase.bio.indiana.edu/.bin/fbidq.html?Sin3A), [mip120](http://flybase.bio.indiana.edu/.bin/fbidq.html?mip120), [jumu](http://flybase.bio.indiana.edu/.bin/fbidq.html?jumu), [Smox](http://flybase.bio.indiana.edu/.bin/fbidq.html?Smox), [Usp7](http://flybase.bio.indiana.edu/.bin/fbidq.html?Usp7), [ush](http://flybase.bio.indiana.edu/.bin/fbidq.html?ush), [fd59A](http://flybase.bio.indiana.edu/.bin/fbidq.html?fd59A), [Gap1](http://flybase.bio.indiana.edu/.bin/fbidq.html?Gap1), [run](http://flybase.bio.indiana.edu/.bin/fbidq.html?run), [FBX011](http://flybase.bio.indiana.edu/.bin/fbidq.html?FBX011), [Antp](http://flybase.bio.indiana.edu/.bin/fbidq.html?Antp), [tara](http://flybase.bio.indiana.edu/.bin/fbidq.html?tara), [Alh](http://flybase.bio.indiana.edu/.bin/fbidq.html?Alh), [Sox102F](http://flybase.bio.indiana.edu/.bin/fbidq.html?Sox102F), [egg](http://flybase.bio.indiana.edu/.bin/fbidq.html?egg), [l(3)neo38](http://flybase.bio.indiana.edu/.bin/fbidq.html?l(3)neo38), [koko](http://flybase.bio.indiana.edu/.bin/fbidq.html?koko), [Ptth](http://flybase.bio.indiana.edu/.bin/fbidq.html?Ptth), [Sp1](http://flybase.bio.indiana.edu/.bin/fbidq.html?Sp1), [Ptx1](http://flybase.bio.indiana.edu/.bin/fbidq.html?Ptx1), [ph-p](http://flybase.bio.indiana.edu/.bin/fbidq.html?ph-p), [Ubx](http://flybase.bio.indiana.edu/.bin/fbidq.html?Ubx), [onecut](http://flybase.bio.indiana.edu/.bin/fbidq.html?onecut), [CG7757](http://flybase.bio.indiana.edu/.bin/fbidq.html?CG7757), [chm](http://flybase.bio.indiana.edu/.bin/fbidq.html?chm), [sna](http://flybase.bio.indiana.edu/.bin/fbidq.html?sna),[Wnt5](http://flybase.bio.indiana.edu/.bin/fbidq.html?Wnt5), [Taf1](http://flybase.bio.indiana.edu/.bin/fbidq.html?Taf1), [hbn](http://flybase.bio.indiana.edu/.bin/fbidq.html?hbn), [MBD-like](http://flybase.bio.indiana.edu/.bin/fbidq.html?MBD-like), [tsh](http://flybase.bio.indiana.edu/.bin/fbidq.html?tsh), [esg](http://flybase.bio.indiana.edu/.bin/fbidq.html?esg), [e(y)3](http://flybase.bio.indiana.edu/.bin/fbidq.html?e(y)3), [CG9007](http://flybase.bio.indiana.edu/.bin/fbidq.html?CG9007), [oc](http://flybase.bio.indiana.edu/.bin/fbidq.html?oc), [spt4](http://flybase.bio.indiana.edu/.bin/fbidq.html?spt4), [CG32532](http://flybase.bio.indiana.edu/.bin/fbidq.html?CG32532), [MTA1-like](http://flybase.bio.indiana.edu/.bin/fbidq.html?MTA1-like), [gol](http://flybase.bio.indiana.edu/.bin/fbidq.html?gol), [ey](http://flybase.bio.indiana.edu/.bin/fbidq.html?ey), [mle](http://flybase.bio.indiana.edu/.bin/fbidq.html?mle), [Hr51](http://flybase.bio.indiana.edu/.bin/fbidq.html?Hr51), [Bgb](http://flybase.bio.indiana.edu/.bin/fbidq.html?Bgb), [l(1)sc](http://flybase.bio.indiana.edu/.bin/fbidq.html?l(1)sc), [B-H2](http://flybase.bio.indiana.edu/.bin/fbidq.html?B-H2), [grh](http://flybase.bio.indiana.edu/.bin/fbidq.html?grh), [Vsx1](http://flybase.bio.indiana.edu/.bin/fbidq.html?Vsx1), [wg](http://flybase.bio.indiana.edu/.bin/fbidq.html?wg), [toy](http://flybase.bio.indiana.edu/.bin/fbidq.html?toy), [fkh](http://flybase.bio.indiana.edu/.bin/fbidq.html?fkh), [Lim3](http://flybase.bio.indiana.edu/.bin/fbidq.html?Lim3), [crol](http://flybase.bio.indiana.edu/.bin/fbidq.html?crol), [sd](http://flybase.bio.indiana.edu/.bin/fbidq.html?sd), [retn](http://flybase.bio.indiana.edu/.bin/fbidq.html?retn), [en](http://flybase.bio.indiana.edu/.bin/fbidq.html?en), [fs(1)h](http://flybase.bio.indiana.edu/.bin/fbidq.html?fs(1)h), [rl](http://flybase.bio.indiana.edu/.bin/fbidq.html?rl), [CG11347](http://flybase.bio.indiana.edu/.bin/fbidq.html?CG11347), [tey](http://flybase.bio.indiana.edu/.bin/fbidq.html?tey), [CG11294](http://flybase.bio.indiana.edu/.bin/fbidq.html?CG11294), [hiw](http://flybase.bio.indiana.edu/.bin/fbidq.html?hiw), [cos](http://flybase.bio.indiana.edu/.bin/fbidq.html?cos), [Ct](http://flybase.bio.indiana.edu/.bin/fbidq.html?Ct), [Atf-2](http://flybase.bio.indiana.edu/.bin/fbidq.html?Atf-2) |
| [regulation of RNA biosynthetic process](http://amigo.geneontology.org/cgi-bin/amigo/go.cgi?view=details&query=GO:2001141) | 195 of 1594 genes, 12.2% | 600 of 7634 genes, 7.9% | 3.28e-11 | 0.00% | 0.00 | [fz2](http://flybase.bio.indiana.edu/.bin/fbidq.html?fz2), [acj6](http://flybase.bio.indiana.edu/.bin/fbidq.html?acj6), [skd](http://flybase.bio.indiana.edu/.bin/fbidq.html?skd), [jing](http://flybase.bio.indiana.edu/.bin/fbidq.html?jing), [caup](http://flybase.bio.indiana.edu/.bin/fbidq.html?caup), [tll](http://flybase.bio.indiana.edu/.bin/fbidq.html?tll), [Camta](http://flybase.bio.indiana.edu/.bin/fbidq.html?Camta), [ato](http://flybase.bio.indiana.edu/.bin/fbidq.html?ato), [Ets65A](http://flybase.bio.indiana.edu/.bin/fbidq.html?Ets65A), [tna](http://flybase.bio.indiana.edu/.bin/fbidq.html?tna), [JIL-1](http://flybase.bio.indiana.edu/.bin/fbidq.html?JIL-1), [Spt6](http://flybase.bio.indiana.edu/.bin/fbidq.html?Spt6), [Iswi](http://flybase.bio.indiana.edu/.bin/fbidq.html?Iswi), [rn](http://flybase.bio.indiana.edu/.bin/fbidq.html?rn), [ap](http://flybase.bio.indiana.edu/.bin/fbidq.html?ap), [br](http://flybase.bio.indiana.edu/.bin/fbidq.html?br), [ksr](http://flybase.bio.indiana.edu/.bin/fbidq.html?ksr), [bi](http://flybase.bio.indiana.edu/.bin/fbidq.html?bi), [chn](http://flybase.bio.indiana.edu/.bin/fbidq.html?chn), [Gsc](http://flybase.bio.indiana.edu/.bin/fbidq.html?Gsc), [Hcf](http://flybase.bio.indiana.edu/.bin/fbidq.html?Hcf), [lola](http://flybase.bio.indiana.edu/.bin/fbidq.html?lola), [HLH4C](http://flybase.bio.indiana.edu/.bin/fbidq.html?HLH4C), [Pcl](http://flybase.bio.indiana.edu/.bin/fbidq.html?Pcl), [simj](http://flybase.bio.indiana.edu/.bin/fbidq.html?simj), [Awh](http://flybase.bio.indiana.edu/.bin/fbidq.html?Awh), [tap](http://flybase.bio.indiana.edu/.bin/fbidq.html?tap), [NC2alpha](http://flybase.bio.indiana.edu/.bin/fbidq.html?NC2alpha), [PHDP](http://flybase.bio.indiana.edu/.bin/fbidq.html?PHDP), [bab1](http://flybase.bio.indiana.edu/.bin/fbidq.html?bab1), [CG4328](http://flybase.bio.indiana.edu/.bin/fbidq.html?CG4328), [Snoo](http://flybase.bio.indiana.edu/.bin/fbidq.html?Snoo), [l(2)NC136](http://flybase.bio.indiana.edu/.bin/fbidq.html?l(2)NC136), [Taf4](http://flybase.bio.indiana.edu/.bin/fbidq.html?Taf4), [Su(var)3-3](http://flybase.bio.indiana.edu/.bin/fbidq.html?Su(var)3-3), [HGTX](http://flybase.bio.indiana.edu/.bin/fbidq.html?HGTX), [not](http://flybase.bio.indiana.edu/.bin/fbidq.html?not), [nvy](http://flybase.bio.indiana.edu/.bin/fbidq.html?nvy), [nub](http://flybase.bio.indiana.edu/.bin/fbidq.html?nub), [nab](http://flybase.bio.indiana.edu/.bin/fbidq.html?nab), [btd](http://flybase.bio.indiana.edu/.bin/fbidq.html?btd),[fd68A](http://flybase.bio.indiana.edu/.bin/fbidq.html?fd68A), [HLHmdelta](http://flybase.bio.indiana.edu/.bin/fbidq.html?HLHmdelta), [B-H1](http://flybase.bio.indiana.edu/.bin/fbidq.html?B-H1), [Nipped-A](http://flybase.bio.indiana.edu/.bin/fbidq.html?Nipped-A), [ATbp](http://flybase.bio.indiana.edu/.bin/fbidq.html?ATbp), [Sos](http://flybase.bio.indiana.edu/.bin/fbidq.html?Sos), [Fer2](http://flybase.bio.indiana.edu/.bin/fbidq.html?Fer2), [fd102C](http://flybase.bio.indiana.edu/.bin/fbidq.html?fd102C), [vg](http://flybase.bio.indiana.edu/.bin/fbidq.html?vg), [hth](http://flybase.bio.indiana.edu/.bin/fbidq.html?hth), [Nf-YC](http://flybase.bio.indiana.edu/.bin/fbidq.html?Nf-YC), [eya](http://flybase.bio.indiana.edu/.bin/fbidq.html?eya), [fz](http://flybase.bio.indiana.edu/.bin/fbidq.html?fz), [brm](http://flybase.bio.indiana.edu/.bin/fbidq.html?brm), [Vsx2](http://flybase.bio.indiana.edu/.bin/fbidq.html?Vsx2), [Hey](http://flybase.bio.indiana.edu/.bin/fbidq.html?Hey), [sno](http://flybase.bio.indiana.edu/.bin/fbidq.html?sno), [Sfmbt](http://flybase.bio.indiana.edu/.bin/fbidq.html?Sfmbt), [ara](http://flybase.bio.indiana.edu/.bin/fbidq.html?ara), [mam](http://flybase.bio.indiana.edu/.bin/fbidq.html?mam), [pan](http://flybase.bio.indiana.edu/.bin/fbidq.html?pan), [Dll](http://flybase.bio.indiana.edu/.bin/fbidq.html?Dll), [disco](http://flybase.bio.indiana.edu/.bin/fbidq.html?disco), [grn](http://flybase.bio.indiana.edu/.bin/fbidq.html?grn), [HLHm5](http://flybase.bio.indiana.edu/.bin/fbidq.html?HLHm5), [D12](http://flybase.bio.indiana.edu/.bin/fbidq.html?D12), [Lis-1](http://flybase.bio.indiana.edu/.bin/fbidq.html?Lis-1), [Brf](http://flybase.bio.indiana.edu/.bin/fbidq.html?Brf), [ems](http://flybase.bio.indiana.edu/.bin/fbidq.html?ems), [pros](http://flybase.bio.indiana.edu/.bin/fbidq.html?pros), [tup](http://flybase.bio.indiana.edu/.bin/fbidq.html?tup), [pdm2](http://flybase.bio.indiana.edu/.bin/fbidq.html?pdm2), [Gug](http://flybase.bio.indiana.edu/.bin/fbidq.html?Gug), [pad](http://flybase.bio.indiana.edu/.bin/fbidq.html?pad), [rgr](http://flybase.bio.indiana.edu/.bin/fbidq.html?rgr), [Wnt4](http://flybase.bio.indiana.edu/.bin/fbidq.html?Wnt4), [Rx](http://flybase.bio.indiana.edu/.bin/fbidq.html?Rx), [MED1](http://flybase.bio.indiana.edu/.bin/fbidq.html?MED1), [salr](http://flybase.bio.indiana.edu/.bin/fbidq.html?salr), [wdn](http://flybase.bio.indiana.edu/.bin/fbidq.html?wdn), [sim](http://flybase.bio.indiana.edu/.bin/fbidq.html?sim),[d4](http://flybase.bio.indiana.edu/.bin/fbidq.html?d4), [dan](http://flybase.bio.indiana.edu/.bin/fbidq.html?dan), [gro](http://flybase.bio.indiana.edu/.bin/fbidq.html?gro), [nerfin-1](http://flybase.bio.indiana.edu/.bin/fbidq.html?nerfin-1), [trx](http://flybase.bio.indiana.edu/.bin/fbidq.html?trx), [Rpd3](http://flybase.bio.indiana.edu/.bin/fbidq.html?Rpd3), [dsx](http://flybase.bio.indiana.edu/.bin/fbidq.html?dsx), [Ssdp](http://flybase.bio.indiana.edu/.bin/fbidq.html?Ssdp), [vfl](http://flybase.bio.indiana.edu/.bin/fbidq.html?vfl), [sqz](http://flybase.bio.indiana.edu/.bin/fbidq.html?sqz), [CG32105](http://flybase.bio.indiana.edu/.bin/fbidq.html?CG32105), [Fer3](http://flybase.bio.indiana.edu/.bin/fbidq.html?Fer3), [pygo](http://flybase.bio.indiana.edu/.bin/fbidq.html?pygo), [dalao](http://flybase.bio.indiana.edu/.bin/fbidq.html?dalao), [HLHm7](http://flybase.bio.indiana.edu/.bin/fbidq.html?HLHm7), [z](http://flybase.bio.indiana.edu/.bin/fbidq.html?z), [HLHmgamma](http://flybase.bio.indiana.edu/.bin/fbidq.html?HLHmgamma), [Sox21b](http://flybase.bio.indiana.edu/.bin/fbidq.html?Sox21b), [sr](http://flybase.bio.indiana.edu/.bin/fbidq.html?sr), [Lim1](http://flybase.bio.indiana.edu/.bin/fbidq.html?Lim1), [tld](http://flybase.bio.indiana.edu/.bin/fbidq.html?tld), [danr](http://flybase.bio.indiana.edu/.bin/fbidq.html?danr), [wor](http://flybase.bio.indiana.edu/.bin/fbidq.html?wor), [vvl](http://flybase.bio.indiana.edu/.bin/fbidq.html?vvl), [wda](http://flybase.bio.indiana.edu/.bin/fbidq.html?wda), [sgg](http://flybase.bio.indiana.edu/.bin/fbidq.html?sgg), [MED14](http://flybase.bio.indiana.edu/.bin/fbidq.html?MED14), [gcm2](http://flybase.bio.indiana.edu/.bin/fbidq.html?gcm2), [Eip93F](http://flybase.bio.indiana.edu/.bin/fbidq.html?Eip93F), [gcm](http://flybase.bio.indiana.edu/.bin/fbidq.html?gcm), [seq](http://flybase.bio.indiana.edu/.bin/fbidq.html?seq), [Dip3](http://flybase.bio.indiana.edu/.bin/fbidq.html?Dip3), [Aef1](http://flybase.bio.indiana.edu/.bin/fbidq.html?Aef1), [HDAC4](http://flybase.bio.indiana.edu/.bin/fbidq.html?HDAC4), [can](http://flybase.bio.indiana.edu/.bin/fbidq.html?can), [elk](http://flybase.bio.indiana.edu/.bin/fbidq.html?elk), [phl](http://flybase.bio.indiana.edu/.bin/fbidq.html?phl), [ase](http://flybase.bio.indiana.edu/.bin/fbidq.html?ase), [CG13253](http://flybase.bio.indiana.edu/.bin/fbidq.html?CG13253),[ovo](http://flybase.bio.indiana.edu/.bin/fbidq.html?ovo), [scro](http://flybase.bio.indiana.edu/.bin/fbidq.html?scro), [Dsp1](http://flybase.bio.indiana.edu/.bin/fbidq.html?Dsp1), [CG14216](http://flybase.bio.indiana.edu/.bin/fbidq.html?CG14216), [Pdp1](http://flybase.bio.indiana.edu/.bin/fbidq.html?Pdp1), [Oli](http://flybase.bio.indiana.edu/.bin/fbidq.html?Oli), [Nelf-A](http://flybase.bio.indiana.edu/.bin/fbidq.html?Nelf-A), [GATAd](http://flybase.bio.indiana.edu/.bin/fbidq.html?GATAd), [Poxn](http://flybase.bio.indiana.edu/.bin/fbidq.html?Poxn), [CG15376](http://flybase.bio.indiana.edu/.bin/fbidq.html?CG15376), [Sin3A](http://flybase.bio.indiana.edu/.bin/fbidq.html?Sin3A), [mip120](http://flybase.bio.indiana.edu/.bin/fbidq.html?mip120), [jumu](http://flybase.bio.indiana.edu/.bin/fbidq.html?jumu), [Smox](http://flybase.bio.indiana.edu/.bin/fbidq.html?Smox), [Usp7](http://flybase.bio.indiana.edu/.bin/fbidq.html?Usp7), [ush](http://flybase.bio.indiana.edu/.bin/fbidq.html?ush), [fd59A](http://flybase.bio.indiana.edu/.bin/fbidq.html?fd59A), [Gap1](http://flybase.bio.indiana.edu/.bin/fbidq.html?Gap1), [run](http://flybase.bio.indiana.edu/.bin/fbidq.html?run), [FBX011](http://flybase.bio.indiana.edu/.bin/fbidq.html?FBX011), [Antp](http://flybase.bio.indiana.edu/.bin/fbidq.html?Antp), [tara](http://flybase.bio.indiana.edu/.bin/fbidq.html?tara), [Alh](http://flybase.bio.indiana.edu/.bin/fbidq.html?Alh), [Sox102F](http://flybase.bio.indiana.edu/.bin/fbidq.html?Sox102F), [egg](http://flybase.bio.indiana.edu/.bin/fbidq.html?egg), [l(3)neo38](http://flybase.bio.indiana.edu/.bin/fbidq.html?l(3)neo38), [koko](http://flybase.bio.indiana.edu/.bin/fbidq.html?koko), [Ptth](http://flybase.bio.indiana.edu/.bin/fbidq.html?Ptth), [Sp1](http://flybase.bio.indiana.edu/.bin/fbidq.html?Sp1), [Ptx1](http://flybase.bio.indiana.edu/.bin/fbidq.html?Ptx1), [ph-p](http://flybase.bio.indiana.edu/.bin/fbidq.html?ph-p), [Ubx](http://flybase.bio.indiana.edu/.bin/fbidq.html?Ubx), [onecut](http://flybase.bio.indiana.edu/.bin/fbidq.html?onecut), [CG7757](http://flybase.bio.indiana.edu/.bin/fbidq.html?CG7757), [chm](http://flybase.bio.indiana.edu/.bin/fbidq.html?chm), [sna](http://flybase.bio.indiana.edu/.bin/fbidq.html?sna),[Wnt5](http://flybase.bio.indiana.edu/.bin/fbidq.html?Wnt5), [Taf1](http://flybase.bio.indiana.edu/.bin/fbidq.html?Taf1), [hbn](http://flybase.bio.indiana.edu/.bin/fbidq.html?hbn), [MBD-like](http://flybase.bio.indiana.edu/.bin/fbidq.html?MBD-like), [tsh](http://flybase.bio.indiana.edu/.bin/fbidq.html?tsh), [esg](http://flybase.bio.indiana.edu/.bin/fbidq.html?esg), [e(y)3](http://flybase.bio.indiana.edu/.bin/fbidq.html?e(y)3), [CG9007](http://flybase.bio.indiana.edu/.bin/fbidq.html?CG9007), [oc](http://flybase.bio.indiana.edu/.bin/fbidq.html?oc), [spt4](http://flybase.bio.indiana.edu/.bin/fbidq.html?spt4), [CG32532](http://flybase.bio.indiana.edu/.bin/fbidq.html?CG32532), [MTA1-like](http://flybase.bio.indiana.edu/.bin/fbidq.html?MTA1-like), [gol](http://flybase.bio.indiana.edu/.bin/fbidq.html?gol), [ey](http://flybase.bio.indiana.edu/.bin/fbidq.html?ey), [mle](http://flybase.bio.indiana.edu/.bin/fbidq.html?mle), [Hr51](http://flybase.bio.indiana.edu/.bin/fbidq.html?Hr51), [Bgb](http://flybase.bio.indiana.edu/.bin/fbidq.html?Bgb), [l(1)sc](http://flybase.bio.indiana.edu/.bin/fbidq.html?l(1)sc), [B-H2](http://flybase.bio.indiana.edu/.bin/fbidq.html?B-H2), [grh](http://flybase.bio.indiana.edu/.bin/fbidq.html?grh), [Vsx1](http://flybase.bio.indiana.edu/.bin/fbidq.html?Vsx1), [wg](http://flybase.bio.indiana.edu/.bin/fbidq.html?wg), [toy](http://flybase.bio.indiana.edu/.bin/fbidq.html?toy), [fkh](http://flybase.bio.indiana.edu/.bin/fbidq.html?fkh), [Lim3](http://flybase.bio.indiana.edu/.bin/fbidq.html?Lim3), [crol](http://flybase.bio.indiana.edu/.bin/fbidq.html?crol), [sd](http://flybase.bio.indiana.edu/.bin/fbidq.html?sd), [retn](http://flybase.bio.indiana.edu/.bin/fbidq.html?retn), [en](http://flybase.bio.indiana.edu/.bin/fbidq.html?en), [fs(1)h](http://flybase.bio.indiana.edu/.bin/fbidq.html?fs(1)h), [rl](http://flybase.bio.indiana.edu/.bin/fbidq.html?rl), [CG11347](http://flybase.bio.indiana.edu/.bin/fbidq.html?CG11347), [tey](http://flybase.bio.indiana.edu/.bin/fbidq.html?tey), [CG11294](http://flybase.bio.indiana.edu/.bin/fbidq.html?CG11294), [hiw](http://flybase.bio.indiana.edu/.bin/fbidq.html?hiw), [cos](http://flybase.bio.indiana.edu/.bin/fbidq.html?cos), [Ct](http://flybase.bio.indiana.edu/.bin/fbidq.html?Ct), [Atf-2](http://flybase.bio.indiana.edu/.bin/fbidq.html?Atf-2) |
| [cell morphogenesis involved in differentiation](http://amigo.geneontology.org/cgi-bin/amigo/go.cgi?view=details&query=GO:0000904) | 137 of 1594 genes, 8.6% | 378 of 7634 genes, 5.0% | 4.16e-11 | 0.00% | 0.00 | [fz2](http://flybase.bio.indiana.edu/.bin/fbidq.html?fz2), [Sh](http://flybase.bio.indiana.edu/.bin/fbidq.html?Sh), [acj6](http://flybase.bio.indiana.edu/.bin/fbidq.html?acj6), [jing](http://flybase.bio.indiana.edu/.bin/fbidq.html?jing), [dock](http://flybase.bio.indiana.edu/.bin/fbidq.html?dock), [ato](http://flybase.bio.indiana.edu/.bin/fbidq.html?ato), [ap](http://flybase.bio.indiana.edu/.bin/fbidq.html?ap), [Iswi](http://flybase.bio.indiana.edu/.bin/fbidq.html?Iswi), [Dg](http://flybase.bio.indiana.edu/.bin/fbidq.html?Dg), [Trim9](http://flybase.bio.indiana.edu/.bin/fbidq.html?Trim9), [neo](http://flybase.bio.indiana.edu/.bin/fbidq.html?neo), [robo3](http://flybase.bio.indiana.edu/.bin/fbidq.html?robo3), [os](http://flybase.bio.indiana.edu/.bin/fbidq.html?os), [tok](http://flybase.bio.indiana.edu/.bin/fbidq.html?tok), [Ptp99A](http://flybase.bio.indiana.edu/.bin/fbidq.html?Ptp99A), [lola](http://flybase.bio.indiana.edu/.bin/fbidq.html?lola), [unc-104](http://flybase.bio.indiana.edu/.bin/fbidq.html?unc-104), [chif](http://flybase.bio.indiana.edu/.bin/fbidq.html?chif), [NetB](http://flybase.bio.indiana.edu/.bin/fbidq.html?NetB), [p130CAS](http://flybase.bio.indiana.edu/.bin/fbidq.html?p130CAS), [Tango10](http://flybase.bio.indiana.edu/.bin/fbidq.html?Tango10), [Nrk](http://flybase.bio.indiana.edu/.bin/fbidq.html?Nrk), [CG4328](http://flybase.bio.indiana.edu/.bin/fbidq.html?CG4328), [Galpha49B](http://flybase.bio.indiana.edu/.bin/fbidq.html?Galpha49B), [Lar](http://flybase.bio.indiana.edu/.bin/fbidq.html?Lar), [Taf4](http://flybase.bio.indiana.edu/.bin/fbidq.html?Taf4), [not](http://flybase.bio.indiana.edu/.bin/fbidq.html?not), [rut](http://flybase.bio.indiana.edu/.bin/fbidq.html?rut), [nvy](http://flybase.bio.indiana.edu/.bin/fbidq.html?nvy), [Nrg](http://flybase.bio.indiana.edu/.bin/fbidq.html?Nrg), [gogo](http://flybase.bio.indiana.edu/.bin/fbidq.html?gogo), [jeb](http://flybase.bio.indiana.edu/.bin/fbidq.html?jeb), [Actbeta](http://flybase.bio.indiana.edu/.bin/fbidq.html?Actbeta), [Sema-1a](http://flybase.bio.indiana.edu/.bin/fbidq.html?Sema-1a), [beat-Ib](http://flybase.bio.indiana.edu/.bin/fbidq.html?beat-Ib), [Traf4](http://flybase.bio.indiana.edu/.bin/fbidq.html?Traf4), [otk](http://flybase.bio.indiana.edu/.bin/fbidq.html?otk), [CG34400](http://flybase.bio.indiana.edu/.bin/fbidq.html?CG34400),[Ulp1](http://flybase.bio.indiana.edu/.bin/fbidq.html?Ulp1), [ena](http://flybase.bio.indiana.edu/.bin/fbidq.html?ena), [dsh](http://flybase.bio.indiana.edu/.bin/fbidq.html?dsh), [Fas2](http://flybase.bio.indiana.edu/.bin/fbidq.html?Fas2), [Nf-YC](http://flybase.bio.indiana.edu/.bin/fbidq.html?Nf-YC), [fz](http://flybase.bio.indiana.edu/.bin/fbidq.html?fz), [robo](http://flybase.bio.indiana.edu/.bin/fbidq.html?robo), [lea](http://flybase.bio.indiana.edu/.bin/fbidq.html?lea), [bsk](http://flybase.bio.indiana.edu/.bin/fbidq.html?bsk), [brm](http://flybase.bio.indiana.edu/.bin/fbidq.html?brm), [beat-Ia](http://flybase.bio.indiana.edu/.bin/fbidq.html?beat-Ia), [kat-60L1](http://flybase.bio.indiana.edu/.bin/fbidq.html?kat-60L1), [ft](http://flybase.bio.indiana.edu/.bin/fbidq.html?ft), [RhoGAPp190](http://flybase.bio.indiana.edu/.bin/fbidq.html?RhoGAPp190), [plexB](http://flybase.bio.indiana.edu/.bin/fbidq.html?plexB), [Klp64D](http://flybase.bio.indiana.edu/.bin/fbidq.html?Klp64D), [grn](http://flybase.bio.indiana.edu/.bin/fbidq.html?grn), [DAAM](http://flybase.bio.indiana.edu/.bin/fbidq.html?DAAM), [mew](http://flybase.bio.indiana.edu/.bin/fbidq.html?mew), [Lis-1](http://flybase.bio.indiana.edu/.bin/fbidq.html?Lis-1), [ems](http://flybase.bio.indiana.edu/.bin/fbidq.html?ems), [pros](http://flybase.bio.indiana.edu/.bin/fbidq.html?pros), [tup](http://flybase.bio.indiana.edu/.bin/fbidq.html?tup), [trio](http://flybase.bio.indiana.edu/.bin/fbidq.html?trio), [daw](http://flybase.bio.indiana.edu/.bin/fbidq.html?daw), [Wnt4](http://flybase.bio.indiana.edu/.bin/fbidq.html?Wnt4), [rictor](http://flybase.bio.indiana.edu/.bin/fbidq.html?rictor), [Apc](http://flybase.bio.indiana.edu/.bin/fbidq.html?Apc), [sim](http://flybase.bio.indiana.edu/.bin/fbidq.html?sim), [d4](http://flybase.bio.indiana.edu/.bin/fbidq.html?d4), [gro](http://flybase.bio.indiana.edu/.bin/fbidq.html?gro), [pigs](http://flybase.bio.indiana.edu/.bin/fbidq.html?pigs), [nerfin-1](http://flybase.bio.indiana.edu/.bin/fbidq.html?nerfin-1), [bchs](http://flybase.bio.indiana.edu/.bin/fbidq.html?bchs), [wnd](http://flybase.bio.indiana.edu/.bin/fbidq.html?wnd), [trx](http://flybase.bio.indiana.edu/.bin/fbidq.html?trx), [Rpd3](http://flybase.bio.indiana.edu/.bin/fbidq.html?Rpd3), [dsx](http://flybase.bio.indiana.edu/.bin/fbidq.html?dsx), [chinmo](http://flybase.bio.indiana.edu/.bin/fbidq.html?chinmo), [unc-5](http://flybase.bio.indiana.edu/.bin/fbidq.html?unc-5),[CG10107](http://flybase.bio.indiana.edu/.bin/fbidq.html?CG10107), [sqz](http://flybase.bio.indiana.edu/.bin/fbidq.html?sqz), [fend](http://flybase.bio.indiana.edu/.bin/fbidq.html?fend), [pygo](http://flybase.bio.indiana.edu/.bin/fbidq.html?pygo), [HLHm7](http://flybase.bio.indiana.edu/.bin/fbidq.html?HLHm7), [wor](http://flybase.bio.indiana.edu/.bin/fbidq.html?wor), [vvl](http://flybase.bio.indiana.edu/.bin/fbidq.html?vvl), [Pten](http://flybase.bio.indiana.edu/.bin/fbidq.html?Pten), [Ptp69D](http://flybase.bio.indiana.edu/.bin/fbidq.html?Ptp69D), [sgg](http://flybase.bio.indiana.edu/.bin/fbidq.html?sgg), [gcm2](http://flybase.bio.indiana.edu/.bin/fbidq.html?gcm2), [hts](http://flybase.bio.indiana.edu/.bin/fbidq.html?hts), [mp](http://flybase.bio.indiana.edu/.bin/fbidq.html?mp), [gcm](http://flybase.bio.indiana.edu/.bin/fbidq.html?gcm), [seq](http://flybase.bio.indiana.edu/.bin/fbidq.html?seq), [ds](http://flybase.bio.indiana.edu/.bin/fbidq.html?ds), [Alk](http://flybase.bio.indiana.edu/.bin/fbidq.html?Alk), [Sin3A](http://flybase.bio.indiana.edu/.bin/fbidq.html?Sin3A), [jumu](http://flybase.bio.indiana.edu/.bin/fbidq.html?jumu), [Smox](http://flybase.bio.indiana.edu/.bin/fbidq.html?Smox), [beat-Ic](http://flybase.bio.indiana.edu/.bin/fbidq.html?beat-Ic), [fd59A](http://flybase.bio.indiana.edu/.bin/fbidq.html?fd59A), [run](http://flybase.bio.indiana.edu/.bin/fbidq.html?run), [brat](http://flybase.bio.indiana.edu/.bin/fbidq.html?brat), [ko](http://flybase.bio.indiana.edu/.bin/fbidq.html?ko), [stan](http://flybase.bio.indiana.edu/.bin/fbidq.html?stan), [CG12424](http://flybase.bio.indiana.edu/.bin/fbidq.html?CG12424), [tutl](http://flybase.bio.indiana.edu/.bin/fbidq.html?tutl), [futsch](http://flybase.bio.indiana.edu/.bin/fbidq.html?futsch), [ph-p](http://flybase.bio.indiana.edu/.bin/fbidq.html?ph-p), [Ptx1](http://flybase.bio.indiana.edu/.bin/fbidq.html?Ptx1), [gukh](http://flybase.bio.indiana.edu/.bin/fbidq.html?gukh), [chm](http://flybase.bio.indiana.edu/.bin/fbidq.html?chm), [sna](http://flybase.bio.indiana.edu/.bin/fbidq.html?sna), [Wnt5](http://flybase.bio.indiana.edu/.bin/fbidq.html?Wnt5), [beat-IIa](http://flybase.bio.indiana.edu/.bin/fbidq.html?beat-IIa), [sm](http://flybase.bio.indiana.edu/.bin/fbidq.html?sm), [dac](http://flybase.bio.indiana.edu/.bin/fbidq.html?dac), [Cdk5](http://flybase.bio.indiana.edu/.bin/fbidq.html?Cdk5), [sif](http://flybase.bio.indiana.edu/.bin/fbidq.html?sif),[dom](http://flybase.bio.indiana.edu/.bin/fbidq.html?dom), [ssh](http://flybase.bio.indiana.edu/.bin/fbidq.html?ssh), [baz](http://flybase.bio.indiana.edu/.bin/fbidq.html?baz), [mle](http://flybase.bio.indiana.edu/.bin/fbidq.html?mle), [Hr51](http://flybase.bio.indiana.edu/.bin/fbidq.html?Hr51), [rst](http://flybase.bio.indiana.edu/.bin/fbidq.html?rst), [grh](http://flybase.bio.indiana.edu/.bin/fbidq.html?grh), [comm](http://flybase.bio.indiana.edu/.bin/fbidq.html?comm), [tyn](http://flybase.bio.indiana.edu/.bin/fbidq.html?tyn), [Cdk5alpha](http://flybase.bio.indiana.edu/.bin/fbidq.html?Cdk5alpha), [Lim3](http://flybase.bio.indiana.edu/.bin/fbidq.html?Lim3), [CadN](http://flybase.bio.indiana.edu/.bin/fbidq.html?CadN), [E(Pc)](http://flybase.bio.indiana.edu/.bin/fbidq.html?E(Pc)), [retn](http://flybase.bio.indiana.edu/.bin/fbidq.html?retn), [E(bx)](http://flybase.bio.indiana.edu/.bin/fbidq.html?E(bx)), [en](http://flybase.bio.indiana.edu/.bin/fbidq.html?en), [trn](http://flybase.bio.indiana.edu/.bin/fbidq.html?trn), [CG33960](http://flybase.bio.indiana.edu/.bin/fbidq.html?CG33960), [Ct](http://flybase.bio.indiana.edu/.bin/fbidq.html?Ct) |
| [response to stimulus](http://amigo.geneontology.org/cgi-bin/amigo/go.cgi?view=details&query=GO:0050896) | 460 of 1594 genes, 28.9% | 1744 of 7634 genes, 22.8% | 4.37e-11 | 0.00% | 0.00 | [fz2](http://flybase.bio.indiana.edu/.bin/fbidq.html?fz2), [CG14375](http://flybase.bio.indiana.edu/.bin/fbidq.html?CG14375), [Ocho](http://flybase.bio.indiana.edu/.bin/fbidq.html?Ocho), [Sh](http://flybase.bio.indiana.edu/.bin/fbidq.html?Sh), [numb](http://flybase.bio.indiana.edu/.bin/fbidq.html?numb), [dock](http://flybase.bio.indiana.edu/.bin/fbidq.html?dock), [mus101](http://flybase.bio.indiana.edu/.bin/fbidq.html?mus101), [CG17734](http://flybase.bio.indiana.edu/.bin/fbidq.html?CG17734), [Sxl](http://flybase.bio.indiana.edu/.bin/fbidq.html?Sxl), [CG8500](http://flybase.bio.indiana.edu/.bin/fbidq.html?CG8500), [ap](http://flybase.bio.indiana.edu/.bin/fbidq.html?ap), [cenG1A](http://flybase.bio.indiana.edu/.bin/fbidq.html?cenG1A), [kkv](http://flybase.bio.indiana.edu/.bin/fbidq.html?kkv), [tlk](http://flybase.bio.indiana.edu/.bin/fbidq.html?tlk), [Nf1](http://flybase.bio.indiana.edu/.bin/fbidq.html?Nf1), [malpha](http://flybase.bio.indiana.edu/.bin/fbidq.html?malpha), [AR-2](http://flybase.bio.indiana.edu/.bin/fbidq.html?AR-2), [sol](http://flybase.bio.indiana.edu/.bin/fbidq.html?sol), [os](http://flybase.bio.indiana.edu/.bin/fbidq.html?os), [Topors](http://flybase.bio.indiana.edu/.bin/fbidq.html?Topors), [Axn](http://flybase.bio.indiana.edu/.bin/fbidq.html?Axn), [tok](http://flybase.bio.indiana.edu/.bin/fbidq.html?tok), [nonA](http://flybase.bio.indiana.edu/.bin/fbidq.html?nonA), [Ptp99A](http://flybase.bio.indiana.edu/.bin/fbidq.html?Ptp99A), [Traf6](http://flybase.bio.indiana.edu/.bin/fbidq.html?Traf6), [Leucokinin](http://flybase.bio.indiana.edu/.bin/fbidq.html?Leucokinin), [NetB](http://flybase.bio.indiana.edu/.bin/fbidq.html?NetB), [Gs1l](http://flybase.bio.indiana.edu/.bin/fbidq.html?Gs1l), [rdgC](http://flybase.bio.indiana.edu/.bin/fbidq.html?rdgC), [CG4476](http://flybase.bio.indiana.edu/.bin/fbidq.html?CG4476), [Galpha49B](http://flybase.bio.indiana.edu/.bin/fbidq.html?Galpha49B), [Snoo](http://flybase.bio.indiana.edu/.bin/fbidq.html?Snoo), [Lar](http://flybase.bio.indiana.edu/.bin/fbidq.html?Lar), [scrib](http://flybase.bio.indiana.edu/.bin/fbidq.html?scrib), [not](http://flybase.bio.indiana.edu/.bin/fbidq.html?not), [rut](http://flybase.bio.indiana.edu/.bin/fbidq.html?rut),[nvy](http://flybase.bio.indiana.edu/.bin/fbidq.html?nvy), [CG7650](http://flybase.bio.indiana.edu/.bin/fbidq.html?CG7650), [RhoGAP100F](http://flybase.bio.indiana.edu/.bin/fbidq.html?RhoGAP100F), [uif](http://flybase.bio.indiana.edu/.bin/fbidq.html?uif), [nab](http://flybase.bio.indiana.edu/.bin/fbidq.html?nab), [Ddc](http://flybase.bio.indiana.edu/.bin/fbidq.html?Ddc), [amon](http://flybase.bio.indiana.edu/.bin/fbidq.html?amon), [Mmp2](http://flybase.bio.indiana.edu/.bin/fbidq.html?Mmp2), [HLHmdelta](http://flybase.bio.indiana.edu/.bin/fbidq.html?HLHmdelta), [Nplp1](http://flybase.bio.indiana.edu/.bin/fbidq.html?Nplp1), [Nipped-A](http://flybase.bio.indiana.edu/.bin/fbidq.html?Nipped-A), [DAT](http://flybase.bio.indiana.edu/.bin/fbidq.html?DAT), [CG32206](http://flybase.bio.indiana.edu/.bin/fbidq.html?CG32206), [Vha68-1](http://flybase.bio.indiana.edu/.bin/fbidq.html?Vha68-1), [7B2](http://flybase.bio.indiana.edu/.bin/fbidq.html?7B2), [alph](http://flybase.bio.indiana.edu/.bin/fbidq.html?alph), [Traf4](http://flybase.bio.indiana.edu/.bin/fbidq.html?Traf4), [TBPH](http://flybase.bio.indiana.edu/.bin/fbidq.html?TBPH), [Spf45](http://flybase.bio.indiana.edu/.bin/fbidq.html?Spf45), [Fer2](http://flybase.bio.indiana.edu/.bin/fbidq.html?Fer2), [CG34400](http://flybase.bio.indiana.edu/.bin/fbidq.html?CG34400), [Ulp1](http://flybase.bio.indiana.edu/.bin/fbidq.html?Ulp1), [ena](http://flybase.bio.indiana.edu/.bin/fbidq.html?ena), [mGluRA](http://flybase.bio.indiana.edu/.bin/fbidq.html?mGluRA), [dsh](http://flybase.bio.indiana.edu/.bin/fbidq.html?dsh), [lid](http://flybase.bio.indiana.edu/.bin/fbidq.html?lid), [pzg](http://flybase.bio.indiana.edu/.bin/fbidq.html?pzg), [Nf-YC](http://flybase.bio.indiana.edu/.bin/fbidq.html?Nf-YC), [14-3-3zeta](http://flybase.bio.indiana.edu/.bin/fbidq.html?14-3-3zeta), [lea](http://flybase.bio.indiana.edu/.bin/fbidq.html?lea), [Pli](http://flybase.bio.indiana.edu/.bin/fbidq.html?Pli), [shakB](http://flybase.bio.indiana.edu/.bin/fbidq.html?shakB),[CG31140](http://flybase.bio.indiana.edu/.bin/fbidq.html?CG31140), [Hey](http://flybase.bio.indiana.edu/.bin/fbidq.html?Hey), [plexB](http://flybase.bio.indiana.edu/.bin/fbidq.html?plexB), [AlstR](http://flybase.bio.indiana.edu/.bin/fbidq.html?AlstR), [PP2A-B](http://flybase.bio.indiana.edu/.bin/fbidq.html?PP2A-B), [Pxn](http://flybase.bio.indiana.edu/.bin/fbidq.html?Pxn), [loqs](http://flybase.bio.indiana.edu/.bin/fbidq.html?loqs), [qvr](http://flybase.bio.indiana.edu/.bin/fbidq.html?qvr), [Klp64D](http://flybase.bio.indiana.edu/.bin/fbidq.html?Klp64D), [jet](http://flybase.bio.indiana.edu/.bin/fbidq.html?jet), [IM10](http://flybase.bio.indiana.edu/.bin/fbidq.html?IM10), [neur](http://flybase.bio.indiana.edu/.bin/fbidq.html?neur), [CycE](http://flybase.bio.indiana.edu/.bin/fbidq.html?CycE), [rho-5](http://flybase.bio.indiana.edu/.bin/fbidq.html?rho-5), [nmo](http://flybase.bio.indiana.edu/.bin/fbidq.html?nmo), [Tusp](http://flybase.bio.indiana.edu/.bin/fbidq.html?Tusp), [5-HT1A](http://flybase.bio.indiana.edu/.bin/fbidq.html?5-HT1A), [CG32149](http://flybase.bio.indiana.edu/.bin/fbidq.html?CG32149), [Vdup1](http://flybase.bio.indiana.edu/.bin/fbidq.html?Vdup1), [Gug](http://flybase.bio.indiana.edu/.bin/fbidq.html?Gug), [Actn](http://flybase.bio.indiana.edu/.bin/fbidq.html?Actn), [ed](http://flybase.bio.indiana.edu/.bin/fbidq.html?ed), [orb2](http://flybase.bio.indiana.edu/.bin/fbidq.html?orb2), [daw](http://flybase.bio.indiana.edu/.bin/fbidq.html?daw), [CG5036](http://flybase.bio.indiana.edu/.bin/fbidq.html?CG5036), [5-HT1B](http://flybase.bio.indiana.edu/.bin/fbidq.html?5-HT1B), [rogdi](http://flybase.bio.indiana.edu/.bin/fbidq.html?rogdi), [Apc](http://flybase.bio.indiana.edu/.bin/fbidq.html?Apc), [sim](http://flybase.bio.indiana.edu/.bin/fbidq.html?sim), [CG8795](http://flybase.bio.indiana.edu/.bin/fbidq.html?CG8795), [VGAT](http://flybase.bio.indiana.edu/.bin/fbidq.html?VGAT), [tau](http://flybase.bio.indiana.edu/.bin/fbidq.html?tau), [gro](http://flybase.bio.indiana.edu/.bin/fbidq.html?gro), [RN-tre](http://flybase.bio.indiana.edu/.bin/fbidq.html?RN-tre), [scyl](http://flybase.bio.indiana.edu/.bin/fbidq.html?scyl), [bin3](http://flybase.bio.indiana.edu/.bin/fbidq.html?bin3), [wnd](http://flybase.bio.indiana.edu/.bin/fbidq.html?wnd),[trx](http://flybase.bio.indiana.edu/.bin/fbidq.html?trx), [Rpd3](http://flybase.bio.indiana.edu/.bin/fbidq.html?Rpd3), [Btk29A](http://flybase.bio.indiana.edu/.bin/fbidq.html?Btk29A), [SMC2](http://flybase.bio.indiana.edu/.bin/fbidq.html?SMC2), [PQBP-1](http://flybase.bio.indiana.edu/.bin/fbidq.html?PQBP-1), [MED25](http://flybase.bio.indiana.edu/.bin/fbidq.html?MED25), [ETH](http://flybase.bio.indiana.edu/.bin/fbidq.html?ETH), [tyf](http://flybase.bio.indiana.edu/.bin/fbidq.html?tyf), [unc-5](http://flybase.bio.indiana.edu/.bin/fbidq.html?unc-5), [cdi](http://flybase.bio.indiana.edu/.bin/fbidq.html?cdi), [CG10107](http://flybase.bio.indiana.edu/.bin/fbidq.html?CG10107), [NPFR1](http://flybase.bio.indiana.edu/.bin/fbidq.html?NPFR1), [aret](http://flybase.bio.indiana.edu/.bin/fbidq.html?aret), [CG4290](http://flybase.bio.indiana.edu/.bin/fbidq.html?CG4290), [pygo](http://flybase.bio.indiana.edu/.bin/fbidq.html?pygo), [HLHm7](http://flybase.bio.indiana.edu/.bin/fbidq.html?HLHm7), [HLHmgamma](http://flybase.bio.indiana.edu/.bin/fbidq.html?HLHmgamma), [CG15556](http://flybase.bio.indiana.edu/.bin/fbidq.html?CG15556), [tow](http://flybase.bio.indiana.edu/.bin/fbidq.html?tow), [Ror](http://flybase.bio.indiana.edu/.bin/fbidq.html?Ror), [CG15609](http://flybase.bio.indiana.edu/.bin/fbidq.html?CG15609), [CG14509](http://flybase.bio.indiana.edu/.bin/fbidq.html?CG14509), [Atg1](http://flybase.bio.indiana.edu/.bin/fbidq.html?Atg1), [Pten](http://flybase.bio.indiana.edu/.bin/fbidq.html?Pten), [unc-13](http://flybase.bio.indiana.edu/.bin/fbidq.html?unc-13), [npf](http://flybase.bio.indiana.edu/.bin/fbidq.html?npf), [Ptp69D](http://flybase.bio.indiana.edu/.bin/fbidq.html?Ptp69D), [hts](http://flybase.bio.indiana.edu/.bin/fbidq.html?hts), [dpr9](http://flybase.bio.indiana.edu/.bin/fbidq.html?dpr9), [seq](http://flybase.bio.indiana.edu/.bin/fbidq.html?seq), [jbug](http://flybase.bio.indiana.edu/.bin/fbidq.html?jbug), [oa2](http://flybase.bio.indiana.edu/.bin/fbidq.html?oa2), [elk](http://flybase.bio.indiana.edu/.bin/fbidq.html?elk), [tay](http://flybase.bio.indiana.edu/.bin/fbidq.html?tay),[l(3)L1231](http://flybase.bio.indiana.edu/.bin/fbidq.html?l(3)L1231), [phl](http://flybase.bio.indiana.edu/.bin/fbidq.html?phl), [Rab26](http://flybase.bio.indiana.edu/.bin/fbidq.html?Rab26), [Cbl](http://flybase.bio.indiana.edu/.bin/fbidq.html?Cbl), [Pde6](http://flybase.bio.indiana.edu/.bin/fbidq.html?Pde6), [sNPF](http://flybase.bio.indiana.edu/.bin/fbidq.html?sNPF), [hang](http://flybase.bio.indiana.edu/.bin/fbidq.html?hang), [pog](http://flybase.bio.indiana.edu/.bin/fbidq.html?pog), [plx](http://flybase.bio.indiana.edu/.bin/fbidq.html?plx), [Pdp1](http://flybase.bio.indiana.edu/.bin/fbidq.html?Pdp1), [CASK](http://flybase.bio.indiana.edu/.bin/fbidq.html?CASK), [CG10251](http://flybase.bio.indiana.edu/.bin/fbidq.html?CG10251), [CG9098](http://flybase.bio.indiana.edu/.bin/fbidq.html?CG9098), [IFa](http://flybase.bio.indiana.edu/.bin/fbidq.html?IFa), [CG8108](http://flybase.bio.indiana.edu/.bin/fbidq.html?CG8108), [CG32758](http://flybase.bio.indiana.edu/.bin/fbidq.html?CG32758), [mtt](http://flybase.bio.indiana.edu/.bin/fbidq.html?mtt), [Alk](http://flybase.bio.indiana.edu/.bin/fbidq.html?Alk), [Pde1c](http://flybase.bio.indiana.edu/.bin/fbidq.html?Pde1c), [disp](http://flybase.bio.indiana.edu/.bin/fbidq.html?disp), [casp](http://flybase.bio.indiana.edu/.bin/fbidq.html?casp), [CG4022](http://flybase.bio.indiana.edu/.bin/fbidq.html?CG4022), [Smox](http://flybase.bio.indiana.edu/.bin/fbidq.html?Smox), [gfA](http://flybase.bio.indiana.edu/.bin/fbidq.html?gfA), [Plc21C](http://flybase.bio.indiana.edu/.bin/fbidq.html?Plc21C), [fd59A](http://flybase.bio.indiana.edu/.bin/fbidq.html?fd59A), [beat-Ic](http://flybase.bio.indiana.edu/.bin/fbidq.html?beat-Ic), [srpk79D](http://flybase.bio.indiana.edu/.bin/fbidq.html?srpk79D), [brat](http://flybase.bio.indiana.edu/.bin/fbidq.html?brat), [GRHRII](http://flybase.bio.indiana.edu/.bin/fbidq.html?GRHRII), [Rdl](http://flybase.bio.indiana.edu/.bin/fbidq.html?Rdl), [CG10362](http://flybase.bio.indiana.edu/.bin/fbidq.html?CG10362), [GABA-B-R2](http://flybase.bio.indiana.edu/.bin/fbidq.html?GABA-B-R2),[Ast-C](http://flybase.bio.indiana.edu/.bin/fbidq.html?Ast-C), [Gbeta5](http://flybase.bio.indiana.edu/.bin/fbidq.html?Gbeta5), [futsch](http://flybase.bio.indiana.edu/.bin/fbidq.html?futsch), [gukh](http://flybase.bio.indiana.edu/.bin/fbidq.html?gukh), [Rpt3R](http://flybase.bio.indiana.edu/.bin/fbidq.html?Rpt3R), [pigeon](http://flybase.bio.indiana.edu/.bin/fbidq.html?pigeon), [hug](http://flybase.bio.indiana.edu/.bin/fbidq.html?hug), [beat-IIa](http://flybase.bio.indiana.edu/.bin/fbidq.html?beat-IIa), [Gpo-1](http://flybase.bio.indiana.edu/.bin/fbidq.html?Gpo-1), [para](http://flybase.bio.indiana.edu/.bin/fbidq.html?para), [sm](http://flybase.bio.indiana.edu/.bin/fbidq.html?sm), [Sur-8](http://flybase.bio.indiana.edu/.bin/fbidq.html?Sur-8), [Rgl](http://flybase.bio.indiana.edu/.bin/fbidq.html?Rgl), [eas](http://flybase.bio.indiana.edu/.bin/fbidq.html?eas), [dac](http://flybase.bio.indiana.edu/.bin/fbidq.html?dac), [e(y)3](http://flybase.bio.indiana.edu/.bin/fbidq.html?e(y)3), [Nrx-1](http://flybase.bio.indiana.edu/.bin/fbidq.html?Nrx-1), [CRMP](http://flybase.bio.indiana.edu/.bin/fbidq.html?CRMP), [Cdk5](http://flybase.bio.indiana.edu/.bin/fbidq.html?Cdk5), [oc](http://flybase.bio.indiana.edu/.bin/fbidq.html?oc), [mav](http://flybase.bio.indiana.edu/.bin/fbidq.html?mav), [Ilp7](http://flybase.bio.indiana.edu/.bin/fbidq.html?Ilp7), [ey](http://flybase.bio.indiana.edu/.bin/fbidq.html?ey), [Mip](http://flybase.bio.indiana.edu/.bin/fbidq.html?Mip), [baz](http://flybase.bio.indiana.edu/.bin/fbidq.html?baz), [CG33275](http://flybase.bio.indiana.edu/.bin/fbidq.html?CG33275), [Oamb](http://flybase.bio.indiana.edu/.bin/fbidq.html?Oamb), [CG34381](http://flybase.bio.indiana.edu/.bin/fbidq.html?CG34381), [grh](http://flybase.bio.indiana.edu/.bin/fbidq.html?grh), [comm](http://flybase.bio.indiana.edu/.bin/fbidq.html?comm), [Cdk5alpha](http://flybase.bio.indiana.edu/.bin/fbidq.html?Cdk5alpha), [CG40351](http://flybase.bio.indiana.edu/.bin/fbidq.html?CG40351), [Vmat](http://flybase.bio.indiana.edu/.bin/fbidq.html?Vmat), [qtc](http://flybase.bio.indiana.edu/.bin/fbidq.html?qtc), [Lim3](http://flybase.bio.indiana.edu/.bin/fbidq.html?Lim3), [crol](http://flybase.bio.indiana.edu/.bin/fbidq.html?crol), [sd](http://flybase.bio.indiana.edu/.bin/fbidq.html?sd),[Aplip1](http://flybase.bio.indiana.edu/.bin/fbidq.html?Aplip1), [retn](http://flybase.bio.indiana.edu/.bin/fbidq.html?retn), [Ac3](http://flybase.bio.indiana.edu/.bin/fbidq.html?Ac3), [rl](http://flybase.bio.indiana.edu/.bin/fbidq.html?rl), [Syt1](http://flybase.bio.indiana.edu/.bin/fbidq.html?Syt1), [CG17760](http://flybase.bio.indiana.edu/.bin/fbidq.html?CG17760), [Nup153](http://flybase.bio.indiana.edu/.bin/fbidq.html?Nup153), [CG32683](http://flybase.bio.indiana.edu/.bin/fbidq.html?CG32683), [CG11347](http://flybase.bio.indiana.edu/.bin/fbidq.html?CG11347), [Ggamma30A](http://flybase.bio.indiana.edu/.bin/fbidq.html?Ggamma30A), [wls](http://flybase.bio.indiana.edu/.bin/fbidq.html?wls), [CG33960](http://flybase.bio.indiana.edu/.bin/fbidq.html?CG33960), [hiw](http://flybase.bio.indiana.edu/.bin/fbidq.html?hiw), [dnr1](http://flybase.bio.indiana.edu/.bin/fbidq.html?dnr1), [Atf-2](http://flybase.bio.indiana.edu/.bin/fbidq.html?Atf-2), [acj6](http://flybase.bio.indiana.edu/.bin/fbidq.html?acj6), [CG16833](http://flybase.bio.indiana.edu/.bin/fbidq.html?CG16833), [skd](http://flybase.bio.indiana.edu/.bin/fbidq.html?skd), [jing](http://flybase.bio.indiana.edu/.bin/fbidq.html?jing), [Cbp80](http://flybase.bio.indiana.edu/.bin/fbidq.html?Cbp80), [Ela](http://flybase.bio.indiana.edu/.bin/fbidq.html?Ela), [Camta](http://flybase.bio.indiana.edu/.bin/fbidq.html?Camta), [tll](http://flybase.bio.indiana.edu/.bin/fbidq.html?tll), [ato](http://flybase.bio.indiana.edu/.bin/fbidq.html?ato), [Spn](http://flybase.bio.indiana.edu/.bin/fbidq.html?Spn), [Hsp67Ba](http://flybase.bio.indiana.edu/.bin/fbidq.html?Hsp67Ba), [Fmrf](http://flybase.bio.indiana.edu/.bin/fbidq.html?Fmrf), [Iswi](http://flybase.bio.indiana.edu/.bin/fbidq.html?Iswi), [Spt6](http://flybase.bio.indiana.edu/.bin/fbidq.html?Spt6), [Dg](http://flybase.bio.indiana.edu/.bin/fbidq.html?Dg), [Trim9](http://flybase.bio.indiana.edu/.bin/fbidq.html?Trim9), [GABA-B-R3](http://flybase.bio.indiana.edu/.bin/fbidq.html?GABA-B-R3), [Pka-R1](http://flybase.bio.indiana.edu/.bin/fbidq.html?Pka-R1),[CG14478](http://flybase.bio.indiana.edu/.bin/fbidq.html?CG14478), [Evi5](http://flybase.bio.indiana.edu/.bin/fbidq.html?Evi5), [mago](http://flybase.bio.indiana.edu/.bin/fbidq.html?mago), [br](http://flybase.bio.indiana.edu/.bin/fbidq.html?br), [Gycalpha99B](http://flybase.bio.indiana.edu/.bin/fbidq.html?Gycalpha99B), [CG42533](http://flybase.bio.indiana.edu/.bin/fbidq.html?CG42533), [ksr](http://flybase.bio.indiana.edu/.bin/fbidq.html?ksr), [robo3](http://flybase.bio.indiana.edu/.bin/fbidq.html?robo3), [CG11376](http://flybase.bio.indiana.edu/.bin/fbidq.html?CG11376), [Gyc-89Da](http://flybase.bio.indiana.edu/.bin/fbidq.html?Gyc-89Da), [brp](http://flybase.bio.indiana.edu/.bin/fbidq.html?brp), [lola](http://flybase.bio.indiana.edu/.bin/fbidq.html?lola), [unc-104](http://flybase.bio.indiana.edu/.bin/fbidq.html?unc-104), [VAChT](http://flybase.bio.indiana.edu/.bin/fbidq.html?VAChT), [CG7627](http://flybase.bio.indiana.edu/.bin/fbidq.html?CG7627), [chif](http://flybase.bio.indiana.edu/.bin/fbidq.html?chif), [D2R](http://flybase.bio.indiana.edu/.bin/fbidq.html?D2R), [CG31158](http://flybase.bio.indiana.edu/.bin/fbidq.html?CG31158), [Appl](http://flybase.bio.indiana.edu/.bin/fbidq.html?Appl), [ewg](http://flybase.bio.indiana.edu/.bin/fbidq.html?ewg), [Nrk](http://flybase.bio.indiana.edu/.bin/fbidq.html?Nrk), [Cirl](http://flybase.bio.indiana.edu/.bin/fbidq.html?Cirl), [Ilp3](http://flybase.bio.indiana.edu/.bin/fbidq.html?Ilp3), [Su(var)3-3](http://flybase.bio.indiana.edu/.bin/fbidq.html?Su(var)3-3), [Bili](http://flybase.bio.indiana.edu/.bin/fbidq.html?Bili), [Nrg](http://flybase.bio.indiana.edu/.bin/fbidq.html?Nrg), [gogo](http://flybase.bio.indiana.edu/.bin/fbidq.html?gogo), [CG8745](http://flybase.bio.indiana.edu/.bin/fbidq.html?CG8745), [CG34384](http://flybase.bio.indiana.edu/.bin/fbidq.html?CG34384), [jeb](http://flybase.bio.indiana.edu/.bin/fbidq.html?jeb), [Dh44-R1](http://flybase.bio.indiana.edu/.bin/fbidq.html?Dh44-R1), [stet](http://flybase.bio.indiana.edu/.bin/fbidq.html?stet),[CG32447](http://flybase.bio.indiana.edu/.bin/fbidq.html?CG32447), [stau](http://flybase.bio.indiana.edu/.bin/fbidq.html?stau), [Sema-1a](http://flybase.bio.indiana.edu/.bin/fbidq.html?Sema-1a), [Eh](http://flybase.bio.indiana.edu/.bin/fbidq.html?Eh), [beat-Ib](http://flybase.bio.indiana.edu/.bin/fbidq.html?beat-Ib), [Hs3st-B](http://flybase.bio.indiana.edu/.bin/fbidq.html?Hs3st-B), [otk](http://flybase.bio.indiana.edu/.bin/fbidq.html?otk), [Sos](http://flybase.bio.indiana.edu/.bin/fbidq.html?Sos), [phyl](http://flybase.bio.indiana.edu/.bin/fbidq.html?phyl), [Fas2](http://flybase.bio.indiana.edu/.bin/fbidq.html?Fas2), [eya](http://flybase.bio.indiana.edu/.bin/fbidq.html?eya), [fz](http://flybase.bio.indiana.edu/.bin/fbidq.html?fz), [robo](http://flybase.bio.indiana.edu/.bin/fbidq.html?robo), [mAcR-60C](http://flybase.bio.indiana.edu/.bin/fbidq.html?mAcR-60C), [CG8557](http://flybase.bio.indiana.edu/.bin/fbidq.html?CG8557), [phr](http://flybase.bio.indiana.edu/.bin/fbidq.html?phr), [bsk](http://flybase.bio.indiana.edu/.bin/fbidq.html?bsk), [brm](http://flybase.bio.indiana.edu/.bin/fbidq.html?brm), [beat-Ia](http://flybase.bio.indiana.edu/.bin/fbidq.html?beat-Ia), [ft](http://flybase.bio.indiana.edu/.bin/fbidq.html?ft), [Crz](http://flybase.bio.indiana.edu/.bin/fbidq.html?Crz), [nAcRalpha-30D](http://flybase.bio.indiana.edu/.bin/fbidq.html?nAcRalpha-30D), [CG13830](http://flybase.bio.indiana.edu/.bin/fbidq.html?CG13830), [sno](http://flybase.bio.indiana.edu/.bin/fbidq.html?sno), [RhoGAPp190](http://flybase.bio.indiana.edu/.bin/fbidq.html?RhoGAPp190), [l(2)k16918](http://flybase.bio.indiana.edu/.bin/fbidq.html?l(2)k16918), [mam](http://flybase.bio.indiana.edu/.bin/fbidq.html?mam), [CG30158](http://flybase.bio.indiana.edu/.bin/fbidq.html?CG30158), [Takr86C](http://flybase.bio.indiana.edu/.bin/fbidq.html?Takr86C), [smi35A](http://flybase.bio.indiana.edu/.bin/fbidq.html?smi35A), [pan](http://flybase.bio.indiana.edu/.bin/fbidq.html?pan),[PKD](http://flybase.bio.indiana.edu/.bin/fbidq.html?PKD), [Dll](http://flybase.bio.indiana.edu/.bin/fbidq.html?Dll), [disco](http://flybase.bio.indiana.edu/.bin/fbidq.html?disco), [grn](http://flybase.bio.indiana.edu/.bin/fbidq.html?grn), [DopR](http://flybase.bio.indiana.edu/.bin/fbidq.html?DopR), [mew](http://flybase.bio.indiana.edu/.bin/fbidq.html?mew), [Lis-1](http://flybase.bio.indiana.edu/.bin/fbidq.html?Lis-1), [CG16896](http://flybase.bio.indiana.edu/.bin/fbidq.html?CG16896), [eys](http://flybase.bio.indiana.edu/.bin/fbidq.html?eys), [Bx](http://flybase.bio.indiana.edu/.bin/fbidq.html?Bx), [Mob2](http://flybase.bio.indiana.edu/.bin/fbidq.html?Mob2), [pros](http://flybase.bio.indiana.edu/.bin/fbidq.html?pros), [tup](http://flybase.bio.indiana.edu/.bin/fbidq.html?tup), [Rab3](http://flybase.bio.indiana.edu/.bin/fbidq.html?Rab3), [Gen](http://flybase.bio.indiana.edu/.bin/fbidq.html?Gen), [trio](http://flybase.bio.indiana.edu/.bin/fbidq.html?trio), [mthl8](http://flybase.bio.indiana.edu/.bin/fbidq.html?mthl8), [RabX4](http://flybase.bio.indiana.edu/.bin/fbidq.html?RabX4), [Lkr](http://flybase.bio.indiana.edu/.bin/fbidq.html?Lkr), [spel1](http://flybase.bio.indiana.edu/.bin/fbidq.html?spel1), [CG31665](http://flybase.bio.indiana.edu/.bin/fbidq.html?CG31665), [Ccap](http://flybase.bio.indiana.edu/.bin/fbidq.html?Ccap), [tipE](http://flybase.bio.indiana.edu/.bin/fbidq.html?tipE), [Gad1](http://flybase.bio.indiana.edu/.bin/fbidq.html?Gad1), [DJ-1alpha](http://flybase.bio.indiana.edu/.bin/fbidq.html?DJ-1alpha), [Wnt4](http://flybase.bio.indiana.edu/.bin/fbidq.html?Wnt4), [CG7918](http://flybase.bio.indiana.edu/.bin/fbidq.html?CG7918), [unc79](http://flybase.bio.indiana.edu/.bin/fbidq.html?unc79), [rictor](http://flybase.bio.indiana.edu/.bin/fbidq.html?rictor), [CaMKII](http://flybase.bio.indiana.edu/.bin/fbidq.html?CaMKII), [DopR2](http://flybase.bio.indiana.edu/.bin/fbidq.html?DopR2), [CG30372](http://flybase.bio.indiana.edu/.bin/fbidq.html?CG30372), [CG10188](http://flybase.bio.indiana.edu/.bin/fbidq.html?CG10188), [CG42629](http://flybase.bio.indiana.edu/.bin/fbidq.html?CG42629),[nerfin-1](http://flybase.bio.indiana.edu/.bin/fbidq.html?nerfin-1), [pigs](http://flybase.bio.indiana.edu/.bin/fbidq.html?pigs), [CG31760](http://flybase.bio.indiana.edu/.bin/fbidq.html?CG31760), [Klp54D](http://flybase.bio.indiana.edu/.bin/fbidq.html?Klp54D), [pr-set7](http://flybase.bio.indiana.edu/.bin/fbidq.html?pr-set7), [5-HT7](http://flybase.bio.indiana.edu/.bin/fbidq.html?5-HT7), [Fancd2](http://flybase.bio.indiana.edu/.bin/fbidq.html?Fancd2), [Lgr3](http://flybase.bio.indiana.edu/.bin/fbidq.html?Lgr3), [dsx](http://flybase.bio.indiana.edu/.bin/fbidq.html?dsx), [mei-9](http://flybase.bio.indiana.edu/.bin/fbidq.html?mei-9), [Dh](http://flybase.bio.indiana.edu/.bin/fbidq.html?Dh), [Ac13E](http://flybase.bio.indiana.edu/.bin/fbidq.html?Ac13E), [klu](http://flybase.bio.indiana.edu/.bin/fbidq.html?klu), [SPR](http://flybase.bio.indiana.edu/.bin/fbidq.html?SPR), [CG8155](http://flybase.bio.indiana.edu/.bin/fbidq.html?CG8155), [CG30467](http://flybase.bio.indiana.edu/.bin/fbidq.html?CG30467), [fend](http://flybase.bio.indiana.edu/.bin/fbidq.html?fend), [nkd](http://flybase.bio.indiana.edu/.bin/fbidq.html?nkd), [tld](http://flybase.bio.indiana.edu/.bin/fbidq.html?tld), [RhoGEF3](http://flybase.bio.indiana.edu/.bin/fbidq.html?RhoGEF3), [NaCP60E](http://flybase.bio.indiana.edu/.bin/fbidq.html?NaCP60E), [Dsk](http://flybase.bio.indiana.edu/.bin/fbidq.html?Dsk), [RhoGEF4](http://flybase.bio.indiana.edu/.bin/fbidq.html?RhoGEF4), [vvl](http://flybase.bio.indiana.edu/.bin/fbidq.html?vvl), [rg](http://flybase.bio.indiana.edu/.bin/fbidq.html?rg), [CG9121](http://flybase.bio.indiana.edu/.bin/fbidq.html?CG9121), [betaInt-nu](http://flybase.bio.indiana.edu/.bin/fbidq.html?betaInt-nu), [sgg](http://flybase.bio.indiana.edu/.bin/fbidq.html?sgg), [Brd](http://flybase.bio.indiana.edu/.bin/fbidq.html?Brd), [mnb](http://flybase.bio.indiana.edu/.bin/fbidq.html?mnb), [Nmdar1](http://flybase.bio.indiana.edu/.bin/fbidq.html?Nmdar1), [Eip93F](http://flybase.bio.indiana.edu/.bin/fbidq.html?Eip93F), [inaE](http://flybase.bio.indiana.edu/.bin/fbidq.html?inaE),[mp](http://flybase.bio.indiana.edu/.bin/fbidq.html?mp), [gcm](http://flybase.bio.indiana.edu/.bin/fbidq.html?gcm), [mus210](http://flybase.bio.indiana.edu/.bin/fbidq.html?mus210), [Hsp67Bc](http://flybase.bio.indiana.edu/.bin/fbidq.html?Hsp67Bc), [Smc5](http://flybase.bio.indiana.edu/.bin/fbidq.html?Smc5), [Pask](http://flybase.bio.indiana.edu/.bin/fbidq.html?Pask), [rab3-GEF](http://flybase.bio.indiana.edu/.bin/fbidq.html?rab3-GEF), [CG13253](http://flybase.bio.indiana.edu/.bin/fbidq.html?CG13253), [ovo](http://flybase.bio.indiana.edu/.bin/fbidq.html?ovo), [MESK2](http://flybase.bio.indiana.edu/.bin/fbidq.html?MESK2), [Dsp1](http://flybase.bio.indiana.edu/.bin/fbidq.html?Dsp1), [Poxn](http://flybase.bio.indiana.edu/.bin/fbidq.html?Poxn), [Dat](http://flybase.bio.indiana.edu/.bin/fbidq.html?Dat), [SIFR](http://flybase.bio.indiana.edu/.bin/fbidq.html?SIFR), [CG2061](http://flybase.bio.indiana.edu/.bin/fbidq.html?CG2061), [Ast](http://flybase.bio.indiana.edu/.bin/fbidq.html?Ast), [fred](http://flybase.bio.indiana.edu/.bin/fbidq.html?fred), [Drl-2](http://flybase.bio.indiana.edu/.bin/fbidq.html?Drl-2), [Tsp42Ej](http://flybase.bio.indiana.edu/.bin/fbidq.html?Tsp42Ej), [ush](http://flybase.bio.indiana.edu/.bin/fbidq.html?ush), [Fak56D](http://flybase.bio.indiana.edu/.bin/fbidq.html?Fak56D), [Tk](http://flybase.bio.indiana.edu/.bin/fbidq.html?Tk), [Ilp5](http://flybase.bio.indiana.edu/.bin/fbidq.html?Ilp5), [CG10336](http://flybase.bio.indiana.edu/.bin/fbidq.html?CG10336), [Gap1](http://flybase.bio.indiana.edu/.bin/fbidq.html?Gap1), [Toll-6](http://flybase.bio.indiana.edu/.bin/fbidq.html?Toll-6), [CG3227](http://flybase.bio.indiana.edu/.bin/fbidq.html?CG3227), [run](http://flybase.bio.indiana.edu/.bin/fbidq.html?run), [MESR4](http://flybase.bio.indiana.edu/.bin/fbidq.html?MESR4), [CG16778](http://flybase.bio.indiana.edu/.bin/fbidq.html?CG16778), [Takr99D](http://flybase.bio.indiana.edu/.bin/fbidq.html?Takr99D), [ko](http://flybase.bio.indiana.edu/.bin/fbidq.html?ko), [sNPF-R](http://flybase.bio.indiana.edu/.bin/fbidq.html?sNPF-R), [Eip71CD](http://flybase.bio.indiana.edu/.bin/fbidq.html?Eip71CD), [Hel89B](http://flybase.bio.indiana.edu/.bin/fbidq.html?Hel89B), [CG13692](http://flybase.bio.indiana.edu/.bin/fbidq.html?CG13692), [CG5337](http://flybase.bio.indiana.edu/.bin/fbidq.html?CG5337), [koko](http://flybase.bio.indiana.edu/.bin/fbidq.html?koko), [stan](http://flybase.bio.indiana.edu/.bin/fbidq.html?stan), [CG12424](http://flybase.bio.indiana.edu/.bin/fbidq.html?CG12424), [tutl](http://flybase.bio.indiana.edu/.bin/fbidq.html?tutl), [Ptth](http://flybase.bio.indiana.edu/.bin/fbidq.html?Ptth), [Rab2](http://flybase.bio.indiana.edu/.bin/fbidq.html?Rab2), [Ilp2](http://flybase.bio.indiana.edu/.bin/fbidq.html?Ilp2), [Fancl](http://flybase.bio.indiana.edu/.bin/fbidq.html?Fancl), [ph-p](http://flybase.bio.indiana.edu/.bin/fbidq.html?ph-p), [CG3967](http://flybase.bio.indiana.edu/.bin/fbidq.html?CG3967), [SPoCk](http://flybase.bio.indiana.edu/.bin/fbidq.html?SPoCk), [Wnt5](http://flybase.bio.indiana.edu/.bin/fbidq.html?Wnt5), [SF2](http://flybase.bio.indiana.edu/.bin/fbidq.html?SF2), [Taf1](http://flybase.bio.indiana.edu/.bin/fbidq.html?Taf1), [Ggamma1](http://flybase.bio.indiana.edu/.bin/fbidq.html?Ggamma1), [esg](http://flybase.bio.indiana.edu/.bin/fbidq.html?esg), [fus](http://flybase.bio.indiana.edu/.bin/fbidq.html?fus), [sif](http://flybase.bio.indiana.edu/.bin/fbidq.html?sif), [RSG7](http://flybase.bio.indiana.edu/.bin/fbidq.html?RSG7), [m4](http://flybase.bio.indiana.edu/.bin/fbidq.html?m4), [dom](http://flybase.bio.indiana.edu/.bin/fbidq.html?dom), [Tbh](http://flybase.bio.indiana.edu/.bin/fbidq.html?Tbh), [Dh31](http://flybase.bio.indiana.edu/.bin/fbidq.html?Dh31), [CG42541](http://flybase.bio.indiana.edu/.bin/fbidq.html?CG42541), [mle](http://flybase.bio.indiana.edu/.bin/fbidq.html?mle), [Hr51](http://flybase.bio.indiana.edu/.bin/fbidq.html?Hr51), [lap](http://flybase.bio.indiana.edu/.bin/fbidq.html?lap), [shi](http://flybase.bio.indiana.edu/.bin/fbidq.html?shi), [Spred](http://flybase.bio.indiana.edu/.bin/fbidq.html?Spred), [Keap1](http://flybase.bio.indiana.edu/.bin/fbidq.html?Keap1), [siz](http://flybase.bio.indiana.edu/.bin/fbidq.html?siz),[wg](http://flybase.bio.indiana.edu/.bin/fbidq.html?wg), [fkh](http://flybase.bio.indiana.edu/.bin/fbidq.html?fkh), [CadN](http://flybase.bio.indiana.edu/.bin/fbidq.html?CadN), [Rhp](http://flybase.bio.indiana.edu/.bin/fbidq.html?Rhp), [fne](http://flybase.bio.indiana.edu/.bin/fbidq.html?fne), [CG13995](http://flybase.bio.indiana.edu/.bin/fbidq.html?CG13995), [klg](http://flybase.bio.indiana.edu/.bin/fbidq.html?klg), [en](http://flybase.bio.indiana.edu/.bin/fbidq.html?en), [E(bx)](http://flybase.bio.indiana.edu/.bin/fbidq.html?E(bx)), [sfl](http://flybase.bio.indiana.edu/.bin/fbidq.html?sfl), [CG12728](http://flybase.bio.indiana.edu/.bin/fbidq.html?CG12728), [Sip1](http://flybase.bio.indiana.edu/.bin/fbidq.html?Sip1), [CG13229](http://flybase.bio.indiana.edu/.bin/fbidq.html?CG13229), [Pde11](http://flybase.bio.indiana.edu/.bin/fbidq.html?Pde11), [Dms](http://flybase.bio.indiana.edu/.bin/fbidq.html?Dms), [trn](http://flybase.bio.indiana.edu/.bin/fbidq.html?trn), [Utx](http://flybase.bio.indiana.edu/.bin/fbidq.html?Utx), [pburs](http://flybase.bio.indiana.edu/.bin/fbidq.html?pburs), [cos](http://flybase.bio.indiana.edu/.bin/fbidq.html?cos), [Ct](http://flybase.bio.indiana.edu/.bin/fbidq.html?Ct) |
| [cell projection morphogenesis](http://amigo.geneontology.org/cgi-bin/amigo/go.cgi?view=details&query=GO:0048858) | 153 of 1594 genes, 9.6% | 439 of 7634 genes, 5.8% | 5.21e-11 | 0.00% | 0.00 | [fz2](http://flybase.bio.indiana.edu/.bin/fbidq.html?fz2), [Sh](http://flybase.bio.indiana.edu/.bin/fbidq.html?Sh), [acj6](http://flybase.bio.indiana.edu/.bin/fbidq.html?acj6), [jing](http://flybase.bio.indiana.edu/.bin/fbidq.html?jing), [dock](http://flybase.bio.indiana.edu/.bin/fbidq.html?dock), [CG42256](http://flybase.bio.indiana.edu/.bin/fbidq.html?CG42256), [CG3703](http://flybase.bio.indiana.edu/.bin/fbidq.html?CG3703), [ato](http://flybase.bio.indiana.edu/.bin/fbidq.html?ato), [ap](http://flybase.bio.indiana.edu/.bin/fbidq.html?ap), [Iswi](http://flybase.bio.indiana.edu/.bin/fbidq.html?Iswi), [Dg](http://flybase.bio.indiana.edu/.bin/fbidq.html?Dg), [Trim9](http://flybase.bio.indiana.edu/.bin/fbidq.html?Trim9), [CG7154](http://flybase.bio.indiana.edu/.bin/fbidq.html?CG7154), [robo3](http://flybase.bio.indiana.edu/.bin/fbidq.html?robo3), [tok](http://flybase.bio.indiana.edu/.bin/fbidq.html?tok), [Ptp99A](http://flybase.bio.indiana.edu/.bin/fbidq.html?Ptp99A), [lola](http://flybase.bio.indiana.edu/.bin/fbidq.html?lola), [unc-104](http://flybase.bio.indiana.edu/.bin/fbidq.html?unc-104), [chif](http://flybase.bio.indiana.edu/.bin/fbidq.html?chif), [NetB](http://flybase.bio.indiana.edu/.bin/fbidq.html?NetB), [p130CAS](http://flybase.bio.indiana.edu/.bin/fbidq.html?p130CAS), [Tango10](http://flybase.bio.indiana.edu/.bin/fbidq.html?Tango10), [Oseg4](http://flybase.bio.indiana.edu/.bin/fbidq.html?Oseg4), [Appl](http://flybase.bio.indiana.edu/.bin/fbidq.html?Appl), [metro](http://flybase.bio.indiana.edu/.bin/fbidq.html?metro), [CG32137](http://flybase.bio.indiana.edu/.bin/fbidq.html?CG32137), [Nrk](http://flybase.bio.indiana.edu/.bin/fbidq.html?Nrk), [CG4328](http://flybase.bio.indiana.edu/.bin/fbidq.html?CG4328), [Galpha49B](http://flybase.bio.indiana.edu/.bin/fbidq.html?Galpha49B), [Lar](http://flybase.bio.indiana.edu/.bin/fbidq.html?Lar), [Taf4](http://flybase.bio.indiana.edu/.bin/fbidq.html?Taf4), [CG10249](http://flybase.bio.indiana.edu/.bin/fbidq.html?CG10249), [not](http://flybase.bio.indiana.edu/.bin/fbidq.html?not), [rut](http://flybase.bio.indiana.edu/.bin/fbidq.html?rut), [nvy](http://flybase.bio.indiana.edu/.bin/fbidq.html?nvy),[Nrg](http://flybase.bio.indiana.edu/.bin/fbidq.html?Nrg), [gogo](http://flybase.bio.indiana.edu/.bin/fbidq.html?gogo), [jeb](http://flybase.bio.indiana.edu/.bin/fbidq.html?jeb), [GluClalpha](http://flybase.bio.indiana.edu/.bin/fbidq.html?GluClalpha), [Actbeta](http://flybase.bio.indiana.edu/.bin/fbidq.html?Actbeta), [Sema-1a](http://flybase.bio.indiana.edu/.bin/fbidq.html?Sema-1a), [beat-Ib](http://flybase.bio.indiana.edu/.bin/fbidq.html?beat-Ib), [TBPH](http://flybase.bio.indiana.edu/.bin/fbidq.html?TBPH), [otk](http://flybase.bio.indiana.edu/.bin/fbidq.html?otk), [CG34400](http://flybase.bio.indiana.edu/.bin/fbidq.html?CG34400), [Ulp1](http://flybase.bio.indiana.edu/.bin/fbidq.html?Ulp1), [ena](http://flybase.bio.indiana.edu/.bin/fbidq.html?ena), [dsh](http://flybase.bio.indiana.edu/.bin/fbidq.html?dsh), [Fas2](http://flybase.bio.indiana.edu/.bin/fbidq.html?Fas2), [Nf-YC](http://flybase.bio.indiana.edu/.bin/fbidq.html?Nf-YC), [fz](http://flybase.bio.indiana.edu/.bin/fbidq.html?fz), [robo](http://flybase.bio.indiana.edu/.bin/fbidq.html?robo), [lea](http://flybase.bio.indiana.edu/.bin/fbidq.html?lea), [bsk](http://flybase.bio.indiana.edu/.bin/fbidq.html?bsk), [brm](http://flybase.bio.indiana.edu/.bin/fbidq.html?brm), [beat-Ia](http://flybase.bio.indiana.edu/.bin/fbidq.html?beat-Ia), [kat-60L1](http://flybase.bio.indiana.edu/.bin/fbidq.html?kat-60L1), [RhoGAPp190](http://flybase.bio.indiana.edu/.bin/fbidq.html?RhoGAPp190), [plexB](http://flybase.bio.indiana.edu/.bin/fbidq.html?plexB), [Klp64D](http://flybase.bio.indiana.edu/.bin/fbidq.html?Klp64D), [grn](http://flybase.bio.indiana.edu/.bin/fbidq.html?grn), [DAAM](http://flybase.bio.indiana.edu/.bin/fbidq.html?DAAM), [mew](http://flybase.bio.indiana.edu/.bin/fbidq.html?mew), [Lis-1](http://flybase.bio.indiana.edu/.bin/fbidq.html?Lis-1), [Brf](http://flybase.bio.indiana.edu/.bin/fbidq.html?Brf), [ems](http://flybase.bio.indiana.edu/.bin/fbidq.html?ems), [pros](http://flybase.bio.indiana.edu/.bin/fbidq.html?pros), [tup](http://flybase.bio.indiana.edu/.bin/fbidq.html?tup), [trio](http://flybase.bio.indiana.edu/.bin/fbidq.html?trio), [CG1463](http://flybase.bio.indiana.edu/.bin/fbidq.html?CG1463),[daw](http://flybase.bio.indiana.edu/.bin/fbidq.html?daw), [Wnt4](http://flybase.bio.indiana.edu/.bin/fbidq.html?Wnt4), [rictor](http://flybase.bio.indiana.edu/.bin/fbidq.html?rictor), [Oseg6](http://flybase.bio.indiana.edu/.bin/fbidq.html?Oseg6), [Apc](http://flybase.bio.indiana.edu/.bin/fbidq.html?Apc), [sim](http://flybase.bio.indiana.edu/.bin/fbidq.html?sim), [d4](http://flybase.bio.indiana.edu/.bin/fbidq.html?d4), [gro](http://flybase.bio.indiana.edu/.bin/fbidq.html?gro), [nerfin-1](http://flybase.bio.indiana.edu/.bin/fbidq.html?nerfin-1), [bchs](http://flybase.bio.indiana.edu/.bin/fbidq.html?bchs), [wnd](http://flybase.bio.indiana.edu/.bin/fbidq.html?wnd), [trx](http://flybase.bio.indiana.edu/.bin/fbidq.html?trx), [Rpd3](http://flybase.bio.indiana.edu/.bin/fbidq.html?Rpd3), [dsx](http://flybase.bio.indiana.edu/.bin/fbidq.html?dsx), [chinmo](http://flybase.bio.indiana.edu/.bin/fbidq.html?chinmo), [CG14367](http://flybase.bio.indiana.edu/.bin/fbidq.html?CG14367), [unc-5](http://flybase.bio.indiana.edu/.bin/fbidq.html?unc-5), [Ssdp](http://flybase.bio.indiana.edu/.bin/fbidq.html?Ssdp), [CG10107](http://flybase.bio.indiana.edu/.bin/fbidq.html?CG10107), [sqz](http://flybase.bio.indiana.edu/.bin/fbidq.html?sqz), [fend](http://flybase.bio.indiana.edu/.bin/fbidq.html?fend), [pygo](http://flybase.bio.indiana.edu/.bin/fbidq.html?pygo), [HLHm7](http://flybase.bio.indiana.edu/.bin/fbidq.html?HLHm7), [wor](http://flybase.bio.indiana.edu/.bin/fbidq.html?wor), [vvl](http://flybase.bio.indiana.edu/.bin/fbidq.html?vvl), [Pten](http://flybase.bio.indiana.edu/.bin/fbidq.html?Pten), [Ptp69D](http://flybase.bio.indiana.edu/.bin/fbidq.html?Ptp69D), [gcm2](http://flybase.bio.indiana.edu/.bin/fbidq.html?gcm2), [hts](http://flybase.bio.indiana.edu/.bin/fbidq.html?hts), [mp](http://flybase.bio.indiana.edu/.bin/fbidq.html?mp), [gcm](http://flybase.bio.indiana.edu/.bin/fbidq.html?gcm), [seq](http://flybase.bio.indiana.edu/.bin/fbidq.html?seq), [CG5890](http://flybase.bio.indiana.edu/.bin/fbidq.html?CG5890), [CG32685](http://flybase.bio.indiana.edu/.bin/fbidq.html?CG32685), [CG4893](http://flybase.bio.indiana.edu/.bin/fbidq.html?CG4893), [CG9098](http://flybase.bio.indiana.edu/.bin/fbidq.html?CG9098),[Alk](http://flybase.bio.indiana.edu/.bin/fbidq.html?Alk), [Sin3A](http://flybase.bio.indiana.edu/.bin/fbidq.html?Sin3A), [jumu](http://flybase.bio.indiana.edu/.bin/fbidq.html?jumu), [Smox](http://flybase.bio.indiana.edu/.bin/fbidq.html?Smox), [CG31475](http://flybase.bio.indiana.edu/.bin/fbidq.html?CG31475), [beat-Ic](http://flybase.bio.indiana.edu/.bin/fbidq.html?beat-Ic), [fd59A](http://flybase.bio.indiana.edu/.bin/fbidq.html?fd59A), [run](http://flybase.bio.indiana.edu/.bin/fbidq.html?run), [brat](http://flybase.bio.indiana.edu/.bin/fbidq.html?brat), [ko](http://flybase.bio.indiana.edu/.bin/fbidq.html?ko), [Kap3](http://flybase.bio.indiana.edu/.bin/fbidq.html?Kap3), [Eip71CD](http://flybase.bio.indiana.edu/.bin/fbidq.html?Eip71CD), [stan](http://flybase.bio.indiana.edu/.bin/fbidq.html?stan), [CG12424](http://flybase.bio.indiana.edu/.bin/fbidq.html?CG12424), [tutl](http://flybase.bio.indiana.edu/.bin/fbidq.html?tutl), [futsch](http://flybase.bio.indiana.edu/.bin/fbidq.html?futsch), [Ptx1](http://flybase.bio.indiana.edu/.bin/fbidq.html?Ptx1), [gukh](http://flybase.bio.indiana.edu/.bin/fbidq.html?gukh), [chm](http://flybase.bio.indiana.edu/.bin/fbidq.html?chm), [sna](http://flybase.bio.indiana.edu/.bin/fbidq.html?sna), [Wnt5](http://flybase.bio.indiana.edu/.bin/fbidq.html?Wnt5), [beat-IIa](http://flybase.bio.indiana.edu/.bin/fbidq.html?beat-IIa), [sm](http://flybase.bio.indiana.edu/.bin/fbidq.html?sm), [dac](http://flybase.bio.indiana.edu/.bin/fbidq.html?dac), [Cdk5](http://flybase.bio.indiana.edu/.bin/fbidq.html?Cdk5), [sif](http://flybase.bio.indiana.edu/.bin/fbidq.html?sif), [Cep135](http://flybase.bio.indiana.edu/.bin/fbidq.html?Cep135), [dom](http://flybase.bio.indiana.edu/.bin/fbidq.html?dom), [Sas-4](http://flybase.bio.indiana.edu/.bin/fbidq.html?Sas-4), [ssh](http://flybase.bio.indiana.edu/.bin/fbidq.html?ssh), [mle](http://flybase.bio.indiana.edu/.bin/fbidq.html?mle), [Hr51](http://flybase.bio.indiana.edu/.bin/fbidq.html?Hr51), [CG11155](http://flybase.bio.indiana.edu/.bin/fbidq.html?CG11155), [rst](http://flybase.bio.indiana.edu/.bin/fbidq.html?rst), [comm](http://flybase.bio.indiana.edu/.bin/fbidq.html?comm), [Cdk5alpha](http://flybase.bio.indiana.edu/.bin/fbidq.html?Cdk5alpha),[Lim3](http://flybase.bio.indiana.edu/.bin/fbidq.html?Lim3), [CadN](http://flybase.bio.indiana.edu/.bin/fbidq.html?CadN), [Aplip1](http://flybase.bio.indiana.edu/.bin/fbidq.html?Aplip1), [E(Pc)](http://flybase.bio.indiana.edu/.bin/fbidq.html?E(Pc)), [retn](http://flybase.bio.indiana.edu/.bin/fbidq.html?retn), [E(bx)](http://flybase.bio.indiana.edu/.bin/fbidq.html?E(bx)), [en](http://flybase.bio.indiana.edu/.bin/fbidq.html?en), [Snap](http://flybase.bio.indiana.edu/.bin/fbidq.html?Snap), [trn](http://flybase.bio.indiana.edu/.bin/fbidq.html?trn), [CG33960](http://flybase.bio.indiana.edu/.bin/fbidq.html?CG33960), [Ct](http://flybase.bio.indiana.edu/.bin/fbidq.html?Ct) |
| [cell part morphogenesis](http://amigo.geneontology.org/cgi-bin/amigo/go.cgi?view=details&query=GO:0032990) | 153 of 1594 genes, 9.6% | 440 of 7634 genes, 5.8% | 6.49e-11 | 0.00% | 0.00 | [fz2](http://flybase.bio.indiana.edu/.bin/fbidq.html?fz2), [Sh](http://flybase.bio.indiana.edu/.bin/fbidq.html?Sh), [acj6](http://flybase.bio.indiana.edu/.bin/fbidq.html?acj6), [jing](http://flybase.bio.indiana.edu/.bin/fbidq.html?jing), [dock](http://flybase.bio.indiana.edu/.bin/fbidq.html?dock), [CG42256](http://flybase.bio.indiana.edu/.bin/fbidq.html?CG42256), [CG3703](http://flybase.bio.indiana.edu/.bin/fbidq.html?CG3703), [ato](http://flybase.bio.indiana.edu/.bin/fbidq.html?ato), [ap](http://flybase.bio.indiana.edu/.bin/fbidq.html?ap), [Iswi](http://flybase.bio.indiana.edu/.bin/fbidq.html?Iswi), [Dg](http://flybase.bio.indiana.edu/.bin/fbidq.html?Dg), [Trim9](http://flybase.bio.indiana.edu/.bin/fbidq.html?Trim9), [CG7154](http://flybase.bio.indiana.edu/.bin/fbidq.html?CG7154), [robo3](http://flybase.bio.indiana.edu/.bin/fbidq.html?robo3), [tok](http://flybase.bio.indiana.edu/.bin/fbidq.html?tok), [Ptp99A](http://flybase.bio.indiana.edu/.bin/fbidq.html?Ptp99A), [lola](http://flybase.bio.indiana.edu/.bin/fbidq.html?lola), [unc-104](http://flybase.bio.indiana.edu/.bin/fbidq.html?unc-104), [chif](http://flybase.bio.indiana.edu/.bin/fbidq.html?chif), [NetB](http://flybase.bio.indiana.edu/.bin/fbidq.html?NetB), [p130CAS](http://flybase.bio.indiana.edu/.bin/fbidq.html?p130CAS), [Tango10](http://flybase.bio.indiana.edu/.bin/fbidq.html?Tango10), [Oseg4](http://flybase.bio.indiana.edu/.bin/fbidq.html?Oseg4), [Appl](http://flybase.bio.indiana.edu/.bin/fbidq.html?Appl), [metro](http://flybase.bio.indiana.edu/.bin/fbidq.html?metro), [CG32137](http://flybase.bio.indiana.edu/.bin/fbidq.html?CG32137), [Nrk](http://flybase.bio.indiana.edu/.bin/fbidq.html?Nrk), [CG4328](http://flybase.bio.indiana.edu/.bin/fbidq.html?CG4328), [Galpha49B](http://flybase.bio.indiana.edu/.bin/fbidq.html?Galpha49B), [Lar](http://flybase.bio.indiana.edu/.bin/fbidq.html?Lar), [Taf4](http://flybase.bio.indiana.edu/.bin/fbidq.html?Taf4), [CG10249](http://flybase.bio.indiana.edu/.bin/fbidq.html?CG10249), [not](http://flybase.bio.indiana.edu/.bin/fbidq.html?not), [rut](http://flybase.bio.indiana.edu/.bin/fbidq.html?rut), [nvy](http://flybase.bio.indiana.edu/.bin/fbidq.html?nvy),[Nrg](http://flybase.bio.indiana.edu/.bin/fbidq.html?Nrg), [gogo](http://flybase.bio.indiana.edu/.bin/fbidq.html?gogo), [jeb](http://flybase.bio.indiana.edu/.bin/fbidq.html?jeb), [GluClalpha](http://flybase.bio.indiana.edu/.bin/fbidq.html?GluClalpha), [Actbeta](http://flybase.bio.indiana.edu/.bin/fbidq.html?Actbeta), [Sema-1a](http://flybase.bio.indiana.edu/.bin/fbidq.html?Sema-1a), [beat-Ib](http://flybase.bio.indiana.edu/.bin/fbidq.html?beat-Ib), [TBPH](http://flybase.bio.indiana.edu/.bin/fbidq.html?TBPH), [otk](http://flybase.bio.indiana.edu/.bin/fbidq.html?otk), [CG34400](http://flybase.bio.indiana.edu/.bin/fbidq.html?CG34400), [Ulp1](http://flybase.bio.indiana.edu/.bin/fbidq.html?Ulp1), [ena](http://flybase.bio.indiana.edu/.bin/fbidq.html?ena), [dsh](http://flybase.bio.indiana.edu/.bin/fbidq.html?dsh), [Fas2](http://flybase.bio.indiana.edu/.bin/fbidq.html?Fas2), [Nf-YC](http://flybase.bio.indiana.edu/.bin/fbidq.html?Nf-YC), [fz](http://flybase.bio.indiana.edu/.bin/fbidq.html?fz), [robo](http://flybase.bio.indiana.edu/.bin/fbidq.html?robo), [lea](http://flybase.bio.indiana.edu/.bin/fbidq.html?lea), [bsk](http://flybase.bio.indiana.edu/.bin/fbidq.html?bsk), [brm](http://flybase.bio.indiana.edu/.bin/fbidq.html?brm), [beat-Ia](http://flybase.bio.indiana.edu/.bin/fbidq.html?beat-Ia), [kat-60L1](http://flybase.bio.indiana.edu/.bin/fbidq.html?kat-60L1), [RhoGAPp190](http://flybase.bio.indiana.edu/.bin/fbidq.html?RhoGAPp190), [plexB](http://flybase.bio.indiana.edu/.bin/fbidq.html?plexB), [Klp64D](http://flybase.bio.indiana.edu/.bin/fbidq.html?Klp64D), [grn](http://flybase.bio.indiana.edu/.bin/fbidq.html?grn), [DAAM](http://flybase.bio.indiana.edu/.bin/fbidq.html?DAAM), [mew](http://flybase.bio.indiana.edu/.bin/fbidq.html?mew), [Lis-1](http://flybase.bio.indiana.edu/.bin/fbidq.html?Lis-1), [Brf](http://flybase.bio.indiana.edu/.bin/fbidq.html?Brf), [ems](http://flybase.bio.indiana.edu/.bin/fbidq.html?ems), [pros](http://flybase.bio.indiana.edu/.bin/fbidq.html?pros), [tup](http://flybase.bio.indiana.edu/.bin/fbidq.html?tup), [trio](http://flybase.bio.indiana.edu/.bin/fbidq.html?trio), [CG1463](http://flybase.bio.indiana.edu/.bin/fbidq.html?CG1463),[daw](http://flybase.bio.indiana.edu/.bin/fbidq.html?daw), [Wnt4](http://flybase.bio.indiana.edu/.bin/fbidq.html?Wnt4), [rictor](http://flybase.bio.indiana.edu/.bin/fbidq.html?rictor), [Oseg6](http://flybase.bio.indiana.edu/.bin/fbidq.html?Oseg6), [Apc](http://flybase.bio.indiana.edu/.bin/fbidq.html?Apc), [sim](http://flybase.bio.indiana.edu/.bin/fbidq.html?sim), [d4](http://flybase.bio.indiana.edu/.bin/fbidq.html?d4), [gro](http://flybase.bio.indiana.edu/.bin/fbidq.html?gro), [nerfin-1](http://flybase.bio.indiana.edu/.bin/fbidq.html?nerfin-1), [bchs](http://flybase.bio.indiana.edu/.bin/fbidq.html?bchs), [wnd](http://flybase.bio.indiana.edu/.bin/fbidq.html?wnd), [trx](http://flybase.bio.indiana.edu/.bin/fbidq.html?trx), [Rpd3](http://flybase.bio.indiana.edu/.bin/fbidq.html?Rpd3), [dsx](http://flybase.bio.indiana.edu/.bin/fbidq.html?dsx), [chinmo](http://flybase.bio.indiana.edu/.bin/fbidq.html?chinmo), [CG14367](http://flybase.bio.indiana.edu/.bin/fbidq.html?CG14367), [unc-5](http://flybase.bio.indiana.edu/.bin/fbidq.html?unc-5), [Ssdp](http://flybase.bio.indiana.edu/.bin/fbidq.html?Ssdp), [CG10107](http://flybase.bio.indiana.edu/.bin/fbidq.html?CG10107), [sqz](http://flybase.bio.indiana.edu/.bin/fbidq.html?sqz), [fend](http://flybase.bio.indiana.edu/.bin/fbidq.html?fend), [pygo](http://flybase.bio.indiana.edu/.bin/fbidq.html?pygo), [HLHm7](http://flybase.bio.indiana.edu/.bin/fbidq.html?HLHm7), [wor](http://flybase.bio.indiana.edu/.bin/fbidq.html?wor), [vvl](http://flybase.bio.indiana.edu/.bin/fbidq.html?vvl), [Pten](http://flybase.bio.indiana.edu/.bin/fbidq.html?Pten), [Ptp69D](http://flybase.bio.indiana.edu/.bin/fbidq.html?Ptp69D), [gcm2](http://flybase.bio.indiana.edu/.bin/fbidq.html?gcm2), [hts](http://flybase.bio.indiana.edu/.bin/fbidq.html?hts), [mp](http://flybase.bio.indiana.edu/.bin/fbidq.html?mp), [gcm](http://flybase.bio.indiana.edu/.bin/fbidq.html?gcm), [seq](http://flybase.bio.indiana.edu/.bin/fbidq.html?seq), [CG5890](http://flybase.bio.indiana.edu/.bin/fbidq.html?CG5890), [CG32685](http://flybase.bio.indiana.edu/.bin/fbidq.html?CG32685), [CG4893](http://flybase.bio.indiana.edu/.bin/fbidq.html?CG4893), [CG9098](http://flybase.bio.indiana.edu/.bin/fbidq.html?CG9098),[Alk](http://flybase.bio.indiana.edu/.bin/fbidq.html?Alk), [Sin3A](http://flybase.bio.indiana.edu/.bin/fbidq.html?Sin3A), [jumu](http://flybase.bio.indiana.edu/.bin/fbidq.html?jumu), [Smox](http://flybase.bio.indiana.edu/.bin/fbidq.html?Smox), [CG31475](http://flybase.bio.indiana.edu/.bin/fbidq.html?CG31475), [beat-Ic](http://flybase.bio.indiana.edu/.bin/fbidq.html?beat-Ic), [fd59A](http://flybase.bio.indiana.edu/.bin/fbidq.html?fd59A), [run](http://flybase.bio.indiana.edu/.bin/fbidq.html?run), [brat](http://flybase.bio.indiana.edu/.bin/fbidq.html?brat), [ko](http://flybase.bio.indiana.edu/.bin/fbidq.html?ko), [Kap3](http://flybase.bio.indiana.edu/.bin/fbidq.html?Kap3), [Eip71CD](http://flybase.bio.indiana.edu/.bin/fbidq.html?Eip71CD), [stan](http://flybase.bio.indiana.edu/.bin/fbidq.html?stan), [CG12424](http://flybase.bio.indiana.edu/.bin/fbidq.html?CG12424), [tutl](http://flybase.bio.indiana.edu/.bin/fbidq.html?tutl), [futsch](http://flybase.bio.indiana.edu/.bin/fbidq.html?futsch), [Ptx1](http://flybase.bio.indiana.edu/.bin/fbidq.html?Ptx1), [gukh](http://flybase.bio.indiana.edu/.bin/fbidq.html?gukh), [chm](http://flybase.bio.indiana.edu/.bin/fbidq.html?chm), [sna](http://flybase.bio.indiana.edu/.bin/fbidq.html?sna), [Wnt5](http://flybase.bio.indiana.edu/.bin/fbidq.html?Wnt5), [beat-IIa](http://flybase.bio.indiana.edu/.bin/fbidq.html?beat-IIa), [sm](http://flybase.bio.indiana.edu/.bin/fbidq.html?sm), [dac](http://flybase.bio.indiana.edu/.bin/fbidq.html?dac), [Cdk5](http://flybase.bio.indiana.edu/.bin/fbidq.html?Cdk5), [sif](http://flybase.bio.indiana.edu/.bin/fbidq.html?sif), [Cep135](http://flybase.bio.indiana.edu/.bin/fbidq.html?Cep135), [dom](http://flybase.bio.indiana.edu/.bin/fbidq.html?dom), [Sas-4](http://flybase.bio.indiana.edu/.bin/fbidq.html?Sas-4), [ssh](http://flybase.bio.indiana.edu/.bin/fbidq.html?ssh), [mle](http://flybase.bio.indiana.edu/.bin/fbidq.html?mle), [Hr51](http://flybase.bio.indiana.edu/.bin/fbidq.html?Hr51), [CG11155](http://flybase.bio.indiana.edu/.bin/fbidq.html?CG11155), [rst](http://flybase.bio.indiana.edu/.bin/fbidq.html?rst), [comm](http://flybase.bio.indiana.edu/.bin/fbidq.html?comm), [Cdk5alpha](http://flybase.bio.indiana.edu/.bin/fbidq.html?Cdk5alpha),[Lim3](http://flybase.bio.indiana.edu/.bin/fbidq.html?Lim3), [CadN](http://flybase.bio.indiana.edu/.bin/fbidq.html?CadN), [Aplip1](http://flybase.bio.indiana.edu/.bin/fbidq.html?Aplip1), [E(Pc)](http://flybase.bio.indiana.edu/.bin/fbidq.html?E(Pc)), [retn](http://flybase.bio.indiana.edu/.bin/fbidq.html?retn), [E(bx)](http://flybase.bio.indiana.edu/.bin/fbidq.html?E(bx)), [en](http://flybase.bio.indiana.edu/.bin/fbidq.html?en), [Snap](http://flybase.bio.indiana.edu/.bin/fbidq.html?Snap), [trn](http://flybase.bio.indiana.edu/.bin/fbidq.html?trn), [CG33960](http://flybase.bio.indiana.edu/.bin/fbidq.html?CG33960), [Ct](http://flybase.bio.indiana.edu/.bin/fbidq.html?Ct) |
| [neuron development](http://amigo.geneontology.org/cgi-bin/amigo/go.cgi?view=details&query=GO:0048666) | 174 of 1594 genes, 10.9% | 521 of 7634 genes, 6.8% | 6.63e-11 | 0.00% | 0.00 | [fz2](http://flybase.bio.indiana.edu/.bin/fbidq.html?fz2), [Sh](http://flybase.bio.indiana.edu/.bin/fbidq.html?Sh), [acj6](http://flybase.bio.indiana.edu/.bin/fbidq.html?acj6), [jing](http://flybase.bio.indiana.edu/.bin/fbidq.html?jing), [dock](http://flybase.bio.indiana.edu/.bin/fbidq.html?dock), [CG42256](http://flybase.bio.indiana.edu/.bin/fbidq.html?CG42256), [CG3703](http://flybase.bio.indiana.edu/.bin/fbidq.html?CG3703), [vimar](http://flybase.bio.indiana.edu/.bin/fbidq.html?vimar), [ato](http://flybase.bio.indiana.edu/.bin/fbidq.html?ato), [Iswi](http://flybase.bio.indiana.edu/.bin/fbidq.html?Iswi), [ap](http://flybase.bio.indiana.edu/.bin/fbidq.html?ap), [Dg](http://flybase.bio.indiana.edu/.bin/fbidq.html?Dg), [Trim9](http://flybase.bio.indiana.edu/.bin/fbidq.html?Trim9), [CG7154](http://flybase.bio.indiana.edu/.bin/fbidq.html?CG7154), [robo3](http://flybase.bio.indiana.edu/.bin/fbidq.html?robo3), [os](http://flybase.bio.indiana.edu/.bin/fbidq.html?os), [tok](http://flybase.bio.indiana.edu/.bin/fbidq.html?tok), [Ptp99A](http://flybase.bio.indiana.edu/.bin/fbidq.html?Ptp99A), [lola](http://flybase.bio.indiana.edu/.bin/fbidq.html?lola), [unc-104](http://flybase.bio.indiana.edu/.bin/fbidq.html?unc-104), [chif](http://flybase.bio.indiana.edu/.bin/fbidq.html?chif), [NetB](http://flybase.bio.indiana.edu/.bin/fbidq.html?NetB), [p130CAS](http://flybase.bio.indiana.edu/.bin/fbidq.html?p130CAS), [Tango10](http://flybase.bio.indiana.edu/.bin/fbidq.html?Tango10), [Appl](http://flybase.bio.indiana.edu/.bin/fbidq.html?Appl), [metro](http://flybase.bio.indiana.edu/.bin/fbidq.html?metro), [CG32137](http://flybase.bio.indiana.edu/.bin/fbidq.html?CG32137), [Nrk](http://flybase.bio.indiana.edu/.bin/fbidq.html?Nrk), [CG4328](http://flybase.bio.indiana.edu/.bin/fbidq.html?CG4328), [Galpha49B](http://flybase.bio.indiana.edu/.bin/fbidq.html?Galpha49B), [Snoo](http://flybase.bio.indiana.edu/.bin/fbidq.html?Snoo), [Lar](http://flybase.bio.indiana.edu/.bin/fbidq.html?Lar), [Taf4](http://flybase.bio.indiana.edu/.bin/fbidq.html?Taf4), [CG10249](http://flybase.bio.indiana.edu/.bin/fbidq.html?CG10249), [not](http://flybase.bio.indiana.edu/.bin/fbidq.html?not),[rut](http://flybase.bio.indiana.edu/.bin/fbidq.html?rut), [nvy](http://flybase.bio.indiana.edu/.bin/fbidq.html?nvy), [Nrg](http://flybase.bio.indiana.edu/.bin/fbidq.html?Nrg), [gogo](http://flybase.bio.indiana.edu/.bin/fbidq.html?gogo), [nab](http://flybase.bio.indiana.edu/.bin/fbidq.html?nab), [jeb](http://flybase.bio.indiana.edu/.bin/fbidq.html?jeb), [GluClalpha](http://flybase.bio.indiana.edu/.bin/fbidq.html?GluClalpha), [Actbeta](http://flybase.bio.indiana.edu/.bin/fbidq.html?Actbeta), [Sema-1a](http://flybase.bio.indiana.edu/.bin/fbidq.html?Sema-1a), [beat-Ib](http://flybase.bio.indiana.edu/.bin/fbidq.html?beat-Ib), [TBPH](http://flybase.bio.indiana.edu/.bin/fbidq.html?TBPH), [otk](http://flybase.bio.indiana.edu/.bin/fbidq.html?otk), [CG34400](http://flybase.bio.indiana.edu/.bin/fbidq.html?CG34400), [phyl](http://flybase.bio.indiana.edu/.bin/fbidq.html?phyl), [Ulp1](http://flybase.bio.indiana.edu/.bin/fbidq.html?Ulp1), [ena](http://flybase.bio.indiana.edu/.bin/fbidq.html?ena), [dsh](http://flybase.bio.indiana.edu/.bin/fbidq.html?dsh), [Fas2](http://flybase.bio.indiana.edu/.bin/fbidq.html?Fas2), [Nf-YC](http://flybase.bio.indiana.edu/.bin/fbidq.html?Nf-YC), [eya](http://flybase.bio.indiana.edu/.bin/fbidq.html?eya), [fz](http://flybase.bio.indiana.edu/.bin/fbidq.html?fz), [robo](http://flybase.bio.indiana.edu/.bin/fbidq.html?robo), [lea](http://flybase.bio.indiana.edu/.bin/fbidq.html?lea), [bsk](http://flybase.bio.indiana.edu/.bin/fbidq.html?bsk), [brm](http://flybase.bio.indiana.edu/.bin/fbidq.html?brm), [beat-Ia](http://flybase.bio.indiana.edu/.bin/fbidq.html?beat-Ia), [kat-60L1](http://flybase.bio.indiana.edu/.bin/fbidq.html?kat-60L1), [hig](http://flybase.bio.indiana.edu/.bin/fbidq.html?hig), [CG12004](http://flybase.bio.indiana.edu/.bin/fbidq.html?CG12004), [RhoGAPp190](http://flybase.bio.indiana.edu/.bin/fbidq.html?RhoGAPp190), [plexB](http://flybase.bio.indiana.edu/.bin/fbidq.html?plexB), [Klp64D](http://flybase.bio.indiana.edu/.bin/fbidq.html?Klp64D), [grn](http://flybase.bio.indiana.edu/.bin/fbidq.html?grn), [DAAM](http://flybase.bio.indiana.edu/.bin/fbidq.html?DAAM), [mew](http://flybase.bio.indiana.edu/.bin/fbidq.html?mew), [neur](http://flybase.bio.indiana.edu/.bin/fbidq.html?neur),[didum](http://flybase.bio.indiana.edu/.bin/fbidq.html?didum), [Lis-1](http://flybase.bio.indiana.edu/.bin/fbidq.html?Lis-1), [Brf](http://flybase.bio.indiana.edu/.bin/fbidq.html?Brf), [ems](http://flybase.bio.indiana.edu/.bin/fbidq.html?ems), [eys](http://flybase.bio.indiana.edu/.bin/fbidq.html?eys), [Mob2](http://flybase.bio.indiana.edu/.bin/fbidq.html?Mob2), [pros](http://flybase.bio.indiana.edu/.bin/fbidq.html?pros), [tup](http://flybase.bio.indiana.edu/.bin/fbidq.html?tup), [trio](http://flybase.bio.indiana.edu/.bin/fbidq.html?trio), [CG1463](http://flybase.bio.indiana.edu/.bin/fbidq.html?CG1463), [daw](http://flybase.bio.indiana.edu/.bin/fbidq.html?daw), [Wnt4](http://flybase.bio.indiana.edu/.bin/fbidq.html?Wnt4), [rictor](http://flybase.bio.indiana.edu/.bin/fbidq.html?rictor), [Apc](http://flybase.bio.indiana.edu/.bin/fbidq.html?Apc), [sim](http://flybase.bio.indiana.edu/.bin/fbidq.html?sim), [d4](http://flybase.bio.indiana.edu/.bin/fbidq.html?d4), [gro](http://flybase.bio.indiana.edu/.bin/fbidq.html?gro), [nerfin-1](http://flybase.bio.indiana.edu/.bin/fbidq.html?nerfin-1), [bchs](http://flybase.bio.indiana.edu/.bin/fbidq.html?bchs), [wnd](http://flybase.bio.indiana.edu/.bin/fbidq.html?wnd), [trx](http://flybase.bio.indiana.edu/.bin/fbidq.html?trx), [Rpd3](http://flybase.bio.indiana.edu/.bin/fbidq.html?Rpd3), [dsx](http://flybase.bio.indiana.edu/.bin/fbidq.html?dsx), [chinmo](http://flybase.bio.indiana.edu/.bin/fbidq.html?chinmo), [unc-5](http://flybase.bio.indiana.edu/.bin/fbidq.html?unc-5), [Ssdp](http://flybase.bio.indiana.edu/.bin/fbidq.html?Ssdp), [CG10107](http://flybase.bio.indiana.edu/.bin/fbidq.html?CG10107), [sqz](http://flybase.bio.indiana.edu/.bin/fbidq.html?sqz), [fend](http://flybase.bio.indiana.edu/.bin/fbidq.html?fend), [pygo](http://flybase.bio.indiana.edu/.bin/fbidq.html?pygo), [HLHm7](http://flybase.bio.indiana.edu/.bin/fbidq.html?HLHm7), [wor](http://flybase.bio.indiana.edu/.bin/fbidq.html?wor), [vvl](http://flybase.bio.indiana.edu/.bin/fbidq.html?vvl), [Pten](http://flybase.bio.indiana.edu/.bin/fbidq.html?Pten), [Ptp69D](http://flybase.bio.indiana.edu/.bin/fbidq.html?Ptp69D), [gcm2](http://flybase.bio.indiana.edu/.bin/fbidq.html?gcm2), [hts](http://flybase.bio.indiana.edu/.bin/fbidq.html?hts), [mp](http://flybase.bio.indiana.edu/.bin/fbidq.html?mp), [gcm](http://flybase.bio.indiana.edu/.bin/fbidq.html?gcm), [seq](http://flybase.bio.indiana.edu/.bin/fbidq.html?seq),[CG5890](http://flybase.bio.indiana.edu/.bin/fbidq.html?CG5890), [Dip3](http://flybase.bio.indiana.edu/.bin/fbidq.html?Dip3), [SP2353](http://flybase.bio.indiana.edu/.bin/fbidq.html?SP2353), [CG32685](http://flybase.bio.indiana.edu/.bin/fbidq.html?CG32685), [CG4893](http://flybase.bio.indiana.edu/.bin/fbidq.html?CG4893), [CG9098](http://flybase.bio.indiana.edu/.bin/fbidq.html?CG9098), [Alk](http://flybase.bio.indiana.edu/.bin/fbidq.html?Alk), [esn](http://flybase.bio.indiana.edu/.bin/fbidq.html?esn), [Vang](http://flybase.bio.indiana.edu/.bin/fbidq.html?Vang), [Sin3A](http://flybase.bio.indiana.edu/.bin/fbidq.html?Sin3A), [pot](http://flybase.bio.indiana.edu/.bin/fbidq.html?pot), [jumu](http://flybase.bio.indiana.edu/.bin/fbidq.html?jumu), [Smox](http://flybase.bio.indiana.edu/.bin/fbidq.html?Smox), [CG31475](http://flybase.bio.indiana.edu/.bin/fbidq.html?CG31475), [beat-Ic](http://flybase.bio.indiana.edu/.bin/fbidq.html?beat-Ic), [fd59A](http://flybase.bio.indiana.edu/.bin/fbidq.html?fd59A), [run](http://flybase.bio.indiana.edu/.bin/fbidq.html?run), [brat](http://flybase.bio.indiana.edu/.bin/fbidq.html?brat), [ko](http://flybase.bio.indiana.edu/.bin/fbidq.html?ko), [Eip71CD](http://flybase.bio.indiana.edu/.bin/fbidq.html?Eip71CD), [stan](http://flybase.bio.indiana.edu/.bin/fbidq.html?stan), [CG12424](http://flybase.bio.indiana.edu/.bin/fbidq.html?CG12424), [tutl](http://flybase.bio.indiana.edu/.bin/fbidq.html?tutl), [futsch](http://flybase.bio.indiana.edu/.bin/fbidq.html?futsch), [Ptx1](http://flybase.bio.indiana.edu/.bin/fbidq.html?Ptx1), [ph-p](http://flybase.bio.indiana.edu/.bin/fbidq.html?ph-p), [gukh](http://flybase.bio.indiana.edu/.bin/fbidq.html?gukh), [chm](http://flybase.bio.indiana.edu/.bin/fbidq.html?chm), [Rim](http://flybase.bio.indiana.edu/.bin/fbidq.html?Rim), [sna](http://flybase.bio.indiana.edu/.bin/fbidq.html?sna), [Wnt5](http://flybase.bio.indiana.edu/.bin/fbidq.html?Wnt5), [beat-IIa](http://flybase.bio.indiana.edu/.bin/fbidq.html?beat-IIa), [sm](http://flybase.bio.indiana.edu/.bin/fbidq.html?sm), [Syn2](http://flybase.bio.indiana.edu/.bin/fbidq.html?Syn2), [dac](http://flybase.bio.indiana.edu/.bin/fbidq.html?dac),[Cdk5](http://flybase.bio.indiana.edu/.bin/fbidq.html?Cdk5), [sif](http://flybase.bio.indiana.edu/.bin/fbidq.html?sif), [oc](http://flybase.bio.indiana.edu/.bin/fbidq.html?oc), [dom](http://flybase.bio.indiana.edu/.bin/fbidq.html?dom), [ssh](http://flybase.bio.indiana.edu/.bin/fbidq.html?ssh), [baz](http://flybase.bio.indiana.edu/.bin/fbidq.html?baz), [klar](http://flybase.bio.indiana.edu/.bin/fbidq.html?klar), [mle](http://flybase.bio.indiana.edu/.bin/fbidq.html?mle), [Hr51](http://flybase.bio.indiana.edu/.bin/fbidq.html?Hr51), [CG11155](http://flybase.bio.indiana.edu/.bin/fbidq.html?CG11155), [rst](http://flybase.bio.indiana.edu/.bin/fbidq.html?rst), [grh](http://flybase.bio.indiana.edu/.bin/fbidq.html?grh), [comm](http://flybase.bio.indiana.edu/.bin/fbidq.html?comm), [Cdk5alpha](http://flybase.bio.indiana.edu/.bin/fbidq.html?Cdk5alpha), [Lim3](http://flybase.bio.indiana.edu/.bin/fbidq.html?Lim3), [CadN](http://flybase.bio.indiana.edu/.bin/fbidq.html?CadN), [Aplip1](http://flybase.bio.indiana.edu/.bin/fbidq.html?Aplip1), [E(Pc)](http://flybase.bio.indiana.edu/.bin/fbidq.html?E(Pc)), [retn](http://flybase.bio.indiana.edu/.bin/fbidq.html?retn), [E(bx)](http://flybase.bio.indiana.edu/.bin/fbidq.html?E(bx)), [en](http://flybase.bio.indiana.edu/.bin/fbidq.html?en), [rl](http://flybase.bio.indiana.edu/.bin/fbidq.html?rl), [Snap](http://flybase.bio.indiana.edu/.bin/fbidq.html?Snap), [trn](http://flybase.bio.indiana.edu/.bin/fbidq.html?trn), [tey](http://flybase.bio.indiana.edu/.bin/fbidq.html?tey), [yrt](http://flybase.bio.indiana.edu/.bin/fbidq.html?yrt), [CG33960](http://flybase.bio.indiana.edu/.bin/fbidq.html?CG33960), [Ct](http://flybase.bio.indiana.edu/.bin/fbidq.html?Ct) |
| [cell morphogenesis](http://amigo.geneontology.org/cgi-bin/amigo/go.cgi?view=details&query=GO:0000902) | 180 of 1594 genes, 11.3% | 548 of 7634 genes, 7.2% | 1.31e-10 | 0.00% | 0.00 | [fz2](http://flybase.bio.indiana.edu/.bin/fbidq.html?fz2), [Sh](http://flybase.bio.indiana.edu/.bin/fbidq.html?Sh), [acj6](http://flybase.bio.indiana.edu/.bin/fbidq.html?acj6), [jing](http://flybase.bio.indiana.edu/.bin/fbidq.html?jing), [dock](http://flybase.bio.indiana.edu/.bin/fbidq.html?dock), [CG42256](http://flybase.bio.indiana.edu/.bin/fbidq.html?CG42256), [CG3703](http://flybase.bio.indiana.edu/.bin/fbidq.html?CG3703), [ato](http://flybase.bio.indiana.edu/.bin/fbidq.html?ato), [Iswi](http://flybase.bio.indiana.edu/.bin/fbidq.html?Iswi), [ap](http://flybase.bio.indiana.edu/.bin/fbidq.html?ap), [Dg](http://flybase.bio.indiana.edu/.bin/fbidq.html?Dg), [Trim9](http://flybase.bio.indiana.edu/.bin/fbidq.html?Trim9), [tlk](http://flybase.bio.indiana.edu/.bin/fbidq.html?tlk), [CG7154](http://flybase.bio.indiana.edu/.bin/fbidq.html?CG7154), [neo](http://flybase.bio.indiana.edu/.bin/fbidq.html?neo), [robo3](http://flybase.bio.indiana.edu/.bin/fbidq.html?robo3), [os](http://flybase.bio.indiana.edu/.bin/fbidq.html?os), [CG3618](http://flybase.bio.indiana.edu/.bin/fbidq.html?CG3618), [tok](http://flybase.bio.indiana.edu/.bin/fbidq.html?tok), [Ptp99A](http://flybase.bio.indiana.edu/.bin/fbidq.html?Ptp99A), [lola](http://flybase.bio.indiana.edu/.bin/fbidq.html?lola), [unc-104](http://flybase.bio.indiana.edu/.bin/fbidq.html?unc-104), [CG31961](http://flybase.bio.indiana.edu/.bin/fbidq.html?CG31961), [chif](http://flybase.bio.indiana.edu/.bin/fbidq.html?chif), [NetB](http://flybase.bio.indiana.edu/.bin/fbidq.html?NetB), [p130CAS](http://flybase.bio.indiana.edu/.bin/fbidq.html?p130CAS), [Tango10](http://flybase.bio.indiana.edu/.bin/fbidq.html?Tango10), [Oseg4](http://flybase.bio.indiana.edu/.bin/fbidq.html?Oseg4), [Appl](http://flybase.bio.indiana.edu/.bin/fbidq.html?Appl), [metro](http://flybase.bio.indiana.edu/.bin/fbidq.html?metro), [CG32137](http://flybase.bio.indiana.edu/.bin/fbidq.html?CG32137), [Nrk](http://flybase.bio.indiana.edu/.bin/fbidq.html?Nrk), [CG4328](http://flybase.bio.indiana.edu/.bin/fbidq.html?CG4328), [Galpha49B](http://flybase.bio.indiana.edu/.bin/fbidq.html?Galpha49B), [Lar](http://flybase.bio.indiana.edu/.bin/fbidq.html?Lar),[Taf4](http://flybase.bio.indiana.edu/.bin/fbidq.html?Taf4), [CG10249](http://flybase.bio.indiana.edu/.bin/fbidq.html?CG10249), [scrib](http://flybase.bio.indiana.edu/.bin/fbidq.html?scrib), [not](http://flybase.bio.indiana.edu/.bin/fbidq.html?not), [rut](http://flybase.bio.indiana.edu/.bin/fbidq.html?rut), [nvy](http://flybase.bio.indiana.edu/.bin/fbidq.html?nvy), [Nrg](http://flybase.bio.indiana.edu/.bin/fbidq.html?Nrg), [gogo](http://flybase.bio.indiana.edu/.bin/fbidq.html?gogo), [jeb](http://flybase.bio.indiana.edu/.bin/fbidq.html?jeb), [GluClalpha](http://flybase.bio.indiana.edu/.bin/fbidq.html?GluClalpha), [Actbeta](http://flybase.bio.indiana.edu/.bin/fbidq.html?Actbeta), [Sema-1a](http://flybase.bio.indiana.edu/.bin/fbidq.html?Sema-1a), [beat-Ib](http://flybase.bio.indiana.edu/.bin/fbidq.html?beat-Ib), [Traf4](http://flybase.bio.indiana.edu/.bin/fbidq.html?Traf4), [TBPH](http://flybase.bio.indiana.edu/.bin/fbidq.html?TBPH), [otk](http://flybase.bio.indiana.edu/.bin/fbidq.html?otk), [Sos](http://flybase.bio.indiana.edu/.bin/fbidq.html?Sos), [CG34400](http://flybase.bio.indiana.edu/.bin/fbidq.html?CG34400), [Ulp1](http://flybase.bio.indiana.edu/.bin/fbidq.html?Ulp1), [ena](http://flybase.bio.indiana.edu/.bin/fbidq.html?ena), [dsh](http://flybase.bio.indiana.edu/.bin/fbidq.html?dsh), [Fas2](http://flybase.bio.indiana.edu/.bin/fbidq.html?Fas2), [Nf-YC](http://flybase.bio.indiana.edu/.bin/fbidq.html?Nf-YC), [fz](http://flybase.bio.indiana.edu/.bin/fbidq.html?fz), [robo](http://flybase.bio.indiana.edu/.bin/fbidq.html?robo), [lea](http://flybase.bio.indiana.edu/.bin/fbidq.html?lea), [bsk](http://flybase.bio.indiana.edu/.bin/fbidq.html?bsk), [brm](http://flybase.bio.indiana.edu/.bin/fbidq.html?brm), [beat-Ia](http://flybase.bio.indiana.edu/.bin/fbidq.html?beat-Ia), [kat-60L1](http://flybase.bio.indiana.edu/.bin/fbidq.html?kat-60L1), [ft](http://flybase.bio.indiana.edu/.bin/fbidq.html?ft), [RhoGAPp190](http://flybase.bio.indiana.edu/.bin/fbidq.html?RhoGAPp190), [plexB](http://flybase.bio.indiana.edu/.bin/fbidq.html?plexB), [Klp64D](http://flybase.bio.indiana.edu/.bin/fbidq.html?Klp64D), [grn](http://flybase.bio.indiana.edu/.bin/fbidq.html?grn), [DAAM](http://flybase.bio.indiana.edu/.bin/fbidq.html?DAAM),[mew](http://flybase.bio.indiana.edu/.bin/fbidq.html?mew), [Lis-1](http://flybase.bio.indiana.edu/.bin/fbidq.html?Lis-1), [CycE](http://flybase.bio.indiana.edu/.bin/fbidq.html?CycE), [Brf](http://flybase.bio.indiana.edu/.bin/fbidq.html?Brf), [ems](http://flybase.bio.indiana.edu/.bin/fbidq.html?ems), [eys](http://flybase.bio.indiana.edu/.bin/fbidq.html?eys), [Mob2](http://flybase.bio.indiana.edu/.bin/fbidq.html?Mob2), [pros](http://flybase.bio.indiana.edu/.bin/fbidq.html?pros), [tup](http://flybase.bio.indiana.edu/.bin/fbidq.html?tup), [trio](http://flybase.bio.indiana.edu/.bin/fbidq.html?trio), [ed](http://flybase.bio.indiana.edu/.bin/fbidq.html?ed), [CG1463](http://flybase.bio.indiana.edu/.bin/fbidq.html?CG1463), [daw](http://flybase.bio.indiana.edu/.bin/fbidq.html?daw), [Wnt4](http://flybase.bio.indiana.edu/.bin/fbidq.html?Wnt4), [rictor](http://flybase.bio.indiana.edu/.bin/fbidq.html?rictor), [Oseg6](http://flybase.bio.indiana.edu/.bin/fbidq.html?Oseg6), [Apc](http://flybase.bio.indiana.edu/.bin/fbidq.html?Apc), [sim](http://flybase.bio.indiana.edu/.bin/fbidq.html?sim), [d4](http://flybase.bio.indiana.edu/.bin/fbidq.html?d4), [gro](http://flybase.bio.indiana.edu/.bin/fbidq.html?gro), [pigs](http://flybase.bio.indiana.edu/.bin/fbidq.html?pigs), [nerfin-1](http://flybase.bio.indiana.edu/.bin/fbidq.html?nerfin-1), [bchs](http://flybase.bio.indiana.edu/.bin/fbidq.html?bchs), [wnd](http://flybase.bio.indiana.edu/.bin/fbidq.html?wnd), [trx](http://flybase.bio.indiana.edu/.bin/fbidq.html?trx), [Rpd3](http://flybase.bio.indiana.edu/.bin/fbidq.html?Rpd3), [dsx](http://flybase.bio.indiana.edu/.bin/fbidq.html?dsx), [chinmo](http://flybase.bio.indiana.edu/.bin/fbidq.html?chinmo), [CG14367](http://flybase.bio.indiana.edu/.bin/fbidq.html?CG14367), [unc-5](http://flybase.bio.indiana.edu/.bin/fbidq.html?unc-5), [Ssdp](http://flybase.bio.indiana.edu/.bin/fbidq.html?Ssdp), [CG10107](http://flybase.bio.indiana.edu/.bin/fbidq.html?CG10107), [sqz](http://flybase.bio.indiana.edu/.bin/fbidq.html?sqz), [fend](http://flybase.bio.indiana.edu/.bin/fbidq.html?fend), [pygo](http://flybase.bio.indiana.edu/.bin/fbidq.html?pygo), [HLHm7](http://flybase.bio.indiana.edu/.bin/fbidq.html?HLHm7), [wor](http://flybase.bio.indiana.edu/.bin/fbidq.html?wor), [vvl](http://flybase.bio.indiana.edu/.bin/fbidq.html?vvl),[RhoGEF4](http://flybase.bio.indiana.edu/.bin/fbidq.html?RhoGEF4), [Pten](http://flybase.bio.indiana.edu/.bin/fbidq.html?Pten), [Ptp69D](http://flybase.bio.indiana.edu/.bin/fbidq.html?Ptp69D), [sgg](http://flybase.bio.indiana.edu/.bin/fbidq.html?sgg), [gcm2](http://flybase.bio.indiana.edu/.bin/fbidq.html?gcm2), [hts](http://flybase.bio.indiana.edu/.bin/fbidq.html?hts), [mp](http://flybase.bio.indiana.edu/.bin/fbidq.html?mp), [gcm](http://flybase.bio.indiana.edu/.bin/fbidq.html?gcm), [seq](http://flybase.bio.indiana.edu/.bin/fbidq.html?seq), [CG5890](http://flybase.bio.indiana.edu/.bin/fbidq.html?CG5890), [CG32685](http://flybase.bio.indiana.edu/.bin/fbidq.html?CG32685), [CG4893](http://flybase.bio.indiana.edu/.bin/fbidq.html?CG4893), [ds](http://flybase.bio.indiana.edu/.bin/fbidq.html?ds), [CASK](http://flybase.bio.indiana.edu/.bin/fbidq.html?CASK), [CG9098](http://flybase.bio.indiana.edu/.bin/fbidq.html?CG9098), [Alk](http://flybase.bio.indiana.edu/.bin/fbidq.html?Alk), [Sin3A](http://flybase.bio.indiana.edu/.bin/fbidq.html?Sin3A), [jumu](http://flybase.bio.indiana.edu/.bin/fbidq.html?jumu), [Smox](http://flybase.bio.indiana.edu/.bin/fbidq.html?Smox), [CG31475](http://flybase.bio.indiana.edu/.bin/fbidq.html?CG31475), [CG32944](http://flybase.bio.indiana.edu/.bin/fbidq.html?CG32944), [beat-Ic](http://flybase.bio.indiana.edu/.bin/fbidq.html?beat-Ic), [fd59A](http://flybase.bio.indiana.edu/.bin/fbidq.html?fd59A), [Fak56D](http://flybase.bio.indiana.edu/.bin/fbidq.html?Fak56D), [run](http://flybase.bio.indiana.edu/.bin/fbidq.html?run), [brat](http://flybase.bio.indiana.edu/.bin/fbidq.html?brat), [ko](http://flybase.bio.indiana.edu/.bin/fbidq.html?ko), [Kap3](http://flybase.bio.indiana.edu/.bin/fbidq.html?Kap3), [Eip71CD](http://flybase.bio.indiana.edu/.bin/fbidq.html?Eip71CD), [stan](http://flybase.bio.indiana.edu/.bin/fbidq.html?stan), [CG12424](http://flybase.bio.indiana.edu/.bin/fbidq.html?CG12424), [tutl](http://flybase.bio.indiana.edu/.bin/fbidq.html?tutl), [futsch](http://flybase.bio.indiana.edu/.bin/fbidq.html?futsch),[Ptx1](http://flybase.bio.indiana.edu/.bin/fbidq.html?Ptx1), [ph-p](http://flybase.bio.indiana.edu/.bin/fbidq.html?ph-p), [gukh](http://flybase.bio.indiana.edu/.bin/fbidq.html?gukh), [chm](http://flybase.bio.indiana.edu/.bin/fbidq.html?chm), [sna](http://flybase.bio.indiana.edu/.bin/fbidq.html?sna), [Wnt5](http://flybase.bio.indiana.edu/.bin/fbidq.html?Wnt5), [beat-IIa](http://flybase.bio.indiana.edu/.bin/fbidq.html?beat-IIa), [Ggamma1](http://flybase.bio.indiana.edu/.bin/fbidq.html?Ggamma1), [sm](http://flybase.bio.indiana.edu/.bin/fbidq.html?sm), [dac](http://flybase.bio.indiana.edu/.bin/fbidq.html?dac), [Cdk5](http://flybase.bio.indiana.edu/.bin/fbidq.html?Cdk5), [sif](http://flybase.bio.indiana.edu/.bin/fbidq.html?sif), [Cep135](http://flybase.bio.indiana.edu/.bin/fbidq.html?Cep135), [dom](http://flybase.bio.indiana.edu/.bin/fbidq.html?dom), [Sas-4](http://flybase.bio.indiana.edu/.bin/fbidq.html?Sas-4), [ssh](http://flybase.bio.indiana.edu/.bin/fbidq.html?ssh), [baz](http://flybase.bio.indiana.edu/.bin/fbidq.html?baz), [mle](http://flybase.bio.indiana.edu/.bin/fbidq.html?mle), [Hr51](http://flybase.bio.indiana.edu/.bin/fbidq.html?Hr51), [CG11155](http://flybase.bio.indiana.edu/.bin/fbidq.html?CG11155), [rst](http://flybase.bio.indiana.edu/.bin/fbidq.html?rst), [grh](http://flybase.bio.indiana.edu/.bin/fbidq.html?grh), [comm](http://flybase.bio.indiana.edu/.bin/fbidq.html?comm), [tyn](http://flybase.bio.indiana.edu/.bin/fbidq.html?tyn), [Cdk5alpha](http://flybase.bio.indiana.edu/.bin/fbidq.html?Cdk5alpha), [wg](http://flybase.bio.indiana.edu/.bin/fbidq.html?wg), [Lim3](http://flybase.bio.indiana.edu/.bin/fbidq.html?Lim3), [CadN](http://flybase.bio.indiana.edu/.bin/fbidq.html?CadN), [Aplip1](http://flybase.bio.indiana.edu/.bin/fbidq.html?Aplip1), [E(Pc)](http://flybase.bio.indiana.edu/.bin/fbidq.html?E(Pc)), [retn](http://flybase.bio.indiana.edu/.bin/fbidq.html?retn), [E(bx)](http://flybase.bio.indiana.edu/.bin/fbidq.html?E(bx)), [en](http://flybase.bio.indiana.edu/.bin/fbidq.html?en), [Snap](http://flybase.bio.indiana.edu/.bin/fbidq.html?Snap), [trn](http://flybase.bio.indiana.edu/.bin/fbidq.html?trn), [Pitslre](http://flybase.bio.indiana.edu/.bin/fbidq.html?Pitslre), [CG33960](http://flybase.bio.indiana.edu/.bin/fbidq.html?CG33960), [Ct](http://flybase.bio.indiana.edu/.bin/fbidq.html?Ct) |
| [cell projection organization](http://amigo.geneontology.org/cgi-bin/amigo/go.cgi?view=details&query=GO:0030030) | 163 of 1594 genes, 10.2% | 484 of 7634 genes, 6.3% | 2.12e-10 | 0.00% | 0.00 | [fz2](http://flybase.bio.indiana.edu/.bin/fbidq.html?fz2), [Sh](http://flybase.bio.indiana.edu/.bin/fbidq.html?Sh), [acj6](http://flybase.bio.indiana.edu/.bin/fbidq.html?acj6), [jing](http://flybase.bio.indiana.edu/.bin/fbidq.html?jing), [dock](http://flybase.bio.indiana.edu/.bin/fbidq.html?dock), [CG42256](http://flybase.bio.indiana.edu/.bin/fbidq.html?CG42256), [CG3703](http://flybase.bio.indiana.edu/.bin/fbidq.html?CG3703), [ato](http://flybase.bio.indiana.edu/.bin/fbidq.html?ato), [ap](http://flybase.bio.indiana.edu/.bin/fbidq.html?ap), [Iswi](http://flybase.bio.indiana.edu/.bin/fbidq.html?Iswi), [Dg](http://flybase.bio.indiana.edu/.bin/fbidq.html?Dg), [Trim9](http://flybase.bio.indiana.edu/.bin/fbidq.html?Trim9), [CG7154](http://flybase.bio.indiana.edu/.bin/fbidq.html?CG7154), [robo3](http://flybase.bio.indiana.edu/.bin/fbidq.html?robo3), [tok](http://flybase.bio.indiana.edu/.bin/fbidq.html?tok), [Ptp99A](http://flybase.bio.indiana.edu/.bin/fbidq.html?Ptp99A), [lola](http://flybase.bio.indiana.edu/.bin/fbidq.html?lola), [unc-104](http://flybase.bio.indiana.edu/.bin/fbidq.html?unc-104), [chif](http://flybase.bio.indiana.edu/.bin/fbidq.html?chif), [NetB](http://flybase.bio.indiana.edu/.bin/fbidq.html?NetB), [p130CAS](http://flybase.bio.indiana.edu/.bin/fbidq.html?p130CAS), [Tango10](http://flybase.bio.indiana.edu/.bin/fbidq.html?Tango10), [Oseg4](http://flybase.bio.indiana.edu/.bin/fbidq.html?Oseg4), [Appl](http://flybase.bio.indiana.edu/.bin/fbidq.html?Appl), [metro](http://flybase.bio.indiana.edu/.bin/fbidq.html?metro), [CG32137](http://flybase.bio.indiana.edu/.bin/fbidq.html?CG32137), [Nrk](http://flybase.bio.indiana.edu/.bin/fbidq.html?Nrk), [CG4328](http://flybase.bio.indiana.edu/.bin/fbidq.html?CG4328), [Galpha49B](http://flybase.bio.indiana.edu/.bin/fbidq.html?Galpha49B), [Lar](http://flybase.bio.indiana.edu/.bin/fbidq.html?Lar), [Taf4](http://flybase.bio.indiana.edu/.bin/fbidq.html?Taf4), [CG10249](http://flybase.bio.indiana.edu/.bin/fbidq.html?CG10249), [scrib](http://flybase.bio.indiana.edu/.bin/fbidq.html?scrib), [not](http://flybase.bio.indiana.edu/.bin/fbidq.html?not), [rut](http://flybase.bio.indiana.edu/.bin/fbidq.html?rut),[nvy](http://flybase.bio.indiana.edu/.bin/fbidq.html?nvy), [Nrg](http://flybase.bio.indiana.edu/.bin/fbidq.html?Nrg), [gogo](http://flybase.bio.indiana.edu/.bin/fbidq.html?gogo), [jeb](http://flybase.bio.indiana.edu/.bin/fbidq.html?jeb), [GluClalpha](http://flybase.bio.indiana.edu/.bin/fbidq.html?GluClalpha), [Actbeta](http://flybase.bio.indiana.edu/.bin/fbidq.html?Actbeta), [Sema-1a](http://flybase.bio.indiana.edu/.bin/fbidq.html?Sema-1a), [beat-Ib](http://flybase.bio.indiana.edu/.bin/fbidq.html?beat-Ib), [TBPH](http://flybase.bio.indiana.edu/.bin/fbidq.html?TBPH), [otk](http://flybase.bio.indiana.edu/.bin/fbidq.html?otk), [CG34400](http://flybase.bio.indiana.edu/.bin/fbidq.html?CG34400), [Ulp1](http://flybase.bio.indiana.edu/.bin/fbidq.html?Ulp1), [ena](http://flybase.bio.indiana.edu/.bin/fbidq.html?ena), [dsh](http://flybase.bio.indiana.edu/.bin/fbidq.html?dsh), [Fas2](http://flybase.bio.indiana.edu/.bin/fbidq.html?Fas2), [Nf-YC](http://flybase.bio.indiana.edu/.bin/fbidq.html?Nf-YC), [fz](http://flybase.bio.indiana.edu/.bin/fbidq.html?fz), [robo](http://flybase.bio.indiana.edu/.bin/fbidq.html?robo), [lea](http://flybase.bio.indiana.edu/.bin/fbidq.html?lea), [bsk](http://flybase.bio.indiana.edu/.bin/fbidq.html?bsk), [brm](http://flybase.bio.indiana.edu/.bin/fbidq.html?brm), [beat-Ia](http://flybase.bio.indiana.edu/.bin/fbidq.html?beat-Ia), [kat-60L1](http://flybase.bio.indiana.edu/.bin/fbidq.html?kat-60L1), [ft](http://flybase.bio.indiana.edu/.bin/fbidq.html?ft), [RhoGAPp190](http://flybase.bio.indiana.edu/.bin/fbidq.html?RhoGAPp190), [plexB](http://flybase.bio.indiana.edu/.bin/fbidq.html?plexB), [Klp64D](http://flybase.bio.indiana.edu/.bin/fbidq.html?Klp64D), [grn](http://flybase.bio.indiana.edu/.bin/fbidq.html?grn), [DAAM](http://flybase.bio.indiana.edu/.bin/fbidq.html?DAAM), [mew](http://flybase.bio.indiana.edu/.bin/fbidq.html?mew), [Lis-1](http://flybase.bio.indiana.edu/.bin/fbidq.html?Lis-1), [Brf](http://flybase.bio.indiana.edu/.bin/fbidq.html?Brf), [ems](http://flybase.bio.indiana.edu/.bin/fbidq.html?ems), [pros](http://flybase.bio.indiana.edu/.bin/fbidq.html?pros), [tup](http://flybase.bio.indiana.edu/.bin/fbidq.html?tup), [trio](http://flybase.bio.indiana.edu/.bin/fbidq.html?trio),[CG1463](http://flybase.bio.indiana.edu/.bin/fbidq.html?CG1463), [daw](http://flybase.bio.indiana.edu/.bin/fbidq.html?daw), [Wnt4](http://flybase.bio.indiana.edu/.bin/fbidq.html?Wnt4), [rictor](http://flybase.bio.indiana.edu/.bin/fbidq.html?rictor), [Oseg6](http://flybase.bio.indiana.edu/.bin/fbidq.html?Oseg6), [Apc](http://flybase.bio.indiana.edu/.bin/fbidq.html?Apc), [sim](http://flybase.bio.indiana.edu/.bin/fbidq.html?sim), [d4](http://flybase.bio.indiana.edu/.bin/fbidq.html?d4), [CaMKII](http://flybase.bio.indiana.edu/.bin/fbidq.html?CaMKII), [gro](http://flybase.bio.indiana.edu/.bin/fbidq.html?gro), [nerfin-1](http://flybase.bio.indiana.edu/.bin/fbidq.html?nerfin-1), [bchs](http://flybase.bio.indiana.edu/.bin/fbidq.html?bchs), [wnd](http://flybase.bio.indiana.edu/.bin/fbidq.html?wnd), [trx](http://flybase.bio.indiana.edu/.bin/fbidq.html?trx), [Rpd3](http://flybase.bio.indiana.edu/.bin/fbidq.html?Rpd3), [pk](http://flybase.bio.indiana.edu/.bin/fbidq.html?pk), [dsx](http://flybase.bio.indiana.edu/.bin/fbidq.html?dsx), [chinmo](http://flybase.bio.indiana.edu/.bin/fbidq.html?chinmo), [CG14367](http://flybase.bio.indiana.edu/.bin/fbidq.html?CG14367), [unc-5](http://flybase.bio.indiana.edu/.bin/fbidq.html?unc-5), [Ssdp](http://flybase.bio.indiana.edu/.bin/fbidq.html?Ssdp), [CG10107](http://flybase.bio.indiana.edu/.bin/fbidq.html?CG10107), [sqz](http://flybase.bio.indiana.edu/.bin/fbidq.html?sqz), [fend](http://flybase.bio.indiana.edu/.bin/fbidq.html?fend), [pygo](http://flybase.bio.indiana.edu/.bin/fbidq.html?pygo), [HLHm7](http://flybase.bio.indiana.edu/.bin/fbidq.html?HLHm7), [tow](http://flybase.bio.indiana.edu/.bin/fbidq.html?tow), [wor](http://flybase.bio.indiana.edu/.bin/fbidq.html?wor), [vvl](http://flybase.bio.indiana.edu/.bin/fbidq.html?vvl), [Pten](http://flybase.bio.indiana.edu/.bin/fbidq.html?Pten), [Ptp69D](http://flybase.bio.indiana.edu/.bin/fbidq.html?Ptp69D), [gcm2](http://flybase.bio.indiana.edu/.bin/fbidq.html?gcm2), [Myo10A](http://flybase.bio.indiana.edu/.bin/fbidq.html?Myo10A), [hts](http://flybase.bio.indiana.edu/.bin/fbidq.html?hts), [mp](http://flybase.bio.indiana.edu/.bin/fbidq.html?mp), [gcm](http://flybase.bio.indiana.edu/.bin/fbidq.html?gcm), [seq](http://flybase.bio.indiana.edu/.bin/fbidq.html?seq),[CG5890](http://flybase.bio.indiana.edu/.bin/fbidq.html?CG5890), [ovo](http://flybase.bio.indiana.edu/.bin/fbidq.html?ovo), [CG32685](http://flybase.bio.indiana.edu/.bin/fbidq.html?CG32685), [CG4893](http://flybase.bio.indiana.edu/.bin/fbidq.html?CG4893), [ds](http://flybase.bio.indiana.edu/.bin/fbidq.html?ds), [CG9098](http://flybase.bio.indiana.edu/.bin/fbidq.html?CG9098), [Alk](http://flybase.bio.indiana.edu/.bin/fbidq.html?Alk), [Sin3A](http://flybase.bio.indiana.edu/.bin/fbidq.html?Sin3A), [jumu](http://flybase.bio.indiana.edu/.bin/fbidq.html?jumu), [Smox](http://flybase.bio.indiana.edu/.bin/fbidq.html?Smox), [CG31475](http://flybase.bio.indiana.edu/.bin/fbidq.html?CG31475), [beat-Ic](http://flybase.bio.indiana.edu/.bin/fbidq.html?beat-Ic), [fd59A](http://flybase.bio.indiana.edu/.bin/fbidq.html?fd59A), [run](http://flybase.bio.indiana.edu/.bin/fbidq.html?run), [brat](http://flybase.bio.indiana.edu/.bin/fbidq.html?brat), [ko](http://flybase.bio.indiana.edu/.bin/fbidq.html?ko), [Kap3](http://flybase.bio.indiana.edu/.bin/fbidq.html?Kap3), [Eip71CD](http://flybase.bio.indiana.edu/.bin/fbidq.html?Eip71CD), [stan](http://flybase.bio.indiana.edu/.bin/fbidq.html?stan), [CG12424](http://flybase.bio.indiana.edu/.bin/fbidq.html?CG12424), [tutl](http://flybase.bio.indiana.edu/.bin/fbidq.html?tutl), [futsch](http://flybase.bio.indiana.edu/.bin/fbidq.html?futsch), [Ptx1](http://flybase.bio.indiana.edu/.bin/fbidq.html?Ptx1), [gukh](http://flybase.bio.indiana.edu/.bin/fbidq.html?gukh), [chm](http://flybase.bio.indiana.edu/.bin/fbidq.html?chm), [sna](http://flybase.bio.indiana.edu/.bin/fbidq.html?sna), [Wnt5](http://flybase.bio.indiana.edu/.bin/fbidq.html?Wnt5), [beat-IIa](http://flybase.bio.indiana.edu/.bin/fbidq.html?beat-IIa), [sm](http://flybase.bio.indiana.edu/.bin/fbidq.html?sm), [dac](http://flybase.bio.indiana.edu/.bin/fbidq.html?dac), [Cdk5](http://flybase.bio.indiana.edu/.bin/fbidq.html?Cdk5), [sif](http://flybase.bio.indiana.edu/.bin/fbidq.html?sif), [Cep135](http://flybase.bio.indiana.edu/.bin/fbidq.html?Cep135), [dom](http://flybase.bio.indiana.edu/.bin/fbidq.html?dom), [Sas-4](http://flybase.bio.indiana.edu/.bin/fbidq.html?Sas-4), [ssh](http://flybase.bio.indiana.edu/.bin/fbidq.html?ssh),[baz](http://flybase.bio.indiana.edu/.bin/fbidq.html?baz), [mle](http://flybase.bio.indiana.edu/.bin/fbidq.html?mle), [Hr51](http://flybase.bio.indiana.edu/.bin/fbidq.html?Hr51), [CG11155](http://flybase.bio.indiana.edu/.bin/fbidq.html?CG11155), [rst](http://flybase.bio.indiana.edu/.bin/fbidq.html?rst), [comm](http://flybase.bio.indiana.edu/.bin/fbidq.html?comm), [Cdk5alpha](http://flybase.bio.indiana.edu/.bin/fbidq.html?Cdk5alpha), [wg](http://flybase.bio.indiana.edu/.bin/fbidq.html?wg), [Lim3](http://flybase.bio.indiana.edu/.bin/fbidq.html?Lim3), [CadN](http://flybase.bio.indiana.edu/.bin/fbidq.html?CadN), [Aplip1](http://flybase.bio.indiana.edu/.bin/fbidq.html?Aplip1), [E(Pc)](http://flybase.bio.indiana.edu/.bin/fbidq.html?E(Pc)), [retn](http://flybase.bio.indiana.edu/.bin/fbidq.html?retn), [E(bx)](http://flybase.bio.indiana.edu/.bin/fbidq.html?E(bx)), [en](http://flybase.bio.indiana.edu/.bin/fbidq.html?en), [Snap](http://flybase.bio.indiana.edu/.bin/fbidq.html?Snap), [trn](http://flybase.bio.indiana.edu/.bin/fbidq.html?trn), [CG33960](http://flybase.bio.indiana.edu/.bin/fbidq.html?CG33960), [Ct](http://flybase.bio.indiana.edu/.bin/fbidq.html?Ct) |
| [regulation of cellular biosynthetic process](http://amigo.geneontology.org/cgi-bin/amigo/go.cgi?view=details&query=GO:0031326) | 214 of 1594 genes, 13.4% | 688 of 7634 genes, 9.0% | 2.53e-10 | 0.00% | 0.00 | [fz2](http://flybase.bio.indiana.edu/.bin/fbidq.html?fz2), [acj6](http://flybase.bio.indiana.edu/.bin/fbidq.html?acj6), [skd](http://flybase.bio.indiana.edu/.bin/fbidq.html?skd), [jing](http://flybase.bio.indiana.edu/.bin/fbidq.html?jing), [caup](http://flybase.bio.indiana.edu/.bin/fbidq.html?caup), [tll](http://flybase.bio.indiana.edu/.bin/fbidq.html?tll), [Camta](http://flybase.bio.indiana.edu/.bin/fbidq.html?Camta), [ato](http://flybase.bio.indiana.edu/.bin/fbidq.html?ato), [Ets65A](http://flybase.bio.indiana.edu/.bin/fbidq.html?Ets65A), [tna](http://flybase.bio.indiana.edu/.bin/fbidq.html?tna), [Sxl](http://flybase.bio.indiana.edu/.bin/fbidq.html?Sxl), [JIL-1](http://flybase.bio.indiana.edu/.bin/fbidq.html?JIL-1), [Spt6](http://flybase.bio.indiana.edu/.bin/fbidq.html?Spt6), [Iswi](http://flybase.bio.indiana.edu/.bin/fbidq.html?Iswi), [rn](http://flybase.bio.indiana.edu/.bin/fbidq.html?rn), [ap](http://flybase.bio.indiana.edu/.bin/fbidq.html?ap), [Nf1](http://flybase.bio.indiana.edu/.bin/fbidq.html?Nf1), [br](http://flybase.bio.indiana.edu/.bin/fbidq.html?br), [ksr](http://flybase.bio.indiana.edu/.bin/fbidq.html?ksr), [bi](http://flybase.bio.indiana.edu/.bin/fbidq.html?bi), [chn](http://flybase.bio.indiana.edu/.bin/fbidq.html?chn), [Gsc](http://flybase.bio.indiana.edu/.bin/fbidq.html?Gsc), [Hcf](http://flybase.bio.indiana.edu/.bin/fbidq.html?Hcf), [lola](http://flybase.bio.indiana.edu/.bin/fbidq.html?lola), [HLH4C](http://flybase.bio.indiana.edu/.bin/fbidq.html?HLH4C), [Pcl](http://flybase.bio.indiana.edu/.bin/fbidq.html?Pcl), [chif](http://flybase.bio.indiana.edu/.bin/fbidq.html?chif), [simj](http://flybase.bio.indiana.edu/.bin/fbidq.html?simj), [Awh](http://flybase.bio.indiana.edu/.bin/fbidq.html?Awh), [tap](http://flybase.bio.indiana.edu/.bin/fbidq.html?tap), [NC2alpha](http://flybase.bio.indiana.edu/.bin/fbidq.html?NC2alpha), [PHDP](http://flybase.bio.indiana.edu/.bin/fbidq.html?PHDP), [bab1](http://flybase.bio.indiana.edu/.bin/fbidq.html?bab1), [CG4328](http://flybase.bio.indiana.edu/.bin/fbidq.html?CG4328), [Snoo](http://flybase.bio.indiana.edu/.bin/fbidq.html?Snoo), [l(2)NC136](http://flybase.bio.indiana.edu/.bin/fbidq.html?l(2)NC136), [Taf4](http://flybase.bio.indiana.edu/.bin/fbidq.html?Taf4), [Su(var)3-3](http://flybase.bio.indiana.edu/.bin/fbidq.html?Su(var)3-3), [scrib](http://flybase.bio.indiana.edu/.bin/fbidq.html?scrib), [HGTX](http://flybase.bio.indiana.edu/.bin/fbidq.html?HGTX), [not](http://flybase.bio.indiana.edu/.bin/fbidq.html?not),[nvy](http://flybase.bio.indiana.edu/.bin/fbidq.html?nvy), [nub](http://flybase.bio.indiana.edu/.bin/fbidq.html?nub), [nab](http://flybase.bio.indiana.edu/.bin/fbidq.html?nab), [btd](http://flybase.bio.indiana.edu/.bin/fbidq.html?btd), [fd68A](http://flybase.bio.indiana.edu/.bin/fbidq.html?fd68A), [HLHmdelta](http://flybase.bio.indiana.edu/.bin/fbidq.html?HLHmdelta), [B-H1](http://flybase.bio.indiana.edu/.bin/fbidq.html?B-H1), [Nipped-A](http://flybase.bio.indiana.edu/.bin/fbidq.html?Nipped-A), [ATbp](http://flybase.bio.indiana.edu/.bin/fbidq.html?ATbp), [stau](http://flybase.bio.indiana.edu/.bin/fbidq.html?stau), [Sos](http://flybase.bio.indiana.edu/.bin/fbidq.html?Sos), [Fer2](http://flybase.bio.indiana.edu/.bin/fbidq.html?Fer2), [fd102C](http://flybase.bio.indiana.edu/.bin/fbidq.html?fd102C), [vg](http://flybase.bio.indiana.edu/.bin/fbidq.html?vg), [hth](http://flybase.bio.indiana.edu/.bin/fbidq.html?hth), [Nf-YC](http://flybase.bio.indiana.edu/.bin/fbidq.html?Nf-YC), [eya](http://flybase.bio.indiana.edu/.bin/fbidq.html?eya), [fz](http://flybase.bio.indiana.edu/.bin/fbidq.html?fz), [brm](http://flybase.bio.indiana.edu/.bin/fbidq.html?brm), [Vsx2](http://flybase.bio.indiana.edu/.bin/fbidq.html?Vsx2), [Hey](http://flybase.bio.indiana.edu/.bin/fbidq.html?Hey), [sno](http://flybase.bio.indiana.edu/.bin/fbidq.html?sno), [Sfmbt](http://flybase.bio.indiana.edu/.bin/fbidq.html?Sfmbt), [ara](http://flybase.bio.indiana.edu/.bin/fbidq.html?ara), [mam](http://flybase.bio.indiana.edu/.bin/fbidq.html?mam), [pan](http://flybase.bio.indiana.edu/.bin/fbidq.html?pan), [Dll](http://flybase.bio.indiana.edu/.bin/fbidq.html?Dll), [disco](http://flybase.bio.indiana.edu/.bin/fbidq.html?disco), [grn](http://flybase.bio.indiana.edu/.bin/fbidq.html?grn), [HLHm5](http://flybase.bio.indiana.edu/.bin/fbidq.html?HLHm5), [D12](http://flybase.bio.indiana.edu/.bin/fbidq.html?D12), [Lis-1](http://flybase.bio.indiana.edu/.bin/fbidq.html?Lis-1), [CycE](http://flybase.bio.indiana.edu/.bin/fbidq.html?CycE), [Brf](http://flybase.bio.indiana.edu/.bin/fbidq.html?Brf), [ems](http://flybase.bio.indiana.edu/.bin/fbidq.html?ems), [pros](http://flybase.bio.indiana.edu/.bin/fbidq.html?pros), [tup](http://flybase.bio.indiana.edu/.bin/fbidq.html?tup), [pdm2](http://flybase.bio.indiana.edu/.bin/fbidq.html?pdm2), [Gug](http://flybase.bio.indiana.edu/.bin/fbidq.html?Gug), [pad](http://flybase.bio.indiana.edu/.bin/fbidq.html?pad), [rgr](http://flybase.bio.indiana.edu/.bin/fbidq.html?rgr),[orb2](http://flybase.bio.indiana.edu/.bin/fbidq.html?orb2), [Wnt4](http://flybase.bio.indiana.edu/.bin/fbidq.html?Wnt4), [Rx](http://flybase.bio.indiana.edu/.bin/fbidq.html?Rx), [MED1](http://flybase.bio.indiana.edu/.bin/fbidq.html?MED1), [salr](http://flybase.bio.indiana.edu/.bin/fbidq.html?salr), [wdn](http://flybase.bio.indiana.edu/.bin/fbidq.html?wdn), [sim](http://flybase.bio.indiana.edu/.bin/fbidq.html?sim), [d4](http://flybase.bio.indiana.edu/.bin/fbidq.html?d4), [dan](http://flybase.bio.indiana.edu/.bin/fbidq.html?dan), [gro](http://flybase.bio.indiana.edu/.bin/fbidq.html?gro), [nerfin-1](http://flybase.bio.indiana.edu/.bin/fbidq.html?nerfin-1), [bin3](http://flybase.bio.indiana.edu/.bin/fbidq.html?bin3), [trx](http://flybase.bio.indiana.edu/.bin/fbidq.html?trx), [Rpd3](http://flybase.bio.indiana.edu/.bin/fbidq.html?Rpd3), [dsx](http://flybase.bio.indiana.edu/.bin/fbidq.html?dsx), [MED25](http://flybase.bio.indiana.edu/.bin/fbidq.html?MED25), [tyf](http://flybase.bio.indiana.edu/.bin/fbidq.html?tyf), [Ssdp](http://flybase.bio.indiana.edu/.bin/fbidq.html?Ssdp), [vfl](http://flybase.bio.indiana.edu/.bin/fbidq.html?vfl), [sqz](http://flybase.bio.indiana.edu/.bin/fbidq.html?sqz), [CG32105](http://flybase.bio.indiana.edu/.bin/fbidq.html?CG32105), [Fer3](http://flybase.bio.indiana.edu/.bin/fbidq.html?Fer3), [aret](http://flybase.bio.indiana.edu/.bin/fbidq.html?aret), [pygo](http://flybase.bio.indiana.edu/.bin/fbidq.html?pygo), [dalao](http://flybase.bio.indiana.edu/.bin/fbidq.html?dalao), [HLHm7](http://flybase.bio.indiana.edu/.bin/fbidq.html?HLHm7), [z](http://flybase.bio.indiana.edu/.bin/fbidq.html?z), [HLHmgamma](http://flybase.bio.indiana.edu/.bin/fbidq.html?HLHmgamma), [Sox21b](http://flybase.bio.indiana.edu/.bin/fbidq.html?Sox21b), [sr](http://flybase.bio.indiana.edu/.bin/fbidq.html?sr), [Lim1](http://flybase.bio.indiana.edu/.bin/fbidq.html?Lim1), [tld](http://flybase.bio.indiana.edu/.bin/fbidq.html?tld), [danr](http://flybase.bio.indiana.edu/.bin/fbidq.html?danr), [wor](http://flybase.bio.indiana.edu/.bin/fbidq.html?wor), [vvl](http://flybase.bio.indiana.edu/.bin/fbidq.html?vvl), [wda](http://flybase.bio.indiana.edu/.bin/fbidq.html?wda), [sgg](http://flybase.bio.indiana.edu/.bin/fbidq.html?sgg), [MED14](http://flybase.bio.indiana.edu/.bin/fbidq.html?MED14), [gcm2](http://flybase.bio.indiana.edu/.bin/fbidq.html?gcm2), [Eip93F](http://flybase.bio.indiana.edu/.bin/fbidq.html?Eip93F),[gcm](http://flybase.bio.indiana.edu/.bin/fbidq.html?gcm), [seq](http://flybase.bio.indiana.edu/.bin/fbidq.html?seq), [Dip3](http://flybase.bio.indiana.edu/.bin/fbidq.html?Dip3), [Aef1](http://flybase.bio.indiana.edu/.bin/fbidq.html?Aef1), [HDAC4](http://flybase.bio.indiana.edu/.bin/fbidq.html?HDAC4), [can](http://flybase.bio.indiana.edu/.bin/fbidq.html?can), [elk](http://flybase.bio.indiana.edu/.bin/fbidq.html?elk), [Hsp67Bc](http://flybase.bio.indiana.edu/.bin/fbidq.html?Hsp67Bc), [phl](http://flybase.bio.indiana.edu/.bin/fbidq.html?phl), [ase](http://flybase.bio.indiana.edu/.bin/fbidq.html?ase), [CG13253](http://flybase.bio.indiana.edu/.bin/fbidq.html?CG13253), [ovo](http://flybase.bio.indiana.edu/.bin/fbidq.html?ovo), [scro](http://flybase.bio.indiana.edu/.bin/fbidq.html?scro), [pog](http://flybase.bio.indiana.edu/.bin/fbidq.html?pog), [Dsp1](http://flybase.bio.indiana.edu/.bin/fbidq.html?Dsp1), [CG14216](http://flybase.bio.indiana.edu/.bin/fbidq.html?CG14216), [Pdp1](http://flybase.bio.indiana.edu/.bin/fbidq.html?Pdp1), [Oli](http://flybase.bio.indiana.edu/.bin/fbidq.html?Oli), [Nelf-A](http://flybase.bio.indiana.edu/.bin/fbidq.html?Nelf-A), [GATAd](http://flybase.bio.indiana.edu/.bin/fbidq.html?GATAd), [Poxn](http://flybase.bio.indiana.edu/.bin/fbidq.html?Poxn), [CG15376](http://flybase.bio.indiana.edu/.bin/fbidq.html?CG15376), [Sin3A](http://flybase.bio.indiana.edu/.bin/fbidq.html?Sin3A), [mip120](http://flybase.bio.indiana.edu/.bin/fbidq.html?mip120), [casp](http://flybase.bio.indiana.edu/.bin/fbidq.html?casp), [jumu](http://flybase.bio.indiana.edu/.bin/fbidq.html?jumu), [Smox](http://flybase.bio.indiana.edu/.bin/fbidq.html?Smox), [Usp7](http://flybase.bio.indiana.edu/.bin/fbidq.html?Usp7), [ush](http://flybase.bio.indiana.edu/.bin/fbidq.html?ush), [fd59A](http://flybase.bio.indiana.edu/.bin/fbidq.html?fd59A), [CG10336](http://flybase.bio.indiana.edu/.bin/fbidq.html?CG10336), [Gap1](http://flybase.bio.indiana.edu/.bin/fbidq.html?Gap1), [run](http://flybase.bio.indiana.edu/.bin/fbidq.html?run), [FBX011](http://flybase.bio.indiana.edu/.bin/fbidq.html?FBX011), [brat](http://flybase.bio.indiana.edu/.bin/fbidq.html?brat),[Antp](http://flybase.bio.indiana.edu/.bin/fbidq.html?Antp), [tara](http://flybase.bio.indiana.edu/.bin/fbidq.html?tara), [Alh](http://flybase.bio.indiana.edu/.bin/fbidq.html?Alh), [Hel89B](http://flybase.bio.indiana.edu/.bin/fbidq.html?Hel89B), [Sox102F](http://flybase.bio.indiana.edu/.bin/fbidq.html?Sox102F), [egg](http://flybase.bio.indiana.edu/.bin/fbidq.html?egg), [l(3)neo38](http://flybase.bio.indiana.edu/.bin/fbidq.html?l(3)neo38), [koko](http://flybase.bio.indiana.edu/.bin/fbidq.html?koko), [Ptth](http://flybase.bio.indiana.edu/.bin/fbidq.html?Ptth), [Ast-C](http://flybase.bio.indiana.edu/.bin/fbidq.html?Ast-C), [Sp1](http://flybase.bio.indiana.edu/.bin/fbidq.html?Sp1), [Ptx1](http://flybase.bio.indiana.edu/.bin/fbidq.html?Ptx1), [ph-p](http://flybase.bio.indiana.edu/.bin/fbidq.html?ph-p), [Ubx](http://flybase.bio.indiana.edu/.bin/fbidq.html?Ubx), [onecut](http://flybase.bio.indiana.edu/.bin/fbidq.html?onecut), [CG7757](http://flybase.bio.indiana.edu/.bin/fbidq.html?CG7757), [chm](http://flybase.bio.indiana.edu/.bin/fbidq.html?chm), [sna](http://flybase.bio.indiana.edu/.bin/fbidq.html?sna), [Wnt5](http://flybase.bio.indiana.edu/.bin/fbidq.html?Wnt5), [Taf1](http://flybase.bio.indiana.edu/.bin/fbidq.html?Taf1), [hbn](http://flybase.bio.indiana.edu/.bin/fbidq.html?hbn), [MBD-like](http://flybase.bio.indiana.edu/.bin/fbidq.html?MBD-like), [tsh](http://flybase.bio.indiana.edu/.bin/fbidq.html?tsh), [esg](http://flybase.bio.indiana.edu/.bin/fbidq.html?esg), [e(y)3](http://flybase.bio.indiana.edu/.bin/fbidq.html?e(y)3), [CG9007](http://flybase.bio.indiana.edu/.bin/fbidq.html?CG9007), [oc](http://flybase.bio.indiana.edu/.bin/fbidq.html?oc), [spt4](http://flybase.bio.indiana.edu/.bin/fbidq.html?spt4), [CG32532](http://flybase.bio.indiana.edu/.bin/fbidq.html?CG32532), [MTA1-like](http://flybase.bio.indiana.edu/.bin/fbidq.html?MTA1-like), [gol](http://flybase.bio.indiana.edu/.bin/fbidq.html?gol), [ey](http://flybase.bio.indiana.edu/.bin/fbidq.html?ey), [mle](http://flybase.bio.indiana.edu/.bin/fbidq.html?mle), [Hr51](http://flybase.bio.indiana.edu/.bin/fbidq.html?Hr51), [Bgb](http://flybase.bio.indiana.edu/.bin/fbidq.html?Bgb), [l(1)sc](http://flybase.bio.indiana.edu/.bin/fbidq.html?l(1)sc), [B-H2](http://flybase.bio.indiana.edu/.bin/fbidq.html?B-H2),[grh](http://flybase.bio.indiana.edu/.bin/fbidq.html?grh), [Vsx1](http://flybase.bio.indiana.edu/.bin/fbidq.html?Vsx1), [wg](http://flybase.bio.indiana.edu/.bin/fbidq.html?wg), [toy](http://flybase.bio.indiana.edu/.bin/fbidq.html?toy), [fkh](http://flybase.bio.indiana.edu/.bin/fbidq.html?fkh), [Lim3](http://flybase.bio.indiana.edu/.bin/fbidq.html?Lim3), [crol](http://flybase.bio.indiana.edu/.bin/fbidq.html?crol), [sd](http://flybase.bio.indiana.edu/.bin/fbidq.html?sd), [retn](http://flybase.bio.indiana.edu/.bin/fbidq.html?retn), [en](http://flybase.bio.indiana.edu/.bin/fbidq.html?en), [fs(1)h](http://flybase.bio.indiana.edu/.bin/fbidq.html?fs(1)h), [rl](http://flybase.bio.indiana.edu/.bin/fbidq.html?rl), [CG11347](http://flybase.bio.indiana.edu/.bin/fbidq.html?CG11347), [tey](http://flybase.bio.indiana.edu/.bin/fbidq.html?tey), [CG11294](http://flybase.bio.indiana.edu/.bin/fbidq.html?CG11294), [hiw](http://flybase.bio.indiana.edu/.bin/fbidq.html?hiw), [cos](http://flybase.bio.indiana.edu/.bin/fbidq.html?cos), [Ct](http://flybase.bio.indiana.edu/.bin/fbidq.html?Ct), [Atf-2](http://flybase.bio.indiana.edu/.bin/fbidq.html?Atf-2), [dnr1](http://flybase.bio.indiana.edu/.bin/fbidq.html?dnr1) |
| [regulation of biosynthetic process](http://amigo.geneontology.org/cgi-bin/amigo/go.cgi?view=details&query=GO:0009889) | 214 of 1594 genes, 13.4% | 689 of 7634 genes, 9.0% | 2.99e-10 | 0.00% | 0.00 | [fz2](http://flybase.bio.indiana.edu/.bin/fbidq.html?fz2), [acj6](http://flybase.bio.indiana.edu/.bin/fbidq.html?acj6), [skd](http://flybase.bio.indiana.edu/.bin/fbidq.html?skd), [jing](http://flybase.bio.indiana.edu/.bin/fbidq.html?jing), [caup](http://flybase.bio.indiana.edu/.bin/fbidq.html?caup), [tll](http://flybase.bio.indiana.edu/.bin/fbidq.html?tll), [Camta](http://flybase.bio.indiana.edu/.bin/fbidq.html?Camta), [ato](http://flybase.bio.indiana.edu/.bin/fbidq.html?ato), [Ets65A](http://flybase.bio.indiana.edu/.bin/fbidq.html?Ets65A), [tna](http://flybase.bio.indiana.edu/.bin/fbidq.html?tna), [Sxl](http://flybase.bio.indiana.edu/.bin/fbidq.html?Sxl), [JIL-1](http://flybase.bio.indiana.edu/.bin/fbidq.html?JIL-1), [Spt6](http://flybase.bio.indiana.edu/.bin/fbidq.html?Spt6), [Iswi](http://flybase.bio.indiana.edu/.bin/fbidq.html?Iswi), [rn](http://flybase.bio.indiana.edu/.bin/fbidq.html?rn), [ap](http://flybase.bio.indiana.edu/.bin/fbidq.html?ap), [Nf1](http://flybase.bio.indiana.edu/.bin/fbidq.html?Nf1), [br](http://flybase.bio.indiana.edu/.bin/fbidq.html?br), [ksr](http://flybase.bio.indiana.edu/.bin/fbidq.html?ksr), [bi](http://flybase.bio.indiana.edu/.bin/fbidq.html?bi), [chn](http://flybase.bio.indiana.edu/.bin/fbidq.html?chn), [Gsc](http://flybase.bio.indiana.edu/.bin/fbidq.html?Gsc), [Hcf](http://flybase.bio.indiana.edu/.bin/fbidq.html?Hcf), [lola](http://flybase.bio.indiana.edu/.bin/fbidq.html?lola), [HLH4C](http://flybase.bio.indiana.edu/.bin/fbidq.html?HLH4C), [Pcl](http://flybase.bio.indiana.edu/.bin/fbidq.html?Pcl), [chif](http://flybase.bio.indiana.edu/.bin/fbidq.html?chif), [simj](http://flybase.bio.indiana.edu/.bin/fbidq.html?simj), [Awh](http://flybase.bio.indiana.edu/.bin/fbidq.html?Awh), [tap](http://flybase.bio.indiana.edu/.bin/fbidq.html?tap), [NC2alpha](http://flybase.bio.indiana.edu/.bin/fbidq.html?NC2alpha), [PHDP](http://flybase.bio.indiana.edu/.bin/fbidq.html?PHDP), [bab1](http://flybase.bio.indiana.edu/.bin/fbidq.html?bab1), [CG4328](http://flybase.bio.indiana.edu/.bin/fbidq.html?CG4328), [Snoo](http://flybase.bio.indiana.edu/.bin/fbidq.html?Snoo), [l(2)NC136](http://flybase.bio.indiana.edu/.bin/fbidq.html?l(2)NC136), [Taf4](http://flybase.bio.indiana.edu/.bin/fbidq.html?Taf4), [Su(var)3-3](http://flybase.bio.indiana.edu/.bin/fbidq.html?Su(var)3-3), [scrib](http://flybase.bio.indiana.edu/.bin/fbidq.html?scrib), [HGTX](http://flybase.bio.indiana.edu/.bin/fbidq.html?HGTX), [not](http://flybase.bio.indiana.edu/.bin/fbidq.html?not),[nvy](http://flybase.bio.indiana.edu/.bin/fbidq.html?nvy), [nub](http://flybase.bio.indiana.edu/.bin/fbidq.html?nub), [nab](http://flybase.bio.indiana.edu/.bin/fbidq.html?nab), [btd](http://flybase.bio.indiana.edu/.bin/fbidq.html?btd), [fd68A](http://flybase.bio.indiana.edu/.bin/fbidq.html?fd68A), [HLHmdelta](http://flybase.bio.indiana.edu/.bin/fbidq.html?HLHmdelta), [B-H1](http://flybase.bio.indiana.edu/.bin/fbidq.html?B-H1), [Nipped-A](http://flybase.bio.indiana.edu/.bin/fbidq.html?Nipped-A), [ATbp](http://flybase.bio.indiana.edu/.bin/fbidq.html?ATbp), [stau](http://flybase.bio.indiana.edu/.bin/fbidq.html?stau), [Sos](http://flybase.bio.indiana.edu/.bin/fbidq.html?Sos), [Fer2](http://flybase.bio.indiana.edu/.bin/fbidq.html?Fer2), [fd102C](http://flybase.bio.indiana.edu/.bin/fbidq.html?fd102C), [vg](http://flybase.bio.indiana.edu/.bin/fbidq.html?vg), [hth](http://flybase.bio.indiana.edu/.bin/fbidq.html?hth), [Nf-YC](http://flybase.bio.indiana.edu/.bin/fbidq.html?Nf-YC), [eya](http://flybase.bio.indiana.edu/.bin/fbidq.html?eya), [fz](http://flybase.bio.indiana.edu/.bin/fbidq.html?fz), [brm](http://flybase.bio.indiana.edu/.bin/fbidq.html?brm), [Vsx2](http://flybase.bio.indiana.edu/.bin/fbidq.html?Vsx2), [Hey](http://flybase.bio.indiana.edu/.bin/fbidq.html?Hey), [sno](http://flybase.bio.indiana.edu/.bin/fbidq.html?sno), [Sfmbt](http://flybase.bio.indiana.edu/.bin/fbidq.html?Sfmbt), [ara](http://flybase.bio.indiana.edu/.bin/fbidq.html?ara), [mam](http://flybase.bio.indiana.edu/.bin/fbidq.html?mam), [pan](http://flybase.bio.indiana.edu/.bin/fbidq.html?pan), [Dll](http://flybase.bio.indiana.edu/.bin/fbidq.html?Dll), [disco](http://flybase.bio.indiana.edu/.bin/fbidq.html?disco), [grn](http://flybase.bio.indiana.edu/.bin/fbidq.html?grn), [HLHm5](http://flybase.bio.indiana.edu/.bin/fbidq.html?HLHm5), [D12](http://flybase.bio.indiana.edu/.bin/fbidq.html?D12), [Lis-1](http://flybase.bio.indiana.edu/.bin/fbidq.html?Lis-1), [CycE](http://flybase.bio.indiana.edu/.bin/fbidq.html?CycE), [Brf](http://flybase.bio.indiana.edu/.bin/fbidq.html?Brf), [ems](http://flybase.bio.indiana.edu/.bin/fbidq.html?ems), [pros](http://flybase.bio.indiana.edu/.bin/fbidq.html?pros), [tup](http://flybase.bio.indiana.edu/.bin/fbidq.html?tup), [pdm2](http://flybase.bio.indiana.edu/.bin/fbidq.html?pdm2), [Gug](http://flybase.bio.indiana.edu/.bin/fbidq.html?Gug), [pad](http://flybase.bio.indiana.edu/.bin/fbidq.html?pad), [rgr](http://flybase.bio.indiana.edu/.bin/fbidq.html?rgr),[orb2](http://flybase.bio.indiana.edu/.bin/fbidq.html?orb2), [Wnt4](http://flybase.bio.indiana.edu/.bin/fbidq.html?Wnt4), [Rx](http://flybase.bio.indiana.edu/.bin/fbidq.html?Rx), [MED1](http://flybase.bio.indiana.edu/.bin/fbidq.html?MED1), [salr](http://flybase.bio.indiana.edu/.bin/fbidq.html?salr), [wdn](http://flybase.bio.indiana.edu/.bin/fbidq.html?wdn), [sim](http://flybase.bio.indiana.edu/.bin/fbidq.html?sim), [d4](http://flybase.bio.indiana.edu/.bin/fbidq.html?d4), [dan](http://flybase.bio.indiana.edu/.bin/fbidq.html?dan), [gro](http://flybase.bio.indiana.edu/.bin/fbidq.html?gro), [nerfin-1](http://flybase.bio.indiana.edu/.bin/fbidq.html?nerfin-1), [bin3](http://flybase.bio.indiana.edu/.bin/fbidq.html?bin3), [trx](http://flybase.bio.indiana.edu/.bin/fbidq.html?trx), [Rpd3](http://flybase.bio.indiana.edu/.bin/fbidq.html?Rpd3), [dsx](http://flybase.bio.indiana.edu/.bin/fbidq.html?dsx), [MED25](http://flybase.bio.indiana.edu/.bin/fbidq.html?MED25), [tyf](http://flybase.bio.indiana.edu/.bin/fbidq.html?tyf), [Ssdp](http://flybase.bio.indiana.edu/.bin/fbidq.html?Ssdp), [vfl](http://flybase.bio.indiana.edu/.bin/fbidq.html?vfl), [sqz](http://flybase.bio.indiana.edu/.bin/fbidq.html?sqz), [CG32105](http://flybase.bio.indiana.edu/.bin/fbidq.html?CG32105), [Fer3](http://flybase.bio.indiana.edu/.bin/fbidq.html?Fer3), [aret](http://flybase.bio.indiana.edu/.bin/fbidq.html?aret), [pygo](http://flybase.bio.indiana.edu/.bin/fbidq.html?pygo), [dalao](http://flybase.bio.indiana.edu/.bin/fbidq.html?dalao), [HLHm7](http://flybase.bio.indiana.edu/.bin/fbidq.html?HLHm7), [z](http://flybase.bio.indiana.edu/.bin/fbidq.html?z), [HLHmgamma](http://flybase.bio.indiana.edu/.bin/fbidq.html?HLHmgamma), [Sox21b](http://flybase.bio.indiana.edu/.bin/fbidq.html?Sox21b), [sr](http://flybase.bio.indiana.edu/.bin/fbidq.html?sr), [Lim1](http://flybase.bio.indiana.edu/.bin/fbidq.html?Lim1), [tld](http://flybase.bio.indiana.edu/.bin/fbidq.html?tld), [danr](http://flybase.bio.indiana.edu/.bin/fbidq.html?danr), [wor](http://flybase.bio.indiana.edu/.bin/fbidq.html?wor), [vvl](http://flybase.bio.indiana.edu/.bin/fbidq.html?vvl), [wda](http://flybase.bio.indiana.edu/.bin/fbidq.html?wda), [sgg](http://flybase.bio.indiana.edu/.bin/fbidq.html?sgg), [MED14](http://flybase.bio.indiana.edu/.bin/fbidq.html?MED14), [gcm2](http://flybase.bio.indiana.edu/.bin/fbidq.html?gcm2), [Eip93F](http://flybase.bio.indiana.edu/.bin/fbidq.html?Eip93F),[gcm](http://flybase.bio.indiana.edu/.bin/fbidq.html?gcm), [seq](http://flybase.bio.indiana.edu/.bin/fbidq.html?seq), [Dip3](http://flybase.bio.indiana.edu/.bin/fbidq.html?Dip3), [Aef1](http://flybase.bio.indiana.edu/.bin/fbidq.html?Aef1), [HDAC4](http://flybase.bio.indiana.edu/.bin/fbidq.html?HDAC4), [can](http://flybase.bio.indiana.edu/.bin/fbidq.html?can), [elk](http://flybase.bio.indiana.edu/.bin/fbidq.html?elk), [Hsp67Bc](http://flybase.bio.indiana.edu/.bin/fbidq.html?Hsp67Bc), [phl](http://flybase.bio.indiana.edu/.bin/fbidq.html?phl), [ase](http://flybase.bio.indiana.edu/.bin/fbidq.html?ase), [CG13253](http://flybase.bio.indiana.edu/.bin/fbidq.html?CG13253), [ovo](http://flybase.bio.indiana.edu/.bin/fbidq.html?ovo), [scro](http://flybase.bio.indiana.edu/.bin/fbidq.html?scro), [pog](http://flybase.bio.indiana.edu/.bin/fbidq.html?pog), [Dsp1](http://flybase.bio.indiana.edu/.bin/fbidq.html?Dsp1), [CG14216](http://flybase.bio.indiana.edu/.bin/fbidq.html?CG14216), [Pdp1](http://flybase.bio.indiana.edu/.bin/fbidq.html?Pdp1), [Oli](http://flybase.bio.indiana.edu/.bin/fbidq.html?Oli), [Nelf-A](http://flybase.bio.indiana.edu/.bin/fbidq.html?Nelf-A), [GATAd](http://flybase.bio.indiana.edu/.bin/fbidq.html?GATAd), [Poxn](http://flybase.bio.indiana.edu/.bin/fbidq.html?Poxn), [CG15376](http://flybase.bio.indiana.edu/.bin/fbidq.html?CG15376), [Sin3A](http://flybase.bio.indiana.edu/.bin/fbidq.html?Sin3A), [mip120](http://flybase.bio.indiana.edu/.bin/fbidq.html?mip120), [casp](http://flybase.bio.indiana.edu/.bin/fbidq.html?casp), [jumu](http://flybase.bio.indiana.edu/.bin/fbidq.html?jumu), [Smox](http://flybase.bio.indiana.edu/.bin/fbidq.html?Smox), [Usp7](http://flybase.bio.indiana.edu/.bin/fbidq.html?Usp7), [ush](http://flybase.bio.indiana.edu/.bin/fbidq.html?ush), [fd59A](http://flybase.bio.indiana.edu/.bin/fbidq.html?fd59A), [CG10336](http://flybase.bio.indiana.edu/.bin/fbidq.html?CG10336), [Gap1](http://flybase.bio.indiana.edu/.bin/fbidq.html?Gap1), [run](http://flybase.bio.indiana.edu/.bin/fbidq.html?run), [FBX011](http://flybase.bio.indiana.edu/.bin/fbidq.html?FBX011), [brat](http://flybase.bio.indiana.edu/.bin/fbidq.html?brat),[Antp](http://flybase.bio.indiana.edu/.bin/fbidq.html?Antp), [tara](http://flybase.bio.indiana.edu/.bin/fbidq.html?tara), [Alh](http://flybase.bio.indiana.edu/.bin/fbidq.html?Alh), [Hel89B](http://flybase.bio.indiana.edu/.bin/fbidq.html?Hel89B), [Sox102F](http://flybase.bio.indiana.edu/.bin/fbidq.html?Sox102F), [egg](http://flybase.bio.indiana.edu/.bin/fbidq.html?egg), [l(3)neo38](http://flybase.bio.indiana.edu/.bin/fbidq.html?l(3)neo38), [koko](http://flybase.bio.indiana.edu/.bin/fbidq.html?koko), [Ptth](http://flybase.bio.indiana.edu/.bin/fbidq.html?Ptth), [Ast-C](http://flybase.bio.indiana.edu/.bin/fbidq.html?Ast-C), [Sp1](http://flybase.bio.indiana.edu/.bin/fbidq.html?Sp1), [Ptx1](http://flybase.bio.indiana.edu/.bin/fbidq.html?Ptx1), [ph-p](http://flybase.bio.indiana.edu/.bin/fbidq.html?ph-p), [Ubx](http://flybase.bio.indiana.edu/.bin/fbidq.html?Ubx), [onecut](http://flybase.bio.indiana.edu/.bin/fbidq.html?onecut), [CG7757](http://flybase.bio.indiana.edu/.bin/fbidq.html?CG7757), [chm](http://flybase.bio.indiana.edu/.bin/fbidq.html?chm), [sna](http://flybase.bio.indiana.edu/.bin/fbidq.html?sna), [Wnt5](http://flybase.bio.indiana.edu/.bin/fbidq.html?Wnt5), [Taf1](http://flybase.bio.indiana.edu/.bin/fbidq.html?Taf1), [hbn](http://flybase.bio.indiana.edu/.bin/fbidq.html?hbn), [MBD-like](http://flybase.bio.indiana.edu/.bin/fbidq.html?MBD-like), [tsh](http://flybase.bio.indiana.edu/.bin/fbidq.html?tsh), [esg](http://flybase.bio.indiana.edu/.bin/fbidq.html?esg), [e(y)3](http://flybase.bio.indiana.edu/.bin/fbidq.html?e(y)3), [CG9007](http://flybase.bio.indiana.edu/.bin/fbidq.html?CG9007), [oc](http://flybase.bio.indiana.edu/.bin/fbidq.html?oc), [spt4](http://flybase.bio.indiana.edu/.bin/fbidq.html?spt4), [CG32532](http://flybase.bio.indiana.edu/.bin/fbidq.html?CG32532), [MTA1-like](http://flybase.bio.indiana.edu/.bin/fbidq.html?MTA1-like), [gol](http://flybase.bio.indiana.edu/.bin/fbidq.html?gol), [ey](http://flybase.bio.indiana.edu/.bin/fbidq.html?ey), [mle](http://flybase.bio.indiana.edu/.bin/fbidq.html?mle), [Hr51](http://flybase.bio.indiana.edu/.bin/fbidq.html?Hr51), [Bgb](http://flybase.bio.indiana.edu/.bin/fbidq.html?Bgb), [l(1)sc](http://flybase.bio.indiana.edu/.bin/fbidq.html?l(1)sc), [B-H2](http://flybase.bio.indiana.edu/.bin/fbidq.html?B-H2),[grh](http://flybase.bio.indiana.edu/.bin/fbidq.html?grh), [Vsx1](http://flybase.bio.indiana.edu/.bin/fbidq.html?Vsx1), [wg](http://flybase.bio.indiana.edu/.bin/fbidq.html?wg), [toy](http://flybase.bio.indiana.edu/.bin/fbidq.html?toy), [fkh](http://flybase.bio.indiana.edu/.bin/fbidq.html?fkh), [Lim3](http://flybase.bio.indiana.edu/.bin/fbidq.html?Lim3), [crol](http://flybase.bio.indiana.edu/.bin/fbidq.html?crol), [sd](http://flybase.bio.indiana.edu/.bin/fbidq.html?sd), [retn](http://flybase.bio.indiana.edu/.bin/fbidq.html?retn), [en](http://flybase.bio.indiana.edu/.bin/fbidq.html?en), [fs(1)h](http://flybase.bio.indiana.edu/.bin/fbidq.html?fs(1)h), [rl](http://flybase.bio.indiana.edu/.bin/fbidq.html?rl), [CG11347](http://flybase.bio.indiana.edu/.bin/fbidq.html?CG11347), [tey](http://flybase.bio.indiana.edu/.bin/fbidq.html?tey), [CG11294](http://flybase.bio.indiana.edu/.bin/fbidq.html?CG11294), [hiw](http://flybase.bio.indiana.edu/.bin/fbidq.html?hiw), [cos](http://flybase.bio.indiana.edu/.bin/fbidq.html?cos), [Ct](http://flybase.bio.indiana.edu/.bin/fbidq.html?Ct), [Atf-2](http://flybase.bio.indiana.edu/.bin/fbidq.html?Atf-2), [dnr1](http://flybase.bio.indiana.edu/.bin/fbidq.html?dnr1) |
| [neuron projection morphogenesis](http://amigo.geneontology.org/cgi-bin/amigo/go.cgi?view=details&query=GO:0048812) | 147 of 1594 genes, 9.2% | 427 of 7634 genes, 5.6% | 5.50e-10 | 0.00% | 0.00 | [fz2](http://flybase.bio.indiana.edu/.bin/fbidq.html?fz2), [Sh](http://flybase.bio.indiana.edu/.bin/fbidq.html?Sh), [acj6](http://flybase.bio.indiana.edu/.bin/fbidq.html?acj6), [jing](http://flybase.bio.indiana.edu/.bin/fbidq.html?jing), [dock](http://flybase.bio.indiana.edu/.bin/fbidq.html?dock), [CG42256](http://flybase.bio.indiana.edu/.bin/fbidq.html?CG42256), [CG3703](http://flybase.bio.indiana.edu/.bin/fbidq.html?CG3703), [ato](http://flybase.bio.indiana.edu/.bin/fbidq.html?ato), [ap](http://flybase.bio.indiana.edu/.bin/fbidq.html?ap), [Iswi](http://flybase.bio.indiana.edu/.bin/fbidq.html?Iswi), [Dg](http://flybase.bio.indiana.edu/.bin/fbidq.html?Dg), [Trim9](http://flybase.bio.indiana.edu/.bin/fbidq.html?Trim9), [CG7154](http://flybase.bio.indiana.edu/.bin/fbidq.html?CG7154), [robo3](http://flybase.bio.indiana.edu/.bin/fbidq.html?robo3), [tok](http://flybase.bio.indiana.edu/.bin/fbidq.html?tok), [Ptp99A](http://flybase.bio.indiana.edu/.bin/fbidq.html?Ptp99A), [lola](http://flybase.bio.indiana.edu/.bin/fbidq.html?lola), [unc-104](http://flybase.bio.indiana.edu/.bin/fbidq.html?unc-104), [chif](http://flybase.bio.indiana.edu/.bin/fbidq.html?chif), [NetB](http://flybase.bio.indiana.edu/.bin/fbidq.html?NetB), [p130CAS](http://flybase.bio.indiana.edu/.bin/fbidq.html?p130CAS), [Tango10](http://flybase.bio.indiana.edu/.bin/fbidq.html?Tango10), [Appl](http://flybase.bio.indiana.edu/.bin/fbidq.html?Appl), [metro](http://flybase.bio.indiana.edu/.bin/fbidq.html?metro), [CG32137](http://flybase.bio.indiana.edu/.bin/fbidq.html?CG32137), [Nrk](http://flybase.bio.indiana.edu/.bin/fbidq.html?Nrk), [CG4328](http://flybase.bio.indiana.edu/.bin/fbidq.html?CG4328), [Galpha49B](http://flybase.bio.indiana.edu/.bin/fbidq.html?Galpha49B), [Lar](http://flybase.bio.indiana.edu/.bin/fbidq.html?Lar), [Taf4](http://flybase.bio.indiana.edu/.bin/fbidq.html?Taf4), [CG10249](http://flybase.bio.indiana.edu/.bin/fbidq.html?CG10249), [not](http://flybase.bio.indiana.edu/.bin/fbidq.html?not), [rut](http://flybase.bio.indiana.edu/.bin/fbidq.html?rut), [nvy](http://flybase.bio.indiana.edu/.bin/fbidq.html?nvy), [Nrg](http://flybase.bio.indiana.edu/.bin/fbidq.html?Nrg), [gogo](http://flybase.bio.indiana.edu/.bin/fbidq.html?gogo),[jeb](http://flybase.bio.indiana.edu/.bin/fbidq.html?jeb), [GluClalpha](http://flybase.bio.indiana.edu/.bin/fbidq.html?GluClalpha), [Actbeta](http://flybase.bio.indiana.edu/.bin/fbidq.html?Actbeta), [Sema-1a](http://flybase.bio.indiana.edu/.bin/fbidq.html?Sema-1a), [beat-Ib](http://flybase.bio.indiana.edu/.bin/fbidq.html?beat-Ib), [TBPH](http://flybase.bio.indiana.edu/.bin/fbidq.html?TBPH), [otk](http://flybase.bio.indiana.edu/.bin/fbidq.html?otk), [CG34400](http://flybase.bio.indiana.edu/.bin/fbidq.html?CG34400), [Ulp1](http://flybase.bio.indiana.edu/.bin/fbidq.html?Ulp1), [ena](http://flybase.bio.indiana.edu/.bin/fbidq.html?ena), [dsh](http://flybase.bio.indiana.edu/.bin/fbidq.html?dsh), [Fas2](http://flybase.bio.indiana.edu/.bin/fbidq.html?Fas2), [Nf-YC](http://flybase.bio.indiana.edu/.bin/fbidq.html?Nf-YC), [fz](http://flybase.bio.indiana.edu/.bin/fbidq.html?fz), [robo](http://flybase.bio.indiana.edu/.bin/fbidq.html?robo), [lea](http://flybase.bio.indiana.edu/.bin/fbidq.html?lea), [bsk](http://flybase.bio.indiana.edu/.bin/fbidq.html?bsk), [brm](http://flybase.bio.indiana.edu/.bin/fbidq.html?brm), [beat-Ia](http://flybase.bio.indiana.edu/.bin/fbidq.html?beat-Ia), [kat-60L1](http://flybase.bio.indiana.edu/.bin/fbidq.html?kat-60L1), [RhoGAPp190](http://flybase.bio.indiana.edu/.bin/fbidq.html?RhoGAPp190), [plexB](http://flybase.bio.indiana.edu/.bin/fbidq.html?plexB), [Klp64D](http://flybase.bio.indiana.edu/.bin/fbidq.html?Klp64D), [grn](http://flybase.bio.indiana.edu/.bin/fbidq.html?grn), [DAAM](http://flybase.bio.indiana.edu/.bin/fbidq.html?DAAM), [mew](http://flybase.bio.indiana.edu/.bin/fbidq.html?mew), [Lis-1](http://flybase.bio.indiana.edu/.bin/fbidq.html?Lis-1), [Brf](http://flybase.bio.indiana.edu/.bin/fbidq.html?Brf), [ems](http://flybase.bio.indiana.edu/.bin/fbidq.html?ems), [pros](http://flybase.bio.indiana.edu/.bin/fbidq.html?pros), [tup](http://flybase.bio.indiana.edu/.bin/fbidq.html?tup), [trio](http://flybase.bio.indiana.edu/.bin/fbidq.html?trio), [CG1463](http://flybase.bio.indiana.edu/.bin/fbidq.html?CG1463), [daw](http://flybase.bio.indiana.edu/.bin/fbidq.html?daw), [Wnt4](http://flybase.bio.indiana.edu/.bin/fbidq.html?Wnt4),[rictor](http://flybase.bio.indiana.edu/.bin/fbidq.html?rictor), [Apc](http://flybase.bio.indiana.edu/.bin/fbidq.html?Apc), [sim](http://flybase.bio.indiana.edu/.bin/fbidq.html?sim), [d4](http://flybase.bio.indiana.edu/.bin/fbidq.html?d4), [gro](http://flybase.bio.indiana.edu/.bin/fbidq.html?gro), [nerfin-1](http://flybase.bio.indiana.edu/.bin/fbidq.html?nerfin-1), [bchs](http://flybase.bio.indiana.edu/.bin/fbidq.html?bchs), [wnd](http://flybase.bio.indiana.edu/.bin/fbidq.html?wnd), [trx](http://flybase.bio.indiana.edu/.bin/fbidq.html?trx), [Rpd3](http://flybase.bio.indiana.edu/.bin/fbidq.html?Rpd3), [dsx](http://flybase.bio.indiana.edu/.bin/fbidq.html?dsx), [chinmo](http://flybase.bio.indiana.edu/.bin/fbidq.html?chinmo), [unc-5](http://flybase.bio.indiana.edu/.bin/fbidq.html?unc-5), [Ssdp](http://flybase.bio.indiana.edu/.bin/fbidq.html?Ssdp), [CG10107](http://flybase.bio.indiana.edu/.bin/fbidq.html?CG10107), [sqz](http://flybase.bio.indiana.edu/.bin/fbidq.html?sqz), [fend](http://flybase.bio.indiana.edu/.bin/fbidq.html?fend), [pygo](http://flybase.bio.indiana.edu/.bin/fbidq.html?pygo), [HLHm7](http://flybase.bio.indiana.edu/.bin/fbidq.html?HLHm7), [wor](http://flybase.bio.indiana.edu/.bin/fbidq.html?wor), [vvl](http://flybase.bio.indiana.edu/.bin/fbidq.html?vvl), [Pten](http://flybase.bio.indiana.edu/.bin/fbidq.html?Pten), [Ptp69D](http://flybase.bio.indiana.edu/.bin/fbidq.html?Ptp69D), [gcm2](http://flybase.bio.indiana.edu/.bin/fbidq.html?gcm2), [hts](http://flybase.bio.indiana.edu/.bin/fbidq.html?hts), [mp](http://flybase.bio.indiana.edu/.bin/fbidq.html?mp), [gcm](http://flybase.bio.indiana.edu/.bin/fbidq.html?gcm), [seq](http://flybase.bio.indiana.edu/.bin/fbidq.html?seq), [CG5890](http://flybase.bio.indiana.edu/.bin/fbidq.html?CG5890), [CG32685](http://flybase.bio.indiana.edu/.bin/fbidq.html?CG32685), [CG4893](http://flybase.bio.indiana.edu/.bin/fbidq.html?CG4893), [CG9098](http://flybase.bio.indiana.edu/.bin/fbidq.html?CG9098), [Alk](http://flybase.bio.indiana.edu/.bin/fbidq.html?Alk), [Sin3A](http://flybase.bio.indiana.edu/.bin/fbidq.html?Sin3A), [jumu](http://flybase.bio.indiana.edu/.bin/fbidq.html?jumu), [Smox](http://flybase.bio.indiana.edu/.bin/fbidq.html?Smox),[CG31475](http://flybase.bio.indiana.edu/.bin/fbidq.html?CG31475), [beat-Ic](http://flybase.bio.indiana.edu/.bin/fbidq.html?beat-Ic), [fd59A](http://flybase.bio.indiana.edu/.bin/fbidq.html?fd59A), [run](http://flybase.bio.indiana.edu/.bin/fbidq.html?run), [brat](http://flybase.bio.indiana.edu/.bin/fbidq.html?brat), [ko](http://flybase.bio.indiana.edu/.bin/fbidq.html?ko), [Eip71CD](http://flybase.bio.indiana.edu/.bin/fbidq.html?Eip71CD), [stan](http://flybase.bio.indiana.edu/.bin/fbidq.html?stan), [CG12424](http://flybase.bio.indiana.edu/.bin/fbidq.html?CG12424), [tutl](http://flybase.bio.indiana.edu/.bin/fbidq.html?tutl), [futsch](http://flybase.bio.indiana.edu/.bin/fbidq.html?futsch), [Ptx1](http://flybase.bio.indiana.edu/.bin/fbidq.html?Ptx1), [gukh](http://flybase.bio.indiana.edu/.bin/fbidq.html?gukh), [chm](http://flybase.bio.indiana.edu/.bin/fbidq.html?chm), [sna](http://flybase.bio.indiana.edu/.bin/fbidq.html?sna), [Wnt5](http://flybase.bio.indiana.edu/.bin/fbidq.html?Wnt5), [beat-IIa](http://flybase.bio.indiana.edu/.bin/fbidq.html?beat-IIa), [sm](http://flybase.bio.indiana.edu/.bin/fbidq.html?sm), [dac](http://flybase.bio.indiana.edu/.bin/fbidq.html?dac), [Cdk5](http://flybase.bio.indiana.edu/.bin/fbidq.html?Cdk5), [sif](http://flybase.bio.indiana.edu/.bin/fbidq.html?sif), [dom](http://flybase.bio.indiana.edu/.bin/fbidq.html?dom), [ssh](http://flybase.bio.indiana.edu/.bin/fbidq.html?ssh), [mle](http://flybase.bio.indiana.edu/.bin/fbidq.html?mle), [Hr51](http://flybase.bio.indiana.edu/.bin/fbidq.html?Hr51), [CG11155](http://flybase.bio.indiana.edu/.bin/fbidq.html?CG11155), [rst](http://flybase.bio.indiana.edu/.bin/fbidq.html?rst), [comm](http://flybase.bio.indiana.edu/.bin/fbidq.html?comm), [Cdk5alpha](http://flybase.bio.indiana.edu/.bin/fbidq.html?Cdk5alpha), [Lim3](http://flybase.bio.indiana.edu/.bin/fbidq.html?Lim3), [CadN](http://flybase.bio.indiana.edu/.bin/fbidq.html?CadN), [Aplip1](http://flybase.bio.indiana.edu/.bin/fbidq.html?Aplip1), [E(Pc)](http://flybase.bio.indiana.edu/.bin/fbidq.html?E(Pc)), [retn](http://flybase.bio.indiana.edu/.bin/fbidq.html?retn), [E(bx)](http://flybase.bio.indiana.edu/.bin/fbidq.html?E(bx)), [en](http://flybase.bio.indiana.edu/.bin/fbidq.html?en), [Snap](http://flybase.bio.indiana.edu/.bin/fbidq.html?Snap),[trn](http://flybase.bio.indiana.edu/.bin/fbidq.html?trn), [CG33960](http://flybase.bio.indiana.edu/.bin/fbidq.html?CG33960), [Ct](http://flybase.bio.indiana.edu/.bin/fbidq.html?Ct) |
| [neuron projection development](http://amigo.geneontology.org/cgi-bin/amigo/go.cgi?view=details&query=GO:0031175) | 147 of 1594 genes, 9.2% | 428 of 7634 genes, 5.6% | 6.81e-10 | 0.00% | 0.00 | [fz2](http://flybase.bio.indiana.edu/.bin/fbidq.html?fz2), [Sh](http://flybase.bio.indiana.edu/.bin/fbidq.html?Sh), [acj6](http://flybase.bio.indiana.edu/.bin/fbidq.html?acj6), [jing](http://flybase.bio.indiana.edu/.bin/fbidq.html?jing), [dock](http://flybase.bio.indiana.edu/.bin/fbidq.html?dock), [CG42256](http://flybase.bio.indiana.edu/.bin/fbidq.html?CG42256), [CG3703](http://flybase.bio.indiana.edu/.bin/fbidq.html?CG3703), [ato](http://flybase.bio.indiana.edu/.bin/fbidq.html?ato), [ap](http://flybase.bio.indiana.edu/.bin/fbidq.html?ap), [Iswi](http://flybase.bio.indiana.edu/.bin/fbidq.html?Iswi), [Dg](http://flybase.bio.indiana.edu/.bin/fbidq.html?Dg), [Trim9](http://flybase.bio.indiana.edu/.bin/fbidq.html?Trim9), [CG7154](http://flybase.bio.indiana.edu/.bin/fbidq.html?CG7154), [robo3](http://flybase.bio.indiana.edu/.bin/fbidq.html?robo3), [tok](http://flybase.bio.indiana.edu/.bin/fbidq.html?tok), [Ptp99A](http://flybase.bio.indiana.edu/.bin/fbidq.html?Ptp99A), [lola](http://flybase.bio.indiana.edu/.bin/fbidq.html?lola), [unc-104](http://flybase.bio.indiana.edu/.bin/fbidq.html?unc-104), [chif](http://flybase.bio.indiana.edu/.bin/fbidq.html?chif), [NetB](http://flybase.bio.indiana.edu/.bin/fbidq.html?NetB), [p130CAS](http://flybase.bio.indiana.edu/.bin/fbidq.html?p130CAS), [Tango10](http://flybase.bio.indiana.edu/.bin/fbidq.html?Tango10), [Appl](http://flybase.bio.indiana.edu/.bin/fbidq.html?Appl), [metro](http://flybase.bio.indiana.edu/.bin/fbidq.html?metro), [CG32137](http://flybase.bio.indiana.edu/.bin/fbidq.html?CG32137), [Nrk](http://flybase.bio.indiana.edu/.bin/fbidq.html?Nrk), [CG4328](http://flybase.bio.indiana.edu/.bin/fbidq.html?CG4328), [Galpha49B](http://flybase.bio.indiana.edu/.bin/fbidq.html?Galpha49B), [Lar](http://flybase.bio.indiana.edu/.bin/fbidq.html?Lar), [Taf4](http://flybase.bio.indiana.edu/.bin/fbidq.html?Taf4), [CG10249](http://flybase.bio.indiana.edu/.bin/fbidq.html?CG10249), [not](http://flybase.bio.indiana.edu/.bin/fbidq.html?not), [rut](http://flybase.bio.indiana.edu/.bin/fbidq.html?rut), [nvy](http://flybase.bio.indiana.edu/.bin/fbidq.html?nvy), [Nrg](http://flybase.bio.indiana.edu/.bin/fbidq.html?Nrg), [gogo](http://flybase.bio.indiana.edu/.bin/fbidq.html?gogo),[jeb](http://flybase.bio.indiana.edu/.bin/fbidq.html?jeb), [GluClalpha](http://flybase.bio.indiana.edu/.bin/fbidq.html?GluClalpha), [Actbeta](http://flybase.bio.indiana.edu/.bin/fbidq.html?Actbeta), [Sema-1a](http://flybase.bio.indiana.edu/.bin/fbidq.html?Sema-1a), [beat-Ib](http://flybase.bio.indiana.edu/.bin/fbidq.html?beat-Ib), [TBPH](http://flybase.bio.indiana.edu/.bin/fbidq.html?TBPH), [otk](http://flybase.bio.indiana.edu/.bin/fbidq.html?otk), [CG34400](http://flybase.bio.indiana.edu/.bin/fbidq.html?CG34400), [Ulp1](http://flybase.bio.indiana.edu/.bin/fbidq.html?Ulp1), [ena](http://flybase.bio.indiana.edu/.bin/fbidq.html?ena), [dsh](http://flybase.bio.indiana.edu/.bin/fbidq.html?dsh), [Fas2](http://flybase.bio.indiana.edu/.bin/fbidq.html?Fas2), [Nf-YC](http://flybase.bio.indiana.edu/.bin/fbidq.html?Nf-YC), [fz](http://flybase.bio.indiana.edu/.bin/fbidq.html?fz), [robo](http://flybase.bio.indiana.edu/.bin/fbidq.html?robo), [lea](http://flybase.bio.indiana.edu/.bin/fbidq.html?lea), [bsk](http://flybase.bio.indiana.edu/.bin/fbidq.html?bsk), [brm](http://flybase.bio.indiana.edu/.bin/fbidq.html?brm), [beat-Ia](http://flybase.bio.indiana.edu/.bin/fbidq.html?beat-Ia), [kat-60L1](http://flybase.bio.indiana.edu/.bin/fbidq.html?kat-60L1), [RhoGAPp190](http://flybase.bio.indiana.edu/.bin/fbidq.html?RhoGAPp190), [plexB](http://flybase.bio.indiana.edu/.bin/fbidq.html?plexB), [Klp64D](http://flybase.bio.indiana.edu/.bin/fbidq.html?Klp64D), [grn](http://flybase.bio.indiana.edu/.bin/fbidq.html?grn), [DAAM](http://flybase.bio.indiana.edu/.bin/fbidq.html?DAAM), [mew](http://flybase.bio.indiana.edu/.bin/fbidq.html?mew), [Lis-1](http://flybase.bio.indiana.edu/.bin/fbidq.html?Lis-1), [Brf](http://flybase.bio.indiana.edu/.bin/fbidq.html?Brf), [ems](http://flybase.bio.indiana.edu/.bin/fbidq.html?ems), [pros](http://flybase.bio.indiana.edu/.bin/fbidq.html?pros), [tup](http://flybase.bio.indiana.edu/.bin/fbidq.html?tup), [trio](http://flybase.bio.indiana.edu/.bin/fbidq.html?trio), [CG1463](http://flybase.bio.indiana.edu/.bin/fbidq.html?CG1463), [daw](http://flybase.bio.indiana.edu/.bin/fbidq.html?daw), [Wnt4](http://flybase.bio.indiana.edu/.bin/fbidq.html?Wnt4),[rictor](http://flybase.bio.indiana.edu/.bin/fbidq.html?rictor), [Apc](http://flybase.bio.indiana.edu/.bin/fbidq.html?Apc), [sim](http://flybase.bio.indiana.edu/.bin/fbidq.html?sim), [d4](http://flybase.bio.indiana.edu/.bin/fbidq.html?d4), [gro](http://flybase.bio.indiana.edu/.bin/fbidq.html?gro), [nerfin-1](http://flybase.bio.indiana.edu/.bin/fbidq.html?nerfin-1), [bchs](http://flybase.bio.indiana.edu/.bin/fbidq.html?bchs), [wnd](http://flybase.bio.indiana.edu/.bin/fbidq.html?wnd), [trx](http://flybase.bio.indiana.edu/.bin/fbidq.html?trx), [Rpd3](http://flybase.bio.indiana.edu/.bin/fbidq.html?Rpd3), [dsx](http://flybase.bio.indiana.edu/.bin/fbidq.html?dsx), [chinmo](http://flybase.bio.indiana.edu/.bin/fbidq.html?chinmo), [unc-5](http://flybase.bio.indiana.edu/.bin/fbidq.html?unc-5), [Ssdp](http://flybase.bio.indiana.edu/.bin/fbidq.html?Ssdp), [CG10107](http://flybase.bio.indiana.edu/.bin/fbidq.html?CG10107), [sqz](http://flybase.bio.indiana.edu/.bin/fbidq.html?sqz), [fend](http://flybase.bio.indiana.edu/.bin/fbidq.html?fend), [pygo](http://flybase.bio.indiana.edu/.bin/fbidq.html?pygo), [HLHm7](http://flybase.bio.indiana.edu/.bin/fbidq.html?HLHm7), [wor](http://flybase.bio.indiana.edu/.bin/fbidq.html?wor), [vvl](http://flybase.bio.indiana.edu/.bin/fbidq.html?vvl), [Pten](http://flybase.bio.indiana.edu/.bin/fbidq.html?Pten), [Ptp69D](http://flybase.bio.indiana.edu/.bin/fbidq.html?Ptp69D), [gcm2](http://flybase.bio.indiana.edu/.bin/fbidq.html?gcm2), [hts](http://flybase.bio.indiana.edu/.bin/fbidq.html?hts), [mp](http://flybase.bio.indiana.edu/.bin/fbidq.html?mp), [gcm](http://flybase.bio.indiana.edu/.bin/fbidq.html?gcm), [seq](http://flybase.bio.indiana.edu/.bin/fbidq.html?seq), [CG5890](http://flybase.bio.indiana.edu/.bin/fbidq.html?CG5890), [CG32685](http://flybase.bio.indiana.edu/.bin/fbidq.html?CG32685), [CG4893](http://flybase.bio.indiana.edu/.bin/fbidq.html?CG4893), [CG9098](http://flybase.bio.indiana.edu/.bin/fbidq.html?CG9098), [Alk](http://flybase.bio.indiana.edu/.bin/fbidq.html?Alk), [Sin3A](http://flybase.bio.indiana.edu/.bin/fbidq.html?Sin3A), [jumu](http://flybase.bio.indiana.edu/.bin/fbidq.html?jumu), [Smox](http://flybase.bio.indiana.edu/.bin/fbidq.html?Smox),[CG31475](http://flybase.bio.indiana.edu/.bin/fbidq.html?CG31475), [beat-Ic](http://flybase.bio.indiana.edu/.bin/fbidq.html?beat-Ic), [fd59A](http://flybase.bio.indiana.edu/.bin/fbidq.html?fd59A), [run](http://flybase.bio.indiana.edu/.bin/fbidq.html?run), [brat](http://flybase.bio.indiana.edu/.bin/fbidq.html?brat), [ko](http://flybase.bio.indiana.edu/.bin/fbidq.html?ko), [Eip71CD](http://flybase.bio.indiana.edu/.bin/fbidq.html?Eip71CD), [stan](http://flybase.bio.indiana.edu/.bin/fbidq.html?stan), [CG12424](http://flybase.bio.indiana.edu/.bin/fbidq.html?CG12424), [tutl](http://flybase.bio.indiana.edu/.bin/fbidq.html?tutl), [futsch](http://flybase.bio.indiana.edu/.bin/fbidq.html?futsch), [Ptx1](http://flybase.bio.indiana.edu/.bin/fbidq.html?Ptx1), [gukh](http://flybase.bio.indiana.edu/.bin/fbidq.html?gukh), [chm](http://flybase.bio.indiana.edu/.bin/fbidq.html?chm), [sna](http://flybase.bio.indiana.edu/.bin/fbidq.html?sna), [Wnt5](http://flybase.bio.indiana.edu/.bin/fbidq.html?Wnt5), [beat-IIa](http://flybase.bio.indiana.edu/.bin/fbidq.html?beat-IIa), [sm](http://flybase.bio.indiana.edu/.bin/fbidq.html?sm), [dac](http://flybase.bio.indiana.edu/.bin/fbidq.html?dac), [Cdk5](http://flybase.bio.indiana.edu/.bin/fbidq.html?Cdk5), [sif](http://flybase.bio.indiana.edu/.bin/fbidq.html?sif), [dom](http://flybase.bio.indiana.edu/.bin/fbidq.html?dom), [ssh](http://flybase.bio.indiana.edu/.bin/fbidq.html?ssh), [mle](http://flybase.bio.indiana.edu/.bin/fbidq.html?mle), [Hr51](http://flybase.bio.indiana.edu/.bin/fbidq.html?Hr51), [CG11155](http://flybase.bio.indiana.edu/.bin/fbidq.html?CG11155), [rst](http://flybase.bio.indiana.edu/.bin/fbidq.html?rst), [comm](http://flybase.bio.indiana.edu/.bin/fbidq.html?comm), [Cdk5alpha](http://flybase.bio.indiana.edu/.bin/fbidq.html?Cdk5alpha), [Lim3](http://flybase.bio.indiana.edu/.bin/fbidq.html?Lim3), [CadN](http://flybase.bio.indiana.edu/.bin/fbidq.html?CadN), [Aplip1](http://flybase.bio.indiana.edu/.bin/fbidq.html?Aplip1), [E(Pc)](http://flybase.bio.indiana.edu/.bin/fbidq.html?E(Pc)), [retn](http://flybase.bio.indiana.edu/.bin/fbidq.html?retn), [E(bx)](http://flybase.bio.indiana.edu/.bin/fbidq.html?E(bx)), [en](http://flybase.bio.indiana.edu/.bin/fbidq.html?en), [Snap](http://flybase.bio.indiana.edu/.bin/fbidq.html?Snap),[trn](http://flybase.bio.indiana.edu/.bin/fbidq.html?trn), [CG33960](http://flybase.bio.indiana.edu/.bin/fbidq.html?CG33960), [Ct](http://flybase.bio.indiana.edu/.bin/fbidq.html?Ct) |
| [axonogenesis](http://amigo.geneontology.org/cgi-bin/amigo/go.cgi?view=details&query=GO:0007409) | 101 of 1594 genes, 6.3% | 259 of 7634 genes, 3.4% | 8.23e-10 | 0.00% | 0.00 | [fz2](http://flybase.bio.indiana.edu/.bin/fbidq.html?fz2), [Sh](http://flybase.bio.indiana.edu/.bin/fbidq.html?Sh), [acj6](http://flybase.bio.indiana.edu/.bin/fbidq.html?acj6), [vvl](http://flybase.bio.indiana.edu/.bin/fbidq.html?vvl), [jing](http://flybase.bio.indiana.edu/.bin/fbidq.html?jing), [dock](http://flybase.bio.indiana.edu/.bin/fbidq.html?dock), [Pten](http://flybase.bio.indiana.edu/.bin/fbidq.html?Pten), [Ptp69D](http://flybase.bio.indiana.edu/.bin/fbidq.html?Ptp69D), [hts](http://flybase.bio.indiana.edu/.bin/fbidq.html?hts), [mp](http://flybase.bio.indiana.edu/.bin/fbidq.html?mp), [ap](http://flybase.bio.indiana.edu/.bin/fbidq.html?ap), [Dg](http://flybase.bio.indiana.edu/.bin/fbidq.html?Dg), [seq](http://flybase.bio.indiana.edu/.bin/fbidq.html?seq), [gcm](http://flybase.bio.indiana.edu/.bin/fbidq.html?gcm), [Trim9](http://flybase.bio.indiana.edu/.bin/fbidq.html?Trim9), [robo3](http://flybase.bio.indiana.edu/.bin/fbidq.html?robo3), [tok](http://flybase.bio.indiana.edu/.bin/fbidq.html?tok), [Ptp99A](http://flybase.bio.indiana.edu/.bin/fbidq.html?Ptp99A), [lola](http://flybase.bio.indiana.edu/.bin/fbidq.html?lola), [unc-104](http://flybase.bio.indiana.edu/.bin/fbidq.html?unc-104), [chif](http://flybase.bio.indiana.edu/.bin/fbidq.html?chif), [NetB](http://flybase.bio.indiana.edu/.bin/fbidq.html?NetB), [p130CAS](http://flybase.bio.indiana.edu/.bin/fbidq.html?p130CAS), [Alk](http://flybase.bio.indiana.edu/.bin/fbidq.html?Alk), [Nrk](http://flybase.bio.indiana.edu/.bin/fbidq.html?Nrk), [Galpha49B](http://flybase.bio.indiana.edu/.bin/fbidq.html?Galpha49B), [Smox](http://flybase.bio.indiana.edu/.bin/fbidq.html?Smox), [Lar](http://flybase.bio.indiana.edu/.bin/fbidq.html?Lar), [not](http://flybase.bio.indiana.edu/.bin/fbidq.html?not), [rut](http://flybase.bio.indiana.edu/.bin/fbidq.html?rut), [nvy](http://flybase.bio.indiana.edu/.bin/fbidq.html?nvy), [Nrg](http://flybase.bio.indiana.edu/.bin/fbidq.html?Nrg), [gogo](http://flybase.bio.indiana.edu/.bin/fbidq.html?gogo), [fd59A](http://flybase.bio.indiana.edu/.bin/fbidq.html?fd59A), [beat-Ic](http://flybase.bio.indiana.edu/.bin/fbidq.html?beat-Ic), [run](http://flybase.bio.indiana.edu/.bin/fbidq.html?run), [jeb](http://flybase.bio.indiana.edu/.bin/fbidq.html?jeb), [brat](http://flybase.bio.indiana.edu/.bin/fbidq.html?brat), [ko](http://flybase.bio.indiana.edu/.bin/fbidq.html?ko), [Sema-1a](http://flybase.bio.indiana.edu/.bin/fbidq.html?Sema-1a), [beat-Ib](http://flybase.bio.indiana.edu/.bin/fbidq.html?beat-Ib),[otk](http://flybase.bio.indiana.edu/.bin/fbidq.html?otk), [stan](http://flybase.bio.indiana.edu/.bin/fbidq.html?stan), [CG12424](http://flybase.bio.indiana.edu/.bin/fbidq.html?CG12424), [CG34400](http://flybase.bio.indiana.edu/.bin/fbidq.html?CG34400), [Ulp1](http://flybase.bio.indiana.edu/.bin/fbidq.html?Ulp1), [tutl](http://flybase.bio.indiana.edu/.bin/fbidq.html?tutl), [ena](http://flybase.bio.indiana.edu/.bin/fbidq.html?ena), [dsh](http://flybase.bio.indiana.edu/.bin/fbidq.html?dsh), [Nf-YC](http://flybase.bio.indiana.edu/.bin/fbidq.html?Nf-YC), [Fas2](http://flybase.bio.indiana.edu/.bin/fbidq.html?Fas2), [fz](http://flybase.bio.indiana.edu/.bin/fbidq.html?fz), [robo](http://flybase.bio.indiana.edu/.bin/fbidq.html?robo), [lea](http://flybase.bio.indiana.edu/.bin/fbidq.html?lea), [bsk](http://flybase.bio.indiana.edu/.bin/fbidq.html?bsk), [brm](http://flybase.bio.indiana.edu/.bin/fbidq.html?brm), [futsch](http://flybase.bio.indiana.edu/.bin/fbidq.html?futsch), [beat-Ia](http://flybase.bio.indiana.edu/.bin/fbidq.html?beat-Ia), [gukh](http://flybase.bio.indiana.edu/.bin/fbidq.html?gukh), [RhoGAPp190](http://flybase.bio.indiana.edu/.bin/fbidq.html?RhoGAPp190), [plexB](http://flybase.bio.indiana.edu/.bin/fbidq.html?plexB), [Wnt5](http://flybase.bio.indiana.edu/.bin/fbidq.html?Wnt5), [Klp64D](http://flybase.bio.indiana.edu/.bin/fbidq.html?Klp64D), [beat-IIa](http://flybase.bio.indiana.edu/.bin/fbidq.html?beat-IIa), [grn](http://flybase.bio.indiana.edu/.bin/fbidq.html?grn), [sm](http://flybase.bio.indiana.edu/.bin/fbidq.html?sm), [DAAM](http://flybase.bio.indiana.edu/.bin/fbidq.html?DAAM), [mew](http://flybase.bio.indiana.edu/.bin/fbidq.html?mew), [Lis-1](http://flybase.bio.indiana.edu/.bin/fbidq.html?Lis-1), [dac](http://flybase.bio.indiana.edu/.bin/fbidq.html?dac), [ems](http://flybase.bio.indiana.edu/.bin/fbidq.html?ems), [sif](http://flybase.bio.indiana.edu/.bin/fbidq.html?sif), [Cdk5](http://flybase.bio.indiana.edu/.bin/fbidq.html?Cdk5), [pros](http://flybase.bio.indiana.edu/.bin/fbidq.html?pros), [tup](http://flybase.bio.indiana.edu/.bin/fbidq.html?tup), [trio](http://flybase.bio.indiana.edu/.bin/fbidq.html?trio), [ssh](http://flybase.bio.indiana.edu/.bin/fbidq.html?ssh), [mle](http://flybase.bio.indiana.edu/.bin/fbidq.html?mle), [Hr51](http://flybase.bio.indiana.edu/.bin/fbidq.html?Hr51), [daw](http://flybase.bio.indiana.edu/.bin/fbidq.html?daw),[rst](http://flybase.bio.indiana.edu/.bin/fbidq.html?rst), [Wnt4](http://flybase.bio.indiana.edu/.bin/fbidq.html?Wnt4), [comm](http://flybase.bio.indiana.edu/.bin/fbidq.html?comm), [Cdk5alpha](http://flybase.bio.indiana.edu/.bin/fbidq.html?Cdk5alpha), [Apc](http://flybase.bio.indiana.edu/.bin/fbidq.html?Apc), [sim](http://flybase.bio.indiana.edu/.bin/fbidq.html?sim), [Lim3](http://flybase.bio.indiana.edu/.bin/fbidq.html?Lim3), [CadN](http://flybase.bio.indiana.edu/.bin/fbidq.html?CadN), [nerfin-1](http://flybase.bio.indiana.edu/.bin/fbidq.html?nerfin-1), [bchs](http://flybase.bio.indiana.edu/.bin/fbidq.html?bchs), [trx](http://flybase.bio.indiana.edu/.bin/fbidq.html?trx), [wnd](http://flybase.bio.indiana.edu/.bin/fbidq.html?wnd), [Rpd3](http://flybase.bio.indiana.edu/.bin/fbidq.html?Rpd3), [retn](http://flybase.bio.indiana.edu/.bin/fbidq.html?retn), [en](http://flybase.bio.indiana.edu/.bin/fbidq.html?en), [dsx](http://flybase.bio.indiana.edu/.bin/fbidq.html?dsx), [unc-5](http://flybase.bio.indiana.edu/.bin/fbidq.html?unc-5), [trn](http://flybase.bio.indiana.edu/.bin/fbidq.html?trn), [CG10107](http://flybase.bio.indiana.edu/.bin/fbidq.html?CG10107), [fend](http://flybase.bio.indiana.edu/.bin/fbidq.html?fend), [CG33960](http://flybase.bio.indiana.edu/.bin/fbidq.html?CG33960) |
| [system process](http://amigo.geneontology.org/cgi-bin/amigo/go.cgi?view=details&query=GO:0003008) | 153 of 1594 genes, 9.6% | 452 of 7634 genes, 5.9% | 8.29e-10 | 0.00% | 0.00 | [endoA](http://flybase.bio.indiana.edu/.bin/fbidq.html?endoA), [Sh](http://flybase.bio.indiana.edu/.bin/fbidq.html?Sh), [n-syb](http://flybase.bio.indiana.edu/.bin/fbidq.html?n-syb), [acj6](http://flybase.bio.indiana.edu/.bin/fbidq.html?acj6), [ato](http://flybase.bio.indiana.edu/.bin/fbidq.html?ato), [Fmrf](http://flybase.bio.indiana.edu/.bin/fbidq.html?Fmrf), [stj](http://flybase.bio.indiana.edu/.bin/fbidq.html?stj), [Dg](http://flybase.bio.indiana.edu/.bin/fbidq.html?Dg), [Nf1](http://flybase.bio.indiana.edu/.bin/fbidq.html?Nf1), [Pka-R1](http://flybase.bio.indiana.edu/.bin/fbidq.html?Pka-R1), [sns](http://flybase.bio.indiana.edu/.bin/fbidq.html?sns), [os](http://flybase.bio.indiana.edu/.bin/fbidq.html?os), [brp](http://flybase.bio.indiana.edu/.bin/fbidq.html?brp), [Sytalpha](http://flybase.bio.indiana.edu/.bin/fbidq.html?Sytalpha), [nonA](http://flybase.bio.indiana.edu/.bin/fbidq.html?nonA), [lola](http://flybase.bio.indiana.edu/.bin/fbidq.html?lola), [unc-104](http://flybase.bio.indiana.edu/.bin/fbidq.html?unc-104), [Khc-73](http://flybase.bio.indiana.edu/.bin/fbidq.html?Khc-73), [rdgC](http://flybase.bio.indiana.edu/.bin/fbidq.html?rdgC), [Appl](http://flybase.bio.indiana.edu/.bin/fbidq.html?Appl), [metro](http://flybase.bio.indiana.edu/.bin/fbidq.html?metro), [ewg](http://flybase.bio.indiana.edu/.bin/fbidq.html?ewg), [Cirl](http://flybase.bio.indiana.edu/.bin/fbidq.html?Cirl), [Galpha49B](http://flybase.bio.indiana.edu/.bin/fbidq.html?Galpha49B), [scrib](http://flybase.bio.indiana.edu/.bin/fbidq.html?scrib), [rut](http://flybase.bio.indiana.edu/.bin/fbidq.html?rut), [Nrg](http://flybase.bio.indiana.edu/.bin/fbidq.html?Nrg), [unc-13-4A](http://flybase.bio.indiana.edu/.bin/fbidq.html?unc-13-4A), [Ddc](http://flybase.bio.indiana.edu/.bin/fbidq.html?Ddc), [stau](http://flybase.bio.indiana.edu/.bin/fbidq.html?stau), [Sema-1a](http://flybase.bio.indiana.edu/.bin/fbidq.html?Sema-1a), [dpr15](http://flybase.bio.indiana.edu/.bin/fbidq.html?dpr15), [TBPH](http://flybase.bio.indiana.edu/.bin/fbidq.html?TBPH), [Snap25](http://flybase.bio.indiana.edu/.bin/fbidq.html?Snap25), [mGluRA](http://flybase.bio.indiana.edu/.bin/fbidq.html?mGluRA), [Fas2](http://flybase.bio.indiana.edu/.bin/fbidq.html?Fas2), [14-3-3zeta](http://flybase.bio.indiana.edu/.bin/fbidq.html?14-3-3zeta), [robo](http://flybase.bio.indiana.edu/.bin/fbidq.html?robo), [dpr20](http://flybase.bio.indiana.edu/.bin/fbidq.html?dpr20), [mtg](http://flybase.bio.indiana.edu/.bin/fbidq.html?mtg), [comt](http://flybase.bio.indiana.edu/.bin/fbidq.html?comt), [Pbprp1](http://flybase.bio.indiana.edu/.bin/fbidq.html?Pbprp1), [PP2A-B](http://flybase.bio.indiana.edu/.bin/fbidq.html?PP2A-B), [qvr](http://flybase.bio.indiana.edu/.bin/fbidq.html?qvr), [Klp64D](http://flybase.bio.indiana.edu/.bin/fbidq.html?Klp64D), [sws](http://flybase.bio.indiana.edu/.bin/fbidq.html?sws), [disco](http://flybase.bio.indiana.edu/.bin/fbidq.html?disco), [DopR](http://flybase.bio.indiana.edu/.bin/fbidq.html?DopR), [CG4019](http://flybase.bio.indiana.edu/.bin/fbidq.html?CG4019), [mew](http://flybase.bio.indiana.edu/.bin/fbidq.html?mew), [neur](http://flybase.bio.indiana.edu/.bin/fbidq.html?neur), [nmo](http://flybase.bio.indiana.edu/.bin/fbidq.html?nmo), [5-HT1A](http://flybase.bio.indiana.edu/.bin/fbidq.html?5-HT1A), [Mob2](http://flybase.bio.indiana.edu/.bin/fbidq.html?Mob2), [pros](http://flybase.bio.indiana.edu/.bin/fbidq.html?pros), [Rab3](http://flybase.bio.indiana.edu/.bin/fbidq.html?Rab3), [trio](http://flybase.bio.indiana.edu/.bin/fbidq.html?trio), [mr](http://flybase.bio.indiana.edu/.bin/fbidq.html?mr), [orb2](http://flybase.bio.indiana.edu/.bin/fbidq.html?orb2), [CG31665](http://flybase.bio.indiana.edu/.bin/fbidq.html?CG31665), [Ccap](http://flybase.bio.indiana.edu/.bin/fbidq.html?Ccap), [Gad1](http://flybase.bio.indiana.edu/.bin/fbidq.html?Gad1), [Fhos](http://flybase.bio.indiana.edu/.bin/fbidq.html?Fhos), [rogdi](http://flybase.bio.indiana.edu/.bin/fbidq.html?rogdi), [salr](http://flybase.bio.indiana.edu/.bin/fbidq.html?salr), [synaptogyrin](http://flybase.bio.indiana.edu/.bin/fbidq.html?synaptogyrin), [CaMKII](http://flybase.bio.indiana.edu/.bin/fbidq.html?CaMKII), [tau](http://flybase.bio.indiana.edu/.bin/fbidq.html?tau), [CG42629](http://flybase.bio.indiana.edu/.bin/fbidq.html?CG42629), [Rya-r44F](http://flybase.bio.indiana.edu/.bin/fbidq.html?Rya-r44F), [bchs](http://flybase.bio.indiana.edu/.bin/fbidq.html?bchs), [king-tubby](http://flybase.bio.indiana.edu/.bin/fbidq.html?king-tubby), [Btk29A](http://flybase.bio.indiana.edu/.bin/fbidq.html?Btk29A), [PQBP-1](http://flybase.bio.indiana.edu/.bin/fbidq.html?PQBP-1), [Syn1](http://flybase.bio.indiana.edu/.bin/fbidq.html?Syn1), [dpr11](http://flybase.bio.indiana.edu/.bin/fbidq.html?dpr11), [HLHm7](http://flybase.bio.indiana.edu/.bin/fbidq.html?HLHm7), [Ca-alpha1D](http://flybase.bio.indiana.edu/.bin/fbidq.html?Ca-alpha1D), [Klp68D](http://flybase.bio.indiana.edu/.bin/fbidq.html?Klp68D), [Dsk](http://flybase.bio.indiana.edu/.bin/fbidq.html?Dsk), [Atg1](http://flybase.bio.indiana.edu/.bin/fbidq.html?Atg1), [sei](http://flybase.bio.indiana.edu/.bin/fbidq.html?sei), [CG12187](http://flybase.bio.indiana.edu/.bin/fbidq.html?CG12187), [rg](http://flybase.bio.indiana.edu/.bin/fbidq.html?rg), [unc-13](http://flybase.bio.indiana.edu/.bin/fbidq.html?unc-13), [betaInt-nu](http://flybase.bio.indiana.edu/.bin/fbidq.html?betaInt-nu), [dpr8](http://flybase.bio.indiana.edu/.bin/fbidq.html?dpr8), [CG1909](http://flybase.bio.indiana.edu/.bin/fbidq.html?CG1909), [sgg](http://flybase.bio.indiana.edu/.bin/fbidq.html?sgg), [mnb](http://flybase.bio.indiana.edu/.bin/fbidq.html?mnb), [Nmdar1](http://flybase.bio.indiana.edu/.bin/fbidq.html?Nmdar1), [dpr9](http://flybase.bio.indiana.edu/.bin/fbidq.html?dpr9), [dpr10](http://flybase.bio.indiana.edu/.bin/fbidq.html?dpr10), [CanB](http://flybase.bio.indiana.edu/.bin/fbidq.html?CanB), [oa2](http://flybase.bio.indiana.edu/.bin/fbidq.html?oa2), [rab3-GEF](http://flybase.bio.indiana.edu/.bin/fbidq.html?rab3-GEF), [CG10251](http://flybase.bio.indiana.edu/.bin/fbidq.html?CG10251), [CASK](http://flybase.bio.indiana.edu/.bin/fbidq.html?CASK), [Syt4](http://flybase.bio.indiana.edu/.bin/fbidq.html?Syt4), [CG7708](http://flybase.bio.indiana.edu/.bin/fbidq.html?CG7708),[Alk](http://flybase.bio.indiana.edu/.bin/fbidq.html?Alk), [Dat](http://flybase.bio.indiana.edu/.bin/fbidq.html?Dat), [dpr3](http://flybase.bio.indiana.edu/.bin/fbidq.html?dpr3), [cpx](http://flybase.bio.indiana.edu/.bin/fbidq.html?cpx), [gfA](http://flybase.bio.indiana.edu/.bin/fbidq.html?gfA), [Tk](http://flybase.bio.indiana.edu/.bin/fbidq.html?Tk), [Fak56D](http://flybase.bio.indiana.edu/.bin/fbidq.html?Fak56D), [Rph](http://flybase.bio.indiana.edu/.bin/fbidq.html?Rph), [srpk79D](http://flybase.bio.indiana.edu/.bin/fbidq.html?srpk79D), [Kap3](http://flybase.bio.indiana.edu/.bin/fbidq.html?Kap3), [dpr17](http://flybase.bio.indiana.edu/.bin/fbidq.html?dpr17), [dpr14](http://flybase.bio.indiana.edu/.bin/fbidq.html?dpr14), [Rdl](http://flybase.bio.indiana.edu/.bin/fbidq.html?Rdl), [stan](http://flybase.bio.indiana.edu/.bin/fbidq.html?stan), [GABA-B-R2](http://flybase.bio.indiana.edu/.bin/fbidq.html?GABA-B-R2), [Ptth](http://flybase.bio.indiana.edu/.bin/fbidq.html?Ptth), [Ast-C](http://flybase.bio.indiana.edu/.bin/fbidq.html?Ast-C), [futsch](http://flybase.bio.indiana.edu/.bin/fbidq.html?futsch), [Frq1](http://flybase.bio.indiana.edu/.bin/fbidq.html?Frq1), [Rim](http://flybase.bio.indiana.edu/.bin/fbidq.html?Rim), [Shal](http://flybase.bio.indiana.edu/.bin/fbidq.html?Shal), [pigeon](http://flybase.bio.indiana.edu/.bin/fbidq.html?pigeon), [para](http://flybase.bio.indiana.edu/.bin/fbidq.html?para), [Syn2](http://flybase.bio.indiana.edu/.bin/fbidq.html?Syn2), [eas](http://flybase.bio.indiana.edu/.bin/fbidq.html?eas), [CRMP](http://flybase.bio.indiana.edu/.bin/fbidq.html?CRMP), [Nrx-1](http://flybase.bio.indiana.edu/.bin/fbidq.html?Nrx-1), [Cdk5](http://flybase.bio.indiana.edu/.bin/fbidq.html?Cdk5), [sif](http://flybase.bio.indiana.edu/.bin/fbidq.html?sif), [CG4641](http://flybase.bio.indiana.edu/.bin/fbidq.html?CG4641), [Tbh](http://flybase.bio.indiana.edu/.bin/fbidq.html?Tbh), [PIP5K59B](http://flybase.bio.indiana.edu/.bin/fbidq.html?PIP5K59B), [Oamb](http://flybase.bio.indiana.edu/.bin/fbidq.html?Oamb), [lap](http://flybase.bio.indiana.edu/.bin/fbidq.html?lap), [shi](http://flybase.bio.indiana.edu/.bin/fbidq.html?shi), [CG11155](http://flybase.bio.indiana.edu/.bin/fbidq.html?CG11155),[CG3822](http://flybase.bio.indiana.edu/.bin/fbidq.html?CG3822), [dpr5](http://flybase.bio.indiana.edu/.bin/fbidq.html?dpr5), [Vmat](http://flybase.bio.indiana.edu/.bin/fbidq.html?Vmat), [Cha](http://flybase.bio.indiana.edu/.bin/fbidq.html?Cha), [Aplip1](http://flybase.bio.indiana.edu/.bin/fbidq.html?Aplip1), [klg](http://flybase.bio.indiana.edu/.bin/fbidq.html?klg), [sfl](http://flybase.bio.indiana.edu/.bin/fbidq.html?sfl), [rl](http://flybase.bio.indiana.edu/.bin/fbidq.html?rl), [Syt1](http://flybase.bio.indiana.edu/.bin/fbidq.html?Syt1), [dpr19](http://flybase.bio.indiana.edu/.bin/fbidq.html?dpr19), [Dms](http://flybase.bio.indiana.edu/.bin/fbidq.html?Dms), [Snap](http://flybase.bio.indiana.edu/.bin/fbidq.html?Snap), [SerT](http://flybase.bio.indiana.edu/.bin/fbidq.html?SerT), [dpr7](http://flybase.bio.indiana.edu/.bin/fbidq.html?dpr7), [dpr18](http://flybase.bio.indiana.edu/.bin/fbidq.html?dpr18), [hiw](http://flybase.bio.indiana.edu/.bin/fbidq.html?hiw), [Ct](http://flybase.bio.indiana.edu/.bin/fbidq.html?Ct) |
| [cell morphogenesis involved in neuron differentiation](http://amigo.geneontology.org/cgi-bin/amigo/go.cgi?view=details&query=GO:0048667) | 127 of 1594 genes, 8.0% | 354 of 7634 genes, 4.6% | 9.54e-10 | 0.00% | 0.00 | [fz2](http://flybase.bio.indiana.edu/.bin/fbidq.html?fz2), [Sh](http://flybase.bio.indiana.edu/.bin/fbidq.html?Sh), [acj6](http://flybase.bio.indiana.edu/.bin/fbidq.html?acj6), [wor](http://flybase.bio.indiana.edu/.bin/fbidq.html?wor), [vvl](http://flybase.bio.indiana.edu/.bin/fbidq.html?vvl), [jing](http://flybase.bio.indiana.edu/.bin/fbidq.html?jing), [dock](http://flybase.bio.indiana.edu/.bin/fbidq.html?dock), [Pten](http://flybase.bio.indiana.edu/.bin/fbidq.html?Pten), [ato](http://flybase.bio.indiana.edu/.bin/fbidq.html?ato), [Ptp69D](http://flybase.bio.indiana.edu/.bin/fbidq.html?Ptp69D), [gcm2](http://flybase.bio.indiana.edu/.bin/fbidq.html?gcm2), [hts](http://flybase.bio.indiana.edu/.bin/fbidq.html?hts), [mp](http://flybase.bio.indiana.edu/.bin/fbidq.html?mp), [ap](http://flybase.bio.indiana.edu/.bin/fbidq.html?ap), [Iswi](http://flybase.bio.indiana.edu/.bin/fbidq.html?Iswi), [Dg](http://flybase.bio.indiana.edu/.bin/fbidq.html?Dg), [seq](http://flybase.bio.indiana.edu/.bin/fbidq.html?seq), [gcm](http://flybase.bio.indiana.edu/.bin/fbidq.html?gcm), [Trim9](http://flybase.bio.indiana.edu/.bin/fbidq.html?Trim9), [robo3](http://flybase.bio.indiana.edu/.bin/fbidq.html?robo3), [tok](http://flybase.bio.indiana.edu/.bin/fbidq.html?tok), [Ptp99A](http://flybase.bio.indiana.edu/.bin/fbidq.html?Ptp99A), [lola](http://flybase.bio.indiana.edu/.bin/fbidq.html?lola), [unc-104](http://flybase.bio.indiana.edu/.bin/fbidq.html?unc-104), [chif](http://flybase.bio.indiana.edu/.bin/fbidq.html?chif), [NetB](http://flybase.bio.indiana.edu/.bin/fbidq.html?NetB), [p130CAS](http://flybase.bio.indiana.edu/.bin/fbidq.html?p130CAS), [Alk](http://flybase.bio.indiana.edu/.bin/fbidq.html?Alk), [Tango10](http://flybase.bio.indiana.edu/.bin/fbidq.html?Tango10), [Sin3A](http://flybase.bio.indiana.edu/.bin/fbidq.html?Sin3A), [Nrk](http://flybase.bio.indiana.edu/.bin/fbidq.html?Nrk), [CG4328](http://flybase.bio.indiana.edu/.bin/fbidq.html?CG4328), [Galpha49B](http://flybase.bio.indiana.edu/.bin/fbidq.html?Galpha49B), [jumu](http://flybase.bio.indiana.edu/.bin/fbidq.html?jumu), [Smox](http://flybase.bio.indiana.edu/.bin/fbidq.html?Smox), [Lar](http://flybase.bio.indiana.edu/.bin/fbidq.html?Lar), [Taf4](http://flybase.bio.indiana.edu/.bin/fbidq.html?Taf4), [not](http://flybase.bio.indiana.edu/.bin/fbidq.html?not), [rut](http://flybase.bio.indiana.edu/.bin/fbidq.html?rut), [nvy](http://flybase.bio.indiana.edu/.bin/fbidq.html?nvy),[Nrg](http://flybase.bio.indiana.edu/.bin/fbidq.html?Nrg), [gogo](http://flybase.bio.indiana.edu/.bin/fbidq.html?gogo), [fd59A](http://flybase.bio.indiana.edu/.bin/fbidq.html?fd59A), [beat-Ic](http://flybase.bio.indiana.edu/.bin/fbidq.html?beat-Ic), [run](http://flybase.bio.indiana.edu/.bin/fbidq.html?run), [jeb](http://flybase.bio.indiana.edu/.bin/fbidq.html?jeb), [brat](http://flybase.bio.indiana.edu/.bin/fbidq.html?brat), [ko](http://flybase.bio.indiana.edu/.bin/fbidq.html?ko), [Actbeta](http://flybase.bio.indiana.edu/.bin/fbidq.html?Actbeta), [Sema-1a](http://flybase.bio.indiana.edu/.bin/fbidq.html?Sema-1a), [beat-Ib](http://flybase.bio.indiana.edu/.bin/fbidq.html?beat-Ib), [otk](http://flybase.bio.indiana.edu/.bin/fbidq.html?otk), [stan](http://flybase.bio.indiana.edu/.bin/fbidq.html?stan), [CG12424](http://flybase.bio.indiana.edu/.bin/fbidq.html?CG12424), [CG34400](http://flybase.bio.indiana.edu/.bin/fbidq.html?CG34400), [Ulp1](http://flybase.bio.indiana.edu/.bin/fbidq.html?Ulp1), [tutl](http://flybase.bio.indiana.edu/.bin/fbidq.html?tutl), [ena](http://flybase.bio.indiana.edu/.bin/fbidq.html?ena), [dsh](http://flybase.bio.indiana.edu/.bin/fbidq.html?dsh), [Fas2](http://flybase.bio.indiana.edu/.bin/fbidq.html?Fas2), [Nf-YC](http://flybase.bio.indiana.edu/.bin/fbidq.html?Nf-YC), [fz](http://flybase.bio.indiana.edu/.bin/fbidq.html?fz), [robo](http://flybase.bio.indiana.edu/.bin/fbidq.html?robo), [lea](http://flybase.bio.indiana.edu/.bin/fbidq.html?lea), [futsch](http://flybase.bio.indiana.edu/.bin/fbidq.html?futsch), [bsk](http://flybase.bio.indiana.edu/.bin/fbidq.html?bsk), [brm](http://flybase.bio.indiana.edu/.bin/fbidq.html?brm), [beat-Ia](http://flybase.bio.indiana.edu/.bin/fbidq.html?beat-Ia), [Ptx1](http://flybase.bio.indiana.edu/.bin/fbidq.html?Ptx1), [kat-60L1](http://flybase.bio.indiana.edu/.bin/fbidq.html?kat-60L1), [gukh](http://flybase.bio.indiana.edu/.bin/fbidq.html?gukh), [RhoGAPp190](http://flybase.bio.indiana.edu/.bin/fbidq.html?RhoGAPp190), [chm](http://flybase.bio.indiana.edu/.bin/fbidq.html?chm), [plexB](http://flybase.bio.indiana.edu/.bin/fbidq.html?plexB), [sna](http://flybase.bio.indiana.edu/.bin/fbidq.html?sna), [Wnt5](http://flybase.bio.indiana.edu/.bin/fbidq.html?Wnt5),[Klp64D](http://flybase.bio.indiana.edu/.bin/fbidq.html?Klp64D), [beat-IIa](http://flybase.bio.indiana.edu/.bin/fbidq.html?beat-IIa), [grn](http://flybase.bio.indiana.edu/.bin/fbidq.html?grn), [sm](http://flybase.bio.indiana.edu/.bin/fbidq.html?sm), [DAAM](http://flybase.bio.indiana.edu/.bin/fbidq.html?DAAM), [mew](http://flybase.bio.indiana.edu/.bin/fbidq.html?mew), [Lis-1](http://flybase.bio.indiana.edu/.bin/fbidq.html?Lis-1), [dac](http://flybase.bio.indiana.edu/.bin/fbidq.html?dac), [ems](http://flybase.bio.indiana.edu/.bin/fbidq.html?ems), [sif](http://flybase.bio.indiana.edu/.bin/fbidq.html?sif), [Cdk5](http://flybase.bio.indiana.edu/.bin/fbidq.html?Cdk5), [dom](http://flybase.bio.indiana.edu/.bin/fbidq.html?dom), [pros](http://flybase.bio.indiana.edu/.bin/fbidq.html?pros), [tup](http://flybase.bio.indiana.edu/.bin/fbidq.html?tup), [trio](http://flybase.bio.indiana.edu/.bin/fbidq.html?trio), [ssh](http://flybase.bio.indiana.edu/.bin/fbidq.html?ssh), [baz](http://flybase.bio.indiana.edu/.bin/fbidq.html?baz), [mle](http://flybase.bio.indiana.edu/.bin/fbidq.html?mle), [Hr51](http://flybase.bio.indiana.edu/.bin/fbidq.html?Hr51), [daw](http://flybase.bio.indiana.edu/.bin/fbidq.html?daw), [rst](http://flybase.bio.indiana.edu/.bin/fbidq.html?rst), [Wnt4](http://flybase.bio.indiana.edu/.bin/fbidq.html?Wnt4), [comm](http://flybase.bio.indiana.edu/.bin/fbidq.html?comm), [rictor](http://flybase.bio.indiana.edu/.bin/fbidq.html?rictor), [Cdk5alpha](http://flybase.bio.indiana.edu/.bin/fbidq.html?Cdk5alpha), [Apc](http://flybase.bio.indiana.edu/.bin/fbidq.html?Apc), [sim](http://flybase.bio.indiana.edu/.bin/fbidq.html?sim), [Lim3](http://flybase.bio.indiana.edu/.bin/fbidq.html?Lim3), [d4](http://flybase.bio.indiana.edu/.bin/fbidq.html?d4), [CadN](http://flybase.bio.indiana.edu/.bin/fbidq.html?CadN), [gro](http://flybase.bio.indiana.edu/.bin/fbidq.html?gro), [nerfin-1](http://flybase.bio.indiana.edu/.bin/fbidq.html?nerfin-1), [bchs](http://flybase.bio.indiana.edu/.bin/fbidq.html?bchs), [E(Pc)](http://flybase.bio.indiana.edu/.bin/fbidq.html?E(Pc)), [trx](http://flybase.bio.indiana.edu/.bin/fbidq.html?trx), [wnd](http://flybase.bio.indiana.edu/.bin/fbidq.html?wnd), [Rpd3](http://flybase.bio.indiana.edu/.bin/fbidq.html?Rpd3), [retn](http://flybase.bio.indiana.edu/.bin/fbidq.html?retn), [en](http://flybase.bio.indiana.edu/.bin/fbidq.html?en), [E(bx)](http://flybase.bio.indiana.edu/.bin/fbidq.html?E(bx)), [dsx](http://flybase.bio.indiana.edu/.bin/fbidq.html?dsx),[chinmo](http://flybase.bio.indiana.edu/.bin/fbidq.html?chinmo), [unc-5](http://flybase.bio.indiana.edu/.bin/fbidq.html?unc-5), [trn](http://flybase.bio.indiana.edu/.bin/fbidq.html?trn), [CG10107](http://flybase.bio.indiana.edu/.bin/fbidq.html?CG10107), [sqz](http://flybase.bio.indiana.edu/.bin/fbidq.html?sqz), [fend](http://flybase.bio.indiana.edu/.bin/fbidq.html?fend), [CG33960](http://flybase.bio.indiana.edu/.bin/fbidq.html?CG33960), [pygo](http://flybase.bio.indiana.edu/.bin/fbidq.html?pygo), [HLHm7](http://flybase.bio.indiana.edu/.bin/fbidq.html?HLHm7), [Ct](http://flybase.bio.indiana.edu/.bin/fbidq.html?Ct) |
| [regulation of macromolecule biosynthetic process](http://amigo.geneontology.org/cgi-bin/amigo/go.cgi?view=details&query=GO:0010556) | 206 of 1594 genes, 12.9% | 664 of 7634 genes, 8.7% | 1.06e-09 | 0.00% | 0.00 | [fz2](http://flybase.bio.indiana.edu/.bin/fbidq.html?fz2), [acj6](http://flybase.bio.indiana.edu/.bin/fbidq.html?acj6), [skd](http://flybase.bio.indiana.edu/.bin/fbidq.html?skd), [jing](http://flybase.bio.indiana.edu/.bin/fbidq.html?jing), [caup](http://flybase.bio.indiana.edu/.bin/fbidq.html?caup), [tll](http://flybase.bio.indiana.edu/.bin/fbidq.html?tll), [Camta](http://flybase.bio.indiana.edu/.bin/fbidq.html?Camta), [ato](http://flybase.bio.indiana.edu/.bin/fbidq.html?ato), [Ets65A](http://flybase.bio.indiana.edu/.bin/fbidq.html?Ets65A), [tna](http://flybase.bio.indiana.edu/.bin/fbidq.html?tna), [Sxl](http://flybase.bio.indiana.edu/.bin/fbidq.html?Sxl), [JIL-1](http://flybase.bio.indiana.edu/.bin/fbidq.html?JIL-1), [Spt6](http://flybase.bio.indiana.edu/.bin/fbidq.html?Spt6), [Iswi](http://flybase.bio.indiana.edu/.bin/fbidq.html?Iswi), [rn](http://flybase.bio.indiana.edu/.bin/fbidq.html?rn), [ap](http://flybase.bio.indiana.edu/.bin/fbidq.html?ap), [br](http://flybase.bio.indiana.edu/.bin/fbidq.html?br), [ksr](http://flybase.bio.indiana.edu/.bin/fbidq.html?ksr), [bi](http://flybase.bio.indiana.edu/.bin/fbidq.html?bi), [chn](http://flybase.bio.indiana.edu/.bin/fbidq.html?chn), [Gsc](http://flybase.bio.indiana.edu/.bin/fbidq.html?Gsc), [Hcf](http://flybase.bio.indiana.edu/.bin/fbidq.html?Hcf), [lola](http://flybase.bio.indiana.edu/.bin/fbidq.html?lola), [HLH4C](http://flybase.bio.indiana.edu/.bin/fbidq.html?HLH4C), [Pcl](http://flybase.bio.indiana.edu/.bin/fbidq.html?Pcl), [chif](http://flybase.bio.indiana.edu/.bin/fbidq.html?chif), [simj](http://flybase.bio.indiana.edu/.bin/fbidq.html?simj), [Awh](http://flybase.bio.indiana.edu/.bin/fbidq.html?Awh), [tap](http://flybase.bio.indiana.edu/.bin/fbidq.html?tap), [NC2alpha](http://flybase.bio.indiana.edu/.bin/fbidq.html?NC2alpha), [PHDP](http://flybase.bio.indiana.edu/.bin/fbidq.html?PHDP), [bab1](http://flybase.bio.indiana.edu/.bin/fbidq.html?bab1), [CG4328](http://flybase.bio.indiana.edu/.bin/fbidq.html?CG4328), [Snoo](http://flybase.bio.indiana.edu/.bin/fbidq.html?Snoo), [l(2)NC136](http://flybase.bio.indiana.edu/.bin/fbidq.html?l(2)NC136), [Taf4](http://flybase.bio.indiana.edu/.bin/fbidq.html?Taf4), [Su(var)3-3](http://flybase.bio.indiana.edu/.bin/fbidq.html?Su(var)3-3), [HGTX](http://flybase.bio.indiana.edu/.bin/fbidq.html?HGTX), [not](http://flybase.bio.indiana.edu/.bin/fbidq.html?not), [nvy](http://flybase.bio.indiana.edu/.bin/fbidq.html?nvy), [nub](http://flybase.bio.indiana.edu/.bin/fbidq.html?nub), [nab](http://flybase.bio.indiana.edu/.bin/fbidq.html?nab),[btd](http://flybase.bio.indiana.edu/.bin/fbidq.html?btd), [fd68A](http://flybase.bio.indiana.edu/.bin/fbidq.html?fd68A), [HLHmdelta](http://flybase.bio.indiana.edu/.bin/fbidq.html?HLHmdelta), [B-H1](http://flybase.bio.indiana.edu/.bin/fbidq.html?B-H1), [Nipped-A](http://flybase.bio.indiana.edu/.bin/fbidq.html?Nipped-A), [ATbp](http://flybase.bio.indiana.edu/.bin/fbidq.html?ATbp), [stau](http://flybase.bio.indiana.edu/.bin/fbidq.html?stau), [Sos](http://flybase.bio.indiana.edu/.bin/fbidq.html?Sos), [Fer2](http://flybase.bio.indiana.edu/.bin/fbidq.html?Fer2), [fd102C](http://flybase.bio.indiana.edu/.bin/fbidq.html?fd102C), [vg](http://flybase.bio.indiana.edu/.bin/fbidq.html?vg), [hth](http://flybase.bio.indiana.edu/.bin/fbidq.html?hth), [Nf-YC](http://flybase.bio.indiana.edu/.bin/fbidq.html?Nf-YC), [eya](http://flybase.bio.indiana.edu/.bin/fbidq.html?eya), [fz](http://flybase.bio.indiana.edu/.bin/fbidq.html?fz), [brm](http://flybase.bio.indiana.edu/.bin/fbidq.html?brm), [Vsx2](http://flybase.bio.indiana.edu/.bin/fbidq.html?Vsx2), [Hey](http://flybase.bio.indiana.edu/.bin/fbidq.html?Hey), [sno](http://flybase.bio.indiana.edu/.bin/fbidq.html?sno), [Sfmbt](http://flybase.bio.indiana.edu/.bin/fbidq.html?Sfmbt), [ara](http://flybase.bio.indiana.edu/.bin/fbidq.html?ara), [mam](http://flybase.bio.indiana.edu/.bin/fbidq.html?mam), [pan](http://flybase.bio.indiana.edu/.bin/fbidq.html?pan), [Dll](http://flybase.bio.indiana.edu/.bin/fbidq.html?Dll), [disco](http://flybase.bio.indiana.edu/.bin/fbidq.html?disco), [grn](http://flybase.bio.indiana.edu/.bin/fbidq.html?grn), [HLHm5](http://flybase.bio.indiana.edu/.bin/fbidq.html?HLHm5), [D12](http://flybase.bio.indiana.edu/.bin/fbidq.html?D12), [Lis-1](http://flybase.bio.indiana.edu/.bin/fbidq.html?Lis-1), [CycE](http://flybase.bio.indiana.edu/.bin/fbidq.html?CycE), [Brf](http://flybase.bio.indiana.edu/.bin/fbidq.html?Brf), [ems](http://flybase.bio.indiana.edu/.bin/fbidq.html?ems), [pros](http://flybase.bio.indiana.edu/.bin/fbidq.html?pros), [tup](http://flybase.bio.indiana.edu/.bin/fbidq.html?tup), [pdm2](http://flybase.bio.indiana.edu/.bin/fbidq.html?pdm2), [Gug](http://flybase.bio.indiana.edu/.bin/fbidq.html?Gug), [pad](http://flybase.bio.indiana.edu/.bin/fbidq.html?pad), [rgr](http://flybase.bio.indiana.edu/.bin/fbidq.html?rgr), [orb2](http://flybase.bio.indiana.edu/.bin/fbidq.html?orb2), [Wnt4](http://flybase.bio.indiana.edu/.bin/fbidq.html?Wnt4), [Rx](http://flybase.bio.indiana.edu/.bin/fbidq.html?Rx),[MED1](http://flybase.bio.indiana.edu/.bin/fbidq.html?MED1), [salr](http://flybase.bio.indiana.edu/.bin/fbidq.html?salr), [wdn](http://flybase.bio.indiana.edu/.bin/fbidq.html?wdn), [sim](http://flybase.bio.indiana.edu/.bin/fbidq.html?sim), [d4](http://flybase.bio.indiana.edu/.bin/fbidq.html?d4), [dan](http://flybase.bio.indiana.edu/.bin/fbidq.html?dan), [gro](http://flybase.bio.indiana.edu/.bin/fbidq.html?gro), [nerfin-1](http://flybase.bio.indiana.edu/.bin/fbidq.html?nerfin-1), [bin3](http://flybase.bio.indiana.edu/.bin/fbidq.html?bin3), [trx](http://flybase.bio.indiana.edu/.bin/fbidq.html?trx), [Rpd3](http://flybase.bio.indiana.edu/.bin/fbidq.html?Rpd3), [dsx](http://flybase.bio.indiana.edu/.bin/fbidq.html?dsx), [tyf](http://flybase.bio.indiana.edu/.bin/fbidq.html?tyf), [Ssdp](http://flybase.bio.indiana.edu/.bin/fbidq.html?Ssdp), [vfl](http://flybase.bio.indiana.edu/.bin/fbidq.html?vfl), [sqz](http://flybase.bio.indiana.edu/.bin/fbidq.html?sqz), [CG32105](http://flybase.bio.indiana.edu/.bin/fbidq.html?CG32105), [Fer3](http://flybase.bio.indiana.edu/.bin/fbidq.html?Fer3), [aret](http://flybase.bio.indiana.edu/.bin/fbidq.html?aret), [pygo](http://flybase.bio.indiana.edu/.bin/fbidq.html?pygo), [dalao](http://flybase.bio.indiana.edu/.bin/fbidq.html?dalao), [HLHm7](http://flybase.bio.indiana.edu/.bin/fbidq.html?HLHm7), [z](http://flybase.bio.indiana.edu/.bin/fbidq.html?z), [HLHmgamma](http://flybase.bio.indiana.edu/.bin/fbidq.html?HLHmgamma), [Sox21b](http://flybase.bio.indiana.edu/.bin/fbidq.html?Sox21b), [sr](http://flybase.bio.indiana.edu/.bin/fbidq.html?sr), [Lim1](http://flybase.bio.indiana.edu/.bin/fbidq.html?Lim1), [tld](http://flybase.bio.indiana.edu/.bin/fbidq.html?tld), [danr](http://flybase.bio.indiana.edu/.bin/fbidq.html?danr), [wor](http://flybase.bio.indiana.edu/.bin/fbidq.html?wor), [vvl](http://flybase.bio.indiana.edu/.bin/fbidq.html?vvl), [wda](http://flybase.bio.indiana.edu/.bin/fbidq.html?wda), [sgg](http://flybase.bio.indiana.edu/.bin/fbidq.html?sgg), [MED14](http://flybase.bio.indiana.edu/.bin/fbidq.html?MED14), [gcm2](http://flybase.bio.indiana.edu/.bin/fbidq.html?gcm2), [Eip93F](http://flybase.bio.indiana.edu/.bin/fbidq.html?Eip93F), [gcm](http://flybase.bio.indiana.edu/.bin/fbidq.html?gcm), [seq](http://flybase.bio.indiana.edu/.bin/fbidq.html?seq), [Dip3](http://flybase.bio.indiana.edu/.bin/fbidq.html?Dip3), [Aef1](http://flybase.bio.indiana.edu/.bin/fbidq.html?Aef1),[HDAC4](http://flybase.bio.indiana.edu/.bin/fbidq.html?HDAC4), [can](http://flybase.bio.indiana.edu/.bin/fbidq.html?can), [elk](http://flybase.bio.indiana.edu/.bin/fbidq.html?elk), [Hsp67Bc](http://flybase.bio.indiana.edu/.bin/fbidq.html?Hsp67Bc), [phl](http://flybase.bio.indiana.edu/.bin/fbidq.html?phl), [ase](http://flybase.bio.indiana.edu/.bin/fbidq.html?ase), [CG13253](http://flybase.bio.indiana.edu/.bin/fbidq.html?CG13253), [ovo](http://flybase.bio.indiana.edu/.bin/fbidq.html?ovo), [scro](http://flybase.bio.indiana.edu/.bin/fbidq.html?scro), [Dsp1](http://flybase.bio.indiana.edu/.bin/fbidq.html?Dsp1), [CG14216](http://flybase.bio.indiana.edu/.bin/fbidq.html?CG14216), [Pdp1](http://flybase.bio.indiana.edu/.bin/fbidq.html?Pdp1), [Oli](http://flybase.bio.indiana.edu/.bin/fbidq.html?Oli), [Nelf-A](http://flybase.bio.indiana.edu/.bin/fbidq.html?Nelf-A), [GATAd](http://flybase.bio.indiana.edu/.bin/fbidq.html?GATAd), [Poxn](http://flybase.bio.indiana.edu/.bin/fbidq.html?Poxn), [CG15376](http://flybase.bio.indiana.edu/.bin/fbidq.html?CG15376), [Sin3A](http://flybase.bio.indiana.edu/.bin/fbidq.html?Sin3A), [mip120](http://flybase.bio.indiana.edu/.bin/fbidq.html?mip120), [jumu](http://flybase.bio.indiana.edu/.bin/fbidq.html?jumu), [Smox](http://flybase.bio.indiana.edu/.bin/fbidq.html?Smox), [Usp7](http://flybase.bio.indiana.edu/.bin/fbidq.html?Usp7), [ush](http://flybase.bio.indiana.edu/.bin/fbidq.html?ush), [fd59A](http://flybase.bio.indiana.edu/.bin/fbidq.html?fd59A), [CG10336](http://flybase.bio.indiana.edu/.bin/fbidq.html?CG10336), [Gap1](http://flybase.bio.indiana.edu/.bin/fbidq.html?Gap1), [run](http://flybase.bio.indiana.edu/.bin/fbidq.html?run), [FBX011](http://flybase.bio.indiana.edu/.bin/fbidq.html?FBX011), [brat](http://flybase.bio.indiana.edu/.bin/fbidq.html?brat), [Antp](http://flybase.bio.indiana.edu/.bin/fbidq.html?Antp), [tara](http://flybase.bio.indiana.edu/.bin/fbidq.html?tara), [Alh](http://flybase.bio.indiana.edu/.bin/fbidq.html?Alh), [Sox102F](http://flybase.bio.indiana.edu/.bin/fbidq.html?Sox102F), [egg](http://flybase.bio.indiana.edu/.bin/fbidq.html?egg),[l(3)neo38](http://flybase.bio.indiana.edu/.bin/fbidq.html?l(3)neo38), [koko](http://flybase.bio.indiana.edu/.bin/fbidq.html?koko), [Ptth](http://flybase.bio.indiana.edu/.bin/fbidq.html?Ptth), [Sp1](http://flybase.bio.indiana.edu/.bin/fbidq.html?Sp1), [Ptx1](http://flybase.bio.indiana.edu/.bin/fbidq.html?Ptx1), [ph-p](http://flybase.bio.indiana.edu/.bin/fbidq.html?ph-p), [Ubx](http://flybase.bio.indiana.edu/.bin/fbidq.html?Ubx), [onecut](http://flybase.bio.indiana.edu/.bin/fbidq.html?onecut), [CG7757](http://flybase.bio.indiana.edu/.bin/fbidq.html?CG7757), [chm](http://flybase.bio.indiana.edu/.bin/fbidq.html?chm), [sna](http://flybase.bio.indiana.edu/.bin/fbidq.html?sna), [Wnt5](http://flybase.bio.indiana.edu/.bin/fbidq.html?Wnt5), [Taf1](http://flybase.bio.indiana.edu/.bin/fbidq.html?Taf1), [hbn](http://flybase.bio.indiana.edu/.bin/fbidq.html?hbn), [MBD-like](http://flybase.bio.indiana.edu/.bin/fbidq.html?MBD-like), [tsh](http://flybase.bio.indiana.edu/.bin/fbidq.html?tsh), [esg](http://flybase.bio.indiana.edu/.bin/fbidq.html?esg), [e(y)3](http://flybase.bio.indiana.edu/.bin/fbidq.html?e(y)3), [CG9007](http://flybase.bio.indiana.edu/.bin/fbidq.html?CG9007), [oc](http://flybase.bio.indiana.edu/.bin/fbidq.html?oc), [spt4](http://flybase.bio.indiana.edu/.bin/fbidq.html?spt4), [CG32532](http://flybase.bio.indiana.edu/.bin/fbidq.html?CG32532), [MTA1-like](http://flybase.bio.indiana.edu/.bin/fbidq.html?MTA1-like), [gol](http://flybase.bio.indiana.edu/.bin/fbidq.html?gol), [ey](http://flybase.bio.indiana.edu/.bin/fbidq.html?ey), [mle](http://flybase.bio.indiana.edu/.bin/fbidq.html?mle), [Hr51](http://flybase.bio.indiana.edu/.bin/fbidq.html?Hr51), [Bgb](http://flybase.bio.indiana.edu/.bin/fbidq.html?Bgb), [l(1)sc](http://flybase.bio.indiana.edu/.bin/fbidq.html?l(1)sc), [B-H2](http://flybase.bio.indiana.edu/.bin/fbidq.html?B-H2), [grh](http://flybase.bio.indiana.edu/.bin/fbidq.html?grh), [Vsx1](http://flybase.bio.indiana.edu/.bin/fbidq.html?Vsx1), [wg](http://flybase.bio.indiana.edu/.bin/fbidq.html?wg), [toy](http://flybase.bio.indiana.edu/.bin/fbidq.html?toy), [fkh](http://flybase.bio.indiana.edu/.bin/fbidq.html?fkh), [Lim3](http://flybase.bio.indiana.edu/.bin/fbidq.html?Lim3), [crol](http://flybase.bio.indiana.edu/.bin/fbidq.html?crol), [sd](http://flybase.bio.indiana.edu/.bin/fbidq.html?sd), [retn](http://flybase.bio.indiana.edu/.bin/fbidq.html?retn), [en](http://flybase.bio.indiana.edu/.bin/fbidq.html?en),[fs(1)h](http://flybase.bio.indiana.edu/.bin/fbidq.html?fs(1)h), [rl](http://flybase.bio.indiana.edu/.bin/fbidq.html?rl), [CG11347](http://flybase.bio.indiana.edu/.bin/fbidq.html?CG11347), [tey](http://flybase.bio.indiana.edu/.bin/fbidq.html?tey), [CG11294](http://flybase.bio.indiana.edu/.bin/fbidq.html?CG11294), [hiw](http://flybase.bio.indiana.edu/.bin/fbidq.html?hiw), [cos](http://flybase.bio.indiana.edu/.bin/fbidq.html?cos), [Ct](http://flybase.bio.indiana.edu/.bin/fbidq.html?Ct), [Atf-2](http://flybase.bio.indiana.edu/.bin/fbidq.html?Atf-2) |
| [regulation of cellular macromolecule biosynthetic process](http://amigo.geneontology.org/cgi-bin/amigo/go.cgi?view=details&query=GO:2000112) | 206 of 1594 genes, 12.9% | 664 of 7634 genes, 8.7% | 1.06e-09 | 0.00% | 0.00 | [fz2](http://flybase.bio.indiana.edu/.bin/fbidq.html?fz2), [acj6](http://flybase.bio.indiana.edu/.bin/fbidq.html?acj6), [skd](http://flybase.bio.indiana.edu/.bin/fbidq.html?skd), [jing](http://flybase.bio.indiana.edu/.bin/fbidq.html?jing), [caup](http://flybase.bio.indiana.edu/.bin/fbidq.html?caup), [tll](http://flybase.bio.indiana.edu/.bin/fbidq.html?tll), [Camta](http://flybase.bio.indiana.edu/.bin/fbidq.html?Camta), [ato](http://flybase.bio.indiana.edu/.bin/fbidq.html?ato), [Ets65A](http://flybase.bio.indiana.edu/.bin/fbidq.html?Ets65A), [tna](http://flybase.bio.indiana.edu/.bin/fbidq.html?tna), [Sxl](http://flybase.bio.indiana.edu/.bin/fbidq.html?Sxl), [JIL-1](http://flybase.bio.indiana.edu/.bin/fbidq.html?JIL-1), [Spt6](http://flybase.bio.indiana.edu/.bin/fbidq.html?Spt6), [Iswi](http://flybase.bio.indiana.edu/.bin/fbidq.html?Iswi), [rn](http://flybase.bio.indiana.edu/.bin/fbidq.html?rn), [ap](http://flybase.bio.indiana.edu/.bin/fbidq.html?ap), [br](http://flybase.bio.indiana.edu/.bin/fbidq.html?br), [ksr](http://flybase.bio.indiana.edu/.bin/fbidq.html?ksr), [bi](http://flybase.bio.indiana.edu/.bin/fbidq.html?bi), [chn](http://flybase.bio.indiana.edu/.bin/fbidq.html?chn), [Gsc](http://flybase.bio.indiana.edu/.bin/fbidq.html?Gsc), [Hcf](http://flybase.bio.indiana.edu/.bin/fbidq.html?Hcf), [lola](http://flybase.bio.indiana.edu/.bin/fbidq.html?lola), [HLH4C](http://flybase.bio.indiana.edu/.bin/fbidq.html?HLH4C), [Pcl](http://flybase.bio.indiana.edu/.bin/fbidq.html?Pcl), [chif](http://flybase.bio.indiana.edu/.bin/fbidq.html?chif), [simj](http://flybase.bio.indiana.edu/.bin/fbidq.html?simj), [Awh](http://flybase.bio.indiana.edu/.bin/fbidq.html?Awh), [tap](http://flybase.bio.indiana.edu/.bin/fbidq.html?tap), [NC2alpha](http://flybase.bio.indiana.edu/.bin/fbidq.html?NC2alpha), [PHDP](http://flybase.bio.indiana.edu/.bin/fbidq.html?PHDP), [bab1](http://flybase.bio.indiana.edu/.bin/fbidq.html?bab1), [CG4328](http://flybase.bio.indiana.edu/.bin/fbidq.html?CG4328), [Snoo](http://flybase.bio.indiana.edu/.bin/fbidq.html?Snoo), [l(2)NC136](http://flybase.bio.indiana.edu/.bin/fbidq.html?l(2)NC136), [Taf4](http://flybase.bio.indiana.edu/.bin/fbidq.html?Taf4), [Su(var)3-3](http://flybase.bio.indiana.edu/.bin/fbidq.html?Su(var)3-3), [HGTX](http://flybase.bio.indiana.edu/.bin/fbidq.html?HGTX), [not](http://flybase.bio.indiana.edu/.bin/fbidq.html?not), [nvy](http://flybase.bio.indiana.edu/.bin/fbidq.html?nvy), [nub](http://flybase.bio.indiana.edu/.bin/fbidq.html?nub), [nab](http://flybase.bio.indiana.edu/.bin/fbidq.html?nab),[btd](http://flybase.bio.indiana.edu/.bin/fbidq.html?btd), [fd68A](http://flybase.bio.indiana.edu/.bin/fbidq.html?fd68A), [HLHmdelta](http://flybase.bio.indiana.edu/.bin/fbidq.html?HLHmdelta), [B-H1](http://flybase.bio.indiana.edu/.bin/fbidq.html?B-H1), [Nipped-A](http://flybase.bio.indiana.edu/.bin/fbidq.html?Nipped-A), [ATbp](http://flybase.bio.indiana.edu/.bin/fbidq.html?ATbp), [stau](http://flybase.bio.indiana.edu/.bin/fbidq.html?stau), [Sos](http://flybase.bio.indiana.edu/.bin/fbidq.html?Sos), [Fer2](http://flybase.bio.indiana.edu/.bin/fbidq.html?Fer2), [fd102C](http://flybase.bio.indiana.edu/.bin/fbidq.html?fd102C), [vg](http://flybase.bio.indiana.edu/.bin/fbidq.html?vg), [hth](http://flybase.bio.indiana.edu/.bin/fbidq.html?hth), [Nf-YC](http://flybase.bio.indiana.edu/.bin/fbidq.html?Nf-YC), [eya](http://flybase.bio.indiana.edu/.bin/fbidq.html?eya), [fz](http://flybase.bio.indiana.edu/.bin/fbidq.html?fz), [brm](http://flybase.bio.indiana.edu/.bin/fbidq.html?brm), [Vsx2](http://flybase.bio.indiana.edu/.bin/fbidq.html?Vsx2), [Hey](http://flybase.bio.indiana.edu/.bin/fbidq.html?Hey), [sno](http://flybase.bio.indiana.edu/.bin/fbidq.html?sno), [Sfmbt](http://flybase.bio.indiana.edu/.bin/fbidq.html?Sfmbt), [ara](http://flybase.bio.indiana.edu/.bin/fbidq.html?ara), [mam](http://flybase.bio.indiana.edu/.bin/fbidq.html?mam), [pan](http://flybase.bio.indiana.edu/.bin/fbidq.html?pan), [Dll](http://flybase.bio.indiana.edu/.bin/fbidq.html?Dll), [disco](http://flybase.bio.indiana.edu/.bin/fbidq.html?disco), [grn](http://flybase.bio.indiana.edu/.bin/fbidq.html?grn), [HLHm5](http://flybase.bio.indiana.edu/.bin/fbidq.html?HLHm5), [D12](http://flybase.bio.indiana.edu/.bin/fbidq.html?D12), [Lis-1](http://flybase.bio.indiana.edu/.bin/fbidq.html?Lis-1), [CycE](http://flybase.bio.indiana.edu/.bin/fbidq.html?CycE), [Brf](http://flybase.bio.indiana.edu/.bin/fbidq.html?Brf), [ems](http://flybase.bio.indiana.edu/.bin/fbidq.html?ems), [pros](http://flybase.bio.indiana.edu/.bin/fbidq.html?pros), [tup](http://flybase.bio.indiana.edu/.bin/fbidq.html?tup), [pdm2](http://flybase.bio.indiana.edu/.bin/fbidq.html?pdm2), [Gug](http://flybase.bio.indiana.edu/.bin/fbidq.html?Gug), [pad](http://flybase.bio.indiana.edu/.bin/fbidq.html?pad), [rgr](http://flybase.bio.indiana.edu/.bin/fbidq.html?rgr), [orb2](http://flybase.bio.indiana.edu/.bin/fbidq.html?orb2), [Wnt4](http://flybase.bio.indiana.edu/.bin/fbidq.html?Wnt4), [Rx](http://flybase.bio.indiana.edu/.bin/fbidq.html?Rx),[MED1](http://flybase.bio.indiana.edu/.bin/fbidq.html?MED1), [salr](http://flybase.bio.indiana.edu/.bin/fbidq.html?salr), [wdn](http://flybase.bio.indiana.edu/.bin/fbidq.html?wdn), [sim](http://flybase.bio.indiana.edu/.bin/fbidq.html?sim), [d4](http://flybase.bio.indiana.edu/.bin/fbidq.html?d4), [dan](http://flybase.bio.indiana.edu/.bin/fbidq.html?dan), [gro](http://flybase.bio.indiana.edu/.bin/fbidq.html?gro), [nerfin-1](http://flybase.bio.indiana.edu/.bin/fbidq.html?nerfin-1), [bin3](http://flybase.bio.indiana.edu/.bin/fbidq.html?bin3), [trx](http://flybase.bio.indiana.edu/.bin/fbidq.html?trx), [Rpd3](http://flybase.bio.indiana.edu/.bin/fbidq.html?Rpd3), [dsx](http://flybase.bio.indiana.edu/.bin/fbidq.html?dsx), [tyf](http://flybase.bio.indiana.edu/.bin/fbidq.html?tyf), [Ssdp](http://flybase.bio.indiana.edu/.bin/fbidq.html?Ssdp), [vfl](http://flybase.bio.indiana.edu/.bin/fbidq.html?vfl), [sqz](http://flybase.bio.indiana.edu/.bin/fbidq.html?sqz), [CG32105](http://flybase.bio.indiana.edu/.bin/fbidq.html?CG32105), [Fer3](http://flybase.bio.indiana.edu/.bin/fbidq.html?Fer3), [aret](http://flybase.bio.indiana.edu/.bin/fbidq.html?aret), [pygo](http://flybase.bio.indiana.edu/.bin/fbidq.html?pygo), [dalao](http://flybase.bio.indiana.edu/.bin/fbidq.html?dalao), [HLHm7](http://flybase.bio.indiana.edu/.bin/fbidq.html?HLHm7), [z](http://flybase.bio.indiana.edu/.bin/fbidq.html?z), [HLHmgamma](http://flybase.bio.indiana.edu/.bin/fbidq.html?HLHmgamma), [Sox21b](http://flybase.bio.indiana.edu/.bin/fbidq.html?Sox21b), [sr](http://flybase.bio.indiana.edu/.bin/fbidq.html?sr), [Lim1](http://flybase.bio.indiana.edu/.bin/fbidq.html?Lim1), [tld](http://flybase.bio.indiana.edu/.bin/fbidq.html?tld), [danr](http://flybase.bio.indiana.edu/.bin/fbidq.html?danr), [wor](http://flybase.bio.indiana.edu/.bin/fbidq.html?wor), [vvl](http://flybase.bio.indiana.edu/.bin/fbidq.html?vvl), [wda](http://flybase.bio.indiana.edu/.bin/fbidq.html?wda), [sgg](http://flybase.bio.indiana.edu/.bin/fbidq.html?sgg), [MED14](http://flybase.bio.indiana.edu/.bin/fbidq.html?MED14), [gcm2](http://flybase.bio.indiana.edu/.bin/fbidq.html?gcm2), [Eip93F](http://flybase.bio.indiana.edu/.bin/fbidq.html?Eip93F), [gcm](http://flybase.bio.indiana.edu/.bin/fbidq.html?gcm), [seq](http://flybase.bio.indiana.edu/.bin/fbidq.html?seq), [Dip3](http://flybase.bio.indiana.edu/.bin/fbidq.html?Dip3), [Aef1](http://flybase.bio.indiana.edu/.bin/fbidq.html?Aef1),[HDAC4](http://flybase.bio.indiana.edu/.bin/fbidq.html?HDAC4), [can](http://flybase.bio.indiana.edu/.bin/fbidq.html?can), [elk](http://flybase.bio.indiana.edu/.bin/fbidq.html?elk), [Hsp67Bc](http://flybase.bio.indiana.edu/.bin/fbidq.html?Hsp67Bc), [phl](http://flybase.bio.indiana.edu/.bin/fbidq.html?phl), [ase](http://flybase.bio.indiana.edu/.bin/fbidq.html?ase), [CG13253](http://flybase.bio.indiana.edu/.bin/fbidq.html?CG13253), [ovo](http://flybase.bio.indiana.edu/.bin/fbidq.html?ovo), [scro](http://flybase.bio.indiana.edu/.bin/fbidq.html?scro), [Dsp1](http://flybase.bio.indiana.edu/.bin/fbidq.html?Dsp1), [CG14216](http://flybase.bio.indiana.edu/.bin/fbidq.html?CG14216), [Pdp1](http://flybase.bio.indiana.edu/.bin/fbidq.html?Pdp1), [Oli](http://flybase.bio.indiana.edu/.bin/fbidq.html?Oli), [Nelf-A](http://flybase.bio.indiana.edu/.bin/fbidq.html?Nelf-A), [GATAd](http://flybase.bio.indiana.edu/.bin/fbidq.html?GATAd), [Poxn](http://flybase.bio.indiana.edu/.bin/fbidq.html?Poxn), [CG15376](http://flybase.bio.indiana.edu/.bin/fbidq.html?CG15376), [Sin3A](http://flybase.bio.indiana.edu/.bin/fbidq.html?Sin3A), [mip120](http://flybase.bio.indiana.edu/.bin/fbidq.html?mip120), [jumu](http://flybase.bio.indiana.edu/.bin/fbidq.html?jumu), [Smox](http://flybase.bio.indiana.edu/.bin/fbidq.html?Smox), [Usp7](http://flybase.bio.indiana.edu/.bin/fbidq.html?Usp7), [ush](http://flybase.bio.indiana.edu/.bin/fbidq.html?ush), [fd59A](http://flybase.bio.indiana.edu/.bin/fbidq.html?fd59A), [CG10336](http://flybase.bio.indiana.edu/.bin/fbidq.html?CG10336), [Gap1](http://flybase.bio.indiana.edu/.bin/fbidq.html?Gap1), [run](http://flybase.bio.indiana.edu/.bin/fbidq.html?run), [FBX011](http://flybase.bio.indiana.edu/.bin/fbidq.html?FBX011), [brat](http://flybase.bio.indiana.edu/.bin/fbidq.html?brat), [Antp](http://flybase.bio.indiana.edu/.bin/fbidq.html?Antp), [tara](http://flybase.bio.indiana.edu/.bin/fbidq.html?tara), [Alh](http://flybase.bio.indiana.edu/.bin/fbidq.html?Alh), [Sox102F](http://flybase.bio.indiana.edu/.bin/fbidq.html?Sox102F), [egg](http://flybase.bio.indiana.edu/.bin/fbidq.html?egg),[l(3)neo38](http://flybase.bio.indiana.edu/.bin/fbidq.html?l(3)neo38), [koko](http://flybase.bio.indiana.edu/.bin/fbidq.html?koko), [Ptth](http://flybase.bio.indiana.edu/.bin/fbidq.html?Ptth), [Sp1](http://flybase.bio.indiana.edu/.bin/fbidq.html?Sp1), [Ptx1](http://flybase.bio.indiana.edu/.bin/fbidq.html?Ptx1), [ph-p](http://flybase.bio.indiana.edu/.bin/fbidq.html?ph-p), [Ubx](http://flybase.bio.indiana.edu/.bin/fbidq.html?Ubx), [onecut](http://flybase.bio.indiana.edu/.bin/fbidq.html?onecut), [CG7757](http://flybase.bio.indiana.edu/.bin/fbidq.html?CG7757), [chm](http://flybase.bio.indiana.edu/.bin/fbidq.html?chm), [sna](http://flybase.bio.indiana.edu/.bin/fbidq.html?sna), [Wnt5](http://flybase.bio.indiana.edu/.bin/fbidq.html?Wnt5), [Taf1](http://flybase.bio.indiana.edu/.bin/fbidq.html?Taf1), [hbn](http://flybase.bio.indiana.edu/.bin/fbidq.html?hbn), [MBD-like](http://flybase.bio.indiana.edu/.bin/fbidq.html?MBD-like), [tsh](http://flybase.bio.indiana.edu/.bin/fbidq.html?tsh), [esg](http://flybase.bio.indiana.edu/.bin/fbidq.html?esg), [e(y)3](http://flybase.bio.indiana.edu/.bin/fbidq.html?e(y)3), [CG9007](http://flybase.bio.indiana.edu/.bin/fbidq.html?CG9007), [oc](http://flybase.bio.indiana.edu/.bin/fbidq.html?oc), [spt4](http://flybase.bio.indiana.edu/.bin/fbidq.html?spt4), [CG32532](http://flybase.bio.indiana.edu/.bin/fbidq.html?CG32532), [MTA1-like](http://flybase.bio.indiana.edu/.bin/fbidq.html?MTA1-like), [gol](http://flybase.bio.indiana.edu/.bin/fbidq.html?gol), [ey](http://flybase.bio.indiana.edu/.bin/fbidq.html?ey), [mle](http://flybase.bio.indiana.edu/.bin/fbidq.html?mle), [Hr51](http://flybase.bio.indiana.edu/.bin/fbidq.html?Hr51), [Bgb](http://flybase.bio.indiana.edu/.bin/fbidq.html?Bgb), [l(1)sc](http://flybase.bio.indiana.edu/.bin/fbidq.html?l(1)sc), [B-H2](http://flybase.bio.indiana.edu/.bin/fbidq.html?B-H2), [grh](http://flybase.bio.indiana.edu/.bin/fbidq.html?grh), [Vsx1](http://flybase.bio.indiana.edu/.bin/fbidq.html?Vsx1), [wg](http://flybase.bio.indiana.edu/.bin/fbidq.html?wg), [toy](http://flybase.bio.indiana.edu/.bin/fbidq.html?toy), [fkh](http://flybase.bio.indiana.edu/.bin/fbidq.html?fkh), [Lim3](http://flybase.bio.indiana.edu/.bin/fbidq.html?Lim3), [crol](http://flybase.bio.indiana.edu/.bin/fbidq.html?crol), [sd](http://flybase.bio.indiana.edu/.bin/fbidq.html?sd), [retn](http://flybase.bio.indiana.edu/.bin/fbidq.html?retn), [en](http://flybase.bio.indiana.edu/.bin/fbidq.html?en),[fs(1)h](http://flybase.bio.indiana.edu/.bin/fbidq.html?fs(1)h), [rl](http://flybase.bio.indiana.edu/.bin/fbidq.html?rl), [CG11347](http://flybase.bio.indiana.edu/.bin/fbidq.html?CG11347), [tey](http://flybase.bio.indiana.edu/.bin/fbidq.html?tey), [CG11294](http://flybase.bio.indiana.edu/.bin/fbidq.html?CG11294), [hiw](http://flybase.bio.indiana.edu/.bin/fbidq.html?hiw), [cos](http://flybase.bio.indiana.edu/.bin/fbidq.html?cos), [Ct](http://flybase.bio.indiana.edu/.bin/fbidq.html?Ct), [Atf-2](http://flybase.bio.indiana.edu/.bin/fbidq.html?Atf-2) |
| [neuropeptide signaling pathway](http://amigo.geneontology.org/cgi-bin/amigo/go.cgi?view=details&query=GO:0007218) | 24 of 1594 genes, 1.5% | 29 of 7634 genes, 0.4% | 1.14e-09 | 0.00% | 0.00 | [CG14375](http://flybase.bio.indiana.edu/.bin/fbidq.html?CG14375), [Leucokinin](http://flybase.bio.indiana.edu/.bin/fbidq.html?Leucokinin), [IFa](http://flybase.bio.indiana.edu/.bin/fbidq.html?IFa), [Dsk](http://flybase.bio.indiana.edu/.bin/fbidq.html?Dsk), [Crz](http://flybase.bio.indiana.edu/.bin/fbidq.html?Crz), [npf](http://flybase.bio.indiana.edu/.bin/fbidq.html?npf), [Cirl](http://flybase.bio.indiana.edu/.bin/fbidq.html?Cirl), [hug](http://flybase.bio.indiana.edu/.bin/fbidq.html?hug), [Fmrf](http://flybase.bio.indiana.edu/.bin/fbidq.html?Fmrf), [Ast](http://flybase.bio.indiana.edu/.bin/fbidq.html?Ast), [Tk](http://flybase.bio.indiana.edu/.bin/fbidq.html?Tk), [Dh](http://flybase.bio.indiana.edu/.bin/fbidq.html?Dh), [Dms](http://flybase.bio.indiana.edu/.bin/fbidq.html?Dms), [ETH](http://flybase.bio.indiana.edu/.bin/fbidq.html?ETH), [Nplp1](http://flybase.bio.indiana.edu/.bin/fbidq.html?Nplp1), [7B2](http://flybase.bio.indiana.edu/.bin/fbidq.html?7B2), [sNPF-R](http://flybase.bio.indiana.edu/.bin/fbidq.html?sNPF-R), [Eh](http://flybase.bio.indiana.edu/.bin/fbidq.html?Eh), [Dh31](http://flybase.bio.indiana.edu/.bin/fbidq.html?Dh31), [sNPF](http://flybase.bio.indiana.edu/.bin/fbidq.html?sNPF), [Mip](http://flybase.bio.indiana.edu/.bin/fbidq.html?Mip), [stan](http://flybase.bio.indiana.edu/.bin/fbidq.html?stan), [Ast-C](http://flybase.bio.indiana.edu/.bin/fbidq.html?Ast-C), [Ccap](http://flybase.bio.indiana.edu/.bin/fbidq.html?Ccap) |
| [single-organism behavior](http://amigo.geneontology.org/cgi-bin/amigo/go.cgi?view=details&query=GO:0044708) | 111 of 1594 genes, 7.0% | 296 of 7634 genes, 3.9% | 1.15e-09 | 0.00% | 0.00 | [Sh](http://flybase.bio.indiana.edu/.bin/fbidq.html?Sh), [numb](http://flybase.bio.indiana.edu/.bin/fbidq.html?numb), [NaCP60E](http://flybase.bio.indiana.edu/.bin/fbidq.html?NaCP60E), [acj6](http://flybase.bio.indiana.edu/.bin/fbidq.html?acj6), [Dsk](http://flybase.bio.indiana.edu/.bin/fbidq.html?Dsk), [CG14509](http://flybase.bio.indiana.edu/.bin/fbidq.html?CG14509), [rg](http://flybase.bio.indiana.edu/.bin/fbidq.html?rg), [npf](http://flybase.bio.indiana.edu/.bin/fbidq.html?npf), [betaInt-nu](http://flybase.bio.indiana.edu/.bin/fbidq.html?betaInt-nu), [Spn](http://flybase.bio.indiana.edu/.bin/fbidq.html?Spn), [sgg](http://flybase.bio.indiana.edu/.bin/fbidq.html?sgg), [CG17734](http://flybase.bio.indiana.edu/.bin/fbidq.html?CG17734), [mnb](http://flybase.bio.indiana.edu/.bin/fbidq.html?mnb), [dpr9](http://flybase.bio.indiana.edu/.bin/fbidq.html?dpr9), [Nmdar1](http://flybase.bio.indiana.edu/.bin/fbidq.html?Nmdar1), [Nf1](http://flybase.bio.indiana.edu/.bin/fbidq.html?Nf1), [Pka-R1](http://flybase.bio.indiana.edu/.bin/fbidq.html?Pka-R1), [tay](http://flybase.bio.indiana.edu/.bin/fbidq.html?tay), [ovo](http://flybase.bio.indiana.edu/.bin/fbidq.html?ovo), [sol](http://flybase.bio.indiana.edu/.bin/fbidq.html?sol), [os](http://flybase.bio.indiana.edu/.bin/fbidq.html?os), [sNPF](http://flybase.bio.indiana.edu/.bin/fbidq.html?sNPF), [hang](http://flybase.bio.indiana.edu/.bin/fbidq.html?hang), [brp](http://flybase.bio.indiana.edu/.bin/fbidq.html?brp), [CASK](http://flybase.bio.indiana.edu/.bin/fbidq.html?CASK), [nonA](http://flybase.bio.indiana.edu/.bin/fbidq.html?nonA), [CG10251](http://flybase.bio.indiana.edu/.bin/fbidq.html?CG10251), [lola](http://flybase.bio.indiana.edu/.bin/fbidq.html?lola), [unc-104](http://flybase.bio.indiana.edu/.bin/fbidq.html?unc-104), [mtt](http://flybase.bio.indiana.edu/.bin/fbidq.html?mtt), [Alk](http://flybase.bio.indiana.edu/.bin/fbidq.html?Alk), [Poxn](http://flybase.bio.indiana.edu/.bin/fbidq.html?Poxn), [Dat](http://flybase.bio.indiana.edu/.bin/fbidq.html?Dat), [Appl](http://flybase.bio.indiana.edu/.bin/fbidq.html?Appl), [CG4476](http://flybase.bio.indiana.edu/.bin/fbidq.html?CG4476), [Galpha49B](http://flybase.bio.indiana.edu/.bin/fbidq.html?Galpha49B),[scrib](http://flybase.bio.indiana.edu/.bin/fbidq.html?scrib), [rut](http://flybase.bio.indiana.edu/.bin/fbidq.html?rut), [gfA](http://flybase.bio.indiana.edu/.bin/fbidq.html?gfA), [Plc21C](http://flybase.bio.indiana.edu/.bin/fbidq.html?Plc21C), [Tk](http://flybase.bio.indiana.edu/.bin/fbidq.html?Tk), [nab](http://flybase.bio.indiana.edu/.bin/fbidq.html?nab), [Ddc](http://flybase.bio.indiana.edu/.bin/fbidq.html?Ddc), [amon](http://flybase.bio.indiana.edu/.bin/fbidq.html?amon), [DAT](http://flybase.bio.indiana.edu/.bin/fbidq.html?DAT), [stau](http://flybase.bio.indiana.edu/.bin/fbidq.html?stau), [Rdl](http://flybase.bio.indiana.edu/.bin/fbidq.html?Rdl), [Sema-1a](http://flybase.bio.indiana.edu/.bin/fbidq.html?Sema-1a), [Eh](http://flybase.bio.indiana.edu/.bin/fbidq.html?Eh), [TBPH](http://flybase.bio.indiana.edu/.bin/fbidq.html?TBPH), [Fer2](http://flybase.bio.indiana.edu/.bin/fbidq.html?Fer2), [CG34400](http://flybase.bio.indiana.edu/.bin/fbidq.html?CG34400), [GABA-B-R2](http://flybase.bio.indiana.edu/.bin/fbidq.html?GABA-B-R2), [tutl](http://flybase.bio.indiana.edu/.bin/fbidq.html?tutl), [lid](http://flybase.bio.indiana.edu/.bin/fbidq.html?lid), [Fas2](http://flybase.bio.indiana.edu/.bin/fbidq.html?Fas2), [14-3-3zeta](http://flybase.bio.indiana.edu/.bin/fbidq.html?14-3-3zeta), [robo](http://flybase.bio.indiana.edu/.bin/fbidq.html?robo), [futsch](http://flybase.bio.indiana.edu/.bin/fbidq.html?futsch), [Ilp2](http://flybase.bio.indiana.edu/.bin/fbidq.html?Ilp2), [shakB](http://flybase.bio.indiana.edu/.bin/fbidq.html?shakB), [CG3967](http://flybase.bio.indiana.edu/.bin/fbidq.html?CG3967), [qvr](http://flybase.bio.indiana.edu/.bin/fbidq.html?qvr), [pigeon](http://flybase.bio.indiana.edu/.bin/fbidq.html?pigeon), [hug](http://flybase.bio.indiana.edu/.bin/fbidq.html?hug), [smi35A](http://flybase.bio.indiana.edu/.bin/fbidq.html?smi35A), [Gpo-1](http://flybase.bio.indiana.edu/.bin/fbidq.html?Gpo-1), [Ggamma1](http://flybase.bio.indiana.edu/.bin/fbidq.html?Ggamma1), [jet](http://flybase.bio.indiana.edu/.bin/fbidq.html?jet), [para](http://flybase.bio.indiana.edu/.bin/fbidq.html?para), [Dll](http://flybase.bio.indiana.edu/.bin/fbidq.html?Dll), [disco](http://flybase.bio.indiana.edu/.bin/fbidq.html?disco),[sm](http://flybase.bio.indiana.edu/.bin/fbidq.html?sm), [DopR](http://flybase.bio.indiana.edu/.bin/fbidq.html?DopR), [neur](http://flybase.bio.indiana.edu/.bin/fbidq.html?neur), [esg](http://flybase.bio.indiana.edu/.bin/fbidq.html?esg), [eas](http://flybase.bio.indiana.edu/.bin/fbidq.html?eas), [Nrx-1](http://flybase.bio.indiana.edu/.bin/fbidq.html?Nrx-1), [CRMP](http://flybase.bio.indiana.edu/.bin/fbidq.html?CRMP), [Cdk5](http://flybase.bio.indiana.edu/.bin/fbidq.html?Cdk5), [5-HT1A](http://flybase.bio.indiana.edu/.bin/fbidq.html?5-HT1A), [oc](http://flybase.bio.indiana.edu/.bin/fbidq.html?oc), [Bx](http://flybase.bio.indiana.edu/.bin/fbidq.html?Bx), [Mob2](http://flybase.bio.indiana.edu/.bin/fbidq.html?Mob2), [Tbh](http://flybase.bio.indiana.edu/.bin/fbidq.html?Tbh), [ey](http://flybase.bio.indiana.edu/.bin/fbidq.html?ey), [Actn](http://flybase.bio.indiana.edu/.bin/fbidq.html?Actn), [Oamb](http://flybase.bio.indiana.edu/.bin/fbidq.html?Oamb), [orb2](http://flybase.bio.indiana.edu/.bin/fbidq.html?orb2), [shi](http://flybase.bio.indiana.edu/.bin/fbidq.html?shi), [Gad1](http://flybase.bio.indiana.edu/.bin/fbidq.html?Gad1), [Cdk5alpha](http://flybase.bio.indiana.edu/.bin/fbidq.html?Cdk5alpha), [rogdi](http://flybase.bio.indiana.edu/.bin/fbidq.html?rogdi), [sim](http://flybase.bio.indiana.edu/.bin/fbidq.html?sim), [VGAT](http://flybase.bio.indiana.edu/.bin/fbidq.html?VGAT), [CaMKII](http://flybase.bio.indiana.edu/.bin/fbidq.html?CaMKII), [tau](http://flybase.bio.indiana.edu/.bin/fbidq.html?tau), [CG42629](http://flybase.bio.indiana.edu/.bin/fbidq.html?CG42629), [bin3](http://flybase.bio.indiana.edu/.bin/fbidq.html?bin3), [klg](http://flybase.bio.indiana.edu/.bin/fbidq.html?klg), [Btk29A](http://flybase.bio.indiana.edu/.bin/fbidq.html?Btk29A), [Ac3](http://flybase.bio.indiana.edu/.bin/fbidq.html?Ac3), [rl](http://flybase.bio.indiana.edu/.bin/fbidq.html?rl), [Syt1](http://flybase.bio.indiana.edu/.bin/fbidq.html?Syt1), [PQBP-1](http://flybase.bio.indiana.edu/.bin/fbidq.html?PQBP-1), [Sip1](http://flybase.bio.indiana.edu/.bin/fbidq.html?Sip1), [tyf](http://flybase.bio.indiana.edu/.bin/fbidq.html?tyf), [klu](http://flybase.bio.indiana.edu/.bin/fbidq.html?klu), [NPFR1](http://flybase.bio.indiana.edu/.bin/fbidq.html?NPFR1), [HLHm7](http://flybase.bio.indiana.edu/.bin/fbidq.html?HLHm7), [hiw](http://flybase.bio.indiana.edu/.bin/fbidq.html?hiw) |
| [transcription, DNA-dependent](http://amigo.geneontology.org/cgi-bin/amigo/go.cgi?view=details&query=GO:0006351) | 204 of 1594 genes, 12.8% | 659 of 7634 genes, 8.6% | 1.78e-09 | 0.00% | 0.00 | [fz2](http://flybase.bio.indiana.edu/.bin/fbidq.html?fz2), [acj6](http://flybase.bio.indiana.edu/.bin/fbidq.html?acj6), [skd](http://flybase.bio.indiana.edu/.bin/fbidq.html?skd), [jing](http://flybase.bio.indiana.edu/.bin/fbidq.html?jing), [caup](http://flybase.bio.indiana.edu/.bin/fbidq.html?caup), [tll](http://flybase.bio.indiana.edu/.bin/fbidq.html?tll), [Camta](http://flybase.bio.indiana.edu/.bin/fbidq.html?Camta), [ato](http://flybase.bio.indiana.edu/.bin/fbidq.html?ato), [Ets65A](http://flybase.bio.indiana.edu/.bin/fbidq.html?Ets65A), [tna](http://flybase.bio.indiana.edu/.bin/fbidq.html?tna), [JIL-1](http://flybase.bio.indiana.edu/.bin/fbidq.html?JIL-1), [Spt6](http://flybase.bio.indiana.edu/.bin/fbidq.html?Spt6), [Iswi](http://flybase.bio.indiana.edu/.bin/fbidq.html?Iswi), [rn](http://flybase.bio.indiana.edu/.bin/fbidq.html?rn), [ap](http://flybase.bio.indiana.edu/.bin/fbidq.html?ap), [br](http://flybase.bio.indiana.edu/.bin/fbidq.html?br), [ksr](http://flybase.bio.indiana.edu/.bin/fbidq.html?ksr), [PNUTS](http://flybase.bio.indiana.edu/.bin/fbidq.html?PNUTS), [bi](http://flybase.bio.indiana.edu/.bin/fbidq.html?bi), [chn](http://flybase.bio.indiana.edu/.bin/fbidq.html?chn), [Gsc](http://flybase.bio.indiana.edu/.bin/fbidq.html?Gsc), [Hcf](http://flybase.bio.indiana.edu/.bin/fbidq.html?Hcf), [lola](http://flybase.bio.indiana.edu/.bin/fbidq.html?lola), [HLH4C](http://flybase.bio.indiana.edu/.bin/fbidq.html?HLH4C), [Pcl](http://flybase.bio.indiana.edu/.bin/fbidq.html?Pcl), [simj](http://flybase.bio.indiana.edu/.bin/fbidq.html?simj), [Awh](http://flybase.bio.indiana.edu/.bin/fbidq.html?Awh), [tap](http://flybase.bio.indiana.edu/.bin/fbidq.html?tap), [CG13550](http://flybase.bio.indiana.edu/.bin/fbidq.html?CG13550), [NC2alpha](http://flybase.bio.indiana.edu/.bin/fbidq.html?NC2alpha), [PHDP](http://flybase.bio.indiana.edu/.bin/fbidq.html?PHDP), [bab1](http://flybase.bio.indiana.edu/.bin/fbidq.html?bab1), [Taf12L](http://flybase.bio.indiana.edu/.bin/fbidq.html?Taf12L), [CG4328](http://flybase.bio.indiana.edu/.bin/fbidq.html?CG4328), [Snoo](http://flybase.bio.indiana.edu/.bin/fbidq.html?Snoo), [l(2)NC136](http://flybase.bio.indiana.edu/.bin/fbidq.html?l(2)NC136), [Taf4](http://flybase.bio.indiana.edu/.bin/fbidq.html?Taf4), [Su(var)3-3](http://flybase.bio.indiana.edu/.bin/fbidq.html?Su(var)3-3), [HGTX](http://flybase.bio.indiana.edu/.bin/fbidq.html?HGTX),[not](http://flybase.bio.indiana.edu/.bin/fbidq.html?not), [nvy](http://flybase.bio.indiana.edu/.bin/fbidq.html?nvy), [nub](http://flybase.bio.indiana.edu/.bin/fbidq.html?nub), [nab](http://flybase.bio.indiana.edu/.bin/fbidq.html?nab), [btd](http://flybase.bio.indiana.edu/.bin/fbidq.html?btd), [fd68A](http://flybase.bio.indiana.edu/.bin/fbidq.html?fd68A), [HLHmdelta](http://flybase.bio.indiana.edu/.bin/fbidq.html?HLHmdelta), [B-H1](http://flybase.bio.indiana.edu/.bin/fbidq.html?B-H1), [Nipped-A](http://flybase.bio.indiana.edu/.bin/fbidq.html?Nipped-A), [ATbp](http://flybase.bio.indiana.edu/.bin/fbidq.html?ATbp), [Sos](http://flybase.bio.indiana.edu/.bin/fbidq.html?Sos), [Fer2](http://flybase.bio.indiana.edu/.bin/fbidq.html?Fer2), [fd102C](http://flybase.bio.indiana.edu/.bin/fbidq.html?fd102C), [vg](http://flybase.bio.indiana.edu/.bin/fbidq.html?vg), [hth](http://flybase.bio.indiana.edu/.bin/fbidq.html?hth), [Nf-YC](http://flybase.bio.indiana.edu/.bin/fbidq.html?Nf-YC), [eya](http://flybase.bio.indiana.edu/.bin/fbidq.html?eya), [fz](http://flybase.bio.indiana.edu/.bin/fbidq.html?fz), [brm](http://flybase.bio.indiana.edu/.bin/fbidq.html?brm), [Vsx2](http://flybase.bio.indiana.edu/.bin/fbidq.html?Vsx2), [Hey](http://flybase.bio.indiana.edu/.bin/fbidq.html?Hey), [sno](http://flybase.bio.indiana.edu/.bin/fbidq.html?sno), [Sfmbt](http://flybase.bio.indiana.edu/.bin/fbidq.html?Sfmbt), [ara](http://flybase.bio.indiana.edu/.bin/fbidq.html?ara), [mam](http://flybase.bio.indiana.edu/.bin/fbidq.html?mam), [pan](http://flybase.bio.indiana.edu/.bin/fbidq.html?pan), [Dll](http://flybase.bio.indiana.edu/.bin/fbidq.html?Dll), [disco](http://flybase.bio.indiana.edu/.bin/fbidq.html?disco), [grn](http://flybase.bio.indiana.edu/.bin/fbidq.html?grn), [HLHm5](http://flybase.bio.indiana.edu/.bin/fbidq.html?HLHm5), [D12](http://flybase.bio.indiana.edu/.bin/fbidq.html?D12), [Lis-1](http://flybase.bio.indiana.edu/.bin/fbidq.html?Lis-1), [Brf](http://flybase.bio.indiana.edu/.bin/fbidq.html?Brf), [ems](http://flybase.bio.indiana.edu/.bin/fbidq.html?ems), [pros](http://flybase.bio.indiana.edu/.bin/fbidq.html?pros), [tup](http://flybase.bio.indiana.edu/.bin/fbidq.html?tup), [pdm2](http://flybase.bio.indiana.edu/.bin/fbidq.html?pdm2), [Gug](http://flybase.bio.indiana.edu/.bin/fbidq.html?Gug), [pad](http://flybase.bio.indiana.edu/.bin/fbidq.html?pad), [rgr](http://flybase.bio.indiana.edu/.bin/fbidq.html?rgr), [Wnt4](http://flybase.bio.indiana.edu/.bin/fbidq.html?Wnt4), [Rx](http://flybase.bio.indiana.edu/.bin/fbidq.html?Rx),[MED1](http://flybase.bio.indiana.edu/.bin/fbidq.html?MED1), [salr](http://flybase.bio.indiana.edu/.bin/fbidq.html?salr), [wdn](http://flybase.bio.indiana.edu/.bin/fbidq.html?wdn), [sim](http://flybase.bio.indiana.edu/.bin/fbidq.html?sim), [d4](http://flybase.bio.indiana.edu/.bin/fbidq.html?d4), [dan](http://flybase.bio.indiana.edu/.bin/fbidq.html?dan), [gro](http://flybase.bio.indiana.edu/.bin/fbidq.html?gro), [nerfin-1](http://flybase.bio.indiana.edu/.bin/fbidq.html?nerfin-1), [trx](http://flybase.bio.indiana.edu/.bin/fbidq.html?trx), [Rpd3](http://flybase.bio.indiana.edu/.bin/fbidq.html?Rpd3), [dsx](http://flybase.bio.indiana.edu/.bin/fbidq.html?dsx), [MED25](http://flybase.bio.indiana.edu/.bin/fbidq.html?MED25), [Ssdp](http://flybase.bio.indiana.edu/.bin/fbidq.html?Ssdp), [vfl](http://flybase.bio.indiana.edu/.bin/fbidq.html?vfl), [RpII140](http://flybase.bio.indiana.edu/.bin/fbidq.html?RpII140), [sqz](http://flybase.bio.indiana.edu/.bin/fbidq.html?sqz), [CG32105](http://flybase.bio.indiana.edu/.bin/fbidq.html?CG32105), [Fer3](http://flybase.bio.indiana.edu/.bin/fbidq.html?Fer3), [pygo](http://flybase.bio.indiana.edu/.bin/fbidq.html?pygo), [dalao](http://flybase.bio.indiana.edu/.bin/fbidq.html?dalao), [HLHm7](http://flybase.bio.indiana.edu/.bin/fbidq.html?HLHm7), [z](http://flybase.bio.indiana.edu/.bin/fbidq.html?z), [HLHmgamma](http://flybase.bio.indiana.edu/.bin/fbidq.html?HLHmgamma), [Sox21b](http://flybase.bio.indiana.edu/.bin/fbidq.html?Sox21b), [sr](http://flybase.bio.indiana.edu/.bin/fbidq.html?sr), [Lim1](http://flybase.bio.indiana.edu/.bin/fbidq.html?Lim1), [tld](http://flybase.bio.indiana.edu/.bin/fbidq.html?tld), [danr](http://flybase.bio.indiana.edu/.bin/fbidq.html?danr), [wor](http://flybase.bio.indiana.edu/.bin/fbidq.html?wor), [vvl](http://flybase.bio.indiana.edu/.bin/fbidq.html?vvl), [wda](http://flybase.bio.indiana.edu/.bin/fbidq.html?wda), [sgg](http://flybase.bio.indiana.edu/.bin/fbidq.html?sgg), [MED14](http://flybase.bio.indiana.edu/.bin/fbidq.html?MED14), [gcm2](http://flybase.bio.indiana.edu/.bin/fbidq.html?gcm2), [Eip93F](http://flybase.bio.indiana.edu/.bin/fbidq.html?Eip93F), [gcm](http://flybase.bio.indiana.edu/.bin/fbidq.html?gcm), [seq](http://flybase.bio.indiana.edu/.bin/fbidq.html?seq), [Dip3](http://flybase.bio.indiana.edu/.bin/fbidq.html?Dip3), [Aef1](http://flybase.bio.indiana.edu/.bin/fbidq.html?Aef1),[HDAC4](http://flybase.bio.indiana.edu/.bin/fbidq.html?HDAC4), [can](http://flybase.bio.indiana.edu/.bin/fbidq.html?can), [elk](http://flybase.bio.indiana.edu/.bin/fbidq.html?elk), [phl](http://flybase.bio.indiana.edu/.bin/fbidq.html?phl), [ase](http://flybase.bio.indiana.edu/.bin/fbidq.html?ase), [CG13253](http://flybase.bio.indiana.edu/.bin/fbidq.html?CG13253), [ovo](http://flybase.bio.indiana.edu/.bin/fbidq.html?ovo), [scro](http://flybase.bio.indiana.edu/.bin/fbidq.html?scro), [Dsp1](http://flybase.bio.indiana.edu/.bin/fbidq.html?Dsp1), [CG14216](http://flybase.bio.indiana.edu/.bin/fbidq.html?CG14216), [Pdp1](http://flybase.bio.indiana.edu/.bin/fbidq.html?Pdp1), [Oli](http://flybase.bio.indiana.edu/.bin/fbidq.html?Oli), [Nelf-A](http://flybase.bio.indiana.edu/.bin/fbidq.html?Nelf-A), [GATAd](http://flybase.bio.indiana.edu/.bin/fbidq.html?GATAd), [Poxn](http://flybase.bio.indiana.edu/.bin/fbidq.html?Poxn), [CG15376](http://flybase.bio.indiana.edu/.bin/fbidq.html?CG15376), [Sin3A](http://flybase.bio.indiana.edu/.bin/fbidq.html?Sin3A), [mip120](http://flybase.bio.indiana.edu/.bin/fbidq.html?mip120), [jumu](http://flybase.bio.indiana.edu/.bin/fbidq.html?jumu), [Smox](http://flybase.bio.indiana.edu/.bin/fbidq.html?Smox), [Usp7](http://flybase.bio.indiana.edu/.bin/fbidq.html?Usp7), [ush](http://flybase.bio.indiana.edu/.bin/fbidq.html?ush), [fd59A](http://flybase.bio.indiana.edu/.bin/fbidq.html?fd59A), [Gap1](http://flybase.bio.indiana.edu/.bin/fbidq.html?Gap1), [run](http://flybase.bio.indiana.edu/.bin/fbidq.html?run), [FBX011](http://flybase.bio.indiana.edu/.bin/fbidq.html?FBX011), [brat](http://flybase.bio.indiana.edu/.bin/fbidq.html?brat), [Antp](http://flybase.bio.indiana.edu/.bin/fbidq.html?Antp), [tara](http://flybase.bio.indiana.edu/.bin/fbidq.html?tara), [Alh](http://flybase.bio.indiana.edu/.bin/fbidq.html?Alh), [Sox102F](http://flybase.bio.indiana.edu/.bin/fbidq.html?Sox102F), [egg](http://flybase.bio.indiana.edu/.bin/fbidq.html?egg), [l(3)neo38](http://flybase.bio.indiana.edu/.bin/fbidq.html?l(3)neo38), [koko](http://flybase.bio.indiana.edu/.bin/fbidq.html?koko), [Ptth](http://flybase.bio.indiana.edu/.bin/fbidq.html?Ptth), [Sp1](http://flybase.bio.indiana.edu/.bin/fbidq.html?Sp1),[Ptx1](http://flybase.bio.indiana.edu/.bin/fbidq.html?Ptx1), [ph-p](http://flybase.bio.indiana.edu/.bin/fbidq.html?ph-p), [Ubx](http://flybase.bio.indiana.edu/.bin/fbidq.html?Ubx), [onecut](http://flybase.bio.indiana.edu/.bin/fbidq.html?onecut), [CG7757](http://flybase.bio.indiana.edu/.bin/fbidq.html?CG7757), [chm](http://flybase.bio.indiana.edu/.bin/fbidq.html?chm), [sna](http://flybase.bio.indiana.edu/.bin/fbidq.html?sna), [Wnt5](http://flybase.bio.indiana.edu/.bin/fbidq.html?Wnt5), [Taf1](http://flybase.bio.indiana.edu/.bin/fbidq.html?Taf1), [hbn](http://flybase.bio.indiana.edu/.bin/fbidq.html?hbn), [MBD-like](http://flybase.bio.indiana.edu/.bin/fbidq.html?MBD-like), [tsh](http://flybase.bio.indiana.edu/.bin/fbidq.html?tsh), [esg](http://flybase.bio.indiana.edu/.bin/fbidq.html?esg), [MED16](http://flybase.bio.indiana.edu/.bin/fbidq.html?MED16), [e(y)3](http://flybase.bio.indiana.edu/.bin/fbidq.html?e(y)3), [dac](http://flybase.bio.indiana.edu/.bin/fbidq.html?dac), [CG9007](http://flybase.bio.indiana.edu/.bin/fbidq.html?CG9007), [oc](http://flybase.bio.indiana.edu/.bin/fbidq.html?oc), [spt4](http://flybase.bio.indiana.edu/.bin/fbidq.html?spt4), [CG32532](http://flybase.bio.indiana.edu/.bin/fbidq.html?CG32532), [MTA1-like](http://flybase.bio.indiana.edu/.bin/fbidq.html?MTA1-like), [gol](http://flybase.bio.indiana.edu/.bin/fbidq.html?gol), [ey](http://flybase.bio.indiana.edu/.bin/fbidq.html?ey), [mle](http://flybase.bio.indiana.edu/.bin/fbidq.html?mle), [Hr51](http://flybase.bio.indiana.edu/.bin/fbidq.html?Hr51), [Bgb](http://flybase.bio.indiana.edu/.bin/fbidq.html?Bgb), [l(1)sc](http://flybase.bio.indiana.edu/.bin/fbidq.html?l(1)sc), [B-H2](http://flybase.bio.indiana.edu/.bin/fbidq.html?B-H2), [grh](http://flybase.bio.indiana.edu/.bin/fbidq.html?grh), [Vsx1](http://flybase.bio.indiana.edu/.bin/fbidq.html?Vsx1), [wg](http://flybase.bio.indiana.edu/.bin/fbidq.html?wg), [toy](http://flybase.bio.indiana.edu/.bin/fbidq.html?toy), [fkh](http://flybase.bio.indiana.edu/.bin/fbidq.html?fkh), [Lim3](http://flybase.bio.indiana.edu/.bin/fbidq.html?Lim3), [crol](http://flybase.bio.indiana.edu/.bin/fbidq.html?crol), [sd](http://flybase.bio.indiana.edu/.bin/fbidq.html?sd), [retn](http://flybase.bio.indiana.edu/.bin/fbidq.html?retn), [en](http://flybase.bio.indiana.edu/.bin/fbidq.html?en), [fs(1)h](http://flybase.bio.indiana.edu/.bin/fbidq.html?fs(1)h), [E(bx)](http://flybase.bio.indiana.edu/.bin/fbidq.html?E(bx)), [rl](http://flybase.bio.indiana.edu/.bin/fbidq.html?rl),[CG11347](http://flybase.bio.indiana.edu/.bin/fbidq.html?CG11347), [tey](http://flybase.bio.indiana.edu/.bin/fbidq.html?tey), [CG11294](http://flybase.bio.indiana.edu/.bin/fbidq.html?CG11294), [hiw](http://flybase.bio.indiana.edu/.bin/fbidq.html?hiw), [cos](http://flybase.bio.indiana.edu/.bin/fbidq.html?cos), [Ct](http://flybase.bio.indiana.edu/.bin/fbidq.html?Ct), [Atf-2](http://flybase.bio.indiana.edu/.bin/fbidq.html?Atf-2) |
| [RNA biosynthetic process](http://amigo.geneontology.org/cgi-bin/amigo/go.cgi?view=details&query=GO:0032774) | 204 of 1594 genes, 12.8% | 661 of 7634 genes, 8.7% | 2.47e-09 | 0.00% | 0.00 | [fz2](http://flybase.bio.indiana.edu/.bin/fbidq.html?fz2), [acj6](http://flybase.bio.indiana.edu/.bin/fbidq.html?acj6), [skd](http://flybase.bio.indiana.edu/.bin/fbidq.html?skd), [jing](http://flybase.bio.indiana.edu/.bin/fbidq.html?jing), [caup](http://flybase.bio.indiana.edu/.bin/fbidq.html?caup), [tll](http://flybase.bio.indiana.edu/.bin/fbidq.html?tll), [Camta](http://flybase.bio.indiana.edu/.bin/fbidq.html?Camta), [ato](http://flybase.bio.indiana.edu/.bin/fbidq.html?ato), [Ets65A](http://flybase.bio.indiana.edu/.bin/fbidq.html?Ets65A), [tna](http://flybase.bio.indiana.edu/.bin/fbidq.html?tna), [JIL-1](http://flybase.bio.indiana.edu/.bin/fbidq.html?JIL-1), [Spt6](http://flybase.bio.indiana.edu/.bin/fbidq.html?Spt6), [Iswi](http://flybase.bio.indiana.edu/.bin/fbidq.html?Iswi), [rn](http://flybase.bio.indiana.edu/.bin/fbidq.html?rn), [ap](http://flybase.bio.indiana.edu/.bin/fbidq.html?ap), [br](http://flybase.bio.indiana.edu/.bin/fbidq.html?br), [ksr](http://flybase.bio.indiana.edu/.bin/fbidq.html?ksr), [PNUTS](http://flybase.bio.indiana.edu/.bin/fbidq.html?PNUTS), [bi](http://flybase.bio.indiana.edu/.bin/fbidq.html?bi), [chn](http://flybase.bio.indiana.edu/.bin/fbidq.html?chn), [Gsc](http://flybase.bio.indiana.edu/.bin/fbidq.html?Gsc), [Hcf](http://flybase.bio.indiana.edu/.bin/fbidq.html?Hcf), [lola](http://flybase.bio.indiana.edu/.bin/fbidq.html?lola), [HLH4C](http://flybase.bio.indiana.edu/.bin/fbidq.html?HLH4C), [Pcl](http://flybase.bio.indiana.edu/.bin/fbidq.html?Pcl), [simj](http://flybase.bio.indiana.edu/.bin/fbidq.html?simj), [Awh](http://flybase.bio.indiana.edu/.bin/fbidq.html?Awh), [tap](http://flybase.bio.indiana.edu/.bin/fbidq.html?tap), [CG13550](http://flybase.bio.indiana.edu/.bin/fbidq.html?CG13550), [NC2alpha](http://flybase.bio.indiana.edu/.bin/fbidq.html?NC2alpha), [PHDP](http://flybase.bio.indiana.edu/.bin/fbidq.html?PHDP), [bab1](http://flybase.bio.indiana.edu/.bin/fbidq.html?bab1), [Taf12L](http://flybase.bio.indiana.edu/.bin/fbidq.html?Taf12L), [CG4328](http://flybase.bio.indiana.edu/.bin/fbidq.html?CG4328), [Snoo](http://flybase.bio.indiana.edu/.bin/fbidq.html?Snoo), [l(2)NC136](http://flybase.bio.indiana.edu/.bin/fbidq.html?l(2)NC136), [Taf4](http://flybase.bio.indiana.edu/.bin/fbidq.html?Taf4), [Su(var)3-3](http://flybase.bio.indiana.edu/.bin/fbidq.html?Su(var)3-3), [HGTX](http://flybase.bio.indiana.edu/.bin/fbidq.html?HGTX),[not](http://flybase.bio.indiana.edu/.bin/fbidq.html?not), [nvy](http://flybase.bio.indiana.edu/.bin/fbidq.html?nvy), [nub](http://flybase.bio.indiana.edu/.bin/fbidq.html?nub), [nab](http://flybase.bio.indiana.edu/.bin/fbidq.html?nab), [btd](http://flybase.bio.indiana.edu/.bin/fbidq.html?btd), [fd68A](http://flybase.bio.indiana.edu/.bin/fbidq.html?fd68A), [HLHmdelta](http://flybase.bio.indiana.edu/.bin/fbidq.html?HLHmdelta), [B-H1](http://flybase.bio.indiana.edu/.bin/fbidq.html?B-H1), [Nipped-A](http://flybase.bio.indiana.edu/.bin/fbidq.html?Nipped-A), [ATbp](http://flybase.bio.indiana.edu/.bin/fbidq.html?ATbp), [Sos](http://flybase.bio.indiana.edu/.bin/fbidq.html?Sos), [Fer2](http://flybase.bio.indiana.edu/.bin/fbidq.html?Fer2), [fd102C](http://flybase.bio.indiana.edu/.bin/fbidq.html?fd102C), [vg](http://flybase.bio.indiana.edu/.bin/fbidq.html?vg), [hth](http://flybase.bio.indiana.edu/.bin/fbidq.html?hth), [Nf-YC](http://flybase.bio.indiana.edu/.bin/fbidq.html?Nf-YC), [eya](http://flybase.bio.indiana.edu/.bin/fbidq.html?eya), [fz](http://flybase.bio.indiana.edu/.bin/fbidq.html?fz), [brm](http://flybase.bio.indiana.edu/.bin/fbidq.html?brm), [Vsx2](http://flybase.bio.indiana.edu/.bin/fbidq.html?Vsx2), [Hey](http://flybase.bio.indiana.edu/.bin/fbidq.html?Hey), [sno](http://flybase.bio.indiana.edu/.bin/fbidq.html?sno), [Sfmbt](http://flybase.bio.indiana.edu/.bin/fbidq.html?Sfmbt), [ara](http://flybase.bio.indiana.edu/.bin/fbidq.html?ara), [mam](http://flybase.bio.indiana.edu/.bin/fbidq.html?mam), [pan](http://flybase.bio.indiana.edu/.bin/fbidq.html?pan), [Dll](http://flybase.bio.indiana.edu/.bin/fbidq.html?Dll), [disco](http://flybase.bio.indiana.edu/.bin/fbidq.html?disco), [grn](http://flybase.bio.indiana.edu/.bin/fbidq.html?grn), [HLHm5](http://flybase.bio.indiana.edu/.bin/fbidq.html?HLHm5), [D12](http://flybase.bio.indiana.edu/.bin/fbidq.html?D12), [Lis-1](http://flybase.bio.indiana.edu/.bin/fbidq.html?Lis-1), [Brf](http://flybase.bio.indiana.edu/.bin/fbidq.html?Brf), [ems](http://flybase.bio.indiana.edu/.bin/fbidq.html?ems), [pros](http://flybase.bio.indiana.edu/.bin/fbidq.html?pros), [tup](http://flybase.bio.indiana.edu/.bin/fbidq.html?tup), [pdm2](http://flybase.bio.indiana.edu/.bin/fbidq.html?pdm2), [Gug](http://flybase.bio.indiana.edu/.bin/fbidq.html?Gug), [pad](http://flybase.bio.indiana.edu/.bin/fbidq.html?pad), [rgr](http://flybase.bio.indiana.edu/.bin/fbidq.html?rgr), [Wnt4](http://flybase.bio.indiana.edu/.bin/fbidq.html?Wnt4), [Rx](http://flybase.bio.indiana.edu/.bin/fbidq.html?Rx),[MED1](http://flybase.bio.indiana.edu/.bin/fbidq.html?MED1), [salr](http://flybase.bio.indiana.edu/.bin/fbidq.html?salr), [wdn](http://flybase.bio.indiana.edu/.bin/fbidq.html?wdn), [sim](http://flybase.bio.indiana.edu/.bin/fbidq.html?sim), [d4](http://flybase.bio.indiana.edu/.bin/fbidq.html?d4), [dan](http://flybase.bio.indiana.edu/.bin/fbidq.html?dan), [gro](http://flybase.bio.indiana.edu/.bin/fbidq.html?gro), [nerfin-1](http://flybase.bio.indiana.edu/.bin/fbidq.html?nerfin-1), [trx](http://flybase.bio.indiana.edu/.bin/fbidq.html?trx), [Rpd3](http://flybase.bio.indiana.edu/.bin/fbidq.html?Rpd3), [dsx](http://flybase.bio.indiana.edu/.bin/fbidq.html?dsx), [MED25](http://flybase.bio.indiana.edu/.bin/fbidq.html?MED25), [Ssdp](http://flybase.bio.indiana.edu/.bin/fbidq.html?Ssdp), [vfl](http://flybase.bio.indiana.edu/.bin/fbidq.html?vfl), [RpII140](http://flybase.bio.indiana.edu/.bin/fbidq.html?RpII140), [sqz](http://flybase.bio.indiana.edu/.bin/fbidq.html?sqz), [CG32105](http://flybase.bio.indiana.edu/.bin/fbidq.html?CG32105), [Fer3](http://flybase.bio.indiana.edu/.bin/fbidq.html?Fer3), [pygo](http://flybase.bio.indiana.edu/.bin/fbidq.html?pygo), [dalao](http://flybase.bio.indiana.edu/.bin/fbidq.html?dalao), [HLHm7](http://flybase.bio.indiana.edu/.bin/fbidq.html?HLHm7), [z](http://flybase.bio.indiana.edu/.bin/fbidq.html?z), [HLHmgamma](http://flybase.bio.indiana.edu/.bin/fbidq.html?HLHmgamma), [Sox21b](http://flybase.bio.indiana.edu/.bin/fbidq.html?Sox21b), [sr](http://flybase.bio.indiana.edu/.bin/fbidq.html?sr), [Lim1](http://flybase.bio.indiana.edu/.bin/fbidq.html?Lim1), [tld](http://flybase.bio.indiana.edu/.bin/fbidq.html?tld), [danr](http://flybase.bio.indiana.edu/.bin/fbidq.html?danr), [wor](http://flybase.bio.indiana.edu/.bin/fbidq.html?wor), [vvl](http://flybase.bio.indiana.edu/.bin/fbidq.html?vvl), [wda](http://flybase.bio.indiana.edu/.bin/fbidq.html?wda), [sgg](http://flybase.bio.indiana.edu/.bin/fbidq.html?sgg), [MED14](http://flybase.bio.indiana.edu/.bin/fbidq.html?MED14), [gcm2](http://flybase.bio.indiana.edu/.bin/fbidq.html?gcm2), [Eip93F](http://flybase.bio.indiana.edu/.bin/fbidq.html?Eip93F), [gcm](http://flybase.bio.indiana.edu/.bin/fbidq.html?gcm), [seq](http://flybase.bio.indiana.edu/.bin/fbidq.html?seq), [Dip3](http://flybase.bio.indiana.edu/.bin/fbidq.html?Dip3), [Aef1](http://flybase.bio.indiana.edu/.bin/fbidq.html?Aef1),[HDAC4](http://flybase.bio.indiana.edu/.bin/fbidq.html?HDAC4), [can](http://flybase.bio.indiana.edu/.bin/fbidq.html?can), [elk](http://flybase.bio.indiana.edu/.bin/fbidq.html?elk), [phl](http://flybase.bio.indiana.edu/.bin/fbidq.html?phl), [ase](http://flybase.bio.indiana.edu/.bin/fbidq.html?ase), [CG13253](http://flybase.bio.indiana.edu/.bin/fbidq.html?CG13253), [ovo](http://flybase.bio.indiana.edu/.bin/fbidq.html?ovo), [scro](http://flybase.bio.indiana.edu/.bin/fbidq.html?scro), [Dsp1](http://flybase.bio.indiana.edu/.bin/fbidq.html?Dsp1), [CG14216](http://flybase.bio.indiana.edu/.bin/fbidq.html?CG14216), [Pdp1](http://flybase.bio.indiana.edu/.bin/fbidq.html?Pdp1), [Oli](http://flybase.bio.indiana.edu/.bin/fbidq.html?Oli), [Nelf-A](http://flybase.bio.indiana.edu/.bin/fbidq.html?Nelf-A), [GATAd](http://flybase.bio.indiana.edu/.bin/fbidq.html?GATAd), [Poxn](http://flybase.bio.indiana.edu/.bin/fbidq.html?Poxn), [CG15376](http://flybase.bio.indiana.edu/.bin/fbidq.html?CG15376), [Sin3A](http://flybase.bio.indiana.edu/.bin/fbidq.html?Sin3A), [mip120](http://flybase.bio.indiana.edu/.bin/fbidq.html?mip120), [jumu](http://flybase.bio.indiana.edu/.bin/fbidq.html?jumu), [Smox](http://flybase.bio.indiana.edu/.bin/fbidq.html?Smox), [Usp7](http://flybase.bio.indiana.edu/.bin/fbidq.html?Usp7), [ush](http://flybase.bio.indiana.edu/.bin/fbidq.html?ush), [fd59A](http://flybase.bio.indiana.edu/.bin/fbidq.html?fd59A), [Gap1](http://flybase.bio.indiana.edu/.bin/fbidq.html?Gap1), [run](http://flybase.bio.indiana.edu/.bin/fbidq.html?run), [FBX011](http://flybase.bio.indiana.edu/.bin/fbidq.html?FBX011), [brat](http://flybase.bio.indiana.edu/.bin/fbidq.html?brat), [Antp](http://flybase.bio.indiana.edu/.bin/fbidq.html?Antp), [tara](http://flybase.bio.indiana.edu/.bin/fbidq.html?tara), [Alh](http://flybase.bio.indiana.edu/.bin/fbidq.html?Alh), [Sox102F](http://flybase.bio.indiana.edu/.bin/fbidq.html?Sox102F), [egg](http://flybase.bio.indiana.edu/.bin/fbidq.html?egg), [l(3)neo38](http://flybase.bio.indiana.edu/.bin/fbidq.html?l(3)neo38), [koko](http://flybase.bio.indiana.edu/.bin/fbidq.html?koko), [Ptth](http://flybase.bio.indiana.edu/.bin/fbidq.html?Ptth), [Sp1](http://flybase.bio.indiana.edu/.bin/fbidq.html?Sp1),[Ptx1](http://flybase.bio.indiana.edu/.bin/fbidq.html?Ptx1), [ph-p](http://flybase.bio.indiana.edu/.bin/fbidq.html?ph-p), [Ubx](http://flybase.bio.indiana.edu/.bin/fbidq.html?Ubx), [onecut](http://flybase.bio.indiana.edu/.bin/fbidq.html?onecut), [CG7757](http://flybase.bio.indiana.edu/.bin/fbidq.html?CG7757), [chm](http://flybase.bio.indiana.edu/.bin/fbidq.html?chm), [sna](http://flybase.bio.indiana.edu/.bin/fbidq.html?sna), [Wnt5](http://flybase.bio.indiana.edu/.bin/fbidq.html?Wnt5), [Taf1](http://flybase.bio.indiana.edu/.bin/fbidq.html?Taf1), [hbn](http://flybase.bio.indiana.edu/.bin/fbidq.html?hbn), [MBD-like](http://flybase.bio.indiana.edu/.bin/fbidq.html?MBD-like), [tsh](http://flybase.bio.indiana.edu/.bin/fbidq.html?tsh), [esg](http://flybase.bio.indiana.edu/.bin/fbidq.html?esg), [MED16](http://flybase.bio.indiana.edu/.bin/fbidq.html?MED16), [e(y)3](http://flybase.bio.indiana.edu/.bin/fbidq.html?e(y)3), [dac](http://flybase.bio.indiana.edu/.bin/fbidq.html?dac), [CG9007](http://flybase.bio.indiana.edu/.bin/fbidq.html?CG9007), [oc](http://flybase.bio.indiana.edu/.bin/fbidq.html?oc), [spt4](http://flybase.bio.indiana.edu/.bin/fbidq.html?spt4), [CG32532](http://flybase.bio.indiana.edu/.bin/fbidq.html?CG32532), [MTA1-like](http://flybase.bio.indiana.edu/.bin/fbidq.html?MTA1-like), [gol](http://flybase.bio.indiana.edu/.bin/fbidq.html?gol), [ey](http://flybase.bio.indiana.edu/.bin/fbidq.html?ey), [mle](http://flybase.bio.indiana.edu/.bin/fbidq.html?mle), [Hr51](http://flybase.bio.indiana.edu/.bin/fbidq.html?Hr51), [Bgb](http://flybase.bio.indiana.edu/.bin/fbidq.html?Bgb), [l(1)sc](http://flybase.bio.indiana.edu/.bin/fbidq.html?l(1)sc), [B-H2](http://flybase.bio.indiana.edu/.bin/fbidq.html?B-H2), [grh](http://flybase.bio.indiana.edu/.bin/fbidq.html?grh), [Vsx1](http://flybase.bio.indiana.edu/.bin/fbidq.html?Vsx1), [wg](http://flybase.bio.indiana.edu/.bin/fbidq.html?wg), [toy](http://flybase.bio.indiana.edu/.bin/fbidq.html?toy), [fkh](http://flybase.bio.indiana.edu/.bin/fbidq.html?fkh), [Lim3](http://flybase.bio.indiana.edu/.bin/fbidq.html?Lim3), [crol](http://flybase.bio.indiana.edu/.bin/fbidq.html?crol), [sd](http://flybase.bio.indiana.edu/.bin/fbidq.html?sd), [retn](http://flybase.bio.indiana.edu/.bin/fbidq.html?retn), [en](http://flybase.bio.indiana.edu/.bin/fbidq.html?en), [fs(1)h](http://flybase.bio.indiana.edu/.bin/fbidq.html?fs(1)h), [E(bx)](http://flybase.bio.indiana.edu/.bin/fbidq.html?E(bx)), [rl](http://flybase.bio.indiana.edu/.bin/fbidq.html?rl),[CG11347](http://flybase.bio.indiana.edu/.bin/fbidq.html?CG11347), [tey](http://flybase.bio.indiana.edu/.bin/fbidq.html?tey), [CG11294](http://flybase.bio.indiana.edu/.bin/fbidq.html?CG11294), [hiw](http://flybase.bio.indiana.edu/.bin/fbidq.html?hiw), [cos](http://flybase.bio.indiana.edu/.bin/fbidq.html?cos), [Ct](http://flybase.bio.indiana.edu/.bin/fbidq.html?Ct), [Atf-2](http://flybase.bio.indiana.edu/.bin/fbidq.html?Atf-2) |
| [cellular component morphogenesis](http://amigo.geneontology.org/cgi-bin/amigo/go.cgi?view=details&query=GO:0032989) | 186 of 1594 genes, 11.7% | 597 of 7634 genes, 7.8% | 1.14e-08 | 0.00% | 0.00 | [fz2](http://flybase.bio.indiana.edu/.bin/fbidq.html?fz2), [Sh](http://flybase.bio.indiana.edu/.bin/fbidq.html?Sh), [acj6](http://flybase.bio.indiana.edu/.bin/fbidq.html?acj6), [jing](http://flybase.bio.indiana.edu/.bin/fbidq.html?jing), [dock](http://flybase.bio.indiana.edu/.bin/fbidq.html?dock), [CG42256](http://flybase.bio.indiana.edu/.bin/fbidq.html?CG42256), [CG3703](http://flybase.bio.indiana.edu/.bin/fbidq.html?CG3703), [ato](http://flybase.bio.indiana.edu/.bin/fbidq.html?ato), [Iswi](http://flybase.bio.indiana.edu/.bin/fbidq.html?Iswi), [ap](http://flybase.bio.indiana.edu/.bin/fbidq.html?ap), [Dg](http://flybase.bio.indiana.edu/.bin/fbidq.html?Dg), [Trim9](http://flybase.bio.indiana.edu/.bin/fbidq.html?Trim9), [tlk](http://flybase.bio.indiana.edu/.bin/fbidq.html?tlk), [CG7154](http://flybase.bio.indiana.edu/.bin/fbidq.html?CG7154), [neo](http://flybase.bio.indiana.edu/.bin/fbidq.html?neo), [robo3](http://flybase.bio.indiana.edu/.bin/fbidq.html?robo3), [os](http://flybase.bio.indiana.edu/.bin/fbidq.html?os), [CG3618](http://flybase.bio.indiana.edu/.bin/fbidq.html?CG3618), [tok](http://flybase.bio.indiana.edu/.bin/fbidq.html?tok), [Ptp99A](http://flybase.bio.indiana.edu/.bin/fbidq.html?Ptp99A), [lola](http://flybase.bio.indiana.edu/.bin/fbidq.html?lola), [unc-104](http://flybase.bio.indiana.edu/.bin/fbidq.html?unc-104), [CG31961](http://flybase.bio.indiana.edu/.bin/fbidq.html?CG31961), [chif](http://flybase.bio.indiana.edu/.bin/fbidq.html?chif), [NetB](http://flybase.bio.indiana.edu/.bin/fbidq.html?NetB), [p130CAS](http://flybase.bio.indiana.edu/.bin/fbidq.html?p130CAS), [Tango10](http://flybase.bio.indiana.edu/.bin/fbidq.html?Tango10), [Oseg4](http://flybase.bio.indiana.edu/.bin/fbidq.html?Oseg4), [Appl](http://flybase.bio.indiana.edu/.bin/fbidq.html?Appl), [metro](http://flybase.bio.indiana.edu/.bin/fbidq.html?metro), [CG32137](http://flybase.bio.indiana.edu/.bin/fbidq.html?CG32137), [Nrk](http://flybase.bio.indiana.edu/.bin/fbidq.html?Nrk), [CG4328](http://flybase.bio.indiana.edu/.bin/fbidq.html?CG4328), [Galpha49B](http://flybase.bio.indiana.edu/.bin/fbidq.html?Galpha49B), [Lar](http://flybase.bio.indiana.edu/.bin/fbidq.html?Lar),[Taf4](http://flybase.bio.indiana.edu/.bin/fbidq.html?Taf4), [CG10249](http://flybase.bio.indiana.edu/.bin/fbidq.html?CG10249), [scrib](http://flybase.bio.indiana.edu/.bin/fbidq.html?scrib), [not](http://flybase.bio.indiana.edu/.bin/fbidq.html?not), [rut](http://flybase.bio.indiana.edu/.bin/fbidq.html?rut), [nvy](http://flybase.bio.indiana.edu/.bin/fbidq.html?nvy), [Nrg](http://flybase.bio.indiana.edu/.bin/fbidq.html?Nrg), [gogo](http://flybase.bio.indiana.edu/.bin/fbidq.html?gogo), [jeb](http://flybase.bio.indiana.edu/.bin/fbidq.html?jeb), [GluClalpha](http://flybase.bio.indiana.edu/.bin/fbidq.html?GluClalpha), [Actbeta](http://flybase.bio.indiana.edu/.bin/fbidq.html?Actbeta), [Sema-1a](http://flybase.bio.indiana.edu/.bin/fbidq.html?Sema-1a), [beat-Ib](http://flybase.bio.indiana.edu/.bin/fbidq.html?beat-Ib), [Traf4](http://flybase.bio.indiana.edu/.bin/fbidq.html?Traf4), [TBPH](http://flybase.bio.indiana.edu/.bin/fbidq.html?TBPH), [otk](http://flybase.bio.indiana.edu/.bin/fbidq.html?otk), [Sos](http://flybase.bio.indiana.edu/.bin/fbidq.html?Sos), [CG34400](http://flybase.bio.indiana.edu/.bin/fbidq.html?CG34400), [Ulp1](http://flybase.bio.indiana.edu/.bin/fbidq.html?Ulp1), [ena](http://flybase.bio.indiana.edu/.bin/fbidq.html?ena), [dsh](http://flybase.bio.indiana.edu/.bin/fbidq.html?dsh), [Fas2](http://flybase.bio.indiana.edu/.bin/fbidq.html?Fas2), [Nf-YC](http://flybase.bio.indiana.edu/.bin/fbidq.html?Nf-YC), [fz](http://flybase.bio.indiana.edu/.bin/fbidq.html?fz), [robo](http://flybase.bio.indiana.edu/.bin/fbidq.html?robo), [lea](http://flybase.bio.indiana.edu/.bin/fbidq.html?lea), [bsk](http://flybase.bio.indiana.edu/.bin/fbidq.html?bsk), [brm](http://flybase.bio.indiana.edu/.bin/fbidq.html?brm), [beat-Ia](http://flybase.bio.indiana.edu/.bin/fbidq.html?beat-Ia), [kat-60L1](http://flybase.bio.indiana.edu/.bin/fbidq.html?kat-60L1), [ft](http://flybase.bio.indiana.edu/.bin/fbidq.html?ft), [RhoGAPp190](http://flybase.bio.indiana.edu/.bin/fbidq.html?RhoGAPp190), [plexB](http://flybase.bio.indiana.edu/.bin/fbidq.html?plexB), [Klp64D](http://flybase.bio.indiana.edu/.bin/fbidq.html?Klp64D), [grn](http://flybase.bio.indiana.edu/.bin/fbidq.html?grn), [DAAM](http://flybase.bio.indiana.edu/.bin/fbidq.html?DAAM),[mew](http://flybase.bio.indiana.edu/.bin/fbidq.html?mew), [Lis-1](http://flybase.bio.indiana.edu/.bin/fbidq.html?Lis-1), [CycE](http://flybase.bio.indiana.edu/.bin/fbidq.html?CycE), [Brf](http://flybase.bio.indiana.edu/.bin/fbidq.html?Brf), [ems](http://flybase.bio.indiana.edu/.bin/fbidq.html?ems), [eys](http://flybase.bio.indiana.edu/.bin/fbidq.html?eys), [Mob2](http://flybase.bio.indiana.edu/.bin/fbidq.html?Mob2), [pros](http://flybase.bio.indiana.edu/.bin/fbidq.html?pros), [tup](http://flybase.bio.indiana.edu/.bin/fbidq.html?tup), [trio](http://flybase.bio.indiana.edu/.bin/fbidq.html?trio), [ed](http://flybase.bio.indiana.edu/.bin/fbidq.html?ed), [Actn](http://flybase.bio.indiana.edu/.bin/fbidq.html?Actn), [CG1463](http://flybase.bio.indiana.edu/.bin/fbidq.html?CG1463), [daw](http://flybase.bio.indiana.edu/.bin/fbidq.html?daw), [Wnt4](http://flybase.bio.indiana.edu/.bin/fbidq.html?Wnt4), [rictor](http://flybase.bio.indiana.edu/.bin/fbidq.html?rictor), [Oseg6](http://flybase.bio.indiana.edu/.bin/fbidq.html?Oseg6), [Apc](http://flybase.bio.indiana.edu/.bin/fbidq.html?Apc), [sim](http://flybase.bio.indiana.edu/.bin/fbidq.html?sim), [d4](http://flybase.bio.indiana.edu/.bin/fbidq.html?d4), [gro](http://flybase.bio.indiana.edu/.bin/fbidq.html?gro), [pigs](http://flybase.bio.indiana.edu/.bin/fbidq.html?pigs), [nerfin-1](http://flybase.bio.indiana.edu/.bin/fbidq.html?nerfin-1), [Mf](http://flybase.bio.indiana.edu/.bin/fbidq.html?Mf), [bchs](http://flybase.bio.indiana.edu/.bin/fbidq.html?bchs), [wnd](http://flybase.bio.indiana.edu/.bin/fbidq.html?wnd), [trx](http://flybase.bio.indiana.edu/.bin/fbidq.html?trx), [Rpd3](http://flybase.bio.indiana.edu/.bin/fbidq.html?Rpd3), [dsx](http://flybase.bio.indiana.edu/.bin/fbidq.html?dsx), [chinmo](http://flybase.bio.indiana.edu/.bin/fbidq.html?chinmo), [CG14367](http://flybase.bio.indiana.edu/.bin/fbidq.html?CG14367), [unc-5](http://flybase.bio.indiana.edu/.bin/fbidq.html?unc-5), [Ssdp](http://flybase.bio.indiana.edu/.bin/fbidq.html?Ssdp), [CG10107](http://flybase.bio.indiana.edu/.bin/fbidq.html?CG10107), [sqz](http://flybase.bio.indiana.edu/.bin/fbidq.html?sqz), [fend](http://flybase.bio.indiana.edu/.bin/fbidq.html?fend), [Cad74A](http://flybase.bio.indiana.edu/.bin/fbidq.html?Cad74A), [pygo](http://flybase.bio.indiana.edu/.bin/fbidq.html?pygo), [HLHm7](http://flybase.bio.indiana.edu/.bin/fbidq.html?HLHm7),[wor](http://flybase.bio.indiana.edu/.bin/fbidq.html?wor), [vvl](http://flybase.bio.indiana.edu/.bin/fbidq.html?vvl), [RhoGEF4](http://flybase.bio.indiana.edu/.bin/fbidq.html?RhoGEF4), [Pten](http://flybase.bio.indiana.edu/.bin/fbidq.html?Pten), [Ptp69D](http://flybase.bio.indiana.edu/.bin/fbidq.html?Ptp69D), [sgg](http://flybase.bio.indiana.edu/.bin/fbidq.html?sgg), [gcm2](http://flybase.bio.indiana.edu/.bin/fbidq.html?gcm2), [hts](http://flybase.bio.indiana.edu/.bin/fbidq.html?hts), [mp](http://flybase.bio.indiana.edu/.bin/fbidq.html?mp), [gcm](http://flybase.bio.indiana.edu/.bin/fbidq.html?gcm), [seq](http://flybase.bio.indiana.edu/.bin/fbidq.html?seq), [CG5890](http://flybase.bio.indiana.edu/.bin/fbidq.html?CG5890), [CG32685](http://flybase.bio.indiana.edu/.bin/fbidq.html?CG32685), [CG4893](http://flybase.bio.indiana.edu/.bin/fbidq.html?CG4893), [ds](http://flybase.bio.indiana.edu/.bin/fbidq.html?ds), [CASK](http://flybase.bio.indiana.edu/.bin/fbidq.html?CASK), [CG9098](http://flybase.bio.indiana.edu/.bin/fbidq.html?CG9098), [Alk](http://flybase.bio.indiana.edu/.bin/fbidq.html?Alk), [sals](http://flybase.bio.indiana.edu/.bin/fbidq.html?sals), [Sin3A](http://flybase.bio.indiana.edu/.bin/fbidq.html?Sin3A), [mip120](http://flybase.bio.indiana.edu/.bin/fbidq.html?mip120), [jumu](http://flybase.bio.indiana.edu/.bin/fbidq.html?jumu), [Smox](http://flybase.bio.indiana.edu/.bin/fbidq.html?Smox), [CG31475](http://flybase.bio.indiana.edu/.bin/fbidq.html?CG31475), [CG32944](http://flybase.bio.indiana.edu/.bin/fbidq.html?CG32944), [beat-Ic](http://flybase.bio.indiana.edu/.bin/fbidq.html?beat-Ic), [fd59A](http://flybase.bio.indiana.edu/.bin/fbidq.html?fd59A), [Fak56D](http://flybase.bio.indiana.edu/.bin/fbidq.html?Fak56D), [run](http://flybase.bio.indiana.edu/.bin/fbidq.html?run), [brat](http://flybase.bio.indiana.edu/.bin/fbidq.html?brat), [ko](http://flybase.bio.indiana.edu/.bin/fbidq.html?ko), [Kap3](http://flybase.bio.indiana.edu/.bin/fbidq.html?Kap3), [Eip71CD](http://flybase.bio.indiana.edu/.bin/fbidq.html?Eip71CD), [stan](http://flybase.bio.indiana.edu/.bin/fbidq.html?stan),[CG12424](http://flybase.bio.indiana.edu/.bin/fbidq.html?CG12424), [tutl](http://flybase.bio.indiana.edu/.bin/fbidq.html?tutl), [futsch](http://flybase.bio.indiana.edu/.bin/fbidq.html?futsch), [Ptx1](http://flybase.bio.indiana.edu/.bin/fbidq.html?Ptx1), [ph-p](http://flybase.bio.indiana.edu/.bin/fbidq.html?ph-p), [gukh](http://flybase.bio.indiana.edu/.bin/fbidq.html?gukh), [chm](http://flybase.bio.indiana.edu/.bin/fbidq.html?chm), [sna](http://flybase.bio.indiana.edu/.bin/fbidq.html?sna), [Wnt5](http://flybase.bio.indiana.edu/.bin/fbidq.html?Wnt5), [beat-IIa](http://flybase.bio.indiana.edu/.bin/fbidq.html?beat-IIa), [Ggamma1](http://flybase.bio.indiana.edu/.bin/fbidq.html?Ggamma1), [sm](http://flybase.bio.indiana.edu/.bin/fbidq.html?sm), [dac](http://flybase.bio.indiana.edu/.bin/fbidq.html?dac), [Cdk5](http://flybase.bio.indiana.edu/.bin/fbidq.html?Cdk5), [sif](http://flybase.bio.indiana.edu/.bin/fbidq.html?sif), [Cep135](http://flybase.bio.indiana.edu/.bin/fbidq.html?Cep135), [dom](http://flybase.bio.indiana.edu/.bin/fbidq.html?dom), [Sas-4](http://flybase.bio.indiana.edu/.bin/fbidq.html?Sas-4), [ssh](http://flybase.bio.indiana.edu/.bin/fbidq.html?ssh), [baz](http://flybase.bio.indiana.edu/.bin/fbidq.html?baz), [mle](http://flybase.bio.indiana.edu/.bin/fbidq.html?mle), [Hr51](http://flybase.bio.indiana.edu/.bin/fbidq.html?Hr51), [CG11155](http://flybase.bio.indiana.edu/.bin/fbidq.html?CG11155), [rst](http://flybase.bio.indiana.edu/.bin/fbidq.html?rst), [grh](http://flybase.bio.indiana.edu/.bin/fbidq.html?grh), [comm](http://flybase.bio.indiana.edu/.bin/fbidq.html?comm), [tyn](http://flybase.bio.indiana.edu/.bin/fbidq.html?tyn), [Cdk5alpha](http://flybase.bio.indiana.edu/.bin/fbidq.html?Cdk5alpha), [wg](http://flybase.bio.indiana.edu/.bin/fbidq.html?wg), [Lim3](http://flybase.bio.indiana.edu/.bin/fbidq.html?Lim3), [CadN](http://flybase.bio.indiana.edu/.bin/fbidq.html?CadN), [Aplip1](http://flybase.bio.indiana.edu/.bin/fbidq.html?Aplip1), [E(Pc)](http://flybase.bio.indiana.edu/.bin/fbidq.html?E(Pc)), [retn](http://flybase.bio.indiana.edu/.bin/fbidq.html?retn), [E(bx)](http://flybase.bio.indiana.edu/.bin/fbidq.html?E(bx)), [en](http://flybase.bio.indiana.edu/.bin/fbidq.html?en), [Snap](http://flybase.bio.indiana.edu/.bin/fbidq.html?Snap), [trn](http://flybase.bio.indiana.edu/.bin/fbidq.html?trn),[Pitslre](http://flybase.bio.indiana.edu/.bin/fbidq.html?Pitslre), [CG33960](http://flybase.bio.indiana.edu/.bin/fbidq.html?CG33960), [bt](http://flybase.bio.indiana.edu/.bin/fbidq.html?bt), [Ct](http://flybase.bio.indiana.edu/.bin/fbidq.html?Ct) |
| [axon guidance](http://amigo.geneontology.org/cgi-bin/amigo/go.cgi?view=details&query=GO:0007411) | 80 of 1594 genes, 5.0% | 195 of 7634 genes, 2.6% | 1.21e-08 | 0.00% | 0.00 | [fz2](http://flybase.bio.indiana.edu/.bin/fbidq.html?fz2), [acj6](http://flybase.bio.indiana.edu/.bin/fbidq.html?acj6), [vvl](http://flybase.bio.indiana.edu/.bin/fbidq.html?vvl), [jing](http://flybase.bio.indiana.edu/.bin/fbidq.html?jing), [dock](http://flybase.bio.indiana.edu/.bin/fbidq.html?dock), [Ptp69D](http://flybase.bio.indiana.edu/.bin/fbidq.html?Ptp69D), [hts](http://flybase.bio.indiana.edu/.bin/fbidq.html?hts), [mp](http://flybase.bio.indiana.edu/.bin/fbidq.html?mp), [ap](http://flybase.bio.indiana.edu/.bin/fbidq.html?ap), [Dg](http://flybase.bio.indiana.edu/.bin/fbidq.html?Dg), [seq](http://flybase.bio.indiana.edu/.bin/fbidq.html?seq), [gcm](http://flybase.bio.indiana.edu/.bin/fbidq.html?gcm), [Trim9](http://flybase.bio.indiana.edu/.bin/fbidq.html?Trim9), [robo3](http://flybase.bio.indiana.edu/.bin/fbidq.html?robo3), [tok](http://flybase.bio.indiana.edu/.bin/fbidq.html?tok), [Ptp99A](http://flybase.bio.indiana.edu/.bin/fbidq.html?Ptp99A), [lola](http://flybase.bio.indiana.edu/.bin/fbidq.html?lola), [unc-104](http://flybase.bio.indiana.edu/.bin/fbidq.html?unc-104), [chif](http://flybase.bio.indiana.edu/.bin/fbidq.html?chif), [NetB](http://flybase.bio.indiana.edu/.bin/fbidq.html?NetB), [Alk](http://flybase.bio.indiana.edu/.bin/fbidq.html?Alk), [Nrk](http://flybase.bio.indiana.edu/.bin/fbidq.html?Nrk), [Galpha49B](http://flybase.bio.indiana.edu/.bin/fbidq.html?Galpha49B), [Smox](http://flybase.bio.indiana.edu/.bin/fbidq.html?Smox), [Lar](http://flybase.bio.indiana.edu/.bin/fbidq.html?Lar), [not](http://flybase.bio.indiana.edu/.bin/fbidq.html?not), [nvy](http://flybase.bio.indiana.edu/.bin/fbidq.html?nvy), [gogo](http://flybase.bio.indiana.edu/.bin/fbidq.html?gogo), [fd59A](http://flybase.bio.indiana.edu/.bin/fbidq.html?fd59A), [beat-Ic](http://flybase.bio.indiana.edu/.bin/fbidq.html?beat-Ic), [run](http://flybase.bio.indiana.edu/.bin/fbidq.html?run), [jeb](http://flybase.bio.indiana.edu/.bin/fbidq.html?jeb), [brat](http://flybase.bio.indiana.edu/.bin/fbidq.html?brat), [ko](http://flybase.bio.indiana.edu/.bin/fbidq.html?ko), [Sema-1a](http://flybase.bio.indiana.edu/.bin/fbidq.html?Sema-1a), [beat-Ib](http://flybase.bio.indiana.edu/.bin/fbidq.html?beat-Ib), [otk](http://flybase.bio.indiana.edu/.bin/fbidq.html?otk), [stan](http://flybase.bio.indiana.edu/.bin/fbidq.html?stan), [CG12424](http://flybase.bio.indiana.edu/.bin/fbidq.html?CG12424),[CG34400](http://flybase.bio.indiana.edu/.bin/fbidq.html?CG34400), [tutl](http://flybase.bio.indiana.edu/.bin/fbidq.html?tutl), [ena](http://flybase.bio.indiana.edu/.bin/fbidq.html?ena), [dsh](http://flybase.bio.indiana.edu/.bin/fbidq.html?dsh), [Nf-YC](http://flybase.bio.indiana.edu/.bin/fbidq.html?Nf-YC), [robo](http://flybase.bio.indiana.edu/.bin/fbidq.html?robo), [bsk](http://flybase.bio.indiana.edu/.bin/fbidq.html?bsk), [lea](http://flybase.bio.indiana.edu/.bin/fbidq.html?lea), [beat-Ia](http://flybase.bio.indiana.edu/.bin/fbidq.html?beat-Ia), [gukh](http://flybase.bio.indiana.edu/.bin/fbidq.html?gukh), [plexB](http://flybase.bio.indiana.edu/.bin/fbidq.html?plexB), [Wnt5](http://flybase.bio.indiana.edu/.bin/fbidq.html?Wnt5), [beat-IIa](http://flybase.bio.indiana.edu/.bin/fbidq.html?beat-IIa), [Klp64D](http://flybase.bio.indiana.edu/.bin/fbidq.html?Klp64D), [grn](http://flybase.bio.indiana.edu/.bin/fbidq.html?grn), [sm](http://flybase.bio.indiana.edu/.bin/fbidq.html?sm), [mew](http://flybase.bio.indiana.edu/.bin/fbidq.html?mew), [Lis-1](http://flybase.bio.indiana.edu/.bin/fbidq.html?Lis-1), [dac](http://flybase.bio.indiana.edu/.bin/fbidq.html?dac), [Cdk5](http://flybase.bio.indiana.edu/.bin/fbidq.html?Cdk5), [pros](http://flybase.bio.indiana.edu/.bin/fbidq.html?pros), [tup](http://flybase.bio.indiana.edu/.bin/fbidq.html?tup), [trio](http://flybase.bio.indiana.edu/.bin/fbidq.html?trio), [daw](http://flybase.bio.indiana.edu/.bin/fbidq.html?daw), [Wnt4](http://flybase.bio.indiana.edu/.bin/fbidq.html?Wnt4), [comm](http://flybase.bio.indiana.edu/.bin/fbidq.html?comm), [Cdk5alpha](http://flybase.bio.indiana.edu/.bin/fbidq.html?Cdk5alpha), [Apc](http://flybase.bio.indiana.edu/.bin/fbidq.html?Apc), [Lim3](http://flybase.bio.indiana.edu/.bin/fbidq.html?Lim3), [sim](http://flybase.bio.indiana.edu/.bin/fbidq.html?sim), [CadN](http://flybase.bio.indiana.edu/.bin/fbidq.html?CadN), [nerfin-1](http://flybase.bio.indiana.edu/.bin/fbidq.html?nerfin-1), [trx](http://flybase.bio.indiana.edu/.bin/fbidq.html?trx), [retn](http://flybase.bio.indiana.edu/.bin/fbidq.html?retn), [en](http://flybase.bio.indiana.edu/.bin/fbidq.html?en), [dsx](http://flybase.bio.indiana.edu/.bin/fbidq.html?dsx), [unc-5](http://flybase.bio.indiana.edu/.bin/fbidq.html?unc-5), [trn](http://flybase.bio.indiana.edu/.bin/fbidq.html?trn),[CG10107](http://flybase.bio.indiana.edu/.bin/fbidq.html?CG10107), [fend](http://flybase.bio.indiana.edu/.bin/fbidq.html?fend), [CG33960](http://flybase.bio.indiana.edu/.bin/fbidq.html?CG33960) |
| [regulation of cellular metabolic process](http://amigo.geneontology.org/cgi-bin/amigo/go.cgi?view=details&query=GO:0031323) | 266 of 1594 genes, 16.7% | 933 of 7634 genes, 12.2% | 1.42e-08 | 0.00% | 0.00 | [fz2](http://flybase.bio.indiana.edu/.bin/fbidq.html?fz2), [Ets65A](http://flybase.bio.indiana.edu/.bin/fbidq.html?Ets65A), [tna](http://flybase.bio.indiana.edu/.bin/fbidq.html?tna), [Sxl](http://flybase.bio.indiana.edu/.bin/fbidq.html?Sxl), [mub](http://flybase.bio.indiana.edu/.bin/fbidq.html?mub), [JIL-1](http://flybase.bio.indiana.edu/.bin/fbidq.html?JIL-1), [rn](http://flybase.bio.indiana.edu/.bin/fbidq.html?rn), [ap](http://flybase.bio.indiana.edu/.bin/fbidq.html?ap), [cenG1A](http://flybase.bio.indiana.edu/.bin/fbidq.html?cenG1A), [Nf1](http://flybase.bio.indiana.edu/.bin/fbidq.html?Nf1), [bi](http://flybase.bio.indiana.edu/.bin/fbidq.html?bi), [Hcf](http://flybase.bio.indiana.edu/.bin/fbidq.html?Hcf), [Traf6](http://flybase.bio.indiana.edu/.bin/fbidq.html?Traf6), [NC2alpha](http://flybase.bio.indiana.edu/.bin/fbidq.html?NC2alpha), [bab1](http://flybase.bio.indiana.edu/.bin/fbidq.html?bab1), [Snoo](http://flybase.bio.indiana.edu/.bin/fbidq.html?Snoo), [scrib](http://flybase.bio.indiana.edu/.bin/fbidq.html?scrib), [HGTX](http://flybase.bio.indiana.edu/.bin/fbidq.html?HGTX), [not](http://flybase.bio.indiana.edu/.bin/fbidq.html?not), [nvy](http://flybase.bio.indiana.edu/.bin/fbidq.html?nvy), [nub](http://flybase.bio.indiana.edu/.bin/fbidq.html?nub), [nab](http://flybase.bio.indiana.edu/.bin/fbidq.html?nab), [btd](http://flybase.bio.indiana.edu/.bin/fbidq.html?btd), [fd68A](http://flybase.bio.indiana.edu/.bin/fbidq.html?fd68A), [U2af38](http://flybase.bio.indiana.edu/.bin/fbidq.html?U2af38), [HLHmdelta](http://flybase.bio.indiana.edu/.bin/fbidq.html?HLHmdelta), [B-H1](http://flybase.bio.indiana.edu/.bin/fbidq.html?B-H1), [Nipped-A](http://flybase.bio.indiana.edu/.bin/fbidq.html?Nipped-A), [ATbp](http://flybase.bio.indiana.edu/.bin/fbidq.html?ATbp), [7B2](http://flybase.bio.indiana.edu/.bin/fbidq.html?7B2), [alph](http://flybase.bio.indiana.edu/.bin/fbidq.html?alph), [Spf45](http://flybase.bio.indiana.edu/.bin/fbidq.html?Spf45), [Fer2](http://flybase.bio.indiana.edu/.bin/fbidq.html?Fer2), [fd102C](http://flybase.bio.indiana.edu/.bin/fbidq.html?fd102C), [hth](http://flybase.bio.indiana.edu/.bin/fbidq.html?hth), [Nf-YC](http://flybase.bio.indiana.edu/.bin/fbidq.html?Nf-YC), [Pli](http://flybase.bio.indiana.edu/.bin/fbidq.html?Pli), [Hel25E](http://flybase.bio.indiana.edu/.bin/fbidq.html?Hel25E), [Hey](http://flybase.bio.indiana.edu/.bin/fbidq.html?Hey),[loqs](http://flybase.bio.indiana.edu/.bin/fbidq.html?loqs), [CycE](http://flybase.bio.indiana.edu/.bin/fbidq.html?CycE), [Brf](http://flybase.bio.indiana.edu/.bin/fbidq.html?Brf), [Psi](http://flybase.bio.indiana.edu/.bin/fbidq.html?Psi), [Gug](http://flybase.bio.indiana.edu/.bin/fbidq.html?Gug), [pad](http://flybase.bio.indiana.edu/.bin/fbidq.html?pad), [orb2](http://flybase.bio.indiana.edu/.bin/fbidq.html?orb2), [Rx](http://flybase.bio.indiana.edu/.bin/fbidq.html?Rx), [MED1](http://flybase.bio.indiana.edu/.bin/fbidq.html?MED1), [salr](http://flybase.bio.indiana.edu/.bin/fbidq.html?salr), [wdn](http://flybase.bio.indiana.edu/.bin/fbidq.html?wdn), [sim](http://flybase.bio.indiana.edu/.bin/fbidq.html?sim), [d4](http://flybase.bio.indiana.edu/.bin/fbidq.html?d4), [cort](http://flybase.bio.indiana.edu/.bin/fbidq.html?cort), [dan](http://flybase.bio.indiana.edu/.bin/fbidq.html?dan), [gro](http://flybase.bio.indiana.edu/.bin/fbidq.html?gro), [Jarid2](http://flybase.bio.indiana.edu/.bin/fbidq.html?Jarid2), [RN-tre](http://flybase.bio.indiana.edu/.bin/fbidq.html?RN-tre), [hkl](http://flybase.bio.indiana.edu/.bin/fbidq.html?hkl), [bin3](http://flybase.bio.indiana.edu/.bin/fbidq.html?bin3), [trx](http://flybase.bio.indiana.edu/.bin/fbidq.html?trx), [Rpd3](http://flybase.bio.indiana.edu/.bin/fbidq.html?Rpd3), [MED25](http://flybase.bio.indiana.edu/.bin/fbidq.html?MED25), [tyf](http://flybase.bio.indiana.edu/.bin/fbidq.html?tyf), [vfl](http://flybase.bio.indiana.edu/.bin/fbidq.html?vfl), [aret](http://flybase.bio.indiana.edu/.bin/fbidq.html?aret), [pygo](http://flybase.bio.indiana.edu/.bin/fbidq.html?pygo), [HLHm7](http://flybase.bio.indiana.edu/.bin/fbidq.html?HLHm7), [z](http://flybase.bio.indiana.edu/.bin/fbidq.html?z), [HLHmgamma](http://flybase.bio.indiana.edu/.bin/fbidq.html?HLHmgamma), [Sox21b](http://flybase.bio.indiana.edu/.bin/fbidq.html?Sox21b), [wor](http://flybase.bio.indiana.edu/.bin/fbidq.html?wor), [Atg1](http://flybase.bio.indiana.edu/.bin/fbidq.html?Atg1), [Pten](http://flybase.bio.indiana.edu/.bin/fbidq.html?Pten), [wda](http://flybase.bio.indiana.edu/.bin/fbidq.html?wda), [MED14](http://flybase.bio.indiana.edu/.bin/fbidq.html?MED14), [pUf68](http://flybase.bio.indiana.edu/.bin/fbidq.html?pUf68), [seq](http://flybase.bio.indiana.edu/.bin/fbidq.html?seq), [Dip3](http://flybase.bio.indiana.edu/.bin/fbidq.html?Dip3), [HDAC4](http://flybase.bio.indiana.edu/.bin/fbidq.html?HDAC4), [elk](http://flybase.bio.indiana.edu/.bin/fbidq.html?elk),[phl](http://flybase.bio.indiana.edu/.bin/fbidq.html?phl), [Cbl](http://flybase.bio.indiana.edu/.bin/fbidq.html?Cbl), [sNPF](http://flybase.bio.indiana.edu/.bin/fbidq.html?sNPF), [pog](http://flybase.bio.indiana.edu/.bin/fbidq.html?pog), [Pdp1](http://flybase.bio.indiana.edu/.bin/fbidq.html?Pdp1), [plx](http://flybase.bio.indiana.edu/.bin/fbidq.html?plx), [Alk](http://flybase.bio.indiana.edu/.bin/fbidq.html?Alk), [Sin3A](http://flybase.bio.indiana.edu/.bin/fbidq.html?Sin3A), [mip120](http://flybase.bio.indiana.edu/.bin/fbidq.html?mip120), [casp](http://flybase.bio.indiana.edu/.bin/fbidq.html?casp), [jumu](http://flybase.bio.indiana.edu/.bin/fbidq.html?jumu), [Smox](http://flybase.bio.indiana.edu/.bin/fbidq.html?Smox), [Usp7](http://flybase.bio.indiana.edu/.bin/fbidq.html?Usp7), [fd59A](http://flybase.bio.indiana.edu/.bin/fbidq.html?fd59A), [brat](http://flybase.bio.indiana.edu/.bin/fbidq.html?brat), [FBX011](http://flybase.bio.indiana.edu/.bin/fbidq.html?FBX011), [Antp](http://flybase.bio.indiana.edu/.bin/fbidq.html?Antp), [l(3)neo38](http://flybase.bio.indiana.edu/.bin/fbidq.html?l(3)neo38), [Ast-C](http://flybase.bio.indiana.edu/.bin/fbidq.html?Ast-C), [Ubx](http://flybase.bio.indiana.edu/.bin/fbidq.html?Ubx), [Ptx1](http://flybase.bio.indiana.edu/.bin/fbidq.html?Ptx1), [chm](http://flybase.bio.indiana.edu/.bin/fbidq.html?chm), [hbn](http://flybase.bio.indiana.edu/.bin/fbidq.html?hbn), [tsh](http://flybase.bio.indiana.edu/.bin/fbidq.html?tsh), [Rgl](http://flybase.bio.indiana.edu/.bin/fbidq.html?Rgl), [e(y)3](http://flybase.bio.indiana.edu/.bin/fbidq.html?e(y)3), [CG9007](http://flybase.bio.indiana.edu/.bin/fbidq.html?CG9007), [oc](http://flybase.bio.indiana.edu/.bin/fbidq.html?oc), [spt4](http://flybase.bio.indiana.edu/.bin/fbidq.html?spt4), [gol](http://flybase.bio.indiana.edu/.bin/fbidq.html?gol), [ey](http://flybase.bio.indiana.edu/.bin/fbidq.html?ey), [Bgb](http://flybase.bio.indiana.edu/.bin/fbidq.html?Bgb), [l(1)sc](http://flybase.bio.indiana.edu/.bin/fbidq.html?l(1)sc), [comm](http://flybase.bio.indiana.edu/.bin/fbidq.html?comm), [grh](http://flybase.bio.indiana.edu/.bin/fbidq.html?grh), [Vsx1](http://flybase.bio.indiana.edu/.bin/fbidq.html?Vsx1), [toy](http://flybase.bio.indiana.edu/.bin/fbidq.html?toy), [Lim3](http://flybase.bio.indiana.edu/.bin/fbidq.html?Lim3), [CG34362](http://flybase.bio.indiana.edu/.bin/fbidq.html?CG34362), [crol](http://flybase.bio.indiana.edu/.bin/fbidq.html?crol),[sd](http://flybase.bio.indiana.edu/.bin/fbidq.html?sd), [retn](http://flybase.bio.indiana.edu/.bin/fbidq.html?retn), [fs(1)h](http://flybase.bio.indiana.edu/.bin/fbidq.html?fs(1)h), [rl](http://flybase.bio.indiana.edu/.bin/fbidq.html?rl), [CG11347](http://flybase.bio.indiana.edu/.bin/fbidq.html?CG11347), [tey](http://flybase.bio.indiana.edu/.bin/fbidq.html?tey), [Saf-B](http://flybase.bio.indiana.edu/.bin/fbidq.html?Saf-B), [CG11294](http://flybase.bio.indiana.edu/.bin/fbidq.html?CG11294), [hiw](http://flybase.bio.indiana.edu/.bin/fbidq.html?hiw), [Atf-2](http://flybase.bio.indiana.edu/.bin/fbidq.html?Atf-2), [dnr1](http://flybase.bio.indiana.edu/.bin/fbidq.html?dnr1), [acj6](http://flybase.bio.indiana.edu/.bin/fbidq.html?acj6), [skd](http://flybase.bio.indiana.edu/.bin/fbidq.html?skd), [jing](http://flybase.bio.indiana.edu/.bin/fbidq.html?jing), [caup](http://flybase.bio.indiana.edu/.bin/fbidq.html?caup), [Camta](http://flybase.bio.indiana.edu/.bin/fbidq.html?Camta), [tll](http://flybase.bio.indiana.edu/.bin/fbidq.html?tll), [ato](http://flybase.bio.indiana.edu/.bin/fbidq.html?ato), [Rox8](http://flybase.bio.indiana.edu/.bin/fbidq.html?Rox8), [Iswi](http://flybase.bio.indiana.edu/.bin/fbidq.html?Iswi), [Spt6](http://flybase.bio.indiana.edu/.bin/fbidq.html?Spt6), [Dg](http://flybase.bio.indiana.edu/.bin/fbidq.html?Dg), [Pka-R1](http://flybase.bio.indiana.edu/.bin/fbidq.html?Pka-R1), [Evi5](http://flybase.bio.indiana.edu/.bin/fbidq.html?Evi5), [br](http://flybase.bio.indiana.edu/.bin/fbidq.html?br), [ksr](http://flybase.bio.indiana.edu/.bin/fbidq.html?ksr), [PNUTS](http://flybase.bio.indiana.edu/.bin/fbidq.html?PNUTS), [chn](http://flybase.bio.indiana.edu/.bin/fbidq.html?chn), [Gsc](http://flybase.bio.indiana.edu/.bin/fbidq.html?Gsc), [CG17124](http://flybase.bio.indiana.edu/.bin/fbidq.html?CG17124), [lola](http://flybase.bio.indiana.edu/.bin/fbidq.html?lola), [chif](http://flybase.bio.indiana.edu/.bin/fbidq.html?chif), [Pcl](http://flybase.bio.indiana.edu/.bin/fbidq.html?Pcl), [HLH4C](http://flybase.bio.indiana.edu/.bin/fbidq.html?HLH4C), [simj](http://flybase.bio.indiana.edu/.bin/fbidq.html?simj), [tap](http://flybase.bio.indiana.edu/.bin/fbidq.html?tap), [Awh](http://flybase.bio.indiana.edu/.bin/fbidq.html?Awh), [PHDP](http://flybase.bio.indiana.edu/.bin/fbidq.html?PHDP), [CG4328](http://flybase.bio.indiana.edu/.bin/fbidq.html?CG4328),[Su(var)3-3](http://flybase.bio.indiana.edu/.bin/fbidq.html?Su(var)3-3), [Taf4](http://flybase.bio.indiana.edu/.bin/fbidq.html?Taf4), [l(2)NC136](http://flybase.bio.indiana.edu/.bin/fbidq.html?l(2)NC136), [stau](http://flybase.bio.indiana.edu/.bin/fbidq.html?stau), [Sos](http://flybase.bio.indiana.edu/.bin/fbidq.html?Sos), [vg](http://flybase.bio.indiana.edu/.bin/fbidq.html?vg), [eya](http://flybase.bio.indiana.edu/.bin/fbidq.html?eya), [bsk](http://flybase.bio.indiana.edu/.bin/fbidq.html?bsk), [brm](http://flybase.bio.indiana.edu/.bin/fbidq.html?brm), [fz](http://flybase.bio.indiana.edu/.bin/fbidq.html?fz), [Vsx2](http://flybase.bio.indiana.edu/.bin/fbidq.html?Vsx2), [sno](http://flybase.bio.indiana.edu/.bin/fbidq.html?sno), [ara](http://flybase.bio.indiana.edu/.bin/fbidq.html?ara), [Sfmbt](http://flybase.bio.indiana.edu/.bin/fbidq.html?Sfmbt), [mam](http://flybase.bio.indiana.edu/.bin/fbidq.html?mam), [pan](http://flybase.bio.indiana.edu/.bin/fbidq.html?pan), [disco](http://flybase.bio.indiana.edu/.bin/fbidq.html?disco), [Dll](http://flybase.bio.indiana.edu/.bin/fbidq.html?Dll), [grn](http://flybase.bio.indiana.edu/.bin/fbidq.html?grn), [D12](http://flybase.bio.indiana.edu/.bin/fbidq.html?D12), [HLHm5](http://flybase.bio.indiana.edu/.bin/fbidq.html?HLHm5), [Lis-1](http://flybase.bio.indiana.edu/.bin/fbidq.html?Lis-1), [ems](http://flybase.bio.indiana.edu/.bin/fbidq.html?ems), [dap](http://flybase.bio.indiana.edu/.bin/fbidq.html?dap), [CG16896](http://flybase.bio.indiana.edu/.bin/fbidq.html?CG16896), [tup](http://flybase.bio.indiana.edu/.bin/fbidq.html?tup), [pros](http://flybase.bio.indiana.edu/.bin/fbidq.html?pros), [pdm2](http://flybase.bio.indiana.edu/.bin/fbidq.html?pdm2), [rgr](http://flybase.bio.indiana.edu/.bin/fbidq.html?rgr), [spel1](http://flybase.bio.indiana.edu/.bin/fbidq.html?spel1), [Wnt4](http://flybase.bio.indiana.edu/.bin/fbidq.html?Wnt4), [CG30372](http://flybase.bio.indiana.edu/.bin/fbidq.html?CG30372), [nerfin-1](http://flybase.bio.indiana.edu/.bin/fbidq.html?nerfin-1), [CG42629](http://flybase.bio.indiana.edu/.bin/fbidq.html?CG42629), [bchs](http://flybase.bio.indiana.edu/.bin/fbidq.html?bchs), [pr-set7](http://flybase.bio.indiana.edu/.bin/fbidq.html?pr-set7), [dsx](http://flybase.bio.indiana.edu/.bin/fbidq.html?dsx),[Ssdp](http://flybase.bio.indiana.edu/.bin/fbidq.html?Ssdp), [sqz](http://flybase.bio.indiana.edu/.bin/fbidq.html?sqz), [CG8155](http://flybase.bio.indiana.edu/.bin/fbidq.html?CG8155), [Fer3](http://flybase.bio.indiana.edu/.bin/fbidq.html?Fer3), [CG32105](http://flybase.bio.indiana.edu/.bin/fbidq.html?CG32105), [dalao](http://flybase.bio.indiana.edu/.bin/fbidq.html?dalao), [Lim1](http://flybase.bio.indiana.edu/.bin/fbidq.html?Lim1), [sr](http://flybase.bio.indiana.edu/.bin/fbidq.html?sr), [tld](http://flybase.bio.indiana.edu/.bin/fbidq.html?tld), [danr](http://flybase.bio.indiana.edu/.bin/fbidq.html?danr), [vvl](http://flybase.bio.indiana.edu/.bin/fbidq.html?vvl), [CG7879](http://flybase.bio.indiana.edu/.bin/fbidq.html?CG7879), [sgg](http://flybase.bio.indiana.edu/.bin/fbidq.html?sgg), [gcm2](http://flybase.bio.indiana.edu/.bin/fbidq.html?gcm2), [Eip93F](http://flybase.bio.indiana.edu/.bin/fbidq.html?Eip93F), [gcm](http://flybase.bio.indiana.edu/.bin/fbidq.html?gcm), [can](http://flybase.bio.indiana.edu/.bin/fbidq.html?can), [Aef1](http://flybase.bio.indiana.edu/.bin/fbidq.html?Aef1), [Hsp67Bc](http://flybase.bio.indiana.edu/.bin/fbidq.html?Hsp67Bc), [rab3-GEF](http://flybase.bio.indiana.edu/.bin/fbidq.html?rab3-GEF), [ase](http://flybase.bio.indiana.edu/.bin/fbidq.html?ase), [ovo](http://flybase.bio.indiana.edu/.bin/fbidq.html?ovo), [CG13253](http://flybase.bio.indiana.edu/.bin/fbidq.html?CG13253), [CG6197](http://flybase.bio.indiana.edu/.bin/fbidq.html?CG6197), [scro](http://flybase.bio.indiana.edu/.bin/fbidq.html?scro), [Dsp1](http://flybase.bio.indiana.edu/.bin/fbidq.html?Dsp1), [elav](http://flybase.bio.indiana.edu/.bin/fbidq.html?elav), [CG14216](http://flybase.bio.indiana.edu/.bin/fbidq.html?CG14216), [Oli](http://flybase.bio.indiana.edu/.bin/fbidq.html?Oli), [Nelf-A](http://flybase.bio.indiana.edu/.bin/fbidq.html?Nelf-A), [GATAd](http://flybase.bio.indiana.edu/.bin/fbidq.html?GATAd), [CG6227](http://flybase.bio.indiana.edu/.bin/fbidq.html?CG6227), [Poxn](http://flybase.bio.indiana.edu/.bin/fbidq.html?Poxn), [fbl6](http://flybase.bio.indiana.edu/.bin/fbidq.html?fbl6),[CG15376](http://flybase.bio.indiana.edu/.bin/fbidq.html?CG15376), [ush](http://flybase.bio.indiana.edu/.bin/fbidq.html?ush), [CG10336](http://flybase.bio.indiana.edu/.bin/fbidq.html?CG10336), [Gap1](http://flybase.bio.indiana.edu/.bin/fbidq.html?Gap1), [run](http://flybase.bio.indiana.edu/.bin/fbidq.html?run), [tara](http://flybase.bio.indiana.edu/.bin/fbidq.html?tara), [Alh](http://flybase.bio.indiana.edu/.bin/fbidq.html?Alh), [Hel89B](http://flybase.bio.indiana.edu/.bin/fbidq.html?Hel89B), [egg](http://flybase.bio.indiana.edu/.bin/fbidq.html?egg), [Sox102F](http://flybase.bio.indiana.edu/.bin/fbidq.html?Sox102F), [CG5337](http://flybase.bio.indiana.edu/.bin/fbidq.html?CG5337), [koko](http://flybase.bio.indiana.edu/.bin/fbidq.html?koko), [Ptth](http://flybase.bio.indiana.edu/.bin/fbidq.html?Ptth), [Sp1](http://flybase.bio.indiana.edu/.bin/fbidq.html?Sp1), [ph-p](http://flybase.bio.indiana.edu/.bin/fbidq.html?ph-p), [onecut](http://flybase.bio.indiana.edu/.bin/fbidq.html?onecut), [CG7757](http://flybase.bio.indiana.edu/.bin/fbidq.html?CG7757), [sna](http://flybase.bio.indiana.edu/.bin/fbidq.html?sna), [Wnt5](http://flybase.bio.indiana.edu/.bin/fbidq.html?Wnt5), [Taf1](http://flybase.bio.indiana.edu/.bin/fbidq.html?Taf1), [MBD-like](http://flybase.bio.indiana.edu/.bin/fbidq.html?MBD-like), [esg](http://flybase.bio.indiana.edu/.bin/fbidq.html?esg), [sif](http://flybase.bio.indiana.edu/.bin/fbidq.html?sif), [dom](http://flybase.bio.indiana.edu/.bin/fbidq.html?dom), [CG32532](http://flybase.bio.indiana.edu/.bin/fbidq.html?CG32532), [MTA1-like](http://flybase.bio.indiana.edu/.bin/fbidq.html?MTA1-like), [mle](http://flybase.bio.indiana.edu/.bin/fbidq.html?mle), [Hr51](http://flybase.bio.indiana.edu/.bin/fbidq.html?Hr51), [B-H2](http://flybase.bio.indiana.edu/.bin/fbidq.html?B-H2), [wg](http://flybase.bio.indiana.edu/.bin/fbidq.html?wg), [fkh](http://flybase.bio.indiana.edu/.bin/fbidq.html?fkh), [fne](http://flybase.bio.indiana.edu/.bin/fbidq.html?fne), [en](http://flybase.bio.indiana.edu/.bin/fbidq.html?en), [Utx](http://flybase.bio.indiana.edu/.bin/fbidq.html?Utx), [cos](http://flybase.bio.indiana.edu/.bin/fbidq.html?cos), [Ct](http://flybase.bio.indiana.edu/.bin/fbidq.html?Ct) |
| [regulation of primary metabolic process](http://amigo.geneontology.org/cgi-bin/amigo/go.cgi?view=details&query=GO:0080090) | 257 of 1594 genes, 16.1% | 896 of 7634 genes, 11.7% | 1.74e-08 | 0.00% | 0.00 | [fz2](http://flybase.bio.indiana.edu/.bin/fbidq.html?fz2), [Ets65A](http://flybase.bio.indiana.edu/.bin/fbidq.html?Ets65A), [tna](http://flybase.bio.indiana.edu/.bin/fbidq.html?tna), [Sxl](http://flybase.bio.indiana.edu/.bin/fbidq.html?Sxl), [mub](http://flybase.bio.indiana.edu/.bin/fbidq.html?mub), [JIL-1](http://flybase.bio.indiana.edu/.bin/fbidq.html?JIL-1), [rn](http://flybase.bio.indiana.edu/.bin/fbidq.html?rn), [ap](http://flybase.bio.indiana.edu/.bin/fbidq.html?ap), [cenG1A](http://flybase.bio.indiana.edu/.bin/fbidq.html?cenG1A), [Nf1](http://flybase.bio.indiana.edu/.bin/fbidq.html?Nf1), [bi](http://flybase.bio.indiana.edu/.bin/fbidq.html?bi), [Hcf](http://flybase.bio.indiana.edu/.bin/fbidq.html?Hcf), [Traf6](http://flybase.bio.indiana.edu/.bin/fbidq.html?Traf6), [NC2alpha](http://flybase.bio.indiana.edu/.bin/fbidq.html?NC2alpha), [bab1](http://flybase.bio.indiana.edu/.bin/fbidq.html?bab1), [Snoo](http://flybase.bio.indiana.edu/.bin/fbidq.html?Snoo), [HGTX](http://flybase.bio.indiana.edu/.bin/fbidq.html?HGTX), [not](http://flybase.bio.indiana.edu/.bin/fbidq.html?not), [nvy](http://flybase.bio.indiana.edu/.bin/fbidq.html?nvy), [nub](http://flybase.bio.indiana.edu/.bin/fbidq.html?nub), [nab](http://flybase.bio.indiana.edu/.bin/fbidq.html?nab), [btd](http://flybase.bio.indiana.edu/.bin/fbidq.html?btd), [fd68A](http://flybase.bio.indiana.edu/.bin/fbidq.html?fd68A), [U2af38](http://flybase.bio.indiana.edu/.bin/fbidq.html?U2af38), [HLHmdelta](http://flybase.bio.indiana.edu/.bin/fbidq.html?HLHmdelta), [B-H1](http://flybase.bio.indiana.edu/.bin/fbidq.html?B-H1), [Nipped-A](http://flybase.bio.indiana.edu/.bin/fbidq.html?Nipped-A), [ATbp](http://flybase.bio.indiana.edu/.bin/fbidq.html?ATbp), [7B2](http://flybase.bio.indiana.edu/.bin/fbidq.html?7B2), [alph](http://flybase.bio.indiana.edu/.bin/fbidq.html?alph), [Spf45](http://flybase.bio.indiana.edu/.bin/fbidq.html?Spf45), [Fer2](http://flybase.bio.indiana.edu/.bin/fbidq.html?Fer2), [fd102C](http://flybase.bio.indiana.edu/.bin/fbidq.html?fd102C), [hth](http://flybase.bio.indiana.edu/.bin/fbidq.html?hth), [Nf-YC](http://flybase.bio.indiana.edu/.bin/fbidq.html?Nf-YC), [Hel25E](http://flybase.bio.indiana.edu/.bin/fbidq.html?Hel25E), [Hey](http://flybase.bio.indiana.edu/.bin/fbidq.html?Hey), [loqs](http://flybase.bio.indiana.edu/.bin/fbidq.html?loqs),[CycE](http://flybase.bio.indiana.edu/.bin/fbidq.html?CycE), [Brf](http://flybase.bio.indiana.edu/.bin/fbidq.html?Brf), [Psi](http://flybase.bio.indiana.edu/.bin/fbidq.html?Psi), [Gug](http://flybase.bio.indiana.edu/.bin/fbidq.html?Gug), [pad](http://flybase.bio.indiana.edu/.bin/fbidq.html?pad), [orb2](http://flybase.bio.indiana.edu/.bin/fbidq.html?orb2), [Rx](http://flybase.bio.indiana.edu/.bin/fbidq.html?Rx), [MED1](http://flybase.bio.indiana.edu/.bin/fbidq.html?MED1), [salr](http://flybase.bio.indiana.edu/.bin/fbidq.html?salr), [wdn](http://flybase.bio.indiana.edu/.bin/fbidq.html?wdn), [sim](http://flybase.bio.indiana.edu/.bin/fbidq.html?sim), [d4](http://flybase.bio.indiana.edu/.bin/fbidq.html?d4), [cort](http://flybase.bio.indiana.edu/.bin/fbidq.html?cort), [dan](http://flybase.bio.indiana.edu/.bin/fbidq.html?dan), [gro](http://flybase.bio.indiana.edu/.bin/fbidq.html?gro), [Jarid2](http://flybase.bio.indiana.edu/.bin/fbidq.html?Jarid2), [RN-tre](http://flybase.bio.indiana.edu/.bin/fbidq.html?RN-tre), [bin3](http://flybase.bio.indiana.edu/.bin/fbidq.html?bin3), [trx](http://flybase.bio.indiana.edu/.bin/fbidq.html?trx), [Rpd3](http://flybase.bio.indiana.edu/.bin/fbidq.html?Rpd3), [tyf](http://flybase.bio.indiana.edu/.bin/fbidq.html?tyf), [vfl](http://flybase.bio.indiana.edu/.bin/fbidq.html?vfl), [aret](http://flybase.bio.indiana.edu/.bin/fbidq.html?aret), [pygo](http://flybase.bio.indiana.edu/.bin/fbidq.html?pygo), [HLHm7](http://flybase.bio.indiana.edu/.bin/fbidq.html?HLHm7), [z](http://flybase.bio.indiana.edu/.bin/fbidq.html?z), [HLHmgamma](http://flybase.bio.indiana.edu/.bin/fbidq.html?HLHmgamma), [Sox21b](http://flybase.bio.indiana.edu/.bin/fbidq.html?Sox21b), [wor](http://flybase.bio.indiana.edu/.bin/fbidq.html?wor), [wda](http://flybase.bio.indiana.edu/.bin/fbidq.html?wda), [mira](http://flybase.bio.indiana.edu/.bin/fbidq.html?mira), [MED14](http://flybase.bio.indiana.edu/.bin/fbidq.html?MED14), [pUf68](http://flybase.bio.indiana.edu/.bin/fbidq.html?pUf68), [seq](http://flybase.bio.indiana.edu/.bin/fbidq.html?seq), [Dip3](http://flybase.bio.indiana.edu/.bin/fbidq.html?Dip3), [HDAC4](http://flybase.bio.indiana.edu/.bin/fbidq.html?HDAC4), [elk](http://flybase.bio.indiana.edu/.bin/fbidq.html?elk), [phl](http://flybase.bio.indiana.edu/.bin/fbidq.html?phl), [Cbl](http://flybase.bio.indiana.edu/.bin/fbidq.html?Cbl), [sNPF](http://flybase.bio.indiana.edu/.bin/fbidq.html?sNPF), [Pdp1](http://flybase.bio.indiana.edu/.bin/fbidq.html?Pdp1),[plx](http://flybase.bio.indiana.edu/.bin/fbidq.html?plx), [Alk](http://flybase.bio.indiana.edu/.bin/fbidq.html?Alk), [Sin3A](http://flybase.bio.indiana.edu/.bin/fbidq.html?Sin3A), [Pde1c](http://flybase.bio.indiana.edu/.bin/fbidq.html?Pde1c), [mip120](http://flybase.bio.indiana.edu/.bin/fbidq.html?mip120), [casp](http://flybase.bio.indiana.edu/.bin/fbidq.html?casp), [jumu](http://flybase.bio.indiana.edu/.bin/fbidq.html?jumu), [Smox](http://flybase.bio.indiana.edu/.bin/fbidq.html?Smox), [Usp7](http://flybase.bio.indiana.edu/.bin/fbidq.html?Usp7), [fd59A](http://flybase.bio.indiana.edu/.bin/fbidq.html?fd59A), [brat](http://flybase.bio.indiana.edu/.bin/fbidq.html?brat), [FBX011](http://flybase.bio.indiana.edu/.bin/fbidq.html?FBX011), [Antp](http://flybase.bio.indiana.edu/.bin/fbidq.html?Antp), [l(3)neo38](http://flybase.bio.indiana.edu/.bin/fbidq.html?l(3)neo38), [Ast-C](http://flybase.bio.indiana.edu/.bin/fbidq.html?Ast-C), [Ubx](http://flybase.bio.indiana.edu/.bin/fbidq.html?Ubx), [Ptx1](http://flybase.bio.indiana.edu/.bin/fbidq.html?Ptx1), [chm](http://flybase.bio.indiana.edu/.bin/fbidq.html?chm), [hbn](http://flybase.bio.indiana.edu/.bin/fbidq.html?hbn), [tsh](http://flybase.bio.indiana.edu/.bin/fbidq.html?tsh), [Rgl](http://flybase.bio.indiana.edu/.bin/fbidq.html?Rgl), [e(y)3](http://flybase.bio.indiana.edu/.bin/fbidq.html?e(y)3), [CG9007](http://flybase.bio.indiana.edu/.bin/fbidq.html?CG9007), [oc](http://flybase.bio.indiana.edu/.bin/fbidq.html?oc), [spt4](http://flybase.bio.indiana.edu/.bin/fbidq.html?spt4), [gol](http://flybase.bio.indiana.edu/.bin/fbidq.html?gol), [ey](http://flybase.bio.indiana.edu/.bin/fbidq.html?ey), [Bgb](http://flybase.bio.indiana.edu/.bin/fbidq.html?Bgb), [l(1)sc](http://flybase.bio.indiana.edu/.bin/fbidq.html?l(1)sc), [grh](http://flybase.bio.indiana.edu/.bin/fbidq.html?grh), [Vsx1](http://flybase.bio.indiana.edu/.bin/fbidq.html?Vsx1), [toy](http://flybase.bio.indiana.edu/.bin/fbidq.html?toy), [Lim3](http://flybase.bio.indiana.edu/.bin/fbidq.html?Lim3), [CG34362](http://flybase.bio.indiana.edu/.bin/fbidq.html?CG34362), [crol](http://flybase.bio.indiana.edu/.bin/fbidq.html?crol), [sd](http://flybase.bio.indiana.edu/.bin/fbidq.html?sd), [retn](http://flybase.bio.indiana.edu/.bin/fbidq.html?retn), [fs(1)h](http://flybase.bio.indiana.edu/.bin/fbidq.html?fs(1)h), [rl](http://flybase.bio.indiana.edu/.bin/fbidq.html?rl),[CG11347](http://flybase.bio.indiana.edu/.bin/fbidq.html?CG11347), [tey](http://flybase.bio.indiana.edu/.bin/fbidq.html?tey), [Saf-B](http://flybase.bio.indiana.edu/.bin/fbidq.html?Saf-B), [CG11294](http://flybase.bio.indiana.edu/.bin/fbidq.html?CG11294), [hiw](http://flybase.bio.indiana.edu/.bin/fbidq.html?hiw), [Atf-2](http://flybase.bio.indiana.edu/.bin/fbidq.html?Atf-2), [acj6](http://flybase.bio.indiana.edu/.bin/fbidq.html?acj6), [skd](http://flybase.bio.indiana.edu/.bin/fbidq.html?skd), [jing](http://flybase.bio.indiana.edu/.bin/fbidq.html?jing), [caup](http://flybase.bio.indiana.edu/.bin/fbidq.html?caup), [Camta](http://flybase.bio.indiana.edu/.bin/fbidq.html?Camta), [tll](http://flybase.bio.indiana.edu/.bin/fbidq.html?tll), [ato](http://flybase.bio.indiana.edu/.bin/fbidq.html?ato), [Rox8](http://flybase.bio.indiana.edu/.bin/fbidq.html?Rox8), [Iswi](http://flybase.bio.indiana.edu/.bin/fbidq.html?Iswi), [Spt6](http://flybase.bio.indiana.edu/.bin/fbidq.html?Spt6), [Dg](http://flybase.bio.indiana.edu/.bin/fbidq.html?Dg), [Pka-R1](http://flybase.bio.indiana.edu/.bin/fbidq.html?Pka-R1), [Evi5](http://flybase.bio.indiana.edu/.bin/fbidq.html?Evi5), [br](http://flybase.bio.indiana.edu/.bin/fbidq.html?br), [ksr](http://flybase.bio.indiana.edu/.bin/fbidq.html?ksr), [PNUTS](http://flybase.bio.indiana.edu/.bin/fbidq.html?PNUTS), [chn](http://flybase.bio.indiana.edu/.bin/fbidq.html?chn), [Gsc](http://flybase.bio.indiana.edu/.bin/fbidq.html?Gsc), [lola](http://flybase.bio.indiana.edu/.bin/fbidq.html?lola), [chif](http://flybase.bio.indiana.edu/.bin/fbidq.html?chif), [Pcl](http://flybase.bio.indiana.edu/.bin/fbidq.html?Pcl), [HLH4C](http://flybase.bio.indiana.edu/.bin/fbidq.html?HLH4C), [simj](http://flybase.bio.indiana.edu/.bin/fbidq.html?simj), [tap](http://flybase.bio.indiana.edu/.bin/fbidq.html?tap), [Awh](http://flybase.bio.indiana.edu/.bin/fbidq.html?Awh), [PHDP](http://flybase.bio.indiana.edu/.bin/fbidq.html?PHDP), [CG4328](http://flybase.bio.indiana.edu/.bin/fbidq.html?CG4328), [Su(var)3-3](http://flybase.bio.indiana.edu/.bin/fbidq.html?Su(var)3-3), [Taf4](http://flybase.bio.indiana.edu/.bin/fbidq.html?Taf4), [l(2)NC136](http://flybase.bio.indiana.edu/.bin/fbidq.html?l(2)NC136), [stau](http://flybase.bio.indiana.edu/.bin/fbidq.html?stau), [Sos](http://flybase.bio.indiana.edu/.bin/fbidq.html?Sos), [vg](http://flybase.bio.indiana.edu/.bin/fbidq.html?vg),[eya](http://flybase.bio.indiana.edu/.bin/fbidq.html?eya), [bsk](http://flybase.bio.indiana.edu/.bin/fbidq.html?bsk), [brm](http://flybase.bio.indiana.edu/.bin/fbidq.html?brm), [fz](http://flybase.bio.indiana.edu/.bin/fbidq.html?fz), [Vsx2](http://flybase.bio.indiana.edu/.bin/fbidq.html?Vsx2), [sno](http://flybase.bio.indiana.edu/.bin/fbidq.html?sno), [ara](http://flybase.bio.indiana.edu/.bin/fbidq.html?ara), [Sfmbt](http://flybase.bio.indiana.edu/.bin/fbidq.html?Sfmbt), [mam](http://flybase.bio.indiana.edu/.bin/fbidq.html?mam), [pan](http://flybase.bio.indiana.edu/.bin/fbidq.html?pan), [disco](http://flybase.bio.indiana.edu/.bin/fbidq.html?disco), [Dll](http://flybase.bio.indiana.edu/.bin/fbidq.html?Dll), [grn](http://flybase.bio.indiana.edu/.bin/fbidq.html?grn), [D12](http://flybase.bio.indiana.edu/.bin/fbidq.html?D12), [HLHm5](http://flybase.bio.indiana.edu/.bin/fbidq.html?HLHm5), [Lis-1](http://flybase.bio.indiana.edu/.bin/fbidq.html?Lis-1), [ems](http://flybase.bio.indiana.edu/.bin/fbidq.html?ems), [dap](http://flybase.bio.indiana.edu/.bin/fbidq.html?dap), [CG16896](http://flybase.bio.indiana.edu/.bin/fbidq.html?CG16896), [tup](http://flybase.bio.indiana.edu/.bin/fbidq.html?tup), [pros](http://flybase.bio.indiana.edu/.bin/fbidq.html?pros), [pdm2](http://flybase.bio.indiana.edu/.bin/fbidq.html?pdm2), [rgr](http://flybase.bio.indiana.edu/.bin/fbidq.html?rgr), [spel1](http://flybase.bio.indiana.edu/.bin/fbidq.html?spel1), [Wnt4](http://flybase.bio.indiana.edu/.bin/fbidq.html?Wnt4), [CG30372](http://flybase.bio.indiana.edu/.bin/fbidq.html?CG30372), [nerfin-1](http://flybase.bio.indiana.edu/.bin/fbidq.html?nerfin-1), [CG42629](http://flybase.bio.indiana.edu/.bin/fbidq.html?CG42629), [bchs](http://flybase.bio.indiana.edu/.bin/fbidq.html?bchs), [pr-set7](http://flybase.bio.indiana.edu/.bin/fbidq.html?pr-set7), [dsx](http://flybase.bio.indiana.edu/.bin/fbidq.html?dsx), [Ssdp](http://flybase.bio.indiana.edu/.bin/fbidq.html?Ssdp), [sqz](http://flybase.bio.indiana.edu/.bin/fbidq.html?sqz), [CG8155](http://flybase.bio.indiana.edu/.bin/fbidq.html?CG8155), [Fer3](http://flybase.bio.indiana.edu/.bin/fbidq.html?Fer3), [CG32105](http://flybase.bio.indiana.edu/.bin/fbidq.html?CG32105), [dalao](http://flybase.bio.indiana.edu/.bin/fbidq.html?dalao),[Lim1](http://flybase.bio.indiana.edu/.bin/fbidq.html?Lim1), [sr](http://flybase.bio.indiana.edu/.bin/fbidq.html?sr), [tld](http://flybase.bio.indiana.edu/.bin/fbidq.html?tld), [danr](http://flybase.bio.indiana.edu/.bin/fbidq.html?danr), [vvl](http://flybase.bio.indiana.edu/.bin/fbidq.html?vvl), [CG7879](http://flybase.bio.indiana.edu/.bin/fbidq.html?CG7879), [sgg](http://flybase.bio.indiana.edu/.bin/fbidq.html?sgg), [gcm2](http://flybase.bio.indiana.edu/.bin/fbidq.html?gcm2), [Eip93F](http://flybase.bio.indiana.edu/.bin/fbidq.html?Eip93F), [gcm](http://flybase.bio.indiana.edu/.bin/fbidq.html?gcm), [can](http://flybase.bio.indiana.edu/.bin/fbidq.html?can), [Aef1](http://flybase.bio.indiana.edu/.bin/fbidq.html?Aef1), [Hsp67Bc](http://flybase.bio.indiana.edu/.bin/fbidq.html?Hsp67Bc), [rab3-GEF](http://flybase.bio.indiana.edu/.bin/fbidq.html?rab3-GEF), [ase](http://flybase.bio.indiana.edu/.bin/fbidq.html?ase), [ovo](http://flybase.bio.indiana.edu/.bin/fbidq.html?ovo), [CG13253](http://flybase.bio.indiana.edu/.bin/fbidq.html?CG13253), [CG6197](http://flybase.bio.indiana.edu/.bin/fbidq.html?CG6197), [scro](http://flybase.bio.indiana.edu/.bin/fbidq.html?scro), [Dsp1](http://flybase.bio.indiana.edu/.bin/fbidq.html?Dsp1), [elav](http://flybase.bio.indiana.edu/.bin/fbidq.html?elav), [CG14216](http://flybase.bio.indiana.edu/.bin/fbidq.html?CG14216), [Oli](http://flybase.bio.indiana.edu/.bin/fbidq.html?Oli), [Nelf-A](http://flybase.bio.indiana.edu/.bin/fbidq.html?Nelf-A), [GATAd](http://flybase.bio.indiana.edu/.bin/fbidq.html?GATAd), [CG6227](http://flybase.bio.indiana.edu/.bin/fbidq.html?CG6227), [Poxn](http://flybase.bio.indiana.edu/.bin/fbidq.html?Poxn), [fbl6](http://flybase.bio.indiana.edu/.bin/fbidq.html?fbl6), [CG15376](http://flybase.bio.indiana.edu/.bin/fbidq.html?CG15376), [ush](http://flybase.bio.indiana.edu/.bin/fbidq.html?ush), [CG10336](http://flybase.bio.indiana.edu/.bin/fbidq.html?CG10336), [Gap1](http://flybase.bio.indiana.edu/.bin/fbidq.html?Gap1), [run](http://flybase.bio.indiana.edu/.bin/fbidq.html?run), [Alh](http://flybase.bio.indiana.edu/.bin/fbidq.html?Alh), [tara](http://flybase.bio.indiana.edu/.bin/fbidq.html?tara),[egg](http://flybase.bio.indiana.edu/.bin/fbidq.html?egg), [Sox102F](http://flybase.bio.indiana.edu/.bin/fbidq.html?Sox102F), [CG5337](http://flybase.bio.indiana.edu/.bin/fbidq.html?CG5337), [koko](http://flybase.bio.indiana.edu/.bin/fbidq.html?koko), [Ptth](http://flybase.bio.indiana.edu/.bin/fbidq.html?Ptth), [Sp1](http://flybase.bio.indiana.edu/.bin/fbidq.html?Sp1), [ph-p](http://flybase.bio.indiana.edu/.bin/fbidq.html?ph-p), [onecut](http://flybase.bio.indiana.edu/.bin/fbidq.html?onecut), [CG7757](http://flybase.bio.indiana.edu/.bin/fbidq.html?CG7757), [sna](http://flybase.bio.indiana.edu/.bin/fbidq.html?sna), [Wnt5](http://flybase.bio.indiana.edu/.bin/fbidq.html?Wnt5), [Taf1](http://flybase.bio.indiana.edu/.bin/fbidq.html?Taf1), [MBD-like](http://flybase.bio.indiana.edu/.bin/fbidq.html?MBD-like), [esg](http://flybase.bio.indiana.edu/.bin/fbidq.html?esg), [sif](http://flybase.bio.indiana.edu/.bin/fbidq.html?sif), [dom](http://flybase.bio.indiana.edu/.bin/fbidq.html?dom), [MTA1-like](http://flybase.bio.indiana.edu/.bin/fbidq.html?MTA1-like), [CG32532](http://flybase.bio.indiana.edu/.bin/fbidq.html?CG32532), [mle](http://flybase.bio.indiana.edu/.bin/fbidq.html?mle), [Hr51](http://flybase.bio.indiana.edu/.bin/fbidq.html?Hr51), [B-H2](http://flybase.bio.indiana.edu/.bin/fbidq.html?B-H2), [wg](http://flybase.bio.indiana.edu/.bin/fbidq.html?wg), [fkh](http://flybase.bio.indiana.edu/.bin/fbidq.html?fkh), [fne](http://flybase.bio.indiana.edu/.bin/fbidq.html?fne), [en](http://flybase.bio.indiana.edu/.bin/fbidq.html?en), [Utx](http://flybase.bio.indiana.edu/.bin/fbidq.html?Utx), [cos](http://flybase.bio.indiana.edu/.bin/fbidq.html?cos), [Ct](http://flybase.bio.indiana.edu/.bin/fbidq.html?Ct) |
| [response to chemical stimulus](http://amigo.geneontology.org/cgi-bin/amigo/go.cgi?view=details&query=GO:0042221) | 166 of 1594 genes, 10.4% | 521 of 7634 genes, 6.8% | 2.48e-08 | 0.00% | 0.00 | [fz2](http://flybase.bio.indiana.edu/.bin/fbidq.html?fz2), [Sh](http://flybase.bio.indiana.edu/.bin/fbidq.html?Sh), [acj6](http://flybase.bio.indiana.edu/.bin/fbidq.html?acj6), [jing](http://flybase.bio.indiana.edu/.bin/fbidq.html?jing), [dock](http://flybase.bio.indiana.edu/.bin/fbidq.html?dock), [Cbp80](http://flybase.bio.indiana.edu/.bin/fbidq.html?Cbp80), [Ela](http://flybase.bio.indiana.edu/.bin/fbidq.html?Ela), [tll](http://flybase.bio.indiana.edu/.bin/fbidq.html?tll), [Spn](http://flybase.bio.indiana.edu/.bin/fbidq.html?Spn), [CG17734](http://flybase.bio.indiana.edu/.bin/fbidq.html?CG17734), [Iswi](http://flybase.bio.indiana.edu/.bin/fbidq.html?Iswi), [ap](http://flybase.bio.indiana.edu/.bin/fbidq.html?ap), [Dg](http://flybase.bio.indiana.edu/.bin/fbidq.html?Dg), [Trim9](http://flybase.bio.indiana.edu/.bin/fbidq.html?Trim9), [Nf1](http://flybase.bio.indiana.edu/.bin/fbidq.html?Nf1), [Pka-R1](http://flybase.bio.indiana.edu/.bin/fbidq.html?Pka-R1), [br](http://flybase.bio.indiana.edu/.bin/fbidq.html?br), [robo3](http://flybase.bio.indiana.edu/.bin/fbidq.html?robo3), [Gyc-89Da](http://flybase.bio.indiana.edu/.bin/fbidq.html?Gyc-89Da), [tok](http://flybase.bio.indiana.edu/.bin/fbidq.html?tok), [Ptp99A](http://flybase.bio.indiana.edu/.bin/fbidq.html?Ptp99A), [Traf6](http://flybase.bio.indiana.edu/.bin/fbidq.html?Traf6), [lola](http://flybase.bio.indiana.edu/.bin/fbidq.html?lola), [unc-104](http://flybase.bio.indiana.edu/.bin/fbidq.html?unc-104), [VAChT](http://flybase.bio.indiana.edu/.bin/fbidq.html?VAChT), [chif](http://flybase.bio.indiana.edu/.bin/fbidq.html?chif), [NetB](http://flybase.bio.indiana.edu/.bin/fbidq.html?NetB), [Nrk](http://flybase.bio.indiana.edu/.bin/fbidq.html?Nrk), [Galpha49B](http://flybase.bio.indiana.edu/.bin/fbidq.html?Galpha49B), [Snoo](http://flybase.bio.indiana.edu/.bin/fbidq.html?Snoo), [Ilp3](http://flybase.bio.indiana.edu/.bin/fbidq.html?Ilp3), [Lar](http://flybase.bio.indiana.edu/.bin/fbidq.html?Lar), [scrib](http://flybase.bio.indiana.edu/.bin/fbidq.html?scrib), [not](http://flybase.bio.indiana.edu/.bin/fbidq.html?not), [rut](http://flybase.bio.indiana.edu/.bin/fbidq.html?rut), [nvy](http://flybase.bio.indiana.edu/.bin/fbidq.html?nvy), [gogo](http://flybase.bio.indiana.edu/.bin/fbidq.html?gogo), [CG8745](http://flybase.bio.indiana.edu/.bin/fbidq.html?CG8745), [Mmp2](http://flybase.bio.indiana.edu/.bin/fbidq.html?Mmp2),[jeb](http://flybase.bio.indiana.edu/.bin/fbidq.html?jeb), [Vha68-1](http://flybase.bio.indiana.edu/.bin/fbidq.html?Vha68-1), [Sema-1a](http://flybase.bio.indiana.edu/.bin/fbidq.html?Sema-1a), [beat-Ib](http://flybase.bio.indiana.edu/.bin/fbidq.html?beat-Ib), [otk](http://flybase.bio.indiana.edu/.bin/fbidq.html?otk), [CG34400](http://flybase.bio.indiana.edu/.bin/fbidq.html?CG34400), [Ulp1](http://flybase.bio.indiana.edu/.bin/fbidq.html?Ulp1), [ena](http://flybase.bio.indiana.edu/.bin/fbidq.html?ena), [dsh](http://flybase.bio.indiana.edu/.bin/fbidq.html?dsh), [pzg](http://flybase.bio.indiana.edu/.bin/fbidq.html?pzg), [Fas2](http://flybase.bio.indiana.edu/.bin/fbidq.html?Fas2), [Nf-YC](http://flybase.bio.indiana.edu/.bin/fbidq.html?Nf-YC), [14-3-3zeta](http://flybase.bio.indiana.edu/.bin/fbidq.html?14-3-3zeta), [robo](http://flybase.bio.indiana.edu/.bin/fbidq.html?robo), [lea](http://flybase.bio.indiana.edu/.bin/fbidq.html?lea), [bsk](http://flybase.bio.indiana.edu/.bin/fbidq.html?bsk), [beat-Ia](http://flybase.bio.indiana.edu/.bin/fbidq.html?beat-Ia), [nAcRalpha-30D](http://flybase.bio.indiana.edu/.bin/fbidq.html?nAcRalpha-30D), [plexB](http://flybase.bio.indiana.edu/.bin/fbidq.html?plexB), [loqs](http://flybase.bio.indiana.edu/.bin/fbidq.html?loqs), [Klp64D](http://flybase.bio.indiana.edu/.bin/fbidq.html?Klp64D), [smi35A](http://flybase.bio.indiana.edu/.bin/fbidq.html?smi35A), [Dll](http://flybase.bio.indiana.edu/.bin/fbidq.html?Dll), [grn](http://flybase.bio.indiana.edu/.bin/fbidq.html?grn), [DopR](http://flybase.bio.indiana.edu/.bin/fbidq.html?DopR), [mew](http://flybase.bio.indiana.edu/.bin/fbidq.html?mew), [neur](http://flybase.bio.indiana.edu/.bin/fbidq.html?neur), [Lis-1](http://flybase.bio.indiana.edu/.bin/fbidq.html?Lis-1), [Bx](http://flybase.bio.indiana.edu/.bin/fbidq.html?Bx), [Mob2](http://flybase.bio.indiana.edu/.bin/fbidq.html?Mob2), [pros](http://flybase.bio.indiana.edu/.bin/fbidq.html?pros), [tup](http://flybase.bio.indiana.edu/.bin/fbidq.html?tup), [trio](http://flybase.bio.indiana.edu/.bin/fbidq.html?trio), [daw](http://flybase.bio.indiana.edu/.bin/fbidq.html?daw), [Gad1](http://flybase.bio.indiana.edu/.bin/fbidq.html?Gad1),[Wnt4](http://flybase.bio.indiana.edu/.bin/fbidq.html?Wnt4), [unc79](http://flybase.bio.indiana.edu/.bin/fbidq.html?unc79), [rogdi](http://flybase.bio.indiana.edu/.bin/fbidq.html?rogdi), [Apc](http://flybase.bio.indiana.edu/.bin/fbidq.html?Apc), [sim](http://flybase.bio.indiana.edu/.bin/fbidq.html?sim), [tau](http://flybase.bio.indiana.edu/.bin/fbidq.html?tau), [CG42629](http://flybase.bio.indiana.edu/.bin/fbidq.html?CG42629), [nerfin-1](http://flybase.bio.indiana.edu/.bin/fbidq.html?nerfin-1), [bin3](http://flybase.bio.indiana.edu/.bin/fbidq.html?bin3), [trx](http://flybase.bio.indiana.edu/.bin/fbidq.html?trx), [dsx](http://flybase.bio.indiana.edu/.bin/fbidq.html?dsx), [PQBP-1](http://flybase.bio.indiana.edu/.bin/fbidq.html?PQBP-1), [unc-5](http://flybase.bio.indiana.edu/.bin/fbidq.html?unc-5), [CG10107](http://flybase.bio.indiana.edu/.bin/fbidq.html?CG10107), [fend](http://flybase.bio.indiana.edu/.bin/fbidq.html?fend), [tld](http://flybase.bio.indiana.edu/.bin/fbidq.html?tld), [NaCP60E](http://flybase.bio.indiana.edu/.bin/fbidq.html?NaCP60E), [CG14509](http://flybase.bio.indiana.edu/.bin/fbidq.html?CG14509), [vvl](http://flybase.bio.indiana.edu/.bin/fbidq.html?vvl), [rg](http://flybase.bio.indiana.edu/.bin/fbidq.html?rg), [Pten](http://flybase.bio.indiana.edu/.bin/fbidq.html?Pten), [betaInt-nu](http://flybase.bio.indiana.edu/.bin/fbidq.html?betaInt-nu), [npf](http://flybase.bio.indiana.edu/.bin/fbidq.html?npf), [Ptp69D](http://flybase.bio.indiana.edu/.bin/fbidq.html?Ptp69D), [sgg](http://flybase.bio.indiana.edu/.bin/fbidq.html?sgg), [mnb](http://flybase.bio.indiana.edu/.bin/fbidq.html?mnb), [Eip93F](http://flybase.bio.indiana.edu/.bin/fbidq.html?Eip93F), [Nmdar1](http://flybase.bio.indiana.edu/.bin/fbidq.html?Nmdar1), [dpr9](http://flybase.bio.indiana.edu/.bin/fbidq.html?dpr9), [hts](http://flybase.bio.indiana.edu/.bin/fbidq.html?hts), [mp](http://flybase.bio.indiana.edu/.bin/fbidq.html?mp), [gcm](http://flybase.bio.indiana.edu/.bin/fbidq.html?gcm), [seq](http://flybase.bio.indiana.edu/.bin/fbidq.html?seq), [Hsp67Bc](http://flybase.bio.indiana.edu/.bin/fbidq.html?Hsp67Bc), [sNPF](http://flybase.bio.indiana.edu/.bin/fbidq.html?sNPF),[hang](http://flybase.bio.indiana.edu/.bin/fbidq.html?hang), [Pdp1](http://flybase.bio.indiana.edu/.bin/fbidq.html?Pdp1), [CG10251](http://flybase.bio.indiana.edu/.bin/fbidq.html?CG10251), [mtt](http://flybase.bio.indiana.edu/.bin/fbidq.html?mtt), [Alk](http://flybase.bio.indiana.edu/.bin/fbidq.html?Alk), [Poxn](http://flybase.bio.indiana.edu/.bin/fbidq.html?Poxn), [Smox](http://flybase.bio.indiana.edu/.bin/fbidq.html?Smox), [beat-Ic](http://flybase.bio.indiana.edu/.bin/fbidq.html?beat-Ic), [fd59A](http://flybase.bio.indiana.edu/.bin/fbidq.html?fd59A), [Ilp5](http://flybase.bio.indiana.edu/.bin/fbidq.html?Ilp5), [run](http://flybase.bio.indiana.edu/.bin/fbidq.html?run), [MESR4](http://flybase.bio.indiana.edu/.bin/fbidq.html?MESR4), [brat](http://flybase.bio.indiana.edu/.bin/fbidq.html?brat), [ko](http://flybase.bio.indiana.edu/.bin/fbidq.html?ko), [Eip71CD](http://flybase.bio.indiana.edu/.bin/fbidq.html?Eip71CD), [Rdl](http://flybase.bio.indiana.edu/.bin/fbidq.html?Rdl), [koko](http://flybase.bio.indiana.edu/.bin/fbidq.html?koko), [stan](http://flybase.bio.indiana.edu/.bin/fbidq.html?stan), [GABA-B-R2](http://flybase.bio.indiana.edu/.bin/fbidq.html?GABA-B-R2), [CG12424](http://flybase.bio.indiana.edu/.bin/fbidq.html?CG12424), [tutl](http://flybase.bio.indiana.edu/.bin/fbidq.html?tutl), [futsch](http://flybase.bio.indiana.edu/.bin/fbidq.html?futsch), [Ilp2](http://flybase.bio.indiana.edu/.bin/fbidq.html?Ilp2), [ph-p](http://flybase.bio.indiana.edu/.bin/fbidq.html?ph-p), [CG3967](http://flybase.bio.indiana.edu/.bin/fbidq.html?CG3967), [gukh](http://flybase.bio.indiana.edu/.bin/fbidq.html?gukh), [Wnt5](http://flybase.bio.indiana.edu/.bin/fbidq.html?Wnt5), [pigeon](http://flybase.bio.indiana.edu/.bin/fbidq.html?pigeon), [beat-IIa](http://flybase.bio.indiana.edu/.bin/fbidq.html?beat-IIa), [Ggamma1](http://flybase.bio.indiana.edu/.bin/fbidq.html?Ggamma1), [para](http://flybase.bio.indiana.edu/.bin/fbidq.html?para), [sm](http://flybase.bio.indiana.edu/.bin/fbidq.html?sm), [esg](http://flybase.bio.indiana.edu/.bin/fbidq.html?esg), [dac](http://flybase.bio.indiana.edu/.bin/fbidq.html?dac), [Cdk5](http://flybase.bio.indiana.edu/.bin/fbidq.html?Cdk5),[mav](http://flybase.bio.indiana.edu/.bin/fbidq.html?mav), [Tbh](http://flybase.bio.indiana.edu/.bin/fbidq.html?Tbh), [Ilp7](http://flybase.bio.indiana.edu/.bin/fbidq.html?Ilp7), [Hr51](http://flybase.bio.indiana.edu/.bin/fbidq.html?Hr51), [shi](http://flybase.bio.indiana.edu/.bin/fbidq.html?shi), [comm](http://flybase.bio.indiana.edu/.bin/fbidq.html?comm), [Cdk5alpha](http://flybase.bio.indiana.edu/.bin/fbidq.html?Cdk5alpha), [Vmat](http://flybase.bio.indiana.edu/.bin/fbidq.html?Vmat), [fkh](http://flybase.bio.indiana.edu/.bin/fbidq.html?fkh), [Lim3](http://flybase.bio.indiana.edu/.bin/fbidq.html?Lim3), [CadN](http://flybase.bio.indiana.edu/.bin/fbidq.html?CadN), [retn](http://flybase.bio.indiana.edu/.bin/fbidq.html?retn), [E(bx)](http://flybase.bio.indiana.edu/.bin/fbidq.html?E(bx)), [en](http://flybase.bio.indiana.edu/.bin/fbidq.html?en), [klg](http://flybase.bio.indiana.edu/.bin/fbidq.html?klg), [sfl](http://flybase.bio.indiana.edu/.bin/fbidq.html?sfl), [rl](http://flybase.bio.indiana.edu/.bin/fbidq.html?rl), [Sip1](http://flybase.bio.indiana.edu/.bin/fbidq.html?Sip1), [trn](http://flybase.bio.indiana.edu/.bin/fbidq.html?trn), [CG11347](http://flybase.bio.indiana.edu/.bin/fbidq.html?CG11347), [Ggamma30A](http://flybase.bio.indiana.edu/.bin/fbidq.html?Ggamma30A), [CG33960](http://flybase.bio.indiana.edu/.bin/fbidq.html?CG33960) |
| [neurological system process](http://amigo.geneontology.org/cgi-bin/amigo/go.cgi?view=details&query=GO:0050877) | 141 of 1594 genes, 8.8% | 424 of 7634 genes, 5.6% | 3.39e-08 | 0.00% | 0.00 | [endoA](http://flybase.bio.indiana.edu/.bin/fbidq.html?endoA), [Sh](http://flybase.bio.indiana.edu/.bin/fbidq.html?Sh), [n-syb](http://flybase.bio.indiana.edu/.bin/fbidq.html?n-syb), [acj6](http://flybase.bio.indiana.edu/.bin/fbidq.html?acj6), [ato](http://flybase.bio.indiana.edu/.bin/fbidq.html?ato), [stj](http://flybase.bio.indiana.edu/.bin/fbidq.html?stj), [Dg](http://flybase.bio.indiana.edu/.bin/fbidq.html?Dg), [Nf1](http://flybase.bio.indiana.edu/.bin/fbidq.html?Nf1), [Pka-R1](http://flybase.bio.indiana.edu/.bin/fbidq.html?Pka-R1), [os](http://flybase.bio.indiana.edu/.bin/fbidq.html?os), [brp](http://flybase.bio.indiana.edu/.bin/fbidq.html?brp), [Sytalpha](http://flybase.bio.indiana.edu/.bin/fbidq.html?Sytalpha), [nonA](http://flybase.bio.indiana.edu/.bin/fbidq.html?nonA), [lola](http://flybase.bio.indiana.edu/.bin/fbidq.html?lola), [unc-104](http://flybase.bio.indiana.edu/.bin/fbidq.html?unc-104), [Khc-73](http://flybase.bio.indiana.edu/.bin/fbidq.html?Khc-73), [rdgC](http://flybase.bio.indiana.edu/.bin/fbidq.html?rdgC), [Appl](http://flybase.bio.indiana.edu/.bin/fbidq.html?Appl), [metro](http://flybase.bio.indiana.edu/.bin/fbidq.html?metro), [ewg](http://flybase.bio.indiana.edu/.bin/fbidq.html?ewg), [Cirl](http://flybase.bio.indiana.edu/.bin/fbidq.html?Cirl), [Galpha49B](http://flybase.bio.indiana.edu/.bin/fbidq.html?Galpha49B), [scrib](http://flybase.bio.indiana.edu/.bin/fbidq.html?scrib), [rut](http://flybase.bio.indiana.edu/.bin/fbidq.html?rut), [Nrg](http://flybase.bio.indiana.edu/.bin/fbidq.html?Nrg), [unc-13-4A](http://flybase.bio.indiana.edu/.bin/fbidq.html?unc-13-4A), [Ddc](http://flybase.bio.indiana.edu/.bin/fbidq.html?Ddc), [stau](http://flybase.bio.indiana.edu/.bin/fbidq.html?stau), [Sema-1a](http://flybase.bio.indiana.edu/.bin/fbidq.html?Sema-1a), [dpr15](http://flybase.bio.indiana.edu/.bin/fbidq.html?dpr15), [TBPH](http://flybase.bio.indiana.edu/.bin/fbidq.html?TBPH), [Snap25](http://flybase.bio.indiana.edu/.bin/fbidq.html?Snap25), [mGluRA](http://flybase.bio.indiana.edu/.bin/fbidq.html?mGluRA), [Fas2](http://flybase.bio.indiana.edu/.bin/fbidq.html?Fas2), [14-3-3zeta](http://flybase.bio.indiana.edu/.bin/fbidq.html?14-3-3zeta),[robo](http://flybase.bio.indiana.edu/.bin/fbidq.html?robo), [dpr20](http://flybase.bio.indiana.edu/.bin/fbidq.html?dpr20), [mtg](http://flybase.bio.indiana.edu/.bin/fbidq.html?mtg), [comt](http://flybase.bio.indiana.edu/.bin/fbidq.html?comt), [Pbprp1](http://flybase.bio.indiana.edu/.bin/fbidq.html?Pbprp1), [PP2A-B](http://flybase.bio.indiana.edu/.bin/fbidq.html?PP2A-B), [qvr](http://flybase.bio.indiana.edu/.bin/fbidq.html?qvr), [Klp64D](http://flybase.bio.indiana.edu/.bin/fbidq.html?Klp64D), [sws](http://flybase.bio.indiana.edu/.bin/fbidq.html?sws), [disco](http://flybase.bio.indiana.edu/.bin/fbidq.html?disco), [DopR](http://flybase.bio.indiana.edu/.bin/fbidq.html?DopR), [mew](http://flybase.bio.indiana.edu/.bin/fbidq.html?mew), [neur](http://flybase.bio.indiana.edu/.bin/fbidq.html?neur), [nmo](http://flybase.bio.indiana.edu/.bin/fbidq.html?nmo), [5-HT1A](http://flybase.bio.indiana.edu/.bin/fbidq.html?5-HT1A), [Mob2](http://flybase.bio.indiana.edu/.bin/fbidq.html?Mob2), [pros](http://flybase.bio.indiana.edu/.bin/fbidq.html?pros), [Rab3](http://flybase.bio.indiana.edu/.bin/fbidq.html?Rab3), [trio](http://flybase.bio.indiana.edu/.bin/fbidq.html?trio), [mr](http://flybase.bio.indiana.edu/.bin/fbidq.html?mr), [orb2](http://flybase.bio.indiana.edu/.bin/fbidq.html?orb2), [Gad1](http://flybase.bio.indiana.edu/.bin/fbidq.html?Gad1), [rogdi](http://flybase.bio.indiana.edu/.bin/fbidq.html?rogdi), [salr](http://flybase.bio.indiana.edu/.bin/fbidq.html?salr), [synaptogyrin](http://flybase.bio.indiana.edu/.bin/fbidq.html?synaptogyrin), [CaMKII](http://flybase.bio.indiana.edu/.bin/fbidq.html?CaMKII), [tau](http://flybase.bio.indiana.edu/.bin/fbidq.html?tau), [CG42629](http://flybase.bio.indiana.edu/.bin/fbidq.html?CG42629), [bchs](http://flybase.bio.indiana.edu/.bin/fbidq.html?bchs), [king-tubby](http://flybase.bio.indiana.edu/.bin/fbidq.html?king-tubby), [Btk29A](http://flybase.bio.indiana.edu/.bin/fbidq.html?Btk29A), [PQBP-1](http://flybase.bio.indiana.edu/.bin/fbidq.html?PQBP-1), [Syn1](http://flybase.bio.indiana.edu/.bin/fbidq.html?Syn1), [dpr11](http://flybase.bio.indiana.edu/.bin/fbidq.html?dpr11),[HLHm7](http://flybase.bio.indiana.edu/.bin/fbidq.html?HLHm7), [Klp68D](http://flybase.bio.indiana.edu/.bin/fbidq.html?Klp68D), [Atg1](http://flybase.bio.indiana.edu/.bin/fbidq.html?Atg1), [sei](http://flybase.bio.indiana.edu/.bin/fbidq.html?sei), [CG12187](http://flybase.bio.indiana.edu/.bin/fbidq.html?CG12187), [rg](http://flybase.bio.indiana.edu/.bin/fbidq.html?rg), [unc-13](http://flybase.bio.indiana.edu/.bin/fbidq.html?unc-13), [betaInt-nu](http://flybase.bio.indiana.edu/.bin/fbidq.html?betaInt-nu), [dpr8](http://flybase.bio.indiana.edu/.bin/fbidq.html?dpr8), [CG1909](http://flybase.bio.indiana.edu/.bin/fbidq.html?CG1909), [sgg](http://flybase.bio.indiana.edu/.bin/fbidq.html?sgg), [mnb](http://flybase.bio.indiana.edu/.bin/fbidq.html?mnb), [Nmdar1](http://flybase.bio.indiana.edu/.bin/fbidq.html?Nmdar1), [dpr9](http://flybase.bio.indiana.edu/.bin/fbidq.html?dpr9), [dpr10](http://flybase.bio.indiana.edu/.bin/fbidq.html?dpr10), [CanB](http://flybase.bio.indiana.edu/.bin/fbidq.html?CanB), [oa2](http://flybase.bio.indiana.edu/.bin/fbidq.html?oa2), [rab3-GEF](http://flybase.bio.indiana.edu/.bin/fbidq.html?rab3-GEF), [CG10251](http://flybase.bio.indiana.edu/.bin/fbidq.html?CG10251), [CASK](http://flybase.bio.indiana.edu/.bin/fbidq.html?CASK), [Syt4](http://flybase.bio.indiana.edu/.bin/fbidq.html?Syt4), [CG7708](http://flybase.bio.indiana.edu/.bin/fbidq.html?CG7708), [Alk](http://flybase.bio.indiana.edu/.bin/fbidq.html?Alk), [Dat](http://flybase.bio.indiana.edu/.bin/fbidq.html?Dat), [dpr3](http://flybase.bio.indiana.edu/.bin/fbidq.html?dpr3), [cpx](http://flybase.bio.indiana.edu/.bin/fbidq.html?cpx), [gfA](http://flybase.bio.indiana.edu/.bin/fbidq.html?gfA), [Tk](http://flybase.bio.indiana.edu/.bin/fbidq.html?Tk), [Fak56D](http://flybase.bio.indiana.edu/.bin/fbidq.html?Fak56D), [Rph](http://flybase.bio.indiana.edu/.bin/fbidq.html?Rph), [srpk79D](http://flybase.bio.indiana.edu/.bin/fbidq.html?srpk79D), [Kap3](http://flybase.bio.indiana.edu/.bin/fbidq.html?Kap3), [dpr17](http://flybase.bio.indiana.edu/.bin/fbidq.html?dpr17), [dpr14](http://flybase.bio.indiana.edu/.bin/fbidq.html?dpr14),[Rdl](http://flybase.bio.indiana.edu/.bin/fbidq.html?Rdl), [stan](http://flybase.bio.indiana.edu/.bin/fbidq.html?stan), [GABA-B-R2](http://flybase.bio.indiana.edu/.bin/fbidq.html?GABA-B-R2), [futsch](http://flybase.bio.indiana.edu/.bin/fbidq.html?futsch), [Frq1](http://flybase.bio.indiana.edu/.bin/fbidq.html?Frq1), [Rim](http://flybase.bio.indiana.edu/.bin/fbidq.html?Rim), [Shal](http://flybase.bio.indiana.edu/.bin/fbidq.html?Shal), [pigeon](http://flybase.bio.indiana.edu/.bin/fbidq.html?pigeon), [para](http://flybase.bio.indiana.edu/.bin/fbidq.html?para), [Syn2](http://flybase.bio.indiana.edu/.bin/fbidq.html?Syn2), [eas](http://flybase.bio.indiana.edu/.bin/fbidq.html?eas), [CRMP](http://flybase.bio.indiana.edu/.bin/fbidq.html?CRMP), [Nrx-1](http://flybase.bio.indiana.edu/.bin/fbidq.html?Nrx-1), [Cdk5](http://flybase.bio.indiana.edu/.bin/fbidq.html?Cdk5), [sif](http://flybase.bio.indiana.edu/.bin/fbidq.html?sif), [Tbh](http://flybase.bio.indiana.edu/.bin/fbidq.html?Tbh), [CG4641](http://flybase.bio.indiana.edu/.bin/fbidq.html?CG4641), [PIP5K59B](http://flybase.bio.indiana.edu/.bin/fbidq.html?PIP5K59B), [Oamb](http://flybase.bio.indiana.edu/.bin/fbidq.html?Oamb), [lap](http://flybase.bio.indiana.edu/.bin/fbidq.html?lap), [shi](http://flybase.bio.indiana.edu/.bin/fbidq.html?shi), [CG11155](http://flybase.bio.indiana.edu/.bin/fbidq.html?CG11155), [CG3822](http://flybase.bio.indiana.edu/.bin/fbidq.html?CG3822), [dpr5](http://flybase.bio.indiana.edu/.bin/fbidq.html?dpr5), [Vmat](http://flybase.bio.indiana.edu/.bin/fbidq.html?Vmat), [Cha](http://flybase.bio.indiana.edu/.bin/fbidq.html?Cha), [Aplip1](http://flybase.bio.indiana.edu/.bin/fbidq.html?Aplip1), [klg](http://flybase.bio.indiana.edu/.bin/fbidq.html?klg), [sfl](http://flybase.bio.indiana.edu/.bin/fbidq.html?sfl), [Syt1](http://flybase.bio.indiana.edu/.bin/fbidq.html?Syt1), [rl](http://flybase.bio.indiana.edu/.bin/fbidq.html?rl), [dpr19](http://flybase.bio.indiana.edu/.bin/fbidq.html?dpr19), [Snap](http://flybase.bio.indiana.edu/.bin/fbidq.html?Snap), [SerT](http://flybase.bio.indiana.edu/.bin/fbidq.html?SerT), [dpr7](http://flybase.bio.indiana.edu/.bin/fbidq.html?dpr7), [hiw](http://flybase.bio.indiana.edu/.bin/fbidq.html?hiw), [dpr18](http://flybase.bio.indiana.edu/.bin/fbidq.html?dpr18), [Ct](http://flybase.bio.indiana.edu/.bin/fbidq.html?Ct) |
| [regulation of gene expression](http://amigo.geneontology.org/cgi-bin/amigo/go.cgi?view=details&query=GO:0010468) | 235 of 1594 genes, 14.7% | 808 of 7634 genes, 10.6% | 3.68e-08 | 0.00% | 0.00 | [fz2](http://flybase.bio.indiana.edu/.bin/fbidq.html?fz2), [acj6](http://flybase.bio.indiana.edu/.bin/fbidq.html?acj6), [skd](http://flybase.bio.indiana.edu/.bin/fbidq.html?skd), [jing](http://flybase.bio.indiana.edu/.bin/fbidq.html?jing), [caup](http://flybase.bio.indiana.edu/.bin/fbidq.html?caup), [Cbp80](http://flybase.bio.indiana.edu/.bin/fbidq.html?Cbp80), [tll](http://flybase.bio.indiana.edu/.bin/fbidq.html?tll), [Camta](http://flybase.bio.indiana.edu/.bin/fbidq.html?Camta), [ato](http://flybase.bio.indiana.edu/.bin/fbidq.html?ato), [Ets65A](http://flybase.bio.indiana.edu/.bin/fbidq.html?Ets65A), [Rox8](http://flybase.bio.indiana.edu/.bin/fbidq.html?Rox8), [tna](http://flybase.bio.indiana.edu/.bin/fbidq.html?tna), [Sxl](http://flybase.bio.indiana.edu/.bin/fbidq.html?Sxl), [mub](http://flybase.bio.indiana.edu/.bin/fbidq.html?mub), [JIL-1](http://flybase.bio.indiana.edu/.bin/fbidq.html?JIL-1), [Spt6](http://flybase.bio.indiana.edu/.bin/fbidq.html?Spt6), [Iswi](http://flybase.bio.indiana.edu/.bin/fbidq.html?Iswi), [rn](http://flybase.bio.indiana.edu/.bin/fbidq.html?rn), [ap](http://flybase.bio.indiana.edu/.bin/fbidq.html?ap), [br](http://flybase.bio.indiana.edu/.bin/fbidq.html?br), [ksr](http://flybase.bio.indiana.edu/.bin/fbidq.html?ksr), [jigr1](http://flybase.bio.indiana.edu/.bin/fbidq.html?jigr1), [tok](http://flybase.bio.indiana.edu/.bin/fbidq.html?tok), [bi](http://flybase.bio.indiana.edu/.bin/fbidq.html?bi), [chn](http://flybase.bio.indiana.edu/.bin/fbidq.html?chn), [Gsc](http://flybase.bio.indiana.edu/.bin/fbidq.html?Gsc), [Hcf](http://flybase.bio.indiana.edu/.bin/fbidq.html?Hcf), [lola](http://flybase.bio.indiana.edu/.bin/fbidq.html?lola), [HLH4C](http://flybase.bio.indiana.edu/.bin/fbidq.html?HLH4C), [Pcl](http://flybase.bio.indiana.edu/.bin/fbidq.html?Pcl), [simj](http://flybase.bio.indiana.edu/.bin/fbidq.html?simj), [Awh](http://flybase.bio.indiana.edu/.bin/fbidq.html?Awh), [tap](http://flybase.bio.indiana.edu/.bin/fbidq.html?tap), [NC2alpha](http://flybase.bio.indiana.edu/.bin/fbidq.html?NC2alpha), [PHDP](http://flybase.bio.indiana.edu/.bin/fbidq.html?PHDP), [bab1](http://flybase.bio.indiana.edu/.bin/fbidq.html?bab1), [CG4328](http://flybase.bio.indiana.edu/.bin/fbidq.html?CG4328), [Snoo](http://flybase.bio.indiana.edu/.bin/fbidq.html?Snoo), [l(2)NC136](http://flybase.bio.indiana.edu/.bin/fbidq.html?l(2)NC136), [Taf4](http://flybase.bio.indiana.edu/.bin/fbidq.html?Taf4), [Su(var)3-3](http://flybase.bio.indiana.edu/.bin/fbidq.html?Su(var)3-3),[HGTX](http://flybase.bio.indiana.edu/.bin/fbidq.html?HGTX), [not](http://flybase.bio.indiana.edu/.bin/fbidq.html?not), [nvy](http://flybase.bio.indiana.edu/.bin/fbidq.html?nvy), [nub](http://flybase.bio.indiana.edu/.bin/fbidq.html?nub), [nab](http://flybase.bio.indiana.edu/.bin/fbidq.html?nab), [btd](http://flybase.bio.indiana.edu/.bin/fbidq.html?btd), [fd68A](http://flybase.bio.indiana.edu/.bin/fbidq.html?fd68A), [U2af38](http://flybase.bio.indiana.edu/.bin/fbidq.html?U2af38), [HLHmdelta](http://flybase.bio.indiana.edu/.bin/fbidq.html?HLHmdelta), [B-H1](http://flybase.bio.indiana.edu/.bin/fbidq.html?B-H1), [Nipped-A](http://flybase.bio.indiana.edu/.bin/fbidq.html?Nipped-A), [ATbp](http://flybase.bio.indiana.edu/.bin/fbidq.html?ATbp), [stau](http://flybase.bio.indiana.edu/.bin/fbidq.html?stau), [Sos](http://flybase.bio.indiana.edu/.bin/fbidq.html?Sos), [Spf45](http://flybase.bio.indiana.edu/.bin/fbidq.html?Spf45), [Fer2](http://flybase.bio.indiana.edu/.bin/fbidq.html?Fer2), [fd102C](http://flybase.bio.indiana.edu/.bin/fbidq.html?fd102C), [vg](http://flybase.bio.indiana.edu/.bin/fbidq.html?vg), [msl-2](http://flybase.bio.indiana.edu/.bin/fbidq.html?msl-2), [hth](http://flybase.bio.indiana.edu/.bin/fbidq.html?hth), [Nf-YC](http://flybase.bio.indiana.edu/.bin/fbidq.html?Nf-YC), [14-3-3zeta](http://flybase.bio.indiana.edu/.bin/fbidq.html?14-3-3zeta), [eya](http://flybase.bio.indiana.edu/.bin/fbidq.html?eya), [fz](http://flybase.bio.indiana.edu/.bin/fbidq.html?fz), [brm](http://flybase.bio.indiana.edu/.bin/fbidq.html?brm), [Vsx2](http://flybase.bio.indiana.edu/.bin/fbidq.html?Vsx2), [Hel25E](http://flybase.bio.indiana.edu/.bin/fbidq.html?Hel25E), [Hey](http://flybase.bio.indiana.edu/.bin/fbidq.html?Hey), [sno](http://flybase.bio.indiana.edu/.bin/fbidq.html?sno), [Sfmbt](http://flybase.bio.indiana.edu/.bin/fbidq.html?Sfmbt), [ara](http://flybase.bio.indiana.edu/.bin/fbidq.html?ara), [mam](http://flybase.bio.indiana.edu/.bin/fbidq.html?mam), [loqs](http://flybase.bio.indiana.edu/.bin/fbidq.html?loqs), [pan](http://flybase.bio.indiana.edu/.bin/fbidq.html?pan), [Dll](http://flybase.bio.indiana.edu/.bin/fbidq.html?Dll), [disco](http://flybase.bio.indiana.edu/.bin/fbidq.html?disco), [grn](http://flybase.bio.indiana.edu/.bin/fbidq.html?grn), [HLHm5](http://flybase.bio.indiana.edu/.bin/fbidq.html?HLHm5),[D12](http://flybase.bio.indiana.edu/.bin/fbidq.html?D12), [Lis-1](http://flybase.bio.indiana.edu/.bin/fbidq.html?Lis-1), [Brf](http://flybase.bio.indiana.edu/.bin/fbidq.html?Brf), [ems](http://flybase.bio.indiana.edu/.bin/fbidq.html?ems), [pros](http://flybase.bio.indiana.edu/.bin/fbidq.html?pros), [tup](http://flybase.bio.indiana.edu/.bin/fbidq.html?tup), [Psi](http://flybase.bio.indiana.edu/.bin/fbidq.html?Psi), [pdm2](http://flybase.bio.indiana.edu/.bin/fbidq.html?pdm2), [Vdup1](http://flybase.bio.indiana.edu/.bin/fbidq.html?Vdup1), [Gug](http://flybase.bio.indiana.edu/.bin/fbidq.html?Gug), [pad](http://flybase.bio.indiana.edu/.bin/fbidq.html?pad), [rgr](http://flybase.bio.indiana.edu/.bin/fbidq.html?rgr), [orb2](http://flybase.bio.indiana.edu/.bin/fbidq.html?orb2), [Wnt4](http://flybase.bio.indiana.edu/.bin/fbidq.html?Wnt4), [Rx](http://flybase.bio.indiana.edu/.bin/fbidq.html?Rx), [MED1](http://flybase.bio.indiana.edu/.bin/fbidq.html?MED1), [salr](http://flybase.bio.indiana.edu/.bin/fbidq.html?salr), [wdn](http://flybase.bio.indiana.edu/.bin/fbidq.html?wdn), [sim](http://flybase.bio.indiana.edu/.bin/fbidq.html?sim), [d4](http://flybase.bio.indiana.edu/.bin/fbidq.html?d4), [dan](http://flybase.bio.indiana.edu/.bin/fbidq.html?dan), [gro](http://flybase.bio.indiana.edu/.bin/fbidq.html?gro), [nerfin-1](http://flybase.bio.indiana.edu/.bin/fbidq.html?nerfin-1), [bin3](http://flybase.bio.indiana.edu/.bin/fbidq.html?bin3), [trx](http://flybase.bio.indiana.edu/.bin/fbidq.html?trx), [Rpd3](http://flybase.bio.indiana.edu/.bin/fbidq.html?Rpd3), [dsx](http://flybase.bio.indiana.edu/.bin/fbidq.html?dsx), [tyf](http://flybase.bio.indiana.edu/.bin/fbidq.html?tyf), [Ssdp](http://flybase.bio.indiana.edu/.bin/fbidq.html?Ssdp), [vfl](http://flybase.bio.indiana.edu/.bin/fbidq.html?vfl), [sqz](http://flybase.bio.indiana.edu/.bin/fbidq.html?sqz), [CG32105](http://flybase.bio.indiana.edu/.bin/fbidq.html?CG32105), [Fer3](http://flybase.bio.indiana.edu/.bin/fbidq.html?Fer3), [aret](http://flybase.bio.indiana.edu/.bin/fbidq.html?aret), [pygo](http://flybase.bio.indiana.edu/.bin/fbidq.html?pygo), [dalao](http://flybase.bio.indiana.edu/.bin/fbidq.html?dalao), [CG1868](http://flybase.bio.indiana.edu/.bin/fbidq.html?CG1868), [HLHm7](http://flybase.bio.indiana.edu/.bin/fbidq.html?HLHm7), [z](http://flybase.bio.indiana.edu/.bin/fbidq.html?z), [HLHmgamma](http://flybase.bio.indiana.edu/.bin/fbidq.html?HLHmgamma),[Sox21b](http://flybase.bio.indiana.edu/.bin/fbidq.html?Sox21b), [sr](http://flybase.bio.indiana.edu/.bin/fbidq.html?sr), [Lim1](http://flybase.bio.indiana.edu/.bin/fbidq.html?Lim1), [tld](http://flybase.bio.indiana.edu/.bin/fbidq.html?tld), [danr](http://flybase.bio.indiana.edu/.bin/fbidq.html?danr), [wor](http://flybase.bio.indiana.edu/.bin/fbidq.html?wor), [vvl](http://flybase.bio.indiana.edu/.bin/fbidq.html?vvl), [CG7879](http://flybase.bio.indiana.edu/.bin/fbidq.html?CG7879), [Brd8](http://flybase.bio.indiana.edu/.bin/fbidq.html?Brd8), [Pten](http://flybase.bio.indiana.edu/.bin/fbidq.html?Pten), [wda](http://flybase.bio.indiana.edu/.bin/fbidq.html?wda), [sgg](http://flybase.bio.indiana.edu/.bin/fbidq.html?sgg), [MED14](http://flybase.bio.indiana.edu/.bin/fbidq.html?MED14), [gcm2](http://flybase.bio.indiana.edu/.bin/fbidq.html?gcm2), [pUf68](http://flybase.bio.indiana.edu/.bin/fbidq.html?pUf68), [Eip93F](http://flybase.bio.indiana.edu/.bin/fbidq.html?Eip93F), [seq](http://flybase.bio.indiana.edu/.bin/fbidq.html?seq), [gcm](http://flybase.bio.indiana.edu/.bin/fbidq.html?gcm), [Dip3](http://flybase.bio.indiana.edu/.bin/fbidq.html?Dip3), [Suv4-20](http://flybase.bio.indiana.edu/.bin/fbidq.html?Suv4-20), [Aef1](http://flybase.bio.indiana.edu/.bin/fbidq.html?Aef1), [HDAC4](http://flybase.bio.indiana.edu/.bin/fbidq.html?HDAC4), [can](http://flybase.bio.indiana.edu/.bin/fbidq.html?can), [elk](http://flybase.bio.indiana.edu/.bin/fbidq.html?elk), [Hsp67Bc](http://flybase.bio.indiana.edu/.bin/fbidq.html?Hsp67Bc), [phl](http://flybase.bio.indiana.edu/.bin/fbidq.html?phl), [ase](http://flybase.bio.indiana.edu/.bin/fbidq.html?ase), [CG13253](http://flybase.bio.indiana.edu/.bin/fbidq.html?CG13253), [ovo](http://flybase.bio.indiana.edu/.bin/fbidq.html?ovo), [scro](http://flybase.bio.indiana.edu/.bin/fbidq.html?scro), [CG6197](http://flybase.bio.indiana.edu/.bin/fbidq.html?CG6197), [phol](http://flybase.bio.indiana.edu/.bin/fbidq.html?phol), [elav](http://flybase.bio.indiana.edu/.bin/fbidq.html?elav), [Dsp1](http://flybase.bio.indiana.edu/.bin/fbidq.html?Dsp1), [CG14216](http://flybase.bio.indiana.edu/.bin/fbidq.html?CG14216), [Pdp1](http://flybase.bio.indiana.edu/.bin/fbidq.html?Pdp1), [Oli](http://flybase.bio.indiana.edu/.bin/fbidq.html?Oli),[Nelf-A](http://flybase.bio.indiana.edu/.bin/fbidq.html?Nelf-A), [GATAd](http://flybase.bio.indiana.edu/.bin/fbidq.html?GATAd), [CG6227](http://flybase.bio.indiana.edu/.bin/fbidq.html?CG6227), [Poxn](http://flybase.bio.indiana.edu/.bin/fbidq.html?Poxn), [fbl6](http://flybase.bio.indiana.edu/.bin/fbidq.html?fbl6), [CG15376](http://flybase.bio.indiana.edu/.bin/fbidq.html?CG15376), [Sin3A](http://flybase.bio.indiana.edu/.bin/fbidq.html?Sin3A), [mip120](http://flybase.bio.indiana.edu/.bin/fbidq.html?mip120), [jumu](http://flybase.bio.indiana.edu/.bin/fbidq.html?jumu), [Smox](http://flybase.bio.indiana.edu/.bin/fbidq.html?Smox), [Su(var)2-HP2](http://flybase.bio.indiana.edu/.bin/fbidq.html?Su(var)2-HP2), [Usp7](http://flybase.bio.indiana.edu/.bin/fbidq.html?Usp7), [ush](http://flybase.bio.indiana.edu/.bin/fbidq.html?ush), [fd59A](http://flybase.bio.indiana.edu/.bin/fbidq.html?fd59A), [Gap1](http://flybase.bio.indiana.edu/.bin/fbidq.html?Gap1), [run](http://flybase.bio.indiana.edu/.bin/fbidq.html?run), [brat](http://flybase.bio.indiana.edu/.bin/fbidq.html?brat), [FBX011](http://flybase.bio.indiana.edu/.bin/fbidq.html?FBX011), [Antp](http://flybase.bio.indiana.edu/.bin/fbidq.html?Antp), [tara](http://flybase.bio.indiana.edu/.bin/fbidq.html?tara), [Alh](http://flybase.bio.indiana.edu/.bin/fbidq.html?Alh), [Sox102F](http://flybase.bio.indiana.edu/.bin/fbidq.html?Sox102F), [egg](http://flybase.bio.indiana.edu/.bin/fbidq.html?egg), [l(3)neo38](http://flybase.bio.indiana.edu/.bin/fbidq.html?l(3)neo38), [koko](http://flybase.bio.indiana.edu/.bin/fbidq.html?koko), [Ptth](http://flybase.bio.indiana.edu/.bin/fbidq.html?Ptth), [Sp1](http://flybase.bio.indiana.edu/.bin/fbidq.html?Sp1), [Ubx](http://flybase.bio.indiana.edu/.bin/fbidq.html?Ubx), [Ptx1](http://flybase.bio.indiana.edu/.bin/fbidq.html?Ptx1), [ph-p](http://flybase.bio.indiana.edu/.bin/fbidq.html?ph-p), [onecut](http://flybase.bio.indiana.edu/.bin/fbidq.html?onecut), [CG7757](http://flybase.bio.indiana.edu/.bin/fbidq.html?CG7757), [chm](http://flybase.bio.indiana.edu/.bin/fbidq.html?chm), [sna](http://flybase.bio.indiana.edu/.bin/fbidq.html?sna),[Wnt5](http://flybase.bio.indiana.edu/.bin/fbidq.html?Wnt5), [msl-1](http://flybase.bio.indiana.edu/.bin/fbidq.html?msl-1), [Taf1](http://flybase.bio.indiana.edu/.bin/fbidq.html?Taf1), [hbn](http://flybase.bio.indiana.edu/.bin/fbidq.html?hbn), [MBD-like](http://flybase.bio.indiana.edu/.bin/fbidq.html?MBD-like), [tsh](http://flybase.bio.indiana.edu/.bin/fbidq.html?tsh), [esg](http://flybase.bio.indiana.edu/.bin/fbidq.html?esg), [dac](http://flybase.bio.indiana.edu/.bin/fbidq.html?dac), [e(y)3](http://flybase.bio.indiana.edu/.bin/fbidq.html?e(y)3), [CG9007](http://flybase.bio.indiana.edu/.bin/fbidq.html?CG9007), [oc](http://flybase.bio.indiana.edu/.bin/fbidq.html?oc), [dom](http://flybase.bio.indiana.edu/.bin/fbidq.html?dom), [spt4](http://flybase.bio.indiana.edu/.bin/fbidq.html?spt4), [CG32532](http://flybase.bio.indiana.edu/.bin/fbidq.html?CG32532), [MTA1-like](http://flybase.bio.indiana.edu/.bin/fbidq.html?MTA1-like), [gol](http://flybase.bio.indiana.edu/.bin/fbidq.html?gol), [ey](http://flybase.bio.indiana.edu/.bin/fbidq.html?ey), [mle](http://flybase.bio.indiana.edu/.bin/fbidq.html?mle), [Hr51](http://flybase.bio.indiana.edu/.bin/fbidq.html?Hr51), [Bgb](http://flybase.bio.indiana.edu/.bin/fbidq.html?Bgb), [CG8786](http://flybase.bio.indiana.edu/.bin/fbidq.html?CG8786), [l(1)sc](http://flybase.bio.indiana.edu/.bin/fbidq.html?l(1)sc), [B-H2](http://flybase.bio.indiana.edu/.bin/fbidq.html?B-H2), [grh](http://flybase.bio.indiana.edu/.bin/fbidq.html?grh), [Vsx1](http://flybase.bio.indiana.edu/.bin/fbidq.html?Vsx1), [wg](http://flybase.bio.indiana.edu/.bin/fbidq.html?wg), [toy](http://flybase.bio.indiana.edu/.bin/fbidq.html?toy), [fkh](http://flybase.bio.indiana.edu/.bin/fbidq.html?fkh), [Lim3](http://flybase.bio.indiana.edu/.bin/fbidq.html?Lim3), [CG34362](http://flybase.bio.indiana.edu/.bin/fbidq.html?CG34362), [crol](http://flybase.bio.indiana.edu/.bin/fbidq.html?crol), [sd](http://flybase.bio.indiana.edu/.bin/fbidq.html?sd), [Spps](http://flybase.bio.indiana.edu/.bin/fbidq.html?Spps), [retn](http://flybase.bio.indiana.edu/.bin/fbidq.html?retn), [en](http://flybase.bio.indiana.edu/.bin/fbidq.html?en), [fs(1)h](http://flybase.bio.indiana.edu/.bin/fbidq.html?fs(1)h), [rl](http://flybase.bio.indiana.edu/.bin/fbidq.html?rl), [CG11347](http://flybase.bio.indiana.edu/.bin/fbidq.html?CG11347), [tey](http://flybase.bio.indiana.edu/.bin/fbidq.html?tey),[Saf-B](http://flybase.bio.indiana.edu/.bin/fbidq.html?Saf-B), [CG11294](http://flybase.bio.indiana.edu/.bin/fbidq.html?CG11294), [hiw](http://flybase.bio.indiana.edu/.bin/fbidq.html?hiw), [cos](http://flybase.bio.indiana.edu/.bin/fbidq.html?cos), [Ct](http://flybase.bio.indiana.edu/.bin/fbidq.html?Ct), [Atf-2](http://flybase.bio.indiana.edu/.bin/fbidq.html?Atf-2) |
| [taxis](http://amigo.geneontology.org/cgi-bin/amigo/go.cgi?view=details&query=GO:0042330) | 89 of 1594 genes, 5.6% | 231 of 7634 genes, 3.0% | 4.75e-08 | 0.00% | 0.00 | [fz2](http://flybase.bio.indiana.edu/.bin/fbidq.html?fz2), [acj6](http://flybase.bio.indiana.edu/.bin/fbidq.html?acj6), [vvl](http://flybase.bio.indiana.edu/.bin/fbidq.html?vvl), [jing](http://flybase.bio.indiana.edu/.bin/fbidq.html?jing), [dock](http://flybase.bio.indiana.edu/.bin/fbidq.html?dock), [Ptp69D](http://flybase.bio.indiana.edu/.bin/fbidq.html?Ptp69D), [hts](http://flybase.bio.indiana.edu/.bin/fbidq.html?hts), [Nmdar1](http://flybase.bio.indiana.edu/.bin/fbidq.html?Nmdar1), [mp](http://flybase.bio.indiana.edu/.bin/fbidq.html?mp), [ap](http://flybase.bio.indiana.edu/.bin/fbidq.html?ap), [Dg](http://flybase.bio.indiana.edu/.bin/fbidq.html?Dg), [seq](http://flybase.bio.indiana.edu/.bin/fbidq.html?seq), [gcm](http://flybase.bio.indiana.edu/.bin/fbidq.html?gcm), [Trim9](http://flybase.bio.indiana.edu/.bin/fbidq.html?Trim9), [Gycalpha99B](http://flybase.bio.indiana.edu/.bin/fbidq.html?Gycalpha99B), [robo3](http://flybase.bio.indiana.edu/.bin/fbidq.html?robo3), [tok](http://flybase.bio.indiana.edu/.bin/fbidq.html?tok), [nonA](http://flybase.bio.indiana.edu/.bin/fbidq.html?nonA), [Ptp99A](http://flybase.bio.indiana.edu/.bin/fbidq.html?Ptp99A), [lola](http://flybase.bio.indiana.edu/.bin/fbidq.html?lola), [unc-104](http://flybase.bio.indiana.edu/.bin/fbidq.html?unc-104), [chif](http://flybase.bio.indiana.edu/.bin/fbidq.html?chif), [NetB](http://flybase.bio.indiana.edu/.bin/fbidq.html?NetB), [Alk](http://flybase.bio.indiana.edu/.bin/fbidq.html?Alk), [rdgC](http://flybase.bio.indiana.edu/.bin/fbidq.html?rdgC), [Nrk](http://flybase.bio.indiana.edu/.bin/fbidq.html?Nrk), [Galpha49B](http://flybase.bio.indiana.edu/.bin/fbidq.html?Galpha49B), [Smox](http://flybase.bio.indiana.edu/.bin/fbidq.html?Smox), [Lar](http://flybase.bio.indiana.edu/.bin/fbidq.html?Lar), [not](http://flybase.bio.indiana.edu/.bin/fbidq.html?not), [nvy](http://flybase.bio.indiana.edu/.bin/fbidq.html?nvy), [gogo](http://flybase.bio.indiana.edu/.bin/fbidq.html?gogo), [fd59A](http://flybase.bio.indiana.edu/.bin/fbidq.html?fd59A), [beat-Ic](http://flybase.bio.indiana.edu/.bin/fbidq.html?beat-Ic), [Ddc](http://flybase.bio.indiana.edu/.bin/fbidq.html?Ddc), [run](http://flybase.bio.indiana.edu/.bin/fbidq.html?run), [jeb](http://flybase.bio.indiana.edu/.bin/fbidq.html?jeb), [brat](http://flybase.bio.indiana.edu/.bin/fbidq.html?brat), [CG16778](http://flybase.bio.indiana.edu/.bin/fbidq.html?CG16778),[ko](http://flybase.bio.indiana.edu/.bin/fbidq.html?ko), [Sema-1a](http://flybase.bio.indiana.edu/.bin/fbidq.html?Sema-1a), [beat-Ib](http://flybase.bio.indiana.edu/.bin/fbidq.html?beat-Ib), [otk](http://flybase.bio.indiana.edu/.bin/fbidq.html?otk), [stan](http://flybase.bio.indiana.edu/.bin/fbidq.html?stan), [CG12424](http://flybase.bio.indiana.edu/.bin/fbidq.html?CG12424), [CG34400](http://flybase.bio.indiana.edu/.bin/fbidq.html?CG34400), [tutl](http://flybase.bio.indiana.edu/.bin/fbidq.html?tutl), [ena](http://flybase.bio.indiana.edu/.bin/fbidq.html?ena), [dsh](http://flybase.bio.indiana.edu/.bin/fbidq.html?dsh), [Nf-YC](http://flybase.bio.indiana.edu/.bin/fbidq.html?Nf-YC), [robo](http://flybase.bio.indiana.edu/.bin/fbidq.html?robo), [bsk](http://flybase.bio.indiana.edu/.bin/fbidq.html?bsk), [lea](http://flybase.bio.indiana.edu/.bin/fbidq.html?lea), [beat-Ia](http://flybase.bio.indiana.edu/.bin/fbidq.html?beat-Ia), [gukh](http://flybase.bio.indiana.edu/.bin/fbidq.html?gukh), [plexB](http://flybase.bio.indiana.edu/.bin/fbidq.html?plexB), [Wnt5](http://flybase.bio.indiana.edu/.bin/fbidq.html?Wnt5), [Klp64D](http://flybase.bio.indiana.edu/.bin/fbidq.html?Klp64D), [beat-IIa](http://flybase.bio.indiana.edu/.bin/fbidq.html?beat-IIa), [grn](http://flybase.bio.indiana.edu/.bin/fbidq.html?grn), [sm](http://flybase.bio.indiana.edu/.bin/fbidq.html?sm), [DopR](http://flybase.bio.indiana.edu/.bin/fbidq.html?DopR), [mew](http://flybase.bio.indiana.edu/.bin/fbidq.html?mew), [Lis-1](http://flybase.bio.indiana.edu/.bin/fbidq.html?Lis-1), [dac](http://flybase.bio.indiana.edu/.bin/fbidq.html?dac), [Cdk5](http://flybase.bio.indiana.edu/.bin/fbidq.html?Cdk5), [nmo](http://flybase.bio.indiana.edu/.bin/fbidq.html?nmo), [pros](http://flybase.bio.indiana.edu/.bin/fbidq.html?pros), [tup](http://flybase.bio.indiana.edu/.bin/fbidq.html?tup), [trio](http://flybase.bio.indiana.edu/.bin/fbidq.html?trio), [daw](http://flybase.bio.indiana.edu/.bin/fbidq.html?daw), [lap](http://flybase.bio.indiana.edu/.bin/fbidq.html?lap), [Wnt4](http://flybase.bio.indiana.edu/.bin/fbidq.html?Wnt4), [comm](http://flybase.bio.indiana.edu/.bin/fbidq.html?comm), [Cdk5alpha](http://flybase.bio.indiana.edu/.bin/fbidq.html?Cdk5alpha), [Apc](http://flybase.bio.indiana.edu/.bin/fbidq.html?Apc),[sim](http://flybase.bio.indiana.edu/.bin/fbidq.html?sim), [Lim3](http://flybase.bio.indiana.edu/.bin/fbidq.html?Lim3), [CadN](http://flybase.bio.indiana.edu/.bin/fbidq.html?CadN), [nerfin-1](http://flybase.bio.indiana.edu/.bin/fbidq.html?nerfin-1), [trx](http://flybase.bio.indiana.edu/.bin/fbidq.html?trx), [retn](http://flybase.bio.indiana.edu/.bin/fbidq.html?retn), [en](http://flybase.bio.indiana.edu/.bin/fbidq.html?en), [dsx](http://flybase.bio.indiana.edu/.bin/fbidq.html?dsx), [unc-5](http://flybase.bio.indiana.edu/.bin/fbidq.html?unc-5), [trn](http://flybase.bio.indiana.edu/.bin/fbidq.html?trn), [CG10107](http://flybase.bio.indiana.edu/.bin/fbidq.html?CG10107), [fend](http://flybase.bio.indiana.edu/.bin/fbidq.html?fend), [CG33960](http://flybase.bio.indiana.edu/.bin/fbidq.html?CG33960) |
| [cell development](http://amigo.geneontology.org/cgi-bin/amigo/go.cgi?view=details&query=GO:0048468) | 264 of 1594 genes, 16.6% | 937 of 7634 genes, 12.3% | 7.21e-08 | 0.00% | 0.00 | [fz2](http://flybase.bio.indiana.edu/.bin/fbidq.html?fz2), [Sh](http://flybase.bio.indiana.edu/.bin/fbidq.html?Sh), [numb](http://flybase.bio.indiana.edu/.bin/fbidq.html?numb), [dock](http://flybase.bio.indiana.edu/.bin/fbidq.html?dock), [CG42256](http://flybase.bio.indiana.edu/.bin/fbidq.html?CG42256), [CG3703](http://flybase.bio.indiana.edu/.bin/fbidq.html?CG3703), [stj](http://flybase.bio.indiana.edu/.bin/fbidq.html?stj), [Sxl](http://flybase.bio.indiana.edu/.bin/fbidq.html?Sxl), [ap](http://flybase.bio.indiana.edu/.bin/fbidq.html?ap), [Nf1](http://flybase.bio.indiana.edu/.bin/fbidq.html?Nf1), [sns](http://flybase.bio.indiana.edu/.bin/fbidq.html?sns), [os](http://flybase.bio.indiana.edu/.bin/fbidq.html?os), [Axn](http://flybase.bio.indiana.edu/.bin/fbidq.html?Axn), [tok](http://flybase.bio.indiana.edu/.bin/fbidq.html?tok), [Ptp99A](http://flybase.bio.indiana.edu/.bin/fbidq.html?Ptp99A), [Traf6](http://flybase.bio.indiana.edu/.bin/fbidq.html?Traf6), [NetB](http://flybase.bio.indiana.edu/.bin/fbidq.html?NetB), [Tango10](http://flybase.bio.indiana.edu/.bin/fbidq.html?Tango10), [CG32137](http://flybase.bio.indiana.edu/.bin/fbidq.html?CG32137), [blue](http://flybase.bio.indiana.edu/.bin/fbidq.html?blue), [Galpha49B](http://flybase.bio.indiana.edu/.bin/fbidq.html?Galpha49B), [Snoo](http://flybase.bio.indiana.edu/.bin/fbidq.html?Snoo), [Lar](http://flybase.bio.indiana.edu/.bin/fbidq.html?Lar), [not](http://flybase.bio.indiana.edu/.bin/fbidq.html?not), [rut](http://flybase.bio.indiana.edu/.bin/fbidq.html?rut), [nvy](http://flybase.bio.indiana.edu/.bin/fbidq.html?nvy), [nab](http://flybase.bio.indiana.edu/.bin/fbidq.html?nab), [pim](http://flybase.bio.indiana.edu/.bin/fbidq.html?pim), [alph](http://flybase.bio.indiana.edu/.bin/fbidq.html?alph), [Traf4](http://flybase.bio.indiana.edu/.bin/fbidq.html?Traf4), [TBPH](http://flybase.bio.indiana.edu/.bin/fbidq.html?TBPH), [CG34400](http://flybase.bio.indiana.edu/.bin/fbidq.html?CG34400), [Ulp1](http://flybase.bio.indiana.edu/.bin/fbidq.html?Ulp1), [mGluRA](http://flybase.bio.indiana.edu/.bin/fbidq.html?mGluRA), [ena](http://flybase.bio.indiana.edu/.bin/fbidq.html?ena), [dsh](http://flybase.bio.indiana.edu/.bin/fbidq.html?dsh), [hth](http://flybase.bio.indiana.edu/.bin/fbidq.html?hth), [Nf-YC](http://flybase.bio.indiana.edu/.bin/fbidq.html?Nf-YC), [14-3-3zeta](http://flybase.bio.indiana.edu/.bin/fbidq.html?14-3-3zeta), [lea](http://flybase.bio.indiana.edu/.bin/fbidq.html?lea), [plexB](http://flybase.bio.indiana.edu/.bin/fbidq.html?plexB), [PP2A-B](http://flybase.bio.indiana.edu/.bin/fbidq.html?PP2A-B), [loqs](http://flybase.bio.indiana.edu/.bin/fbidq.html?loqs), [Klp64D](http://flybase.bio.indiana.edu/.bin/fbidq.html?Klp64D), [DAAM](http://flybase.bio.indiana.edu/.bin/fbidq.html?DAAM), [neur](http://flybase.bio.indiana.edu/.bin/fbidq.html?neur), [CycE](http://flybase.bio.indiana.edu/.bin/fbidq.html?CycE), [Brf](http://flybase.bio.indiana.edu/.bin/fbidq.html?Brf), [nmo](http://flybase.bio.indiana.edu/.bin/fbidq.html?nmo), [ed](http://flybase.bio.indiana.edu/.bin/fbidq.html?ed), [Actn](http://flybase.bio.indiana.edu/.bin/fbidq.html?Actn), [mr](http://flybase.bio.indiana.edu/.bin/fbidq.html?mr), [daw](http://flybase.bio.indiana.edu/.bin/fbidq.html?daw), [Apc](http://flybase.bio.indiana.edu/.bin/fbidq.html?Apc), [sim](http://flybase.bio.indiana.edu/.bin/fbidq.html?sim), [d4](http://flybase.bio.indiana.edu/.bin/fbidq.html?d4), [cort](http://flybase.bio.indiana.edu/.bin/fbidq.html?cort), [gro](http://flybase.bio.indiana.edu/.bin/fbidq.html?gro), [RN-tre](http://flybase.bio.indiana.edu/.bin/fbidq.html?RN-tre), [Mf](http://flybase.bio.indiana.edu/.bin/fbidq.html?Mf), [wnd](http://flybase.bio.indiana.edu/.bin/fbidq.html?wnd), [trx](http://flybase.bio.indiana.edu/.bin/fbidq.html?trx), [Rpd3](http://flybase.bio.indiana.edu/.bin/fbidq.html?Rpd3), [unc-5](http://flybase.bio.indiana.edu/.bin/fbidq.html?unc-5), [cdi](http://flybase.bio.indiana.edu/.bin/fbidq.html?cdi), [CG10107](http://flybase.bio.indiana.edu/.bin/fbidq.html?CG10107), [aret](http://flybase.bio.indiana.edu/.bin/fbidq.html?aret), [pygo](http://flybase.bio.indiana.edu/.bin/fbidq.html?pygo), [HLHm7](http://flybase.bio.indiana.edu/.bin/fbidq.html?HLHm7), [HLHmgamma](http://flybase.bio.indiana.edu/.bin/fbidq.html?HLHmgamma), [jar](http://flybase.bio.indiana.edu/.bin/fbidq.html?jar), [wor](http://flybase.bio.indiana.edu/.bin/fbidq.html?wor), [Atg1](http://flybase.bio.indiana.edu/.bin/fbidq.html?Atg1), [Pten](http://flybase.bio.indiana.edu/.bin/fbidq.html?Pten), [mira](http://flybase.bio.indiana.edu/.bin/fbidq.html?mira), [Ptp69D](http://flybase.bio.indiana.edu/.bin/fbidq.html?Ptp69D), [hts](http://flybase.bio.indiana.edu/.bin/fbidq.html?hts),[pUf68](http://flybase.bio.indiana.edu/.bin/fbidq.html?pUf68), [seq](http://flybase.bio.indiana.edu/.bin/fbidq.html?seq), [Dip3](http://flybase.bio.indiana.edu/.bin/fbidq.html?Dip3), [oa2](http://flybase.bio.indiana.edu/.bin/fbidq.html?oa2), [phl](http://flybase.bio.indiana.edu/.bin/fbidq.html?phl), [Cbl](http://flybase.bio.indiana.edu/.bin/fbidq.html?Cbl), [CG4893](http://flybase.bio.indiana.edu/.bin/fbidq.html?CG4893), [CG9098](http://flybase.bio.indiana.edu/.bin/fbidq.html?CG9098), [Alk](http://flybase.bio.indiana.edu/.bin/fbidq.html?Alk), [esn](http://flybase.bio.indiana.edu/.bin/fbidq.html?esn), [Vang](http://flybase.bio.indiana.edu/.bin/fbidq.html?Vang), [Sin3A](http://flybase.bio.indiana.edu/.bin/fbidq.html?Sin3A), [pot](http://flybase.bio.indiana.edu/.bin/fbidq.html?pot), [mip120](http://flybase.bio.indiana.edu/.bin/fbidq.html?mip120), [cpx](http://flybase.bio.indiana.edu/.bin/fbidq.html?cpx), [jumu](http://flybase.bio.indiana.edu/.bin/fbidq.html?jumu), [Smox](http://flybase.bio.indiana.edu/.bin/fbidq.html?Smox), [CG31475](http://flybase.bio.indiana.edu/.bin/fbidq.html?CG31475), [fd59A](http://flybase.bio.indiana.edu/.bin/fbidq.html?fd59A), [beat-Ic](http://flybase.bio.indiana.edu/.bin/fbidq.html?beat-Ic), [brat](http://flybase.bio.indiana.edu/.bin/fbidq.html?brat), [ST6Gal](http://flybase.bio.indiana.edu/.bin/fbidq.html?ST6Gal), [Antp](http://flybase.bio.indiana.edu/.bin/fbidq.html?Antp), [cindr](http://flybase.bio.indiana.edu/.bin/fbidq.html?cindr), [futsch](http://flybase.bio.indiana.edu/.bin/fbidq.html?futsch), [Ptx1](http://flybase.bio.indiana.edu/.bin/fbidq.html?Ptx1), [gukh](http://flybase.bio.indiana.edu/.bin/fbidq.html?gukh), [chm](http://flybase.bio.indiana.edu/.bin/fbidq.html?chm), [CG6114](http://flybase.bio.indiana.edu/.bin/fbidq.html?CG6114), [Rim](http://flybase.bio.indiana.edu/.bin/fbidq.html?Rim), [beat-IIa](http://flybase.bio.indiana.edu/.bin/fbidq.html?beat-IIa), [sm](http://flybase.bio.indiana.edu/.bin/fbidq.html?sm), [Syn2](http://flybase.bio.indiana.edu/.bin/fbidq.html?Syn2), [dac](http://flybase.bio.indiana.edu/.bin/fbidq.html?dac), [Cdk5](http://flybase.bio.indiana.edu/.bin/fbidq.html?Cdk5), [Cep135](http://flybase.bio.indiana.edu/.bin/fbidq.html?Cep135), [oc](http://flybase.bio.indiana.edu/.bin/fbidq.html?oc),[CG4641](http://flybase.bio.indiana.edu/.bin/fbidq.html?CG4641), [baz](http://flybase.bio.indiana.edu/.bin/fbidq.html?baz), [rst](http://flybase.bio.indiana.edu/.bin/fbidq.html?rst), [comm](http://flybase.bio.indiana.edu/.bin/fbidq.html?comm), [grh](http://flybase.bio.indiana.edu/.bin/fbidq.html?grh), [Cdk5alpha](http://flybase.bio.indiana.edu/.bin/fbidq.html?Cdk5alpha), [Lim3](http://flybase.bio.indiana.edu/.bin/fbidq.html?Lim3), [sd](http://flybase.bio.indiana.edu/.bin/fbidq.html?sd), [Aplip1](http://flybase.bio.indiana.edu/.bin/fbidq.html?Aplip1), [E(Pc)](http://flybase.bio.indiana.edu/.bin/fbidq.html?E(Pc)), [retn](http://flybase.bio.indiana.edu/.bin/fbidq.html?retn), [rl](http://flybase.bio.indiana.edu/.bin/fbidq.html?rl), [Syt1](http://flybase.bio.indiana.edu/.bin/fbidq.html?Syt1), [tey](http://flybase.bio.indiana.edu/.bin/fbidq.html?tey), [yrt](http://flybase.bio.indiana.edu/.bin/fbidq.html?yrt), [CG33960](http://flybase.bio.indiana.edu/.bin/fbidq.html?CG33960), [hiw](http://flybase.bio.indiana.edu/.bin/fbidq.html?hiw), [acj6](http://flybase.bio.indiana.edu/.bin/fbidq.html?acj6), [jing](http://flybase.bio.indiana.edu/.bin/fbidq.html?jing), [ato](http://flybase.bio.indiana.edu/.bin/fbidq.html?ato), [vimar](http://flybase.bio.indiana.edu/.bin/fbidq.html?vimar), [Iswi](http://flybase.bio.indiana.edu/.bin/fbidq.html?Iswi), [Dg](http://flybase.bio.indiana.edu/.bin/fbidq.html?Dg), [Trim9](http://flybase.bio.indiana.edu/.bin/fbidq.html?Trim9), [Pka-R1](http://flybase.bio.indiana.edu/.bin/fbidq.html?Pka-R1), [mago](http://flybase.bio.indiana.edu/.bin/fbidq.html?mago), [Evi5](http://flybase.bio.indiana.edu/.bin/fbidq.html?Evi5), [enc](http://flybase.bio.indiana.edu/.bin/fbidq.html?enc), [CG7154](http://flybase.bio.indiana.edu/.bin/fbidq.html?CG7154), [br](http://flybase.bio.indiana.edu/.bin/fbidq.html?br), [robo3](http://flybase.bio.indiana.edu/.bin/fbidq.html?robo3), [cuff](http://flybase.bio.indiana.edu/.bin/fbidq.html?cuff), [neo](http://flybase.bio.indiana.edu/.bin/fbidq.html?neo), [lola](http://flybase.bio.indiana.edu/.bin/fbidq.html?lola), [unc-104](http://flybase.bio.indiana.edu/.bin/fbidq.html?unc-104), [chif](http://flybase.bio.indiana.edu/.bin/fbidq.html?chif), [p130CAS](http://flybase.bio.indiana.edu/.bin/fbidq.html?p130CAS), [Appl](http://flybase.bio.indiana.edu/.bin/fbidq.html?Appl), [ewg](http://flybase.bio.indiana.edu/.bin/fbidq.html?ewg),[metro](http://flybase.bio.indiana.edu/.bin/fbidq.html?metro), [Nrk](http://flybase.bio.indiana.edu/.bin/fbidq.html?Nrk), [erm](http://flybase.bio.indiana.edu/.bin/fbidq.html?erm), [CG4328](http://flybase.bio.indiana.edu/.bin/fbidq.html?CG4328), [CG10249](http://flybase.bio.indiana.edu/.bin/fbidq.html?CG10249), [Taf4](http://flybase.bio.indiana.edu/.bin/fbidq.html?Taf4), [gogo](http://flybase.bio.indiana.edu/.bin/fbidq.html?gogo), [Nrg](http://flybase.bio.indiana.edu/.bin/fbidq.html?Nrg), [jeb](http://flybase.bio.indiana.edu/.bin/fbidq.html?jeb), [GluClalpha](http://flybase.bio.indiana.edu/.bin/fbidq.html?GluClalpha), [Actbeta](http://flybase.bio.indiana.edu/.bin/fbidq.html?Actbeta), [beat-Ib](http://flybase.bio.indiana.edu/.bin/fbidq.html?beat-Ib), [Sema-1a](http://flybase.bio.indiana.edu/.bin/fbidq.html?Sema-1a), [stau](http://flybase.bio.indiana.edu/.bin/fbidq.html?stau), [Hs3st-B](http://flybase.bio.indiana.edu/.bin/fbidq.html?Hs3st-B), [otk](http://flybase.bio.indiana.edu/.bin/fbidq.html?otk), [phyl](http://flybase.bio.indiana.edu/.bin/fbidq.html?phyl), [eya](http://flybase.bio.indiana.edu/.bin/fbidq.html?eya), [Fas2](http://flybase.bio.indiana.edu/.bin/fbidq.html?Fas2), [bsk](http://flybase.bio.indiana.edu/.bin/fbidq.html?bsk), [brm](http://flybase.bio.indiana.edu/.bin/fbidq.html?brm), [robo](http://flybase.bio.indiana.edu/.bin/fbidq.html?robo), [fz](http://flybase.bio.indiana.edu/.bin/fbidq.html?fz), [beat-Ia](http://flybase.bio.indiana.edu/.bin/fbidq.html?beat-Ia), [ft](http://flybase.bio.indiana.edu/.bin/fbidq.html?ft), [kat-60L1](http://flybase.bio.indiana.edu/.bin/fbidq.html?kat-60L1), [CG12004](http://flybase.bio.indiana.edu/.bin/fbidq.html?CG12004), [RhoGAPp190](http://flybase.bio.indiana.edu/.bin/fbidq.html?RhoGAPp190), [hig](http://flybase.bio.indiana.edu/.bin/fbidq.html?hig), [mam](http://flybase.bio.indiana.edu/.bin/fbidq.html?mam), [grn](http://flybase.bio.indiana.edu/.bin/fbidq.html?grn), [mew](http://flybase.bio.indiana.edu/.bin/fbidq.html?mew), [Lis-1](http://flybase.bio.indiana.edu/.bin/fbidq.html?Lis-1), [didum](http://flybase.bio.indiana.edu/.bin/fbidq.html?didum), [ems](http://flybase.bio.indiana.edu/.bin/fbidq.html?ems),[eys](http://flybase.bio.indiana.edu/.bin/fbidq.html?eys), [tup](http://flybase.bio.indiana.edu/.bin/fbidq.html?tup), [pros](http://flybase.bio.indiana.edu/.bin/fbidq.html?pros), [Mob2](http://flybase.bio.indiana.edu/.bin/fbidq.html?Mob2), [pdm2](http://flybase.bio.indiana.edu/.bin/fbidq.html?pdm2), [trio](http://flybase.bio.indiana.edu/.bin/fbidq.html?trio), [CG1463](http://flybase.bio.indiana.edu/.bin/fbidq.html?CG1463), [Gad1](http://flybase.bio.indiana.edu/.bin/fbidq.html?Gad1), [Wnt4](http://flybase.bio.indiana.edu/.bin/fbidq.html?Wnt4), [rictor](http://flybase.bio.indiana.edu/.bin/fbidq.html?rictor), [CaMKII](http://flybase.bio.indiana.edu/.bin/fbidq.html?CaMKII), [pigs](http://flybase.bio.indiana.edu/.bin/fbidq.html?pigs), [nerfin-1](http://flybase.bio.indiana.edu/.bin/fbidq.html?nerfin-1), [bchs](http://flybase.bio.indiana.edu/.bin/fbidq.html?bchs), [polo](http://flybase.bio.indiana.edu/.bin/fbidq.html?polo), [dsx](http://flybase.bio.indiana.edu/.bin/fbidq.html?dsx), [chinmo](http://flybase.bio.indiana.edu/.bin/fbidq.html?chinmo), [Syn1](http://flybase.bio.indiana.edu/.bin/fbidq.html?Syn1), [Ssdp](http://flybase.bio.indiana.edu/.bin/fbidq.html?Ssdp), [sqz](http://flybase.bio.indiana.edu/.bin/fbidq.html?sqz), [klu](http://flybase.bio.indiana.edu/.bin/fbidq.html?klu), [CG8155](http://flybase.bio.indiana.edu/.bin/fbidq.html?CG8155), [fend](http://flybase.bio.indiana.edu/.bin/fbidq.html?fend), [Cad74A](http://flybase.bio.indiana.edu/.bin/fbidq.html?Cad74A), [tld](http://flybase.bio.indiana.edu/.bin/fbidq.html?tld), [Dsk](http://flybase.bio.indiana.edu/.bin/fbidq.html?Dsk), [vvl](http://flybase.bio.indiana.edu/.bin/fbidq.html?vvl), [rg](http://flybase.bio.indiana.edu/.bin/fbidq.html?rg), [betaInt-nu](http://flybase.bio.indiana.edu/.bin/fbidq.html?betaInt-nu), [sgg](http://flybase.bio.indiana.edu/.bin/fbidq.html?sgg), [gcm2](http://flybase.bio.indiana.edu/.bin/fbidq.html?gcm2), [mp](http://flybase.bio.indiana.edu/.bin/fbidq.html?mp), [gcm](http://flybase.bio.indiana.edu/.bin/fbidq.html?gcm), [CG5890](http://flybase.bio.indiana.edu/.bin/fbidq.html?CG5890), [can](http://flybase.bio.indiana.edu/.bin/fbidq.html?can), [SP2353](http://flybase.bio.indiana.edu/.bin/fbidq.html?SP2353), [CG13253](http://flybase.bio.indiana.edu/.bin/fbidq.html?CG13253),[CG32685](http://flybase.bio.indiana.edu/.bin/fbidq.html?CG32685), [ds](http://flybase.bio.indiana.edu/.bin/fbidq.html?ds), [mei-P26](http://flybase.bio.indiana.edu/.bin/fbidq.html?mei-P26), [sals](http://flybase.bio.indiana.edu/.bin/fbidq.html?sals), [Rbp9](http://flybase.bio.indiana.edu/.bin/fbidq.html?Rbp9), [Fak56D](http://flybase.bio.indiana.edu/.bin/fbidq.html?Fak56D), [Gap1](http://flybase.bio.indiana.edu/.bin/fbidq.html?Gap1), [run](http://flybase.bio.indiana.edu/.bin/fbidq.html?run), [ko](http://flybase.bio.indiana.edu/.bin/fbidq.html?ko), [Eip71CD](http://flybase.bio.indiana.edu/.bin/fbidq.html?Eip71CD), [egg](http://flybase.bio.indiana.edu/.bin/fbidq.html?egg), [stan](http://flybase.bio.indiana.edu/.bin/fbidq.html?stan), [koko](http://flybase.bio.indiana.edu/.bin/fbidq.html?koko), [CG12424](http://flybase.bio.indiana.edu/.bin/fbidq.html?CG12424), [tutl](http://flybase.bio.indiana.edu/.bin/fbidq.html?tutl), [ph-p](http://flybase.bio.indiana.edu/.bin/fbidq.html?ph-p), [Frq1](http://flybase.bio.indiana.edu/.bin/fbidq.html?Frq1), [sna](http://flybase.bio.indiana.edu/.bin/fbidq.html?sna), [Wnt5](http://flybase.bio.indiana.edu/.bin/fbidq.html?Wnt5), [esg](http://flybase.bio.indiana.edu/.bin/fbidq.html?esg), [sif](http://flybase.bio.indiana.edu/.bin/fbidq.html?sif), [dom](http://flybase.bio.indiana.edu/.bin/fbidq.html?dom), [ssh](http://flybase.bio.indiana.edu/.bin/fbidq.html?ssh), [klar](http://flybase.bio.indiana.edu/.bin/fbidq.html?klar), [PIP5K59B](http://flybase.bio.indiana.edu/.bin/fbidq.html?PIP5K59B), [mle](http://flybase.bio.indiana.edu/.bin/fbidq.html?mle), [Hr51](http://flybase.bio.indiana.edu/.bin/fbidq.html?Hr51), [CG11155](http://flybase.bio.indiana.edu/.bin/fbidq.html?CG11155), [shi](http://flybase.bio.indiana.edu/.bin/fbidq.html?shi), [tyn](http://flybase.bio.indiana.edu/.bin/fbidq.html?tyn), [wg](http://flybase.bio.indiana.edu/.bin/fbidq.html?wg), [CadN](http://flybase.bio.indiana.edu/.bin/fbidq.html?CadN), [E(bx)](http://flybase.bio.indiana.edu/.bin/fbidq.html?E(bx)), [en](http://flybase.bio.indiana.edu/.bin/fbidq.html?en), [Snap](http://flybase.bio.indiana.edu/.bin/fbidq.html?Snap), [trn](http://flybase.bio.indiana.edu/.bin/fbidq.html?trn), [bt](http://flybase.bio.indiana.edu/.bin/fbidq.html?bt), [cos](http://flybase.bio.indiana.edu/.bin/fbidq.html?cos), [Ct](http://flybase.bio.indiana.edu/.bin/fbidq.html?Ct) |
| [chemotaxis](http://amigo.geneontology.org/cgi-bin/amigo/go.cgi?view=details&query=GO:0006935) | 80 of 1594 genes, 5.0% | 203 of 7634 genes, 2.7% | 1.36e-07 | 0.00% | 0.00 | [fz2](http://flybase.bio.indiana.edu/.bin/fbidq.html?fz2), [acj6](http://flybase.bio.indiana.edu/.bin/fbidq.html?acj6), [vvl](http://flybase.bio.indiana.edu/.bin/fbidq.html?vvl), [jing](http://flybase.bio.indiana.edu/.bin/fbidq.html?jing), [dock](http://flybase.bio.indiana.edu/.bin/fbidq.html?dock), [Ptp69D](http://flybase.bio.indiana.edu/.bin/fbidq.html?Ptp69D), [hts](http://flybase.bio.indiana.edu/.bin/fbidq.html?hts), [mp](http://flybase.bio.indiana.edu/.bin/fbidq.html?mp), [ap](http://flybase.bio.indiana.edu/.bin/fbidq.html?ap), [Dg](http://flybase.bio.indiana.edu/.bin/fbidq.html?Dg), [seq](http://flybase.bio.indiana.edu/.bin/fbidq.html?seq), [gcm](http://flybase.bio.indiana.edu/.bin/fbidq.html?gcm), [Trim9](http://flybase.bio.indiana.edu/.bin/fbidq.html?Trim9), [robo3](http://flybase.bio.indiana.edu/.bin/fbidq.html?robo3), [tok](http://flybase.bio.indiana.edu/.bin/fbidq.html?tok), [Ptp99A](http://flybase.bio.indiana.edu/.bin/fbidq.html?Ptp99A), [lola](http://flybase.bio.indiana.edu/.bin/fbidq.html?lola), [unc-104](http://flybase.bio.indiana.edu/.bin/fbidq.html?unc-104), [chif](http://flybase.bio.indiana.edu/.bin/fbidq.html?chif), [NetB](http://flybase.bio.indiana.edu/.bin/fbidq.html?NetB), [Alk](http://flybase.bio.indiana.edu/.bin/fbidq.html?Alk), [Nrk](http://flybase.bio.indiana.edu/.bin/fbidq.html?Nrk), [Galpha49B](http://flybase.bio.indiana.edu/.bin/fbidq.html?Galpha49B), [Smox](http://flybase.bio.indiana.edu/.bin/fbidq.html?Smox), [Lar](http://flybase.bio.indiana.edu/.bin/fbidq.html?Lar), [not](http://flybase.bio.indiana.edu/.bin/fbidq.html?not), [nvy](http://flybase.bio.indiana.edu/.bin/fbidq.html?nvy), [gogo](http://flybase.bio.indiana.edu/.bin/fbidq.html?gogo), [fd59A](http://flybase.bio.indiana.edu/.bin/fbidq.html?fd59A), [beat-Ic](http://flybase.bio.indiana.edu/.bin/fbidq.html?beat-Ic), [run](http://flybase.bio.indiana.edu/.bin/fbidq.html?run), [jeb](http://flybase.bio.indiana.edu/.bin/fbidq.html?jeb), [brat](http://flybase.bio.indiana.edu/.bin/fbidq.html?brat), [ko](http://flybase.bio.indiana.edu/.bin/fbidq.html?ko), [Sema-1a](http://flybase.bio.indiana.edu/.bin/fbidq.html?Sema-1a), [beat-Ib](http://flybase.bio.indiana.edu/.bin/fbidq.html?beat-Ib), [otk](http://flybase.bio.indiana.edu/.bin/fbidq.html?otk), [stan](http://flybase.bio.indiana.edu/.bin/fbidq.html?stan), [CG12424](http://flybase.bio.indiana.edu/.bin/fbidq.html?CG12424),[CG34400](http://flybase.bio.indiana.edu/.bin/fbidq.html?CG34400), [tutl](http://flybase.bio.indiana.edu/.bin/fbidq.html?tutl), [ena](http://flybase.bio.indiana.edu/.bin/fbidq.html?ena), [dsh](http://flybase.bio.indiana.edu/.bin/fbidq.html?dsh), [Nf-YC](http://flybase.bio.indiana.edu/.bin/fbidq.html?Nf-YC), [robo](http://flybase.bio.indiana.edu/.bin/fbidq.html?robo), [bsk](http://flybase.bio.indiana.edu/.bin/fbidq.html?bsk), [lea](http://flybase.bio.indiana.edu/.bin/fbidq.html?lea), [beat-Ia](http://flybase.bio.indiana.edu/.bin/fbidq.html?beat-Ia), [gukh](http://flybase.bio.indiana.edu/.bin/fbidq.html?gukh), [plexB](http://flybase.bio.indiana.edu/.bin/fbidq.html?plexB), [Wnt5](http://flybase.bio.indiana.edu/.bin/fbidq.html?Wnt5), [beat-IIa](http://flybase.bio.indiana.edu/.bin/fbidq.html?beat-IIa), [Klp64D](http://flybase.bio.indiana.edu/.bin/fbidq.html?Klp64D), [grn](http://flybase.bio.indiana.edu/.bin/fbidq.html?grn), [sm](http://flybase.bio.indiana.edu/.bin/fbidq.html?sm), [mew](http://flybase.bio.indiana.edu/.bin/fbidq.html?mew), [Lis-1](http://flybase.bio.indiana.edu/.bin/fbidq.html?Lis-1), [dac](http://flybase.bio.indiana.edu/.bin/fbidq.html?dac), [Cdk5](http://flybase.bio.indiana.edu/.bin/fbidq.html?Cdk5), [pros](http://flybase.bio.indiana.edu/.bin/fbidq.html?pros), [tup](http://flybase.bio.indiana.edu/.bin/fbidq.html?tup), [trio](http://flybase.bio.indiana.edu/.bin/fbidq.html?trio), [daw](http://flybase.bio.indiana.edu/.bin/fbidq.html?daw), [Wnt4](http://flybase.bio.indiana.edu/.bin/fbidq.html?Wnt4), [comm](http://flybase.bio.indiana.edu/.bin/fbidq.html?comm), [Cdk5alpha](http://flybase.bio.indiana.edu/.bin/fbidq.html?Cdk5alpha), [Apc](http://flybase.bio.indiana.edu/.bin/fbidq.html?Apc), [Lim3](http://flybase.bio.indiana.edu/.bin/fbidq.html?Lim3), [sim](http://flybase.bio.indiana.edu/.bin/fbidq.html?sim), [CadN](http://flybase.bio.indiana.edu/.bin/fbidq.html?CadN), [nerfin-1](http://flybase.bio.indiana.edu/.bin/fbidq.html?nerfin-1), [trx](http://flybase.bio.indiana.edu/.bin/fbidq.html?trx), [retn](http://flybase.bio.indiana.edu/.bin/fbidq.html?retn), [en](http://flybase.bio.indiana.edu/.bin/fbidq.html?en), [dsx](http://flybase.bio.indiana.edu/.bin/fbidq.html?dsx), [unc-5](http://flybase.bio.indiana.edu/.bin/fbidq.html?unc-5), [trn](http://flybase.bio.indiana.edu/.bin/fbidq.html?trn),[CG10107](http://flybase.bio.indiana.edu/.bin/fbidq.html?CG10107), [fend](http://flybase.bio.indiana.edu/.bin/fbidq.html?fend), [CG33960](http://flybase.bio.indiana.edu/.bin/fbidq.html?CG33960) |
| [anatomical structure morphogenesis](http://amigo.geneontology.org/cgi-bin/amigo/go.cgi?view=details&query=GO:0009653) | 325 of 1594 genes, 20.4% | 1210 of 7634 genes, 15.9% | 1.41e-07 | 0.00% | 0.00 | [fz2](http://flybase.bio.indiana.edu/.bin/fbidq.html?fz2), [Sh](http://flybase.bio.indiana.edu/.bin/fbidq.html?Sh), [numb](http://flybase.bio.indiana.edu/.bin/fbidq.html?numb), [dock](http://flybase.bio.indiana.edu/.bin/fbidq.html?dock), [CG42256](http://flybase.bio.indiana.edu/.bin/fbidq.html?CG42256), [CG3703](http://flybase.bio.indiana.edu/.bin/fbidq.html?CG3703), [CG33007](http://flybase.bio.indiana.edu/.bin/fbidq.html?CG33007), [rn](http://flybase.bio.indiana.edu/.bin/fbidq.html?rn), [ap](http://flybase.bio.indiana.edu/.bin/fbidq.html?ap), [kkv](http://flybase.bio.indiana.edu/.bin/fbidq.html?kkv), [tlk](http://flybase.bio.indiana.edu/.bin/fbidq.html?tlk), [sns](http://flybase.bio.indiana.edu/.bin/fbidq.html?sns), [os](http://flybase.bio.indiana.edu/.bin/fbidq.html?os), [Axn](http://flybase.bio.indiana.edu/.bin/fbidq.html?Axn), [tok](http://flybase.bio.indiana.edu/.bin/fbidq.html?tok), [bi](http://flybase.bio.indiana.edu/.bin/fbidq.html?bi), [Ptp99A](http://flybase.bio.indiana.edu/.bin/fbidq.html?Ptp99A), [Traf6](http://flybase.bio.indiana.edu/.bin/fbidq.html?Traf6), [CG31961](http://flybase.bio.indiana.edu/.bin/fbidq.html?CG31961), [NetB](http://flybase.bio.indiana.edu/.bin/fbidq.html?NetB), [Tango10](http://flybase.bio.indiana.edu/.bin/fbidq.html?Tango10), [Oseg4](http://flybase.bio.indiana.edu/.bin/fbidq.html?Oseg4), [CG32137](http://flybase.bio.indiana.edu/.bin/fbidq.html?CG32137), [bab1](http://flybase.bio.indiana.edu/.bin/fbidq.html?bab1), [Galpha49B](http://flybase.bio.indiana.edu/.bin/fbidq.html?Galpha49B), [Lar](http://flybase.bio.indiana.edu/.bin/fbidq.html?Lar), [scrib](http://flybase.bio.indiana.edu/.bin/fbidq.html?scrib), [not](http://flybase.bio.indiana.edu/.bin/fbidq.html?not), [rut](http://flybase.bio.indiana.edu/.bin/fbidq.html?rut), [nvy](http://flybase.bio.indiana.edu/.bin/fbidq.html?nvy), [Ect3](http://flybase.bio.indiana.edu/.bin/fbidq.html?Ect3), [btd](http://flybase.bio.indiana.edu/.bin/fbidq.html?btd), [Mmp2](http://flybase.bio.indiana.edu/.bin/fbidq.html?Mmp2), [HLHmdelta](http://flybase.bio.indiana.edu/.bin/fbidq.html?HLHmdelta), [B-H1](http://flybase.bio.indiana.edu/.bin/fbidq.html?B-H1), [pim](http://flybase.bio.indiana.edu/.bin/fbidq.html?pim), [alph](http://flybase.bio.indiana.edu/.bin/fbidq.html?alph),[Traf4](http://flybase.bio.indiana.edu/.bin/fbidq.html?Traf4), [TBPH](http://flybase.bio.indiana.edu/.bin/fbidq.html?TBPH), [CG34400](http://flybase.bio.indiana.edu/.bin/fbidq.html?CG34400), [Ulp1](http://flybase.bio.indiana.edu/.bin/fbidq.html?Ulp1), [ena](http://flybase.bio.indiana.edu/.bin/fbidq.html?ena), [dsh](http://flybase.bio.indiana.edu/.bin/fbidq.html?dsh), [hth](http://flybase.bio.indiana.edu/.bin/fbidq.html?hth), [Nf-YC](http://flybase.bio.indiana.edu/.bin/fbidq.html?Nf-YC), [14-3-3zeta](http://flybase.bio.indiana.edu/.bin/fbidq.html?14-3-3zeta), [lea](http://flybase.bio.indiana.edu/.bin/fbidq.html?lea), [plexB](http://flybase.bio.indiana.edu/.bin/fbidq.html?plexB), [Klp64D](http://flybase.bio.indiana.edu/.bin/fbidq.html?Klp64D), [DAAM](http://flybase.bio.indiana.edu/.bin/fbidq.html?DAAM), [neur](http://flybase.bio.indiana.edu/.bin/fbidq.html?neur), [CycE](http://flybase.bio.indiana.edu/.bin/fbidq.html?CycE), [Brf](http://flybase.bio.indiana.edu/.bin/fbidq.html?Brf), [nmo](http://flybase.bio.indiana.edu/.bin/fbidq.html?nmo), [CG32149](http://flybase.bio.indiana.edu/.bin/fbidq.html?CG32149), [Gug](http://flybase.bio.indiana.edu/.bin/fbidq.html?Gug), [ed](http://flybase.bio.indiana.edu/.bin/fbidq.html?ed), [Actn](http://flybase.bio.indiana.edu/.bin/fbidq.html?Actn), [pad](http://flybase.bio.indiana.edu/.bin/fbidq.html?pad), [daw](http://flybase.bio.indiana.edu/.bin/fbidq.html?daw), [ImpE2](http://flybase.bio.indiana.edu/.bin/fbidq.html?ImpE2), [salr](http://flybase.bio.indiana.edu/.bin/fbidq.html?salr), [Apc](http://flybase.bio.indiana.edu/.bin/fbidq.html?Apc), [sim](http://flybase.bio.indiana.edu/.bin/fbidq.html?sim), [d4](http://flybase.bio.indiana.edu/.bin/fbidq.html?d4), [cort](http://flybase.bio.indiana.edu/.bin/fbidq.html?cort), [dan](http://flybase.bio.indiana.edu/.bin/fbidq.html?dan), [gro](http://flybase.bio.indiana.edu/.bin/fbidq.html?gro), [Mf](http://flybase.bio.indiana.edu/.bin/fbidq.html?Mf), [scyl](http://flybase.bio.indiana.edu/.bin/fbidq.html?scyl), [wnd](http://flybase.bio.indiana.edu/.bin/fbidq.html?wnd), [trx](http://flybase.bio.indiana.edu/.bin/fbidq.html?trx), [Rpd3](http://flybase.bio.indiana.edu/.bin/fbidq.html?Rpd3), [Btk29A](http://flybase.bio.indiana.edu/.bin/fbidq.html?Btk29A), [CG14367](http://flybase.bio.indiana.edu/.bin/fbidq.html?CG14367), [unc-5](http://flybase.bio.indiana.edu/.bin/fbidq.html?unc-5),[cdi](http://flybase.bio.indiana.edu/.bin/fbidq.html?cdi), [vfl](http://flybase.bio.indiana.edu/.bin/fbidq.html?vfl), [CG10107](http://flybase.bio.indiana.edu/.bin/fbidq.html?CG10107), [barr](http://flybase.bio.indiana.edu/.bin/fbidq.html?barr), [aret](http://flybase.bio.indiana.edu/.bin/fbidq.html?aret), [pygo](http://flybase.bio.indiana.edu/.bin/fbidq.html?pygo), [HLHm7](http://flybase.bio.indiana.edu/.bin/fbidq.html?HLHm7), [spag](http://flybase.bio.indiana.edu/.bin/fbidq.html?spag), [jar](http://flybase.bio.indiana.edu/.bin/fbidq.html?jar), [tow](http://flybase.bio.indiana.edu/.bin/fbidq.html?tow), [wor](http://flybase.bio.indiana.edu/.bin/fbidq.html?wor), [Atg1](http://flybase.bio.indiana.edu/.bin/fbidq.html?Atg1), [l(1)G0148](http://flybase.bio.indiana.edu/.bin/fbidq.html?l(1)G0148), [Pten](http://flybase.bio.indiana.edu/.bin/fbidq.html?Pten), [Ptp69D](http://flybase.bio.indiana.edu/.bin/fbidq.html?Ptp69D), [hts](http://flybase.bio.indiana.edu/.bin/fbidq.html?hts), [Myo10A](http://flybase.bio.indiana.edu/.bin/fbidq.html?Myo10A), [bowl](http://flybase.bio.indiana.edu/.bin/fbidq.html?bowl), [seq](http://flybase.bio.indiana.edu/.bin/fbidq.html?seq), [Dip3](http://flybase.bio.indiana.edu/.bin/fbidq.html?Dip3), [jbug](http://flybase.bio.indiana.edu/.bin/fbidq.html?jbug), [phl](http://flybase.bio.indiana.edu/.bin/fbidq.html?phl), [Cbl](http://flybase.bio.indiana.edu/.bin/fbidq.html?Cbl), [thr](http://flybase.bio.indiana.edu/.bin/fbidq.html?thr), [CG4893](http://flybase.bio.indiana.edu/.bin/fbidq.html?CG4893), [pog](http://flybase.bio.indiana.edu/.bin/fbidq.html?pog), [CASK](http://flybase.bio.indiana.edu/.bin/fbidq.html?CASK), [CG9098](http://flybase.bio.indiana.edu/.bin/fbidq.html?CG9098), [Alk](http://flybase.bio.indiana.edu/.bin/fbidq.html?Alk), [Vang](http://flybase.bio.indiana.edu/.bin/fbidq.html?Vang), [Sin3A](http://flybase.bio.indiana.edu/.bin/fbidq.html?Sin3A), [pot](http://flybase.bio.indiana.edu/.bin/fbidq.html?pot), [mip120](http://flybase.bio.indiana.edu/.bin/fbidq.html?mip120), [jumu](http://flybase.bio.indiana.edu/.bin/fbidq.html?jumu), [Smox](http://flybase.bio.indiana.edu/.bin/fbidq.html?Smox), [CG31475](http://flybase.bio.indiana.edu/.bin/fbidq.html?CG31475), [fd59A](http://flybase.bio.indiana.edu/.bin/fbidq.html?fd59A),[beat-Ic](http://flybase.bio.indiana.edu/.bin/fbidq.html?beat-Ic), [brat](http://flybase.bio.indiana.edu/.bin/fbidq.html?brat), [l(3)neo38](http://flybase.bio.indiana.edu/.bin/fbidq.html?l(3)neo38), [cindr](http://flybase.bio.indiana.edu/.bin/fbidq.html?cindr), [futsch](http://flybase.bio.indiana.edu/.bin/fbidq.html?futsch), [comm3](http://flybase.bio.indiana.edu/.bin/fbidq.html?comm3), [Ubx](http://flybase.bio.indiana.edu/.bin/fbidq.html?Ubx), [Ptx1](http://flybase.bio.indiana.edu/.bin/fbidq.html?Ptx1), [gukh](http://flybase.bio.indiana.edu/.bin/fbidq.html?gukh), [chm](http://flybase.bio.indiana.edu/.bin/fbidq.html?chm), [beat-IIa](http://flybase.bio.indiana.edu/.bin/fbidq.html?beat-IIa), [tsh](http://flybase.bio.indiana.edu/.bin/fbidq.html?tsh), [sm](http://flybase.bio.indiana.edu/.bin/fbidq.html?sm), [Syn2](http://flybase.bio.indiana.edu/.bin/fbidq.html?Syn2), [dac](http://flybase.bio.indiana.edu/.bin/fbidq.html?dac), [e(y)3](http://flybase.bio.indiana.edu/.bin/fbidq.html?e(y)3), [Sry-alpha](http://flybase.bio.indiana.edu/.bin/fbidq.html?Sry-alpha), [Cdk5](http://flybase.bio.indiana.edu/.bin/fbidq.html?Cdk5), [Cep135](http://flybase.bio.indiana.edu/.bin/fbidq.html?Cep135), [oc](http://flybase.bio.indiana.edu/.bin/fbidq.html?oc), [ey](http://flybase.bio.indiana.edu/.bin/fbidq.html?ey), [baz](http://flybase.bio.indiana.edu/.bin/fbidq.html?baz), [CG33275](http://flybase.bio.indiana.edu/.bin/fbidq.html?CG33275), [rst](http://flybase.bio.indiana.edu/.bin/fbidq.html?rst), [CG6398](http://flybase.bio.indiana.edu/.bin/fbidq.html?CG6398), [l(1)sc](http://flybase.bio.indiana.edu/.bin/fbidq.html?l(1)sc), [comm](http://flybase.bio.indiana.edu/.bin/fbidq.html?comm), [grh](http://flybase.bio.indiana.edu/.bin/fbidq.html?grh), [Cdk5alpha](http://flybase.bio.indiana.edu/.bin/fbidq.html?Cdk5alpha), [toy](http://flybase.bio.indiana.edu/.bin/fbidq.html?toy), [Lim3](http://flybase.bio.indiana.edu/.bin/fbidq.html?Lim3), [crol](http://flybase.bio.indiana.edu/.bin/fbidq.html?crol), [sd](http://flybase.bio.indiana.edu/.bin/fbidq.html?sd), [Aplip1](http://flybase.bio.indiana.edu/.bin/fbidq.html?Aplip1), [E(Pc)](http://flybase.bio.indiana.edu/.bin/fbidq.html?E(Pc)), [retn](http://flybase.bio.indiana.edu/.bin/fbidq.html?retn),[fs(1)h](http://flybase.bio.indiana.edu/.bin/fbidq.html?fs(1)h), [Eip63F-1](http://flybase.bio.indiana.edu/.bin/fbidq.html?Eip63F-1), [rl](http://flybase.bio.indiana.edu/.bin/fbidq.html?rl), [yrt](http://flybase.bio.indiana.edu/.bin/fbidq.html?yrt), [wls](http://flybase.bio.indiana.edu/.bin/fbidq.html?wls), [CG33960](http://flybase.bio.indiana.edu/.bin/fbidq.html?CG33960), [unk](http://flybase.bio.indiana.edu/.bin/fbidq.html?unk), [CG32653](http://flybase.bio.indiana.edu/.bin/fbidq.html?CG32653), [dnr1](http://flybase.bio.indiana.edu/.bin/fbidq.html?dnr1), [acj6](http://flybase.bio.indiana.edu/.bin/fbidq.html?acj6), [skd](http://flybase.bio.indiana.edu/.bin/fbidq.html?skd), [jing](http://flybase.bio.indiana.edu/.bin/fbidq.html?jing), [caup](http://flybase.bio.indiana.edu/.bin/fbidq.html?caup), [tll](http://flybase.bio.indiana.edu/.bin/fbidq.html?tll), [vimar](http://flybase.bio.indiana.edu/.bin/fbidq.html?vimar), [ato](http://flybase.bio.indiana.edu/.bin/fbidq.html?ato), [Iswi](http://flybase.bio.indiana.edu/.bin/fbidq.html?Iswi), [Dg](http://flybase.bio.indiana.edu/.bin/fbidq.html?Dg), [Trim9](http://flybase.bio.indiana.edu/.bin/fbidq.html?Trim9), [CG14478](http://flybase.bio.indiana.edu/.bin/fbidq.html?CG14478), [br](http://flybase.bio.indiana.edu/.bin/fbidq.html?br), [enc](http://flybase.bio.indiana.edu/.bin/fbidq.html?enc), [CG7154](http://flybase.bio.indiana.edu/.bin/fbidq.html?CG7154), [neo](http://flybase.bio.indiana.edu/.bin/fbidq.html?neo), [ksr](http://flybase.bio.indiana.edu/.bin/fbidq.html?ksr), [robo3](http://flybase.bio.indiana.edu/.bin/fbidq.html?robo3), [cuff](http://flybase.bio.indiana.edu/.bin/fbidq.html?cuff), [CG3618](http://flybase.bio.indiana.edu/.bin/fbidq.html?CG3618), [chn](http://flybase.bio.indiana.edu/.bin/fbidq.html?chn), [lola](http://flybase.bio.indiana.edu/.bin/fbidq.html?lola), [unc-104](http://flybase.bio.indiana.edu/.bin/fbidq.html?unc-104), [chif](http://flybase.bio.indiana.edu/.bin/fbidq.html?chif), [p130CAS](http://flybase.bio.indiana.edu/.bin/fbidq.html?p130CAS), [CG15312](http://flybase.bio.indiana.edu/.bin/fbidq.html?CG15312), [Appl](http://flybase.bio.indiana.edu/.bin/fbidq.html?Appl), [metro](http://flybase.bio.indiana.edu/.bin/fbidq.html?metro), [ewg](http://flybase.bio.indiana.edu/.bin/fbidq.html?ewg), [Nrk](http://flybase.bio.indiana.edu/.bin/fbidq.html?Nrk),[sano](http://flybase.bio.indiana.edu/.bin/fbidq.html?sano), [CG4328](http://flybase.bio.indiana.edu/.bin/fbidq.html?CG4328), [Su(var)3-3](http://flybase.bio.indiana.edu/.bin/fbidq.html?Su(var)3-3), [CG10249](http://flybase.bio.indiana.edu/.bin/fbidq.html?CG10249), [Taf4](http://flybase.bio.indiana.edu/.bin/fbidq.html?Taf4), [Nrg](http://flybase.bio.indiana.edu/.bin/fbidq.html?Nrg), [gogo](http://flybase.bio.indiana.edu/.bin/fbidq.html?gogo), [jeb](http://flybase.bio.indiana.edu/.bin/fbidq.html?jeb), [GluClalpha](http://flybase.bio.indiana.edu/.bin/fbidq.html?GluClalpha), [Actbeta](http://flybase.bio.indiana.edu/.bin/fbidq.html?Actbeta), [beat-Ib](http://flybase.bio.indiana.edu/.bin/fbidq.html?beat-Ib), [Sema-1a](http://flybase.bio.indiana.edu/.bin/fbidq.html?Sema-1a), [Hs3st-B](http://flybase.bio.indiana.edu/.bin/fbidq.html?Hs3st-B), [otk](http://flybase.bio.indiana.edu/.bin/fbidq.html?otk), [Sos](http://flybase.bio.indiana.edu/.bin/fbidq.html?Sos), [phyl](http://flybase.bio.indiana.edu/.bin/fbidq.html?phyl), [vg](http://flybase.bio.indiana.edu/.bin/fbidq.html?vg), [eya](http://flybase.bio.indiana.edu/.bin/fbidq.html?eya), [Fas2](http://flybase.bio.indiana.edu/.bin/fbidq.html?Fas2), [fz](http://flybase.bio.indiana.edu/.bin/fbidq.html?fz), [robo](http://flybase.bio.indiana.edu/.bin/fbidq.html?robo), [CG8557](http://flybase.bio.indiana.edu/.bin/fbidq.html?CG8557), [bsk](http://flybase.bio.indiana.edu/.bin/fbidq.html?bsk), [brm](http://flybase.bio.indiana.edu/.bin/fbidq.html?brm), [beat-Ia](http://flybase.bio.indiana.edu/.bin/fbidq.html?beat-Ia), [ft](http://flybase.bio.indiana.edu/.bin/fbidq.html?ft), [CG42268](http://flybase.bio.indiana.edu/.bin/fbidq.html?CG42268), [kat-60L1](http://flybase.bio.indiana.edu/.bin/fbidq.html?kat-60L1), [lft](http://flybase.bio.indiana.edu/.bin/fbidq.html?lft), [sno](http://flybase.bio.indiana.edu/.bin/fbidq.html?sno), [RhoGAPp190](http://flybase.bio.indiana.edu/.bin/fbidq.html?RhoGAPp190), [ara](http://flybase.bio.indiana.edu/.bin/fbidq.html?ara), [mam](http://flybase.bio.indiana.edu/.bin/fbidq.html?mam), [pan](http://flybase.bio.indiana.edu/.bin/fbidq.html?pan), [Dll](http://flybase.bio.indiana.edu/.bin/fbidq.html?Dll),[grn](http://flybase.bio.indiana.edu/.bin/fbidq.html?grn), [mew](http://flybase.bio.indiana.edu/.bin/fbidq.html?mew), [Lis-1](http://flybase.bio.indiana.edu/.bin/fbidq.html?Lis-1), [didum](http://flybase.bio.indiana.edu/.bin/fbidq.html?didum), [dap](http://flybase.bio.indiana.edu/.bin/fbidq.html?dap), [ems](http://flybase.bio.indiana.edu/.bin/fbidq.html?ems), [eys](http://flybase.bio.indiana.edu/.bin/fbidq.html?eys), [Bx](http://flybase.bio.indiana.edu/.bin/fbidq.html?Bx), [Mob2](http://flybase.bio.indiana.edu/.bin/fbidq.html?Mob2), [pros](http://flybase.bio.indiana.edu/.bin/fbidq.html?pros), [tup](http://flybase.bio.indiana.edu/.bin/fbidq.html?tup), [trio](http://flybase.bio.indiana.edu/.bin/fbidq.html?trio), [CG1463](http://flybase.bio.indiana.edu/.bin/fbidq.html?CG1463), [Wnt4](http://flybase.bio.indiana.edu/.bin/fbidq.html?Wnt4), [rictor](http://flybase.bio.indiana.edu/.bin/fbidq.html?rictor), [Oseg6](http://flybase.bio.indiana.edu/.bin/fbidq.html?Oseg6), [Glut4EF](http://flybase.bio.indiana.edu/.bin/fbidq.html?Glut4EF), [pigs](http://flybase.bio.indiana.edu/.bin/fbidq.html?pigs), [nerfin-1](http://flybase.bio.indiana.edu/.bin/fbidq.html?nerfin-1), [bchs](http://flybase.bio.indiana.edu/.bin/fbidq.html?bchs), [pk](http://flybase.bio.indiana.edu/.bin/fbidq.html?pk), [dsx](http://flybase.bio.indiana.edu/.bin/fbidq.html?dsx), [chinmo](http://flybase.bio.indiana.edu/.bin/fbidq.html?chinmo), [betaTub85D](http://flybase.bio.indiana.edu/.bin/fbidq.html?betaTub85D), [Ssdp](http://flybase.bio.indiana.edu/.bin/fbidq.html?Ssdp), [klu](http://flybase.bio.indiana.edu/.bin/fbidq.html?klu), [sqz](http://flybase.bio.indiana.edu/.bin/fbidq.html?sqz), [fend](http://flybase.bio.indiana.edu/.bin/fbidq.html?fend), [Cad74A](http://flybase.bio.indiana.edu/.bin/fbidq.html?Cad74A), [Lim1](http://flybase.bio.indiana.edu/.bin/fbidq.html?Lim1), [sr](http://flybase.bio.indiana.edu/.bin/fbidq.html?sr), [nkd](http://flybase.bio.indiana.edu/.bin/fbidq.html?nkd), [tld](http://flybase.bio.indiana.edu/.bin/fbidq.html?tld), [danr](http://flybase.bio.indiana.edu/.bin/fbidq.html?danr), [vvl](http://flybase.bio.indiana.edu/.bin/fbidq.html?vvl), [RhoGEF4](http://flybase.bio.indiana.edu/.bin/fbidq.html?RhoGEF4), [rg](http://flybase.bio.indiana.edu/.bin/fbidq.html?rg), [betaInt-nu](http://flybase.bio.indiana.edu/.bin/fbidq.html?betaInt-nu),[Brd](http://flybase.bio.indiana.edu/.bin/fbidq.html?Brd), [sgg](http://flybase.bio.indiana.edu/.bin/fbidq.html?sgg), [gcm2](http://flybase.bio.indiana.edu/.bin/fbidq.html?gcm2), [Nmdar1](http://flybase.bio.indiana.edu/.bin/fbidq.html?Nmdar1), [Eip93F](http://flybase.bio.indiana.edu/.bin/fbidq.html?Eip93F), [mp](http://flybase.bio.indiana.edu/.bin/fbidq.html?mp), [gcm](http://flybase.bio.indiana.edu/.bin/fbidq.html?gcm), [CG5890](http://flybase.bio.indiana.edu/.bin/fbidq.html?CG5890), [SP2353](http://flybase.bio.indiana.edu/.bin/fbidq.html?SP2353), [ase](http://flybase.bio.indiana.edu/.bin/fbidq.html?ase), [ovo](http://flybase.bio.indiana.edu/.bin/fbidq.html?ovo), [CG32685](http://flybase.bio.indiana.edu/.bin/fbidq.html?CG32685), [ds](http://flybase.bio.indiana.edu/.bin/fbidq.html?ds), [Poxn](http://flybase.bio.indiana.edu/.bin/fbidq.html?Poxn), [sals](http://flybase.bio.indiana.edu/.bin/fbidq.html?sals), [Rbp9](http://flybase.bio.indiana.edu/.bin/fbidq.html?Rbp9), [fred](http://flybase.bio.indiana.edu/.bin/fbidq.html?fred), [Drl-2](http://flybase.bio.indiana.edu/.bin/fbidq.html?Drl-2), [eIF4G2](http://flybase.bio.indiana.edu/.bin/fbidq.html?eIF4G2), [CG32944](http://flybase.bio.indiana.edu/.bin/fbidq.html?CG32944), [MYPT-75D](http://flybase.bio.indiana.edu/.bin/fbidq.html?MYPT-75D), [ush](http://flybase.bio.indiana.edu/.bin/fbidq.html?ush), [Fak56D](http://flybase.bio.indiana.edu/.bin/fbidq.html?Fak56D), [Gap1](http://flybase.bio.indiana.edu/.bin/fbidq.html?Gap1), [run](http://flybase.bio.indiana.edu/.bin/fbidq.html?run), [ko](http://flybase.bio.indiana.edu/.bin/fbidq.html?ko), [Kap3](http://flybase.bio.indiana.edu/.bin/fbidq.html?Kap3), [Eip71CD](http://flybase.bio.indiana.edu/.bin/fbidq.html?Eip71CD), [stan](http://flybase.bio.indiana.edu/.bin/fbidq.html?stan), [CG12424](http://flybase.bio.indiana.edu/.bin/fbidq.html?CG12424), [tutl](http://flybase.bio.indiana.edu/.bin/fbidq.html?tutl), [Ptth](http://flybase.bio.indiana.edu/.bin/fbidq.html?Ptth), [Sp1](http://flybase.bio.indiana.edu/.bin/fbidq.html?Sp1), [ph-p](http://flybase.bio.indiana.edu/.bin/fbidq.html?ph-p), [sna](http://flybase.bio.indiana.edu/.bin/fbidq.html?sna),[Wnt5](http://flybase.bio.indiana.edu/.bin/fbidq.html?Wnt5), [Ggamma1](http://flybase.bio.indiana.edu/.bin/fbidq.html?Ggamma1), [esg](http://flybase.bio.indiana.edu/.bin/fbidq.html?esg), [sif](http://flybase.bio.indiana.edu/.bin/fbidq.html?sif), [dom](http://flybase.bio.indiana.edu/.bin/fbidq.html?dom), [Sas-4](http://flybase.bio.indiana.edu/.bin/fbidq.html?Sas-4), [ssh](http://flybase.bio.indiana.edu/.bin/fbidq.html?ssh), [klar](http://flybase.bio.indiana.edu/.bin/fbidq.html?klar), [mle](http://flybase.bio.indiana.edu/.bin/fbidq.html?mle), [Hr51](http://flybase.bio.indiana.edu/.bin/fbidq.html?Hr51), [shi](http://flybase.bio.indiana.edu/.bin/fbidq.html?shi), [CG11155](http://flybase.bio.indiana.edu/.bin/fbidq.html?CG11155), [siz](http://flybase.bio.indiana.edu/.bin/fbidq.html?siz), [B-H2](http://flybase.bio.indiana.edu/.bin/fbidq.html?B-H2), [tyn](http://flybase.bio.indiana.edu/.bin/fbidq.html?tyn), [wg](http://flybase.bio.indiana.edu/.bin/fbidq.html?wg), [fkh](http://flybase.bio.indiana.edu/.bin/fbidq.html?fkh), [CadN](http://flybase.bio.indiana.edu/.bin/fbidq.html?CadN), [E(bx)](http://flybase.bio.indiana.edu/.bin/fbidq.html?E(bx)), [en](http://flybase.bio.indiana.edu/.bin/fbidq.html?en), [klg](http://flybase.bio.indiana.edu/.bin/fbidq.html?klg), [sfl](http://flybase.bio.indiana.edu/.bin/fbidq.html?sfl), [Sip1](http://flybase.bio.indiana.edu/.bin/fbidq.html?Sip1), [Snap](http://flybase.bio.indiana.edu/.bin/fbidq.html?Snap), [trn](http://flybase.bio.indiana.edu/.bin/fbidq.html?trn), [Pitslre](http://flybase.bio.indiana.edu/.bin/fbidq.html?Pitslre), [bt](http://flybase.bio.indiana.edu/.bin/fbidq.html?bt), [pburs](http://flybase.bio.indiana.edu/.bin/fbidq.html?pburs), [cos](http://flybase.bio.indiana.edu/.bin/fbidq.html?cos), [Ct](http://flybase.bio.indiana.edu/.bin/fbidq.html?Ct) |
| [regulation of macromolecule metabolic process](http://amigo.geneontology.org/cgi-bin/amigo/go.cgi?view=details&query=GO:0060255) | 260 of 1594 genes, 16.3% | 926 of 7634 genes, 12.1% | 1.58e-07 | 0.00% | 0.00 | [fz2](http://flybase.bio.indiana.edu/.bin/fbidq.html?fz2), [Ets65A](http://flybase.bio.indiana.edu/.bin/fbidq.html?Ets65A), [tna](http://flybase.bio.indiana.edu/.bin/fbidq.html?tna), [Sxl](http://flybase.bio.indiana.edu/.bin/fbidq.html?Sxl), [mub](http://flybase.bio.indiana.edu/.bin/fbidq.html?mub), [JIL-1](http://flybase.bio.indiana.edu/.bin/fbidq.html?JIL-1), [rn](http://flybase.bio.indiana.edu/.bin/fbidq.html?rn), [ap](http://flybase.bio.indiana.edu/.bin/fbidq.html?ap), [jigr1](http://flybase.bio.indiana.edu/.bin/fbidq.html?jigr1), [tok](http://flybase.bio.indiana.edu/.bin/fbidq.html?tok), [bi](http://flybase.bio.indiana.edu/.bin/fbidq.html?bi), [Hcf](http://flybase.bio.indiana.edu/.bin/fbidq.html?Hcf), [Traf6](http://flybase.bio.indiana.edu/.bin/fbidq.html?Traf6), [NC2alpha](http://flybase.bio.indiana.edu/.bin/fbidq.html?NC2alpha), [bab1](http://flybase.bio.indiana.edu/.bin/fbidq.html?bab1), [Snoo](http://flybase.bio.indiana.edu/.bin/fbidq.html?Snoo), [HGTX](http://flybase.bio.indiana.edu/.bin/fbidq.html?HGTX), [not](http://flybase.bio.indiana.edu/.bin/fbidq.html?not), [nvy](http://flybase.bio.indiana.edu/.bin/fbidq.html?nvy), [nub](http://flybase.bio.indiana.edu/.bin/fbidq.html?nub), [nab](http://flybase.bio.indiana.edu/.bin/fbidq.html?nab), [btd](http://flybase.bio.indiana.edu/.bin/fbidq.html?btd), [fd68A](http://flybase.bio.indiana.edu/.bin/fbidq.html?fd68A), [U2af38](http://flybase.bio.indiana.edu/.bin/fbidq.html?U2af38), [HLHmdelta](http://flybase.bio.indiana.edu/.bin/fbidq.html?HLHmdelta), [B-H1](http://flybase.bio.indiana.edu/.bin/fbidq.html?B-H1), [Nipped-A](http://flybase.bio.indiana.edu/.bin/fbidq.html?Nipped-A), [ATbp](http://flybase.bio.indiana.edu/.bin/fbidq.html?ATbp), [7B2](http://flybase.bio.indiana.edu/.bin/fbidq.html?7B2), [alph](http://flybase.bio.indiana.edu/.bin/fbidq.html?alph), [Spf45](http://flybase.bio.indiana.edu/.bin/fbidq.html?Spf45), [Fer2](http://flybase.bio.indiana.edu/.bin/fbidq.html?Fer2), [fd102C](http://flybase.bio.indiana.edu/.bin/fbidq.html?fd102C), [hth](http://flybase.bio.indiana.edu/.bin/fbidq.html?hth), [Nf-YC](http://flybase.bio.indiana.edu/.bin/fbidq.html?Nf-YC), [14-3-3zeta](http://flybase.bio.indiana.edu/.bin/fbidq.html?14-3-3zeta), [Hel25E](http://flybase.bio.indiana.edu/.bin/fbidq.html?Hel25E), [Hey](http://flybase.bio.indiana.edu/.bin/fbidq.html?Hey),[loqs](http://flybase.bio.indiana.edu/.bin/fbidq.html?loqs), [CycE](http://flybase.bio.indiana.edu/.bin/fbidq.html?CycE), [Brf](http://flybase.bio.indiana.edu/.bin/fbidq.html?Brf), [Psi](http://flybase.bio.indiana.edu/.bin/fbidq.html?Psi), [Vdup1](http://flybase.bio.indiana.edu/.bin/fbidq.html?Vdup1), [Gug](http://flybase.bio.indiana.edu/.bin/fbidq.html?Gug), [pad](http://flybase.bio.indiana.edu/.bin/fbidq.html?pad), [orb2](http://flybase.bio.indiana.edu/.bin/fbidq.html?orb2), [Rx](http://flybase.bio.indiana.edu/.bin/fbidq.html?Rx), [MED1](http://flybase.bio.indiana.edu/.bin/fbidq.html?MED1), [salr](http://flybase.bio.indiana.edu/.bin/fbidq.html?salr), [wdn](http://flybase.bio.indiana.edu/.bin/fbidq.html?wdn), [sim](http://flybase.bio.indiana.edu/.bin/fbidq.html?sim), [d4](http://flybase.bio.indiana.edu/.bin/fbidq.html?d4), [cort](http://flybase.bio.indiana.edu/.bin/fbidq.html?cort), [dan](http://flybase.bio.indiana.edu/.bin/fbidq.html?dan), [gro](http://flybase.bio.indiana.edu/.bin/fbidq.html?gro), [Jarid2](http://flybase.bio.indiana.edu/.bin/fbidq.html?Jarid2), [bin3](http://flybase.bio.indiana.edu/.bin/fbidq.html?bin3), [trx](http://flybase.bio.indiana.edu/.bin/fbidq.html?trx), [Rpd3](http://flybase.bio.indiana.edu/.bin/fbidq.html?Rpd3), [tyf](http://flybase.bio.indiana.edu/.bin/fbidq.html?tyf), [vfl](http://flybase.bio.indiana.edu/.bin/fbidq.html?vfl), [aret](http://flybase.bio.indiana.edu/.bin/fbidq.html?aret), [pygo](http://flybase.bio.indiana.edu/.bin/fbidq.html?pygo), [HLHm7](http://flybase.bio.indiana.edu/.bin/fbidq.html?HLHm7), [z](http://flybase.bio.indiana.edu/.bin/fbidq.html?z), [HLHmgamma](http://flybase.bio.indiana.edu/.bin/fbidq.html?HLHmgamma), [Sox21b](http://flybase.bio.indiana.edu/.bin/fbidq.html?Sox21b), [wor](http://flybase.bio.indiana.edu/.bin/fbidq.html?wor), [Pten](http://flybase.bio.indiana.edu/.bin/fbidq.html?Pten), [wda](http://flybase.bio.indiana.edu/.bin/fbidq.html?wda), [mira](http://flybase.bio.indiana.edu/.bin/fbidq.html?mira), [MED14](http://flybase.bio.indiana.edu/.bin/fbidq.html?MED14), [pUf68](http://flybase.bio.indiana.edu/.bin/fbidq.html?pUf68), [seq](http://flybase.bio.indiana.edu/.bin/fbidq.html?seq), [Dip3](http://flybase.bio.indiana.edu/.bin/fbidq.html?Dip3), [HDAC4](http://flybase.bio.indiana.edu/.bin/fbidq.html?HDAC4), [elk](http://flybase.bio.indiana.edu/.bin/fbidq.html?elk), [phl](http://flybase.bio.indiana.edu/.bin/fbidq.html?phl), [Cbl](http://flybase.bio.indiana.edu/.bin/fbidq.html?Cbl),[Pdp1](http://flybase.bio.indiana.edu/.bin/fbidq.html?Pdp1), [Alk](http://flybase.bio.indiana.edu/.bin/fbidq.html?Alk), [Sin3A](http://flybase.bio.indiana.edu/.bin/fbidq.html?Sin3A), [Pde1c](http://flybase.bio.indiana.edu/.bin/fbidq.html?Pde1c), [mip120](http://flybase.bio.indiana.edu/.bin/fbidq.html?mip120), [casp](http://flybase.bio.indiana.edu/.bin/fbidq.html?casp), [jumu](http://flybase.bio.indiana.edu/.bin/fbidq.html?jumu), [Smox](http://flybase.bio.indiana.edu/.bin/fbidq.html?Smox), [Su(var)2-HP2](http://flybase.bio.indiana.edu/.bin/fbidq.html?Su(var)2-HP2), [Usp7](http://flybase.bio.indiana.edu/.bin/fbidq.html?Usp7), [fd59A](http://flybase.bio.indiana.edu/.bin/fbidq.html?fd59A), [brat](http://flybase.bio.indiana.edu/.bin/fbidq.html?brat), [FBX011](http://flybase.bio.indiana.edu/.bin/fbidq.html?FBX011), [Antp](http://flybase.bio.indiana.edu/.bin/fbidq.html?Antp), [l(3)neo38](http://flybase.bio.indiana.edu/.bin/fbidq.html?l(3)neo38), [Ubx](http://flybase.bio.indiana.edu/.bin/fbidq.html?Ubx), [Ptx1](http://flybase.bio.indiana.edu/.bin/fbidq.html?Ptx1), [chm](http://flybase.bio.indiana.edu/.bin/fbidq.html?chm), [msl-1](http://flybase.bio.indiana.edu/.bin/fbidq.html?msl-1), [hbn](http://flybase.bio.indiana.edu/.bin/fbidq.html?hbn), [tsh](http://flybase.bio.indiana.edu/.bin/fbidq.html?tsh), [dac](http://flybase.bio.indiana.edu/.bin/fbidq.html?dac), [e(y)3](http://flybase.bio.indiana.edu/.bin/fbidq.html?e(y)3), [CG9007](http://flybase.bio.indiana.edu/.bin/fbidq.html?CG9007), [oc](http://flybase.bio.indiana.edu/.bin/fbidq.html?oc), [spt4](http://flybase.bio.indiana.edu/.bin/fbidq.html?spt4), [gol](http://flybase.bio.indiana.edu/.bin/fbidq.html?gol), [ey](http://flybase.bio.indiana.edu/.bin/fbidq.html?ey), [Bgb](http://flybase.bio.indiana.edu/.bin/fbidq.html?Bgb), [l(1)sc](http://flybase.bio.indiana.edu/.bin/fbidq.html?l(1)sc), [comm](http://flybase.bio.indiana.edu/.bin/fbidq.html?comm), [grh](http://flybase.bio.indiana.edu/.bin/fbidq.html?grh), [Vsx1](http://flybase.bio.indiana.edu/.bin/fbidq.html?Vsx1), [toy](http://flybase.bio.indiana.edu/.bin/fbidq.html?toy), [Lim3](http://flybase.bio.indiana.edu/.bin/fbidq.html?Lim3), [CG34362](http://flybase.bio.indiana.edu/.bin/fbidq.html?CG34362), [crol](http://flybase.bio.indiana.edu/.bin/fbidq.html?crol),[sd](http://flybase.bio.indiana.edu/.bin/fbidq.html?sd), [retn](http://flybase.bio.indiana.edu/.bin/fbidq.html?retn), [fs(1)h](http://flybase.bio.indiana.edu/.bin/fbidq.html?fs(1)h), [rl](http://flybase.bio.indiana.edu/.bin/fbidq.html?rl), [CG11347](http://flybase.bio.indiana.edu/.bin/fbidq.html?CG11347), [tey](http://flybase.bio.indiana.edu/.bin/fbidq.html?tey), [Saf-B](http://flybase.bio.indiana.edu/.bin/fbidq.html?Saf-B), [CG11294](http://flybase.bio.indiana.edu/.bin/fbidq.html?CG11294), [hiw](http://flybase.bio.indiana.edu/.bin/fbidq.html?hiw), [Atf-2](http://flybase.bio.indiana.edu/.bin/fbidq.html?Atf-2), [acj6](http://flybase.bio.indiana.edu/.bin/fbidq.html?acj6), [skd](http://flybase.bio.indiana.edu/.bin/fbidq.html?skd), [jing](http://flybase.bio.indiana.edu/.bin/fbidq.html?jing), [caup](http://flybase.bio.indiana.edu/.bin/fbidq.html?caup), [Camta](http://flybase.bio.indiana.edu/.bin/fbidq.html?Camta), [tll](http://flybase.bio.indiana.edu/.bin/fbidq.html?tll), [Cbp80](http://flybase.bio.indiana.edu/.bin/fbidq.html?Cbp80), [ato](http://flybase.bio.indiana.edu/.bin/fbidq.html?ato), [Rox8](http://flybase.bio.indiana.edu/.bin/fbidq.html?Rox8), [Iswi](http://flybase.bio.indiana.edu/.bin/fbidq.html?Iswi), [Spt6](http://flybase.bio.indiana.edu/.bin/fbidq.html?Spt6), [Dg](http://flybase.bio.indiana.edu/.bin/fbidq.html?Dg), [Pka-R1](http://flybase.bio.indiana.edu/.bin/fbidq.html?Pka-R1), [br](http://flybase.bio.indiana.edu/.bin/fbidq.html?br), [ksr](http://flybase.bio.indiana.edu/.bin/fbidq.html?ksr), [PNUTS](http://flybase.bio.indiana.edu/.bin/fbidq.html?PNUTS), [chn](http://flybase.bio.indiana.edu/.bin/fbidq.html?chn), [Gsc](http://flybase.bio.indiana.edu/.bin/fbidq.html?Gsc), [lola](http://flybase.bio.indiana.edu/.bin/fbidq.html?lola), [chif](http://flybase.bio.indiana.edu/.bin/fbidq.html?chif), [Pcl](http://flybase.bio.indiana.edu/.bin/fbidq.html?Pcl), [HLH4C](http://flybase.bio.indiana.edu/.bin/fbidq.html?HLH4C), [simj](http://flybase.bio.indiana.edu/.bin/fbidq.html?simj), [tap](http://flybase.bio.indiana.edu/.bin/fbidq.html?tap), [Awh](http://flybase.bio.indiana.edu/.bin/fbidq.html?Awh), [PHDP](http://flybase.bio.indiana.edu/.bin/fbidq.html?PHDP), [CG4328](http://flybase.bio.indiana.edu/.bin/fbidq.html?CG4328), [Su(var)3-3](http://flybase.bio.indiana.edu/.bin/fbidq.html?Su(var)3-3), [Taf4](http://flybase.bio.indiana.edu/.bin/fbidq.html?Taf4),[l(2)NC136](http://flybase.bio.indiana.edu/.bin/fbidq.html?l(2)NC136), [stau](http://flybase.bio.indiana.edu/.bin/fbidq.html?stau), [Sos](http://flybase.bio.indiana.edu/.bin/fbidq.html?Sos), [msl-2](http://flybase.bio.indiana.edu/.bin/fbidq.html?msl-2), [vg](http://flybase.bio.indiana.edu/.bin/fbidq.html?vg), [eya](http://flybase.bio.indiana.edu/.bin/fbidq.html?eya), [bsk](http://flybase.bio.indiana.edu/.bin/fbidq.html?bsk), [brm](http://flybase.bio.indiana.edu/.bin/fbidq.html?brm), [fz](http://flybase.bio.indiana.edu/.bin/fbidq.html?fz), [Vsx2](http://flybase.bio.indiana.edu/.bin/fbidq.html?Vsx2), [sno](http://flybase.bio.indiana.edu/.bin/fbidq.html?sno), [ara](http://flybase.bio.indiana.edu/.bin/fbidq.html?ara), [Sfmbt](http://flybase.bio.indiana.edu/.bin/fbidq.html?Sfmbt), [mam](http://flybase.bio.indiana.edu/.bin/fbidq.html?mam), [pan](http://flybase.bio.indiana.edu/.bin/fbidq.html?pan), [disco](http://flybase.bio.indiana.edu/.bin/fbidq.html?disco), [Dll](http://flybase.bio.indiana.edu/.bin/fbidq.html?Dll), [grn](http://flybase.bio.indiana.edu/.bin/fbidq.html?grn), [D12](http://flybase.bio.indiana.edu/.bin/fbidq.html?D12), [HLHm5](http://flybase.bio.indiana.edu/.bin/fbidq.html?HLHm5), [Lis-1](http://flybase.bio.indiana.edu/.bin/fbidq.html?Lis-1), [ems](http://flybase.bio.indiana.edu/.bin/fbidq.html?ems), [dap](http://flybase.bio.indiana.edu/.bin/fbidq.html?dap), [tup](http://flybase.bio.indiana.edu/.bin/fbidq.html?tup), [pros](http://flybase.bio.indiana.edu/.bin/fbidq.html?pros), [pdm2](http://flybase.bio.indiana.edu/.bin/fbidq.html?pdm2), [rgr](http://flybase.bio.indiana.edu/.bin/fbidq.html?rgr), [spel1](http://flybase.bio.indiana.edu/.bin/fbidq.html?spel1), [Wnt4](http://flybase.bio.indiana.edu/.bin/fbidq.html?Wnt4), [nerfin-1](http://flybase.bio.indiana.edu/.bin/fbidq.html?nerfin-1), [bchs](http://flybase.bio.indiana.edu/.bin/fbidq.html?bchs), [pr-set7](http://flybase.bio.indiana.edu/.bin/fbidq.html?pr-set7), [dsx](http://flybase.bio.indiana.edu/.bin/fbidq.html?dsx), [Ssdp](http://flybase.bio.indiana.edu/.bin/fbidq.html?Ssdp), [sqz](http://flybase.bio.indiana.edu/.bin/fbidq.html?sqz), [Fer3](http://flybase.bio.indiana.edu/.bin/fbidq.html?Fer3), [CG32105](http://flybase.bio.indiana.edu/.bin/fbidq.html?CG32105), [CG1868](http://flybase.bio.indiana.edu/.bin/fbidq.html?CG1868), [dalao](http://flybase.bio.indiana.edu/.bin/fbidq.html?dalao),[Lim1](http://flybase.bio.indiana.edu/.bin/fbidq.html?Lim1), [sr](http://flybase.bio.indiana.edu/.bin/fbidq.html?sr), [tld](http://flybase.bio.indiana.edu/.bin/fbidq.html?tld), [danr](http://flybase.bio.indiana.edu/.bin/fbidq.html?danr), [vvl](http://flybase.bio.indiana.edu/.bin/fbidq.html?vvl), [Brd8](http://flybase.bio.indiana.edu/.bin/fbidq.html?Brd8), [CG7879](http://flybase.bio.indiana.edu/.bin/fbidq.html?CG7879), [sgg](http://flybase.bio.indiana.edu/.bin/fbidq.html?sgg), [gcm2](http://flybase.bio.indiana.edu/.bin/fbidq.html?gcm2), [Eip93F](http://flybase.bio.indiana.edu/.bin/fbidq.html?Eip93F), [gcm](http://flybase.bio.indiana.edu/.bin/fbidq.html?gcm), [Suv4-20](http://flybase.bio.indiana.edu/.bin/fbidq.html?Suv4-20), [can](http://flybase.bio.indiana.edu/.bin/fbidq.html?can), [Aef1](http://flybase.bio.indiana.edu/.bin/fbidq.html?Aef1), [Hsp67Bc](http://flybase.bio.indiana.edu/.bin/fbidq.html?Hsp67Bc), [rab3-GEF](http://flybase.bio.indiana.edu/.bin/fbidq.html?rab3-GEF), [ase](http://flybase.bio.indiana.edu/.bin/fbidq.html?ase), [ovo](http://flybase.bio.indiana.edu/.bin/fbidq.html?ovo), [CG13253](http://flybase.bio.indiana.edu/.bin/fbidq.html?CG13253), [CG6197](http://flybase.bio.indiana.edu/.bin/fbidq.html?CG6197), [scro](http://flybase.bio.indiana.edu/.bin/fbidq.html?scro), [phol](http://flybase.bio.indiana.edu/.bin/fbidq.html?phol), [Dsp1](http://flybase.bio.indiana.edu/.bin/fbidq.html?Dsp1), [elav](http://flybase.bio.indiana.edu/.bin/fbidq.html?elav), [CG14216](http://flybase.bio.indiana.edu/.bin/fbidq.html?CG14216), [Oli](http://flybase.bio.indiana.edu/.bin/fbidq.html?Oli), [Nelf-A](http://flybase.bio.indiana.edu/.bin/fbidq.html?Nelf-A), [GATAd](http://flybase.bio.indiana.edu/.bin/fbidq.html?GATAd), [CG6227](http://flybase.bio.indiana.edu/.bin/fbidq.html?CG6227), [Poxn](http://flybase.bio.indiana.edu/.bin/fbidq.html?Poxn), [fbl6](http://flybase.bio.indiana.edu/.bin/fbidq.html?fbl6), [CG15376](http://flybase.bio.indiana.edu/.bin/fbidq.html?CG15376), [ush](http://flybase.bio.indiana.edu/.bin/fbidq.html?ush), [CG10336](http://flybase.bio.indiana.edu/.bin/fbidq.html?CG10336),[Gap1](http://flybase.bio.indiana.edu/.bin/fbidq.html?Gap1), [run](http://flybase.bio.indiana.edu/.bin/fbidq.html?run), [Alh](http://flybase.bio.indiana.edu/.bin/fbidq.html?Alh), [tara](http://flybase.bio.indiana.edu/.bin/fbidq.html?tara), [egg](http://flybase.bio.indiana.edu/.bin/fbidq.html?egg), [Sox102F](http://flybase.bio.indiana.edu/.bin/fbidq.html?Sox102F), [koko](http://flybase.bio.indiana.edu/.bin/fbidq.html?koko), [Ptth](http://flybase.bio.indiana.edu/.bin/fbidq.html?Ptth), [Sp1](http://flybase.bio.indiana.edu/.bin/fbidq.html?Sp1), [ph-p](http://flybase.bio.indiana.edu/.bin/fbidq.html?ph-p), [onecut](http://flybase.bio.indiana.edu/.bin/fbidq.html?onecut), [CG7757](http://flybase.bio.indiana.edu/.bin/fbidq.html?CG7757), [sna](http://flybase.bio.indiana.edu/.bin/fbidq.html?sna), [Wnt5](http://flybase.bio.indiana.edu/.bin/fbidq.html?Wnt5), [Taf1](http://flybase.bio.indiana.edu/.bin/fbidq.html?Taf1), [MBD-like](http://flybase.bio.indiana.edu/.bin/fbidq.html?MBD-like), [esg](http://flybase.bio.indiana.edu/.bin/fbidq.html?esg), [dom](http://flybase.bio.indiana.edu/.bin/fbidq.html?dom), [CG32532](http://flybase.bio.indiana.edu/.bin/fbidq.html?CG32532), [MTA1-like](http://flybase.bio.indiana.edu/.bin/fbidq.html?MTA1-like), [mle](http://flybase.bio.indiana.edu/.bin/fbidq.html?mle), [Hr51](http://flybase.bio.indiana.edu/.bin/fbidq.html?Hr51), [CG8786](http://flybase.bio.indiana.edu/.bin/fbidq.html?CG8786), [B-H2](http://flybase.bio.indiana.edu/.bin/fbidq.html?B-H2), [wg](http://flybase.bio.indiana.edu/.bin/fbidq.html?wg), [fkh](http://flybase.bio.indiana.edu/.bin/fbidq.html?fkh), [Spps](http://flybase.bio.indiana.edu/.bin/fbidq.html?Spps), [fne](http://flybase.bio.indiana.edu/.bin/fbidq.html?fne), [en](http://flybase.bio.indiana.edu/.bin/fbidq.html?en), [Utx](http://flybase.bio.indiana.edu/.bin/fbidq.html?Utx), [cos](http://flybase.bio.indiana.edu/.bin/fbidq.html?cos), [Ct](http://flybase.bio.indiana.edu/.bin/fbidq.html?Ct) |
| [single-organism process](http://amigo.geneontology.org/cgi-bin/amigo/go.cgi?view=details&query=GO:0044699) | 869 of 1594 genes, 54.5% | 3814 of 7634 genes, 50.0% | 1.60e-07 | 0.00% | 0.00 | [endoA](http://flybase.bio.indiana.edu/.bin/fbidq.html?endoA), [Sh](http://flybase.bio.indiana.edu/.bin/fbidq.html?Sh), [CG9279](http://flybase.bio.indiana.edu/.bin/fbidq.html?CG9279), [dock](http://flybase.bio.indiana.edu/.bin/fbidq.html?dock), [CG5808](http://flybase.bio.indiana.edu/.bin/fbidq.html?CG5808), [CG9004](http://flybase.bio.indiana.edu/.bin/fbidq.html?CG9004), [CG3703](http://flybase.bio.indiana.edu/.bin/fbidq.html?CG3703), [CG32135](http://flybase.bio.indiana.edu/.bin/fbidq.html?CG32135), [tna](http://flybase.bio.indiana.edu/.bin/fbidq.html?tna), [CG8500](http://flybase.bio.indiana.edu/.bin/fbidq.html?CG8500), [rn](http://flybase.bio.indiana.edu/.bin/fbidq.html?rn), [ap](http://flybase.bio.indiana.edu/.bin/fbidq.html?ap), [cenG1A](http://flybase.bio.indiana.edu/.bin/fbidq.html?cenG1A), [kkv](http://flybase.bio.indiana.edu/.bin/fbidq.html?kkv), [tlk](http://flybase.bio.indiana.edu/.bin/fbidq.html?tlk), [malpha](http://flybase.bio.indiana.edu/.bin/fbidq.html?malpha), [os](http://flybase.bio.indiana.edu/.bin/fbidq.html?os), [Axn](http://flybase.bio.indiana.edu/.bin/fbidq.html?Axn), [Gs1](http://flybase.bio.indiana.edu/.bin/fbidq.html?Gs1), [mtrm](http://flybase.bio.indiana.edu/.bin/fbidq.html?mtrm), [Hcf](http://flybase.bio.indiana.edu/.bin/fbidq.html?Hcf), [Traf6](http://flybase.bio.indiana.edu/.bin/fbidq.html?Traf6), [Leucokinin](http://flybase.bio.indiana.edu/.bin/fbidq.html?Leucokinin), [NetB](http://flybase.bio.indiana.edu/.bin/fbidq.html?NetB), [rdgC](http://flybase.bio.indiana.edu/.bin/fbidq.html?rdgC), [cid](http://flybase.bio.indiana.edu/.bin/fbidq.html?cid), [CG32137](http://flybase.bio.indiana.edu/.bin/fbidq.html?CG32137), [lute](http://flybase.bio.indiana.edu/.bin/fbidq.html?lute), [CG4476](http://flybase.bio.indiana.edu/.bin/fbidq.html?CG4476), [Ssrp](http://flybase.bio.indiana.edu/.bin/fbidq.html?Ssrp), [Lar](http://flybase.bio.indiana.edu/.bin/fbidq.html?Lar), [scrib](http://flybase.bio.indiana.edu/.bin/fbidq.html?scrib), [HGTX](http://flybase.bio.indiana.edu/.bin/fbidq.html?HGTX), [not](http://flybase.bio.indiana.edu/.bin/fbidq.html?not), [CG7650](http://flybase.bio.indiana.edu/.bin/fbidq.html?CG7650),[nvy](http://flybase.bio.indiana.edu/.bin/fbidq.html?nvy), [Ect3](http://flybase.bio.indiana.edu/.bin/fbidq.html?Ect3), [nab](http://flybase.bio.indiana.edu/.bin/fbidq.html?nab), [Ddc](http://flybase.bio.indiana.edu/.bin/fbidq.html?Ddc), [HLHmdelta](http://flybase.bio.indiana.edu/.bin/fbidq.html?HLHmdelta), [U2af38](http://flybase.bio.indiana.edu/.bin/fbidq.html?U2af38), [7B2](http://flybase.bio.indiana.edu/.bin/fbidq.html?7B2), [alph](http://flybase.bio.indiana.edu/.bin/fbidq.html?alph), [Traf4](http://flybase.bio.indiana.edu/.bin/fbidq.html?Traf4), [TBPH](http://flybase.bio.indiana.edu/.bin/fbidq.html?TBPH), [Snap25](http://flybase.bio.indiana.edu/.bin/fbidq.html?Snap25), [Teh2](http://flybase.bio.indiana.edu/.bin/fbidq.html?Teh2), [Fer2](http://flybase.bio.indiana.edu/.bin/fbidq.html?Fer2), [CG34400](http://flybase.bio.indiana.edu/.bin/fbidq.html?CG34400), [Ulp1](http://flybase.bio.indiana.edu/.bin/fbidq.html?Ulp1), [CG42330](http://flybase.bio.indiana.edu/.bin/fbidq.html?CG42330), [mGluRA](http://flybase.bio.indiana.edu/.bin/fbidq.html?mGluRA), [ena](http://flybase.bio.indiana.edu/.bin/fbidq.html?ena), [dsh](http://flybase.bio.indiana.edu/.bin/fbidq.html?dsh), [Nf-YC](http://flybase.bio.indiana.edu/.bin/fbidq.html?Nf-YC), [dpr20](http://flybase.bio.indiana.edu/.bin/fbidq.html?dpr20), [Pli](http://flybase.bio.indiana.edu/.bin/fbidq.html?Pli), [qkr58E-1](http://flybase.bio.indiana.edu/.bin/fbidq.html?qkr58E-1), [Hel25E](http://flybase.bio.indiana.edu/.bin/fbidq.html?Hel25E), [comt](http://flybase.bio.indiana.edu/.bin/fbidq.html?comt), [CG31140](http://flybase.bio.indiana.edu/.bin/fbidq.html?CG31140), [Pbprp1](http://flybase.bio.indiana.edu/.bin/fbidq.html?Pbprp1), [PP2A-B](http://flybase.bio.indiana.edu/.bin/fbidq.html?PP2A-B), [Pxn](http://flybase.bio.indiana.edu/.bin/fbidq.html?Pxn), [qvr](http://flybase.bio.indiana.edu/.bin/fbidq.html?qvr), [jet](http://flybase.bio.indiana.edu/.bin/fbidq.html?jet), [DAAM](http://flybase.bio.indiana.edu/.bin/fbidq.html?DAAM), [pall](http://flybase.bio.indiana.edu/.bin/fbidq.html?pall), [rho-5](http://flybase.bio.indiana.edu/.bin/fbidq.html?rho-5),[nmo](http://flybase.bio.indiana.edu/.bin/fbidq.html?nmo), [Tusp](http://flybase.bio.indiana.edu/.bin/fbidq.html?Tusp), [5-HT1A](http://flybase.bio.indiana.edu/.bin/fbidq.html?5-HT1A), [CG13248](http://flybase.bio.indiana.edu/.bin/fbidq.html?CG13248), [Psi](http://flybase.bio.indiana.edu/.bin/fbidq.html?Psi), [Gug](http://flybase.bio.indiana.edu/.bin/fbidq.html?Gug), [Actn](http://flybase.bio.indiana.edu/.bin/fbidq.html?Actn), [ed](http://flybase.bio.indiana.edu/.bin/fbidq.html?ed), [pad](http://flybase.bio.indiana.edu/.bin/fbidq.html?pad), [CG11317](http://flybase.bio.indiana.edu/.bin/fbidq.html?CG11317), [CG33181](http://flybase.bio.indiana.edu/.bin/fbidq.html?CG33181), [daw](http://flybase.bio.indiana.edu/.bin/fbidq.html?daw), [CG5036](http://flybase.bio.indiana.edu/.bin/fbidq.html?CG5036), [5-HT1B](http://flybase.bio.indiana.edu/.bin/fbidq.html?5-HT1B), [Poc1](http://flybase.bio.indiana.edu/.bin/fbidq.html?Poc1), [Fhos](http://flybase.bio.indiana.edu/.bin/fbidq.html?Fhos), [Rx](http://flybase.bio.indiana.edu/.bin/fbidq.html?Rx), [rogdi](http://flybase.bio.indiana.edu/.bin/fbidq.html?rogdi), [salr](http://flybase.bio.indiana.edu/.bin/fbidq.html?salr), [Apc](http://flybase.bio.indiana.edu/.bin/fbidq.html?Apc), [CG8795](http://flybase.bio.indiana.edu/.bin/fbidq.html?CG8795), [VGAT](http://flybase.bio.indiana.edu/.bin/fbidq.html?VGAT), [d4](http://flybase.bio.indiana.edu/.bin/fbidq.html?d4), [dan](http://flybase.bio.indiana.edu/.bin/fbidq.html?dan), [cort](http://flybase.bio.indiana.edu/.bin/fbidq.html?cort), [tau](http://flybase.bio.indiana.edu/.bin/fbidq.html?tau), [beat-Vb](http://flybase.bio.indiana.edu/.bin/fbidq.html?beat-Vb), [Jarid2](http://flybase.bio.indiana.edu/.bin/fbidq.html?Jarid2), [RN-tre](http://flybase.bio.indiana.edu/.bin/fbidq.html?RN-tre), [Mf](http://flybase.bio.indiana.edu/.bin/fbidq.html?Mf), [wnd](http://flybase.bio.indiana.edu/.bin/fbidq.html?wnd), [trx](http://flybase.bio.indiana.edu/.bin/fbidq.html?trx), [rdo](http://flybase.bio.indiana.edu/.bin/fbidq.html?rdo), [Btk29A](http://flybase.bio.indiana.edu/.bin/fbidq.html?Btk29A), [SMC2](http://flybase.bio.indiana.edu/.bin/fbidq.html?SMC2), [PQBP-1](http://flybase.bio.indiana.edu/.bin/fbidq.html?PQBP-1),[MED25](http://flybase.bio.indiana.edu/.bin/fbidq.html?MED25), [ETH](http://flybase.bio.indiana.edu/.bin/fbidq.html?ETH), [unc-5](http://flybase.bio.indiana.edu/.bin/fbidq.html?unc-5), [cdi](http://flybase.bio.indiana.edu/.bin/fbidq.html?cdi), [CG13907](http://flybase.bio.indiana.edu/.bin/fbidq.html?CG13907), [vfl](http://flybase.bio.indiana.edu/.bin/fbidq.html?vfl), [CG1139](http://flybase.bio.indiana.edu/.bin/fbidq.html?CG1139), [barr](http://flybase.bio.indiana.edu/.bin/fbidq.html?barr), [CG42321](http://flybase.bio.indiana.edu/.bin/fbidq.html?CG42321), [NPFR1](http://flybase.bio.indiana.edu/.bin/fbidq.html?NPFR1), [pygo](http://flybase.bio.indiana.edu/.bin/fbidq.html?pygo), [z](http://flybase.bio.indiana.edu/.bin/fbidq.html?z), [HLHmgamma](http://flybase.bio.indiana.edu/.bin/fbidq.html?HLHmgamma), [spag](http://flybase.bio.indiana.edu/.bin/fbidq.html?spag), [CG15556](http://flybase.bio.indiana.edu/.bin/fbidq.html?CG15556), [jar](http://flybase.bio.indiana.edu/.bin/fbidq.html?jar), [Klp68D](http://flybase.bio.indiana.edu/.bin/fbidq.html?Klp68D), [Ror](http://flybase.bio.indiana.edu/.bin/fbidq.html?Ror), [CG7368](http://flybase.bio.indiana.edu/.bin/fbidq.html?CG7368), [wor](http://flybase.bio.indiana.edu/.bin/fbidq.html?wor), [Atg1](http://flybase.bio.indiana.edu/.bin/fbidq.html?Atg1), [Fcp1](http://flybase.bio.indiana.edu/.bin/fbidq.html?Fcp1), [Ptp69D](http://flybase.bio.indiana.edu/.bin/fbidq.html?Ptp69D), [hts](http://flybase.bio.indiana.edu/.bin/fbidq.html?hts), [dpr9](http://flybase.bio.indiana.edu/.bin/fbidq.html?dpr9), [pUf68](http://flybase.bio.indiana.edu/.bin/fbidq.html?pUf68), [bowl](http://flybase.bio.indiana.edu/.bin/fbidq.html?bowl), [seq](http://flybase.bio.indiana.edu/.bin/fbidq.html?seq), [jbug](http://flybase.bio.indiana.edu/.bin/fbidq.html?jbug), [HDAC4](http://flybase.bio.indiana.edu/.bin/fbidq.html?HDAC4), [CG42575](http://flybase.bio.indiana.edu/.bin/fbidq.html?CG42575), [tay](http://flybase.bio.indiana.edu/.bin/fbidq.html?tay), [CG1671](http://flybase.bio.indiana.edu/.bin/fbidq.html?CG1671), [Cbl](http://flybase.bio.indiana.edu/.bin/fbidq.html?Cbl),[thr](http://flybase.bio.indiana.edu/.bin/fbidq.html?thr), [pog](http://flybase.bio.indiana.edu/.bin/fbidq.html?pog), [CG12071](http://flybase.bio.indiana.edu/.bin/fbidq.html?CG12071), [Pdp1](http://flybase.bio.indiana.edu/.bin/fbidq.html?Pdp1), [CASK](http://flybase.bio.indiana.edu/.bin/fbidq.html?CASK), [CG10251](http://flybase.bio.indiana.edu/.bin/fbidq.html?CG10251), [CG13743](http://flybase.bio.indiana.edu/.bin/fbidq.html?CG13743), [CG32758](http://flybase.bio.indiana.edu/.bin/fbidq.html?CG32758), [mtt](http://flybase.bio.indiana.edu/.bin/fbidq.html?mtt), [CG5535](http://flybase.bio.indiana.edu/.bin/fbidq.html?CG5535), [disp](http://flybase.bio.indiana.edu/.bin/fbidq.html?disp), [mip120](http://flybase.bio.indiana.edu/.bin/fbidq.html?mip120), [cpx](http://flybase.bio.indiana.edu/.bin/fbidq.html?cpx), [CG4022](http://flybase.bio.indiana.edu/.bin/fbidq.html?CG4022), [LpR1](http://flybase.bio.indiana.edu/.bin/fbidq.html?LpR1), [jumu](http://flybase.bio.indiana.edu/.bin/fbidq.html?jumu), [Su(var)2-HP2](http://flybase.bio.indiana.edu/.bin/fbidq.html?Su(var)2-HP2), [gfA](http://flybase.bio.indiana.edu/.bin/fbidq.html?gfA), [Usp7](http://flybase.bio.indiana.edu/.bin/fbidq.html?Usp7), [CG31781](http://flybase.bio.indiana.edu/.bin/fbidq.html?CG31781), [fd59A](http://flybase.bio.indiana.edu/.bin/fbidq.html?fd59A), [beat-Ic](http://flybase.bio.indiana.edu/.bin/fbidq.html?beat-Ic), [Rph](http://flybase.bio.indiana.edu/.bin/fbidq.html?Rph), [brat](http://flybase.bio.indiana.edu/.bin/fbidq.html?brat), [FBX011](http://flybase.bio.indiana.edu/.bin/fbidq.html?FBX011), [ST6Gal](http://flybase.bio.indiana.edu/.bin/fbidq.html?ST6Gal), [CG17343](http://flybase.bio.indiana.edu/.bin/fbidq.html?CG17343), [dpr14](http://flybase.bio.indiana.edu/.bin/fbidq.html?dpr14), [GABA-B-R2](http://flybase.bio.indiana.edu/.bin/fbidq.html?GABA-B-R2),[CG6231](http://flybase.bio.indiana.edu/.bin/fbidq.html?CG6231), [CG7051](http://flybase.bio.indiana.edu/.bin/fbidq.html?CG7051), [futsch](http://flybase.bio.indiana.edu/.bin/fbidq.html?futsch), [Ubx](http://flybase.bio.indiana.edu/.bin/fbidq.html?Ubx), [Cbp53E](http://flybase.bio.indiana.edu/.bin/fbidq.html?Cbp53E), [gukh](http://flybase.bio.indiana.edu/.bin/fbidq.html?gukh), [Dscam3](http://flybase.bio.indiana.edu/.bin/fbidq.html?Dscam3), [Jupiter](http://flybase.bio.indiana.edu/.bin/fbidq.html?Jupiter), [chm](http://flybase.bio.indiana.edu/.bin/fbidq.html?chm), [Rim](http://flybase.bio.indiana.edu/.bin/fbidq.html?Rim), [Rpt3R](http://flybase.bio.indiana.edu/.bin/fbidq.html?Rpt3R), [msl-1](http://flybase.bio.indiana.edu/.bin/fbidq.html?msl-1), [beat-IIa](http://flybase.bio.indiana.edu/.bin/fbidq.html?beat-IIa), [corn](http://flybase.bio.indiana.edu/.bin/fbidq.html?corn), [VhaM9.7-a](http://flybase.bio.indiana.edu/.bin/fbidq.html?VhaM9.7-a), [CG11597](http://flybase.bio.indiana.edu/.bin/fbidq.html?CG11597), [Sur-8](http://flybase.bio.indiana.edu/.bin/fbidq.html?Sur-8), [CG42574](http://flybase.bio.indiana.edu/.bin/fbidq.html?CG42574), [Rgl](http://flybase.bio.indiana.edu/.bin/fbidq.html?Rgl), [dac](http://flybase.bio.indiana.edu/.bin/fbidq.html?dac), [Nrx-1](http://flybase.bio.indiana.edu/.bin/fbidq.html?Nrx-1), [CG9007](http://flybase.bio.indiana.edu/.bin/fbidq.html?CG9007), [CG33203](http://flybase.bio.indiana.edu/.bin/fbidq.html?CG33203), [mav](http://flybase.bio.indiana.edu/.bin/fbidq.html?mav), [Ilp7](http://flybase.bio.indiana.edu/.bin/fbidq.html?Ilp7), [CG31122](http://flybase.bio.indiana.edu/.bin/fbidq.html?CG31122), [CG15439](http://flybase.bio.indiana.edu/.bin/fbidq.html?CG15439), [CG33275](http://flybase.bio.indiana.edu/.bin/fbidq.html?CG33275), [Eaat2](http://flybase.bio.indiana.edu/.bin/fbidq.html?Eaat2),[ventrally-expressed-protein-D](http://flybase.bio.indiana.edu/.bin/fbidq.html?ventrally-expressed-protein-D), [toy](http://flybase.bio.indiana.edu/.bin/fbidq.html?toy), [CG40351](http://flybase.bio.indiana.edu/.bin/fbidq.html?CG40351), [Lim3](http://flybase.bio.indiana.edu/.bin/fbidq.html?Lim3), [CG14681](http://flybase.bio.indiana.edu/.bin/fbidq.html?CG14681), [sd](http://flybase.bio.indiana.edu/.bin/fbidq.html?sd), [Aplip1](http://flybase.bio.indiana.edu/.bin/fbidq.html?Aplip1), [CG1239](http://flybase.bio.indiana.edu/.bin/fbidq.html?CG1239), [fs(1)h](http://flybase.bio.indiana.edu/.bin/fbidq.html?fs(1)h), [Eip63F-1](http://flybase.bio.indiana.edu/.bin/fbidq.html?Eip63F-1), [CG11347](http://flybase.bio.indiana.edu/.bin/fbidq.html?CG11347), [CG5853](http://flybase.bio.indiana.edu/.bin/fbidq.html?CG5853), [Ggamma30A](http://flybase.bio.indiana.edu/.bin/fbidq.html?Ggamma30A), [yrt](http://flybase.bio.indiana.edu/.bin/fbidq.html?yrt), [dpr7](http://flybase.bio.indiana.edu/.bin/fbidq.html?dpr7), [wls](http://flybase.bio.indiana.edu/.bin/fbidq.html?wls), [skl](http://flybase.bio.indiana.edu/.bin/fbidq.html?skl), [CG1688](http://flybase.bio.indiana.edu/.bin/fbidq.html?CG1688), [hiw](http://flybase.bio.indiana.edu/.bin/fbidq.html?hiw), [CG32653](http://flybase.bio.indiana.edu/.bin/fbidq.html?CG32653), [Atf-2](http://flybase.bio.indiana.edu/.bin/fbidq.html?Atf-2), [n-syb](http://flybase.bio.indiana.edu/.bin/fbidq.html?n-syb), [skd](http://flybase.bio.indiana.edu/.bin/fbidq.html?skd), [jing](http://flybase.bio.indiana.edu/.bin/fbidq.html?jing), [CG9850](http://flybase.bio.indiana.edu/.bin/fbidq.html?CG9850), [katanin-60](http://flybase.bio.indiana.edu/.bin/fbidq.html?katanin-60), [Camta](http://flybase.bio.indiana.edu/.bin/fbidq.html?Camta),[CG8177](http://flybase.bio.indiana.edu/.bin/fbidq.html?CG8177), [tll](http://flybase.bio.indiana.edu/.bin/fbidq.html?tll), [ato](http://flybase.bio.indiana.edu/.bin/fbidq.html?ato), [Fmrf](http://flybase.bio.indiana.edu/.bin/fbidq.html?Fmrf), [Iswi](http://flybase.bio.indiana.edu/.bin/fbidq.html?Iswi), [CG5742](http://flybase.bio.indiana.edu/.bin/fbidq.html?CG5742), [obst-A](http://flybase.bio.indiana.edu/.bin/fbidq.html?obst-A), [Pka-R1](http://flybase.bio.indiana.edu/.bin/fbidq.html?Pka-R1), [CG14478](http://flybase.bio.indiana.edu/.bin/fbidq.html?CG14478), [CG9867](http://flybase.bio.indiana.edu/.bin/fbidq.html?CG9867), [mago](http://flybase.bio.indiana.edu/.bin/fbidq.html?mago), [enc](http://flybase.bio.indiana.edu/.bin/fbidq.html?enc), [cuff](http://flybase.bio.indiana.edu/.bin/fbidq.html?cuff), [CG42533](http://flybase.bio.indiana.edu/.bin/fbidq.html?CG42533), [ksr](http://flybase.bio.indiana.edu/.bin/fbidq.html?ksr), [CG7837](http://flybase.bio.indiana.edu/.bin/fbidq.html?CG7837), [PMCA](http://flybase.bio.indiana.edu/.bin/fbidq.html?PMCA), [CG3618](http://flybase.bio.indiana.edu/.bin/fbidq.html?CG3618), [Gyc-89Da](http://flybase.bio.indiana.edu/.bin/fbidq.html?Gyc-89Da), [brp](http://flybase.bio.indiana.edu/.bin/fbidq.html?brp), [chn](http://flybase.bio.indiana.edu/.bin/fbidq.html?chn), [ssp3](http://flybase.bio.indiana.edu/.bin/fbidq.html?ssp3), [Khc-73](http://flybase.bio.indiana.edu/.bin/fbidq.html?Khc-73), [chif](http://flybase.bio.indiana.edu/.bin/fbidq.html?chif), [D2R](http://flybase.bio.indiana.edu/.bin/fbidq.html?D2R), [p130CAS](http://flybase.bio.indiana.edu/.bin/fbidq.html?p130CAS), [CG7033](http://flybase.bio.indiana.edu/.bin/fbidq.html?CG7033), [CG13908](http://flybase.bio.indiana.edu/.bin/fbidq.html?CG13908), [CG30007](http://flybase.bio.indiana.edu/.bin/fbidq.html?CG30007), [ewg](http://flybase.bio.indiana.edu/.bin/fbidq.html?ewg), [CG13550](http://flybase.bio.indiana.edu/.bin/fbidq.html?CG13550),[sano](http://flybase.bio.indiana.edu/.bin/fbidq.html?sano), [Cirl](http://flybase.bio.indiana.edu/.bin/fbidq.html?Cirl), [APC4](http://flybase.bio.indiana.edu/.bin/fbidq.html?APC4), [l(2)NC136](http://flybase.bio.indiana.edu/.bin/fbidq.html?l(2)NC136), [Su(var)3-3](http://flybase.bio.indiana.edu/.bin/fbidq.html?Su(var)3-3), [Bili](http://flybase.bio.indiana.edu/.bin/fbidq.html?Bili), [gogo](http://flybase.bio.indiana.edu/.bin/fbidq.html?gogo), [unc-13-4A](http://flybase.bio.indiana.edu/.bin/fbidq.html?unc-13-4A), [Dh44-R1](http://flybase.bio.indiana.edu/.bin/fbidq.html?Dh44-R1), [stet](http://flybase.bio.indiana.edu/.bin/fbidq.html?stet), [GluClalpha](http://flybase.bio.indiana.edu/.bin/fbidq.html?GluClalpha), [Sema-1a](http://flybase.bio.indiana.edu/.bin/fbidq.html?Sema-1a), [dpr15](http://flybase.bio.indiana.edu/.bin/fbidq.html?dpr15), [Eh](http://flybase.bio.indiana.edu/.bin/fbidq.html?Eh), [Hs3st-B](http://flybase.bio.indiana.edu/.bin/fbidq.html?Hs3st-B), [Sos](http://flybase.bio.indiana.edu/.bin/fbidq.html?Sos), [Fas2](http://flybase.bio.indiana.edu/.bin/fbidq.html?Fas2), [Klp61F](http://flybase.bio.indiana.edu/.bin/fbidq.html?Klp61F), [eya](http://flybase.bio.indiana.edu/.bin/fbidq.html?eya), [mAcR-60C](http://flybase.bio.indiana.edu/.bin/fbidq.html?mAcR-60C), [CG8557](http://flybase.bio.indiana.edu/.bin/fbidq.html?CG8557), [beat-Ia](http://flybase.bio.indiana.edu/.bin/fbidq.html?beat-Ia), [Vsx2](http://flybase.bio.indiana.edu/.bin/fbidq.html?Vsx2), [kat-60L1](http://flybase.bio.indiana.edu/.bin/fbidq.html?kat-60L1), [mtg](http://flybase.bio.indiana.edu/.bin/fbidq.html?mtg), [ft](http://flybase.bio.indiana.edu/.bin/fbidq.html?ft), [Crz](http://flybase.bio.indiana.edu/.bin/fbidq.html?Crz), [CG12004](http://flybase.bio.indiana.edu/.bin/fbidq.html?CG12004), [CG32392](http://flybase.bio.indiana.edu/.bin/fbidq.html?CG32392), [l(2)k16918](http://flybase.bio.indiana.edu/.bin/fbidq.html?l(2)k16918),[Takr86C](http://flybase.bio.indiana.edu/.bin/fbidq.html?Takr86C), [sws](http://flybase.bio.indiana.edu/.bin/fbidq.html?sws), [Dll](http://flybase.bio.indiana.edu/.bin/fbidq.html?Dll), [disco](http://flybase.bio.indiana.edu/.bin/fbidq.html?disco), [DopR](http://flybase.bio.indiana.edu/.bin/fbidq.html?DopR), [D12](http://flybase.bio.indiana.edu/.bin/fbidq.html?D12), [mew](http://flybase.bio.indiana.edu/.bin/fbidq.html?mew), [didum](http://flybase.bio.indiana.edu/.bin/fbidq.html?didum), [Lis-1](http://flybase.bio.indiana.edu/.bin/fbidq.html?Lis-1), [bmm](http://flybase.bio.indiana.edu/.bin/fbidq.html?bmm), [ems](http://flybase.bio.indiana.edu/.bin/fbidq.html?ems), [CG16896](http://flybase.bio.indiana.edu/.bin/fbidq.html?CG16896), [eys](http://flybase.bio.indiana.edu/.bin/fbidq.html?eys), [tup](http://flybase.bio.indiana.edu/.bin/fbidq.html?tup), [Rab3](http://flybase.bio.indiana.edu/.bin/fbidq.html?Rab3), [CG6191](http://flybase.bio.indiana.edu/.bin/fbidq.html?CG6191), [alpha-catenin-related](http://flybase.bio.indiana.edu/.bin/fbidq.html?alpha-catenin-related), [trio](http://flybase.bio.indiana.edu/.bin/fbidq.html?trio), [pdm2](http://flybase.bio.indiana.edu/.bin/fbidq.html?pdm2), [RabX4](http://flybase.bio.indiana.edu/.bin/fbidq.html?RabX4), [Lkr](http://flybase.bio.indiana.edu/.bin/fbidq.html?Lkr), [spel1](http://flybase.bio.indiana.edu/.bin/fbidq.html?spel1), [CG17922](http://flybase.bio.indiana.edu/.bin/fbidq.html?CG17922), [CG31665](http://flybase.bio.indiana.edu/.bin/fbidq.html?CG31665), [Gad1](http://flybase.bio.indiana.edu/.bin/fbidq.html?Gad1), [CG15828](http://flybase.bio.indiana.edu/.bin/fbidq.html?CG15828), [CG7918](http://flybase.bio.indiana.edu/.bin/fbidq.html?CG7918), [Oseg6](http://flybase.bio.indiana.edu/.bin/fbidq.html?Oseg6), [nAcRalpha-80B](http://flybase.bio.indiana.edu/.bin/fbidq.html?nAcRalpha-80B),[CaMKII](http://flybase.bio.indiana.edu/.bin/fbidq.html?CaMKII), [DopR2](http://flybase.bio.indiana.edu/.bin/fbidq.html?DopR2), [l(3)psg2](http://flybase.bio.indiana.edu/.bin/fbidq.html?l(3)psg2), [nerfin-1](http://flybase.bio.indiana.edu/.bin/fbidq.html?nerfin-1), [pigs](http://flybase.bio.indiana.edu/.bin/fbidq.html?pigs), [5-HT7](http://flybase.bio.indiana.edu/.bin/fbidq.html?5-HT7), [Fancd2](http://flybase.bio.indiana.edu/.bin/fbidq.html?Fancd2), [Lgr3](http://flybase.bio.indiana.edu/.bin/fbidq.html?Lgr3), [polo](http://flybase.bio.indiana.edu/.bin/fbidq.html?polo), [chinmo](http://flybase.bio.indiana.edu/.bin/fbidq.html?chinmo), [Syn1](http://flybase.bio.indiana.edu/.bin/fbidq.html?Syn1), [mei-9](http://flybase.bio.indiana.edu/.bin/fbidq.html?mei-9), [Ac13E](http://flybase.bio.indiana.edu/.bin/fbidq.html?Ac13E), [Ssdp](http://flybase.bio.indiana.edu/.bin/fbidq.html?Ssdp), [CG8155](http://flybase.bio.indiana.edu/.bin/fbidq.html?CG8155), [CG10019](http://flybase.bio.indiana.edu/.bin/fbidq.html?CG10019), [fend](http://flybase.bio.indiana.edu/.bin/fbidq.html?fend), [dalao](http://flybase.bio.indiana.edu/.bin/fbidq.html?dalao), [cngl](http://flybase.bio.indiana.edu/.bin/fbidq.html?cngl), [wapl](http://flybase.bio.indiana.edu/.bin/fbidq.html?wapl), [sr](http://flybase.bio.indiana.edu/.bin/fbidq.html?sr), [Lim1](http://flybase.bio.indiana.edu/.bin/fbidq.html?Lim1), [nkd](http://flybase.bio.indiana.edu/.bin/fbidq.html?nkd), [RhoGEF3](http://flybase.bio.indiana.edu/.bin/fbidq.html?RhoGEF3), [nrv3](http://flybase.bio.indiana.edu/.bin/fbidq.html?nrv3), [danr](http://flybase.bio.indiana.edu/.bin/fbidq.html?danr), [Dsk](http://flybase.bio.indiana.edu/.bin/fbidq.html?Dsk), [RhoGEF4](http://flybase.bio.indiana.edu/.bin/fbidq.html?RhoGEF4), [vvl](http://flybase.bio.indiana.edu/.bin/fbidq.html?vvl), [Unc-76](http://flybase.bio.indiana.edu/.bin/fbidq.html?Unc-76), [betaInt-nu](http://flybase.bio.indiana.edu/.bin/fbidq.html?betaInt-nu), [sgg](http://flybase.bio.indiana.edu/.bin/fbidq.html?sgg), [Brd](http://flybase.bio.indiana.edu/.bin/fbidq.html?Brd),[mnb](http://flybase.bio.indiana.edu/.bin/fbidq.html?mnb), [Eip93F](http://flybase.bio.indiana.edu/.bin/fbidq.html?Eip93F), [PICK1](http://flybase.bio.indiana.edu/.bin/fbidq.html?PICK1), [Nmdar1](http://flybase.bio.indiana.edu/.bin/fbidq.html?Nmdar1), [His4r](http://flybase.bio.indiana.edu/.bin/fbidq.html?His4r), [gcm](http://flybase.bio.indiana.edu/.bin/fbidq.html?gcm), [mus210](http://flybase.bio.indiana.edu/.bin/fbidq.html?mus210), [dpr10](http://flybase.bio.indiana.edu/.bin/fbidq.html?dpr10), [Suv4-20](http://flybase.bio.indiana.edu/.bin/fbidq.html?Suv4-20), [Ccn](http://flybase.bio.indiana.edu/.bin/fbidq.html?Ccn), [Hsp67Bc](http://flybase.bio.indiana.edu/.bin/fbidq.html?Hsp67Bc), [ase](http://flybase.bio.indiana.edu/.bin/fbidq.html?ase), [Lerp](http://flybase.bio.indiana.edu/.bin/fbidq.html?Lerp), [CG8771](http://flybase.bio.indiana.edu/.bin/fbidq.html?CG8771), [elav](http://flybase.bio.indiana.edu/.bin/fbidq.html?elav), [MESK2](http://flybase.bio.indiana.edu/.bin/fbidq.html?MESK2), [Poxn](http://flybase.bio.indiana.edu/.bin/fbidq.html?Poxn), [sals](http://flybase.bio.indiana.edu/.bin/fbidq.html?sals), [Dat](http://flybase.bio.indiana.edu/.bin/fbidq.html?Dat), [Rat1](http://flybase.bio.indiana.edu/.bin/fbidq.html?Rat1), [dpr3](http://flybase.bio.indiana.edu/.bin/fbidq.html?dpr3), [Task6](http://flybase.bio.indiana.edu/.bin/fbidq.html?Task6), [CG18273](http://flybase.bio.indiana.edu/.bin/fbidq.html?CG18273), [eIF4G2](http://flybase.bio.indiana.edu/.bin/fbidq.html?eIF4G2), [fred](http://flybase.bio.indiana.edu/.bin/fbidq.html?fred), [Ca-beta](http://flybase.bio.indiana.edu/.bin/fbidq.html?Ca-beta), [ush](http://flybase.bio.indiana.edu/.bin/fbidq.html?ush), [Fak56D](http://flybase.bio.indiana.edu/.bin/fbidq.html?Fak56D), [CG16778](http://flybase.bio.indiana.edu/.bin/fbidq.html?CG16778), [Task7](http://flybase.bio.indiana.edu/.bin/fbidq.html?Task7), [Kap3](http://flybase.bio.indiana.edu/.bin/fbidq.html?Kap3), [ko](http://flybase.bio.indiana.edu/.bin/fbidq.html?ko),[Takr99D](http://flybase.bio.indiana.edu/.bin/fbidq.html?Takr99D), [dpr17](http://flybase.bio.indiana.edu/.bin/fbidq.html?dpr17), [Teh4](http://flybase.bio.indiana.edu/.bin/fbidq.html?Teh4), [Eip71CD](http://flybase.bio.indiana.edu/.bin/fbidq.html?Eip71CD), [Alh](http://flybase.bio.indiana.edu/.bin/fbidq.html?Alh), [Hex-A](http://flybase.bio.indiana.edu/.bin/fbidq.html?Hex-A), [egg](http://flybase.bio.indiana.edu/.bin/fbidq.html?egg), [CG13692](http://flybase.bio.indiana.edu/.bin/fbidq.html?CG13692), [CG5337](http://flybase.bio.indiana.edu/.bin/fbidq.html?CG5337), [rtet](http://flybase.bio.indiana.edu/.bin/fbidq.html?rtet), [CG11750](http://flybase.bio.indiana.edu/.bin/fbidq.html?CG11750), [CG12424](http://flybase.bio.indiana.edu/.bin/fbidq.html?CG12424), [Sp1](http://flybase.bio.indiana.edu/.bin/fbidq.html?Sp1), [GlcAT-P](http://flybase.bio.indiana.edu/.bin/fbidq.html?GlcAT-P), [CG3967](http://flybase.bio.indiana.edu/.bin/fbidq.html?CG3967), [Wnt5](http://flybase.bio.indiana.edu/.bin/fbidq.html?Wnt5), [SF2](http://flybase.bio.indiana.edu/.bin/fbidq.html?SF2), [Sln](http://flybase.bio.indiana.edu/.bin/fbidq.html?Sln), [nAcRbeta-64B](http://flybase.bio.indiana.edu/.bin/fbidq.html?nAcRbeta-64B), [CG11897](http://flybase.bio.indiana.edu/.bin/fbidq.html?CG11897), [fus](http://flybase.bio.indiana.edu/.bin/fbidq.html?fus), [sif](http://flybase.bio.indiana.edu/.bin/fbidq.html?sif), [Tbh](http://flybase.bio.indiana.edu/.bin/fbidq.html?Tbh), [Aac11](http://flybase.bio.indiana.edu/.bin/fbidq.html?Aac11), [MTA1-like](http://flybase.bio.indiana.edu/.bin/fbidq.html?MTA1-like), [Sas-4](http://flybase.bio.indiana.edu/.bin/fbidq.html?Sas-4), [cal1](http://flybase.bio.indiana.edu/.bin/fbidq.html?cal1), [ssh](http://flybase.bio.indiana.edu/.bin/fbidq.html?ssh), [tadr](http://flybase.bio.indiana.edu/.bin/fbidq.html?tadr), [CG42541](http://flybase.bio.indiana.edu/.bin/fbidq.html?CG42541), [PIP5K59B](http://flybase.bio.indiana.edu/.bin/fbidq.html?PIP5K59B),[mle](http://flybase.bio.indiana.edu/.bin/fbidq.html?mle), [Hr51](http://flybase.bio.indiana.edu/.bin/fbidq.html?Hr51), [lap](http://flybase.bio.indiana.edu/.bin/fbidq.html?lap), [CG11155](http://flybase.bio.indiana.edu/.bin/fbidq.html?CG11155), [wg](http://flybase.bio.indiana.edu/.bin/fbidq.html?wg), [CG31030](http://flybase.bio.indiana.edu/.bin/fbidq.html?CG31030), [CG11077](http://flybase.bio.indiana.edu/.bin/fbidq.html?CG11077), [fkh](http://flybase.bio.indiana.edu/.bin/fbidq.html?fkh), [Spps](http://flybase.bio.indiana.edu/.bin/fbidq.html?Spps), [Rhp](http://flybase.bio.indiana.edu/.bin/fbidq.html?Rhp), [RpL28](http://flybase.bio.indiana.edu/.bin/fbidq.html?RpL28), [CG32000](http://flybase.bio.indiana.edu/.bin/fbidq.html?CG32000), [klg](http://flybase.bio.indiana.edu/.bin/fbidq.html?klg), [en](http://flybase.bio.indiana.edu/.bin/fbidq.html?en), [sfl](http://flybase.bio.indiana.edu/.bin/fbidq.html?sfl), [CG13229](http://flybase.bio.indiana.edu/.bin/fbidq.html?CG13229), [Sip1](http://flybase.bio.indiana.edu/.bin/fbidq.html?Sip1), [Pde11](http://flybase.bio.indiana.edu/.bin/fbidq.html?Pde11), [SerT](http://flybase.bio.indiana.edu/.bin/fbidq.html?SerT), [Utx](http://flybase.bio.indiana.edu/.bin/fbidq.html?Utx), [bt](http://flybase.bio.indiana.edu/.bin/fbidq.html?bt), [Rnp4F](http://flybase.bio.indiana.edu/.bin/fbidq.html?Rnp4F), [Ct](http://flybase.bio.indiana.edu/.bin/fbidq.html?Ct), [fz2](http://flybase.bio.indiana.edu/.bin/fbidq.html?fz2), [CG8407](http://flybase.bio.indiana.edu/.bin/fbidq.html?CG8407), [CG14375](http://flybase.bio.indiana.edu/.bin/fbidq.html?CG14375), [Ocho](http://flybase.bio.indiana.edu/.bin/fbidq.html?Ocho), [numb](http://flybase.bio.indiana.edu/.bin/fbidq.html?numb), [Nnf1b](http://flybase.bio.indiana.edu/.bin/fbidq.html?Nnf1b), [CG42256](http://flybase.bio.indiana.edu/.bin/fbidq.html?CG42256), [nAcRalpha-96Ab](http://flybase.bio.indiana.edu/.bin/fbidq.html?nAcRalpha-96Ab), [mus101](http://flybase.bio.indiana.edu/.bin/fbidq.html?mus101),[CG17734](http://flybase.bio.indiana.edu/.bin/fbidq.html?CG17734), [stj](http://flybase.bio.indiana.edu/.bin/fbidq.html?stj), [Sxl](http://flybase.bio.indiana.edu/.bin/fbidq.html?Sxl), [CG33007](http://flybase.bio.indiana.edu/.bin/fbidq.html?CG33007), [JIL-1](http://flybase.bio.indiana.edu/.bin/fbidq.html?JIL-1), [Nf1](http://flybase.bio.indiana.edu/.bin/fbidq.html?Nf1), [Drep-3](http://flybase.bio.indiana.edu/.bin/fbidq.html?Drep-3), [sns](http://flybase.bio.indiana.edu/.bin/fbidq.html?sns), [AR-2](http://flybase.bio.indiana.edu/.bin/fbidq.html?AR-2), [sol](http://flybase.bio.indiana.edu/.bin/fbidq.html?sol), [Topors](http://flybase.bio.indiana.edu/.bin/fbidq.html?Topors), [tok](http://flybase.bio.indiana.edu/.bin/fbidq.html?tok), [bi](http://flybase.bio.indiana.edu/.bin/fbidq.html?bi), [nonA](http://flybase.bio.indiana.edu/.bin/fbidq.html?nonA), [Sytalpha](http://flybase.bio.indiana.edu/.bin/fbidq.html?Sytalpha), [Ptp99A](http://flybase.bio.indiana.edu/.bin/fbidq.html?Ptp99A), [CG31961](http://flybase.bio.indiana.edu/.bin/fbidq.html?CG31961), [Tango10](http://flybase.bio.indiana.edu/.bin/fbidq.html?Tango10), [Oseg4](http://flybase.bio.indiana.edu/.bin/fbidq.html?Oseg4), [NC2alpha](http://flybase.bio.indiana.edu/.bin/fbidq.html?NC2alpha), [blue](http://flybase.bio.indiana.edu/.bin/fbidq.html?blue), [mod(mdg4)](http://flybase.bio.indiana.edu/.bin/fbidq.html?mod(mdg4)), [bab1](http://flybase.bio.indiana.edu/.bin/fbidq.html?bab1), [Taf12L](http://flybase.bio.indiana.edu/.bin/fbidq.html?Taf12L), [Galpha49B](http://flybase.bio.indiana.edu/.bin/fbidq.html?Galpha49B), [Snoo](http://flybase.bio.indiana.edu/.bin/fbidq.html?Snoo), [rut](http://flybase.bio.indiana.edu/.bin/fbidq.html?rut), [RhoGAP100F](http://flybase.bio.indiana.edu/.bin/fbidq.html?RhoGAP100F), [nub](http://flybase.bio.indiana.edu/.bin/fbidq.html?nub), [uif](http://flybase.bio.indiana.edu/.bin/fbidq.html?uif), [btd](http://flybase.bio.indiana.edu/.bin/fbidq.html?btd), [fd68A](http://flybase.bio.indiana.edu/.bin/fbidq.html?fd68A),[Mmp2](http://flybase.bio.indiana.edu/.bin/fbidq.html?Mmp2), [amon](http://flybase.bio.indiana.edu/.bin/fbidq.html?amon), [B-H1](http://flybase.bio.indiana.edu/.bin/fbidq.html?B-H1), [opa1-like](http://flybase.bio.indiana.edu/.bin/fbidq.html?opa1-like), [Nplp1](http://flybase.bio.indiana.edu/.bin/fbidq.html?Nplp1), [Nipped-A](http://flybase.bio.indiana.edu/.bin/fbidq.html?Nipped-A), [DAT](http://flybase.bio.indiana.edu/.bin/fbidq.html?DAT), [pim](http://flybase.bio.indiana.edu/.bin/fbidq.html?pim), [Vha68-1](http://flybase.bio.indiana.edu/.bin/fbidq.html?Vha68-1), [CG32206](http://flybase.bio.indiana.edu/.bin/fbidq.html?CG32206), [CG10804](http://flybase.bio.indiana.edu/.bin/fbidq.html?CG10804), [Spf45](http://flybase.bio.indiana.edu/.bin/fbidq.html?Spf45), [fd102C](http://flybase.bio.indiana.edu/.bin/fbidq.html?fd102C), [lid](http://flybase.bio.indiana.edu/.bin/fbidq.html?lid), [hth](http://flybase.bio.indiana.edu/.bin/fbidq.html?hth), [pzg](http://flybase.bio.indiana.edu/.bin/fbidq.html?pzg), [14-3-3zeta](http://flybase.bio.indiana.edu/.bin/fbidq.html?14-3-3zeta), [lea](http://flybase.bio.indiana.edu/.bin/fbidq.html?lea), [shakB](http://flybase.bio.indiana.edu/.bin/fbidq.html?shakB), [Glu-RI](http://flybase.bio.indiana.edu/.bin/fbidq.html?Glu-RI), [Hey](http://flybase.bio.indiana.edu/.bin/fbidq.html?Hey), [plexB](http://flybase.bio.indiana.edu/.bin/fbidq.html?plexB), [AlstR](http://flybase.bio.indiana.edu/.bin/fbidq.html?AlstR), [loqs](http://flybase.bio.indiana.edu/.bin/fbidq.html?loqs), [Klp64D](http://flybase.bio.indiana.edu/.bin/fbidq.html?Klp64D), [icln](http://flybase.bio.indiana.edu/.bin/fbidq.html?icln), [mars](http://flybase.bio.indiana.edu/.bin/fbidq.html?mars), [CG9095](http://flybase.bio.indiana.edu/.bin/fbidq.html?CG9095), [CG32226](http://flybase.bio.indiana.edu/.bin/fbidq.html?CG32226), [IM10](http://flybase.bio.indiana.edu/.bin/fbidq.html?IM10), [beat-Vc](http://flybase.bio.indiana.edu/.bin/fbidq.html?beat-Vc), [neur](http://flybase.bio.indiana.edu/.bin/fbidq.html?neur),[CG33543](http://flybase.bio.indiana.edu/.bin/fbidq.html?CG33543), [CycE](http://flybase.bio.indiana.edu/.bin/fbidq.html?CycE), [Brf](http://flybase.bio.indiana.edu/.bin/fbidq.html?Brf), [Cep97](http://flybase.bio.indiana.edu/.bin/fbidq.html?Cep97), [escl](http://flybase.bio.indiana.edu/.bin/fbidq.html?escl), [CG32149](http://flybase.bio.indiana.edu/.bin/fbidq.html?CG32149), [Vdup1](http://flybase.bio.indiana.edu/.bin/fbidq.html?Vdup1), [mr](http://flybase.bio.indiana.edu/.bin/fbidq.html?mr), [orb2](http://flybase.bio.indiana.edu/.bin/fbidq.html?orb2), [ImpE2](http://flybase.bio.indiana.edu/.bin/fbidq.html?ImpE2), [nAcRbeta-96A](http://flybase.bio.indiana.edu/.bin/fbidq.html?nAcRbeta-96A), [synaptogyrin](http://flybase.bio.indiana.edu/.bin/fbidq.html?synaptogyrin), [sim](http://flybase.bio.indiana.edu/.bin/fbidq.html?sim), [gro](http://flybase.bio.indiana.edu/.bin/fbidq.html?gro), [Rya-r44F](http://flybase.bio.indiana.edu/.bin/fbidq.html?Rya-r44F), [scyl](http://flybase.bio.indiana.edu/.bin/fbidq.html?scyl), [hkl](http://flybase.bio.indiana.edu/.bin/fbidq.html?hkl), [Rpd3](http://flybase.bio.indiana.edu/.bin/fbidq.html?Rpd3), [CG14367](http://flybase.bio.indiana.edu/.bin/fbidq.html?CG14367), [tyf](http://flybase.bio.indiana.edu/.bin/fbidq.html?tyf), [CG10107](http://flybase.bio.indiana.edu/.bin/fbidq.html?CG10107), [aret](http://flybase.bio.indiana.edu/.bin/fbidq.html?aret), [HLHm7](http://flybase.bio.indiana.edu/.bin/fbidq.html?HLHm7), [tow](http://flybase.bio.indiana.edu/.bin/fbidq.html?tow), [CG15609](http://flybase.bio.indiana.edu/.bin/fbidq.html?CG15609), [l(1)G0148](http://flybase.bio.indiana.edu/.bin/fbidq.html?l(1)G0148), [sei](http://flybase.bio.indiana.edu/.bin/fbidq.html?sei), [Lcch3](http://flybase.bio.indiana.edu/.bin/fbidq.html?Lcch3), [CG12187](http://flybase.bio.indiana.edu/.bin/fbidq.html?CG12187), [Pten](http://flybase.bio.indiana.edu/.bin/fbidq.html?Pten), [wda](http://flybase.bio.indiana.edu/.bin/fbidq.html?wda),[unc-13](http://flybase.bio.indiana.edu/.bin/fbidq.html?unc-13), [dpr8](http://flybase.bio.indiana.edu/.bin/fbidq.html?dpr8), [npf](http://flybase.bio.indiana.edu/.bin/fbidq.html?npf), [mira](http://flybase.bio.indiana.edu/.bin/fbidq.html?mira), [Myo10A](http://flybase.bio.indiana.edu/.bin/fbidq.html?Myo10A), [CG12194](http://flybase.bio.indiana.edu/.bin/fbidq.html?CG12194), [Sug](http://flybase.bio.indiana.edu/.bin/fbidq.html?Sug), [Spindly](http://flybase.bio.indiana.edu/.bin/fbidq.html?Spindly), [Dip3](http://flybase.bio.indiana.edu/.bin/fbidq.html?Dip3), [Drep-2](http://flybase.bio.indiana.edu/.bin/fbidq.html?Drep-2), [oa2](http://flybase.bio.indiana.edu/.bin/fbidq.html?oa2), [elk](http://flybase.bio.indiana.edu/.bin/fbidq.html?elk), [RpS11](http://flybase.bio.indiana.edu/.bin/fbidq.html?RpS11), [phl](http://flybase.bio.indiana.edu/.bin/fbidq.html?phl), [Rab26](http://flybase.bio.indiana.edu/.bin/fbidq.html?Rab26), [Pde6](http://flybase.bio.indiana.edu/.bin/fbidq.html?Pde6), [sNPF](http://flybase.bio.indiana.edu/.bin/fbidq.html?sNPF), [CG4893](http://flybase.bio.indiana.edu/.bin/fbidq.html?CG4893), [hang](http://flybase.bio.indiana.edu/.bin/fbidq.html?hang), [plx](http://flybase.bio.indiana.edu/.bin/fbidq.html?plx), [CG9098](http://flybase.bio.indiana.edu/.bin/fbidq.html?CG9098), [CG8468](http://flybase.bio.indiana.edu/.bin/fbidq.html?CG8468), [CG8108](http://flybase.bio.indiana.edu/.bin/fbidq.html?CG8108), [IFa](http://flybase.bio.indiana.edu/.bin/fbidq.html?IFa), [Alk](http://flybase.bio.indiana.edu/.bin/fbidq.html?Alk), [CG7708](http://flybase.bio.indiana.edu/.bin/fbidq.html?CG7708), [esn](http://flybase.bio.indiana.edu/.bin/fbidq.html?esn), [Vang](http://flybase.bio.indiana.edu/.bin/fbidq.html?Vang), [Sin3A](http://flybase.bio.indiana.edu/.bin/fbidq.html?Sin3A), [pot](http://flybase.bio.indiana.edu/.bin/fbidq.html?pot), [Pde1c](http://flybase.bio.indiana.edu/.bin/fbidq.html?Pde1c), [casp](http://flybase.bio.indiana.edu/.bin/fbidq.html?casp), [Smox](http://flybase.bio.indiana.edu/.bin/fbidq.html?Smox), [CG31475](http://flybase.bio.indiana.edu/.bin/fbidq.html?CG31475),[Shaw](http://flybase.bio.indiana.edu/.bin/fbidq.html?Shaw), [mei-S332](http://flybase.bio.indiana.edu/.bin/fbidq.html?mei-S332), [Plc21C](http://flybase.bio.indiana.edu/.bin/fbidq.html?Plc21C), [srpk79D](http://flybase.bio.indiana.edu/.bin/fbidq.html?srpk79D), [CG6208](http://flybase.bio.indiana.edu/.bin/fbidq.html?CG6208), [Antp](http://flybase.bio.indiana.edu/.bin/fbidq.html?Antp), [CAP-D2](http://flybase.bio.indiana.edu/.bin/fbidq.html?CAP-D2), [GRHRII](http://flybase.bio.indiana.edu/.bin/fbidq.html?GRHRII), [glec](http://flybase.bio.indiana.edu/.bin/fbidq.html?glec), [Rdl](http://flybase.bio.indiana.edu/.bin/fbidq.html?Rdl), [l(3)neo38](http://flybase.bio.indiana.edu/.bin/fbidq.html?l(3)neo38), [CG10362](http://flybase.bio.indiana.edu/.bin/fbidq.html?CG10362), [Ast-C](http://flybase.bio.indiana.edu/.bin/fbidq.html?Ast-C), [Gbeta5](http://flybase.bio.indiana.edu/.bin/fbidq.html?Gbeta5), [cindr](http://flybase.bio.indiana.edu/.bin/fbidq.html?cindr), [comm3](http://flybase.bio.indiana.edu/.bin/fbidq.html?comm3), [beat-VI](http://flybase.bio.indiana.edu/.bin/fbidq.html?beat-VI), [Nhe3](http://flybase.bio.indiana.edu/.bin/fbidq.html?Nhe3), [Ptx1](http://flybase.bio.indiana.edu/.bin/fbidq.html?Ptx1), [CG3556](http://flybase.bio.indiana.edu/.bin/fbidq.html?CG3556), [CG11071](http://flybase.bio.indiana.edu/.bin/fbidq.html?CG11071), [CG6114](http://flybase.bio.indiana.edu/.bin/fbidq.html?CG6114), [Shal](http://flybase.bio.indiana.edu/.bin/fbidq.html?Shal), [pigeon](http://flybase.bio.indiana.edu/.bin/fbidq.html?pigeon), [hug](http://flybase.bio.indiana.edu/.bin/fbidq.html?hug), [Gpo-1](http://flybase.bio.indiana.edu/.bin/fbidq.html?Gpo-1), [para](http://flybase.bio.indiana.edu/.bin/fbidq.html?para), [tsh](http://flybase.bio.indiana.edu/.bin/fbidq.html?tsh), [sm](http://flybase.bio.indiana.edu/.bin/fbidq.html?sm), [Syn2](http://flybase.bio.indiana.edu/.bin/fbidq.html?Syn2), [eas](http://flybase.bio.indiana.edu/.bin/fbidq.html?eas),[e(y)3](http://flybase.bio.indiana.edu/.bin/fbidq.html?e(y)3), [Sry-alpha](http://flybase.bio.indiana.edu/.bin/fbidq.html?Sry-alpha), [CRMP](http://flybase.bio.indiana.edu/.bin/fbidq.html?CRMP), [Cdk5](http://flybase.bio.indiana.edu/.bin/fbidq.html?Cdk5), [Cep135](http://flybase.bio.indiana.edu/.bin/fbidq.html?Cep135), [oc](http://flybase.bio.indiana.edu/.bin/fbidq.html?oc), [CG4641](http://flybase.bio.indiana.edu/.bin/fbidq.html?CG4641), [spt4](http://flybase.bio.indiana.edu/.bin/fbidq.html?spt4), [CG3964](http://flybase.bio.indiana.edu/.bin/fbidq.html?CG3964), [ey](http://flybase.bio.indiana.edu/.bin/fbidq.html?ey), [Mip](http://flybase.bio.indiana.edu/.bin/fbidq.html?Mip), [baz](http://flybase.bio.indiana.edu/.bin/fbidq.html?baz), [Oamb](http://flybase.bio.indiana.edu/.bin/fbidq.html?Oamb), [SK](http://flybase.bio.indiana.edu/.bin/fbidq.html?SK), [msd5](http://flybase.bio.indiana.edu/.bin/fbidq.html?msd5), [CG34381](http://flybase.bio.indiana.edu/.bin/fbidq.html?CG34381), [rst](http://flybase.bio.indiana.edu/.bin/fbidq.html?rst), [CG6398](http://flybase.bio.indiana.edu/.bin/fbidq.html?CG6398), [Bgb](http://flybase.bio.indiana.edu/.bin/fbidq.html?Bgb), [l(1)sc](http://flybase.bio.indiana.edu/.bin/fbidq.html?l(1)sc), [comm](http://flybase.bio.indiana.edu/.bin/fbidq.html?comm), [grh](http://flybase.bio.indiana.edu/.bin/fbidq.html?grh), [Vsx1](http://flybase.bio.indiana.edu/.bin/fbidq.html?Vsx1), [Cdk5alpha](http://flybase.bio.indiana.edu/.bin/fbidq.html?Cdk5alpha), [yemalpha](http://flybase.bio.indiana.edu/.bin/fbidq.html?yemalpha), [Vmat](http://flybase.bio.indiana.edu/.bin/fbidq.html?Vmat), [qtc](http://flybase.bio.indiana.edu/.bin/fbidq.html?qtc), [Cha](http://flybase.bio.indiana.edu/.bin/fbidq.html?Cha), [crol](http://flybase.bio.indiana.edu/.bin/fbidq.html?crol), [E(Pc)](http://flybase.bio.indiana.edu/.bin/fbidq.html?E(Pc)), [retn](http://flybase.bio.indiana.edu/.bin/fbidq.html?retn), [Sap130](http://flybase.bio.indiana.edu/.bin/fbidq.html?Sap130), [Ac3](http://flybase.bio.indiana.edu/.bin/fbidq.html?Ac3), [rl](http://flybase.bio.indiana.edu/.bin/fbidq.html?rl), [Grip163](http://flybase.bio.indiana.edu/.bin/fbidq.html?Grip163),[Syt1](http://flybase.bio.indiana.edu/.bin/fbidq.html?Syt1), [CG17760](http://flybase.bio.indiana.edu/.bin/fbidq.html?CG17760), [Kdm4B](http://flybase.bio.indiana.edu/.bin/fbidq.html?Kdm4B), [Nup153](http://flybase.bio.indiana.edu/.bin/fbidq.html?Nup153), [CG32683](http://flybase.bio.indiana.edu/.bin/fbidq.html?CG32683), [tey](http://flybase.bio.indiana.edu/.bin/fbidq.html?tey), [CG33960](http://flybase.bio.indiana.edu/.bin/fbidq.html?CG33960), [dpr18](http://flybase.bio.indiana.edu/.bin/fbidq.html?dpr18), [CG15765](http://flybase.bio.indiana.edu/.bin/fbidq.html?CG15765), [unk](http://flybase.bio.indiana.edu/.bin/fbidq.html?unk), [CG1962](http://flybase.bio.indiana.edu/.bin/fbidq.html?CG1962), [dnr1](http://flybase.bio.indiana.edu/.bin/fbidq.html?dnr1), [Pabp2](http://flybase.bio.indiana.edu/.bin/fbidq.html?Pabp2), [Arp8](http://flybase.bio.indiana.edu/.bin/fbidq.html?Arp8), [acj6](http://flybase.bio.indiana.edu/.bin/fbidq.html?acj6), [nAcRalpha-34E](http://flybase.bio.indiana.edu/.bin/fbidq.html?nAcRalpha-34E), [ana3](http://flybase.bio.indiana.edu/.bin/fbidq.html?ana3), [caup](http://flybase.bio.indiana.edu/.bin/fbidq.html?caup), [Cbp80](http://flybase.bio.indiana.edu/.bin/fbidq.html?Cbp80), [vimar](http://flybase.bio.indiana.edu/.bin/fbidq.html?vimar), [Ndae1](http://flybase.bio.indiana.edu/.bin/fbidq.html?Ndae1), [Dg](http://flybase.bio.indiana.edu/.bin/fbidq.html?Dg), [Trim9](http://flybase.bio.indiana.edu/.bin/fbidq.html?Trim9), [nAcRalpha-96Aa](http://flybase.bio.indiana.edu/.bin/fbidq.html?nAcRalpha-96Aa), [GABA-B-R3](http://flybase.bio.indiana.edu/.bin/fbidq.html?GABA-B-R3), [Evi5](http://flybase.bio.indiana.edu/.bin/fbidq.html?Evi5), [CG34127](http://flybase.bio.indiana.edu/.bin/fbidq.html?CG34127), [br](http://flybase.bio.indiana.edu/.bin/fbidq.html?br),[Gycalpha99B](http://flybase.bio.indiana.edu/.bin/fbidq.html?Gycalpha99B), [CG7154](http://flybase.bio.indiana.edu/.bin/fbidq.html?CG7154), [neo](http://flybase.bio.indiana.edu/.bin/fbidq.html?neo), [robo3](http://flybase.bio.indiana.edu/.bin/fbidq.html?robo3), [CG11376](http://flybase.bio.indiana.edu/.bin/fbidq.html?CG11376), [Fim](http://flybase.bio.indiana.edu/.bin/fbidq.html?Fim), [CG6126](http://flybase.bio.indiana.edu/.bin/fbidq.html?CG6126), [PNUTS](http://flybase.bio.indiana.edu/.bin/fbidq.html?PNUTS), [lola](http://flybase.bio.indiana.edu/.bin/fbidq.html?lola), [unc-104](http://flybase.bio.indiana.edu/.bin/fbidq.html?unc-104), [VAChT](http://flybase.bio.indiana.edu/.bin/fbidq.html?VAChT), [CG7627](http://flybase.bio.indiana.edu/.bin/fbidq.html?CG7627), [Pcl](http://flybase.bio.indiana.edu/.bin/fbidq.html?Pcl), [CG15312](http://flybase.bio.indiana.edu/.bin/fbidq.html?CG15312), [Spc105R](http://flybase.bio.indiana.edu/.bin/fbidq.html?Spc105R), [CG31158](http://flybase.bio.indiana.edu/.bin/fbidq.html?CG31158), [Awh](http://flybase.bio.indiana.edu/.bin/fbidq.html?Awh), [Appl](http://flybase.bio.indiana.edu/.bin/fbidq.html?Appl), [tap](http://flybase.bio.indiana.edu/.bin/fbidq.html?tap), [metro](http://flybase.bio.indiana.edu/.bin/fbidq.html?metro), [erm](http://flybase.bio.indiana.edu/.bin/fbidq.html?erm), [Nrk](http://flybase.bio.indiana.edu/.bin/fbidq.html?Nrk), [dmt](http://flybase.bio.indiana.edu/.bin/fbidq.html?dmt), [CG4328](http://flybase.bio.indiana.edu/.bin/fbidq.html?CG4328), [CG11399](http://flybase.bio.indiana.edu/.bin/fbidq.html?CG11399), [Ilp3](http://flybase.bio.indiana.edu/.bin/fbidq.html?Ilp3), [CG10249](http://flybase.bio.indiana.edu/.bin/fbidq.html?CG10249), [Taf4](http://flybase.bio.indiana.edu/.bin/fbidq.html?Taf4), [Nrg](http://flybase.bio.indiana.edu/.bin/fbidq.html?Nrg), [insc](http://flybase.bio.indiana.edu/.bin/fbidq.html?insc), [CG34384](http://flybase.bio.indiana.edu/.bin/fbidq.html?CG34384),[jeb](http://flybase.bio.indiana.edu/.bin/fbidq.html?jeb), [CG32447](http://flybase.bio.indiana.edu/.bin/fbidq.html?CG32447), [CG31530](http://flybase.bio.indiana.edu/.bin/fbidq.html?CG31530), [Actbeta](http://flybase.bio.indiana.edu/.bin/fbidq.html?Actbeta), [stau](http://flybase.bio.indiana.edu/.bin/fbidq.html?stau), [beat-Ib](http://flybase.bio.indiana.edu/.bin/fbidq.html?beat-Ib), [beat-IIb](http://flybase.bio.indiana.edu/.bin/fbidq.html?beat-IIb), [CG31064](http://flybase.bio.indiana.edu/.bin/fbidq.html?CG31064), [otk](http://flybase.bio.indiana.edu/.bin/fbidq.html?otk), [phyl](http://flybase.bio.indiana.edu/.bin/fbidq.html?phyl), [vg](http://flybase.bio.indiana.edu/.bin/fbidq.html?vg), [fz](http://flybase.bio.indiana.edu/.bin/fbidq.html?fz), [robo](http://flybase.bio.indiana.edu/.bin/fbidq.html?robo), [phr](http://flybase.bio.indiana.edu/.bin/fbidq.html?phr), [bsk](http://flybase.bio.indiana.edu/.bin/fbidq.html?bsk), [brm](http://flybase.bio.indiana.edu/.bin/fbidq.html?brm), [CG42268](http://flybase.bio.indiana.edu/.bin/fbidq.html?CG42268), [E(var)3-9](http://flybase.bio.indiana.edu/.bin/fbidq.html?E(var)3-9), [lft](http://flybase.bio.indiana.edu/.bin/fbidq.html?lft), [nAcRalpha-30D](http://flybase.bio.indiana.edu/.bin/fbidq.html?nAcRalpha-30D), [CG11652](http://flybase.bio.indiana.edu/.bin/fbidq.html?CG11652), [CG13830](http://flybase.bio.indiana.edu/.bin/fbidq.html?CG13830), [CG6006](http://flybase.bio.indiana.edu/.bin/fbidq.html?CG6006), [sno](http://flybase.bio.indiana.edu/.bin/fbidq.html?sno), [hig](http://flybase.bio.indiana.edu/.bin/fbidq.html?hig), [RhoGAPp190](http://flybase.bio.indiana.edu/.bin/fbidq.html?RhoGAPp190), [Cad96Ca](http://flybase.bio.indiana.edu/.bin/fbidq.html?Cad96Ca), [Atac1](http://flybase.bio.indiana.edu/.bin/fbidq.html?Atac1), [Sfmbt](http://flybase.bio.indiana.edu/.bin/fbidq.html?Sfmbt), [ara](http://flybase.bio.indiana.edu/.bin/fbidq.html?ara), [pHCl](http://flybase.bio.indiana.edu/.bin/fbidq.html?pHCl),[mam](http://flybase.bio.indiana.edu/.bin/fbidq.html?mam), [CG30158](http://flybase.bio.indiana.edu/.bin/fbidq.html?CG30158), [pan](http://flybase.bio.indiana.edu/.bin/fbidq.html?pan), [PKD](http://flybase.bio.indiana.edu/.bin/fbidq.html?PKD), [grn](http://flybase.bio.indiana.edu/.bin/fbidq.html?grn), [CG4019](http://flybase.bio.indiana.edu/.bin/fbidq.html?CG4019), [HLHm5](http://flybase.bio.indiana.edu/.bin/fbidq.html?HLHm5), [beat-IIIc](http://flybase.bio.indiana.edu/.bin/fbidq.html?beat-IIIc), [dap](http://flybase.bio.indiana.edu/.bin/fbidq.html?dap), [Bx](http://flybase.bio.indiana.edu/.bin/fbidq.html?Bx), [Mob2](http://flybase.bio.indiana.edu/.bin/fbidq.html?Mob2), [pros](http://flybase.bio.indiana.edu/.bin/fbidq.html?pros), [CG2993](http://flybase.bio.indiana.edu/.bin/fbidq.html?CG2993), [Gen](http://flybase.bio.indiana.edu/.bin/fbidq.html?Gen), [mthl8](http://flybase.bio.indiana.edu/.bin/fbidq.html?mthl8), [CG1463](http://flybase.bio.indiana.edu/.bin/fbidq.html?CG1463), [Ccap](http://flybase.bio.indiana.edu/.bin/fbidq.html?Ccap), [tipE](http://flybase.bio.indiana.edu/.bin/fbidq.html?tipE), [Wnt4](http://flybase.bio.indiana.edu/.bin/fbidq.html?Wnt4), [rictor](http://flybase.bio.indiana.edu/.bin/fbidq.html?rictor), [Mad1](http://flybase.bio.indiana.edu/.bin/fbidq.html?Mad1), [Glut4EF](http://flybase.bio.indiana.edu/.bin/fbidq.html?Glut4EF), [CG30372](http://flybase.bio.indiana.edu/.bin/fbidq.html?CG30372), [CG10188](http://flybase.bio.indiana.edu/.bin/fbidq.html?CG10188), [CG42629](http://flybase.bio.indiana.edu/.bin/fbidq.html?CG42629), [CG31760](http://flybase.bio.indiana.edu/.bin/fbidq.html?CG31760), [Klp54D](http://flybase.bio.indiana.edu/.bin/fbidq.html?Klp54D), [pr-set7](http://flybase.bio.indiana.edu/.bin/fbidq.html?pr-set7), [bchs](http://flybase.bio.indiana.edu/.bin/fbidq.html?bchs), [RpS5b](http://flybase.bio.indiana.edu/.bin/fbidq.html?RpS5b), [king-tubby](http://flybase.bio.indiana.edu/.bin/fbidq.html?king-tubby),[pk](http://flybase.bio.indiana.edu/.bin/fbidq.html?pk), [dsx](http://flybase.bio.indiana.edu/.bin/fbidq.html?dsx), [Dh](http://flybase.bio.indiana.edu/.bin/fbidq.html?Dh), [Scgbeta](http://flybase.bio.indiana.edu/.bin/fbidq.html?Scgbeta), [betaTub85D](http://flybase.bio.indiana.edu/.bin/fbidq.html?betaTub85D), [klu](http://flybase.bio.indiana.edu/.bin/fbidq.html?klu), [SPR](http://flybase.bio.indiana.edu/.bin/fbidq.html?SPR), [sqz](http://flybase.bio.indiana.edu/.bin/fbidq.html?sqz), [Cad74A](http://flybase.bio.indiana.edu/.bin/fbidq.html?Cad74A), [dpr11](http://flybase.bio.indiana.edu/.bin/fbidq.html?dpr11), [Ca-alpha1D](http://flybase.bio.indiana.edu/.bin/fbidq.html?Ca-alpha1D), [Atet](http://flybase.bio.indiana.edu/.bin/fbidq.html?Atet), [tld](http://flybase.bio.indiana.edu/.bin/fbidq.html?tld), [NaCP60E](http://flybase.bio.indiana.edu/.bin/fbidq.html?NaCP60E), [rg](http://flybase.bio.indiana.edu/.bin/fbidq.html?rg), [CG9121](http://flybase.bio.indiana.edu/.bin/fbidq.html?CG9121), [CG1909](http://flybase.bio.indiana.edu/.bin/fbidq.html?CG1909), [gcm2](http://flybase.bio.indiana.edu/.bin/fbidq.html?gcm2), [Ent3](http://flybase.bio.indiana.edu/.bin/fbidq.html?Ent3), [inaE](http://flybase.bio.indiana.edu/.bin/fbidq.html?inaE), [mp](http://flybase.bio.indiana.edu/.bin/fbidq.html?mp), [CG5890](http://flybase.bio.indiana.edu/.bin/fbidq.html?CG5890), [CanB](http://flybase.bio.indiana.edu/.bin/fbidq.html?CanB), [SP2353](http://flybase.bio.indiana.edu/.bin/fbidq.html?SP2353), [can](http://flybase.bio.indiana.edu/.bin/fbidq.html?can), [Smc5](http://flybase.bio.indiana.edu/.bin/fbidq.html?Smc5), [Pask](http://flybase.bio.indiana.edu/.bin/fbidq.html?Pask), [rab3-GEF](http://flybase.bio.indiana.edu/.bin/fbidq.html?rab3-GEF), [CG13253](http://flybase.bio.indiana.edu/.bin/fbidq.html?CG13253), [ovo](http://flybase.bio.indiana.edu/.bin/fbidq.html?ovo), [CG6197](http://flybase.bio.indiana.edu/.bin/fbidq.html?CG6197), [phol](http://flybase.bio.indiana.edu/.bin/fbidq.html?phol),[CG32685](http://flybase.bio.indiana.edu/.bin/fbidq.html?CG32685), [Dsp1](http://flybase.bio.indiana.edu/.bin/fbidq.html?Dsp1), [alphaTub84D](http://flybase.bio.indiana.edu/.bin/fbidq.html?alphaTub84D), [ds](http://flybase.bio.indiana.edu/.bin/fbidq.html?ds), [mei-P26](http://flybase.bio.indiana.edu/.bin/fbidq.html?mei-P26), [Syt4](http://flybase.bio.indiana.edu/.bin/fbidq.html?Syt4), [CG11033](http://flybase.bio.indiana.edu/.bin/fbidq.html?CG11033), [Incenp](http://flybase.bio.indiana.edu/.bin/fbidq.html?Incenp), [Rbp9](http://flybase.bio.indiana.edu/.bin/fbidq.html?Rbp9), [SIFR](http://flybase.bio.indiana.edu/.bin/fbidq.html?SIFR), [CG2061](http://flybase.bio.indiana.edu/.bin/fbidq.html?CG2061), [Ast](http://flybase.bio.indiana.edu/.bin/fbidq.html?Ast), [Drl-2](http://flybase.bio.indiana.edu/.bin/fbidq.html?Drl-2), [CG12858](http://flybase.bio.indiana.edu/.bin/fbidq.html?CG12858), [Tsp42Ej](http://flybase.bio.indiana.edu/.bin/fbidq.html?Tsp42Ej), [CG32944](http://flybase.bio.indiana.edu/.bin/fbidq.html?CG32944), [MYPT-75D](http://flybase.bio.indiana.edu/.bin/fbidq.html?MYPT-75D), [Tk](http://flybase.bio.indiana.edu/.bin/fbidq.html?Tk), [Ilp5](http://flybase.bio.indiana.edu/.bin/fbidq.html?Ilp5), [CG10336](http://flybase.bio.indiana.edu/.bin/fbidq.html?CG10336), [Gap1](http://flybase.bio.indiana.edu/.bin/fbidq.html?Gap1), [Toll-6](http://flybase.bio.indiana.edu/.bin/fbidq.html?Toll-6), [CG3227](http://flybase.bio.indiana.edu/.bin/fbidq.html?CG3227), [beat-IV](http://flybase.bio.indiana.edu/.bin/fbidq.html?beat-IV), [run](http://flybase.bio.indiana.edu/.bin/fbidq.html?run), [MESR4](http://flybase.bio.indiana.edu/.bin/fbidq.html?MESR4), [sNPF-R](http://flybase.bio.indiana.edu/.bin/fbidq.html?sNPF-R), [tara](http://flybase.bio.indiana.edu/.bin/fbidq.html?tara), [Hel89B](http://flybase.bio.indiana.edu/.bin/fbidq.html?Hel89B), [koko](http://flybase.bio.indiana.edu/.bin/fbidq.html?koko),[stan](http://flybase.bio.indiana.edu/.bin/fbidq.html?stan), [CanA-14F](http://flybase.bio.indiana.edu/.bin/fbidq.html?CanA-14F), [Rh50](http://flybase.bio.indiana.edu/.bin/fbidq.html?Rh50), [tutl](http://flybase.bio.indiana.edu/.bin/fbidq.html?tutl), [Ptth](http://flybase.bio.indiana.edu/.bin/fbidq.html?Ptth), [Rab2](http://flybase.bio.indiana.edu/.bin/fbidq.html?Rab2), [CG1753](http://flybase.bio.indiana.edu/.bin/fbidq.html?CG1753), [Ilp2](http://flybase.bio.indiana.edu/.bin/fbidq.html?Ilp2), [Fancl](http://flybase.bio.indiana.edu/.bin/fbidq.html?Fancl), [ph-p](http://flybase.bio.indiana.edu/.bin/fbidq.html?ph-p), [Frq1](http://flybase.bio.indiana.edu/.bin/fbidq.html?Frq1), [CG7757](http://flybase.bio.indiana.edu/.bin/fbidq.html?CG7757), [SPoCk](http://flybase.bio.indiana.edu/.bin/fbidq.html?SPoCk), [sna](http://flybase.bio.indiana.edu/.bin/fbidq.html?sna), [CG12972](http://flybase.bio.indiana.edu/.bin/fbidq.html?CG12972), [Bre1](http://flybase.bio.indiana.edu/.bin/fbidq.html?Bre1), [Taf1](http://flybase.bio.indiana.edu/.bin/fbidq.html?Taf1), [Ggamma1](http://flybase.bio.indiana.edu/.bin/fbidq.html?Ggamma1), [CG7597](http://flybase.bio.indiana.edu/.bin/fbidq.html?CG7597), [CG5466](http://flybase.bio.indiana.edu/.bin/fbidq.html?CG5466), [MBD-like](http://flybase.bio.indiana.edu/.bin/fbidq.html?MBD-like), [esg](http://flybase.bio.indiana.edu/.bin/fbidq.html?esg), [RSG7](http://flybase.bio.indiana.edu/.bin/fbidq.html?RSG7), [m4](http://flybase.bio.indiana.edu/.bin/fbidq.html?m4), [dom](http://flybase.bio.indiana.edu/.bin/fbidq.html?dom), [Dh31](http://flybase.bio.indiana.edu/.bin/fbidq.html?Dh31), [klar](http://flybase.bio.indiana.edu/.bin/fbidq.html?klar), [shi](http://flybase.bio.indiana.edu/.bin/fbidq.html?shi), [Spred](http://flybase.bio.indiana.edu/.bin/fbidq.html?Spred), [siz](http://flybase.bio.indiana.edu/.bin/fbidq.html?siz), [B-H2](http://flybase.bio.indiana.edu/.bin/fbidq.html?B-H2), [tyn](http://flybase.bio.indiana.edu/.bin/fbidq.html?tyn), [CG3822](http://flybase.bio.indiana.edu/.bin/fbidq.html?CG3822), [dpr5](http://flybase.bio.indiana.edu/.bin/fbidq.html?dpr5),[CadN](http://flybase.bio.indiana.edu/.bin/fbidq.html?CadN), [CG34139](http://flybase.bio.indiana.edu/.bin/fbidq.html?CG34139), [Mfap1](http://flybase.bio.indiana.edu/.bin/fbidq.html?Mfap1), [CG13995](http://flybase.bio.indiana.edu/.bin/fbidq.html?CG13995), [E(bx)](http://flybase.bio.indiana.edu/.bin/fbidq.html?E(bx)), [CG12728](http://flybase.bio.indiana.edu/.bin/fbidq.html?CG12728), [dpr19](http://flybase.bio.indiana.edu/.bin/fbidq.html?dpr19), [Dms](http://flybase.bio.indiana.edu/.bin/fbidq.html?Dms), [Rbp2](http://flybase.bio.indiana.edu/.bin/fbidq.html?Rbp2), [Snap](http://flybase.bio.indiana.edu/.bin/fbidq.html?Snap), [trn](http://flybase.bio.indiana.edu/.bin/fbidq.html?trn), [Pitslre](http://flybase.bio.indiana.edu/.bin/fbidq.html?Pitslre), [CG3085](http://flybase.bio.indiana.edu/.bin/fbidq.html?CG3085), [pburs](http://flybase.bio.indiana.edu/.bin/fbidq.html?pburs), [cos](http://flybase.bio.indiana.edu/.bin/fbidq.html?cos) |
| [regulation of transcription from RNA polymerase II promoter](http://amigo.geneontology.org/cgi-bin/amigo/go.cgi?view=details&query=GO:0006357) | 112 of 1594 genes, 7.0% | 321 of 7634 genes, 4.2% | 1.86e-07 | 0.00% | 0.00 | [fz2](http://flybase.bio.indiana.edu/.bin/fbidq.html?fz2), [tld](http://flybase.bio.indiana.edu/.bin/fbidq.html?tld), [acj6](http://flybase.bio.indiana.edu/.bin/fbidq.html?acj6), [skd](http://flybase.bio.indiana.edu/.bin/fbidq.html?skd), [wor](http://flybase.bio.indiana.edu/.bin/fbidq.html?wor), [vvl](http://flybase.bio.indiana.edu/.bin/fbidq.html?vvl), [jing](http://flybase.bio.indiana.edu/.bin/fbidq.html?jing), [caup](http://flybase.bio.indiana.edu/.bin/fbidq.html?caup), [tll](http://flybase.bio.indiana.edu/.bin/fbidq.html?tll), [Camta](http://flybase.bio.indiana.edu/.bin/fbidq.html?Camta), [ato](http://flybase.bio.indiana.edu/.bin/fbidq.html?ato), [MED14](http://flybase.bio.indiana.edu/.bin/fbidq.html?MED14), [Eip93F](http://flybase.bio.indiana.edu/.bin/fbidq.html?Eip93F), [Spt6](http://flybase.bio.indiana.edu/.bin/fbidq.html?Spt6), [Iswi](http://flybase.bio.indiana.edu/.bin/fbidq.html?Iswi), [rn](http://flybase.bio.indiana.edu/.bin/fbidq.html?rn), [ap](http://flybase.bio.indiana.edu/.bin/fbidq.html?ap), [seq](http://flybase.bio.indiana.edu/.bin/fbidq.html?seq), [gcm](http://flybase.bio.indiana.edu/.bin/fbidq.html?gcm), [Dip3](http://flybase.bio.indiana.edu/.bin/fbidq.html?Dip3), [Aef1](http://flybase.bio.indiana.edu/.bin/fbidq.html?Aef1), [phl](http://flybase.bio.indiana.edu/.bin/fbidq.html?phl), [ase](http://flybase.bio.indiana.edu/.bin/fbidq.html?ase), [CG13253](http://flybase.bio.indiana.edu/.bin/fbidq.html?CG13253), [br](http://flybase.bio.indiana.edu/.bin/fbidq.html?br), [ovo](http://flybase.bio.indiana.edu/.bin/fbidq.html?ovo), [ksr](http://flybase.bio.indiana.edu/.bin/fbidq.html?ksr), [Dsp1](http://flybase.bio.indiana.edu/.bin/fbidq.html?Dsp1), [CG14216](http://flybase.bio.indiana.edu/.bin/fbidq.html?CG14216), [bi](http://flybase.bio.indiana.edu/.bin/fbidq.html?bi), [chn](http://flybase.bio.indiana.edu/.bin/fbidq.html?chn), [lola](http://flybase.bio.indiana.edu/.bin/fbidq.html?lola), [Nelf-A](http://flybase.bio.indiana.edu/.bin/fbidq.html?Nelf-A), [simj](http://flybase.bio.indiana.edu/.bin/fbidq.html?simj), [tap](http://flybase.bio.indiana.edu/.bin/fbidq.html?tap), [NC2alpha](http://flybase.bio.indiana.edu/.bin/fbidq.html?NC2alpha), [mip120](http://flybase.bio.indiana.edu/.bin/fbidq.html?mip120), [Snoo](http://flybase.bio.indiana.edu/.bin/fbidq.html?Snoo), [jumu](http://flybase.bio.indiana.edu/.bin/fbidq.html?jumu), [Taf4](http://flybase.bio.indiana.edu/.bin/fbidq.html?Taf4), [HGTX](http://flybase.bio.indiana.edu/.bin/fbidq.html?HGTX), [ush](http://flybase.bio.indiana.edu/.bin/fbidq.html?ush),[fd59A](http://flybase.bio.indiana.edu/.bin/fbidq.html?fd59A), [nub](http://flybase.bio.indiana.edu/.bin/fbidq.html?nub), [Gap1](http://flybase.bio.indiana.edu/.bin/fbidq.html?Gap1), [btd](http://flybase.bio.indiana.edu/.bin/fbidq.html?btd), [fd68A](http://flybase.bio.indiana.edu/.bin/fbidq.html?fd68A), [HLHmdelta](http://flybase.bio.indiana.edu/.bin/fbidq.html?HLHmdelta), [run](http://flybase.bio.indiana.edu/.bin/fbidq.html?run), [B-H1](http://flybase.bio.indiana.edu/.bin/fbidq.html?B-H1), [Antp](http://flybase.bio.indiana.edu/.bin/fbidq.html?Antp), [ATbp](http://flybase.bio.indiana.edu/.bin/fbidq.html?ATbp), [Sos](http://flybase.bio.indiana.edu/.bin/fbidq.html?Sos), [fd102C](http://flybase.bio.indiana.edu/.bin/fbidq.html?fd102C), [Ptth](http://flybase.bio.indiana.edu/.bin/fbidq.html?Ptth), [hth](http://flybase.bio.indiana.edu/.bin/fbidq.html?hth), [vg](http://flybase.bio.indiana.edu/.bin/fbidq.html?vg), [eya](http://flybase.bio.indiana.edu/.bin/fbidq.html?eya), [fz](http://flybase.bio.indiana.edu/.bin/fbidq.html?fz), [Ubx](http://flybase.bio.indiana.edu/.bin/fbidq.html?Ubx), [Vsx2](http://flybase.bio.indiana.edu/.bin/fbidq.html?Vsx2), [Ptx1](http://flybase.bio.indiana.edu/.bin/fbidq.html?Ptx1), [onecut](http://flybase.bio.indiana.edu/.bin/fbidq.html?onecut), [sno](http://flybase.bio.indiana.edu/.bin/fbidq.html?sno), [chm](http://flybase.bio.indiana.edu/.bin/fbidq.html?chm), [ara](http://flybase.bio.indiana.edu/.bin/fbidq.html?ara), [sna](http://flybase.bio.indiana.edu/.bin/fbidq.html?sna), [Wnt5](http://flybase.bio.indiana.edu/.bin/fbidq.html?Wnt5), [mam](http://flybase.bio.indiana.edu/.bin/fbidq.html?mam), [Taf1](http://flybase.bio.indiana.edu/.bin/fbidq.html?Taf1), [pan](http://flybase.bio.indiana.edu/.bin/fbidq.html?pan), [disco](http://flybase.bio.indiana.edu/.bin/fbidq.html?disco), [grn](http://flybase.bio.indiana.edu/.bin/fbidq.html?grn), [tsh](http://flybase.bio.indiana.edu/.bin/fbidq.html?tsh), [esg](http://flybase.bio.indiana.edu/.bin/fbidq.html?esg), [Lis-1](http://flybase.bio.indiana.edu/.bin/fbidq.html?Lis-1), [oc](http://flybase.bio.indiana.edu/.bin/fbidq.html?oc), [pros](http://flybase.bio.indiana.edu/.bin/fbidq.html?pros), [tup](http://flybase.bio.indiana.edu/.bin/fbidq.html?tup), [ey](http://flybase.bio.indiana.edu/.bin/fbidq.html?ey), [pad](http://flybase.bio.indiana.edu/.bin/fbidq.html?pad), [mle](http://flybase.bio.indiana.edu/.bin/fbidq.html?mle), [Bgb](http://flybase.bio.indiana.edu/.bin/fbidq.html?Bgb), [l(1)sc](http://flybase.bio.indiana.edu/.bin/fbidq.html?l(1)sc), [Wnt4](http://flybase.bio.indiana.edu/.bin/fbidq.html?Wnt4),[B-H2](http://flybase.bio.indiana.edu/.bin/fbidq.html?B-H2), [grh](http://flybase.bio.indiana.edu/.bin/fbidq.html?grh), [Rx](http://flybase.bio.indiana.edu/.bin/fbidq.html?Rx), [MED1](http://flybase.bio.indiana.edu/.bin/fbidq.html?MED1), [Vsx1](http://flybase.bio.indiana.edu/.bin/fbidq.html?Vsx1), [wg](http://flybase.bio.indiana.edu/.bin/fbidq.html?wg), [toy](http://flybase.bio.indiana.edu/.bin/fbidq.html?toy), [fkh](http://flybase.bio.indiana.edu/.bin/fbidq.html?fkh), [sim](http://flybase.bio.indiana.edu/.bin/fbidq.html?sim), [gro](http://flybase.bio.indiana.edu/.bin/fbidq.html?gro), [crol](http://flybase.bio.indiana.edu/.bin/fbidq.html?crol), [nerfin-1](http://flybase.bio.indiana.edu/.bin/fbidq.html?nerfin-1), [sd](http://flybase.bio.indiana.edu/.bin/fbidq.html?sd), [trx](http://flybase.bio.indiana.edu/.bin/fbidq.html?trx), [Rpd3](http://flybase.bio.indiana.edu/.bin/fbidq.html?Rpd3), [retn](http://flybase.bio.indiana.edu/.bin/fbidq.html?retn), [en](http://flybase.bio.indiana.edu/.bin/fbidq.html?en), [fs(1)h](http://flybase.bio.indiana.edu/.bin/fbidq.html?fs(1)h), [dsx](http://flybase.bio.indiana.edu/.bin/fbidq.html?dsx), [rl](http://flybase.bio.indiana.edu/.bin/fbidq.html?rl), [CG11347](http://flybase.bio.indiana.edu/.bin/fbidq.html?CG11347), [pygo](http://flybase.bio.indiana.edu/.bin/fbidq.html?pygo), [HLHm7](http://flybase.bio.indiana.edu/.bin/fbidq.html?HLHm7), [HLHmgamma](http://flybase.bio.indiana.edu/.bin/fbidq.html?HLHmgamma), [hiw](http://flybase.bio.indiana.edu/.bin/fbidq.html?hiw), [Ct](http://flybase.bio.indiana.edu/.bin/fbidq.html?Ct), [Atf-2](http://flybase.bio.indiana.edu/.bin/fbidq.html?Atf-2) |
| [organ morphogenesis](http://amigo.geneontology.org/cgi-bin/amigo/go.cgi?view=details&query=GO:0009887) | 174 of 1594 genes, 10.9% | 567 of 7634 genes, 7.4% | 2.43e-07 | 0.00% | 0.00 | [fz2](http://flybase.bio.indiana.edu/.bin/fbidq.html?fz2), [numb](http://flybase.bio.indiana.edu/.bin/fbidq.html?numb), [skd](http://flybase.bio.indiana.edu/.bin/fbidq.html?skd), [jing](http://flybase.bio.indiana.edu/.bin/fbidq.html?jing), [caup](http://flybase.bio.indiana.edu/.bin/fbidq.html?caup), [tll](http://flybase.bio.indiana.edu/.bin/fbidq.html?tll), [vimar](http://flybase.bio.indiana.edu/.bin/fbidq.html?vimar), [ato](http://flybase.bio.indiana.edu/.bin/fbidq.html?ato), [rn](http://flybase.bio.indiana.edu/.bin/fbidq.html?rn), [ap](http://flybase.bio.indiana.edu/.bin/fbidq.html?ap), [Dg](http://flybase.bio.indiana.edu/.bin/fbidq.html?Dg), [kkv](http://flybase.bio.indiana.edu/.bin/fbidq.html?kkv), [CG14478](http://flybase.bio.indiana.edu/.bin/fbidq.html?CG14478), [br](http://flybase.bio.indiana.edu/.bin/fbidq.html?br), [sns](http://flybase.bio.indiana.edu/.bin/fbidq.html?sns), [ksr](http://flybase.bio.indiana.edu/.bin/fbidq.html?ksr), [os](http://flybase.bio.indiana.edu/.bin/fbidq.html?os), [Axn](http://flybase.bio.indiana.edu/.bin/fbidq.html?Axn), [tok](http://flybase.bio.indiana.edu/.bin/fbidq.html?tok), [bi](http://flybase.bio.indiana.edu/.bin/fbidq.html?bi), [chn](http://flybase.bio.indiana.edu/.bin/fbidq.html?chn), [Traf6](http://flybase.bio.indiana.edu/.bin/fbidq.html?Traf6), [lola](http://flybase.bio.indiana.edu/.bin/fbidq.html?lola), [NetB](http://flybase.bio.indiana.edu/.bin/fbidq.html?NetB), [CG15312](http://flybase.bio.indiana.edu/.bin/fbidq.html?CG15312), [ewg](http://flybase.bio.indiana.edu/.bin/fbidq.html?ewg), [Nrk](http://flybase.bio.indiana.edu/.bin/fbidq.html?Nrk), [sano](http://flybase.bio.indiana.edu/.bin/fbidq.html?sano), [bab1](http://flybase.bio.indiana.edu/.bin/fbidq.html?bab1), [Galpha49B](http://flybase.bio.indiana.edu/.bin/fbidq.html?Galpha49B), [Lar](http://flybase.bio.indiana.edu/.bin/fbidq.html?Lar), [scrib](http://flybase.bio.indiana.edu/.bin/fbidq.html?scrib), [Su(var)3-3](http://flybase.bio.indiana.edu/.bin/fbidq.html?Su(var)3-3), [nvy](http://flybase.bio.indiana.edu/.bin/fbidq.html?nvy), [Nrg](http://flybase.bio.indiana.edu/.bin/fbidq.html?Nrg), [btd](http://flybase.bio.indiana.edu/.bin/fbidq.html?btd), [Mmp2](http://flybase.bio.indiana.edu/.bin/fbidq.html?Mmp2), [HLHmdelta](http://flybase.bio.indiana.edu/.bin/fbidq.html?HLHmdelta), [jeb](http://flybase.bio.indiana.edu/.bin/fbidq.html?jeb), [B-H1](http://flybase.bio.indiana.edu/.bin/fbidq.html?B-H1), [alph](http://flybase.bio.indiana.edu/.bin/fbidq.html?alph),[Sema-1a](http://flybase.bio.indiana.edu/.bin/fbidq.html?Sema-1a), [Hs3st-B](http://flybase.bio.indiana.edu/.bin/fbidq.html?Hs3st-B), [Traf4](http://flybase.bio.indiana.edu/.bin/fbidq.html?Traf4), [Sos](http://flybase.bio.indiana.edu/.bin/fbidq.html?Sos), [CG34400](http://flybase.bio.indiana.edu/.bin/fbidq.html?CG34400), [phyl](http://flybase.bio.indiana.edu/.bin/fbidq.html?phyl), [ena](http://flybase.bio.indiana.edu/.bin/fbidq.html?ena), [dsh](http://flybase.bio.indiana.edu/.bin/fbidq.html?dsh), [hth](http://flybase.bio.indiana.edu/.bin/fbidq.html?hth), [vg](http://flybase.bio.indiana.edu/.bin/fbidq.html?vg), [Fas2](http://flybase.bio.indiana.edu/.bin/fbidq.html?Fas2), [eya](http://flybase.bio.indiana.edu/.bin/fbidq.html?eya), [14-3-3zeta](http://flybase.bio.indiana.edu/.bin/fbidq.html?14-3-3zeta), [fz](http://flybase.bio.indiana.edu/.bin/fbidq.html?fz), [robo](http://flybase.bio.indiana.edu/.bin/fbidq.html?robo), [lea](http://flybase.bio.indiana.edu/.bin/fbidq.html?lea), [brm](http://flybase.bio.indiana.edu/.bin/fbidq.html?brm), [CG8557](http://flybase.bio.indiana.edu/.bin/fbidq.html?CG8557), [bsk](http://flybase.bio.indiana.edu/.bin/fbidq.html?bsk), [beat-Ia](http://flybase.bio.indiana.edu/.bin/fbidq.html?beat-Ia), [CG42268](http://flybase.bio.indiana.edu/.bin/fbidq.html?CG42268), [ft](http://flybase.bio.indiana.edu/.bin/fbidq.html?ft), [lft](http://flybase.bio.indiana.edu/.bin/fbidq.html?lft), [sno](http://flybase.bio.indiana.edu/.bin/fbidq.html?sno), [ara](http://flybase.bio.indiana.edu/.bin/fbidq.html?ara), [mam](http://flybase.bio.indiana.edu/.bin/fbidq.html?mam), [pan](http://flybase.bio.indiana.edu/.bin/fbidq.html?pan), [Dll](http://flybase.bio.indiana.edu/.bin/fbidq.html?Dll), [grn](http://flybase.bio.indiana.edu/.bin/fbidq.html?grn), [mew](http://flybase.bio.indiana.edu/.bin/fbidq.html?mew), [neur](http://flybase.bio.indiana.edu/.bin/fbidq.html?neur), [didum](http://flybase.bio.indiana.edu/.bin/fbidq.html?didum), [Lis-1](http://flybase.bio.indiana.edu/.bin/fbidq.html?Lis-1), [dap](http://flybase.bio.indiana.edu/.bin/fbidq.html?dap), [nmo](http://flybase.bio.indiana.edu/.bin/fbidq.html?nmo), [Bx](http://flybase.bio.indiana.edu/.bin/fbidq.html?Bx), [eys](http://flybase.bio.indiana.edu/.bin/fbidq.html?eys), [CG32149](http://flybase.bio.indiana.edu/.bin/fbidq.html?CG32149), [Mob2](http://flybase.bio.indiana.edu/.bin/fbidq.html?Mob2),[pros](http://flybase.bio.indiana.edu/.bin/fbidq.html?pros), [tup](http://flybase.bio.indiana.edu/.bin/fbidq.html?tup), [trio](http://flybase.bio.indiana.edu/.bin/fbidq.html?trio), [Gug](http://flybase.bio.indiana.edu/.bin/fbidq.html?Gug), [ed](http://flybase.bio.indiana.edu/.bin/fbidq.html?ed), [pad](http://flybase.bio.indiana.edu/.bin/fbidq.html?pad), [ImpE2](http://flybase.bio.indiana.edu/.bin/fbidq.html?ImpE2), [Wnt4](http://flybase.bio.indiana.edu/.bin/fbidq.html?Wnt4), [salr](http://flybase.bio.indiana.edu/.bin/fbidq.html?salr), [Apc](http://flybase.bio.indiana.edu/.bin/fbidq.html?Apc), [Glut4EF](http://flybase.bio.indiana.edu/.bin/fbidq.html?Glut4EF), [pk](http://flybase.bio.indiana.edu/.bin/fbidq.html?pk), [Btk29A](http://flybase.bio.indiana.edu/.bin/fbidq.html?Btk29A), [dsx](http://flybase.bio.indiana.edu/.bin/fbidq.html?dsx), [chinmo](http://flybase.bio.indiana.edu/.bin/fbidq.html?chinmo), [unc-5](http://flybase.bio.indiana.edu/.bin/fbidq.html?unc-5), [cdi](http://flybase.bio.indiana.edu/.bin/fbidq.html?cdi), [klu](http://flybase.bio.indiana.edu/.bin/fbidq.html?klu), [aret](http://flybase.bio.indiana.edu/.bin/fbidq.html?aret), [pygo](http://flybase.bio.indiana.edu/.bin/fbidq.html?pygo), [HLHm7](http://flybase.bio.indiana.edu/.bin/fbidq.html?HLHm7), [spag](http://flybase.bio.indiana.edu/.bin/fbidq.html?spag), [Lim1](http://flybase.bio.indiana.edu/.bin/fbidq.html?Lim1), [nkd](http://flybase.bio.indiana.edu/.bin/fbidq.html?nkd), [tld](http://flybase.bio.indiana.edu/.bin/fbidq.html?tld), [jar](http://flybase.bio.indiana.edu/.bin/fbidq.html?jar), [tow](http://flybase.bio.indiana.edu/.bin/fbidq.html?tow), [rg](http://flybase.bio.indiana.edu/.bin/fbidq.html?rg), [Pten](http://flybase.bio.indiana.edu/.bin/fbidq.html?Pten), [betaInt-nu](http://flybase.bio.indiana.edu/.bin/fbidq.html?betaInt-nu), [Brd](http://flybase.bio.indiana.edu/.bin/fbidq.html?Brd), [sgg](http://flybase.bio.indiana.edu/.bin/fbidq.html?sgg), [Nmdar1](http://flybase.bio.indiana.edu/.bin/fbidq.html?Nmdar1), [bowl](http://flybase.bio.indiana.edu/.bin/fbidq.html?bowl), [Dip3](http://flybase.bio.indiana.edu/.bin/fbidq.html?Dip3), [SP2353](http://flybase.bio.indiana.edu/.bin/fbidq.html?SP2353), [phl](http://flybase.bio.indiana.edu/.bin/fbidq.html?phl), [Cbl](http://flybase.bio.indiana.edu/.bin/fbidq.html?Cbl), [ase](http://flybase.bio.indiana.edu/.bin/fbidq.html?ase), [ovo](http://flybase.bio.indiana.edu/.bin/fbidq.html?ovo), [ds](http://flybase.bio.indiana.edu/.bin/fbidq.html?ds), [Alk](http://flybase.bio.indiana.edu/.bin/fbidq.html?Alk),[Poxn](http://flybase.bio.indiana.edu/.bin/fbidq.html?Poxn), [Vang](http://flybase.bio.indiana.edu/.bin/fbidq.html?Vang), [pot](http://flybase.bio.indiana.edu/.bin/fbidq.html?pot), [jumu](http://flybase.bio.indiana.edu/.bin/fbidq.html?jumu), [Smox](http://flybase.bio.indiana.edu/.bin/fbidq.html?Smox), [fred](http://flybase.bio.indiana.edu/.bin/fbidq.html?fred), [MYPT-75D](http://flybase.bio.indiana.edu/.bin/fbidq.html?MYPT-75D), [Gap1](http://flybase.bio.indiana.edu/.bin/fbidq.html?Gap1), [run](http://flybase.bio.indiana.edu/.bin/fbidq.html?run), [l(3)neo38](http://flybase.bio.indiana.edu/.bin/fbidq.html?l(3)neo38), [stan](http://flybase.bio.indiana.edu/.bin/fbidq.html?stan), [cindr](http://flybase.bio.indiana.edu/.bin/fbidq.html?cindr), [Sp1](http://flybase.bio.indiana.edu/.bin/fbidq.html?Sp1), [Ubx](http://flybase.bio.indiana.edu/.bin/fbidq.html?Ubx), [tsh](http://flybase.bio.indiana.edu/.bin/fbidq.html?tsh), [Syn2](http://flybase.bio.indiana.edu/.bin/fbidq.html?Syn2), [esg](http://flybase.bio.indiana.edu/.bin/fbidq.html?esg), [e(y)3](http://flybase.bio.indiana.edu/.bin/fbidq.html?e(y)3), [dac](http://flybase.bio.indiana.edu/.bin/fbidq.html?dac), [oc](http://flybase.bio.indiana.edu/.bin/fbidq.html?oc), [ey](http://flybase.bio.indiana.edu/.bin/fbidq.html?ey), [baz](http://flybase.bio.indiana.edu/.bin/fbidq.html?baz), [klar](http://flybase.bio.indiana.edu/.bin/fbidq.html?klar), [CG33275](http://flybase.bio.indiana.edu/.bin/fbidq.html?CG33275), [Hr51](http://flybase.bio.indiana.edu/.bin/fbidq.html?Hr51), [shi](http://flybase.bio.indiana.edu/.bin/fbidq.html?shi), [siz](http://flybase.bio.indiana.edu/.bin/fbidq.html?siz), [rst](http://flybase.bio.indiana.edu/.bin/fbidq.html?rst), [CG6398](http://flybase.bio.indiana.edu/.bin/fbidq.html?CG6398), [B-H2](http://flybase.bio.indiana.edu/.bin/fbidq.html?B-H2), [grh](http://flybase.bio.indiana.edu/.bin/fbidq.html?grh), [toy](http://flybase.bio.indiana.edu/.bin/fbidq.html?toy), [wg](http://flybase.bio.indiana.edu/.bin/fbidq.html?wg), [fkh](http://flybase.bio.indiana.edu/.bin/fbidq.html?fkh), [CadN](http://flybase.bio.indiana.edu/.bin/fbidq.html?CadN), [crol](http://flybase.bio.indiana.edu/.bin/fbidq.html?crol), [sd](http://flybase.bio.indiana.edu/.bin/fbidq.html?sd), [fs(1)h](http://flybase.bio.indiana.edu/.bin/fbidq.html?fs(1)h), [klg](http://flybase.bio.indiana.edu/.bin/fbidq.html?klg), [sfl](http://flybase.bio.indiana.edu/.bin/fbidq.html?sfl), [rl](http://flybase.bio.indiana.edu/.bin/fbidq.html?rl), [Sip1](http://flybase.bio.indiana.edu/.bin/fbidq.html?Sip1), [Snap](http://flybase.bio.indiana.edu/.bin/fbidq.html?Snap),[trn](http://flybase.bio.indiana.edu/.bin/fbidq.html?trn), [yrt](http://flybase.bio.indiana.edu/.bin/fbidq.html?yrt), [wls](http://flybase.bio.indiana.edu/.bin/fbidq.html?wls), [pburs](http://flybase.bio.indiana.edu/.bin/fbidq.html?pburs), [unk](http://flybase.bio.indiana.edu/.bin/fbidq.html?unk), [cos](http://flybase.bio.indiana.edu/.bin/fbidq.html?cos), [dnr1](http://flybase.bio.indiana.edu/.bin/fbidq.html?dnr1), [CG32653](http://flybase.bio.indiana.edu/.bin/fbidq.html?CG32653), [Ct](http://flybase.bio.indiana.edu/.bin/fbidq.html?Ct) |
| [organ development](http://amigo.geneontology.org/cgi-bin/amigo/go.cgi?view=details&query=GO:0048513) | 280 of 1594 genes, 17.6% | 1019 of 7634 genes, 13.3% | 3.28e-07 | 0.00% | 0.00 | [fz2](http://flybase.bio.indiana.edu/.bin/fbidq.html?fz2), [Ocho](http://flybase.bio.indiana.edu/.bin/fbidq.html?Ocho), [numb](http://flybase.bio.indiana.edu/.bin/fbidq.html?numb), [stj](http://flybase.bio.indiana.edu/.bin/fbidq.html?stj), [Sxl](http://flybase.bio.indiana.edu/.bin/fbidq.html?Sxl), [CG33007](http://flybase.bio.indiana.edu/.bin/fbidq.html?CG33007), [rn](http://flybase.bio.indiana.edu/.bin/fbidq.html?rn), [ap](http://flybase.bio.indiana.edu/.bin/fbidq.html?ap), [kkv](http://flybase.bio.indiana.edu/.bin/fbidq.html?kkv), [malpha](http://flybase.bio.indiana.edu/.bin/fbidq.html?malpha), [sns](http://flybase.bio.indiana.edu/.bin/fbidq.html?sns), [os](http://flybase.bio.indiana.edu/.bin/fbidq.html?os), [Axn](http://flybase.bio.indiana.edu/.bin/fbidq.html?Axn), [tok](http://flybase.bio.indiana.edu/.bin/fbidq.html?tok), [bi](http://flybase.bio.indiana.edu/.bin/fbidq.html?bi), [Traf6](http://flybase.bio.indiana.edu/.bin/fbidq.html?Traf6), [NetB](http://flybase.bio.indiana.edu/.bin/fbidq.html?NetB), [Tango10](http://flybase.bio.indiana.edu/.bin/fbidq.html?Tango10), [bab1](http://flybase.bio.indiana.edu/.bin/fbidq.html?bab1), [Galpha49B](http://flybase.bio.indiana.edu/.bin/fbidq.html?Galpha49B), [Lar](http://flybase.bio.indiana.edu/.bin/fbidq.html?Lar), [scrib](http://flybase.bio.indiana.edu/.bin/fbidq.html?scrib), [rut](http://flybase.bio.indiana.edu/.bin/fbidq.html?rut), [nvy](http://flybase.bio.indiana.edu/.bin/fbidq.html?nvy), [Ect3](http://flybase.bio.indiana.edu/.bin/fbidq.html?Ect3), [nub](http://flybase.bio.indiana.edu/.bin/fbidq.html?nub), [nab](http://flybase.bio.indiana.edu/.bin/fbidq.html?nab), [Ddc](http://flybase.bio.indiana.edu/.bin/fbidq.html?Ddc), [btd](http://flybase.bio.indiana.edu/.bin/fbidq.html?btd), [Mmp2](http://flybase.bio.indiana.edu/.bin/fbidq.html?Mmp2), [HLHmdelta](http://flybase.bio.indiana.edu/.bin/fbidq.html?HLHmdelta), [B-H1](http://flybase.bio.indiana.edu/.bin/fbidq.html?B-H1), [Nipped-A](http://flybase.bio.indiana.edu/.bin/fbidq.html?Nipped-A), [pim](http://flybase.bio.indiana.edu/.bin/fbidq.html?pim), [alph](http://flybase.bio.indiana.edu/.bin/fbidq.html?alph), [Traf4](http://flybase.bio.indiana.edu/.bin/fbidq.html?Traf4), [TBPH](http://flybase.bio.indiana.edu/.bin/fbidq.html?TBPH), [CG34400](http://flybase.bio.indiana.edu/.bin/fbidq.html?CG34400), [ena](http://flybase.bio.indiana.edu/.bin/fbidq.html?ena),[mGluRA](http://flybase.bio.indiana.edu/.bin/fbidq.html?mGluRA), [dsh](http://flybase.bio.indiana.edu/.bin/fbidq.html?dsh), [hth](http://flybase.bio.indiana.edu/.bin/fbidq.html?hth), [Nf-YC](http://flybase.bio.indiana.edu/.bin/fbidq.html?Nf-YC), [14-3-3zeta](http://flybase.bio.indiana.edu/.bin/fbidq.html?14-3-3zeta), [lea](http://flybase.bio.indiana.edu/.bin/fbidq.html?lea), [PP2A-B](http://flybase.bio.indiana.edu/.bin/fbidq.html?PP2A-B), [neur](http://flybase.bio.indiana.edu/.bin/fbidq.html?neur), [CycE](http://flybase.bio.indiana.edu/.bin/fbidq.html?CycE), [nmo](http://flybase.bio.indiana.edu/.bin/fbidq.html?nmo), [CG32149](http://flybase.bio.indiana.edu/.bin/fbidq.html?CG32149), [Gug](http://flybase.bio.indiana.edu/.bin/fbidq.html?Gug), [ed](http://flybase.bio.indiana.edu/.bin/fbidq.html?ed), [pad](http://flybase.bio.indiana.edu/.bin/fbidq.html?pad), [mr](http://flybase.bio.indiana.edu/.bin/fbidq.html?mr), [ImpE2](http://flybase.bio.indiana.edu/.bin/fbidq.html?ImpE2), [Rx](http://flybase.bio.indiana.edu/.bin/fbidq.html?Rx), [salr](http://flybase.bio.indiana.edu/.bin/fbidq.html?salr), [Apc](http://flybase.bio.indiana.edu/.bin/fbidq.html?Apc), [sim](http://flybase.bio.indiana.edu/.bin/fbidq.html?sim), [d4](http://flybase.bio.indiana.edu/.bin/fbidq.html?d4), [dan](http://flybase.bio.indiana.edu/.bin/fbidq.html?dan), [Mf](http://flybase.bio.indiana.edu/.bin/fbidq.html?Mf), [rdo](http://flybase.bio.indiana.edu/.bin/fbidq.html?rdo), [Rpd3](http://flybase.bio.indiana.edu/.bin/fbidq.html?Rpd3), [Btk29A](http://flybase.bio.indiana.edu/.bin/fbidq.html?Btk29A), [unc-5](http://flybase.bio.indiana.edu/.bin/fbidq.html?unc-5), [cdi](http://flybase.bio.indiana.edu/.bin/fbidq.html?cdi), [barr](http://flybase.bio.indiana.edu/.bin/fbidq.html?barr), [aret](http://flybase.bio.indiana.edu/.bin/fbidq.html?aret), [pygo](http://flybase.bio.indiana.edu/.bin/fbidq.html?pygo), [HLHm7](http://flybase.bio.indiana.edu/.bin/fbidq.html?HLHm7), [HLHmgamma](http://flybase.bio.indiana.edu/.bin/fbidq.html?HLHmgamma), [spag](http://flybase.bio.indiana.edu/.bin/fbidq.html?spag), [jar](http://flybase.bio.indiana.edu/.bin/fbidq.html?jar), [tow](http://flybase.bio.indiana.edu/.bin/fbidq.html?tow), [CG15609](http://flybase.bio.indiana.edu/.bin/fbidq.html?CG15609), [wor](http://flybase.bio.indiana.edu/.bin/fbidq.html?wor),[Atg1](http://flybase.bio.indiana.edu/.bin/fbidq.html?Atg1), [l(1)G0148](http://flybase.bio.indiana.edu/.bin/fbidq.html?l(1)G0148), [Pten](http://flybase.bio.indiana.edu/.bin/fbidq.html?Pten), [bowl](http://flybase.bio.indiana.edu/.bin/fbidq.html?bowl), [seq](http://flybase.bio.indiana.edu/.bin/fbidq.html?seq), [Dip3](http://flybase.bio.indiana.edu/.bin/fbidq.html?Dip3), [oa2](http://flybase.bio.indiana.edu/.bin/fbidq.html?oa2), [phl](http://flybase.bio.indiana.edu/.bin/fbidq.html?phl), [Cbl](http://flybase.bio.indiana.edu/.bin/fbidq.html?Cbl), [thr](http://flybase.bio.indiana.edu/.bin/fbidq.html?thr), [Alk](http://flybase.bio.indiana.edu/.bin/fbidq.html?Alk), [Vang](http://flybase.bio.indiana.edu/.bin/fbidq.html?Vang), [Sin3A](http://flybase.bio.indiana.edu/.bin/fbidq.html?Sin3A), [pot](http://flybase.bio.indiana.edu/.bin/fbidq.html?pot), [cpx](http://flybase.bio.indiana.edu/.bin/fbidq.html?cpx), [jumu](http://flybase.bio.indiana.edu/.bin/fbidq.html?jumu), [Smox](http://flybase.bio.indiana.edu/.bin/fbidq.html?Smox), [brat](http://flybase.bio.indiana.edu/.bin/fbidq.html?brat), [ST6Gal](http://flybase.bio.indiana.edu/.bin/fbidq.html?ST6Gal), [Antp](http://flybase.bio.indiana.edu/.bin/fbidq.html?Antp), [l(3)neo38](http://flybase.bio.indiana.edu/.bin/fbidq.html?l(3)neo38), [cindr](http://flybase.bio.indiana.edu/.bin/fbidq.html?cindr), [futsch](http://flybase.bio.indiana.edu/.bin/fbidq.html?futsch), [comm3](http://flybase.bio.indiana.edu/.bin/fbidq.html?comm3), [Ubx](http://flybase.bio.indiana.edu/.bin/fbidq.html?Ubx), [chm](http://flybase.bio.indiana.edu/.bin/fbidq.html?chm), [CG6114](http://flybase.bio.indiana.edu/.bin/fbidq.html?CG6114), [tsh](http://flybase.bio.indiana.edu/.bin/fbidq.html?tsh), [Syn2](http://flybase.bio.indiana.edu/.bin/fbidq.html?Syn2), [eas](http://flybase.bio.indiana.edu/.bin/fbidq.html?eas), [dac](http://flybase.bio.indiana.edu/.bin/fbidq.html?dac), [e(y)3](http://flybase.bio.indiana.edu/.bin/fbidq.html?e(y)3), [Cdk5](http://flybase.bio.indiana.edu/.bin/fbidq.html?Cdk5), [oc](http://flybase.bio.indiana.edu/.bin/fbidq.html?oc), [CG4641](http://flybase.bio.indiana.edu/.bin/fbidq.html?CG4641), [ey](http://flybase.bio.indiana.edu/.bin/fbidq.html?ey), [baz](http://flybase.bio.indiana.edu/.bin/fbidq.html?baz), [CG33275](http://flybase.bio.indiana.edu/.bin/fbidq.html?CG33275), [rst](http://flybase.bio.indiana.edu/.bin/fbidq.html?rst),[CG6398](http://flybase.bio.indiana.edu/.bin/fbidq.html?CG6398), [l(1)sc](http://flybase.bio.indiana.edu/.bin/fbidq.html?l(1)sc), [grh](http://flybase.bio.indiana.edu/.bin/fbidq.html?grh), [toy](http://flybase.bio.indiana.edu/.bin/fbidq.html?toy), [crol](http://flybase.bio.indiana.edu/.bin/fbidq.html?crol), [sd](http://flybase.bio.indiana.edu/.bin/fbidq.html?sd), [retn](http://flybase.bio.indiana.edu/.bin/fbidq.html?retn), [fs(1)h](http://flybase.bio.indiana.edu/.bin/fbidq.html?fs(1)h), [Eip63F-1](http://flybase.bio.indiana.edu/.bin/fbidq.html?Eip63F-1), [rl](http://flybase.bio.indiana.edu/.bin/fbidq.html?rl), [yrt](http://flybase.bio.indiana.edu/.bin/fbidq.html?yrt), [wls](http://flybase.bio.indiana.edu/.bin/fbidq.html?wls), [hiw](http://flybase.bio.indiana.edu/.bin/fbidq.html?hiw), [unk](http://flybase.bio.indiana.edu/.bin/fbidq.html?unk), [CG32653](http://flybase.bio.indiana.edu/.bin/fbidq.html?CG32653), [dnr1](http://flybase.bio.indiana.edu/.bin/fbidq.html?dnr1), [skd](http://flybase.bio.indiana.edu/.bin/fbidq.html?skd), [jing](http://flybase.bio.indiana.edu/.bin/fbidq.html?jing), [katanin-60](http://flybase.bio.indiana.edu/.bin/fbidq.html?katanin-60), [caup](http://flybase.bio.indiana.edu/.bin/fbidq.html?caup), [tll](http://flybase.bio.indiana.edu/.bin/fbidq.html?tll), [ato](http://flybase.bio.indiana.edu/.bin/fbidq.html?ato), [vimar](http://flybase.bio.indiana.edu/.bin/fbidq.html?vimar), [Iswi](http://flybase.bio.indiana.edu/.bin/fbidq.html?Iswi), [Dg](http://flybase.bio.indiana.edu/.bin/fbidq.html?Dg), [CG14478](http://flybase.bio.indiana.edu/.bin/fbidq.html?CG14478), [br](http://flybase.bio.indiana.edu/.bin/fbidq.html?br), [ksr](http://flybase.bio.indiana.edu/.bin/fbidq.html?ksr), [robo3](http://flybase.bio.indiana.edu/.bin/fbidq.html?robo3), [chn](http://flybase.bio.indiana.edu/.bin/fbidq.html?chn), [lola](http://flybase.bio.indiana.edu/.bin/fbidq.html?lola), [unc-104](http://flybase.bio.indiana.edu/.bin/fbidq.html?unc-104), [CG15312](http://flybase.bio.indiana.edu/.bin/fbidq.html?CG15312), [tap](http://flybase.bio.indiana.edu/.bin/fbidq.html?tap), [Awh](http://flybase.bio.indiana.edu/.bin/fbidq.html?Awh), [ewg](http://flybase.bio.indiana.edu/.bin/fbidq.html?ewg), [metro](http://flybase.bio.indiana.edu/.bin/fbidq.html?metro), [Nrk](http://flybase.bio.indiana.edu/.bin/fbidq.html?Nrk), [sano](http://flybase.bio.indiana.edu/.bin/fbidq.html?sano),[CG11399](http://flybase.bio.indiana.edu/.bin/fbidq.html?CG11399), [Su(var)3-3](http://flybase.bio.indiana.edu/.bin/fbidq.html?Su(var)3-3), [Taf4](http://flybase.bio.indiana.edu/.bin/fbidq.html?Taf4), [Nrg](http://flybase.bio.indiana.edu/.bin/fbidq.html?Nrg), [insc](http://flybase.bio.indiana.edu/.bin/fbidq.html?insc), [jeb](http://flybase.bio.indiana.edu/.bin/fbidq.html?jeb), [Sema-1a](http://flybase.bio.indiana.edu/.bin/fbidq.html?Sema-1a), [Hs3st-B](http://flybase.bio.indiana.edu/.bin/fbidq.html?Hs3st-B), [Sos](http://flybase.bio.indiana.edu/.bin/fbidq.html?Sos), [phyl](http://flybase.bio.indiana.edu/.bin/fbidq.html?phyl), [vg](http://flybase.bio.indiana.edu/.bin/fbidq.html?vg), [eya](http://flybase.bio.indiana.edu/.bin/fbidq.html?eya), [Fas2](http://flybase.bio.indiana.edu/.bin/fbidq.html?Fas2), [CG8557](http://flybase.bio.indiana.edu/.bin/fbidq.html?CG8557), [bsk](http://flybase.bio.indiana.edu/.bin/fbidq.html?bsk), [brm](http://flybase.bio.indiana.edu/.bin/fbidq.html?brm), [robo](http://flybase.bio.indiana.edu/.bin/fbidq.html?robo), [fz](http://flybase.bio.indiana.edu/.bin/fbidq.html?fz), [beat-Ia](http://flybase.bio.indiana.edu/.bin/fbidq.html?beat-Ia), [ft](http://flybase.bio.indiana.edu/.bin/fbidq.html?ft), [CG42268](http://flybase.bio.indiana.edu/.bin/fbidq.html?CG42268), [lft](http://flybase.bio.indiana.edu/.bin/fbidq.html?lft), [sno](http://flybase.bio.indiana.edu/.bin/fbidq.html?sno), [RhoGAPp190](http://flybase.bio.indiana.edu/.bin/fbidq.html?RhoGAPp190), [ara](http://flybase.bio.indiana.edu/.bin/fbidq.html?ara), [Sfmbt](http://flybase.bio.indiana.edu/.bin/fbidq.html?Sfmbt), [mam](http://flybase.bio.indiana.edu/.bin/fbidq.html?mam), [sws](http://flybase.bio.indiana.edu/.bin/fbidq.html?sws), [pan](http://flybase.bio.indiana.edu/.bin/fbidq.html?pan), [disco](http://flybase.bio.indiana.edu/.bin/fbidq.html?disco), [Dll](http://flybase.bio.indiana.edu/.bin/fbidq.html?Dll), [grn](http://flybase.bio.indiana.edu/.bin/fbidq.html?grn), [mew](http://flybase.bio.indiana.edu/.bin/fbidq.html?mew), [HLHm5](http://flybase.bio.indiana.edu/.bin/fbidq.html?HLHm5), [Lis-1](http://flybase.bio.indiana.edu/.bin/fbidq.html?Lis-1), [didum](http://flybase.bio.indiana.edu/.bin/fbidq.html?didum), [ems](http://flybase.bio.indiana.edu/.bin/fbidq.html?ems),[dap](http://flybase.bio.indiana.edu/.bin/fbidq.html?dap), [eys](http://flybase.bio.indiana.edu/.bin/fbidq.html?eys), [Bx](http://flybase.bio.indiana.edu/.bin/fbidq.html?Bx), [Mob2](http://flybase.bio.indiana.edu/.bin/fbidq.html?Mob2), [pros](http://flybase.bio.indiana.edu/.bin/fbidq.html?pros), [tup](http://flybase.bio.indiana.edu/.bin/fbidq.html?tup), [trio](http://flybase.bio.indiana.edu/.bin/fbidq.html?trio), [Gad1](http://flybase.bio.indiana.edu/.bin/fbidq.html?Gad1), [Wnt4](http://flybase.bio.indiana.edu/.bin/fbidq.html?Wnt4), [rictor](http://flybase.bio.indiana.edu/.bin/fbidq.html?rictor), [Glut4EF](http://flybase.bio.indiana.edu/.bin/fbidq.html?Glut4EF), [CaMKII](http://flybase.bio.indiana.edu/.bin/fbidq.html?CaMKII), [bchs](http://flybase.bio.indiana.edu/.bin/fbidq.html?bchs), [polo](http://flybase.bio.indiana.edu/.bin/fbidq.html?polo), [pk](http://flybase.bio.indiana.edu/.bin/fbidq.html?pk), [dsx](http://flybase.bio.indiana.edu/.bin/fbidq.html?dsx), [chinmo](http://flybase.bio.indiana.edu/.bin/fbidq.html?chinmo), [Syn1](http://flybase.bio.indiana.edu/.bin/fbidq.html?Syn1), [betaTub85D](http://flybase.bio.indiana.edu/.bin/fbidq.html?betaTub85D), [Ssdp](http://flybase.bio.indiana.edu/.bin/fbidq.html?Ssdp), [klu](http://flybase.bio.indiana.edu/.bin/fbidq.html?klu), [sqz](http://flybase.bio.indiana.edu/.bin/fbidq.html?sqz), [Lim1](http://flybase.bio.indiana.edu/.bin/fbidq.html?Lim1), [sr](http://flybase.bio.indiana.edu/.bin/fbidq.html?sr), [nkd](http://flybase.bio.indiana.edu/.bin/fbidq.html?nkd), [tld](http://flybase.bio.indiana.edu/.bin/fbidq.html?tld), [danr](http://flybase.bio.indiana.edu/.bin/fbidq.html?danr), [Dsk](http://flybase.bio.indiana.edu/.bin/fbidq.html?Dsk), [vvl](http://flybase.bio.indiana.edu/.bin/fbidq.html?vvl), [rg](http://flybase.bio.indiana.edu/.bin/fbidq.html?rg), [betaInt-nu](http://flybase.bio.indiana.edu/.bin/fbidq.html?betaInt-nu), [Brd](http://flybase.bio.indiana.edu/.bin/fbidq.html?Brd), [sgg](http://flybase.bio.indiana.edu/.bin/fbidq.html?sgg), [mnb](http://flybase.bio.indiana.edu/.bin/fbidq.html?mnb), [gcm2](http://flybase.bio.indiana.edu/.bin/fbidq.html?gcm2), [Nmdar1](http://flybase.bio.indiana.edu/.bin/fbidq.html?Nmdar1), [Eip93F](http://flybase.bio.indiana.edu/.bin/fbidq.html?Eip93F), [gcm](http://flybase.bio.indiana.edu/.bin/fbidq.html?gcm), [CG5890](http://flybase.bio.indiana.edu/.bin/fbidq.html?CG5890),[SP2353](http://flybase.bio.indiana.edu/.bin/fbidq.html?SP2353), [ase](http://flybase.bio.indiana.edu/.bin/fbidq.html?ase), [CG13253](http://flybase.bio.indiana.edu/.bin/fbidq.html?CG13253), [ovo](http://flybase.bio.indiana.edu/.bin/fbidq.html?ovo), [Dsp1](http://flybase.bio.indiana.edu/.bin/fbidq.html?Dsp1), [ds](http://flybase.bio.indiana.edu/.bin/fbidq.html?ds), [Poxn](http://flybase.bio.indiana.edu/.bin/fbidq.html?Poxn), [Drl-2](http://flybase.bio.indiana.edu/.bin/fbidq.html?Drl-2), [fred](http://flybase.bio.indiana.edu/.bin/fbidq.html?fred), [eIF4G2](http://flybase.bio.indiana.edu/.bin/fbidq.html?eIF4G2), [MYPT-75D](http://flybase.bio.indiana.edu/.bin/fbidq.html?MYPT-75D), [ush](http://flybase.bio.indiana.edu/.bin/fbidq.html?ush), [Fak56D](http://flybase.bio.indiana.edu/.bin/fbidq.html?Fak56D), [Gap1](http://flybase.bio.indiana.edu/.bin/fbidq.html?Gap1), [run](http://flybase.bio.indiana.edu/.bin/fbidq.html?run), [tara](http://flybase.bio.indiana.edu/.bin/fbidq.html?tara), [Alh](http://flybase.bio.indiana.edu/.bin/fbidq.html?Alh), [Eip71CD](http://flybase.bio.indiana.edu/.bin/fbidq.html?Eip71CD), [egg](http://flybase.bio.indiana.edu/.bin/fbidq.html?egg), [stan](http://flybase.bio.indiana.edu/.bin/fbidq.html?stan), [Sp1](http://flybase.bio.indiana.edu/.bin/fbidq.html?Sp1), [Frq1](http://flybase.bio.indiana.edu/.bin/fbidq.html?Frq1), [sna](http://flybase.bio.indiana.edu/.bin/fbidq.html?sna), [Wnt5](http://flybase.bio.indiana.edu/.bin/fbidq.html?Wnt5), [Ggamma1](http://flybase.bio.indiana.edu/.bin/fbidq.html?Ggamma1), [esg](http://flybase.bio.indiana.edu/.bin/fbidq.html?esg), [m4](http://flybase.bio.indiana.edu/.bin/fbidq.html?m4), [dom](http://flybase.bio.indiana.edu/.bin/fbidq.html?dom), [ssh](http://flybase.bio.indiana.edu/.bin/fbidq.html?ssh), [klar](http://flybase.bio.indiana.edu/.bin/fbidq.html?klar), [PIP5K59B](http://flybase.bio.indiana.edu/.bin/fbidq.html?PIP5K59B), [Hr51](http://flybase.bio.indiana.edu/.bin/fbidq.html?Hr51), [shi](http://flybase.bio.indiana.edu/.bin/fbidq.html?shi), [siz](http://flybase.bio.indiana.edu/.bin/fbidq.html?siz), [B-H2](http://flybase.bio.indiana.edu/.bin/fbidq.html?B-H2), [wg](http://flybase.bio.indiana.edu/.bin/fbidq.html?wg), [fkh](http://flybase.bio.indiana.edu/.bin/fbidq.html?fkh),[CadN](http://flybase.bio.indiana.edu/.bin/fbidq.html?CadN), [E(bx)](http://flybase.bio.indiana.edu/.bin/fbidq.html?E(bx)), [en](http://flybase.bio.indiana.edu/.bin/fbidq.html?en), [klg](http://flybase.bio.indiana.edu/.bin/fbidq.html?klg), [sfl](http://flybase.bio.indiana.edu/.bin/fbidq.html?sfl), [Sip1](http://flybase.bio.indiana.edu/.bin/fbidq.html?Sip1), [Snap](http://flybase.bio.indiana.edu/.bin/fbidq.html?Snap), [trn](http://flybase.bio.indiana.edu/.bin/fbidq.html?trn), [Utx](http://flybase.bio.indiana.edu/.bin/fbidq.html?Utx), [pburs](http://flybase.bio.indiana.edu/.bin/fbidq.html?pburs), [cos](http://flybase.bio.indiana.edu/.bin/fbidq.html?cos), [Ct](http://flybase.bio.indiana.edu/.bin/fbidq.html?Ct) |
| [multicellular organismal process](http://amigo.geneontology.org/cgi-bin/amigo/go.cgi?view=details&query=GO:0032501) | 588 of 1594 genes, 36.9% | 2439 of 7634 genes, 31.9% | 3.54e-07 | 0.00% | 0.00 | [endoA](http://flybase.bio.indiana.edu/.bin/fbidq.html?endoA), [Sh](http://flybase.bio.indiana.edu/.bin/fbidq.html?Sh), [dock](http://flybase.bio.indiana.edu/.bin/fbidq.html?dock), [CG5808](http://flybase.bio.indiana.edu/.bin/fbidq.html?CG5808), [CG9004](http://flybase.bio.indiana.edu/.bin/fbidq.html?CG9004), [CG3703](http://flybase.bio.indiana.edu/.bin/fbidq.html?CG3703), [rn](http://flybase.bio.indiana.edu/.bin/fbidq.html?rn), [ap](http://flybase.bio.indiana.edu/.bin/fbidq.html?ap), [kkv](http://flybase.bio.indiana.edu/.bin/fbidq.html?kkv), [tlk](http://flybase.bio.indiana.edu/.bin/fbidq.html?tlk), [malpha](http://flybase.bio.indiana.edu/.bin/fbidq.html?malpha), [os](http://flybase.bio.indiana.edu/.bin/fbidq.html?os), [Axn](http://flybase.bio.indiana.edu/.bin/fbidq.html?Axn), [Traf6](http://flybase.bio.indiana.edu/.bin/fbidq.html?Traf6), [Leucokinin](http://flybase.bio.indiana.edu/.bin/fbidq.html?Leucokinin), [NetB](http://flybase.bio.indiana.edu/.bin/fbidq.html?NetB), [rdgC](http://flybase.bio.indiana.edu/.bin/fbidq.html?rdgC), [cid](http://flybase.bio.indiana.edu/.bin/fbidq.html?cid), [CG32137](http://flybase.bio.indiana.edu/.bin/fbidq.html?CG32137), [lute](http://flybase.bio.indiana.edu/.bin/fbidq.html?lute), [CG4476](http://flybase.bio.indiana.edu/.bin/fbidq.html?CG4476), [Ssrp](http://flybase.bio.indiana.edu/.bin/fbidq.html?Ssrp), [Lar](http://flybase.bio.indiana.edu/.bin/fbidq.html?Lar), [scrib](http://flybase.bio.indiana.edu/.bin/fbidq.html?scrib), [HGTX](http://flybase.bio.indiana.edu/.bin/fbidq.html?HGTX), [not](http://flybase.bio.indiana.edu/.bin/fbidq.html?not), [nvy](http://flybase.bio.indiana.edu/.bin/fbidq.html?nvy), [Ect3](http://flybase.bio.indiana.edu/.bin/fbidq.html?Ect3), [nab](http://flybase.bio.indiana.edu/.bin/fbidq.html?nab), [Ddc](http://flybase.bio.indiana.edu/.bin/fbidq.html?Ddc), [HLHmdelta](http://flybase.bio.indiana.edu/.bin/fbidq.html?HLHmdelta), [alph](http://flybase.bio.indiana.edu/.bin/fbidq.html?alph), [Traf4](http://flybase.bio.indiana.edu/.bin/fbidq.html?Traf4), [TBPH](http://flybase.bio.indiana.edu/.bin/fbidq.html?TBPH), [Snap25](http://flybase.bio.indiana.edu/.bin/fbidq.html?Snap25), [Fer2](http://flybase.bio.indiana.edu/.bin/fbidq.html?Fer2),[CG34400](http://flybase.bio.indiana.edu/.bin/fbidq.html?CG34400), [Ulp1](http://flybase.bio.indiana.edu/.bin/fbidq.html?Ulp1), [mGluRA](http://flybase.bio.indiana.edu/.bin/fbidq.html?mGluRA), [ena](http://flybase.bio.indiana.edu/.bin/fbidq.html?ena), [dsh](http://flybase.bio.indiana.edu/.bin/fbidq.html?dsh), [Nf-YC](http://flybase.bio.indiana.edu/.bin/fbidq.html?Nf-YC), [dpr20](http://flybase.bio.indiana.edu/.bin/fbidq.html?dpr20), [qkr58E-1](http://flybase.bio.indiana.edu/.bin/fbidq.html?qkr58E-1), [comt](http://flybase.bio.indiana.edu/.bin/fbidq.html?comt), [Pbprp1](http://flybase.bio.indiana.edu/.bin/fbidq.html?Pbprp1), [PP2A-B](http://flybase.bio.indiana.edu/.bin/fbidq.html?PP2A-B), [qvr](http://flybase.bio.indiana.edu/.bin/fbidq.html?qvr), [DAAM](http://flybase.bio.indiana.edu/.bin/fbidq.html?DAAM), [rho-5](http://flybase.bio.indiana.edu/.bin/fbidq.html?rho-5), [nmo](http://flybase.bio.indiana.edu/.bin/fbidq.html?nmo), [5-HT1A](http://flybase.bio.indiana.edu/.bin/fbidq.html?5-HT1A), [Psi](http://flybase.bio.indiana.edu/.bin/fbidq.html?Psi), [Gug](http://flybase.bio.indiana.edu/.bin/fbidq.html?Gug), [Actn](http://flybase.bio.indiana.edu/.bin/fbidq.html?Actn), [ed](http://flybase.bio.indiana.edu/.bin/fbidq.html?ed), [pad](http://flybase.bio.indiana.edu/.bin/fbidq.html?pad), [CG11317](http://flybase.bio.indiana.edu/.bin/fbidq.html?CG11317), [daw](http://flybase.bio.indiana.edu/.bin/fbidq.html?daw), [Fhos](http://flybase.bio.indiana.edu/.bin/fbidq.html?Fhos), [Rx](http://flybase.bio.indiana.edu/.bin/fbidq.html?Rx), [rogdi](http://flybase.bio.indiana.edu/.bin/fbidq.html?rogdi), [salr](http://flybase.bio.indiana.edu/.bin/fbidq.html?salr), [Apc](http://flybase.bio.indiana.edu/.bin/fbidq.html?Apc), [VGAT](http://flybase.bio.indiana.edu/.bin/fbidq.html?VGAT), [d4](http://flybase.bio.indiana.edu/.bin/fbidq.html?d4), [dan](http://flybase.bio.indiana.edu/.bin/fbidq.html?dan), [cort](http://flybase.bio.indiana.edu/.bin/fbidq.html?cort), [tau](http://flybase.bio.indiana.edu/.bin/fbidq.html?tau), [RN-tre](http://flybase.bio.indiana.edu/.bin/fbidq.html?RN-tre), [Mf](http://flybase.bio.indiana.edu/.bin/fbidq.html?Mf), [wnd](http://flybase.bio.indiana.edu/.bin/fbidq.html?wnd), [trx](http://flybase.bio.indiana.edu/.bin/fbidq.html?trx), [rdo](http://flybase.bio.indiana.edu/.bin/fbidq.html?rdo),[Btk29A](http://flybase.bio.indiana.edu/.bin/fbidq.html?Btk29A), [SMC2](http://flybase.bio.indiana.edu/.bin/fbidq.html?SMC2), [PQBP-1](http://flybase.bio.indiana.edu/.bin/fbidq.html?PQBP-1), [MED25](http://flybase.bio.indiana.edu/.bin/fbidq.html?MED25), [ETH](http://flybase.bio.indiana.edu/.bin/fbidq.html?ETH), [unc-5](http://flybase.bio.indiana.edu/.bin/fbidq.html?unc-5), [cdi](http://flybase.bio.indiana.edu/.bin/fbidq.html?cdi), [vfl](http://flybase.bio.indiana.edu/.bin/fbidq.html?vfl), [barr](http://flybase.bio.indiana.edu/.bin/fbidq.html?barr), [NPFR1](http://flybase.bio.indiana.edu/.bin/fbidq.html?NPFR1), [pygo](http://flybase.bio.indiana.edu/.bin/fbidq.html?pygo), [HLHmgamma](http://flybase.bio.indiana.edu/.bin/fbidq.html?HLHmgamma), [spag](http://flybase.bio.indiana.edu/.bin/fbidq.html?spag), [jar](http://flybase.bio.indiana.edu/.bin/fbidq.html?jar), [Klp68D](http://flybase.bio.indiana.edu/.bin/fbidq.html?Klp68D), [Ror](http://flybase.bio.indiana.edu/.bin/fbidq.html?Ror), [wor](http://flybase.bio.indiana.edu/.bin/fbidq.html?wor), [Atg1](http://flybase.bio.indiana.edu/.bin/fbidq.html?Atg1), [Fcp1](http://flybase.bio.indiana.edu/.bin/fbidq.html?Fcp1), [Ptp69D](http://flybase.bio.indiana.edu/.bin/fbidq.html?Ptp69D), [hts](http://flybase.bio.indiana.edu/.bin/fbidq.html?hts), [dpr9](http://flybase.bio.indiana.edu/.bin/fbidq.html?dpr9), [pUf68](http://flybase.bio.indiana.edu/.bin/fbidq.html?pUf68), [bowl](http://flybase.bio.indiana.edu/.bin/fbidq.html?bowl), [seq](http://flybase.bio.indiana.edu/.bin/fbidq.html?seq), [tay](http://flybase.bio.indiana.edu/.bin/fbidq.html?tay), [CG1671](http://flybase.bio.indiana.edu/.bin/fbidq.html?CG1671), [Cbl](http://flybase.bio.indiana.edu/.bin/fbidq.html?Cbl), [thr](http://flybase.bio.indiana.edu/.bin/fbidq.html?thr), [pog](http://flybase.bio.indiana.edu/.bin/fbidq.html?pog), [CASK](http://flybase.bio.indiana.edu/.bin/fbidq.html?CASK), [CG10251](http://flybase.bio.indiana.edu/.bin/fbidq.html?CG10251), [mtt](http://flybase.bio.indiana.edu/.bin/fbidq.html?mtt), [disp](http://flybase.bio.indiana.edu/.bin/fbidq.html?disp), [mip120](http://flybase.bio.indiana.edu/.bin/fbidq.html?mip120), [cpx](http://flybase.bio.indiana.edu/.bin/fbidq.html?cpx), [jumu](http://flybase.bio.indiana.edu/.bin/fbidq.html?jumu),[Su(var)2-HP2](http://flybase.bio.indiana.edu/.bin/fbidq.html?Su(var)2-HP2), [gfA](http://flybase.bio.indiana.edu/.bin/fbidq.html?gfA), [fd59A](http://flybase.bio.indiana.edu/.bin/fbidq.html?fd59A), [beat-Ic](http://flybase.bio.indiana.edu/.bin/fbidq.html?beat-Ic), [Rph](http://flybase.bio.indiana.edu/.bin/fbidq.html?Rph), [brat](http://flybase.bio.indiana.edu/.bin/fbidq.html?brat), [ST6Gal](http://flybase.bio.indiana.edu/.bin/fbidq.html?ST6Gal), [dpr14](http://flybase.bio.indiana.edu/.bin/fbidq.html?dpr14), [GABA-B-R2](http://flybase.bio.indiana.edu/.bin/fbidq.html?GABA-B-R2), [CG7051](http://flybase.bio.indiana.edu/.bin/fbidq.html?CG7051), [futsch](http://flybase.bio.indiana.edu/.bin/fbidq.html?futsch), [Ubx](http://flybase.bio.indiana.edu/.bin/fbidq.html?Ubx), [gukh](http://flybase.bio.indiana.edu/.bin/fbidq.html?gukh), [chm](http://flybase.bio.indiana.edu/.bin/fbidq.html?chm), [Rim](http://flybase.bio.indiana.edu/.bin/fbidq.html?Rim), [beat-IIa](http://flybase.bio.indiana.edu/.bin/fbidq.html?beat-IIa), [CG42574](http://flybase.bio.indiana.edu/.bin/fbidq.html?CG42574), [dac](http://flybase.bio.indiana.edu/.bin/fbidq.html?dac), [Nrx-1](http://flybase.bio.indiana.edu/.bin/fbidq.html?Nrx-1), [CG9007](http://flybase.bio.indiana.edu/.bin/fbidq.html?CG9007), [CG33275](http://flybase.bio.indiana.edu/.bin/fbidq.html?CG33275), [ventrally-expressed-protein-D](http://flybase.bio.indiana.edu/.bin/fbidq.html?ventrally-expressed-protein-D), [toy](http://flybase.bio.indiana.edu/.bin/fbidq.html?toy), [Lim3](http://flybase.bio.indiana.edu/.bin/fbidq.html?Lim3), [sd](http://flybase.bio.indiana.edu/.bin/fbidq.html?sd), [Aplip1](http://flybase.bio.indiana.edu/.bin/fbidq.html?Aplip1), [CG1239](http://flybase.bio.indiana.edu/.bin/fbidq.html?CG1239), [fs(1)h](http://flybase.bio.indiana.edu/.bin/fbidq.html?fs(1)h),[Eip63F-1](http://flybase.bio.indiana.edu/.bin/fbidq.html?Eip63F-1), [yrt](http://flybase.bio.indiana.edu/.bin/fbidq.html?yrt), [dpr7](http://flybase.bio.indiana.edu/.bin/fbidq.html?dpr7), [wls](http://flybase.bio.indiana.edu/.bin/fbidq.html?wls), [hiw](http://flybase.bio.indiana.edu/.bin/fbidq.html?hiw), [CG32653](http://flybase.bio.indiana.edu/.bin/fbidq.html?CG32653), [Atf-2](http://flybase.bio.indiana.edu/.bin/fbidq.html?Atf-2), [n-syb](http://flybase.bio.indiana.edu/.bin/fbidq.html?n-syb), [skd](http://flybase.bio.indiana.edu/.bin/fbidq.html?skd), [jing](http://flybase.bio.indiana.edu/.bin/fbidq.html?jing), [katanin-60](http://flybase.bio.indiana.edu/.bin/fbidq.html?katanin-60), [tll](http://flybase.bio.indiana.edu/.bin/fbidq.html?tll), [ato](http://flybase.bio.indiana.edu/.bin/fbidq.html?ato), [Fmrf](http://flybase.bio.indiana.edu/.bin/fbidq.html?Fmrf), [Iswi](http://flybase.bio.indiana.edu/.bin/fbidq.html?Iswi), [CG5742](http://flybase.bio.indiana.edu/.bin/fbidq.html?CG5742), [obst-A](http://flybase.bio.indiana.edu/.bin/fbidq.html?obst-A), [Pka-R1](http://flybase.bio.indiana.edu/.bin/fbidq.html?Pka-R1), [CG14478](http://flybase.bio.indiana.edu/.bin/fbidq.html?CG14478), [CG9867](http://flybase.bio.indiana.edu/.bin/fbidq.html?CG9867), [mago](http://flybase.bio.indiana.edu/.bin/fbidq.html?mago), [enc](http://flybase.bio.indiana.edu/.bin/fbidq.html?enc), [cuff](http://flybase.bio.indiana.edu/.bin/fbidq.html?cuff), [ksr](http://flybase.bio.indiana.edu/.bin/fbidq.html?ksr), [brp](http://flybase.bio.indiana.edu/.bin/fbidq.html?brp), [chn](http://flybase.bio.indiana.edu/.bin/fbidq.html?chn), [Khc-73](http://flybase.bio.indiana.edu/.bin/fbidq.html?Khc-73), [chif](http://flybase.bio.indiana.edu/.bin/fbidq.html?chif), [p130CAS](http://flybase.bio.indiana.edu/.bin/fbidq.html?p130CAS), [CG7033](http://flybase.bio.indiana.edu/.bin/fbidq.html?CG7033), [CG30007](http://flybase.bio.indiana.edu/.bin/fbidq.html?CG30007), [ewg](http://flybase.bio.indiana.edu/.bin/fbidq.html?ewg), [CG13550](http://flybase.bio.indiana.edu/.bin/fbidq.html?CG13550), [sano](http://flybase.bio.indiana.edu/.bin/fbidq.html?sano), [Cirl](http://flybase.bio.indiana.edu/.bin/fbidq.html?Cirl),[l(2)NC136](http://flybase.bio.indiana.edu/.bin/fbidq.html?l(2)NC136), [Su(var)3-3](http://flybase.bio.indiana.edu/.bin/fbidq.html?Su(var)3-3), [gogo](http://flybase.bio.indiana.edu/.bin/fbidq.html?gogo), [unc-13-4A](http://flybase.bio.indiana.edu/.bin/fbidq.html?unc-13-4A), [GluClalpha](http://flybase.bio.indiana.edu/.bin/fbidq.html?GluClalpha), [Sema-1a](http://flybase.bio.indiana.edu/.bin/fbidq.html?Sema-1a), [dpr15](http://flybase.bio.indiana.edu/.bin/fbidq.html?dpr15), [Eh](http://flybase.bio.indiana.edu/.bin/fbidq.html?Eh), [Hs3st-B](http://flybase.bio.indiana.edu/.bin/fbidq.html?Hs3st-B), [Sos](http://flybase.bio.indiana.edu/.bin/fbidq.html?Sos), [Fas2](http://flybase.bio.indiana.edu/.bin/fbidq.html?Fas2), [Klp61F](http://flybase.bio.indiana.edu/.bin/fbidq.html?Klp61F), [eya](http://flybase.bio.indiana.edu/.bin/fbidq.html?eya), [CG8557](http://flybase.bio.indiana.edu/.bin/fbidq.html?CG8557), [beat-Ia](http://flybase.bio.indiana.edu/.bin/fbidq.html?beat-Ia), [kat-60L1](http://flybase.bio.indiana.edu/.bin/fbidq.html?kat-60L1), [mtg](http://flybase.bio.indiana.edu/.bin/fbidq.html?mtg), [ft](http://flybase.bio.indiana.edu/.bin/fbidq.html?ft), [CG12004](http://flybase.bio.indiana.edu/.bin/fbidq.html?CG12004), [l(2)k16918](http://flybase.bio.indiana.edu/.bin/fbidq.html?l(2)k16918), [sws](http://flybase.bio.indiana.edu/.bin/fbidq.html?sws), [Dll](http://flybase.bio.indiana.edu/.bin/fbidq.html?Dll), [disco](http://flybase.bio.indiana.edu/.bin/fbidq.html?disco), [DopR](http://flybase.bio.indiana.edu/.bin/fbidq.html?DopR), [mew](http://flybase.bio.indiana.edu/.bin/fbidq.html?mew), [didum](http://flybase.bio.indiana.edu/.bin/fbidq.html?didum), [Lis-1](http://flybase.bio.indiana.edu/.bin/fbidq.html?Lis-1), [bmm](http://flybase.bio.indiana.edu/.bin/fbidq.html?bmm), [ems](http://flybase.bio.indiana.edu/.bin/fbidq.html?ems), [eys](http://flybase.bio.indiana.edu/.bin/fbidq.html?eys), [tup](http://flybase.bio.indiana.edu/.bin/fbidq.html?tup), [Rab3](http://flybase.bio.indiana.edu/.bin/fbidq.html?Rab3),[trio](http://flybase.bio.indiana.edu/.bin/fbidq.html?trio), [pdm2](http://flybase.bio.indiana.edu/.bin/fbidq.html?pdm2), [CG31659](http://flybase.bio.indiana.edu/.bin/fbidq.html?CG31659), [CG31665](http://flybase.bio.indiana.edu/.bin/fbidq.html?CG31665), [Gad1](http://flybase.bio.indiana.edu/.bin/fbidq.html?Gad1), [CaMKII](http://flybase.bio.indiana.edu/.bin/fbidq.html?CaMKII), [nerfin-1](http://flybase.bio.indiana.edu/.bin/fbidq.html?nerfin-1), [pigs](http://flybase.bio.indiana.edu/.bin/fbidq.html?pigs), [5-HT7](http://flybase.bio.indiana.edu/.bin/fbidq.html?5-HT7), [polo](http://flybase.bio.indiana.edu/.bin/fbidq.html?polo), [chinmo](http://flybase.bio.indiana.edu/.bin/fbidq.html?chinmo), [Syn1](http://flybase.bio.indiana.edu/.bin/fbidq.html?Syn1), [Ssdp](http://flybase.bio.indiana.edu/.bin/fbidq.html?Ssdp), [CG8155](http://flybase.bio.indiana.edu/.bin/fbidq.html?CG8155), [fend](http://flybase.bio.indiana.edu/.bin/fbidq.html?fend), [sr](http://flybase.bio.indiana.edu/.bin/fbidq.html?sr), [Lim1](http://flybase.bio.indiana.edu/.bin/fbidq.html?Lim1), [nkd](http://flybase.bio.indiana.edu/.bin/fbidq.html?nkd), [danr](http://flybase.bio.indiana.edu/.bin/fbidq.html?danr), [Dsk](http://flybase.bio.indiana.edu/.bin/fbidq.html?Dsk), [vvl](http://flybase.bio.indiana.edu/.bin/fbidq.html?vvl), [Unc-76](http://flybase.bio.indiana.edu/.bin/fbidq.html?Unc-76), [betaInt-nu](http://flybase.bio.indiana.edu/.bin/fbidq.html?betaInt-nu), [sgg](http://flybase.bio.indiana.edu/.bin/fbidq.html?sgg), [Brd](http://flybase.bio.indiana.edu/.bin/fbidq.html?Brd), [mnb](http://flybase.bio.indiana.edu/.bin/fbidq.html?mnb), [Nmdar1](http://flybase.bio.indiana.edu/.bin/fbidq.html?Nmdar1), [Eip93F](http://flybase.bio.indiana.edu/.bin/fbidq.html?Eip93F), [gcm](http://flybase.bio.indiana.edu/.bin/fbidq.html?gcm), [dpr10](http://flybase.bio.indiana.edu/.bin/fbidq.html?dpr10), [Ccn](http://flybase.bio.indiana.edu/.bin/fbidq.html?Ccn), [ase](http://flybase.bio.indiana.edu/.bin/fbidq.html?ase), [elav](http://flybase.bio.indiana.edu/.bin/fbidq.html?elav), [Poxn](http://flybase.bio.indiana.edu/.bin/fbidq.html?Poxn), [Dat](http://flybase.bio.indiana.edu/.bin/fbidq.html?Dat), [dpr3](http://flybase.bio.indiana.edu/.bin/fbidq.html?dpr3),[Rat1](http://flybase.bio.indiana.edu/.bin/fbidq.html?Rat1), [CG18273](http://flybase.bio.indiana.edu/.bin/fbidq.html?CG18273), [eIF4G2](http://flybase.bio.indiana.edu/.bin/fbidq.html?eIF4G2), [fred](http://flybase.bio.indiana.edu/.bin/fbidq.html?fred), [ush](http://flybase.bio.indiana.edu/.bin/fbidq.html?ush), [Fak56D](http://flybase.bio.indiana.edu/.bin/fbidq.html?Fak56D), [CG16778](http://flybase.bio.indiana.edu/.bin/fbidq.html?CG16778), [Kap3](http://flybase.bio.indiana.edu/.bin/fbidq.html?Kap3), [ko](http://flybase.bio.indiana.edu/.bin/fbidq.html?ko), [dpr17](http://flybase.bio.indiana.edu/.bin/fbidq.html?dpr17), [Eip71CD](http://flybase.bio.indiana.edu/.bin/fbidq.html?Eip71CD), [Alh](http://flybase.bio.indiana.edu/.bin/fbidq.html?Alh), [egg](http://flybase.bio.indiana.edu/.bin/fbidq.html?egg), [rtet](http://flybase.bio.indiana.edu/.bin/fbidq.html?rtet), [CG12424](http://flybase.bio.indiana.edu/.bin/fbidq.html?CG12424), [Sp1](http://flybase.bio.indiana.edu/.bin/fbidq.html?Sp1), [GlcAT-P](http://flybase.bio.indiana.edu/.bin/fbidq.html?GlcAT-P), [CG3967](http://flybase.bio.indiana.edu/.bin/fbidq.html?CG3967), [Wnt5](http://flybase.bio.indiana.edu/.bin/fbidq.html?Wnt5), [SF2](http://flybase.bio.indiana.edu/.bin/fbidq.html?SF2), [sif](http://flybase.bio.indiana.edu/.bin/fbidq.html?sif), [Tbh](http://flybase.bio.indiana.edu/.bin/fbidq.html?Tbh), [Aac11](http://flybase.bio.indiana.edu/.bin/fbidq.html?Aac11), [Sas-4](http://flybase.bio.indiana.edu/.bin/fbidq.html?Sas-4), [ssh](http://flybase.bio.indiana.edu/.bin/fbidq.html?ssh), [PIP5K59B](http://flybase.bio.indiana.edu/.bin/fbidq.html?PIP5K59B), [mle](http://flybase.bio.indiana.edu/.bin/fbidq.html?mle), [Hr51](http://flybase.bio.indiana.edu/.bin/fbidq.html?Hr51), [lap](http://flybase.bio.indiana.edu/.bin/fbidq.html?lap), [CG11155](http://flybase.bio.indiana.edu/.bin/fbidq.html?CG11155), [wg](http://flybase.bio.indiana.edu/.bin/fbidq.html?wg), [fkh](http://flybase.bio.indiana.edu/.bin/fbidq.html?fkh), [RpL28](http://flybase.bio.indiana.edu/.bin/fbidq.html?RpL28), [fne](http://flybase.bio.indiana.edu/.bin/fbidq.html?fne), [klg](http://flybase.bio.indiana.edu/.bin/fbidq.html?klg), [en](http://flybase.bio.indiana.edu/.bin/fbidq.html?en),[sfl](http://flybase.bio.indiana.edu/.bin/fbidq.html?sfl), [Sip1](http://flybase.bio.indiana.edu/.bin/fbidq.html?Sip1), [SerT](http://flybase.bio.indiana.edu/.bin/fbidq.html?SerT), [Utx](http://flybase.bio.indiana.edu/.bin/fbidq.html?Utx), [Rnp4F](http://flybase.bio.indiana.edu/.bin/fbidq.html?Rnp4F), [Ct](http://flybase.bio.indiana.edu/.bin/fbidq.html?Ct), [fz2](http://flybase.bio.indiana.edu/.bin/fbidq.html?fz2), [numb](http://flybase.bio.indiana.edu/.bin/fbidq.html?numb), [Ocho](http://flybase.bio.indiana.edu/.bin/fbidq.html?Ocho), [CG42256](http://flybase.bio.indiana.edu/.bin/fbidq.html?CG42256), [mus101](http://flybase.bio.indiana.edu/.bin/fbidq.html?mus101), [CG17734](http://flybase.bio.indiana.edu/.bin/fbidq.html?CG17734), [Sxl](http://flybase.bio.indiana.edu/.bin/fbidq.html?Sxl), [stj](http://flybase.bio.indiana.edu/.bin/fbidq.html?stj), [JIL-1](http://flybase.bio.indiana.edu/.bin/fbidq.html?JIL-1), [CG33007](http://flybase.bio.indiana.edu/.bin/fbidq.html?CG33007), [Nf1](http://flybase.bio.indiana.edu/.bin/fbidq.html?Nf1), [Drep-3](http://flybase.bio.indiana.edu/.bin/fbidq.html?Drep-3), [sol](http://flybase.bio.indiana.edu/.bin/fbidq.html?sol), [sns](http://flybase.bio.indiana.edu/.bin/fbidq.html?sns), [bi](http://flybase.bio.indiana.edu/.bin/fbidq.html?bi), [tok](http://flybase.bio.indiana.edu/.bin/fbidq.html?tok), [nonA](http://flybase.bio.indiana.edu/.bin/fbidq.html?nonA), [Sytalpha](http://flybase.bio.indiana.edu/.bin/fbidq.html?Sytalpha), [Ptp99A](http://flybase.bio.indiana.edu/.bin/fbidq.html?Ptp99A), [CG31961](http://flybase.bio.indiana.edu/.bin/fbidq.html?CG31961), [Tango10](http://flybase.bio.indiana.edu/.bin/fbidq.html?Tango10), [Oseg4](http://flybase.bio.indiana.edu/.bin/fbidq.html?Oseg4), [blue](http://flybase.bio.indiana.edu/.bin/fbidq.html?blue), [mod(mdg4)](http://flybase.bio.indiana.edu/.bin/fbidq.html?mod(mdg4)), [Taf12L](http://flybase.bio.indiana.edu/.bin/fbidq.html?Taf12L), [bab1](http://flybase.bio.indiana.edu/.bin/fbidq.html?bab1), [Galpha49B](http://flybase.bio.indiana.edu/.bin/fbidq.html?Galpha49B), [Snoo](http://flybase.bio.indiana.edu/.bin/fbidq.html?Snoo),[rut](http://flybase.bio.indiana.edu/.bin/fbidq.html?rut), [uif](http://flybase.bio.indiana.edu/.bin/fbidq.html?uif), [nub](http://flybase.bio.indiana.edu/.bin/fbidq.html?nub), [btd](http://flybase.bio.indiana.edu/.bin/fbidq.html?btd), [Mmp2](http://flybase.bio.indiana.edu/.bin/fbidq.html?Mmp2), [amon](http://flybase.bio.indiana.edu/.bin/fbidq.html?amon), [fd68A](http://flybase.bio.indiana.edu/.bin/fbidq.html?fd68A), [opa1-like](http://flybase.bio.indiana.edu/.bin/fbidq.html?opa1-like), [B-H1](http://flybase.bio.indiana.edu/.bin/fbidq.html?B-H1), [pim](http://flybase.bio.indiana.edu/.bin/fbidq.html?pim), [CG32206](http://flybase.bio.indiana.edu/.bin/fbidq.html?CG32206), [DAT](http://flybase.bio.indiana.edu/.bin/fbidq.html?DAT), [Nipped-A](http://flybase.bio.indiana.edu/.bin/fbidq.html?Nipped-A), [fd102C](http://flybase.bio.indiana.edu/.bin/fbidq.html?fd102C), [hth](http://flybase.bio.indiana.edu/.bin/fbidq.html?hth), [14-3-3zeta](http://flybase.bio.indiana.edu/.bin/fbidq.html?14-3-3zeta), [pzg](http://flybase.bio.indiana.edu/.bin/fbidq.html?pzg), [lea](http://flybase.bio.indiana.edu/.bin/fbidq.html?lea), [shakB](http://flybase.bio.indiana.edu/.bin/fbidq.html?shakB), [plexB](http://flybase.bio.indiana.edu/.bin/fbidq.html?plexB), [loqs](http://flybase.bio.indiana.edu/.bin/fbidq.html?loqs), [Klp64D](http://flybase.bio.indiana.edu/.bin/fbidq.html?Klp64D), [neur](http://flybase.bio.indiana.edu/.bin/fbidq.html?neur), [Brf](http://flybase.bio.indiana.edu/.bin/fbidq.html?Brf), [CycE](http://flybase.bio.indiana.edu/.bin/fbidq.html?CycE), [CG32149](http://flybase.bio.indiana.edu/.bin/fbidq.html?CG32149), [mr](http://flybase.bio.indiana.edu/.bin/fbidq.html?mr), [orb2](http://flybase.bio.indiana.edu/.bin/fbidq.html?orb2), [ImpE2](http://flybase.bio.indiana.edu/.bin/fbidq.html?ImpE2), [synaptogyrin](http://flybase.bio.indiana.edu/.bin/fbidq.html?synaptogyrin), [sim](http://flybase.bio.indiana.edu/.bin/fbidq.html?sim), [gro](http://flybase.bio.indiana.edu/.bin/fbidq.html?gro), [Rya-r44F](http://flybase.bio.indiana.edu/.bin/fbidq.html?Rya-r44F), [scyl](http://flybase.bio.indiana.edu/.bin/fbidq.html?scyl), [Rpd3](http://flybase.bio.indiana.edu/.bin/fbidq.html?Rpd3),[CG10107](http://flybase.bio.indiana.edu/.bin/fbidq.html?CG10107), [aret](http://flybase.bio.indiana.edu/.bin/fbidq.html?aret), [CG3662](http://flybase.bio.indiana.edu/.bin/fbidq.html?CG3662), [HLHm7](http://flybase.bio.indiana.edu/.bin/fbidq.html?HLHm7), [tow](http://flybase.bio.indiana.edu/.bin/fbidq.html?tow), [CG15609](http://flybase.bio.indiana.edu/.bin/fbidq.html?CG15609), [sei](http://flybase.bio.indiana.edu/.bin/fbidq.html?sei), [l(1)G0148](http://flybase.bio.indiana.edu/.bin/fbidq.html?l(1)G0148), [CG12187](http://flybase.bio.indiana.edu/.bin/fbidq.html?CG12187), [Pten](http://flybase.bio.indiana.edu/.bin/fbidq.html?Pten), [unc-13](http://flybase.bio.indiana.edu/.bin/fbidq.html?unc-13), [npf](http://flybase.bio.indiana.edu/.bin/fbidq.html?npf), [dpr8](http://flybase.bio.indiana.edu/.bin/fbidq.html?dpr8), [mira](http://flybase.bio.indiana.edu/.bin/fbidq.html?mira), [Myo10A](http://flybase.bio.indiana.edu/.bin/fbidq.html?Myo10A), [Sug](http://flybase.bio.indiana.edu/.bin/fbidq.html?Sug), [Drep-2](http://flybase.bio.indiana.edu/.bin/fbidq.html?Drep-2), [Dip3](http://flybase.bio.indiana.edu/.bin/fbidq.html?Dip3), [oa2](http://flybase.bio.indiana.edu/.bin/fbidq.html?oa2), [RpS11](http://flybase.bio.indiana.edu/.bin/fbidq.html?RpS11), [phl](http://flybase.bio.indiana.edu/.bin/fbidq.html?phl), [hang](http://flybase.bio.indiana.edu/.bin/fbidq.html?hang), [CG4893](http://flybase.bio.indiana.edu/.bin/fbidq.html?CG4893), [sNPF](http://flybase.bio.indiana.edu/.bin/fbidq.html?sNPF), [CG9098](http://flybase.bio.indiana.edu/.bin/fbidq.html?CG9098), [CG7708](http://flybase.bio.indiana.edu/.bin/fbidq.html?CG7708), [Alk](http://flybase.bio.indiana.edu/.bin/fbidq.html?Alk), [Vang](http://flybase.bio.indiana.edu/.bin/fbidq.html?Vang), [esn](http://flybase.bio.indiana.edu/.bin/fbidq.html?esn), [pot](http://flybase.bio.indiana.edu/.bin/fbidq.html?pot), [Pde1c](http://flybase.bio.indiana.edu/.bin/fbidq.html?Pde1c), [Sin3A](http://flybase.bio.indiana.edu/.bin/fbidq.html?Sin3A), [Smox](http://flybase.bio.indiana.edu/.bin/fbidq.html?Smox),[Shaw](http://flybase.bio.indiana.edu/.bin/fbidq.html?Shaw), [CG31475](http://flybase.bio.indiana.edu/.bin/fbidq.html?CG31475), [Plc21C](http://flybase.bio.indiana.edu/.bin/fbidq.html?Plc21C), [srpk79D](http://flybase.bio.indiana.edu/.bin/fbidq.html?srpk79D), [Antp](http://flybase.bio.indiana.edu/.bin/fbidq.html?Antp), [Rdl](http://flybase.bio.indiana.edu/.bin/fbidq.html?Rdl), [glec](http://flybase.bio.indiana.edu/.bin/fbidq.html?glec), [l(3)neo38](http://flybase.bio.indiana.edu/.bin/fbidq.html?l(3)neo38), [Ast-C](http://flybase.bio.indiana.edu/.bin/fbidq.html?Ast-C), [cindr](http://flybase.bio.indiana.edu/.bin/fbidq.html?cindr), [Ptx1](http://flybase.bio.indiana.edu/.bin/fbidq.html?Ptx1), [comm3](http://flybase.bio.indiana.edu/.bin/fbidq.html?comm3), [CG11071](http://flybase.bio.indiana.edu/.bin/fbidq.html?CG11071), [CG6114](http://flybase.bio.indiana.edu/.bin/fbidq.html?CG6114), [Shal](http://flybase.bio.indiana.edu/.bin/fbidq.html?Shal), [hug](http://flybase.bio.indiana.edu/.bin/fbidq.html?hug), [pigeon](http://flybase.bio.indiana.edu/.bin/fbidq.html?pigeon), [Gpo-1](http://flybase.bio.indiana.edu/.bin/fbidq.html?Gpo-1), [para](http://flybase.bio.indiana.edu/.bin/fbidq.html?para), [Syn2](http://flybase.bio.indiana.edu/.bin/fbidq.html?Syn2), [sm](http://flybase.bio.indiana.edu/.bin/fbidq.html?sm), [tsh](http://flybase.bio.indiana.edu/.bin/fbidq.html?tsh), [e(y)3](http://flybase.bio.indiana.edu/.bin/fbidq.html?e(y)3), [eas](http://flybase.bio.indiana.edu/.bin/fbidq.html?eas), [CRMP](http://flybase.bio.indiana.edu/.bin/fbidq.html?CRMP), [Sry-alpha](http://flybase.bio.indiana.edu/.bin/fbidq.html?Sry-alpha), [Cdk5](http://flybase.bio.indiana.edu/.bin/fbidq.html?Cdk5), [oc](http://flybase.bio.indiana.edu/.bin/fbidq.html?oc), [Cep135](http://flybase.bio.indiana.edu/.bin/fbidq.html?Cep135), [CG4641](http://flybase.bio.indiana.edu/.bin/fbidq.html?CG4641), [CG3964](http://flybase.bio.indiana.edu/.bin/fbidq.html?CG3964), [ey](http://flybase.bio.indiana.edu/.bin/fbidq.html?ey), [baz](http://flybase.bio.indiana.edu/.bin/fbidq.html?baz),[Oamb](http://flybase.bio.indiana.edu/.bin/fbidq.html?Oamb), [rst](http://flybase.bio.indiana.edu/.bin/fbidq.html?rst), [l(1)sc](http://flybase.bio.indiana.edu/.bin/fbidq.html?l(1)sc), [CG6398](http://flybase.bio.indiana.edu/.bin/fbidq.html?CG6398), [comm](http://flybase.bio.indiana.edu/.bin/fbidq.html?comm), [grh](http://flybase.bio.indiana.edu/.bin/fbidq.html?grh), [yemalpha](http://flybase.bio.indiana.edu/.bin/fbidq.html?yemalpha), [Cdk5alpha](http://flybase.bio.indiana.edu/.bin/fbidq.html?Cdk5alpha), [Vmat](http://flybase.bio.indiana.edu/.bin/fbidq.html?Vmat), [qtc](http://flybase.bio.indiana.edu/.bin/fbidq.html?qtc), [Cha](http://flybase.bio.indiana.edu/.bin/fbidq.html?Cha), [crol](http://flybase.bio.indiana.edu/.bin/fbidq.html?crol), [E(Pc)](http://flybase.bio.indiana.edu/.bin/fbidq.html?E(Pc)), [retn](http://flybase.bio.indiana.edu/.bin/fbidq.html?retn), [Syt1](http://flybase.bio.indiana.edu/.bin/fbidq.html?Syt1), [rl](http://flybase.bio.indiana.edu/.bin/fbidq.html?rl), [Nup153](http://flybase.bio.indiana.edu/.bin/fbidq.html?Nup153), [tey](http://flybase.bio.indiana.edu/.bin/fbidq.html?tey), [CG33960](http://flybase.bio.indiana.edu/.bin/fbidq.html?CG33960), [CG15765](http://flybase.bio.indiana.edu/.bin/fbidq.html?CG15765), [dpr18](http://flybase.bio.indiana.edu/.bin/fbidq.html?dpr18), [CG1962](http://flybase.bio.indiana.edu/.bin/fbidq.html?CG1962), [unk](http://flybase.bio.indiana.edu/.bin/fbidq.html?unk), [dnr1](http://flybase.bio.indiana.edu/.bin/fbidq.html?dnr1), [Pabp2](http://flybase.bio.indiana.edu/.bin/fbidq.html?Pabp2), [acj6](http://flybase.bio.indiana.edu/.bin/fbidq.html?acj6), [caup](http://flybase.bio.indiana.edu/.bin/fbidq.html?caup), [Cbp80](http://flybase.bio.indiana.edu/.bin/fbidq.html?Cbp80), [vimar](http://flybase.bio.indiana.edu/.bin/fbidq.html?vimar), [Dg](http://flybase.bio.indiana.edu/.bin/fbidq.html?Dg), [Trim9](http://flybase.bio.indiana.edu/.bin/fbidq.html?Trim9), [CG34127](http://flybase.bio.indiana.edu/.bin/fbidq.html?CG34127), [Evi5](http://flybase.bio.indiana.edu/.bin/fbidq.html?Evi5), [br](http://flybase.bio.indiana.edu/.bin/fbidq.html?br), [CG7154](http://flybase.bio.indiana.edu/.bin/fbidq.html?CG7154),[neo](http://flybase.bio.indiana.edu/.bin/fbidq.html?neo), [robo3](http://flybase.bio.indiana.edu/.bin/fbidq.html?robo3), [lola](http://flybase.bio.indiana.edu/.bin/fbidq.html?lola), [unc-104](http://flybase.bio.indiana.edu/.bin/fbidq.html?unc-104), [Pcl](http://flybase.bio.indiana.edu/.bin/fbidq.html?Pcl), [CG15312](http://flybase.bio.indiana.edu/.bin/fbidq.html?CG15312), [Spc105R](http://flybase.bio.indiana.edu/.bin/fbidq.html?Spc105R), [CG31158](http://flybase.bio.indiana.edu/.bin/fbidq.html?CG31158), [Awh](http://flybase.bio.indiana.edu/.bin/fbidq.html?Awh), [tap](http://flybase.bio.indiana.edu/.bin/fbidq.html?tap), [Appl](http://flybase.bio.indiana.edu/.bin/fbidq.html?Appl), [metro](http://flybase.bio.indiana.edu/.bin/fbidq.html?metro), [erm](http://flybase.bio.indiana.edu/.bin/fbidq.html?erm), [Nrk](http://flybase.bio.indiana.edu/.bin/fbidq.html?Nrk), [dmt](http://flybase.bio.indiana.edu/.bin/fbidq.html?dmt), [CG4328](http://flybase.bio.indiana.edu/.bin/fbidq.html?CG4328), [CG11399](http://flybase.bio.indiana.edu/.bin/fbidq.html?CG11399), [CG34002](http://flybase.bio.indiana.edu/.bin/fbidq.html?CG34002), [Ilp3](http://flybase.bio.indiana.edu/.bin/fbidq.html?Ilp3), [Taf4](http://flybase.bio.indiana.edu/.bin/fbidq.html?Taf4), [CG10249](http://flybase.bio.indiana.edu/.bin/fbidq.html?CG10249), [Nrg](http://flybase.bio.indiana.edu/.bin/fbidq.html?Nrg), [insc](http://flybase.bio.indiana.edu/.bin/fbidq.html?insc), [jeb](http://flybase.bio.indiana.edu/.bin/fbidq.html?jeb), [Actbeta](http://flybase.bio.indiana.edu/.bin/fbidq.html?Actbeta), [stau](http://flybase.bio.indiana.edu/.bin/fbidq.html?stau), [beat-Ib](http://flybase.bio.indiana.edu/.bin/fbidq.html?beat-Ib), [otk](http://flybase.bio.indiana.edu/.bin/fbidq.html?otk), [phyl](http://flybase.bio.indiana.edu/.bin/fbidq.html?phyl), [vg](http://flybase.bio.indiana.edu/.bin/fbidq.html?vg), [fz](http://flybase.bio.indiana.edu/.bin/fbidq.html?fz), [robo](http://flybase.bio.indiana.edu/.bin/fbidq.html?robo), [brm](http://flybase.bio.indiana.edu/.bin/fbidq.html?brm), [bsk](http://flybase.bio.indiana.edu/.bin/fbidq.html?bsk), [CG42268](http://flybase.bio.indiana.edu/.bin/fbidq.html?CG42268), [lft](http://flybase.bio.indiana.edu/.bin/fbidq.html?lft), [sno](http://flybase.bio.indiana.edu/.bin/fbidq.html?sno),[hig](http://flybase.bio.indiana.edu/.bin/fbidq.html?hig), [RhoGAPp190](http://flybase.bio.indiana.edu/.bin/fbidq.html?RhoGAPp190), [Sfmbt](http://flybase.bio.indiana.edu/.bin/fbidq.html?Sfmbt), [ara](http://flybase.bio.indiana.edu/.bin/fbidq.html?ara), [pHCl](http://flybase.bio.indiana.edu/.bin/fbidq.html?pHCl), [mam](http://flybase.bio.indiana.edu/.bin/fbidq.html?mam), [pan](http://flybase.bio.indiana.edu/.bin/fbidq.html?pan), [grn](http://flybase.bio.indiana.edu/.bin/fbidq.html?grn), [CG4019](http://flybase.bio.indiana.edu/.bin/fbidq.html?CG4019), [HLHm5](http://flybase.bio.indiana.edu/.bin/fbidq.html?HLHm5), [dap](http://flybase.bio.indiana.edu/.bin/fbidq.html?dap), [Bx](http://flybase.bio.indiana.edu/.bin/fbidq.html?Bx), [Mob2](http://flybase.bio.indiana.edu/.bin/fbidq.html?Mob2), [pros](http://flybase.bio.indiana.edu/.bin/fbidq.html?pros), [mthl8](http://flybase.bio.indiana.edu/.bin/fbidq.html?mthl8), [CG1463](http://flybase.bio.indiana.edu/.bin/fbidq.html?CG1463), [Ccap](http://flybase.bio.indiana.edu/.bin/fbidq.html?Ccap), [tipE](http://flybase.bio.indiana.edu/.bin/fbidq.html?tipE), [Wnt4](http://flybase.bio.indiana.edu/.bin/fbidq.html?Wnt4), [rictor](http://flybase.bio.indiana.edu/.bin/fbidq.html?rictor), [Glut4EF](http://flybase.bio.indiana.edu/.bin/fbidq.html?Glut4EF), [CG42629](http://flybase.bio.indiana.edu/.bin/fbidq.html?CG42629), [bchs](http://flybase.bio.indiana.edu/.bin/fbidq.html?bchs), [RpS5b](http://flybase.bio.indiana.edu/.bin/fbidq.html?RpS5b), [king-tubby](http://flybase.bio.indiana.edu/.bin/fbidq.html?king-tubby), [pk](http://flybase.bio.indiana.edu/.bin/fbidq.html?pk), [dsx](http://flybase.bio.indiana.edu/.bin/fbidq.html?dsx), [Dh](http://flybase.bio.indiana.edu/.bin/fbidq.html?Dh), [betaTub85D](http://flybase.bio.indiana.edu/.bin/fbidq.html?betaTub85D), [klu](http://flybase.bio.indiana.edu/.bin/fbidq.html?klu), [SPR](http://flybase.bio.indiana.edu/.bin/fbidq.html?SPR), [sqz](http://flybase.bio.indiana.edu/.bin/fbidq.html?sqz), [Cad74A](http://flybase.bio.indiana.edu/.bin/fbidq.html?Cad74A), [dpr11](http://flybase.bio.indiana.edu/.bin/fbidq.html?dpr11),[Ca-alpha1D](http://flybase.bio.indiana.edu/.bin/fbidq.html?Ca-alpha1D), [Atet](http://flybase.bio.indiana.edu/.bin/fbidq.html?Atet), [tld](http://flybase.bio.indiana.edu/.bin/fbidq.html?tld), [rg](http://flybase.bio.indiana.edu/.bin/fbidq.html?rg), [CG1909](http://flybase.bio.indiana.edu/.bin/fbidq.html?CG1909), [gcm2](http://flybase.bio.indiana.edu/.bin/fbidq.html?gcm2), [mp](http://flybase.bio.indiana.edu/.bin/fbidq.html?mp), [CG5890](http://flybase.bio.indiana.edu/.bin/fbidq.html?CG5890), [CanB](http://flybase.bio.indiana.edu/.bin/fbidq.html?CanB), [can](http://flybase.bio.indiana.edu/.bin/fbidq.html?can), [SP2353](http://flybase.bio.indiana.edu/.bin/fbidq.html?SP2353), [rab3-GEF](http://flybase.bio.indiana.edu/.bin/fbidq.html?rab3-GEF), [CG13253](http://flybase.bio.indiana.edu/.bin/fbidq.html?CG13253), [ovo](http://flybase.bio.indiana.edu/.bin/fbidq.html?ovo), [CG32685](http://flybase.bio.indiana.edu/.bin/fbidq.html?CG32685), [Dsp1](http://flybase.bio.indiana.edu/.bin/fbidq.html?Dsp1), [ds](http://flybase.bio.indiana.edu/.bin/fbidq.html?ds), [mei-P26](http://flybase.bio.indiana.edu/.bin/fbidq.html?mei-P26), [Syt4](http://flybase.bio.indiana.edu/.bin/fbidq.html?Syt4), [CG11033](http://flybase.bio.indiana.edu/.bin/fbidq.html?CG11033), [Incenp](http://flybase.bio.indiana.edu/.bin/fbidq.html?Incenp), [SIFR](http://flybase.bio.indiana.edu/.bin/fbidq.html?SIFR), [Rbp9](http://flybase.bio.indiana.edu/.bin/fbidq.html?Rbp9), [Drl-2](http://flybase.bio.indiana.edu/.bin/fbidq.html?Drl-2), [MYPT-75D](http://flybase.bio.indiana.edu/.bin/fbidq.html?MYPT-75D), [Tk](http://flybase.bio.indiana.edu/.bin/fbidq.html?Tk), [Ilp5](http://flybase.bio.indiana.edu/.bin/fbidq.html?Ilp5), [Gap1](http://flybase.bio.indiana.edu/.bin/fbidq.html?Gap1), [run](http://flybase.bio.indiana.edu/.bin/fbidq.html?run), [sNPF-R](http://flybase.bio.indiana.edu/.bin/fbidq.html?sNPF-R), [tara](http://flybase.bio.indiana.edu/.bin/fbidq.html?tara), [koko](http://flybase.bio.indiana.edu/.bin/fbidq.html?koko), [stan](http://flybase.bio.indiana.edu/.bin/fbidq.html?stan),[CanA-14F](http://flybase.bio.indiana.edu/.bin/fbidq.html?CanA-14F), [tutl](http://flybase.bio.indiana.edu/.bin/fbidq.html?tutl), [Ptth](http://flybase.bio.indiana.edu/.bin/fbidq.html?Ptth), [CG1753](http://flybase.bio.indiana.edu/.bin/fbidq.html?CG1753), [Ilp2](http://flybase.bio.indiana.edu/.bin/fbidq.html?Ilp2), [ph-p](http://flybase.bio.indiana.edu/.bin/fbidq.html?ph-p), [Frq1](http://flybase.bio.indiana.edu/.bin/fbidq.html?Frq1), [sna](http://flybase.bio.indiana.edu/.bin/fbidq.html?sna), [Bre1](http://flybase.bio.indiana.edu/.bin/fbidq.html?Bre1), [Taf1](http://flybase.bio.indiana.edu/.bin/fbidq.html?Taf1), [Ggamma1](http://flybase.bio.indiana.edu/.bin/fbidq.html?Ggamma1), [CG7597](http://flybase.bio.indiana.edu/.bin/fbidq.html?CG7597), [CG5466](http://flybase.bio.indiana.edu/.bin/fbidq.html?CG5466), [MBD-like](http://flybase.bio.indiana.edu/.bin/fbidq.html?MBD-like), [esg](http://flybase.bio.indiana.edu/.bin/fbidq.html?esg), [m4](http://flybase.bio.indiana.edu/.bin/fbidq.html?m4), [dom](http://flybase.bio.indiana.edu/.bin/fbidq.html?dom), [Dh31](http://flybase.bio.indiana.edu/.bin/fbidq.html?Dh31), [klar](http://flybase.bio.indiana.edu/.bin/fbidq.html?klar), [shi](http://flybase.bio.indiana.edu/.bin/fbidq.html?shi), [Spred](http://flybase.bio.indiana.edu/.bin/fbidq.html?Spred), [siz](http://flybase.bio.indiana.edu/.bin/fbidq.html?siz), [B-H2](http://flybase.bio.indiana.edu/.bin/fbidq.html?B-H2), [tyn](http://flybase.bio.indiana.edu/.bin/fbidq.html?tyn), [CG3822](http://flybase.bio.indiana.edu/.bin/fbidq.html?CG3822), [dpr5](http://flybase.bio.indiana.edu/.bin/fbidq.html?dpr5), [CadN](http://flybase.bio.indiana.edu/.bin/fbidq.html?CadN), [CG34139](http://flybase.bio.indiana.edu/.bin/fbidq.html?CG34139), [Mfap1](http://flybase.bio.indiana.edu/.bin/fbidq.html?Mfap1), [E(bx)](http://flybase.bio.indiana.edu/.bin/fbidq.html?E(bx)), [dpr19](http://flybase.bio.indiana.edu/.bin/fbidq.html?dpr19), [Dms](http://flybase.bio.indiana.edu/.bin/fbidq.html?Dms), [Snap](http://flybase.bio.indiana.edu/.bin/fbidq.html?Snap), [trn](http://flybase.bio.indiana.edu/.bin/fbidq.html?trn), [pburs](http://flybase.bio.indiana.edu/.bin/fbidq.html?pburs), [cos](http://flybase.bio.indiana.edu/.bin/fbidq.html?cos) |
| [single-multicellular organism process](http://amigo.geneontology.org/cgi-bin/amigo/go.cgi?view=details&query=GO:0044707) | 557 of 1594 genes, 34.9% | 2294 of 7634 genes, 30.0% | 4.57e-07 | 0.00% | 0.00 | [endoA](http://flybase.bio.indiana.edu/.bin/fbidq.html?endoA), [Sh](http://flybase.bio.indiana.edu/.bin/fbidq.html?Sh), [dock](http://flybase.bio.indiana.edu/.bin/fbidq.html?dock), [CG5808](http://flybase.bio.indiana.edu/.bin/fbidq.html?CG5808), [CG9004](http://flybase.bio.indiana.edu/.bin/fbidq.html?CG9004), [CG3703](http://flybase.bio.indiana.edu/.bin/fbidq.html?CG3703), [rn](http://flybase.bio.indiana.edu/.bin/fbidq.html?rn), [ap](http://flybase.bio.indiana.edu/.bin/fbidq.html?ap), [kkv](http://flybase.bio.indiana.edu/.bin/fbidq.html?kkv), [tlk](http://flybase.bio.indiana.edu/.bin/fbidq.html?tlk), [malpha](http://flybase.bio.indiana.edu/.bin/fbidq.html?malpha), [os](http://flybase.bio.indiana.edu/.bin/fbidq.html?os), [Axn](http://flybase.bio.indiana.edu/.bin/fbidq.html?Axn), [Traf6](http://flybase.bio.indiana.edu/.bin/fbidq.html?Traf6), [Leucokinin](http://flybase.bio.indiana.edu/.bin/fbidq.html?Leucokinin), [NetB](http://flybase.bio.indiana.edu/.bin/fbidq.html?NetB), [rdgC](http://flybase.bio.indiana.edu/.bin/fbidq.html?rdgC), [cid](http://flybase.bio.indiana.edu/.bin/fbidq.html?cid), [CG32137](http://flybase.bio.indiana.edu/.bin/fbidq.html?CG32137), [lute](http://flybase.bio.indiana.edu/.bin/fbidq.html?lute), [CG4476](http://flybase.bio.indiana.edu/.bin/fbidq.html?CG4476), [Ssrp](http://flybase.bio.indiana.edu/.bin/fbidq.html?Ssrp), [Lar](http://flybase.bio.indiana.edu/.bin/fbidq.html?Lar), [scrib](http://flybase.bio.indiana.edu/.bin/fbidq.html?scrib), [HGTX](http://flybase.bio.indiana.edu/.bin/fbidq.html?HGTX), [not](http://flybase.bio.indiana.edu/.bin/fbidq.html?not), [nvy](http://flybase.bio.indiana.edu/.bin/fbidq.html?nvy), [Ect3](http://flybase.bio.indiana.edu/.bin/fbidq.html?Ect3), [nab](http://flybase.bio.indiana.edu/.bin/fbidq.html?nab), [Ddc](http://flybase.bio.indiana.edu/.bin/fbidq.html?Ddc), [HLHmdelta](http://flybase.bio.indiana.edu/.bin/fbidq.html?HLHmdelta), [alph](http://flybase.bio.indiana.edu/.bin/fbidq.html?alph), [Traf4](http://flybase.bio.indiana.edu/.bin/fbidq.html?Traf4), [TBPH](http://flybase.bio.indiana.edu/.bin/fbidq.html?TBPH), [Snap25](http://flybase.bio.indiana.edu/.bin/fbidq.html?Snap25), [Fer2](http://flybase.bio.indiana.edu/.bin/fbidq.html?Fer2),[CG34400](http://flybase.bio.indiana.edu/.bin/fbidq.html?CG34400), [Ulp1](http://flybase.bio.indiana.edu/.bin/fbidq.html?Ulp1), [mGluRA](http://flybase.bio.indiana.edu/.bin/fbidq.html?mGluRA), [ena](http://flybase.bio.indiana.edu/.bin/fbidq.html?ena), [dsh](http://flybase.bio.indiana.edu/.bin/fbidq.html?dsh), [Nf-YC](http://flybase.bio.indiana.edu/.bin/fbidq.html?Nf-YC), [dpr20](http://flybase.bio.indiana.edu/.bin/fbidq.html?dpr20), [qkr58E-1](http://flybase.bio.indiana.edu/.bin/fbidq.html?qkr58E-1), [comt](http://flybase.bio.indiana.edu/.bin/fbidq.html?comt), [Pbprp1](http://flybase.bio.indiana.edu/.bin/fbidq.html?Pbprp1), [PP2A-B](http://flybase.bio.indiana.edu/.bin/fbidq.html?PP2A-B), [qvr](http://flybase.bio.indiana.edu/.bin/fbidq.html?qvr), [DAAM](http://flybase.bio.indiana.edu/.bin/fbidq.html?DAAM), [rho-5](http://flybase.bio.indiana.edu/.bin/fbidq.html?rho-5), [nmo](http://flybase.bio.indiana.edu/.bin/fbidq.html?nmo), [5-HT1A](http://flybase.bio.indiana.edu/.bin/fbidq.html?5-HT1A), [Gug](http://flybase.bio.indiana.edu/.bin/fbidq.html?Gug), [Actn](http://flybase.bio.indiana.edu/.bin/fbidq.html?Actn), [ed](http://flybase.bio.indiana.edu/.bin/fbidq.html?ed), [pad](http://flybase.bio.indiana.edu/.bin/fbidq.html?pad), [CG11317](http://flybase.bio.indiana.edu/.bin/fbidq.html?CG11317), [daw](http://flybase.bio.indiana.edu/.bin/fbidq.html?daw), [Fhos](http://flybase.bio.indiana.edu/.bin/fbidq.html?Fhos), [Rx](http://flybase.bio.indiana.edu/.bin/fbidq.html?Rx), [rogdi](http://flybase.bio.indiana.edu/.bin/fbidq.html?rogdi), [salr](http://flybase.bio.indiana.edu/.bin/fbidq.html?salr), [Apc](http://flybase.bio.indiana.edu/.bin/fbidq.html?Apc), [VGAT](http://flybase.bio.indiana.edu/.bin/fbidq.html?VGAT), [d4](http://flybase.bio.indiana.edu/.bin/fbidq.html?d4), [dan](http://flybase.bio.indiana.edu/.bin/fbidq.html?dan), [cort](http://flybase.bio.indiana.edu/.bin/fbidq.html?cort), [tau](http://flybase.bio.indiana.edu/.bin/fbidq.html?tau), [Mf](http://flybase.bio.indiana.edu/.bin/fbidq.html?Mf), [wnd](http://flybase.bio.indiana.edu/.bin/fbidq.html?wnd), [trx](http://flybase.bio.indiana.edu/.bin/fbidq.html?trx), [rdo](http://flybase.bio.indiana.edu/.bin/fbidq.html?rdo), [Btk29A](http://flybase.bio.indiana.edu/.bin/fbidq.html?Btk29A), [SMC2](http://flybase.bio.indiana.edu/.bin/fbidq.html?SMC2),[PQBP-1](http://flybase.bio.indiana.edu/.bin/fbidq.html?PQBP-1), [MED25](http://flybase.bio.indiana.edu/.bin/fbidq.html?MED25), [ETH](http://flybase.bio.indiana.edu/.bin/fbidq.html?ETH), [unc-5](http://flybase.bio.indiana.edu/.bin/fbidq.html?unc-5), [cdi](http://flybase.bio.indiana.edu/.bin/fbidq.html?cdi), [vfl](http://flybase.bio.indiana.edu/.bin/fbidq.html?vfl), [barr](http://flybase.bio.indiana.edu/.bin/fbidq.html?barr), [NPFR1](http://flybase.bio.indiana.edu/.bin/fbidq.html?NPFR1), [pygo](http://flybase.bio.indiana.edu/.bin/fbidq.html?pygo), [HLHmgamma](http://flybase.bio.indiana.edu/.bin/fbidq.html?HLHmgamma), [spag](http://flybase.bio.indiana.edu/.bin/fbidq.html?spag), [jar](http://flybase.bio.indiana.edu/.bin/fbidq.html?jar), [Klp68D](http://flybase.bio.indiana.edu/.bin/fbidq.html?Klp68D), [Ror](http://flybase.bio.indiana.edu/.bin/fbidq.html?Ror), [wor](http://flybase.bio.indiana.edu/.bin/fbidq.html?wor), [Atg1](http://flybase.bio.indiana.edu/.bin/fbidq.html?Atg1), [Fcp1](http://flybase.bio.indiana.edu/.bin/fbidq.html?Fcp1), [Ptp69D](http://flybase.bio.indiana.edu/.bin/fbidq.html?Ptp69D), [hts](http://flybase.bio.indiana.edu/.bin/fbidq.html?hts), [dpr9](http://flybase.bio.indiana.edu/.bin/fbidq.html?dpr9), [pUf68](http://flybase.bio.indiana.edu/.bin/fbidq.html?pUf68), [bowl](http://flybase.bio.indiana.edu/.bin/fbidq.html?bowl), [seq](http://flybase.bio.indiana.edu/.bin/fbidq.html?seq), [tay](http://flybase.bio.indiana.edu/.bin/fbidq.html?tay), [CG1671](http://flybase.bio.indiana.edu/.bin/fbidq.html?CG1671), [Cbl](http://flybase.bio.indiana.edu/.bin/fbidq.html?Cbl), [thr](http://flybase.bio.indiana.edu/.bin/fbidq.html?thr), [pog](http://flybase.bio.indiana.edu/.bin/fbidq.html?pog), [CASK](http://flybase.bio.indiana.edu/.bin/fbidq.html?CASK), [CG10251](http://flybase.bio.indiana.edu/.bin/fbidq.html?CG10251), [mtt](http://flybase.bio.indiana.edu/.bin/fbidq.html?mtt), [mip120](http://flybase.bio.indiana.edu/.bin/fbidq.html?mip120), [cpx](http://flybase.bio.indiana.edu/.bin/fbidq.html?cpx), [jumu](http://flybase.bio.indiana.edu/.bin/fbidq.html?jumu), [Su(var)2-HP2](http://flybase.bio.indiana.edu/.bin/fbidq.html?Su(var)2-HP2), [gfA](http://flybase.bio.indiana.edu/.bin/fbidq.html?gfA),[fd59A](http://flybase.bio.indiana.edu/.bin/fbidq.html?fd59A), [beat-Ic](http://flybase.bio.indiana.edu/.bin/fbidq.html?beat-Ic), [Rph](http://flybase.bio.indiana.edu/.bin/fbidq.html?Rph), [brat](http://flybase.bio.indiana.edu/.bin/fbidq.html?brat), [ST6Gal](http://flybase.bio.indiana.edu/.bin/fbidq.html?ST6Gal), [dpr14](http://flybase.bio.indiana.edu/.bin/fbidq.html?dpr14), [GABA-B-R2](http://flybase.bio.indiana.edu/.bin/fbidq.html?GABA-B-R2), [futsch](http://flybase.bio.indiana.edu/.bin/fbidq.html?futsch), [Ubx](http://flybase.bio.indiana.edu/.bin/fbidq.html?Ubx), [gukh](http://flybase.bio.indiana.edu/.bin/fbidq.html?gukh), [chm](http://flybase.bio.indiana.edu/.bin/fbidq.html?chm), [Rim](http://flybase.bio.indiana.edu/.bin/fbidq.html?Rim), [beat-IIa](http://flybase.bio.indiana.edu/.bin/fbidq.html?beat-IIa), [CG42574](http://flybase.bio.indiana.edu/.bin/fbidq.html?CG42574), [dac](http://flybase.bio.indiana.edu/.bin/fbidq.html?dac), [Nrx-1](http://flybase.bio.indiana.edu/.bin/fbidq.html?Nrx-1), [CG33275](http://flybase.bio.indiana.edu/.bin/fbidq.html?CG33275), [ventrally-expressed-protein-D](http://flybase.bio.indiana.edu/.bin/fbidq.html?ventrally-expressed-protein-D), [toy](http://flybase.bio.indiana.edu/.bin/fbidq.html?toy), [Lim3](http://flybase.bio.indiana.edu/.bin/fbidq.html?Lim3), [sd](http://flybase.bio.indiana.edu/.bin/fbidq.html?sd), [Aplip1](http://flybase.bio.indiana.edu/.bin/fbidq.html?Aplip1), [CG1239](http://flybase.bio.indiana.edu/.bin/fbidq.html?CG1239), [fs(1)h](http://flybase.bio.indiana.edu/.bin/fbidq.html?fs(1)h), [Eip63F-1](http://flybase.bio.indiana.edu/.bin/fbidq.html?Eip63F-1), [yrt](http://flybase.bio.indiana.edu/.bin/fbidq.html?yrt), [dpr7](http://flybase.bio.indiana.edu/.bin/fbidq.html?dpr7), [wls](http://flybase.bio.indiana.edu/.bin/fbidq.html?wls), [hiw](http://flybase.bio.indiana.edu/.bin/fbidq.html?hiw), [CG32653](http://flybase.bio.indiana.edu/.bin/fbidq.html?CG32653), [Atf-2](http://flybase.bio.indiana.edu/.bin/fbidq.html?Atf-2),[n-syb](http://flybase.bio.indiana.edu/.bin/fbidq.html?n-syb), [skd](http://flybase.bio.indiana.edu/.bin/fbidq.html?skd), [jing](http://flybase.bio.indiana.edu/.bin/fbidq.html?jing), [katanin-60](http://flybase.bio.indiana.edu/.bin/fbidq.html?katanin-60), [tll](http://flybase.bio.indiana.edu/.bin/fbidq.html?tll), [ato](http://flybase.bio.indiana.edu/.bin/fbidq.html?ato), [Fmrf](http://flybase.bio.indiana.edu/.bin/fbidq.html?Fmrf), [Iswi](http://flybase.bio.indiana.edu/.bin/fbidq.html?Iswi), [CG5742](http://flybase.bio.indiana.edu/.bin/fbidq.html?CG5742), [obst-A](http://flybase.bio.indiana.edu/.bin/fbidq.html?obst-A), [Pka-R1](http://flybase.bio.indiana.edu/.bin/fbidq.html?Pka-R1), [CG14478](http://flybase.bio.indiana.edu/.bin/fbidq.html?CG14478), [CG9867](http://flybase.bio.indiana.edu/.bin/fbidq.html?CG9867), [mago](http://flybase.bio.indiana.edu/.bin/fbidq.html?mago), [enc](http://flybase.bio.indiana.edu/.bin/fbidq.html?enc), [cuff](http://flybase.bio.indiana.edu/.bin/fbidq.html?cuff), [ksr](http://flybase.bio.indiana.edu/.bin/fbidq.html?ksr), [brp](http://flybase.bio.indiana.edu/.bin/fbidq.html?brp), [chn](http://flybase.bio.indiana.edu/.bin/fbidq.html?chn), [Khc-73](http://flybase.bio.indiana.edu/.bin/fbidq.html?Khc-73), [chif](http://flybase.bio.indiana.edu/.bin/fbidq.html?chif), [p130CAS](http://flybase.bio.indiana.edu/.bin/fbidq.html?p130CAS), [CG7033](http://flybase.bio.indiana.edu/.bin/fbidq.html?CG7033), [CG30007](http://flybase.bio.indiana.edu/.bin/fbidq.html?CG30007), [ewg](http://flybase.bio.indiana.edu/.bin/fbidq.html?ewg), [CG13550](http://flybase.bio.indiana.edu/.bin/fbidq.html?CG13550), [sano](http://flybase.bio.indiana.edu/.bin/fbidq.html?sano), [Cirl](http://flybase.bio.indiana.edu/.bin/fbidq.html?Cirl), [l(2)NC136](http://flybase.bio.indiana.edu/.bin/fbidq.html?l(2)NC136), [Su(var)3-3](http://flybase.bio.indiana.edu/.bin/fbidq.html?Su(var)3-3), [gogo](http://flybase.bio.indiana.edu/.bin/fbidq.html?gogo), [unc-13-4A](http://flybase.bio.indiana.edu/.bin/fbidq.html?unc-13-4A),[GluClalpha](http://flybase.bio.indiana.edu/.bin/fbidq.html?GluClalpha), [Sema-1a](http://flybase.bio.indiana.edu/.bin/fbidq.html?Sema-1a), [dpr15](http://flybase.bio.indiana.edu/.bin/fbidq.html?dpr15), [Eh](http://flybase.bio.indiana.edu/.bin/fbidq.html?Eh), [Hs3st-B](http://flybase.bio.indiana.edu/.bin/fbidq.html?Hs3st-B), [Sos](http://flybase.bio.indiana.edu/.bin/fbidq.html?Sos), [Fas2](http://flybase.bio.indiana.edu/.bin/fbidq.html?Fas2), [Klp61F](http://flybase.bio.indiana.edu/.bin/fbidq.html?Klp61F), [eya](http://flybase.bio.indiana.edu/.bin/fbidq.html?eya), [CG8557](http://flybase.bio.indiana.edu/.bin/fbidq.html?CG8557), [beat-Ia](http://flybase.bio.indiana.edu/.bin/fbidq.html?beat-Ia), [kat-60L1](http://flybase.bio.indiana.edu/.bin/fbidq.html?kat-60L1), [mtg](http://flybase.bio.indiana.edu/.bin/fbidq.html?mtg), [ft](http://flybase.bio.indiana.edu/.bin/fbidq.html?ft), [CG12004](http://flybase.bio.indiana.edu/.bin/fbidq.html?CG12004), [l(2)k16918](http://flybase.bio.indiana.edu/.bin/fbidq.html?l(2)k16918), [sws](http://flybase.bio.indiana.edu/.bin/fbidq.html?sws), [Dll](http://flybase.bio.indiana.edu/.bin/fbidq.html?Dll), [disco](http://flybase.bio.indiana.edu/.bin/fbidq.html?disco), [DopR](http://flybase.bio.indiana.edu/.bin/fbidq.html?DopR), [mew](http://flybase.bio.indiana.edu/.bin/fbidq.html?mew), [didum](http://flybase.bio.indiana.edu/.bin/fbidq.html?didum), [Lis-1](http://flybase.bio.indiana.edu/.bin/fbidq.html?Lis-1), [bmm](http://flybase.bio.indiana.edu/.bin/fbidq.html?bmm), [ems](http://flybase.bio.indiana.edu/.bin/fbidq.html?ems), [eys](http://flybase.bio.indiana.edu/.bin/fbidq.html?eys), [tup](http://flybase.bio.indiana.edu/.bin/fbidq.html?tup), [Rab3](http://flybase.bio.indiana.edu/.bin/fbidq.html?Rab3), [trio](http://flybase.bio.indiana.edu/.bin/fbidq.html?trio), [pdm2](http://flybase.bio.indiana.edu/.bin/fbidq.html?pdm2), [CG31665](http://flybase.bio.indiana.edu/.bin/fbidq.html?CG31665), [Gad1](http://flybase.bio.indiana.edu/.bin/fbidq.html?Gad1), [CaMKII](http://flybase.bio.indiana.edu/.bin/fbidq.html?CaMKII),[nerfin-1](http://flybase.bio.indiana.edu/.bin/fbidq.html?nerfin-1), [polo](http://flybase.bio.indiana.edu/.bin/fbidq.html?polo), [chinmo](http://flybase.bio.indiana.edu/.bin/fbidq.html?chinmo), [Syn1](http://flybase.bio.indiana.edu/.bin/fbidq.html?Syn1), [Ssdp](http://flybase.bio.indiana.edu/.bin/fbidq.html?Ssdp), [CG8155](http://flybase.bio.indiana.edu/.bin/fbidq.html?CG8155), [fend](http://flybase.bio.indiana.edu/.bin/fbidq.html?fend), [sr](http://flybase.bio.indiana.edu/.bin/fbidq.html?sr), [Lim1](http://flybase.bio.indiana.edu/.bin/fbidq.html?Lim1), [nkd](http://flybase.bio.indiana.edu/.bin/fbidq.html?nkd), [danr](http://flybase.bio.indiana.edu/.bin/fbidq.html?danr), [Dsk](http://flybase.bio.indiana.edu/.bin/fbidq.html?Dsk), [vvl](http://flybase.bio.indiana.edu/.bin/fbidq.html?vvl), [Unc-76](http://flybase.bio.indiana.edu/.bin/fbidq.html?Unc-76), [betaInt-nu](http://flybase.bio.indiana.edu/.bin/fbidq.html?betaInt-nu), [sgg](http://flybase.bio.indiana.edu/.bin/fbidq.html?sgg), [Brd](http://flybase.bio.indiana.edu/.bin/fbidq.html?Brd), [mnb](http://flybase.bio.indiana.edu/.bin/fbidq.html?mnb), [Nmdar1](http://flybase.bio.indiana.edu/.bin/fbidq.html?Nmdar1), [Eip93F](http://flybase.bio.indiana.edu/.bin/fbidq.html?Eip93F), [gcm](http://flybase.bio.indiana.edu/.bin/fbidq.html?gcm), [dpr10](http://flybase.bio.indiana.edu/.bin/fbidq.html?dpr10), [Ccn](http://flybase.bio.indiana.edu/.bin/fbidq.html?Ccn), [ase](http://flybase.bio.indiana.edu/.bin/fbidq.html?ase), [elav](http://flybase.bio.indiana.edu/.bin/fbidq.html?elav), [Poxn](http://flybase.bio.indiana.edu/.bin/fbidq.html?Poxn), [Dat](http://flybase.bio.indiana.edu/.bin/fbidq.html?Dat), [dpr3](http://flybase.bio.indiana.edu/.bin/fbidq.html?dpr3), [Rat1](http://flybase.bio.indiana.edu/.bin/fbidq.html?Rat1), [CG18273](http://flybase.bio.indiana.edu/.bin/fbidq.html?CG18273), [eIF4G2](http://flybase.bio.indiana.edu/.bin/fbidq.html?eIF4G2), [fred](http://flybase.bio.indiana.edu/.bin/fbidq.html?fred), [ush](http://flybase.bio.indiana.edu/.bin/fbidq.html?ush), [Fak56D](http://flybase.bio.indiana.edu/.bin/fbidq.html?Fak56D), [CG16778](http://flybase.bio.indiana.edu/.bin/fbidq.html?CG16778), [Kap3](http://flybase.bio.indiana.edu/.bin/fbidq.html?Kap3),[ko](http://flybase.bio.indiana.edu/.bin/fbidq.html?ko), [dpr17](http://flybase.bio.indiana.edu/.bin/fbidq.html?dpr17), [Eip71CD](http://flybase.bio.indiana.edu/.bin/fbidq.html?Eip71CD), [Alh](http://flybase.bio.indiana.edu/.bin/fbidq.html?Alh), [egg](http://flybase.bio.indiana.edu/.bin/fbidq.html?egg), [CG12424](http://flybase.bio.indiana.edu/.bin/fbidq.html?CG12424), [Sp1](http://flybase.bio.indiana.edu/.bin/fbidq.html?Sp1), [GlcAT-P](http://flybase.bio.indiana.edu/.bin/fbidq.html?GlcAT-P), [CG3967](http://flybase.bio.indiana.edu/.bin/fbidq.html?CG3967), [Wnt5](http://flybase.bio.indiana.edu/.bin/fbidq.html?Wnt5), [SF2](http://flybase.bio.indiana.edu/.bin/fbidq.html?SF2), [sif](http://flybase.bio.indiana.edu/.bin/fbidq.html?sif), [Tbh](http://flybase.bio.indiana.edu/.bin/fbidq.html?Tbh), [Aac11](http://flybase.bio.indiana.edu/.bin/fbidq.html?Aac11), [Sas-4](http://flybase.bio.indiana.edu/.bin/fbidq.html?Sas-4), [ssh](http://flybase.bio.indiana.edu/.bin/fbidq.html?ssh), [PIP5K59B](http://flybase.bio.indiana.edu/.bin/fbidq.html?PIP5K59B), [mle](http://flybase.bio.indiana.edu/.bin/fbidq.html?mle), [Hr51](http://flybase.bio.indiana.edu/.bin/fbidq.html?Hr51), [lap](http://flybase.bio.indiana.edu/.bin/fbidq.html?lap), [CG11155](http://flybase.bio.indiana.edu/.bin/fbidq.html?CG11155), [wg](http://flybase.bio.indiana.edu/.bin/fbidq.html?wg), [fkh](http://flybase.bio.indiana.edu/.bin/fbidq.html?fkh), [RpL28](http://flybase.bio.indiana.edu/.bin/fbidq.html?RpL28), [klg](http://flybase.bio.indiana.edu/.bin/fbidq.html?klg), [en](http://flybase.bio.indiana.edu/.bin/fbidq.html?en), [sfl](http://flybase.bio.indiana.edu/.bin/fbidq.html?sfl), [Sip1](http://flybase.bio.indiana.edu/.bin/fbidq.html?Sip1), [SerT](http://flybase.bio.indiana.edu/.bin/fbidq.html?SerT), [Utx](http://flybase.bio.indiana.edu/.bin/fbidq.html?Utx), [Rnp4F](http://flybase.bio.indiana.edu/.bin/fbidq.html?Rnp4F), [Ct](http://flybase.bio.indiana.edu/.bin/fbidq.html?Ct), [fz2](http://flybase.bio.indiana.edu/.bin/fbidq.html?fz2), [numb](http://flybase.bio.indiana.edu/.bin/fbidq.html?numb), [Ocho](http://flybase.bio.indiana.edu/.bin/fbidq.html?Ocho), [CG42256](http://flybase.bio.indiana.edu/.bin/fbidq.html?CG42256), [CG17734](http://flybase.bio.indiana.edu/.bin/fbidq.html?CG17734),[Sxl](http://flybase.bio.indiana.edu/.bin/fbidq.html?Sxl), [stj](http://flybase.bio.indiana.edu/.bin/fbidq.html?stj), [CG33007](http://flybase.bio.indiana.edu/.bin/fbidq.html?CG33007), [Nf1](http://flybase.bio.indiana.edu/.bin/fbidq.html?Nf1), [Drep-3](http://flybase.bio.indiana.edu/.bin/fbidq.html?Drep-3), [sol](http://flybase.bio.indiana.edu/.bin/fbidq.html?sol), [sns](http://flybase.bio.indiana.edu/.bin/fbidq.html?sns), [bi](http://flybase.bio.indiana.edu/.bin/fbidq.html?bi), [tok](http://flybase.bio.indiana.edu/.bin/fbidq.html?tok), [nonA](http://flybase.bio.indiana.edu/.bin/fbidq.html?nonA), [Sytalpha](http://flybase.bio.indiana.edu/.bin/fbidq.html?Sytalpha), [Ptp99A](http://flybase.bio.indiana.edu/.bin/fbidq.html?Ptp99A), [CG31961](http://flybase.bio.indiana.edu/.bin/fbidq.html?CG31961), [Tango10](http://flybase.bio.indiana.edu/.bin/fbidq.html?Tango10), [Oseg4](http://flybase.bio.indiana.edu/.bin/fbidq.html?Oseg4), [blue](http://flybase.bio.indiana.edu/.bin/fbidq.html?blue), [bab1](http://flybase.bio.indiana.edu/.bin/fbidq.html?bab1), [Galpha49B](http://flybase.bio.indiana.edu/.bin/fbidq.html?Galpha49B), [Snoo](http://flybase.bio.indiana.edu/.bin/fbidq.html?Snoo), [rut](http://flybase.bio.indiana.edu/.bin/fbidq.html?rut), [uif](http://flybase.bio.indiana.edu/.bin/fbidq.html?uif), [nub](http://flybase.bio.indiana.edu/.bin/fbidq.html?nub), [btd](http://flybase.bio.indiana.edu/.bin/fbidq.html?btd), [Mmp2](http://flybase.bio.indiana.edu/.bin/fbidq.html?Mmp2), [amon](http://flybase.bio.indiana.edu/.bin/fbidq.html?amon), [fd68A](http://flybase.bio.indiana.edu/.bin/fbidq.html?fd68A), [opa1-like](http://flybase.bio.indiana.edu/.bin/fbidq.html?opa1-like), [B-H1](http://flybase.bio.indiana.edu/.bin/fbidq.html?B-H1), [pim](http://flybase.bio.indiana.edu/.bin/fbidq.html?pim), [CG32206](http://flybase.bio.indiana.edu/.bin/fbidq.html?CG32206), [DAT](http://flybase.bio.indiana.edu/.bin/fbidq.html?DAT), [Nipped-A](http://flybase.bio.indiana.edu/.bin/fbidq.html?Nipped-A), [fd102C](http://flybase.bio.indiana.edu/.bin/fbidq.html?fd102C), [hth](http://flybase.bio.indiana.edu/.bin/fbidq.html?hth), [14-3-3zeta](http://flybase.bio.indiana.edu/.bin/fbidq.html?14-3-3zeta), [pzg](http://flybase.bio.indiana.edu/.bin/fbidq.html?pzg), [lea](http://flybase.bio.indiana.edu/.bin/fbidq.html?lea), [shakB](http://flybase.bio.indiana.edu/.bin/fbidq.html?shakB), [plexB](http://flybase.bio.indiana.edu/.bin/fbidq.html?plexB), [loqs](http://flybase.bio.indiana.edu/.bin/fbidq.html?loqs), [Klp64D](http://flybase.bio.indiana.edu/.bin/fbidq.html?Klp64D), [neur](http://flybase.bio.indiana.edu/.bin/fbidq.html?neur), [Brf](http://flybase.bio.indiana.edu/.bin/fbidq.html?Brf), [CycE](http://flybase.bio.indiana.edu/.bin/fbidq.html?CycE), [CG32149](http://flybase.bio.indiana.edu/.bin/fbidq.html?CG32149), [mr](http://flybase.bio.indiana.edu/.bin/fbidq.html?mr), [orb2](http://flybase.bio.indiana.edu/.bin/fbidq.html?orb2), [ImpE2](http://flybase.bio.indiana.edu/.bin/fbidq.html?ImpE2), [synaptogyrin](http://flybase.bio.indiana.edu/.bin/fbidq.html?synaptogyrin), [sim](http://flybase.bio.indiana.edu/.bin/fbidq.html?sim), [gro](http://flybase.bio.indiana.edu/.bin/fbidq.html?gro), [Rya-r44F](http://flybase.bio.indiana.edu/.bin/fbidq.html?Rya-r44F), [scyl](http://flybase.bio.indiana.edu/.bin/fbidq.html?scyl), [Rpd3](http://flybase.bio.indiana.edu/.bin/fbidq.html?Rpd3), [CG10107](http://flybase.bio.indiana.edu/.bin/fbidq.html?CG10107), [aret](http://flybase.bio.indiana.edu/.bin/fbidq.html?aret), [HLHm7](http://flybase.bio.indiana.edu/.bin/fbidq.html?HLHm7), [tow](http://flybase.bio.indiana.edu/.bin/fbidq.html?tow), [CG15609](http://flybase.bio.indiana.edu/.bin/fbidq.html?CG15609), [sei](http://flybase.bio.indiana.edu/.bin/fbidq.html?sei), [l(1)G0148](http://flybase.bio.indiana.edu/.bin/fbidq.html?l(1)G0148), [CG12187](http://flybase.bio.indiana.edu/.bin/fbidq.html?CG12187), [Pten](http://flybase.bio.indiana.edu/.bin/fbidq.html?Pten), [unc-13](http://flybase.bio.indiana.edu/.bin/fbidq.html?unc-13), [npf](http://flybase.bio.indiana.edu/.bin/fbidq.html?npf), [dpr8](http://flybase.bio.indiana.edu/.bin/fbidq.html?dpr8), [mira](http://flybase.bio.indiana.edu/.bin/fbidq.html?mira), [Myo10A](http://flybase.bio.indiana.edu/.bin/fbidq.html?Myo10A),[Sug](http://flybase.bio.indiana.edu/.bin/fbidq.html?Sug), [Drep-2](http://flybase.bio.indiana.edu/.bin/fbidq.html?Drep-2), [Dip3](http://flybase.bio.indiana.edu/.bin/fbidq.html?Dip3), [oa2](http://flybase.bio.indiana.edu/.bin/fbidq.html?oa2), [RpS11](http://flybase.bio.indiana.edu/.bin/fbidq.html?RpS11), [phl](http://flybase.bio.indiana.edu/.bin/fbidq.html?phl), [hang](http://flybase.bio.indiana.edu/.bin/fbidq.html?hang), [CG4893](http://flybase.bio.indiana.edu/.bin/fbidq.html?CG4893), [sNPF](http://flybase.bio.indiana.edu/.bin/fbidq.html?sNPF), [CG9098](http://flybase.bio.indiana.edu/.bin/fbidq.html?CG9098), [CG7708](http://flybase.bio.indiana.edu/.bin/fbidq.html?CG7708), [Alk](http://flybase.bio.indiana.edu/.bin/fbidq.html?Alk), [Vang](http://flybase.bio.indiana.edu/.bin/fbidq.html?Vang), [esn](http://flybase.bio.indiana.edu/.bin/fbidq.html?esn), [pot](http://flybase.bio.indiana.edu/.bin/fbidq.html?pot), [Sin3A](http://flybase.bio.indiana.edu/.bin/fbidq.html?Sin3A), [Smox](http://flybase.bio.indiana.edu/.bin/fbidq.html?Smox), [Shaw](http://flybase.bio.indiana.edu/.bin/fbidq.html?Shaw), [CG31475](http://flybase.bio.indiana.edu/.bin/fbidq.html?CG31475), [Plc21C](http://flybase.bio.indiana.edu/.bin/fbidq.html?Plc21C), [srpk79D](http://flybase.bio.indiana.edu/.bin/fbidq.html?srpk79D), [Antp](http://flybase.bio.indiana.edu/.bin/fbidq.html?Antp), [Rdl](http://flybase.bio.indiana.edu/.bin/fbidq.html?Rdl), [glec](http://flybase.bio.indiana.edu/.bin/fbidq.html?glec), [l(3)neo38](http://flybase.bio.indiana.edu/.bin/fbidq.html?l(3)neo38), [Ast-C](http://flybase.bio.indiana.edu/.bin/fbidq.html?Ast-C), [cindr](http://flybase.bio.indiana.edu/.bin/fbidq.html?cindr), [Ptx1](http://flybase.bio.indiana.edu/.bin/fbidq.html?Ptx1), [comm3](http://flybase.bio.indiana.edu/.bin/fbidq.html?comm3), [CG6114](http://flybase.bio.indiana.edu/.bin/fbidq.html?CG6114), [Shal](http://flybase.bio.indiana.edu/.bin/fbidq.html?Shal), [hug](http://flybase.bio.indiana.edu/.bin/fbidq.html?hug), [pigeon](http://flybase.bio.indiana.edu/.bin/fbidq.html?pigeon), [Gpo-1](http://flybase.bio.indiana.edu/.bin/fbidq.html?Gpo-1),[para](http://flybase.bio.indiana.edu/.bin/fbidq.html?para), [Syn2](http://flybase.bio.indiana.edu/.bin/fbidq.html?Syn2), [sm](http://flybase.bio.indiana.edu/.bin/fbidq.html?sm), [tsh](http://flybase.bio.indiana.edu/.bin/fbidq.html?tsh), [e(y)3](http://flybase.bio.indiana.edu/.bin/fbidq.html?e(y)3), [eas](http://flybase.bio.indiana.edu/.bin/fbidq.html?eas), [CRMP](http://flybase.bio.indiana.edu/.bin/fbidq.html?CRMP), [Sry-alpha](http://flybase.bio.indiana.edu/.bin/fbidq.html?Sry-alpha), [Cdk5](http://flybase.bio.indiana.edu/.bin/fbidq.html?Cdk5), [oc](http://flybase.bio.indiana.edu/.bin/fbidq.html?oc), [CG4641](http://flybase.bio.indiana.edu/.bin/fbidq.html?CG4641), [CG3964](http://flybase.bio.indiana.edu/.bin/fbidq.html?CG3964), [ey](http://flybase.bio.indiana.edu/.bin/fbidq.html?ey), [baz](http://flybase.bio.indiana.edu/.bin/fbidq.html?baz), [Oamb](http://flybase.bio.indiana.edu/.bin/fbidq.html?Oamb), [rst](http://flybase.bio.indiana.edu/.bin/fbidq.html?rst), [l(1)sc](http://flybase.bio.indiana.edu/.bin/fbidq.html?l(1)sc), [CG6398](http://flybase.bio.indiana.edu/.bin/fbidq.html?CG6398), [comm](http://flybase.bio.indiana.edu/.bin/fbidq.html?comm), [grh](http://flybase.bio.indiana.edu/.bin/fbidq.html?grh), [Cdk5alpha](http://flybase.bio.indiana.edu/.bin/fbidq.html?Cdk5alpha), [Vmat](http://flybase.bio.indiana.edu/.bin/fbidq.html?Vmat), [Cha](http://flybase.bio.indiana.edu/.bin/fbidq.html?Cha), [crol](http://flybase.bio.indiana.edu/.bin/fbidq.html?crol), [E(Pc)](http://flybase.bio.indiana.edu/.bin/fbidq.html?E(Pc)), [retn](http://flybase.bio.indiana.edu/.bin/fbidq.html?retn), [Syt1](http://flybase.bio.indiana.edu/.bin/fbidq.html?Syt1), [rl](http://flybase.bio.indiana.edu/.bin/fbidq.html?rl), [Nup153](http://flybase.bio.indiana.edu/.bin/fbidq.html?Nup153), [tey](http://flybase.bio.indiana.edu/.bin/fbidq.html?tey), [CG33960](http://flybase.bio.indiana.edu/.bin/fbidq.html?CG33960), [CG15765](http://flybase.bio.indiana.edu/.bin/fbidq.html?CG15765), [dpr18](http://flybase.bio.indiana.edu/.bin/fbidq.html?dpr18), [CG1962](http://flybase.bio.indiana.edu/.bin/fbidq.html?CG1962), [unk](http://flybase.bio.indiana.edu/.bin/fbidq.html?unk), [dnr1](http://flybase.bio.indiana.edu/.bin/fbidq.html?dnr1),[Pabp2](http://flybase.bio.indiana.edu/.bin/fbidq.html?Pabp2), [acj6](http://flybase.bio.indiana.edu/.bin/fbidq.html?acj6), [caup](http://flybase.bio.indiana.edu/.bin/fbidq.html?caup), [Cbp80](http://flybase.bio.indiana.edu/.bin/fbidq.html?Cbp80), [vimar](http://flybase.bio.indiana.edu/.bin/fbidq.html?vimar), [Dg](http://flybase.bio.indiana.edu/.bin/fbidq.html?Dg), [Trim9](http://flybase.bio.indiana.edu/.bin/fbidq.html?Trim9), [CG34127](http://flybase.bio.indiana.edu/.bin/fbidq.html?CG34127), [CG7154](http://flybase.bio.indiana.edu/.bin/fbidq.html?CG7154), [br](http://flybase.bio.indiana.edu/.bin/fbidq.html?br), [robo3](http://flybase.bio.indiana.edu/.bin/fbidq.html?robo3), [neo](http://flybase.bio.indiana.edu/.bin/fbidq.html?neo), [lola](http://flybase.bio.indiana.edu/.bin/fbidq.html?lola), [unc-104](http://flybase.bio.indiana.edu/.bin/fbidq.html?unc-104), [Pcl](http://flybase.bio.indiana.edu/.bin/fbidq.html?Pcl), [CG15312](http://flybase.bio.indiana.edu/.bin/fbidq.html?CG15312), [Spc105R](http://flybase.bio.indiana.edu/.bin/fbidq.html?Spc105R), [CG31158](http://flybase.bio.indiana.edu/.bin/fbidq.html?CG31158), [tap](http://flybase.bio.indiana.edu/.bin/fbidq.html?tap), [Appl](http://flybase.bio.indiana.edu/.bin/fbidq.html?Appl), [Awh](http://flybase.bio.indiana.edu/.bin/fbidq.html?Awh), [metro](http://flybase.bio.indiana.edu/.bin/fbidq.html?metro), [Nrk](http://flybase.bio.indiana.edu/.bin/fbidq.html?Nrk), [erm](http://flybase.bio.indiana.edu/.bin/fbidq.html?erm), [CG4328](http://flybase.bio.indiana.edu/.bin/fbidq.html?CG4328), [dmt](http://flybase.bio.indiana.edu/.bin/fbidq.html?dmt), [CG11399](http://flybase.bio.indiana.edu/.bin/fbidq.html?CG11399), [Taf4](http://flybase.bio.indiana.edu/.bin/fbidq.html?Taf4), [CG10249](http://flybase.bio.indiana.edu/.bin/fbidq.html?CG10249), [Nrg](http://flybase.bio.indiana.edu/.bin/fbidq.html?Nrg), [insc](http://flybase.bio.indiana.edu/.bin/fbidq.html?insc), [jeb](http://flybase.bio.indiana.edu/.bin/fbidq.html?jeb), [Actbeta](http://flybase.bio.indiana.edu/.bin/fbidq.html?Actbeta), [beat-Ib](http://flybase.bio.indiana.edu/.bin/fbidq.html?beat-Ib),[stau](http://flybase.bio.indiana.edu/.bin/fbidq.html?stau), [otk](http://flybase.bio.indiana.edu/.bin/fbidq.html?otk), [phyl](http://flybase.bio.indiana.edu/.bin/fbidq.html?phyl), [vg](http://flybase.bio.indiana.edu/.bin/fbidq.html?vg), [brm](http://flybase.bio.indiana.edu/.bin/fbidq.html?brm), [bsk](http://flybase.bio.indiana.edu/.bin/fbidq.html?bsk), [robo](http://flybase.bio.indiana.edu/.bin/fbidq.html?robo), [fz](http://flybase.bio.indiana.edu/.bin/fbidq.html?fz), [CG42268](http://flybase.bio.indiana.edu/.bin/fbidq.html?CG42268), [lft](http://flybase.bio.indiana.edu/.bin/fbidq.html?lft), [sno](http://flybase.bio.indiana.edu/.bin/fbidq.html?sno), [RhoGAPp190](http://flybase.bio.indiana.edu/.bin/fbidq.html?RhoGAPp190), [hig](http://flybase.bio.indiana.edu/.bin/fbidq.html?hig), [pHCl](http://flybase.bio.indiana.edu/.bin/fbidq.html?pHCl), [ara](http://flybase.bio.indiana.edu/.bin/fbidq.html?ara), [Sfmbt](http://flybase.bio.indiana.edu/.bin/fbidq.html?Sfmbt), [mam](http://flybase.bio.indiana.edu/.bin/fbidq.html?mam), [pan](http://flybase.bio.indiana.edu/.bin/fbidq.html?pan), [grn](http://flybase.bio.indiana.edu/.bin/fbidq.html?grn), [HLHm5](http://flybase.bio.indiana.edu/.bin/fbidq.html?HLHm5), [CG4019](http://flybase.bio.indiana.edu/.bin/fbidq.html?CG4019), [dap](http://flybase.bio.indiana.edu/.bin/fbidq.html?dap), [Bx](http://flybase.bio.indiana.edu/.bin/fbidq.html?Bx), [Mob2](http://flybase.bio.indiana.edu/.bin/fbidq.html?Mob2), [pros](http://flybase.bio.indiana.edu/.bin/fbidq.html?pros), [mthl8](http://flybase.bio.indiana.edu/.bin/fbidq.html?mthl8), [CG1463](http://flybase.bio.indiana.edu/.bin/fbidq.html?CG1463), [Ccap](http://flybase.bio.indiana.edu/.bin/fbidq.html?Ccap), [Wnt4](http://flybase.bio.indiana.edu/.bin/fbidq.html?Wnt4), [rictor](http://flybase.bio.indiana.edu/.bin/fbidq.html?rictor), [Glut4EF](http://flybase.bio.indiana.edu/.bin/fbidq.html?Glut4EF), [CG42629](http://flybase.bio.indiana.edu/.bin/fbidq.html?CG42629), [bchs](http://flybase.bio.indiana.edu/.bin/fbidq.html?bchs), [RpS5b](http://flybase.bio.indiana.edu/.bin/fbidq.html?RpS5b), [king-tubby](http://flybase.bio.indiana.edu/.bin/fbidq.html?king-tubby), [pk](http://flybase.bio.indiana.edu/.bin/fbidq.html?pk), [dsx](http://flybase.bio.indiana.edu/.bin/fbidq.html?dsx),[Dh](http://flybase.bio.indiana.edu/.bin/fbidq.html?Dh), [betaTub85D](http://flybase.bio.indiana.edu/.bin/fbidq.html?betaTub85D), [klu](http://flybase.bio.indiana.edu/.bin/fbidq.html?klu), [sqz](http://flybase.bio.indiana.edu/.bin/fbidq.html?sqz), [dpr11](http://flybase.bio.indiana.edu/.bin/fbidq.html?dpr11), [Ca-alpha1D](http://flybase.bio.indiana.edu/.bin/fbidq.html?Ca-alpha1D), [Atet](http://flybase.bio.indiana.edu/.bin/fbidq.html?Atet), [tld](http://flybase.bio.indiana.edu/.bin/fbidq.html?tld), [rg](http://flybase.bio.indiana.edu/.bin/fbidq.html?rg), [CG1909](http://flybase.bio.indiana.edu/.bin/fbidq.html?CG1909), [gcm2](http://flybase.bio.indiana.edu/.bin/fbidq.html?gcm2), [mp](http://flybase.bio.indiana.edu/.bin/fbidq.html?mp), [CG5890](http://flybase.bio.indiana.edu/.bin/fbidq.html?CG5890), [CanB](http://flybase.bio.indiana.edu/.bin/fbidq.html?CanB), [SP2353](http://flybase.bio.indiana.edu/.bin/fbidq.html?SP2353), [rab3-GEF](http://flybase.bio.indiana.edu/.bin/fbidq.html?rab3-GEF), [CG13253](http://flybase.bio.indiana.edu/.bin/fbidq.html?CG13253), [ovo](http://flybase.bio.indiana.edu/.bin/fbidq.html?ovo), [CG32685](http://flybase.bio.indiana.edu/.bin/fbidq.html?CG32685), [Dsp1](http://flybase.bio.indiana.edu/.bin/fbidq.html?Dsp1), [ds](http://flybase.bio.indiana.edu/.bin/fbidq.html?ds), [Syt4](http://flybase.bio.indiana.edu/.bin/fbidq.html?Syt4), [CG11033](http://flybase.bio.indiana.edu/.bin/fbidq.html?CG11033), [Incenp](http://flybase.bio.indiana.edu/.bin/fbidq.html?Incenp), [SIFR](http://flybase.bio.indiana.edu/.bin/fbidq.html?SIFR), [Drl-2](http://flybase.bio.indiana.edu/.bin/fbidq.html?Drl-2), [MYPT-75D](http://flybase.bio.indiana.edu/.bin/fbidq.html?MYPT-75D), [Tk](http://flybase.bio.indiana.edu/.bin/fbidq.html?Tk), [Gap1](http://flybase.bio.indiana.edu/.bin/fbidq.html?Gap1), [run](http://flybase.bio.indiana.edu/.bin/fbidq.html?run), [sNPF-R](http://flybase.bio.indiana.edu/.bin/fbidq.html?sNPF-R), [tara](http://flybase.bio.indiana.edu/.bin/fbidq.html?tara), [stan](http://flybase.bio.indiana.edu/.bin/fbidq.html?stan),[CanA-14F](http://flybase.bio.indiana.edu/.bin/fbidq.html?CanA-14F), [tutl](http://flybase.bio.indiana.edu/.bin/fbidq.html?tutl), [Ptth](http://flybase.bio.indiana.edu/.bin/fbidq.html?Ptth), [CG1753](http://flybase.bio.indiana.edu/.bin/fbidq.html?CG1753), [Ilp2](http://flybase.bio.indiana.edu/.bin/fbidq.html?Ilp2), [ph-p](http://flybase.bio.indiana.edu/.bin/fbidq.html?ph-p), [Frq1](http://flybase.bio.indiana.edu/.bin/fbidq.html?Frq1), [sna](http://flybase.bio.indiana.edu/.bin/fbidq.html?sna), [Taf1](http://flybase.bio.indiana.edu/.bin/fbidq.html?Taf1), [Bre1](http://flybase.bio.indiana.edu/.bin/fbidq.html?Bre1), [Ggamma1](http://flybase.bio.indiana.edu/.bin/fbidq.html?Ggamma1), [CG7597](http://flybase.bio.indiana.edu/.bin/fbidq.html?CG7597), [CG5466](http://flybase.bio.indiana.edu/.bin/fbidq.html?CG5466), [MBD-like](http://flybase.bio.indiana.edu/.bin/fbidq.html?MBD-like), [esg](http://flybase.bio.indiana.edu/.bin/fbidq.html?esg), [m4](http://flybase.bio.indiana.edu/.bin/fbidq.html?m4), [dom](http://flybase.bio.indiana.edu/.bin/fbidq.html?dom), [Dh31](http://flybase.bio.indiana.edu/.bin/fbidq.html?Dh31), [klar](http://flybase.bio.indiana.edu/.bin/fbidq.html?klar), [shi](http://flybase.bio.indiana.edu/.bin/fbidq.html?shi), [Spred](http://flybase.bio.indiana.edu/.bin/fbidq.html?Spred), [siz](http://flybase.bio.indiana.edu/.bin/fbidq.html?siz), [B-H2](http://flybase.bio.indiana.edu/.bin/fbidq.html?B-H2), [tyn](http://flybase.bio.indiana.edu/.bin/fbidq.html?tyn), [CG3822](http://flybase.bio.indiana.edu/.bin/fbidq.html?CG3822), [dpr5](http://flybase.bio.indiana.edu/.bin/fbidq.html?dpr5), [CadN](http://flybase.bio.indiana.edu/.bin/fbidq.html?CadN), [CG34139](http://flybase.bio.indiana.edu/.bin/fbidq.html?CG34139), [Mfap1](http://flybase.bio.indiana.edu/.bin/fbidq.html?Mfap1), [E(bx)](http://flybase.bio.indiana.edu/.bin/fbidq.html?E(bx)), [dpr19](http://flybase.bio.indiana.edu/.bin/fbidq.html?dpr19), [Dms](http://flybase.bio.indiana.edu/.bin/fbidq.html?Dms), [Snap](http://flybase.bio.indiana.edu/.bin/fbidq.html?Snap), [trn](http://flybase.bio.indiana.edu/.bin/fbidq.html?trn), [pburs](http://flybase.bio.indiana.edu/.bin/fbidq.html?pburs), [cos](http://flybase.bio.indiana.edu/.bin/fbidq.html?cos) |
| [nucleobase-containing compound biosynthetic process](http://amigo.geneontology.org/cgi-bin/amigo/go.cgi?view=details&query=GO:0034654) | 216 of 1594 genes, 13.6% | 748 of 7634 genes, 9.8% | 5.94e-07 | 0.00% | 0.00 | [fz2](http://flybase.bio.indiana.edu/.bin/fbidq.html?fz2), [acj6](http://flybase.bio.indiana.edu/.bin/fbidq.html?acj6), [skd](http://flybase.bio.indiana.edu/.bin/fbidq.html?skd), [jing](http://flybase.bio.indiana.edu/.bin/fbidq.html?jing), [caup](http://flybase.bio.indiana.edu/.bin/fbidq.html?caup), [Tgt](http://flybase.bio.indiana.edu/.bin/fbidq.html?Tgt), [tll](http://flybase.bio.indiana.edu/.bin/fbidq.html?tll), [Camta](http://flybase.bio.indiana.edu/.bin/fbidq.html?Camta), [ato](http://flybase.bio.indiana.edu/.bin/fbidq.html?ato), [Ets65A](http://flybase.bio.indiana.edu/.bin/fbidq.html?Ets65A), [tna](http://flybase.bio.indiana.edu/.bin/fbidq.html?tna), [Prat2](http://flybase.bio.indiana.edu/.bin/fbidq.html?Prat2), [JIL-1](http://flybase.bio.indiana.edu/.bin/fbidq.html?JIL-1), [Spt6](http://flybase.bio.indiana.edu/.bin/fbidq.html?Spt6), [Iswi](http://flybase.bio.indiana.edu/.bin/fbidq.html?Iswi), [rn](http://flybase.bio.indiana.edu/.bin/fbidq.html?rn), [ap](http://flybase.bio.indiana.edu/.bin/fbidq.html?ap), [Nf1](http://flybase.bio.indiana.edu/.bin/fbidq.html?Nf1), [br](http://flybase.bio.indiana.edu/.bin/fbidq.html?br), [Gycalpha99B](http://flybase.bio.indiana.edu/.bin/fbidq.html?Gycalpha99B), [ksr](http://flybase.bio.indiana.edu/.bin/fbidq.html?ksr), [PNUTS](http://flybase.bio.indiana.edu/.bin/fbidq.html?PNUTS), [Gyc-89Da](http://flybase.bio.indiana.edu/.bin/fbidq.html?Gyc-89Da), [bi](http://flybase.bio.indiana.edu/.bin/fbidq.html?bi), [chn](http://flybase.bio.indiana.edu/.bin/fbidq.html?chn), [Gsc](http://flybase.bio.indiana.edu/.bin/fbidq.html?Gsc), [Hcf](http://flybase.bio.indiana.edu/.bin/fbidq.html?Hcf), [lola](http://flybase.bio.indiana.edu/.bin/fbidq.html?lola), [HLH4C](http://flybase.bio.indiana.edu/.bin/fbidq.html?HLH4C), [Pcl](http://flybase.bio.indiana.edu/.bin/fbidq.html?Pcl), [chif](http://flybase.bio.indiana.edu/.bin/fbidq.html?chif), [simj](http://flybase.bio.indiana.edu/.bin/fbidq.html?simj), [Awh](http://flybase.bio.indiana.edu/.bin/fbidq.html?Awh), [tap](http://flybase.bio.indiana.edu/.bin/fbidq.html?tap), [CG13550](http://flybase.bio.indiana.edu/.bin/fbidq.html?CG13550), [NC2alpha](http://flybase.bio.indiana.edu/.bin/fbidq.html?NC2alpha), [PHDP](http://flybase.bio.indiana.edu/.bin/fbidq.html?PHDP), [bab1](http://flybase.bio.indiana.edu/.bin/fbidq.html?bab1), [Taf12L](http://flybase.bio.indiana.edu/.bin/fbidq.html?Taf12L),[CG4328](http://flybase.bio.indiana.edu/.bin/fbidq.html?CG4328), [Snoo](http://flybase.bio.indiana.edu/.bin/fbidq.html?Snoo), [l(2)NC136](http://flybase.bio.indiana.edu/.bin/fbidq.html?l(2)NC136), [Taf4](http://flybase.bio.indiana.edu/.bin/fbidq.html?Taf4), [Su(var)3-3](http://flybase.bio.indiana.edu/.bin/fbidq.html?Su(var)3-3), [HGTX](http://flybase.bio.indiana.edu/.bin/fbidq.html?HGTX), [not](http://flybase.bio.indiana.edu/.bin/fbidq.html?not), [rut](http://flybase.bio.indiana.edu/.bin/fbidq.html?rut), [nvy](http://flybase.bio.indiana.edu/.bin/fbidq.html?nvy), [nub](http://flybase.bio.indiana.edu/.bin/fbidq.html?nub), [nab](http://flybase.bio.indiana.edu/.bin/fbidq.html?nab), [btd](http://flybase.bio.indiana.edu/.bin/fbidq.html?btd), [fd68A](http://flybase.bio.indiana.edu/.bin/fbidq.html?fd68A), [HLHmdelta](http://flybase.bio.indiana.edu/.bin/fbidq.html?HLHmdelta), [B-H1](http://flybase.bio.indiana.edu/.bin/fbidq.html?B-H1), [Nipped-A](http://flybase.bio.indiana.edu/.bin/fbidq.html?Nipped-A), [ATbp](http://flybase.bio.indiana.edu/.bin/fbidq.html?ATbp), [Sos](http://flybase.bio.indiana.edu/.bin/fbidq.html?Sos), [Fer2](http://flybase.bio.indiana.edu/.bin/fbidq.html?Fer2), [fd102C](http://flybase.bio.indiana.edu/.bin/fbidq.html?fd102C), [vg](http://flybase.bio.indiana.edu/.bin/fbidq.html?vg), [hth](http://flybase.bio.indiana.edu/.bin/fbidq.html?hth), [Nf-YC](http://flybase.bio.indiana.edu/.bin/fbidq.html?Nf-YC), [eya](http://flybase.bio.indiana.edu/.bin/fbidq.html?eya), [fz](http://flybase.bio.indiana.edu/.bin/fbidq.html?fz), [brm](http://flybase.bio.indiana.edu/.bin/fbidq.html?brm), [Vsx2](http://flybase.bio.indiana.edu/.bin/fbidq.html?Vsx2), [Hey](http://flybase.bio.indiana.edu/.bin/fbidq.html?Hey), [sno](http://flybase.bio.indiana.edu/.bin/fbidq.html?sno), [Sfmbt](http://flybase.bio.indiana.edu/.bin/fbidq.html?Sfmbt), [ara](http://flybase.bio.indiana.edu/.bin/fbidq.html?ara), [mam](http://flybase.bio.indiana.edu/.bin/fbidq.html?mam), [pan](http://flybase.bio.indiana.edu/.bin/fbidq.html?pan), [Dll](http://flybase.bio.indiana.edu/.bin/fbidq.html?Dll), [disco](http://flybase.bio.indiana.edu/.bin/fbidq.html?disco), [grn](http://flybase.bio.indiana.edu/.bin/fbidq.html?grn), [HLHm5](http://flybase.bio.indiana.edu/.bin/fbidq.html?HLHm5), [D12](http://flybase.bio.indiana.edu/.bin/fbidq.html?D12),[Lis-1](http://flybase.bio.indiana.edu/.bin/fbidq.html?Lis-1), [CycE](http://flybase.bio.indiana.edu/.bin/fbidq.html?CycE), [Brf](http://flybase.bio.indiana.edu/.bin/fbidq.html?Brf), [ems](http://flybase.bio.indiana.edu/.bin/fbidq.html?ems), [pros](http://flybase.bio.indiana.edu/.bin/fbidq.html?pros), [tup](http://flybase.bio.indiana.edu/.bin/fbidq.html?tup), [pdm2](http://flybase.bio.indiana.edu/.bin/fbidq.html?pdm2), [Gug](http://flybase.bio.indiana.edu/.bin/fbidq.html?Gug), [pad](http://flybase.bio.indiana.edu/.bin/fbidq.html?pad), [rgr](http://flybase.bio.indiana.edu/.bin/fbidq.html?rgr), [Wnt4](http://flybase.bio.indiana.edu/.bin/fbidq.html?Wnt4), [CG3714](http://flybase.bio.indiana.edu/.bin/fbidq.html?CG3714), [Rx](http://flybase.bio.indiana.edu/.bin/fbidq.html?Rx), [MED1](http://flybase.bio.indiana.edu/.bin/fbidq.html?MED1), [salr](http://flybase.bio.indiana.edu/.bin/fbidq.html?salr), [wdn](http://flybase.bio.indiana.edu/.bin/fbidq.html?wdn), [sim](http://flybase.bio.indiana.edu/.bin/fbidq.html?sim), [d4](http://flybase.bio.indiana.edu/.bin/fbidq.html?d4), [dan](http://flybase.bio.indiana.edu/.bin/fbidq.html?dan), [gro](http://flybase.bio.indiana.edu/.bin/fbidq.html?gro), [nerfin-1](http://flybase.bio.indiana.edu/.bin/fbidq.html?nerfin-1), [trx](http://flybase.bio.indiana.edu/.bin/fbidq.html?trx), [Rpd3](http://flybase.bio.indiana.edu/.bin/fbidq.html?Rpd3), [dsx](http://flybase.bio.indiana.edu/.bin/fbidq.html?dsx), [MED25](http://flybase.bio.indiana.edu/.bin/fbidq.html?MED25), [Ac13E](http://flybase.bio.indiana.edu/.bin/fbidq.html?Ac13E), [Ssdp](http://flybase.bio.indiana.edu/.bin/fbidq.html?Ssdp), [vfl](http://flybase.bio.indiana.edu/.bin/fbidq.html?vfl), [RpII140](http://flybase.bio.indiana.edu/.bin/fbidq.html?RpII140), [sqz](http://flybase.bio.indiana.edu/.bin/fbidq.html?sqz), [CG32105](http://flybase.bio.indiana.edu/.bin/fbidq.html?CG32105), [Fer3](http://flybase.bio.indiana.edu/.bin/fbidq.html?Fer3), [pygo](http://flybase.bio.indiana.edu/.bin/fbidq.html?pygo), [dalao](http://flybase.bio.indiana.edu/.bin/fbidq.html?dalao), [HLHm7](http://flybase.bio.indiana.edu/.bin/fbidq.html?HLHm7), [z](http://flybase.bio.indiana.edu/.bin/fbidq.html?z), [HLHmgamma](http://flybase.bio.indiana.edu/.bin/fbidq.html?HLHmgamma), [Sox21b](http://flybase.bio.indiana.edu/.bin/fbidq.html?Sox21b),[sr](http://flybase.bio.indiana.edu/.bin/fbidq.html?sr), [Lim1](http://flybase.bio.indiana.edu/.bin/fbidq.html?Lim1), [tld](http://flybase.bio.indiana.edu/.bin/fbidq.html?tld), [danr](http://flybase.bio.indiana.edu/.bin/fbidq.html?danr), [wor](http://flybase.bio.indiana.edu/.bin/fbidq.html?wor), [vvl](http://flybase.bio.indiana.edu/.bin/fbidq.html?vvl), [wda](http://flybase.bio.indiana.edu/.bin/fbidq.html?wda), [sgg](http://flybase.bio.indiana.edu/.bin/fbidq.html?sgg), [MED14](http://flybase.bio.indiana.edu/.bin/fbidq.html?MED14), [gcm2](http://flybase.bio.indiana.edu/.bin/fbidq.html?gcm2), [Eip93F](http://flybase.bio.indiana.edu/.bin/fbidq.html?Eip93F), [gcm](http://flybase.bio.indiana.edu/.bin/fbidq.html?gcm), [seq](http://flybase.bio.indiana.edu/.bin/fbidq.html?seq), [Dip3](http://flybase.bio.indiana.edu/.bin/fbidq.html?Dip3), [Aef1](http://flybase.bio.indiana.edu/.bin/fbidq.html?Aef1), [HDAC4](http://flybase.bio.indiana.edu/.bin/fbidq.html?HDAC4), [can](http://flybase.bio.indiana.edu/.bin/fbidq.html?can), [elk](http://flybase.bio.indiana.edu/.bin/fbidq.html?elk), [phl](http://flybase.bio.indiana.edu/.bin/fbidq.html?phl), [ase](http://flybase.bio.indiana.edu/.bin/fbidq.html?ase), [CG13253](http://flybase.bio.indiana.edu/.bin/fbidq.html?CG13253), [ovo](http://flybase.bio.indiana.edu/.bin/fbidq.html?ovo), [scro](http://flybase.bio.indiana.edu/.bin/fbidq.html?scro), [Dsp1](http://flybase.bio.indiana.edu/.bin/fbidq.html?Dsp1), [CG14216](http://flybase.bio.indiana.edu/.bin/fbidq.html?CG14216), [Pdp1](http://flybase.bio.indiana.edu/.bin/fbidq.html?Pdp1), [Oli](http://flybase.bio.indiana.edu/.bin/fbidq.html?Oli), [Nelf-A](http://flybase.bio.indiana.edu/.bin/fbidq.html?Nelf-A), [GATAd](http://flybase.bio.indiana.edu/.bin/fbidq.html?GATAd), [Poxn](http://flybase.bio.indiana.edu/.bin/fbidq.html?Poxn), [CG15376](http://flybase.bio.indiana.edu/.bin/fbidq.html?CG15376), [Sin3A](http://flybase.bio.indiana.edu/.bin/fbidq.html?Sin3A), [mip120](http://flybase.bio.indiana.edu/.bin/fbidq.html?mip120), [jumu](http://flybase.bio.indiana.edu/.bin/fbidq.html?jumu), [Smox](http://flybase.bio.indiana.edu/.bin/fbidq.html?Smox), [Usp7](http://flybase.bio.indiana.edu/.bin/fbidq.html?Usp7), [ush](http://flybase.bio.indiana.edu/.bin/fbidq.html?ush), [fd59A](http://flybase.bio.indiana.edu/.bin/fbidq.html?fd59A),[Gap1](http://flybase.bio.indiana.edu/.bin/fbidq.html?Gap1), [run](http://flybase.bio.indiana.edu/.bin/fbidq.html?run), [FBX011](http://flybase.bio.indiana.edu/.bin/fbidq.html?FBX011), [brat](http://flybase.bio.indiana.edu/.bin/fbidq.html?brat), [Antp](http://flybase.bio.indiana.edu/.bin/fbidq.html?Antp), [tara](http://flybase.bio.indiana.edu/.bin/fbidq.html?tara), [Alh](http://flybase.bio.indiana.edu/.bin/fbidq.html?Alh), [Sox102F](http://flybase.bio.indiana.edu/.bin/fbidq.html?Sox102F), [egg](http://flybase.bio.indiana.edu/.bin/fbidq.html?egg), [l(3)neo38](http://flybase.bio.indiana.edu/.bin/fbidq.html?l(3)neo38), [koko](http://flybase.bio.indiana.edu/.bin/fbidq.html?koko), [Ada](http://flybase.bio.indiana.edu/.bin/fbidq.html?Ada), [Ptth](http://flybase.bio.indiana.edu/.bin/fbidq.html?Ptth), [Sp1](http://flybase.bio.indiana.edu/.bin/fbidq.html?Sp1), [Ptx1](http://flybase.bio.indiana.edu/.bin/fbidq.html?Ptx1), [ph-p](http://flybase.bio.indiana.edu/.bin/fbidq.html?ph-p), [Ubx](http://flybase.bio.indiana.edu/.bin/fbidq.html?Ubx), [onecut](http://flybase.bio.indiana.edu/.bin/fbidq.html?onecut), [CG7757](http://flybase.bio.indiana.edu/.bin/fbidq.html?CG7757), [chm](http://flybase.bio.indiana.edu/.bin/fbidq.html?chm), [sna](http://flybase.bio.indiana.edu/.bin/fbidq.html?sna), [Wnt5](http://flybase.bio.indiana.edu/.bin/fbidq.html?Wnt5), [Taf1](http://flybase.bio.indiana.edu/.bin/fbidq.html?Taf1), [hbn](http://flybase.bio.indiana.edu/.bin/fbidq.html?hbn), [MBD-like](http://flybase.bio.indiana.edu/.bin/fbidq.html?MBD-like), [tsh](http://flybase.bio.indiana.edu/.bin/fbidq.html?tsh), [esg](http://flybase.bio.indiana.edu/.bin/fbidq.html?esg), [MED16](http://flybase.bio.indiana.edu/.bin/fbidq.html?MED16), [e(y)3](http://flybase.bio.indiana.edu/.bin/fbidq.html?e(y)3), [dac](http://flybase.bio.indiana.edu/.bin/fbidq.html?dac), [CG9007](http://flybase.bio.indiana.edu/.bin/fbidq.html?CG9007), [oc](http://flybase.bio.indiana.edu/.bin/fbidq.html?oc), [spt4](http://flybase.bio.indiana.edu/.bin/fbidq.html?spt4), [CG32532](http://flybase.bio.indiana.edu/.bin/fbidq.html?CG32532), [MTA1-like](http://flybase.bio.indiana.edu/.bin/fbidq.html?MTA1-like), [gol](http://flybase.bio.indiana.edu/.bin/fbidq.html?gol), [ey](http://flybase.bio.indiana.edu/.bin/fbidq.html?ey),[mle](http://flybase.bio.indiana.edu/.bin/fbidq.html?mle), [Hr51](http://flybase.bio.indiana.edu/.bin/fbidq.html?Hr51), [Bgb](http://flybase.bio.indiana.edu/.bin/fbidq.html?Bgb), [l(1)sc](http://flybase.bio.indiana.edu/.bin/fbidq.html?l(1)sc), [B-H2](http://flybase.bio.indiana.edu/.bin/fbidq.html?B-H2), [grh](http://flybase.bio.indiana.edu/.bin/fbidq.html?grh), [Vsx1](http://flybase.bio.indiana.edu/.bin/fbidq.html?Vsx1), [wg](http://flybase.bio.indiana.edu/.bin/fbidq.html?wg), [toy](http://flybase.bio.indiana.edu/.bin/fbidq.html?toy), [fkh](http://flybase.bio.indiana.edu/.bin/fbidq.html?fkh), [Lim3](http://flybase.bio.indiana.edu/.bin/fbidq.html?Lim3), [crol](http://flybase.bio.indiana.edu/.bin/fbidq.html?crol), [sd](http://flybase.bio.indiana.edu/.bin/fbidq.html?sd), [retn](http://flybase.bio.indiana.edu/.bin/fbidq.html?retn), [en](http://flybase.bio.indiana.edu/.bin/fbidq.html?en), [fs(1)h](http://flybase.bio.indiana.edu/.bin/fbidq.html?fs(1)h), [Ac3](http://flybase.bio.indiana.edu/.bin/fbidq.html?Ac3), [E(bx)](http://flybase.bio.indiana.edu/.bin/fbidq.html?E(bx)), [rl](http://flybase.bio.indiana.edu/.bin/fbidq.html?rl), [CG11347](http://flybase.bio.indiana.edu/.bin/fbidq.html?CG11347), [tey](http://flybase.bio.indiana.edu/.bin/fbidq.html?tey), [CG11294](http://flybase.bio.indiana.edu/.bin/fbidq.html?CG11294), [hiw](http://flybase.bio.indiana.edu/.bin/fbidq.html?hiw), [cos](http://flybase.bio.indiana.edu/.bin/fbidq.html?cos), [Ct](http://flybase.bio.indiana.edu/.bin/fbidq.html?Ct), [Atf-2](http://flybase.bio.indiana.edu/.bin/fbidq.html?Atf-2) |
| [regulation of multicellular organismal process](http://amigo.geneontology.org/cgi-bin/amigo/go.cgi?view=details&query=GO:0051239) | 136 of 1594 genes, 8.5% | 426 of 7634 genes, 5.6% | 1.88e-06 | 0.00% | 0.00 | [fz2](http://flybase.bio.indiana.edu/.bin/fbidq.html?fz2), [Sh](http://flybase.bio.indiana.edu/.bin/fbidq.html?Sh), [numb](http://flybase.bio.indiana.edu/.bin/fbidq.html?numb), [Fmrf](http://flybase.bio.indiana.edu/.bin/fbidq.html?Fmrf), [stj](http://flybase.bio.indiana.edu/.bin/fbidq.html?stj), [Dg](http://flybase.bio.indiana.edu/.bin/fbidq.html?Dg), [Nf1](http://flybase.bio.indiana.edu/.bin/fbidq.html?Nf1), [mago](http://flybase.bio.indiana.edu/.bin/fbidq.html?mago), [enc](http://flybase.bio.indiana.edu/.bin/fbidq.html?enc), [neo](http://flybase.bio.indiana.edu/.bin/fbidq.html?neo), [os](http://flybase.bio.indiana.edu/.bin/fbidq.html?os), [brp](http://flybase.bio.indiana.edu/.bin/fbidq.html?brp), [chn](http://flybase.bio.indiana.edu/.bin/fbidq.html?chn), [Traf6](http://flybase.bio.indiana.edu/.bin/fbidq.html?Traf6), [NetB](http://flybase.bio.indiana.edu/.bin/fbidq.html?NetB), [metro](http://flybase.bio.indiana.edu/.bin/fbidq.html?metro), [ewg](http://flybase.bio.indiana.edu/.bin/fbidq.html?ewg), [erm](http://flybase.bio.indiana.edu/.bin/fbidq.html?erm), [blue](http://flybase.bio.indiana.edu/.bin/fbidq.html?blue), [bab1](http://flybase.bio.indiana.edu/.bin/fbidq.html?bab1), [Galpha49B](http://flybase.bio.indiana.edu/.bin/fbidq.html?Galpha49B), [Lar](http://flybase.bio.indiana.edu/.bin/fbidq.html?Lar), [scrib](http://flybase.bio.indiana.edu/.bin/fbidq.html?scrib), [Ddc](http://flybase.bio.indiana.edu/.bin/fbidq.html?Ddc), [insc](http://flybase.bio.indiana.edu/.bin/fbidq.html?insc), [alph](http://flybase.bio.indiana.edu/.bin/fbidq.html?alph), [stau](http://flybase.bio.indiana.edu/.bin/fbidq.html?stau), [Sema-1a](http://flybase.bio.indiana.edu/.bin/fbidq.html?Sema-1a), [Eh](http://flybase.bio.indiana.edu/.bin/fbidq.html?Eh), [Hs3st-B](http://flybase.bio.indiana.edu/.bin/fbidq.html?Hs3st-B), [TBPH](http://flybase.bio.indiana.edu/.bin/fbidq.html?TBPH), [Snap25](http://flybase.bio.indiana.edu/.bin/fbidq.html?Snap25), [mGluRA](http://flybase.bio.indiana.edu/.bin/fbidq.html?mGluRA), [hth](http://flybase.bio.indiana.edu/.bin/fbidq.html?hth), [dsh](http://flybase.bio.indiana.edu/.bin/fbidq.html?dsh), [Fas2](http://flybase.bio.indiana.edu/.bin/fbidq.html?Fas2), [fz](http://flybase.bio.indiana.edu/.bin/fbidq.html?fz), [robo](http://flybase.bio.indiana.edu/.bin/fbidq.html?robo), [lea](http://flybase.bio.indiana.edu/.bin/fbidq.html?lea), [kat-60L1](http://flybase.bio.indiana.edu/.bin/fbidq.html?kat-60L1),[comt](http://flybase.bio.indiana.edu/.bin/fbidq.html?comt), [ft](http://flybase.bio.indiana.edu/.bin/fbidq.html?ft), [RhoGAPp190](http://flybase.bio.indiana.edu/.bin/fbidq.html?RhoGAPp190), [qvr](http://flybase.bio.indiana.edu/.bin/fbidq.html?qvr), [pan](http://flybase.bio.indiana.edu/.bin/fbidq.html?pan), [DAAM](http://flybase.bio.indiana.edu/.bin/fbidq.html?DAAM), [neur](http://flybase.bio.indiana.edu/.bin/fbidq.html?neur), [didum](http://flybase.bio.indiana.edu/.bin/fbidq.html?didum), [Lis-1](http://flybase.bio.indiana.edu/.bin/fbidq.html?Lis-1), [nmo](http://flybase.bio.indiana.edu/.bin/fbidq.html?nmo), [5-HT1A](http://flybase.bio.indiana.edu/.bin/fbidq.html?5-HT1A), [pros](http://flybase.bio.indiana.edu/.bin/fbidq.html?pros), [trio](http://flybase.bio.indiana.edu/.bin/fbidq.html?trio), [ed](http://flybase.bio.indiana.edu/.bin/fbidq.html?ed), [mr](http://flybase.bio.indiana.edu/.bin/fbidq.html?mr), [Ccap](http://flybase.bio.indiana.edu/.bin/fbidq.html?Ccap), [Wnt4](http://flybase.bio.indiana.edu/.bin/fbidq.html?Wnt4), [Apc](http://flybase.bio.indiana.edu/.bin/fbidq.html?Apc), [CaMKII](http://flybase.bio.indiana.edu/.bin/fbidq.html?CaMKII), [CG42629](http://flybase.bio.indiana.edu/.bin/fbidq.html?CG42629), [Rya-r44F](http://flybase.bio.indiana.edu/.bin/fbidq.html?Rya-r44F), [Rpd3](http://flybase.bio.indiana.edu/.bin/fbidq.html?Rpd3), [polo](http://flybase.bio.indiana.edu/.bin/fbidq.html?polo), [dsx](http://flybase.bio.indiana.edu/.bin/fbidq.html?dsx), [Syn1](http://flybase.bio.indiana.edu/.bin/fbidq.html?Syn1), [cdi](http://flybase.bio.indiana.edu/.bin/fbidq.html?cdi), [klu](http://flybase.bio.indiana.edu/.bin/fbidq.html?klu), [SPR](http://flybase.bio.indiana.edu/.bin/fbidq.html?SPR), [HLHmgamma](http://flybase.bio.indiana.edu/.bin/fbidq.html?HLHmgamma), [nkd](http://flybase.bio.indiana.edu/.bin/fbidq.html?nkd), [Dsk](http://flybase.bio.indiana.edu/.bin/fbidq.html?Dsk), [Atg1](http://flybase.bio.indiana.edu/.bin/fbidq.html?Atg1), [vvl](http://flybase.bio.indiana.edu/.bin/fbidq.html?vvl), [CG12187](http://flybase.bio.indiana.edu/.bin/fbidq.html?CG12187), [Pten](http://flybase.bio.indiana.edu/.bin/fbidq.html?Pten), [betaInt-nu](http://flybase.bio.indiana.edu/.bin/fbidq.html?betaInt-nu),[sgg](http://flybase.bio.indiana.edu/.bin/fbidq.html?sgg), [mira](http://flybase.bio.indiana.edu/.bin/fbidq.html?mira), [gcm2](http://flybase.bio.indiana.edu/.bin/fbidq.html?gcm2), [gcm](http://flybase.bio.indiana.edu/.bin/fbidq.html?gcm), [seq](http://flybase.bio.indiana.edu/.bin/fbidq.html?seq), [Dip3](http://flybase.bio.indiana.edu/.bin/fbidq.html?Dip3), [oa2](http://flybase.bio.indiana.edu/.bin/fbidq.html?oa2), [phl](http://flybase.bio.indiana.edu/.bin/fbidq.html?phl), [sNPF](http://flybase.bio.indiana.edu/.bin/fbidq.html?sNPF), [CASK](http://flybase.bio.indiana.edu/.bin/fbidq.html?CASK), [ds](http://flybase.bio.indiana.edu/.bin/fbidq.html?ds), [Syt4](http://flybase.bio.indiana.edu/.bin/fbidq.html?Syt4), [Alk](http://flybase.bio.indiana.edu/.bin/fbidq.html?Alk), [Dat](http://flybase.bio.indiana.edu/.bin/fbidq.html?Dat), [Vang](http://flybase.bio.indiana.edu/.bin/fbidq.html?Vang), [Sin3A](http://flybase.bio.indiana.edu/.bin/fbidq.html?Sin3A), [cpx](http://flybase.bio.indiana.edu/.bin/fbidq.html?cpx), [ush](http://flybase.bio.indiana.edu/.bin/fbidq.html?ush), [Fak56D](http://flybase.bio.indiana.edu/.bin/fbidq.html?Fak56D), [Gap1](http://flybase.bio.indiana.edu/.bin/fbidq.html?Gap1), [brat](http://flybase.bio.indiana.edu/.bin/fbidq.html?brat), [sNPF-R](http://flybase.bio.indiana.edu/.bin/fbidq.html?sNPF-R), [Rdl](http://flybase.bio.indiana.edu/.bin/fbidq.html?Rdl), [stan](http://flybase.bio.indiana.edu/.bin/fbidq.html?stan), [GABA-B-R2](http://flybase.bio.indiana.edu/.bin/fbidq.html?GABA-B-R2), [tutl](http://flybase.bio.indiana.edu/.bin/fbidq.html?tutl), [Ptth](http://flybase.bio.indiana.edu/.bin/fbidq.html?Ptth), [Ast-C](http://flybase.bio.indiana.edu/.bin/fbidq.html?Ast-C), [futsch](http://flybase.bio.indiana.edu/.bin/fbidq.html?futsch), [Ilp2](http://flybase.bio.indiana.edu/.bin/fbidq.html?Ilp2), [Ubx](http://flybase.bio.indiana.edu/.bin/fbidq.html?Ubx), [Frq1](http://flybase.bio.indiana.edu/.bin/fbidq.html?Frq1), [Syn2](http://flybase.bio.indiana.edu/.bin/fbidq.html?Syn2), [tsh](http://flybase.bio.indiana.edu/.bin/fbidq.html?tsh), [Cdk5](http://flybase.bio.indiana.edu/.bin/fbidq.html?Cdk5), [sif](http://flybase.bio.indiana.edu/.bin/fbidq.html?sif), [oc](http://flybase.bio.indiana.edu/.bin/fbidq.html?oc), [dom](http://flybase.bio.indiana.edu/.bin/fbidq.html?dom), [CG4641](http://flybase.bio.indiana.edu/.bin/fbidq.html?CG4641), [ssh](http://flybase.bio.indiana.edu/.bin/fbidq.html?ssh), [ey](http://flybase.bio.indiana.edu/.bin/fbidq.html?ey),[PIP5K59B](http://flybase.bio.indiana.edu/.bin/fbidq.html?PIP5K59B), [baz](http://flybase.bio.indiana.edu/.bin/fbidq.html?baz), [Oamb](http://flybase.bio.indiana.edu/.bin/fbidq.html?Oamb), [shi](http://flybase.bio.indiana.edu/.bin/fbidq.html?shi), [CG11155](http://flybase.bio.indiana.edu/.bin/fbidq.html?CG11155), [rst](http://flybase.bio.indiana.edu/.bin/fbidq.html?rst), [Bgb](http://flybase.bio.indiana.edu/.bin/fbidq.html?Bgb), [tyn](http://flybase.bio.indiana.edu/.bin/fbidq.html?tyn), [CG3822](http://flybase.bio.indiana.edu/.bin/fbidq.html?CG3822), [wg](http://flybase.bio.indiana.edu/.bin/fbidq.html?wg), [fkh](http://flybase.bio.indiana.edu/.bin/fbidq.html?fkh), [CadN](http://flybase.bio.indiana.edu/.bin/fbidq.html?CadN), [sfl](http://flybase.bio.indiana.edu/.bin/fbidq.html?sfl), [Syt1](http://flybase.bio.indiana.edu/.bin/fbidq.html?Syt1), [rl](http://flybase.bio.indiana.edu/.bin/fbidq.html?rl), [Dms](http://flybase.bio.indiana.edu/.bin/fbidq.html?Dms), [pburs](http://flybase.bio.indiana.edu/.bin/fbidq.html?pburs), [hiw](http://flybase.bio.indiana.edu/.bin/fbidq.html?hiw), [CG32653](http://flybase.bio.indiana.edu/.bin/fbidq.html?CG32653) |
| [regulation of metabolic process](http://amigo.geneontology.org/cgi-bin/amigo/go.cgi?view=details&query=GO:0019222) | 284 of 1594 genes, 17.8% | 1062 of 7634 genes, 13.9% | 5.54e-06 | 0.00% | 0.00 | [fz2](http://flybase.bio.indiana.edu/.bin/fbidq.html?fz2), [Ets65A](http://flybase.bio.indiana.edu/.bin/fbidq.html?Ets65A), [tna](http://flybase.bio.indiana.edu/.bin/fbidq.html?tna), [Sxl](http://flybase.bio.indiana.edu/.bin/fbidq.html?Sxl), [mub](http://flybase.bio.indiana.edu/.bin/fbidq.html?mub), [JIL-1](http://flybase.bio.indiana.edu/.bin/fbidq.html?JIL-1), [rn](http://flybase.bio.indiana.edu/.bin/fbidq.html?rn), [ap](http://flybase.bio.indiana.edu/.bin/fbidq.html?ap), [cenG1A](http://flybase.bio.indiana.edu/.bin/fbidq.html?cenG1A), [Nf1](http://flybase.bio.indiana.edu/.bin/fbidq.html?Nf1), [jigr1](http://flybase.bio.indiana.edu/.bin/fbidq.html?jigr1), [tok](http://flybase.bio.indiana.edu/.bin/fbidq.html?tok), [bi](http://flybase.bio.indiana.edu/.bin/fbidq.html?bi), [Hcf](http://flybase.bio.indiana.edu/.bin/fbidq.html?Hcf), [Traf6](http://flybase.bio.indiana.edu/.bin/fbidq.html?Traf6), [NC2alpha](http://flybase.bio.indiana.edu/.bin/fbidq.html?NC2alpha), [bab1](http://flybase.bio.indiana.edu/.bin/fbidq.html?bab1), [Galpha49B](http://flybase.bio.indiana.edu/.bin/fbidq.html?Galpha49B), [Snoo](http://flybase.bio.indiana.edu/.bin/fbidq.html?Snoo), [scrib](http://flybase.bio.indiana.edu/.bin/fbidq.html?scrib), [HGTX](http://flybase.bio.indiana.edu/.bin/fbidq.html?HGTX), [not](http://flybase.bio.indiana.edu/.bin/fbidq.html?not), [nvy](http://flybase.bio.indiana.edu/.bin/fbidq.html?nvy), [nub](http://flybase.bio.indiana.edu/.bin/fbidq.html?nub), [nab](http://flybase.bio.indiana.edu/.bin/fbidq.html?nab), [btd](http://flybase.bio.indiana.edu/.bin/fbidq.html?btd), [fd68A](http://flybase.bio.indiana.edu/.bin/fbidq.html?fd68A), [U2af38](http://flybase.bio.indiana.edu/.bin/fbidq.html?U2af38), [HLHmdelta](http://flybase.bio.indiana.edu/.bin/fbidq.html?HLHmdelta), [B-H1](http://flybase.bio.indiana.edu/.bin/fbidq.html?B-H1), [Nipped-A](http://flybase.bio.indiana.edu/.bin/fbidq.html?Nipped-A), [ATbp](http://flybase.bio.indiana.edu/.bin/fbidq.html?ATbp), [7B2](http://flybase.bio.indiana.edu/.bin/fbidq.html?7B2), [alph](http://flybase.bio.indiana.edu/.bin/fbidq.html?alph), [Spf45](http://flybase.bio.indiana.edu/.bin/fbidq.html?Spf45), [Fer2](http://flybase.bio.indiana.edu/.bin/fbidq.html?Fer2), [fd102C](http://flybase.bio.indiana.edu/.bin/fbidq.html?fd102C), [hth](http://flybase.bio.indiana.edu/.bin/fbidq.html?hth),[Nf-YC](http://flybase.bio.indiana.edu/.bin/fbidq.html?Nf-YC), [14-3-3zeta](http://flybase.bio.indiana.edu/.bin/fbidq.html?14-3-3zeta), [Pli](http://flybase.bio.indiana.edu/.bin/fbidq.html?Pli), [Hel25E](http://flybase.bio.indiana.edu/.bin/fbidq.html?Hel25E), [Hey](http://flybase.bio.indiana.edu/.bin/fbidq.html?Hey), [loqs](http://flybase.bio.indiana.edu/.bin/fbidq.html?loqs), [CycE](http://flybase.bio.indiana.edu/.bin/fbidq.html?CycE), [Brf](http://flybase.bio.indiana.edu/.bin/fbidq.html?Brf), [Psi](http://flybase.bio.indiana.edu/.bin/fbidq.html?Psi), [Vdup1](http://flybase.bio.indiana.edu/.bin/fbidq.html?Vdup1), [Gug](http://flybase.bio.indiana.edu/.bin/fbidq.html?Gug), [pad](http://flybase.bio.indiana.edu/.bin/fbidq.html?pad), [orb2](http://flybase.bio.indiana.edu/.bin/fbidq.html?orb2), [Rx](http://flybase.bio.indiana.edu/.bin/fbidq.html?Rx), [MED1](http://flybase.bio.indiana.edu/.bin/fbidq.html?MED1), [salr](http://flybase.bio.indiana.edu/.bin/fbidq.html?salr), [wdn](http://flybase.bio.indiana.edu/.bin/fbidq.html?wdn), [sim](http://flybase.bio.indiana.edu/.bin/fbidq.html?sim), [d4](http://flybase.bio.indiana.edu/.bin/fbidq.html?d4), [cort](http://flybase.bio.indiana.edu/.bin/fbidq.html?cort), [dan](http://flybase.bio.indiana.edu/.bin/fbidq.html?dan), [gro](http://flybase.bio.indiana.edu/.bin/fbidq.html?gro), [Jarid2](http://flybase.bio.indiana.edu/.bin/fbidq.html?Jarid2), [RN-tre](http://flybase.bio.indiana.edu/.bin/fbidq.html?RN-tre), [hkl](http://flybase.bio.indiana.edu/.bin/fbidq.html?hkl), [bin3](http://flybase.bio.indiana.edu/.bin/fbidq.html?bin3), [trx](http://flybase.bio.indiana.edu/.bin/fbidq.html?trx), [Rpd3](http://flybase.bio.indiana.edu/.bin/fbidq.html?Rpd3), [MED25](http://flybase.bio.indiana.edu/.bin/fbidq.html?MED25), [tyf](http://flybase.bio.indiana.edu/.bin/fbidq.html?tyf), [vfl](http://flybase.bio.indiana.edu/.bin/fbidq.html?vfl), [aret](http://flybase.bio.indiana.edu/.bin/fbidq.html?aret), [pygo](http://flybase.bio.indiana.edu/.bin/fbidq.html?pygo), [HLHm7](http://flybase.bio.indiana.edu/.bin/fbidq.html?HLHm7), [z](http://flybase.bio.indiana.edu/.bin/fbidq.html?z), [HLHmgamma](http://flybase.bio.indiana.edu/.bin/fbidq.html?HLHmgamma), [Sox21b](http://flybase.bio.indiana.edu/.bin/fbidq.html?Sox21b), [wor](http://flybase.bio.indiana.edu/.bin/fbidq.html?wor), [Atg1](http://flybase.bio.indiana.edu/.bin/fbidq.html?Atg1), [Pten](http://flybase.bio.indiana.edu/.bin/fbidq.html?Pten),[wda](http://flybase.bio.indiana.edu/.bin/fbidq.html?wda), [mira](http://flybase.bio.indiana.edu/.bin/fbidq.html?mira), [MED14](http://flybase.bio.indiana.edu/.bin/fbidq.html?MED14), [pUf68](http://flybase.bio.indiana.edu/.bin/fbidq.html?pUf68), [seq](http://flybase.bio.indiana.edu/.bin/fbidq.html?seq), [Dip3](http://flybase.bio.indiana.edu/.bin/fbidq.html?Dip3), [HDAC4](http://flybase.bio.indiana.edu/.bin/fbidq.html?HDAC4), [elk](http://flybase.bio.indiana.edu/.bin/fbidq.html?elk), [phl](http://flybase.bio.indiana.edu/.bin/fbidq.html?phl), [Cbl](http://flybase.bio.indiana.edu/.bin/fbidq.html?Cbl), [sNPF](http://flybase.bio.indiana.edu/.bin/fbidq.html?sNPF), [pog](http://flybase.bio.indiana.edu/.bin/fbidq.html?pog), [Pdp1](http://flybase.bio.indiana.edu/.bin/fbidq.html?Pdp1), [plx](http://flybase.bio.indiana.edu/.bin/fbidq.html?plx), [Alk](http://flybase.bio.indiana.edu/.bin/fbidq.html?Alk), [Sin3A](http://flybase.bio.indiana.edu/.bin/fbidq.html?Sin3A), [Pde1c](http://flybase.bio.indiana.edu/.bin/fbidq.html?Pde1c), [mip120](http://flybase.bio.indiana.edu/.bin/fbidq.html?mip120), [casp](http://flybase.bio.indiana.edu/.bin/fbidq.html?casp), [jumu](http://flybase.bio.indiana.edu/.bin/fbidq.html?jumu), [Smox](http://flybase.bio.indiana.edu/.bin/fbidq.html?Smox), [Su(var)2-HP2](http://flybase.bio.indiana.edu/.bin/fbidq.html?Su(var)2-HP2), [Usp7](http://flybase.bio.indiana.edu/.bin/fbidq.html?Usp7), [fd59A](http://flybase.bio.indiana.edu/.bin/fbidq.html?fd59A), [brat](http://flybase.bio.indiana.edu/.bin/fbidq.html?brat), [FBX011](http://flybase.bio.indiana.edu/.bin/fbidq.html?FBX011), [Antp](http://flybase.bio.indiana.edu/.bin/fbidq.html?Antp), [l(3)neo38](http://flybase.bio.indiana.edu/.bin/fbidq.html?l(3)neo38), [Ast-C](http://flybase.bio.indiana.edu/.bin/fbidq.html?Ast-C), [Ubx](http://flybase.bio.indiana.edu/.bin/fbidq.html?Ubx), [Ptx1](http://flybase.bio.indiana.edu/.bin/fbidq.html?Ptx1), [chm](http://flybase.bio.indiana.edu/.bin/fbidq.html?chm), [msl-1](http://flybase.bio.indiana.edu/.bin/fbidq.html?msl-1), [hbn](http://flybase.bio.indiana.edu/.bin/fbidq.html?hbn), [tsh](http://flybase.bio.indiana.edu/.bin/fbidq.html?tsh), [Rgl](http://flybase.bio.indiana.edu/.bin/fbidq.html?Rgl), [dac](http://flybase.bio.indiana.edu/.bin/fbidq.html?dac),[e(y)3](http://flybase.bio.indiana.edu/.bin/fbidq.html?e(y)3), [CG9007](http://flybase.bio.indiana.edu/.bin/fbidq.html?CG9007), [oc](http://flybase.bio.indiana.edu/.bin/fbidq.html?oc), [spt4](http://flybase.bio.indiana.edu/.bin/fbidq.html?spt4), [gol](http://flybase.bio.indiana.edu/.bin/fbidq.html?gol), [ey](http://flybase.bio.indiana.edu/.bin/fbidq.html?ey), [Bgb](http://flybase.bio.indiana.edu/.bin/fbidq.html?Bgb), [l(1)sc](http://flybase.bio.indiana.edu/.bin/fbidq.html?l(1)sc), [comm](http://flybase.bio.indiana.edu/.bin/fbidq.html?comm), [grh](http://flybase.bio.indiana.edu/.bin/fbidq.html?grh), [Vsx1](http://flybase.bio.indiana.edu/.bin/fbidq.html?Vsx1), [toy](http://flybase.bio.indiana.edu/.bin/fbidq.html?toy), [Lim3](http://flybase.bio.indiana.edu/.bin/fbidq.html?Lim3), [CG34362](http://flybase.bio.indiana.edu/.bin/fbidq.html?CG34362), [crol](http://flybase.bio.indiana.edu/.bin/fbidq.html?crol), [sd](http://flybase.bio.indiana.edu/.bin/fbidq.html?sd), [retn](http://flybase.bio.indiana.edu/.bin/fbidq.html?retn), [fs(1)h](http://flybase.bio.indiana.edu/.bin/fbidq.html?fs(1)h), [rl](http://flybase.bio.indiana.edu/.bin/fbidq.html?rl), [CG11347](http://flybase.bio.indiana.edu/.bin/fbidq.html?CG11347), [tey](http://flybase.bio.indiana.edu/.bin/fbidq.html?tey), [Saf-B](http://flybase.bio.indiana.edu/.bin/fbidq.html?Saf-B), [CG11294](http://flybase.bio.indiana.edu/.bin/fbidq.html?CG11294), [hiw](http://flybase.bio.indiana.edu/.bin/fbidq.html?hiw), [Atf-2](http://flybase.bio.indiana.edu/.bin/fbidq.html?Atf-2), [dnr1](http://flybase.bio.indiana.edu/.bin/fbidq.html?dnr1), [acj6](http://flybase.bio.indiana.edu/.bin/fbidq.html?acj6), [skd](http://flybase.bio.indiana.edu/.bin/fbidq.html?skd), [jing](http://flybase.bio.indiana.edu/.bin/fbidq.html?jing), [caup](http://flybase.bio.indiana.edu/.bin/fbidq.html?caup), [Camta](http://flybase.bio.indiana.edu/.bin/fbidq.html?Camta), [tll](http://flybase.bio.indiana.edu/.bin/fbidq.html?tll), [Cbp80](http://flybase.bio.indiana.edu/.bin/fbidq.html?Cbp80), [ato](http://flybase.bio.indiana.edu/.bin/fbidq.html?ato), [Rox8](http://flybase.bio.indiana.edu/.bin/fbidq.html?Rox8), [Iswi](http://flybase.bio.indiana.edu/.bin/fbidq.html?Iswi), [Spt6](http://flybase.bio.indiana.edu/.bin/fbidq.html?Spt6), [Dg](http://flybase.bio.indiana.edu/.bin/fbidq.html?Dg), [Pka-R1](http://flybase.bio.indiana.edu/.bin/fbidq.html?Pka-R1), [Evi5](http://flybase.bio.indiana.edu/.bin/fbidq.html?Evi5), [br](http://flybase.bio.indiana.edu/.bin/fbidq.html?br),[ksr](http://flybase.bio.indiana.edu/.bin/fbidq.html?ksr), [PNUTS](http://flybase.bio.indiana.edu/.bin/fbidq.html?PNUTS), [chn](http://flybase.bio.indiana.edu/.bin/fbidq.html?chn), [Gsc](http://flybase.bio.indiana.edu/.bin/fbidq.html?Gsc), [CG17124](http://flybase.bio.indiana.edu/.bin/fbidq.html?CG17124), [lola](http://flybase.bio.indiana.edu/.bin/fbidq.html?lola), [chif](http://flybase.bio.indiana.edu/.bin/fbidq.html?chif), [Pcl](http://flybase.bio.indiana.edu/.bin/fbidq.html?Pcl), [HLH4C](http://flybase.bio.indiana.edu/.bin/fbidq.html?HLH4C), [simj](http://flybase.bio.indiana.edu/.bin/fbidq.html?simj), [tap](http://flybase.bio.indiana.edu/.bin/fbidq.html?tap), [Awh](http://flybase.bio.indiana.edu/.bin/fbidq.html?Awh), [PHDP](http://flybase.bio.indiana.edu/.bin/fbidq.html?PHDP), [CG4328](http://flybase.bio.indiana.edu/.bin/fbidq.html?CG4328), [Su(var)3-3](http://flybase.bio.indiana.edu/.bin/fbidq.html?Su(var)3-3), [Taf4](http://flybase.bio.indiana.edu/.bin/fbidq.html?Taf4), [l(2)NC136](http://flybase.bio.indiana.edu/.bin/fbidq.html?l(2)NC136), [stau](http://flybase.bio.indiana.edu/.bin/fbidq.html?stau), [Sos](http://flybase.bio.indiana.edu/.bin/fbidq.html?Sos), [msl-2](http://flybase.bio.indiana.edu/.bin/fbidq.html?msl-2), [vg](http://flybase.bio.indiana.edu/.bin/fbidq.html?vg), [eya](http://flybase.bio.indiana.edu/.bin/fbidq.html?eya), [bsk](http://flybase.bio.indiana.edu/.bin/fbidq.html?bsk), [brm](http://flybase.bio.indiana.edu/.bin/fbidq.html?brm), [fz](http://flybase.bio.indiana.edu/.bin/fbidq.html?fz), [Vsx2](http://flybase.bio.indiana.edu/.bin/fbidq.html?Vsx2), [sno](http://flybase.bio.indiana.edu/.bin/fbidq.html?sno), [ara](http://flybase.bio.indiana.edu/.bin/fbidq.html?ara), [Sfmbt](http://flybase.bio.indiana.edu/.bin/fbidq.html?Sfmbt), [mam](http://flybase.bio.indiana.edu/.bin/fbidq.html?mam), [pan](http://flybase.bio.indiana.edu/.bin/fbidq.html?pan), [disco](http://flybase.bio.indiana.edu/.bin/fbidq.html?disco), [Dll](http://flybase.bio.indiana.edu/.bin/fbidq.html?Dll), [grn](http://flybase.bio.indiana.edu/.bin/fbidq.html?grn), [D12](http://flybase.bio.indiana.edu/.bin/fbidq.html?D12), [HLHm5](http://flybase.bio.indiana.edu/.bin/fbidq.html?HLHm5), [Lis-1](http://flybase.bio.indiana.edu/.bin/fbidq.html?Lis-1), [dap](http://flybase.bio.indiana.edu/.bin/fbidq.html?dap), [ems](http://flybase.bio.indiana.edu/.bin/fbidq.html?ems),[CG16896](http://flybase.bio.indiana.edu/.bin/fbidq.html?CG16896), [pros](http://flybase.bio.indiana.edu/.bin/fbidq.html?pros), [tup](http://flybase.bio.indiana.edu/.bin/fbidq.html?tup), [pdm2](http://flybase.bio.indiana.edu/.bin/fbidq.html?pdm2), [rgr](http://flybase.bio.indiana.edu/.bin/fbidq.html?rgr), [spel1](http://flybase.bio.indiana.edu/.bin/fbidq.html?spel1), [Wnt4](http://flybase.bio.indiana.edu/.bin/fbidq.html?Wnt4), [CG30372](http://flybase.bio.indiana.edu/.bin/fbidq.html?CG30372), [CG42629](http://flybase.bio.indiana.edu/.bin/fbidq.html?CG42629), [nerfin-1](http://flybase.bio.indiana.edu/.bin/fbidq.html?nerfin-1), [pr-set7](http://flybase.bio.indiana.edu/.bin/fbidq.html?pr-set7), [bchs](http://flybase.bio.indiana.edu/.bin/fbidq.html?bchs), [dsx](http://flybase.bio.indiana.edu/.bin/fbidq.html?dsx), [Ssdp](http://flybase.bio.indiana.edu/.bin/fbidq.html?Ssdp), [sqz](http://flybase.bio.indiana.edu/.bin/fbidq.html?sqz), [CG8155](http://flybase.bio.indiana.edu/.bin/fbidq.html?CG8155), [Fer3](http://flybase.bio.indiana.edu/.bin/fbidq.html?Fer3), [CG32105](http://flybase.bio.indiana.edu/.bin/fbidq.html?CG32105), [CG1868](http://flybase.bio.indiana.edu/.bin/fbidq.html?CG1868), [dalao](http://flybase.bio.indiana.edu/.bin/fbidq.html?dalao), [Lim1](http://flybase.bio.indiana.edu/.bin/fbidq.html?Lim1), [sr](http://flybase.bio.indiana.edu/.bin/fbidq.html?sr), [tld](http://flybase.bio.indiana.edu/.bin/fbidq.html?tld), [danr](http://flybase.bio.indiana.edu/.bin/fbidq.html?danr), [vvl](http://flybase.bio.indiana.edu/.bin/fbidq.html?vvl), [CG7879](http://flybase.bio.indiana.edu/.bin/fbidq.html?CG7879), [Brd8](http://flybase.bio.indiana.edu/.bin/fbidq.html?Brd8), [sgg](http://flybase.bio.indiana.edu/.bin/fbidq.html?sgg), [gcm2](http://flybase.bio.indiana.edu/.bin/fbidq.html?gcm2), [Eip93F](http://flybase.bio.indiana.edu/.bin/fbidq.html?Eip93F), [gcm](http://flybase.bio.indiana.edu/.bin/fbidq.html?gcm), [Suv4-20](http://flybase.bio.indiana.edu/.bin/fbidq.html?Suv4-20), [Aef1](http://flybase.bio.indiana.edu/.bin/fbidq.html?Aef1), [can](http://flybase.bio.indiana.edu/.bin/fbidq.html?can),[Hsp67Bc](http://flybase.bio.indiana.edu/.bin/fbidq.html?Hsp67Bc), [rab3-GEF](http://flybase.bio.indiana.edu/.bin/fbidq.html?rab3-GEF), [ase](http://flybase.bio.indiana.edu/.bin/fbidq.html?ase), [CG13253](http://flybase.bio.indiana.edu/.bin/fbidq.html?CG13253), [ovo](http://flybase.bio.indiana.edu/.bin/fbidq.html?ovo), [CG6197](http://flybase.bio.indiana.edu/.bin/fbidq.html?CG6197), [scro](http://flybase.bio.indiana.edu/.bin/fbidq.html?scro), [phol](http://flybase.bio.indiana.edu/.bin/fbidq.html?phol), [Dsp1](http://flybase.bio.indiana.edu/.bin/fbidq.html?Dsp1), [elav](http://flybase.bio.indiana.edu/.bin/fbidq.html?elav), [CG14216](http://flybase.bio.indiana.edu/.bin/fbidq.html?CG14216), [Oli](http://flybase.bio.indiana.edu/.bin/fbidq.html?Oli), [Nelf-A](http://flybase.bio.indiana.edu/.bin/fbidq.html?Nelf-A), [GATAd](http://flybase.bio.indiana.edu/.bin/fbidq.html?GATAd), [CG6227](http://flybase.bio.indiana.edu/.bin/fbidq.html?CG6227), [Poxn](http://flybase.bio.indiana.edu/.bin/fbidq.html?Poxn), [fbl6](http://flybase.bio.indiana.edu/.bin/fbidq.html?fbl6), [CG15376](http://flybase.bio.indiana.edu/.bin/fbidq.html?CG15376), [ush](http://flybase.bio.indiana.edu/.bin/fbidq.html?ush), [CG10336](http://flybase.bio.indiana.edu/.bin/fbidq.html?CG10336), [Gap1](http://flybase.bio.indiana.edu/.bin/fbidq.html?Gap1), [run](http://flybase.bio.indiana.edu/.bin/fbidq.html?run), [tara](http://flybase.bio.indiana.edu/.bin/fbidq.html?tara), [Alh](http://flybase.bio.indiana.edu/.bin/fbidq.html?Alh), [Hel89B](http://flybase.bio.indiana.edu/.bin/fbidq.html?Hel89B), [egg](http://flybase.bio.indiana.edu/.bin/fbidq.html?egg), [Sox102F](http://flybase.bio.indiana.edu/.bin/fbidq.html?Sox102F), [CG5337](http://flybase.bio.indiana.edu/.bin/fbidq.html?CG5337), [koko](http://flybase.bio.indiana.edu/.bin/fbidq.html?koko), [Ptth](http://flybase.bio.indiana.edu/.bin/fbidq.html?Ptth), [Sp1](http://flybase.bio.indiana.edu/.bin/fbidq.html?Sp1), [ph-p](http://flybase.bio.indiana.edu/.bin/fbidq.html?ph-p), [onecut](http://flybase.bio.indiana.edu/.bin/fbidq.html?onecut),[CG7757](http://flybase.bio.indiana.edu/.bin/fbidq.html?CG7757), [sna](http://flybase.bio.indiana.edu/.bin/fbidq.html?sna), [Wnt5](http://flybase.bio.indiana.edu/.bin/fbidq.html?Wnt5), [Taf1](http://flybase.bio.indiana.edu/.bin/fbidq.html?Taf1), [MBD-like](http://flybase.bio.indiana.edu/.bin/fbidq.html?MBD-like), [esg](http://flybase.bio.indiana.edu/.bin/fbidq.html?esg), [sif](http://flybase.bio.indiana.edu/.bin/fbidq.html?sif), [dom](http://flybase.bio.indiana.edu/.bin/fbidq.html?dom), [CG32532](http://flybase.bio.indiana.edu/.bin/fbidq.html?CG32532), [MTA1-like](http://flybase.bio.indiana.edu/.bin/fbidq.html?MTA1-like), [mle](http://flybase.bio.indiana.edu/.bin/fbidq.html?mle), [Hr51](http://flybase.bio.indiana.edu/.bin/fbidq.html?Hr51), [CG8786](http://flybase.bio.indiana.edu/.bin/fbidq.html?CG8786), [B-H2](http://flybase.bio.indiana.edu/.bin/fbidq.html?B-H2), [wg](http://flybase.bio.indiana.edu/.bin/fbidq.html?wg), [fkh](http://flybase.bio.indiana.edu/.bin/fbidq.html?fkh), [Spps](http://flybase.bio.indiana.edu/.bin/fbidq.html?Spps), [fne](http://flybase.bio.indiana.edu/.bin/fbidq.html?fne), [en](http://flybase.bio.indiana.edu/.bin/fbidq.html?en), [Utx](http://flybase.bio.indiana.edu/.bin/fbidq.html?Utx), [cos](http://flybase.bio.indiana.edu/.bin/fbidq.html?cos), [Ct](http://flybase.bio.indiana.edu/.bin/fbidq.html?Ct) |
| [response to external stimulus](http://amigo.geneontology.org/cgi-bin/amigo/go.cgi?view=details&query=GO:0009605) | 112 of 1594 genes, 7.0% | 338 of 7634 genes, 4.4% | 6.12e-06 | 0.00% | 0.00 | [fz2](http://flybase.bio.indiana.edu/.bin/fbidq.html?fz2), [Sh](http://flybase.bio.indiana.edu/.bin/fbidq.html?Sh), [acj6](http://flybase.bio.indiana.edu/.bin/fbidq.html?acj6), [Atg1](http://flybase.bio.indiana.edu/.bin/fbidq.html?Atg1), [vvl](http://flybase.bio.indiana.edu/.bin/fbidq.html?vvl), [jing](http://flybase.bio.indiana.edu/.bin/fbidq.html?jing), [dock](http://flybase.bio.indiana.edu/.bin/fbidq.html?dock), [Pten](http://flybase.bio.indiana.edu/.bin/fbidq.html?Pten), [npf](http://flybase.bio.indiana.edu/.bin/fbidq.html?npf), [Camta](http://flybase.bio.indiana.edu/.bin/fbidq.html?Camta), [sgg](http://flybase.bio.indiana.edu/.bin/fbidq.html?sgg), [Ptp69D](http://flybase.bio.indiana.edu/.bin/fbidq.html?Ptp69D), [hts](http://flybase.bio.indiana.edu/.bin/fbidq.html?hts), [inaE](http://flybase.bio.indiana.edu/.bin/fbidq.html?inaE), [Nmdar1](http://flybase.bio.indiana.edu/.bin/fbidq.html?Nmdar1), [mp](http://flybase.bio.indiana.edu/.bin/fbidq.html?mp), [ap](http://flybase.bio.indiana.edu/.bin/fbidq.html?ap), [Dg](http://flybase.bio.indiana.edu/.bin/fbidq.html?Dg), [seq](http://flybase.bio.indiana.edu/.bin/fbidq.html?seq), [gcm](http://flybase.bio.indiana.edu/.bin/fbidq.html?gcm), [Trim9](http://flybase.bio.indiana.edu/.bin/fbidq.html?Trim9), [jbug](http://flybase.bio.indiana.edu/.bin/fbidq.html?jbug), [Gycalpha99B](http://flybase.bio.indiana.edu/.bin/fbidq.html?Gycalpha99B), [robo3](http://flybase.bio.indiana.edu/.bin/fbidq.html?robo3), [tok](http://flybase.bio.indiana.edu/.bin/fbidq.html?tok), [Pdp1](http://flybase.bio.indiana.edu/.bin/fbidq.html?Pdp1), [nonA](http://flybase.bio.indiana.edu/.bin/fbidq.html?nonA), [Ptp99A](http://flybase.bio.indiana.edu/.bin/fbidq.html?Ptp99A), [lola](http://flybase.bio.indiana.edu/.bin/fbidq.html?lola), [unc-104](http://flybase.bio.indiana.edu/.bin/fbidq.html?unc-104), [chif](http://flybase.bio.indiana.edu/.bin/fbidq.html?chif), [NetB](http://flybase.bio.indiana.edu/.bin/fbidq.html?NetB), [Alk](http://flybase.bio.indiana.edu/.bin/fbidq.html?Alk), [rdgC](http://flybase.bio.indiana.edu/.bin/fbidq.html?rdgC), [Nrk](http://flybase.bio.indiana.edu/.bin/fbidq.html?Nrk), [Galpha49B](http://flybase.bio.indiana.edu/.bin/fbidq.html?Galpha49B), [Smox](http://flybase.bio.indiana.edu/.bin/fbidq.html?Smox), [Lar](http://flybase.bio.indiana.edu/.bin/fbidq.html?Lar), [not](http://flybase.bio.indiana.edu/.bin/fbidq.html?not), [nvy](http://flybase.bio.indiana.edu/.bin/fbidq.html?nvy),[CG7650](http://flybase.bio.indiana.edu/.bin/fbidq.html?CG7650), [gogo](http://flybase.bio.indiana.edu/.bin/fbidq.html?gogo), [fd59A](http://flybase.bio.indiana.edu/.bin/fbidq.html?fd59A), [beat-Ic](http://flybase.bio.indiana.edu/.bin/fbidq.html?beat-Ic), [Ddc](http://flybase.bio.indiana.edu/.bin/fbidq.html?Ddc), [run](http://flybase.bio.indiana.edu/.bin/fbidq.html?run), [jeb](http://flybase.bio.indiana.edu/.bin/fbidq.html?jeb), [brat](http://flybase.bio.indiana.edu/.bin/fbidq.html?brat), [CG16778](http://flybase.bio.indiana.edu/.bin/fbidq.html?CG16778), [ko](http://flybase.bio.indiana.edu/.bin/fbidq.html?ko), [Rdl](http://flybase.bio.indiana.edu/.bin/fbidq.html?Rdl), [Sema-1a](http://flybase.bio.indiana.edu/.bin/fbidq.html?Sema-1a), [beat-Ib](http://flybase.bio.indiana.edu/.bin/fbidq.html?beat-Ib), [otk](http://flybase.bio.indiana.edu/.bin/fbidq.html?otk), [stan](http://flybase.bio.indiana.edu/.bin/fbidq.html?stan), [CG12424](http://flybase.bio.indiana.edu/.bin/fbidq.html?CG12424), [CG34400](http://flybase.bio.indiana.edu/.bin/fbidq.html?CG34400), [tutl](http://flybase.bio.indiana.edu/.bin/fbidq.html?tutl), [ena](http://flybase.bio.indiana.edu/.bin/fbidq.html?ena), [dsh](http://flybase.bio.indiana.edu/.bin/fbidq.html?dsh), [Nf-YC](http://flybase.bio.indiana.edu/.bin/fbidq.html?Nf-YC), [robo](http://flybase.bio.indiana.edu/.bin/fbidq.html?robo), [lea](http://flybase.bio.indiana.edu/.bin/fbidq.html?lea), [bsk](http://flybase.bio.indiana.edu/.bin/fbidq.html?bsk), [beat-Ia](http://flybase.bio.indiana.edu/.bin/fbidq.html?beat-Ia), [shakB](http://flybase.bio.indiana.edu/.bin/fbidq.html?shakB), [gukh](http://flybase.bio.indiana.edu/.bin/fbidq.html?gukh), [plexB](http://flybase.bio.indiana.edu/.bin/fbidq.html?plexB), [Wnt5](http://flybase.bio.indiana.edu/.bin/fbidq.html?Wnt5), [Klp64D](http://flybase.bio.indiana.edu/.bin/fbidq.html?Klp64D), [beat-IIa](http://flybase.bio.indiana.edu/.bin/fbidq.html?beat-IIa), [Ggamma1](http://flybase.bio.indiana.edu/.bin/fbidq.html?Ggamma1), [jet](http://flybase.bio.indiana.edu/.bin/fbidq.html?jet), [para](http://flybase.bio.indiana.edu/.bin/fbidq.html?para), [grn](http://flybase.bio.indiana.edu/.bin/fbidq.html?grn), [sm](http://flybase.bio.indiana.edu/.bin/fbidq.html?sm),[DopR](http://flybase.bio.indiana.edu/.bin/fbidq.html?DopR), [mew](http://flybase.bio.indiana.edu/.bin/fbidq.html?mew), [neur](http://flybase.bio.indiana.edu/.bin/fbidq.html?neur), [Lis-1](http://flybase.bio.indiana.edu/.bin/fbidq.html?Lis-1), [eas](http://flybase.bio.indiana.edu/.bin/fbidq.html?eas), [dac](http://flybase.bio.indiana.edu/.bin/fbidq.html?dac), [Cdk5](http://flybase.bio.indiana.edu/.bin/fbidq.html?Cdk5), [nmo](http://flybase.bio.indiana.edu/.bin/fbidq.html?nmo), [pros](http://flybase.bio.indiana.edu/.bin/fbidq.html?pros), [tup](http://flybase.bio.indiana.edu/.bin/fbidq.html?tup), [trio](http://flybase.bio.indiana.edu/.bin/fbidq.html?trio), [lap](http://flybase.bio.indiana.edu/.bin/fbidq.html?lap), [daw](http://flybase.bio.indiana.edu/.bin/fbidq.html?daw), [shi](http://flybase.bio.indiana.edu/.bin/fbidq.html?shi), [Gad1](http://flybase.bio.indiana.edu/.bin/fbidq.html?Gad1), [5-HT1B](http://flybase.bio.indiana.edu/.bin/fbidq.html?5-HT1B), [Wnt4](http://flybase.bio.indiana.edu/.bin/fbidq.html?Wnt4), [comm](http://flybase.bio.indiana.edu/.bin/fbidq.html?comm), [Cdk5alpha](http://flybase.bio.indiana.edu/.bin/fbidq.html?Cdk5alpha), [Apc](http://flybase.bio.indiana.edu/.bin/fbidq.html?Apc), [sim](http://flybase.bio.indiana.edu/.bin/fbidq.html?sim), [Lim3](http://flybase.bio.indiana.edu/.bin/fbidq.html?Lim3), [CadN](http://flybase.bio.indiana.edu/.bin/fbidq.html?CadN), [nerfin-1](http://flybase.bio.indiana.edu/.bin/fbidq.html?nerfin-1), [trx](http://flybase.bio.indiana.edu/.bin/fbidq.html?trx), [retn](http://flybase.bio.indiana.edu/.bin/fbidq.html?retn), [en](http://flybase.bio.indiana.edu/.bin/fbidq.html?en), [dsx](http://flybase.bio.indiana.edu/.bin/fbidq.html?dsx), [unc-5](http://flybase.bio.indiana.edu/.bin/fbidq.html?unc-5), [trn](http://flybase.bio.indiana.edu/.bin/fbidq.html?trn), [CG10107](http://flybase.bio.indiana.edu/.bin/fbidq.html?CG10107), [Ggamma30A](http://flybase.bio.indiana.edu/.bin/fbidq.html?Ggamma30A), [fend](http://flybase.bio.indiana.edu/.bin/fbidq.html?fend), [CG33960](http://flybase.bio.indiana.edu/.bin/fbidq.html?CG33960), [CG4290](http://flybase.bio.indiana.edu/.bin/fbidq.html?CG4290), [HLHm7](http://flybase.bio.indiana.edu/.bin/fbidq.html?HLHm7) |
| [cellular nitrogen compound biosynthetic process](http://amigo.geneontology.org/cgi-bin/amigo/go.cgi?view=details&query=GO:0044271) | 228 of 1594 genes, 14.3% | 818 of 7634 genes, 10.7% | 6.33e-06 | 0.00% | 0.00 | [fz2](http://flybase.bio.indiana.edu/.bin/fbidq.html?fz2), [acj6](http://flybase.bio.indiana.edu/.bin/fbidq.html?acj6), [skd](http://flybase.bio.indiana.edu/.bin/fbidq.html?skd), [jing](http://flybase.bio.indiana.edu/.bin/fbidq.html?jing), [caup](http://flybase.bio.indiana.edu/.bin/fbidq.html?caup), [Tgt](http://flybase.bio.indiana.edu/.bin/fbidq.html?Tgt), [tll](http://flybase.bio.indiana.edu/.bin/fbidq.html?tll), [Camta](http://flybase.bio.indiana.edu/.bin/fbidq.html?Camta), [ato](http://flybase.bio.indiana.edu/.bin/fbidq.html?ato), [Ets65A](http://flybase.bio.indiana.edu/.bin/fbidq.html?Ets65A), [tna](http://flybase.bio.indiana.edu/.bin/fbidq.html?tna), [Prat2](http://flybase.bio.indiana.edu/.bin/fbidq.html?Prat2), [JIL-1](http://flybase.bio.indiana.edu/.bin/fbidq.html?JIL-1), [Spt6](http://flybase.bio.indiana.edu/.bin/fbidq.html?Spt6), [Iswi](http://flybase.bio.indiana.edu/.bin/fbidq.html?Iswi), [rn](http://flybase.bio.indiana.edu/.bin/fbidq.html?rn), [ap](http://flybase.bio.indiana.edu/.bin/fbidq.html?ap), [Nf1](http://flybase.bio.indiana.edu/.bin/fbidq.html?Nf1), [br](http://flybase.bio.indiana.edu/.bin/fbidq.html?br), [Gycalpha99B](http://flybase.bio.indiana.edu/.bin/fbidq.html?Gycalpha99B), [ksr](http://flybase.bio.indiana.edu/.bin/fbidq.html?ksr), [PNUTS](http://flybase.bio.indiana.edu/.bin/fbidq.html?PNUTS), [Gyc-89Da](http://flybase.bio.indiana.edu/.bin/fbidq.html?Gyc-89Da), [bi](http://flybase.bio.indiana.edu/.bin/fbidq.html?bi), [chn](http://flybase.bio.indiana.edu/.bin/fbidq.html?chn), [Gsc](http://flybase.bio.indiana.edu/.bin/fbidq.html?Gsc), [Hcf](http://flybase.bio.indiana.edu/.bin/fbidq.html?Hcf), [lola](http://flybase.bio.indiana.edu/.bin/fbidq.html?lola), [HLH4C](http://flybase.bio.indiana.edu/.bin/fbidq.html?HLH4C), [Pcl](http://flybase.bio.indiana.edu/.bin/fbidq.html?Pcl), [chif](http://flybase.bio.indiana.edu/.bin/fbidq.html?chif), [simj](http://flybase.bio.indiana.edu/.bin/fbidq.html?simj), [Awh](http://flybase.bio.indiana.edu/.bin/fbidq.html?Awh), [tap](http://flybase.bio.indiana.edu/.bin/fbidq.html?tap), [CG13550](http://flybase.bio.indiana.edu/.bin/fbidq.html?CG13550), [NC2alpha](http://flybase.bio.indiana.edu/.bin/fbidq.html?NC2alpha), [PHDP](http://flybase.bio.indiana.edu/.bin/fbidq.html?PHDP), [bab1](http://flybase.bio.indiana.edu/.bin/fbidq.html?bab1), [Taf12L](http://flybase.bio.indiana.edu/.bin/fbidq.html?Taf12L),[CG4328](http://flybase.bio.indiana.edu/.bin/fbidq.html?CG4328), [Snoo](http://flybase.bio.indiana.edu/.bin/fbidq.html?Snoo), [l(2)NC136](http://flybase.bio.indiana.edu/.bin/fbidq.html?l(2)NC136), [Taf4](http://flybase.bio.indiana.edu/.bin/fbidq.html?Taf4), [Su(var)3-3](http://flybase.bio.indiana.edu/.bin/fbidq.html?Su(var)3-3), [scrib](http://flybase.bio.indiana.edu/.bin/fbidq.html?scrib), [HGTX](http://flybase.bio.indiana.edu/.bin/fbidq.html?HGTX), [not](http://flybase.bio.indiana.edu/.bin/fbidq.html?not), [rut](http://flybase.bio.indiana.edu/.bin/fbidq.html?rut), [nvy](http://flybase.bio.indiana.edu/.bin/fbidq.html?nvy), [nub](http://flybase.bio.indiana.edu/.bin/fbidq.html?nub), [nab](http://flybase.bio.indiana.edu/.bin/fbidq.html?nab), [Ddc](http://flybase.bio.indiana.edu/.bin/fbidq.html?Ddc), [btd](http://flybase.bio.indiana.edu/.bin/fbidq.html?btd), [fd68A](http://flybase.bio.indiana.edu/.bin/fbidq.html?fd68A), [HLHmdelta](http://flybase.bio.indiana.edu/.bin/fbidq.html?HLHmdelta), [B-H1](http://flybase.bio.indiana.edu/.bin/fbidq.html?B-H1), [Nipped-A](http://flybase.bio.indiana.edu/.bin/fbidq.html?Nipped-A), [ATbp](http://flybase.bio.indiana.edu/.bin/fbidq.html?ATbp), [Sos](http://flybase.bio.indiana.edu/.bin/fbidq.html?Sos), [Fer2](http://flybase.bio.indiana.edu/.bin/fbidq.html?Fer2), [fd102C](http://flybase.bio.indiana.edu/.bin/fbidq.html?fd102C), [vg](http://flybase.bio.indiana.edu/.bin/fbidq.html?vg), [hth](http://flybase.bio.indiana.edu/.bin/fbidq.html?hth), [Nf-YC](http://flybase.bio.indiana.edu/.bin/fbidq.html?Nf-YC), [14-3-3zeta](http://flybase.bio.indiana.edu/.bin/fbidq.html?14-3-3zeta), [eya](http://flybase.bio.indiana.edu/.bin/fbidq.html?eya), [fz](http://flybase.bio.indiana.edu/.bin/fbidq.html?fz), [brm](http://flybase.bio.indiana.edu/.bin/fbidq.html?brm), [Vsx2](http://flybase.bio.indiana.edu/.bin/fbidq.html?Vsx2), [Hey](http://flybase.bio.indiana.edu/.bin/fbidq.html?Hey), [sno](http://flybase.bio.indiana.edu/.bin/fbidq.html?sno), [Sfmbt](http://flybase.bio.indiana.edu/.bin/fbidq.html?Sfmbt), [ara](http://flybase.bio.indiana.edu/.bin/fbidq.html?ara), [mam](http://flybase.bio.indiana.edu/.bin/fbidq.html?mam), [pan](http://flybase.bio.indiana.edu/.bin/fbidq.html?pan), [Dll](http://flybase.bio.indiana.edu/.bin/fbidq.html?Dll),[disco](http://flybase.bio.indiana.edu/.bin/fbidq.html?disco), [grn](http://flybase.bio.indiana.edu/.bin/fbidq.html?grn), [HLHm5](http://flybase.bio.indiana.edu/.bin/fbidq.html?HLHm5), [D12](http://flybase.bio.indiana.edu/.bin/fbidq.html?D12), [Lis-1](http://flybase.bio.indiana.edu/.bin/fbidq.html?Lis-1), [CycE](http://flybase.bio.indiana.edu/.bin/fbidq.html?CycE), [Brf](http://flybase.bio.indiana.edu/.bin/fbidq.html?Brf), [ems](http://flybase.bio.indiana.edu/.bin/fbidq.html?ems), [pros](http://flybase.bio.indiana.edu/.bin/fbidq.html?pros), [tup](http://flybase.bio.indiana.edu/.bin/fbidq.html?tup), [pdm2](http://flybase.bio.indiana.edu/.bin/fbidq.html?pdm2), [Gug](http://flybase.bio.indiana.edu/.bin/fbidq.html?Gug), [pad](http://flybase.bio.indiana.edu/.bin/fbidq.html?pad), [rgr](http://flybase.bio.indiana.edu/.bin/fbidq.html?rgr), [Gad1](http://flybase.bio.indiana.edu/.bin/fbidq.html?Gad1), [Wnt4](http://flybase.bio.indiana.edu/.bin/fbidq.html?Wnt4), [CG3714](http://flybase.bio.indiana.edu/.bin/fbidq.html?CG3714), [Rx](http://flybase.bio.indiana.edu/.bin/fbidq.html?Rx), [MED1](http://flybase.bio.indiana.edu/.bin/fbidq.html?MED1), [salr](http://flybase.bio.indiana.edu/.bin/fbidq.html?salr), [wdn](http://flybase.bio.indiana.edu/.bin/fbidq.html?wdn), [sim](http://flybase.bio.indiana.edu/.bin/fbidq.html?sim), [d4](http://flybase.bio.indiana.edu/.bin/fbidq.html?d4), [dan](http://flybase.bio.indiana.edu/.bin/fbidq.html?dan), [gro](http://flybase.bio.indiana.edu/.bin/fbidq.html?gro), [nerfin-1](http://flybase.bio.indiana.edu/.bin/fbidq.html?nerfin-1), [trx](http://flybase.bio.indiana.edu/.bin/fbidq.html?trx), [SamDC](http://flybase.bio.indiana.edu/.bin/fbidq.html?SamDC), [Rpd3](http://flybase.bio.indiana.edu/.bin/fbidq.html?Rpd3), [dsx](http://flybase.bio.indiana.edu/.bin/fbidq.html?dsx), [MED25](http://flybase.bio.indiana.edu/.bin/fbidq.html?MED25), [Ac13E](http://flybase.bio.indiana.edu/.bin/fbidq.html?Ac13E), [Ssdp](http://flybase.bio.indiana.edu/.bin/fbidq.html?Ssdp), [vfl](http://flybase.bio.indiana.edu/.bin/fbidq.html?vfl), [RpII140](http://flybase.bio.indiana.edu/.bin/fbidq.html?RpII140), [sqz](http://flybase.bio.indiana.edu/.bin/fbidq.html?sqz), [CG32105](http://flybase.bio.indiana.edu/.bin/fbidq.html?CG32105), [Fer3](http://flybase.bio.indiana.edu/.bin/fbidq.html?Fer3), [pygo](http://flybase.bio.indiana.edu/.bin/fbidq.html?pygo),[dalao](http://flybase.bio.indiana.edu/.bin/fbidq.html?dalao), [HLHm7](http://flybase.bio.indiana.edu/.bin/fbidq.html?HLHm7), [z](http://flybase.bio.indiana.edu/.bin/fbidq.html?z), [HLHmgamma](http://flybase.bio.indiana.edu/.bin/fbidq.html?HLHmgamma), [Sox21b](http://flybase.bio.indiana.edu/.bin/fbidq.html?Sox21b), [sr](http://flybase.bio.indiana.edu/.bin/fbidq.html?sr), [Lim1](http://flybase.bio.indiana.edu/.bin/fbidq.html?Lim1), [tld](http://flybase.bio.indiana.edu/.bin/fbidq.html?tld), [danr](http://flybase.bio.indiana.edu/.bin/fbidq.html?danr), [wor](http://flybase.bio.indiana.edu/.bin/fbidq.html?wor), [vvl](http://flybase.bio.indiana.edu/.bin/fbidq.html?vvl), [wda](http://flybase.bio.indiana.edu/.bin/fbidq.html?wda), [sgg](http://flybase.bio.indiana.edu/.bin/fbidq.html?sgg), [MED14](http://flybase.bio.indiana.edu/.bin/fbidq.html?MED14), [gcm2](http://flybase.bio.indiana.edu/.bin/fbidq.html?gcm2), [Eip93F](http://flybase.bio.indiana.edu/.bin/fbidq.html?Eip93F), [gcm](http://flybase.bio.indiana.edu/.bin/fbidq.html?gcm), [seq](http://flybase.bio.indiana.edu/.bin/fbidq.html?seq), [Dip3](http://flybase.bio.indiana.edu/.bin/fbidq.html?Dip3), [Aef1](http://flybase.bio.indiana.edu/.bin/fbidq.html?Aef1), [HDAC4](http://flybase.bio.indiana.edu/.bin/fbidq.html?HDAC4), [can](http://flybase.bio.indiana.edu/.bin/fbidq.html?can), [elk](http://flybase.bio.indiana.edu/.bin/fbidq.html?elk), [phl](http://flybase.bio.indiana.edu/.bin/fbidq.html?phl), [ase](http://flybase.bio.indiana.edu/.bin/fbidq.html?ase), [CG13253](http://flybase.bio.indiana.edu/.bin/fbidq.html?CG13253), [ovo](http://flybase.bio.indiana.edu/.bin/fbidq.html?ovo), [scro](http://flybase.bio.indiana.edu/.bin/fbidq.html?scro), [pog](http://flybase.bio.indiana.edu/.bin/fbidq.html?pog), [Dsp1](http://flybase.bio.indiana.edu/.bin/fbidq.html?Dsp1), [CG14216](http://flybase.bio.indiana.edu/.bin/fbidq.html?CG14216), [Pdp1](http://flybase.bio.indiana.edu/.bin/fbidq.html?Pdp1), [Oli](http://flybase.bio.indiana.edu/.bin/fbidq.html?Oli), [Nelf-A](http://flybase.bio.indiana.edu/.bin/fbidq.html?Nelf-A), [GATAd](http://flybase.bio.indiana.edu/.bin/fbidq.html?GATAd), [Poxn](http://flybase.bio.indiana.edu/.bin/fbidq.html?Poxn), [CG15376](http://flybase.bio.indiana.edu/.bin/fbidq.html?CG15376),[Sin3A](http://flybase.bio.indiana.edu/.bin/fbidq.html?Sin3A), [mip120](http://flybase.bio.indiana.edu/.bin/fbidq.html?mip120), [casp](http://flybase.bio.indiana.edu/.bin/fbidq.html?casp), [jumu](http://flybase.bio.indiana.edu/.bin/fbidq.html?jumu), [Smox](http://flybase.bio.indiana.edu/.bin/fbidq.html?Smox), [Usp7](http://flybase.bio.indiana.edu/.bin/fbidq.html?Usp7), [ush](http://flybase.bio.indiana.edu/.bin/fbidq.html?ush), [fd59A](http://flybase.bio.indiana.edu/.bin/fbidq.html?fd59A), [CG4300](http://flybase.bio.indiana.edu/.bin/fbidq.html?CG4300), [Gap1](http://flybase.bio.indiana.edu/.bin/fbidq.html?Gap1), [run](http://flybase.bio.indiana.edu/.bin/fbidq.html?run), [brat](http://flybase.bio.indiana.edu/.bin/fbidq.html?brat), [FBX011](http://flybase.bio.indiana.edu/.bin/fbidq.html?FBX011), [Antp](http://flybase.bio.indiana.edu/.bin/fbidq.html?Antp), [tara](http://flybase.bio.indiana.edu/.bin/fbidq.html?tara), [Alh](http://flybase.bio.indiana.edu/.bin/fbidq.html?Alh), [Hel89B](http://flybase.bio.indiana.edu/.bin/fbidq.html?Hel89B), [Sox102F](http://flybase.bio.indiana.edu/.bin/fbidq.html?Sox102F), [egg](http://flybase.bio.indiana.edu/.bin/fbidq.html?egg), [l(3)neo38](http://flybase.bio.indiana.edu/.bin/fbidq.html?l(3)neo38), [koko](http://flybase.bio.indiana.edu/.bin/fbidq.html?koko), [Ada](http://flybase.bio.indiana.edu/.bin/fbidq.html?Ada), [Ptth](http://flybase.bio.indiana.edu/.bin/fbidq.html?Ptth), [Sp1](http://flybase.bio.indiana.edu/.bin/fbidq.html?Sp1), [Ubx](http://flybase.bio.indiana.edu/.bin/fbidq.html?Ubx), [Ptx1](http://flybase.bio.indiana.edu/.bin/fbidq.html?Ptx1), [ph-p](http://flybase.bio.indiana.edu/.bin/fbidq.html?ph-p), [onecut](http://flybase.bio.indiana.edu/.bin/fbidq.html?onecut), [CG7757](http://flybase.bio.indiana.edu/.bin/fbidq.html?CG7757), [chm](http://flybase.bio.indiana.edu/.bin/fbidq.html?chm), [sna](http://flybase.bio.indiana.edu/.bin/fbidq.html?sna), [Wnt5](http://flybase.bio.indiana.edu/.bin/fbidq.html?Wnt5), [Taf1](http://flybase.bio.indiana.edu/.bin/fbidq.html?Taf1), [hbn](http://flybase.bio.indiana.edu/.bin/fbidq.html?hbn), [MBD-like](http://flybase.bio.indiana.edu/.bin/fbidq.html?MBD-like), [tsh](http://flybase.bio.indiana.edu/.bin/fbidq.html?tsh),[esg](http://flybase.bio.indiana.edu/.bin/fbidq.html?esg), [MED16](http://flybase.bio.indiana.edu/.bin/fbidq.html?MED16), [dac](http://flybase.bio.indiana.edu/.bin/fbidq.html?dac), [e(y)3](http://flybase.bio.indiana.edu/.bin/fbidq.html?e(y)3), [CRMP](http://flybase.bio.indiana.edu/.bin/fbidq.html?CRMP), [CG9007](http://flybase.bio.indiana.edu/.bin/fbidq.html?CG9007), [oc](http://flybase.bio.indiana.edu/.bin/fbidq.html?oc), [spt4](http://flybase.bio.indiana.edu/.bin/fbidq.html?spt4), [Tbh](http://flybase.bio.indiana.edu/.bin/fbidq.html?Tbh), [CG32532](http://flybase.bio.indiana.edu/.bin/fbidq.html?CG32532), [MTA1-like](http://flybase.bio.indiana.edu/.bin/fbidq.html?MTA1-like), [gol](http://flybase.bio.indiana.edu/.bin/fbidq.html?gol), [ey](http://flybase.bio.indiana.edu/.bin/fbidq.html?ey), [mle](http://flybase.bio.indiana.edu/.bin/fbidq.html?mle), [Hr51](http://flybase.bio.indiana.edu/.bin/fbidq.html?Hr51), [Bgb](http://flybase.bio.indiana.edu/.bin/fbidq.html?Bgb), [l(1)sc](http://flybase.bio.indiana.edu/.bin/fbidq.html?l(1)sc), [B-H2](http://flybase.bio.indiana.edu/.bin/fbidq.html?B-H2), [grh](http://flybase.bio.indiana.edu/.bin/fbidq.html?grh), [Vsx1](http://flybase.bio.indiana.edu/.bin/fbidq.html?Vsx1), [wg](http://flybase.bio.indiana.edu/.bin/fbidq.html?wg), [toy](http://flybase.bio.indiana.edu/.bin/fbidq.html?toy), [fkh](http://flybase.bio.indiana.edu/.bin/fbidq.html?fkh), [Lim3](http://flybase.bio.indiana.edu/.bin/fbidq.html?Lim3), [crol](http://flybase.bio.indiana.edu/.bin/fbidq.html?crol), [sd](http://flybase.bio.indiana.edu/.bin/fbidq.html?sd), [retn](http://flybase.bio.indiana.edu/.bin/fbidq.html?retn), [en](http://flybase.bio.indiana.edu/.bin/fbidq.html?en), [fs(1)h](http://flybase.bio.indiana.edu/.bin/fbidq.html?fs(1)h), [Ac3](http://flybase.bio.indiana.edu/.bin/fbidq.html?Ac3), [E(bx)](http://flybase.bio.indiana.edu/.bin/fbidq.html?E(bx)), [rl](http://flybase.bio.indiana.edu/.bin/fbidq.html?rl), [CG11347](http://flybase.bio.indiana.edu/.bin/fbidq.html?CG11347), [tey](http://flybase.bio.indiana.edu/.bin/fbidq.html?tey), [CG11294](http://flybase.bio.indiana.edu/.bin/fbidq.html?CG11294), [hiw](http://flybase.bio.indiana.edu/.bin/fbidq.html?hiw), [cos](http://flybase.bio.indiana.edu/.bin/fbidq.html?cos), [Ct](http://flybase.bio.indiana.edu/.bin/fbidq.html?Ct), [Atf-2](http://flybase.bio.indiana.edu/.bin/fbidq.html?Atf-2), [dnr1](http://flybase.bio.indiana.edu/.bin/fbidq.html?dnr1) |
| [sensory organ development](http://amigo.geneontology.org/cgi-bin/amigo/go.cgi?view=details&query=GO:0007423) | 121 of 1594 genes, 7.6% | 375 of 7634 genes, 4.9% | 8.44e-06 | 0.00% | 0.00 | [fz2](http://flybase.bio.indiana.edu/.bin/fbidq.html?fz2), [Ocho](http://flybase.bio.indiana.edu/.bin/fbidq.html?Ocho), [numb](http://flybase.bio.indiana.edu/.bin/fbidq.html?numb), [CG15609](http://flybase.bio.indiana.edu/.bin/fbidq.html?CG15609), [danr](http://flybase.bio.indiana.edu/.bin/fbidq.html?danr), [skd](http://flybase.bio.indiana.edu/.bin/fbidq.html?skd), [katanin-60](http://flybase.bio.indiana.edu/.bin/fbidq.html?katanin-60), [rg](http://flybase.bio.indiana.edu/.bin/fbidq.html?rg), [Pten](http://flybase.bio.indiana.edu/.bin/fbidq.html?Pten), [caup](http://flybase.bio.indiana.edu/.bin/fbidq.html?caup), [tll](http://flybase.bio.indiana.edu/.bin/fbidq.html?tll), [vimar](http://flybase.bio.indiana.edu/.bin/fbidq.html?vimar), [ato](http://flybase.bio.indiana.edu/.bin/fbidq.html?ato), [sgg](http://flybase.bio.indiana.edu/.bin/fbidq.html?sgg), [Brd](http://flybase.bio.indiana.edu/.bin/fbidq.html?Brd), [rn](http://flybase.bio.indiana.edu/.bin/fbidq.html?rn), [seq](http://flybase.bio.indiana.edu/.bin/fbidq.html?seq), [Dip3](http://flybase.bio.indiana.edu/.bin/fbidq.html?Dip3), [SP2353](http://flybase.bio.indiana.edu/.bin/fbidq.html?SP2353), [ase](http://flybase.bio.indiana.edu/.bin/fbidq.html?ase), [Cbl](http://flybase.bio.indiana.edu/.bin/fbidq.html?Cbl), [br](http://flybase.bio.indiana.edu/.bin/fbidq.html?br), [malpha](http://flybase.bio.indiana.edu/.bin/fbidq.html?malpha), [sns](http://flybase.bio.indiana.edu/.bin/fbidq.html?sns), [os](http://flybase.bio.indiana.edu/.bin/fbidq.html?os), [bi](http://flybase.bio.indiana.edu/.bin/fbidq.html?bi), [chn](http://flybase.bio.indiana.edu/.bin/fbidq.html?chn), [ds](http://flybase.bio.indiana.edu/.bin/fbidq.html?ds), [lola](http://flybase.bio.indiana.edu/.bin/fbidq.html?lola), [Alk](http://flybase.bio.indiana.edu/.bin/fbidq.html?Alk), [Poxn](http://flybase.bio.indiana.edu/.bin/fbidq.html?Poxn), [Vang](http://flybase.bio.indiana.edu/.bin/fbidq.html?Vang), [tap](http://flybase.bio.indiana.edu/.bin/fbidq.html?tap), [Nrk](http://flybase.bio.indiana.edu/.bin/fbidq.html?Nrk), [sano](http://flybase.bio.indiana.edu/.bin/fbidq.html?sano), [Galpha49B](http://flybase.bio.indiana.edu/.bin/fbidq.html?Galpha49B), [jumu](http://flybase.bio.indiana.edu/.bin/fbidq.html?jumu), [Lar](http://flybase.bio.indiana.edu/.bin/fbidq.html?Lar), [scrib](http://flybase.bio.indiana.edu/.bin/fbidq.html?scrib), [fred](http://flybase.bio.indiana.edu/.bin/fbidq.html?fred), [nvy](http://flybase.bio.indiana.edu/.bin/fbidq.html?nvy), [ush](http://flybase.bio.indiana.edu/.bin/fbidq.html?ush),[Gap1](http://flybase.bio.indiana.edu/.bin/fbidq.html?Gap1), [insc](http://flybase.bio.indiana.edu/.bin/fbidq.html?insc), [HLHmdelta](http://flybase.bio.indiana.edu/.bin/fbidq.html?HLHmdelta), [run](http://flybase.bio.indiana.edu/.bin/fbidq.html?run), [jeb](http://flybase.bio.indiana.edu/.bin/fbidq.html?jeb), [B-H1](http://flybase.bio.indiana.edu/.bin/fbidq.html?B-H1), [alph](http://flybase.bio.indiana.edu/.bin/fbidq.html?alph), [Hs3st-B](http://flybase.bio.indiana.edu/.bin/fbidq.html?Hs3st-B), [Traf4](http://flybase.bio.indiana.edu/.bin/fbidq.html?Traf4), [stan](http://flybase.bio.indiana.edu/.bin/fbidq.html?stan), [Sos](http://flybase.bio.indiana.edu/.bin/fbidq.html?Sos), [CG34400](http://flybase.bio.indiana.edu/.bin/fbidq.html?CG34400), [phyl](http://flybase.bio.indiana.edu/.bin/fbidq.html?phyl), [ena](http://flybase.bio.indiana.edu/.bin/fbidq.html?ena), [hth](http://flybase.bio.indiana.edu/.bin/fbidq.html?hth), [cindr](http://flybase.bio.indiana.edu/.bin/fbidq.html?cindr), [vg](http://flybase.bio.indiana.edu/.bin/fbidq.html?vg), [dsh](http://flybase.bio.indiana.edu/.bin/fbidq.html?dsh), [Fas2](http://flybase.bio.indiana.edu/.bin/fbidq.html?Fas2), [eya](http://flybase.bio.indiana.edu/.bin/fbidq.html?eya), [14-3-3zeta](http://flybase.bio.indiana.edu/.bin/fbidq.html?14-3-3zeta), [fz](http://flybase.bio.indiana.edu/.bin/fbidq.html?fz), [futsch](http://flybase.bio.indiana.edu/.bin/fbidq.html?futsch), [beat-Ia](http://flybase.bio.indiana.edu/.bin/fbidq.html?beat-Ia), [ft](http://flybase.bio.indiana.edu/.bin/fbidq.html?ft), [sno](http://flybase.bio.indiana.edu/.bin/fbidq.html?sno), [ara](http://flybase.bio.indiana.edu/.bin/fbidq.html?ara), [sna](http://flybase.bio.indiana.edu/.bin/fbidq.html?sna), [mam](http://flybase.bio.indiana.edu/.bin/fbidq.html?mam), [tsh](http://flybase.bio.indiana.edu/.bin/fbidq.html?tsh), [Syn2](http://flybase.bio.indiana.edu/.bin/fbidq.html?Syn2), [HLHm5](http://flybase.bio.indiana.edu/.bin/fbidq.html?HLHm5), [neur](http://flybase.bio.indiana.edu/.bin/fbidq.html?neur), [didum](http://flybase.bio.indiana.edu/.bin/fbidq.html?didum), [Lis-1](http://flybase.bio.indiana.edu/.bin/fbidq.html?Lis-1), [dac](http://flybase.bio.indiana.edu/.bin/fbidq.html?dac), [dap](http://flybase.bio.indiana.edu/.bin/fbidq.html?dap), [CycE](http://flybase.bio.indiana.edu/.bin/fbidq.html?CycE), [nmo](http://flybase.bio.indiana.edu/.bin/fbidq.html?nmo),[eys](http://flybase.bio.indiana.edu/.bin/fbidq.html?eys), [oc](http://flybase.bio.indiana.edu/.bin/fbidq.html?oc), [m4](http://flybase.bio.indiana.edu/.bin/fbidq.html?m4), [Mob2](http://flybase.bio.indiana.edu/.bin/fbidq.html?Mob2), [pros](http://flybase.bio.indiana.edu/.bin/fbidq.html?pros), [tup](http://flybase.bio.indiana.edu/.bin/fbidq.html?tup), [ey](http://flybase.bio.indiana.edu/.bin/fbidq.html?ey), [ssh](http://flybase.bio.indiana.edu/.bin/fbidq.html?ssh), [ed](http://flybase.bio.indiana.edu/.bin/fbidq.html?ed), [Gug](http://flybase.bio.indiana.edu/.bin/fbidq.html?Gug), [pad](http://flybase.bio.indiana.edu/.bin/fbidq.html?pad), [klar](http://flybase.bio.indiana.edu/.bin/fbidq.html?klar), [shi](http://flybase.bio.indiana.edu/.bin/fbidq.html?shi), [rst](http://flybase.bio.indiana.edu/.bin/fbidq.html?rst), [Wnt4](http://flybase.bio.indiana.edu/.bin/fbidq.html?Wnt4), [B-H2](http://flybase.bio.indiana.edu/.bin/fbidq.html?B-H2), [grh](http://flybase.bio.indiana.edu/.bin/fbidq.html?grh), [wg](http://flybase.bio.indiana.edu/.bin/fbidq.html?wg), [toy](http://flybase.bio.indiana.edu/.bin/fbidq.html?toy), [salr](http://flybase.bio.indiana.edu/.bin/fbidq.html?salr), [Apc](http://flybase.bio.indiana.edu/.bin/fbidq.html?Apc), [CadN](http://flybase.bio.indiana.edu/.bin/fbidq.html?CadN), [dan](http://flybase.bio.indiana.edu/.bin/fbidq.html?dan), [sd](http://flybase.bio.indiana.edu/.bin/fbidq.html?sd), [Mf](http://flybase.bio.indiana.edu/.bin/fbidq.html?Mf), [bchs](http://flybase.bio.indiana.edu/.bin/fbidq.html?bchs), [klg](http://flybase.bio.indiana.edu/.bin/fbidq.html?klg), [pk](http://flybase.bio.indiana.edu/.bin/fbidq.html?pk), [dsx](http://flybase.bio.indiana.edu/.bin/fbidq.html?dsx), [rl](http://flybase.bio.indiana.edu/.bin/fbidq.html?rl), [Snap](http://flybase.bio.indiana.edu/.bin/fbidq.html?Snap), [cdi](http://flybase.bio.indiana.edu/.bin/fbidq.html?cdi), [klu](http://flybase.bio.indiana.edu/.bin/fbidq.html?klu), [Utx](http://flybase.bio.indiana.edu/.bin/fbidq.html?Utx), [yrt](http://flybase.bio.indiana.edu/.bin/fbidq.html?yrt), [wls](http://flybase.bio.indiana.edu/.bin/fbidq.html?wls), [HLHm7](http://flybase.bio.indiana.edu/.bin/fbidq.html?HLHm7), [HLHmgamma](http://flybase.bio.indiana.edu/.bin/fbidq.html?HLHmgamma), [unk](http://flybase.bio.indiana.edu/.bin/fbidq.html?unk), [Ct](http://flybase.bio.indiana.edu/.bin/fbidq.html?Ct) |
| [central nervous system development](http://amigo.geneontology.org/cgi-bin/amigo/go.cgi?view=details&query=GO:0007417) | 71 of 1594 genes, 4.5% | 188 of 7634 genes, 2.5% | 1.45e-05 | 0.00% | 0.00 | [fz2](http://flybase.bio.indiana.edu/.bin/fbidq.html?fz2), [numb](http://flybase.bio.indiana.edu/.bin/fbidq.html?numb), [Ror](http://flybase.bio.indiana.edu/.bin/fbidq.html?Ror), [danr](http://flybase.bio.indiana.edu/.bin/fbidq.html?danr), [wor](http://flybase.bio.indiana.edu/.bin/fbidq.html?wor), [vvl](http://flybase.bio.indiana.edu/.bin/fbidq.html?vvl), [jing](http://flybase.bio.indiana.edu/.bin/fbidq.html?jing), [rg](http://flybase.bio.indiana.edu/.bin/fbidq.html?rg), [ato](http://flybase.bio.indiana.edu/.bin/fbidq.html?ato), [mnb](http://flybase.bio.indiana.edu/.bin/fbidq.html?mnb), [ase](http://flybase.bio.indiana.edu/.bin/fbidq.html?ase), [br](http://flybase.bio.indiana.edu/.bin/fbidq.html?br), [robo3](http://flybase.bio.indiana.edu/.bin/fbidq.html?robo3), [elav](http://flybase.bio.indiana.edu/.bin/fbidq.html?elav), [lola](http://flybase.bio.indiana.edu/.bin/fbidq.html?lola), [ewg](http://flybase.bio.indiana.edu/.bin/fbidq.html?ewg), [Smox](http://flybase.bio.indiana.edu/.bin/fbidq.html?Smox), [HGTX](http://flybase.bio.indiana.edu/.bin/fbidq.html?HGTX), [Nrg](http://flybase.bio.indiana.edu/.bin/fbidq.html?Nrg), [nub](http://flybase.bio.indiana.edu/.bin/fbidq.html?nub), [run](http://flybase.bio.indiana.edu/.bin/fbidq.html?run), [brat](http://flybase.bio.indiana.edu/.bin/fbidq.html?brat), [CG16778](http://flybase.bio.indiana.edu/.bin/fbidq.html?CG16778), [Sema-1a](http://flybase.bio.indiana.edu/.bin/fbidq.html?Sema-1a), [TBPH](http://flybase.bio.indiana.edu/.bin/fbidq.html?TBPH), [stan](http://flybase.bio.indiana.edu/.bin/fbidq.html?stan), [Ulp1](http://flybase.bio.indiana.edu/.bin/fbidq.html?Ulp1), [hth](http://flybase.bio.indiana.edu/.bin/fbidq.html?hth), [Fas2](http://flybase.bio.indiana.edu/.bin/fbidq.html?Fas2), [fz](http://flybase.bio.indiana.edu/.bin/fbidq.html?fz), [lea](http://flybase.bio.indiana.edu/.bin/fbidq.html?lea), [robo](http://flybase.bio.indiana.edu/.bin/fbidq.html?robo), [ph-p](http://flybase.bio.indiana.edu/.bin/fbidq.html?ph-p), [RhoGAPp190](http://flybase.bio.indiana.edu/.bin/fbidq.html?RhoGAPp190), [sna](http://flybase.bio.indiana.edu/.bin/fbidq.html?sna), [mam](http://flybase.bio.indiana.edu/.bin/fbidq.html?mam), [loqs](http://flybase.bio.indiana.edu/.bin/fbidq.html?loqs), [sws](http://flybase.bio.indiana.edu/.bin/fbidq.html?sws), [Dll](http://flybase.bio.indiana.edu/.bin/fbidq.html?Dll), [disco](http://flybase.bio.indiana.edu/.bin/fbidq.html?disco), [neur](http://flybase.bio.indiana.edu/.bin/fbidq.html?neur), [esg](http://flybase.bio.indiana.edu/.bin/fbidq.html?esg),[Lis-1](http://flybase.bio.indiana.edu/.bin/fbidq.html?Lis-1), [dac](http://flybase.bio.indiana.edu/.bin/fbidq.html?dac), [eas](http://flybase.bio.indiana.edu/.bin/fbidq.html?eas), [ems](http://flybase.bio.indiana.edu/.bin/fbidq.html?ems), [oc](http://flybase.bio.indiana.edu/.bin/fbidq.html?oc), [pros](http://flybase.bio.indiana.edu/.bin/fbidq.html?pros), [pdm2](http://flybase.bio.indiana.edu/.bin/fbidq.html?pdm2), [trio](http://flybase.bio.indiana.edu/.bin/fbidq.html?trio), [ed](http://flybase.bio.indiana.edu/.bin/fbidq.html?ed), [ssh](http://flybase.bio.indiana.edu/.bin/fbidq.html?ssh), [ey](http://flybase.bio.indiana.edu/.bin/fbidq.html?ey), [Hr51](http://flybase.bio.indiana.edu/.bin/fbidq.html?Hr51), [siz](http://flybase.bio.indiana.edu/.bin/fbidq.html?siz), [l(1)sc](http://flybase.bio.indiana.edu/.bin/fbidq.html?l(1)sc), [grh](http://flybase.bio.indiana.edu/.bin/fbidq.html?grh), [Rx](http://flybase.bio.indiana.edu/.bin/fbidq.html?Rx), [toy](http://flybase.bio.indiana.edu/.bin/fbidq.html?toy), [wg](http://flybase.bio.indiana.edu/.bin/fbidq.html?wg), [Apc](http://flybase.bio.indiana.edu/.bin/fbidq.html?Apc), [sim](http://flybase.bio.indiana.edu/.bin/fbidq.html?sim), [dan](http://flybase.bio.indiana.edu/.bin/fbidq.html?dan), [en](http://flybase.bio.indiana.edu/.bin/fbidq.html?en), [dsx](http://flybase.bio.indiana.edu/.bin/fbidq.html?dsx), [chinmo](http://flybase.bio.indiana.edu/.bin/fbidq.html?chinmo), [CG10107](http://flybase.bio.indiana.edu/.bin/fbidq.html?CG10107), [HLHm7](http://flybase.bio.indiana.edu/.bin/fbidq.html?HLHm7), [Rnp4F](http://flybase.bio.indiana.edu/.bin/fbidq.html?Rnp4F), [sr](http://flybase.bio.indiana.edu/.bin/fbidq.html?sr), [Ct](http://flybase.bio.indiana.edu/.bin/fbidq.html?Ct) |
| [transcription from RNA polymerase II promoter](http://amigo.geneontology.org/cgi-bin/amigo/go.cgi?view=details&query=GO:0006366) | 118 of 1594 genes, 7.4% | 367 of 7634 genes, 4.8% | 1.68e-05 | 0.00% | 0.00 | [fz2](http://flybase.bio.indiana.edu/.bin/fbidq.html?fz2), [tld](http://flybase.bio.indiana.edu/.bin/fbidq.html?tld), [acj6](http://flybase.bio.indiana.edu/.bin/fbidq.html?acj6), [skd](http://flybase.bio.indiana.edu/.bin/fbidq.html?skd), [wor](http://flybase.bio.indiana.edu/.bin/fbidq.html?wor), [vvl](http://flybase.bio.indiana.edu/.bin/fbidq.html?vvl), [jing](http://flybase.bio.indiana.edu/.bin/fbidq.html?jing), [caup](http://flybase.bio.indiana.edu/.bin/fbidq.html?caup), [tll](http://flybase.bio.indiana.edu/.bin/fbidq.html?tll), [Camta](http://flybase.bio.indiana.edu/.bin/fbidq.html?Camta), [ato](http://flybase.bio.indiana.edu/.bin/fbidq.html?ato), [MED14](http://flybase.bio.indiana.edu/.bin/fbidq.html?MED14), [Eip93F](http://flybase.bio.indiana.edu/.bin/fbidq.html?Eip93F), [Spt6](http://flybase.bio.indiana.edu/.bin/fbidq.html?Spt6), [Iswi](http://flybase.bio.indiana.edu/.bin/fbidq.html?Iswi), [rn](http://flybase.bio.indiana.edu/.bin/fbidq.html?rn), [ap](http://flybase.bio.indiana.edu/.bin/fbidq.html?ap), [seq](http://flybase.bio.indiana.edu/.bin/fbidq.html?seq), [gcm](http://flybase.bio.indiana.edu/.bin/fbidq.html?gcm), [Dip3](http://flybase.bio.indiana.edu/.bin/fbidq.html?Dip3), [Aef1](http://flybase.bio.indiana.edu/.bin/fbidq.html?Aef1), [can](http://flybase.bio.indiana.edu/.bin/fbidq.html?can), [phl](http://flybase.bio.indiana.edu/.bin/fbidq.html?phl), [ase](http://flybase.bio.indiana.edu/.bin/fbidq.html?ase), [CG13253](http://flybase.bio.indiana.edu/.bin/fbidq.html?CG13253), [br](http://flybase.bio.indiana.edu/.bin/fbidq.html?br), [ovo](http://flybase.bio.indiana.edu/.bin/fbidq.html?ovo), [ksr](http://flybase.bio.indiana.edu/.bin/fbidq.html?ksr), [Dsp1](http://flybase.bio.indiana.edu/.bin/fbidq.html?Dsp1), [CG14216](http://flybase.bio.indiana.edu/.bin/fbidq.html?CG14216), [bi](http://flybase.bio.indiana.edu/.bin/fbidq.html?bi), [chn](http://flybase.bio.indiana.edu/.bin/fbidq.html?chn), [lola](http://flybase.bio.indiana.edu/.bin/fbidq.html?lola), [Nelf-A](http://flybase.bio.indiana.edu/.bin/fbidq.html?Nelf-A), [simj](http://flybase.bio.indiana.edu/.bin/fbidq.html?simj), [tap](http://flybase.bio.indiana.edu/.bin/fbidq.html?tap), [CG13550](http://flybase.bio.indiana.edu/.bin/fbidq.html?CG13550), [NC2alpha](http://flybase.bio.indiana.edu/.bin/fbidq.html?NC2alpha), [mip120](http://flybase.bio.indiana.edu/.bin/fbidq.html?mip120), [Taf12L](http://flybase.bio.indiana.edu/.bin/fbidq.html?Taf12L), [Snoo](http://flybase.bio.indiana.edu/.bin/fbidq.html?Snoo),[jumu](http://flybase.bio.indiana.edu/.bin/fbidq.html?jumu), [Taf4](http://flybase.bio.indiana.edu/.bin/fbidq.html?Taf4), [HGTX](http://flybase.bio.indiana.edu/.bin/fbidq.html?HGTX), [ush](http://flybase.bio.indiana.edu/.bin/fbidq.html?ush), [fd59A](http://flybase.bio.indiana.edu/.bin/fbidq.html?fd59A), [nub](http://flybase.bio.indiana.edu/.bin/fbidq.html?nub), [Gap1](http://flybase.bio.indiana.edu/.bin/fbidq.html?Gap1), [btd](http://flybase.bio.indiana.edu/.bin/fbidq.html?btd), [fd68A](http://flybase.bio.indiana.edu/.bin/fbidq.html?fd68A), [HLHmdelta](http://flybase.bio.indiana.edu/.bin/fbidq.html?HLHmdelta), [run](http://flybase.bio.indiana.edu/.bin/fbidq.html?run), [B-H1](http://flybase.bio.indiana.edu/.bin/fbidq.html?B-H1), [Antp](http://flybase.bio.indiana.edu/.bin/fbidq.html?Antp), [ATbp](http://flybase.bio.indiana.edu/.bin/fbidq.html?ATbp), [Sos](http://flybase.bio.indiana.edu/.bin/fbidq.html?Sos), [fd102C](http://flybase.bio.indiana.edu/.bin/fbidq.html?fd102C), [Ptth](http://flybase.bio.indiana.edu/.bin/fbidq.html?Ptth), [hth](http://flybase.bio.indiana.edu/.bin/fbidq.html?hth), [vg](http://flybase.bio.indiana.edu/.bin/fbidq.html?vg), [eya](http://flybase.bio.indiana.edu/.bin/fbidq.html?eya), [fz](http://flybase.bio.indiana.edu/.bin/fbidq.html?fz), [Ubx](http://flybase.bio.indiana.edu/.bin/fbidq.html?Ubx), [Vsx2](http://flybase.bio.indiana.edu/.bin/fbidq.html?Vsx2), [Ptx1](http://flybase.bio.indiana.edu/.bin/fbidq.html?Ptx1), [onecut](http://flybase.bio.indiana.edu/.bin/fbidq.html?onecut), [sno](http://flybase.bio.indiana.edu/.bin/fbidq.html?sno), [chm](http://flybase.bio.indiana.edu/.bin/fbidq.html?chm), [ara](http://flybase.bio.indiana.edu/.bin/fbidq.html?ara), [sna](http://flybase.bio.indiana.edu/.bin/fbidq.html?sna), [Wnt5](http://flybase.bio.indiana.edu/.bin/fbidq.html?Wnt5), [mam](http://flybase.bio.indiana.edu/.bin/fbidq.html?mam), [Taf1](http://flybase.bio.indiana.edu/.bin/fbidq.html?Taf1), [pan](http://flybase.bio.indiana.edu/.bin/fbidq.html?pan), [disco](http://flybase.bio.indiana.edu/.bin/fbidq.html?disco), [grn](http://flybase.bio.indiana.edu/.bin/fbidq.html?grn), [tsh](http://flybase.bio.indiana.edu/.bin/fbidq.html?tsh), [esg](http://flybase.bio.indiana.edu/.bin/fbidq.html?esg), [Lis-1](http://flybase.bio.indiana.edu/.bin/fbidq.html?Lis-1), [MED16](http://flybase.bio.indiana.edu/.bin/fbidq.html?MED16), [oc](http://flybase.bio.indiana.edu/.bin/fbidq.html?oc), [pros](http://flybase.bio.indiana.edu/.bin/fbidq.html?pros), [tup](http://flybase.bio.indiana.edu/.bin/fbidq.html?tup),[ey](http://flybase.bio.indiana.edu/.bin/fbidq.html?ey), [pad](http://flybase.bio.indiana.edu/.bin/fbidq.html?pad), [mle](http://flybase.bio.indiana.edu/.bin/fbidq.html?mle), [Bgb](http://flybase.bio.indiana.edu/.bin/fbidq.html?Bgb), [l(1)sc](http://flybase.bio.indiana.edu/.bin/fbidq.html?l(1)sc), [Wnt4](http://flybase.bio.indiana.edu/.bin/fbidq.html?Wnt4), [B-H2](http://flybase.bio.indiana.edu/.bin/fbidq.html?B-H2), [grh](http://flybase.bio.indiana.edu/.bin/fbidq.html?grh), [Rx](http://flybase.bio.indiana.edu/.bin/fbidq.html?Rx), [MED1](http://flybase.bio.indiana.edu/.bin/fbidq.html?MED1), [Vsx1](http://flybase.bio.indiana.edu/.bin/fbidq.html?Vsx1), [wg](http://flybase.bio.indiana.edu/.bin/fbidq.html?wg), [toy](http://flybase.bio.indiana.edu/.bin/fbidq.html?toy), [fkh](http://flybase.bio.indiana.edu/.bin/fbidq.html?fkh), [sim](http://flybase.bio.indiana.edu/.bin/fbidq.html?sim), [gro](http://flybase.bio.indiana.edu/.bin/fbidq.html?gro), [crol](http://flybase.bio.indiana.edu/.bin/fbidq.html?crol), [nerfin-1](http://flybase.bio.indiana.edu/.bin/fbidq.html?nerfin-1), [sd](http://flybase.bio.indiana.edu/.bin/fbidq.html?sd), [trx](http://flybase.bio.indiana.edu/.bin/fbidq.html?trx), [Rpd3](http://flybase.bio.indiana.edu/.bin/fbidq.html?Rpd3), [retn](http://flybase.bio.indiana.edu/.bin/fbidq.html?retn), [en](http://flybase.bio.indiana.edu/.bin/fbidq.html?en), [fs(1)h](http://flybase.bio.indiana.edu/.bin/fbidq.html?fs(1)h), [dsx](http://flybase.bio.indiana.edu/.bin/fbidq.html?dsx), [rl](http://flybase.bio.indiana.edu/.bin/fbidq.html?rl), [MED25](http://flybase.bio.indiana.edu/.bin/fbidq.html?MED25), [RpII140](http://flybase.bio.indiana.edu/.bin/fbidq.html?RpII140), [CG11347](http://flybase.bio.indiana.edu/.bin/fbidq.html?CG11347), [pygo](http://flybase.bio.indiana.edu/.bin/fbidq.html?pygo), [HLHm7](http://flybase.bio.indiana.edu/.bin/fbidq.html?HLHm7), [HLHmgamma](http://flybase.bio.indiana.edu/.bin/fbidq.html?HLHmgamma), [hiw](http://flybase.bio.indiana.edu/.bin/fbidq.html?hiw), [Ct](http://flybase.bio.indiana.edu/.bin/fbidq.html?Ct), [Atf-2](http://flybase.bio.indiana.edu/.bin/fbidq.html?Atf-2) |
| [aromatic compound biosynthetic process](http://amigo.geneontology.org/cgi-bin/amigo/go.cgi?view=details&query=GO:0019438) | 219 of 1594 genes, 13.7% | 791 of 7634 genes, 10.4% | 2.59e-05 | 0.00% | 0.00 | [fz2](http://flybase.bio.indiana.edu/.bin/fbidq.html?fz2), [acj6](http://flybase.bio.indiana.edu/.bin/fbidq.html?acj6), [skd](http://flybase.bio.indiana.edu/.bin/fbidq.html?skd), [jing](http://flybase.bio.indiana.edu/.bin/fbidq.html?jing), [caup](http://flybase.bio.indiana.edu/.bin/fbidq.html?caup), [Tgt](http://flybase.bio.indiana.edu/.bin/fbidq.html?Tgt), [tll](http://flybase.bio.indiana.edu/.bin/fbidq.html?tll), [Camta](http://flybase.bio.indiana.edu/.bin/fbidq.html?Camta), [ato](http://flybase.bio.indiana.edu/.bin/fbidq.html?ato), [Ets65A](http://flybase.bio.indiana.edu/.bin/fbidq.html?Ets65A), [tna](http://flybase.bio.indiana.edu/.bin/fbidq.html?tna), [Prat2](http://flybase.bio.indiana.edu/.bin/fbidq.html?Prat2), [JIL-1](http://flybase.bio.indiana.edu/.bin/fbidq.html?JIL-1), [Spt6](http://flybase.bio.indiana.edu/.bin/fbidq.html?Spt6), [Iswi](http://flybase.bio.indiana.edu/.bin/fbidq.html?Iswi), [rn](http://flybase.bio.indiana.edu/.bin/fbidq.html?rn), [ap](http://flybase.bio.indiana.edu/.bin/fbidq.html?ap), [Nf1](http://flybase.bio.indiana.edu/.bin/fbidq.html?Nf1), [br](http://flybase.bio.indiana.edu/.bin/fbidq.html?br), [Gycalpha99B](http://flybase.bio.indiana.edu/.bin/fbidq.html?Gycalpha99B), [ksr](http://flybase.bio.indiana.edu/.bin/fbidq.html?ksr), [PNUTS](http://flybase.bio.indiana.edu/.bin/fbidq.html?PNUTS), [Gyc-89Da](http://flybase.bio.indiana.edu/.bin/fbidq.html?Gyc-89Da), [bi](http://flybase.bio.indiana.edu/.bin/fbidq.html?bi), [chn](http://flybase.bio.indiana.edu/.bin/fbidq.html?chn), [Gsc](http://flybase.bio.indiana.edu/.bin/fbidq.html?Gsc), [Hcf](http://flybase.bio.indiana.edu/.bin/fbidq.html?Hcf), [lola](http://flybase.bio.indiana.edu/.bin/fbidq.html?lola), [HLH4C](http://flybase.bio.indiana.edu/.bin/fbidq.html?HLH4C), [Pcl](http://flybase.bio.indiana.edu/.bin/fbidq.html?Pcl), [chif](http://flybase.bio.indiana.edu/.bin/fbidq.html?chif), [simj](http://flybase.bio.indiana.edu/.bin/fbidq.html?simj), [Awh](http://flybase.bio.indiana.edu/.bin/fbidq.html?Awh), [tap](http://flybase.bio.indiana.edu/.bin/fbidq.html?tap), [CG13550](http://flybase.bio.indiana.edu/.bin/fbidq.html?CG13550), [NC2alpha](http://flybase.bio.indiana.edu/.bin/fbidq.html?NC2alpha), [PHDP](http://flybase.bio.indiana.edu/.bin/fbidq.html?PHDP), [bab1](http://flybase.bio.indiana.edu/.bin/fbidq.html?bab1), [Taf12L](http://flybase.bio.indiana.edu/.bin/fbidq.html?Taf12L),[CG4328](http://flybase.bio.indiana.edu/.bin/fbidq.html?CG4328), [Snoo](http://flybase.bio.indiana.edu/.bin/fbidq.html?Snoo), [l(2)NC136](http://flybase.bio.indiana.edu/.bin/fbidq.html?l(2)NC136), [Taf4](http://flybase.bio.indiana.edu/.bin/fbidq.html?Taf4), [Su(var)3-3](http://flybase.bio.indiana.edu/.bin/fbidq.html?Su(var)3-3), [HGTX](http://flybase.bio.indiana.edu/.bin/fbidq.html?HGTX), [not](http://flybase.bio.indiana.edu/.bin/fbidq.html?not), [rut](http://flybase.bio.indiana.edu/.bin/fbidq.html?rut), [nvy](http://flybase.bio.indiana.edu/.bin/fbidq.html?nvy), [nub](http://flybase.bio.indiana.edu/.bin/fbidq.html?nub), [nab](http://flybase.bio.indiana.edu/.bin/fbidq.html?nab), [Ddc](http://flybase.bio.indiana.edu/.bin/fbidq.html?Ddc), [btd](http://flybase.bio.indiana.edu/.bin/fbidq.html?btd), [fd68A](http://flybase.bio.indiana.edu/.bin/fbidq.html?fd68A), [HLHmdelta](http://flybase.bio.indiana.edu/.bin/fbidq.html?HLHmdelta), [B-H1](http://flybase.bio.indiana.edu/.bin/fbidq.html?B-H1), [Nipped-A](http://flybase.bio.indiana.edu/.bin/fbidq.html?Nipped-A), [ATbp](http://flybase.bio.indiana.edu/.bin/fbidq.html?ATbp), [Sos](http://flybase.bio.indiana.edu/.bin/fbidq.html?Sos), [Fer2](http://flybase.bio.indiana.edu/.bin/fbidq.html?Fer2), [fd102C](http://flybase.bio.indiana.edu/.bin/fbidq.html?fd102C), [vg](http://flybase.bio.indiana.edu/.bin/fbidq.html?vg), [hth](http://flybase.bio.indiana.edu/.bin/fbidq.html?hth), [Nf-YC](http://flybase.bio.indiana.edu/.bin/fbidq.html?Nf-YC), [14-3-3zeta](http://flybase.bio.indiana.edu/.bin/fbidq.html?14-3-3zeta), [eya](http://flybase.bio.indiana.edu/.bin/fbidq.html?eya), [fz](http://flybase.bio.indiana.edu/.bin/fbidq.html?fz), [brm](http://flybase.bio.indiana.edu/.bin/fbidq.html?brm), [Vsx2](http://flybase.bio.indiana.edu/.bin/fbidq.html?Vsx2), [Hey](http://flybase.bio.indiana.edu/.bin/fbidq.html?Hey), [sno](http://flybase.bio.indiana.edu/.bin/fbidq.html?sno), [Sfmbt](http://flybase.bio.indiana.edu/.bin/fbidq.html?Sfmbt), [ara](http://flybase.bio.indiana.edu/.bin/fbidq.html?ara), [mam](http://flybase.bio.indiana.edu/.bin/fbidq.html?mam), [pan](http://flybase.bio.indiana.edu/.bin/fbidq.html?pan), [Dll](http://flybase.bio.indiana.edu/.bin/fbidq.html?Dll), [disco](http://flybase.bio.indiana.edu/.bin/fbidq.html?disco), [grn](http://flybase.bio.indiana.edu/.bin/fbidq.html?grn),[HLHm5](http://flybase.bio.indiana.edu/.bin/fbidq.html?HLHm5), [D12](http://flybase.bio.indiana.edu/.bin/fbidq.html?D12), [Lis-1](http://flybase.bio.indiana.edu/.bin/fbidq.html?Lis-1), [CycE](http://flybase.bio.indiana.edu/.bin/fbidq.html?CycE), [Brf](http://flybase.bio.indiana.edu/.bin/fbidq.html?Brf), [ems](http://flybase.bio.indiana.edu/.bin/fbidq.html?ems), [pros](http://flybase.bio.indiana.edu/.bin/fbidq.html?pros), [tup](http://flybase.bio.indiana.edu/.bin/fbidq.html?tup), [pdm2](http://flybase.bio.indiana.edu/.bin/fbidq.html?pdm2), [Gug](http://flybase.bio.indiana.edu/.bin/fbidq.html?Gug), [pad](http://flybase.bio.indiana.edu/.bin/fbidq.html?pad), [rgr](http://flybase.bio.indiana.edu/.bin/fbidq.html?rgr), [Wnt4](http://flybase.bio.indiana.edu/.bin/fbidq.html?Wnt4), [CG3714](http://flybase.bio.indiana.edu/.bin/fbidq.html?CG3714), [Rx](http://flybase.bio.indiana.edu/.bin/fbidq.html?Rx), [MED1](http://flybase.bio.indiana.edu/.bin/fbidq.html?MED1), [salr](http://flybase.bio.indiana.edu/.bin/fbidq.html?salr), [wdn](http://flybase.bio.indiana.edu/.bin/fbidq.html?wdn), [sim](http://flybase.bio.indiana.edu/.bin/fbidq.html?sim), [d4](http://flybase.bio.indiana.edu/.bin/fbidq.html?d4), [dan](http://flybase.bio.indiana.edu/.bin/fbidq.html?dan), [gro](http://flybase.bio.indiana.edu/.bin/fbidq.html?gro), [nerfin-1](http://flybase.bio.indiana.edu/.bin/fbidq.html?nerfin-1), [trx](http://flybase.bio.indiana.edu/.bin/fbidq.html?trx), [Rpd3](http://flybase.bio.indiana.edu/.bin/fbidq.html?Rpd3), [dsx](http://flybase.bio.indiana.edu/.bin/fbidq.html?dsx), [MED25](http://flybase.bio.indiana.edu/.bin/fbidq.html?MED25), [Ac13E](http://flybase.bio.indiana.edu/.bin/fbidq.html?Ac13E), [Ssdp](http://flybase.bio.indiana.edu/.bin/fbidq.html?Ssdp), [vfl](http://flybase.bio.indiana.edu/.bin/fbidq.html?vfl), [RpII140](http://flybase.bio.indiana.edu/.bin/fbidq.html?RpII140), [sqz](http://flybase.bio.indiana.edu/.bin/fbidq.html?sqz), [CG32105](http://flybase.bio.indiana.edu/.bin/fbidq.html?CG32105), [Fer3](http://flybase.bio.indiana.edu/.bin/fbidq.html?Fer3), [pygo](http://flybase.bio.indiana.edu/.bin/fbidq.html?pygo), [dalao](http://flybase.bio.indiana.edu/.bin/fbidq.html?dalao), [HLHm7](http://flybase.bio.indiana.edu/.bin/fbidq.html?HLHm7), [z](http://flybase.bio.indiana.edu/.bin/fbidq.html?z),[HLHmgamma](http://flybase.bio.indiana.edu/.bin/fbidq.html?HLHmgamma), [Sox21b](http://flybase.bio.indiana.edu/.bin/fbidq.html?Sox21b), [sr](http://flybase.bio.indiana.edu/.bin/fbidq.html?sr), [Lim1](http://flybase.bio.indiana.edu/.bin/fbidq.html?Lim1), [tld](http://flybase.bio.indiana.edu/.bin/fbidq.html?tld), [danr](http://flybase.bio.indiana.edu/.bin/fbidq.html?danr), [wor](http://flybase.bio.indiana.edu/.bin/fbidq.html?wor), [vvl](http://flybase.bio.indiana.edu/.bin/fbidq.html?vvl), [wda](http://flybase.bio.indiana.edu/.bin/fbidq.html?wda), [sgg](http://flybase.bio.indiana.edu/.bin/fbidq.html?sgg), [MED14](http://flybase.bio.indiana.edu/.bin/fbidq.html?MED14), [gcm2](http://flybase.bio.indiana.edu/.bin/fbidq.html?gcm2), [Eip93F](http://flybase.bio.indiana.edu/.bin/fbidq.html?Eip93F), [gcm](http://flybase.bio.indiana.edu/.bin/fbidq.html?gcm), [seq](http://flybase.bio.indiana.edu/.bin/fbidq.html?seq), [Dip3](http://flybase.bio.indiana.edu/.bin/fbidq.html?Dip3), [Aef1](http://flybase.bio.indiana.edu/.bin/fbidq.html?Aef1), [HDAC4](http://flybase.bio.indiana.edu/.bin/fbidq.html?HDAC4), [can](http://flybase.bio.indiana.edu/.bin/fbidq.html?can), [elk](http://flybase.bio.indiana.edu/.bin/fbidq.html?elk), [phl](http://flybase.bio.indiana.edu/.bin/fbidq.html?phl), [ase](http://flybase.bio.indiana.edu/.bin/fbidq.html?ase), [CG13253](http://flybase.bio.indiana.edu/.bin/fbidq.html?CG13253), [ovo](http://flybase.bio.indiana.edu/.bin/fbidq.html?ovo), [scro](http://flybase.bio.indiana.edu/.bin/fbidq.html?scro), [Dsp1](http://flybase.bio.indiana.edu/.bin/fbidq.html?Dsp1), [CG14216](http://flybase.bio.indiana.edu/.bin/fbidq.html?CG14216), [Pdp1](http://flybase.bio.indiana.edu/.bin/fbidq.html?Pdp1), [Oli](http://flybase.bio.indiana.edu/.bin/fbidq.html?Oli), [Nelf-A](http://flybase.bio.indiana.edu/.bin/fbidq.html?Nelf-A), [GATAd](http://flybase.bio.indiana.edu/.bin/fbidq.html?GATAd), [Poxn](http://flybase.bio.indiana.edu/.bin/fbidq.html?Poxn), [CG15376](http://flybase.bio.indiana.edu/.bin/fbidq.html?CG15376), [Sin3A](http://flybase.bio.indiana.edu/.bin/fbidq.html?Sin3A), [mip120](http://flybase.bio.indiana.edu/.bin/fbidq.html?mip120), [jumu](http://flybase.bio.indiana.edu/.bin/fbidq.html?jumu),[Smox](http://flybase.bio.indiana.edu/.bin/fbidq.html?Smox), [Usp7](http://flybase.bio.indiana.edu/.bin/fbidq.html?Usp7), [ush](http://flybase.bio.indiana.edu/.bin/fbidq.html?ush), [fd59A](http://flybase.bio.indiana.edu/.bin/fbidq.html?fd59A), [Gap1](http://flybase.bio.indiana.edu/.bin/fbidq.html?Gap1), [run](http://flybase.bio.indiana.edu/.bin/fbidq.html?run), [FBX011](http://flybase.bio.indiana.edu/.bin/fbidq.html?FBX011), [brat](http://flybase.bio.indiana.edu/.bin/fbidq.html?brat), [Antp](http://flybase.bio.indiana.edu/.bin/fbidq.html?Antp), [tara](http://flybase.bio.indiana.edu/.bin/fbidq.html?tara), [Alh](http://flybase.bio.indiana.edu/.bin/fbidq.html?Alh), [Sox102F](http://flybase.bio.indiana.edu/.bin/fbidq.html?Sox102F), [egg](http://flybase.bio.indiana.edu/.bin/fbidq.html?egg), [l(3)neo38](http://flybase.bio.indiana.edu/.bin/fbidq.html?l(3)neo38), [koko](http://flybase.bio.indiana.edu/.bin/fbidq.html?koko), [Ada](http://flybase.bio.indiana.edu/.bin/fbidq.html?Ada), [Ptth](http://flybase.bio.indiana.edu/.bin/fbidq.html?Ptth), [Sp1](http://flybase.bio.indiana.edu/.bin/fbidq.html?Sp1), [Ptx1](http://flybase.bio.indiana.edu/.bin/fbidq.html?Ptx1), [ph-p](http://flybase.bio.indiana.edu/.bin/fbidq.html?ph-p), [Ubx](http://flybase.bio.indiana.edu/.bin/fbidq.html?Ubx), [onecut](http://flybase.bio.indiana.edu/.bin/fbidq.html?onecut), [CG7757](http://flybase.bio.indiana.edu/.bin/fbidq.html?CG7757), [chm](http://flybase.bio.indiana.edu/.bin/fbidq.html?chm), [sna](http://flybase.bio.indiana.edu/.bin/fbidq.html?sna), [Wnt5](http://flybase.bio.indiana.edu/.bin/fbidq.html?Wnt5), [Taf1](http://flybase.bio.indiana.edu/.bin/fbidq.html?Taf1), [hbn](http://flybase.bio.indiana.edu/.bin/fbidq.html?hbn), [MBD-like](http://flybase.bio.indiana.edu/.bin/fbidq.html?MBD-like), [tsh](http://flybase.bio.indiana.edu/.bin/fbidq.html?tsh), [esg](http://flybase.bio.indiana.edu/.bin/fbidq.html?esg), [MED16](http://flybase.bio.indiana.edu/.bin/fbidq.html?MED16), [e(y)3](http://flybase.bio.indiana.edu/.bin/fbidq.html?e(y)3), [dac](http://flybase.bio.indiana.edu/.bin/fbidq.html?dac), [CG9007](http://flybase.bio.indiana.edu/.bin/fbidq.html?CG9007), [oc](http://flybase.bio.indiana.edu/.bin/fbidq.html?oc), [spt4](http://flybase.bio.indiana.edu/.bin/fbidq.html?spt4), [Tbh](http://flybase.bio.indiana.edu/.bin/fbidq.html?Tbh),[CG32532](http://flybase.bio.indiana.edu/.bin/fbidq.html?CG32532), [MTA1-like](http://flybase.bio.indiana.edu/.bin/fbidq.html?MTA1-like), [gol](http://flybase.bio.indiana.edu/.bin/fbidq.html?gol), [ey](http://flybase.bio.indiana.edu/.bin/fbidq.html?ey), [mle](http://flybase.bio.indiana.edu/.bin/fbidq.html?mle), [Hr51](http://flybase.bio.indiana.edu/.bin/fbidq.html?Hr51), [Bgb](http://flybase.bio.indiana.edu/.bin/fbidq.html?Bgb), [l(1)sc](http://flybase.bio.indiana.edu/.bin/fbidq.html?l(1)sc), [B-H2](http://flybase.bio.indiana.edu/.bin/fbidq.html?B-H2), [grh](http://flybase.bio.indiana.edu/.bin/fbidq.html?grh), [Vsx1](http://flybase.bio.indiana.edu/.bin/fbidq.html?Vsx1), [wg](http://flybase.bio.indiana.edu/.bin/fbidq.html?wg), [toy](http://flybase.bio.indiana.edu/.bin/fbidq.html?toy), [fkh](http://flybase.bio.indiana.edu/.bin/fbidq.html?fkh), [Lim3](http://flybase.bio.indiana.edu/.bin/fbidq.html?Lim3), [crol](http://flybase.bio.indiana.edu/.bin/fbidq.html?crol), [sd](http://flybase.bio.indiana.edu/.bin/fbidq.html?sd), [retn](http://flybase.bio.indiana.edu/.bin/fbidq.html?retn), [en](http://flybase.bio.indiana.edu/.bin/fbidq.html?en), [fs(1)h](http://flybase.bio.indiana.edu/.bin/fbidq.html?fs(1)h), [Ac3](http://flybase.bio.indiana.edu/.bin/fbidq.html?Ac3), [E(bx)](http://flybase.bio.indiana.edu/.bin/fbidq.html?E(bx)), [rl](http://flybase.bio.indiana.edu/.bin/fbidq.html?rl), [CG11347](http://flybase.bio.indiana.edu/.bin/fbidq.html?CG11347), [tey](http://flybase.bio.indiana.edu/.bin/fbidq.html?tey), [CG11294](http://flybase.bio.indiana.edu/.bin/fbidq.html?CG11294), [hiw](http://flybase.bio.indiana.edu/.bin/fbidq.html?hiw), [cos](http://flybase.bio.indiana.edu/.bin/fbidq.html?cos), [Ct](http://flybase.bio.indiana.edu/.bin/fbidq.html?Ct), [Atf-2](http://flybase.bio.indiana.edu/.bin/fbidq.html?Atf-2) |
| [positive regulation of transcription, DNA-dependent](http://amigo.geneontology.org/cgi-bin/amigo/go.cgi?view=details&query=GO:0045893) | 68 of 1594 genes, 4.3% | 182 of 7634 genes, 2.4% | 5.03e-05 | 0.00% | 0.00 | [acj6](http://flybase.bio.indiana.edu/.bin/fbidq.html?acj6), [wor](http://flybase.bio.indiana.edu/.bin/fbidq.html?wor), [vvl](http://flybase.bio.indiana.edu/.bin/fbidq.html?vvl), [caup](http://flybase.bio.indiana.edu/.bin/fbidq.html?caup), [Camta](http://flybase.bio.indiana.edu/.bin/fbidq.html?Camta), [tll](http://flybase.bio.indiana.edu/.bin/fbidq.html?tll), [ato](http://flybase.bio.indiana.edu/.bin/fbidq.html?ato), [tna](http://flybase.bio.indiana.edu/.bin/fbidq.html?tna), [Eip93F](http://flybase.bio.indiana.edu/.bin/fbidq.html?Eip93F), [JIL-1](http://flybase.bio.indiana.edu/.bin/fbidq.html?JIL-1), [ap](http://flybase.bio.indiana.edu/.bin/fbidq.html?ap), [Iswi](http://flybase.bio.indiana.edu/.bin/fbidq.html?Iswi), [gcm](http://flybase.bio.indiana.edu/.bin/fbidq.html?gcm), [Dip3](http://flybase.bio.indiana.edu/.bin/fbidq.html?Dip3), [ase](http://flybase.bio.indiana.edu/.bin/fbidq.html?ase), [br](http://flybase.bio.indiana.edu/.bin/fbidq.html?br), [ovo](http://flybase.bio.indiana.edu/.bin/fbidq.html?ovo), [Pdp1](http://flybase.bio.indiana.edu/.bin/fbidq.html?Pdp1), [chn](http://flybase.bio.indiana.edu/.bin/fbidq.html?chn), [Hcf](http://flybase.bio.indiana.edu/.bin/fbidq.html?Hcf), [lola](http://flybase.bio.indiana.edu/.bin/fbidq.html?lola), [NC2alpha](http://flybase.bio.indiana.edu/.bin/fbidq.html?NC2alpha), [Taf4](http://flybase.bio.indiana.edu/.bin/fbidq.html?Taf4), [HGTX](http://flybase.bio.indiana.edu/.bin/fbidq.html?HGTX), [fd59A](http://flybase.bio.indiana.edu/.bin/fbidq.html?fd59A), [btd](http://flybase.bio.indiana.edu/.bin/fbidq.html?btd), [run](http://flybase.bio.indiana.edu/.bin/fbidq.html?run), [Antp](http://flybase.bio.indiana.edu/.bin/fbidq.html?Antp), [ATbp](http://flybase.bio.indiana.edu/.bin/fbidq.html?ATbp), [tara](http://flybase.bio.indiana.edu/.bin/fbidq.html?tara), [hth](http://flybase.bio.indiana.edu/.bin/fbidq.html?hth), [vg](http://flybase.bio.indiana.edu/.bin/fbidq.html?vg), [eya](http://flybase.bio.indiana.edu/.bin/fbidq.html?eya), [brm](http://flybase.bio.indiana.edu/.bin/fbidq.html?brm), [onecut](http://flybase.bio.indiana.edu/.bin/fbidq.html?onecut), [sno](http://flybase.bio.indiana.edu/.bin/fbidq.html?sno), [chm](http://flybase.bio.indiana.edu/.bin/fbidq.html?chm), [sna](http://flybase.bio.indiana.edu/.bin/fbidq.html?sna), [ara](http://flybase.bio.indiana.edu/.bin/fbidq.html?ara), [mam](http://flybase.bio.indiana.edu/.bin/fbidq.html?mam), [Taf1](http://flybase.bio.indiana.edu/.bin/fbidq.html?Taf1), [pan](http://flybase.bio.indiana.edu/.bin/fbidq.html?pan), [disco](http://flybase.bio.indiana.edu/.bin/fbidq.html?disco), [esg](http://flybase.bio.indiana.edu/.bin/fbidq.html?esg),[e(y)3](http://flybase.bio.indiana.edu/.bin/fbidq.html?e(y)3), [Brf](http://flybase.bio.indiana.edu/.bin/fbidq.html?Brf), [oc](http://flybase.bio.indiana.edu/.bin/fbidq.html?oc), [tup](http://flybase.bio.indiana.edu/.bin/fbidq.html?tup), [ey](http://flybase.bio.indiana.edu/.bin/fbidq.html?ey), [mle](http://flybase.bio.indiana.edu/.bin/fbidq.html?mle), [l(1)sc](http://flybase.bio.indiana.edu/.bin/fbidq.html?l(1)sc), [Bgb](http://flybase.bio.indiana.edu/.bin/fbidq.html?Bgb), [grh](http://flybase.bio.indiana.edu/.bin/fbidq.html?grh), [toy](http://flybase.bio.indiana.edu/.bin/fbidq.html?toy), [wg](http://flybase.bio.indiana.edu/.bin/fbidq.html?wg), [fkh](http://flybase.bio.indiana.edu/.bin/fbidq.html?fkh), [sim](http://flybase.bio.indiana.edu/.bin/fbidq.html?sim), [sd](http://flybase.bio.indiana.edu/.bin/fbidq.html?sd), [trx](http://flybase.bio.indiana.edu/.bin/fbidq.html?trx), [retn](http://flybase.bio.indiana.edu/.bin/fbidq.html?retn), [dsx](http://flybase.bio.indiana.edu/.bin/fbidq.html?dsx), [vfl](http://flybase.bio.indiana.edu/.bin/fbidq.html?vfl), [CG11347](http://flybase.bio.indiana.edu/.bin/fbidq.html?CG11347), [dalao](http://flybase.bio.indiana.edu/.bin/fbidq.html?dalao), [pygo](http://flybase.bio.indiana.edu/.bin/fbidq.html?pygo), [z](http://flybase.bio.indiana.edu/.bin/fbidq.html?z), [Lim1](http://flybase.bio.indiana.edu/.bin/fbidq.html?Lim1), [Atf-2](http://flybase.bio.indiana.edu/.bin/fbidq.html?Atf-2) |
| [nervous system development](http://amigo.geneontology.org/cgi-bin/amigo/go.cgi?view=details&query=GO:0007399) | 317 of 1594 genes, 19.9% | 1234 of 7634 genes, 16.2% | 5.92e-05 | 0.00% | 0.00 | [fz2](http://flybase.bio.indiana.edu/.bin/fbidq.html?fz2), [Sh](http://flybase.bio.indiana.edu/.bin/fbidq.html?Sh), [numb](http://flybase.bio.indiana.edu/.bin/fbidq.html?numb), [dock](http://flybase.bio.indiana.edu/.bin/fbidq.html?dock), [CG5808](http://flybase.bio.indiana.edu/.bin/fbidq.html?CG5808), [CG9004](http://flybase.bio.indiana.edu/.bin/fbidq.html?CG9004), [CG42256](http://flybase.bio.indiana.edu/.bin/fbidq.html?CG42256), [CG3703](http://flybase.bio.indiana.edu/.bin/fbidq.html?CG3703), [stj](http://flybase.bio.indiana.edu/.bin/fbidq.html?stj), [ap](http://flybase.bio.indiana.edu/.bin/fbidq.html?ap), [Nf1](http://flybase.bio.indiana.edu/.bin/fbidq.html?Nf1), [Drep-3](http://flybase.bio.indiana.edu/.bin/fbidq.html?Drep-3), [sol](http://flybase.bio.indiana.edu/.bin/fbidq.html?sol), [os](http://flybase.bio.indiana.edu/.bin/fbidq.html?os), [tok](http://flybase.bio.indiana.edu/.bin/fbidq.html?tok), [Ptp99A](http://flybase.bio.indiana.edu/.bin/fbidq.html?Ptp99A), [CG31961](http://flybase.bio.indiana.edu/.bin/fbidq.html?CG31961), [NetB](http://flybase.bio.indiana.edu/.bin/fbidq.html?NetB), [Tango10](http://flybase.bio.indiana.edu/.bin/fbidq.html?Tango10), [Oseg4](http://flybase.bio.indiana.edu/.bin/fbidq.html?Oseg4), [cid](http://flybase.bio.indiana.edu/.bin/fbidq.html?cid), [CG32137](http://flybase.bio.indiana.edu/.bin/fbidq.html?CG32137), [lute](http://flybase.bio.indiana.edu/.bin/fbidq.html?lute), [Ssrp](http://flybase.bio.indiana.edu/.bin/fbidq.html?Ssrp), [Galpha49B](http://flybase.bio.indiana.edu/.bin/fbidq.html?Galpha49B), [Snoo](http://flybase.bio.indiana.edu/.bin/fbidq.html?Snoo), [Lar](http://flybase.bio.indiana.edu/.bin/fbidq.html?Lar), [scrib](http://flybase.bio.indiana.edu/.bin/fbidq.html?scrib), [HGTX](http://flybase.bio.indiana.edu/.bin/fbidq.html?HGTX), [not](http://flybase.bio.indiana.edu/.bin/fbidq.html?not), [rut](http://flybase.bio.indiana.edu/.bin/fbidq.html?rut), [nvy](http://flybase.bio.indiana.edu/.bin/fbidq.html?nvy), [nub](http://flybase.bio.indiana.edu/.bin/fbidq.html?nub), [nab](http://flybase.bio.indiana.edu/.bin/fbidq.html?nab), [HLHmdelta](http://flybase.bio.indiana.edu/.bin/fbidq.html?HLHmdelta), [B-H1](http://flybase.bio.indiana.edu/.bin/fbidq.html?B-H1), [pim](http://flybase.bio.indiana.edu/.bin/fbidq.html?pim), [alph](http://flybase.bio.indiana.edu/.bin/fbidq.html?alph), [TBPH](http://flybase.bio.indiana.edu/.bin/fbidq.html?TBPH), [Fer2](http://flybase.bio.indiana.edu/.bin/fbidq.html?Fer2), [CG34400](http://flybase.bio.indiana.edu/.bin/fbidq.html?CG34400), [Ulp1](http://flybase.bio.indiana.edu/.bin/fbidq.html?Ulp1), [ena](http://flybase.bio.indiana.edu/.bin/fbidq.html?ena), [dsh](http://flybase.bio.indiana.edu/.bin/fbidq.html?dsh), [hth](http://flybase.bio.indiana.edu/.bin/fbidq.html?hth), [pzg](http://flybase.bio.indiana.edu/.bin/fbidq.html?pzg), [Nf-YC](http://flybase.bio.indiana.edu/.bin/fbidq.html?Nf-YC), [14-3-3zeta](http://flybase.bio.indiana.edu/.bin/fbidq.html?14-3-3zeta), [lea](http://flybase.bio.indiana.edu/.bin/fbidq.html?lea), [qkr58E-1](http://flybase.bio.indiana.edu/.bin/fbidq.html?qkr58E-1), [plexB](http://flybase.bio.indiana.edu/.bin/fbidq.html?plexB), [loqs](http://flybase.bio.indiana.edu/.bin/fbidq.html?loqs), [Klp64D](http://flybase.bio.indiana.edu/.bin/fbidq.html?Klp64D), [DAAM](http://flybase.bio.indiana.edu/.bin/fbidq.html?DAAM), [neur](http://flybase.bio.indiana.edu/.bin/fbidq.html?neur), [CycE](http://flybase.bio.indiana.edu/.bin/fbidq.html?CycE), [Brf](http://flybase.bio.indiana.edu/.bin/fbidq.html?Brf), [nmo](http://flybase.bio.indiana.edu/.bin/fbidq.html?nmo), [ed](http://flybase.bio.indiana.edu/.bin/fbidq.html?ed), [mr](http://flybase.bio.indiana.edu/.bin/fbidq.html?mr), [CG11317](http://flybase.bio.indiana.edu/.bin/fbidq.html?CG11317), [daw](http://flybase.bio.indiana.edu/.bin/fbidq.html?daw), [Rx](http://flybase.bio.indiana.edu/.bin/fbidq.html?Rx), [salr](http://flybase.bio.indiana.edu/.bin/fbidq.html?salr), [synaptogyrin](http://flybase.bio.indiana.edu/.bin/fbidq.html?synaptogyrin), [Apc](http://flybase.bio.indiana.edu/.bin/fbidq.html?Apc), [sim](http://flybase.bio.indiana.edu/.bin/fbidq.html?sim), [d4](http://flybase.bio.indiana.edu/.bin/fbidq.html?d4), [dan](http://flybase.bio.indiana.edu/.bin/fbidq.html?dan), [gro](http://flybase.bio.indiana.edu/.bin/fbidq.html?gro), [wnd](http://flybase.bio.indiana.edu/.bin/fbidq.html?wnd), [trx](http://flybase.bio.indiana.edu/.bin/fbidq.html?trx), [Rpd3](http://flybase.bio.indiana.edu/.bin/fbidq.html?Rpd3), [SMC2](http://flybase.bio.indiana.edu/.bin/fbidq.html?SMC2),[MED25](http://flybase.bio.indiana.edu/.bin/fbidq.html?MED25), [unc-5](http://flybase.bio.indiana.edu/.bin/fbidq.html?unc-5), [cdi](http://flybase.bio.indiana.edu/.bin/fbidq.html?cdi), [CG10107](http://flybase.bio.indiana.edu/.bin/fbidq.html?CG10107), [barr](http://flybase.bio.indiana.edu/.bin/fbidq.html?barr), [pygo](http://flybase.bio.indiana.edu/.bin/fbidq.html?pygo), [HLHm7](http://flybase.bio.indiana.edu/.bin/fbidq.html?HLHm7), [HLHmgamma](http://flybase.bio.indiana.edu/.bin/fbidq.html?HLHmgamma), [jar](http://flybase.bio.indiana.edu/.bin/fbidq.html?jar), [Ror](http://flybase.bio.indiana.edu/.bin/fbidq.html?Ror), [wor](http://flybase.bio.indiana.edu/.bin/fbidq.html?wor), [Atg1](http://flybase.bio.indiana.edu/.bin/fbidq.html?Atg1), [Pten](http://flybase.bio.indiana.edu/.bin/fbidq.html?Pten), [Fcp1](http://flybase.bio.indiana.edu/.bin/fbidq.html?Fcp1), [mira](http://flybase.bio.indiana.edu/.bin/fbidq.html?mira), [Ptp69D](http://flybase.bio.indiana.edu/.bin/fbidq.html?Ptp69D), [hts](http://flybase.bio.indiana.edu/.bin/fbidq.html?hts), [pUf68](http://flybase.bio.indiana.edu/.bin/fbidq.html?pUf68), [seq](http://flybase.bio.indiana.edu/.bin/fbidq.html?seq), [Dip3](http://flybase.bio.indiana.edu/.bin/fbidq.html?Dip3), [Drep-2](http://flybase.bio.indiana.edu/.bin/fbidq.html?Drep-2), [oa2](http://flybase.bio.indiana.edu/.bin/fbidq.html?oa2), [RpS11](http://flybase.bio.indiana.edu/.bin/fbidq.html?RpS11), [phl](http://flybase.bio.indiana.edu/.bin/fbidq.html?phl), [CG1671](http://flybase.bio.indiana.edu/.bin/fbidq.html?CG1671), [Cbl](http://flybase.bio.indiana.edu/.bin/fbidq.html?Cbl), [thr](http://flybase.bio.indiana.edu/.bin/fbidq.html?thr), [CG4893](http://flybase.bio.indiana.edu/.bin/fbidq.html?CG4893), [CG9098](http://flybase.bio.indiana.edu/.bin/fbidq.html?CG9098), [Alk](http://flybase.bio.indiana.edu/.bin/fbidq.html?Alk), [esn](http://flybase.bio.indiana.edu/.bin/fbidq.html?esn), [Vang](http://flybase.bio.indiana.edu/.bin/fbidq.html?Vang), [Sin3A](http://flybase.bio.indiana.edu/.bin/fbidq.html?Sin3A), [pot](http://flybase.bio.indiana.edu/.bin/fbidq.html?pot), [cpx](http://flybase.bio.indiana.edu/.bin/fbidq.html?cpx), [jumu](http://flybase.bio.indiana.edu/.bin/fbidq.html?jumu),[Smox](http://flybase.bio.indiana.edu/.bin/fbidq.html?Smox), [Su(var)2-HP2](http://flybase.bio.indiana.edu/.bin/fbidq.html?Su(var)2-HP2), [CG31475](http://flybase.bio.indiana.edu/.bin/fbidq.html?CG31475), [fd59A](http://flybase.bio.indiana.edu/.bin/fbidq.html?fd59A), [beat-Ic](http://flybase.bio.indiana.edu/.bin/fbidq.html?beat-Ic), [brat](http://flybase.bio.indiana.edu/.bin/fbidq.html?brat), [Antp](http://flybase.bio.indiana.edu/.bin/fbidq.html?Antp), [glec](http://flybase.bio.indiana.edu/.bin/fbidq.html?glec), [futsch](http://flybase.bio.indiana.edu/.bin/fbidq.html?futsch), [Ptx1](http://flybase.bio.indiana.edu/.bin/fbidq.html?Ptx1), [gukh](http://flybase.bio.indiana.edu/.bin/fbidq.html?gukh), [chm](http://flybase.bio.indiana.edu/.bin/fbidq.html?chm), [Rim](http://flybase.bio.indiana.edu/.bin/fbidq.html?Rim), [beat-IIa](http://flybase.bio.indiana.edu/.bin/fbidq.html?beat-IIa), [Gpo-1](http://flybase.bio.indiana.edu/.bin/fbidq.html?Gpo-1), [sm](http://flybase.bio.indiana.edu/.bin/fbidq.html?sm), [Syn2](http://flybase.bio.indiana.edu/.bin/fbidq.html?Syn2), [CG42574](http://flybase.bio.indiana.edu/.bin/fbidq.html?CG42574), [eas](http://flybase.bio.indiana.edu/.bin/fbidq.html?eas), [dac](http://flybase.bio.indiana.edu/.bin/fbidq.html?dac), [Nrx-1](http://flybase.bio.indiana.edu/.bin/fbidq.html?Nrx-1), [Cdk5](http://flybase.bio.indiana.edu/.bin/fbidq.html?Cdk5), [oc](http://flybase.bio.indiana.edu/.bin/fbidq.html?oc), [CG4641](http://flybase.bio.indiana.edu/.bin/fbidq.html?CG4641), [CG3964](http://flybase.bio.indiana.edu/.bin/fbidq.html?CG3964), [ey](http://flybase.bio.indiana.edu/.bin/fbidq.html?ey), [baz](http://flybase.bio.indiana.edu/.bin/fbidq.html?baz), [rst](http://flybase.bio.indiana.edu/.bin/fbidq.html?rst), [l(1)sc](http://flybase.bio.indiana.edu/.bin/fbidq.html?l(1)sc), [comm](http://flybase.bio.indiana.edu/.bin/fbidq.html?comm), [grh](http://flybase.bio.indiana.edu/.bin/fbidq.html?grh), [Cdk5alpha](http://flybase.bio.indiana.edu/.bin/fbidq.html?Cdk5alpha), [toy](http://flybase.bio.indiana.edu/.bin/fbidq.html?toy), [Lim3](http://flybase.bio.indiana.edu/.bin/fbidq.html?Lim3), [sd](http://flybase.bio.indiana.edu/.bin/fbidq.html?sd),[Aplip1](http://flybase.bio.indiana.edu/.bin/fbidq.html?Aplip1), [E(Pc)](http://flybase.bio.indiana.edu/.bin/fbidq.html?E(Pc)), [CG1239](http://flybase.bio.indiana.edu/.bin/fbidq.html?CG1239), [retn](http://flybase.bio.indiana.edu/.bin/fbidq.html?retn), [rl](http://flybase.bio.indiana.edu/.bin/fbidq.html?rl), [Nup153](http://flybase.bio.indiana.edu/.bin/fbidq.html?Nup153), [tey](http://flybase.bio.indiana.edu/.bin/fbidq.html?tey), [yrt](http://flybase.bio.indiana.edu/.bin/fbidq.html?yrt), [CG33960](http://flybase.bio.indiana.edu/.bin/fbidq.html?CG33960), [hiw](http://flybase.bio.indiana.edu/.bin/fbidq.html?hiw), [CG15765](http://flybase.bio.indiana.edu/.bin/fbidq.html?CG15765), [Pabp2](http://flybase.bio.indiana.edu/.bin/fbidq.html?Pabp2), [acj6](http://flybase.bio.indiana.edu/.bin/fbidq.html?acj6), [jing](http://flybase.bio.indiana.edu/.bin/fbidq.html?jing), [Cbp80](http://flybase.bio.indiana.edu/.bin/fbidq.html?Cbp80), [tll](http://flybase.bio.indiana.edu/.bin/fbidq.html?tll), [vimar](http://flybase.bio.indiana.edu/.bin/fbidq.html?vimar), [ato](http://flybase.bio.indiana.edu/.bin/fbidq.html?ato), [Iswi](http://flybase.bio.indiana.edu/.bin/fbidq.html?Iswi), [Dg](http://flybase.bio.indiana.edu/.bin/fbidq.html?Dg), [Trim9](http://flybase.bio.indiana.edu/.bin/fbidq.html?Trim9), [CG5742](http://flybase.bio.indiana.edu/.bin/fbidq.html?CG5742), [CG34127](http://flybase.bio.indiana.edu/.bin/fbidq.html?CG34127), [br](http://flybase.bio.indiana.edu/.bin/fbidq.html?br), [CG7154](http://flybase.bio.indiana.edu/.bin/fbidq.html?CG7154), [robo3](http://flybase.bio.indiana.edu/.bin/fbidq.html?robo3), [chn](http://flybase.bio.indiana.edu/.bin/fbidq.html?chn), [lola](http://flybase.bio.indiana.edu/.bin/fbidq.html?lola), [unc-104](http://flybase.bio.indiana.edu/.bin/fbidq.html?unc-104), [chif](http://flybase.bio.indiana.edu/.bin/fbidq.html?chif), [Pcl](http://flybase.bio.indiana.edu/.bin/fbidq.html?Pcl), [p130CAS](http://flybase.bio.indiana.edu/.bin/fbidq.html?p130CAS), [CG7033](http://flybase.bio.indiana.edu/.bin/fbidq.html?CG7033), [Spc105R](http://flybase.bio.indiana.edu/.bin/fbidq.html?Spc105R),[CG31158](http://flybase.bio.indiana.edu/.bin/fbidq.html?CG31158), [Appl](http://flybase.bio.indiana.edu/.bin/fbidq.html?Appl), [metro](http://flybase.bio.indiana.edu/.bin/fbidq.html?metro), [CG13550](http://flybase.bio.indiana.edu/.bin/fbidq.html?CG13550), [ewg](http://flybase.bio.indiana.edu/.bin/fbidq.html?ewg), [CG30007](http://flybase.bio.indiana.edu/.bin/fbidq.html?CG30007), [erm](http://flybase.bio.indiana.edu/.bin/fbidq.html?erm), [Nrk](http://flybase.bio.indiana.edu/.bin/fbidq.html?Nrk), [dmt](http://flybase.bio.indiana.edu/.bin/fbidq.html?dmt), [CG4328](http://flybase.bio.indiana.edu/.bin/fbidq.html?CG4328), [CG10249](http://flybase.bio.indiana.edu/.bin/fbidq.html?CG10249), [Taf4](http://flybase.bio.indiana.edu/.bin/fbidq.html?Taf4), [l(2)NC136](http://flybase.bio.indiana.edu/.bin/fbidq.html?l(2)NC136), [Nrg](http://flybase.bio.indiana.edu/.bin/fbidq.html?Nrg), [gogo](http://flybase.bio.indiana.edu/.bin/fbidq.html?gogo), [insc](http://flybase.bio.indiana.edu/.bin/fbidq.html?insc), [jeb](http://flybase.bio.indiana.edu/.bin/fbidq.html?jeb), [GluClalpha](http://flybase.bio.indiana.edu/.bin/fbidq.html?GluClalpha), [Actbeta](http://flybase.bio.indiana.edu/.bin/fbidq.html?Actbeta), [stau](http://flybase.bio.indiana.edu/.bin/fbidq.html?stau), [beat-Ib](http://flybase.bio.indiana.edu/.bin/fbidq.html?beat-Ib), [Sema-1a](http://flybase.bio.indiana.edu/.bin/fbidq.html?Sema-1a), [Hs3st-B](http://flybase.bio.indiana.edu/.bin/fbidq.html?Hs3st-B), [otk](http://flybase.bio.indiana.edu/.bin/fbidq.html?otk), [Sos](http://flybase.bio.indiana.edu/.bin/fbidq.html?Sos), [phyl](http://flybase.bio.indiana.edu/.bin/fbidq.html?phyl), [eya](http://flybase.bio.indiana.edu/.bin/fbidq.html?eya), [Klp61F](http://flybase.bio.indiana.edu/.bin/fbidq.html?Klp61F), [Fas2](http://flybase.bio.indiana.edu/.bin/fbidq.html?Fas2), [fz](http://flybase.bio.indiana.edu/.bin/fbidq.html?fz), [robo](http://flybase.bio.indiana.edu/.bin/fbidq.html?robo), [bsk](http://flybase.bio.indiana.edu/.bin/fbidq.html?bsk), [brm](http://flybase.bio.indiana.edu/.bin/fbidq.html?brm), [beat-Ia](http://flybase.bio.indiana.edu/.bin/fbidq.html?beat-Ia),[mtg](http://flybase.bio.indiana.edu/.bin/fbidq.html?mtg), [kat-60L1](http://flybase.bio.indiana.edu/.bin/fbidq.html?kat-60L1), [hig](http://flybase.bio.indiana.edu/.bin/fbidq.html?hig), [CG12004](http://flybase.bio.indiana.edu/.bin/fbidq.html?CG12004), [RhoGAPp190](http://flybase.bio.indiana.edu/.bin/fbidq.html?RhoGAPp190), [pHCl](http://flybase.bio.indiana.edu/.bin/fbidq.html?pHCl), [l(2)k16918](http://flybase.bio.indiana.edu/.bin/fbidq.html?l(2)k16918), [mam](http://flybase.bio.indiana.edu/.bin/fbidq.html?mam), [sws](http://flybase.bio.indiana.edu/.bin/fbidq.html?sws), [disco](http://flybase.bio.indiana.edu/.bin/fbidq.html?disco), [Dll](http://flybase.bio.indiana.edu/.bin/fbidq.html?Dll), [grn](http://flybase.bio.indiana.edu/.bin/fbidq.html?grn), [HLHm5](http://flybase.bio.indiana.edu/.bin/fbidq.html?HLHm5), [mew](http://flybase.bio.indiana.edu/.bin/fbidq.html?mew), [Lis-1](http://flybase.bio.indiana.edu/.bin/fbidq.html?Lis-1), [didum](http://flybase.bio.indiana.edu/.bin/fbidq.html?didum), [ems](http://flybase.bio.indiana.edu/.bin/fbidq.html?ems), [eys](http://flybase.bio.indiana.edu/.bin/fbidq.html?eys), [Mob2](http://flybase.bio.indiana.edu/.bin/fbidq.html?Mob2), [pros](http://flybase.bio.indiana.edu/.bin/fbidq.html?pros), [tup](http://flybase.bio.indiana.edu/.bin/fbidq.html?tup), [pdm2](http://flybase.bio.indiana.edu/.bin/fbidq.html?pdm2), [trio](http://flybase.bio.indiana.edu/.bin/fbidq.html?trio), [CG1463](http://flybase.bio.indiana.edu/.bin/fbidq.html?CG1463), [Gad1](http://flybase.bio.indiana.edu/.bin/fbidq.html?Gad1), [Wnt4](http://flybase.bio.indiana.edu/.bin/fbidq.html?Wnt4), [rictor](http://flybase.bio.indiana.edu/.bin/fbidq.html?rictor), [nerfin-1](http://flybase.bio.indiana.edu/.bin/fbidq.html?nerfin-1), [bchs](http://flybase.bio.indiana.edu/.bin/fbidq.html?bchs), [RpS5b](http://flybase.bio.indiana.edu/.bin/fbidq.html?RpS5b), [polo](http://flybase.bio.indiana.edu/.bin/fbidq.html?polo), [dsx](http://flybase.bio.indiana.edu/.bin/fbidq.html?dsx), [chinmo](http://flybase.bio.indiana.edu/.bin/fbidq.html?chinmo), [Syn1](http://flybase.bio.indiana.edu/.bin/fbidq.html?Syn1),[Ssdp](http://flybase.bio.indiana.edu/.bin/fbidq.html?Ssdp), [klu](http://flybase.bio.indiana.edu/.bin/fbidq.html?klu), [sqz](http://flybase.bio.indiana.edu/.bin/fbidq.html?sqz), [fend](http://flybase.bio.indiana.edu/.bin/fbidq.html?fend), [Atet](http://flybase.bio.indiana.edu/.bin/fbidq.html?Atet), [sr](http://flybase.bio.indiana.edu/.bin/fbidq.html?sr), [nkd](http://flybase.bio.indiana.edu/.bin/fbidq.html?nkd), [danr](http://flybase.bio.indiana.edu/.bin/fbidq.html?danr), [vvl](http://flybase.bio.indiana.edu/.bin/fbidq.html?vvl), [Unc-76](http://flybase.bio.indiana.edu/.bin/fbidq.html?Unc-76), [rg](http://flybase.bio.indiana.edu/.bin/fbidq.html?rg), [betaInt-nu](http://flybase.bio.indiana.edu/.bin/fbidq.html?betaInt-nu), [sgg](http://flybase.bio.indiana.edu/.bin/fbidq.html?sgg), [mnb](http://flybase.bio.indiana.edu/.bin/fbidq.html?mnb), [gcm2](http://flybase.bio.indiana.edu/.bin/fbidq.html?gcm2), [mp](http://flybase.bio.indiana.edu/.bin/fbidq.html?mp), [gcm](http://flybase.bio.indiana.edu/.bin/fbidq.html?gcm), [CG5890](http://flybase.bio.indiana.edu/.bin/fbidq.html?CG5890), [SP2353](http://flybase.bio.indiana.edu/.bin/fbidq.html?SP2353), [Ccn](http://flybase.bio.indiana.edu/.bin/fbidq.html?Ccn), [ase](http://flybase.bio.indiana.edu/.bin/fbidq.html?ase), [CG32685](http://flybase.bio.indiana.edu/.bin/fbidq.html?CG32685), [elav](http://flybase.bio.indiana.edu/.bin/fbidq.html?elav), [Poxn](http://flybase.bio.indiana.edu/.bin/fbidq.html?Poxn), [Incenp](http://flybase.bio.indiana.edu/.bin/fbidq.html?Incenp), [Rat1](http://flybase.bio.indiana.edu/.bin/fbidq.html?Rat1), [CG18273](http://flybase.bio.indiana.edu/.bin/fbidq.html?CG18273), [Fak56D](http://flybase.bio.indiana.edu/.bin/fbidq.html?Fak56D), [Gap1](http://flybase.bio.indiana.edu/.bin/fbidq.html?Gap1), [run](http://flybase.bio.indiana.edu/.bin/fbidq.html?run), [ko](http://flybase.bio.indiana.edu/.bin/fbidq.html?ko), [CG16778](http://flybase.bio.indiana.edu/.bin/fbidq.html?CG16778), [Eip71CD](http://flybase.bio.indiana.edu/.bin/fbidq.html?Eip71CD), [stan](http://flybase.bio.indiana.edu/.bin/fbidq.html?stan), [CG12424](http://flybase.bio.indiana.edu/.bin/fbidq.html?CG12424), [tutl](http://flybase.bio.indiana.edu/.bin/fbidq.html?tutl), [ph-p](http://flybase.bio.indiana.edu/.bin/fbidq.html?ph-p), [GlcAT-P](http://flybase.bio.indiana.edu/.bin/fbidq.html?GlcAT-P), [sna](http://flybase.bio.indiana.edu/.bin/fbidq.html?sna), [Wnt5](http://flybase.bio.indiana.edu/.bin/fbidq.html?Wnt5), [Bre1](http://flybase.bio.indiana.edu/.bin/fbidq.html?Bre1), [Taf1](http://flybase.bio.indiana.edu/.bin/fbidq.html?Taf1), [SF2](http://flybase.bio.indiana.edu/.bin/fbidq.html?SF2), [Ggamma1](http://flybase.bio.indiana.edu/.bin/fbidq.html?Ggamma1), [CG7597](http://flybase.bio.indiana.edu/.bin/fbidq.html?CG7597), [CG5466](http://flybase.bio.indiana.edu/.bin/fbidq.html?CG5466), [esg](http://flybase.bio.indiana.edu/.bin/fbidq.html?esg), [sif](http://flybase.bio.indiana.edu/.bin/fbidq.html?sif), [dom](http://flybase.bio.indiana.edu/.bin/fbidq.html?dom), [Aac11](http://flybase.bio.indiana.edu/.bin/fbidq.html?Aac11), [ssh](http://flybase.bio.indiana.edu/.bin/fbidq.html?ssh), [klar](http://flybase.bio.indiana.edu/.bin/fbidq.html?klar), [PIP5K59B](http://flybase.bio.indiana.edu/.bin/fbidq.html?PIP5K59B), [mle](http://flybase.bio.indiana.edu/.bin/fbidq.html?mle), [Hr51](http://flybase.bio.indiana.edu/.bin/fbidq.html?Hr51), [CG11155](http://flybase.bio.indiana.edu/.bin/fbidq.html?CG11155), [siz](http://flybase.bio.indiana.edu/.bin/fbidq.html?siz), [B-H2](http://flybase.bio.indiana.edu/.bin/fbidq.html?B-H2), [wg](http://flybase.bio.indiana.edu/.bin/fbidq.html?wg), [CadN](http://flybase.bio.indiana.edu/.bin/fbidq.html?CadN), [CG34139](http://flybase.bio.indiana.edu/.bin/fbidq.html?CG34139), [Mfap1](http://flybase.bio.indiana.edu/.bin/fbidq.html?Mfap1), [RpL28](http://flybase.bio.indiana.edu/.bin/fbidq.html?RpL28), [E(bx)](http://flybase.bio.indiana.edu/.bin/fbidq.html?E(bx)), [en](http://flybase.bio.indiana.edu/.bin/fbidq.html?en), [klg](http://flybase.bio.indiana.edu/.bin/fbidq.html?klg), [Snap](http://flybase.bio.indiana.edu/.bin/fbidq.html?Snap), [trn](http://flybase.bio.indiana.edu/.bin/fbidq.html?trn), [Rnp4F](http://flybase.bio.indiana.edu/.bin/fbidq.html?Rnp4F), [Ct](http://flybase.bio.indiana.edu/.bin/fbidq.html?Ct) |
| [heterocycle biosynthetic process](http://amigo.geneontology.org/cgi-bin/amigo/go.cgi?view=details&query=GO:0018130) | 219 of 1594 genes, 13.7% | 799 of 7634 genes, 10.5% | 6.50e-05 | 0.00% | 0.00 | [fz2](http://flybase.bio.indiana.edu/.bin/fbidq.html?fz2), [acj6](http://flybase.bio.indiana.edu/.bin/fbidq.html?acj6), [skd](http://flybase.bio.indiana.edu/.bin/fbidq.html?skd), [jing](http://flybase.bio.indiana.edu/.bin/fbidq.html?jing), [caup](http://flybase.bio.indiana.edu/.bin/fbidq.html?caup), [Tgt](http://flybase.bio.indiana.edu/.bin/fbidq.html?Tgt), [tll](http://flybase.bio.indiana.edu/.bin/fbidq.html?tll), [Camta](http://flybase.bio.indiana.edu/.bin/fbidq.html?Camta), [ato](http://flybase.bio.indiana.edu/.bin/fbidq.html?ato), [Ets65A](http://flybase.bio.indiana.edu/.bin/fbidq.html?Ets65A), [tna](http://flybase.bio.indiana.edu/.bin/fbidq.html?tna), [Prat2](http://flybase.bio.indiana.edu/.bin/fbidq.html?Prat2), [JIL-1](http://flybase.bio.indiana.edu/.bin/fbidq.html?JIL-1), [Spt6](http://flybase.bio.indiana.edu/.bin/fbidq.html?Spt6), [Iswi](http://flybase.bio.indiana.edu/.bin/fbidq.html?Iswi), [rn](http://flybase.bio.indiana.edu/.bin/fbidq.html?rn), [ap](http://flybase.bio.indiana.edu/.bin/fbidq.html?ap), [Nf1](http://flybase.bio.indiana.edu/.bin/fbidq.html?Nf1), [br](http://flybase.bio.indiana.edu/.bin/fbidq.html?br), [Gycalpha99B](http://flybase.bio.indiana.edu/.bin/fbidq.html?Gycalpha99B), [ksr](http://flybase.bio.indiana.edu/.bin/fbidq.html?ksr), [PNUTS](http://flybase.bio.indiana.edu/.bin/fbidq.html?PNUTS), [Gyc-89Da](http://flybase.bio.indiana.edu/.bin/fbidq.html?Gyc-89Da), [bi](http://flybase.bio.indiana.edu/.bin/fbidq.html?bi), [chn](http://flybase.bio.indiana.edu/.bin/fbidq.html?chn), [Gsc](http://flybase.bio.indiana.edu/.bin/fbidq.html?Gsc), [Hcf](http://flybase.bio.indiana.edu/.bin/fbidq.html?Hcf), [lola](http://flybase.bio.indiana.edu/.bin/fbidq.html?lola), [HLH4C](http://flybase.bio.indiana.edu/.bin/fbidq.html?HLH4C), [Pcl](http://flybase.bio.indiana.edu/.bin/fbidq.html?Pcl), [chif](http://flybase.bio.indiana.edu/.bin/fbidq.html?chif), [simj](http://flybase.bio.indiana.edu/.bin/fbidq.html?simj), [Awh](http://flybase.bio.indiana.edu/.bin/fbidq.html?Awh), [tap](http://flybase.bio.indiana.edu/.bin/fbidq.html?tap), [CG13550](http://flybase.bio.indiana.edu/.bin/fbidq.html?CG13550), [NC2alpha](http://flybase.bio.indiana.edu/.bin/fbidq.html?NC2alpha), [PHDP](http://flybase.bio.indiana.edu/.bin/fbidq.html?PHDP), [bab1](http://flybase.bio.indiana.edu/.bin/fbidq.html?bab1), [Taf12L](http://flybase.bio.indiana.edu/.bin/fbidq.html?Taf12L),[CG4328](http://flybase.bio.indiana.edu/.bin/fbidq.html?CG4328), [Snoo](http://flybase.bio.indiana.edu/.bin/fbidq.html?Snoo), [l(2)NC136](http://flybase.bio.indiana.edu/.bin/fbidq.html?l(2)NC136), [Taf4](http://flybase.bio.indiana.edu/.bin/fbidq.html?Taf4), [Su(var)3-3](http://flybase.bio.indiana.edu/.bin/fbidq.html?Su(var)3-3), [HGTX](http://flybase.bio.indiana.edu/.bin/fbidq.html?HGTX), [not](http://flybase.bio.indiana.edu/.bin/fbidq.html?not), [rut](http://flybase.bio.indiana.edu/.bin/fbidq.html?rut), [nvy](http://flybase.bio.indiana.edu/.bin/fbidq.html?nvy), [nub](http://flybase.bio.indiana.edu/.bin/fbidq.html?nub), [nab](http://flybase.bio.indiana.edu/.bin/fbidq.html?nab), [Ddc](http://flybase.bio.indiana.edu/.bin/fbidq.html?Ddc), [btd](http://flybase.bio.indiana.edu/.bin/fbidq.html?btd), [fd68A](http://flybase.bio.indiana.edu/.bin/fbidq.html?fd68A), [HLHmdelta](http://flybase.bio.indiana.edu/.bin/fbidq.html?HLHmdelta), [B-H1](http://flybase.bio.indiana.edu/.bin/fbidq.html?B-H1), [Nipped-A](http://flybase.bio.indiana.edu/.bin/fbidq.html?Nipped-A), [ATbp](http://flybase.bio.indiana.edu/.bin/fbidq.html?ATbp), [Sos](http://flybase.bio.indiana.edu/.bin/fbidq.html?Sos), [Fer2](http://flybase.bio.indiana.edu/.bin/fbidq.html?Fer2), [fd102C](http://flybase.bio.indiana.edu/.bin/fbidq.html?fd102C), [vg](http://flybase.bio.indiana.edu/.bin/fbidq.html?vg), [hth](http://flybase.bio.indiana.edu/.bin/fbidq.html?hth), [Nf-YC](http://flybase.bio.indiana.edu/.bin/fbidq.html?Nf-YC), [14-3-3zeta](http://flybase.bio.indiana.edu/.bin/fbidq.html?14-3-3zeta), [eya](http://flybase.bio.indiana.edu/.bin/fbidq.html?eya), [fz](http://flybase.bio.indiana.edu/.bin/fbidq.html?fz), [brm](http://flybase.bio.indiana.edu/.bin/fbidq.html?brm), [Vsx2](http://flybase.bio.indiana.edu/.bin/fbidq.html?Vsx2), [Hey](http://flybase.bio.indiana.edu/.bin/fbidq.html?Hey), [sno](http://flybase.bio.indiana.edu/.bin/fbidq.html?sno), [Sfmbt](http://flybase.bio.indiana.edu/.bin/fbidq.html?Sfmbt), [ara](http://flybase.bio.indiana.edu/.bin/fbidq.html?ara), [mam](http://flybase.bio.indiana.edu/.bin/fbidq.html?mam), [pan](http://flybase.bio.indiana.edu/.bin/fbidq.html?pan), [Dll](http://flybase.bio.indiana.edu/.bin/fbidq.html?Dll), [disco](http://flybase.bio.indiana.edu/.bin/fbidq.html?disco), [grn](http://flybase.bio.indiana.edu/.bin/fbidq.html?grn),[HLHm5](http://flybase.bio.indiana.edu/.bin/fbidq.html?HLHm5), [D12](http://flybase.bio.indiana.edu/.bin/fbidq.html?D12), [Lis-1](http://flybase.bio.indiana.edu/.bin/fbidq.html?Lis-1), [CycE](http://flybase.bio.indiana.edu/.bin/fbidq.html?CycE), [Brf](http://flybase.bio.indiana.edu/.bin/fbidq.html?Brf), [ems](http://flybase.bio.indiana.edu/.bin/fbidq.html?ems), [pros](http://flybase.bio.indiana.edu/.bin/fbidq.html?pros), [tup](http://flybase.bio.indiana.edu/.bin/fbidq.html?tup), [pdm2](http://flybase.bio.indiana.edu/.bin/fbidq.html?pdm2), [Gug](http://flybase.bio.indiana.edu/.bin/fbidq.html?Gug), [pad](http://flybase.bio.indiana.edu/.bin/fbidq.html?pad), [rgr](http://flybase.bio.indiana.edu/.bin/fbidq.html?rgr), [Wnt4](http://flybase.bio.indiana.edu/.bin/fbidq.html?Wnt4), [CG3714](http://flybase.bio.indiana.edu/.bin/fbidq.html?CG3714), [Rx](http://flybase.bio.indiana.edu/.bin/fbidq.html?Rx), [MED1](http://flybase.bio.indiana.edu/.bin/fbidq.html?MED1), [salr](http://flybase.bio.indiana.edu/.bin/fbidq.html?salr), [wdn](http://flybase.bio.indiana.edu/.bin/fbidq.html?wdn), [sim](http://flybase.bio.indiana.edu/.bin/fbidq.html?sim), [d4](http://flybase.bio.indiana.edu/.bin/fbidq.html?d4), [dan](http://flybase.bio.indiana.edu/.bin/fbidq.html?dan), [gro](http://flybase.bio.indiana.edu/.bin/fbidq.html?gro), [nerfin-1](http://flybase.bio.indiana.edu/.bin/fbidq.html?nerfin-1), [trx](http://flybase.bio.indiana.edu/.bin/fbidq.html?trx), [Rpd3](http://flybase.bio.indiana.edu/.bin/fbidq.html?Rpd3), [dsx](http://flybase.bio.indiana.edu/.bin/fbidq.html?dsx), [MED25](http://flybase.bio.indiana.edu/.bin/fbidq.html?MED25), [Ac13E](http://flybase.bio.indiana.edu/.bin/fbidq.html?Ac13E), [Ssdp](http://flybase.bio.indiana.edu/.bin/fbidq.html?Ssdp), [vfl](http://flybase.bio.indiana.edu/.bin/fbidq.html?vfl), [RpII140](http://flybase.bio.indiana.edu/.bin/fbidq.html?RpII140), [sqz](http://flybase.bio.indiana.edu/.bin/fbidq.html?sqz), [CG32105](http://flybase.bio.indiana.edu/.bin/fbidq.html?CG32105), [Fer3](http://flybase.bio.indiana.edu/.bin/fbidq.html?Fer3), [pygo](http://flybase.bio.indiana.edu/.bin/fbidq.html?pygo), [dalao](http://flybase.bio.indiana.edu/.bin/fbidq.html?dalao), [HLHm7](http://flybase.bio.indiana.edu/.bin/fbidq.html?HLHm7), [z](http://flybase.bio.indiana.edu/.bin/fbidq.html?z),[HLHmgamma](http://flybase.bio.indiana.edu/.bin/fbidq.html?HLHmgamma), [Sox21b](http://flybase.bio.indiana.edu/.bin/fbidq.html?Sox21b), [sr](http://flybase.bio.indiana.edu/.bin/fbidq.html?sr), [Lim1](http://flybase.bio.indiana.edu/.bin/fbidq.html?Lim1), [tld](http://flybase.bio.indiana.edu/.bin/fbidq.html?tld), [danr](http://flybase.bio.indiana.edu/.bin/fbidq.html?danr), [wor](http://flybase.bio.indiana.edu/.bin/fbidq.html?wor), [vvl](http://flybase.bio.indiana.edu/.bin/fbidq.html?vvl), [wda](http://flybase.bio.indiana.edu/.bin/fbidq.html?wda), [sgg](http://flybase.bio.indiana.edu/.bin/fbidq.html?sgg), [MED14](http://flybase.bio.indiana.edu/.bin/fbidq.html?MED14), [gcm2](http://flybase.bio.indiana.edu/.bin/fbidq.html?gcm2), [Eip93F](http://flybase.bio.indiana.edu/.bin/fbidq.html?Eip93F), [gcm](http://flybase.bio.indiana.edu/.bin/fbidq.html?gcm), [seq](http://flybase.bio.indiana.edu/.bin/fbidq.html?seq), [Dip3](http://flybase.bio.indiana.edu/.bin/fbidq.html?Dip3), [Aef1](http://flybase.bio.indiana.edu/.bin/fbidq.html?Aef1), [HDAC4](http://flybase.bio.indiana.edu/.bin/fbidq.html?HDAC4), [can](http://flybase.bio.indiana.edu/.bin/fbidq.html?can), [elk](http://flybase.bio.indiana.edu/.bin/fbidq.html?elk), [phl](http://flybase.bio.indiana.edu/.bin/fbidq.html?phl), [ase](http://flybase.bio.indiana.edu/.bin/fbidq.html?ase), [CG13253](http://flybase.bio.indiana.edu/.bin/fbidq.html?CG13253), [ovo](http://flybase.bio.indiana.edu/.bin/fbidq.html?ovo), [scro](http://flybase.bio.indiana.edu/.bin/fbidq.html?scro), [Dsp1](http://flybase.bio.indiana.edu/.bin/fbidq.html?Dsp1), [CG14216](http://flybase.bio.indiana.edu/.bin/fbidq.html?CG14216), [Pdp1](http://flybase.bio.indiana.edu/.bin/fbidq.html?Pdp1), [Oli](http://flybase.bio.indiana.edu/.bin/fbidq.html?Oli), [Nelf-A](http://flybase.bio.indiana.edu/.bin/fbidq.html?Nelf-A), [GATAd](http://flybase.bio.indiana.edu/.bin/fbidq.html?GATAd), [Poxn](http://flybase.bio.indiana.edu/.bin/fbidq.html?Poxn), [CG15376](http://flybase.bio.indiana.edu/.bin/fbidq.html?CG15376), [Sin3A](http://flybase.bio.indiana.edu/.bin/fbidq.html?Sin3A), [mip120](http://flybase.bio.indiana.edu/.bin/fbidq.html?mip120), [jumu](http://flybase.bio.indiana.edu/.bin/fbidq.html?jumu),[Smox](http://flybase.bio.indiana.edu/.bin/fbidq.html?Smox), [Usp7](http://flybase.bio.indiana.edu/.bin/fbidq.html?Usp7), [ush](http://flybase.bio.indiana.edu/.bin/fbidq.html?ush), [fd59A](http://flybase.bio.indiana.edu/.bin/fbidq.html?fd59A), [Gap1](http://flybase.bio.indiana.edu/.bin/fbidq.html?Gap1), [run](http://flybase.bio.indiana.edu/.bin/fbidq.html?run), [FBX011](http://flybase.bio.indiana.edu/.bin/fbidq.html?FBX011), [brat](http://flybase.bio.indiana.edu/.bin/fbidq.html?brat), [Antp](http://flybase.bio.indiana.edu/.bin/fbidq.html?Antp), [tara](http://flybase.bio.indiana.edu/.bin/fbidq.html?tara), [Alh](http://flybase.bio.indiana.edu/.bin/fbidq.html?Alh), [Sox102F](http://flybase.bio.indiana.edu/.bin/fbidq.html?Sox102F), [egg](http://flybase.bio.indiana.edu/.bin/fbidq.html?egg), [l(3)neo38](http://flybase.bio.indiana.edu/.bin/fbidq.html?l(3)neo38), [koko](http://flybase.bio.indiana.edu/.bin/fbidq.html?koko), [Ada](http://flybase.bio.indiana.edu/.bin/fbidq.html?Ada), [Ptth](http://flybase.bio.indiana.edu/.bin/fbidq.html?Ptth), [Sp1](http://flybase.bio.indiana.edu/.bin/fbidq.html?Sp1), [Ptx1](http://flybase.bio.indiana.edu/.bin/fbidq.html?Ptx1), [ph-p](http://flybase.bio.indiana.edu/.bin/fbidq.html?ph-p), [Ubx](http://flybase.bio.indiana.edu/.bin/fbidq.html?Ubx), [onecut](http://flybase.bio.indiana.edu/.bin/fbidq.html?onecut), [CG7757](http://flybase.bio.indiana.edu/.bin/fbidq.html?CG7757), [chm](http://flybase.bio.indiana.edu/.bin/fbidq.html?chm), [sna](http://flybase.bio.indiana.edu/.bin/fbidq.html?sna), [Wnt5](http://flybase.bio.indiana.edu/.bin/fbidq.html?Wnt5), [Taf1](http://flybase.bio.indiana.edu/.bin/fbidq.html?Taf1), [hbn](http://flybase.bio.indiana.edu/.bin/fbidq.html?hbn), [MBD-like](http://flybase.bio.indiana.edu/.bin/fbidq.html?MBD-like), [tsh](http://flybase.bio.indiana.edu/.bin/fbidq.html?tsh), [esg](http://flybase.bio.indiana.edu/.bin/fbidq.html?esg), [MED16](http://flybase.bio.indiana.edu/.bin/fbidq.html?MED16), [dac](http://flybase.bio.indiana.edu/.bin/fbidq.html?dac), [e(y)3](http://flybase.bio.indiana.edu/.bin/fbidq.html?e(y)3), [CRMP](http://flybase.bio.indiana.edu/.bin/fbidq.html?CRMP), [CG9007](http://flybase.bio.indiana.edu/.bin/fbidq.html?CG9007), [oc](http://flybase.bio.indiana.edu/.bin/fbidq.html?oc), [spt4](http://flybase.bio.indiana.edu/.bin/fbidq.html?spt4),[CG32532](http://flybase.bio.indiana.edu/.bin/fbidq.html?CG32532), [MTA1-like](http://flybase.bio.indiana.edu/.bin/fbidq.html?MTA1-like), [gol](http://flybase.bio.indiana.edu/.bin/fbidq.html?gol), [ey](http://flybase.bio.indiana.edu/.bin/fbidq.html?ey), [mle](http://flybase.bio.indiana.edu/.bin/fbidq.html?mle), [Hr51](http://flybase.bio.indiana.edu/.bin/fbidq.html?Hr51), [Bgb](http://flybase.bio.indiana.edu/.bin/fbidq.html?Bgb), [l(1)sc](http://flybase.bio.indiana.edu/.bin/fbidq.html?l(1)sc), [B-H2](http://flybase.bio.indiana.edu/.bin/fbidq.html?B-H2), [grh](http://flybase.bio.indiana.edu/.bin/fbidq.html?grh), [Vsx1](http://flybase.bio.indiana.edu/.bin/fbidq.html?Vsx1), [wg](http://flybase.bio.indiana.edu/.bin/fbidq.html?wg), [toy](http://flybase.bio.indiana.edu/.bin/fbidq.html?toy), [fkh](http://flybase.bio.indiana.edu/.bin/fbidq.html?fkh), [Lim3](http://flybase.bio.indiana.edu/.bin/fbidq.html?Lim3), [crol](http://flybase.bio.indiana.edu/.bin/fbidq.html?crol), [sd](http://flybase.bio.indiana.edu/.bin/fbidq.html?sd), [retn](http://flybase.bio.indiana.edu/.bin/fbidq.html?retn), [en](http://flybase.bio.indiana.edu/.bin/fbidq.html?en), [fs(1)h](http://flybase.bio.indiana.edu/.bin/fbidq.html?fs(1)h), [Ac3](http://flybase.bio.indiana.edu/.bin/fbidq.html?Ac3), [E(bx)](http://flybase.bio.indiana.edu/.bin/fbidq.html?E(bx)), [rl](http://flybase.bio.indiana.edu/.bin/fbidq.html?rl), [CG11347](http://flybase.bio.indiana.edu/.bin/fbidq.html?CG11347), [tey](http://flybase.bio.indiana.edu/.bin/fbidq.html?tey), [CG11294](http://flybase.bio.indiana.edu/.bin/fbidq.html?CG11294), [hiw](http://flybase.bio.indiana.edu/.bin/fbidq.html?hiw), [cos](http://flybase.bio.indiana.edu/.bin/fbidq.html?cos), [Ct](http://flybase.bio.indiana.edu/.bin/fbidq.html?Ct), [Atf-2](http://flybase.bio.indiana.edu/.bin/fbidq.html?Atf-2) |
| [mating behavior](http://amigo.geneontology.org/cgi-bin/amigo/go.cgi?view=details&query=GO:0007617) | 29 of 1594 genes, 1.8% | 54 of 7634 genes, 0.7% | 7.09e-05 | 0.00% | 0.00 | [tipE](http://flybase.bio.indiana.edu/.bin/fbidq.html?tipE), [Sh](http://flybase.bio.indiana.edu/.bin/fbidq.html?Sh), [Ilp2](http://flybase.bio.indiana.edu/.bin/fbidq.html?Ilp2), [Poxn](http://flybase.bio.indiana.edu/.bin/fbidq.html?Poxn), [qtc](http://flybase.bio.indiana.edu/.bin/fbidq.html?qtc), [Pde1c](http://flybase.bio.indiana.edu/.bin/fbidq.html?Pde1c), [CaMKII](http://flybase.bio.indiana.edu/.bin/fbidq.html?CaMKII), [npf](http://flybase.bio.indiana.edu/.bin/fbidq.html?npf), [ato](http://flybase.bio.indiana.edu/.bin/fbidq.html?ato), [fne](http://flybase.bio.indiana.edu/.bin/fbidq.html?fne), [Ilp3](http://flybase.bio.indiana.edu/.bin/fbidq.html?Ilp3), [para](http://flybase.bio.indiana.edu/.bin/fbidq.html?para), [retn](http://flybase.bio.indiana.edu/.bin/fbidq.html?retn), [5-HT7](http://flybase.bio.indiana.edu/.bin/fbidq.html?5-HT7), [rut](http://flybase.bio.indiana.edu/.bin/fbidq.html?rut), [Btk29A](http://flybase.bio.indiana.edu/.bin/fbidq.html?Btk29A), [dsx](http://flybase.bio.indiana.edu/.bin/fbidq.html?dsx), [Nrg](http://flybase.bio.indiana.edu/.bin/fbidq.html?Nrg), [Ilp5](http://flybase.bio.indiana.edu/.bin/fbidq.html?Ilp5), [Ddc](http://flybase.bio.indiana.edu/.bin/fbidq.html?Ddc), [SPR](http://flybase.bio.indiana.edu/.bin/fbidq.html?SPR), [pros](http://flybase.bio.indiana.edu/.bin/fbidq.html?pros), [Tbh](http://flybase.bio.indiana.edu/.bin/fbidq.html?Tbh), [Oamb](http://flybase.bio.indiana.edu/.bin/fbidq.html?Oamb), [mle](http://flybase.bio.indiana.edu/.bin/fbidq.html?mle), [orb2](http://flybase.bio.indiana.edu/.bin/fbidq.html?orb2), [nonA](http://flybase.bio.indiana.edu/.bin/fbidq.html?nonA), [CG10251](http://flybase.bio.indiana.edu/.bin/fbidq.html?CG10251), [CASK](http://flybase.bio.indiana.edu/.bin/fbidq.html?CASK) |
| [positive regulation of RNA metabolic process](http://amigo.geneontology.org/cgi-bin/amigo/go.cgi?view=details&query=GO:0051254) | 70 of 1594 genes, 4.4% | 191 of 7634 genes, 2.5% | 7.77e-05 | 0.00% | 0.00 | [acj6](http://flybase.bio.indiana.edu/.bin/fbidq.html?acj6), [wor](http://flybase.bio.indiana.edu/.bin/fbidq.html?wor), [vvl](http://flybase.bio.indiana.edu/.bin/fbidq.html?vvl), [caup](http://flybase.bio.indiana.edu/.bin/fbidq.html?caup), [Camta](http://flybase.bio.indiana.edu/.bin/fbidq.html?Camta), [tll](http://flybase.bio.indiana.edu/.bin/fbidq.html?tll), [ato](http://flybase.bio.indiana.edu/.bin/fbidq.html?ato), [tna](http://flybase.bio.indiana.edu/.bin/fbidq.html?tna), [Eip93F](http://flybase.bio.indiana.edu/.bin/fbidq.html?Eip93F), [JIL-1](http://flybase.bio.indiana.edu/.bin/fbidq.html?JIL-1), [ap](http://flybase.bio.indiana.edu/.bin/fbidq.html?ap), [Iswi](http://flybase.bio.indiana.edu/.bin/fbidq.html?Iswi), [Spt6](http://flybase.bio.indiana.edu/.bin/fbidq.html?Spt6), [gcm](http://flybase.bio.indiana.edu/.bin/fbidq.html?gcm), [Dip3](http://flybase.bio.indiana.edu/.bin/fbidq.html?Dip3), [ase](http://flybase.bio.indiana.edu/.bin/fbidq.html?ase), [br](http://flybase.bio.indiana.edu/.bin/fbidq.html?br), [ovo](http://flybase.bio.indiana.edu/.bin/fbidq.html?ovo), [Pdp1](http://flybase.bio.indiana.edu/.bin/fbidq.html?Pdp1), [chn](http://flybase.bio.indiana.edu/.bin/fbidq.html?chn), [Hcf](http://flybase.bio.indiana.edu/.bin/fbidq.html?Hcf), [lola](http://flybase.bio.indiana.edu/.bin/fbidq.html?lola), [NC2alpha](http://flybase.bio.indiana.edu/.bin/fbidq.html?NC2alpha), [Taf4](http://flybase.bio.indiana.edu/.bin/fbidq.html?Taf4), [HGTX](http://flybase.bio.indiana.edu/.bin/fbidq.html?HGTX), [fd59A](http://flybase.bio.indiana.edu/.bin/fbidq.html?fd59A), [btd](http://flybase.bio.indiana.edu/.bin/fbidq.html?btd), [run](http://flybase.bio.indiana.edu/.bin/fbidq.html?run), [Antp](http://flybase.bio.indiana.edu/.bin/fbidq.html?Antp), [ATbp](http://flybase.bio.indiana.edu/.bin/fbidq.html?ATbp), [tara](http://flybase.bio.indiana.edu/.bin/fbidq.html?tara), [hth](http://flybase.bio.indiana.edu/.bin/fbidq.html?hth), [vg](http://flybase.bio.indiana.edu/.bin/fbidq.html?vg), [eya](http://flybase.bio.indiana.edu/.bin/fbidq.html?eya), [brm](http://flybase.bio.indiana.edu/.bin/fbidq.html?brm), [onecut](http://flybase.bio.indiana.edu/.bin/fbidq.html?onecut), [sno](http://flybase.bio.indiana.edu/.bin/fbidq.html?sno), [chm](http://flybase.bio.indiana.edu/.bin/fbidq.html?chm), [ara](http://flybase.bio.indiana.edu/.bin/fbidq.html?ara), [sna](http://flybase.bio.indiana.edu/.bin/fbidq.html?sna), [mam](http://flybase.bio.indiana.edu/.bin/fbidq.html?mam), [Taf1](http://flybase.bio.indiana.edu/.bin/fbidq.html?Taf1), [pan](http://flybase.bio.indiana.edu/.bin/fbidq.html?pan), [disco](http://flybase.bio.indiana.edu/.bin/fbidq.html?disco),[esg](http://flybase.bio.indiana.edu/.bin/fbidq.html?esg), [e(y)3](http://flybase.bio.indiana.edu/.bin/fbidq.html?e(y)3), [Brf](http://flybase.bio.indiana.edu/.bin/fbidq.html?Brf), [oc](http://flybase.bio.indiana.edu/.bin/fbidq.html?oc), [tup](http://flybase.bio.indiana.edu/.bin/fbidq.html?tup), [spt4](http://flybase.bio.indiana.edu/.bin/fbidq.html?spt4), [ey](http://flybase.bio.indiana.edu/.bin/fbidq.html?ey), [mle](http://flybase.bio.indiana.edu/.bin/fbidq.html?mle), [l(1)sc](http://flybase.bio.indiana.edu/.bin/fbidq.html?l(1)sc), [Bgb](http://flybase.bio.indiana.edu/.bin/fbidq.html?Bgb), [grh](http://flybase.bio.indiana.edu/.bin/fbidq.html?grh), [toy](http://flybase.bio.indiana.edu/.bin/fbidq.html?toy), [wg](http://flybase.bio.indiana.edu/.bin/fbidq.html?wg), [fkh](http://flybase.bio.indiana.edu/.bin/fbidq.html?fkh), [sim](http://flybase.bio.indiana.edu/.bin/fbidq.html?sim), [sd](http://flybase.bio.indiana.edu/.bin/fbidq.html?sd), [trx](http://flybase.bio.indiana.edu/.bin/fbidq.html?trx), [retn](http://flybase.bio.indiana.edu/.bin/fbidq.html?retn), [dsx](http://flybase.bio.indiana.edu/.bin/fbidq.html?dsx), [vfl](http://flybase.bio.indiana.edu/.bin/fbidq.html?vfl), [CG11347](http://flybase.bio.indiana.edu/.bin/fbidq.html?CG11347), [dalao](http://flybase.bio.indiana.edu/.bin/fbidq.html?dalao), [pygo](http://flybase.bio.indiana.edu/.bin/fbidq.html?pygo), [z](http://flybase.bio.indiana.edu/.bin/fbidq.html?z), [Lim1](http://flybase.bio.indiana.edu/.bin/fbidq.html?Lim1), [Atf-2](http://flybase.bio.indiana.edu/.bin/fbidq.html?Atf-2) |
| [positive regulation of nucleobase-containing compound metabolic process](http://amigo.geneontology.org/cgi-bin/amigo/go.cgi?view=details&query=GO:0045935) | 71 of 1594 genes, 4.5% | 195 of 7634 genes, 2.6% | 8.44e-05 | 0.00% | 0.00 | [acj6](http://flybase.bio.indiana.edu/.bin/fbidq.html?acj6), [wor](http://flybase.bio.indiana.edu/.bin/fbidq.html?wor), [vvl](http://flybase.bio.indiana.edu/.bin/fbidq.html?vvl), [caup](http://flybase.bio.indiana.edu/.bin/fbidq.html?caup), [Camta](http://flybase.bio.indiana.edu/.bin/fbidq.html?Camta), [tll](http://flybase.bio.indiana.edu/.bin/fbidq.html?tll), [ato](http://flybase.bio.indiana.edu/.bin/fbidq.html?ato), [tna](http://flybase.bio.indiana.edu/.bin/fbidq.html?tna), [Eip93F](http://flybase.bio.indiana.edu/.bin/fbidq.html?Eip93F), [JIL-1](http://flybase.bio.indiana.edu/.bin/fbidq.html?JIL-1), [ap](http://flybase.bio.indiana.edu/.bin/fbidq.html?ap), [Iswi](http://flybase.bio.indiana.edu/.bin/fbidq.html?Iswi), [Spt6](http://flybase.bio.indiana.edu/.bin/fbidq.html?Spt6), [gcm](http://flybase.bio.indiana.edu/.bin/fbidq.html?gcm), [Dip3](http://flybase.bio.indiana.edu/.bin/fbidq.html?Dip3), [Nf1](http://flybase.bio.indiana.edu/.bin/fbidq.html?Nf1), [ase](http://flybase.bio.indiana.edu/.bin/fbidq.html?ase), [br](http://flybase.bio.indiana.edu/.bin/fbidq.html?br), [ovo](http://flybase.bio.indiana.edu/.bin/fbidq.html?ovo), [Pdp1](http://flybase.bio.indiana.edu/.bin/fbidq.html?Pdp1), [chn](http://flybase.bio.indiana.edu/.bin/fbidq.html?chn), [Hcf](http://flybase.bio.indiana.edu/.bin/fbidq.html?Hcf), [lola](http://flybase.bio.indiana.edu/.bin/fbidq.html?lola), [NC2alpha](http://flybase.bio.indiana.edu/.bin/fbidq.html?NC2alpha), [Taf4](http://flybase.bio.indiana.edu/.bin/fbidq.html?Taf4), [HGTX](http://flybase.bio.indiana.edu/.bin/fbidq.html?HGTX), [fd59A](http://flybase.bio.indiana.edu/.bin/fbidq.html?fd59A), [btd](http://flybase.bio.indiana.edu/.bin/fbidq.html?btd), [run](http://flybase.bio.indiana.edu/.bin/fbidq.html?run), [Antp](http://flybase.bio.indiana.edu/.bin/fbidq.html?Antp), [ATbp](http://flybase.bio.indiana.edu/.bin/fbidq.html?ATbp), [tara](http://flybase.bio.indiana.edu/.bin/fbidq.html?tara), [hth](http://flybase.bio.indiana.edu/.bin/fbidq.html?hth), [vg](http://flybase.bio.indiana.edu/.bin/fbidq.html?vg), [eya](http://flybase.bio.indiana.edu/.bin/fbidq.html?eya), [brm](http://flybase.bio.indiana.edu/.bin/fbidq.html?brm), [onecut](http://flybase.bio.indiana.edu/.bin/fbidq.html?onecut), [sno](http://flybase.bio.indiana.edu/.bin/fbidq.html?sno), [chm](http://flybase.bio.indiana.edu/.bin/fbidq.html?chm), [ara](http://flybase.bio.indiana.edu/.bin/fbidq.html?ara), [sna](http://flybase.bio.indiana.edu/.bin/fbidq.html?sna), [mam](http://flybase.bio.indiana.edu/.bin/fbidq.html?mam), [Taf1](http://flybase.bio.indiana.edu/.bin/fbidq.html?Taf1), [pan](http://flybase.bio.indiana.edu/.bin/fbidq.html?pan),[disco](http://flybase.bio.indiana.edu/.bin/fbidq.html?disco), [esg](http://flybase.bio.indiana.edu/.bin/fbidq.html?esg), [e(y)3](http://flybase.bio.indiana.edu/.bin/fbidq.html?e(y)3), [Brf](http://flybase.bio.indiana.edu/.bin/fbidq.html?Brf), [oc](http://flybase.bio.indiana.edu/.bin/fbidq.html?oc), [tup](http://flybase.bio.indiana.edu/.bin/fbidq.html?tup), [spt4](http://flybase.bio.indiana.edu/.bin/fbidq.html?spt4), [ey](http://flybase.bio.indiana.edu/.bin/fbidq.html?ey), [mle](http://flybase.bio.indiana.edu/.bin/fbidq.html?mle), [l(1)sc](http://flybase.bio.indiana.edu/.bin/fbidq.html?l(1)sc), [Bgb](http://flybase.bio.indiana.edu/.bin/fbidq.html?Bgb), [grh](http://flybase.bio.indiana.edu/.bin/fbidq.html?grh), [toy](http://flybase.bio.indiana.edu/.bin/fbidq.html?toy), [wg](http://flybase.bio.indiana.edu/.bin/fbidq.html?wg), [fkh](http://flybase.bio.indiana.edu/.bin/fbidq.html?fkh), [sim](http://flybase.bio.indiana.edu/.bin/fbidq.html?sim), [sd](http://flybase.bio.indiana.edu/.bin/fbidq.html?sd), [trx](http://flybase.bio.indiana.edu/.bin/fbidq.html?trx), [retn](http://flybase.bio.indiana.edu/.bin/fbidq.html?retn), [dsx](http://flybase.bio.indiana.edu/.bin/fbidq.html?dsx), [vfl](http://flybase.bio.indiana.edu/.bin/fbidq.html?vfl), [CG11347](http://flybase.bio.indiana.edu/.bin/fbidq.html?CG11347), [dalao](http://flybase.bio.indiana.edu/.bin/fbidq.html?dalao), [pygo](http://flybase.bio.indiana.edu/.bin/fbidq.html?pygo), [z](http://flybase.bio.indiana.edu/.bin/fbidq.html?z), [Lim1](http://flybase.bio.indiana.edu/.bin/fbidq.html?Lim1), [Atf-2](http://flybase.bio.indiana.edu/.bin/fbidq.html?Atf-2) |
| [positive regulation of gene expression](http://amigo.geneontology.org/cgi-bin/amigo/go.cgi?view=details&query=GO:0010628) | 69 of 1594 genes, 4.3% | 188 of 7634 genes, 2.5% | 9.12e-05 | 0.00% | 0.00 | [acj6](http://flybase.bio.indiana.edu/.bin/fbidq.html?acj6), [wor](http://flybase.bio.indiana.edu/.bin/fbidq.html?wor), [vvl](http://flybase.bio.indiana.edu/.bin/fbidq.html?vvl), [caup](http://flybase.bio.indiana.edu/.bin/fbidq.html?caup), [Camta](http://flybase.bio.indiana.edu/.bin/fbidq.html?Camta), [tll](http://flybase.bio.indiana.edu/.bin/fbidq.html?tll), [ato](http://flybase.bio.indiana.edu/.bin/fbidq.html?ato), [tna](http://flybase.bio.indiana.edu/.bin/fbidq.html?tna), [Eip93F](http://flybase.bio.indiana.edu/.bin/fbidq.html?Eip93F), [JIL-1](http://flybase.bio.indiana.edu/.bin/fbidq.html?JIL-1), [ap](http://flybase.bio.indiana.edu/.bin/fbidq.html?ap), [Iswi](http://flybase.bio.indiana.edu/.bin/fbidq.html?Iswi), [gcm](http://flybase.bio.indiana.edu/.bin/fbidq.html?gcm), [Dip3](http://flybase.bio.indiana.edu/.bin/fbidq.html?Dip3), [ase](http://flybase.bio.indiana.edu/.bin/fbidq.html?ase), [br](http://flybase.bio.indiana.edu/.bin/fbidq.html?br), [ovo](http://flybase.bio.indiana.edu/.bin/fbidq.html?ovo), [Pdp1](http://flybase.bio.indiana.edu/.bin/fbidq.html?Pdp1), [chn](http://flybase.bio.indiana.edu/.bin/fbidq.html?chn), [Hcf](http://flybase.bio.indiana.edu/.bin/fbidq.html?Hcf), [lola](http://flybase.bio.indiana.edu/.bin/fbidq.html?lola), [NC2alpha](http://flybase.bio.indiana.edu/.bin/fbidq.html?NC2alpha), [Taf4](http://flybase.bio.indiana.edu/.bin/fbidq.html?Taf4), [HGTX](http://flybase.bio.indiana.edu/.bin/fbidq.html?HGTX), [fd59A](http://flybase.bio.indiana.edu/.bin/fbidq.html?fd59A), [btd](http://flybase.bio.indiana.edu/.bin/fbidq.html?btd), [run](http://flybase.bio.indiana.edu/.bin/fbidq.html?run), [Antp](http://flybase.bio.indiana.edu/.bin/fbidq.html?Antp), [ATbp](http://flybase.bio.indiana.edu/.bin/fbidq.html?ATbp), [tara](http://flybase.bio.indiana.edu/.bin/fbidq.html?tara), [Fer2](http://flybase.bio.indiana.edu/.bin/fbidq.html?Fer2), [hth](http://flybase.bio.indiana.edu/.bin/fbidq.html?hth), [vg](http://flybase.bio.indiana.edu/.bin/fbidq.html?vg), [eya](http://flybase.bio.indiana.edu/.bin/fbidq.html?eya), [brm](http://flybase.bio.indiana.edu/.bin/fbidq.html?brm), [onecut](http://flybase.bio.indiana.edu/.bin/fbidq.html?onecut), [sno](http://flybase.bio.indiana.edu/.bin/fbidq.html?sno), [chm](http://flybase.bio.indiana.edu/.bin/fbidq.html?chm), [sna](http://flybase.bio.indiana.edu/.bin/fbidq.html?sna), [ara](http://flybase.bio.indiana.edu/.bin/fbidq.html?ara), [mam](http://flybase.bio.indiana.edu/.bin/fbidq.html?mam), [Taf1](http://flybase.bio.indiana.edu/.bin/fbidq.html?Taf1), [pan](http://flybase.bio.indiana.edu/.bin/fbidq.html?pan), [disco](http://flybase.bio.indiana.edu/.bin/fbidq.html?disco),[esg](http://flybase.bio.indiana.edu/.bin/fbidq.html?esg), [e(y)3](http://flybase.bio.indiana.edu/.bin/fbidq.html?e(y)3), [Brf](http://flybase.bio.indiana.edu/.bin/fbidq.html?Brf), [oc](http://flybase.bio.indiana.edu/.bin/fbidq.html?oc), [tup](http://flybase.bio.indiana.edu/.bin/fbidq.html?tup), [ey](http://flybase.bio.indiana.edu/.bin/fbidq.html?ey), [mle](http://flybase.bio.indiana.edu/.bin/fbidq.html?mle), [l(1)sc](http://flybase.bio.indiana.edu/.bin/fbidq.html?l(1)sc), [Bgb](http://flybase.bio.indiana.edu/.bin/fbidq.html?Bgb), [grh](http://flybase.bio.indiana.edu/.bin/fbidq.html?grh), [toy](http://flybase.bio.indiana.edu/.bin/fbidq.html?toy), [wg](http://flybase.bio.indiana.edu/.bin/fbidq.html?wg), [fkh](http://flybase.bio.indiana.edu/.bin/fbidq.html?fkh), [sim](http://flybase.bio.indiana.edu/.bin/fbidq.html?sim), [sd](http://flybase.bio.indiana.edu/.bin/fbidq.html?sd), [trx](http://flybase.bio.indiana.edu/.bin/fbidq.html?trx), [retn](http://flybase.bio.indiana.edu/.bin/fbidq.html?retn), [dsx](http://flybase.bio.indiana.edu/.bin/fbidq.html?dsx), [vfl](http://flybase.bio.indiana.edu/.bin/fbidq.html?vfl), [CG11347](http://flybase.bio.indiana.edu/.bin/fbidq.html?CG11347), [dalao](http://flybase.bio.indiana.edu/.bin/fbidq.html?dalao), [pygo](http://flybase.bio.indiana.edu/.bin/fbidq.html?pygo), [z](http://flybase.bio.indiana.edu/.bin/fbidq.html?z), [Lim1](http://flybase.bio.indiana.edu/.bin/fbidq.html?Lim1), [Atf-2](http://flybase.bio.indiana.edu/.bin/fbidq.html?Atf-2) |
| [cellular response to stimulus](http://amigo.geneontology.org/cgi-bin/amigo/go.cgi?view=details&query=GO:0051716) | 310 of 1594 genes, 19.4% | 1209 of 7634 genes, 15.8% | 0.00010 | 0.00% | 0.00 | [fz2](http://flybase.bio.indiana.edu/.bin/fbidq.html?fz2), [CG14375](http://flybase.bio.indiana.edu/.bin/fbidq.html?CG14375), [Ocho](http://flybase.bio.indiana.edu/.bin/fbidq.html?Ocho), [numb](http://flybase.bio.indiana.edu/.bin/fbidq.html?numb), [dock](http://flybase.bio.indiana.edu/.bin/fbidq.html?dock), [mus101](http://flybase.bio.indiana.edu/.bin/fbidq.html?mus101), [Sxl](http://flybase.bio.indiana.edu/.bin/fbidq.html?Sxl), [CG8500](http://flybase.bio.indiana.edu/.bin/fbidq.html?CG8500), [cenG1A](http://flybase.bio.indiana.edu/.bin/fbidq.html?cenG1A), [Nf1](http://flybase.bio.indiana.edu/.bin/fbidq.html?Nf1), [malpha](http://flybase.bio.indiana.edu/.bin/fbidq.html?malpha), [AR-2](http://flybase.bio.indiana.edu/.bin/fbidq.html?AR-2), [os](http://flybase.bio.indiana.edu/.bin/fbidq.html?os), [Topors](http://flybase.bio.indiana.edu/.bin/fbidq.html?Topors), [Axn](http://flybase.bio.indiana.edu/.bin/fbidq.html?Axn), [Traf6](http://flybase.bio.indiana.edu/.bin/fbidq.html?Traf6), [Leucokinin](http://flybase.bio.indiana.edu/.bin/fbidq.html?Leucokinin), [rdgC](http://flybase.bio.indiana.edu/.bin/fbidq.html?rdgC), [Galpha49B](http://flybase.bio.indiana.edu/.bin/fbidq.html?Galpha49B), [Snoo](http://flybase.bio.indiana.edu/.bin/fbidq.html?Snoo), [rut](http://flybase.bio.indiana.edu/.bin/fbidq.html?rut), [CG7650](http://flybase.bio.indiana.edu/.bin/fbidq.html?CG7650), [RhoGAP100F](http://flybase.bio.indiana.edu/.bin/fbidq.html?RhoGAP100F), [uif](http://flybase.bio.indiana.edu/.bin/fbidq.html?uif), [Mmp2](http://flybase.bio.indiana.edu/.bin/fbidq.html?Mmp2), [HLHmdelta](http://flybase.bio.indiana.edu/.bin/fbidq.html?HLHmdelta), [Nplp1](http://flybase.bio.indiana.edu/.bin/fbidq.html?Nplp1), [Nipped-A](http://flybase.bio.indiana.edu/.bin/fbidq.html?Nipped-A), [CG32206](http://flybase.bio.indiana.edu/.bin/fbidq.html?CG32206), [Vha68-1](http://flybase.bio.indiana.edu/.bin/fbidq.html?Vha68-1),[7B2](http://flybase.bio.indiana.edu/.bin/fbidq.html?7B2), [alph](http://flybase.bio.indiana.edu/.bin/fbidq.html?alph), [Traf4](http://flybase.bio.indiana.edu/.bin/fbidq.html?Traf4), [Spf45](http://flybase.bio.indiana.edu/.bin/fbidq.html?Spf45), [Ulp1](http://flybase.bio.indiana.edu/.bin/fbidq.html?Ulp1), [mGluRA](http://flybase.bio.indiana.edu/.bin/fbidq.html?mGluRA), [dsh](http://flybase.bio.indiana.edu/.bin/fbidq.html?dsh), [pzg](http://flybase.bio.indiana.edu/.bin/fbidq.html?pzg), [Nf-YC](http://flybase.bio.indiana.edu/.bin/fbidq.html?Nf-YC), [14-3-3zeta](http://flybase.bio.indiana.edu/.bin/fbidq.html?14-3-3zeta), [Pli](http://flybase.bio.indiana.edu/.bin/fbidq.html?Pli), [shakB](http://flybase.bio.indiana.edu/.bin/fbidq.html?shakB), [CG31140](http://flybase.bio.indiana.edu/.bin/fbidq.html?CG31140), [Hey](http://flybase.bio.indiana.edu/.bin/fbidq.html?Hey), [plexB](http://flybase.bio.indiana.edu/.bin/fbidq.html?plexB), [AlstR](http://flybase.bio.indiana.edu/.bin/fbidq.html?AlstR), [PP2A-B](http://flybase.bio.indiana.edu/.bin/fbidq.html?PP2A-B), [loqs](http://flybase.bio.indiana.edu/.bin/fbidq.html?loqs), [IM10](http://flybase.bio.indiana.edu/.bin/fbidq.html?IM10), [neur](http://flybase.bio.indiana.edu/.bin/fbidq.html?neur), [CycE](http://flybase.bio.indiana.edu/.bin/fbidq.html?CycE), [rho-5](http://flybase.bio.indiana.edu/.bin/fbidq.html?rho-5), [nmo](http://flybase.bio.indiana.edu/.bin/fbidq.html?nmo), [Tusp](http://flybase.bio.indiana.edu/.bin/fbidq.html?Tusp), [5-HT1A](http://flybase.bio.indiana.edu/.bin/fbidq.html?5-HT1A), [CG32149](http://flybase.bio.indiana.edu/.bin/fbidq.html?CG32149), [Vdup1](http://flybase.bio.indiana.edu/.bin/fbidq.html?Vdup1), [Gug](http://flybase.bio.indiana.edu/.bin/fbidq.html?Gug), [ed](http://flybase.bio.indiana.edu/.bin/fbidq.html?ed), [daw](http://flybase.bio.indiana.edu/.bin/fbidq.html?daw), [CG5036](http://flybase.bio.indiana.edu/.bin/fbidq.html?CG5036), [5-HT1B](http://flybase.bio.indiana.edu/.bin/fbidq.html?5-HT1B), [Apc](http://flybase.bio.indiana.edu/.bin/fbidq.html?Apc), [sim](http://flybase.bio.indiana.edu/.bin/fbidq.html?sim), [CG8795](http://flybase.bio.indiana.edu/.bin/fbidq.html?CG8795),[gro](http://flybase.bio.indiana.edu/.bin/fbidq.html?gro), [RN-tre](http://flybase.bio.indiana.edu/.bin/fbidq.html?RN-tre), [scyl](http://flybase.bio.indiana.edu/.bin/fbidq.html?scyl), [wnd](http://flybase.bio.indiana.edu/.bin/fbidq.html?wnd), [trx](http://flybase.bio.indiana.edu/.bin/fbidq.html?trx), [Btk29A](http://flybase.bio.indiana.edu/.bin/fbidq.html?Btk29A), [SMC2](http://flybase.bio.indiana.edu/.bin/fbidq.html?SMC2), [ETH](http://flybase.bio.indiana.edu/.bin/fbidq.html?ETH), [unc-5](http://flybase.bio.indiana.edu/.bin/fbidq.html?unc-5), [cdi](http://flybase.bio.indiana.edu/.bin/fbidq.html?cdi), [NPFR1](http://flybase.bio.indiana.edu/.bin/fbidq.html?NPFR1), [pygo](http://flybase.bio.indiana.edu/.bin/fbidq.html?pygo), [HLHm7](http://flybase.bio.indiana.edu/.bin/fbidq.html?HLHm7), [HLHmgamma](http://flybase.bio.indiana.edu/.bin/fbidq.html?HLHmgamma), [CG15556](http://flybase.bio.indiana.edu/.bin/fbidq.html?CG15556), [Ror](http://flybase.bio.indiana.edu/.bin/fbidq.html?Ror), [tow](http://flybase.bio.indiana.edu/.bin/fbidq.html?tow), [CG15609](http://flybase.bio.indiana.edu/.bin/fbidq.html?CG15609), [Atg1](http://flybase.bio.indiana.edu/.bin/fbidq.html?Atg1), [Pten](http://flybase.bio.indiana.edu/.bin/fbidq.html?Pten), [unc-13](http://flybase.bio.indiana.edu/.bin/fbidq.html?unc-13), [npf](http://flybase.bio.indiana.edu/.bin/fbidq.html?npf), [oa2](http://flybase.bio.indiana.edu/.bin/fbidq.html?oa2), [elk](http://flybase.bio.indiana.edu/.bin/fbidq.html?elk), [phl](http://flybase.bio.indiana.edu/.bin/fbidq.html?phl), [Cbl](http://flybase.bio.indiana.edu/.bin/fbidq.html?Cbl), [Rab26](http://flybase.bio.indiana.edu/.bin/fbidq.html?Rab26), [Pde6](http://flybase.bio.indiana.edu/.bin/fbidq.html?Pde6), [sNPF](http://flybase.bio.indiana.edu/.bin/fbidq.html?sNPF), [pog](http://flybase.bio.indiana.edu/.bin/fbidq.html?pog), [plx](http://flybase.bio.indiana.edu/.bin/fbidq.html?plx), [CG9098](http://flybase.bio.indiana.edu/.bin/fbidq.html?CG9098), [CG8108](http://flybase.bio.indiana.edu/.bin/fbidq.html?CG8108), [IFa](http://flybase.bio.indiana.edu/.bin/fbidq.html?IFa), [mtt](http://flybase.bio.indiana.edu/.bin/fbidq.html?mtt), [CG32758](http://flybase.bio.indiana.edu/.bin/fbidq.html?CG32758),[Alk](http://flybase.bio.indiana.edu/.bin/fbidq.html?Alk), [Pde1c](http://flybase.bio.indiana.edu/.bin/fbidq.html?Pde1c), [disp](http://flybase.bio.indiana.edu/.bin/fbidq.html?disp), [casp](http://flybase.bio.indiana.edu/.bin/fbidq.html?casp), [CG4022](http://flybase.bio.indiana.edu/.bin/fbidq.html?CG4022), [Smox](http://flybase.bio.indiana.edu/.bin/fbidq.html?Smox), [Plc21C](http://flybase.bio.indiana.edu/.bin/fbidq.html?Plc21C), [GRHRII](http://flybase.bio.indiana.edu/.bin/fbidq.html?GRHRII), [CG10362](http://flybase.bio.indiana.edu/.bin/fbidq.html?CG10362), [GABA-B-R2](http://flybase.bio.indiana.edu/.bin/fbidq.html?GABA-B-R2), [Ast-C](http://flybase.bio.indiana.edu/.bin/fbidq.html?Ast-C), [Gbeta5](http://flybase.bio.indiana.edu/.bin/fbidq.html?Gbeta5), [Rpt3R](http://flybase.bio.indiana.edu/.bin/fbidq.html?Rpt3R), [hug](http://flybase.bio.indiana.edu/.bin/fbidq.html?hug), [Sur-8](http://flybase.bio.indiana.edu/.bin/fbidq.html?Sur-8), [Rgl](http://flybase.bio.indiana.edu/.bin/fbidq.html?Rgl), [e(y)3](http://flybase.bio.indiana.edu/.bin/fbidq.html?e(y)3), [CRMP](http://flybase.bio.indiana.edu/.bin/fbidq.html?CRMP), [Cdk5](http://flybase.bio.indiana.edu/.bin/fbidq.html?Cdk5), [mav](http://flybase.bio.indiana.edu/.bin/fbidq.html?mav), [Ilp7](http://flybase.bio.indiana.edu/.bin/fbidq.html?Ilp7), [ey](http://flybase.bio.indiana.edu/.bin/fbidq.html?ey), [Mip](http://flybase.bio.indiana.edu/.bin/fbidq.html?Mip), [baz](http://flybase.bio.indiana.edu/.bin/fbidq.html?baz), [CG33275](http://flybase.bio.indiana.edu/.bin/fbidq.html?CG33275), [Oamb](http://flybase.bio.indiana.edu/.bin/fbidq.html?Oamb), [CG34381](http://flybase.bio.indiana.edu/.bin/fbidq.html?CG34381), [Cdk5alpha](http://flybase.bio.indiana.edu/.bin/fbidq.html?Cdk5alpha), [CG40351](http://flybase.bio.indiana.edu/.bin/fbidq.html?CG40351), [crol](http://flybase.bio.indiana.edu/.bin/fbidq.html?crol), [sd](http://flybase.bio.indiana.edu/.bin/fbidq.html?sd), [Aplip1](http://flybase.bio.indiana.edu/.bin/fbidq.html?Aplip1), [Ac3](http://flybase.bio.indiana.edu/.bin/fbidq.html?Ac3), [rl](http://flybase.bio.indiana.edu/.bin/fbidq.html?rl),[CG17760](http://flybase.bio.indiana.edu/.bin/fbidq.html?CG17760), [Nup153](http://flybase.bio.indiana.edu/.bin/fbidq.html?Nup153), [CG32683](http://flybase.bio.indiana.edu/.bin/fbidq.html?CG32683), [CG11347](http://flybase.bio.indiana.edu/.bin/fbidq.html?CG11347), [Ggamma30A](http://flybase.bio.indiana.edu/.bin/fbidq.html?Ggamma30A), [wls](http://flybase.bio.indiana.edu/.bin/fbidq.html?wls), [CG33960](http://flybase.bio.indiana.edu/.bin/fbidq.html?CG33960), [hiw](http://flybase.bio.indiana.edu/.bin/fbidq.html?hiw), [Atf-2](http://flybase.bio.indiana.edu/.bin/fbidq.html?Atf-2), [skd](http://flybase.bio.indiana.edu/.bin/fbidq.html?skd), [Cbp80](http://flybase.bio.indiana.edu/.bin/fbidq.html?Cbp80), [Camta](http://flybase.bio.indiana.edu/.bin/fbidq.html?Camta), [tll](http://flybase.bio.indiana.edu/.bin/fbidq.html?tll), [ato](http://flybase.bio.indiana.edu/.bin/fbidq.html?ato), [Fmrf](http://flybase.bio.indiana.edu/.bin/fbidq.html?Fmrf), [Iswi](http://flybase.bio.indiana.edu/.bin/fbidq.html?Iswi), [Trim9](http://flybase.bio.indiana.edu/.bin/fbidq.html?Trim9), [GABA-B-R3](http://flybase.bio.indiana.edu/.bin/fbidq.html?GABA-B-R3), [Pka-R1](http://flybase.bio.indiana.edu/.bin/fbidq.html?Pka-R1), [Evi5](http://flybase.bio.indiana.edu/.bin/fbidq.html?Evi5), [mago](http://flybase.bio.indiana.edu/.bin/fbidq.html?mago), [br](http://flybase.bio.indiana.edu/.bin/fbidq.html?br), [Gycalpha99B](http://flybase.bio.indiana.edu/.bin/fbidq.html?Gycalpha99B), [ksr](http://flybase.bio.indiana.edu/.bin/fbidq.html?ksr), [CG42533](http://flybase.bio.indiana.edu/.bin/fbidq.html?CG42533), [CG11376](http://flybase.bio.indiana.edu/.bin/fbidq.html?CG11376), [Gyc-89Da](http://flybase.bio.indiana.edu/.bin/fbidq.html?Gyc-89Da), [D2R](http://flybase.bio.indiana.edu/.bin/fbidq.html?D2R), [CG31158](http://flybase.bio.indiana.edu/.bin/fbidq.html?CG31158), [ewg](http://flybase.bio.indiana.edu/.bin/fbidq.html?ewg),[Nrk](http://flybase.bio.indiana.edu/.bin/fbidq.html?Nrk), [Cirl](http://flybase.bio.indiana.edu/.bin/fbidq.html?Cirl), [Ilp3](http://flybase.bio.indiana.edu/.bin/fbidq.html?Ilp3), [Su(var)3-3](http://flybase.bio.indiana.edu/.bin/fbidq.html?Su(var)3-3), [Bili](http://flybase.bio.indiana.edu/.bin/fbidq.html?Bili), [Nrg](http://flybase.bio.indiana.edu/.bin/fbidq.html?Nrg), [CG34384](http://flybase.bio.indiana.edu/.bin/fbidq.html?CG34384), [stet](http://flybase.bio.indiana.edu/.bin/fbidq.html?stet), [Dh44-R1](http://flybase.bio.indiana.edu/.bin/fbidq.html?Dh44-R1), [CG32447](http://flybase.bio.indiana.edu/.bin/fbidq.html?CG32447), [Eh](http://flybase.bio.indiana.edu/.bin/fbidq.html?Eh), [Sema-1a](http://flybase.bio.indiana.edu/.bin/fbidq.html?Sema-1a), [Hs3st-B](http://flybase.bio.indiana.edu/.bin/fbidq.html?Hs3st-B), [otk](http://flybase.bio.indiana.edu/.bin/fbidq.html?otk), [Sos](http://flybase.bio.indiana.edu/.bin/fbidq.html?Sos), [phyl](http://flybase.bio.indiana.edu/.bin/fbidq.html?phyl), [Fas2](http://flybase.bio.indiana.edu/.bin/fbidq.html?Fas2), [fz](http://flybase.bio.indiana.edu/.bin/fbidq.html?fz), [robo](http://flybase.bio.indiana.edu/.bin/fbidq.html?robo), [CG8557](http://flybase.bio.indiana.edu/.bin/fbidq.html?CG8557), [phr](http://flybase.bio.indiana.edu/.bin/fbidq.html?phr), [bsk](http://flybase.bio.indiana.edu/.bin/fbidq.html?bsk), [brm](http://flybase.bio.indiana.edu/.bin/fbidq.html?brm), [mAcR-60C](http://flybase.bio.indiana.edu/.bin/fbidq.html?mAcR-60C), [Crz](http://flybase.bio.indiana.edu/.bin/fbidq.html?Crz), [ft](http://flybase.bio.indiana.edu/.bin/fbidq.html?ft), [CG13830](http://flybase.bio.indiana.edu/.bin/fbidq.html?CG13830), [sno](http://flybase.bio.indiana.edu/.bin/fbidq.html?sno), [RhoGAPp190](http://flybase.bio.indiana.edu/.bin/fbidq.html?RhoGAPp190), [l(2)k16918](http://flybase.bio.indiana.edu/.bin/fbidq.html?l(2)k16918), [mam](http://flybase.bio.indiana.edu/.bin/fbidq.html?mam), [CG30158](http://flybase.bio.indiana.edu/.bin/fbidq.html?CG30158), [Takr86C](http://flybase.bio.indiana.edu/.bin/fbidq.html?Takr86C),[pan](http://flybase.bio.indiana.edu/.bin/fbidq.html?pan), [PKD](http://flybase.bio.indiana.edu/.bin/fbidq.html?PKD), [DopR](http://flybase.bio.indiana.edu/.bin/fbidq.html?DopR), [Lis-1](http://flybase.bio.indiana.edu/.bin/fbidq.html?Lis-1), [CG16896](http://flybase.bio.indiana.edu/.bin/fbidq.html?CG16896), [Rab3](http://flybase.bio.indiana.edu/.bin/fbidq.html?Rab3), [tup](http://flybase.bio.indiana.edu/.bin/fbidq.html?tup), [Gen](http://flybase.bio.indiana.edu/.bin/fbidq.html?Gen), [mthl8](http://flybase.bio.indiana.edu/.bin/fbidq.html?mthl8), [trio](http://flybase.bio.indiana.edu/.bin/fbidq.html?trio), [RabX4](http://flybase.bio.indiana.edu/.bin/fbidq.html?RabX4), [spel1](http://flybase.bio.indiana.edu/.bin/fbidq.html?spel1), [Lkr](http://flybase.bio.indiana.edu/.bin/fbidq.html?Lkr), [CG31665](http://flybase.bio.indiana.edu/.bin/fbidq.html?CG31665), [Ccap](http://flybase.bio.indiana.edu/.bin/fbidq.html?Ccap), [tipE](http://flybase.bio.indiana.edu/.bin/fbidq.html?tipE), [Wnt4](http://flybase.bio.indiana.edu/.bin/fbidq.html?Wnt4), [CG7918](http://flybase.bio.indiana.edu/.bin/fbidq.html?CG7918), [rictor](http://flybase.bio.indiana.edu/.bin/fbidq.html?rictor), [DopR2](http://flybase.bio.indiana.edu/.bin/fbidq.html?DopR2), [CG30372](http://flybase.bio.indiana.edu/.bin/fbidq.html?CG30372), [CG10188](http://flybase.bio.indiana.edu/.bin/fbidq.html?CG10188), [CG42629](http://flybase.bio.indiana.edu/.bin/fbidq.html?CG42629), [pigs](http://flybase.bio.indiana.edu/.bin/fbidq.html?pigs), [CG31760](http://flybase.bio.indiana.edu/.bin/fbidq.html?CG31760), [Klp54D](http://flybase.bio.indiana.edu/.bin/fbidq.html?Klp54D), [pr-set7](http://flybase.bio.indiana.edu/.bin/fbidq.html?pr-set7), [Fancd2](http://flybase.bio.indiana.edu/.bin/fbidq.html?Fancd2), [5-HT7](http://flybase.bio.indiana.edu/.bin/fbidq.html?5-HT7), [Lgr3](http://flybase.bio.indiana.edu/.bin/fbidq.html?Lgr3), [Dh](http://flybase.bio.indiana.edu/.bin/fbidq.html?Dh), [mei-9](http://flybase.bio.indiana.edu/.bin/fbidq.html?mei-9),[Ac13E](http://flybase.bio.indiana.edu/.bin/fbidq.html?Ac13E), [klu](http://flybase.bio.indiana.edu/.bin/fbidq.html?klu), [SPR](http://flybase.bio.indiana.edu/.bin/fbidq.html?SPR), [CG8155](http://flybase.bio.indiana.edu/.bin/fbidq.html?CG8155), [nkd](http://flybase.bio.indiana.edu/.bin/fbidq.html?nkd), [tld](http://flybase.bio.indiana.edu/.bin/fbidq.html?tld), [RhoGEF3](http://flybase.bio.indiana.edu/.bin/fbidq.html?RhoGEF3), [Dsk](http://flybase.bio.indiana.edu/.bin/fbidq.html?Dsk), [RhoGEF4](http://flybase.bio.indiana.edu/.bin/fbidq.html?RhoGEF4), [CG9121](http://flybase.bio.indiana.edu/.bin/fbidq.html?CG9121), [Brd](http://flybase.bio.indiana.edu/.bin/fbidq.html?Brd), [sgg](http://flybase.bio.indiana.edu/.bin/fbidq.html?sgg), [Nmdar1](http://flybase.bio.indiana.edu/.bin/fbidq.html?Nmdar1), [Eip93F](http://flybase.bio.indiana.edu/.bin/fbidq.html?Eip93F), [inaE](http://flybase.bio.indiana.edu/.bin/fbidq.html?inaE), [mus210](http://flybase.bio.indiana.edu/.bin/fbidq.html?mus210), [Hsp67Bc](http://flybase.bio.indiana.edu/.bin/fbidq.html?Hsp67Bc), [Smc5](http://flybase.bio.indiana.edu/.bin/fbidq.html?Smc5), [Pask](http://flybase.bio.indiana.edu/.bin/fbidq.html?Pask), [rab3-GEF](http://flybase.bio.indiana.edu/.bin/fbidq.html?rab3-GEF), [CG13253](http://flybase.bio.indiana.edu/.bin/fbidq.html?CG13253), [MESK2](http://flybase.bio.indiana.edu/.bin/fbidq.html?MESK2), [SIFR](http://flybase.bio.indiana.edu/.bin/fbidq.html?SIFR), [CG2061](http://flybase.bio.indiana.edu/.bin/fbidq.html?CG2061), [Ast](http://flybase.bio.indiana.edu/.bin/fbidq.html?Ast), [fred](http://flybase.bio.indiana.edu/.bin/fbidq.html?fred), [Drl-2](http://flybase.bio.indiana.edu/.bin/fbidq.html?Drl-2), [Tsp42Ej](http://flybase.bio.indiana.edu/.bin/fbidq.html?Tsp42Ej), [ush](http://flybase.bio.indiana.edu/.bin/fbidq.html?ush), [Tk](http://flybase.bio.indiana.edu/.bin/fbidq.html?Tk), [Fak56D](http://flybase.bio.indiana.edu/.bin/fbidq.html?Fak56D), [Ilp5](http://flybase.bio.indiana.edu/.bin/fbidq.html?Ilp5),[CG10336](http://flybase.bio.indiana.edu/.bin/fbidq.html?CG10336), [Gap1](http://flybase.bio.indiana.edu/.bin/fbidq.html?Gap1), [Toll-6](http://flybase.bio.indiana.edu/.bin/fbidq.html?Toll-6), [CG3227](http://flybase.bio.indiana.edu/.bin/fbidq.html?CG3227), [MESR4](http://flybase.bio.indiana.edu/.bin/fbidq.html?MESR4), [Takr99D](http://flybase.bio.indiana.edu/.bin/fbidq.html?Takr99D), [sNPF-R](http://flybase.bio.indiana.edu/.bin/fbidq.html?sNPF-R), [Eip71CD](http://flybase.bio.indiana.edu/.bin/fbidq.html?Eip71CD), [Hel89B](http://flybase.bio.indiana.edu/.bin/fbidq.html?Hel89B), [CG5337](http://flybase.bio.indiana.edu/.bin/fbidq.html?CG5337), [CG13692](http://flybase.bio.indiana.edu/.bin/fbidq.html?CG13692), [koko](http://flybase.bio.indiana.edu/.bin/fbidq.html?koko), [stan](http://flybase.bio.indiana.edu/.bin/fbidq.html?stan), [CG12424](http://flybase.bio.indiana.edu/.bin/fbidq.html?CG12424), [Ptth](http://flybase.bio.indiana.edu/.bin/fbidq.html?Ptth), [Rab2](http://flybase.bio.indiana.edu/.bin/fbidq.html?Rab2), [Ilp2](http://flybase.bio.indiana.edu/.bin/fbidq.html?Ilp2), [Fancl](http://flybase.bio.indiana.edu/.bin/fbidq.html?Fancl), [SPoCk](http://flybase.bio.indiana.edu/.bin/fbidq.html?SPoCk), [Wnt5](http://flybase.bio.indiana.edu/.bin/fbidq.html?Wnt5), [Taf1](http://flybase.bio.indiana.edu/.bin/fbidq.html?Taf1), [SF2](http://flybase.bio.indiana.edu/.bin/fbidq.html?SF2), [Ggamma1](http://flybase.bio.indiana.edu/.bin/fbidq.html?Ggamma1), [fus](http://flybase.bio.indiana.edu/.bin/fbidq.html?fus), [sif](http://flybase.bio.indiana.edu/.bin/fbidq.html?sif), [RSG7](http://flybase.bio.indiana.edu/.bin/fbidq.html?RSG7), [m4](http://flybase.bio.indiana.edu/.bin/fbidq.html?m4), [dom](http://flybase.bio.indiana.edu/.bin/fbidq.html?dom), [Tbh](http://flybase.bio.indiana.edu/.bin/fbidq.html?Tbh), [Dh31](http://flybase.bio.indiana.edu/.bin/fbidq.html?Dh31), [CG42541](http://flybase.bio.indiana.edu/.bin/fbidq.html?CG42541), [Hr51](http://flybase.bio.indiana.edu/.bin/fbidq.html?Hr51),[shi](http://flybase.bio.indiana.edu/.bin/fbidq.html?shi), [Spred](http://flybase.bio.indiana.edu/.bin/fbidq.html?Spred), [siz](http://flybase.bio.indiana.edu/.bin/fbidq.html?siz), [wg](http://flybase.bio.indiana.edu/.bin/fbidq.html?wg), [Rhp](http://flybase.bio.indiana.edu/.bin/fbidq.html?Rhp), [CG13995](http://flybase.bio.indiana.edu/.bin/fbidq.html?CG13995), [E(bx)](http://flybase.bio.indiana.edu/.bin/fbidq.html?E(bx)), [sfl](http://flybase.bio.indiana.edu/.bin/fbidq.html?sfl), [CG12728](http://flybase.bio.indiana.edu/.bin/fbidq.html?CG12728), [CG13229](http://flybase.bio.indiana.edu/.bin/fbidq.html?CG13229), [Dms](http://flybase.bio.indiana.edu/.bin/fbidq.html?Dms), [Pde11](http://flybase.bio.indiana.edu/.bin/fbidq.html?Pde11), [Utx](http://flybase.bio.indiana.edu/.bin/fbidq.html?Utx), [pburs](http://flybase.bio.indiana.edu/.bin/fbidq.html?pburs), [cos](http://flybase.bio.indiana.edu/.bin/fbidq.html?cos), [Ct](http://flybase.bio.indiana.edu/.bin/fbidq.html?Ct) |
| [mating](http://amigo.geneontology.org/cgi-bin/amigo/go.cgi?view=details&query=GO:0007618) | 29 of 1594 genes, 1.8% | 55 of 7634 genes, 0.7% | 0.00012 | 0.00% | 0.00 | [tipE](http://flybase.bio.indiana.edu/.bin/fbidq.html?tipE), [Sh](http://flybase.bio.indiana.edu/.bin/fbidq.html?Sh), [Ilp2](http://flybase.bio.indiana.edu/.bin/fbidq.html?Ilp2), [Poxn](http://flybase.bio.indiana.edu/.bin/fbidq.html?Poxn), [qtc](http://flybase.bio.indiana.edu/.bin/fbidq.html?qtc), [Pde1c](http://flybase.bio.indiana.edu/.bin/fbidq.html?Pde1c), [CaMKII](http://flybase.bio.indiana.edu/.bin/fbidq.html?CaMKII), [npf](http://flybase.bio.indiana.edu/.bin/fbidq.html?npf), [ato](http://flybase.bio.indiana.edu/.bin/fbidq.html?ato), [fne](http://flybase.bio.indiana.edu/.bin/fbidq.html?fne), [Ilp3](http://flybase.bio.indiana.edu/.bin/fbidq.html?Ilp3), [para](http://flybase.bio.indiana.edu/.bin/fbidq.html?para), [retn](http://flybase.bio.indiana.edu/.bin/fbidq.html?retn), [5-HT7](http://flybase.bio.indiana.edu/.bin/fbidq.html?5-HT7), [rut](http://flybase.bio.indiana.edu/.bin/fbidq.html?rut), [Btk29A](http://flybase.bio.indiana.edu/.bin/fbidq.html?Btk29A), [dsx](http://flybase.bio.indiana.edu/.bin/fbidq.html?dsx), [Nrg](http://flybase.bio.indiana.edu/.bin/fbidq.html?Nrg), [Ilp5](http://flybase.bio.indiana.edu/.bin/fbidq.html?Ilp5), [Ddc](http://flybase.bio.indiana.edu/.bin/fbidq.html?Ddc), [SPR](http://flybase.bio.indiana.edu/.bin/fbidq.html?SPR), [pros](http://flybase.bio.indiana.edu/.bin/fbidq.html?pros), [Tbh](http://flybase.bio.indiana.edu/.bin/fbidq.html?Tbh), [Oamb](http://flybase.bio.indiana.edu/.bin/fbidq.html?Oamb), [mle](http://flybase.bio.indiana.edu/.bin/fbidq.html?mle), [orb2](http://flybase.bio.indiana.edu/.bin/fbidq.html?orb2), [nonA](http://flybase.bio.indiana.edu/.bin/fbidq.html?nonA), [CG10251](http://flybase.bio.indiana.edu/.bin/fbidq.html?CG10251), [CASK](http://flybase.bio.indiana.edu/.bin/fbidq.html?CASK) |
| [eye development](http://amigo.geneontology.org/cgi-bin/amigo/go.cgi?view=details&query=GO:0001654) | 96 of 1594 genes, 6.0% | 292 of 7634 genes, 3.8% | 0.00013 | 0.00% | 0.00 | [danr](http://flybase.bio.indiana.edu/.bin/fbidq.html?danr), [skd](http://flybase.bio.indiana.edu/.bin/fbidq.html?skd), [rg](http://flybase.bio.indiana.edu/.bin/fbidq.html?rg), [Pten](http://flybase.bio.indiana.edu/.bin/fbidq.html?Pten), [caup](http://flybase.bio.indiana.edu/.bin/fbidq.html?caup), [tll](http://flybase.bio.indiana.edu/.bin/fbidq.html?tll), [vimar](http://flybase.bio.indiana.edu/.bin/fbidq.html?vimar), [ato](http://flybase.bio.indiana.edu/.bin/fbidq.html?ato), [rn](http://flybase.bio.indiana.edu/.bin/fbidq.html?rn), [Dip3](http://flybase.bio.indiana.edu/.bin/fbidq.html?Dip3), [SP2353](http://flybase.bio.indiana.edu/.bin/fbidq.html?SP2353), [Cbl](http://flybase.bio.indiana.edu/.bin/fbidq.html?Cbl), [br](http://flybase.bio.indiana.edu/.bin/fbidq.html?br), [sns](http://flybase.bio.indiana.edu/.bin/fbidq.html?sns), [os](http://flybase.bio.indiana.edu/.bin/fbidq.html?os), [bi](http://flybase.bio.indiana.edu/.bin/fbidq.html?bi), [chn](http://flybase.bio.indiana.edu/.bin/fbidq.html?chn), [ds](http://flybase.bio.indiana.edu/.bin/fbidq.html?ds), [lola](http://flybase.bio.indiana.edu/.bin/fbidq.html?lola), [Alk](http://flybase.bio.indiana.edu/.bin/fbidq.html?Alk), [Vang](http://flybase.bio.indiana.edu/.bin/fbidq.html?Vang), [Nrk](http://flybase.bio.indiana.edu/.bin/fbidq.html?Nrk), [sano](http://flybase.bio.indiana.edu/.bin/fbidq.html?sano), [Galpha49B](http://flybase.bio.indiana.edu/.bin/fbidq.html?Galpha49B), [jumu](http://flybase.bio.indiana.edu/.bin/fbidq.html?jumu), [Lar](http://flybase.bio.indiana.edu/.bin/fbidq.html?Lar), [scrib](http://flybase.bio.indiana.edu/.bin/fbidq.html?scrib), [fred](http://flybase.bio.indiana.edu/.bin/fbidq.html?fred), [ush](http://flybase.bio.indiana.edu/.bin/fbidq.html?ush), [Gap1](http://flybase.bio.indiana.edu/.bin/fbidq.html?Gap1), [HLHmdelta](http://flybase.bio.indiana.edu/.bin/fbidq.html?HLHmdelta), [run](http://flybase.bio.indiana.edu/.bin/fbidq.html?run), [jeb](http://flybase.bio.indiana.edu/.bin/fbidq.html?jeb), [B-H1](http://flybase.bio.indiana.edu/.bin/fbidq.html?B-H1), [alph](http://flybase.bio.indiana.edu/.bin/fbidq.html?alph), [Hs3st-B](http://flybase.bio.indiana.edu/.bin/fbidq.html?Hs3st-B), [Traf4](http://flybase.bio.indiana.edu/.bin/fbidq.html?Traf4), [stan](http://flybase.bio.indiana.edu/.bin/fbidq.html?stan), [Sos](http://flybase.bio.indiana.edu/.bin/fbidq.html?Sos), [CG34400](http://flybase.bio.indiana.edu/.bin/fbidq.html?CG34400), [phyl](http://flybase.bio.indiana.edu/.bin/fbidq.html?phyl), [ena](http://flybase.bio.indiana.edu/.bin/fbidq.html?ena),[hth](http://flybase.bio.indiana.edu/.bin/fbidq.html?hth), [cindr](http://flybase.bio.indiana.edu/.bin/fbidq.html?cindr), [vg](http://flybase.bio.indiana.edu/.bin/fbidq.html?vg), [dsh](http://flybase.bio.indiana.edu/.bin/fbidq.html?dsh), [eya](http://flybase.bio.indiana.edu/.bin/fbidq.html?eya), [14-3-3zeta](http://flybase.bio.indiana.edu/.bin/fbidq.html?14-3-3zeta), [Fas2](http://flybase.bio.indiana.edu/.bin/fbidq.html?Fas2), [fz](http://flybase.bio.indiana.edu/.bin/fbidq.html?fz), [futsch](http://flybase.bio.indiana.edu/.bin/fbidq.html?futsch), [beat-Ia](http://flybase.bio.indiana.edu/.bin/fbidq.html?beat-Ia), [ft](http://flybase.bio.indiana.edu/.bin/fbidq.html?ft), [sno](http://flybase.bio.indiana.edu/.bin/fbidq.html?sno), [ara](http://flybase.bio.indiana.edu/.bin/fbidq.html?ara), [sna](http://flybase.bio.indiana.edu/.bin/fbidq.html?sna), [mam](http://flybase.bio.indiana.edu/.bin/fbidq.html?mam), [tsh](http://flybase.bio.indiana.edu/.bin/fbidq.html?tsh), [Syn2](http://flybase.bio.indiana.edu/.bin/fbidq.html?Syn2), [HLHm5](http://flybase.bio.indiana.edu/.bin/fbidq.html?HLHm5), [neur](http://flybase.bio.indiana.edu/.bin/fbidq.html?neur), [didum](http://flybase.bio.indiana.edu/.bin/fbidq.html?didum), [Lis-1](http://flybase.bio.indiana.edu/.bin/fbidq.html?Lis-1), [dac](http://flybase.bio.indiana.edu/.bin/fbidq.html?dac), [nmo](http://flybase.bio.indiana.edu/.bin/fbidq.html?nmo), [eys](http://flybase.bio.indiana.edu/.bin/fbidq.html?eys), [oc](http://flybase.bio.indiana.edu/.bin/fbidq.html?oc), [Mob2](http://flybase.bio.indiana.edu/.bin/fbidq.html?Mob2), [pros](http://flybase.bio.indiana.edu/.bin/fbidq.html?pros), [ey](http://flybase.bio.indiana.edu/.bin/fbidq.html?ey), [ssh](http://flybase.bio.indiana.edu/.bin/fbidq.html?ssh), [Gug](http://flybase.bio.indiana.edu/.bin/fbidq.html?Gug), [klar](http://flybase.bio.indiana.edu/.bin/fbidq.html?klar), [shi](http://flybase.bio.indiana.edu/.bin/fbidq.html?shi), [rst](http://flybase.bio.indiana.edu/.bin/fbidq.html?rst), [Wnt4](http://flybase.bio.indiana.edu/.bin/fbidq.html?Wnt4), [B-H2](http://flybase.bio.indiana.edu/.bin/fbidq.html?B-H2), [grh](http://flybase.bio.indiana.edu/.bin/fbidq.html?grh), [wg](http://flybase.bio.indiana.edu/.bin/fbidq.html?wg), [toy](http://flybase.bio.indiana.edu/.bin/fbidq.html?toy), [Apc](http://flybase.bio.indiana.edu/.bin/fbidq.html?Apc), [CadN](http://flybase.bio.indiana.edu/.bin/fbidq.html?CadN), [dan](http://flybase.bio.indiana.edu/.bin/fbidq.html?dan), [sd](http://flybase.bio.indiana.edu/.bin/fbidq.html?sd), [bchs](http://flybase.bio.indiana.edu/.bin/fbidq.html?bchs), [klg](http://flybase.bio.indiana.edu/.bin/fbidq.html?klg), [pk](http://flybase.bio.indiana.edu/.bin/fbidq.html?pk),[rl](http://flybase.bio.indiana.edu/.bin/fbidq.html?rl), [Snap](http://flybase.bio.indiana.edu/.bin/fbidq.html?Snap), [cdi](http://flybase.bio.indiana.edu/.bin/fbidq.html?cdi), [klu](http://flybase.bio.indiana.edu/.bin/fbidq.html?klu), [yrt](http://flybase.bio.indiana.edu/.bin/fbidq.html?yrt), [wls](http://flybase.bio.indiana.edu/.bin/fbidq.html?wls), [HLHm7](http://flybase.bio.indiana.edu/.bin/fbidq.html?HLHm7), [HLHmgamma](http://flybase.bio.indiana.edu/.bin/fbidq.html?HLHmgamma), [unk](http://flybase.bio.indiana.edu/.bin/fbidq.html?unk) |
| [sensory perception of chemical stimulus](http://amigo.geneontology.org/cgi-bin/amigo/go.cgi?view=details&query=GO:0007606) | 25 of 1594 genes, 1.6% | 44 of 7634 genes, 0.6% | 0.00014 | 0.00% | 0.00 | [Sh](http://flybase.bio.indiana.edu/.bin/fbidq.html?Sh), [Klp68D](http://flybase.bio.indiana.edu/.bin/fbidq.html?Klp68D), [dpr20](http://flybase.bio.indiana.edu/.bin/fbidq.html?dpr20), [acj6](http://flybase.bio.indiana.edu/.bin/fbidq.html?acj6), [dpr5](http://flybase.bio.indiana.edu/.bin/fbidq.html?dpr5), [Pbprp1](http://flybase.bio.indiana.edu/.bin/fbidq.html?Pbprp1), [dpr3](http://flybase.bio.indiana.edu/.bin/fbidq.html?dpr3), [dpr8](http://flybase.bio.indiana.edu/.bin/fbidq.html?dpr8), [Klp64D](http://flybase.bio.indiana.edu/.bin/fbidq.html?Klp64D), [Galpha49B](http://flybase.bio.indiana.edu/.bin/fbidq.html?Galpha49B), [sws](http://flybase.bio.indiana.edu/.bin/fbidq.html?sws), [dpr9](http://flybase.bio.indiana.edu/.bin/fbidq.html?dpr9), [king-tubby](http://flybase.bio.indiana.edu/.bin/fbidq.html?king-tubby), [mew](http://flybase.bio.indiana.edu/.bin/fbidq.html?mew), [Tk](http://flybase.bio.indiana.edu/.bin/fbidq.html?Tk), [dpr10](http://flybase.bio.indiana.edu/.bin/fbidq.html?dpr10), [dpr19](http://flybase.bio.indiana.edu/.bin/fbidq.html?dpr19), [Kap3](http://flybase.bio.indiana.edu/.bin/fbidq.html?Kap3), [pros](http://flybase.bio.indiana.edu/.bin/fbidq.html?pros), [dpr17](http://flybase.bio.indiana.edu/.bin/fbidq.html?dpr17), [dpr14](http://flybase.bio.indiana.edu/.bin/fbidq.html?dpr14), [dpr7](http://flybase.bio.indiana.edu/.bin/fbidq.html?dpr7), [dpr15](http://flybase.bio.indiana.edu/.bin/fbidq.html?dpr15), [dpr11](http://flybase.bio.indiana.edu/.bin/fbidq.html?dpr11), [dpr18](http://flybase.bio.indiana.edu/.bin/fbidq.html?dpr18) |
| [positive regulation of macromolecule biosynthetic process](http://amigo.geneontology.org/cgi-bin/amigo/go.cgi?view=details&query=GO:0010557) | 72 of 1594 genes, 4.5% | 203 of 7634 genes, 2.7% | 0.00023 | 0.00% | 0.00 | [acj6](http://flybase.bio.indiana.edu/.bin/fbidq.html?acj6), [wor](http://flybase.bio.indiana.edu/.bin/fbidq.html?wor), [vvl](http://flybase.bio.indiana.edu/.bin/fbidq.html?vvl), [caup](http://flybase.bio.indiana.edu/.bin/fbidq.html?caup), [Camta](http://flybase.bio.indiana.edu/.bin/fbidq.html?Camta), [tll](http://flybase.bio.indiana.edu/.bin/fbidq.html?tll), [ato](http://flybase.bio.indiana.edu/.bin/fbidq.html?ato), [tna](http://flybase.bio.indiana.edu/.bin/fbidq.html?tna), [Eip93F](http://flybase.bio.indiana.edu/.bin/fbidq.html?Eip93F), [JIL-1](http://flybase.bio.indiana.edu/.bin/fbidq.html?JIL-1), [ap](http://flybase.bio.indiana.edu/.bin/fbidq.html?ap), [Iswi](http://flybase.bio.indiana.edu/.bin/fbidq.html?Iswi), [Spt6](http://flybase.bio.indiana.edu/.bin/fbidq.html?Spt6), [gcm](http://flybase.bio.indiana.edu/.bin/fbidq.html?gcm), [Dip3](http://flybase.bio.indiana.edu/.bin/fbidq.html?Dip3), [ase](http://flybase.bio.indiana.edu/.bin/fbidq.html?ase), [br](http://flybase.bio.indiana.edu/.bin/fbidq.html?br), [ovo](http://flybase.bio.indiana.edu/.bin/fbidq.html?ovo), [Pdp1](http://flybase.bio.indiana.edu/.bin/fbidq.html?Pdp1), [chn](http://flybase.bio.indiana.edu/.bin/fbidq.html?chn), [Hcf](http://flybase.bio.indiana.edu/.bin/fbidq.html?Hcf), [lola](http://flybase.bio.indiana.edu/.bin/fbidq.html?lola), [NC2alpha](http://flybase.bio.indiana.edu/.bin/fbidq.html?NC2alpha), [Taf4](http://flybase.bio.indiana.edu/.bin/fbidq.html?Taf4), [HGTX](http://flybase.bio.indiana.edu/.bin/fbidq.html?HGTX), [fd59A](http://flybase.bio.indiana.edu/.bin/fbidq.html?fd59A), [btd](http://flybase.bio.indiana.edu/.bin/fbidq.html?btd), [run](http://flybase.bio.indiana.edu/.bin/fbidq.html?run), [Antp](http://flybase.bio.indiana.edu/.bin/fbidq.html?Antp), [ATbp](http://flybase.bio.indiana.edu/.bin/fbidq.html?ATbp), [tara](http://flybase.bio.indiana.edu/.bin/fbidq.html?tara), [stau](http://flybase.bio.indiana.edu/.bin/fbidq.html?stau), [hth](http://flybase.bio.indiana.edu/.bin/fbidq.html?hth), [vg](http://flybase.bio.indiana.edu/.bin/fbidq.html?vg), [eya](http://flybase.bio.indiana.edu/.bin/fbidq.html?eya), [brm](http://flybase.bio.indiana.edu/.bin/fbidq.html?brm), [onecut](http://flybase.bio.indiana.edu/.bin/fbidq.html?onecut), [sno](http://flybase.bio.indiana.edu/.bin/fbidq.html?sno), [chm](http://flybase.bio.indiana.edu/.bin/fbidq.html?chm), [ara](http://flybase.bio.indiana.edu/.bin/fbidq.html?ara), [sna](http://flybase.bio.indiana.edu/.bin/fbidq.html?sna), [mam](http://flybase.bio.indiana.edu/.bin/fbidq.html?mam), [Taf1](http://flybase.bio.indiana.edu/.bin/fbidq.html?Taf1), [pan](http://flybase.bio.indiana.edu/.bin/fbidq.html?pan),[disco](http://flybase.bio.indiana.edu/.bin/fbidq.html?disco), [esg](http://flybase.bio.indiana.edu/.bin/fbidq.html?esg), [e(y)3](http://flybase.bio.indiana.edu/.bin/fbidq.html?e(y)3), [Brf](http://flybase.bio.indiana.edu/.bin/fbidq.html?Brf), [oc](http://flybase.bio.indiana.edu/.bin/fbidq.html?oc), [tup](http://flybase.bio.indiana.edu/.bin/fbidq.html?tup), [spt4](http://flybase.bio.indiana.edu/.bin/fbidq.html?spt4), [ey](http://flybase.bio.indiana.edu/.bin/fbidq.html?ey), [mle](http://flybase.bio.indiana.edu/.bin/fbidq.html?mle), [l(1)sc](http://flybase.bio.indiana.edu/.bin/fbidq.html?l(1)sc), [Bgb](http://flybase.bio.indiana.edu/.bin/fbidq.html?Bgb), [grh](http://flybase.bio.indiana.edu/.bin/fbidq.html?grh), [toy](http://flybase.bio.indiana.edu/.bin/fbidq.html?toy), [wg](http://flybase.bio.indiana.edu/.bin/fbidq.html?wg), [fkh](http://flybase.bio.indiana.edu/.bin/fbidq.html?fkh), [sim](http://flybase.bio.indiana.edu/.bin/fbidq.html?sim), [sd](http://flybase.bio.indiana.edu/.bin/fbidq.html?sd), [trx](http://flybase.bio.indiana.edu/.bin/fbidq.html?trx), [retn](http://flybase.bio.indiana.edu/.bin/fbidq.html?retn), [dsx](http://flybase.bio.indiana.edu/.bin/fbidq.html?dsx), [tyf](http://flybase.bio.indiana.edu/.bin/fbidq.html?tyf), [vfl](http://flybase.bio.indiana.edu/.bin/fbidq.html?vfl), [CG11347](http://flybase.bio.indiana.edu/.bin/fbidq.html?CG11347), [dalao](http://flybase.bio.indiana.edu/.bin/fbidq.html?dalao), [pygo](http://flybase.bio.indiana.edu/.bin/fbidq.html?pygo), [z](http://flybase.bio.indiana.edu/.bin/fbidq.html?z), [Lim1](http://flybase.bio.indiana.edu/.bin/fbidq.html?Lim1), [Atf-2](http://flybase.bio.indiana.edu/.bin/fbidq.html?Atf-2) |
| [organic cyclic compound biosynthetic process](http://amigo.geneontology.org/cgi-bin/amigo/go.cgi?view=details&query=GO:1901362) | 220 of 1594 genes, 13.8% | 815 of 7634 genes, 10.7% | 0.00023 | 0.00% | 0.00 | [fz2](http://flybase.bio.indiana.edu/.bin/fbidq.html?fz2), [acj6](http://flybase.bio.indiana.edu/.bin/fbidq.html?acj6), [skd](http://flybase.bio.indiana.edu/.bin/fbidq.html?skd), [jing](http://flybase.bio.indiana.edu/.bin/fbidq.html?jing), [caup](http://flybase.bio.indiana.edu/.bin/fbidq.html?caup), [Tgt](http://flybase.bio.indiana.edu/.bin/fbidq.html?Tgt), [tll](http://flybase.bio.indiana.edu/.bin/fbidq.html?tll), [Camta](http://flybase.bio.indiana.edu/.bin/fbidq.html?Camta), [ato](http://flybase.bio.indiana.edu/.bin/fbidq.html?ato), [Ets65A](http://flybase.bio.indiana.edu/.bin/fbidq.html?Ets65A), [tna](http://flybase.bio.indiana.edu/.bin/fbidq.html?tna), [Prat2](http://flybase.bio.indiana.edu/.bin/fbidq.html?Prat2), [JIL-1](http://flybase.bio.indiana.edu/.bin/fbidq.html?JIL-1), [Spt6](http://flybase.bio.indiana.edu/.bin/fbidq.html?Spt6), [Iswi](http://flybase.bio.indiana.edu/.bin/fbidq.html?Iswi), [rn](http://flybase.bio.indiana.edu/.bin/fbidq.html?rn), [ap](http://flybase.bio.indiana.edu/.bin/fbidq.html?ap), [Nf1](http://flybase.bio.indiana.edu/.bin/fbidq.html?Nf1), [br](http://flybase.bio.indiana.edu/.bin/fbidq.html?br), [Gycalpha99B](http://flybase.bio.indiana.edu/.bin/fbidq.html?Gycalpha99B), [ksr](http://flybase.bio.indiana.edu/.bin/fbidq.html?ksr), [PNUTS](http://flybase.bio.indiana.edu/.bin/fbidq.html?PNUTS), [Gyc-89Da](http://flybase.bio.indiana.edu/.bin/fbidq.html?Gyc-89Da), [bi](http://flybase.bio.indiana.edu/.bin/fbidq.html?bi), [chn](http://flybase.bio.indiana.edu/.bin/fbidq.html?chn), [Gsc](http://flybase.bio.indiana.edu/.bin/fbidq.html?Gsc), [Hcf](http://flybase.bio.indiana.edu/.bin/fbidq.html?Hcf), [lola](http://flybase.bio.indiana.edu/.bin/fbidq.html?lola), [HLH4C](http://flybase.bio.indiana.edu/.bin/fbidq.html?HLH4C), [Pcl](http://flybase.bio.indiana.edu/.bin/fbidq.html?Pcl), [chif](http://flybase.bio.indiana.edu/.bin/fbidq.html?chif), [simj](http://flybase.bio.indiana.edu/.bin/fbidq.html?simj), [Awh](http://flybase.bio.indiana.edu/.bin/fbidq.html?Awh), [tap](http://flybase.bio.indiana.edu/.bin/fbidq.html?tap), [CG13550](http://flybase.bio.indiana.edu/.bin/fbidq.html?CG13550), [NC2alpha](http://flybase.bio.indiana.edu/.bin/fbidq.html?NC2alpha), [PHDP](http://flybase.bio.indiana.edu/.bin/fbidq.html?PHDP), [bab1](http://flybase.bio.indiana.edu/.bin/fbidq.html?bab1), [Taf12L](http://flybase.bio.indiana.edu/.bin/fbidq.html?Taf12L),[CG4328](http://flybase.bio.indiana.edu/.bin/fbidq.html?CG4328), [Snoo](http://flybase.bio.indiana.edu/.bin/fbidq.html?Snoo), [l(2)NC136](http://flybase.bio.indiana.edu/.bin/fbidq.html?l(2)NC136), [Taf4](http://flybase.bio.indiana.edu/.bin/fbidq.html?Taf4), [Su(var)3-3](http://flybase.bio.indiana.edu/.bin/fbidq.html?Su(var)3-3), [HGTX](http://flybase.bio.indiana.edu/.bin/fbidq.html?HGTX), [not](http://flybase.bio.indiana.edu/.bin/fbidq.html?not), [rut](http://flybase.bio.indiana.edu/.bin/fbidq.html?rut), [nvy](http://flybase.bio.indiana.edu/.bin/fbidq.html?nvy), [nub](http://flybase.bio.indiana.edu/.bin/fbidq.html?nub), [nab](http://flybase.bio.indiana.edu/.bin/fbidq.html?nab), [Ddc](http://flybase.bio.indiana.edu/.bin/fbidq.html?Ddc), [btd](http://flybase.bio.indiana.edu/.bin/fbidq.html?btd), [fd68A](http://flybase.bio.indiana.edu/.bin/fbidq.html?fd68A), [HLHmdelta](http://flybase.bio.indiana.edu/.bin/fbidq.html?HLHmdelta), [B-H1](http://flybase.bio.indiana.edu/.bin/fbidq.html?B-H1), [Nipped-A](http://flybase.bio.indiana.edu/.bin/fbidq.html?Nipped-A), [ATbp](http://flybase.bio.indiana.edu/.bin/fbidq.html?ATbp), [Sos](http://flybase.bio.indiana.edu/.bin/fbidq.html?Sos), [Fer2](http://flybase.bio.indiana.edu/.bin/fbidq.html?Fer2), [fd102C](http://flybase.bio.indiana.edu/.bin/fbidq.html?fd102C), [vg](http://flybase.bio.indiana.edu/.bin/fbidq.html?vg), [hth](http://flybase.bio.indiana.edu/.bin/fbidq.html?hth), [Nf-YC](http://flybase.bio.indiana.edu/.bin/fbidq.html?Nf-YC), [14-3-3zeta](http://flybase.bio.indiana.edu/.bin/fbidq.html?14-3-3zeta), [eya](http://flybase.bio.indiana.edu/.bin/fbidq.html?eya), [fz](http://flybase.bio.indiana.edu/.bin/fbidq.html?fz), [brm](http://flybase.bio.indiana.edu/.bin/fbidq.html?brm), [Vsx2](http://flybase.bio.indiana.edu/.bin/fbidq.html?Vsx2), [Hey](http://flybase.bio.indiana.edu/.bin/fbidq.html?Hey), [sno](http://flybase.bio.indiana.edu/.bin/fbidq.html?sno), [Sfmbt](http://flybase.bio.indiana.edu/.bin/fbidq.html?Sfmbt), [ara](http://flybase.bio.indiana.edu/.bin/fbidq.html?ara), [mam](http://flybase.bio.indiana.edu/.bin/fbidq.html?mam), [pan](http://flybase.bio.indiana.edu/.bin/fbidq.html?pan), [Dll](http://flybase.bio.indiana.edu/.bin/fbidq.html?Dll), [disco](http://flybase.bio.indiana.edu/.bin/fbidq.html?disco), [grn](http://flybase.bio.indiana.edu/.bin/fbidq.html?grn),[HLHm5](http://flybase.bio.indiana.edu/.bin/fbidq.html?HLHm5), [D12](http://flybase.bio.indiana.edu/.bin/fbidq.html?D12), [Lis-1](http://flybase.bio.indiana.edu/.bin/fbidq.html?Lis-1), [CycE](http://flybase.bio.indiana.edu/.bin/fbidq.html?CycE), [Brf](http://flybase.bio.indiana.edu/.bin/fbidq.html?Brf), [ems](http://flybase.bio.indiana.edu/.bin/fbidq.html?ems), [pros](http://flybase.bio.indiana.edu/.bin/fbidq.html?pros), [tup](http://flybase.bio.indiana.edu/.bin/fbidq.html?tup), [pdm2](http://flybase.bio.indiana.edu/.bin/fbidq.html?pdm2), [Gug](http://flybase.bio.indiana.edu/.bin/fbidq.html?Gug), [pad](http://flybase.bio.indiana.edu/.bin/fbidq.html?pad), [rgr](http://flybase.bio.indiana.edu/.bin/fbidq.html?rgr), [Wnt4](http://flybase.bio.indiana.edu/.bin/fbidq.html?Wnt4), [CG3714](http://flybase.bio.indiana.edu/.bin/fbidq.html?CG3714), [Rx](http://flybase.bio.indiana.edu/.bin/fbidq.html?Rx), [MED1](http://flybase.bio.indiana.edu/.bin/fbidq.html?MED1), [salr](http://flybase.bio.indiana.edu/.bin/fbidq.html?salr), [wdn](http://flybase.bio.indiana.edu/.bin/fbidq.html?wdn), [sim](http://flybase.bio.indiana.edu/.bin/fbidq.html?sim), [d4](http://flybase.bio.indiana.edu/.bin/fbidq.html?d4), [dan](http://flybase.bio.indiana.edu/.bin/fbidq.html?dan), [gro](http://flybase.bio.indiana.edu/.bin/fbidq.html?gro), [nerfin-1](http://flybase.bio.indiana.edu/.bin/fbidq.html?nerfin-1), [trx](http://flybase.bio.indiana.edu/.bin/fbidq.html?trx), [Rpd3](http://flybase.bio.indiana.edu/.bin/fbidq.html?Rpd3), [dsx](http://flybase.bio.indiana.edu/.bin/fbidq.html?dsx), [MED25](http://flybase.bio.indiana.edu/.bin/fbidq.html?MED25), [Ac13E](http://flybase.bio.indiana.edu/.bin/fbidq.html?Ac13E), [Ssdp](http://flybase.bio.indiana.edu/.bin/fbidq.html?Ssdp), [vfl](http://flybase.bio.indiana.edu/.bin/fbidq.html?vfl), [RpII140](http://flybase.bio.indiana.edu/.bin/fbidq.html?RpII140), [sqz](http://flybase.bio.indiana.edu/.bin/fbidq.html?sqz), [CG32105](http://flybase.bio.indiana.edu/.bin/fbidq.html?CG32105), [Fer3](http://flybase.bio.indiana.edu/.bin/fbidq.html?Fer3), [pygo](http://flybase.bio.indiana.edu/.bin/fbidq.html?pygo), [dalao](http://flybase.bio.indiana.edu/.bin/fbidq.html?dalao), [HLHm7](http://flybase.bio.indiana.edu/.bin/fbidq.html?HLHm7), [z](http://flybase.bio.indiana.edu/.bin/fbidq.html?z),[HLHmgamma](http://flybase.bio.indiana.edu/.bin/fbidq.html?HLHmgamma), [Sox21b](http://flybase.bio.indiana.edu/.bin/fbidq.html?Sox21b), [sr](http://flybase.bio.indiana.edu/.bin/fbidq.html?sr), [Lim1](http://flybase.bio.indiana.edu/.bin/fbidq.html?Lim1), [tld](http://flybase.bio.indiana.edu/.bin/fbidq.html?tld), [danr](http://flybase.bio.indiana.edu/.bin/fbidq.html?danr), [wor](http://flybase.bio.indiana.edu/.bin/fbidq.html?wor), [vvl](http://flybase.bio.indiana.edu/.bin/fbidq.html?vvl), [wda](http://flybase.bio.indiana.edu/.bin/fbidq.html?wda), [sgg](http://flybase.bio.indiana.edu/.bin/fbidq.html?sgg), [MED14](http://flybase.bio.indiana.edu/.bin/fbidq.html?MED14), [gcm2](http://flybase.bio.indiana.edu/.bin/fbidq.html?gcm2), [Eip93F](http://flybase.bio.indiana.edu/.bin/fbidq.html?Eip93F), [gcm](http://flybase.bio.indiana.edu/.bin/fbidq.html?gcm), [seq](http://flybase.bio.indiana.edu/.bin/fbidq.html?seq), [Dip3](http://flybase.bio.indiana.edu/.bin/fbidq.html?Dip3), [Aef1](http://flybase.bio.indiana.edu/.bin/fbidq.html?Aef1), [HDAC4](http://flybase.bio.indiana.edu/.bin/fbidq.html?HDAC4), [can](http://flybase.bio.indiana.edu/.bin/fbidq.html?can), [elk](http://flybase.bio.indiana.edu/.bin/fbidq.html?elk), [phl](http://flybase.bio.indiana.edu/.bin/fbidq.html?phl), [ase](http://flybase.bio.indiana.edu/.bin/fbidq.html?ase), [CG13253](http://flybase.bio.indiana.edu/.bin/fbidq.html?CG13253), [ovo](http://flybase.bio.indiana.edu/.bin/fbidq.html?ovo), [scro](http://flybase.bio.indiana.edu/.bin/fbidq.html?scro), [Dsp1](http://flybase.bio.indiana.edu/.bin/fbidq.html?Dsp1), [CG14216](http://flybase.bio.indiana.edu/.bin/fbidq.html?CG14216), [Pdp1](http://flybase.bio.indiana.edu/.bin/fbidq.html?Pdp1), [Oli](http://flybase.bio.indiana.edu/.bin/fbidq.html?Oli), [Nelf-A](http://flybase.bio.indiana.edu/.bin/fbidq.html?Nelf-A), [GATAd](http://flybase.bio.indiana.edu/.bin/fbidq.html?GATAd), [Poxn](http://flybase.bio.indiana.edu/.bin/fbidq.html?Poxn), [CG15376](http://flybase.bio.indiana.edu/.bin/fbidq.html?CG15376), [Sin3A](http://flybase.bio.indiana.edu/.bin/fbidq.html?Sin3A), [mip120](http://flybase.bio.indiana.edu/.bin/fbidq.html?mip120), [jumu](http://flybase.bio.indiana.edu/.bin/fbidq.html?jumu),[Smox](http://flybase.bio.indiana.edu/.bin/fbidq.html?Smox), [Usp7](http://flybase.bio.indiana.edu/.bin/fbidq.html?Usp7), [ush](http://flybase.bio.indiana.edu/.bin/fbidq.html?ush), [fd59A](http://flybase.bio.indiana.edu/.bin/fbidq.html?fd59A), [Gap1](http://flybase.bio.indiana.edu/.bin/fbidq.html?Gap1), [run](http://flybase.bio.indiana.edu/.bin/fbidq.html?run), [FBX011](http://flybase.bio.indiana.edu/.bin/fbidq.html?FBX011), [brat](http://flybase.bio.indiana.edu/.bin/fbidq.html?brat), [Antp](http://flybase.bio.indiana.edu/.bin/fbidq.html?Antp), [tara](http://flybase.bio.indiana.edu/.bin/fbidq.html?tara), [Alh](http://flybase.bio.indiana.edu/.bin/fbidq.html?Alh), [Sox102F](http://flybase.bio.indiana.edu/.bin/fbidq.html?Sox102F), [egg](http://flybase.bio.indiana.edu/.bin/fbidq.html?egg), [l(3)neo38](http://flybase.bio.indiana.edu/.bin/fbidq.html?l(3)neo38), [koko](http://flybase.bio.indiana.edu/.bin/fbidq.html?koko), [Ada](http://flybase.bio.indiana.edu/.bin/fbidq.html?Ada), [Ptth](http://flybase.bio.indiana.edu/.bin/fbidq.html?Ptth), [Sp1](http://flybase.bio.indiana.edu/.bin/fbidq.html?Sp1), [Ptx1](http://flybase.bio.indiana.edu/.bin/fbidq.html?Ptx1), [ph-p](http://flybase.bio.indiana.edu/.bin/fbidq.html?ph-p), [Ubx](http://flybase.bio.indiana.edu/.bin/fbidq.html?Ubx), [onecut](http://flybase.bio.indiana.edu/.bin/fbidq.html?onecut), [CG7757](http://flybase.bio.indiana.edu/.bin/fbidq.html?CG7757), [chm](http://flybase.bio.indiana.edu/.bin/fbidq.html?chm), [sna](http://flybase.bio.indiana.edu/.bin/fbidq.html?sna), [Wnt5](http://flybase.bio.indiana.edu/.bin/fbidq.html?Wnt5), [Taf1](http://flybase.bio.indiana.edu/.bin/fbidq.html?Taf1), [hbn](http://flybase.bio.indiana.edu/.bin/fbidq.html?hbn), [MBD-like](http://flybase.bio.indiana.edu/.bin/fbidq.html?MBD-like), [tsh](http://flybase.bio.indiana.edu/.bin/fbidq.html?tsh), [esg](http://flybase.bio.indiana.edu/.bin/fbidq.html?esg), [MED16](http://flybase.bio.indiana.edu/.bin/fbidq.html?MED16), [dac](http://flybase.bio.indiana.edu/.bin/fbidq.html?dac), [e(y)3](http://flybase.bio.indiana.edu/.bin/fbidq.html?e(y)3), [CRMP](http://flybase.bio.indiana.edu/.bin/fbidq.html?CRMP), [CG9007](http://flybase.bio.indiana.edu/.bin/fbidq.html?CG9007), [oc](http://flybase.bio.indiana.edu/.bin/fbidq.html?oc), [spt4](http://flybase.bio.indiana.edu/.bin/fbidq.html?spt4),[Tbh](http://flybase.bio.indiana.edu/.bin/fbidq.html?Tbh), [CG32532](http://flybase.bio.indiana.edu/.bin/fbidq.html?CG32532), [MTA1-like](http://flybase.bio.indiana.edu/.bin/fbidq.html?MTA1-like), [gol](http://flybase.bio.indiana.edu/.bin/fbidq.html?gol), [ey](http://flybase.bio.indiana.edu/.bin/fbidq.html?ey), [mle](http://flybase.bio.indiana.edu/.bin/fbidq.html?mle), [Hr51](http://flybase.bio.indiana.edu/.bin/fbidq.html?Hr51), [Bgb](http://flybase.bio.indiana.edu/.bin/fbidq.html?Bgb), [l(1)sc](http://flybase.bio.indiana.edu/.bin/fbidq.html?l(1)sc), [B-H2](http://flybase.bio.indiana.edu/.bin/fbidq.html?B-H2), [grh](http://flybase.bio.indiana.edu/.bin/fbidq.html?grh), [Vsx1](http://flybase.bio.indiana.edu/.bin/fbidq.html?Vsx1), [wg](http://flybase.bio.indiana.edu/.bin/fbidq.html?wg), [toy](http://flybase.bio.indiana.edu/.bin/fbidq.html?toy), [fkh](http://flybase.bio.indiana.edu/.bin/fbidq.html?fkh), [Lim3](http://flybase.bio.indiana.edu/.bin/fbidq.html?Lim3), [crol](http://flybase.bio.indiana.edu/.bin/fbidq.html?crol), [sd](http://flybase.bio.indiana.edu/.bin/fbidq.html?sd), [retn](http://flybase.bio.indiana.edu/.bin/fbidq.html?retn), [en](http://flybase.bio.indiana.edu/.bin/fbidq.html?en), [fs(1)h](http://flybase.bio.indiana.edu/.bin/fbidq.html?fs(1)h), [Ac3](http://flybase.bio.indiana.edu/.bin/fbidq.html?Ac3), [E(bx)](http://flybase.bio.indiana.edu/.bin/fbidq.html?E(bx)), [rl](http://flybase.bio.indiana.edu/.bin/fbidq.html?rl), [CG11347](http://flybase.bio.indiana.edu/.bin/fbidq.html?CG11347), [tey](http://flybase.bio.indiana.edu/.bin/fbidq.html?tey), [CG11294](http://flybase.bio.indiana.edu/.bin/fbidq.html?CG11294), [hiw](http://flybase.bio.indiana.edu/.bin/fbidq.html?hiw), [cos](http://flybase.bio.indiana.edu/.bin/fbidq.html?cos), [Ct](http://flybase.bio.indiana.edu/.bin/fbidq.html?Ct), [Atf-2](http://flybase.bio.indiana.edu/.bin/fbidq.html?Atf-2) |
| [positive regulation of transcription from RNA polymerase II promoter](http://amigo.geneontology.org/cgi-bin/amigo/go.cgi?view=details&query=GO:0045944) | 53 of 1594 genes, 3.3% | 135 of 7634 genes, 1.8% | 0.00028 | 0.00% | 0.00 | [vg](http://flybase.bio.indiana.edu/.bin/fbidq.html?vg), [hth](http://flybase.bio.indiana.edu/.bin/fbidq.html?hth), [eya](http://flybase.bio.indiana.edu/.bin/fbidq.html?eya), [acj6](http://flybase.bio.indiana.edu/.bin/fbidq.html?acj6), [onecut](http://flybase.bio.indiana.edu/.bin/fbidq.html?onecut), [wor](http://flybase.bio.indiana.edu/.bin/fbidq.html?wor), [sno](http://flybase.bio.indiana.edu/.bin/fbidq.html?sno), [vvl](http://flybase.bio.indiana.edu/.bin/fbidq.html?vvl), [chm](http://flybase.bio.indiana.edu/.bin/fbidq.html?chm), [ara](http://flybase.bio.indiana.edu/.bin/fbidq.html?ara), [sna](http://flybase.bio.indiana.edu/.bin/fbidq.html?sna), [caup](http://flybase.bio.indiana.edu/.bin/fbidq.html?caup), [mam](http://flybase.bio.indiana.edu/.bin/fbidq.html?mam), [Camta](http://flybase.bio.indiana.edu/.bin/fbidq.html?Camta), [tll](http://flybase.bio.indiana.edu/.bin/fbidq.html?tll), [ato](http://flybase.bio.indiana.edu/.bin/fbidq.html?ato), [Taf1](http://flybase.bio.indiana.edu/.bin/fbidq.html?Taf1), [pan](http://flybase.bio.indiana.edu/.bin/fbidq.html?pan), [Eip93F](http://flybase.bio.indiana.edu/.bin/fbidq.html?Eip93F), [disco](http://flybase.bio.indiana.edu/.bin/fbidq.html?disco), [ap](http://flybase.bio.indiana.edu/.bin/fbidq.html?ap), [Iswi](http://flybase.bio.indiana.edu/.bin/fbidq.html?Iswi), [gcm](http://flybase.bio.indiana.edu/.bin/fbidq.html?gcm), [esg](http://flybase.bio.indiana.edu/.bin/fbidq.html?esg), [Dip3](http://flybase.bio.indiana.edu/.bin/fbidq.html?Dip3), [oc](http://flybase.bio.indiana.edu/.bin/fbidq.html?oc), [ase](http://flybase.bio.indiana.edu/.bin/fbidq.html?ase), [tup](http://flybase.bio.indiana.edu/.bin/fbidq.html?tup), [br](http://flybase.bio.indiana.edu/.bin/fbidq.html?br), [ovo](http://flybase.bio.indiana.edu/.bin/fbidq.html?ovo), [ey](http://flybase.bio.indiana.edu/.bin/fbidq.html?ey), [chn](http://flybase.bio.indiana.edu/.bin/fbidq.html?chn), [mle](http://flybase.bio.indiana.edu/.bin/fbidq.html?mle), [Bgb](http://flybase.bio.indiana.edu/.bin/fbidq.html?Bgb), [l(1)sc](http://flybase.bio.indiana.edu/.bin/fbidq.html?l(1)sc), [grh](http://flybase.bio.indiana.edu/.bin/fbidq.html?grh), [wg](http://flybase.bio.indiana.edu/.bin/fbidq.html?wg), [toy](http://flybase.bio.indiana.edu/.bin/fbidq.html?toy), [fkh](http://flybase.bio.indiana.edu/.bin/fbidq.html?fkh), [sim](http://flybase.bio.indiana.edu/.bin/fbidq.html?sim), [NC2alpha](http://flybase.bio.indiana.edu/.bin/fbidq.html?NC2alpha), [sd](http://flybase.bio.indiana.edu/.bin/fbidq.html?sd), [Taf4](http://flybase.bio.indiana.edu/.bin/fbidq.html?Taf4), [HGTX](http://flybase.bio.indiana.edu/.bin/fbidq.html?HGTX), [dsx](http://flybase.bio.indiana.edu/.bin/fbidq.html?dsx), [fd59A](http://flybase.bio.indiana.edu/.bin/fbidq.html?fd59A),[btd](http://flybase.bio.indiana.edu/.bin/fbidq.html?btd), [run](http://flybase.bio.indiana.edu/.bin/fbidq.html?run), [Antp](http://flybase.bio.indiana.edu/.bin/fbidq.html?Antp), [CG11347](http://flybase.bio.indiana.edu/.bin/fbidq.html?CG11347), [ATbp](http://flybase.bio.indiana.edu/.bin/fbidq.html?ATbp), [pygo](http://flybase.bio.indiana.edu/.bin/fbidq.html?pygo), [Atf-2](http://flybase.bio.indiana.edu/.bin/fbidq.html?Atf-2) |
| [negative regulation of nitrogen compound metabolic process](http://amigo.geneontology.org/cgi-bin/amigo/go.cgi?view=details&query=GO:0051172) | 76 of 1594 genes, 4.8% | 220 of 7634 genes, 2.9% | 0.00035 | 0.00% | 0.00 | [jing](http://flybase.bio.indiana.edu/.bin/fbidq.html?jing), [Sxl](http://flybase.bio.indiana.edu/.bin/fbidq.html?Sxl), [JIL-1](http://flybase.bio.indiana.edu/.bin/fbidq.html?JIL-1), [rn](http://flybase.bio.indiana.edu/.bin/fbidq.html?rn), [Iswi](http://flybase.bio.indiana.edu/.bin/fbidq.html?Iswi), [seq](http://flybase.bio.indiana.edu/.bin/fbidq.html?seq), [Aef1](http://flybase.bio.indiana.edu/.bin/fbidq.html?Aef1), [ovo](http://flybase.bio.indiana.edu/.bin/fbidq.html?ovo), [elav](http://flybase.bio.indiana.edu/.bin/fbidq.html?elav), [Dsp1](http://flybase.bio.indiana.edu/.bin/fbidq.html?Dsp1), [bi](http://flybase.bio.indiana.edu/.bin/fbidq.html?bi), [chn](http://flybase.bio.indiana.edu/.bin/fbidq.html?chn), [Gsc](http://flybase.bio.indiana.edu/.bin/fbidq.html?Gsc), [Nelf-A](http://flybase.bio.indiana.edu/.bin/fbidq.html?Nelf-A), [Pcl](http://flybase.bio.indiana.edu/.bin/fbidq.html?Pcl), [simj](http://flybase.bio.indiana.edu/.bin/fbidq.html?simj), [Sin3A](http://flybase.bio.indiana.edu/.bin/fbidq.html?Sin3A), [NC2alpha](http://flybase.bio.indiana.edu/.bin/fbidq.html?NC2alpha), [mip120](http://flybase.bio.indiana.edu/.bin/fbidq.html?mip120), [casp](http://flybase.bio.indiana.edu/.bin/fbidq.html?casp), [Snoo](http://flybase.bio.indiana.edu/.bin/fbidq.html?Snoo), [jumu](http://flybase.bio.indiana.edu/.bin/fbidq.html?jumu), [HGTX](http://flybase.bio.indiana.edu/.bin/fbidq.html?HGTX), [ush](http://flybase.bio.indiana.edu/.bin/fbidq.html?ush), [Usp7](http://flybase.bio.indiana.edu/.bin/fbidq.html?Usp7), [fd59A](http://flybase.bio.indiana.edu/.bin/fbidq.html?fd59A), [nab](http://flybase.bio.indiana.edu/.bin/fbidq.html?nab), [CG10336](http://flybase.bio.indiana.edu/.bin/fbidq.html?CG10336), [btd](http://flybase.bio.indiana.edu/.bin/fbidq.html?btd), [fd68A](http://flybase.bio.indiana.edu/.bin/fbidq.html?fd68A), [HLHmdelta](http://flybase.bio.indiana.edu/.bin/fbidq.html?HLHmdelta), [run](http://flybase.bio.indiana.edu/.bin/fbidq.html?run), [B-H1](http://flybase.bio.indiana.edu/.bin/fbidq.html?B-H1), [FBX011](http://flybase.bio.indiana.edu/.bin/fbidq.html?FBX011), [egg](http://flybase.bio.indiana.edu/.bin/fbidq.html?egg), [l(3)neo38](http://flybase.bio.indiana.edu/.bin/fbidq.html?l(3)neo38), [fd102C](http://flybase.bio.indiana.edu/.bin/fbidq.html?fd102C),[Vsx2](http://flybase.bio.indiana.edu/.bin/fbidq.html?Vsx2), [ph-p](http://flybase.bio.indiana.edu/.bin/fbidq.html?ph-p), [Ubx](http://flybase.bio.indiana.edu/.bin/fbidq.html?Ubx), [CG7757](http://flybase.bio.indiana.edu/.bin/fbidq.html?CG7757), [chm](http://flybase.bio.indiana.edu/.bin/fbidq.html?chm), [sna](http://flybase.bio.indiana.edu/.bin/fbidq.html?sna), [Sfmbt](http://flybase.bio.indiana.edu/.bin/fbidq.html?Sfmbt), [Wnt5](http://flybase.bio.indiana.edu/.bin/fbidq.html?Wnt5), [pan](http://flybase.bio.indiana.edu/.bin/fbidq.html?pan), [MBD-like](http://flybase.bio.indiana.edu/.bin/fbidq.html?MBD-like), [tsh](http://flybase.bio.indiana.edu/.bin/fbidq.html?tsh), [HLHm5](http://flybase.bio.indiana.edu/.bin/fbidq.html?HLHm5), [esg](http://flybase.bio.indiana.edu/.bin/fbidq.html?esg), [e(y)3](http://flybase.bio.indiana.edu/.bin/fbidq.html?e(y)3), [oc](http://flybase.bio.indiana.edu/.bin/fbidq.html?oc), [pros](http://flybase.bio.indiana.edu/.bin/fbidq.html?pros), [Psi](http://flybase.bio.indiana.edu/.bin/fbidq.html?Psi), [Gug](http://flybase.bio.indiana.edu/.bin/fbidq.html?Gug), [pad](http://flybase.bio.indiana.edu/.bin/fbidq.html?pad), [spel1](http://flybase.bio.indiana.edu/.bin/fbidq.html?spel1), [Wnt4](http://flybase.bio.indiana.edu/.bin/fbidq.html?Wnt4), [B-H2](http://flybase.bio.indiana.edu/.bin/fbidq.html?B-H2), [wg](http://flybase.bio.indiana.edu/.bin/fbidq.html?wg), [Vsx1](http://flybase.bio.indiana.edu/.bin/fbidq.html?Vsx1), [fkh](http://flybase.bio.indiana.edu/.bin/fbidq.html?fkh), [gro](http://flybase.bio.indiana.edu/.bin/fbidq.html?gro), [crol](http://flybase.bio.indiana.edu/.bin/fbidq.html?crol), [Rpd3](http://flybase.bio.indiana.edu/.bin/fbidq.html?Rpd3), [retn](http://flybase.bio.indiana.edu/.bin/fbidq.html?retn), [fs(1)h](http://flybase.bio.indiana.edu/.bin/fbidq.html?fs(1)h), [en](http://flybase.bio.indiana.edu/.bin/fbidq.html?en), [dsx](http://flybase.bio.indiana.edu/.bin/fbidq.html?dsx), [tey](http://flybase.bio.indiana.edu/.bin/fbidq.html?tey), [HLHmgamma](http://flybase.bio.indiana.edu/.bin/fbidq.html?HLHmgamma), [z](http://flybase.bio.indiana.edu/.bin/fbidq.html?z), [HLHm7](http://flybase.bio.indiana.edu/.bin/fbidq.html?HLHm7), [Lim1](http://flybase.bio.indiana.edu/.bin/fbidq.html?Lim1), [dnr1](http://flybase.bio.indiana.edu/.bin/fbidq.html?dnr1), [Ct](http://flybase.bio.indiana.edu/.bin/fbidq.html?Ct) |
| [negative regulation of transcription from RNA polymerase II promoter](http://amigo.geneontology.org/cgi-bin/amigo/go.cgi?view=details&query=GO:0000122) | 44 of 1594 genes, 2.8% | 105 of 7634 genes, 1.4% | 0.00037 | 0.00% | 0.00 | [Ubx](http://flybase.bio.indiana.edu/.bin/fbidq.html?Ubx), [Vsx2](http://flybase.bio.indiana.edu/.bin/fbidq.html?Vsx2), [jing](http://flybase.bio.indiana.edu/.bin/fbidq.html?jing), [sna](http://flybase.bio.indiana.edu/.bin/fbidq.html?sna), [Wnt5](http://flybase.bio.indiana.edu/.bin/fbidq.html?Wnt5), [tsh](http://flybase.bio.indiana.edu/.bin/fbidq.html?tsh), [rn](http://flybase.bio.indiana.edu/.bin/fbidq.html?rn), [seq](http://flybase.bio.indiana.edu/.bin/fbidq.html?seq), [esg](http://flybase.bio.indiana.edu/.bin/fbidq.html?esg), [Aef1](http://flybase.bio.indiana.edu/.bin/fbidq.html?Aef1), [oc](http://flybase.bio.indiana.edu/.bin/fbidq.html?oc), [pros](http://flybase.bio.indiana.edu/.bin/fbidq.html?pros), [ovo](http://flybase.bio.indiana.edu/.bin/fbidq.html?ovo), [Dsp1](http://flybase.bio.indiana.edu/.bin/fbidq.html?Dsp1), [pad](http://flybase.bio.indiana.edu/.bin/fbidq.html?pad), [bi](http://flybase.bio.indiana.edu/.bin/fbidq.html?bi), [chn](http://flybase.bio.indiana.edu/.bin/fbidq.html?chn), [Nelf-A](http://flybase.bio.indiana.edu/.bin/fbidq.html?Nelf-A), [Wnt4](http://flybase.bio.indiana.edu/.bin/fbidq.html?Wnt4), [B-H2](http://flybase.bio.indiana.edu/.bin/fbidq.html?B-H2), [Vsx1](http://flybase.bio.indiana.edu/.bin/fbidq.html?Vsx1), [simj](http://flybase.bio.indiana.edu/.bin/fbidq.html?simj), [wg](http://flybase.bio.indiana.edu/.bin/fbidq.html?wg), [fkh](http://flybase.bio.indiana.edu/.bin/fbidq.html?fkh), [NC2alpha](http://flybase.bio.indiana.edu/.bin/fbidq.html?NC2alpha), [mip120](http://flybase.bio.indiana.edu/.bin/fbidq.html?mip120), [gro](http://flybase.bio.indiana.edu/.bin/fbidq.html?gro), [Snoo](http://flybase.bio.indiana.edu/.bin/fbidq.html?Snoo), [jumu](http://flybase.bio.indiana.edu/.bin/fbidq.html?jumu), [Rpd3](http://flybase.bio.indiana.edu/.bin/fbidq.html?Rpd3), [retn](http://flybase.bio.indiana.edu/.bin/fbidq.html?retn), [HGTX](http://flybase.bio.indiana.edu/.bin/fbidq.html?HGTX), [ush](http://flybase.bio.indiana.edu/.bin/fbidq.html?ush), [en](http://flybase.bio.indiana.edu/.bin/fbidq.html?en), [dsx](http://flybase.bio.indiana.edu/.bin/fbidq.html?dsx), [fd59A](http://flybase.bio.indiana.edu/.bin/fbidq.html?fd59A), [btd](http://flybase.bio.indiana.edu/.bin/fbidq.html?btd), [fd68A](http://flybase.bio.indiana.edu/.bin/fbidq.html?fd68A), [HLHmdelta](http://flybase.bio.indiana.edu/.bin/fbidq.html?HLHmdelta), [B-H1](http://flybase.bio.indiana.edu/.bin/fbidq.html?B-H1), [HLHm7](http://flybase.bio.indiana.edu/.bin/fbidq.html?HLHm7),[HLHmgamma](http://flybase.bio.indiana.edu/.bin/fbidq.html?HLHmgamma), [fd102C](http://flybase.bio.indiana.edu/.bin/fbidq.html?fd102C), [Ct](http://flybase.bio.indiana.edu/.bin/fbidq.html?Ct) |
| [neuroblast differentiation](http://amigo.geneontology.org/cgi-bin/amigo/go.cgi?view=details&query=GO:0014016) | 21 of 1594 genes, 1.3% | 35 of 7634 genes, 0.5% | 0.00046 | 0.00% | 0.00 | [numb](http://flybase.bio.indiana.edu/.bin/fbidq.html?numb), [l(1)sc](http://flybase.bio.indiana.edu/.bin/fbidq.html?l(1)sc), [wg](http://flybase.bio.indiana.edu/.bin/fbidq.html?wg), [mira](http://flybase.bio.indiana.edu/.bin/fbidq.html?mira), [jumu](http://flybase.bio.indiana.edu/.bin/fbidq.html?jumu), [en](http://flybase.bio.indiana.edu/.bin/fbidq.html?en), [nab](http://flybase.bio.indiana.edu/.bin/fbidq.html?nab), [nub](http://flybase.bio.indiana.edu/.bin/fbidq.html?nub), [ems](http://flybase.bio.indiana.edu/.bin/fbidq.html?ems), [insc](http://flybase.bio.indiana.edu/.bin/fbidq.html?insc), [CycE](http://flybase.bio.indiana.edu/.bin/fbidq.html?CycE), [run](http://flybase.bio.indiana.edu/.bin/fbidq.html?run), [brat](http://flybase.bio.indiana.edu/.bin/fbidq.html?brat), [klu](http://flybase.bio.indiana.edu/.bin/fbidq.html?klu), [sqz](http://flybase.bio.indiana.edu/.bin/fbidq.html?sqz), [Antp](http://flybase.bio.indiana.edu/.bin/fbidq.html?Antp), [pros](http://flybase.bio.indiana.edu/.bin/fbidq.html?pros), [ase](http://flybase.bio.indiana.edu/.bin/fbidq.html?ase), [stau](http://flybase.bio.indiana.edu/.bin/fbidq.html?stau), [pdm2](http://flybase.bio.indiana.edu/.bin/fbidq.html?pdm2), [nkd](http://flybase.bio.indiana.edu/.bin/fbidq.html?nkd) |
| [courtship behavior](http://amigo.geneontology.org/cgi-bin/amigo/go.cgi?view=details&query=GO:0007619) | 23 of 1594 genes, 1.4% | 41 of 7634 genes, 0.5% | 0.00067 | 0.00% | 0.00 | [tipE](http://flybase.bio.indiana.edu/.bin/fbidq.html?tipE), [Sh](http://flybase.bio.indiana.edu/.bin/fbidq.html?Sh), [Poxn](http://flybase.bio.indiana.edu/.bin/fbidq.html?Poxn), [qtc](http://flybase.bio.indiana.edu/.bin/fbidq.html?qtc), [CaMKII](http://flybase.bio.indiana.edu/.bin/fbidq.html?CaMKII), [npf](http://flybase.bio.indiana.edu/.bin/fbidq.html?npf), [ato](http://flybase.bio.indiana.edu/.bin/fbidq.html?ato), [fne](http://flybase.bio.indiana.edu/.bin/fbidq.html?fne), [para](http://flybase.bio.indiana.edu/.bin/fbidq.html?para), [retn](http://flybase.bio.indiana.edu/.bin/fbidq.html?retn), [5-HT7](http://flybase.bio.indiana.edu/.bin/fbidq.html?5-HT7), [Btk29A](http://flybase.bio.indiana.edu/.bin/fbidq.html?Btk29A), [rut](http://flybase.bio.indiana.edu/.bin/fbidq.html?rut), [dsx](http://flybase.bio.indiana.edu/.bin/fbidq.html?dsx), [Nrg](http://flybase.bio.indiana.edu/.bin/fbidq.html?Nrg), [Ddc](http://flybase.bio.indiana.edu/.bin/fbidq.html?Ddc), [pros](http://flybase.bio.indiana.edu/.bin/fbidq.html?pros), [Tbh](http://flybase.bio.indiana.edu/.bin/fbidq.html?Tbh), [Oamb](http://flybase.bio.indiana.edu/.bin/fbidq.html?Oamb), [mle](http://flybase.bio.indiana.edu/.bin/fbidq.html?mle), [CASK](http://flybase.bio.indiana.edu/.bin/fbidq.html?CASK), [nonA](http://flybase.bio.indiana.edu/.bin/fbidq.html?nonA), [orb2](http://flybase.bio.indiana.edu/.bin/fbidq.html?orb2) |
| [imaginal disc morphogenesis](http://amigo.geneontology.org/cgi-bin/amigo/go.cgi?view=details&query=GO:0007560) | 110 of 1594 genes, 6.9% | 357 of 7634 genes, 4.7% | 0.00067 | 0.00% | 0.00 | [fz2](http://flybase.bio.indiana.edu/.bin/fbidq.html?fz2), [tld](http://flybase.bio.indiana.edu/.bin/fbidq.html?tld), [jar](http://flybase.bio.indiana.edu/.bin/fbidq.html?jar), [tow](http://flybase.bio.indiana.edu/.bin/fbidq.html?tow), [skd](http://flybase.bio.indiana.edu/.bin/fbidq.html?skd), [jing](http://flybase.bio.indiana.edu/.bin/fbidq.html?jing), [caup](http://flybase.bio.indiana.edu/.bin/fbidq.html?caup), [betaInt-nu](http://flybase.bio.indiana.edu/.bin/fbidq.html?betaInt-nu), [ato](http://flybase.bio.indiana.edu/.bin/fbidq.html?ato), [sgg](http://flybase.bio.indiana.edu/.bin/fbidq.html?sgg), [Nmdar1](http://flybase.bio.indiana.edu/.bin/fbidq.html?Nmdar1), [bowl](http://flybase.bio.indiana.edu/.bin/fbidq.html?bowl), [rn](http://flybase.bio.indiana.edu/.bin/fbidq.html?rn), [ap](http://flybase.bio.indiana.edu/.bin/fbidq.html?ap), [Dg](http://flybase.bio.indiana.edu/.bin/fbidq.html?Dg), [CG14478](http://flybase.bio.indiana.edu/.bin/fbidq.html?CG14478), [phl](http://flybase.bio.indiana.edu/.bin/fbidq.html?phl), [Cbl](http://flybase.bio.indiana.edu/.bin/fbidq.html?Cbl), [ovo](http://flybase.bio.indiana.edu/.bin/fbidq.html?ovo), [ksr](http://flybase.bio.indiana.edu/.bin/fbidq.html?ksr), [os](http://flybase.bio.indiana.edu/.bin/fbidq.html?os), [Axn](http://flybase.bio.indiana.edu/.bin/fbidq.html?Axn), [tok](http://flybase.bio.indiana.edu/.bin/fbidq.html?tok), [bi](http://flybase.bio.indiana.edu/.bin/fbidq.html?bi), [ds](http://flybase.bio.indiana.edu/.bin/fbidq.html?ds), [CG15312](http://flybase.bio.indiana.edu/.bin/fbidq.html?CG15312), [Poxn](http://flybase.bio.indiana.edu/.bin/fbidq.html?Poxn), [ewg](http://flybase.bio.indiana.edu/.bin/fbidq.html?ewg), [pot](http://flybase.bio.indiana.edu/.bin/fbidq.html?pot), [bab1](http://flybase.bio.indiana.edu/.bin/fbidq.html?bab1), [jumu](http://flybase.bio.indiana.edu/.bin/fbidq.html?jumu), [Smox](http://flybase.bio.indiana.edu/.bin/fbidq.html?Smox), [scrib](http://flybase.bio.indiana.edu/.bin/fbidq.html?scrib), [Su(var)3-3](http://flybase.bio.indiana.edu/.bin/fbidq.html?Su(var)3-3), [MYPT-75D](http://flybase.bio.indiana.edu/.bin/fbidq.html?MYPT-75D), [Nrg](http://flybase.bio.indiana.edu/.bin/fbidq.html?Nrg), [Gap1](http://flybase.bio.indiana.edu/.bin/fbidq.html?Gap1), [btd](http://flybase.bio.indiana.edu/.bin/fbidq.html?btd), [Mmp2](http://flybase.bio.indiana.edu/.bin/fbidq.html?Mmp2), [B-H1](http://flybase.bio.indiana.edu/.bin/fbidq.html?B-H1), [alph](http://flybase.bio.indiana.edu/.bin/fbidq.html?alph),[Hs3st-B](http://flybase.bio.indiana.edu/.bin/fbidq.html?Hs3st-B), [Traf4](http://flybase.bio.indiana.edu/.bin/fbidq.html?Traf4), [l(3)neo38](http://flybase.bio.indiana.edu/.bin/fbidq.html?l(3)neo38), [Sos](http://flybase.bio.indiana.edu/.bin/fbidq.html?Sos), [Sp1](http://flybase.bio.indiana.edu/.bin/fbidq.html?Sp1), [hth](http://flybase.bio.indiana.edu/.bin/fbidq.html?hth), [vg](http://flybase.bio.indiana.edu/.bin/fbidq.html?vg), [dsh](http://flybase.bio.indiana.edu/.bin/fbidq.html?dsh), [Fas2](http://flybase.bio.indiana.edu/.bin/fbidq.html?Fas2), [eya](http://flybase.bio.indiana.edu/.bin/fbidq.html?eya), [fz](http://flybase.bio.indiana.edu/.bin/fbidq.html?fz), [CG8557](http://flybase.bio.indiana.edu/.bin/fbidq.html?CG8557), [bsk](http://flybase.bio.indiana.edu/.bin/fbidq.html?bsk), [brm](http://flybase.bio.indiana.edu/.bin/fbidq.html?brm), [Ubx](http://flybase.bio.indiana.edu/.bin/fbidq.html?Ubx), [ft](http://flybase.bio.indiana.edu/.bin/fbidq.html?ft), [CG42268](http://flybase.bio.indiana.edu/.bin/fbidq.html?CG42268), [lft](http://flybase.bio.indiana.edu/.bin/fbidq.html?lft), [sno](http://flybase.bio.indiana.edu/.bin/fbidq.html?sno), [ara](http://flybase.bio.indiana.edu/.bin/fbidq.html?ara), [mam](http://flybase.bio.indiana.edu/.bin/fbidq.html?mam), [pan](http://flybase.bio.indiana.edu/.bin/fbidq.html?pan), [Dll](http://flybase.bio.indiana.edu/.bin/fbidq.html?Dll), [tsh](http://flybase.bio.indiana.edu/.bin/fbidq.html?tsh), [mew](http://flybase.bio.indiana.edu/.bin/fbidq.html?mew), [neur](http://flybase.bio.indiana.edu/.bin/fbidq.html?neur), [esg](http://flybase.bio.indiana.edu/.bin/fbidq.html?esg), [e(y)3](http://flybase.bio.indiana.edu/.bin/fbidq.html?e(y)3), [dac](http://flybase.bio.indiana.edu/.bin/fbidq.html?dac), [dap](http://flybase.bio.indiana.edu/.bin/fbidq.html?dap), [nmo](http://flybase.bio.indiana.edu/.bin/fbidq.html?nmo), [oc](http://flybase.bio.indiana.edu/.bin/fbidq.html?oc), [Bx](http://flybase.bio.indiana.edu/.bin/fbidq.html?Bx), [CG32149](http://flybase.bio.indiana.edu/.bin/fbidq.html?CG32149), [trio](http://flybase.bio.indiana.edu/.bin/fbidq.html?trio), [ey](http://flybase.bio.indiana.edu/.bin/fbidq.html?ey), [ed](http://flybase.bio.indiana.edu/.bin/fbidq.html?ed), [Gug](http://flybase.bio.indiana.edu/.bin/fbidq.html?Gug), [CG33275](http://flybase.bio.indiana.edu/.bin/fbidq.html?CG33275), [Hr51](http://flybase.bio.indiana.edu/.bin/fbidq.html?Hr51), [ImpE2](http://flybase.bio.indiana.edu/.bin/fbidq.html?ImpE2), [siz](http://flybase.bio.indiana.edu/.bin/fbidq.html?siz),[CG6398](http://flybase.bio.indiana.edu/.bin/fbidq.html?CG6398), [Wnt4](http://flybase.bio.indiana.edu/.bin/fbidq.html?Wnt4), [B-H2](http://flybase.bio.indiana.edu/.bin/fbidq.html?B-H2), [wg](http://flybase.bio.indiana.edu/.bin/fbidq.html?wg), [salr](http://flybase.bio.indiana.edu/.bin/fbidq.html?salr), [Glut4EF](http://flybase.bio.indiana.edu/.bin/fbidq.html?Glut4EF), [crol](http://flybase.bio.indiana.edu/.bin/fbidq.html?crol), [sd](http://flybase.bio.indiana.edu/.bin/fbidq.html?sd), [Btk29A](http://flybase.bio.indiana.edu/.bin/fbidq.html?Btk29A), [fs(1)h](http://flybase.bio.indiana.edu/.bin/fbidq.html?fs(1)h), [pk](http://flybase.bio.indiana.edu/.bin/fbidq.html?pk), [dsx](http://flybase.bio.indiana.edu/.bin/fbidq.html?dsx), [sfl](http://flybase.bio.indiana.edu/.bin/fbidq.html?sfl), [rl](http://flybase.bio.indiana.edu/.bin/fbidq.html?rl), [chinmo](http://flybase.bio.indiana.edu/.bin/fbidq.html?chinmo), [Sip1](http://flybase.bio.indiana.edu/.bin/fbidq.html?Sip1), [Snap](http://flybase.bio.indiana.edu/.bin/fbidq.html?Snap), [trn](http://flybase.bio.indiana.edu/.bin/fbidq.html?trn), [aret](http://flybase.bio.indiana.edu/.bin/fbidq.html?aret), [wls](http://flybase.bio.indiana.edu/.bin/fbidq.html?wls), [pygo](http://flybase.bio.indiana.edu/.bin/fbidq.html?pygo), [spag](http://flybase.bio.indiana.edu/.bin/fbidq.html?spag), [unk](http://flybase.bio.indiana.edu/.bin/fbidq.html?unk), [Lim1](http://flybase.bio.indiana.edu/.bin/fbidq.html?Lim1), [cos](http://flybase.bio.indiana.edu/.bin/fbidq.html?cos), [Ct](http://flybase.bio.indiana.edu/.bin/fbidq.html?Ct), [dnr1](http://flybase.bio.indiana.edu/.bin/fbidq.html?dnr1) |
| [post-embryonic organ morphogenesis](http://amigo.geneontology.org/cgi-bin/amigo/go.cgi?view=details&query=GO:0048563) | 110 of 1594 genes, 6.9% | 357 of 7634 genes, 4.7% | 0.00067 | 0.00% | 0.00 | [fz2](http://flybase.bio.indiana.edu/.bin/fbidq.html?fz2), [tld](http://flybase.bio.indiana.edu/.bin/fbidq.html?tld), [jar](http://flybase.bio.indiana.edu/.bin/fbidq.html?jar), [tow](http://flybase.bio.indiana.edu/.bin/fbidq.html?tow), [skd](http://flybase.bio.indiana.edu/.bin/fbidq.html?skd), [jing](http://flybase.bio.indiana.edu/.bin/fbidq.html?jing), [caup](http://flybase.bio.indiana.edu/.bin/fbidq.html?caup), [betaInt-nu](http://flybase.bio.indiana.edu/.bin/fbidq.html?betaInt-nu), [ato](http://flybase.bio.indiana.edu/.bin/fbidq.html?ato), [sgg](http://flybase.bio.indiana.edu/.bin/fbidq.html?sgg), [Nmdar1](http://flybase.bio.indiana.edu/.bin/fbidq.html?Nmdar1), [bowl](http://flybase.bio.indiana.edu/.bin/fbidq.html?bowl), [rn](http://flybase.bio.indiana.edu/.bin/fbidq.html?rn), [ap](http://flybase.bio.indiana.edu/.bin/fbidq.html?ap), [Dg](http://flybase.bio.indiana.edu/.bin/fbidq.html?Dg), [CG14478](http://flybase.bio.indiana.edu/.bin/fbidq.html?CG14478), [phl](http://flybase.bio.indiana.edu/.bin/fbidq.html?phl), [Cbl](http://flybase.bio.indiana.edu/.bin/fbidq.html?Cbl), [ovo](http://flybase.bio.indiana.edu/.bin/fbidq.html?ovo), [ksr](http://flybase.bio.indiana.edu/.bin/fbidq.html?ksr), [os](http://flybase.bio.indiana.edu/.bin/fbidq.html?os), [Axn](http://flybase.bio.indiana.edu/.bin/fbidq.html?Axn), [tok](http://flybase.bio.indiana.edu/.bin/fbidq.html?tok), [bi](http://flybase.bio.indiana.edu/.bin/fbidq.html?bi), [ds](http://flybase.bio.indiana.edu/.bin/fbidq.html?ds), [CG15312](http://flybase.bio.indiana.edu/.bin/fbidq.html?CG15312), [Poxn](http://flybase.bio.indiana.edu/.bin/fbidq.html?Poxn), [ewg](http://flybase.bio.indiana.edu/.bin/fbidq.html?ewg), [pot](http://flybase.bio.indiana.edu/.bin/fbidq.html?pot), [bab1](http://flybase.bio.indiana.edu/.bin/fbidq.html?bab1), [jumu](http://flybase.bio.indiana.edu/.bin/fbidq.html?jumu), [Smox](http://flybase.bio.indiana.edu/.bin/fbidq.html?Smox), [scrib](http://flybase.bio.indiana.edu/.bin/fbidq.html?scrib), [Su(var)3-3](http://flybase.bio.indiana.edu/.bin/fbidq.html?Su(var)3-3), [MYPT-75D](http://flybase.bio.indiana.edu/.bin/fbidq.html?MYPT-75D), [Nrg](http://flybase.bio.indiana.edu/.bin/fbidq.html?Nrg), [Gap1](http://flybase.bio.indiana.edu/.bin/fbidq.html?Gap1), [btd](http://flybase.bio.indiana.edu/.bin/fbidq.html?btd), [Mmp2](http://flybase.bio.indiana.edu/.bin/fbidq.html?Mmp2), [B-H1](http://flybase.bio.indiana.edu/.bin/fbidq.html?B-H1), [alph](http://flybase.bio.indiana.edu/.bin/fbidq.html?alph),[Hs3st-B](http://flybase.bio.indiana.edu/.bin/fbidq.html?Hs3st-B), [Traf4](http://flybase.bio.indiana.edu/.bin/fbidq.html?Traf4), [l(3)neo38](http://flybase.bio.indiana.edu/.bin/fbidq.html?l(3)neo38), [Sos](http://flybase.bio.indiana.edu/.bin/fbidq.html?Sos), [Sp1](http://flybase.bio.indiana.edu/.bin/fbidq.html?Sp1), [hth](http://flybase.bio.indiana.edu/.bin/fbidq.html?hth), [vg](http://flybase.bio.indiana.edu/.bin/fbidq.html?vg), [dsh](http://flybase.bio.indiana.edu/.bin/fbidq.html?dsh), [Fas2](http://flybase.bio.indiana.edu/.bin/fbidq.html?Fas2), [eya](http://flybase.bio.indiana.edu/.bin/fbidq.html?eya), [fz](http://flybase.bio.indiana.edu/.bin/fbidq.html?fz), [CG8557](http://flybase.bio.indiana.edu/.bin/fbidq.html?CG8557), [bsk](http://flybase.bio.indiana.edu/.bin/fbidq.html?bsk), [brm](http://flybase.bio.indiana.edu/.bin/fbidq.html?brm), [Ubx](http://flybase.bio.indiana.edu/.bin/fbidq.html?Ubx), [ft](http://flybase.bio.indiana.edu/.bin/fbidq.html?ft), [CG42268](http://flybase.bio.indiana.edu/.bin/fbidq.html?CG42268), [lft](http://flybase.bio.indiana.edu/.bin/fbidq.html?lft), [sno](http://flybase.bio.indiana.edu/.bin/fbidq.html?sno), [ara](http://flybase.bio.indiana.edu/.bin/fbidq.html?ara), [mam](http://flybase.bio.indiana.edu/.bin/fbidq.html?mam), [pan](http://flybase.bio.indiana.edu/.bin/fbidq.html?pan), [Dll](http://flybase.bio.indiana.edu/.bin/fbidq.html?Dll), [tsh](http://flybase.bio.indiana.edu/.bin/fbidq.html?tsh), [mew](http://flybase.bio.indiana.edu/.bin/fbidq.html?mew), [neur](http://flybase.bio.indiana.edu/.bin/fbidq.html?neur), [esg](http://flybase.bio.indiana.edu/.bin/fbidq.html?esg), [e(y)3](http://flybase.bio.indiana.edu/.bin/fbidq.html?e(y)3), [dac](http://flybase.bio.indiana.edu/.bin/fbidq.html?dac), [dap](http://flybase.bio.indiana.edu/.bin/fbidq.html?dap), [nmo](http://flybase.bio.indiana.edu/.bin/fbidq.html?nmo), [oc](http://flybase.bio.indiana.edu/.bin/fbidq.html?oc), [Bx](http://flybase.bio.indiana.edu/.bin/fbidq.html?Bx), [CG32149](http://flybase.bio.indiana.edu/.bin/fbidq.html?CG32149), [trio](http://flybase.bio.indiana.edu/.bin/fbidq.html?trio), [ey](http://flybase.bio.indiana.edu/.bin/fbidq.html?ey), [ed](http://flybase.bio.indiana.edu/.bin/fbidq.html?ed), [Gug](http://flybase.bio.indiana.edu/.bin/fbidq.html?Gug), [CG33275](http://flybase.bio.indiana.edu/.bin/fbidq.html?CG33275), [Hr51](http://flybase.bio.indiana.edu/.bin/fbidq.html?Hr51), [ImpE2](http://flybase.bio.indiana.edu/.bin/fbidq.html?ImpE2), [siz](http://flybase.bio.indiana.edu/.bin/fbidq.html?siz),[CG6398](http://flybase.bio.indiana.edu/.bin/fbidq.html?CG6398), [Wnt4](http://flybase.bio.indiana.edu/.bin/fbidq.html?Wnt4), [B-H2](http://flybase.bio.indiana.edu/.bin/fbidq.html?B-H2), [wg](http://flybase.bio.indiana.edu/.bin/fbidq.html?wg), [salr](http://flybase.bio.indiana.edu/.bin/fbidq.html?salr), [Glut4EF](http://flybase.bio.indiana.edu/.bin/fbidq.html?Glut4EF), [crol](http://flybase.bio.indiana.edu/.bin/fbidq.html?crol), [sd](http://flybase.bio.indiana.edu/.bin/fbidq.html?sd), [Btk29A](http://flybase.bio.indiana.edu/.bin/fbidq.html?Btk29A), [fs(1)h](http://flybase.bio.indiana.edu/.bin/fbidq.html?fs(1)h), [pk](http://flybase.bio.indiana.edu/.bin/fbidq.html?pk), [dsx](http://flybase.bio.indiana.edu/.bin/fbidq.html?dsx), [sfl](http://flybase.bio.indiana.edu/.bin/fbidq.html?sfl), [rl](http://flybase.bio.indiana.edu/.bin/fbidq.html?rl), [chinmo](http://flybase.bio.indiana.edu/.bin/fbidq.html?chinmo), [Sip1](http://flybase.bio.indiana.edu/.bin/fbidq.html?Sip1), [Snap](http://flybase.bio.indiana.edu/.bin/fbidq.html?Snap), [trn](http://flybase.bio.indiana.edu/.bin/fbidq.html?trn), [aret](http://flybase.bio.indiana.edu/.bin/fbidq.html?aret), [wls](http://flybase.bio.indiana.edu/.bin/fbidq.html?wls), [pygo](http://flybase.bio.indiana.edu/.bin/fbidq.html?pygo), [spag](http://flybase.bio.indiana.edu/.bin/fbidq.html?spag), [unk](http://flybase.bio.indiana.edu/.bin/fbidq.html?unk), [Lim1](http://flybase.bio.indiana.edu/.bin/fbidq.html?Lim1), [cos](http://flybase.bio.indiana.edu/.bin/fbidq.html?cos), [Ct](http://flybase.bio.indiana.edu/.bin/fbidq.html?Ct), [dnr1](http://flybase.bio.indiana.edu/.bin/fbidq.html?dnr1) |
| [negative regulation of RNA metabolic process](http://amigo.geneontology.org/cgi-bin/amigo/go.cgi?view=details&query=GO:0051253) | 72 of 1594 genes, 4.5% | 208 of 7634 genes, 2.7% | 0.00069 | 0.00% | 0.00 | [jing](http://flybase.bio.indiana.edu/.bin/fbidq.html?jing), [Sxl](http://flybase.bio.indiana.edu/.bin/fbidq.html?Sxl), [JIL-1](http://flybase.bio.indiana.edu/.bin/fbidq.html?JIL-1), [rn](http://flybase.bio.indiana.edu/.bin/fbidq.html?rn), [Iswi](http://flybase.bio.indiana.edu/.bin/fbidq.html?Iswi), [seq](http://flybase.bio.indiana.edu/.bin/fbidq.html?seq), [Aef1](http://flybase.bio.indiana.edu/.bin/fbidq.html?Aef1), [ovo](http://flybase.bio.indiana.edu/.bin/fbidq.html?ovo), [elav](http://flybase.bio.indiana.edu/.bin/fbidq.html?elav), [Dsp1](http://flybase.bio.indiana.edu/.bin/fbidq.html?Dsp1), [bi](http://flybase.bio.indiana.edu/.bin/fbidq.html?bi), [chn](http://flybase.bio.indiana.edu/.bin/fbidq.html?chn), [Gsc](http://flybase.bio.indiana.edu/.bin/fbidq.html?Gsc), [Nelf-A](http://flybase.bio.indiana.edu/.bin/fbidq.html?Nelf-A), [Pcl](http://flybase.bio.indiana.edu/.bin/fbidq.html?Pcl), [simj](http://flybase.bio.indiana.edu/.bin/fbidq.html?simj), [Sin3A](http://flybase.bio.indiana.edu/.bin/fbidq.html?Sin3A), [NC2alpha](http://flybase.bio.indiana.edu/.bin/fbidq.html?NC2alpha), [mip120](http://flybase.bio.indiana.edu/.bin/fbidq.html?mip120), [Snoo](http://flybase.bio.indiana.edu/.bin/fbidq.html?Snoo), [jumu](http://flybase.bio.indiana.edu/.bin/fbidq.html?jumu), [HGTX](http://flybase.bio.indiana.edu/.bin/fbidq.html?HGTX), [ush](http://flybase.bio.indiana.edu/.bin/fbidq.html?ush), [Usp7](http://flybase.bio.indiana.edu/.bin/fbidq.html?Usp7), [fd59A](http://flybase.bio.indiana.edu/.bin/fbidq.html?fd59A), [nab](http://flybase.bio.indiana.edu/.bin/fbidq.html?nab), [btd](http://flybase.bio.indiana.edu/.bin/fbidq.html?btd), [fd68A](http://flybase.bio.indiana.edu/.bin/fbidq.html?fd68A), [HLHmdelta](http://flybase.bio.indiana.edu/.bin/fbidq.html?HLHmdelta), [run](http://flybase.bio.indiana.edu/.bin/fbidq.html?run), [B-H1](http://flybase.bio.indiana.edu/.bin/fbidq.html?B-H1), [FBX011](http://flybase.bio.indiana.edu/.bin/fbidq.html?FBX011), [egg](http://flybase.bio.indiana.edu/.bin/fbidq.html?egg), [l(3)neo38](http://flybase.bio.indiana.edu/.bin/fbidq.html?l(3)neo38), [fd102C](http://flybase.bio.indiana.edu/.bin/fbidq.html?fd102C), [Vsx2](http://flybase.bio.indiana.edu/.bin/fbidq.html?Vsx2), [ph-p](http://flybase.bio.indiana.edu/.bin/fbidq.html?ph-p), [Ubx](http://flybase.bio.indiana.edu/.bin/fbidq.html?Ubx),[CG7757](http://flybase.bio.indiana.edu/.bin/fbidq.html?CG7757), [chm](http://flybase.bio.indiana.edu/.bin/fbidq.html?chm), [sna](http://flybase.bio.indiana.edu/.bin/fbidq.html?sna), [Sfmbt](http://flybase.bio.indiana.edu/.bin/fbidq.html?Sfmbt), [Wnt5](http://flybase.bio.indiana.edu/.bin/fbidq.html?Wnt5), [pan](http://flybase.bio.indiana.edu/.bin/fbidq.html?pan), [MBD-like](http://flybase.bio.indiana.edu/.bin/fbidq.html?MBD-like), [tsh](http://flybase.bio.indiana.edu/.bin/fbidq.html?tsh), [HLHm5](http://flybase.bio.indiana.edu/.bin/fbidq.html?HLHm5), [esg](http://flybase.bio.indiana.edu/.bin/fbidq.html?esg), [e(y)3](http://flybase.bio.indiana.edu/.bin/fbidq.html?e(y)3), [oc](http://flybase.bio.indiana.edu/.bin/fbidq.html?oc), [pros](http://flybase.bio.indiana.edu/.bin/fbidq.html?pros), [Psi](http://flybase.bio.indiana.edu/.bin/fbidq.html?Psi), [Gug](http://flybase.bio.indiana.edu/.bin/fbidq.html?Gug), [pad](http://flybase.bio.indiana.edu/.bin/fbidq.html?pad), [Wnt4](http://flybase.bio.indiana.edu/.bin/fbidq.html?Wnt4), [B-H2](http://flybase.bio.indiana.edu/.bin/fbidq.html?B-H2), [wg](http://flybase.bio.indiana.edu/.bin/fbidq.html?wg), [Vsx1](http://flybase.bio.indiana.edu/.bin/fbidq.html?Vsx1), [fkh](http://flybase.bio.indiana.edu/.bin/fbidq.html?fkh), [gro](http://flybase.bio.indiana.edu/.bin/fbidq.html?gro), [crol](http://flybase.bio.indiana.edu/.bin/fbidq.html?crol), [Rpd3](http://flybase.bio.indiana.edu/.bin/fbidq.html?Rpd3), [retn](http://flybase.bio.indiana.edu/.bin/fbidq.html?retn), [fs(1)h](http://flybase.bio.indiana.edu/.bin/fbidq.html?fs(1)h), [en](http://flybase.bio.indiana.edu/.bin/fbidq.html?en), [dsx](http://flybase.bio.indiana.edu/.bin/fbidq.html?dsx), [tey](http://flybase.bio.indiana.edu/.bin/fbidq.html?tey), [HLHmgamma](http://flybase.bio.indiana.edu/.bin/fbidq.html?HLHmgamma), [z](http://flybase.bio.indiana.edu/.bin/fbidq.html?z), [HLHm7](http://flybase.bio.indiana.edu/.bin/fbidq.html?HLHm7), [Lim1](http://flybase.bio.indiana.edu/.bin/fbidq.html?Lim1), [Ct](http://flybase.bio.indiana.edu/.bin/fbidq.html?Ct) |
| [positive regulation of nitrogen compound metabolic process](http://amigo.geneontology.org/cgi-bin/amigo/go.cgi?view=details&query=GO:0051173) | 73 of 1594 genes, 4.6% | 212 of 7634 genes, 2.8% | 0.00072 | 0.00% | 0.00 | [acj6](http://flybase.bio.indiana.edu/.bin/fbidq.html?acj6), [wor](http://flybase.bio.indiana.edu/.bin/fbidq.html?wor), [vvl](http://flybase.bio.indiana.edu/.bin/fbidq.html?vvl), [caup](http://flybase.bio.indiana.edu/.bin/fbidq.html?caup), [tll](http://flybase.bio.indiana.edu/.bin/fbidq.html?tll), [Camta](http://flybase.bio.indiana.edu/.bin/fbidq.html?Camta), [ato](http://flybase.bio.indiana.edu/.bin/fbidq.html?ato), [tna](http://flybase.bio.indiana.edu/.bin/fbidq.html?tna), [Eip93F](http://flybase.bio.indiana.edu/.bin/fbidq.html?Eip93F), [JIL-1](http://flybase.bio.indiana.edu/.bin/fbidq.html?JIL-1), [ap](http://flybase.bio.indiana.edu/.bin/fbidq.html?ap), [Iswi](http://flybase.bio.indiana.edu/.bin/fbidq.html?Iswi), [Spt6](http://flybase.bio.indiana.edu/.bin/fbidq.html?Spt6), [gcm](http://flybase.bio.indiana.edu/.bin/fbidq.html?gcm), [Dip3](http://flybase.bio.indiana.edu/.bin/fbidq.html?Dip3), [Nf1](http://flybase.bio.indiana.edu/.bin/fbidq.html?Nf1), [ase](http://flybase.bio.indiana.edu/.bin/fbidq.html?ase), [br](http://flybase.bio.indiana.edu/.bin/fbidq.html?br), [ovo](http://flybase.bio.indiana.edu/.bin/fbidq.html?ovo), [Pdp1](http://flybase.bio.indiana.edu/.bin/fbidq.html?Pdp1), [chn](http://flybase.bio.indiana.edu/.bin/fbidq.html?chn), [Hcf](http://flybase.bio.indiana.edu/.bin/fbidq.html?Hcf), [lola](http://flybase.bio.indiana.edu/.bin/fbidq.html?lola), [NC2alpha](http://flybase.bio.indiana.edu/.bin/fbidq.html?NC2alpha), [Taf4](http://flybase.bio.indiana.edu/.bin/fbidq.html?Taf4), [scrib](http://flybase.bio.indiana.edu/.bin/fbidq.html?scrib), [HGTX](http://flybase.bio.indiana.edu/.bin/fbidq.html?HGTX), [fd59A](http://flybase.bio.indiana.edu/.bin/fbidq.html?fd59A), [btd](http://flybase.bio.indiana.edu/.bin/fbidq.html?btd), [run](http://flybase.bio.indiana.edu/.bin/fbidq.html?run), [Antp](http://flybase.bio.indiana.edu/.bin/fbidq.html?Antp), [ATbp](http://flybase.bio.indiana.edu/.bin/fbidq.html?ATbp), [tara](http://flybase.bio.indiana.edu/.bin/fbidq.html?tara), [Hel89B](http://flybase.bio.indiana.edu/.bin/fbidq.html?Hel89B), [hth](http://flybase.bio.indiana.edu/.bin/fbidq.html?hth), [vg](http://flybase.bio.indiana.edu/.bin/fbidq.html?vg), [eya](http://flybase.bio.indiana.edu/.bin/fbidq.html?eya), [brm](http://flybase.bio.indiana.edu/.bin/fbidq.html?brm), [onecut](http://flybase.bio.indiana.edu/.bin/fbidq.html?onecut), [sno](http://flybase.bio.indiana.edu/.bin/fbidq.html?sno), [chm](http://flybase.bio.indiana.edu/.bin/fbidq.html?chm), [ara](http://flybase.bio.indiana.edu/.bin/fbidq.html?ara), [sna](http://flybase.bio.indiana.edu/.bin/fbidq.html?sna),[mam](http://flybase.bio.indiana.edu/.bin/fbidq.html?mam), [Taf1](http://flybase.bio.indiana.edu/.bin/fbidq.html?Taf1), [pan](http://flybase.bio.indiana.edu/.bin/fbidq.html?pan), [disco](http://flybase.bio.indiana.edu/.bin/fbidq.html?disco), [esg](http://flybase.bio.indiana.edu/.bin/fbidq.html?esg), [e(y)3](http://flybase.bio.indiana.edu/.bin/fbidq.html?e(y)3), [Brf](http://flybase.bio.indiana.edu/.bin/fbidq.html?Brf), [oc](http://flybase.bio.indiana.edu/.bin/fbidq.html?oc), [tup](http://flybase.bio.indiana.edu/.bin/fbidq.html?tup), [spt4](http://flybase.bio.indiana.edu/.bin/fbidq.html?spt4), [ey](http://flybase.bio.indiana.edu/.bin/fbidq.html?ey), [mle](http://flybase.bio.indiana.edu/.bin/fbidq.html?mle), [l(1)sc](http://flybase.bio.indiana.edu/.bin/fbidq.html?l(1)sc), [Bgb](http://flybase.bio.indiana.edu/.bin/fbidq.html?Bgb), [grh](http://flybase.bio.indiana.edu/.bin/fbidq.html?grh), [toy](http://flybase.bio.indiana.edu/.bin/fbidq.html?toy), [wg](http://flybase.bio.indiana.edu/.bin/fbidq.html?wg), [fkh](http://flybase.bio.indiana.edu/.bin/fbidq.html?fkh), [sim](http://flybase.bio.indiana.edu/.bin/fbidq.html?sim), [sd](http://flybase.bio.indiana.edu/.bin/fbidq.html?sd), [trx](http://flybase.bio.indiana.edu/.bin/fbidq.html?trx), [retn](http://flybase.bio.indiana.edu/.bin/fbidq.html?retn), [dsx](http://flybase.bio.indiana.edu/.bin/fbidq.html?dsx), [vfl](http://flybase.bio.indiana.edu/.bin/fbidq.html?vfl), [CG11347](http://flybase.bio.indiana.edu/.bin/fbidq.html?CG11347), [dalao](http://flybase.bio.indiana.edu/.bin/fbidq.html?dalao), [pygo](http://flybase.bio.indiana.edu/.bin/fbidq.html?pygo), [z](http://flybase.bio.indiana.edu/.bin/fbidq.html?z), [Lim1](http://flybase.bio.indiana.edu/.bin/fbidq.html?Lim1), [Atf-2](http://flybase.bio.indiana.edu/.bin/fbidq.html?Atf-2) |
| [post-embryonic organ development](http://amigo.geneontology.org/cgi-bin/amigo/go.cgi?view=details&query=GO:0048569) | 114 of 1594 genes, 7.2% | 374 of 7634 genes, 4.9% | 0.00075 | 0.00% | 0.00 | [fz2](http://flybase.bio.indiana.edu/.bin/fbidq.html?fz2), [tld](http://flybase.bio.indiana.edu/.bin/fbidq.html?tld), [jar](http://flybase.bio.indiana.edu/.bin/fbidq.html?jar), [tow](http://flybase.bio.indiana.edu/.bin/fbidq.html?tow), [skd](http://flybase.bio.indiana.edu/.bin/fbidq.html?skd), [jing](http://flybase.bio.indiana.edu/.bin/fbidq.html?jing), [caup](http://flybase.bio.indiana.edu/.bin/fbidq.html?caup), [betaInt-nu](http://flybase.bio.indiana.edu/.bin/fbidq.html?betaInt-nu), [ato](http://flybase.bio.indiana.edu/.bin/fbidq.html?ato), [sgg](http://flybase.bio.indiana.edu/.bin/fbidq.html?sgg), [Nmdar1](http://flybase.bio.indiana.edu/.bin/fbidq.html?Nmdar1), [bowl](http://flybase.bio.indiana.edu/.bin/fbidq.html?bowl), [rn](http://flybase.bio.indiana.edu/.bin/fbidq.html?rn), [ap](http://flybase.bio.indiana.edu/.bin/fbidq.html?ap), [Dg](http://flybase.bio.indiana.edu/.bin/fbidq.html?Dg), [gcm](http://flybase.bio.indiana.edu/.bin/fbidq.html?gcm), [CG14478](http://flybase.bio.indiana.edu/.bin/fbidq.html?CG14478), [phl](http://flybase.bio.indiana.edu/.bin/fbidq.html?phl), [Cbl](http://flybase.bio.indiana.edu/.bin/fbidq.html?Cbl), [ovo](http://flybase.bio.indiana.edu/.bin/fbidq.html?ovo), [sns](http://flybase.bio.indiana.edu/.bin/fbidq.html?sns), [ksr](http://flybase.bio.indiana.edu/.bin/fbidq.html?ksr), [os](http://flybase.bio.indiana.edu/.bin/fbidq.html?os), [Axn](http://flybase.bio.indiana.edu/.bin/fbidq.html?Axn), [tok](http://flybase.bio.indiana.edu/.bin/fbidq.html?tok), [bi](http://flybase.bio.indiana.edu/.bin/fbidq.html?bi), [ds](http://flybase.bio.indiana.edu/.bin/fbidq.html?ds), [CG15312](http://flybase.bio.indiana.edu/.bin/fbidq.html?CG15312), [Poxn](http://flybase.bio.indiana.edu/.bin/fbidq.html?Poxn), [ewg](http://flybase.bio.indiana.edu/.bin/fbidq.html?ewg), [pot](http://flybase.bio.indiana.edu/.bin/fbidq.html?pot), [bab1](http://flybase.bio.indiana.edu/.bin/fbidq.html?bab1), [jumu](http://flybase.bio.indiana.edu/.bin/fbidq.html?jumu), [Smox](http://flybase.bio.indiana.edu/.bin/fbidq.html?Smox), [scrib](http://flybase.bio.indiana.edu/.bin/fbidq.html?scrib), [Su(var)3-3](http://flybase.bio.indiana.edu/.bin/fbidq.html?Su(var)3-3), [MYPT-75D](http://flybase.bio.indiana.edu/.bin/fbidq.html?MYPT-75D), [ush](http://flybase.bio.indiana.edu/.bin/fbidq.html?ush), [Nrg](http://flybase.bio.indiana.edu/.bin/fbidq.html?Nrg), [Gap1](http://flybase.bio.indiana.edu/.bin/fbidq.html?Gap1), [btd](http://flybase.bio.indiana.edu/.bin/fbidq.html?btd),[Mmp2](http://flybase.bio.indiana.edu/.bin/fbidq.html?Mmp2), [B-H1](http://flybase.bio.indiana.edu/.bin/fbidq.html?B-H1), [alph](http://flybase.bio.indiana.edu/.bin/fbidq.html?alph), [Hs3st-B](http://flybase.bio.indiana.edu/.bin/fbidq.html?Hs3st-B), [Traf4](http://flybase.bio.indiana.edu/.bin/fbidq.html?Traf4), [l(3)neo38](http://flybase.bio.indiana.edu/.bin/fbidq.html?l(3)neo38), [Sos](http://flybase.bio.indiana.edu/.bin/fbidq.html?Sos), [Sp1](http://flybase.bio.indiana.edu/.bin/fbidq.html?Sp1), [hth](http://flybase.bio.indiana.edu/.bin/fbidq.html?hth), [vg](http://flybase.bio.indiana.edu/.bin/fbidq.html?vg), [dsh](http://flybase.bio.indiana.edu/.bin/fbidq.html?dsh), [Fas2](http://flybase.bio.indiana.edu/.bin/fbidq.html?Fas2), [eya](http://flybase.bio.indiana.edu/.bin/fbidq.html?eya), [fz](http://flybase.bio.indiana.edu/.bin/fbidq.html?fz), [CG8557](http://flybase.bio.indiana.edu/.bin/fbidq.html?CG8557), [bsk](http://flybase.bio.indiana.edu/.bin/fbidq.html?bsk), [brm](http://flybase.bio.indiana.edu/.bin/fbidq.html?brm), [Ubx](http://flybase.bio.indiana.edu/.bin/fbidq.html?Ubx), [ft](http://flybase.bio.indiana.edu/.bin/fbidq.html?ft), [CG42268](http://flybase.bio.indiana.edu/.bin/fbidq.html?CG42268), [lft](http://flybase.bio.indiana.edu/.bin/fbidq.html?lft), [sno](http://flybase.bio.indiana.edu/.bin/fbidq.html?sno), [ara](http://flybase.bio.indiana.edu/.bin/fbidq.html?ara), [mam](http://flybase.bio.indiana.edu/.bin/fbidq.html?mam), [pan](http://flybase.bio.indiana.edu/.bin/fbidq.html?pan), [Dll](http://flybase.bio.indiana.edu/.bin/fbidq.html?Dll), [tsh](http://flybase.bio.indiana.edu/.bin/fbidq.html?tsh), [mew](http://flybase.bio.indiana.edu/.bin/fbidq.html?mew), [neur](http://flybase.bio.indiana.edu/.bin/fbidq.html?neur), [esg](http://flybase.bio.indiana.edu/.bin/fbidq.html?esg), [e(y)3](http://flybase.bio.indiana.edu/.bin/fbidq.html?e(y)3), [dac](http://flybase.bio.indiana.edu/.bin/fbidq.html?dac), [dap](http://flybase.bio.indiana.edu/.bin/fbidq.html?dap), [nmo](http://flybase.bio.indiana.edu/.bin/fbidq.html?nmo), [oc](http://flybase.bio.indiana.edu/.bin/fbidq.html?oc), [Bx](http://flybase.bio.indiana.edu/.bin/fbidq.html?Bx), [CG32149](http://flybase.bio.indiana.edu/.bin/fbidq.html?CG32149), [trio](http://flybase.bio.indiana.edu/.bin/fbidq.html?trio), [ey](http://flybase.bio.indiana.edu/.bin/fbidq.html?ey), [ed](http://flybase.bio.indiana.edu/.bin/fbidq.html?ed), [Gug](http://flybase.bio.indiana.edu/.bin/fbidq.html?Gug), [klar](http://flybase.bio.indiana.edu/.bin/fbidq.html?klar),[CG33275](http://flybase.bio.indiana.edu/.bin/fbidq.html?CG33275), [Hr51](http://flybase.bio.indiana.edu/.bin/fbidq.html?Hr51), [ImpE2](http://flybase.bio.indiana.edu/.bin/fbidq.html?ImpE2), [siz](http://flybase.bio.indiana.edu/.bin/fbidq.html?siz), [CG6398](http://flybase.bio.indiana.edu/.bin/fbidq.html?CG6398), [Wnt4](http://flybase.bio.indiana.edu/.bin/fbidq.html?Wnt4), [B-H2](http://flybase.bio.indiana.edu/.bin/fbidq.html?B-H2), [wg](http://flybase.bio.indiana.edu/.bin/fbidq.html?wg), [salr](http://flybase.bio.indiana.edu/.bin/fbidq.html?salr), [Glut4EF](http://flybase.bio.indiana.edu/.bin/fbidq.html?Glut4EF), [crol](http://flybase.bio.indiana.edu/.bin/fbidq.html?crol), [sd](http://flybase.bio.indiana.edu/.bin/fbidq.html?sd), [Btk29A](http://flybase.bio.indiana.edu/.bin/fbidq.html?Btk29A), [fs(1)h](http://flybase.bio.indiana.edu/.bin/fbidq.html?fs(1)h), [pk](http://flybase.bio.indiana.edu/.bin/fbidq.html?pk), [dsx](http://flybase.bio.indiana.edu/.bin/fbidq.html?dsx), [sfl](http://flybase.bio.indiana.edu/.bin/fbidq.html?sfl), [rl](http://flybase.bio.indiana.edu/.bin/fbidq.html?rl), [chinmo](http://flybase.bio.indiana.edu/.bin/fbidq.html?chinmo), [Sip1](http://flybase.bio.indiana.edu/.bin/fbidq.html?Sip1), [Snap](http://flybase.bio.indiana.edu/.bin/fbidq.html?Snap), [trn](http://flybase.bio.indiana.edu/.bin/fbidq.html?trn), [aret](http://flybase.bio.indiana.edu/.bin/fbidq.html?aret), [wls](http://flybase.bio.indiana.edu/.bin/fbidq.html?wls), [pygo](http://flybase.bio.indiana.edu/.bin/fbidq.html?pygo), [spag](http://flybase.bio.indiana.edu/.bin/fbidq.html?spag), [unk](http://flybase.bio.indiana.edu/.bin/fbidq.html?unk), [Lim1](http://flybase.bio.indiana.edu/.bin/fbidq.html?Lim1), [cos](http://flybase.bio.indiana.edu/.bin/fbidq.html?cos), [Ct](http://flybase.bio.indiana.edu/.bin/fbidq.html?Ct), [dnr1](http://flybase.bio.indiana.edu/.bin/fbidq.html?dnr1) |
| [single-organism developmental process](http://amigo.geneontology.org/cgi-bin/amigo/go.cgi?view=details&query=GO:0044767) | 411 of 1594 genes, 25.8% | 1701 of 7634 genes, 22.3% | 0.00085 | 0.00% | 0.00 | [fz2](http://flybase.bio.indiana.edu/.bin/fbidq.html?fz2), [Ocho](http://flybase.bio.indiana.edu/.bin/fbidq.html?Ocho), [Sh](http://flybase.bio.indiana.edu/.bin/fbidq.html?Sh), [numb](http://flybase.bio.indiana.edu/.bin/fbidq.html?numb), [dock](http://flybase.bio.indiana.edu/.bin/fbidq.html?dock), [CG42256](http://flybase.bio.indiana.edu/.bin/fbidq.html?CG42256), [CG3703](http://flybase.bio.indiana.edu/.bin/fbidq.html?CG3703), [stj](http://flybase.bio.indiana.edu/.bin/fbidq.html?stj), [Sxl](http://flybase.bio.indiana.edu/.bin/fbidq.html?Sxl), [CG33007](http://flybase.bio.indiana.edu/.bin/fbidq.html?CG33007), [rn](http://flybase.bio.indiana.edu/.bin/fbidq.html?rn), [ap](http://flybase.bio.indiana.edu/.bin/fbidq.html?ap), [kkv](http://flybase.bio.indiana.edu/.bin/fbidq.html?kkv), [tlk](http://flybase.bio.indiana.edu/.bin/fbidq.html?tlk), [Nf1](http://flybase.bio.indiana.edu/.bin/fbidq.html?Nf1), [malpha](http://flybase.bio.indiana.edu/.bin/fbidq.html?malpha), [sns](http://flybase.bio.indiana.edu/.bin/fbidq.html?sns), [os](http://flybase.bio.indiana.edu/.bin/fbidq.html?os), [Axn](http://flybase.bio.indiana.edu/.bin/fbidq.html?Axn), [tok](http://flybase.bio.indiana.edu/.bin/fbidq.html?tok), [bi](http://flybase.bio.indiana.edu/.bin/fbidq.html?bi), [Ptp99A](http://flybase.bio.indiana.edu/.bin/fbidq.html?Ptp99A), [Traf6](http://flybase.bio.indiana.edu/.bin/fbidq.html?Traf6), [CG31961](http://flybase.bio.indiana.edu/.bin/fbidq.html?CG31961), [NetB](http://flybase.bio.indiana.edu/.bin/fbidq.html?NetB), [Tango10](http://flybase.bio.indiana.edu/.bin/fbidq.html?Tango10), [Oseg4](http://flybase.bio.indiana.edu/.bin/fbidq.html?Oseg4), [CG32137](http://flybase.bio.indiana.edu/.bin/fbidq.html?CG32137), [blue](http://flybase.bio.indiana.edu/.bin/fbidq.html?blue), [bab1](http://flybase.bio.indiana.edu/.bin/fbidq.html?bab1), [Galpha49B](http://flybase.bio.indiana.edu/.bin/fbidq.html?Galpha49B), [Snoo](http://flybase.bio.indiana.edu/.bin/fbidq.html?Snoo), [Lar](http://flybase.bio.indiana.edu/.bin/fbidq.html?Lar), [scrib](http://flybase.bio.indiana.edu/.bin/fbidq.html?scrib), [not](http://flybase.bio.indiana.edu/.bin/fbidq.html?not), [rut](http://flybase.bio.indiana.edu/.bin/fbidq.html?rut), [nvy](http://flybase.bio.indiana.edu/.bin/fbidq.html?nvy), [Ect3](http://flybase.bio.indiana.edu/.bin/fbidq.html?Ect3),[nub](http://flybase.bio.indiana.edu/.bin/fbidq.html?nub), [nab](http://flybase.bio.indiana.edu/.bin/fbidq.html?nab), [Ddc](http://flybase.bio.indiana.edu/.bin/fbidq.html?Ddc), [btd](http://flybase.bio.indiana.edu/.bin/fbidq.html?btd), [fd68A](http://flybase.bio.indiana.edu/.bin/fbidq.html?fd68A), [Mmp2](http://flybase.bio.indiana.edu/.bin/fbidq.html?Mmp2), [HLHmdelta](http://flybase.bio.indiana.edu/.bin/fbidq.html?HLHmdelta), [B-H1](http://flybase.bio.indiana.edu/.bin/fbidq.html?B-H1), [Nipped-A](http://flybase.bio.indiana.edu/.bin/fbidq.html?Nipped-A), [pim](http://flybase.bio.indiana.edu/.bin/fbidq.html?pim), [alph](http://flybase.bio.indiana.edu/.bin/fbidq.html?alph), [Traf4](http://flybase.bio.indiana.edu/.bin/fbidq.html?Traf4), [TBPH](http://flybase.bio.indiana.edu/.bin/fbidq.html?TBPH), [CG34400](http://flybase.bio.indiana.edu/.bin/fbidq.html?CG34400), [fd102C](http://flybase.bio.indiana.edu/.bin/fbidq.html?fd102C), [Ulp1](http://flybase.bio.indiana.edu/.bin/fbidq.html?Ulp1), [ena](http://flybase.bio.indiana.edu/.bin/fbidq.html?ena), [mGluRA](http://flybase.bio.indiana.edu/.bin/fbidq.html?mGluRA), [dsh](http://flybase.bio.indiana.edu/.bin/fbidq.html?dsh), [hth](http://flybase.bio.indiana.edu/.bin/fbidq.html?hth), [Nf-YC](http://flybase.bio.indiana.edu/.bin/fbidq.html?Nf-YC), [14-3-3zeta](http://flybase.bio.indiana.edu/.bin/fbidq.html?14-3-3zeta), [lea](http://flybase.bio.indiana.edu/.bin/fbidq.html?lea), [plexB](http://flybase.bio.indiana.edu/.bin/fbidq.html?plexB), [PP2A-B](http://flybase.bio.indiana.edu/.bin/fbidq.html?PP2A-B), [loqs](http://flybase.bio.indiana.edu/.bin/fbidq.html?loqs), [Klp64D](http://flybase.bio.indiana.edu/.bin/fbidq.html?Klp64D), [DAAM](http://flybase.bio.indiana.edu/.bin/fbidq.html?DAAM), [neur](http://flybase.bio.indiana.edu/.bin/fbidq.html?neur), [CycE](http://flybase.bio.indiana.edu/.bin/fbidq.html?CycE), [Brf](http://flybase.bio.indiana.edu/.bin/fbidq.html?Brf), [nmo](http://flybase.bio.indiana.edu/.bin/fbidq.html?nmo), [CG32149](http://flybase.bio.indiana.edu/.bin/fbidq.html?CG32149), [Gug](http://flybase.bio.indiana.edu/.bin/fbidq.html?Gug), [ed](http://flybase.bio.indiana.edu/.bin/fbidq.html?ed),[Actn](http://flybase.bio.indiana.edu/.bin/fbidq.html?Actn), [pad](http://flybase.bio.indiana.edu/.bin/fbidq.html?pad), [mr](http://flybase.bio.indiana.edu/.bin/fbidq.html?mr), [daw](http://flybase.bio.indiana.edu/.bin/fbidq.html?daw), [ImpE2](http://flybase.bio.indiana.edu/.bin/fbidq.html?ImpE2), [Rx](http://flybase.bio.indiana.edu/.bin/fbidq.html?Rx), [salr](http://flybase.bio.indiana.edu/.bin/fbidq.html?salr), [Apc](http://flybase.bio.indiana.edu/.bin/fbidq.html?Apc), [sim](http://flybase.bio.indiana.edu/.bin/fbidq.html?sim), [d4](http://flybase.bio.indiana.edu/.bin/fbidq.html?d4), [cort](http://flybase.bio.indiana.edu/.bin/fbidq.html?cort), [dan](http://flybase.bio.indiana.edu/.bin/fbidq.html?dan), [gro](http://flybase.bio.indiana.edu/.bin/fbidq.html?gro), [RN-tre](http://flybase.bio.indiana.edu/.bin/fbidq.html?RN-tre), [Mf](http://flybase.bio.indiana.edu/.bin/fbidq.html?Mf), [scyl](http://flybase.bio.indiana.edu/.bin/fbidq.html?scyl), [wnd](http://flybase.bio.indiana.edu/.bin/fbidq.html?wnd), [trx](http://flybase.bio.indiana.edu/.bin/fbidq.html?trx), [rdo](http://flybase.bio.indiana.edu/.bin/fbidq.html?rdo), [Rpd3](http://flybase.bio.indiana.edu/.bin/fbidq.html?Rpd3), [Btk29A](http://flybase.bio.indiana.edu/.bin/fbidq.html?Btk29A), [CG14367](http://flybase.bio.indiana.edu/.bin/fbidq.html?CG14367), [unc-5](http://flybase.bio.indiana.edu/.bin/fbidq.html?unc-5), [cdi](http://flybase.bio.indiana.edu/.bin/fbidq.html?cdi), [vfl](http://flybase.bio.indiana.edu/.bin/fbidq.html?vfl), [CG10107](http://flybase.bio.indiana.edu/.bin/fbidq.html?CG10107), [barr](http://flybase.bio.indiana.edu/.bin/fbidq.html?barr), [aret](http://flybase.bio.indiana.edu/.bin/fbidq.html?aret), [pygo](http://flybase.bio.indiana.edu/.bin/fbidq.html?pygo), [HLHm7](http://flybase.bio.indiana.edu/.bin/fbidq.html?HLHm7), [HLHmgamma](http://flybase.bio.indiana.edu/.bin/fbidq.html?HLHmgamma), [spag](http://flybase.bio.indiana.edu/.bin/fbidq.html?spag), [jar](http://flybase.bio.indiana.edu/.bin/fbidq.html?jar), [tow](http://flybase.bio.indiana.edu/.bin/fbidq.html?tow), [CG15609](http://flybase.bio.indiana.edu/.bin/fbidq.html?CG15609), [wor](http://flybase.bio.indiana.edu/.bin/fbidq.html?wor), [Atg1](http://flybase.bio.indiana.edu/.bin/fbidq.html?Atg1), [l(1)G0148](http://flybase.bio.indiana.edu/.bin/fbidq.html?l(1)G0148), [Pten](http://flybase.bio.indiana.edu/.bin/fbidq.html?Pten),[mira](http://flybase.bio.indiana.edu/.bin/fbidq.html?mira), [Ptp69D](http://flybase.bio.indiana.edu/.bin/fbidq.html?Ptp69D), [hts](http://flybase.bio.indiana.edu/.bin/fbidq.html?hts), [pUf68](http://flybase.bio.indiana.edu/.bin/fbidq.html?pUf68), [Myo10A](http://flybase.bio.indiana.edu/.bin/fbidq.html?Myo10A), [bowl](http://flybase.bio.indiana.edu/.bin/fbidq.html?bowl), [seq](http://flybase.bio.indiana.edu/.bin/fbidq.html?seq), [Sug](http://flybase.bio.indiana.edu/.bin/fbidq.html?Sug), [Dip3](http://flybase.bio.indiana.edu/.bin/fbidq.html?Dip3), [jbug](http://flybase.bio.indiana.edu/.bin/fbidq.html?jbug), [oa2](http://flybase.bio.indiana.edu/.bin/fbidq.html?oa2), [phl](http://flybase.bio.indiana.edu/.bin/fbidq.html?phl), [Cbl](http://flybase.bio.indiana.edu/.bin/fbidq.html?Cbl), [thr](http://flybase.bio.indiana.edu/.bin/fbidq.html?thr), [sNPF](http://flybase.bio.indiana.edu/.bin/fbidq.html?sNPF), [CG4893](http://flybase.bio.indiana.edu/.bin/fbidq.html?CG4893), [pog](http://flybase.bio.indiana.edu/.bin/fbidq.html?pog), [CASK](http://flybase.bio.indiana.edu/.bin/fbidq.html?CASK), [CG9098](http://flybase.bio.indiana.edu/.bin/fbidq.html?CG9098), [Alk](http://flybase.bio.indiana.edu/.bin/fbidq.html?Alk), [esn](http://flybase.bio.indiana.edu/.bin/fbidq.html?esn), [Vang](http://flybase.bio.indiana.edu/.bin/fbidq.html?Vang), [Sin3A](http://flybase.bio.indiana.edu/.bin/fbidq.html?Sin3A), [pot](http://flybase.bio.indiana.edu/.bin/fbidq.html?pot), [mip120](http://flybase.bio.indiana.edu/.bin/fbidq.html?mip120), [cpx](http://flybase.bio.indiana.edu/.bin/fbidq.html?cpx), [jumu](http://flybase.bio.indiana.edu/.bin/fbidq.html?jumu), [Smox](http://flybase.bio.indiana.edu/.bin/fbidq.html?Smox), [CG31475](http://flybase.bio.indiana.edu/.bin/fbidq.html?CG31475), [fd59A](http://flybase.bio.indiana.edu/.bin/fbidq.html?fd59A), [beat-Ic](http://flybase.bio.indiana.edu/.bin/fbidq.html?beat-Ic), [brat](http://flybase.bio.indiana.edu/.bin/fbidq.html?brat), [ST6Gal](http://flybase.bio.indiana.edu/.bin/fbidq.html?ST6Gal), [Antp](http://flybase.bio.indiana.edu/.bin/fbidq.html?Antp), [l(3)neo38](http://flybase.bio.indiana.edu/.bin/fbidq.html?l(3)neo38), [cindr](http://flybase.bio.indiana.edu/.bin/fbidq.html?cindr),[futsch](http://flybase.bio.indiana.edu/.bin/fbidq.html?futsch), [comm3](http://flybase.bio.indiana.edu/.bin/fbidq.html?comm3), [Ubx](http://flybase.bio.indiana.edu/.bin/fbidq.html?Ubx), [Ptx1](http://flybase.bio.indiana.edu/.bin/fbidq.html?Ptx1), [gukh](http://flybase.bio.indiana.edu/.bin/fbidq.html?gukh), [chm](http://flybase.bio.indiana.edu/.bin/fbidq.html?chm), [CG6114](http://flybase.bio.indiana.edu/.bin/fbidq.html?CG6114), [Rim](http://flybase.bio.indiana.edu/.bin/fbidq.html?Rim), [beat-IIa](http://flybase.bio.indiana.edu/.bin/fbidq.html?beat-IIa), [tsh](http://flybase.bio.indiana.edu/.bin/fbidq.html?tsh), [sm](http://flybase.bio.indiana.edu/.bin/fbidq.html?sm), [Syn2](http://flybase.bio.indiana.edu/.bin/fbidq.html?Syn2), [eas](http://flybase.bio.indiana.edu/.bin/fbidq.html?eas), [dac](http://flybase.bio.indiana.edu/.bin/fbidq.html?dac), [e(y)3](http://flybase.bio.indiana.edu/.bin/fbidq.html?e(y)3), [Sry-alpha](http://flybase.bio.indiana.edu/.bin/fbidq.html?Sry-alpha), [Cdk5](http://flybase.bio.indiana.edu/.bin/fbidq.html?Cdk5), [Cep135](http://flybase.bio.indiana.edu/.bin/fbidq.html?Cep135), [oc](http://flybase.bio.indiana.edu/.bin/fbidq.html?oc), [CG4641](http://flybase.bio.indiana.edu/.bin/fbidq.html?CG4641), [ey](http://flybase.bio.indiana.edu/.bin/fbidq.html?ey), [baz](http://flybase.bio.indiana.edu/.bin/fbidq.html?baz), [CG33275](http://flybase.bio.indiana.edu/.bin/fbidq.html?CG33275), [rst](http://flybase.bio.indiana.edu/.bin/fbidq.html?rst), [CG6398](http://flybase.bio.indiana.edu/.bin/fbidq.html?CG6398), [l(1)sc](http://flybase.bio.indiana.edu/.bin/fbidq.html?l(1)sc), [grh](http://flybase.bio.indiana.edu/.bin/fbidq.html?grh), [comm](http://flybase.bio.indiana.edu/.bin/fbidq.html?comm), [Vsx1](http://flybase.bio.indiana.edu/.bin/fbidq.html?Vsx1), [Cdk5alpha](http://flybase.bio.indiana.edu/.bin/fbidq.html?Cdk5alpha), [toy](http://flybase.bio.indiana.edu/.bin/fbidq.html?toy), [Lim3](http://flybase.bio.indiana.edu/.bin/fbidq.html?Lim3), [crol](http://flybase.bio.indiana.edu/.bin/fbidq.html?crol), [sd](http://flybase.bio.indiana.edu/.bin/fbidq.html?sd), [Aplip1](http://flybase.bio.indiana.edu/.bin/fbidq.html?Aplip1), [E(Pc)](http://flybase.bio.indiana.edu/.bin/fbidq.html?E(Pc)), [retn](http://flybase.bio.indiana.edu/.bin/fbidq.html?retn),[fs(1)h](http://flybase.bio.indiana.edu/.bin/fbidq.html?fs(1)h), [Eip63F-1](http://flybase.bio.indiana.edu/.bin/fbidq.html?Eip63F-1), [rl](http://flybase.bio.indiana.edu/.bin/fbidq.html?rl), [Syt1](http://flybase.bio.indiana.edu/.bin/fbidq.html?Syt1), [tey](http://flybase.bio.indiana.edu/.bin/fbidq.html?tey), [yrt](http://flybase.bio.indiana.edu/.bin/fbidq.html?yrt), [wls](http://flybase.bio.indiana.edu/.bin/fbidq.html?wls), [CG33960](http://flybase.bio.indiana.edu/.bin/fbidq.html?CG33960), [hiw](http://flybase.bio.indiana.edu/.bin/fbidq.html?hiw), [unk](http://flybase.bio.indiana.edu/.bin/fbidq.html?unk), [CG1962](http://flybase.bio.indiana.edu/.bin/fbidq.html?CG1962), [CG32653](http://flybase.bio.indiana.edu/.bin/fbidq.html?CG32653), [dnr1](http://flybase.bio.indiana.edu/.bin/fbidq.html?dnr1), [acj6](http://flybase.bio.indiana.edu/.bin/fbidq.html?acj6), [skd](http://flybase.bio.indiana.edu/.bin/fbidq.html?skd), [jing](http://flybase.bio.indiana.edu/.bin/fbidq.html?jing), [katanin-60](http://flybase.bio.indiana.edu/.bin/fbidq.html?katanin-60), [caup](http://flybase.bio.indiana.edu/.bin/fbidq.html?caup), [tll](http://flybase.bio.indiana.edu/.bin/fbidq.html?tll), [vimar](http://flybase.bio.indiana.edu/.bin/fbidq.html?vimar), [ato](http://flybase.bio.indiana.edu/.bin/fbidq.html?ato), [Iswi](http://flybase.bio.indiana.edu/.bin/fbidq.html?Iswi), [Dg](http://flybase.bio.indiana.edu/.bin/fbidq.html?Dg), [Trim9](http://flybase.bio.indiana.edu/.bin/fbidq.html?Trim9), [obst-A](http://flybase.bio.indiana.edu/.bin/fbidq.html?obst-A), [CG14478](http://flybase.bio.indiana.edu/.bin/fbidq.html?CG14478), [Pka-R1](http://flybase.bio.indiana.edu/.bin/fbidq.html?Pka-R1), [Evi5](http://flybase.bio.indiana.edu/.bin/fbidq.html?Evi5), [mago](http://flybase.bio.indiana.edu/.bin/fbidq.html?mago), [CG9867](http://flybase.bio.indiana.edu/.bin/fbidq.html?CG9867), [br](http://flybase.bio.indiana.edu/.bin/fbidq.html?br), [enc](http://flybase.bio.indiana.edu/.bin/fbidq.html?enc), [CG7154](http://flybase.bio.indiana.edu/.bin/fbidq.html?CG7154), [neo](http://flybase.bio.indiana.edu/.bin/fbidq.html?neo), [ksr](http://flybase.bio.indiana.edu/.bin/fbidq.html?ksr), [robo3](http://flybase.bio.indiana.edu/.bin/fbidq.html?robo3), [cuff](http://flybase.bio.indiana.edu/.bin/fbidq.html?cuff),[CG3618](http://flybase.bio.indiana.edu/.bin/fbidq.html?CG3618), [chn](http://flybase.bio.indiana.edu/.bin/fbidq.html?chn), [lola](http://flybase.bio.indiana.edu/.bin/fbidq.html?lola), [unc-104](http://flybase.bio.indiana.edu/.bin/fbidq.html?unc-104), [chif](http://flybase.bio.indiana.edu/.bin/fbidq.html?chif), [p130CAS](http://flybase.bio.indiana.edu/.bin/fbidq.html?p130CAS), [CG15312](http://flybase.bio.indiana.edu/.bin/fbidq.html?CG15312), [Awh](http://flybase.bio.indiana.edu/.bin/fbidq.html?Awh), [Appl](http://flybase.bio.indiana.edu/.bin/fbidq.html?Appl), [tap](http://flybase.bio.indiana.edu/.bin/fbidq.html?tap), [metro](http://flybase.bio.indiana.edu/.bin/fbidq.html?metro), [ewg](http://flybase.bio.indiana.edu/.bin/fbidq.html?ewg), [erm](http://flybase.bio.indiana.edu/.bin/fbidq.html?erm), [Nrk](http://flybase.bio.indiana.edu/.bin/fbidq.html?Nrk), [sano](http://flybase.bio.indiana.edu/.bin/fbidq.html?sano), [CG4328](http://flybase.bio.indiana.edu/.bin/fbidq.html?CG4328), [CG11399](http://flybase.bio.indiana.edu/.bin/fbidq.html?CG11399), [Su(var)3-3](http://flybase.bio.indiana.edu/.bin/fbidq.html?Su(var)3-3), [CG10249](http://flybase.bio.indiana.edu/.bin/fbidq.html?CG10249), [Taf4](http://flybase.bio.indiana.edu/.bin/fbidq.html?Taf4), [Nrg](http://flybase.bio.indiana.edu/.bin/fbidq.html?Nrg), [gogo](http://flybase.bio.indiana.edu/.bin/fbidq.html?gogo), [insc](http://flybase.bio.indiana.edu/.bin/fbidq.html?insc), [jeb](http://flybase.bio.indiana.edu/.bin/fbidq.html?jeb), [GluClalpha](http://flybase.bio.indiana.edu/.bin/fbidq.html?GluClalpha), [Actbeta](http://flybase.bio.indiana.edu/.bin/fbidq.html?Actbeta), [stau](http://flybase.bio.indiana.edu/.bin/fbidq.html?stau), [beat-Ib](http://flybase.bio.indiana.edu/.bin/fbidq.html?beat-Ib), [Sema-1a](http://flybase.bio.indiana.edu/.bin/fbidq.html?Sema-1a), [Hs3st-B](http://flybase.bio.indiana.edu/.bin/fbidq.html?Hs3st-B), [otk](http://flybase.bio.indiana.edu/.bin/fbidq.html?otk), [Sos](http://flybase.bio.indiana.edu/.bin/fbidq.html?Sos), [phyl](http://flybase.bio.indiana.edu/.bin/fbidq.html?phyl),[vg](http://flybase.bio.indiana.edu/.bin/fbidq.html?vg), [eya](http://flybase.bio.indiana.edu/.bin/fbidq.html?eya), [Fas2](http://flybase.bio.indiana.edu/.bin/fbidq.html?Fas2), [fz](http://flybase.bio.indiana.edu/.bin/fbidq.html?fz), [robo](http://flybase.bio.indiana.edu/.bin/fbidq.html?robo), [CG8557](http://flybase.bio.indiana.edu/.bin/fbidq.html?CG8557), [bsk](http://flybase.bio.indiana.edu/.bin/fbidq.html?bsk), [brm](http://flybase.bio.indiana.edu/.bin/fbidq.html?brm), [Vsx2](http://flybase.bio.indiana.edu/.bin/fbidq.html?Vsx2), [beat-Ia](http://flybase.bio.indiana.edu/.bin/fbidq.html?beat-Ia), [ft](http://flybase.bio.indiana.edu/.bin/fbidq.html?ft), [CG42268](http://flybase.bio.indiana.edu/.bin/fbidq.html?CG42268), [kat-60L1](http://flybase.bio.indiana.edu/.bin/fbidq.html?kat-60L1), [lft](http://flybase.bio.indiana.edu/.bin/fbidq.html?lft), [sno](http://flybase.bio.indiana.edu/.bin/fbidq.html?sno), [hig](http://flybase.bio.indiana.edu/.bin/fbidq.html?hig), [CG12004](http://flybase.bio.indiana.edu/.bin/fbidq.html?CG12004), [RhoGAPp190](http://flybase.bio.indiana.edu/.bin/fbidq.html?RhoGAPp190), [Sfmbt](http://flybase.bio.indiana.edu/.bin/fbidq.html?Sfmbt), [ara](http://flybase.bio.indiana.edu/.bin/fbidq.html?ara), [mam](http://flybase.bio.indiana.edu/.bin/fbidq.html?mam), [pan](http://flybase.bio.indiana.edu/.bin/fbidq.html?pan), [sws](http://flybase.bio.indiana.edu/.bin/fbidq.html?sws), [disco](http://flybase.bio.indiana.edu/.bin/fbidq.html?disco), [Dll](http://flybase.bio.indiana.edu/.bin/fbidq.html?Dll), [grn](http://flybase.bio.indiana.edu/.bin/fbidq.html?grn), [HLHm5](http://flybase.bio.indiana.edu/.bin/fbidq.html?HLHm5), [mew](http://flybase.bio.indiana.edu/.bin/fbidq.html?mew), [didum](http://flybase.bio.indiana.edu/.bin/fbidq.html?didum), [Lis-1](http://flybase.bio.indiana.edu/.bin/fbidq.html?Lis-1), [dap](http://flybase.bio.indiana.edu/.bin/fbidq.html?dap), [bmm](http://flybase.bio.indiana.edu/.bin/fbidq.html?bmm), [ems](http://flybase.bio.indiana.edu/.bin/fbidq.html?ems), [eys](http://flybase.bio.indiana.edu/.bin/fbidq.html?eys), [Bx](http://flybase.bio.indiana.edu/.bin/fbidq.html?Bx), [Mob2](http://flybase.bio.indiana.edu/.bin/fbidq.html?Mob2), [pros](http://flybase.bio.indiana.edu/.bin/fbidq.html?pros), [tup](http://flybase.bio.indiana.edu/.bin/fbidq.html?tup), [trio](http://flybase.bio.indiana.edu/.bin/fbidq.html?trio),[pdm2](http://flybase.bio.indiana.edu/.bin/fbidq.html?pdm2), [mthl8](http://flybase.bio.indiana.edu/.bin/fbidq.html?mthl8), [CG1463](http://flybase.bio.indiana.edu/.bin/fbidq.html?CG1463), [Gad1](http://flybase.bio.indiana.edu/.bin/fbidq.html?Gad1), [Wnt4](http://flybase.bio.indiana.edu/.bin/fbidq.html?Wnt4), [rictor](http://flybase.bio.indiana.edu/.bin/fbidq.html?rictor), [Oseg6](http://flybase.bio.indiana.edu/.bin/fbidq.html?Oseg6), [Glut4EF](http://flybase.bio.indiana.edu/.bin/fbidq.html?Glut4EF), [CaMKII](http://flybase.bio.indiana.edu/.bin/fbidq.html?CaMKII), [nerfin-1](http://flybase.bio.indiana.edu/.bin/fbidq.html?nerfin-1), [pigs](http://flybase.bio.indiana.edu/.bin/fbidq.html?pigs), [bchs](http://flybase.bio.indiana.edu/.bin/fbidq.html?bchs), [polo](http://flybase.bio.indiana.edu/.bin/fbidq.html?polo), [pk](http://flybase.bio.indiana.edu/.bin/fbidq.html?pk), [dsx](http://flybase.bio.indiana.edu/.bin/fbidq.html?dsx), [chinmo](http://flybase.bio.indiana.edu/.bin/fbidq.html?chinmo), [Syn1](http://flybase.bio.indiana.edu/.bin/fbidq.html?Syn1), [betaTub85D](http://flybase.bio.indiana.edu/.bin/fbidq.html?betaTub85D), [Ssdp](http://flybase.bio.indiana.edu/.bin/fbidq.html?Ssdp), [klu](http://flybase.bio.indiana.edu/.bin/fbidq.html?klu), [sqz](http://flybase.bio.indiana.edu/.bin/fbidq.html?sqz), [CG8155](http://flybase.bio.indiana.edu/.bin/fbidq.html?CG8155), [fend](http://flybase.bio.indiana.edu/.bin/fbidq.html?fend), [Cad74A](http://flybase.bio.indiana.edu/.bin/fbidq.html?Cad74A), [Ca-alpha1D](http://flybase.bio.indiana.edu/.bin/fbidq.html?Ca-alpha1D), [sr](http://flybase.bio.indiana.edu/.bin/fbidq.html?sr), [Lim1](http://flybase.bio.indiana.edu/.bin/fbidq.html?Lim1), [nkd](http://flybase.bio.indiana.edu/.bin/fbidq.html?nkd), [tld](http://flybase.bio.indiana.edu/.bin/fbidq.html?tld), [danr](http://flybase.bio.indiana.edu/.bin/fbidq.html?danr), [Dsk](http://flybase.bio.indiana.edu/.bin/fbidq.html?Dsk), [RhoGEF4](http://flybase.bio.indiana.edu/.bin/fbidq.html?RhoGEF4), [vvl](http://flybase.bio.indiana.edu/.bin/fbidq.html?vvl), [rg](http://flybase.bio.indiana.edu/.bin/fbidq.html?rg), [betaInt-nu](http://flybase.bio.indiana.edu/.bin/fbidq.html?betaInt-nu), [sgg](http://flybase.bio.indiana.edu/.bin/fbidq.html?sgg), [Brd](http://flybase.bio.indiana.edu/.bin/fbidq.html?Brd), [mnb](http://flybase.bio.indiana.edu/.bin/fbidq.html?mnb), [gcm2](http://flybase.bio.indiana.edu/.bin/fbidq.html?gcm2), [Nmdar1](http://flybase.bio.indiana.edu/.bin/fbidq.html?Nmdar1), [Eip93F](http://flybase.bio.indiana.edu/.bin/fbidq.html?Eip93F), [mp](http://flybase.bio.indiana.edu/.bin/fbidq.html?mp), [gcm](http://flybase.bio.indiana.edu/.bin/fbidq.html?gcm), [CG5890](http://flybase.bio.indiana.edu/.bin/fbidq.html?CG5890), [SP2353](http://flybase.bio.indiana.edu/.bin/fbidq.html?SP2353), [can](http://flybase.bio.indiana.edu/.bin/fbidq.html?can), [ase](http://flybase.bio.indiana.edu/.bin/fbidq.html?ase), [CG13253](http://flybase.bio.indiana.edu/.bin/fbidq.html?CG13253), [ovo](http://flybase.bio.indiana.edu/.bin/fbidq.html?ovo), [CG32685](http://flybase.bio.indiana.edu/.bin/fbidq.html?CG32685), [Dsp1](http://flybase.bio.indiana.edu/.bin/fbidq.html?Dsp1), [ds](http://flybase.bio.indiana.edu/.bin/fbidq.html?ds), [mei-P26](http://flybase.bio.indiana.edu/.bin/fbidq.html?mei-P26), [Poxn](http://flybase.bio.indiana.edu/.bin/fbidq.html?Poxn), [sals](http://flybase.bio.indiana.edu/.bin/fbidq.html?sals), [Rbp9](http://flybase.bio.indiana.edu/.bin/fbidq.html?Rbp9), [SIFR](http://flybase.bio.indiana.edu/.bin/fbidq.html?SIFR), [eIF4G2](http://flybase.bio.indiana.edu/.bin/fbidq.html?eIF4G2), [fred](http://flybase.bio.indiana.edu/.bin/fbidq.html?fred), [Drl-2](http://flybase.bio.indiana.edu/.bin/fbidq.html?Drl-2), [CG32944](http://flybase.bio.indiana.edu/.bin/fbidq.html?CG32944), [MYPT-75D](http://flybase.bio.indiana.edu/.bin/fbidq.html?MYPT-75D), [ush](http://flybase.bio.indiana.edu/.bin/fbidq.html?ush), [Fak56D](http://flybase.bio.indiana.edu/.bin/fbidq.html?Fak56D), [Gap1](http://flybase.bio.indiana.edu/.bin/fbidq.html?Gap1), [run](http://flybase.bio.indiana.edu/.bin/fbidq.html?run), [Kap3](http://flybase.bio.indiana.edu/.bin/fbidq.html?Kap3), [ko](http://flybase.bio.indiana.edu/.bin/fbidq.html?ko), [tara](http://flybase.bio.indiana.edu/.bin/fbidq.html?tara),[Eip71CD](http://flybase.bio.indiana.edu/.bin/fbidq.html?Eip71CD), [Alh](http://flybase.bio.indiana.edu/.bin/fbidq.html?Alh), [egg](http://flybase.bio.indiana.edu/.bin/fbidq.html?egg), [koko](http://flybase.bio.indiana.edu/.bin/fbidq.html?koko), [stan](http://flybase.bio.indiana.edu/.bin/fbidq.html?stan), [CG12424](http://flybase.bio.indiana.edu/.bin/fbidq.html?CG12424), [tutl](http://flybase.bio.indiana.edu/.bin/fbidq.html?tutl), [Ptth](http://flybase.bio.indiana.edu/.bin/fbidq.html?Ptth), [Sp1](http://flybase.bio.indiana.edu/.bin/fbidq.html?Sp1), [CG1753](http://flybase.bio.indiana.edu/.bin/fbidq.html?CG1753), [Ilp2](http://flybase.bio.indiana.edu/.bin/fbidq.html?Ilp2), [ph-p](http://flybase.bio.indiana.edu/.bin/fbidq.html?ph-p), [Frq1](http://flybase.bio.indiana.edu/.bin/fbidq.html?Frq1), [sna](http://flybase.bio.indiana.edu/.bin/fbidq.html?sna), [CG12972](http://flybase.bio.indiana.edu/.bin/fbidq.html?CG12972), [Wnt5](http://flybase.bio.indiana.edu/.bin/fbidq.html?Wnt5), [Ggamma1](http://flybase.bio.indiana.edu/.bin/fbidq.html?Ggamma1), [esg](http://flybase.bio.indiana.edu/.bin/fbidq.html?esg), [sif](http://flybase.bio.indiana.edu/.bin/fbidq.html?sif), [m4](http://flybase.bio.indiana.edu/.bin/fbidq.html?m4), [dom](http://flybase.bio.indiana.edu/.bin/fbidq.html?dom), [Sas-4](http://flybase.bio.indiana.edu/.bin/fbidq.html?Sas-4), [ssh](http://flybase.bio.indiana.edu/.bin/fbidq.html?ssh), [PIP5K59B](http://flybase.bio.indiana.edu/.bin/fbidq.html?PIP5K59B), [klar](http://flybase.bio.indiana.edu/.bin/fbidq.html?klar), [mle](http://flybase.bio.indiana.edu/.bin/fbidq.html?mle), [Hr51](http://flybase.bio.indiana.edu/.bin/fbidq.html?Hr51), [shi](http://flybase.bio.indiana.edu/.bin/fbidq.html?shi), [CG11155](http://flybase.bio.indiana.edu/.bin/fbidq.html?CG11155), [siz](http://flybase.bio.indiana.edu/.bin/fbidq.html?siz), [B-H2](http://flybase.bio.indiana.edu/.bin/fbidq.html?B-H2), [tyn](http://flybase.bio.indiana.edu/.bin/fbidq.html?tyn), [wg](http://flybase.bio.indiana.edu/.bin/fbidq.html?wg), [fkh](http://flybase.bio.indiana.edu/.bin/fbidq.html?fkh), [CadN](http://flybase.bio.indiana.edu/.bin/fbidq.html?CadN), [klg](http://flybase.bio.indiana.edu/.bin/fbidq.html?klg), [en](http://flybase.bio.indiana.edu/.bin/fbidq.html?en), [E(bx)](http://flybase.bio.indiana.edu/.bin/fbidq.html?E(bx)), [sfl](http://flybase.bio.indiana.edu/.bin/fbidq.html?sfl),[Sip1](http://flybase.bio.indiana.edu/.bin/fbidq.html?Sip1), [Snap](http://flybase.bio.indiana.edu/.bin/fbidq.html?Snap), [trn](http://flybase.bio.indiana.edu/.bin/fbidq.html?trn), [Pitslre](http://flybase.bio.indiana.edu/.bin/fbidq.html?Pitslre), [Utx](http://flybase.bio.indiana.edu/.bin/fbidq.html?Utx), [bt](http://flybase.bio.indiana.edu/.bin/fbidq.html?bt), [pburs](http://flybase.bio.indiana.edu/.bin/fbidq.html?pburs), [cos](http://flybase.bio.indiana.edu/.bin/fbidq.html?cos), [Ct](http://flybase.bio.indiana.edu/.bin/fbidq.html?Ct) |
| [multi-organism reproductive process](http://amigo.geneontology.org/cgi-bin/amigo/go.cgi?view=details&query=GO:0044703) | 30 of 1594 genes, 1.9% | 62 of 7634 genes, 0.8% | 0.00086 | 0.00% | 0.00 | [tipE](http://flybase.bio.indiana.edu/.bin/fbidq.html?tipE), [Sh](http://flybase.bio.indiana.edu/.bin/fbidq.html?Sh), [Ilp2](http://flybase.bio.indiana.edu/.bin/fbidq.html?Ilp2), [Poxn](http://flybase.bio.indiana.edu/.bin/fbidq.html?Poxn), [qtc](http://flybase.bio.indiana.edu/.bin/fbidq.html?qtc), [Pde1c](http://flybase.bio.indiana.edu/.bin/fbidq.html?Pde1c), [Cbp80](http://flybase.bio.indiana.edu/.bin/fbidq.html?Cbp80), [CaMKII](http://flybase.bio.indiana.edu/.bin/fbidq.html?CaMKII), [npf](http://flybase.bio.indiana.edu/.bin/fbidq.html?npf), [ato](http://flybase.bio.indiana.edu/.bin/fbidq.html?ato), [fne](http://flybase.bio.indiana.edu/.bin/fbidq.html?fne), [Ilp3](http://flybase.bio.indiana.edu/.bin/fbidq.html?Ilp3), [para](http://flybase.bio.indiana.edu/.bin/fbidq.html?para), [retn](http://flybase.bio.indiana.edu/.bin/fbidq.html?retn), [5-HT7](http://flybase.bio.indiana.edu/.bin/fbidq.html?5-HT7), [rut](http://flybase.bio.indiana.edu/.bin/fbidq.html?rut), [Btk29A](http://flybase.bio.indiana.edu/.bin/fbidq.html?Btk29A), [dsx](http://flybase.bio.indiana.edu/.bin/fbidq.html?dsx), [Nrg](http://flybase.bio.indiana.edu/.bin/fbidq.html?Nrg), [Ilp5](http://flybase.bio.indiana.edu/.bin/fbidq.html?Ilp5), [Ddc](http://flybase.bio.indiana.edu/.bin/fbidq.html?Ddc), [SPR](http://flybase.bio.indiana.edu/.bin/fbidq.html?SPR), [pros](http://flybase.bio.indiana.edu/.bin/fbidq.html?pros), [Tbh](http://flybase.bio.indiana.edu/.bin/fbidq.html?Tbh), [Oamb](http://flybase.bio.indiana.edu/.bin/fbidq.html?Oamb), [mle](http://flybase.bio.indiana.edu/.bin/fbidq.html?mle), [orb2](http://flybase.bio.indiana.edu/.bin/fbidq.html?orb2), [nonA](http://flybase.bio.indiana.edu/.bin/fbidq.html?nonA), [CG10251](http://flybase.bio.indiana.edu/.bin/fbidq.html?CG10251), [CASK](http://flybase.bio.indiana.edu/.bin/fbidq.html?CASK) |
| [brain development](http://amigo.geneontology.org/cgi-bin/amigo/go.cgi?view=details&query=GO:0007420) | 42 of 1594 genes, 2.6% | 101 of 7634 genes, 1.3% | 0.00091 | 0.00% | 0.00 | [fz2](http://flybase.bio.indiana.edu/.bin/fbidq.html?fz2), [hth](http://flybase.bio.indiana.edu/.bin/fbidq.html?hth), [Fas2](http://flybase.bio.indiana.edu/.bin/fbidq.html?Fas2), [fz](http://flybase.bio.indiana.edu/.bin/fbidq.html?fz), [robo](http://flybase.bio.indiana.edu/.bin/fbidq.html?robo), [lea](http://flybase.bio.indiana.edu/.bin/fbidq.html?lea), [wor](http://flybase.bio.indiana.edu/.bin/fbidq.html?wor), [vvl](http://flybase.bio.indiana.edu/.bin/fbidq.html?vvl), [rg](http://flybase.bio.indiana.edu/.bin/fbidq.html?rg), [RhoGAPp190](http://flybase.bio.indiana.edu/.bin/fbidq.html?RhoGAPp190), [ato](http://flybase.bio.indiana.edu/.bin/fbidq.html?ato), [mnb](http://flybase.bio.indiana.edu/.bin/fbidq.html?mnb), [sws](http://flybase.bio.indiana.edu/.bin/fbidq.html?sws), [Dll](http://flybase.bio.indiana.edu/.bin/fbidq.html?Dll), [disco](http://flybase.bio.indiana.edu/.bin/fbidq.html?disco), [neur](http://flybase.bio.indiana.edu/.bin/fbidq.html?neur), [Lis-1](http://flybase.bio.indiana.edu/.bin/fbidq.html?Lis-1), [eas](http://flybase.bio.indiana.edu/.bin/fbidq.html?eas), [dac](http://flybase.bio.indiana.edu/.bin/fbidq.html?dac), [ems](http://flybase.bio.indiana.edu/.bin/fbidq.html?ems), [oc](http://flybase.bio.indiana.edu/.bin/fbidq.html?oc), [pros](http://flybase.bio.indiana.edu/.bin/fbidq.html?pros), [trio](http://flybase.bio.indiana.edu/.bin/fbidq.html?trio), [robo3](http://flybase.bio.indiana.edu/.bin/fbidq.html?robo3), [ey](http://flybase.bio.indiana.edu/.bin/fbidq.html?ey), [ssh](http://flybase.bio.indiana.edu/.bin/fbidq.html?ssh), [ed](http://flybase.bio.indiana.edu/.bin/fbidq.html?ed), [Hr51](http://flybase.bio.indiana.edu/.bin/fbidq.html?Hr51), [lola](http://flybase.bio.indiana.edu/.bin/fbidq.html?lola), [Rx](http://flybase.bio.indiana.edu/.bin/fbidq.html?Rx), [toy](http://flybase.bio.indiana.edu/.bin/fbidq.html?toy), [wg](http://flybase.bio.indiana.edu/.bin/fbidq.html?wg), [Apc](http://flybase.bio.indiana.edu/.bin/fbidq.html?Apc), [sim](http://flybase.bio.indiana.edu/.bin/fbidq.html?sim), [Smox](http://flybase.bio.indiana.edu/.bin/fbidq.html?Smox), [en](http://flybase.bio.indiana.edu/.bin/fbidq.html?en), [Nrg](http://flybase.bio.indiana.edu/.bin/fbidq.html?Nrg), [chinmo](http://flybase.bio.indiana.edu/.bin/fbidq.html?chinmo), [brat](http://flybase.bio.indiana.edu/.bin/fbidq.html?brat), [Sema-1a](http://flybase.bio.indiana.edu/.bin/fbidq.html?Sema-1a), [HLHm7](http://flybase.bio.indiana.edu/.bin/fbidq.html?HLHm7), [stan](http://flybase.bio.indiana.edu/.bin/fbidq.html?stan) |
| [negative regulation of nucleobase-containing compound metabolic process](http://amigo.geneontology.org/cgi-bin/amigo/go.cgi?view=details&query=GO:0045934) | 74 of 1594 genes, 4.6% | 217 of 7634 genes, 2.8% | 0.00093 | 0.00% | 0.00 | [jing](http://flybase.bio.indiana.edu/.bin/fbidq.html?jing), [Sxl](http://flybase.bio.indiana.edu/.bin/fbidq.html?Sxl), [JIL-1](http://flybase.bio.indiana.edu/.bin/fbidq.html?JIL-1), [rn](http://flybase.bio.indiana.edu/.bin/fbidq.html?rn), [Iswi](http://flybase.bio.indiana.edu/.bin/fbidq.html?Iswi), [seq](http://flybase.bio.indiana.edu/.bin/fbidq.html?seq), [Aef1](http://flybase.bio.indiana.edu/.bin/fbidq.html?Aef1), [ovo](http://flybase.bio.indiana.edu/.bin/fbidq.html?ovo), [elav](http://flybase.bio.indiana.edu/.bin/fbidq.html?elav), [Dsp1](http://flybase.bio.indiana.edu/.bin/fbidq.html?Dsp1), [bi](http://flybase.bio.indiana.edu/.bin/fbidq.html?bi), [chn](http://flybase.bio.indiana.edu/.bin/fbidq.html?chn), [Gsc](http://flybase.bio.indiana.edu/.bin/fbidq.html?Gsc), [Nelf-A](http://flybase.bio.indiana.edu/.bin/fbidq.html?Nelf-A), [Pcl](http://flybase.bio.indiana.edu/.bin/fbidq.html?Pcl), [simj](http://flybase.bio.indiana.edu/.bin/fbidq.html?simj), [Sin3A](http://flybase.bio.indiana.edu/.bin/fbidq.html?Sin3A), [NC2alpha](http://flybase.bio.indiana.edu/.bin/fbidq.html?NC2alpha), [mip120](http://flybase.bio.indiana.edu/.bin/fbidq.html?mip120), [Snoo](http://flybase.bio.indiana.edu/.bin/fbidq.html?Snoo), [jumu](http://flybase.bio.indiana.edu/.bin/fbidq.html?jumu), [HGTX](http://flybase.bio.indiana.edu/.bin/fbidq.html?HGTX), [ush](http://flybase.bio.indiana.edu/.bin/fbidq.html?ush), [Usp7](http://flybase.bio.indiana.edu/.bin/fbidq.html?Usp7), [fd59A](http://flybase.bio.indiana.edu/.bin/fbidq.html?fd59A), [nab](http://flybase.bio.indiana.edu/.bin/fbidq.html?nab), [CG10336](http://flybase.bio.indiana.edu/.bin/fbidq.html?CG10336), [btd](http://flybase.bio.indiana.edu/.bin/fbidq.html?btd), [fd68A](http://flybase.bio.indiana.edu/.bin/fbidq.html?fd68A), [HLHmdelta](http://flybase.bio.indiana.edu/.bin/fbidq.html?HLHmdelta), [run](http://flybase.bio.indiana.edu/.bin/fbidq.html?run), [B-H1](http://flybase.bio.indiana.edu/.bin/fbidq.html?B-H1), [FBX011](http://flybase.bio.indiana.edu/.bin/fbidq.html?FBX011), [egg](http://flybase.bio.indiana.edu/.bin/fbidq.html?egg), [l(3)neo38](http://flybase.bio.indiana.edu/.bin/fbidq.html?l(3)neo38), [fd102C](http://flybase.bio.indiana.edu/.bin/fbidq.html?fd102C), [Vsx2](http://flybase.bio.indiana.edu/.bin/fbidq.html?Vsx2),[ph-p](http://flybase.bio.indiana.edu/.bin/fbidq.html?ph-p), [Ubx](http://flybase.bio.indiana.edu/.bin/fbidq.html?Ubx), [CG7757](http://flybase.bio.indiana.edu/.bin/fbidq.html?CG7757), [chm](http://flybase.bio.indiana.edu/.bin/fbidq.html?chm), [sna](http://flybase.bio.indiana.edu/.bin/fbidq.html?sna), [Sfmbt](http://flybase.bio.indiana.edu/.bin/fbidq.html?Sfmbt), [Wnt5](http://flybase.bio.indiana.edu/.bin/fbidq.html?Wnt5), [pan](http://flybase.bio.indiana.edu/.bin/fbidq.html?pan), [MBD-like](http://flybase.bio.indiana.edu/.bin/fbidq.html?MBD-like), [tsh](http://flybase.bio.indiana.edu/.bin/fbidq.html?tsh), [HLHm5](http://flybase.bio.indiana.edu/.bin/fbidq.html?HLHm5), [esg](http://flybase.bio.indiana.edu/.bin/fbidq.html?esg), [e(y)3](http://flybase.bio.indiana.edu/.bin/fbidq.html?e(y)3), [oc](http://flybase.bio.indiana.edu/.bin/fbidq.html?oc), [pros](http://flybase.bio.indiana.edu/.bin/fbidq.html?pros), [Psi](http://flybase.bio.indiana.edu/.bin/fbidq.html?Psi), [Gug](http://flybase.bio.indiana.edu/.bin/fbidq.html?Gug), [pad](http://flybase.bio.indiana.edu/.bin/fbidq.html?pad), [spel1](http://flybase.bio.indiana.edu/.bin/fbidq.html?spel1), [Wnt4](http://flybase.bio.indiana.edu/.bin/fbidq.html?Wnt4), [B-H2](http://flybase.bio.indiana.edu/.bin/fbidq.html?B-H2), [wg](http://flybase.bio.indiana.edu/.bin/fbidq.html?wg), [Vsx1](http://flybase.bio.indiana.edu/.bin/fbidq.html?Vsx1), [fkh](http://flybase.bio.indiana.edu/.bin/fbidq.html?fkh), [gro](http://flybase.bio.indiana.edu/.bin/fbidq.html?gro), [crol](http://flybase.bio.indiana.edu/.bin/fbidq.html?crol), [Rpd3](http://flybase.bio.indiana.edu/.bin/fbidq.html?Rpd3), [retn](http://flybase.bio.indiana.edu/.bin/fbidq.html?retn), [fs(1)h](http://flybase.bio.indiana.edu/.bin/fbidq.html?fs(1)h), [en](http://flybase.bio.indiana.edu/.bin/fbidq.html?en), [dsx](http://flybase.bio.indiana.edu/.bin/fbidq.html?dsx), [tey](http://flybase.bio.indiana.edu/.bin/fbidq.html?tey), [HLHmgamma](http://flybase.bio.indiana.edu/.bin/fbidq.html?HLHmgamma), [z](http://flybase.bio.indiana.edu/.bin/fbidq.html?z), [HLHm7](http://flybase.bio.indiana.edu/.bin/fbidq.html?HLHm7), [Lim1](http://flybase.bio.indiana.edu/.bin/fbidq.html?Lim1), [Ct](http://flybase.bio.indiana.edu/.bin/fbidq.html?Ct) |
| [system development](http://amigo.geneontology.org/cgi-bin/amigo/go.cgi?view=details&query=GO:0048731) | 421 of 1594 genes, 26.4% | 1750 of 7634 genes, 22.9% | 0.00098 | 0.00% | 0.00 | [fz2](http://flybase.bio.indiana.edu/.bin/fbidq.html?fz2), [Ocho](http://flybase.bio.indiana.edu/.bin/fbidq.html?Ocho), [Sh](http://flybase.bio.indiana.edu/.bin/fbidq.html?Sh), [numb](http://flybase.bio.indiana.edu/.bin/fbidq.html?numb), [dock](http://flybase.bio.indiana.edu/.bin/fbidq.html?dock), [CG5808](http://flybase.bio.indiana.edu/.bin/fbidq.html?CG5808), [CG9004](http://flybase.bio.indiana.edu/.bin/fbidq.html?CG9004), [CG42256](http://flybase.bio.indiana.edu/.bin/fbidq.html?CG42256), [CG3703](http://flybase.bio.indiana.edu/.bin/fbidq.html?CG3703), [stj](http://flybase.bio.indiana.edu/.bin/fbidq.html?stj), [Sxl](http://flybase.bio.indiana.edu/.bin/fbidq.html?Sxl), [CG33007](http://flybase.bio.indiana.edu/.bin/fbidq.html?CG33007), [rn](http://flybase.bio.indiana.edu/.bin/fbidq.html?rn), [ap](http://flybase.bio.indiana.edu/.bin/fbidq.html?ap), [kkv](http://flybase.bio.indiana.edu/.bin/fbidq.html?kkv), [Nf1](http://flybase.bio.indiana.edu/.bin/fbidq.html?Nf1), [Drep-3](http://flybase.bio.indiana.edu/.bin/fbidq.html?Drep-3), [malpha](http://flybase.bio.indiana.edu/.bin/fbidq.html?malpha), [sns](http://flybase.bio.indiana.edu/.bin/fbidq.html?sns), [sol](http://flybase.bio.indiana.edu/.bin/fbidq.html?sol), [os](http://flybase.bio.indiana.edu/.bin/fbidq.html?os), [Axn](http://flybase.bio.indiana.edu/.bin/fbidq.html?Axn), [tok](http://flybase.bio.indiana.edu/.bin/fbidq.html?tok), [bi](http://flybase.bio.indiana.edu/.bin/fbidq.html?bi), [Ptp99A](http://flybase.bio.indiana.edu/.bin/fbidq.html?Ptp99A), [Traf6](http://flybase.bio.indiana.edu/.bin/fbidq.html?Traf6), [CG31961](http://flybase.bio.indiana.edu/.bin/fbidq.html?CG31961), [NetB](http://flybase.bio.indiana.edu/.bin/fbidq.html?NetB), [Tango10](http://flybase.bio.indiana.edu/.bin/fbidq.html?Tango10), [Oseg4](http://flybase.bio.indiana.edu/.bin/fbidq.html?Oseg4), [cid](http://flybase.bio.indiana.edu/.bin/fbidq.html?cid), [CG32137](http://flybase.bio.indiana.edu/.bin/fbidq.html?CG32137), [lute](http://flybase.bio.indiana.edu/.bin/fbidq.html?lute), [bab1](http://flybase.bio.indiana.edu/.bin/fbidq.html?bab1), [Ssrp](http://flybase.bio.indiana.edu/.bin/fbidq.html?Ssrp), [Galpha49B](http://flybase.bio.indiana.edu/.bin/fbidq.html?Galpha49B),[Snoo](http://flybase.bio.indiana.edu/.bin/fbidq.html?Snoo), [Lar](http://flybase.bio.indiana.edu/.bin/fbidq.html?Lar), [scrib](http://flybase.bio.indiana.edu/.bin/fbidq.html?scrib), [HGTX](http://flybase.bio.indiana.edu/.bin/fbidq.html?HGTX), [not](http://flybase.bio.indiana.edu/.bin/fbidq.html?not), [rut](http://flybase.bio.indiana.edu/.bin/fbidq.html?rut), [nvy](http://flybase.bio.indiana.edu/.bin/fbidq.html?nvy), [Ect3](http://flybase.bio.indiana.edu/.bin/fbidq.html?Ect3), [nub](http://flybase.bio.indiana.edu/.bin/fbidq.html?nub), [uif](http://flybase.bio.indiana.edu/.bin/fbidq.html?uif), [nab](http://flybase.bio.indiana.edu/.bin/fbidq.html?nab), [Ddc](http://flybase.bio.indiana.edu/.bin/fbidq.html?Ddc), [btd](http://flybase.bio.indiana.edu/.bin/fbidq.html?btd), [Mmp2](http://flybase.bio.indiana.edu/.bin/fbidq.html?Mmp2), [HLHmdelta](http://flybase.bio.indiana.edu/.bin/fbidq.html?HLHmdelta), [B-H1](http://flybase.bio.indiana.edu/.bin/fbidq.html?B-H1), [Nipped-A](http://flybase.bio.indiana.edu/.bin/fbidq.html?Nipped-A), [pim](http://flybase.bio.indiana.edu/.bin/fbidq.html?pim), [alph](http://flybase.bio.indiana.edu/.bin/fbidq.html?alph), [Traf4](http://flybase.bio.indiana.edu/.bin/fbidq.html?Traf4), [TBPH](http://flybase.bio.indiana.edu/.bin/fbidq.html?TBPH), [Fer2](http://flybase.bio.indiana.edu/.bin/fbidq.html?Fer2), [CG34400](http://flybase.bio.indiana.edu/.bin/fbidq.html?CG34400), [Ulp1](http://flybase.bio.indiana.edu/.bin/fbidq.html?Ulp1), [mGluRA](http://flybase.bio.indiana.edu/.bin/fbidq.html?mGluRA), [ena](http://flybase.bio.indiana.edu/.bin/fbidq.html?ena), [dsh](http://flybase.bio.indiana.edu/.bin/fbidq.html?dsh), [hth](http://flybase.bio.indiana.edu/.bin/fbidq.html?hth), [pzg](http://flybase.bio.indiana.edu/.bin/fbidq.html?pzg), [Nf-YC](http://flybase.bio.indiana.edu/.bin/fbidq.html?Nf-YC), [14-3-3zeta](http://flybase.bio.indiana.edu/.bin/fbidq.html?14-3-3zeta), [lea](http://flybase.bio.indiana.edu/.bin/fbidq.html?lea), [qkr58E-1](http://flybase.bio.indiana.edu/.bin/fbidq.html?qkr58E-1), [plexB](http://flybase.bio.indiana.edu/.bin/fbidq.html?plexB), [PP2A-B](http://flybase.bio.indiana.edu/.bin/fbidq.html?PP2A-B), [loqs](http://flybase.bio.indiana.edu/.bin/fbidq.html?loqs),[Klp64D](http://flybase.bio.indiana.edu/.bin/fbidq.html?Klp64D), [DAAM](http://flybase.bio.indiana.edu/.bin/fbidq.html?DAAM), [neur](http://flybase.bio.indiana.edu/.bin/fbidq.html?neur), [CycE](http://flybase.bio.indiana.edu/.bin/fbidq.html?CycE), [Brf](http://flybase.bio.indiana.edu/.bin/fbidq.html?Brf), [nmo](http://flybase.bio.indiana.edu/.bin/fbidq.html?nmo), [CG32149](http://flybase.bio.indiana.edu/.bin/fbidq.html?CG32149), [Gug](http://flybase.bio.indiana.edu/.bin/fbidq.html?Gug), [ed](http://flybase.bio.indiana.edu/.bin/fbidq.html?ed), [pad](http://flybase.bio.indiana.edu/.bin/fbidq.html?pad), [mr](http://flybase.bio.indiana.edu/.bin/fbidq.html?mr), [CG11317](http://flybase.bio.indiana.edu/.bin/fbidq.html?CG11317), [daw](http://flybase.bio.indiana.edu/.bin/fbidq.html?daw), [ImpE2](http://flybase.bio.indiana.edu/.bin/fbidq.html?ImpE2), [Rx](http://flybase.bio.indiana.edu/.bin/fbidq.html?Rx), [salr](http://flybase.bio.indiana.edu/.bin/fbidq.html?salr), [synaptogyrin](http://flybase.bio.indiana.edu/.bin/fbidq.html?synaptogyrin), [Apc](http://flybase.bio.indiana.edu/.bin/fbidq.html?Apc), [sim](http://flybase.bio.indiana.edu/.bin/fbidq.html?sim), [d4](http://flybase.bio.indiana.edu/.bin/fbidq.html?d4), [dan](http://flybase.bio.indiana.edu/.bin/fbidq.html?dan), [gro](http://flybase.bio.indiana.edu/.bin/fbidq.html?gro), [Mf](http://flybase.bio.indiana.edu/.bin/fbidq.html?Mf), [wnd](http://flybase.bio.indiana.edu/.bin/fbidq.html?wnd), [trx](http://flybase.bio.indiana.edu/.bin/fbidq.html?trx), [rdo](http://flybase.bio.indiana.edu/.bin/fbidq.html?rdo), [Rpd3](http://flybase.bio.indiana.edu/.bin/fbidq.html?Rpd3), [Btk29A](http://flybase.bio.indiana.edu/.bin/fbidq.html?Btk29A), [SMC2](http://flybase.bio.indiana.edu/.bin/fbidq.html?SMC2), [MED25](http://flybase.bio.indiana.edu/.bin/fbidq.html?MED25), [unc-5](http://flybase.bio.indiana.edu/.bin/fbidq.html?unc-5), [cdi](http://flybase.bio.indiana.edu/.bin/fbidq.html?cdi), [CG10107](http://flybase.bio.indiana.edu/.bin/fbidq.html?CG10107), [barr](http://flybase.bio.indiana.edu/.bin/fbidq.html?barr), [aret](http://flybase.bio.indiana.edu/.bin/fbidq.html?aret), [pygo](http://flybase.bio.indiana.edu/.bin/fbidq.html?pygo), [HLHm7](http://flybase.bio.indiana.edu/.bin/fbidq.html?HLHm7),[HLHmgamma](http://flybase.bio.indiana.edu/.bin/fbidq.html?HLHmgamma), [spag](http://flybase.bio.indiana.edu/.bin/fbidq.html?spag), [jar](http://flybase.bio.indiana.edu/.bin/fbidq.html?jar), [Ror](http://flybase.bio.indiana.edu/.bin/fbidq.html?Ror), [tow](http://flybase.bio.indiana.edu/.bin/fbidq.html?tow), [CG15609](http://flybase.bio.indiana.edu/.bin/fbidq.html?CG15609), [wor](http://flybase.bio.indiana.edu/.bin/fbidq.html?wor), [Atg1](http://flybase.bio.indiana.edu/.bin/fbidq.html?Atg1), [l(1)G0148](http://flybase.bio.indiana.edu/.bin/fbidq.html?l(1)G0148), [Pten](http://flybase.bio.indiana.edu/.bin/fbidq.html?Pten), [Fcp1](http://flybase.bio.indiana.edu/.bin/fbidq.html?Fcp1), [mira](http://flybase.bio.indiana.edu/.bin/fbidq.html?mira), [Ptp69D](http://flybase.bio.indiana.edu/.bin/fbidq.html?Ptp69D), [hts](http://flybase.bio.indiana.edu/.bin/fbidq.html?hts), [pUf68](http://flybase.bio.indiana.edu/.bin/fbidq.html?pUf68), [bowl](http://flybase.bio.indiana.edu/.bin/fbidq.html?bowl), [seq](http://flybase.bio.indiana.edu/.bin/fbidq.html?seq), [Dip3](http://flybase.bio.indiana.edu/.bin/fbidq.html?Dip3), [Drep-2](http://flybase.bio.indiana.edu/.bin/fbidq.html?Drep-2), [oa2](http://flybase.bio.indiana.edu/.bin/fbidq.html?oa2), [RpS11](http://flybase.bio.indiana.edu/.bin/fbidq.html?RpS11), [phl](http://flybase.bio.indiana.edu/.bin/fbidq.html?phl), [CG1671](http://flybase.bio.indiana.edu/.bin/fbidq.html?CG1671), [Cbl](http://flybase.bio.indiana.edu/.bin/fbidq.html?Cbl), [thr](http://flybase.bio.indiana.edu/.bin/fbidq.html?thr), [CG4893](http://flybase.bio.indiana.edu/.bin/fbidq.html?CG4893), [CG9098](http://flybase.bio.indiana.edu/.bin/fbidq.html?CG9098), [Alk](http://flybase.bio.indiana.edu/.bin/fbidq.html?Alk), [esn](http://flybase.bio.indiana.edu/.bin/fbidq.html?esn), [Vang](http://flybase.bio.indiana.edu/.bin/fbidq.html?Vang), [Sin3A](http://flybase.bio.indiana.edu/.bin/fbidq.html?Sin3A), [pot](http://flybase.bio.indiana.edu/.bin/fbidq.html?pot), [cpx](http://flybase.bio.indiana.edu/.bin/fbidq.html?cpx), [jumu](http://flybase.bio.indiana.edu/.bin/fbidq.html?jumu), [Smox](http://flybase.bio.indiana.edu/.bin/fbidq.html?Smox), [Su(var)2-HP2](http://flybase.bio.indiana.edu/.bin/fbidq.html?Su(var)2-HP2), [CG31475](http://flybase.bio.indiana.edu/.bin/fbidq.html?CG31475), [fd59A](http://flybase.bio.indiana.edu/.bin/fbidq.html?fd59A), [beat-Ic](http://flybase.bio.indiana.edu/.bin/fbidq.html?beat-Ic), [brat](http://flybase.bio.indiana.edu/.bin/fbidq.html?brat), [ST6Gal](http://flybase.bio.indiana.edu/.bin/fbidq.html?ST6Gal), [Antp](http://flybase.bio.indiana.edu/.bin/fbidq.html?Antp), [glec](http://flybase.bio.indiana.edu/.bin/fbidq.html?glec), [l(3)neo38](http://flybase.bio.indiana.edu/.bin/fbidq.html?l(3)neo38), [cindr](http://flybase.bio.indiana.edu/.bin/fbidq.html?cindr), [futsch](http://flybase.bio.indiana.edu/.bin/fbidq.html?futsch), [comm3](http://flybase.bio.indiana.edu/.bin/fbidq.html?comm3), [Ubx](http://flybase.bio.indiana.edu/.bin/fbidq.html?Ubx), [Ptx1](http://flybase.bio.indiana.edu/.bin/fbidq.html?Ptx1), [gukh](http://flybase.bio.indiana.edu/.bin/fbidq.html?gukh), [chm](http://flybase.bio.indiana.edu/.bin/fbidq.html?chm), [CG6114](http://flybase.bio.indiana.edu/.bin/fbidq.html?CG6114), [Rim](http://flybase.bio.indiana.edu/.bin/fbidq.html?Rim), [beat-IIa](http://flybase.bio.indiana.edu/.bin/fbidq.html?beat-IIa), [Gpo-1](http://flybase.bio.indiana.edu/.bin/fbidq.html?Gpo-1), [tsh](http://flybase.bio.indiana.edu/.bin/fbidq.html?tsh), [sm](http://flybase.bio.indiana.edu/.bin/fbidq.html?sm), [Syn2](http://flybase.bio.indiana.edu/.bin/fbidq.html?Syn2), [CG42574](http://flybase.bio.indiana.edu/.bin/fbidq.html?CG42574), [eas](http://flybase.bio.indiana.edu/.bin/fbidq.html?eas), [dac](http://flybase.bio.indiana.edu/.bin/fbidq.html?dac), [e(y)3](http://flybase.bio.indiana.edu/.bin/fbidq.html?e(y)3), [Nrx-1](http://flybase.bio.indiana.edu/.bin/fbidq.html?Nrx-1), [Cdk5](http://flybase.bio.indiana.edu/.bin/fbidq.html?Cdk5), [oc](http://flybase.bio.indiana.edu/.bin/fbidq.html?oc), [CG4641](http://flybase.bio.indiana.edu/.bin/fbidq.html?CG4641), [CG3964](http://flybase.bio.indiana.edu/.bin/fbidq.html?CG3964), [ey](http://flybase.bio.indiana.edu/.bin/fbidq.html?ey), [baz](http://flybase.bio.indiana.edu/.bin/fbidq.html?baz), [CG33275](http://flybase.bio.indiana.edu/.bin/fbidq.html?CG33275),[rst](http://flybase.bio.indiana.edu/.bin/fbidq.html?rst), [CG6398](http://flybase.bio.indiana.edu/.bin/fbidq.html?CG6398), [l(1)sc](http://flybase.bio.indiana.edu/.bin/fbidq.html?l(1)sc), [grh](http://flybase.bio.indiana.edu/.bin/fbidq.html?grh), [comm](http://flybase.bio.indiana.edu/.bin/fbidq.html?comm), [Cdk5alpha](http://flybase.bio.indiana.edu/.bin/fbidq.html?Cdk5alpha), [toy](http://flybase.bio.indiana.edu/.bin/fbidq.html?toy), [Lim3](http://flybase.bio.indiana.edu/.bin/fbidq.html?Lim3), [crol](http://flybase.bio.indiana.edu/.bin/fbidq.html?crol), [sd](http://flybase.bio.indiana.edu/.bin/fbidq.html?sd), [Aplip1](http://flybase.bio.indiana.edu/.bin/fbidq.html?Aplip1), [E(Pc)](http://flybase.bio.indiana.edu/.bin/fbidq.html?E(Pc)), [CG1239](http://flybase.bio.indiana.edu/.bin/fbidq.html?CG1239), [retn](http://flybase.bio.indiana.edu/.bin/fbidq.html?retn), [fs(1)h](http://flybase.bio.indiana.edu/.bin/fbidq.html?fs(1)h), [Eip63F-1](http://flybase.bio.indiana.edu/.bin/fbidq.html?Eip63F-1), [rl](http://flybase.bio.indiana.edu/.bin/fbidq.html?rl), [Nup153](http://flybase.bio.indiana.edu/.bin/fbidq.html?Nup153), [tey](http://flybase.bio.indiana.edu/.bin/fbidq.html?tey), [yrt](http://flybase.bio.indiana.edu/.bin/fbidq.html?yrt), [wls](http://flybase.bio.indiana.edu/.bin/fbidq.html?wls), [CG33960](http://flybase.bio.indiana.edu/.bin/fbidq.html?CG33960), [CG15765](http://flybase.bio.indiana.edu/.bin/fbidq.html?CG15765), [hiw](http://flybase.bio.indiana.edu/.bin/fbidq.html?hiw), [unk](http://flybase.bio.indiana.edu/.bin/fbidq.html?unk), [CG32653](http://flybase.bio.indiana.edu/.bin/fbidq.html?CG32653), [dnr1](http://flybase.bio.indiana.edu/.bin/fbidq.html?dnr1), [Pabp2](http://flybase.bio.indiana.edu/.bin/fbidq.html?Pabp2), [acj6](http://flybase.bio.indiana.edu/.bin/fbidq.html?acj6), [skd](http://flybase.bio.indiana.edu/.bin/fbidq.html?skd), [jing](http://flybase.bio.indiana.edu/.bin/fbidq.html?jing), [katanin-60](http://flybase.bio.indiana.edu/.bin/fbidq.html?katanin-60), [caup](http://flybase.bio.indiana.edu/.bin/fbidq.html?caup), [Cbp80](http://flybase.bio.indiana.edu/.bin/fbidq.html?Cbp80), [tll](http://flybase.bio.indiana.edu/.bin/fbidq.html?tll), [vimar](http://flybase.bio.indiana.edu/.bin/fbidq.html?vimar),[ato](http://flybase.bio.indiana.edu/.bin/fbidq.html?ato), [Iswi](http://flybase.bio.indiana.edu/.bin/fbidq.html?Iswi), [Dg](http://flybase.bio.indiana.edu/.bin/fbidq.html?Dg), [Trim9](http://flybase.bio.indiana.edu/.bin/fbidq.html?Trim9), [CG5742](http://flybase.bio.indiana.edu/.bin/fbidq.html?CG5742), [CG14478](http://flybase.bio.indiana.edu/.bin/fbidq.html?CG14478), [CG34127](http://flybase.bio.indiana.edu/.bin/fbidq.html?CG34127), [br](http://flybase.bio.indiana.edu/.bin/fbidq.html?br), [CG7154](http://flybase.bio.indiana.edu/.bin/fbidq.html?CG7154), [ksr](http://flybase.bio.indiana.edu/.bin/fbidq.html?ksr), [robo3](http://flybase.bio.indiana.edu/.bin/fbidq.html?robo3), [chn](http://flybase.bio.indiana.edu/.bin/fbidq.html?chn), [lola](http://flybase.bio.indiana.edu/.bin/fbidq.html?lola), [unc-104](http://flybase.bio.indiana.edu/.bin/fbidq.html?unc-104), [chif](http://flybase.bio.indiana.edu/.bin/fbidq.html?chif), [Pcl](http://flybase.bio.indiana.edu/.bin/fbidq.html?Pcl), [p130CAS](http://flybase.bio.indiana.edu/.bin/fbidq.html?p130CAS), [CG15312](http://flybase.bio.indiana.edu/.bin/fbidq.html?CG15312), [CG7033](http://flybase.bio.indiana.edu/.bin/fbidq.html?CG7033), [Spc105R](http://flybase.bio.indiana.edu/.bin/fbidq.html?Spc105R), [CG31158](http://flybase.bio.indiana.edu/.bin/fbidq.html?CG31158), [Awh](http://flybase.bio.indiana.edu/.bin/fbidq.html?Awh), [Appl](http://flybase.bio.indiana.edu/.bin/fbidq.html?Appl), [tap](http://flybase.bio.indiana.edu/.bin/fbidq.html?tap), [metro](http://flybase.bio.indiana.edu/.bin/fbidq.html?metro), [CG13550](http://flybase.bio.indiana.edu/.bin/fbidq.html?CG13550), [ewg](http://flybase.bio.indiana.edu/.bin/fbidq.html?ewg), [CG30007](http://flybase.bio.indiana.edu/.bin/fbidq.html?CG30007), [erm](http://flybase.bio.indiana.edu/.bin/fbidq.html?erm), [Nrk](http://flybase.bio.indiana.edu/.bin/fbidq.html?Nrk), [sano](http://flybase.bio.indiana.edu/.bin/fbidq.html?sano), [dmt](http://flybase.bio.indiana.edu/.bin/fbidq.html?dmt), [CG4328](http://flybase.bio.indiana.edu/.bin/fbidq.html?CG4328),[CG11399](http://flybase.bio.indiana.edu/.bin/fbidq.html?CG11399), [l(2)NC136](http://flybase.bio.indiana.edu/.bin/fbidq.html?l(2)NC136), [Su(var)3-3](http://flybase.bio.indiana.edu/.bin/fbidq.html?Su(var)3-3), [CG10249](http://flybase.bio.indiana.edu/.bin/fbidq.html?CG10249), [Taf4](http://flybase.bio.indiana.edu/.bin/fbidq.html?Taf4), [Nrg](http://flybase.bio.indiana.edu/.bin/fbidq.html?Nrg), [gogo](http://flybase.bio.indiana.edu/.bin/fbidq.html?gogo), [insc](http://flybase.bio.indiana.edu/.bin/fbidq.html?insc), [jeb](http://flybase.bio.indiana.edu/.bin/fbidq.html?jeb), [GluClalpha](http://flybase.bio.indiana.edu/.bin/fbidq.html?GluClalpha), [Actbeta](http://flybase.bio.indiana.edu/.bin/fbidq.html?Actbeta), [stau](http://flybase.bio.indiana.edu/.bin/fbidq.html?stau), [Sema-1a](http://flybase.bio.indiana.edu/.bin/fbidq.html?Sema-1a), [beat-Ib](http://flybase.bio.indiana.edu/.bin/fbidq.html?beat-Ib), [Hs3st-B](http://flybase.bio.indiana.edu/.bin/fbidq.html?Hs3st-B), [otk](http://flybase.bio.indiana.edu/.bin/fbidq.html?otk), [Sos](http://flybase.bio.indiana.edu/.bin/fbidq.html?Sos), [phyl](http://flybase.bio.indiana.edu/.bin/fbidq.html?phyl), [vg](http://flybase.bio.indiana.edu/.bin/fbidq.html?vg), [Fas2](http://flybase.bio.indiana.edu/.bin/fbidq.html?Fas2), [Klp61F](http://flybase.bio.indiana.edu/.bin/fbidq.html?Klp61F), [eya](http://flybase.bio.indiana.edu/.bin/fbidq.html?eya), [fz](http://flybase.bio.indiana.edu/.bin/fbidq.html?fz), [robo](http://flybase.bio.indiana.edu/.bin/fbidq.html?robo), [CG8557](http://flybase.bio.indiana.edu/.bin/fbidq.html?CG8557), [bsk](http://flybase.bio.indiana.edu/.bin/fbidq.html?bsk), [brm](http://flybase.bio.indiana.edu/.bin/fbidq.html?brm), [beat-Ia](http://flybase.bio.indiana.edu/.bin/fbidq.html?beat-Ia), [kat-60L1](http://flybase.bio.indiana.edu/.bin/fbidq.html?kat-60L1), [mtg](http://flybase.bio.indiana.edu/.bin/fbidq.html?mtg), [ft](http://flybase.bio.indiana.edu/.bin/fbidq.html?ft), [CG42268](http://flybase.bio.indiana.edu/.bin/fbidq.html?CG42268), [lft](http://flybase.bio.indiana.edu/.bin/fbidq.html?lft), [sno](http://flybase.bio.indiana.edu/.bin/fbidq.html?sno),[hig](http://flybase.bio.indiana.edu/.bin/fbidq.html?hig), [CG12004](http://flybase.bio.indiana.edu/.bin/fbidq.html?CG12004), [RhoGAPp190](http://flybase.bio.indiana.edu/.bin/fbidq.html?RhoGAPp190), [Sfmbt](http://flybase.bio.indiana.edu/.bin/fbidq.html?Sfmbt), [ara](http://flybase.bio.indiana.edu/.bin/fbidq.html?ara), [pHCl](http://flybase.bio.indiana.edu/.bin/fbidq.html?pHCl), [l(2)k16918](http://flybase.bio.indiana.edu/.bin/fbidq.html?l(2)k16918), [mam](http://flybase.bio.indiana.edu/.bin/fbidq.html?mam), [pan](http://flybase.bio.indiana.edu/.bin/fbidq.html?pan), [sws](http://flybase.bio.indiana.edu/.bin/fbidq.html?sws), [Dll](http://flybase.bio.indiana.edu/.bin/fbidq.html?Dll), [disco](http://flybase.bio.indiana.edu/.bin/fbidq.html?disco), [grn](http://flybase.bio.indiana.edu/.bin/fbidq.html?grn), [HLHm5](http://flybase.bio.indiana.edu/.bin/fbidq.html?HLHm5), [mew](http://flybase.bio.indiana.edu/.bin/fbidq.html?mew), [didum](http://flybase.bio.indiana.edu/.bin/fbidq.html?didum), [Lis-1](http://flybase.bio.indiana.edu/.bin/fbidq.html?Lis-1), [dap](http://flybase.bio.indiana.edu/.bin/fbidq.html?dap), [ems](http://flybase.bio.indiana.edu/.bin/fbidq.html?ems), [eys](http://flybase.bio.indiana.edu/.bin/fbidq.html?eys), [Bx](http://flybase.bio.indiana.edu/.bin/fbidq.html?Bx), [Mob2](http://flybase.bio.indiana.edu/.bin/fbidq.html?Mob2), [pros](http://flybase.bio.indiana.edu/.bin/fbidq.html?pros), [tup](http://flybase.bio.indiana.edu/.bin/fbidq.html?tup), [trio](http://flybase.bio.indiana.edu/.bin/fbidq.html?trio), [pdm2](http://flybase.bio.indiana.edu/.bin/fbidq.html?pdm2), [CG1463](http://flybase.bio.indiana.edu/.bin/fbidq.html?CG1463), [Gad1](http://flybase.bio.indiana.edu/.bin/fbidq.html?Gad1), [Wnt4](http://flybase.bio.indiana.edu/.bin/fbidq.html?Wnt4), [rictor](http://flybase.bio.indiana.edu/.bin/fbidq.html?rictor), [Glut4EF](http://flybase.bio.indiana.edu/.bin/fbidq.html?Glut4EF), [CaMKII](http://flybase.bio.indiana.edu/.bin/fbidq.html?CaMKII), [nerfin-1](http://flybase.bio.indiana.edu/.bin/fbidq.html?nerfin-1), [bchs](http://flybase.bio.indiana.edu/.bin/fbidq.html?bchs), [RpS5b](http://flybase.bio.indiana.edu/.bin/fbidq.html?RpS5b),[polo](http://flybase.bio.indiana.edu/.bin/fbidq.html?polo), [pk](http://flybase.bio.indiana.edu/.bin/fbidq.html?pk), [dsx](http://flybase.bio.indiana.edu/.bin/fbidq.html?dsx), [chinmo](http://flybase.bio.indiana.edu/.bin/fbidq.html?chinmo), [Syn1](http://flybase.bio.indiana.edu/.bin/fbidq.html?Syn1), [betaTub85D](http://flybase.bio.indiana.edu/.bin/fbidq.html?betaTub85D), [Ssdp](http://flybase.bio.indiana.edu/.bin/fbidq.html?Ssdp), [klu](http://flybase.bio.indiana.edu/.bin/fbidq.html?klu), [sqz](http://flybase.bio.indiana.edu/.bin/fbidq.html?sqz), [fend](http://flybase.bio.indiana.edu/.bin/fbidq.html?fend), [Atet](http://flybase.bio.indiana.edu/.bin/fbidq.html?Atet), [sr](http://flybase.bio.indiana.edu/.bin/fbidq.html?sr), [Lim1](http://flybase.bio.indiana.edu/.bin/fbidq.html?Lim1), [nkd](http://flybase.bio.indiana.edu/.bin/fbidq.html?nkd), [tld](http://flybase.bio.indiana.edu/.bin/fbidq.html?tld), [danr](http://flybase.bio.indiana.edu/.bin/fbidq.html?danr), [Dsk](http://flybase.bio.indiana.edu/.bin/fbidq.html?Dsk), [vvl](http://flybase.bio.indiana.edu/.bin/fbidq.html?vvl), [Unc-76](http://flybase.bio.indiana.edu/.bin/fbidq.html?Unc-76), [rg](http://flybase.bio.indiana.edu/.bin/fbidq.html?rg), [betaInt-nu](http://flybase.bio.indiana.edu/.bin/fbidq.html?betaInt-nu), [sgg](http://flybase.bio.indiana.edu/.bin/fbidq.html?sgg), [Brd](http://flybase.bio.indiana.edu/.bin/fbidq.html?Brd), [mnb](http://flybase.bio.indiana.edu/.bin/fbidq.html?mnb), [gcm2](http://flybase.bio.indiana.edu/.bin/fbidq.html?gcm2), [Nmdar1](http://flybase.bio.indiana.edu/.bin/fbidq.html?Nmdar1), [Eip93F](http://flybase.bio.indiana.edu/.bin/fbidq.html?Eip93F), [mp](http://flybase.bio.indiana.edu/.bin/fbidq.html?mp), [gcm](http://flybase.bio.indiana.edu/.bin/fbidq.html?gcm), [CG5890](http://flybase.bio.indiana.edu/.bin/fbidq.html?CG5890), [Ccn](http://flybase.bio.indiana.edu/.bin/fbidq.html?Ccn), [SP2353](http://flybase.bio.indiana.edu/.bin/fbidq.html?SP2353), [ase](http://flybase.bio.indiana.edu/.bin/fbidq.html?ase), [CG13253](http://flybase.bio.indiana.edu/.bin/fbidq.html?CG13253), [ovo](http://flybase.bio.indiana.edu/.bin/fbidq.html?ovo), [CG32685](http://flybase.bio.indiana.edu/.bin/fbidq.html?CG32685), [elav](http://flybase.bio.indiana.edu/.bin/fbidq.html?elav),[Dsp1](http://flybase.bio.indiana.edu/.bin/fbidq.html?Dsp1), [ds](http://flybase.bio.indiana.edu/.bin/fbidq.html?ds), [Poxn](http://flybase.bio.indiana.edu/.bin/fbidq.html?Poxn), [Incenp](http://flybase.bio.indiana.edu/.bin/fbidq.html?Incenp), [Rat1](http://flybase.bio.indiana.edu/.bin/fbidq.html?Rat1), [CG18273](http://flybase.bio.indiana.edu/.bin/fbidq.html?CG18273), [eIF4G2](http://flybase.bio.indiana.edu/.bin/fbidq.html?eIF4G2), [fred](http://flybase.bio.indiana.edu/.bin/fbidq.html?fred), [Drl-2](http://flybase.bio.indiana.edu/.bin/fbidq.html?Drl-2), [MYPT-75D](http://flybase.bio.indiana.edu/.bin/fbidq.html?MYPT-75D), [ush](http://flybase.bio.indiana.edu/.bin/fbidq.html?ush), [Fak56D](http://flybase.bio.indiana.edu/.bin/fbidq.html?Fak56D), [Gap1](http://flybase.bio.indiana.edu/.bin/fbidq.html?Gap1), [run](http://flybase.bio.indiana.edu/.bin/fbidq.html?run), [CG16778](http://flybase.bio.indiana.edu/.bin/fbidq.html?CG16778), [ko](http://flybase.bio.indiana.edu/.bin/fbidq.html?ko), [tara](http://flybase.bio.indiana.edu/.bin/fbidq.html?tara), [Eip71CD](http://flybase.bio.indiana.edu/.bin/fbidq.html?Eip71CD), [Alh](http://flybase.bio.indiana.edu/.bin/fbidq.html?Alh), [egg](http://flybase.bio.indiana.edu/.bin/fbidq.html?egg), [stan](http://flybase.bio.indiana.edu/.bin/fbidq.html?stan), [CG12424](http://flybase.bio.indiana.edu/.bin/fbidq.html?CG12424), [tutl](http://flybase.bio.indiana.edu/.bin/fbidq.html?tutl), [Sp1](http://flybase.bio.indiana.edu/.bin/fbidq.html?Sp1), [ph-p](http://flybase.bio.indiana.edu/.bin/fbidq.html?ph-p), [Frq1](http://flybase.bio.indiana.edu/.bin/fbidq.html?Frq1), [GlcAT-P](http://flybase.bio.indiana.edu/.bin/fbidq.html?GlcAT-P), [sna](http://flybase.bio.indiana.edu/.bin/fbidq.html?sna), [Wnt5](http://flybase.bio.indiana.edu/.bin/fbidq.html?Wnt5), [SF2](http://flybase.bio.indiana.edu/.bin/fbidq.html?SF2), [Bre1](http://flybase.bio.indiana.edu/.bin/fbidq.html?Bre1), [Taf1](http://flybase.bio.indiana.edu/.bin/fbidq.html?Taf1), [Ggamma1](http://flybase.bio.indiana.edu/.bin/fbidq.html?Ggamma1), [CG7597](http://flybase.bio.indiana.edu/.bin/fbidq.html?CG7597),[CG5466](http://flybase.bio.indiana.edu/.bin/fbidq.html?CG5466), [esg](http://flybase.bio.indiana.edu/.bin/fbidq.html?esg), [sif](http://flybase.bio.indiana.edu/.bin/fbidq.html?sif), [m4](http://flybase.bio.indiana.edu/.bin/fbidq.html?m4), [dom](http://flybase.bio.indiana.edu/.bin/fbidq.html?dom), [Aac11](http://flybase.bio.indiana.edu/.bin/fbidq.html?Aac11), [ssh](http://flybase.bio.indiana.edu/.bin/fbidq.html?ssh), [PIP5K59B](http://flybase.bio.indiana.edu/.bin/fbidq.html?PIP5K59B), [klar](http://flybase.bio.indiana.edu/.bin/fbidq.html?klar), [mle](http://flybase.bio.indiana.edu/.bin/fbidq.html?mle), [Hr51](http://flybase.bio.indiana.edu/.bin/fbidq.html?Hr51), [shi](http://flybase.bio.indiana.edu/.bin/fbidq.html?shi), [CG11155](http://flybase.bio.indiana.edu/.bin/fbidq.html?CG11155), [siz](http://flybase.bio.indiana.edu/.bin/fbidq.html?siz), [B-H2](http://flybase.bio.indiana.edu/.bin/fbidq.html?B-H2), [wg](http://flybase.bio.indiana.edu/.bin/fbidq.html?wg), [fkh](http://flybase.bio.indiana.edu/.bin/fbidq.html?fkh), [CadN](http://flybase.bio.indiana.edu/.bin/fbidq.html?CadN), [CG34139](http://flybase.bio.indiana.edu/.bin/fbidq.html?CG34139), [Mfap1](http://flybase.bio.indiana.edu/.bin/fbidq.html?Mfap1), [RpL28](http://flybase.bio.indiana.edu/.bin/fbidq.html?RpL28), [klg](http://flybase.bio.indiana.edu/.bin/fbidq.html?klg), [en](http://flybase.bio.indiana.edu/.bin/fbidq.html?en), [E(bx)](http://flybase.bio.indiana.edu/.bin/fbidq.html?E(bx)), [sfl](http://flybase.bio.indiana.edu/.bin/fbidq.html?sfl), [Sip1](http://flybase.bio.indiana.edu/.bin/fbidq.html?Sip1), [Snap](http://flybase.bio.indiana.edu/.bin/fbidq.html?Snap), [trn](http://flybase.bio.indiana.edu/.bin/fbidq.html?trn), [Utx](http://flybase.bio.indiana.edu/.bin/fbidq.html?Utx), [pburs](http://flybase.bio.indiana.edu/.bin/fbidq.html?pburs), [Rnp4F](http://flybase.bio.indiana.edu/.bin/fbidq.html?Rnp4F), [cos](http://flybase.bio.indiana.edu/.bin/fbidq.html?cos), [Ct](http://flybase.bio.indiana.edu/.bin/fbidq.html?Ct) |
| [single-organism cellular process](http://amigo.geneontology.org/cgi-bin/amigo/go.cgi?view=details&query=GO:0044763) | 764 of 1594 genes, 47.9% | 3417 of 7634 genes, 44.8% | 0.00099 | 0.00% | 0.00 | [endoA](http://flybase.bio.indiana.edu/.bin/fbidq.html?endoA), [Sh](http://flybase.bio.indiana.edu/.bin/fbidq.html?Sh), [CG9279](http://flybase.bio.indiana.edu/.bin/fbidq.html?CG9279), [dock](http://flybase.bio.indiana.edu/.bin/fbidq.html?dock), [CG5808](http://flybase.bio.indiana.edu/.bin/fbidq.html?CG5808), [CG9004](http://flybase.bio.indiana.edu/.bin/fbidq.html?CG9004), [CG3703](http://flybase.bio.indiana.edu/.bin/fbidq.html?CG3703), [CG32135](http://flybase.bio.indiana.edu/.bin/fbidq.html?CG32135), [tna](http://flybase.bio.indiana.edu/.bin/fbidq.html?tna), [CG8500](http://flybase.bio.indiana.edu/.bin/fbidq.html?CG8500), [ap](http://flybase.bio.indiana.edu/.bin/fbidq.html?ap), [cenG1A](http://flybase.bio.indiana.edu/.bin/fbidq.html?cenG1A), [tlk](http://flybase.bio.indiana.edu/.bin/fbidq.html?tlk), [malpha](http://flybase.bio.indiana.edu/.bin/fbidq.html?malpha), [os](http://flybase.bio.indiana.edu/.bin/fbidq.html?os), [Axn](http://flybase.bio.indiana.edu/.bin/fbidq.html?Axn), [Gs1](http://flybase.bio.indiana.edu/.bin/fbidq.html?Gs1), [mtrm](http://flybase.bio.indiana.edu/.bin/fbidq.html?mtrm), [Hcf](http://flybase.bio.indiana.edu/.bin/fbidq.html?Hcf), [Traf6](http://flybase.bio.indiana.edu/.bin/fbidq.html?Traf6), [Leucokinin](http://flybase.bio.indiana.edu/.bin/fbidq.html?Leucokinin), [NetB](http://flybase.bio.indiana.edu/.bin/fbidq.html?NetB), [rdgC](http://flybase.bio.indiana.edu/.bin/fbidq.html?rdgC), [cid](http://flybase.bio.indiana.edu/.bin/fbidq.html?cid), [CG32137](http://flybase.bio.indiana.edu/.bin/fbidq.html?CG32137), [lute](http://flybase.bio.indiana.edu/.bin/fbidq.html?lute), [Ssrp](http://flybase.bio.indiana.edu/.bin/fbidq.html?Ssrp), [Lar](http://flybase.bio.indiana.edu/.bin/fbidq.html?Lar), [scrib](http://flybase.bio.indiana.edu/.bin/fbidq.html?scrib), [HGTX](http://flybase.bio.indiana.edu/.bin/fbidq.html?HGTX), [not](http://flybase.bio.indiana.edu/.bin/fbidq.html?not), [CG7650](http://flybase.bio.indiana.edu/.bin/fbidq.html?CG7650), [nvy](http://flybase.bio.indiana.edu/.bin/fbidq.html?nvy), [Ect3](http://flybase.bio.indiana.edu/.bin/fbidq.html?Ect3), [nab](http://flybase.bio.indiana.edu/.bin/fbidq.html?nab),[HLHmdelta](http://flybase.bio.indiana.edu/.bin/fbidq.html?HLHmdelta), [U2af38](http://flybase.bio.indiana.edu/.bin/fbidq.html?U2af38), [7B2](http://flybase.bio.indiana.edu/.bin/fbidq.html?7B2), [alph](http://flybase.bio.indiana.edu/.bin/fbidq.html?alph), [Traf4](http://flybase.bio.indiana.edu/.bin/fbidq.html?Traf4), [TBPH](http://flybase.bio.indiana.edu/.bin/fbidq.html?TBPH), [Snap25](http://flybase.bio.indiana.edu/.bin/fbidq.html?Snap25), [Fer2](http://flybase.bio.indiana.edu/.bin/fbidq.html?Fer2), [CG34400](http://flybase.bio.indiana.edu/.bin/fbidq.html?CG34400), [Ulp1](http://flybase.bio.indiana.edu/.bin/fbidq.html?Ulp1), [CG42330](http://flybase.bio.indiana.edu/.bin/fbidq.html?CG42330), [mGluRA](http://flybase.bio.indiana.edu/.bin/fbidq.html?mGluRA), [ena](http://flybase.bio.indiana.edu/.bin/fbidq.html?ena), [dsh](http://flybase.bio.indiana.edu/.bin/fbidq.html?dsh), [Nf-YC](http://flybase.bio.indiana.edu/.bin/fbidq.html?Nf-YC), [Pli](http://flybase.bio.indiana.edu/.bin/fbidq.html?Pli), [qkr58E-1](http://flybase.bio.indiana.edu/.bin/fbidq.html?qkr58E-1), [Hel25E](http://flybase.bio.indiana.edu/.bin/fbidq.html?Hel25E), [comt](http://flybase.bio.indiana.edu/.bin/fbidq.html?comt), [CG31140](http://flybase.bio.indiana.edu/.bin/fbidq.html?CG31140), [PP2A-B](http://flybase.bio.indiana.edu/.bin/fbidq.html?PP2A-B), [Pxn](http://flybase.bio.indiana.edu/.bin/fbidq.html?Pxn), [qvr](http://flybase.bio.indiana.edu/.bin/fbidq.html?qvr), [DAAM](http://flybase.bio.indiana.edu/.bin/fbidq.html?DAAM), [pall](http://flybase.bio.indiana.edu/.bin/fbidq.html?pall), [rho-5](http://flybase.bio.indiana.edu/.bin/fbidq.html?rho-5), [nmo](http://flybase.bio.indiana.edu/.bin/fbidq.html?nmo), [Tusp](http://flybase.bio.indiana.edu/.bin/fbidq.html?Tusp), [5-HT1A](http://flybase.bio.indiana.edu/.bin/fbidq.html?5-HT1A), [CG13248](http://flybase.bio.indiana.edu/.bin/fbidq.html?CG13248), [Gug](http://flybase.bio.indiana.edu/.bin/fbidq.html?Gug), [Actn](http://flybase.bio.indiana.edu/.bin/fbidq.html?Actn), [ed](http://flybase.bio.indiana.edu/.bin/fbidq.html?ed),[CG11317](http://flybase.bio.indiana.edu/.bin/fbidq.html?CG11317), [daw](http://flybase.bio.indiana.edu/.bin/fbidq.html?daw), [CG5036](http://flybase.bio.indiana.edu/.bin/fbidq.html?CG5036), [5-HT1B](http://flybase.bio.indiana.edu/.bin/fbidq.html?5-HT1B), [Poc1](http://flybase.bio.indiana.edu/.bin/fbidq.html?Poc1), [Fhos](http://flybase.bio.indiana.edu/.bin/fbidq.html?Fhos), [Rx](http://flybase.bio.indiana.edu/.bin/fbidq.html?Rx), [salr](http://flybase.bio.indiana.edu/.bin/fbidq.html?salr), [Apc](http://flybase.bio.indiana.edu/.bin/fbidq.html?Apc), [CG8795](http://flybase.bio.indiana.edu/.bin/fbidq.html?CG8795), [VGAT](http://flybase.bio.indiana.edu/.bin/fbidq.html?VGAT), [d4](http://flybase.bio.indiana.edu/.bin/fbidq.html?d4), [cort](http://flybase.bio.indiana.edu/.bin/fbidq.html?cort), [tau](http://flybase.bio.indiana.edu/.bin/fbidq.html?tau), [beat-Vb](http://flybase.bio.indiana.edu/.bin/fbidq.html?beat-Vb), [Jarid2](http://flybase.bio.indiana.edu/.bin/fbidq.html?Jarid2), [RN-tre](http://flybase.bio.indiana.edu/.bin/fbidq.html?RN-tre), [Mf](http://flybase.bio.indiana.edu/.bin/fbidq.html?Mf), [wnd](http://flybase.bio.indiana.edu/.bin/fbidq.html?wnd), [trx](http://flybase.bio.indiana.edu/.bin/fbidq.html?trx), [Btk29A](http://flybase.bio.indiana.edu/.bin/fbidq.html?Btk29A), [SMC2](http://flybase.bio.indiana.edu/.bin/fbidq.html?SMC2), [ETH](http://flybase.bio.indiana.edu/.bin/fbidq.html?ETH), [unc-5](http://flybase.bio.indiana.edu/.bin/fbidq.html?unc-5), [cdi](http://flybase.bio.indiana.edu/.bin/fbidq.html?cdi), [CG13907](http://flybase.bio.indiana.edu/.bin/fbidq.html?CG13907), [vfl](http://flybase.bio.indiana.edu/.bin/fbidq.html?vfl), [CG1139](http://flybase.bio.indiana.edu/.bin/fbidq.html?CG1139), [barr](http://flybase.bio.indiana.edu/.bin/fbidq.html?barr), [NPFR1](http://flybase.bio.indiana.edu/.bin/fbidq.html?NPFR1), [pygo](http://flybase.bio.indiana.edu/.bin/fbidq.html?pygo), [z](http://flybase.bio.indiana.edu/.bin/fbidq.html?z), [HLHmgamma](http://flybase.bio.indiana.edu/.bin/fbidq.html?HLHmgamma), [CG15556](http://flybase.bio.indiana.edu/.bin/fbidq.html?CG15556), [jar](http://flybase.bio.indiana.edu/.bin/fbidq.html?jar),[Klp68D](http://flybase.bio.indiana.edu/.bin/fbidq.html?Klp68D), [Ror](http://flybase.bio.indiana.edu/.bin/fbidq.html?Ror), [CG7368](http://flybase.bio.indiana.edu/.bin/fbidq.html?CG7368), [wor](http://flybase.bio.indiana.edu/.bin/fbidq.html?wor), [Atg1](http://flybase.bio.indiana.edu/.bin/fbidq.html?Atg1), [Fcp1](http://flybase.bio.indiana.edu/.bin/fbidq.html?Fcp1), [Ptp69D](http://flybase.bio.indiana.edu/.bin/fbidq.html?Ptp69D), [hts](http://flybase.bio.indiana.edu/.bin/fbidq.html?hts), [pUf68](http://flybase.bio.indiana.edu/.bin/fbidq.html?pUf68), [bowl](http://flybase.bio.indiana.edu/.bin/fbidq.html?bowl), [seq](http://flybase.bio.indiana.edu/.bin/fbidq.html?seq), [HDAC4](http://flybase.bio.indiana.edu/.bin/fbidq.html?HDAC4), [CG1671](http://flybase.bio.indiana.edu/.bin/fbidq.html?CG1671), [Cbl](http://flybase.bio.indiana.edu/.bin/fbidq.html?Cbl), [thr](http://flybase.bio.indiana.edu/.bin/fbidq.html?thr), [pog](http://flybase.bio.indiana.edu/.bin/fbidq.html?pog), [CG12071](http://flybase.bio.indiana.edu/.bin/fbidq.html?CG12071), [Pdp1](http://flybase.bio.indiana.edu/.bin/fbidq.html?Pdp1), [CASK](http://flybase.bio.indiana.edu/.bin/fbidq.html?CASK), [CG10251](http://flybase.bio.indiana.edu/.bin/fbidq.html?CG10251), [CG13743](http://flybase.bio.indiana.edu/.bin/fbidq.html?CG13743), [CG32758](http://flybase.bio.indiana.edu/.bin/fbidq.html?CG32758), [mtt](http://flybase.bio.indiana.edu/.bin/fbidq.html?mtt), [CG5535](http://flybase.bio.indiana.edu/.bin/fbidq.html?CG5535), [disp](http://flybase.bio.indiana.edu/.bin/fbidq.html?disp), [mip120](http://flybase.bio.indiana.edu/.bin/fbidq.html?mip120), [cpx](http://flybase.bio.indiana.edu/.bin/fbidq.html?cpx), [CG4022](http://flybase.bio.indiana.edu/.bin/fbidq.html?CG4022), [jumu](http://flybase.bio.indiana.edu/.bin/fbidq.html?jumu), [Su(var)2-HP2](http://flybase.bio.indiana.edu/.bin/fbidq.html?Su(var)2-HP2), [gfA](http://flybase.bio.indiana.edu/.bin/fbidq.html?gfA), [Usp7](http://flybase.bio.indiana.edu/.bin/fbidq.html?Usp7),[CG31781](http://flybase.bio.indiana.edu/.bin/fbidq.html?CG31781), [fd59A](http://flybase.bio.indiana.edu/.bin/fbidq.html?fd59A), [beat-Ic](http://flybase.bio.indiana.edu/.bin/fbidq.html?beat-Ic), [Rph](http://flybase.bio.indiana.edu/.bin/fbidq.html?Rph), [brat](http://flybase.bio.indiana.edu/.bin/fbidq.html?brat), [FBX011](http://flybase.bio.indiana.edu/.bin/fbidq.html?FBX011), [ST6Gal](http://flybase.bio.indiana.edu/.bin/fbidq.html?ST6Gal), [CG17343](http://flybase.bio.indiana.edu/.bin/fbidq.html?CG17343), [GABA-B-R2](http://flybase.bio.indiana.edu/.bin/fbidq.html?GABA-B-R2), [CG6231](http://flybase.bio.indiana.edu/.bin/fbidq.html?CG6231), [CG7051](http://flybase.bio.indiana.edu/.bin/fbidq.html?CG7051), [futsch](http://flybase.bio.indiana.edu/.bin/fbidq.html?futsch), [Ubx](http://flybase.bio.indiana.edu/.bin/fbidq.html?Ubx), [Cbp53E](http://flybase.bio.indiana.edu/.bin/fbidq.html?Cbp53E), [gukh](http://flybase.bio.indiana.edu/.bin/fbidq.html?gukh), [Dscam3](http://flybase.bio.indiana.edu/.bin/fbidq.html?Dscam3), [Jupiter](http://flybase.bio.indiana.edu/.bin/fbidq.html?Jupiter), [chm](http://flybase.bio.indiana.edu/.bin/fbidq.html?chm), [Rim](http://flybase.bio.indiana.edu/.bin/fbidq.html?Rim), [Rpt3R](http://flybase.bio.indiana.edu/.bin/fbidq.html?Rpt3R), [msl-1](http://flybase.bio.indiana.edu/.bin/fbidq.html?msl-1), [beat-IIa](http://flybase.bio.indiana.edu/.bin/fbidq.html?beat-IIa), [corn](http://flybase.bio.indiana.edu/.bin/fbidq.html?corn), [VhaM9.7-a](http://flybase.bio.indiana.edu/.bin/fbidq.html?VhaM9.7-a), [CG11597](http://flybase.bio.indiana.edu/.bin/fbidq.html?CG11597), [Sur-8](http://flybase.bio.indiana.edu/.bin/fbidq.html?Sur-8), [CG42574](http://flybase.bio.indiana.edu/.bin/fbidq.html?CG42574), [Rgl](http://flybase.bio.indiana.edu/.bin/fbidq.html?Rgl), [dac](http://flybase.bio.indiana.edu/.bin/fbidq.html?dac), [Nrx-1](http://flybase.bio.indiana.edu/.bin/fbidq.html?Nrx-1),[CG9007](http://flybase.bio.indiana.edu/.bin/fbidq.html?CG9007), [CG33203](http://flybase.bio.indiana.edu/.bin/fbidq.html?CG33203), [mav](http://flybase.bio.indiana.edu/.bin/fbidq.html?mav), [Ilp7](http://flybase.bio.indiana.edu/.bin/fbidq.html?Ilp7), [CG31122](http://flybase.bio.indiana.edu/.bin/fbidq.html?CG31122), [CG15439](http://flybase.bio.indiana.edu/.bin/fbidq.html?CG15439), [CG33275](http://flybase.bio.indiana.edu/.bin/fbidq.html?CG33275), [CG40351](http://flybase.bio.indiana.edu/.bin/fbidq.html?CG40351), [Lim3](http://flybase.bio.indiana.edu/.bin/fbidq.html?Lim3), [CG14681](http://flybase.bio.indiana.edu/.bin/fbidq.html?CG14681), [sd](http://flybase.bio.indiana.edu/.bin/fbidq.html?sd), [Aplip1](http://flybase.bio.indiana.edu/.bin/fbidq.html?Aplip1), [CG1239](http://flybase.bio.indiana.edu/.bin/fbidq.html?CG1239), [Eip63F-1](http://flybase.bio.indiana.edu/.bin/fbidq.html?Eip63F-1), [CG11347](http://flybase.bio.indiana.edu/.bin/fbidq.html?CG11347), [CG5853](http://flybase.bio.indiana.edu/.bin/fbidq.html?CG5853), [Ggamma30A](http://flybase.bio.indiana.edu/.bin/fbidq.html?Ggamma30A), [yrt](http://flybase.bio.indiana.edu/.bin/fbidq.html?yrt), [wls](http://flybase.bio.indiana.edu/.bin/fbidq.html?wls), [skl](http://flybase.bio.indiana.edu/.bin/fbidq.html?skl), [CG1688](http://flybase.bio.indiana.edu/.bin/fbidq.html?CG1688), [hiw](http://flybase.bio.indiana.edu/.bin/fbidq.html?hiw), [Atf-2](http://flybase.bio.indiana.edu/.bin/fbidq.html?Atf-2), [n-syb](http://flybase.bio.indiana.edu/.bin/fbidq.html?n-syb), [skd](http://flybase.bio.indiana.edu/.bin/fbidq.html?skd), [jing](http://flybase.bio.indiana.edu/.bin/fbidq.html?jing), [katanin-60](http://flybase.bio.indiana.edu/.bin/fbidq.html?katanin-60), [Camta](http://flybase.bio.indiana.edu/.bin/fbidq.html?Camta), [tll](http://flybase.bio.indiana.edu/.bin/fbidq.html?tll), [ato](http://flybase.bio.indiana.edu/.bin/fbidq.html?ato), [Fmrf](http://flybase.bio.indiana.edu/.bin/fbidq.html?Fmrf),[Iswi](http://flybase.bio.indiana.edu/.bin/fbidq.html?Iswi), [CG5742](http://flybase.bio.indiana.edu/.bin/fbidq.html?CG5742), [obst-A](http://flybase.bio.indiana.edu/.bin/fbidq.html?obst-A), [Pka-R1](http://flybase.bio.indiana.edu/.bin/fbidq.html?Pka-R1), [mago](http://flybase.bio.indiana.edu/.bin/fbidq.html?mago), [enc](http://flybase.bio.indiana.edu/.bin/fbidq.html?enc), [cuff](http://flybase.bio.indiana.edu/.bin/fbidq.html?cuff), [CG42533](http://flybase.bio.indiana.edu/.bin/fbidq.html?CG42533), [ksr](http://flybase.bio.indiana.edu/.bin/fbidq.html?ksr), [CG7837](http://flybase.bio.indiana.edu/.bin/fbidq.html?CG7837), [PMCA](http://flybase.bio.indiana.edu/.bin/fbidq.html?PMCA), [CG3618](http://flybase.bio.indiana.edu/.bin/fbidq.html?CG3618), [Gyc-89Da](http://flybase.bio.indiana.edu/.bin/fbidq.html?Gyc-89Da), [brp](http://flybase.bio.indiana.edu/.bin/fbidq.html?brp), [chn](http://flybase.bio.indiana.edu/.bin/fbidq.html?chn), [ssp3](http://flybase.bio.indiana.edu/.bin/fbidq.html?ssp3), [Khc-73](http://flybase.bio.indiana.edu/.bin/fbidq.html?Khc-73), [chif](http://flybase.bio.indiana.edu/.bin/fbidq.html?chif), [D2R](http://flybase.bio.indiana.edu/.bin/fbidq.html?D2R), [p130CAS](http://flybase.bio.indiana.edu/.bin/fbidq.html?p130CAS), [CG7033](http://flybase.bio.indiana.edu/.bin/fbidq.html?CG7033), [CG13908](http://flybase.bio.indiana.edu/.bin/fbidq.html?CG13908), [CG30007](http://flybase.bio.indiana.edu/.bin/fbidq.html?CG30007), [ewg](http://flybase.bio.indiana.edu/.bin/fbidq.html?ewg), [CG13550](http://flybase.bio.indiana.edu/.bin/fbidq.html?CG13550), [sano](http://flybase.bio.indiana.edu/.bin/fbidq.html?sano), [Cirl](http://flybase.bio.indiana.edu/.bin/fbidq.html?Cirl), [APC4](http://flybase.bio.indiana.edu/.bin/fbidq.html?APC4), [l(2)NC136](http://flybase.bio.indiana.edu/.bin/fbidq.html?l(2)NC136), [Su(var)3-3](http://flybase.bio.indiana.edu/.bin/fbidq.html?Su(var)3-3), [Bili](http://flybase.bio.indiana.edu/.bin/fbidq.html?Bili),[gogo](http://flybase.bio.indiana.edu/.bin/fbidq.html?gogo), [unc-13-4A](http://flybase.bio.indiana.edu/.bin/fbidq.html?unc-13-4A), [Dh44-R1](http://flybase.bio.indiana.edu/.bin/fbidq.html?Dh44-R1), [stet](http://flybase.bio.indiana.edu/.bin/fbidq.html?stet), [GluClalpha](http://flybase.bio.indiana.edu/.bin/fbidq.html?GluClalpha), [Sema-1a](http://flybase.bio.indiana.edu/.bin/fbidq.html?Sema-1a), [Eh](http://flybase.bio.indiana.edu/.bin/fbidq.html?Eh), [Hs3st-B](http://flybase.bio.indiana.edu/.bin/fbidq.html?Hs3st-B), [Sos](http://flybase.bio.indiana.edu/.bin/fbidq.html?Sos), [Fas2](http://flybase.bio.indiana.edu/.bin/fbidq.html?Fas2), [Klp61F](http://flybase.bio.indiana.edu/.bin/fbidq.html?Klp61F), [eya](http://flybase.bio.indiana.edu/.bin/fbidq.html?eya), [mAcR-60C](http://flybase.bio.indiana.edu/.bin/fbidq.html?mAcR-60C), [CG8557](http://flybase.bio.indiana.edu/.bin/fbidq.html?CG8557), [beat-Ia](http://flybase.bio.indiana.edu/.bin/fbidq.html?beat-Ia), [kat-60L1](http://flybase.bio.indiana.edu/.bin/fbidq.html?kat-60L1), [mtg](http://flybase.bio.indiana.edu/.bin/fbidq.html?mtg), [ft](http://flybase.bio.indiana.edu/.bin/fbidq.html?ft), [Crz](http://flybase.bio.indiana.edu/.bin/fbidq.html?Crz), [CG12004](http://flybase.bio.indiana.edu/.bin/fbidq.html?CG12004), [CG32392](http://flybase.bio.indiana.edu/.bin/fbidq.html?CG32392), [l(2)k16918](http://flybase.bio.indiana.edu/.bin/fbidq.html?l(2)k16918), [Takr86C](http://flybase.bio.indiana.edu/.bin/fbidq.html?Takr86C), [sws](http://flybase.bio.indiana.edu/.bin/fbidq.html?sws), [disco](http://flybase.bio.indiana.edu/.bin/fbidq.html?disco), [DopR](http://flybase.bio.indiana.edu/.bin/fbidq.html?DopR), [D12](http://flybase.bio.indiana.edu/.bin/fbidq.html?D12), [mew](http://flybase.bio.indiana.edu/.bin/fbidq.html?mew), [didum](http://flybase.bio.indiana.edu/.bin/fbidq.html?didum), [Lis-1](http://flybase.bio.indiana.edu/.bin/fbidq.html?Lis-1),[ems](http://flybase.bio.indiana.edu/.bin/fbidq.html?ems), [CG16896](http://flybase.bio.indiana.edu/.bin/fbidq.html?CG16896), [eys](http://flybase.bio.indiana.edu/.bin/fbidq.html?eys), [tup](http://flybase.bio.indiana.edu/.bin/fbidq.html?tup), [Rab3](http://flybase.bio.indiana.edu/.bin/fbidq.html?Rab3), [CG6191](http://flybase.bio.indiana.edu/.bin/fbidq.html?CG6191), [alpha-catenin-related](http://flybase.bio.indiana.edu/.bin/fbidq.html?alpha-catenin-related), [trio](http://flybase.bio.indiana.edu/.bin/fbidq.html?trio), [pdm2](http://flybase.bio.indiana.edu/.bin/fbidq.html?pdm2), [RabX4](http://flybase.bio.indiana.edu/.bin/fbidq.html?RabX4), [Lkr](http://flybase.bio.indiana.edu/.bin/fbidq.html?Lkr), [spel1](http://flybase.bio.indiana.edu/.bin/fbidq.html?spel1), [CG17922](http://flybase.bio.indiana.edu/.bin/fbidq.html?CG17922), [CG31665](http://flybase.bio.indiana.edu/.bin/fbidq.html?CG31665), [Gad1](http://flybase.bio.indiana.edu/.bin/fbidq.html?Gad1), [CG7918](http://flybase.bio.indiana.edu/.bin/fbidq.html?CG7918), [Oseg6](http://flybase.bio.indiana.edu/.bin/fbidq.html?Oseg6), [CaMKII](http://flybase.bio.indiana.edu/.bin/fbidq.html?CaMKII), [DopR2](http://flybase.bio.indiana.edu/.bin/fbidq.html?DopR2), [l(3)psg2](http://flybase.bio.indiana.edu/.bin/fbidq.html?l(3)psg2), [nerfin-1](http://flybase.bio.indiana.edu/.bin/fbidq.html?nerfin-1), [pigs](http://flybase.bio.indiana.edu/.bin/fbidq.html?pigs), [5-HT7](http://flybase.bio.indiana.edu/.bin/fbidq.html?5-HT7), [Fancd2](http://flybase.bio.indiana.edu/.bin/fbidq.html?Fancd2), [Lgr3](http://flybase.bio.indiana.edu/.bin/fbidq.html?Lgr3), [polo](http://flybase.bio.indiana.edu/.bin/fbidq.html?polo), [chinmo](http://flybase.bio.indiana.edu/.bin/fbidq.html?chinmo), [Syn1](http://flybase.bio.indiana.edu/.bin/fbidq.html?Syn1), [mei-9](http://flybase.bio.indiana.edu/.bin/fbidq.html?mei-9), [Ac13E](http://flybase.bio.indiana.edu/.bin/fbidq.html?Ac13E),[Ssdp](http://flybase.bio.indiana.edu/.bin/fbidq.html?Ssdp), [CG8155](http://flybase.bio.indiana.edu/.bin/fbidq.html?CG8155), [CG10019](http://flybase.bio.indiana.edu/.bin/fbidq.html?CG10019), [fend](http://flybase.bio.indiana.edu/.bin/fbidq.html?fend), [dalao](http://flybase.bio.indiana.edu/.bin/fbidq.html?dalao), [cngl](http://flybase.bio.indiana.edu/.bin/fbidq.html?cngl), [wapl](http://flybase.bio.indiana.edu/.bin/fbidq.html?wapl), [sr](http://flybase.bio.indiana.edu/.bin/fbidq.html?sr), [nkd](http://flybase.bio.indiana.edu/.bin/fbidq.html?nkd), [RhoGEF3](http://flybase.bio.indiana.edu/.bin/fbidq.html?RhoGEF3), [Dsk](http://flybase.bio.indiana.edu/.bin/fbidq.html?Dsk), [RhoGEF4](http://flybase.bio.indiana.edu/.bin/fbidq.html?RhoGEF4), [vvl](http://flybase.bio.indiana.edu/.bin/fbidq.html?vvl), [Unc-76](http://flybase.bio.indiana.edu/.bin/fbidq.html?Unc-76), [betaInt-nu](http://flybase.bio.indiana.edu/.bin/fbidq.html?betaInt-nu), [sgg](http://flybase.bio.indiana.edu/.bin/fbidq.html?sgg), [Brd](http://flybase.bio.indiana.edu/.bin/fbidq.html?Brd), [Eip93F](http://flybase.bio.indiana.edu/.bin/fbidq.html?Eip93F), [PICK1](http://flybase.bio.indiana.edu/.bin/fbidq.html?PICK1), [Nmdar1](http://flybase.bio.indiana.edu/.bin/fbidq.html?Nmdar1), [His4r](http://flybase.bio.indiana.edu/.bin/fbidq.html?His4r), [gcm](http://flybase.bio.indiana.edu/.bin/fbidq.html?gcm), [mus210](http://flybase.bio.indiana.edu/.bin/fbidq.html?mus210), [Suv4-20](http://flybase.bio.indiana.edu/.bin/fbidq.html?Suv4-20), [Ccn](http://flybase.bio.indiana.edu/.bin/fbidq.html?Ccn), [Hsp67Bc](http://flybase.bio.indiana.edu/.bin/fbidq.html?Hsp67Bc), [ase](http://flybase.bio.indiana.edu/.bin/fbidq.html?ase), [Lerp](http://flybase.bio.indiana.edu/.bin/fbidq.html?Lerp), [CG8771](http://flybase.bio.indiana.edu/.bin/fbidq.html?CG8771), [MESK2](http://flybase.bio.indiana.edu/.bin/fbidq.html?MESK2), [sals](http://flybase.bio.indiana.edu/.bin/fbidq.html?sals), [Dat](http://flybase.bio.indiana.edu/.bin/fbidq.html?Dat), [Rat1](http://flybase.bio.indiana.edu/.bin/fbidq.html?Rat1),[Task6](http://flybase.bio.indiana.edu/.bin/fbidq.html?Task6), [CG18273](http://flybase.bio.indiana.edu/.bin/fbidq.html?CG18273), [eIF4G2](http://flybase.bio.indiana.edu/.bin/fbidq.html?eIF4G2), [fred](http://flybase.bio.indiana.edu/.bin/fbidq.html?fred), [Ca-beta](http://flybase.bio.indiana.edu/.bin/fbidq.html?Ca-beta), [ush](http://flybase.bio.indiana.edu/.bin/fbidq.html?ush), [Fak56D](http://flybase.bio.indiana.edu/.bin/fbidq.html?Fak56D), [Task7](http://flybase.bio.indiana.edu/.bin/fbidq.html?Task7), [Kap3](http://flybase.bio.indiana.edu/.bin/fbidq.html?Kap3), [ko](http://flybase.bio.indiana.edu/.bin/fbidq.html?ko), [Takr99D](http://flybase.bio.indiana.edu/.bin/fbidq.html?Takr99D), [Eip71CD](http://flybase.bio.indiana.edu/.bin/fbidq.html?Eip71CD), [Hex-A](http://flybase.bio.indiana.edu/.bin/fbidq.html?Hex-A), [egg](http://flybase.bio.indiana.edu/.bin/fbidq.html?egg), [CG13692](http://flybase.bio.indiana.edu/.bin/fbidq.html?CG13692), [CG5337](http://flybase.bio.indiana.edu/.bin/fbidq.html?CG5337), [rtet](http://flybase.bio.indiana.edu/.bin/fbidq.html?rtet), [CG11750](http://flybase.bio.indiana.edu/.bin/fbidq.html?CG11750), [CG12424](http://flybase.bio.indiana.edu/.bin/fbidq.html?CG12424), [Wnt5](http://flybase.bio.indiana.edu/.bin/fbidq.html?Wnt5), [SF2](http://flybase.bio.indiana.edu/.bin/fbidq.html?SF2), [Sln](http://flybase.bio.indiana.edu/.bin/fbidq.html?Sln), [CG11897](http://flybase.bio.indiana.edu/.bin/fbidq.html?CG11897), [fus](http://flybase.bio.indiana.edu/.bin/fbidq.html?fus), [sif](http://flybase.bio.indiana.edu/.bin/fbidq.html?sif), [Tbh](http://flybase.bio.indiana.edu/.bin/fbidq.html?Tbh), [Aac11](http://flybase.bio.indiana.edu/.bin/fbidq.html?Aac11), [MTA1-like](http://flybase.bio.indiana.edu/.bin/fbidq.html?MTA1-like), [Sas-4](http://flybase.bio.indiana.edu/.bin/fbidq.html?Sas-4), [cal1](http://flybase.bio.indiana.edu/.bin/fbidq.html?cal1), [ssh](http://flybase.bio.indiana.edu/.bin/fbidq.html?ssh), [tadr](http://flybase.bio.indiana.edu/.bin/fbidq.html?tadr),[CG42541](http://flybase.bio.indiana.edu/.bin/fbidq.html?CG42541), [PIP5K59B](http://flybase.bio.indiana.edu/.bin/fbidq.html?PIP5K59B), [mle](http://flybase.bio.indiana.edu/.bin/fbidq.html?mle), [Hr51](http://flybase.bio.indiana.edu/.bin/fbidq.html?Hr51), [lap](http://flybase.bio.indiana.edu/.bin/fbidq.html?lap), [CG11155](http://flybase.bio.indiana.edu/.bin/fbidq.html?CG11155), [wg](http://flybase.bio.indiana.edu/.bin/fbidq.html?wg), [CG31030](http://flybase.bio.indiana.edu/.bin/fbidq.html?CG31030), [CG11077](http://flybase.bio.indiana.edu/.bin/fbidq.html?CG11077), [fkh](http://flybase.bio.indiana.edu/.bin/fbidq.html?fkh), [Spps](http://flybase.bio.indiana.edu/.bin/fbidq.html?Spps), [Rhp](http://flybase.bio.indiana.edu/.bin/fbidq.html?Rhp), [RpL28](http://flybase.bio.indiana.edu/.bin/fbidq.html?RpL28), [klg](http://flybase.bio.indiana.edu/.bin/fbidq.html?klg), [en](http://flybase.bio.indiana.edu/.bin/fbidq.html?en), [sfl](http://flybase.bio.indiana.edu/.bin/fbidq.html?sfl), [CG13229](http://flybase.bio.indiana.edu/.bin/fbidq.html?CG13229), [Sip1](http://flybase.bio.indiana.edu/.bin/fbidq.html?Sip1), [Pde11](http://flybase.bio.indiana.edu/.bin/fbidq.html?Pde11), [SerT](http://flybase.bio.indiana.edu/.bin/fbidq.html?SerT), [Utx](http://flybase.bio.indiana.edu/.bin/fbidq.html?Utx), [bt](http://flybase.bio.indiana.edu/.bin/fbidq.html?bt), [Ct](http://flybase.bio.indiana.edu/.bin/fbidq.html?Ct), [fz2](http://flybase.bio.indiana.edu/.bin/fbidq.html?fz2), [CG8407](http://flybase.bio.indiana.edu/.bin/fbidq.html?CG8407), [CG14375](http://flybase.bio.indiana.edu/.bin/fbidq.html?CG14375), [Ocho](http://flybase.bio.indiana.edu/.bin/fbidq.html?Ocho), [numb](http://flybase.bio.indiana.edu/.bin/fbidq.html?numb), [Nnf1b](http://flybase.bio.indiana.edu/.bin/fbidq.html?Nnf1b), [CG42256](http://flybase.bio.indiana.edu/.bin/fbidq.html?CG42256), [mus101](http://flybase.bio.indiana.edu/.bin/fbidq.html?mus101), [stj](http://flybase.bio.indiana.edu/.bin/fbidq.html?stj), [Sxl](http://flybase.bio.indiana.edu/.bin/fbidq.html?Sxl), [CG33007](http://flybase.bio.indiana.edu/.bin/fbidq.html?CG33007), [JIL-1](http://flybase.bio.indiana.edu/.bin/fbidq.html?JIL-1), [Nf1](http://flybase.bio.indiana.edu/.bin/fbidq.html?Nf1), [Drep-3](http://flybase.bio.indiana.edu/.bin/fbidq.html?Drep-3), [sns](http://flybase.bio.indiana.edu/.bin/fbidq.html?sns), [AR-2](http://flybase.bio.indiana.edu/.bin/fbidq.html?AR-2), [Topors](http://flybase.bio.indiana.edu/.bin/fbidq.html?Topors), [tok](http://flybase.bio.indiana.edu/.bin/fbidq.html?tok), [Sytalpha](http://flybase.bio.indiana.edu/.bin/fbidq.html?Sytalpha), [Ptp99A](http://flybase.bio.indiana.edu/.bin/fbidq.html?Ptp99A), [CG31961](http://flybase.bio.indiana.edu/.bin/fbidq.html?CG31961), [Tango10](http://flybase.bio.indiana.edu/.bin/fbidq.html?Tango10), [Oseg4](http://flybase.bio.indiana.edu/.bin/fbidq.html?Oseg4), [NC2alpha](http://flybase.bio.indiana.edu/.bin/fbidq.html?NC2alpha), [blue](http://flybase.bio.indiana.edu/.bin/fbidq.html?blue), [mod(mdg4)](http://flybase.bio.indiana.edu/.bin/fbidq.html?mod(mdg4)), [Taf12L](http://flybase.bio.indiana.edu/.bin/fbidq.html?Taf12L), [Galpha49B](http://flybase.bio.indiana.edu/.bin/fbidq.html?Galpha49B), [Snoo](http://flybase.bio.indiana.edu/.bin/fbidq.html?Snoo), [rut](http://flybase.bio.indiana.edu/.bin/fbidq.html?rut), [RhoGAP100F](http://flybase.bio.indiana.edu/.bin/fbidq.html?RhoGAP100F), [nub](http://flybase.bio.indiana.edu/.bin/fbidq.html?nub), [uif](http://flybase.bio.indiana.edu/.bin/fbidq.html?uif), [Mmp2](http://flybase.bio.indiana.edu/.bin/fbidq.html?Mmp2), [B-H1](http://flybase.bio.indiana.edu/.bin/fbidq.html?B-H1), [opa1-like](http://flybase.bio.indiana.edu/.bin/fbidq.html?opa1-like), [Nplp1](http://flybase.bio.indiana.edu/.bin/fbidq.html?Nplp1), [Nipped-A](http://flybase.bio.indiana.edu/.bin/fbidq.html?Nipped-A), [pim](http://flybase.bio.indiana.edu/.bin/fbidq.html?pim), [Vha68-1](http://flybase.bio.indiana.edu/.bin/fbidq.html?Vha68-1), [CG32206](http://flybase.bio.indiana.edu/.bin/fbidq.html?CG32206),[Spf45](http://flybase.bio.indiana.edu/.bin/fbidq.html?Spf45), [lid](http://flybase.bio.indiana.edu/.bin/fbidq.html?lid), [hth](http://flybase.bio.indiana.edu/.bin/fbidq.html?hth), [pzg](http://flybase.bio.indiana.edu/.bin/fbidq.html?pzg), [14-3-3zeta](http://flybase.bio.indiana.edu/.bin/fbidq.html?14-3-3zeta), [lea](http://flybase.bio.indiana.edu/.bin/fbidq.html?lea), [shakB](http://flybase.bio.indiana.edu/.bin/fbidq.html?shakB), [Hey](http://flybase.bio.indiana.edu/.bin/fbidq.html?Hey), [plexB](http://flybase.bio.indiana.edu/.bin/fbidq.html?plexB), [AlstR](http://flybase.bio.indiana.edu/.bin/fbidq.html?AlstR), [loqs](http://flybase.bio.indiana.edu/.bin/fbidq.html?loqs), [Klp64D](http://flybase.bio.indiana.edu/.bin/fbidq.html?Klp64D), [icln](http://flybase.bio.indiana.edu/.bin/fbidq.html?icln), [mars](http://flybase.bio.indiana.edu/.bin/fbidq.html?mars), [CG9095](http://flybase.bio.indiana.edu/.bin/fbidq.html?CG9095), [CG32226](http://flybase.bio.indiana.edu/.bin/fbidq.html?CG32226), [IM10](http://flybase.bio.indiana.edu/.bin/fbidq.html?IM10), [beat-Vc](http://flybase.bio.indiana.edu/.bin/fbidq.html?beat-Vc), [neur](http://flybase.bio.indiana.edu/.bin/fbidq.html?neur), [CG33543](http://flybase.bio.indiana.edu/.bin/fbidq.html?CG33543), [CycE](http://flybase.bio.indiana.edu/.bin/fbidq.html?CycE), [Brf](http://flybase.bio.indiana.edu/.bin/fbidq.html?Brf), [Cep97](http://flybase.bio.indiana.edu/.bin/fbidq.html?Cep97), [escl](http://flybase.bio.indiana.edu/.bin/fbidq.html?escl), [CG32149](http://flybase.bio.indiana.edu/.bin/fbidq.html?CG32149), [Vdup1](http://flybase.bio.indiana.edu/.bin/fbidq.html?Vdup1), [mr](http://flybase.bio.indiana.edu/.bin/fbidq.html?mr), [orb2](http://flybase.bio.indiana.edu/.bin/fbidq.html?orb2), [synaptogyrin](http://flybase.bio.indiana.edu/.bin/fbidq.html?synaptogyrin), [sim](http://flybase.bio.indiana.edu/.bin/fbidq.html?sim), [gro](http://flybase.bio.indiana.edu/.bin/fbidq.html?gro), [Rya-r44F](http://flybase.bio.indiana.edu/.bin/fbidq.html?Rya-r44F), [scyl](http://flybase.bio.indiana.edu/.bin/fbidq.html?scyl), [hkl](http://flybase.bio.indiana.edu/.bin/fbidq.html?hkl),[Rpd3](http://flybase.bio.indiana.edu/.bin/fbidq.html?Rpd3), [CG14367](http://flybase.bio.indiana.edu/.bin/fbidq.html?CG14367), [CG10107](http://flybase.bio.indiana.edu/.bin/fbidq.html?CG10107), [aret](http://flybase.bio.indiana.edu/.bin/fbidq.html?aret), [HLHm7](http://flybase.bio.indiana.edu/.bin/fbidq.html?HLHm7), [tow](http://flybase.bio.indiana.edu/.bin/fbidq.html?tow), [CG15609](http://flybase.bio.indiana.edu/.bin/fbidq.html?CG15609), [l(1)G0148](http://flybase.bio.indiana.edu/.bin/fbidq.html?l(1)G0148), [sei](http://flybase.bio.indiana.edu/.bin/fbidq.html?sei), [CG12187](http://flybase.bio.indiana.edu/.bin/fbidq.html?CG12187), [Pten](http://flybase.bio.indiana.edu/.bin/fbidq.html?Pten), [wda](http://flybase.bio.indiana.edu/.bin/fbidq.html?wda), [unc-13](http://flybase.bio.indiana.edu/.bin/fbidq.html?unc-13), [npf](http://flybase.bio.indiana.edu/.bin/fbidq.html?npf), [mira](http://flybase.bio.indiana.edu/.bin/fbidq.html?mira), [Myo10A](http://flybase.bio.indiana.edu/.bin/fbidq.html?Myo10A), [CG12194](http://flybase.bio.indiana.edu/.bin/fbidq.html?CG12194), [Sug](http://flybase.bio.indiana.edu/.bin/fbidq.html?Sug), [Spindly](http://flybase.bio.indiana.edu/.bin/fbidq.html?Spindly), [Dip3](http://flybase.bio.indiana.edu/.bin/fbidq.html?Dip3), [Drep-2](http://flybase.bio.indiana.edu/.bin/fbidq.html?Drep-2), [oa2](http://flybase.bio.indiana.edu/.bin/fbidq.html?oa2), [elk](http://flybase.bio.indiana.edu/.bin/fbidq.html?elk), [RpS11](http://flybase.bio.indiana.edu/.bin/fbidq.html?RpS11), [phl](http://flybase.bio.indiana.edu/.bin/fbidq.html?phl), [Rab26](http://flybase.bio.indiana.edu/.bin/fbidq.html?Rab26), [Pde6](http://flybase.bio.indiana.edu/.bin/fbidq.html?Pde6), [sNPF](http://flybase.bio.indiana.edu/.bin/fbidq.html?sNPF), [CG4893](http://flybase.bio.indiana.edu/.bin/fbidq.html?CG4893), [plx](http://flybase.bio.indiana.edu/.bin/fbidq.html?plx), [CG9098](http://flybase.bio.indiana.edu/.bin/fbidq.html?CG9098), [CG8468](http://flybase.bio.indiana.edu/.bin/fbidq.html?CG8468),[CG8108](http://flybase.bio.indiana.edu/.bin/fbidq.html?CG8108), [IFa](http://flybase.bio.indiana.edu/.bin/fbidq.html?IFa), [Alk](http://flybase.bio.indiana.edu/.bin/fbidq.html?Alk), [CG7708](http://flybase.bio.indiana.edu/.bin/fbidq.html?CG7708), [esn](http://flybase.bio.indiana.edu/.bin/fbidq.html?esn), [Vang](http://flybase.bio.indiana.edu/.bin/fbidq.html?Vang), [Sin3A](http://flybase.bio.indiana.edu/.bin/fbidq.html?Sin3A), [pot](http://flybase.bio.indiana.edu/.bin/fbidq.html?pot), [Pde1c](http://flybase.bio.indiana.edu/.bin/fbidq.html?Pde1c), [casp](http://flybase.bio.indiana.edu/.bin/fbidq.html?casp), [Smox](http://flybase.bio.indiana.edu/.bin/fbidq.html?Smox), [CG31475](http://flybase.bio.indiana.edu/.bin/fbidq.html?CG31475), [Shaw](http://flybase.bio.indiana.edu/.bin/fbidq.html?Shaw), [mei-S332](http://flybase.bio.indiana.edu/.bin/fbidq.html?mei-S332), [Plc21C](http://flybase.bio.indiana.edu/.bin/fbidq.html?Plc21C), [srpk79D](http://flybase.bio.indiana.edu/.bin/fbidq.html?srpk79D), [CG6208](http://flybase.bio.indiana.edu/.bin/fbidq.html?CG6208), [Antp](http://flybase.bio.indiana.edu/.bin/fbidq.html?Antp), [CAP-D2](http://flybase.bio.indiana.edu/.bin/fbidq.html?CAP-D2), [GRHRII](http://flybase.bio.indiana.edu/.bin/fbidq.html?GRHRII), [glec](http://flybase.bio.indiana.edu/.bin/fbidq.html?glec), [Rdl](http://flybase.bio.indiana.edu/.bin/fbidq.html?Rdl), [l(3)neo38](http://flybase.bio.indiana.edu/.bin/fbidq.html?l(3)neo38), [CG10362](http://flybase.bio.indiana.edu/.bin/fbidq.html?CG10362), [Ast-C](http://flybase.bio.indiana.edu/.bin/fbidq.html?Ast-C), [Gbeta5](http://flybase.bio.indiana.edu/.bin/fbidq.html?Gbeta5), [cindr](http://flybase.bio.indiana.edu/.bin/fbidq.html?cindr), [comm3](http://flybase.bio.indiana.edu/.bin/fbidq.html?comm3), [beat-VI](http://flybase.bio.indiana.edu/.bin/fbidq.html?beat-VI), [Nhe3](http://flybase.bio.indiana.edu/.bin/fbidq.html?Nhe3), [Ptx1](http://flybase.bio.indiana.edu/.bin/fbidq.html?Ptx1),[CG11071](http://flybase.bio.indiana.edu/.bin/fbidq.html?CG11071), [CG6114](http://flybase.bio.indiana.edu/.bin/fbidq.html?CG6114), [Shal](http://flybase.bio.indiana.edu/.bin/fbidq.html?Shal), [hug](http://flybase.bio.indiana.edu/.bin/fbidq.html?hug), [Gpo-1](http://flybase.bio.indiana.edu/.bin/fbidq.html?Gpo-1), [para](http://flybase.bio.indiana.edu/.bin/fbidq.html?para), [tsh](http://flybase.bio.indiana.edu/.bin/fbidq.html?tsh), [sm](http://flybase.bio.indiana.edu/.bin/fbidq.html?sm), [Syn2](http://flybase.bio.indiana.edu/.bin/fbidq.html?Syn2), [eas](http://flybase.bio.indiana.edu/.bin/fbidq.html?eas), [e(y)3](http://flybase.bio.indiana.edu/.bin/fbidq.html?e(y)3), [Sry-alpha](http://flybase.bio.indiana.edu/.bin/fbidq.html?Sry-alpha), [CRMP](http://flybase.bio.indiana.edu/.bin/fbidq.html?CRMP), [Cdk5](http://flybase.bio.indiana.edu/.bin/fbidq.html?Cdk5), [Cep135](http://flybase.bio.indiana.edu/.bin/fbidq.html?Cep135), [oc](http://flybase.bio.indiana.edu/.bin/fbidq.html?oc), [CG4641](http://flybase.bio.indiana.edu/.bin/fbidq.html?CG4641), [spt4](http://flybase.bio.indiana.edu/.bin/fbidq.html?spt4), [CG3964](http://flybase.bio.indiana.edu/.bin/fbidq.html?CG3964), [ey](http://flybase.bio.indiana.edu/.bin/fbidq.html?ey), [Mip](http://flybase.bio.indiana.edu/.bin/fbidq.html?Mip), [baz](http://flybase.bio.indiana.edu/.bin/fbidq.html?baz), [Oamb](http://flybase.bio.indiana.edu/.bin/fbidq.html?Oamb), [SK](http://flybase.bio.indiana.edu/.bin/fbidq.html?SK), [msd5](http://flybase.bio.indiana.edu/.bin/fbidq.html?msd5), [CG34381](http://flybase.bio.indiana.edu/.bin/fbidq.html?CG34381), [rst](http://flybase.bio.indiana.edu/.bin/fbidq.html?rst), [l(1)sc](http://flybase.bio.indiana.edu/.bin/fbidq.html?l(1)sc), [comm](http://flybase.bio.indiana.edu/.bin/fbidq.html?comm), [grh](http://flybase.bio.indiana.edu/.bin/fbidq.html?grh), [Vsx1](http://flybase.bio.indiana.edu/.bin/fbidq.html?Vsx1), [Cdk5alpha](http://flybase.bio.indiana.edu/.bin/fbidq.html?Cdk5alpha), [yemalpha](http://flybase.bio.indiana.edu/.bin/fbidq.html?yemalpha), [Vmat](http://flybase.bio.indiana.edu/.bin/fbidq.html?Vmat), [qtc](http://flybase.bio.indiana.edu/.bin/fbidq.html?qtc),[Cha](http://flybase.bio.indiana.edu/.bin/fbidq.html?Cha), [crol](http://flybase.bio.indiana.edu/.bin/fbidq.html?crol), [E(Pc)](http://flybase.bio.indiana.edu/.bin/fbidq.html?E(Pc)), [retn](http://flybase.bio.indiana.edu/.bin/fbidq.html?retn), [Sap130](http://flybase.bio.indiana.edu/.bin/fbidq.html?Sap130), [Ac3](http://flybase.bio.indiana.edu/.bin/fbidq.html?Ac3), [rl](http://flybase.bio.indiana.edu/.bin/fbidq.html?rl), [Grip163](http://flybase.bio.indiana.edu/.bin/fbidq.html?Grip163), [Syt1](http://flybase.bio.indiana.edu/.bin/fbidq.html?Syt1), [CG17760](http://flybase.bio.indiana.edu/.bin/fbidq.html?CG17760), [Kdm4B](http://flybase.bio.indiana.edu/.bin/fbidq.html?Kdm4B), [Nup153](http://flybase.bio.indiana.edu/.bin/fbidq.html?Nup153), [CG32683](http://flybase.bio.indiana.edu/.bin/fbidq.html?CG32683), [tey](http://flybase.bio.indiana.edu/.bin/fbidq.html?tey), [CG33960](http://flybase.bio.indiana.edu/.bin/fbidq.html?CG33960), [CG1962](http://flybase.bio.indiana.edu/.bin/fbidq.html?CG1962), [Pabp2](http://flybase.bio.indiana.edu/.bin/fbidq.html?Pabp2), [Arp8](http://flybase.bio.indiana.edu/.bin/fbidq.html?Arp8), [acj6](http://flybase.bio.indiana.edu/.bin/fbidq.html?acj6), [ana3](http://flybase.bio.indiana.edu/.bin/fbidq.html?ana3), [caup](http://flybase.bio.indiana.edu/.bin/fbidq.html?caup), [Cbp80](http://flybase.bio.indiana.edu/.bin/fbidq.html?Cbp80), [vimar](http://flybase.bio.indiana.edu/.bin/fbidq.html?vimar), [Dg](http://flybase.bio.indiana.edu/.bin/fbidq.html?Dg), [Trim9](http://flybase.bio.indiana.edu/.bin/fbidq.html?Trim9), [GABA-B-R3](http://flybase.bio.indiana.edu/.bin/fbidq.html?GABA-B-R3), [Evi5](http://flybase.bio.indiana.edu/.bin/fbidq.html?Evi5), [CG34127](http://flybase.bio.indiana.edu/.bin/fbidq.html?CG34127), [br](http://flybase.bio.indiana.edu/.bin/fbidq.html?br), [Gycalpha99B](http://flybase.bio.indiana.edu/.bin/fbidq.html?Gycalpha99B), [CG7154](http://flybase.bio.indiana.edu/.bin/fbidq.html?CG7154), [neo](http://flybase.bio.indiana.edu/.bin/fbidq.html?neo),[robo3](http://flybase.bio.indiana.edu/.bin/fbidq.html?robo3), [CG11376](http://flybase.bio.indiana.edu/.bin/fbidq.html?CG11376), [Fim](http://flybase.bio.indiana.edu/.bin/fbidq.html?Fim), [CG6126](http://flybase.bio.indiana.edu/.bin/fbidq.html?CG6126), [PNUTS](http://flybase.bio.indiana.edu/.bin/fbidq.html?PNUTS), [lola](http://flybase.bio.indiana.edu/.bin/fbidq.html?lola), [unc-104](http://flybase.bio.indiana.edu/.bin/fbidq.html?unc-104), [VAChT](http://flybase.bio.indiana.edu/.bin/fbidq.html?VAChT), [CG7627](http://flybase.bio.indiana.edu/.bin/fbidq.html?CG7627), [Pcl](http://flybase.bio.indiana.edu/.bin/fbidq.html?Pcl), [Spc105R](http://flybase.bio.indiana.edu/.bin/fbidq.html?Spc105R), [CG31158](http://flybase.bio.indiana.edu/.bin/fbidq.html?CG31158), [Appl](http://flybase.bio.indiana.edu/.bin/fbidq.html?Appl), [metro](http://flybase.bio.indiana.edu/.bin/fbidq.html?metro), [erm](http://flybase.bio.indiana.edu/.bin/fbidq.html?erm), [Nrk](http://flybase.bio.indiana.edu/.bin/fbidq.html?Nrk), [dmt](http://flybase.bio.indiana.edu/.bin/fbidq.html?dmt), [CG4328](http://flybase.bio.indiana.edu/.bin/fbidq.html?CG4328), [Ilp3](http://flybase.bio.indiana.edu/.bin/fbidq.html?Ilp3), [CG10249](http://flybase.bio.indiana.edu/.bin/fbidq.html?CG10249), [Taf4](http://flybase.bio.indiana.edu/.bin/fbidq.html?Taf4), [Nrg](http://flybase.bio.indiana.edu/.bin/fbidq.html?Nrg), [insc](http://flybase.bio.indiana.edu/.bin/fbidq.html?insc), [CG34384](http://flybase.bio.indiana.edu/.bin/fbidq.html?CG34384), [jeb](http://flybase.bio.indiana.edu/.bin/fbidq.html?jeb), [CG32447](http://flybase.bio.indiana.edu/.bin/fbidq.html?CG32447), [CG31530](http://flybase.bio.indiana.edu/.bin/fbidq.html?CG31530), [Actbeta](http://flybase.bio.indiana.edu/.bin/fbidq.html?Actbeta), [stau](http://flybase.bio.indiana.edu/.bin/fbidq.html?stau), [beat-Ib](http://flybase.bio.indiana.edu/.bin/fbidq.html?beat-Ib), [beat-IIb](http://flybase.bio.indiana.edu/.bin/fbidq.html?beat-IIb),[CG31064](http://flybase.bio.indiana.edu/.bin/fbidq.html?CG31064), [otk](http://flybase.bio.indiana.edu/.bin/fbidq.html?otk), [phyl](http://flybase.bio.indiana.edu/.bin/fbidq.html?phyl), [vg](http://flybase.bio.indiana.edu/.bin/fbidq.html?vg), [fz](http://flybase.bio.indiana.edu/.bin/fbidq.html?fz), [robo](http://flybase.bio.indiana.edu/.bin/fbidq.html?robo), [phr](http://flybase.bio.indiana.edu/.bin/fbidq.html?phr), [bsk](http://flybase.bio.indiana.edu/.bin/fbidq.html?bsk), [brm](http://flybase.bio.indiana.edu/.bin/fbidq.html?brm), [E(var)3-9](http://flybase.bio.indiana.edu/.bin/fbidq.html?E(var)3-9), [nAcRalpha-30D](http://flybase.bio.indiana.edu/.bin/fbidq.html?nAcRalpha-30D), [CG11652](http://flybase.bio.indiana.edu/.bin/fbidq.html?CG11652), [CG13830](http://flybase.bio.indiana.edu/.bin/fbidq.html?CG13830), [CG6006](http://flybase.bio.indiana.edu/.bin/fbidq.html?CG6006), [sno](http://flybase.bio.indiana.edu/.bin/fbidq.html?sno), [hig](http://flybase.bio.indiana.edu/.bin/fbidq.html?hig), [RhoGAPp190](http://flybase.bio.indiana.edu/.bin/fbidq.html?RhoGAPp190), [Cad96Ca](http://flybase.bio.indiana.edu/.bin/fbidq.html?Cad96Ca), [Atac1](http://flybase.bio.indiana.edu/.bin/fbidq.html?Atac1), [Sfmbt](http://flybase.bio.indiana.edu/.bin/fbidq.html?Sfmbt), [ara](http://flybase.bio.indiana.edu/.bin/fbidq.html?ara), [pHCl](http://flybase.bio.indiana.edu/.bin/fbidq.html?pHCl), [mam](http://flybase.bio.indiana.edu/.bin/fbidq.html?mam), [CG30158](http://flybase.bio.indiana.edu/.bin/fbidq.html?CG30158), [pan](http://flybase.bio.indiana.edu/.bin/fbidq.html?pan), [PKD](http://flybase.bio.indiana.edu/.bin/fbidq.html?PKD), [grn](http://flybase.bio.indiana.edu/.bin/fbidq.html?grn), [CG4019](http://flybase.bio.indiana.edu/.bin/fbidq.html?CG4019), [HLHm5](http://flybase.bio.indiana.edu/.bin/fbidq.html?HLHm5), [beat-IIIc](http://flybase.bio.indiana.edu/.bin/fbidq.html?beat-IIIc), [dap](http://flybase.bio.indiana.edu/.bin/fbidq.html?dap), [Bx](http://flybase.bio.indiana.edu/.bin/fbidq.html?Bx),[Mob2](http://flybase.bio.indiana.edu/.bin/fbidq.html?Mob2), [pros](http://flybase.bio.indiana.edu/.bin/fbidq.html?pros), [CG2993](http://flybase.bio.indiana.edu/.bin/fbidq.html?CG2993), [Gen](http://flybase.bio.indiana.edu/.bin/fbidq.html?Gen), [mthl8](http://flybase.bio.indiana.edu/.bin/fbidq.html?mthl8), [CG1463](http://flybase.bio.indiana.edu/.bin/fbidq.html?CG1463), [Ccap](http://flybase.bio.indiana.edu/.bin/fbidq.html?Ccap), [tipE](http://flybase.bio.indiana.edu/.bin/fbidq.html?tipE), [Wnt4](http://flybase.bio.indiana.edu/.bin/fbidq.html?Wnt4), [rictor](http://flybase.bio.indiana.edu/.bin/fbidq.html?rictor), [Mad1](http://flybase.bio.indiana.edu/.bin/fbidq.html?Mad1), [CG30372](http://flybase.bio.indiana.edu/.bin/fbidq.html?CG30372), [CG10188](http://flybase.bio.indiana.edu/.bin/fbidq.html?CG10188), [CG42629](http://flybase.bio.indiana.edu/.bin/fbidq.html?CG42629), [CG31760](http://flybase.bio.indiana.edu/.bin/fbidq.html?CG31760), [Klp54D](http://flybase.bio.indiana.edu/.bin/fbidq.html?Klp54D), [pr-set7](http://flybase.bio.indiana.edu/.bin/fbidq.html?pr-set7), [bchs](http://flybase.bio.indiana.edu/.bin/fbidq.html?bchs), [RpS5b](http://flybase.bio.indiana.edu/.bin/fbidq.html?RpS5b), [pk](http://flybase.bio.indiana.edu/.bin/fbidq.html?pk), [dsx](http://flybase.bio.indiana.edu/.bin/fbidq.html?dsx), [Dh](http://flybase.bio.indiana.edu/.bin/fbidq.html?Dh), [Scgbeta](http://flybase.bio.indiana.edu/.bin/fbidq.html?Scgbeta), [betaTub85D](http://flybase.bio.indiana.edu/.bin/fbidq.html?betaTub85D), [klu](http://flybase.bio.indiana.edu/.bin/fbidq.html?klu), [SPR](http://flybase.bio.indiana.edu/.bin/fbidq.html?SPR), [sqz](http://flybase.bio.indiana.edu/.bin/fbidq.html?sqz), [Cad74A](http://flybase.bio.indiana.edu/.bin/fbidq.html?Cad74A), [Ca-alpha1D](http://flybase.bio.indiana.edu/.bin/fbidq.html?Ca-alpha1D), [Atet](http://flybase.bio.indiana.edu/.bin/fbidq.html?Atet), [tld](http://flybase.bio.indiana.edu/.bin/fbidq.html?tld),[NaCP60E](http://flybase.bio.indiana.edu/.bin/fbidq.html?NaCP60E), [rg](http://flybase.bio.indiana.edu/.bin/fbidq.html?rg), [CG9121](http://flybase.bio.indiana.edu/.bin/fbidq.html?CG9121), [CG1909](http://flybase.bio.indiana.edu/.bin/fbidq.html?CG1909), [gcm2](http://flybase.bio.indiana.edu/.bin/fbidq.html?gcm2), [inaE](http://flybase.bio.indiana.edu/.bin/fbidq.html?inaE), [mp](http://flybase.bio.indiana.edu/.bin/fbidq.html?mp), [CG5890](http://flybase.bio.indiana.edu/.bin/fbidq.html?CG5890), [CanB](http://flybase.bio.indiana.edu/.bin/fbidq.html?CanB), [SP2353](http://flybase.bio.indiana.edu/.bin/fbidq.html?SP2353), [can](http://flybase.bio.indiana.edu/.bin/fbidq.html?can), [Smc5](http://flybase.bio.indiana.edu/.bin/fbidq.html?Smc5), [Pask](http://flybase.bio.indiana.edu/.bin/fbidq.html?Pask), [rab3-GEF](http://flybase.bio.indiana.edu/.bin/fbidq.html?rab3-GEF), [CG13253](http://flybase.bio.indiana.edu/.bin/fbidq.html?CG13253), [ovo](http://flybase.bio.indiana.edu/.bin/fbidq.html?ovo), [CG6197](http://flybase.bio.indiana.edu/.bin/fbidq.html?CG6197), [phol](http://flybase.bio.indiana.edu/.bin/fbidq.html?phol), [CG32685](http://flybase.bio.indiana.edu/.bin/fbidq.html?CG32685), [Dsp1](http://flybase.bio.indiana.edu/.bin/fbidq.html?Dsp1), [alphaTub84D](http://flybase.bio.indiana.edu/.bin/fbidq.html?alphaTub84D), [ds](http://flybase.bio.indiana.edu/.bin/fbidq.html?ds), [mei-P26](http://flybase.bio.indiana.edu/.bin/fbidq.html?mei-P26), [Syt4](http://flybase.bio.indiana.edu/.bin/fbidq.html?Syt4), [CG11033](http://flybase.bio.indiana.edu/.bin/fbidq.html?CG11033), [Incenp](http://flybase.bio.indiana.edu/.bin/fbidq.html?Incenp), [Rbp9](http://flybase.bio.indiana.edu/.bin/fbidq.html?Rbp9), [SIFR](http://flybase.bio.indiana.edu/.bin/fbidq.html?SIFR), [CG2061](http://flybase.bio.indiana.edu/.bin/fbidq.html?CG2061), [Ast](http://flybase.bio.indiana.edu/.bin/fbidq.html?Ast), [Drl-2](http://flybase.bio.indiana.edu/.bin/fbidq.html?Drl-2), [CG12858](http://flybase.bio.indiana.edu/.bin/fbidq.html?CG12858), [Tsp42Ej](http://flybase.bio.indiana.edu/.bin/fbidq.html?Tsp42Ej), [CG32944](http://flybase.bio.indiana.edu/.bin/fbidq.html?CG32944), [Tk](http://flybase.bio.indiana.edu/.bin/fbidq.html?Tk), [Ilp5](http://flybase.bio.indiana.edu/.bin/fbidq.html?Ilp5), [CG10336](http://flybase.bio.indiana.edu/.bin/fbidq.html?CG10336), [Gap1](http://flybase.bio.indiana.edu/.bin/fbidq.html?Gap1), [Toll-6](http://flybase.bio.indiana.edu/.bin/fbidq.html?Toll-6), [CG3227](http://flybase.bio.indiana.edu/.bin/fbidq.html?CG3227), [beat-IV](http://flybase.bio.indiana.edu/.bin/fbidq.html?beat-IV), [run](http://flybase.bio.indiana.edu/.bin/fbidq.html?run), [MESR4](http://flybase.bio.indiana.edu/.bin/fbidq.html?MESR4), [sNPF-R](http://flybase.bio.indiana.edu/.bin/fbidq.html?sNPF-R), [tara](http://flybase.bio.indiana.edu/.bin/fbidq.html?tara), [Hel89B](http://flybase.bio.indiana.edu/.bin/fbidq.html?Hel89B), [koko](http://flybase.bio.indiana.edu/.bin/fbidq.html?koko), [stan](http://flybase.bio.indiana.edu/.bin/fbidq.html?stan), [CanA-14F](http://flybase.bio.indiana.edu/.bin/fbidq.html?CanA-14F), [tutl](http://flybase.bio.indiana.edu/.bin/fbidq.html?tutl), [Ptth](http://flybase.bio.indiana.edu/.bin/fbidq.html?Ptth), [Rab2](http://flybase.bio.indiana.edu/.bin/fbidq.html?Rab2), [Ilp2](http://flybase.bio.indiana.edu/.bin/fbidq.html?Ilp2), [Fancl](http://flybase.bio.indiana.edu/.bin/fbidq.html?Fancl), [ph-p](http://flybase.bio.indiana.edu/.bin/fbidq.html?ph-p), [Frq1](http://flybase.bio.indiana.edu/.bin/fbidq.html?Frq1), [CG7757](http://flybase.bio.indiana.edu/.bin/fbidq.html?CG7757), [SPoCk](http://flybase.bio.indiana.edu/.bin/fbidq.html?SPoCk), [sna](http://flybase.bio.indiana.edu/.bin/fbidq.html?sna), [Bre1](http://flybase.bio.indiana.edu/.bin/fbidq.html?Bre1), [Taf1](http://flybase.bio.indiana.edu/.bin/fbidq.html?Taf1), [Ggamma1](http://flybase.bio.indiana.edu/.bin/fbidq.html?Ggamma1), [CG7597](http://flybase.bio.indiana.edu/.bin/fbidq.html?CG7597),[CG5466](http://flybase.bio.indiana.edu/.bin/fbidq.html?CG5466), [esg](http://flybase.bio.indiana.edu/.bin/fbidq.html?esg), [RSG7](http://flybase.bio.indiana.edu/.bin/fbidq.html?RSG7), [m4](http://flybase.bio.indiana.edu/.bin/fbidq.html?m4), [dom](http://flybase.bio.indiana.edu/.bin/fbidq.html?dom), [Dh31](http://flybase.bio.indiana.edu/.bin/fbidq.html?Dh31), [klar](http://flybase.bio.indiana.edu/.bin/fbidq.html?klar), [shi](http://flybase.bio.indiana.edu/.bin/fbidq.html?shi), [Spred](http://flybase.bio.indiana.edu/.bin/fbidq.html?Spred), [siz](http://flybase.bio.indiana.edu/.bin/fbidq.html?siz), [B-H2](http://flybase.bio.indiana.edu/.bin/fbidq.html?B-H2), [tyn](http://flybase.bio.indiana.edu/.bin/fbidq.html?tyn), [CG3822](http://flybase.bio.indiana.edu/.bin/fbidq.html?CG3822), [CadN](http://flybase.bio.indiana.edu/.bin/fbidq.html?CadN), [CG34139](http://flybase.bio.indiana.edu/.bin/fbidq.html?CG34139), [Mfap1](http://flybase.bio.indiana.edu/.bin/fbidq.html?Mfap1), [CG13995](http://flybase.bio.indiana.edu/.bin/fbidq.html?CG13995), [E(bx)](http://flybase.bio.indiana.edu/.bin/fbidq.html?E(bx)), [CG12728](http://flybase.bio.indiana.edu/.bin/fbidq.html?CG12728), [Dms](http://flybase.bio.indiana.edu/.bin/fbidq.html?Dms), [Rbp2](http://flybase.bio.indiana.edu/.bin/fbidq.html?Rbp2), [Snap](http://flybase.bio.indiana.edu/.bin/fbidq.html?Snap), [trn](http://flybase.bio.indiana.edu/.bin/fbidq.html?trn), [Pitslre](http://flybase.bio.indiana.edu/.bin/fbidq.html?Pitslre), [CG3085](http://flybase.bio.indiana.edu/.bin/fbidq.html?CG3085), [pburs](http://flybase.bio.indiana.edu/.bin/fbidq.html?pburs), [cos](http://flybase.bio.indiana.edu/.bin/fbidq.html?cos) |
| [compound eye development](http://amigo.geneontology.org/cgi-bin/amigo/go.cgi?view=details&query=GO:0048749) | 90 of 1594 genes, 5.6% | 280 of 7634 genes, 3.7% | 0.00110 | 0.00% | 0.00 | [danr](http://flybase.bio.indiana.edu/.bin/fbidq.html?danr), [skd](http://flybase.bio.indiana.edu/.bin/fbidq.html?skd), [rg](http://flybase.bio.indiana.edu/.bin/fbidq.html?rg), [Pten](http://flybase.bio.indiana.edu/.bin/fbidq.html?Pten), [caup](http://flybase.bio.indiana.edu/.bin/fbidq.html?caup), [tll](http://flybase.bio.indiana.edu/.bin/fbidq.html?tll), [vimar](http://flybase.bio.indiana.edu/.bin/fbidq.html?vimar), [ato](http://flybase.bio.indiana.edu/.bin/fbidq.html?ato), [rn](http://flybase.bio.indiana.edu/.bin/fbidq.html?rn), [Dip3](http://flybase.bio.indiana.edu/.bin/fbidq.html?Dip3), [SP2353](http://flybase.bio.indiana.edu/.bin/fbidq.html?SP2353), [Cbl](http://flybase.bio.indiana.edu/.bin/fbidq.html?Cbl), [br](http://flybase.bio.indiana.edu/.bin/fbidq.html?br), [sns](http://flybase.bio.indiana.edu/.bin/fbidq.html?sns), [os](http://flybase.bio.indiana.edu/.bin/fbidq.html?os), [bi](http://flybase.bio.indiana.edu/.bin/fbidq.html?bi), [chn](http://flybase.bio.indiana.edu/.bin/fbidq.html?chn), [ds](http://flybase.bio.indiana.edu/.bin/fbidq.html?ds), [lola](http://flybase.bio.indiana.edu/.bin/fbidq.html?lola), [Alk](http://flybase.bio.indiana.edu/.bin/fbidq.html?Alk), [Vang](http://flybase.bio.indiana.edu/.bin/fbidq.html?Vang), [Nrk](http://flybase.bio.indiana.edu/.bin/fbidq.html?Nrk), [sano](http://flybase.bio.indiana.edu/.bin/fbidq.html?sano), [Galpha49B](http://flybase.bio.indiana.edu/.bin/fbidq.html?Galpha49B), [jumu](http://flybase.bio.indiana.edu/.bin/fbidq.html?jumu), [Lar](http://flybase.bio.indiana.edu/.bin/fbidq.html?Lar), [scrib](http://flybase.bio.indiana.edu/.bin/fbidq.html?scrib), [fred](http://flybase.bio.indiana.edu/.bin/fbidq.html?fred), [ush](http://flybase.bio.indiana.edu/.bin/fbidq.html?ush), [Gap1](http://flybase.bio.indiana.edu/.bin/fbidq.html?Gap1), [HLHmdelta](http://flybase.bio.indiana.edu/.bin/fbidq.html?HLHmdelta), [jeb](http://flybase.bio.indiana.edu/.bin/fbidq.html?jeb), [B-H1](http://flybase.bio.indiana.edu/.bin/fbidq.html?B-H1), [alph](http://flybase.bio.indiana.edu/.bin/fbidq.html?alph), [Hs3st-B](http://flybase.bio.indiana.edu/.bin/fbidq.html?Hs3st-B), [stan](http://flybase.bio.indiana.edu/.bin/fbidq.html?stan), [Sos](http://flybase.bio.indiana.edu/.bin/fbidq.html?Sos), [CG34400](http://flybase.bio.indiana.edu/.bin/fbidq.html?CG34400), [phyl](http://flybase.bio.indiana.edu/.bin/fbidq.html?phyl), [ena](http://flybase.bio.indiana.edu/.bin/fbidq.html?ena), [hth](http://flybase.bio.indiana.edu/.bin/fbidq.html?hth), [cindr](http://flybase.bio.indiana.edu/.bin/fbidq.html?cindr),[vg](http://flybase.bio.indiana.edu/.bin/fbidq.html?vg), [dsh](http://flybase.bio.indiana.edu/.bin/fbidq.html?dsh), [eya](http://flybase.bio.indiana.edu/.bin/fbidq.html?eya), [14-3-3zeta](http://flybase.bio.indiana.edu/.bin/fbidq.html?14-3-3zeta), [fz](http://flybase.bio.indiana.edu/.bin/fbidq.html?fz), [futsch](http://flybase.bio.indiana.edu/.bin/fbidq.html?futsch), [ft](http://flybase.bio.indiana.edu/.bin/fbidq.html?ft), [sno](http://flybase.bio.indiana.edu/.bin/fbidq.html?sno), [ara](http://flybase.bio.indiana.edu/.bin/fbidq.html?ara), [sna](http://flybase.bio.indiana.edu/.bin/fbidq.html?sna), [mam](http://flybase.bio.indiana.edu/.bin/fbidq.html?mam), [tsh](http://flybase.bio.indiana.edu/.bin/fbidq.html?tsh), [Syn2](http://flybase.bio.indiana.edu/.bin/fbidq.html?Syn2), [HLHm5](http://flybase.bio.indiana.edu/.bin/fbidq.html?HLHm5), [neur](http://flybase.bio.indiana.edu/.bin/fbidq.html?neur), [didum](http://flybase.bio.indiana.edu/.bin/fbidq.html?didum), [Lis-1](http://flybase.bio.indiana.edu/.bin/fbidq.html?Lis-1), [dac](http://flybase.bio.indiana.edu/.bin/fbidq.html?dac), [nmo](http://flybase.bio.indiana.edu/.bin/fbidq.html?nmo), [eys](http://flybase.bio.indiana.edu/.bin/fbidq.html?eys), [oc](http://flybase.bio.indiana.edu/.bin/fbidq.html?oc), [Mob2](http://flybase.bio.indiana.edu/.bin/fbidq.html?Mob2), [pros](http://flybase.bio.indiana.edu/.bin/fbidq.html?pros), [ey](http://flybase.bio.indiana.edu/.bin/fbidq.html?ey), [ssh](http://flybase.bio.indiana.edu/.bin/fbidq.html?ssh), [klar](http://flybase.bio.indiana.edu/.bin/fbidq.html?klar), [shi](http://flybase.bio.indiana.edu/.bin/fbidq.html?shi), [rst](http://flybase.bio.indiana.edu/.bin/fbidq.html?rst), [Wnt4](http://flybase.bio.indiana.edu/.bin/fbidq.html?Wnt4), [B-H2](http://flybase.bio.indiana.edu/.bin/fbidq.html?B-H2), [grh](http://flybase.bio.indiana.edu/.bin/fbidq.html?grh), [toy](http://flybase.bio.indiana.edu/.bin/fbidq.html?toy), [wg](http://flybase.bio.indiana.edu/.bin/fbidq.html?wg), [Apc](http://flybase.bio.indiana.edu/.bin/fbidq.html?Apc), [CadN](http://flybase.bio.indiana.edu/.bin/fbidq.html?CadN), [dan](http://flybase.bio.indiana.edu/.bin/fbidq.html?dan), [sd](http://flybase.bio.indiana.edu/.bin/fbidq.html?sd), [bchs](http://flybase.bio.indiana.edu/.bin/fbidq.html?bchs), [pk](http://flybase.bio.indiana.edu/.bin/fbidq.html?pk), [klg](http://flybase.bio.indiana.edu/.bin/fbidq.html?klg), [rl](http://flybase.bio.indiana.edu/.bin/fbidq.html?rl), [Snap](http://flybase.bio.indiana.edu/.bin/fbidq.html?Snap), [cdi](http://flybase.bio.indiana.edu/.bin/fbidq.html?cdi), [klu](http://flybase.bio.indiana.edu/.bin/fbidq.html?klu), [wls](http://flybase.bio.indiana.edu/.bin/fbidq.html?wls), [HLHm7](http://flybase.bio.indiana.edu/.bin/fbidq.html?HLHm7),[HLHmgamma](http://flybase.bio.indiana.edu/.bin/fbidq.html?HLHmgamma), [unk](http://flybase.bio.indiana.edu/.bin/fbidq.html?unk) |
| [cell-cell adhesion](http://amigo.geneontology.org/cgi-bin/amigo/go.cgi?view=details&query=GO:0016337) | 33 of 1594 genes, 2.1% | 72 of 7634 genes, 0.9% | 0.00112 | 0.00% | 0.00 | [Fas2](http://flybase.bio.indiana.edu/.bin/fbidq.html?Fas2), [fz](http://flybase.bio.indiana.edu/.bin/fbidq.html?fz), [robo](http://flybase.bio.indiana.edu/.bin/fbidq.html?robo), [lea](http://flybase.bio.indiana.edu/.bin/fbidq.html?lea), [beat-VI](http://flybase.bio.indiana.edu/.bin/fbidq.html?beat-VI), [ft](http://flybase.bio.indiana.edu/.bin/fbidq.html?ft), [Dscam3](http://flybase.bio.indiana.edu/.bin/fbidq.html?Dscam3), [Cad96Ca](http://flybase.bio.indiana.edu/.bin/fbidq.html?Cad96Ca), [CG42256](http://flybase.bio.indiana.edu/.bin/fbidq.html?CG42256), [betaInt-nu](http://flybase.bio.indiana.edu/.bin/fbidq.html?betaInt-nu), [beat-IIa](http://flybase.bio.indiana.edu/.bin/fbidq.html?beat-IIa), [beat-Vc](http://flybase.bio.indiana.edu/.bin/fbidq.html?beat-Vc), [mew](http://flybase.bio.indiana.edu/.bin/fbidq.html?mew), [beat-IIIc](http://flybase.bio.indiana.edu/.bin/fbidq.html?beat-IIIc), [sns](http://flybase.bio.indiana.edu/.bin/fbidq.html?sns), [ed](http://flybase.bio.indiana.edu/.bin/fbidq.html?ed), [baz](http://flybase.bio.indiana.edu/.bin/fbidq.html?baz), [ds](http://flybase.bio.indiana.edu/.bin/fbidq.html?ds), [rst](http://flybase.bio.indiana.edu/.bin/fbidq.html?rst), [p130CAS](http://flybase.bio.indiana.edu/.bin/fbidq.html?p130CAS), [Apc](http://flybase.bio.indiana.edu/.bin/fbidq.html?Apc), [CadN](http://flybase.bio.indiana.edu/.bin/fbidq.html?CadN), [beat-Vb](http://flybase.bio.indiana.edu/.bin/fbidq.html?beat-Vb), [klg](http://flybase.bio.indiana.edu/.bin/fbidq.html?klg), [beat-Ic](http://flybase.bio.indiana.edu/.bin/fbidq.html?beat-Ic), [Nrg](http://flybase.bio.indiana.edu/.bin/fbidq.html?Nrg), [beat-IV](http://flybase.bio.indiana.edu/.bin/fbidq.html?beat-IV), [beat-IIb](http://flybase.bio.indiana.edu/.bin/fbidq.html?beat-IIb), [beat-Ib](http://flybase.bio.indiana.edu/.bin/fbidq.html?beat-Ib), [glec](http://flybase.bio.indiana.edu/.bin/fbidq.html?glec), [Cad74A](http://flybase.bio.indiana.edu/.bin/fbidq.html?Cad74A), [pygo](http://flybase.bio.indiana.edu/.bin/fbidq.html?pygo), [stan](http://flybase.bio.indiana.edu/.bin/fbidq.html?stan) |
| [positive regulation of biosynthetic process](http://amigo.geneontology.org/cgi-bin/amigo/go.cgi?view=details&query=GO:0009891) | 75 of 1594 genes, 4.7% | 222 of 7634 genes, 2.9% | 0.00118 | 0.00% | 0.00 | [acj6](http://flybase.bio.indiana.edu/.bin/fbidq.html?acj6), [wor](http://flybase.bio.indiana.edu/.bin/fbidq.html?wor), [vvl](http://flybase.bio.indiana.edu/.bin/fbidq.html?vvl), [caup](http://flybase.bio.indiana.edu/.bin/fbidq.html?caup), [tll](http://flybase.bio.indiana.edu/.bin/fbidq.html?tll), [Camta](http://flybase.bio.indiana.edu/.bin/fbidq.html?Camta), [ato](http://flybase.bio.indiana.edu/.bin/fbidq.html?ato), [tna](http://flybase.bio.indiana.edu/.bin/fbidq.html?tna), [Eip93F](http://flybase.bio.indiana.edu/.bin/fbidq.html?Eip93F), [JIL-1](http://flybase.bio.indiana.edu/.bin/fbidq.html?JIL-1), [ap](http://flybase.bio.indiana.edu/.bin/fbidq.html?ap), [Iswi](http://flybase.bio.indiana.edu/.bin/fbidq.html?Iswi), [Spt6](http://flybase.bio.indiana.edu/.bin/fbidq.html?Spt6), [gcm](http://flybase.bio.indiana.edu/.bin/fbidq.html?gcm), [Dip3](http://flybase.bio.indiana.edu/.bin/fbidq.html?Dip3), [Nf1](http://flybase.bio.indiana.edu/.bin/fbidq.html?Nf1), [ase](http://flybase.bio.indiana.edu/.bin/fbidq.html?ase), [br](http://flybase.bio.indiana.edu/.bin/fbidq.html?br), [ovo](http://flybase.bio.indiana.edu/.bin/fbidq.html?ovo), [Pdp1](http://flybase.bio.indiana.edu/.bin/fbidq.html?Pdp1), [chn](http://flybase.bio.indiana.edu/.bin/fbidq.html?chn), [Hcf](http://flybase.bio.indiana.edu/.bin/fbidq.html?Hcf), [lola](http://flybase.bio.indiana.edu/.bin/fbidq.html?lola), [NC2alpha](http://flybase.bio.indiana.edu/.bin/fbidq.html?NC2alpha), [Taf4](http://flybase.bio.indiana.edu/.bin/fbidq.html?Taf4), [scrib](http://flybase.bio.indiana.edu/.bin/fbidq.html?scrib), [HGTX](http://flybase.bio.indiana.edu/.bin/fbidq.html?HGTX), [fd59A](http://flybase.bio.indiana.edu/.bin/fbidq.html?fd59A), [btd](http://flybase.bio.indiana.edu/.bin/fbidq.html?btd), [run](http://flybase.bio.indiana.edu/.bin/fbidq.html?run), [Antp](http://flybase.bio.indiana.edu/.bin/fbidq.html?Antp), [ATbp](http://flybase.bio.indiana.edu/.bin/fbidq.html?ATbp), [tara](http://flybase.bio.indiana.edu/.bin/fbidq.html?tara), [Hel89B](http://flybase.bio.indiana.edu/.bin/fbidq.html?Hel89B), [stau](http://flybase.bio.indiana.edu/.bin/fbidq.html?stau), [hth](http://flybase.bio.indiana.edu/.bin/fbidq.html?hth), [vg](http://flybase.bio.indiana.edu/.bin/fbidq.html?vg), [eya](http://flybase.bio.indiana.edu/.bin/fbidq.html?eya), [brm](http://flybase.bio.indiana.edu/.bin/fbidq.html?brm), [onecut](http://flybase.bio.indiana.edu/.bin/fbidq.html?onecut), [sno](http://flybase.bio.indiana.edu/.bin/fbidq.html?sno), [chm](http://flybase.bio.indiana.edu/.bin/fbidq.html?chm), [ara](http://flybase.bio.indiana.edu/.bin/fbidq.html?ara),[sna](http://flybase.bio.indiana.edu/.bin/fbidq.html?sna), [mam](http://flybase.bio.indiana.edu/.bin/fbidq.html?mam), [Taf1](http://flybase.bio.indiana.edu/.bin/fbidq.html?Taf1), [pan](http://flybase.bio.indiana.edu/.bin/fbidq.html?pan), [disco](http://flybase.bio.indiana.edu/.bin/fbidq.html?disco), [esg](http://flybase.bio.indiana.edu/.bin/fbidq.html?esg), [e(y)3](http://flybase.bio.indiana.edu/.bin/fbidq.html?e(y)3), [Brf](http://flybase.bio.indiana.edu/.bin/fbidq.html?Brf), [oc](http://flybase.bio.indiana.edu/.bin/fbidq.html?oc), [tup](http://flybase.bio.indiana.edu/.bin/fbidq.html?tup), [spt4](http://flybase.bio.indiana.edu/.bin/fbidq.html?spt4), [ey](http://flybase.bio.indiana.edu/.bin/fbidq.html?ey), [mle](http://flybase.bio.indiana.edu/.bin/fbidq.html?mle), [l(1)sc](http://flybase.bio.indiana.edu/.bin/fbidq.html?l(1)sc), [Bgb](http://flybase.bio.indiana.edu/.bin/fbidq.html?Bgb), [grh](http://flybase.bio.indiana.edu/.bin/fbidq.html?grh), [toy](http://flybase.bio.indiana.edu/.bin/fbidq.html?toy), [wg](http://flybase.bio.indiana.edu/.bin/fbidq.html?wg), [fkh](http://flybase.bio.indiana.edu/.bin/fbidq.html?fkh), [sim](http://flybase.bio.indiana.edu/.bin/fbidq.html?sim), [sd](http://flybase.bio.indiana.edu/.bin/fbidq.html?sd), [trx](http://flybase.bio.indiana.edu/.bin/fbidq.html?trx), [retn](http://flybase.bio.indiana.edu/.bin/fbidq.html?retn), [dsx](http://flybase.bio.indiana.edu/.bin/fbidq.html?dsx), [tyf](http://flybase.bio.indiana.edu/.bin/fbidq.html?tyf), [vfl](http://flybase.bio.indiana.edu/.bin/fbidq.html?vfl), [CG11347](http://flybase.bio.indiana.edu/.bin/fbidq.html?CG11347), [dalao](http://flybase.bio.indiana.edu/.bin/fbidq.html?dalao), [pygo](http://flybase.bio.indiana.edu/.bin/fbidq.html?pygo), [z](http://flybase.bio.indiana.edu/.bin/fbidq.html?z), [Lim1](http://flybase.bio.indiana.edu/.bin/fbidq.html?Lim1), [Atf-2](http://flybase.bio.indiana.edu/.bin/fbidq.html?Atf-2) |
| [positive regulation of cellular biosynthetic process](http://amigo.geneontology.org/cgi-bin/amigo/go.cgi?view=details&query=GO:0031328) | 75 of 1594 genes, 4.7% | 222 of 7634 genes, 2.9% | 0.00118 | 0.00% | 0.00 | [acj6](http://flybase.bio.indiana.edu/.bin/fbidq.html?acj6), [wor](http://flybase.bio.indiana.edu/.bin/fbidq.html?wor), [vvl](http://flybase.bio.indiana.edu/.bin/fbidq.html?vvl), [caup](http://flybase.bio.indiana.edu/.bin/fbidq.html?caup), [tll](http://flybase.bio.indiana.edu/.bin/fbidq.html?tll), [Camta](http://flybase.bio.indiana.edu/.bin/fbidq.html?Camta), [ato](http://flybase.bio.indiana.edu/.bin/fbidq.html?ato), [tna](http://flybase.bio.indiana.edu/.bin/fbidq.html?tna), [Eip93F](http://flybase.bio.indiana.edu/.bin/fbidq.html?Eip93F), [JIL-1](http://flybase.bio.indiana.edu/.bin/fbidq.html?JIL-1), [ap](http://flybase.bio.indiana.edu/.bin/fbidq.html?ap), [Iswi](http://flybase.bio.indiana.edu/.bin/fbidq.html?Iswi), [Spt6](http://flybase.bio.indiana.edu/.bin/fbidq.html?Spt6), [gcm](http://flybase.bio.indiana.edu/.bin/fbidq.html?gcm), [Dip3](http://flybase.bio.indiana.edu/.bin/fbidq.html?Dip3), [Nf1](http://flybase.bio.indiana.edu/.bin/fbidq.html?Nf1), [ase](http://flybase.bio.indiana.edu/.bin/fbidq.html?ase), [br](http://flybase.bio.indiana.edu/.bin/fbidq.html?br), [ovo](http://flybase.bio.indiana.edu/.bin/fbidq.html?ovo), [Pdp1](http://flybase.bio.indiana.edu/.bin/fbidq.html?Pdp1), [chn](http://flybase.bio.indiana.edu/.bin/fbidq.html?chn), [Hcf](http://flybase.bio.indiana.edu/.bin/fbidq.html?Hcf), [lola](http://flybase.bio.indiana.edu/.bin/fbidq.html?lola), [NC2alpha](http://flybase.bio.indiana.edu/.bin/fbidq.html?NC2alpha), [Taf4](http://flybase.bio.indiana.edu/.bin/fbidq.html?Taf4), [scrib](http://flybase.bio.indiana.edu/.bin/fbidq.html?scrib), [HGTX](http://flybase.bio.indiana.edu/.bin/fbidq.html?HGTX), [fd59A](http://flybase.bio.indiana.edu/.bin/fbidq.html?fd59A), [btd](http://flybase.bio.indiana.edu/.bin/fbidq.html?btd), [run](http://flybase.bio.indiana.edu/.bin/fbidq.html?run), [Antp](http://flybase.bio.indiana.edu/.bin/fbidq.html?Antp), [ATbp](http://flybase.bio.indiana.edu/.bin/fbidq.html?ATbp), [tara](http://flybase.bio.indiana.edu/.bin/fbidq.html?tara), [Hel89B](http://flybase.bio.indiana.edu/.bin/fbidq.html?Hel89B), [stau](http://flybase.bio.indiana.edu/.bin/fbidq.html?stau), [hth](http://flybase.bio.indiana.edu/.bin/fbidq.html?hth), [vg](http://flybase.bio.indiana.edu/.bin/fbidq.html?vg), [eya](http://flybase.bio.indiana.edu/.bin/fbidq.html?eya), [brm](http://flybase.bio.indiana.edu/.bin/fbidq.html?brm), [onecut](http://flybase.bio.indiana.edu/.bin/fbidq.html?onecut), [sno](http://flybase.bio.indiana.edu/.bin/fbidq.html?sno), [chm](http://flybase.bio.indiana.edu/.bin/fbidq.html?chm), [ara](http://flybase.bio.indiana.edu/.bin/fbidq.html?ara),[sna](http://flybase.bio.indiana.edu/.bin/fbidq.html?sna), [mam](http://flybase.bio.indiana.edu/.bin/fbidq.html?mam), [Taf1](http://flybase.bio.indiana.edu/.bin/fbidq.html?Taf1), [pan](http://flybase.bio.indiana.edu/.bin/fbidq.html?pan), [disco](http://flybase.bio.indiana.edu/.bin/fbidq.html?disco), [esg](http://flybase.bio.indiana.edu/.bin/fbidq.html?esg), [e(y)3](http://flybase.bio.indiana.edu/.bin/fbidq.html?e(y)3), [Brf](http://flybase.bio.indiana.edu/.bin/fbidq.html?Brf), [oc](http://flybase.bio.indiana.edu/.bin/fbidq.html?oc), [tup](http://flybase.bio.indiana.edu/.bin/fbidq.html?tup), [spt4](http://flybase.bio.indiana.edu/.bin/fbidq.html?spt4), [ey](http://flybase.bio.indiana.edu/.bin/fbidq.html?ey), [mle](http://flybase.bio.indiana.edu/.bin/fbidq.html?mle), [l(1)sc](http://flybase.bio.indiana.edu/.bin/fbidq.html?l(1)sc), [Bgb](http://flybase.bio.indiana.edu/.bin/fbidq.html?Bgb), [grh](http://flybase.bio.indiana.edu/.bin/fbidq.html?grh), [toy](http://flybase.bio.indiana.edu/.bin/fbidq.html?toy), [wg](http://flybase.bio.indiana.edu/.bin/fbidq.html?wg), [fkh](http://flybase.bio.indiana.edu/.bin/fbidq.html?fkh), [sim](http://flybase.bio.indiana.edu/.bin/fbidq.html?sim), [sd](http://flybase.bio.indiana.edu/.bin/fbidq.html?sd), [trx](http://flybase.bio.indiana.edu/.bin/fbidq.html?trx), [retn](http://flybase.bio.indiana.edu/.bin/fbidq.html?retn), [dsx](http://flybase.bio.indiana.edu/.bin/fbidq.html?dsx), [tyf](http://flybase.bio.indiana.edu/.bin/fbidq.html?tyf), [vfl](http://flybase.bio.indiana.edu/.bin/fbidq.html?vfl), [CG11347](http://flybase.bio.indiana.edu/.bin/fbidq.html?CG11347), [dalao](http://flybase.bio.indiana.edu/.bin/fbidq.html?dalao), [pygo](http://flybase.bio.indiana.edu/.bin/fbidq.html?pygo), [z](http://flybase.bio.indiana.edu/.bin/fbidq.html?z), [Lim1](http://flybase.bio.indiana.edu/.bin/fbidq.html?Lim1), [Atf-2](http://flybase.bio.indiana.edu/.bin/fbidq.html?Atf-2) |
| [multi-organism behavior](http://amigo.geneontology.org/cgi-bin/amigo/go.cgi?view=details&query=GO:0051705) | 38 of 1594 genes, 2.4% | 89 of 7634 genes, 1.2% | 0.00146 | 0.00% | 0.00 | [Sh](http://flybase.bio.indiana.edu/.bin/fbidq.html?Sh), [Ilp2](http://flybase.bio.indiana.edu/.bin/fbidq.html?Ilp2), [npf](http://flybase.bio.indiana.edu/.bin/fbidq.html?npf), [ato](http://flybase.bio.indiana.edu/.bin/fbidq.html?ato), [Spn](http://flybase.bio.indiana.edu/.bin/fbidq.html?Spn), [para](http://flybase.bio.indiana.edu/.bin/fbidq.html?para), [neur](http://flybase.bio.indiana.edu/.bin/fbidq.html?neur), [CG14478](http://flybase.bio.indiana.edu/.bin/fbidq.html?CG14478), [Bx](http://flybase.bio.indiana.edu/.bin/fbidq.html?Bx), [l(3)L1231](http://flybase.bio.indiana.edu/.bin/fbidq.html?l(3)L1231), [pros](http://flybase.bio.indiana.edu/.bin/fbidq.html?pros), [Tbh](http://flybase.bio.indiana.edu/.bin/fbidq.html?Tbh), [ed](http://flybase.bio.indiana.edu/.bin/fbidq.html?ed), [mle](http://flybase.bio.indiana.edu/.bin/fbidq.html?mle), [Oamb](http://flybase.bio.indiana.edu/.bin/fbidq.html?Oamb), [orb2](http://flybase.bio.indiana.edu/.bin/fbidq.html?orb2), [nonA](http://flybase.bio.indiana.edu/.bin/fbidq.html?nonA), [CG10251](http://flybase.bio.indiana.edu/.bin/fbidq.html?CG10251), [CASK](http://flybase.bio.indiana.edu/.bin/fbidq.html?CASK), [tipE](http://flybase.bio.indiana.edu/.bin/fbidq.html?tipE), [lola](http://flybase.bio.indiana.edu/.bin/fbidq.html?lola), [siz](http://flybase.bio.indiana.edu/.bin/fbidq.html?siz), [Poxn](http://flybase.bio.indiana.edu/.bin/fbidq.html?Poxn), [qtc](http://flybase.bio.indiana.edu/.bin/fbidq.html?qtc), [Pde1c](http://flybase.bio.indiana.edu/.bin/fbidq.html?Pde1c), [CaMKII](http://flybase.bio.indiana.edu/.bin/fbidq.html?CaMKII), [fne](http://flybase.bio.indiana.edu/.bin/fbidq.html?fne), [Ilp3](http://flybase.bio.indiana.edu/.bin/fbidq.html?Ilp3), [5-HT7](http://flybase.bio.indiana.edu/.bin/fbidq.html?5-HT7), [retn](http://flybase.bio.indiana.edu/.bin/fbidq.html?retn), [Btk29A](http://flybase.bio.indiana.edu/.bin/fbidq.html?Btk29A), [rut](http://flybase.bio.indiana.edu/.bin/fbidq.html?rut), [dsx](http://flybase.bio.indiana.edu/.bin/fbidq.html?dsx), [Nrg](http://flybase.bio.indiana.edu/.bin/fbidq.html?Nrg), [Ilp5](http://flybase.bio.indiana.edu/.bin/fbidq.html?Ilp5), [Ddc](http://flybase.bio.indiana.edu/.bin/fbidq.html?Ddc), [SPR](http://flybase.bio.indiana.edu/.bin/fbidq.html?SPR), [aret](http://flybase.bio.indiana.edu/.bin/fbidq.html?aret) |
| [chemosensory behavior](http://amigo.geneontology.org/cgi-bin/amigo/go.cgi?view=details&query=GO:0007635) | 37 of 1594 genes, 2.3% | 86 of 7634 genes, 1.1% | 0.00163 | 0.00% | 0.00 | [Fas2](http://flybase.bio.indiana.edu/.bin/fbidq.html?Fas2), [Sh](http://flybase.bio.indiana.edu/.bin/fbidq.html?Sh), [14-3-3zeta](http://flybase.bio.indiana.edu/.bin/fbidq.html?14-3-3zeta), [futsch](http://flybase.bio.indiana.edu/.bin/fbidq.html?futsch), [NaCP60E](http://flybase.bio.indiana.edu/.bin/fbidq.html?NaCP60E), [acj6](http://flybase.bio.indiana.edu/.bin/fbidq.html?acj6), [CG14509](http://flybase.bio.indiana.edu/.bin/fbidq.html?CG14509), [rg](http://flybase.bio.indiana.edu/.bin/fbidq.html?rg), [betaInt-nu](http://flybase.bio.indiana.edu/.bin/fbidq.html?betaInt-nu), [Spn](http://flybase.bio.indiana.edu/.bin/fbidq.html?Spn), [pigeon](http://flybase.bio.indiana.edu/.bin/fbidq.html?pigeon), [sgg](http://flybase.bio.indiana.edu/.bin/fbidq.html?sgg), [smi35A](http://flybase.bio.indiana.edu/.bin/fbidq.html?smi35A), [mnb](http://flybase.bio.indiana.edu/.bin/fbidq.html?mnb), [Ggamma1](http://flybase.bio.indiana.edu/.bin/fbidq.html?Ggamma1), [Dll](http://flybase.bio.indiana.edu/.bin/fbidq.html?Dll), [Nmdar1](http://flybase.bio.indiana.edu/.bin/fbidq.html?Nmdar1), [neur](http://flybase.bio.indiana.edu/.bin/fbidq.html?neur), [esg](http://flybase.bio.indiana.edu/.bin/fbidq.html?esg), [Nf1](http://flybase.bio.indiana.edu/.bin/fbidq.html?Nf1), [Pka-R1](http://flybase.bio.indiana.edu/.bin/fbidq.html?Pka-R1), [Mob2](http://flybase.bio.indiana.edu/.bin/fbidq.html?Mob2), [CG10251](http://flybase.bio.indiana.edu/.bin/fbidq.html?CG10251), [shi](http://flybase.bio.indiana.edu/.bin/fbidq.html?shi), [Gad1](http://flybase.bio.indiana.edu/.bin/fbidq.html?Gad1), [Alk](http://flybase.bio.indiana.edu/.bin/fbidq.html?Alk), [rogdi](http://flybase.bio.indiana.edu/.bin/fbidq.html?rogdi), [tau](http://flybase.bio.indiana.edu/.bin/fbidq.html?tau), [CG42629](http://flybase.bio.indiana.edu/.bin/fbidq.html?CG42629), [bin3](http://flybase.bio.indiana.edu/.bin/fbidq.html?bin3), [scrib](http://flybase.bio.indiana.edu/.bin/fbidq.html?scrib), [klg](http://flybase.bio.indiana.edu/.bin/fbidq.html?klg), [rut](http://flybase.bio.indiana.edu/.bin/fbidq.html?rut), [PQBP-1](http://flybase.bio.indiana.edu/.bin/fbidq.html?PQBP-1),[Sip1](http://flybase.bio.indiana.edu/.bin/fbidq.html?Sip1), [Rdl](http://flybase.bio.indiana.edu/.bin/fbidq.html?Rdl), [GABA-B-R2](http://flybase.bio.indiana.edu/.bin/fbidq.html?GABA-B-R2) |
| [locomotion](http://amigo.geneontology.org/cgi-bin/amigo/go.cgi?view=details&query=GO:0040011) | 120 of 1594 genes, 7.5% | 404 of 7634 genes, 5.3% | 0.00168 | 0.00% | 0.00 | [fz2](http://flybase.bio.indiana.edu/.bin/fbidq.html?fz2), [jar](http://flybase.bio.indiana.edu/.bin/fbidq.html?jar), [numb](http://flybase.bio.indiana.edu/.bin/fbidq.html?numb), [acj6](http://flybase.bio.indiana.edu/.bin/fbidq.html?acj6), [vvl](http://flybase.bio.indiana.edu/.bin/fbidq.html?vvl), [jing](http://flybase.bio.indiana.edu/.bin/fbidq.html?jing), [dock](http://flybase.bio.indiana.edu/.bin/fbidq.html?dock), [katanin-60](http://flybase.bio.indiana.edu/.bin/fbidq.html?katanin-60), [Pten](http://flybase.bio.indiana.edu/.bin/fbidq.html?Pten), [Ptp69D](http://flybase.bio.indiana.edu/.bin/fbidq.html?Ptp69D), [hts](http://flybase.bio.indiana.edu/.bin/fbidq.html?hts), [Nmdar1](http://flybase.bio.indiana.edu/.bin/fbidq.html?Nmdar1), [mp](http://flybase.bio.indiana.edu/.bin/fbidq.html?mp), [ap](http://flybase.bio.indiana.edu/.bin/fbidq.html?ap), [Dg](http://flybase.bio.indiana.edu/.bin/fbidq.html?Dg), [seq](http://flybase.bio.indiana.edu/.bin/fbidq.html?seq), [gcm](http://flybase.bio.indiana.edu/.bin/fbidq.html?gcm), [Trim9](http://flybase.bio.indiana.edu/.bin/fbidq.html?Trim9), [Evi5](http://flybase.bio.indiana.edu/.bin/fbidq.html?Evi5), [phl](http://flybase.bio.indiana.edu/.bin/fbidq.html?phl), [Cbl](http://flybase.bio.indiana.edu/.bin/fbidq.html?Cbl), [Gycalpha99B](http://flybase.bio.indiana.edu/.bin/fbidq.html?Gycalpha99B), [robo3](http://flybase.bio.indiana.edu/.bin/fbidq.html?robo3), [os](http://flybase.bio.indiana.edu/.bin/fbidq.html?os), [tok](http://flybase.bio.indiana.edu/.bin/fbidq.html?tok), [nonA](http://flybase.bio.indiana.edu/.bin/fbidq.html?nonA), [Ptp99A](http://flybase.bio.indiana.edu/.bin/fbidq.html?Ptp99A), [lola](http://flybase.bio.indiana.edu/.bin/fbidq.html?lola), [unc-104](http://flybase.bio.indiana.edu/.bin/fbidq.html?unc-104), [chif](http://flybase.bio.indiana.edu/.bin/fbidq.html?chif), [NetB](http://flybase.bio.indiana.edu/.bin/fbidq.html?NetB), [Alk](http://flybase.bio.indiana.edu/.bin/fbidq.html?Alk), [Vang](http://flybase.bio.indiana.edu/.bin/fbidq.html?Vang), [rdgC](http://flybase.bio.indiana.edu/.bin/fbidq.html?rdgC), [Nrk](http://flybase.bio.indiana.edu/.bin/fbidq.html?Nrk), [mod(mdg4)](http://flybase.bio.indiana.edu/.bin/fbidq.html?mod(mdg4)), [disp](http://flybase.bio.indiana.edu/.bin/fbidq.html?disp), [mip120](http://flybase.bio.indiana.edu/.bin/fbidq.html?mip120),[Galpha49B](http://flybase.bio.indiana.edu/.bin/fbidq.html?Galpha49B), [Smox](http://flybase.bio.indiana.edu/.bin/fbidq.html?Smox), [Lar](http://flybase.bio.indiana.edu/.bin/fbidq.html?Lar), [scrib](http://flybase.bio.indiana.edu/.bin/fbidq.html?scrib), [not](http://flybase.bio.indiana.edu/.bin/fbidq.html?not), [nvy](http://flybase.bio.indiana.edu/.bin/fbidq.html?nvy), [gogo](http://flybase.bio.indiana.edu/.bin/fbidq.html?gogo), [fd59A](http://flybase.bio.indiana.edu/.bin/fbidq.html?fd59A), [beat-Ic](http://flybase.bio.indiana.edu/.bin/fbidq.html?beat-Ic), [Ddc](http://flybase.bio.indiana.edu/.bin/fbidq.html?Ddc), [run](http://flybase.bio.indiana.edu/.bin/fbidq.html?run), [jeb](http://flybase.bio.indiana.edu/.bin/fbidq.html?jeb), [brat](http://flybase.bio.indiana.edu/.bin/fbidq.html?brat), [CG16778](http://flybase.bio.indiana.edu/.bin/fbidq.html?CG16778), [ko](http://flybase.bio.indiana.edu/.bin/fbidq.html?ko), [Hex-A](http://flybase.bio.indiana.edu/.bin/fbidq.html?Hex-A), [Sema-1a](http://flybase.bio.indiana.edu/.bin/fbidq.html?Sema-1a), [beat-Ib](http://flybase.bio.indiana.edu/.bin/fbidq.html?beat-Ib), [otk](http://flybase.bio.indiana.edu/.bin/fbidq.html?otk), [stan](http://flybase.bio.indiana.edu/.bin/fbidq.html?stan), [CG12424](http://flybase.bio.indiana.edu/.bin/fbidq.html?CG12424), [CG34400](http://flybase.bio.indiana.edu/.bin/fbidq.html?CG34400), [tutl](http://flybase.bio.indiana.edu/.bin/fbidq.html?tutl), [ena](http://flybase.bio.indiana.edu/.bin/fbidq.html?ena), [cindr](http://flybase.bio.indiana.edu/.bin/fbidq.html?cindr), [dsh](http://flybase.bio.indiana.edu/.bin/fbidq.html?dsh), [eya](http://flybase.bio.indiana.edu/.bin/fbidq.html?eya), [Nf-YC](http://flybase.bio.indiana.edu/.bin/fbidq.html?Nf-YC), [fz](http://flybase.bio.indiana.edu/.bin/fbidq.html?fz), [robo](http://flybase.bio.indiana.edu/.bin/fbidq.html?robo), [lea](http://flybase.bio.indiana.edu/.bin/fbidq.html?lea), [bsk](http://flybase.bio.indiana.edu/.bin/fbidq.html?bsk), [beat-Ia](http://flybase.bio.indiana.edu/.bin/fbidq.html?beat-Ia), [gukh](http://flybase.bio.indiana.edu/.bin/fbidq.html?gukh), [plexB](http://flybase.bio.indiana.edu/.bin/fbidq.html?plexB), [Wnt5](http://flybase.bio.indiana.edu/.bin/fbidq.html?Wnt5), [Klp64D](http://flybase.bio.indiana.edu/.bin/fbidq.html?Klp64D),[beat-IIa](http://flybase.bio.indiana.edu/.bin/fbidq.html?beat-IIa), [grn](http://flybase.bio.indiana.edu/.bin/fbidq.html?grn), [sm](http://flybase.bio.indiana.edu/.bin/fbidq.html?sm), [DopR](http://flybase.bio.indiana.edu/.bin/fbidq.html?DopR), [mew](http://flybase.bio.indiana.edu/.bin/fbidq.html?mew), [neur](http://flybase.bio.indiana.edu/.bin/fbidq.html?neur), [esg](http://flybase.bio.indiana.edu/.bin/fbidq.html?esg), [Lis-1](http://flybase.bio.indiana.edu/.bin/fbidq.html?Lis-1), [dac](http://flybase.bio.indiana.edu/.bin/fbidq.html?dac), [Cdk5](http://flybase.bio.indiana.edu/.bin/fbidq.html?Cdk5), [nmo](http://flybase.bio.indiana.edu/.bin/fbidq.html?nmo), [pros](http://flybase.bio.indiana.edu/.bin/fbidq.html?pros), [Tbh](http://flybase.bio.indiana.edu/.bin/fbidq.html?Tbh), [tup](http://flybase.bio.indiana.edu/.bin/fbidq.html?tup), [trio](http://flybase.bio.indiana.edu/.bin/fbidq.html?trio), [Sas-4](http://flybase.bio.indiana.edu/.bin/fbidq.html?Sas-4), [ey](http://flybase.bio.indiana.edu/.bin/fbidq.html?ey), [baz](http://flybase.bio.indiana.edu/.bin/fbidq.html?baz), [klar](http://flybase.bio.indiana.edu/.bin/fbidq.html?klar), [lap](http://flybase.bio.indiana.edu/.bin/fbidq.html?lap), [daw](http://flybase.bio.indiana.edu/.bin/fbidq.html?daw), [shi](http://flybase.bio.indiana.edu/.bin/fbidq.html?shi), [Wnt4](http://flybase.bio.indiana.edu/.bin/fbidq.html?Wnt4), [comm](http://flybase.bio.indiana.edu/.bin/fbidq.html?comm), [Cdk5alpha](http://flybase.bio.indiana.edu/.bin/fbidq.html?Cdk5alpha), [Apc](http://flybase.bio.indiana.edu/.bin/fbidq.html?Apc), [sim](http://flybase.bio.indiana.edu/.bin/fbidq.html?sim), [Lim3](http://flybase.bio.indiana.edu/.bin/fbidq.html?Lim3), [CadN](http://flybase.bio.indiana.edu/.bin/fbidq.html?CadN), [nerfin-1](http://flybase.bio.indiana.edu/.bin/fbidq.html?nerfin-1), [RN-tre](http://flybase.bio.indiana.edu/.bin/fbidq.html?RN-tre), [trx](http://flybase.bio.indiana.edu/.bin/fbidq.html?trx), [retn](http://flybase.bio.indiana.edu/.bin/fbidq.html?retn), [en](http://flybase.bio.indiana.edu/.bin/fbidq.html?en), [dsx](http://flybase.bio.indiana.edu/.bin/fbidq.html?dsx), [sfl](http://flybase.bio.indiana.edu/.bin/fbidq.html?sfl), [unc-5](http://flybase.bio.indiana.edu/.bin/fbidq.html?unc-5), [trn](http://flybase.bio.indiana.edu/.bin/fbidq.html?trn), [CG10107](http://flybase.bio.indiana.edu/.bin/fbidq.html?CG10107), [CG8155](http://flybase.bio.indiana.edu/.bin/fbidq.html?CG8155),[fend](http://flybase.bio.indiana.edu/.bin/fbidq.html?fend), [CG33960](http://flybase.bio.indiana.edu/.bin/fbidq.html?CG33960), [HLHm7](http://flybase.bio.indiana.edu/.bin/fbidq.html?HLHm7), [hiw](http://flybase.bio.indiana.edu/.bin/fbidq.html?hiw), [sr](http://flybase.bio.indiana.edu/.bin/fbidq.html?sr) |
| [negative regulation of transcription, DNA-dependent](http://amigo.geneontology.org/cgi-bin/amigo/go.cgi?view=details&query=GO:0045892) | 69 of 1594 genes, 4.3% | 201 of 7634 genes, 2.6% | 0.00174 | 0.00% | 0.00 | [jing](http://flybase.bio.indiana.edu/.bin/fbidq.html?jing), [rn](http://flybase.bio.indiana.edu/.bin/fbidq.html?rn), [Iswi](http://flybase.bio.indiana.edu/.bin/fbidq.html?Iswi), [JIL-1](http://flybase.bio.indiana.edu/.bin/fbidq.html?JIL-1), [seq](http://flybase.bio.indiana.edu/.bin/fbidq.html?seq), [Aef1](http://flybase.bio.indiana.edu/.bin/fbidq.html?Aef1), [ovo](http://flybase.bio.indiana.edu/.bin/fbidq.html?ovo), [Dsp1](http://flybase.bio.indiana.edu/.bin/fbidq.html?Dsp1), [bi](http://flybase.bio.indiana.edu/.bin/fbidq.html?bi), [chn](http://flybase.bio.indiana.edu/.bin/fbidq.html?chn), [Gsc](http://flybase.bio.indiana.edu/.bin/fbidq.html?Gsc), [Nelf-A](http://flybase.bio.indiana.edu/.bin/fbidq.html?Nelf-A), [Pcl](http://flybase.bio.indiana.edu/.bin/fbidq.html?Pcl), [simj](http://flybase.bio.indiana.edu/.bin/fbidq.html?simj), [Sin3A](http://flybase.bio.indiana.edu/.bin/fbidq.html?Sin3A), [NC2alpha](http://flybase.bio.indiana.edu/.bin/fbidq.html?NC2alpha), [mip120](http://flybase.bio.indiana.edu/.bin/fbidq.html?mip120), [Snoo](http://flybase.bio.indiana.edu/.bin/fbidq.html?Snoo), [jumu](http://flybase.bio.indiana.edu/.bin/fbidq.html?jumu), [HGTX](http://flybase.bio.indiana.edu/.bin/fbidq.html?HGTX), [ush](http://flybase.bio.indiana.edu/.bin/fbidq.html?ush), [Usp7](http://flybase.bio.indiana.edu/.bin/fbidq.html?Usp7), [fd59A](http://flybase.bio.indiana.edu/.bin/fbidq.html?fd59A), [nab](http://flybase.bio.indiana.edu/.bin/fbidq.html?nab), [btd](http://flybase.bio.indiana.edu/.bin/fbidq.html?btd), [fd68A](http://flybase.bio.indiana.edu/.bin/fbidq.html?fd68A), [HLHmdelta](http://flybase.bio.indiana.edu/.bin/fbidq.html?HLHmdelta), [run](http://flybase.bio.indiana.edu/.bin/fbidq.html?run), [B-H1](http://flybase.bio.indiana.edu/.bin/fbidq.html?B-H1), [FBX011](http://flybase.bio.indiana.edu/.bin/fbidq.html?FBX011), [egg](http://flybase.bio.indiana.edu/.bin/fbidq.html?egg), [l(3)neo38](http://flybase.bio.indiana.edu/.bin/fbidq.html?l(3)neo38), [fd102C](http://flybase.bio.indiana.edu/.bin/fbidq.html?fd102C), [Vsx2](http://flybase.bio.indiana.edu/.bin/fbidq.html?Vsx2), [ph-p](http://flybase.bio.indiana.edu/.bin/fbidq.html?ph-p), [Ubx](http://flybase.bio.indiana.edu/.bin/fbidq.html?Ubx), [CG7757](http://flybase.bio.indiana.edu/.bin/fbidq.html?CG7757),[chm](http://flybase.bio.indiana.edu/.bin/fbidq.html?chm), [sna](http://flybase.bio.indiana.edu/.bin/fbidq.html?sna), [Sfmbt](http://flybase.bio.indiana.edu/.bin/fbidq.html?Sfmbt), [Wnt5](http://flybase.bio.indiana.edu/.bin/fbidq.html?Wnt5), [pan](http://flybase.bio.indiana.edu/.bin/fbidq.html?pan), [MBD-like](http://flybase.bio.indiana.edu/.bin/fbidq.html?MBD-like), [tsh](http://flybase.bio.indiana.edu/.bin/fbidq.html?tsh), [HLHm5](http://flybase.bio.indiana.edu/.bin/fbidq.html?HLHm5), [esg](http://flybase.bio.indiana.edu/.bin/fbidq.html?esg), [e(y)3](http://flybase.bio.indiana.edu/.bin/fbidq.html?e(y)3), [oc](http://flybase.bio.indiana.edu/.bin/fbidq.html?oc), [pros](http://flybase.bio.indiana.edu/.bin/fbidq.html?pros), [Gug](http://flybase.bio.indiana.edu/.bin/fbidq.html?Gug), [pad](http://flybase.bio.indiana.edu/.bin/fbidq.html?pad), [Wnt4](http://flybase.bio.indiana.edu/.bin/fbidq.html?Wnt4), [B-H2](http://flybase.bio.indiana.edu/.bin/fbidq.html?B-H2), [wg](http://flybase.bio.indiana.edu/.bin/fbidq.html?wg), [Vsx1](http://flybase.bio.indiana.edu/.bin/fbidq.html?Vsx1), [fkh](http://flybase.bio.indiana.edu/.bin/fbidq.html?fkh), [gro](http://flybase.bio.indiana.edu/.bin/fbidq.html?gro), [crol](http://flybase.bio.indiana.edu/.bin/fbidq.html?crol), [Rpd3](http://flybase.bio.indiana.edu/.bin/fbidq.html?Rpd3), [retn](http://flybase.bio.indiana.edu/.bin/fbidq.html?retn), [fs(1)h](http://flybase.bio.indiana.edu/.bin/fbidq.html?fs(1)h), [en](http://flybase.bio.indiana.edu/.bin/fbidq.html?en), [dsx](http://flybase.bio.indiana.edu/.bin/fbidq.html?dsx), [tey](http://flybase.bio.indiana.edu/.bin/fbidq.html?tey), [HLHmgamma](http://flybase.bio.indiana.edu/.bin/fbidq.html?HLHmgamma), [z](http://flybase.bio.indiana.edu/.bin/fbidq.html?z), [HLHm7](http://flybase.bio.indiana.edu/.bin/fbidq.html?HLHm7), [Lim1](http://flybase.bio.indiana.edu/.bin/fbidq.html?Lim1), [Ct](http://flybase.bio.indiana.edu/.bin/fbidq.html?Ct) |
| [reproductive behavior](http://amigo.geneontology.org/cgi-bin/amigo/go.cgi?view=details&query=GO:0019098) | 36 of 1594 genes, 2.3% | 83 of 7634 genes, 1.1% | 0.00182 | 0.00% | 0.00 | [Sh](http://flybase.bio.indiana.edu/.bin/fbidq.html?Sh), [Ilp2](http://flybase.bio.indiana.edu/.bin/fbidq.html?Ilp2), [npf](http://flybase.bio.indiana.edu/.bin/fbidq.html?npf), [ato](http://flybase.bio.indiana.edu/.bin/fbidq.html?ato), [mnb](http://flybase.bio.indiana.edu/.bin/fbidq.html?mnb), [para](http://flybase.bio.indiana.edu/.bin/fbidq.html?para), [DopR](http://flybase.bio.indiana.edu/.bin/fbidq.html?DopR), [tay](http://flybase.bio.indiana.edu/.bin/fbidq.html?tay), [pros](http://flybase.bio.indiana.edu/.bin/fbidq.html?pros), [Tbh](http://flybase.bio.indiana.edu/.bin/fbidq.html?Tbh), [sol](http://flybase.bio.indiana.edu/.bin/fbidq.html?sol), [mle](http://flybase.bio.indiana.edu/.bin/fbidq.html?mle), [Oamb](http://flybase.bio.indiana.edu/.bin/fbidq.html?Oamb), [orb2](http://flybase.bio.indiana.edu/.bin/fbidq.html?orb2), [nonA](http://flybase.bio.indiana.edu/.bin/fbidq.html?nonA), [CG10251](http://flybase.bio.indiana.edu/.bin/fbidq.html?CG10251), [CASK](http://flybase.bio.indiana.edu/.bin/fbidq.html?CASK), [tipE](http://flybase.bio.indiana.edu/.bin/fbidq.html?tipE), [Poxn](http://flybase.bio.indiana.edu/.bin/fbidq.html?Poxn), [qtc](http://flybase.bio.indiana.edu/.bin/fbidq.html?qtc), [Pde1c](http://flybase.bio.indiana.edu/.bin/fbidq.html?Pde1c), [VGAT](http://flybase.bio.indiana.edu/.bin/fbidq.html?VGAT), [CaMKII](http://flybase.bio.indiana.edu/.bin/fbidq.html?CaMKII), [CG4476](http://flybase.bio.indiana.edu/.bin/fbidq.html?CG4476), [fne](http://flybase.bio.indiana.edu/.bin/fbidq.html?fne), [Ilp3](http://flybase.bio.indiana.edu/.bin/fbidq.html?Ilp3), [5-HT7](http://flybase.bio.indiana.edu/.bin/fbidq.html?5-HT7), [retn](http://flybase.bio.indiana.edu/.bin/fbidq.html?retn), [gfA](http://flybase.bio.indiana.edu/.bin/fbidq.html?gfA), [Btk29A](http://flybase.bio.indiana.edu/.bin/fbidq.html?Btk29A), [rut](http://flybase.bio.indiana.edu/.bin/fbidq.html?rut), [dsx](http://flybase.bio.indiana.edu/.bin/fbidq.html?dsx), [Nrg](http://flybase.bio.indiana.edu/.bin/fbidq.html?Nrg), [Ilp5](http://flybase.bio.indiana.edu/.bin/fbidq.html?Ilp5), [Ddc](http://flybase.bio.indiana.edu/.bin/fbidq.html?Ddc), [SPR](http://flybase.bio.indiana.edu/.bin/fbidq.html?SPR) |
| [regulation of signaling](http://amigo.geneontology.org/cgi-bin/amigo/go.cgi?view=details&query=GO:0023051) | 129 of 1594 genes, 8.1% | 442 of 7634 genes, 5.8% | 0.00183 | 0.00% | 0.00 | [Sh](http://flybase.bio.indiana.edu/.bin/fbidq.html?Sh), [numb](http://flybase.bio.indiana.edu/.bin/fbidq.html?numb), [skd](http://flybase.bio.indiana.edu/.bin/fbidq.html?skd), [Camta](http://flybase.bio.indiana.edu/.bin/fbidq.html?Camta), [ato](http://flybase.bio.indiana.edu/.bin/fbidq.html?ato), [Dg](http://flybase.bio.indiana.edu/.bin/fbidq.html?Dg), [cenG1A](http://flybase.bio.indiana.edu/.bin/fbidq.html?cenG1A), [Nf1](http://flybase.bio.indiana.edu/.bin/fbidq.html?Nf1), [Evi5](http://flybase.bio.indiana.edu/.bin/fbidq.html?Evi5), [CG42533](http://flybase.bio.indiana.edu/.bin/fbidq.html?CG42533), [os](http://flybase.bio.indiana.edu/.bin/fbidq.html?os), [CG11376](http://flybase.bio.indiana.edu/.bin/fbidq.html?CG11376), [Axn](http://flybase.bio.indiana.edu/.bin/fbidq.html?Axn), [brp](http://flybase.bio.indiana.edu/.bin/fbidq.html?brp), [Traf6](http://flybase.bio.indiana.edu/.bin/fbidq.html?Traf6), [CG31158](http://flybase.bio.indiana.edu/.bin/fbidq.html?CG31158), [rdgC](http://flybase.bio.indiana.edu/.bin/fbidq.html?rdgC), [ewg](http://flybase.bio.indiana.edu/.bin/fbidq.html?ewg), [Galpha49B](http://flybase.bio.indiana.edu/.bin/fbidq.html?Galpha49B), [Snoo](http://flybase.bio.indiana.edu/.bin/fbidq.html?Snoo), [Su(var)3-3](http://flybase.bio.indiana.edu/.bin/fbidq.html?Su(var)3-3), [Bili](http://flybase.bio.indiana.edu/.bin/fbidq.html?Bili), [uif](http://flybase.bio.indiana.edu/.bin/fbidq.html?uif), [Mmp2](http://flybase.bio.indiana.edu/.bin/fbidq.html?Mmp2), [Nipped-A](http://flybase.bio.indiana.edu/.bin/fbidq.html?Nipped-A), [alph](http://flybase.bio.indiana.edu/.bin/fbidq.html?alph), [Traf4](http://flybase.bio.indiana.edu/.bin/fbidq.html?Traf4), [otk](http://flybase.bio.indiana.edu/.bin/fbidq.html?otk), [Snap25](http://flybase.bio.indiana.edu/.bin/fbidq.html?Snap25), [Sos](http://flybase.bio.indiana.edu/.bin/fbidq.html?Sos), [phyl](http://flybase.bio.indiana.edu/.bin/fbidq.html?phyl), [mGluRA](http://flybase.bio.indiana.edu/.bin/fbidq.html?mGluRA), [dsh](http://flybase.bio.indiana.edu/.bin/fbidq.html?dsh), [pzg](http://flybase.bio.indiana.edu/.bin/fbidq.html?pzg), [Fas2](http://flybase.bio.indiana.edu/.bin/fbidq.html?Fas2), [Nf-YC](http://flybase.bio.indiana.edu/.bin/fbidq.html?Nf-YC),[CG8557](http://flybase.bio.indiana.edu/.bin/fbidq.html?CG8557), [bsk](http://flybase.bio.indiana.edu/.bin/fbidq.html?bsk), [brm](http://flybase.bio.indiana.edu/.bin/fbidq.html?brm), [comt](http://flybase.bio.indiana.edu/.bin/fbidq.html?comt), [ft](http://flybase.bio.indiana.edu/.bin/fbidq.html?ft), [Hey](http://flybase.bio.indiana.edu/.bin/fbidq.html?Hey), [plexB](http://flybase.bio.indiana.edu/.bin/fbidq.html?plexB), [qvr](http://flybase.bio.indiana.edu/.bin/fbidq.html?qvr), [pan](http://flybase.bio.indiana.edu/.bin/fbidq.html?pan), [neur](http://flybase.bio.indiana.edu/.bin/fbidq.html?neur), [Lis-1](http://flybase.bio.indiana.edu/.bin/fbidq.html?Lis-1), [rho-5](http://flybase.bio.indiana.edu/.bin/fbidq.html?rho-5), [nmo](http://flybase.bio.indiana.edu/.bin/fbidq.html?nmo), [CG16896](http://flybase.bio.indiana.edu/.bin/fbidq.html?CG16896), [trio](http://flybase.bio.indiana.edu/.bin/fbidq.html?trio), [Gug](http://flybase.bio.indiana.edu/.bin/fbidq.html?Gug), [ed](http://flybase.bio.indiana.edu/.bin/fbidq.html?ed), [mr](http://flybase.bio.indiana.edu/.bin/fbidq.html?mr), [daw](http://flybase.bio.indiana.edu/.bin/fbidq.html?daw), [CG5036](http://flybase.bio.indiana.edu/.bin/fbidq.html?CG5036), [Apc](http://flybase.bio.indiana.edu/.bin/fbidq.html?Apc), [CG30372](http://flybase.bio.indiana.edu/.bin/fbidq.html?CG30372), [gro](http://flybase.bio.indiana.edu/.bin/fbidq.html?gro), [CG10188](http://flybase.bio.indiana.edu/.bin/fbidq.html?CG10188), [CG42629](http://flybase.bio.indiana.edu/.bin/fbidq.html?CG42629), [RN-tre](http://flybase.bio.indiana.edu/.bin/fbidq.html?RN-tre), [pigs](http://flybase.bio.indiana.edu/.bin/fbidq.html?pigs), [scyl](http://flybase.bio.indiana.edu/.bin/fbidq.html?scyl), [cdi](http://flybase.bio.indiana.edu/.bin/fbidq.html?cdi), [klu](http://flybase.bio.indiana.edu/.bin/fbidq.html?klu), [CG8155](http://flybase.bio.indiana.edu/.bin/fbidq.html?CG8155), [pygo](http://flybase.bio.indiana.edu/.bin/fbidq.html?pygo), [tld](http://flybase.bio.indiana.edu/.bin/fbidq.html?tld), [tow](http://flybase.bio.indiana.edu/.bin/fbidq.html?tow), [RhoGEF3](http://flybase.bio.indiana.edu/.bin/fbidq.html?RhoGEF3), [CG15609](http://flybase.bio.indiana.edu/.bin/fbidq.html?CG15609), [Atg1](http://flybase.bio.indiana.edu/.bin/fbidq.html?Atg1),[RhoGEF4](http://flybase.bio.indiana.edu/.bin/fbidq.html?RhoGEF4), [CG12187](http://flybase.bio.indiana.edu/.bin/fbidq.html?CG12187), [Pten](http://flybase.bio.indiana.edu/.bin/fbidq.html?Pten), [sgg](http://flybase.bio.indiana.edu/.bin/fbidq.html?sgg), [Cbl](http://flybase.bio.indiana.edu/.bin/fbidq.html?Cbl), [rab3-GEF](http://flybase.bio.indiana.edu/.bin/fbidq.html?rab3-GEF), [CG13253](http://flybase.bio.indiana.edu/.bin/fbidq.html?CG13253), [MESK2](http://flybase.bio.indiana.edu/.bin/fbidq.html?MESK2), [sNPF](http://flybase.bio.indiana.edu/.bin/fbidq.html?sNPF), [plx](http://flybase.bio.indiana.edu/.bin/fbidq.html?plx), [CASK](http://flybase.bio.indiana.edu/.bin/fbidq.html?CASK), [CG8108](http://flybase.bio.indiana.edu/.bin/fbidq.html?CG8108), [Syt4](http://flybase.bio.indiana.edu/.bin/fbidq.html?Syt4), [Alk](http://flybase.bio.indiana.edu/.bin/fbidq.html?Alk), [cpx](http://flybase.bio.indiana.edu/.bin/fbidq.html?cpx), [casp](http://flybase.bio.indiana.edu/.bin/fbidq.html?casp), [CG4022](http://flybase.bio.indiana.edu/.bin/fbidq.html?CG4022), [fred](http://flybase.bio.indiana.edu/.bin/fbidq.html?fred), [Fak56D](http://flybase.bio.indiana.edu/.bin/fbidq.html?Fak56D), [CG3227](http://flybase.bio.indiana.edu/.bin/fbidq.html?CG3227), [Gap1](http://flybase.bio.indiana.edu/.bin/fbidq.html?Gap1), [MESR4](http://flybase.bio.indiana.edu/.bin/fbidq.html?MESR4), [sNPF-R](http://flybase.bio.indiana.edu/.bin/fbidq.html?sNPF-R), [Rdl](http://flybase.bio.indiana.edu/.bin/fbidq.html?Rdl), [CG5337](http://flybase.bio.indiana.edu/.bin/fbidq.html?CG5337), [stan](http://flybase.bio.indiana.edu/.bin/fbidq.html?stan), [GABA-B-R2](http://flybase.bio.indiana.edu/.bin/fbidq.html?GABA-B-R2), [Frq1](http://flybase.bio.indiana.edu/.bin/fbidq.html?Frq1), [Taf1](http://flybase.bio.indiana.edu/.bin/fbidq.html?Taf1), [Sur-8](http://flybase.bio.indiana.edu/.bin/fbidq.html?Sur-8), [Rgl](http://flybase.bio.indiana.edu/.bin/fbidq.html?Rgl), [e(y)3](http://flybase.bio.indiana.edu/.bin/fbidq.html?e(y)3), [sif](http://flybase.bio.indiana.edu/.bin/fbidq.html?sif),[RSG7](http://flybase.bio.indiana.edu/.bin/fbidq.html?RSG7), [dom](http://flybase.bio.indiana.edu/.bin/fbidq.html?dom), [ey](http://flybase.bio.indiana.edu/.bin/fbidq.html?ey), [CG33275](http://flybase.bio.indiana.edu/.bin/fbidq.html?CG33275), [baz](http://flybase.bio.indiana.edu/.bin/fbidq.html?baz), [Spred](http://flybase.bio.indiana.edu/.bin/fbidq.html?Spred), [CG11155](http://flybase.bio.indiana.edu/.bin/fbidq.html?CG11155), [shi](http://flybase.bio.indiana.edu/.bin/fbidq.html?shi), [siz](http://flybase.bio.indiana.edu/.bin/fbidq.html?siz), [CG3822](http://flybase.bio.indiana.edu/.bin/fbidq.html?CG3822), [CG40351](http://flybase.bio.indiana.edu/.bin/fbidq.html?CG40351), [wg](http://flybase.bio.indiana.edu/.bin/fbidq.html?wg), [crol](http://flybase.bio.indiana.edu/.bin/fbidq.html?crol), [Aplip1](http://flybase.bio.indiana.edu/.bin/fbidq.html?Aplip1), [E(bx)](http://flybase.bio.indiana.edu/.bin/fbidq.html?E(bx)), [sfl](http://flybase.bio.indiana.edu/.bin/fbidq.html?sfl), [Syt1](http://flybase.bio.indiana.edu/.bin/fbidq.html?Syt1), [rl](http://flybase.bio.indiana.edu/.bin/fbidq.html?rl), [Utx](http://flybase.bio.indiana.edu/.bin/fbidq.html?Utx), [wls](http://flybase.bio.indiana.edu/.bin/fbidq.html?wls), [hiw](http://flybase.bio.indiana.edu/.bin/fbidq.html?hiw), [cos](http://flybase.bio.indiana.edu/.bin/fbidq.html?cos), [Ct](http://flybase.bio.indiana.edu/.bin/fbidq.html?Ct) |
| [regulation of system process](http://amigo.geneontology.org/cgi-bin/amigo/go.cgi?view=details&query=GO:0044057) | 29 of 1594 genes, 1.8% | 61 of 7634 genes, 0.8% | 0.00214 | 0.00% | 0.00 | [shi](http://flybase.bio.indiana.edu/.bin/fbidq.html?shi), [CG11155](http://flybase.bio.indiana.edu/.bin/fbidq.html?CG11155), [Sh](http://flybase.bio.indiana.edu/.bin/fbidq.html?Sh), [Syt4](http://flybase.bio.indiana.edu/.bin/fbidq.html?Syt4), [Dsk](http://flybase.bio.indiana.edu/.bin/fbidq.html?Dsk), [comt](http://flybase.bio.indiana.edu/.bin/fbidq.html?comt), [Frq1](http://flybase.bio.indiana.edu/.bin/fbidq.html?Frq1), [Alk](http://flybase.bio.indiana.edu/.bin/fbidq.html?Alk), [CG3822](http://flybase.bio.indiana.edu/.bin/fbidq.html?CG3822), [Atg1](http://flybase.bio.indiana.edu/.bin/fbidq.html?Atg1), [CG12187](http://flybase.bio.indiana.edu/.bin/fbidq.html?CG12187), [qvr](http://flybase.bio.indiana.edu/.bin/fbidq.html?qvr), [CG42629](http://flybase.bio.indiana.edu/.bin/fbidq.html?CG42629), [cpx](http://flybase.bio.indiana.edu/.bin/fbidq.html?cpx), [Fmrf](http://flybase.bio.indiana.edu/.bin/fbidq.html?Fmrf), [Dg](http://flybase.bio.indiana.edu/.bin/fbidq.html?Dg), [rl](http://flybase.bio.indiana.edu/.bin/fbidq.html?rl), [Syt1](http://flybase.bio.indiana.edu/.bin/fbidq.html?Syt1), [Dms](http://flybase.bio.indiana.edu/.bin/fbidq.html?Dms), [Rdl](http://flybase.bio.indiana.edu/.bin/fbidq.html?Rdl), [Snap25](http://flybase.bio.indiana.edu/.bin/fbidq.html?Snap25), [brp](http://flybase.bio.indiana.edu/.bin/fbidq.html?brp), [mr](http://flybase.bio.indiana.edu/.bin/fbidq.html?mr), [GABA-B-R2](http://flybase.bio.indiana.edu/.bin/fbidq.html?GABA-B-R2), [Ptth](http://flybase.bio.indiana.edu/.bin/fbidq.html?Ptth), [CASK](http://flybase.bio.indiana.edu/.bin/fbidq.html?CASK), [mGluRA](http://flybase.bio.indiana.edu/.bin/fbidq.html?mGluRA), [Ast-C](http://flybase.bio.indiana.edu/.bin/fbidq.html?Ast-C), [Ccap](http://flybase.bio.indiana.edu/.bin/fbidq.html?Ccap) |
| [axonal defasciculation](http://amigo.geneontology.org/cgi-bin/amigo/go.cgi?view=details&query=GO:0007414) | 11 of 1594 genes, 0.7% | 13 of 7634 genes, 0.2% | 0.00216 | 0.00% | 0.00 | [Ptp99A](http://flybase.bio.indiana.edu/.bin/fbidq.html?Ptp99A), [mew](http://flybase.bio.indiana.edu/.bin/fbidq.html?mew), [beat-Ic](http://flybase.bio.indiana.edu/.bin/fbidq.html?beat-Ic), [beat-Ia](http://flybase.bio.indiana.edu/.bin/fbidq.html?beat-Ia), [p130CAS](http://flybase.bio.indiana.edu/.bin/fbidq.html?p130CAS), [RhoGAPp190](http://flybase.bio.indiana.edu/.bin/fbidq.html?RhoGAPp190), [beat-Ib](http://flybase.bio.indiana.edu/.bin/fbidq.html?beat-Ib), [beat-IIa](http://flybase.bio.indiana.edu/.bin/fbidq.html?beat-IIa), [Ptp69D](http://flybase.bio.indiana.edu/.bin/fbidq.html?Ptp69D), [tok](http://flybase.bio.indiana.edu/.bin/fbidq.html?tok), [tutl](http://flybase.bio.indiana.edu/.bin/fbidq.html?tutl) |
| [leg disc development](http://amigo.geneontology.org/cgi-bin/amigo/go.cgi?view=details&query=GO:0035218) | 34 of 1594 genes, 2.1% | 77 of 7634 genes, 1.0% | 0.00222 | 0.00% | 0.00 | [Sp1](http://flybase.bio.indiana.edu/.bin/fbidq.html?Sp1), [vg](http://flybase.bio.indiana.edu/.bin/fbidq.html?vg), [hth](http://flybase.bio.indiana.edu/.bin/fbidq.html?hth), [brm](http://flybase.bio.indiana.edu/.bin/fbidq.html?brm), [CG8557](http://flybase.bio.indiana.edu/.bin/fbidq.html?CG8557), [Ubx](http://flybase.bio.indiana.edu/.bin/fbidq.html?Ubx), [skd](http://flybase.bio.indiana.edu/.bin/fbidq.html?skd), [jing](http://flybase.bio.indiana.edu/.bin/fbidq.html?jing), [Dll](http://flybase.bio.indiana.edu/.bin/fbidq.html?Dll), [tsh](http://flybase.bio.indiana.edu/.bin/fbidq.html?tsh), [bowl](http://flybase.bio.indiana.edu/.bin/fbidq.html?bowl), [rn](http://flybase.bio.indiana.edu/.bin/fbidq.html?rn), [ap](http://flybase.bio.indiana.edu/.bin/fbidq.html?ap), [dac](http://flybase.bio.indiana.edu/.bin/fbidq.html?dac), [e(y)3](http://flybase.bio.indiana.edu/.bin/fbidq.html?e(y)3), [Bx](http://flybase.bio.indiana.edu/.bin/fbidq.html?Bx), [CG32149](http://flybase.bio.indiana.edu/.bin/fbidq.html?CG32149), [ovo](http://flybase.bio.indiana.edu/.bin/fbidq.html?ovo), [trio](http://flybase.bio.indiana.edu/.bin/fbidq.html?trio), [Gug](http://flybase.bio.indiana.edu/.bin/fbidq.html?Gug), [Dsp1](http://flybase.bio.indiana.edu/.bin/fbidq.html?Dsp1), [CG33275](http://flybase.bio.indiana.edu/.bin/fbidq.html?CG33275), [ds](http://flybase.bio.indiana.edu/.bin/fbidq.html?ds), [B-H2](http://flybase.bio.indiana.edu/.bin/fbidq.html?B-H2), [Poxn](http://flybase.bio.indiana.edu/.bin/fbidq.html?Poxn), [wg](http://flybase.bio.indiana.edu/.bin/fbidq.html?wg), [sd](http://flybase.bio.indiana.edu/.bin/fbidq.html?sd), [bab1](http://flybase.bio.indiana.edu/.bin/fbidq.html?bab1), [btd](http://flybase.bio.indiana.edu/.bin/fbidq.html?btd), [B-H1](http://flybase.bio.indiana.edu/.bin/fbidq.html?B-H1), [wls](http://flybase.bio.indiana.edu/.bin/fbidq.html?wls), [Hs3st-B](http://flybase.bio.indiana.edu/.bin/fbidq.html?Hs3st-B), [Sos](http://flybase.bio.indiana.edu/.bin/fbidq.html?Sos), [Lim1](http://flybase.bio.indiana.edu/.bin/fbidq.html?Lim1) |
| [dendrite morphogenesis](http://amigo.geneontology.org/cgi-bin/amigo/go.cgi?view=details&query=GO:0048813) | 54 of 1594 genes, 3.4% | 147 of 7634 genes, 1.9% | 0.00264 | 0.00% | 0.00 | [robo](http://flybase.bio.indiana.edu/.bin/fbidq.html?robo), [futsch](http://flybase.bio.indiana.edu/.bin/fbidq.html?futsch), [brm](http://flybase.bio.indiana.edu/.bin/fbidq.html?brm), [acj6](http://flybase.bio.indiana.edu/.bin/fbidq.html?acj6), [Ptx1](http://flybase.bio.indiana.edu/.bin/fbidq.html?Ptx1), [kat-60L1](http://flybase.bio.indiana.edu/.bin/fbidq.html?kat-60L1), [wor](http://flybase.bio.indiana.edu/.bin/fbidq.html?wor), [vvl](http://flybase.bio.indiana.edu/.bin/fbidq.html?vvl), [Pten](http://flybase.bio.indiana.edu/.bin/fbidq.html?Pten), [chm](http://flybase.bio.indiana.edu/.bin/fbidq.html?chm), [sna](http://flybase.bio.indiana.edu/.bin/fbidq.html?sna), [ato](http://flybase.bio.indiana.edu/.bin/fbidq.html?ato), [Ptp69D](http://flybase.bio.indiana.edu/.bin/fbidq.html?Ptp69D), [gcm2](http://flybase.bio.indiana.edu/.bin/fbidq.html?gcm2), [Iswi](http://flybase.bio.indiana.edu/.bin/fbidq.html?Iswi), [seq](http://flybase.bio.indiana.edu/.bin/fbidq.html?seq), [gcm](http://flybase.bio.indiana.edu/.bin/fbidq.html?gcm), [Lis-1](http://flybase.bio.indiana.edu/.bin/fbidq.html?Lis-1), [ems](http://flybase.bio.indiana.edu/.bin/fbidq.html?ems), [dom](http://flybase.bio.indiana.edu/.bin/fbidq.html?dom), [pros](http://flybase.bio.indiana.edu/.bin/fbidq.html?pros), [tup](http://flybase.bio.indiana.edu/.bin/fbidq.html?tup), [comm](http://flybase.bio.indiana.edu/.bin/fbidq.html?comm), [NetB](http://flybase.bio.indiana.edu/.bin/fbidq.html?NetB), [Tango10](http://flybase.bio.indiana.edu/.bin/fbidq.html?Tango10), [rictor](http://flybase.bio.indiana.edu/.bin/fbidq.html?rictor), [Sin3A](http://flybase.bio.indiana.edu/.bin/fbidq.html?Sin3A), [d4](http://flybase.bio.indiana.edu/.bin/fbidq.html?d4), [CadN](http://flybase.bio.indiana.edu/.bin/fbidq.html?CadN), [gro](http://flybase.bio.indiana.edu/.bin/fbidq.html?gro), [nerfin-1](http://flybase.bio.indiana.edu/.bin/fbidq.html?nerfin-1), [CG4328](http://flybase.bio.indiana.edu/.bin/fbidq.html?CG4328), [E(Pc)](http://flybase.bio.indiana.edu/.bin/fbidq.html?E(Pc)), [jumu](http://flybase.bio.indiana.edu/.bin/fbidq.html?jumu), [Smox](http://flybase.bio.indiana.edu/.bin/fbidq.html?Smox), [Rpd3](http://flybase.bio.indiana.edu/.bin/fbidq.html?Rpd3), [Taf4](http://flybase.bio.indiana.edu/.bin/fbidq.html?Taf4), [nvy](http://flybase.bio.indiana.edu/.bin/fbidq.html?nvy),[E(bx)](http://flybase.bio.indiana.edu/.bin/fbidq.html?E(bx)), [Nrg](http://flybase.bio.indiana.edu/.bin/fbidq.html?Nrg), [chinmo](http://flybase.bio.indiana.edu/.bin/fbidq.html?chinmo), [run](http://flybase.bio.indiana.edu/.bin/fbidq.html?run), [CG10107](http://flybase.bio.indiana.edu/.bin/fbidq.html?CG10107), [sqz](http://flybase.bio.indiana.edu/.bin/fbidq.html?sqz), [Actbeta](http://flybase.bio.indiana.edu/.bin/fbidq.html?Actbeta), [Sema-1a](http://flybase.bio.indiana.edu/.bin/fbidq.html?Sema-1a), [CG33960](http://flybase.bio.indiana.edu/.bin/fbidq.html?CG33960), [pygo](http://flybase.bio.indiana.edu/.bin/fbidq.html?pygo), [HLHm7](http://flybase.bio.indiana.edu/.bin/fbidq.html?HLHm7), [stan](http://flybase.bio.indiana.edu/.bin/fbidq.html?stan), [Ulp1](http://flybase.bio.indiana.edu/.bin/fbidq.html?Ulp1), [tutl](http://flybase.bio.indiana.edu/.bin/fbidq.html?tutl), [Ct](http://flybase.bio.indiana.edu/.bin/fbidq.html?Ct), [ena](http://flybase.bio.indiana.edu/.bin/fbidq.html?ena) |
| [regulation of cell communication](http://amigo.geneontology.org/cgi-bin/amigo/go.cgi?view=details&query=GO:0010646) | 129 of 1594 genes, 8.1% | 445 of 7634 genes, 5.8% | 0.00273 | 0.00% | 0.00 | [Sh](http://flybase.bio.indiana.edu/.bin/fbidq.html?Sh), [numb](http://flybase.bio.indiana.edu/.bin/fbidq.html?numb), [skd](http://flybase.bio.indiana.edu/.bin/fbidq.html?skd), [Camta](http://flybase.bio.indiana.edu/.bin/fbidq.html?Camta), [ato](http://flybase.bio.indiana.edu/.bin/fbidq.html?ato), [Dg](http://flybase.bio.indiana.edu/.bin/fbidq.html?Dg), [cenG1A](http://flybase.bio.indiana.edu/.bin/fbidq.html?cenG1A), [Nf1](http://flybase.bio.indiana.edu/.bin/fbidq.html?Nf1), [Evi5](http://flybase.bio.indiana.edu/.bin/fbidq.html?Evi5), [CG42533](http://flybase.bio.indiana.edu/.bin/fbidq.html?CG42533), [os](http://flybase.bio.indiana.edu/.bin/fbidq.html?os), [CG11376](http://flybase.bio.indiana.edu/.bin/fbidq.html?CG11376), [Axn](http://flybase.bio.indiana.edu/.bin/fbidq.html?Axn), [brp](http://flybase.bio.indiana.edu/.bin/fbidq.html?brp), [Traf6](http://flybase.bio.indiana.edu/.bin/fbidq.html?Traf6), [CG31158](http://flybase.bio.indiana.edu/.bin/fbidq.html?CG31158), [rdgC](http://flybase.bio.indiana.edu/.bin/fbidq.html?rdgC), [ewg](http://flybase.bio.indiana.edu/.bin/fbidq.html?ewg), [Galpha49B](http://flybase.bio.indiana.edu/.bin/fbidq.html?Galpha49B), [Snoo](http://flybase.bio.indiana.edu/.bin/fbidq.html?Snoo), [Su(var)3-3](http://flybase.bio.indiana.edu/.bin/fbidq.html?Su(var)3-3), [Bili](http://flybase.bio.indiana.edu/.bin/fbidq.html?Bili), [uif](http://flybase.bio.indiana.edu/.bin/fbidq.html?uif), [Mmp2](http://flybase.bio.indiana.edu/.bin/fbidq.html?Mmp2), [Nipped-A](http://flybase.bio.indiana.edu/.bin/fbidq.html?Nipped-A), [alph](http://flybase.bio.indiana.edu/.bin/fbidq.html?alph), [Traf4](http://flybase.bio.indiana.edu/.bin/fbidq.html?Traf4), [otk](http://flybase.bio.indiana.edu/.bin/fbidq.html?otk), [Snap25](http://flybase.bio.indiana.edu/.bin/fbidq.html?Snap25), [Sos](http://flybase.bio.indiana.edu/.bin/fbidq.html?Sos), [phyl](http://flybase.bio.indiana.edu/.bin/fbidq.html?phyl), [mGluRA](http://flybase.bio.indiana.edu/.bin/fbidq.html?mGluRA), [dsh](http://flybase.bio.indiana.edu/.bin/fbidq.html?dsh), [pzg](http://flybase.bio.indiana.edu/.bin/fbidq.html?pzg), [Fas2](http://flybase.bio.indiana.edu/.bin/fbidq.html?Fas2), [Nf-YC](http://flybase.bio.indiana.edu/.bin/fbidq.html?Nf-YC),[CG8557](http://flybase.bio.indiana.edu/.bin/fbidq.html?CG8557), [bsk](http://flybase.bio.indiana.edu/.bin/fbidq.html?bsk), [brm](http://flybase.bio.indiana.edu/.bin/fbidq.html?brm), [comt](http://flybase.bio.indiana.edu/.bin/fbidq.html?comt), [ft](http://flybase.bio.indiana.edu/.bin/fbidq.html?ft), [Hey](http://flybase.bio.indiana.edu/.bin/fbidq.html?Hey), [plexB](http://flybase.bio.indiana.edu/.bin/fbidq.html?plexB), [qvr](http://flybase.bio.indiana.edu/.bin/fbidq.html?qvr), [pan](http://flybase.bio.indiana.edu/.bin/fbidq.html?pan), [neur](http://flybase.bio.indiana.edu/.bin/fbidq.html?neur), [Lis-1](http://flybase.bio.indiana.edu/.bin/fbidq.html?Lis-1), [rho-5](http://flybase.bio.indiana.edu/.bin/fbidq.html?rho-5), [nmo](http://flybase.bio.indiana.edu/.bin/fbidq.html?nmo), [CG16896](http://flybase.bio.indiana.edu/.bin/fbidq.html?CG16896), [trio](http://flybase.bio.indiana.edu/.bin/fbidq.html?trio), [Gug](http://flybase.bio.indiana.edu/.bin/fbidq.html?Gug), [ed](http://flybase.bio.indiana.edu/.bin/fbidq.html?ed), [mr](http://flybase.bio.indiana.edu/.bin/fbidq.html?mr), [daw](http://flybase.bio.indiana.edu/.bin/fbidq.html?daw), [CG5036](http://flybase.bio.indiana.edu/.bin/fbidq.html?CG5036), [Apc](http://flybase.bio.indiana.edu/.bin/fbidq.html?Apc), [CG30372](http://flybase.bio.indiana.edu/.bin/fbidq.html?CG30372), [gro](http://flybase.bio.indiana.edu/.bin/fbidq.html?gro), [CG10188](http://flybase.bio.indiana.edu/.bin/fbidq.html?CG10188), [CG42629](http://flybase.bio.indiana.edu/.bin/fbidq.html?CG42629), [RN-tre](http://flybase.bio.indiana.edu/.bin/fbidq.html?RN-tre), [pigs](http://flybase.bio.indiana.edu/.bin/fbidq.html?pigs), [scyl](http://flybase.bio.indiana.edu/.bin/fbidq.html?scyl), [cdi](http://flybase.bio.indiana.edu/.bin/fbidq.html?cdi), [klu](http://flybase.bio.indiana.edu/.bin/fbidq.html?klu), [CG8155](http://flybase.bio.indiana.edu/.bin/fbidq.html?CG8155), [pygo](http://flybase.bio.indiana.edu/.bin/fbidq.html?pygo), [tld](http://flybase.bio.indiana.edu/.bin/fbidq.html?tld), [tow](http://flybase.bio.indiana.edu/.bin/fbidq.html?tow), [RhoGEF3](http://flybase.bio.indiana.edu/.bin/fbidq.html?RhoGEF3), [CG15609](http://flybase.bio.indiana.edu/.bin/fbidq.html?CG15609), [Atg1](http://flybase.bio.indiana.edu/.bin/fbidq.html?Atg1),[RhoGEF4](http://flybase.bio.indiana.edu/.bin/fbidq.html?RhoGEF4), [CG12187](http://flybase.bio.indiana.edu/.bin/fbidq.html?CG12187), [Pten](http://flybase.bio.indiana.edu/.bin/fbidq.html?Pten), [sgg](http://flybase.bio.indiana.edu/.bin/fbidq.html?sgg), [Cbl](http://flybase.bio.indiana.edu/.bin/fbidq.html?Cbl), [rab3-GEF](http://flybase.bio.indiana.edu/.bin/fbidq.html?rab3-GEF), [CG13253](http://flybase.bio.indiana.edu/.bin/fbidq.html?CG13253), [MESK2](http://flybase.bio.indiana.edu/.bin/fbidq.html?MESK2), [sNPF](http://flybase.bio.indiana.edu/.bin/fbidq.html?sNPF), [plx](http://flybase.bio.indiana.edu/.bin/fbidq.html?plx), [CASK](http://flybase.bio.indiana.edu/.bin/fbidq.html?CASK), [CG8108](http://flybase.bio.indiana.edu/.bin/fbidq.html?CG8108), [Syt4](http://flybase.bio.indiana.edu/.bin/fbidq.html?Syt4), [Alk](http://flybase.bio.indiana.edu/.bin/fbidq.html?Alk), [cpx](http://flybase.bio.indiana.edu/.bin/fbidq.html?cpx), [casp](http://flybase.bio.indiana.edu/.bin/fbidq.html?casp), [CG4022](http://flybase.bio.indiana.edu/.bin/fbidq.html?CG4022), [fred](http://flybase.bio.indiana.edu/.bin/fbidq.html?fred), [Fak56D](http://flybase.bio.indiana.edu/.bin/fbidq.html?Fak56D), [CG3227](http://flybase.bio.indiana.edu/.bin/fbidq.html?CG3227), [Gap1](http://flybase.bio.indiana.edu/.bin/fbidq.html?Gap1), [MESR4](http://flybase.bio.indiana.edu/.bin/fbidq.html?MESR4), [sNPF-R](http://flybase.bio.indiana.edu/.bin/fbidq.html?sNPF-R), [Rdl](http://flybase.bio.indiana.edu/.bin/fbidq.html?Rdl), [CG5337](http://flybase.bio.indiana.edu/.bin/fbidq.html?CG5337), [stan](http://flybase.bio.indiana.edu/.bin/fbidq.html?stan), [GABA-B-R2](http://flybase.bio.indiana.edu/.bin/fbidq.html?GABA-B-R2), [Frq1](http://flybase.bio.indiana.edu/.bin/fbidq.html?Frq1), [Taf1](http://flybase.bio.indiana.edu/.bin/fbidq.html?Taf1), [Sur-8](http://flybase.bio.indiana.edu/.bin/fbidq.html?Sur-8), [Rgl](http://flybase.bio.indiana.edu/.bin/fbidq.html?Rgl), [e(y)3](http://flybase.bio.indiana.edu/.bin/fbidq.html?e(y)3), [sif](http://flybase.bio.indiana.edu/.bin/fbidq.html?sif),[RSG7](http://flybase.bio.indiana.edu/.bin/fbidq.html?RSG7), [dom](http://flybase.bio.indiana.edu/.bin/fbidq.html?dom), [ey](http://flybase.bio.indiana.edu/.bin/fbidq.html?ey), [CG33275](http://flybase.bio.indiana.edu/.bin/fbidq.html?CG33275), [baz](http://flybase.bio.indiana.edu/.bin/fbidq.html?baz), [Spred](http://flybase.bio.indiana.edu/.bin/fbidq.html?Spred), [CG11155](http://flybase.bio.indiana.edu/.bin/fbidq.html?CG11155), [shi](http://flybase.bio.indiana.edu/.bin/fbidq.html?shi), [siz](http://flybase.bio.indiana.edu/.bin/fbidq.html?siz), [CG3822](http://flybase.bio.indiana.edu/.bin/fbidq.html?CG3822), [CG40351](http://flybase.bio.indiana.edu/.bin/fbidq.html?CG40351), [wg](http://flybase.bio.indiana.edu/.bin/fbidq.html?wg), [crol](http://flybase.bio.indiana.edu/.bin/fbidq.html?crol), [Aplip1](http://flybase.bio.indiana.edu/.bin/fbidq.html?Aplip1), [E(bx)](http://flybase.bio.indiana.edu/.bin/fbidq.html?E(bx)), [sfl](http://flybase.bio.indiana.edu/.bin/fbidq.html?sfl), [Syt1](http://flybase.bio.indiana.edu/.bin/fbidq.html?Syt1), [rl](http://flybase.bio.indiana.edu/.bin/fbidq.html?rl), [Utx](http://flybase.bio.indiana.edu/.bin/fbidq.html?Utx), [wls](http://flybase.bio.indiana.edu/.bin/fbidq.html?wls), [hiw](http://flybase.bio.indiana.edu/.bin/fbidq.html?hiw), [cos](http://flybase.bio.indiana.edu/.bin/fbidq.html?cos), [Ct](http://flybase.bio.indiana.edu/.bin/fbidq.html?Ct) |
| [negative regulation of biosynthetic process](http://amigo.geneontology.org/cgi-bin/amigo/go.cgi?view=details&query=GO:0009890) | 77 of 1594 genes, 4.8% | 235 of 7634 genes, 3.1% | 0.00327 | 0.00% | 0.00 | [jing](http://flybase.bio.indiana.edu/.bin/fbidq.html?jing), [Sxl](http://flybase.bio.indiana.edu/.bin/fbidq.html?Sxl), [JIL-1](http://flybase.bio.indiana.edu/.bin/fbidq.html?JIL-1), [rn](http://flybase.bio.indiana.edu/.bin/fbidq.html?rn), [Iswi](http://flybase.bio.indiana.edu/.bin/fbidq.html?Iswi), [seq](http://flybase.bio.indiana.edu/.bin/fbidq.html?seq), [Aef1](http://flybase.bio.indiana.edu/.bin/fbidq.html?Aef1), [ovo](http://flybase.bio.indiana.edu/.bin/fbidq.html?ovo), [Dsp1](http://flybase.bio.indiana.edu/.bin/fbidq.html?Dsp1), [bi](http://flybase.bio.indiana.edu/.bin/fbidq.html?bi), [chn](http://flybase.bio.indiana.edu/.bin/fbidq.html?chn), [Gsc](http://flybase.bio.indiana.edu/.bin/fbidq.html?Gsc), [Nelf-A](http://flybase.bio.indiana.edu/.bin/fbidq.html?Nelf-A), [Pcl](http://flybase.bio.indiana.edu/.bin/fbidq.html?Pcl), [simj](http://flybase.bio.indiana.edu/.bin/fbidq.html?simj), [Sin3A](http://flybase.bio.indiana.edu/.bin/fbidq.html?Sin3A), [NC2alpha](http://flybase.bio.indiana.edu/.bin/fbidq.html?NC2alpha), [mip120](http://flybase.bio.indiana.edu/.bin/fbidq.html?mip120), [casp](http://flybase.bio.indiana.edu/.bin/fbidq.html?casp), [Snoo](http://flybase.bio.indiana.edu/.bin/fbidq.html?Snoo), [jumu](http://flybase.bio.indiana.edu/.bin/fbidq.html?jumu), [HGTX](http://flybase.bio.indiana.edu/.bin/fbidq.html?HGTX), [ush](http://flybase.bio.indiana.edu/.bin/fbidq.html?ush), [Usp7](http://flybase.bio.indiana.edu/.bin/fbidq.html?Usp7), [fd59A](http://flybase.bio.indiana.edu/.bin/fbidq.html?fd59A), [nab](http://flybase.bio.indiana.edu/.bin/fbidq.html?nab), [CG10336](http://flybase.bio.indiana.edu/.bin/fbidq.html?CG10336), [btd](http://flybase.bio.indiana.edu/.bin/fbidq.html?btd), [fd68A](http://flybase.bio.indiana.edu/.bin/fbidq.html?fd68A), [HLHmdelta](http://flybase.bio.indiana.edu/.bin/fbidq.html?HLHmdelta), [run](http://flybase.bio.indiana.edu/.bin/fbidq.html?run), [B-H1](http://flybase.bio.indiana.edu/.bin/fbidq.html?B-H1), [FBX011](http://flybase.bio.indiana.edu/.bin/fbidq.html?FBX011), [egg](http://flybase.bio.indiana.edu/.bin/fbidq.html?egg), [l(3)neo38](http://flybase.bio.indiana.edu/.bin/fbidq.html?l(3)neo38), [fd102C](http://flybase.bio.indiana.edu/.bin/fbidq.html?fd102C), [Ast-C](http://flybase.bio.indiana.edu/.bin/fbidq.html?Ast-C),[Vsx2](http://flybase.bio.indiana.edu/.bin/fbidq.html?Vsx2), [ph-p](http://flybase.bio.indiana.edu/.bin/fbidq.html?ph-p), [Ubx](http://flybase.bio.indiana.edu/.bin/fbidq.html?Ubx), [CG7757](http://flybase.bio.indiana.edu/.bin/fbidq.html?CG7757), [chm](http://flybase.bio.indiana.edu/.bin/fbidq.html?chm), [Sfmbt](http://flybase.bio.indiana.edu/.bin/fbidq.html?Sfmbt), [sna](http://flybase.bio.indiana.edu/.bin/fbidq.html?sna), [Wnt5](http://flybase.bio.indiana.edu/.bin/fbidq.html?Wnt5), [pan](http://flybase.bio.indiana.edu/.bin/fbidq.html?pan), [MBD-like](http://flybase.bio.indiana.edu/.bin/fbidq.html?MBD-like), [tsh](http://flybase.bio.indiana.edu/.bin/fbidq.html?tsh), [HLHm5](http://flybase.bio.indiana.edu/.bin/fbidq.html?HLHm5), [esg](http://flybase.bio.indiana.edu/.bin/fbidq.html?esg), [e(y)3](http://flybase.bio.indiana.edu/.bin/fbidq.html?e(y)3), [oc](http://flybase.bio.indiana.edu/.bin/fbidq.html?oc), [pros](http://flybase.bio.indiana.edu/.bin/fbidq.html?pros), [Gug](http://flybase.bio.indiana.edu/.bin/fbidq.html?Gug), [pad](http://flybase.bio.indiana.edu/.bin/fbidq.html?pad), [orb2](http://flybase.bio.indiana.edu/.bin/fbidq.html?orb2), [Wnt4](http://flybase.bio.indiana.edu/.bin/fbidq.html?Wnt4), [B-H2](http://flybase.bio.indiana.edu/.bin/fbidq.html?B-H2), [wg](http://flybase.bio.indiana.edu/.bin/fbidq.html?wg), [Vsx1](http://flybase.bio.indiana.edu/.bin/fbidq.html?Vsx1), [fkh](http://flybase.bio.indiana.edu/.bin/fbidq.html?fkh), [gro](http://flybase.bio.indiana.edu/.bin/fbidq.html?gro), [crol](http://flybase.bio.indiana.edu/.bin/fbidq.html?crol), [bin3](http://flybase.bio.indiana.edu/.bin/fbidq.html?bin3), [Rpd3](http://flybase.bio.indiana.edu/.bin/fbidq.html?Rpd3), [retn](http://flybase.bio.indiana.edu/.bin/fbidq.html?retn), [fs(1)h](http://flybase.bio.indiana.edu/.bin/fbidq.html?fs(1)h), [en](http://flybase.bio.indiana.edu/.bin/fbidq.html?en), [dsx](http://flybase.bio.indiana.edu/.bin/fbidq.html?dsx), [tey](http://flybase.bio.indiana.edu/.bin/fbidq.html?tey), [aret](http://flybase.bio.indiana.edu/.bin/fbidq.html?aret), [HLHmgamma](http://flybase.bio.indiana.edu/.bin/fbidq.html?HLHmgamma), [z](http://flybase.bio.indiana.edu/.bin/fbidq.html?z), [HLHm7](http://flybase.bio.indiana.edu/.bin/fbidq.html?HLHm7), [Lim1](http://flybase.bio.indiana.edu/.bin/fbidq.html?Lim1), [dnr1](http://flybase.bio.indiana.edu/.bin/fbidq.html?dnr1), [Ct](http://flybase.bio.indiana.edu/.bin/fbidq.html?Ct) |
| [negative regulation of cellular biosynthetic process](http://amigo.geneontology.org/cgi-bin/amigo/go.cgi?view=details&query=GO:0031327) | 77 of 1594 genes, 4.8% | 235 of 7634 genes, 3.1% | 0.00327 | 0.00% | 0.00 | [jing](http://flybase.bio.indiana.edu/.bin/fbidq.html?jing), [Sxl](http://flybase.bio.indiana.edu/.bin/fbidq.html?Sxl), [JIL-1](http://flybase.bio.indiana.edu/.bin/fbidq.html?JIL-1), [rn](http://flybase.bio.indiana.edu/.bin/fbidq.html?rn), [Iswi](http://flybase.bio.indiana.edu/.bin/fbidq.html?Iswi), [seq](http://flybase.bio.indiana.edu/.bin/fbidq.html?seq), [Aef1](http://flybase.bio.indiana.edu/.bin/fbidq.html?Aef1), [ovo](http://flybase.bio.indiana.edu/.bin/fbidq.html?ovo), [Dsp1](http://flybase.bio.indiana.edu/.bin/fbidq.html?Dsp1), [bi](http://flybase.bio.indiana.edu/.bin/fbidq.html?bi), [chn](http://flybase.bio.indiana.edu/.bin/fbidq.html?chn), [Gsc](http://flybase.bio.indiana.edu/.bin/fbidq.html?Gsc), [Nelf-A](http://flybase.bio.indiana.edu/.bin/fbidq.html?Nelf-A), [Pcl](http://flybase.bio.indiana.edu/.bin/fbidq.html?Pcl), [simj](http://flybase.bio.indiana.edu/.bin/fbidq.html?simj), [Sin3A](http://flybase.bio.indiana.edu/.bin/fbidq.html?Sin3A), [NC2alpha](http://flybase.bio.indiana.edu/.bin/fbidq.html?NC2alpha), [mip120](http://flybase.bio.indiana.edu/.bin/fbidq.html?mip120), [casp](http://flybase.bio.indiana.edu/.bin/fbidq.html?casp), [Snoo](http://flybase.bio.indiana.edu/.bin/fbidq.html?Snoo), [jumu](http://flybase.bio.indiana.edu/.bin/fbidq.html?jumu), [HGTX](http://flybase.bio.indiana.edu/.bin/fbidq.html?HGTX), [ush](http://flybase.bio.indiana.edu/.bin/fbidq.html?ush), [Usp7](http://flybase.bio.indiana.edu/.bin/fbidq.html?Usp7), [fd59A](http://flybase.bio.indiana.edu/.bin/fbidq.html?fd59A), [nab](http://flybase.bio.indiana.edu/.bin/fbidq.html?nab), [CG10336](http://flybase.bio.indiana.edu/.bin/fbidq.html?CG10336), [btd](http://flybase.bio.indiana.edu/.bin/fbidq.html?btd), [fd68A](http://flybase.bio.indiana.edu/.bin/fbidq.html?fd68A), [HLHmdelta](http://flybase.bio.indiana.edu/.bin/fbidq.html?HLHmdelta), [run](http://flybase.bio.indiana.edu/.bin/fbidq.html?run), [B-H1](http://flybase.bio.indiana.edu/.bin/fbidq.html?B-H1), [FBX011](http://flybase.bio.indiana.edu/.bin/fbidq.html?FBX011), [egg](http://flybase.bio.indiana.edu/.bin/fbidq.html?egg), [l(3)neo38](http://flybase.bio.indiana.edu/.bin/fbidq.html?l(3)neo38), [fd102C](http://flybase.bio.indiana.edu/.bin/fbidq.html?fd102C), [Ast-C](http://flybase.bio.indiana.edu/.bin/fbidq.html?Ast-C),[Vsx2](http://flybase.bio.indiana.edu/.bin/fbidq.html?Vsx2), [ph-p](http://flybase.bio.indiana.edu/.bin/fbidq.html?ph-p), [Ubx](http://flybase.bio.indiana.edu/.bin/fbidq.html?Ubx), [CG7757](http://flybase.bio.indiana.edu/.bin/fbidq.html?CG7757), [chm](http://flybase.bio.indiana.edu/.bin/fbidq.html?chm), [Sfmbt](http://flybase.bio.indiana.edu/.bin/fbidq.html?Sfmbt), [sna](http://flybase.bio.indiana.edu/.bin/fbidq.html?sna), [Wnt5](http://flybase.bio.indiana.edu/.bin/fbidq.html?Wnt5), [pan](http://flybase.bio.indiana.edu/.bin/fbidq.html?pan), [MBD-like](http://flybase.bio.indiana.edu/.bin/fbidq.html?MBD-like), [tsh](http://flybase.bio.indiana.edu/.bin/fbidq.html?tsh), [HLHm5](http://flybase.bio.indiana.edu/.bin/fbidq.html?HLHm5), [esg](http://flybase.bio.indiana.edu/.bin/fbidq.html?esg), [e(y)3](http://flybase.bio.indiana.edu/.bin/fbidq.html?e(y)3), [oc](http://flybase.bio.indiana.edu/.bin/fbidq.html?oc), [pros](http://flybase.bio.indiana.edu/.bin/fbidq.html?pros), [Gug](http://flybase.bio.indiana.edu/.bin/fbidq.html?Gug), [pad](http://flybase.bio.indiana.edu/.bin/fbidq.html?pad), [orb2](http://flybase.bio.indiana.edu/.bin/fbidq.html?orb2), [Wnt4](http://flybase.bio.indiana.edu/.bin/fbidq.html?Wnt4), [B-H2](http://flybase.bio.indiana.edu/.bin/fbidq.html?B-H2), [wg](http://flybase.bio.indiana.edu/.bin/fbidq.html?wg), [Vsx1](http://flybase.bio.indiana.edu/.bin/fbidq.html?Vsx1), [fkh](http://flybase.bio.indiana.edu/.bin/fbidq.html?fkh), [gro](http://flybase.bio.indiana.edu/.bin/fbidq.html?gro), [crol](http://flybase.bio.indiana.edu/.bin/fbidq.html?crol), [bin3](http://flybase.bio.indiana.edu/.bin/fbidq.html?bin3), [Rpd3](http://flybase.bio.indiana.edu/.bin/fbidq.html?Rpd3), [retn](http://flybase.bio.indiana.edu/.bin/fbidq.html?retn), [fs(1)h](http://flybase.bio.indiana.edu/.bin/fbidq.html?fs(1)h), [en](http://flybase.bio.indiana.edu/.bin/fbidq.html?en), [dsx](http://flybase.bio.indiana.edu/.bin/fbidq.html?dsx), [tey](http://flybase.bio.indiana.edu/.bin/fbidq.html?tey), [aret](http://flybase.bio.indiana.edu/.bin/fbidq.html?aret), [HLHmgamma](http://flybase.bio.indiana.edu/.bin/fbidq.html?HLHmgamma), [z](http://flybase.bio.indiana.edu/.bin/fbidq.html?z), [HLHm7](http://flybase.bio.indiana.edu/.bin/fbidq.html?HLHm7), [Lim1](http://flybase.bio.indiana.edu/.bin/fbidq.html?Lim1), [dnr1](http://flybase.bio.indiana.edu/.bin/fbidq.html?dnr1), [Ct](http://flybase.bio.indiana.edu/.bin/fbidq.html?Ct) |
| [dendrite development](http://amigo.geneontology.org/cgi-bin/amigo/go.cgi?view=details&query=GO:0016358) | 54 of 1594 genes, 3.4% | 148 of 7634 genes, 1.9% | 0.00337 | 0.00% | 0.00 | [robo](http://flybase.bio.indiana.edu/.bin/fbidq.html?robo), [futsch](http://flybase.bio.indiana.edu/.bin/fbidq.html?futsch), [brm](http://flybase.bio.indiana.edu/.bin/fbidq.html?brm), [acj6](http://flybase.bio.indiana.edu/.bin/fbidq.html?acj6), [Ptx1](http://flybase.bio.indiana.edu/.bin/fbidq.html?Ptx1), [kat-60L1](http://flybase.bio.indiana.edu/.bin/fbidq.html?kat-60L1), [wor](http://flybase.bio.indiana.edu/.bin/fbidq.html?wor), [vvl](http://flybase.bio.indiana.edu/.bin/fbidq.html?vvl), [Pten](http://flybase.bio.indiana.edu/.bin/fbidq.html?Pten), [chm](http://flybase.bio.indiana.edu/.bin/fbidq.html?chm), [sna](http://flybase.bio.indiana.edu/.bin/fbidq.html?sna), [ato](http://flybase.bio.indiana.edu/.bin/fbidq.html?ato), [Ptp69D](http://flybase.bio.indiana.edu/.bin/fbidq.html?Ptp69D), [gcm2](http://flybase.bio.indiana.edu/.bin/fbidq.html?gcm2), [Iswi](http://flybase.bio.indiana.edu/.bin/fbidq.html?Iswi), [seq](http://flybase.bio.indiana.edu/.bin/fbidq.html?seq), [gcm](http://flybase.bio.indiana.edu/.bin/fbidq.html?gcm), [Lis-1](http://flybase.bio.indiana.edu/.bin/fbidq.html?Lis-1), [ems](http://flybase.bio.indiana.edu/.bin/fbidq.html?ems), [dom](http://flybase.bio.indiana.edu/.bin/fbidq.html?dom), [pros](http://flybase.bio.indiana.edu/.bin/fbidq.html?pros), [tup](http://flybase.bio.indiana.edu/.bin/fbidq.html?tup), [comm](http://flybase.bio.indiana.edu/.bin/fbidq.html?comm), [NetB](http://flybase.bio.indiana.edu/.bin/fbidq.html?NetB), [Tango10](http://flybase.bio.indiana.edu/.bin/fbidq.html?Tango10), [rictor](http://flybase.bio.indiana.edu/.bin/fbidq.html?rictor), [Sin3A](http://flybase.bio.indiana.edu/.bin/fbidq.html?Sin3A), [d4](http://flybase.bio.indiana.edu/.bin/fbidq.html?d4), [CadN](http://flybase.bio.indiana.edu/.bin/fbidq.html?CadN), [gro](http://flybase.bio.indiana.edu/.bin/fbidq.html?gro), [nerfin-1](http://flybase.bio.indiana.edu/.bin/fbidq.html?nerfin-1), [CG4328](http://flybase.bio.indiana.edu/.bin/fbidq.html?CG4328), [E(Pc)](http://flybase.bio.indiana.edu/.bin/fbidq.html?E(Pc)), [jumu](http://flybase.bio.indiana.edu/.bin/fbidq.html?jumu), [Smox](http://flybase.bio.indiana.edu/.bin/fbidq.html?Smox), [Rpd3](http://flybase.bio.indiana.edu/.bin/fbidq.html?Rpd3), [Taf4](http://flybase.bio.indiana.edu/.bin/fbidq.html?Taf4), [nvy](http://flybase.bio.indiana.edu/.bin/fbidq.html?nvy),[E(bx)](http://flybase.bio.indiana.edu/.bin/fbidq.html?E(bx)), [Nrg](http://flybase.bio.indiana.edu/.bin/fbidq.html?Nrg), [chinmo](http://flybase.bio.indiana.edu/.bin/fbidq.html?chinmo), [run](http://flybase.bio.indiana.edu/.bin/fbidq.html?run), [CG10107](http://flybase.bio.indiana.edu/.bin/fbidq.html?CG10107), [sqz](http://flybase.bio.indiana.edu/.bin/fbidq.html?sqz), [Actbeta](http://flybase.bio.indiana.edu/.bin/fbidq.html?Actbeta), [Sema-1a](http://flybase.bio.indiana.edu/.bin/fbidq.html?Sema-1a), [CG33960](http://flybase.bio.indiana.edu/.bin/fbidq.html?CG33960), [pygo](http://flybase.bio.indiana.edu/.bin/fbidq.html?pygo), [HLHm7](http://flybase.bio.indiana.edu/.bin/fbidq.html?HLHm7), [stan](http://flybase.bio.indiana.edu/.bin/fbidq.html?stan), [Ulp1](http://flybase.bio.indiana.edu/.bin/fbidq.html?Ulp1), [tutl](http://flybase.bio.indiana.edu/.bin/fbidq.html?tutl), [Ct](http://flybase.bio.indiana.edu/.bin/fbidq.html?Ct), [ena](http://flybase.bio.indiana.edu/.bin/fbidq.html?ena) |
| [synaptic transmission](http://amigo.geneontology.org/cgi-bin/amigo/go.cgi?view=details&query=GO:0007268) | 79 of 1594 genes, 5.0% | 244 of 7634 genes, 3.2% | 0.00406 | 0.00% | 0.00 | [endoA](http://flybase.bio.indiana.edu/.bin/fbidq.html?endoA), [Sh](http://flybase.bio.indiana.edu/.bin/fbidq.html?Sh), [n-syb](http://flybase.bio.indiana.edu/.bin/fbidq.html?n-syb), [Atg1](http://flybase.bio.indiana.edu/.bin/fbidq.html?Atg1), [CG12187](http://flybase.bio.indiana.edu/.bin/fbidq.html?CG12187), [unc-13](http://flybase.bio.indiana.edu/.bin/fbidq.html?unc-13), [betaInt-nu](http://flybase.bio.indiana.edu/.bin/fbidq.html?betaInt-nu), [CG1909](http://flybase.bio.indiana.edu/.bin/fbidq.html?CG1909), [sgg](http://flybase.bio.indiana.edu/.bin/fbidq.html?sgg), [stj](http://flybase.bio.indiana.edu/.bin/fbidq.html?stj), [Dg](http://flybase.bio.indiana.edu/.bin/fbidq.html?Dg), [CanB](http://flybase.bio.indiana.edu/.bin/fbidq.html?CanB), [oa2](http://flybase.bio.indiana.edu/.bin/fbidq.html?oa2), [Pka-R1](http://flybase.bio.indiana.edu/.bin/fbidq.html?Pka-R1), [rab3-GEF](http://flybase.bio.indiana.edu/.bin/fbidq.html?rab3-GEF), [brp](http://flybase.bio.indiana.edu/.bin/fbidq.html?brp), [CASK](http://flybase.bio.indiana.edu/.bin/fbidq.html?CASK), [Sytalpha](http://flybase.bio.indiana.edu/.bin/fbidq.html?Sytalpha), [unc-104](http://flybase.bio.indiana.edu/.bin/fbidq.html?unc-104), [Khc-73](http://flybase.bio.indiana.edu/.bin/fbidq.html?Khc-73), [Syt4](http://flybase.bio.indiana.edu/.bin/fbidq.html?Syt4), [CG7708](http://flybase.bio.indiana.edu/.bin/fbidq.html?CG7708), [Dat](http://flybase.bio.indiana.edu/.bin/fbidq.html?Dat), [metro](http://flybase.bio.indiana.edu/.bin/fbidq.html?metro), [ewg](http://flybase.bio.indiana.edu/.bin/fbidq.html?ewg), [Cirl](http://flybase.bio.indiana.edu/.bin/fbidq.html?Cirl), [cpx](http://flybase.bio.indiana.edu/.bin/fbidq.html?cpx), [scrib](http://flybase.bio.indiana.edu/.bin/fbidq.html?scrib), [rut](http://flybase.bio.indiana.edu/.bin/fbidq.html?rut), [gfA](http://flybase.bio.indiana.edu/.bin/fbidq.html?gfA), [Fak56D](http://flybase.bio.indiana.edu/.bin/fbidq.html?Fak56D), [unc-13-4A](http://flybase.bio.indiana.edu/.bin/fbidq.html?unc-13-4A), [Rph](http://flybase.bio.indiana.edu/.bin/fbidq.html?Rph), [srpk79D](http://flybase.bio.indiana.edu/.bin/fbidq.html?srpk79D),[Rdl](http://flybase.bio.indiana.edu/.bin/fbidq.html?Rdl), [TBPH](http://flybase.bio.indiana.edu/.bin/fbidq.html?TBPH), [Snap25](http://flybase.bio.indiana.edu/.bin/fbidq.html?Snap25), [stan](http://flybase.bio.indiana.edu/.bin/fbidq.html?stan), [GABA-B-R2](http://flybase.bio.indiana.edu/.bin/fbidq.html?GABA-B-R2), [mGluRA](http://flybase.bio.indiana.edu/.bin/fbidq.html?mGluRA), [Fas2](http://flybase.bio.indiana.edu/.bin/fbidq.html?Fas2), [futsch](http://flybase.bio.indiana.edu/.bin/fbidq.html?futsch), [comt](http://flybase.bio.indiana.edu/.bin/fbidq.html?comt), [mtg](http://flybase.bio.indiana.edu/.bin/fbidq.html?mtg), [Frq1](http://flybase.bio.indiana.edu/.bin/fbidq.html?Frq1), [PP2A-B](http://flybase.bio.indiana.edu/.bin/fbidq.html?PP2A-B), [Rim](http://flybase.bio.indiana.edu/.bin/fbidq.html?Rim), [Shal](http://flybase.bio.indiana.edu/.bin/fbidq.html?Shal), [qvr](http://flybase.bio.indiana.edu/.bin/fbidq.html?qvr), [para](http://flybase.bio.indiana.edu/.bin/fbidq.html?para), [Syn2](http://flybase.bio.indiana.edu/.bin/fbidq.html?Syn2), [Nrx-1](http://flybase.bio.indiana.edu/.bin/fbidq.html?Nrx-1), [Cdk5](http://flybase.bio.indiana.edu/.bin/fbidq.html?Cdk5), [nmo](http://flybase.bio.indiana.edu/.bin/fbidq.html?nmo), [sif](http://flybase.bio.indiana.edu/.bin/fbidq.html?sif), [CG4641](http://flybase.bio.indiana.edu/.bin/fbidq.html?CG4641), [Rab3](http://flybase.bio.indiana.edu/.bin/fbidq.html?Rab3), [Tbh](http://flybase.bio.indiana.edu/.bin/fbidq.html?Tbh), [trio](http://flybase.bio.indiana.edu/.bin/fbidq.html?trio), [PIP5K59B](http://flybase.bio.indiana.edu/.bin/fbidq.html?PIP5K59B), [mr](http://flybase.bio.indiana.edu/.bin/fbidq.html?mr), [lap](http://flybase.bio.indiana.edu/.bin/fbidq.html?lap), [shi](http://flybase.bio.indiana.edu/.bin/fbidq.html?shi), [CG11155](http://flybase.bio.indiana.edu/.bin/fbidq.html?CG11155), [Gad1](http://flybase.bio.indiana.edu/.bin/fbidq.html?Gad1), [CG3822](http://flybase.bio.indiana.edu/.bin/fbidq.html?CG3822), [Vmat](http://flybase.bio.indiana.edu/.bin/fbidq.html?Vmat), [synaptogyrin](http://flybase.bio.indiana.edu/.bin/fbidq.html?synaptogyrin),[Cha](http://flybase.bio.indiana.edu/.bin/fbidq.html?Cha), [CaMKII](http://flybase.bio.indiana.edu/.bin/fbidq.html?CaMKII), [Aplip1](http://flybase.bio.indiana.edu/.bin/fbidq.html?Aplip1), [bchs](http://flybase.bio.indiana.edu/.bin/fbidq.html?bchs), [sfl](http://flybase.bio.indiana.edu/.bin/fbidq.html?sfl), [rl](http://flybase.bio.indiana.edu/.bin/fbidq.html?rl), [Syt1](http://flybase.bio.indiana.edu/.bin/fbidq.html?Syt1), [Syn1](http://flybase.bio.indiana.edu/.bin/fbidq.html?Syn1), [Snap](http://flybase.bio.indiana.edu/.bin/fbidq.html?Snap), [SerT](http://flybase.bio.indiana.edu/.bin/fbidq.html?SerT), [hiw](http://flybase.bio.indiana.edu/.bin/fbidq.html?hiw) |
| [eye morphogenesis](http://amigo.geneontology.org/cgi-bin/amigo/go.cgi?view=details&query=GO:0048592) | 76 of 1594 genes, 4.8% | 233 of 7634 genes, 3.1% | 0.00465 | 0.00% | 0.00 | [rg](http://flybase.bio.indiana.edu/.bin/fbidq.html?rg), [Pten](http://flybase.bio.indiana.edu/.bin/fbidq.html?Pten), [caup](http://flybase.bio.indiana.edu/.bin/fbidq.html?caup), [tll](http://flybase.bio.indiana.edu/.bin/fbidq.html?tll), [vimar](http://flybase.bio.indiana.edu/.bin/fbidq.html?vimar), [ato](http://flybase.bio.indiana.edu/.bin/fbidq.html?ato), [Dip3](http://flybase.bio.indiana.edu/.bin/fbidq.html?Dip3), [SP2353](http://flybase.bio.indiana.edu/.bin/fbidq.html?SP2353), [Cbl](http://flybase.bio.indiana.edu/.bin/fbidq.html?Cbl), [br](http://flybase.bio.indiana.edu/.bin/fbidq.html?br), [sns](http://flybase.bio.indiana.edu/.bin/fbidq.html?sns), [os](http://flybase.bio.indiana.edu/.bin/fbidq.html?os), [bi](http://flybase.bio.indiana.edu/.bin/fbidq.html?bi), [chn](http://flybase.bio.indiana.edu/.bin/fbidq.html?chn), [ds](http://flybase.bio.indiana.edu/.bin/fbidq.html?ds), [lola](http://flybase.bio.indiana.edu/.bin/fbidq.html?lola), [Alk](http://flybase.bio.indiana.edu/.bin/fbidq.html?Alk), [Vang](http://flybase.bio.indiana.edu/.bin/fbidq.html?Vang), [Nrk](http://flybase.bio.indiana.edu/.bin/fbidq.html?Nrk), [sano](http://flybase.bio.indiana.edu/.bin/fbidq.html?sano), [Galpha49B](http://flybase.bio.indiana.edu/.bin/fbidq.html?Galpha49B), [Lar](http://flybase.bio.indiana.edu/.bin/fbidq.html?Lar), [scrib](http://flybase.bio.indiana.edu/.bin/fbidq.html?scrib), [fred](http://flybase.bio.indiana.edu/.bin/fbidq.html?fred), [Gap1](http://flybase.bio.indiana.edu/.bin/fbidq.html?Gap1), [HLHmdelta](http://flybase.bio.indiana.edu/.bin/fbidq.html?HLHmdelta), [run](http://flybase.bio.indiana.edu/.bin/fbidq.html?run), [jeb](http://flybase.bio.indiana.edu/.bin/fbidq.html?jeb), [B-H1](http://flybase.bio.indiana.edu/.bin/fbidq.html?B-H1), [alph](http://flybase.bio.indiana.edu/.bin/fbidq.html?alph), [Hs3st-B](http://flybase.bio.indiana.edu/.bin/fbidq.html?Hs3st-B), [stan](http://flybase.bio.indiana.edu/.bin/fbidq.html?stan), [Sos](http://flybase.bio.indiana.edu/.bin/fbidq.html?Sos), [CG34400](http://flybase.bio.indiana.edu/.bin/fbidq.html?CG34400), [phyl](http://flybase.bio.indiana.edu/.bin/fbidq.html?phyl), [hth](http://flybase.bio.indiana.edu/.bin/fbidq.html?hth), [cindr](http://flybase.bio.indiana.edu/.bin/fbidq.html?cindr), [vg](http://flybase.bio.indiana.edu/.bin/fbidq.html?vg), [dsh](http://flybase.bio.indiana.edu/.bin/fbidq.html?dsh), [eya](http://flybase.bio.indiana.edu/.bin/fbidq.html?eya), [14-3-3zeta](http://flybase.bio.indiana.edu/.bin/fbidq.html?14-3-3zeta),[Fas2](http://flybase.bio.indiana.edu/.bin/fbidq.html?Fas2), [fz](http://flybase.bio.indiana.edu/.bin/fbidq.html?fz), [beat-Ia](http://flybase.bio.indiana.edu/.bin/fbidq.html?beat-Ia), [ft](http://flybase.bio.indiana.edu/.bin/fbidq.html?ft), [sno](http://flybase.bio.indiana.edu/.bin/fbidq.html?sno), [ara](http://flybase.bio.indiana.edu/.bin/fbidq.html?ara), [Syn2](http://flybase.bio.indiana.edu/.bin/fbidq.html?Syn2), [neur](http://flybase.bio.indiana.edu/.bin/fbidq.html?neur), [Lis-1](http://flybase.bio.indiana.edu/.bin/fbidq.html?Lis-1), [didum](http://flybase.bio.indiana.edu/.bin/fbidq.html?didum), [dac](http://flybase.bio.indiana.edu/.bin/fbidq.html?dac), [nmo](http://flybase.bio.indiana.edu/.bin/fbidq.html?nmo), [eys](http://flybase.bio.indiana.edu/.bin/fbidq.html?eys), [oc](http://flybase.bio.indiana.edu/.bin/fbidq.html?oc), [Mob2](http://flybase.bio.indiana.edu/.bin/fbidq.html?Mob2), [pros](http://flybase.bio.indiana.edu/.bin/fbidq.html?pros), [ey](http://flybase.bio.indiana.edu/.bin/fbidq.html?ey), [klar](http://flybase.bio.indiana.edu/.bin/fbidq.html?klar), [shi](http://flybase.bio.indiana.edu/.bin/fbidq.html?shi), [rst](http://flybase.bio.indiana.edu/.bin/fbidq.html?rst), [Wnt4](http://flybase.bio.indiana.edu/.bin/fbidq.html?Wnt4), [B-H2](http://flybase.bio.indiana.edu/.bin/fbidq.html?B-H2), [grh](http://flybase.bio.indiana.edu/.bin/fbidq.html?grh), [wg](http://flybase.bio.indiana.edu/.bin/fbidq.html?wg), [Apc](http://flybase.bio.indiana.edu/.bin/fbidq.html?Apc), [CadN](http://flybase.bio.indiana.edu/.bin/fbidq.html?CadN), [sd](http://flybase.bio.indiana.edu/.bin/fbidq.html?sd), [pk](http://flybase.bio.indiana.edu/.bin/fbidq.html?pk), [klg](http://flybase.bio.indiana.edu/.bin/fbidq.html?klg), [rl](http://flybase.bio.indiana.edu/.bin/fbidq.html?rl), [Snap](http://flybase.bio.indiana.edu/.bin/fbidq.html?Snap), [cdi](http://flybase.bio.indiana.edu/.bin/fbidq.html?cdi), [klu](http://flybase.bio.indiana.edu/.bin/fbidq.html?klu), [yrt](http://flybase.bio.indiana.edu/.bin/fbidq.html?yrt), [wls](http://flybase.bio.indiana.edu/.bin/fbidq.html?wls) |
| [regulation of multicellular organismal development](http://amigo.geneontology.org/cgi-bin/amigo/go.cgi?view=details&query=GO:2000026) | 90 of 1594 genes, 5.6% | 289 of 7634 genes, 3.8% | 0.00507 | 0.00% | 0.00 | [numb](http://flybase.bio.indiana.edu/.bin/fbidq.html?numb), [vvl](http://flybase.bio.indiana.edu/.bin/fbidq.html?vvl), [Pten](http://flybase.bio.indiana.edu/.bin/fbidq.html?Pten), [betaInt-nu](http://flybase.bio.indiana.edu/.bin/fbidq.html?betaInt-nu), [mira](http://flybase.bio.indiana.edu/.bin/fbidq.html?mira), [sgg](http://flybase.bio.indiana.edu/.bin/fbidq.html?sgg), [stj](http://flybase.bio.indiana.edu/.bin/fbidq.html?stj), [seq](http://flybase.bio.indiana.edu/.bin/fbidq.html?seq), [gcm](http://flybase.bio.indiana.edu/.bin/fbidq.html?gcm), [Dip3](http://flybase.bio.indiana.edu/.bin/fbidq.html?Dip3), [oa2](http://flybase.bio.indiana.edu/.bin/fbidq.html?oa2), [phl](http://flybase.bio.indiana.edu/.bin/fbidq.html?phl), [mago](http://flybase.bio.indiana.edu/.bin/fbidq.html?mago), [enc](http://flybase.bio.indiana.edu/.bin/fbidq.html?enc), [neo](http://flybase.bio.indiana.edu/.bin/fbidq.html?neo), [os](http://flybase.bio.indiana.edu/.bin/fbidq.html?os), [chn](http://flybase.bio.indiana.edu/.bin/fbidq.html?chn), [ds](http://flybase.bio.indiana.edu/.bin/fbidq.html?ds), [Traf6](http://flybase.bio.indiana.edu/.bin/fbidq.html?Traf6), [NetB](http://flybase.bio.indiana.edu/.bin/fbidq.html?NetB), [Vang](http://flybase.bio.indiana.edu/.bin/fbidq.html?Vang), [metro](http://flybase.bio.indiana.edu/.bin/fbidq.html?metro), [ewg](http://flybase.bio.indiana.edu/.bin/fbidq.html?ewg), [erm](http://flybase.bio.indiana.edu/.bin/fbidq.html?erm), [blue](http://flybase.bio.indiana.edu/.bin/fbidq.html?blue), [bab1](http://flybase.bio.indiana.edu/.bin/fbidq.html?bab1), [Galpha49B](http://flybase.bio.indiana.edu/.bin/fbidq.html?Galpha49B), [Lar](http://flybase.bio.indiana.edu/.bin/fbidq.html?Lar), [scrib](http://flybase.bio.indiana.edu/.bin/fbidq.html?scrib), [Fak56D](http://flybase.bio.indiana.edu/.bin/fbidq.html?Fak56D), [Gap1](http://flybase.bio.indiana.edu/.bin/fbidq.html?Gap1), [Ddc](http://flybase.bio.indiana.edu/.bin/fbidq.html?Ddc), [insc](http://flybase.bio.indiana.edu/.bin/fbidq.html?insc), [brat](http://flybase.bio.indiana.edu/.bin/fbidq.html?brat), [alph](http://flybase.bio.indiana.edu/.bin/fbidq.html?alph), [stau](http://flybase.bio.indiana.edu/.bin/fbidq.html?stau), [Sema-1a](http://flybase.bio.indiana.edu/.bin/fbidq.html?Sema-1a), [Eh](http://flybase.bio.indiana.edu/.bin/fbidq.html?Eh), [Hs3st-B](http://flybase.bio.indiana.edu/.bin/fbidq.html?Hs3st-B),[TBPH](http://flybase.bio.indiana.edu/.bin/fbidq.html?TBPH), [stan](http://flybase.bio.indiana.edu/.bin/fbidq.html?stan), [tutl](http://flybase.bio.indiana.edu/.bin/fbidq.html?tutl), [hth](http://flybase.bio.indiana.edu/.bin/fbidq.html?hth), [dsh](http://flybase.bio.indiana.edu/.bin/fbidq.html?dsh), [Fas2](http://flybase.bio.indiana.edu/.bin/fbidq.html?Fas2), [robo](http://flybase.bio.indiana.edu/.bin/fbidq.html?robo), [lea](http://flybase.bio.indiana.edu/.bin/fbidq.html?lea), [futsch](http://flybase.bio.indiana.edu/.bin/fbidq.html?futsch), [Ubx](http://flybase.bio.indiana.edu/.bin/fbidq.html?Ubx), [ft](http://flybase.bio.indiana.edu/.bin/fbidq.html?ft), [kat-60L1](http://flybase.bio.indiana.edu/.bin/fbidq.html?kat-60L1), [RhoGAPp190](http://flybase.bio.indiana.edu/.bin/fbidq.html?RhoGAPp190), [tsh](http://flybase.bio.indiana.edu/.bin/fbidq.html?tsh), [Syn2](http://flybase.bio.indiana.edu/.bin/fbidq.html?Syn2), [DAAM](http://flybase.bio.indiana.edu/.bin/fbidq.html?DAAM), [neur](http://flybase.bio.indiana.edu/.bin/fbidq.html?neur), [Lis-1](http://flybase.bio.indiana.edu/.bin/fbidq.html?Lis-1), [didum](http://flybase.bio.indiana.edu/.bin/fbidq.html?didum), [Cdk5](http://flybase.bio.indiana.edu/.bin/fbidq.html?Cdk5), [nmo](http://flybase.bio.indiana.edu/.bin/fbidq.html?nmo), [sif](http://flybase.bio.indiana.edu/.bin/fbidq.html?sif), [oc](http://flybase.bio.indiana.edu/.bin/fbidq.html?oc), [pros](http://flybase.bio.indiana.edu/.bin/fbidq.html?pros), [CG4641](http://flybase.bio.indiana.edu/.bin/fbidq.html?CG4641), [trio](http://flybase.bio.indiana.edu/.bin/fbidq.html?trio), [ed](http://flybase.bio.indiana.edu/.bin/fbidq.html?ed), [ssh](http://flybase.bio.indiana.edu/.bin/fbidq.html?ssh), [baz](http://flybase.bio.indiana.edu/.bin/fbidq.html?baz), [PIP5K59B](http://flybase.bio.indiana.edu/.bin/fbidq.html?PIP5K59B), [mr](http://flybase.bio.indiana.edu/.bin/fbidq.html?mr), [rst](http://flybase.bio.indiana.edu/.bin/fbidq.html?rst), [Wnt4](http://flybase.bio.indiana.edu/.bin/fbidq.html?Wnt4), [tyn](http://flybase.bio.indiana.edu/.bin/fbidq.html?tyn), [wg](http://flybase.bio.indiana.edu/.bin/fbidq.html?wg), [Apc](http://flybase.bio.indiana.edu/.bin/fbidq.html?Apc), [CadN](http://flybase.bio.indiana.edu/.bin/fbidq.html?CadN), [Rpd3](http://flybase.bio.indiana.edu/.bin/fbidq.html?Rpd3), [polo](http://flybase.bio.indiana.edu/.bin/fbidq.html?polo), [dsx](http://flybase.bio.indiana.edu/.bin/fbidq.html?dsx), [sfl](http://flybase.bio.indiana.edu/.bin/fbidq.html?sfl), [rl](http://flybase.bio.indiana.edu/.bin/fbidq.html?rl),[Syt1](http://flybase.bio.indiana.edu/.bin/fbidq.html?Syt1), [Syn1](http://flybase.bio.indiana.edu/.bin/fbidq.html?Syn1), [cdi](http://flybase.bio.indiana.edu/.bin/fbidq.html?cdi), [klu](http://flybase.bio.indiana.edu/.bin/fbidq.html?klu), [pburs](http://flybase.bio.indiana.edu/.bin/fbidq.html?pburs), [HLHmgamma](http://flybase.bio.indiana.edu/.bin/fbidq.html?HLHmgamma), [hiw](http://flybase.bio.indiana.edu/.bin/fbidq.html?hiw), [CG32653](http://flybase.bio.indiana.edu/.bin/fbidq.html?CG32653), [nkd](http://flybase.bio.indiana.edu/.bin/fbidq.html?nkd) |
| [male mating behavior](http://amigo.geneontology.org/cgi-bin/amigo/go.cgi?view=details&query=GO:0060179) | 21 of 1594 genes, 1.3% | 39 of 7634 genes, 0.5% | 0.00545 | 0.00% | 0.00 | [tipE](http://flybase.bio.indiana.edu/.bin/fbidq.html?tipE), [Poxn](http://flybase.bio.indiana.edu/.bin/fbidq.html?Poxn), [qtc](http://flybase.bio.indiana.edu/.bin/fbidq.html?qtc), [Pde1c](http://flybase.bio.indiana.edu/.bin/fbidq.html?Pde1c), [CaMKII](http://flybase.bio.indiana.edu/.bin/fbidq.html?CaMKII), [npf](http://flybase.bio.indiana.edu/.bin/fbidq.html?npf), [ato](http://flybase.bio.indiana.edu/.bin/fbidq.html?ato), [fne](http://flybase.bio.indiana.edu/.bin/fbidq.html?fne), [para](http://flybase.bio.indiana.edu/.bin/fbidq.html?para), [retn](http://flybase.bio.indiana.edu/.bin/fbidq.html?retn), [5-HT7](http://flybase.bio.indiana.edu/.bin/fbidq.html?5-HT7), [dsx](http://flybase.bio.indiana.edu/.bin/fbidq.html?dsx), [Nrg](http://flybase.bio.indiana.edu/.bin/fbidq.html?Nrg), [pros](http://flybase.bio.indiana.edu/.bin/fbidq.html?pros), [Tbh](http://flybase.bio.indiana.edu/.bin/fbidq.html?Tbh), [Oamb](http://flybase.bio.indiana.edu/.bin/fbidq.html?Oamb), [mle](http://flybase.bio.indiana.edu/.bin/fbidq.html?mle), [CASK](http://flybase.bio.indiana.edu/.bin/fbidq.html?CASK), [nonA](http://flybase.bio.indiana.edu/.bin/fbidq.html?nonA), [CG10251](http://flybase.bio.indiana.edu/.bin/fbidq.html?CG10251), [orb2](http://flybase.bio.indiana.edu/.bin/fbidq.html?orb2) |
| [imaginal disc-derived appendage development](http://amigo.geneontology.org/cgi-bin/amigo/go.cgi?view=details&query=GO:0048737) | 94 of 1594 genes, 5.9% | 306 of 7634 genes, 4.0% | 0.00583 | 0.00% | 0.00 | [fz2](http://flybase.bio.indiana.edu/.bin/fbidq.html?fz2), [tld](http://flybase.bio.indiana.edu/.bin/fbidq.html?tld), [tow](http://flybase.bio.indiana.edu/.bin/fbidq.html?tow), [skd](http://flybase.bio.indiana.edu/.bin/fbidq.html?skd), [jing](http://flybase.bio.indiana.edu/.bin/fbidq.html?jing), [caup](http://flybase.bio.indiana.edu/.bin/fbidq.html?caup), [betaInt-nu](http://flybase.bio.indiana.edu/.bin/fbidq.html?betaInt-nu), [ato](http://flybase.bio.indiana.edu/.bin/fbidq.html?ato), [sgg](http://flybase.bio.indiana.edu/.bin/fbidq.html?sgg), [Nmdar1](http://flybase.bio.indiana.edu/.bin/fbidq.html?Nmdar1), [bowl](http://flybase.bio.indiana.edu/.bin/fbidq.html?bowl), [rn](http://flybase.bio.indiana.edu/.bin/fbidq.html?rn), [ap](http://flybase.bio.indiana.edu/.bin/fbidq.html?ap), [Dg](http://flybase.bio.indiana.edu/.bin/fbidq.html?Dg), [Dip3](http://flybase.bio.indiana.edu/.bin/fbidq.html?Dip3), [CG14478](http://flybase.bio.indiana.edu/.bin/fbidq.html?CG14478), [phl](http://flybase.bio.indiana.edu/.bin/fbidq.html?phl), [Cbl](http://flybase.bio.indiana.edu/.bin/fbidq.html?Cbl), [ovo](http://flybase.bio.indiana.edu/.bin/fbidq.html?ovo), [ksr](http://flybase.bio.indiana.edu/.bin/fbidq.html?ksr), [Axn](http://flybase.bio.indiana.edu/.bin/fbidq.html?Axn), [tok](http://flybase.bio.indiana.edu/.bin/fbidq.html?tok), [bi](http://flybase.bio.indiana.edu/.bin/fbidq.html?bi), [ds](http://flybase.bio.indiana.edu/.bin/fbidq.html?ds), [CG15312](http://flybase.bio.indiana.edu/.bin/fbidq.html?CG15312), [Poxn](http://flybase.bio.indiana.edu/.bin/fbidq.html?Poxn), [pot](http://flybase.bio.indiana.edu/.bin/fbidq.html?pot), [bab1](http://flybase.bio.indiana.edu/.bin/fbidq.html?bab1), [jumu](http://flybase.bio.indiana.edu/.bin/fbidq.html?jumu), [Smox](http://flybase.bio.indiana.edu/.bin/fbidq.html?Smox), [scrib](http://flybase.bio.indiana.edu/.bin/fbidq.html?scrib), [Su(var)3-3](http://flybase.bio.indiana.edu/.bin/fbidq.html?Su(var)3-3), [MYPT-75D](http://flybase.bio.indiana.edu/.bin/fbidq.html?MYPT-75D), [Gap1](http://flybase.bio.indiana.edu/.bin/fbidq.html?Gap1), [btd](http://flybase.bio.indiana.edu/.bin/fbidq.html?btd), [alph](http://flybase.bio.indiana.edu/.bin/fbidq.html?alph), [Alh](http://flybase.bio.indiana.edu/.bin/fbidq.html?Alh), [Hs3st-B](http://flybase.bio.indiana.edu/.bin/fbidq.html?Hs3st-B), [l(3)neo38](http://flybase.bio.indiana.edu/.bin/fbidq.html?l(3)neo38),[Sos](http://flybase.bio.indiana.edu/.bin/fbidq.html?Sos), [hth](http://flybase.bio.indiana.edu/.bin/fbidq.html?hth), [vg](http://flybase.bio.indiana.edu/.bin/fbidq.html?vg), [dsh](http://flybase.bio.indiana.edu/.bin/fbidq.html?dsh), [Sp1](http://flybase.bio.indiana.edu/.bin/fbidq.html?Sp1), [fz](http://flybase.bio.indiana.edu/.bin/fbidq.html?fz), [CG8557](http://flybase.bio.indiana.edu/.bin/fbidq.html?CG8557), [brm](http://flybase.bio.indiana.edu/.bin/fbidq.html?brm), [Ubx](http://flybase.bio.indiana.edu/.bin/fbidq.html?Ubx), [ft](http://flybase.bio.indiana.edu/.bin/fbidq.html?ft), [CG42268](http://flybase.bio.indiana.edu/.bin/fbidq.html?CG42268), [lft](http://flybase.bio.indiana.edu/.bin/fbidq.html?lft), [sno](http://flybase.bio.indiana.edu/.bin/fbidq.html?sno), [ara](http://flybase.bio.indiana.edu/.bin/fbidq.html?ara), [mam](http://flybase.bio.indiana.edu/.bin/fbidq.html?mam), [pan](http://flybase.bio.indiana.edu/.bin/fbidq.html?pan), [Dll](http://flybase.bio.indiana.edu/.bin/fbidq.html?Dll), [tsh](http://flybase.bio.indiana.edu/.bin/fbidq.html?tsh), [mew](http://flybase.bio.indiana.edu/.bin/fbidq.html?mew), [neur](http://flybase.bio.indiana.edu/.bin/fbidq.html?neur), [e(y)3](http://flybase.bio.indiana.edu/.bin/fbidq.html?e(y)3), [dac](http://flybase.bio.indiana.edu/.bin/fbidq.html?dac), [nmo](http://flybase.bio.indiana.edu/.bin/fbidq.html?nmo), [Bx](http://flybase.bio.indiana.edu/.bin/fbidq.html?Bx), [CG32149](http://flybase.bio.indiana.edu/.bin/fbidq.html?CG32149), [trio](http://flybase.bio.indiana.edu/.bin/fbidq.html?trio), [ed](http://flybase.bio.indiana.edu/.bin/fbidq.html?ed), [Gug](http://flybase.bio.indiana.edu/.bin/fbidq.html?Gug), [CG33275](http://flybase.bio.indiana.edu/.bin/fbidq.html?CG33275), [Hr51](http://flybase.bio.indiana.edu/.bin/fbidq.html?Hr51), [siz](http://flybase.bio.indiana.edu/.bin/fbidq.html?siz), [CG6398](http://flybase.bio.indiana.edu/.bin/fbidq.html?CG6398), [Wnt4](http://flybase.bio.indiana.edu/.bin/fbidq.html?Wnt4), [salr](http://flybase.bio.indiana.edu/.bin/fbidq.html?salr), [wg](http://flybase.bio.indiana.edu/.bin/fbidq.html?wg), [Glut4EF](http://flybase.bio.indiana.edu/.bin/fbidq.html?Glut4EF), [crol](http://flybase.bio.indiana.edu/.bin/fbidq.html?crol), [sd](http://flybase.bio.indiana.edu/.bin/fbidq.html?sd), [Btk29A](http://flybase.bio.indiana.edu/.bin/fbidq.html?Btk29A), [fs(1)h](http://flybase.bio.indiana.edu/.bin/fbidq.html?fs(1)h), [pk](http://flybase.bio.indiana.edu/.bin/fbidq.html?pk), [sfl](http://flybase.bio.indiana.edu/.bin/fbidq.html?sfl), [rl](http://flybase.bio.indiana.edu/.bin/fbidq.html?rl),[chinmo](http://flybase.bio.indiana.edu/.bin/fbidq.html?chinmo), [Sip1](http://flybase.bio.indiana.edu/.bin/fbidq.html?Sip1), [Snap](http://flybase.bio.indiana.edu/.bin/fbidq.html?Snap), [trn](http://flybase.bio.indiana.edu/.bin/fbidq.html?trn), [aret](http://flybase.bio.indiana.edu/.bin/fbidq.html?aret), [wls](http://flybase.bio.indiana.edu/.bin/fbidq.html?wls), [pygo](http://flybase.bio.indiana.edu/.bin/fbidq.html?pygo), [unk](http://flybase.bio.indiana.edu/.bin/fbidq.html?unk), [Lim1](http://flybase.bio.indiana.edu/.bin/fbidq.html?Lim1), [cos](http://flybase.bio.indiana.edu/.bin/fbidq.html?cos), [Ct](http://flybase.bio.indiana.edu/.bin/fbidq.html?Ct), [dnr1](http://flybase.bio.indiana.edu/.bin/fbidq.html?dnr1) |
| [positive regulation of macromolecule metabolic process](http://amigo.geneontology.org/cgi-bin/amigo/go.cgi?view=details&query=GO:0010604) | 80 of 1594 genes, 5.0% | 250 of 7634 genes, 3.3% | 0.00585 | 0.00% | 0.00 | [acj6](http://flybase.bio.indiana.edu/.bin/fbidq.html?acj6), [wor](http://flybase.bio.indiana.edu/.bin/fbidq.html?wor), [vvl](http://flybase.bio.indiana.edu/.bin/fbidq.html?vvl), [caup](http://flybase.bio.indiana.edu/.bin/fbidq.html?caup), [tll](http://flybase.bio.indiana.edu/.bin/fbidq.html?tll), [Camta](http://flybase.bio.indiana.edu/.bin/fbidq.html?Camta), [ato](http://flybase.bio.indiana.edu/.bin/fbidq.html?ato), [tna](http://flybase.bio.indiana.edu/.bin/fbidq.html?tna), [Eip93F](http://flybase.bio.indiana.edu/.bin/fbidq.html?Eip93F), [JIL-1](http://flybase.bio.indiana.edu/.bin/fbidq.html?JIL-1), [ap](http://flybase.bio.indiana.edu/.bin/fbidq.html?ap), [Iswi](http://flybase.bio.indiana.edu/.bin/fbidq.html?Iswi), [Spt6](http://flybase.bio.indiana.edu/.bin/fbidq.html?Spt6), [gcm](http://flybase.bio.indiana.edu/.bin/fbidq.html?gcm), [Dip3](http://flybase.bio.indiana.edu/.bin/fbidq.html?Dip3), [ase](http://flybase.bio.indiana.edu/.bin/fbidq.html?ase), [rab3-GEF](http://flybase.bio.indiana.edu/.bin/fbidq.html?rab3-GEF), [br](http://flybase.bio.indiana.edu/.bin/fbidq.html?br), [ovo](http://flybase.bio.indiana.edu/.bin/fbidq.html?ovo), [Pdp1](http://flybase.bio.indiana.edu/.bin/fbidq.html?Pdp1), [chn](http://flybase.bio.indiana.edu/.bin/fbidq.html?chn), [Hcf](http://flybase.bio.indiana.edu/.bin/fbidq.html?Hcf), [Traf6](http://flybase.bio.indiana.edu/.bin/fbidq.html?Traf6), [lola](http://flybase.bio.indiana.edu/.bin/fbidq.html?lola), [Alk](http://flybase.bio.indiana.edu/.bin/fbidq.html?Alk), [fbl6](http://flybase.bio.indiana.edu/.bin/fbidq.html?fbl6), [NC2alpha](http://flybase.bio.indiana.edu/.bin/fbidq.html?NC2alpha), [Taf4](http://flybase.bio.indiana.edu/.bin/fbidq.html?Taf4), [HGTX](http://flybase.bio.indiana.edu/.bin/fbidq.html?HGTX), [fd59A](http://flybase.bio.indiana.edu/.bin/fbidq.html?fd59A), [btd](http://flybase.bio.indiana.edu/.bin/fbidq.html?btd), [run](http://flybase.bio.indiana.edu/.bin/fbidq.html?run), [Antp](http://flybase.bio.indiana.edu/.bin/fbidq.html?Antp), [ATbp](http://flybase.bio.indiana.edu/.bin/fbidq.html?ATbp), [7B2](http://flybase.bio.indiana.edu/.bin/fbidq.html?7B2), [tara](http://flybase.bio.indiana.edu/.bin/fbidq.html?tara), [stau](http://flybase.bio.indiana.edu/.bin/fbidq.html?stau), [Fer2](http://flybase.bio.indiana.edu/.bin/fbidq.html?Fer2), [hth](http://flybase.bio.indiana.edu/.bin/fbidq.html?hth), [vg](http://flybase.bio.indiana.edu/.bin/fbidq.html?vg), [eya](http://flybase.bio.indiana.edu/.bin/fbidq.html?eya), [brm](http://flybase.bio.indiana.edu/.bin/fbidq.html?brm), [onecut](http://flybase.bio.indiana.edu/.bin/fbidq.html?onecut),[sno](http://flybase.bio.indiana.edu/.bin/fbidq.html?sno), [chm](http://flybase.bio.indiana.edu/.bin/fbidq.html?chm), [ara](http://flybase.bio.indiana.edu/.bin/fbidq.html?ara), [sna](http://flybase.bio.indiana.edu/.bin/fbidq.html?sna), [mam](http://flybase.bio.indiana.edu/.bin/fbidq.html?mam), [Taf1](http://flybase.bio.indiana.edu/.bin/fbidq.html?Taf1), [pan](http://flybase.bio.indiana.edu/.bin/fbidq.html?pan), [disco](http://flybase.bio.indiana.edu/.bin/fbidq.html?disco), [esg](http://flybase.bio.indiana.edu/.bin/fbidq.html?esg), [e(y)3](http://flybase.bio.indiana.edu/.bin/fbidq.html?e(y)3), [Brf](http://flybase.bio.indiana.edu/.bin/fbidq.html?Brf), [oc](http://flybase.bio.indiana.edu/.bin/fbidq.html?oc), [spt4](http://flybase.bio.indiana.edu/.bin/fbidq.html?spt4), [tup](http://flybase.bio.indiana.edu/.bin/fbidq.html?tup), [ey](http://flybase.bio.indiana.edu/.bin/fbidq.html?ey), [mle](http://flybase.bio.indiana.edu/.bin/fbidq.html?mle), [Bgb](http://flybase.bio.indiana.edu/.bin/fbidq.html?Bgb), [l(1)sc](http://flybase.bio.indiana.edu/.bin/fbidq.html?l(1)sc), [grh](http://flybase.bio.indiana.edu/.bin/fbidq.html?grh), [comm](http://flybase.bio.indiana.edu/.bin/fbidq.html?comm), [toy](http://flybase.bio.indiana.edu/.bin/fbidq.html?toy), [wg](http://flybase.bio.indiana.edu/.bin/fbidq.html?wg), [fkh](http://flybase.bio.indiana.edu/.bin/fbidq.html?fkh), [sim](http://flybase.bio.indiana.edu/.bin/fbidq.html?sim), [cort](http://flybase.bio.indiana.edu/.bin/fbidq.html?cort), [sd](http://flybase.bio.indiana.edu/.bin/fbidq.html?sd), [trx](http://flybase.bio.indiana.edu/.bin/fbidq.html?trx), [retn](http://flybase.bio.indiana.edu/.bin/fbidq.html?retn), [dsx](http://flybase.bio.indiana.edu/.bin/fbidq.html?dsx), [tyf](http://flybase.bio.indiana.edu/.bin/fbidq.html?tyf), [vfl](http://flybase.bio.indiana.edu/.bin/fbidq.html?vfl), [CG11347](http://flybase.bio.indiana.edu/.bin/fbidq.html?CG11347), [dalao](http://flybase.bio.indiana.edu/.bin/fbidq.html?dalao), [pygo](http://flybase.bio.indiana.edu/.bin/fbidq.html?pygo), [z](http://flybase.bio.indiana.edu/.bin/fbidq.html?z), [Lim1](http://flybase.bio.indiana.edu/.bin/fbidq.html?Lim1), [Atf-2](http://flybase.bio.indiana.edu/.bin/fbidq.html?Atf-2) |
| [transmission of nerve impulse](http://amigo.geneontology.org/cgi-bin/amigo/go.cgi?view=details&query=GO:0019226) | 80 of 1594 genes, 5.0% | 250 of 7634 genes, 3.3% | 0.00585 | 0.00% | 0.00 | [endoA](http://flybase.bio.indiana.edu/.bin/fbidq.html?endoA), [Sh](http://flybase.bio.indiana.edu/.bin/fbidq.html?Sh), [n-syb](http://flybase.bio.indiana.edu/.bin/fbidq.html?n-syb), [Atg1](http://flybase.bio.indiana.edu/.bin/fbidq.html?Atg1), [CG12187](http://flybase.bio.indiana.edu/.bin/fbidq.html?CG12187), [unc-13](http://flybase.bio.indiana.edu/.bin/fbidq.html?unc-13), [betaInt-nu](http://flybase.bio.indiana.edu/.bin/fbidq.html?betaInt-nu), [CG1909](http://flybase.bio.indiana.edu/.bin/fbidq.html?CG1909), [sgg](http://flybase.bio.indiana.edu/.bin/fbidq.html?sgg), [stj](http://flybase.bio.indiana.edu/.bin/fbidq.html?stj), [Dg](http://flybase.bio.indiana.edu/.bin/fbidq.html?Dg), [CanB](http://flybase.bio.indiana.edu/.bin/fbidq.html?CanB), [oa2](http://flybase.bio.indiana.edu/.bin/fbidq.html?oa2), [Pka-R1](http://flybase.bio.indiana.edu/.bin/fbidq.html?Pka-R1), [rab3-GEF](http://flybase.bio.indiana.edu/.bin/fbidq.html?rab3-GEF), [brp](http://flybase.bio.indiana.edu/.bin/fbidq.html?brp), [CASK](http://flybase.bio.indiana.edu/.bin/fbidq.html?CASK), [Sytalpha](http://flybase.bio.indiana.edu/.bin/fbidq.html?Sytalpha), [unc-104](http://flybase.bio.indiana.edu/.bin/fbidq.html?unc-104), [Khc-73](http://flybase.bio.indiana.edu/.bin/fbidq.html?Khc-73), [Syt4](http://flybase.bio.indiana.edu/.bin/fbidq.html?Syt4), [CG7708](http://flybase.bio.indiana.edu/.bin/fbidq.html?CG7708), [Dat](http://flybase.bio.indiana.edu/.bin/fbidq.html?Dat), [metro](http://flybase.bio.indiana.edu/.bin/fbidq.html?metro), [ewg](http://flybase.bio.indiana.edu/.bin/fbidq.html?ewg), [Cirl](http://flybase.bio.indiana.edu/.bin/fbidq.html?Cirl), [cpx](http://flybase.bio.indiana.edu/.bin/fbidq.html?cpx), [scrib](http://flybase.bio.indiana.edu/.bin/fbidq.html?scrib), [rut](http://flybase.bio.indiana.edu/.bin/fbidq.html?rut), [gfA](http://flybase.bio.indiana.edu/.bin/fbidq.html?gfA), [Nrg](http://flybase.bio.indiana.edu/.bin/fbidq.html?Nrg), [Fak56D](http://flybase.bio.indiana.edu/.bin/fbidq.html?Fak56D), [unc-13-4A](http://flybase.bio.indiana.edu/.bin/fbidq.html?unc-13-4A), [Rph](http://flybase.bio.indiana.edu/.bin/fbidq.html?Rph),[srpk79D](http://flybase.bio.indiana.edu/.bin/fbidq.html?srpk79D), [Rdl](http://flybase.bio.indiana.edu/.bin/fbidq.html?Rdl), [TBPH](http://flybase.bio.indiana.edu/.bin/fbidq.html?TBPH), [Snap25](http://flybase.bio.indiana.edu/.bin/fbidq.html?Snap25), [stan](http://flybase.bio.indiana.edu/.bin/fbidq.html?stan), [GABA-B-R2](http://flybase.bio.indiana.edu/.bin/fbidq.html?GABA-B-R2), [mGluRA](http://flybase.bio.indiana.edu/.bin/fbidq.html?mGluRA), [Fas2](http://flybase.bio.indiana.edu/.bin/fbidq.html?Fas2), [futsch](http://flybase.bio.indiana.edu/.bin/fbidq.html?futsch), [comt](http://flybase.bio.indiana.edu/.bin/fbidq.html?comt), [mtg](http://flybase.bio.indiana.edu/.bin/fbidq.html?mtg), [Frq1](http://flybase.bio.indiana.edu/.bin/fbidq.html?Frq1), [PP2A-B](http://flybase.bio.indiana.edu/.bin/fbidq.html?PP2A-B), [Rim](http://flybase.bio.indiana.edu/.bin/fbidq.html?Rim), [Shal](http://flybase.bio.indiana.edu/.bin/fbidq.html?Shal), [qvr](http://flybase.bio.indiana.edu/.bin/fbidq.html?qvr), [para](http://flybase.bio.indiana.edu/.bin/fbidq.html?para), [Syn2](http://flybase.bio.indiana.edu/.bin/fbidq.html?Syn2), [Nrx-1](http://flybase.bio.indiana.edu/.bin/fbidq.html?Nrx-1), [Cdk5](http://flybase.bio.indiana.edu/.bin/fbidq.html?Cdk5), [nmo](http://flybase.bio.indiana.edu/.bin/fbidq.html?nmo), [sif](http://flybase.bio.indiana.edu/.bin/fbidq.html?sif), [CG4641](http://flybase.bio.indiana.edu/.bin/fbidq.html?CG4641), [Rab3](http://flybase.bio.indiana.edu/.bin/fbidq.html?Rab3), [Tbh](http://flybase.bio.indiana.edu/.bin/fbidq.html?Tbh), [trio](http://flybase.bio.indiana.edu/.bin/fbidq.html?trio), [PIP5K59B](http://flybase.bio.indiana.edu/.bin/fbidq.html?PIP5K59B), [mr](http://flybase.bio.indiana.edu/.bin/fbidq.html?mr), [lap](http://flybase.bio.indiana.edu/.bin/fbidq.html?lap), [shi](http://flybase.bio.indiana.edu/.bin/fbidq.html?shi), [CG11155](http://flybase.bio.indiana.edu/.bin/fbidq.html?CG11155), [Gad1](http://flybase.bio.indiana.edu/.bin/fbidq.html?Gad1), [CG3822](http://flybase.bio.indiana.edu/.bin/fbidq.html?CG3822), [Vmat](http://flybase.bio.indiana.edu/.bin/fbidq.html?Vmat),[synaptogyrin](http://flybase.bio.indiana.edu/.bin/fbidq.html?synaptogyrin), [Cha](http://flybase.bio.indiana.edu/.bin/fbidq.html?Cha), [CaMKII](http://flybase.bio.indiana.edu/.bin/fbidq.html?CaMKII), [Aplip1](http://flybase.bio.indiana.edu/.bin/fbidq.html?Aplip1), [bchs](http://flybase.bio.indiana.edu/.bin/fbidq.html?bchs), [sfl](http://flybase.bio.indiana.edu/.bin/fbidq.html?sfl), [rl](http://flybase.bio.indiana.edu/.bin/fbidq.html?rl), [Syt1](http://flybase.bio.indiana.edu/.bin/fbidq.html?Syt1), [Syn1](http://flybase.bio.indiana.edu/.bin/fbidq.html?Syn1), [Snap](http://flybase.bio.indiana.edu/.bin/fbidq.html?Snap), [SerT](http://flybase.bio.indiana.edu/.bin/fbidq.html?SerT), [hiw](http://flybase.bio.indiana.edu/.bin/fbidq.html?hiw) |
| [multicellular organismal signaling](http://amigo.geneontology.org/cgi-bin/amigo/go.cgi?view=details&query=GO:0035637) | 80 of 1594 genes, 5.0% | 250 of 7634 genes, 3.3% | 0.00585 | 0.00% | 0.00 | [endoA](http://flybase.bio.indiana.edu/.bin/fbidq.html?endoA), [Sh](http://flybase.bio.indiana.edu/.bin/fbidq.html?Sh), [n-syb](http://flybase.bio.indiana.edu/.bin/fbidq.html?n-syb), [Atg1](http://flybase.bio.indiana.edu/.bin/fbidq.html?Atg1), [CG12187](http://flybase.bio.indiana.edu/.bin/fbidq.html?CG12187), [unc-13](http://flybase.bio.indiana.edu/.bin/fbidq.html?unc-13), [betaInt-nu](http://flybase.bio.indiana.edu/.bin/fbidq.html?betaInt-nu), [CG1909](http://flybase.bio.indiana.edu/.bin/fbidq.html?CG1909), [sgg](http://flybase.bio.indiana.edu/.bin/fbidq.html?sgg), [stj](http://flybase.bio.indiana.edu/.bin/fbidq.html?stj), [Dg](http://flybase.bio.indiana.edu/.bin/fbidq.html?Dg), [CanB](http://flybase.bio.indiana.edu/.bin/fbidq.html?CanB), [oa2](http://flybase.bio.indiana.edu/.bin/fbidq.html?oa2), [Pka-R1](http://flybase.bio.indiana.edu/.bin/fbidq.html?Pka-R1), [rab3-GEF](http://flybase.bio.indiana.edu/.bin/fbidq.html?rab3-GEF), [brp](http://flybase.bio.indiana.edu/.bin/fbidq.html?brp), [CASK](http://flybase.bio.indiana.edu/.bin/fbidq.html?CASK), [Sytalpha](http://flybase.bio.indiana.edu/.bin/fbidq.html?Sytalpha), [unc-104](http://flybase.bio.indiana.edu/.bin/fbidq.html?unc-104), [Khc-73](http://flybase.bio.indiana.edu/.bin/fbidq.html?Khc-73), [Syt4](http://flybase.bio.indiana.edu/.bin/fbidq.html?Syt4), [CG7708](http://flybase.bio.indiana.edu/.bin/fbidq.html?CG7708), [Dat](http://flybase.bio.indiana.edu/.bin/fbidq.html?Dat), [metro](http://flybase.bio.indiana.edu/.bin/fbidq.html?metro), [ewg](http://flybase.bio.indiana.edu/.bin/fbidq.html?ewg), [Cirl](http://flybase.bio.indiana.edu/.bin/fbidq.html?Cirl), [cpx](http://flybase.bio.indiana.edu/.bin/fbidq.html?cpx), [scrib](http://flybase.bio.indiana.edu/.bin/fbidq.html?scrib), [rut](http://flybase.bio.indiana.edu/.bin/fbidq.html?rut), [gfA](http://flybase.bio.indiana.edu/.bin/fbidq.html?gfA), [Nrg](http://flybase.bio.indiana.edu/.bin/fbidq.html?Nrg), [Fak56D](http://flybase.bio.indiana.edu/.bin/fbidq.html?Fak56D), [unc-13-4A](http://flybase.bio.indiana.edu/.bin/fbidq.html?unc-13-4A), [Rph](http://flybase.bio.indiana.edu/.bin/fbidq.html?Rph),[srpk79D](http://flybase.bio.indiana.edu/.bin/fbidq.html?srpk79D), [Rdl](http://flybase.bio.indiana.edu/.bin/fbidq.html?Rdl), [TBPH](http://flybase.bio.indiana.edu/.bin/fbidq.html?TBPH), [Snap25](http://flybase.bio.indiana.edu/.bin/fbidq.html?Snap25), [stan](http://flybase.bio.indiana.edu/.bin/fbidq.html?stan), [GABA-B-R2](http://flybase.bio.indiana.edu/.bin/fbidq.html?GABA-B-R2), [mGluRA](http://flybase.bio.indiana.edu/.bin/fbidq.html?mGluRA), [Fas2](http://flybase.bio.indiana.edu/.bin/fbidq.html?Fas2), [futsch](http://flybase.bio.indiana.edu/.bin/fbidq.html?futsch), [comt](http://flybase.bio.indiana.edu/.bin/fbidq.html?comt), [mtg](http://flybase.bio.indiana.edu/.bin/fbidq.html?mtg), [Frq1](http://flybase.bio.indiana.edu/.bin/fbidq.html?Frq1), [PP2A-B](http://flybase.bio.indiana.edu/.bin/fbidq.html?PP2A-B), [Rim](http://flybase.bio.indiana.edu/.bin/fbidq.html?Rim), [Shal](http://flybase.bio.indiana.edu/.bin/fbidq.html?Shal), [qvr](http://flybase.bio.indiana.edu/.bin/fbidq.html?qvr), [para](http://flybase.bio.indiana.edu/.bin/fbidq.html?para), [Syn2](http://flybase.bio.indiana.edu/.bin/fbidq.html?Syn2), [Nrx-1](http://flybase.bio.indiana.edu/.bin/fbidq.html?Nrx-1), [Cdk5](http://flybase.bio.indiana.edu/.bin/fbidq.html?Cdk5), [nmo](http://flybase.bio.indiana.edu/.bin/fbidq.html?nmo), [sif](http://flybase.bio.indiana.edu/.bin/fbidq.html?sif), [CG4641](http://flybase.bio.indiana.edu/.bin/fbidq.html?CG4641), [Rab3](http://flybase.bio.indiana.edu/.bin/fbidq.html?Rab3), [Tbh](http://flybase.bio.indiana.edu/.bin/fbidq.html?Tbh), [trio](http://flybase.bio.indiana.edu/.bin/fbidq.html?trio), [PIP5K59B](http://flybase.bio.indiana.edu/.bin/fbidq.html?PIP5K59B), [mr](http://flybase.bio.indiana.edu/.bin/fbidq.html?mr), [lap](http://flybase.bio.indiana.edu/.bin/fbidq.html?lap), [shi](http://flybase.bio.indiana.edu/.bin/fbidq.html?shi), [CG11155](http://flybase.bio.indiana.edu/.bin/fbidq.html?CG11155), [Gad1](http://flybase.bio.indiana.edu/.bin/fbidq.html?Gad1), [CG3822](http://flybase.bio.indiana.edu/.bin/fbidq.html?CG3822), [Vmat](http://flybase.bio.indiana.edu/.bin/fbidq.html?Vmat),[synaptogyrin](http://flybase.bio.indiana.edu/.bin/fbidq.html?synaptogyrin), [Cha](http://flybase.bio.indiana.edu/.bin/fbidq.html?Cha), [CaMKII](http://flybase.bio.indiana.edu/.bin/fbidq.html?CaMKII), [Aplip1](http://flybase.bio.indiana.edu/.bin/fbidq.html?Aplip1), [bchs](http://flybase.bio.indiana.edu/.bin/fbidq.html?bchs), [sfl](http://flybase.bio.indiana.edu/.bin/fbidq.html?sfl), [rl](http://flybase.bio.indiana.edu/.bin/fbidq.html?rl), [Syt1](http://flybase.bio.indiana.edu/.bin/fbidq.html?Syt1), [Syn1](http://flybase.bio.indiana.edu/.bin/fbidq.html?Syn1), [Snap](http://flybase.bio.indiana.edu/.bin/fbidq.html?Snap), [SerT](http://flybase.bio.indiana.edu/.bin/fbidq.html?SerT), [hiw](http://flybase.bio.indiana.edu/.bin/fbidq.html?hiw) |
| [regulation of cell development](http://amigo.geneontology.org/cgi-bin/amigo/go.cgi?view=details&query=GO:0060284) | 65 of 1594 genes, 4.1% | 192 of 7634 genes, 2.5% | 0.00626 | 0.00% | 0.00 | [numb](http://flybase.bio.indiana.edu/.bin/fbidq.html?numb), [vvl](http://flybase.bio.indiana.edu/.bin/fbidq.html?vvl), [Pten](http://flybase.bio.indiana.edu/.bin/fbidq.html?Pten), [betaInt-nu](http://flybase.bio.indiana.edu/.bin/fbidq.html?betaInt-nu), [mira](http://flybase.bio.indiana.edu/.bin/fbidq.html?mira), [sgg](http://flybase.bio.indiana.edu/.bin/fbidq.html?sgg), [stj](http://flybase.bio.indiana.edu/.bin/fbidq.html?stj), [seq](http://flybase.bio.indiana.edu/.bin/fbidq.html?seq), [gcm](http://flybase.bio.indiana.edu/.bin/fbidq.html?gcm), [Dip3](http://flybase.bio.indiana.edu/.bin/fbidq.html?Dip3), [oa2](http://flybase.bio.indiana.edu/.bin/fbidq.html?oa2), [phl](http://flybase.bio.indiana.edu/.bin/fbidq.html?phl), [mago](http://flybase.bio.indiana.edu/.bin/fbidq.html?mago), [enc](http://flybase.bio.indiana.edu/.bin/fbidq.html?enc), [NetB](http://flybase.bio.indiana.edu/.bin/fbidq.html?NetB), [sals](http://flybase.bio.indiana.edu/.bin/fbidq.html?sals), [metro](http://flybase.bio.indiana.edu/.bin/fbidq.html?metro), [ewg](http://flybase.bio.indiana.edu/.bin/fbidq.html?ewg), [erm](http://flybase.bio.indiana.edu/.bin/fbidq.html?erm), [blue](http://flybase.bio.indiana.edu/.bin/fbidq.html?blue), [Galpha49B](http://flybase.bio.indiana.edu/.bin/fbidq.html?Galpha49B), [Lar](http://flybase.bio.indiana.edu/.bin/fbidq.html?Lar), [Fak56D](http://flybase.bio.indiana.edu/.bin/fbidq.html?Fak56D), [Gap1](http://flybase.bio.indiana.edu/.bin/fbidq.html?Gap1), [brat](http://flybase.bio.indiana.edu/.bin/fbidq.html?brat), [alph](http://flybase.bio.indiana.edu/.bin/fbidq.html?alph), [stau](http://flybase.bio.indiana.edu/.bin/fbidq.html?stau), [Sema-1a](http://flybase.bio.indiana.edu/.bin/fbidq.html?Sema-1a), [Hs3st-B](http://flybase.bio.indiana.edu/.bin/fbidq.html?Hs3st-B), [TBPH](http://flybase.bio.indiana.edu/.bin/fbidq.html?TBPH), [stan](http://flybase.bio.indiana.edu/.bin/fbidq.html?stan), [tutl](http://flybase.bio.indiana.edu/.bin/fbidq.html?tutl), [hth](http://flybase.bio.indiana.edu/.bin/fbidq.html?hth), [Fas2](http://flybase.bio.indiana.edu/.bin/fbidq.html?Fas2), [futsch](http://flybase.bio.indiana.edu/.bin/fbidq.html?futsch), [kat-60L1](http://flybase.bio.indiana.edu/.bin/fbidq.html?kat-60L1),[RhoGAPp190](http://flybase.bio.indiana.edu/.bin/fbidq.html?RhoGAPp190), [Syn2](http://flybase.bio.indiana.edu/.bin/fbidq.html?Syn2), [DAAM](http://flybase.bio.indiana.edu/.bin/fbidq.html?DAAM), [neur](http://flybase.bio.indiana.edu/.bin/fbidq.html?neur), [Lis-1](http://flybase.bio.indiana.edu/.bin/fbidq.html?Lis-1), [didum](http://flybase.bio.indiana.edu/.bin/fbidq.html?didum), [Cdk5](http://flybase.bio.indiana.edu/.bin/fbidq.html?Cdk5), [nmo](http://flybase.bio.indiana.edu/.bin/fbidq.html?nmo), [sif](http://flybase.bio.indiana.edu/.bin/fbidq.html?sif), [oc](http://flybase.bio.indiana.edu/.bin/fbidq.html?oc), [CG4641](http://flybase.bio.indiana.edu/.bin/fbidq.html?CG4641), [pros](http://flybase.bio.indiana.edu/.bin/fbidq.html?pros), [trio](http://flybase.bio.indiana.edu/.bin/fbidq.html?trio), [ed](http://flybase.bio.indiana.edu/.bin/fbidq.html?ed), [ssh](http://flybase.bio.indiana.edu/.bin/fbidq.html?ssh), [PIP5K59B](http://flybase.bio.indiana.edu/.bin/fbidq.html?PIP5K59B), [mr](http://flybase.bio.indiana.edu/.bin/fbidq.html?mr), [wg](http://flybase.bio.indiana.edu/.bin/fbidq.html?wg), [Apc](http://flybase.bio.indiana.edu/.bin/fbidq.html?Apc), [CadN](http://flybase.bio.indiana.edu/.bin/fbidq.html?CadN), [Rpd3](http://flybase.bio.indiana.edu/.bin/fbidq.html?Rpd3), [polo](http://flybase.bio.indiana.edu/.bin/fbidq.html?polo), [Syt1](http://flybase.bio.indiana.edu/.bin/fbidq.html?Syt1), [rl](http://flybase.bio.indiana.edu/.bin/fbidq.html?rl), [Syn1](http://flybase.bio.indiana.edu/.bin/fbidq.html?Syn1), [cdi](http://flybase.bio.indiana.edu/.bin/fbidq.html?cdi), [klu](http://flybase.bio.indiana.edu/.bin/fbidq.html?klu), [HLHmgamma](http://flybase.bio.indiana.edu/.bin/fbidq.html?HLHmgamma), [hiw](http://flybase.bio.indiana.edu/.bin/fbidq.html?hiw) |
| [instar larval or pupal morphogenesis](http://amigo.geneontology.org/cgi-bin/amigo/go.cgi?view=details&query=GO:0048707) | 121 of 1594 genes, 7.6% | 418 of 7634 genes, 5.5% | 0.00644 | 0.00% | 0.00 | [fz2](http://flybase.bio.indiana.edu/.bin/fbidq.html?fz2), [tld](http://flybase.bio.indiana.edu/.bin/fbidq.html?tld), [jar](http://flybase.bio.indiana.edu/.bin/fbidq.html?jar), [tow](http://flybase.bio.indiana.edu/.bin/fbidq.html?tow), [skd](http://flybase.bio.indiana.edu/.bin/fbidq.html?skd), [Atg1](http://flybase.bio.indiana.edu/.bin/fbidq.html?Atg1), [l(1)G0148](http://flybase.bio.indiana.edu/.bin/fbidq.html?l(1)G0148), [jing](http://flybase.bio.indiana.edu/.bin/fbidq.html?jing), [caup](http://flybase.bio.indiana.edu/.bin/fbidq.html?caup), [betaInt-nu](http://flybase.bio.indiana.edu/.bin/fbidq.html?betaInt-nu), [ato](http://flybase.bio.indiana.edu/.bin/fbidq.html?ato), [sgg](http://flybase.bio.indiana.edu/.bin/fbidq.html?sgg), [Nmdar1](http://flybase.bio.indiana.edu/.bin/fbidq.html?Nmdar1), [Eip93F](http://flybase.bio.indiana.edu/.bin/fbidq.html?Eip93F), [CG33007](http://flybase.bio.indiana.edu/.bin/fbidq.html?CG33007), [bowl](http://flybase.bio.indiana.edu/.bin/fbidq.html?bowl), [rn](http://flybase.bio.indiana.edu/.bin/fbidq.html?rn), [ap](http://flybase.bio.indiana.edu/.bin/fbidq.html?ap), [Dg](http://flybase.bio.indiana.edu/.bin/fbidq.html?Dg), [CG14478](http://flybase.bio.indiana.edu/.bin/fbidq.html?CG14478), [phl](http://flybase.bio.indiana.edu/.bin/fbidq.html?phl), [Cbl](http://flybase.bio.indiana.edu/.bin/fbidq.html?Cbl), [br](http://flybase.bio.indiana.edu/.bin/fbidq.html?br), [ovo](http://flybase.bio.indiana.edu/.bin/fbidq.html?ovo), [ksr](http://flybase.bio.indiana.edu/.bin/fbidq.html?ksr), [os](http://flybase.bio.indiana.edu/.bin/fbidq.html?os), [Axn](http://flybase.bio.indiana.edu/.bin/fbidq.html?Axn), [tok](http://flybase.bio.indiana.edu/.bin/fbidq.html?tok), [bi](http://flybase.bio.indiana.edu/.bin/fbidq.html?bi), [ds](http://flybase.bio.indiana.edu/.bin/fbidq.html?ds), [CG15312](http://flybase.bio.indiana.edu/.bin/fbidq.html?CG15312), [Poxn](http://flybase.bio.indiana.edu/.bin/fbidq.html?Poxn), [ewg](http://flybase.bio.indiana.edu/.bin/fbidq.html?ewg), [pot](http://flybase.bio.indiana.edu/.bin/fbidq.html?pot), [bab1](http://flybase.bio.indiana.edu/.bin/fbidq.html?bab1), [jumu](http://flybase.bio.indiana.edu/.bin/fbidq.html?jumu), [Smox](http://flybase.bio.indiana.edu/.bin/fbidq.html?Smox), [eIF4G2](http://flybase.bio.indiana.edu/.bin/fbidq.html?eIF4G2), [scrib](http://flybase.bio.indiana.edu/.bin/fbidq.html?scrib), [Su(var)3-3](http://flybase.bio.indiana.edu/.bin/fbidq.html?Su(var)3-3),[MYPT-75D](http://flybase.bio.indiana.edu/.bin/fbidq.html?MYPT-75D), [Nrg](http://flybase.bio.indiana.edu/.bin/fbidq.html?Nrg), [Ect3](http://flybase.bio.indiana.edu/.bin/fbidq.html?Ect3), [Gap1](http://flybase.bio.indiana.edu/.bin/fbidq.html?Gap1), [btd](http://flybase.bio.indiana.edu/.bin/fbidq.html?btd), [Mmp2](http://flybase.bio.indiana.edu/.bin/fbidq.html?Mmp2), [B-H1](http://flybase.bio.indiana.edu/.bin/fbidq.html?B-H1), [alph](http://flybase.bio.indiana.edu/.bin/fbidq.html?alph), [Eip71CD](http://flybase.bio.indiana.edu/.bin/fbidq.html?Eip71CD), [Hs3st-B](http://flybase.bio.indiana.edu/.bin/fbidq.html?Hs3st-B), [Traf4](http://flybase.bio.indiana.edu/.bin/fbidq.html?Traf4), [l(3)neo38](http://flybase.bio.indiana.edu/.bin/fbidq.html?l(3)neo38), [Sos](http://flybase.bio.indiana.edu/.bin/fbidq.html?Sos), [Sp1](http://flybase.bio.indiana.edu/.bin/fbidq.html?Sp1), [hth](http://flybase.bio.indiana.edu/.bin/fbidq.html?hth), [vg](http://flybase.bio.indiana.edu/.bin/fbidq.html?vg), [dsh](http://flybase.bio.indiana.edu/.bin/fbidq.html?dsh), [Fas2](http://flybase.bio.indiana.edu/.bin/fbidq.html?Fas2), [eya](http://flybase.bio.indiana.edu/.bin/fbidq.html?eya), [fz](http://flybase.bio.indiana.edu/.bin/fbidq.html?fz), [CG8557](http://flybase.bio.indiana.edu/.bin/fbidq.html?CG8557), [bsk](http://flybase.bio.indiana.edu/.bin/fbidq.html?bsk), [brm](http://flybase.bio.indiana.edu/.bin/fbidq.html?brm), [comm3](http://flybase.bio.indiana.edu/.bin/fbidq.html?comm3), [Ubx](http://flybase.bio.indiana.edu/.bin/fbidq.html?Ubx), [ft](http://flybase.bio.indiana.edu/.bin/fbidq.html?ft), [CG42268](http://flybase.bio.indiana.edu/.bin/fbidq.html?CG42268), [lft](http://flybase.bio.indiana.edu/.bin/fbidq.html?lft), [sno](http://flybase.bio.indiana.edu/.bin/fbidq.html?sno), [ara](http://flybase.bio.indiana.edu/.bin/fbidq.html?ara), [mam](http://flybase.bio.indiana.edu/.bin/fbidq.html?mam), [pan](http://flybase.bio.indiana.edu/.bin/fbidq.html?pan), [Dll](http://flybase.bio.indiana.edu/.bin/fbidq.html?Dll), [tsh](http://flybase.bio.indiana.edu/.bin/fbidq.html?tsh), [mew](http://flybase.bio.indiana.edu/.bin/fbidq.html?mew), [neur](http://flybase.bio.indiana.edu/.bin/fbidq.html?neur), [esg](http://flybase.bio.indiana.edu/.bin/fbidq.html?esg), [e(y)3](http://flybase.bio.indiana.edu/.bin/fbidq.html?e(y)3), [dac](http://flybase.bio.indiana.edu/.bin/fbidq.html?dac), [dap](http://flybase.bio.indiana.edu/.bin/fbidq.html?dap),[nmo](http://flybase.bio.indiana.edu/.bin/fbidq.html?nmo), [oc](http://flybase.bio.indiana.edu/.bin/fbidq.html?oc), [Bx](http://flybase.bio.indiana.edu/.bin/fbidq.html?Bx), [CG32149](http://flybase.bio.indiana.edu/.bin/fbidq.html?CG32149), [trio](http://flybase.bio.indiana.edu/.bin/fbidq.html?trio), [ey](http://flybase.bio.indiana.edu/.bin/fbidq.html?ey), [ed](http://flybase.bio.indiana.edu/.bin/fbidq.html?ed), [Gug](http://flybase.bio.indiana.edu/.bin/fbidq.html?Gug), [CG33275](http://flybase.bio.indiana.edu/.bin/fbidq.html?CG33275), [Hr51](http://flybase.bio.indiana.edu/.bin/fbidq.html?Hr51), [ImpE2](http://flybase.bio.indiana.edu/.bin/fbidq.html?ImpE2), [siz](http://flybase.bio.indiana.edu/.bin/fbidq.html?siz), [CG6398](http://flybase.bio.indiana.edu/.bin/fbidq.html?CG6398), [Wnt4](http://flybase.bio.indiana.edu/.bin/fbidq.html?Wnt4), [B-H2](http://flybase.bio.indiana.edu/.bin/fbidq.html?B-H2), [wg](http://flybase.bio.indiana.edu/.bin/fbidq.html?wg), [salr](http://flybase.bio.indiana.edu/.bin/fbidq.html?salr), [fkh](http://flybase.bio.indiana.edu/.bin/fbidq.html?fkh), [Glut4EF](http://flybase.bio.indiana.edu/.bin/fbidq.html?Glut4EF), [crol](http://flybase.bio.indiana.edu/.bin/fbidq.html?crol), [sd](http://flybase.bio.indiana.edu/.bin/fbidq.html?sd), [Btk29A](http://flybase.bio.indiana.edu/.bin/fbidq.html?Btk29A), [fs(1)h](http://flybase.bio.indiana.edu/.bin/fbidq.html?fs(1)h), [pk](http://flybase.bio.indiana.edu/.bin/fbidq.html?pk), [dsx](http://flybase.bio.indiana.edu/.bin/fbidq.html?dsx), [Eip63F-1](http://flybase.bio.indiana.edu/.bin/fbidq.html?Eip63F-1), [sfl](http://flybase.bio.indiana.edu/.bin/fbidq.html?sfl), [rl](http://flybase.bio.indiana.edu/.bin/fbidq.html?rl), [chinmo](http://flybase.bio.indiana.edu/.bin/fbidq.html?chinmo), [Sip1](http://flybase.bio.indiana.edu/.bin/fbidq.html?Sip1), [Snap](http://flybase.bio.indiana.edu/.bin/fbidq.html?Snap), [trn](http://flybase.bio.indiana.edu/.bin/fbidq.html?trn), [aret](http://flybase.bio.indiana.edu/.bin/fbidq.html?aret), [wls](http://flybase.bio.indiana.edu/.bin/fbidq.html?wls), [pygo](http://flybase.bio.indiana.edu/.bin/fbidq.html?pygo), [spag](http://flybase.bio.indiana.edu/.bin/fbidq.html?spag), [unk](http://flybase.bio.indiana.edu/.bin/fbidq.html?unk), [Lim1](http://flybase.bio.indiana.edu/.bin/fbidq.html?Lim1), [cos](http://flybase.bio.indiana.edu/.bin/fbidq.html?cos), [Ct](http://flybase.bio.indiana.edu/.bin/fbidq.html?Ct), [dnr1](http://flybase.bio.indiana.edu/.bin/fbidq.html?dnr1) |
| [neurogenesis](http://amigo.geneontology.org/cgi-bin/amigo/go.cgi?view=details&query=GO:0022008) | 270 of 1594 genes, 16.9% | 1075 of 7634 genes, 14.1% | 0.00680 | 0.00% | 0.00 | [fz2](http://flybase.bio.indiana.edu/.bin/fbidq.html?fz2), [Sh](http://flybase.bio.indiana.edu/.bin/fbidq.html?Sh), [numb](http://flybase.bio.indiana.edu/.bin/fbidq.html?numb), [dock](http://flybase.bio.indiana.edu/.bin/fbidq.html?dock), [CG5808](http://flybase.bio.indiana.edu/.bin/fbidq.html?CG5808), [CG9004](http://flybase.bio.indiana.edu/.bin/fbidq.html?CG9004), [CG42256](http://flybase.bio.indiana.edu/.bin/fbidq.html?CG42256), [CG3703](http://flybase.bio.indiana.edu/.bin/fbidq.html?CG3703), [ap](http://flybase.bio.indiana.edu/.bin/fbidq.html?ap), [Nf1](http://flybase.bio.indiana.edu/.bin/fbidq.html?Nf1), [Drep-3](http://flybase.bio.indiana.edu/.bin/fbidq.html?Drep-3), [os](http://flybase.bio.indiana.edu/.bin/fbidq.html?os), [tok](http://flybase.bio.indiana.edu/.bin/fbidq.html?tok), [Ptp99A](http://flybase.bio.indiana.edu/.bin/fbidq.html?Ptp99A), [CG31961](http://flybase.bio.indiana.edu/.bin/fbidq.html?CG31961), [NetB](http://flybase.bio.indiana.edu/.bin/fbidq.html?NetB), [Tango10](http://flybase.bio.indiana.edu/.bin/fbidq.html?Tango10), [Oseg4](http://flybase.bio.indiana.edu/.bin/fbidq.html?Oseg4), [cid](http://flybase.bio.indiana.edu/.bin/fbidq.html?cid), [CG32137](http://flybase.bio.indiana.edu/.bin/fbidq.html?CG32137), [lute](http://flybase.bio.indiana.edu/.bin/fbidq.html?lute), [Ssrp](http://flybase.bio.indiana.edu/.bin/fbidq.html?Ssrp), [Galpha49B](http://flybase.bio.indiana.edu/.bin/fbidq.html?Galpha49B), [Snoo](http://flybase.bio.indiana.edu/.bin/fbidq.html?Snoo), [Lar](http://flybase.bio.indiana.edu/.bin/fbidq.html?Lar), [scrib](http://flybase.bio.indiana.edu/.bin/fbidq.html?scrib), [HGTX](http://flybase.bio.indiana.edu/.bin/fbidq.html?HGTX), [not](http://flybase.bio.indiana.edu/.bin/fbidq.html?not), [rut](http://flybase.bio.indiana.edu/.bin/fbidq.html?rut), [nvy](http://flybase.bio.indiana.edu/.bin/fbidq.html?nvy), [nub](http://flybase.bio.indiana.edu/.bin/fbidq.html?nub), [nab](http://flybase.bio.indiana.edu/.bin/fbidq.html?nab), [HLHmdelta](http://flybase.bio.indiana.edu/.bin/fbidq.html?HLHmdelta), [B-H1](http://flybase.bio.indiana.edu/.bin/fbidq.html?B-H1), [pim](http://flybase.bio.indiana.edu/.bin/fbidq.html?pim),[alph](http://flybase.bio.indiana.edu/.bin/fbidq.html?alph), [TBPH](http://flybase.bio.indiana.edu/.bin/fbidq.html?TBPH), [Fer2](http://flybase.bio.indiana.edu/.bin/fbidq.html?Fer2), [CG34400](http://flybase.bio.indiana.edu/.bin/fbidq.html?CG34400), [Ulp1](http://flybase.bio.indiana.edu/.bin/fbidq.html?Ulp1), [ena](http://flybase.bio.indiana.edu/.bin/fbidq.html?ena), [dsh](http://flybase.bio.indiana.edu/.bin/fbidq.html?dsh), [hth](http://flybase.bio.indiana.edu/.bin/fbidq.html?hth), [pzg](http://flybase.bio.indiana.edu/.bin/fbidq.html?pzg), [Nf-YC](http://flybase.bio.indiana.edu/.bin/fbidq.html?Nf-YC), [14-3-3zeta](http://flybase.bio.indiana.edu/.bin/fbidq.html?14-3-3zeta), [lea](http://flybase.bio.indiana.edu/.bin/fbidq.html?lea), [qkr58E-1](http://flybase.bio.indiana.edu/.bin/fbidq.html?qkr58E-1), [plexB](http://flybase.bio.indiana.edu/.bin/fbidq.html?plexB), [Klp64D](http://flybase.bio.indiana.edu/.bin/fbidq.html?Klp64D), [DAAM](http://flybase.bio.indiana.edu/.bin/fbidq.html?DAAM), [neur](http://flybase.bio.indiana.edu/.bin/fbidq.html?neur), [CycE](http://flybase.bio.indiana.edu/.bin/fbidq.html?CycE), [Brf](http://flybase.bio.indiana.edu/.bin/fbidq.html?Brf), [ed](http://flybase.bio.indiana.edu/.bin/fbidq.html?ed), [CG11317](http://flybase.bio.indiana.edu/.bin/fbidq.html?CG11317), [daw](http://flybase.bio.indiana.edu/.bin/fbidq.html?daw), [Rx](http://flybase.bio.indiana.edu/.bin/fbidq.html?Rx), [salr](http://flybase.bio.indiana.edu/.bin/fbidq.html?salr), [synaptogyrin](http://flybase.bio.indiana.edu/.bin/fbidq.html?synaptogyrin), [Apc](http://flybase.bio.indiana.edu/.bin/fbidq.html?Apc), [sim](http://flybase.bio.indiana.edu/.bin/fbidq.html?sim), [d4](http://flybase.bio.indiana.edu/.bin/fbidq.html?d4), [gro](http://flybase.bio.indiana.edu/.bin/fbidq.html?gro), [wnd](http://flybase.bio.indiana.edu/.bin/fbidq.html?wnd), [trx](http://flybase.bio.indiana.edu/.bin/fbidq.html?trx), [Rpd3](http://flybase.bio.indiana.edu/.bin/fbidq.html?Rpd3), [SMC2](http://flybase.bio.indiana.edu/.bin/fbidq.html?SMC2), [unc-5](http://flybase.bio.indiana.edu/.bin/fbidq.html?unc-5), [cdi](http://flybase.bio.indiana.edu/.bin/fbidq.html?cdi), [CG10107](http://flybase.bio.indiana.edu/.bin/fbidq.html?CG10107), [barr](http://flybase.bio.indiana.edu/.bin/fbidq.html?barr),[pygo](http://flybase.bio.indiana.edu/.bin/fbidq.html?pygo), [HLHm7](http://flybase.bio.indiana.edu/.bin/fbidq.html?HLHm7), [HLHmgamma](http://flybase.bio.indiana.edu/.bin/fbidq.html?HLHmgamma), [jar](http://flybase.bio.indiana.edu/.bin/fbidq.html?jar), [wor](http://flybase.bio.indiana.edu/.bin/fbidq.html?wor), [Pten](http://flybase.bio.indiana.edu/.bin/fbidq.html?Pten), [Fcp1](http://flybase.bio.indiana.edu/.bin/fbidq.html?Fcp1), [mira](http://flybase.bio.indiana.edu/.bin/fbidq.html?mira), [Ptp69D](http://flybase.bio.indiana.edu/.bin/fbidq.html?Ptp69D), [hts](http://flybase.bio.indiana.edu/.bin/fbidq.html?hts), [pUf68](http://flybase.bio.indiana.edu/.bin/fbidq.html?pUf68), [seq](http://flybase.bio.indiana.edu/.bin/fbidq.html?seq), [Dip3](http://flybase.bio.indiana.edu/.bin/fbidq.html?Dip3), [Drep-2](http://flybase.bio.indiana.edu/.bin/fbidq.html?Drep-2), [RpS11](http://flybase.bio.indiana.edu/.bin/fbidq.html?RpS11), [phl](http://flybase.bio.indiana.edu/.bin/fbidq.html?phl), [CG1671](http://flybase.bio.indiana.edu/.bin/fbidq.html?CG1671), [Cbl](http://flybase.bio.indiana.edu/.bin/fbidq.html?Cbl), [CG4893](http://flybase.bio.indiana.edu/.bin/fbidq.html?CG4893), [CG9098](http://flybase.bio.indiana.edu/.bin/fbidq.html?CG9098), [Alk](http://flybase.bio.indiana.edu/.bin/fbidq.html?Alk), [esn](http://flybase.bio.indiana.edu/.bin/fbidq.html?esn), [Vang](http://flybase.bio.indiana.edu/.bin/fbidq.html?Vang), [Sin3A](http://flybase.bio.indiana.edu/.bin/fbidq.html?Sin3A), [pot](http://flybase.bio.indiana.edu/.bin/fbidq.html?pot), [jumu](http://flybase.bio.indiana.edu/.bin/fbidq.html?jumu), [Smox](http://flybase.bio.indiana.edu/.bin/fbidq.html?Smox), [Su(var)2-HP2](http://flybase.bio.indiana.edu/.bin/fbidq.html?Su(var)2-HP2), [CG31475](http://flybase.bio.indiana.edu/.bin/fbidq.html?CG31475), [fd59A](http://flybase.bio.indiana.edu/.bin/fbidq.html?fd59A), [beat-Ic](http://flybase.bio.indiana.edu/.bin/fbidq.html?beat-Ic), [brat](http://flybase.bio.indiana.edu/.bin/fbidq.html?brat), [Antp](http://flybase.bio.indiana.edu/.bin/fbidq.html?Antp),[futsch](http://flybase.bio.indiana.edu/.bin/fbidq.html?futsch), [Ptx1](http://flybase.bio.indiana.edu/.bin/fbidq.html?Ptx1), [gukh](http://flybase.bio.indiana.edu/.bin/fbidq.html?gukh), [chm](http://flybase.bio.indiana.edu/.bin/fbidq.html?chm), [Rim](http://flybase.bio.indiana.edu/.bin/fbidq.html?Rim), [beat-IIa](http://flybase.bio.indiana.edu/.bin/fbidq.html?beat-IIa), [Gpo-1](http://flybase.bio.indiana.edu/.bin/fbidq.html?Gpo-1), [sm](http://flybase.bio.indiana.edu/.bin/fbidq.html?sm), [Syn2](http://flybase.bio.indiana.edu/.bin/fbidq.html?Syn2), [CG42574](http://flybase.bio.indiana.edu/.bin/fbidq.html?CG42574), [eas](http://flybase.bio.indiana.edu/.bin/fbidq.html?eas), [dac](http://flybase.bio.indiana.edu/.bin/fbidq.html?dac), [Cdk5](http://flybase.bio.indiana.edu/.bin/fbidq.html?Cdk5), [oc](http://flybase.bio.indiana.edu/.bin/fbidq.html?oc), [CG3964](http://flybase.bio.indiana.edu/.bin/fbidq.html?CG3964), [ey](http://flybase.bio.indiana.edu/.bin/fbidq.html?ey), [baz](http://flybase.bio.indiana.edu/.bin/fbidq.html?baz), [rst](http://flybase.bio.indiana.edu/.bin/fbidq.html?rst), [l(1)sc](http://flybase.bio.indiana.edu/.bin/fbidq.html?l(1)sc), [comm](http://flybase.bio.indiana.edu/.bin/fbidq.html?comm), [grh](http://flybase.bio.indiana.edu/.bin/fbidq.html?grh), [Cdk5alpha](http://flybase.bio.indiana.edu/.bin/fbidq.html?Cdk5alpha), [Lim3](http://flybase.bio.indiana.edu/.bin/fbidq.html?Lim3), [Aplip1](http://flybase.bio.indiana.edu/.bin/fbidq.html?Aplip1), [E(Pc)](http://flybase.bio.indiana.edu/.bin/fbidq.html?E(Pc)), [CG1239](http://flybase.bio.indiana.edu/.bin/fbidq.html?CG1239), [retn](http://flybase.bio.indiana.edu/.bin/fbidq.html?retn), [rl](http://flybase.bio.indiana.edu/.bin/fbidq.html?rl), [Nup153](http://flybase.bio.indiana.edu/.bin/fbidq.html?Nup153), [tey](http://flybase.bio.indiana.edu/.bin/fbidq.html?tey), [yrt](http://flybase.bio.indiana.edu/.bin/fbidq.html?yrt), [CG33960](http://flybase.bio.indiana.edu/.bin/fbidq.html?CG33960), [Pabp2](http://flybase.bio.indiana.edu/.bin/fbidq.html?Pabp2), [acj6](http://flybase.bio.indiana.edu/.bin/fbidq.html?acj6), [jing](http://flybase.bio.indiana.edu/.bin/fbidq.html?jing), [tll](http://flybase.bio.indiana.edu/.bin/fbidq.html?tll), [Cbp80](http://flybase.bio.indiana.edu/.bin/fbidq.html?Cbp80), [ato](http://flybase.bio.indiana.edu/.bin/fbidq.html?ato),[vimar](http://flybase.bio.indiana.edu/.bin/fbidq.html?vimar), [Iswi](http://flybase.bio.indiana.edu/.bin/fbidq.html?Iswi), [Dg](http://flybase.bio.indiana.edu/.bin/fbidq.html?Dg), [CG5742](http://flybase.bio.indiana.edu/.bin/fbidq.html?CG5742), [Trim9](http://flybase.bio.indiana.edu/.bin/fbidq.html?Trim9), [CG34127](http://flybase.bio.indiana.edu/.bin/fbidq.html?CG34127), [CG7154](http://flybase.bio.indiana.edu/.bin/fbidq.html?CG7154), [br](http://flybase.bio.indiana.edu/.bin/fbidq.html?br), [robo3](http://flybase.bio.indiana.edu/.bin/fbidq.html?robo3), [lola](http://flybase.bio.indiana.edu/.bin/fbidq.html?lola), [unc-104](http://flybase.bio.indiana.edu/.bin/fbidq.html?unc-104), [chif](http://flybase.bio.indiana.edu/.bin/fbidq.html?chif), [Pcl](http://flybase.bio.indiana.edu/.bin/fbidq.html?Pcl), [p130CAS](http://flybase.bio.indiana.edu/.bin/fbidq.html?p130CAS), [CG7033](http://flybase.bio.indiana.edu/.bin/fbidq.html?CG7033), [Spc105R](http://flybase.bio.indiana.edu/.bin/fbidq.html?Spc105R), [CG31158](http://flybase.bio.indiana.edu/.bin/fbidq.html?CG31158), [Appl](http://flybase.bio.indiana.edu/.bin/fbidq.html?Appl), [CG13550](http://flybase.bio.indiana.edu/.bin/fbidq.html?CG13550), [CG30007](http://flybase.bio.indiana.edu/.bin/fbidq.html?CG30007), [metro](http://flybase.bio.indiana.edu/.bin/fbidq.html?metro), [Nrk](http://flybase.bio.indiana.edu/.bin/fbidq.html?Nrk), [erm](http://flybase.bio.indiana.edu/.bin/fbidq.html?erm), [CG4328](http://flybase.bio.indiana.edu/.bin/fbidq.html?CG4328), [CG10249](http://flybase.bio.indiana.edu/.bin/fbidq.html?CG10249), [Taf4](http://flybase.bio.indiana.edu/.bin/fbidq.html?Taf4), [l(2)NC136](http://flybase.bio.indiana.edu/.bin/fbidq.html?l(2)NC136), [gogo](http://flybase.bio.indiana.edu/.bin/fbidq.html?gogo), [Nrg](http://flybase.bio.indiana.edu/.bin/fbidq.html?Nrg), [insc](http://flybase.bio.indiana.edu/.bin/fbidq.html?insc), [jeb](http://flybase.bio.indiana.edu/.bin/fbidq.html?jeb),[GluClalpha](http://flybase.bio.indiana.edu/.bin/fbidq.html?GluClalpha), [Actbeta](http://flybase.bio.indiana.edu/.bin/fbidq.html?Actbeta), [beat-Ib](http://flybase.bio.indiana.edu/.bin/fbidq.html?beat-Ib), [Sema-1a](http://flybase.bio.indiana.edu/.bin/fbidq.html?Sema-1a), [stau](http://flybase.bio.indiana.edu/.bin/fbidq.html?stau), [Hs3st-B](http://flybase.bio.indiana.edu/.bin/fbidq.html?Hs3st-B), [otk](http://flybase.bio.indiana.edu/.bin/fbidq.html?otk), [Sos](http://flybase.bio.indiana.edu/.bin/fbidq.html?Sos), [phyl](http://flybase.bio.indiana.edu/.bin/fbidq.html?phyl), [eya](http://flybase.bio.indiana.edu/.bin/fbidq.html?eya), [Klp61F](http://flybase.bio.indiana.edu/.bin/fbidq.html?Klp61F), [Fas2](http://flybase.bio.indiana.edu/.bin/fbidq.html?Fas2), [bsk](http://flybase.bio.indiana.edu/.bin/fbidq.html?bsk), [brm](http://flybase.bio.indiana.edu/.bin/fbidq.html?brm), [robo](http://flybase.bio.indiana.edu/.bin/fbidq.html?robo), [fz](http://flybase.bio.indiana.edu/.bin/fbidq.html?fz), [beat-Ia](http://flybase.bio.indiana.edu/.bin/fbidq.html?beat-Ia), [kat-60L1](http://flybase.bio.indiana.edu/.bin/fbidq.html?kat-60L1), [CG12004](http://flybase.bio.indiana.edu/.bin/fbidq.html?CG12004), [RhoGAPp190](http://flybase.bio.indiana.edu/.bin/fbidq.html?RhoGAPp190), [hig](http://flybase.bio.indiana.edu/.bin/fbidq.html?hig), [pHCl](http://flybase.bio.indiana.edu/.bin/fbidq.html?pHCl), [grn](http://flybase.bio.indiana.edu/.bin/fbidq.html?grn), [mew](http://flybase.bio.indiana.edu/.bin/fbidq.html?mew), [Lis-1](http://flybase.bio.indiana.edu/.bin/fbidq.html?Lis-1), [didum](http://flybase.bio.indiana.edu/.bin/fbidq.html?didum), [ems](http://flybase.bio.indiana.edu/.bin/fbidq.html?ems), [eys](http://flybase.bio.indiana.edu/.bin/fbidq.html?eys), [tup](http://flybase.bio.indiana.edu/.bin/fbidq.html?tup), [pros](http://flybase.bio.indiana.edu/.bin/fbidq.html?pros), [Mob2](http://flybase.bio.indiana.edu/.bin/fbidq.html?Mob2), [pdm2](http://flybase.bio.indiana.edu/.bin/fbidq.html?pdm2), [trio](http://flybase.bio.indiana.edu/.bin/fbidq.html?trio), [CG1463](http://flybase.bio.indiana.edu/.bin/fbidq.html?CG1463),[Wnt4](http://flybase.bio.indiana.edu/.bin/fbidq.html?Wnt4), [rictor](http://flybase.bio.indiana.edu/.bin/fbidq.html?rictor), [nerfin-1](http://flybase.bio.indiana.edu/.bin/fbidq.html?nerfin-1), [bchs](http://flybase.bio.indiana.edu/.bin/fbidq.html?bchs), [RpS5b](http://flybase.bio.indiana.edu/.bin/fbidq.html?RpS5b), [polo](http://flybase.bio.indiana.edu/.bin/fbidq.html?polo), [dsx](http://flybase.bio.indiana.edu/.bin/fbidq.html?dsx), [chinmo](http://flybase.bio.indiana.edu/.bin/fbidq.html?chinmo), [Ssdp](http://flybase.bio.indiana.edu/.bin/fbidq.html?Ssdp), [sqz](http://flybase.bio.indiana.edu/.bin/fbidq.html?sqz), [klu](http://flybase.bio.indiana.edu/.bin/fbidq.html?klu), [fend](http://flybase.bio.indiana.edu/.bin/fbidq.html?fend), [Atet](http://flybase.bio.indiana.edu/.bin/fbidq.html?Atet), [nkd](http://flybase.bio.indiana.edu/.bin/fbidq.html?nkd), [vvl](http://flybase.bio.indiana.edu/.bin/fbidq.html?vvl), [Unc-76](http://flybase.bio.indiana.edu/.bin/fbidq.html?Unc-76), [gcm2](http://flybase.bio.indiana.edu/.bin/fbidq.html?gcm2), [mp](http://flybase.bio.indiana.edu/.bin/fbidq.html?mp), [gcm](http://flybase.bio.indiana.edu/.bin/fbidq.html?gcm), [CG5890](http://flybase.bio.indiana.edu/.bin/fbidq.html?CG5890), [SP2353](http://flybase.bio.indiana.edu/.bin/fbidq.html?SP2353), [Ccn](http://flybase.bio.indiana.edu/.bin/fbidq.html?Ccn), [ase](http://flybase.bio.indiana.edu/.bin/fbidq.html?ase), [CG32685](http://flybase.bio.indiana.edu/.bin/fbidq.html?CG32685), [Incenp](http://flybase.bio.indiana.edu/.bin/fbidq.html?Incenp), [Rat1](http://flybase.bio.indiana.edu/.bin/fbidq.html?Rat1), [CG18273](http://flybase.bio.indiana.edu/.bin/fbidq.html?CG18273), [Gap1](http://flybase.bio.indiana.edu/.bin/fbidq.html?Gap1), [run](http://flybase.bio.indiana.edu/.bin/fbidq.html?run), [ko](http://flybase.bio.indiana.edu/.bin/fbidq.html?ko), [Eip71CD](http://flybase.bio.indiana.edu/.bin/fbidq.html?Eip71CD), [stan](http://flybase.bio.indiana.edu/.bin/fbidq.html?stan), [CG12424](http://flybase.bio.indiana.edu/.bin/fbidq.html?CG12424), [tutl](http://flybase.bio.indiana.edu/.bin/fbidq.html?tutl), [ph-p](http://flybase.bio.indiana.edu/.bin/fbidq.html?ph-p), [sna](http://flybase.bio.indiana.edu/.bin/fbidq.html?sna),[Wnt5](http://flybase.bio.indiana.edu/.bin/fbidq.html?Wnt5), [Taf1](http://flybase.bio.indiana.edu/.bin/fbidq.html?Taf1), [SF2](http://flybase.bio.indiana.edu/.bin/fbidq.html?SF2), [Ggamma1](http://flybase.bio.indiana.edu/.bin/fbidq.html?Ggamma1), [CG7597](http://flybase.bio.indiana.edu/.bin/fbidq.html?CG7597), [CG5466](http://flybase.bio.indiana.edu/.bin/fbidq.html?CG5466), [esg](http://flybase.bio.indiana.edu/.bin/fbidq.html?esg), [sif](http://flybase.bio.indiana.edu/.bin/fbidq.html?sif), [dom](http://flybase.bio.indiana.edu/.bin/fbidq.html?dom), [Aac11](http://flybase.bio.indiana.edu/.bin/fbidq.html?Aac11), [ssh](http://flybase.bio.indiana.edu/.bin/fbidq.html?ssh), [klar](http://flybase.bio.indiana.edu/.bin/fbidq.html?klar), [mle](http://flybase.bio.indiana.edu/.bin/fbidq.html?mle), [Hr51](http://flybase.bio.indiana.edu/.bin/fbidq.html?Hr51), [CG11155](http://flybase.bio.indiana.edu/.bin/fbidq.html?CG11155), [B-H2](http://flybase.bio.indiana.edu/.bin/fbidq.html?B-H2), [wg](http://flybase.bio.indiana.edu/.bin/fbidq.html?wg), [CadN](http://flybase.bio.indiana.edu/.bin/fbidq.html?CadN), [Mfap1](http://flybase.bio.indiana.edu/.bin/fbidq.html?Mfap1), [RpL28](http://flybase.bio.indiana.edu/.bin/fbidq.html?RpL28), [E(bx)](http://flybase.bio.indiana.edu/.bin/fbidq.html?E(bx)), [en](http://flybase.bio.indiana.edu/.bin/fbidq.html?en), [klg](http://flybase.bio.indiana.edu/.bin/fbidq.html?klg), [Snap](http://flybase.bio.indiana.edu/.bin/fbidq.html?Snap), [trn](http://flybase.bio.indiana.edu/.bin/fbidq.html?trn), [Ct](http://flybase.bio.indiana.edu/.bin/fbidq.html?Ct) |
| [imaginal disc-derived appendage morphogenesis](http://amigo.geneontology.org/cgi-bin/amigo/go.cgi?view=details&query=GO:0035114) | 93 of 1594 genes, 5.8% | 303 of 7634 genes, 4.0% | 0.00686 | 0.00% | 0.00 | [fz2](http://flybase.bio.indiana.edu/.bin/fbidq.html?fz2), [tld](http://flybase.bio.indiana.edu/.bin/fbidq.html?tld), [tow](http://flybase.bio.indiana.edu/.bin/fbidq.html?tow), [skd](http://flybase.bio.indiana.edu/.bin/fbidq.html?skd), [jing](http://flybase.bio.indiana.edu/.bin/fbidq.html?jing), [caup](http://flybase.bio.indiana.edu/.bin/fbidq.html?caup), [betaInt-nu](http://flybase.bio.indiana.edu/.bin/fbidq.html?betaInt-nu), [ato](http://flybase.bio.indiana.edu/.bin/fbidq.html?ato), [sgg](http://flybase.bio.indiana.edu/.bin/fbidq.html?sgg), [Nmdar1](http://flybase.bio.indiana.edu/.bin/fbidq.html?Nmdar1), [bowl](http://flybase.bio.indiana.edu/.bin/fbidq.html?bowl), [rn](http://flybase.bio.indiana.edu/.bin/fbidq.html?rn), [ap](http://flybase.bio.indiana.edu/.bin/fbidq.html?ap), [Dg](http://flybase.bio.indiana.edu/.bin/fbidq.html?Dg), [Dip3](http://flybase.bio.indiana.edu/.bin/fbidq.html?Dip3), [CG14478](http://flybase.bio.indiana.edu/.bin/fbidq.html?CG14478), [phl](http://flybase.bio.indiana.edu/.bin/fbidq.html?phl), [Cbl](http://flybase.bio.indiana.edu/.bin/fbidq.html?Cbl), [ovo](http://flybase.bio.indiana.edu/.bin/fbidq.html?ovo), [ksr](http://flybase.bio.indiana.edu/.bin/fbidq.html?ksr), [Axn](http://flybase.bio.indiana.edu/.bin/fbidq.html?Axn), [tok](http://flybase.bio.indiana.edu/.bin/fbidq.html?tok), [bi](http://flybase.bio.indiana.edu/.bin/fbidq.html?bi), [ds](http://flybase.bio.indiana.edu/.bin/fbidq.html?ds), [CG15312](http://flybase.bio.indiana.edu/.bin/fbidq.html?CG15312), [Poxn](http://flybase.bio.indiana.edu/.bin/fbidq.html?Poxn), [pot](http://flybase.bio.indiana.edu/.bin/fbidq.html?pot), [bab1](http://flybase.bio.indiana.edu/.bin/fbidq.html?bab1), [jumu](http://flybase.bio.indiana.edu/.bin/fbidq.html?jumu), [Smox](http://flybase.bio.indiana.edu/.bin/fbidq.html?Smox), [scrib](http://flybase.bio.indiana.edu/.bin/fbidq.html?scrib), [Su(var)3-3](http://flybase.bio.indiana.edu/.bin/fbidq.html?Su(var)3-3), [MYPT-75D](http://flybase.bio.indiana.edu/.bin/fbidq.html?MYPT-75D), [Gap1](http://flybase.bio.indiana.edu/.bin/fbidq.html?Gap1), [btd](http://flybase.bio.indiana.edu/.bin/fbidq.html?btd), [alph](http://flybase.bio.indiana.edu/.bin/fbidq.html?alph), [Hs3st-B](http://flybase.bio.indiana.edu/.bin/fbidq.html?Hs3st-B), [l(3)neo38](http://flybase.bio.indiana.edu/.bin/fbidq.html?l(3)neo38), [Sos](http://flybase.bio.indiana.edu/.bin/fbidq.html?Sos),[hth](http://flybase.bio.indiana.edu/.bin/fbidq.html?hth), [vg](http://flybase.bio.indiana.edu/.bin/fbidq.html?vg), [dsh](http://flybase.bio.indiana.edu/.bin/fbidq.html?dsh), [Sp1](http://flybase.bio.indiana.edu/.bin/fbidq.html?Sp1), [fz](http://flybase.bio.indiana.edu/.bin/fbidq.html?fz), [CG8557](http://flybase.bio.indiana.edu/.bin/fbidq.html?CG8557), [brm](http://flybase.bio.indiana.edu/.bin/fbidq.html?brm), [Ubx](http://flybase.bio.indiana.edu/.bin/fbidq.html?Ubx), [ft](http://flybase.bio.indiana.edu/.bin/fbidq.html?ft), [CG42268](http://flybase.bio.indiana.edu/.bin/fbidq.html?CG42268), [lft](http://flybase.bio.indiana.edu/.bin/fbidq.html?lft), [sno](http://flybase.bio.indiana.edu/.bin/fbidq.html?sno), [ara](http://flybase.bio.indiana.edu/.bin/fbidq.html?ara), [mam](http://flybase.bio.indiana.edu/.bin/fbidq.html?mam), [pan](http://flybase.bio.indiana.edu/.bin/fbidq.html?pan), [Dll](http://flybase.bio.indiana.edu/.bin/fbidq.html?Dll), [tsh](http://flybase.bio.indiana.edu/.bin/fbidq.html?tsh), [mew](http://flybase.bio.indiana.edu/.bin/fbidq.html?mew), [neur](http://flybase.bio.indiana.edu/.bin/fbidq.html?neur), [e(y)3](http://flybase.bio.indiana.edu/.bin/fbidq.html?e(y)3), [dac](http://flybase.bio.indiana.edu/.bin/fbidq.html?dac), [nmo](http://flybase.bio.indiana.edu/.bin/fbidq.html?nmo), [Bx](http://flybase.bio.indiana.edu/.bin/fbidq.html?Bx), [CG32149](http://flybase.bio.indiana.edu/.bin/fbidq.html?CG32149), [trio](http://flybase.bio.indiana.edu/.bin/fbidq.html?trio), [ed](http://flybase.bio.indiana.edu/.bin/fbidq.html?ed), [Gug](http://flybase.bio.indiana.edu/.bin/fbidq.html?Gug), [CG33275](http://flybase.bio.indiana.edu/.bin/fbidq.html?CG33275), [Hr51](http://flybase.bio.indiana.edu/.bin/fbidq.html?Hr51), [siz](http://flybase.bio.indiana.edu/.bin/fbidq.html?siz), [CG6398](http://flybase.bio.indiana.edu/.bin/fbidq.html?CG6398), [Wnt4](http://flybase.bio.indiana.edu/.bin/fbidq.html?Wnt4), [salr](http://flybase.bio.indiana.edu/.bin/fbidq.html?salr), [wg](http://flybase.bio.indiana.edu/.bin/fbidq.html?wg), [Glut4EF](http://flybase.bio.indiana.edu/.bin/fbidq.html?Glut4EF), [crol](http://flybase.bio.indiana.edu/.bin/fbidq.html?crol), [sd](http://flybase.bio.indiana.edu/.bin/fbidq.html?sd), [Btk29A](http://flybase.bio.indiana.edu/.bin/fbidq.html?Btk29A), [fs(1)h](http://flybase.bio.indiana.edu/.bin/fbidq.html?fs(1)h), [pk](http://flybase.bio.indiana.edu/.bin/fbidq.html?pk), [sfl](http://flybase.bio.indiana.edu/.bin/fbidq.html?sfl), [rl](http://flybase.bio.indiana.edu/.bin/fbidq.html?rl), [chinmo](http://flybase.bio.indiana.edu/.bin/fbidq.html?chinmo),[Sip1](http://flybase.bio.indiana.edu/.bin/fbidq.html?Sip1), [Snap](http://flybase.bio.indiana.edu/.bin/fbidq.html?Snap), [trn](http://flybase.bio.indiana.edu/.bin/fbidq.html?trn), [aret](http://flybase.bio.indiana.edu/.bin/fbidq.html?aret), [wls](http://flybase.bio.indiana.edu/.bin/fbidq.html?wls), [pygo](http://flybase.bio.indiana.edu/.bin/fbidq.html?pygo), [unk](http://flybase.bio.indiana.edu/.bin/fbidq.html?unk), [Lim1](http://flybase.bio.indiana.edu/.bin/fbidq.html?Lim1), [cos](http://flybase.bio.indiana.edu/.bin/fbidq.html?cos), [Ct](http://flybase.bio.indiana.edu/.bin/fbidq.html?Ct), [dnr1](http://flybase.bio.indiana.edu/.bin/fbidq.html?dnr1) |
| [regulation of nervous system development](http://amigo.geneontology.org/cgi-bin/amigo/go.cgi?view=details&query=GO:0051960) | 55 of 1594 genes, 3.5% | 155 of 7634 genes, 2.0% | 0.00732 | 0.00% | 0.00 | [hth](http://flybase.bio.indiana.edu/.bin/fbidq.html?hth), [Fas2](http://flybase.bio.indiana.edu/.bin/fbidq.html?Fas2), [numb](http://flybase.bio.indiana.edu/.bin/fbidq.html?numb), [futsch](http://flybase.bio.indiana.edu/.bin/fbidq.html?futsch), [kat-60L1](http://flybase.bio.indiana.edu/.bin/fbidq.html?kat-60L1), [vvl](http://flybase.bio.indiana.edu/.bin/fbidq.html?vvl), [RhoGAPp190](http://flybase.bio.indiana.edu/.bin/fbidq.html?RhoGAPp190), [Pten](http://flybase.bio.indiana.edu/.bin/fbidq.html?Pten), [betaInt-nu](http://flybase.bio.indiana.edu/.bin/fbidq.html?betaInt-nu), [sgg](http://flybase.bio.indiana.edu/.bin/fbidq.html?sgg), [stj](http://flybase.bio.indiana.edu/.bin/fbidq.html?stj), [DAAM](http://flybase.bio.indiana.edu/.bin/fbidq.html?DAAM), [Syn2](http://flybase.bio.indiana.edu/.bin/fbidq.html?Syn2), [seq](http://flybase.bio.indiana.edu/.bin/fbidq.html?seq), [gcm](http://flybase.bio.indiana.edu/.bin/fbidq.html?gcm), [neur](http://flybase.bio.indiana.edu/.bin/fbidq.html?neur), [Lis-1](http://flybase.bio.indiana.edu/.bin/fbidq.html?Lis-1), [Dip3](http://flybase.bio.indiana.edu/.bin/fbidq.html?Dip3), [sif](http://flybase.bio.indiana.edu/.bin/fbidq.html?sif), [nmo](http://flybase.bio.indiana.edu/.bin/fbidq.html?nmo), [oa2](http://flybase.bio.indiana.edu/.bin/fbidq.html?oa2), [Cdk5](http://flybase.bio.indiana.edu/.bin/fbidq.html?Cdk5), [oc](http://flybase.bio.indiana.edu/.bin/fbidq.html?oc), [pros](http://flybase.bio.indiana.edu/.bin/fbidq.html?pros), [CG4641](http://flybase.bio.indiana.edu/.bin/fbidq.html?CG4641), [phl](http://flybase.bio.indiana.edu/.bin/fbidq.html?phl), [trio](http://flybase.bio.indiana.edu/.bin/fbidq.html?trio), [ssh](http://flybase.bio.indiana.edu/.bin/fbidq.html?ssh), [ed](http://flybase.bio.indiana.edu/.bin/fbidq.html?ed), [PIP5K59B](http://flybase.bio.indiana.edu/.bin/fbidq.html?PIP5K59B), [mr](http://flybase.bio.indiana.edu/.bin/fbidq.html?mr), [NetB](http://flybase.bio.indiana.edu/.bin/fbidq.html?NetB), [metro](http://flybase.bio.indiana.edu/.bin/fbidq.html?metro), [ewg](http://flybase.bio.indiana.edu/.bin/fbidq.html?ewg), [CadN](http://flybase.bio.indiana.edu/.bin/fbidq.html?CadN), [erm](http://flybase.bio.indiana.edu/.bin/fbidq.html?erm), [Rpd3](http://flybase.bio.indiana.edu/.bin/fbidq.html?Rpd3), [Lar](http://flybase.bio.indiana.edu/.bin/fbidq.html?Lar),[polo](http://flybase.bio.indiana.edu/.bin/fbidq.html?polo), [Fak56D](http://flybase.bio.indiana.edu/.bin/fbidq.html?Fak56D), [rl](http://flybase.bio.indiana.edu/.bin/fbidq.html?rl), [Gap1](http://flybase.bio.indiana.edu/.bin/fbidq.html?Gap1), [Syn1](http://flybase.bio.indiana.edu/.bin/fbidq.html?Syn1), [insc](http://flybase.bio.indiana.edu/.bin/fbidq.html?insc), [cdi](http://flybase.bio.indiana.edu/.bin/fbidq.html?cdi), [brat](http://flybase.bio.indiana.edu/.bin/fbidq.html?brat), [klu](http://flybase.bio.indiana.edu/.bin/fbidq.html?klu), [alph](http://flybase.bio.indiana.edu/.bin/fbidq.html?alph), [Sema-1a](http://flybase.bio.indiana.edu/.bin/fbidq.html?Sema-1a), [TBPH](http://flybase.bio.indiana.edu/.bin/fbidq.html?TBPH), [Hs3st-B](http://flybase.bio.indiana.edu/.bin/fbidq.html?Hs3st-B), [HLHmgamma](http://flybase.bio.indiana.edu/.bin/fbidq.html?HLHmgamma), [stan](http://flybase.bio.indiana.edu/.bin/fbidq.html?stan), [hiw](http://flybase.bio.indiana.edu/.bin/fbidq.html?hiw), [tutl](http://flybase.bio.indiana.edu/.bin/fbidq.html?tutl) |
| [male courtship behavior](http://amigo.geneontology.org/cgi-bin/amigo/go.cgi?view=details&query=GO:0008049) | 19 of 1594 genes, 1.2% | 34 of 7634 genes, 0.4% | 0.00792 | 0.00% | 0.00 | [tipE](http://flybase.bio.indiana.edu/.bin/fbidq.html?tipE), [Poxn](http://flybase.bio.indiana.edu/.bin/fbidq.html?Poxn), [qtc](http://flybase.bio.indiana.edu/.bin/fbidq.html?qtc), [CaMKII](http://flybase.bio.indiana.edu/.bin/fbidq.html?CaMKII), [npf](http://flybase.bio.indiana.edu/.bin/fbidq.html?npf), [ato](http://flybase.bio.indiana.edu/.bin/fbidq.html?ato), [fne](http://flybase.bio.indiana.edu/.bin/fbidq.html?fne), [para](http://flybase.bio.indiana.edu/.bin/fbidq.html?para), [5-HT7](http://flybase.bio.indiana.edu/.bin/fbidq.html?5-HT7), [retn](http://flybase.bio.indiana.edu/.bin/fbidq.html?retn), [dsx](http://flybase.bio.indiana.edu/.bin/fbidq.html?dsx), [Nrg](http://flybase.bio.indiana.edu/.bin/fbidq.html?Nrg), [Tbh](http://flybase.bio.indiana.edu/.bin/fbidq.html?Tbh), [pros](http://flybase.bio.indiana.edu/.bin/fbidq.html?pros), [Oamb](http://flybase.bio.indiana.edu/.bin/fbidq.html?Oamb), [mle](http://flybase.bio.indiana.edu/.bin/fbidq.html?mle), [CASK](http://flybase.bio.indiana.edu/.bin/fbidq.html?CASK), [nonA](http://flybase.bio.indiana.edu/.bin/fbidq.html?nonA), [orb2](http://flybase.bio.indiana.edu/.bin/fbidq.html?orb2) |
| [appendage development](http://amigo.geneontology.org/cgi-bin/amigo/go.cgi?view=details&query=GO:0048736) | 94 of 1594 genes, 5.9% | 308 of 7634 genes, 4.0% | 0.00794 | 0.00% | 0.00 | [fz2](http://flybase.bio.indiana.edu/.bin/fbidq.html?fz2), [tld](http://flybase.bio.indiana.edu/.bin/fbidq.html?tld), [tow](http://flybase.bio.indiana.edu/.bin/fbidq.html?tow), [skd](http://flybase.bio.indiana.edu/.bin/fbidq.html?skd), [jing](http://flybase.bio.indiana.edu/.bin/fbidq.html?jing), [caup](http://flybase.bio.indiana.edu/.bin/fbidq.html?caup), [betaInt-nu](http://flybase.bio.indiana.edu/.bin/fbidq.html?betaInt-nu), [ato](http://flybase.bio.indiana.edu/.bin/fbidq.html?ato), [sgg](http://flybase.bio.indiana.edu/.bin/fbidq.html?sgg), [Nmdar1](http://flybase.bio.indiana.edu/.bin/fbidq.html?Nmdar1), [bowl](http://flybase.bio.indiana.edu/.bin/fbidq.html?bowl), [rn](http://flybase.bio.indiana.edu/.bin/fbidq.html?rn), [ap](http://flybase.bio.indiana.edu/.bin/fbidq.html?ap), [Dg](http://flybase.bio.indiana.edu/.bin/fbidq.html?Dg), [Dip3](http://flybase.bio.indiana.edu/.bin/fbidq.html?Dip3), [CG14478](http://flybase.bio.indiana.edu/.bin/fbidq.html?CG14478), [phl](http://flybase.bio.indiana.edu/.bin/fbidq.html?phl), [Cbl](http://flybase.bio.indiana.edu/.bin/fbidq.html?Cbl), [ovo](http://flybase.bio.indiana.edu/.bin/fbidq.html?ovo), [ksr](http://flybase.bio.indiana.edu/.bin/fbidq.html?ksr), [Axn](http://flybase.bio.indiana.edu/.bin/fbidq.html?Axn), [tok](http://flybase.bio.indiana.edu/.bin/fbidq.html?tok), [bi](http://flybase.bio.indiana.edu/.bin/fbidq.html?bi), [ds](http://flybase.bio.indiana.edu/.bin/fbidq.html?ds), [CG15312](http://flybase.bio.indiana.edu/.bin/fbidq.html?CG15312), [Poxn](http://flybase.bio.indiana.edu/.bin/fbidq.html?Poxn), [pot](http://flybase.bio.indiana.edu/.bin/fbidq.html?pot), [bab1](http://flybase.bio.indiana.edu/.bin/fbidq.html?bab1), [jumu](http://flybase.bio.indiana.edu/.bin/fbidq.html?jumu), [Smox](http://flybase.bio.indiana.edu/.bin/fbidq.html?Smox), [scrib](http://flybase.bio.indiana.edu/.bin/fbidq.html?scrib), [Su(var)3-3](http://flybase.bio.indiana.edu/.bin/fbidq.html?Su(var)3-3), [MYPT-75D](http://flybase.bio.indiana.edu/.bin/fbidq.html?MYPT-75D), [Gap1](http://flybase.bio.indiana.edu/.bin/fbidq.html?Gap1), [btd](http://flybase.bio.indiana.edu/.bin/fbidq.html?btd), [alph](http://flybase.bio.indiana.edu/.bin/fbidq.html?alph), [Alh](http://flybase.bio.indiana.edu/.bin/fbidq.html?Alh), [Hs3st-B](http://flybase.bio.indiana.edu/.bin/fbidq.html?Hs3st-B), [l(3)neo38](http://flybase.bio.indiana.edu/.bin/fbidq.html?l(3)neo38),[Sos](http://flybase.bio.indiana.edu/.bin/fbidq.html?Sos), [hth](http://flybase.bio.indiana.edu/.bin/fbidq.html?hth), [vg](http://flybase.bio.indiana.edu/.bin/fbidq.html?vg), [dsh](http://flybase.bio.indiana.edu/.bin/fbidq.html?dsh), [Sp1](http://flybase.bio.indiana.edu/.bin/fbidq.html?Sp1), [fz](http://flybase.bio.indiana.edu/.bin/fbidq.html?fz), [CG8557](http://flybase.bio.indiana.edu/.bin/fbidq.html?CG8557), [brm](http://flybase.bio.indiana.edu/.bin/fbidq.html?brm), [Ubx](http://flybase.bio.indiana.edu/.bin/fbidq.html?Ubx), [ft](http://flybase.bio.indiana.edu/.bin/fbidq.html?ft), [CG42268](http://flybase.bio.indiana.edu/.bin/fbidq.html?CG42268), [lft](http://flybase.bio.indiana.edu/.bin/fbidq.html?lft), [sno](http://flybase.bio.indiana.edu/.bin/fbidq.html?sno), [ara](http://flybase.bio.indiana.edu/.bin/fbidq.html?ara), [mam](http://flybase.bio.indiana.edu/.bin/fbidq.html?mam), [pan](http://flybase.bio.indiana.edu/.bin/fbidq.html?pan), [Dll](http://flybase.bio.indiana.edu/.bin/fbidq.html?Dll), [tsh](http://flybase.bio.indiana.edu/.bin/fbidq.html?tsh), [mew](http://flybase.bio.indiana.edu/.bin/fbidq.html?mew), [neur](http://flybase.bio.indiana.edu/.bin/fbidq.html?neur), [e(y)3](http://flybase.bio.indiana.edu/.bin/fbidq.html?e(y)3), [dac](http://flybase.bio.indiana.edu/.bin/fbidq.html?dac), [nmo](http://flybase.bio.indiana.edu/.bin/fbidq.html?nmo), [Bx](http://flybase.bio.indiana.edu/.bin/fbidq.html?Bx), [CG32149](http://flybase.bio.indiana.edu/.bin/fbidq.html?CG32149), [trio](http://flybase.bio.indiana.edu/.bin/fbidq.html?trio), [ed](http://flybase.bio.indiana.edu/.bin/fbidq.html?ed), [Gug](http://flybase.bio.indiana.edu/.bin/fbidq.html?Gug), [CG33275](http://flybase.bio.indiana.edu/.bin/fbidq.html?CG33275), [Hr51](http://flybase.bio.indiana.edu/.bin/fbidq.html?Hr51), [siz](http://flybase.bio.indiana.edu/.bin/fbidq.html?siz), [CG6398](http://flybase.bio.indiana.edu/.bin/fbidq.html?CG6398), [Wnt4](http://flybase.bio.indiana.edu/.bin/fbidq.html?Wnt4), [salr](http://flybase.bio.indiana.edu/.bin/fbidq.html?salr), [wg](http://flybase.bio.indiana.edu/.bin/fbidq.html?wg), [Glut4EF](http://flybase.bio.indiana.edu/.bin/fbidq.html?Glut4EF), [crol](http://flybase.bio.indiana.edu/.bin/fbidq.html?crol), [sd](http://flybase.bio.indiana.edu/.bin/fbidq.html?sd), [Btk29A](http://flybase.bio.indiana.edu/.bin/fbidq.html?Btk29A), [fs(1)h](http://flybase.bio.indiana.edu/.bin/fbidq.html?fs(1)h), [pk](http://flybase.bio.indiana.edu/.bin/fbidq.html?pk), [sfl](http://flybase.bio.indiana.edu/.bin/fbidq.html?sfl), [rl](http://flybase.bio.indiana.edu/.bin/fbidq.html?rl),[chinmo](http://flybase.bio.indiana.edu/.bin/fbidq.html?chinmo), [Sip1](http://flybase.bio.indiana.edu/.bin/fbidq.html?Sip1), [Snap](http://flybase.bio.indiana.edu/.bin/fbidq.html?Snap), [trn](http://flybase.bio.indiana.edu/.bin/fbidq.html?trn), [aret](http://flybase.bio.indiana.edu/.bin/fbidq.html?aret), [wls](http://flybase.bio.indiana.edu/.bin/fbidq.html?wls), [pygo](http://flybase.bio.indiana.edu/.bin/fbidq.html?pygo), [unk](http://flybase.bio.indiana.edu/.bin/fbidq.html?unk), [Lim1](http://flybase.bio.indiana.edu/.bin/fbidq.html?Lim1), [cos](http://flybase.bio.indiana.edu/.bin/fbidq.html?cos), [Ct](http://flybase.bio.indiana.edu/.bin/fbidq.html?Ct), [dnr1](http://flybase.bio.indiana.edu/.bin/fbidq.html?dnr1) |
| [olfactory behavior](http://amigo.geneontology.org/cgi-bin/amigo/go.cgi?view=details&query=GO:0042048) | 34 of 1594 genes, 2.1% | 81 of 7634 genes, 1.1% | 0.00899 | 0.00% | 0.00 | [shi](http://flybase.bio.indiana.edu/.bin/fbidq.html?shi), [Gad1](http://flybase.bio.indiana.edu/.bin/fbidq.html?Gad1), [Fas2](http://flybase.bio.indiana.edu/.bin/fbidq.html?Fas2), [14-3-3zeta](http://flybase.bio.indiana.edu/.bin/fbidq.html?14-3-3zeta), [futsch](http://flybase.bio.indiana.edu/.bin/fbidq.html?futsch), [NaCP60E](http://flybase.bio.indiana.edu/.bin/fbidq.html?NaCP60E), [CG14509](http://flybase.bio.indiana.edu/.bin/fbidq.html?CG14509), [Alk](http://flybase.bio.indiana.edu/.bin/fbidq.html?Alk), [rogdi](http://flybase.bio.indiana.edu/.bin/fbidq.html?rogdi), [rg](http://flybase.bio.indiana.edu/.bin/fbidq.html?rg), [betaInt-nu](http://flybase.bio.indiana.edu/.bin/fbidq.html?betaInt-nu), [tau](http://flybase.bio.indiana.edu/.bin/fbidq.html?tau), [Spn](http://flybase.bio.indiana.edu/.bin/fbidq.html?Spn), [CG42629](http://flybase.bio.indiana.edu/.bin/fbidq.html?CG42629), [pigeon](http://flybase.bio.indiana.edu/.bin/fbidq.html?pigeon), [sgg](http://flybase.bio.indiana.edu/.bin/fbidq.html?sgg), [smi35A](http://flybase.bio.indiana.edu/.bin/fbidq.html?smi35A), [mnb](http://flybase.bio.indiana.edu/.bin/fbidq.html?mnb), [bin3](http://flybase.bio.indiana.edu/.bin/fbidq.html?bin3), [scrib](http://flybase.bio.indiana.edu/.bin/fbidq.html?scrib), [Dll](http://flybase.bio.indiana.edu/.bin/fbidq.html?Dll), [Nmdar1](http://flybase.bio.indiana.edu/.bin/fbidq.html?Nmdar1), [rut](http://flybase.bio.indiana.edu/.bin/fbidq.html?rut), [klg](http://flybase.bio.indiana.edu/.bin/fbidq.html?klg), [neur](http://flybase.bio.indiana.edu/.bin/fbidq.html?neur), [esg](http://flybase.bio.indiana.edu/.bin/fbidq.html?esg), [PQBP-1](http://flybase.bio.indiana.edu/.bin/fbidq.html?PQBP-1), [Sip1](http://flybase.bio.indiana.edu/.bin/fbidq.html?Sip1), [Nf1](http://flybase.bio.indiana.edu/.bin/fbidq.html?Nf1), [Pka-R1](http://flybase.bio.indiana.edu/.bin/fbidq.html?Pka-R1), [Mob2](http://flybase.bio.indiana.edu/.bin/fbidq.html?Mob2), [Rdl](http://flybase.bio.indiana.edu/.bin/fbidq.html?Rdl), [GABA-B-R2](http://flybase.bio.indiana.edu/.bin/fbidq.html?GABA-B-R2), [CG10251](http://flybase.bio.indiana.edu/.bin/fbidq.html?CG10251) |
| [appendage morphogenesis](http://amigo.geneontology.org/cgi-bin/amigo/go.cgi?view=details&query=GO:0035107) | 93 of 1594 genes, 5.8% | 305 of 7634 genes, 4.0% | 0.00934 | 0.00% | 0.00 | [fz2](http://flybase.bio.indiana.edu/.bin/fbidq.html?fz2), [tld](http://flybase.bio.indiana.edu/.bin/fbidq.html?tld), [tow](http://flybase.bio.indiana.edu/.bin/fbidq.html?tow), [skd](http://flybase.bio.indiana.edu/.bin/fbidq.html?skd), [jing](http://flybase.bio.indiana.edu/.bin/fbidq.html?jing), [caup](http://flybase.bio.indiana.edu/.bin/fbidq.html?caup), [betaInt-nu](http://flybase.bio.indiana.edu/.bin/fbidq.html?betaInt-nu), [ato](http://flybase.bio.indiana.edu/.bin/fbidq.html?ato), [sgg](http://flybase.bio.indiana.edu/.bin/fbidq.html?sgg), [Nmdar1](http://flybase.bio.indiana.edu/.bin/fbidq.html?Nmdar1), [bowl](http://flybase.bio.indiana.edu/.bin/fbidq.html?bowl), [rn](http://flybase.bio.indiana.edu/.bin/fbidq.html?rn), [ap](http://flybase.bio.indiana.edu/.bin/fbidq.html?ap), [Dg](http://flybase.bio.indiana.edu/.bin/fbidq.html?Dg), [Dip3](http://flybase.bio.indiana.edu/.bin/fbidq.html?Dip3), [CG14478](http://flybase.bio.indiana.edu/.bin/fbidq.html?CG14478), [phl](http://flybase.bio.indiana.edu/.bin/fbidq.html?phl), [Cbl](http://flybase.bio.indiana.edu/.bin/fbidq.html?Cbl), [ovo](http://flybase.bio.indiana.edu/.bin/fbidq.html?ovo), [ksr](http://flybase.bio.indiana.edu/.bin/fbidq.html?ksr), [Axn](http://flybase.bio.indiana.edu/.bin/fbidq.html?Axn), [tok](http://flybase.bio.indiana.edu/.bin/fbidq.html?tok), [bi](http://flybase.bio.indiana.edu/.bin/fbidq.html?bi), [ds](http://flybase.bio.indiana.edu/.bin/fbidq.html?ds), [CG15312](http://flybase.bio.indiana.edu/.bin/fbidq.html?CG15312), [Poxn](http://flybase.bio.indiana.edu/.bin/fbidq.html?Poxn), [pot](http://flybase.bio.indiana.edu/.bin/fbidq.html?pot), [bab1](http://flybase.bio.indiana.edu/.bin/fbidq.html?bab1), [jumu](http://flybase.bio.indiana.edu/.bin/fbidq.html?jumu), [Smox](http://flybase.bio.indiana.edu/.bin/fbidq.html?Smox), [scrib](http://flybase.bio.indiana.edu/.bin/fbidq.html?scrib), [Su(var)3-3](http://flybase.bio.indiana.edu/.bin/fbidq.html?Su(var)3-3), [MYPT-75D](http://flybase.bio.indiana.edu/.bin/fbidq.html?MYPT-75D), [Gap1](http://flybase.bio.indiana.edu/.bin/fbidq.html?Gap1), [btd](http://flybase.bio.indiana.edu/.bin/fbidq.html?btd), [alph](http://flybase.bio.indiana.edu/.bin/fbidq.html?alph), [Hs3st-B](http://flybase.bio.indiana.edu/.bin/fbidq.html?Hs3st-B), [l(3)neo38](http://flybase.bio.indiana.edu/.bin/fbidq.html?l(3)neo38), [Sos](http://flybase.bio.indiana.edu/.bin/fbidq.html?Sos),[hth](http://flybase.bio.indiana.edu/.bin/fbidq.html?hth), [vg](http://flybase.bio.indiana.edu/.bin/fbidq.html?vg), [dsh](http://flybase.bio.indiana.edu/.bin/fbidq.html?dsh), [Sp1](http://flybase.bio.indiana.edu/.bin/fbidq.html?Sp1), [fz](http://flybase.bio.indiana.edu/.bin/fbidq.html?fz), [CG8557](http://flybase.bio.indiana.edu/.bin/fbidq.html?CG8557), [brm](http://flybase.bio.indiana.edu/.bin/fbidq.html?brm), [Ubx](http://flybase.bio.indiana.edu/.bin/fbidq.html?Ubx), [ft](http://flybase.bio.indiana.edu/.bin/fbidq.html?ft), [CG42268](http://flybase.bio.indiana.edu/.bin/fbidq.html?CG42268), [lft](http://flybase.bio.indiana.edu/.bin/fbidq.html?lft), [sno](http://flybase.bio.indiana.edu/.bin/fbidq.html?sno), [ara](http://flybase.bio.indiana.edu/.bin/fbidq.html?ara), [mam](http://flybase.bio.indiana.edu/.bin/fbidq.html?mam), [pan](http://flybase.bio.indiana.edu/.bin/fbidq.html?pan), [Dll](http://flybase.bio.indiana.edu/.bin/fbidq.html?Dll), [tsh](http://flybase.bio.indiana.edu/.bin/fbidq.html?tsh), [mew](http://flybase.bio.indiana.edu/.bin/fbidq.html?mew), [neur](http://flybase.bio.indiana.edu/.bin/fbidq.html?neur), [e(y)3](http://flybase.bio.indiana.edu/.bin/fbidq.html?e(y)3), [dac](http://flybase.bio.indiana.edu/.bin/fbidq.html?dac), [nmo](http://flybase.bio.indiana.edu/.bin/fbidq.html?nmo), [Bx](http://flybase.bio.indiana.edu/.bin/fbidq.html?Bx), [CG32149](http://flybase.bio.indiana.edu/.bin/fbidq.html?CG32149), [trio](http://flybase.bio.indiana.edu/.bin/fbidq.html?trio), [ed](http://flybase.bio.indiana.edu/.bin/fbidq.html?ed), [Gug](http://flybase.bio.indiana.edu/.bin/fbidq.html?Gug), [CG33275](http://flybase.bio.indiana.edu/.bin/fbidq.html?CG33275), [Hr51](http://flybase.bio.indiana.edu/.bin/fbidq.html?Hr51), [siz](http://flybase.bio.indiana.edu/.bin/fbidq.html?siz), [CG6398](http://flybase.bio.indiana.edu/.bin/fbidq.html?CG6398), [Wnt4](http://flybase.bio.indiana.edu/.bin/fbidq.html?Wnt4), [salr](http://flybase.bio.indiana.edu/.bin/fbidq.html?salr), [wg](http://flybase.bio.indiana.edu/.bin/fbidq.html?wg), [Glut4EF](http://flybase.bio.indiana.edu/.bin/fbidq.html?Glut4EF), [crol](http://flybase.bio.indiana.edu/.bin/fbidq.html?crol), [sd](http://flybase.bio.indiana.edu/.bin/fbidq.html?sd), [Btk29A](http://flybase.bio.indiana.edu/.bin/fbidq.html?Btk29A), [fs(1)h](http://flybase.bio.indiana.edu/.bin/fbidq.html?fs(1)h), [pk](http://flybase.bio.indiana.edu/.bin/fbidq.html?pk), [sfl](http://flybase.bio.indiana.edu/.bin/fbidq.html?sfl), [rl](http://flybase.bio.indiana.edu/.bin/fbidq.html?rl), [chinmo](http://flybase.bio.indiana.edu/.bin/fbidq.html?chinmo),[Sip1](http://flybase.bio.indiana.edu/.bin/fbidq.html?Sip1), [Snap](http://flybase.bio.indiana.edu/.bin/fbidq.html?Snap), [trn](http://flybase.bio.indiana.edu/.bin/fbidq.html?trn), [aret](http://flybase.bio.indiana.edu/.bin/fbidq.html?aret), [wls](http://flybase.bio.indiana.edu/.bin/fbidq.html?wls), [pygo](http://flybase.bio.indiana.edu/.bin/fbidq.html?pygo), [unk](http://flybase.bio.indiana.edu/.bin/fbidq.html?unk), [Lim1](http://flybase.bio.indiana.edu/.bin/fbidq.html?Lim1), [cos](http://flybase.bio.indiana.edu/.bin/fbidq.html?cos), [Ct](http://flybase.bio.indiana.edu/.bin/fbidq.html?Ct), [dnr1](http://flybase.bio.indiana.edu/.bin/fbidq.html?dnr1) |
| [regulation of neurological system process](http://amigo.geneontology.org/cgi-bin/amigo/go.cgi?view=details&query=GO:0031644) | 22 of 1594 genes, 1.4% | 43 of 7634 genes, 0.6% | 0.00965 | 0.00% | 0.00 | [shi](http://flybase.bio.indiana.edu/.bin/fbidq.html?shi), [CG11155](http://flybase.bio.indiana.edu/.bin/fbidq.html?CG11155), [Sh](http://flybase.bio.indiana.edu/.bin/fbidq.html?Sh), [Syt4](http://flybase.bio.indiana.edu/.bin/fbidq.html?Syt4), [comt](http://flybase.bio.indiana.edu/.bin/fbidq.html?comt), [Frq1](http://flybase.bio.indiana.edu/.bin/fbidq.html?Frq1), [Alk](http://flybase.bio.indiana.edu/.bin/fbidq.html?Alk), [CG3822](http://flybase.bio.indiana.edu/.bin/fbidq.html?CG3822), [Atg1](http://flybase.bio.indiana.edu/.bin/fbidq.html?Atg1), [CG12187](http://flybase.bio.indiana.edu/.bin/fbidq.html?CG12187), [qvr](http://flybase.bio.indiana.edu/.bin/fbidq.html?qvr), [cpx](http://flybase.bio.indiana.edu/.bin/fbidq.html?cpx), [Dg](http://flybase.bio.indiana.edu/.bin/fbidq.html?Dg), [rl](http://flybase.bio.indiana.edu/.bin/fbidq.html?rl), [Syt1](http://flybase.bio.indiana.edu/.bin/fbidq.html?Syt1), [Rdl](http://flybase.bio.indiana.edu/.bin/fbidq.html?Rdl), [Snap25](http://flybase.bio.indiana.edu/.bin/fbidq.html?Snap25), [brp](http://flybase.bio.indiana.edu/.bin/fbidq.html?brp), [GABA-B-R2](http://flybase.bio.indiana.edu/.bin/fbidq.html?GABA-B-R2), [mr](http://flybase.bio.indiana.edu/.bin/fbidq.html?mr), [mGluRA](http://flybase.bio.indiana.edu/.bin/fbidq.html?mGluRA), [CASK](http://flybase.bio.indiana.edu/.bin/fbidq.html?CASK) |
| [formation of anatomical boundary](http://amigo.geneontology.org/cgi-bin/amigo/go.cgi?view=details&query=GO:0048859) | 23 of 1594 genes, 1.4% | 46 of 7634 genes, 0.6% | 0.00972 | 0.00% | 0.00 | [hth](http://flybase.bio.indiana.edu/.bin/fbidq.html?hth), [numb](http://flybase.bio.indiana.edu/.bin/fbidq.html?numb), [fz](http://flybase.bio.indiana.edu/.bin/fbidq.html?fz), [robo](http://flybase.bio.indiana.edu/.bin/fbidq.html?robo), [lea](http://flybase.bio.indiana.edu/.bin/fbidq.html?lea), [B-H2](http://flybase.bio.indiana.edu/.bin/fbidq.html?B-H2), [NetB](http://flybase.bio.indiana.edu/.bin/fbidq.html?NetB), [ft](http://flybase.bio.indiana.edu/.bin/fbidq.html?ft), [fkh](http://flybase.bio.indiana.edu/.bin/fbidq.html?fkh), [ara](http://flybase.bio.indiana.edu/.bin/fbidq.html?ara), [caup](http://flybase.bio.indiana.edu/.bin/fbidq.html?caup), [ato](http://flybase.bio.indiana.edu/.bin/fbidq.html?ato), [Brd](http://flybase.bio.indiana.edu/.bin/fbidq.html?Brd), [tsh](http://flybase.bio.indiana.edu/.bin/fbidq.html?tsh), [mew](http://flybase.bio.indiana.edu/.bin/fbidq.html?mew), [neur](http://flybase.bio.indiana.edu/.bin/fbidq.html?neur), [unc-5](http://flybase.bio.indiana.edu/.bin/fbidq.html?unc-5), [B-H1](http://flybase.bio.indiana.edu/.bin/fbidq.html?B-H1), [klu](http://flybase.bio.indiana.edu/.bin/fbidq.html?klu), [phyl](http://flybase.bio.indiana.edu/.bin/fbidq.html?phyl), [ds](http://flybase.bio.indiana.edu/.bin/fbidq.html?ds), [ena](http://flybase.bio.indiana.edu/.bin/fbidq.html?ena), [Ct](http://flybase.bio.indiana.edu/.bin/fbidq.html?Ct) |
