# Supplementary material for: Glial enriched gene expression profiling identifies novel factors regulating the proliferation of specific glial subtypes in the Drosophila brain
Source: Gene Expr Patterns. 2014 Sep;16(1):61–8. doi: 10.1016/j.gep.2014.09.001 (PMC4222725; doi:10.1016/j.gep.2014.09.001)
Supplement: Table S11 — GO analysis (cellular processes) of genes with significantly decreased expression ≤1.5 fold in repo-Gal4, UAS-InR CNS tissue. p-value ≤0.01. [file mmc11.docx]

*Supplementary table S11. GO analysis (cellular processes) of genes with significantly decreased expression ≤1.5 fold in repo-Gal4, UAS-InR CNS tissue. p-value ≤0.01.*

| **Gene Ontology term** | **Cluster frequency** | **Genome frequency** | **Corrected P-value** | **FDR** | **False Positives** | **Genes annotated to the term** |
| --- | --- | --- | --- | --- | --- | --- |
| [neuropeptide signaling pathway](http://amigo.geneontology.org/cgi-bin/amigo/go.cgi?view=details&query=GO:0007218) | 8 of 226 genes, 3.5% | 30 of 7732 genes, 0.4% | 0.00075 | 0.00% | 0.00 | [CG14375](http://flybase.bio.indiana.edu/.bin/fbidq.html?CG14375), [Dsk](http://flybase.bio.indiana.edu/.bin/fbidq.html?Dsk), [Dh](http://flybase.bio.indiana.edu/.bin/fbidq.html?Dh), [Crz](http://flybase.bio.indiana.edu/.bin/fbidq.html?Crz), [hug](http://flybase.bio.indiana.edu/.bin/fbidq.html?hug), [Fmrf](http://flybase.bio.indiana.edu/.bin/fbidq.html?Fmrf), [Mip](http://flybase.bio.indiana.edu/.bin/fbidq.html?Mip), [Ast-C](http://flybase.bio.indiana.edu/.bin/fbidq.html?Ast-C) |
| [regulation of system process](http://amigo.geneontology.org/cgi-bin/amigo/go.cgi?view=details&query=GO:0044057) | 10 of 226 genes, 4.4% | 62 of 7732 genes, 0.8% | 0.00477 | 0.00% | 0.00 | [Dg](http://flybase.bio.indiana.edu/.bin/fbidq.html?Dg), [Syt1](http://flybase.bio.indiana.edu/.bin/fbidq.html?Syt1), [Dsk](http://flybase.bio.indiana.edu/.bin/fbidq.html?Dsk), [comt](http://flybase.bio.indiana.edu/.bin/fbidq.html?comt), [CG3822](http://flybase.bio.indiana.edu/.bin/fbidq.html?CG3822), [CG16976](http://flybase.bio.indiana.edu/.bin/fbidq.html?CG16976), [Snap25](http://flybase.bio.indiana.edu/.bin/fbidq.html?Snap25), [CG42629](http://flybase.bio.indiana.edu/.bin/fbidq.html?CG42629), [Fmrf](http://flybase.bio.indiana.edu/.bin/fbidq.html?Fmrf), [Ast-C](http://flybase.bio.indiana.edu/.bin/fbidq.html?Ast-C) |
| [G-protein coupled receptor signaling pathway](http://amigo.geneontology.org/cgi-bin/amigo/go.cgi?view=details&query=GO:0007186) | 14 of 226 genes, 6.2% | 130 of 7732 genes, 1.7% | 0.00958 | 0.00% | 0.00 | [CG14375](http://flybase.bio.indiana.edu/.bin/fbidq.html?CG14375), [Dsk](http://flybase.bio.indiana.edu/.bin/fbidq.html?Dsk), [Dh](http://flybase.bio.indiana.edu/.bin/fbidq.html?Dh), [Crz](http://flybase.bio.indiana.edu/.bin/fbidq.html?Crz), [Dh31-R1](http://flybase.bio.indiana.edu/.bin/fbidq.html?Dh31-R1), [GABA-B-R3](http://flybase.bio.indiana.edu/.bin/fbidq.html?GABA-B-R3), [Dh44-R1](http://flybase.bio.indiana.edu/.bin/fbidq.html?Dh44-R1), [DopR2](http://flybase.bio.indiana.edu/.bin/fbidq.html?DopR2), [hug](http://flybase.bio.indiana.edu/.bin/fbidq.html?hug), [Mip](http://flybase.bio.indiana.edu/.bin/fbidq.html?Mip), [Ggamma1](http://flybase.bio.indiana.edu/.bin/fbidq.html?Ggamma1), [Fmrf](http://flybase.bio.indiana.edu/.bin/fbidq.html?Fmrf), [CG31760](http://flybase.bio.indiana.edu/.bin/fbidq.html?CG31760), [Ast-C](http://flybase.bio.indiana.edu/.bin/fbidq.html?Ast-C) |
